# Identification and characterization of cichlid TAAR genes and comparison with other teleost TAAR repertoires

**DOI:** 10.1186/s12864-015-1478-4

**Published:** 2015-04-23

**Authors:** Naoual Azzouzi, Frederique Barloy-Hubler, Francis Galibert

**Affiliations:** UMR CNRS/Institut de Génétique et Développement de Rennes, Faculté de Médecine, Université de Rennes 1, 2 avenue Léon Bernard, Rennes, 35000 France

**Keywords:** Cichlids, Zebrafish, Medaka, Stickleback, Fugu, Tetraodon, Trace amine-associated receptors, Splice exons

## Abstract

**Background:**

TAARs (trace amine-associated receptors) are among the principal receptors expressed by the olfactory epithelium. We used the recent BROAD Institute release of the genome sequences of five representative fishes of the cichlid family to establish the complete TAAR repertoires of these species and to compare them with five other fish TAAR repertoires.

**Results:**

The genome sequences of *O. niloticus, P. nyererei, H. burtoni, N. brichardi and M. zebra* were analyzed by exhaustive TBLASTN searches with a set of published TAAR gene sequences used as positive bait. A second TBLASTN analysis was then performed on the candidate genes, with a set of non-TAAR class A GPCR (G protein-coupled receptors) used as negative bait. The resulting cichlid repertoire contained 44 complete TAAR genes from *O. niloticus,* 18 from *P. nyererei,* 23 from *H. burtoni,* 12 from *N. brichardi and* 20 from *M. zebra*, plus a number of pseudogenes, edge genes and fragments. A large proportion of these sequences (80%) consisted of two coding exons, separated in all but two cases by an intron in the interloop 1 coding sequence. We constructed phylogenetic trees. These trees indicated that TAARs constitute a distinct clade, well separated from ORs (olfactory receptors) and other class A GPCRs. Also these repertoires consist of several families and subfamilies, a number of which are common to fugu, tetraodon, stickleback and medaka. Like all other TAARs identified to date, cichlid TAARs have a characteristic two-dimensional structure and contain a number of amino-acid motifs or amino acids, such cysteine, in particular conserved positions.

**Conclusions:**

Little is known about the functions of TAARs: in most cases their ligands have yet to be identified, partly because appropriate methods for such investigations have not been developed. Sequences analyses and comparisons of TAARs in several animal species, here fishes living in the same environment, should help reveal their roles and whether they are complementary to that of ORs.

**Electronic supplementary material:**

The online version of this article (doi:10.1186/s12864-015-1478-4) contains supplementary material, which is available to authorized users.

## Background

Trace amine-associated receptors (TAARs) are a class of chemoreceptors belonging to the G protein-coupled receptor (GPCR) superfamily [1-3]. They have been found in the olfactory epithelium, where, unlike human and zebrafish TAAR1, they are expressed together with the main olfactory receptors (ORs), each by a specific subset of neurons [4]. Mammalian genomes contain only small numbers of TAAR genes: 15 in mice, 17 in rat and six in humans [5-7]. In contrast, 109 TAAR genes have been identified in zebrafish, 50 in stickleback and 27 in medaka, although only 13 such genes have been identified in fugu [8]. Inversely, mammals have thousands of OR genes [9-11], whereas many fish have fewer than a hundred such genes [12-14].

Ligands have been identified for only a small number of ORs and TAARs; this is at least partly because the methods required are complex and suitable high-throughput techniques are not available. It is therefore difficult to interpret and explain the very different numbers of receptors and OR/TAAR ratios in mammals and fishes. Possibly, these differences reflect differences in physiology and/or environment or different agonist distributions. It is also possible that some ligands are recognized by TAARs in fish and ORs in mammals. Alternatively, these differences may simply be a consequence of the techniques applied to define and characterize genes. For example, Hashigushi and Nishida [8] reported the presence of 21 TAAR genes and Libants et al. 28 genes [15] in the lamprey genome; other researchers carrying out phylogenetic studies suggested that this species had only two TAAR genes [16]. Several mouse TAARs respond to isoamylamine, trimethylamine, and β-phenylethylamine, all of which are present in mouse urine and are thought to act as sex pheromones suggesting that TAARs may be involved in the detection of social cues [4,17,18]. In the goldfish, *Carassius auratus*, olfactory sensitivity to catecholamines (epinephrine, norepinephrine, and dopamine) and their metabolites has been confirmed, and goldfish may communicate chemically, through the release of catecholamines into the water [19]. In the masu salmon, *Oncorhynchus masou masou*, L-kynurenine, a metabolite of L-tryptophan, acts as a sex pheromone [20]. If TAARs are not merely biogenic amine receptors but also have other functions, then the size and diversity of TAAR repertoires in different species could provide insight into the relative complexity and species specificity of pheromone-based behavior. TAARs are evolutionarily very ancient [15] and the long evolutionary processes that have occurred in the TAAR gene family may reflect the evolution of chemical communication in reproduction and social interaction in vertebrates. Cichlids, particularly those of the Great East African Lakes, display astonishing phenotypic diversity: hundreds of species may coexist in a single lake [21,22] without interbreeding, even though fertile descendants can be obtained from laboratory crosses between different species. Efforts to unravel the molecular mechanisms underlying the remarkable phenotypic diversity of cichlid fishes have recently focused on sequencing of the genomes of the Nile tilapia (*Oreochromis niloticus*) and four East African cichlids: *Astatotilapia burtoni*, *Pundamilia nyererei*, *Metriaclima zebra* and *Neolamprologus brichardi/pulcher.* Transcriptomic analyses have been performed and a general annotation of these five genome sequences was recently published [23]. To investigate the role of social communication in the development and coexistence of such large numbers of closely related species in the Great African Lakes, we established the complete TAAR gene repertoires of these five cichlid fishes. We present here a list of the TAAR genes identified, and some of their structural characteristics. Many of these TAAR genes contained two coding exons, a characteristic shared by some other teleost TAAR repertoires, such as those of stickleback, medaka, fugu and tetraodon.

## Results and discussion

### Cichlid TAAR repertoires

We carried out a TBLASTN search of the five cichlid genome sequences determined by the BROAD Institute [23], with a set of 199 sequences corresponding to 109 zebrafish, 27 medaka, 50 stickleback and 13 fugu annotated TAAR genes retrieved from the GenBank and ENSEMBL databases [8] (Additional file 1). This initial search, with a cut-off of 1e^−50^, identified a number of candidate receptors; false candidates were identified by a second TBLASTN search with 247 fish class A non-TAAR GPCR sequences (Additional file 2) and were excluded. Finally, we performed a TBLASTX search against the fish database (NCBI, taxiD: 7898).

Table 1 shows the number of TAAR genes identified in the five cichlid genomes (this work) and in the genome sequences of zebrafish, medaka and fugu [8]. Tetraodon and stickleback TAAR gene sequences were extracted from the ENSEMBL database and curated by hand (this work). Direct comparison of gene content between different genome sequences could be biased by the differences in completeness and accuracy of the sequence data. Nevertheless, the numbers of TAAR genes differ very substantially between these ten species: only 12 genes were identified in tetraodon and 109 in zebrafish. The other species were intermediate, from 12 for *N. brichardi* to 44 for *O. niloticus.* In addition to “complete” genes, for which a putative ATG start site and a stop codon could be identified, we detected a number of pseudogenes and edge genes. Pseudogenes are genes with an interrupted open reading frame (ORF), and mostly do not encode active proteins. They may result from a mutation changing a sense codon into a stop codon or from the introduction into (or loss from) the reading frame of one or several nucleotides leading to a shift of translation frame. The pseudogenes we detected were more the result of frameshift than nonsense mutations (Table 2). Edge genes are gene fragments encoding either the N-terminal or the C-terminal part of the protein. Their existence reflects the fragmented nature of genome assembly, which gave many contigs of a mean N50 size of 23.5 kb [23].

**Table 1:** **TAAR genes identified in the genomes of five cichlids and five other model fishes**

	***O. niloticus***	***H. burtoni***	***M. zebra***	***N. brichardi***	***P. nyererei***	***O. Latipes***	***G. aculeatus***	***T. Rubripes***	***T. nigroviridis***	***D. rerio***
Total	44	23	20	12	18	27	50	13	12	109
1 codingexon	9	3	4	3	3	7	7 (5 + 2)	5	10	109
2 coding exons	35	20	16	9	15	20 (17 + 3)	43 (40 + 3)	8	2	0
Pseudo	8	3	5	2	3	7	15	6	4	10
Edge	13	4	5	13	7					
Fragment	1	2	1	0	2					

Cichid TAAR genes were retrieved from the genome sequences determined by the BROAD Institute [23], as explained in the Methods section. Medaka, stickleback, fugu and zebrafish TAAR genes were obtained from Hashiguchi and Nishida [8]. The stickleback and medaka repertoires were updated for this work. The first numbers in brackets correspond to the gene numbers retrieved from Hashiguchi and Nishida [8] and the second number is the gene number assigned in this work. The tetraodon TAAR genes were retrieved from ENSEMBL and further characterized in this work.

**Table 2:** **Distribution of frame-shift and in-frame mutations leading to pseudogenes in the five cichlid TAAR repertoires**

	***O. niloticus***	***M. zebra***	***P. nyererei***	***N. brichardi***	***H. burtoni***
Frameshift	7	1	2	2	2
In-frame stop	1	3	2	0	1

A large proportion (~81%; range: 75 and 86%) of cichlid TAAR genes has two coding exons and encodes a functional receptor (Table 1). The functionality of these genes has not been conclusively demonstrated, but there are three lines of evidence to suggest that they are real genes rather than the result of sequencing errors.

**Figure 1:**
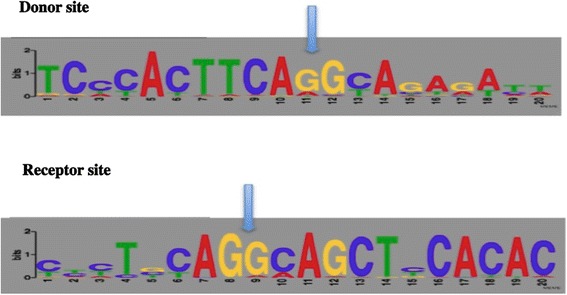
Donor and acceptor splice site sequences. Sequence Logo representation of the donor and acceptor splice sites identified in cichlid TAAR genes aligned with their genomic sequences and manually corrected by both MAFFT multiple alignment and use of the FSPICE tool [24,25].

The first line of evidence is provided by the analysis of nucleotide sequences at exon-intron junctions. The nucleotide sequences present at exon-intron boundaries, as determined by the alignment of the gene nucleotide sequences determined by the TBLASTN search with their cognate contigs, were found to be highly conserved (Figure 1 and Additional file 3). Delimitation of the positions of the exon-intron boundaries, as indicated by the arrows, keeps the reading frame open. Most of these donor/acceptor splice sites were also identified with the FSPLICE program [24] on the FISH model weight matrix (data not shown).

**Figure 2:**
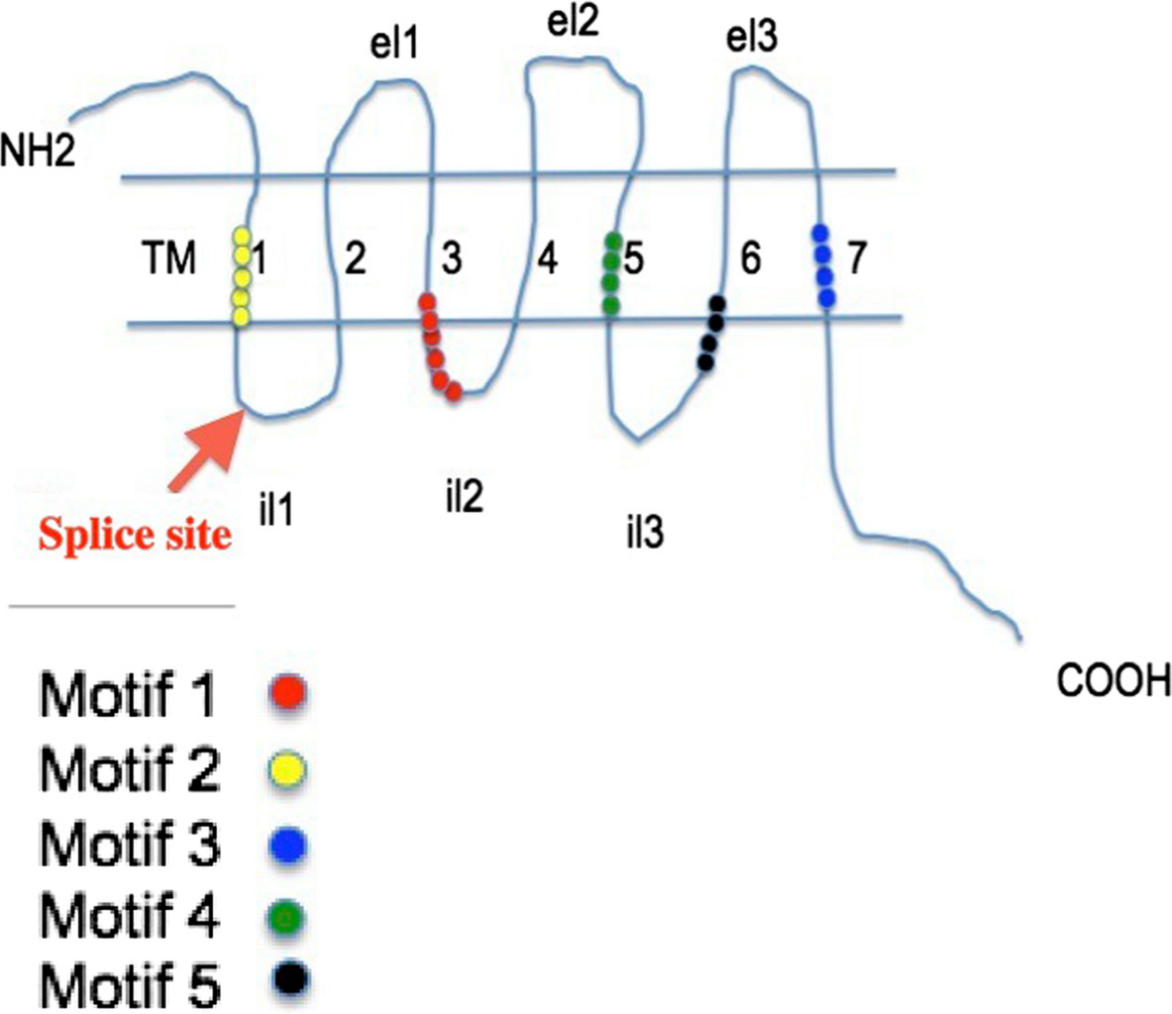
2D schematic view of TAAR structure. The different parts of the molecules are shown: external N terminus, the 7 transmembrane segments (TM1 to TM7), the external loops (EL), the internal loops (IL) and the internal C terminus. The position of the intron disrupting the sequences to most TAAR genes, corresponding to that is indicated.

The second line of evidence relates to intron position: (1) in all cases, the introns are in phase 0. They therefore interrupt the reading frame but not the last codon of the first exon at the donor splice site; (2) the intron sequences interrupt the coding sequences at an almost fixed position, close to codon 55, within the first internal loop defined by the seven transmembrane domains (Figure 2 and Additional file 4). Only two of the 95 TAAR genes identified did not follow this pattern. Interestingly, these two receptors, BurTAR.A016 and BriTAR.A014, have a sequence encoding a different dipeptide at the junction of the two exons. In 92 genes, the intron interrupts a DNA sequence encoding a dipeptide consisting of an aromatic residue at the end of the first exon and a basic residue at the start of the second exon (75 Phe-Arg, 13 Phe-Lys and 4 Tyr-Arg). In contrast, in BurTAR.A016 and BriTAR.A014, the dipeptide sequences are Leu-Thr and Asn-Leu. Finally, TAAR gene TilTAR.A026 is also an exception. Its intron is located just after codon 57, as most of the other introns, but the dipeptide encoded at the junction of the two exons is Phe-Glu. The basic amino acid is therefore replaced with an acidic amino acid (Table 3 and Additional file 5).

**Table 3:** **Dipeptides encoded by the mRNA splice junctions in the various cichlid TAARs**

	**FR**	**FK**	**FE**	**YR**	**LT**	**NL**
***O. niloticus***	30	3	1	1		
***P. nyererei***	11	4				
***H. burtoni***	15	3		1	1	
***N. brichardi***	5	2		1		1
***M. zebra***	14	1		1		

Of the 95 pairs of amino-acids encoded at the splice sites, 92 have a basic amino-acid encoded by the first codon of the second exon and 88 have a phenylalanine encoded by the last codon of the first exon. The one-letter amino-acids code is used.

**Figure 3:**
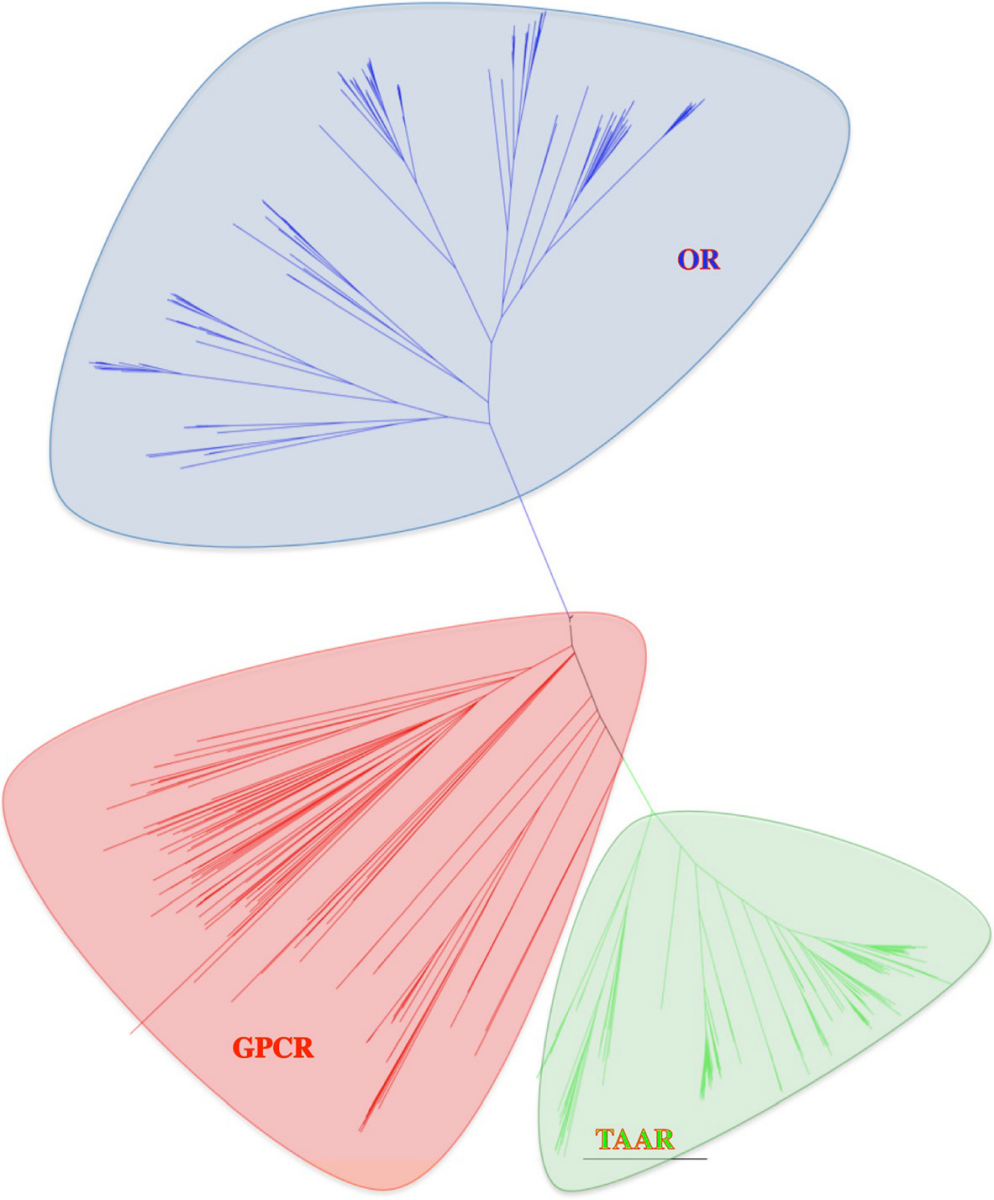
Phylogenetic tree constructed with OR and TAAR cichlid repertoires and non TAAR-class A GPCRs. Amino-acid sequences of 117 TAARs, 247 non- TAAR class A GPCRs (Additional file 2) and 506 cichlid ORs [14] were aligned with MAFFT [25] and PHYML [26] and the tree constructed with FigTree [28]. Each group of receptors constitutes well separated clade in this tree.

Additional evidence was provided by BLASTX and phylogenetic analyses, which demonstrated that these genes encoded true TAARs and not other GPCRs, which are generally encoded by more than one coding exon. All the candidate TAAR genes identified by the TBLASTN search against the positive and negative query sets were analyzed further, by a TBLASTX search against the non-redundant NCBI protein database. We retained only proteins giving a strong hit with TAAR proteins and no hit or a meaningless hit with other GPCRs. Finally, a phylogenetic tree was constructed with all the cichlid TAARs identified in this study and 753 class A GPCRs (247 non-TAAR GPCRs and 506 ORs). All of the TAAR sequences clustered in clades independent of those formed by all class A non-TAAR GPCRs (Figure 3).

### Cichlid TAAR families and subfamilies

**Figure 4:**
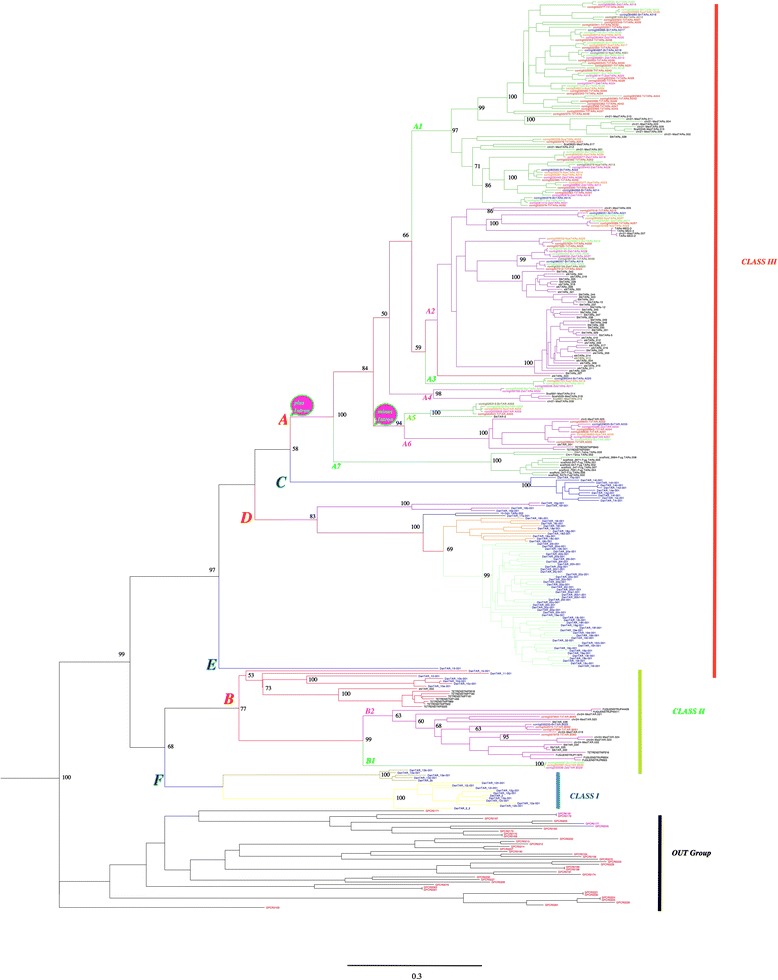
Ciclid TAAR phylogenetic tree. Phylogenetic tree constructed with the cichlid TAARs (*n* = 117) and the model fish TAARs (*n* = 211). TAAR names are color-coded according to the fish species: Till in red, Bur in green, Bri in blue, Nye in orange, Zebra in purple and model fish TAARs in black. The percentage of identity shared by each adjacent gene pair was calculated with CLUSTAL W [51] and thresholds of 40% and 60% were used to define families and sub-families (see Methods section). Families are designated by letters (A to F) and sub-families by numbers (1 – 7). Classes I, II and III, as defined by Hussain *et al.* [16] are indicated. Gains and losses of introns are indicated at the roots of family A and subfamilies 5 and 6, respectively. The out group made of 38 class A nonTAAR GPCRs is at the bottom of the tree. Number above branches are bootstrap values based on 1,000 replicates, Values below 50% are not shown. The scalebar represents the average number of nucleotide substitutions per site.

We used MAFFT [25] and PHYML [26] to align the TAAR amino-acid sequences and construct a phylogenetic tree with the five cichlid repertoires (*n* = 117) and 211 TAAR sequences from five other model fish species: zebrafish (*Danio rerio n* =109), medaka (*Oryzias latipes n* = 27*),* stickleback (*Gasterosteus aculeatus n* = 50), takifugu (*Takifugu rubripes n* = 13) and tetraodon (*Tetraodon nigroviridis* n = 12) (Figure 4). Using 40% and 60% as amino-acid identity thresholds for the comparison of different receptors, as recommended in a previous study [27], we identified six families (A to F) and 17 subfamilies. The largest family, family A, contained 109 cichlid receptors and 78 model fish receptors (45 from stickleback, 20 from medaka, 8 from fugu, and 4 from tetraodon, but none from zebrafish). This family comprised seven subfamilies, six of which were common to the cichlids and the other fishes and contained a number of medaka, stickleback and tetraodon receptors. The remaining subfamily (A7) contained only two tetraodon and eight fugu receptors. Family B was relatively small and consisted of six subfamilies. It contained eight cichlid receptors, forming two subfamilies (B1 and B2) and 47 model fish receptors, 13 of which belonged to subfamily B1, the others belonging to subfamilies B3 to B6, which contained no cichlid receptors. The other families (C, D, E and F) contained 122 model fish receptors but no cichlid receptors (Table 4).

**Table 4:** **Family and sub-family gene distribution**

		**Cichlids**					**Fish models**					
		***N. bri.***	***N. bur.***	***P. nye.***	***O. nil.***	***M. zeb.***	***G. acu.***	***O. Lat.***	***T. rub.***	***T. nig.***	***D. rer.***	
CLASS III	A1	6 s(6e,1p)	12 s(2e,3p,2f)	11 s(5e,1f)	29 s(7e,2p,2sp)	11 s(1e,2ep1fs,3p)	1 s	11 s				
CLASS III	A2	2 s(1e,1ep)	6 s	3 s(2e,1p)	6 s(3e,1es,1p)	3 s(3e)	42 s	5 s				
CLASS III	A3	1 s(1e)	1 s(1e)	1 s(1 sp)	(1p)	1(1e)						
CLASS III	A4	(1e)	1 s	(1f)	(1f)	1 s		4 s				
CLASS III	A5	1	1	1	1	1	1					
CLASS III	A6	1 (2e)	1 (1e)	1 (1p)	4 (1e)	2	1	1	2			
CLASS III	A7								2 s	8 s		
CLASS III	C										10	Group III
CLASS III	D										64	Group V, VI & VII
CLASS III	E										1	
CLASS I	B1	1 (1e)			4 (1e)		4	5	1	3		
CLASS I	B2	(1e)	1	1	(2p)	1						
CLASS I	B3							1		2		
CLASS I	B4						1		7			
CLASS I	B5										5	Group I
CLASS I	B6										2	Group I
CLASS II	F										16	Group XII & XIV
	Total	12 (13e,2p)	23 (4e,3p,2f)	18 (7e,3p,2f)	44 (13e,8p,1f)	20 (5e,5p,1f)	50	27	12	13	98	

Distribution of TAAR genes, pseudogenes and edge genes identified in the five cichlids and the other five model fishes (Table 1), between the various families and subfamilies. The left column shows the corresponding classes (I to III) defined by Hussain *et al.* [16] and the right column shows groups I to XIV defined by Gloriam *et al.* [43]. Lower case letters e, f, p and s are for edge, fragment, pseudo and spliced gene, respectively.

A large proportion (~80%) of the cichlid TAAR genes had an intron interrupting the ORF (Figure 2 and Additional file 4). The phylogenetic tree drawn with FigTree [28] grouped all the cichlid receptors and the other fish TAARs (from medaka, tetraodon, stickleback and fugu) encoded by two exons together in subfamilies 1 to 4 and 7 of family A (Table 4). These subfamilies contained only spliced genes. Visual inspection of the phylogenic tree and computation of amino-acid sequence identity between close pairs of TAARs enabled the identification of a number of orthologs sharing 99% identity or more. The number (*n* = 8) of orthologous pairs was largest between *H. burtoni* and *M. zebra*. We also identified four triplets common to *H. burtoni*, *M. zebra, P. nyereri* and one triplet common to *H. burtoni, P. nyereri* and *N. brichardi* (Table 5, Additional file 6). This finding is reminiscent of our observations for cichlid OR repertoires, although the number of almost identical orthologous OR genes was greatest between *H. burtoni, P. nyereri* and *M. zebra* [14].

**Table 5:** **Pairs and triplets of orthologous genes with high percentage of identity**

**Pairs**		***N. bur.***	***M. zeb.***	***N. bri.***	***P. nye.***	***O. nil.***
	*N. bur.*	0	8	0	1	0
	*M. zeb.*		0	0	0	0
	*N. bri.*			1	1	0
	*P. nye.*				0	0
	*O. nil.*					1
**Triplets**						
	*N. bur.*	*M. zeb.*	*P. nye.*	4		
	*N. bur.*	*P. nye.*	*N. bri.*	1		

Distribution of pairs and triplets of orthologous TAAR genes with high percentage nucleotide and amino-acid sequence identities.

Distribution of TAAR gene pairs and triplets displaying at least 99% amino-acid sequence identity, as calculated with ClustalW [51], were identified from the phylogenetic tree.

### Gene localization

Using RH and FISH mapping data [29], we were able to anchor most of the tilapia genome sequence [23] onto its karyotype. We also localized the 66 *N. tilapia* TAAR genes, edge genes and pseudogenes identified in this study (Additional file 7). All these sequences were located in a single RH group, RH2, which is part of LG16-21. This RH group itself consists of 10 scaffolds, the largest of which (scaffold 78), contains 39 TAAR genes. Within RH2, the TAAR genes are flanked by four OR genes on one side and 32 on the other [14]. On the RH map, RH2 is followed, at an estimated distance of 13 MB, by RH4, which contains another set of 48 OR genes and edge genes. Despite the fragmented nature of the genome sequence available, six tilapia TAAR gene pairs and one gene triplet were found to be on the same contigs. These genes were all oriented tail-to-head, with intergene distances of a few kilobases (1153 nt to 6146 nt). The orientation of the contigs within the different scaffolds and that of the scaffolds themselves indicate that most of the TAAR genes are located on the same DNA strand, with very few organized head-to-head or tail-to-tail (Additional file 7). Interestingly, the genes for tilapia TAARs from the same subfamily tend to be located on the same scaffold, as illustrated by scaffold 78, which contains only receptors of subfamily A1 and includes 49 of the 51 receptors in this subfamily. Similarly, TAARs located close together on the phylogenic tree tend to be located side-by-side on the genome, as shown by a group of six Tilapia genes (TilTAR.0042, 43, 44, 45, 46, and 47). This arrangement corresponds to classic genome expansion, consistent with the *cis*-duplication event that gave rise to the TAAR repertoire.

**Figure 5:**
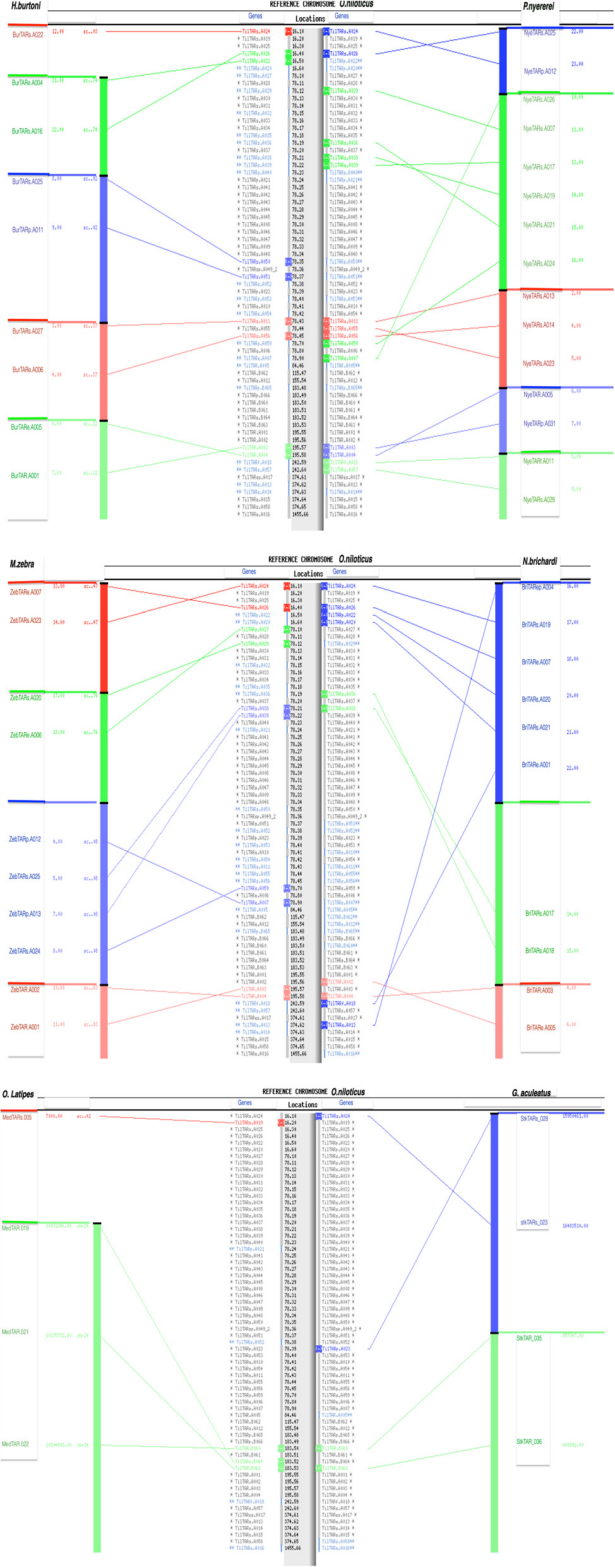
Genome contig localization. A large cluster of TAAR genes (n = 44) mapped to LG16-21 (see Additional file 7) is shown in the central part of the Figure with their names on the right and their coordinates on the left. On either side of the top panel, are the *H. burtoni* (left) and *P. nyereri* (right) scaffolds identified with AutoGraph [52], These scaffolds contain TAAR genes identified by phylogenetic analysis as orthologous to the tilapia genes indicated in the central part see for example the orthologous gene pairs *(BurTARe.A004/TilTARp.A022; BurTARs.A016/TilTARs.A026; BurTARs.A025/TilTARs.A050; BurTARp.A011/TilTARs.A051; BurTARs.A027/TilTARe.A011; BurTARe.A006/TilTARs.A056; BurTARe.A005/TilTAR.A004; BurTAR.A001/TilTAR.A00)*. Similarly, *M. zebra* (left) and *N. brichardi* (right) scaffolds are aligned with the middle panel, and *O. latipes* chr 24 (left) and *G. aculeatus* (right) group XVIII [8] scaffolds are aligned with the lower panel.

In the absence of RH mapping and FISH experiments, it was not possible to anchor the sequences of the other four fish genomes to their cognate karyotypes. However, comparison of the TAAR gene contents of the largest scaffolds present in each genome sequence and alignment of the most closely related genes, as identified in the phylogenic tree, suggested that TAAR genes were organized similarly in the five cichlids (Figure 5).

### Evolution and dN/dS ratios

Pairwise comparison of the TAAR nucleotide sequences revealed the mutations that had occurred during the development of these repertoires; this comparison also made possible to distinguish between silent and nonsense mutations and to calculate the dN/dS ratios (summarized in Table 6). The mean values for these ratios were 0.425 for family A and 0.514 for family B, calculated by the Nei-Gojobori method, as modified by Zhang [30] (Additional file 8). Although well below 1, the theoretical threshold used to distinguish between negative and positive selection, these two values are clearly above the value of 0.11 calculated for 1,880 human/rodent orthologs. Therefore, they suggest at least a tendency towards positive selection, favoring TAAR diversification, as reported for other fishes [16,31]. Interestingly, there were considerable differences between pairs of orthologs, as indicated by the range of the values obtained, extending from 0.12 for Bur TARs.A014/Zeb TARs.A015 to 1.98 for Nye TARs.A028/Til TARs.A057 to even higher values for the BurTAR.B032/NyeTAR.B030 pair, for which one nonsense mutation and no silent mutation were observed. As for OR repertoires [14], intraspecies TAAR dN/dS ratios (paralogous comparisons) were similar to interspecies TAAR dN/dS ratios (ortholog comparisons), indicating similar rates of evolution for the five TAAR cichlid repertoires (Table 7).

**Table 6:** **dN/dS ratios**

**Family names**	**Number of sub-families**	**Number of genes**	**Means**	**Min.**	**Max.**
Fam A	7	99	0.425	0.12	1.98
Fam B	2	8	0.514	0.32	>10

dN/dS ratios for the various TAAR gene pairs in families A and B. dN/dS ratios were calculated by the method of Nei-Gojobori, as modified by Zhang *et al*. [30].

**Table 7:** **Comparison of inter- and intra-species dN/dS ratios**

	**Family A**		
*N. bri.*/*N. bri.*	0.426	*N. bri.*/cichlid	0.428
*N. bur.*/*N. bur.*	0.417	*N. bur.*/cichlid	0.422
*M. zeb*/*M. zeb*	0.400	*M. zeb*/cichlid	0.417
*P. nye.*/*P. nye.*	0.463	*P. nye.*/cichlid	0.440
*O. nil.*/*O. nil.*	0.424	*O. nil.*/cichlid	0.425
	**Family B**		
*N. bri.*/*N. bri.*		*N. bri.*/cichlid	0.564
*N. bur.*/*N. bur.*		*N. bur.*/cichlid	0.467
*M. zeb*/*M. zeb*		*M. zeb*/cichlid	0.469
*P. nye.*/*P. nye.*		*P. nye.*/cichlid	0.531
*O. nil.*/*O. nil.*	0.389	*O. nil.*/cichlid	0.557

dN/dS ratios have been calculated with the method of Nei-Gojobori, as modified by Zhang *et al*. [30] for each pair of genes belonging to Families A and B which contained all cichlid TAAR genes identified in this study.

### Conserved amino-acid motifs and other features

**Figure 6:**
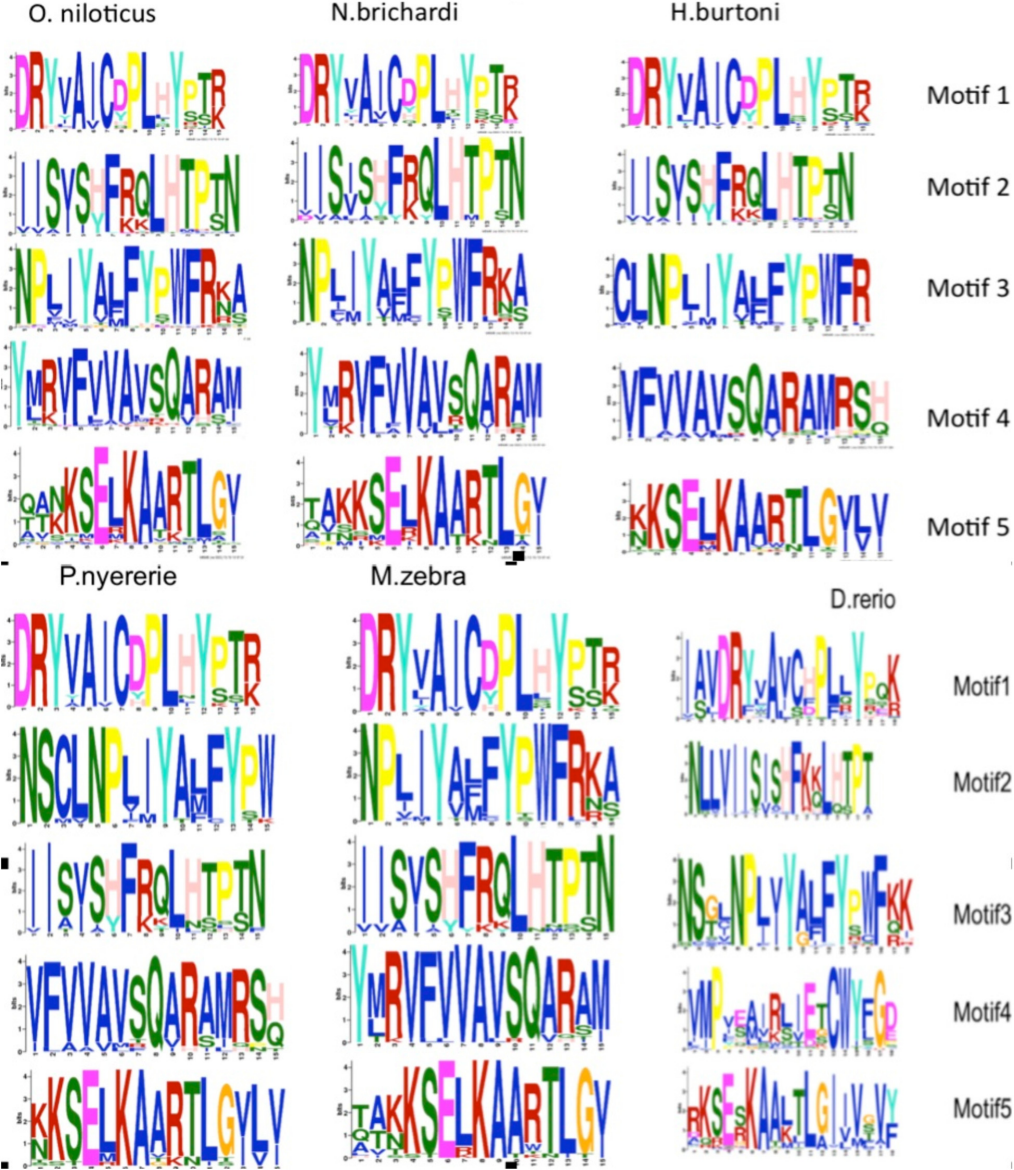
TAAR amino acid motifs. WebLogo graphical representation of the five most significant motifs identified by MEME in the cichlid and *D. rerio* TAAR repertoires. These motifs are located as follows: motif 1: internal loop 2; motif 2: TM1; motif 3: TM7; motif 4: TM5 and motif 5: internal loop 3/TM6.

TAARs are GPCRs of the rhodopsin or class A superfamily. They are characterized by a number of features, such as a specific two-dimensional structure involving seven transmembrane domains, with an extracellular N terminus and an intracellular C terminus [32], and several amino-acid motifs; these motifs include the MAYDRY or, more precisely, the DRY motif, which plays a key role in regulating the conformational state of GPCRs and is responsible for G-protein coupling [33-35]. This DRY motif, located at the junction of the third transmembrane domain and the second internal loop, is the most emblematic motif. We used the MEME program [36] to search for the five best motifs in each of the five cichlid TAAR repertoires and in the *D. rerio* TAAR repertoire. In each of the six repertoires, we identified five very highly conserved motifs (Figure 6) at fixed positions relative to the two-dimensional structure of the corresponding proteins [37] (Additional file 9).

In addition to these motifs, the amino-acid alignment generated with MAFFT [25] enabled us to identify several conserved amino acids, including a cysteine residue present in the N-terminal part of all molecules, a threonine residue in TM1 and two other cysteine residues located in the first external loop (Additional file 10). We also submitted the total set of complete cichlid TAAR sequences to the NetGLY server [38], for the identification of potential signal peptides and N-glycosylation sites (Asn-X-Ser/Thr). As also noted for other TAARs and ORs, these molecules contained no classical signal peptide, despite being anchored to the cell membrane. However, a very high-scoring predicted N-glycosylation site was identified in the N-terminal part of the receptors (Additional file 11). No functional role has yet been suggested for this N-glycosylation site in TAARs, but an identical site has been implicated in the trafficking of ORs to the membrane [39]. A similar role in TAARs is highly likely.

## Conclusions

The main olfactory epithelium was initially thought to detect only volatile compounds. However, following several studies indicating that it also responds to social cues carried by volatile pheromones and small peptides [40-42], Liberles and Buck carried out a large-scale search for additional receptors and identified a second class of receptors expressed by this epithelium [4]. These receptors were then found to correspond to the amine receptors originally identified in the brain and named trace amine-associated receptors (TAARs) [2]. TAARs are found in all vertebrates, but their numbers differ considerably between species. Fifteen such receptors have been identified in mouse, but only five have been found in humans and none have been detected in dogs; by contrast, the zebrafish has more than one hundred [43]. All of the genes identified in this study encode proteins with several characteristic properties common to all of the TAARs identified to date. They all consist of seven hydrophobic transmembrane segments, each 21 to 28 amino acids long. TAAR proteins are translocated to the cell membrane despite lacking a classical signal peptide. Instead, they carry a putative N-glycosylation site (Asn-X-Ser/Thr), identical to that implicated in the trafficking of ORs to the membrane [39]. As for most class A GPCRs, multiple alignments of TAAR amino-acid sequences revealed a number of conserved amino acids at specific locations in the two-dimensional structure. A search with the MEME algorithm identified several amino-acid motifs, including the DRY motif. In addition to these features common to other GPCRs, a phylogenic tree constructed with 506 ORs and 247 non-OR non-TAAR class A GPCRs indicated that the TAAR family constituted a specific clade, well separated from those of ORs and other class A GPCRs.

The size of the TAAR repertoires varied considerably between fish species, essentially with genome size, although there were notable exceptions [44,45]: the zebrafish has more than a hundred TAAR genes, whereas tetraodon has only 12. Variability was also observed in the cichlids but to a much lesser extent (Table 1). There are probably a few more as yet unidentified TAAR genes because some genes currently identified as edge genes may be upgraded to complete, functional gene status when the genome sequences are complete. This variability in the number of TAAR genes is similar to that observed for OR repertoires [14,46], but is less consistent with the higher rate of gene duplication in East African cichlids than in other teleosts as revealed by genome sequence analysis [23].

All mammalian and zebrafish TAAR genes have a single exon and no intron. In contrast, introns were found in 86% of stickleback, 74% of medaka and 61% of fugu TAAR genes [8] (Table 1). A re-analysis of the data in the Genbank database revealed the presence of spliced TAAR genes in the genome of tetraodon, albeit at a much lower frequency (16%).

The numbers of TAAR subfamilies shared between some of the 10 species (stickleback, medaka, fugu, tetraodon, zebrafish and five species of the cichlid family) and the percentage of spliced TAAR genes have evolved in parallel (Table 4). However, this parallel evolution is not entirely consistent with the relative phylogenetic positions of these species. In particular, medaka, which has a number of TAAR subfamilies in common with cichlids and a high percentage of spliced TAAR genes, does not belong to the percomorph group. Inversely, tetraodon and fugu, both of which belong to the tetraodontiform group (a sub-order of the percomorph) share very few TAAR sub-families and have a very different proportion of spliced TAAR genes. Subfamilies A1 to A4 and A7 include only, and all of, the spliced TAAR genes (Table 4 and Figure 4). A most parsimonious hypothesis concerning this distribution is that a gain-of-intron event gave rise to family A, and the subsequent loss of this intron resulting in the creation of subfamilies A5 and A6.

Most documented intron gains and losses have been identified from the analysis of a large number of phyla corresponding to a long period of evolution [47] and such events have been shown to be rare [48,49]. The gain and loss of introns observed in this group of fishes is therefore intriguing. A similar phenomenon has occurred in the development of the olfactory repertoires of this group of fishes [14]. A detailed analysis or re-analysis focusing particularly on this phenomenon during the evolutionary development of the TAAR and OR repertoires would be of considerable interest. Such an analysis may reveal the extent of these gains and losses, the reasons for these events, and their possible consequences for fish behavior.

## Methods

The sequences of the five cichlid genomes were determined by the BROAD Institute [23]. For each species except *M. zebra*, a DNA sample was prepared from one double-haploid individual. In the case of *M. zebra*, DNA was extracted from one individual caught in the wild. For determination of the five TAAR repertoires, we followed the strategy used previously for the OR repertoires [14]. A positive dataset of 109 zebrafish, 50 stickleback, 27 medaka and 13 takifugu TAARs [8] (Additional file 1) was used as bait and an exhaustive TBLASTN search was performed (http://blast.ncbi.nlm.nih.gov/Blast.cgi). The resulting candidate genes were then compared with a negative dataset of 247 non-OR and non-TAAR GPCRs retrieved from the NCBI and ENSEMBL databases (Additional file 2). TBLASTN results were filtered with a homemade Python script to ensure that the sequences retained as actual TAARs met the two following criteria: (1) one or more matches with the positive dataset and (2) no match with the negative dataset, using an e-value cut-off of 1.e^−50^. The candidates retained were rechecked by both BLASTX and BLASTP analyses against the fish protein database (NCBI, taxiD: 7898), using default parameters with a cut-off of 1.e^−100^.

All genes were collected, curated manually and translated into protein sequences with Geneious software 6.1 [50]. Incomplete TAAR genes at the ends of contigs were annotated as “edge genes”, and incomplete TAAR genes located within contigs were called “fragments”. Genes with disruptive frame shifts or stop codons were annotated as pseudogenes. For spliced TAAR genes, predicted sequences and splice sites were deduced by alignment, with MAFFT 7 [25] and FSPLICE [24] and corrected manually. The list and sequences of the complete TAAR genes (spliced and unspliced), pseudogenes, edges and fragments are available as supplementary information (Additional file 5).

Tetraodon TAAR genes were identified from the tetraodon genome sequence (ENSEMBL database), and characterized by the same strategy (Additional file 5). The whole set of cichlid TAARs (Additional file 5) was used as a positive query, and the non-TAAR GPCR genes (Additional file 2) were used as a negative query.

The positions of transmembrane domains were determined with PolyPhobius [37]. The deduced amino-acid sequences of all cichlid, tetraodon (Additional file 5) zebrafish, stickleback, takifugu and medaka TAARs (Additional file 1) were aligned, with the E-INS version of MAFFT 7 [25] (optimal for sequences with conserved motifs and carrying multiple domains), using the default parameters. A classification was proposed on the basis of the percentage identity, calculated with ClustalW [51], between pairs of receptors identified on a bootstrapped maximum likelihood unrooted tree generated by PHYML (1,000 rounds of bootstrapping) and drawn with FigTree 1.3.1. Thresholds of 40% and 60% amino-acid similarity were used to distinguish between families and subfamilies, respectively, as described by Glusman *et al*. [27]. The cichlid TAAR sequences were named according to their phylogenetic positions, as follows: Fish symbol (Bri, Bur, Nye, Til or Zeb for *N. brichardi, H. burtoni, P. nyererei, O. niloticus* and *M. zebra*, respectively) then “TAR”, then s for splice gene, p for pseudogene, e for edge or f for fragment followed by a letter to designate the family and three digits to designate the gene itself. For example, BriTARe.A005 designates TAAR edge gene 005 belonging to family A.

Ratios of non-synonymous to synonymous nucleotide substitutions (ω = dN/dS) were calculated with the method of Nei-Gojobori, as modified by Zhang *et al*. [30], with Perl and Python scripts used to automate the entire process. Conserved motifs in predicted TAAR protein sequences were identified with the online program Multiple Expectation Maximization for Motif Elicitation (MEME) v.4.9.0 [36]. Potential N-glycosylation sites were detected with NetNGlycserver [38]. Only N-glycosylation sites with a “potential” score > 0.5 and board agreement of “++” or higher were considered positive in our analyses.

## Additional files

They are available as additional files numbered 1 to 11. Gene and protein sequences have been deposited to GenBank under the following accession numbers:

file Pundamilianeyereri.sqn:

KP899269 - KP899286

file MetriaclimaZebra.sqn:

KP899287 - KP899306

file Neolaprologusbrichardi.sqn:

KP899307 - KP899318

file Oreochromisniloticus.sqn:

KP899319 - KP899362

file Haplochromisburtoni.sqn:

KP899363 - KP899385.

and the main phylogenetic tree deposited to TreeBase (ref: ID 17227). Additional file 1:
**Positive dataset.** This dataset is made of 109 zebrafish, 27 medaka, 50 stickleback and 13 fugu TAAR genes retrieved from Hashigushi and Nishida [8]. Additional file 2:
**Negative dataset.** This dataset contains 247 fish class A non-TAAR GPCRs retrieved from NCBI. Additional file 3:
**TAAR gene sequence alignment.** TAAR gene sequences were aligned with their cognate genome sequences, with MultiAlin [53], to identify the position of the genes on each contig and the positions of the 2 exons of spliced genes. Additional file 4:
**Intron positions within TAAR genes.** The nature of the last codon of the first coding exon, and its position and its phase are reported. Additional file 5:
**Sequences of the cichlid and tetraodon TAAR genes and their corresponding receptors.** Dipeptides at the splice junction are shown in red. Additional file 6:
**List of pairs and triplets of orthologous genes displaying at least 99% identity.**
Additional file 7:
**List of contigs and scaffolds harboring TAAR genes, with their positions.**
Additional file 8:
**Details of dN/dS ratios calculated for the gene pairs of families A and B.**
Additional file 9:
**Prediction of the** **two-dimensional** **structure of cichlid TAARs by PolyPhobius [** 37 **].**
Additional file 10:
**Multiple alignment and LOGO presentation.** Cichlid TAAR sequences were aligned with MAFFT [25]. The Logo was generated with Geneious [50]. Additional file 11:
**N-glycosylation sites, as predicted by the NetNGly Server [** 38 **], for each cichlid TAAR.**

## Abbreviations

TAAR: Trace amine-associated receptor
OR: Olfactory receptor
GPCR: G protein-coupled receptor
RH: Radiation hybrid
FISH: Fluorescent in situ hybridization
