# Supplementary material for: Identification and characterization of cichlid TAAR genes and comparison with other teleost TAAR repertoires
Source: BMC Genomics. 2015 Apr 23;16(1):335. doi: 10.1186/s12864-015-1478-4 (PMC4415300; doi:10.1186/s12864-015-1478-4)
Supplement: Additional file 1: — Positive dataset. This dataset is made of 109 zebrafish, 27 medaka, 50 stickleback and 13 fugu TAAR genes retrieved from Hashigushi and Nishida [8]. [file 12864_2015_1478_MOESM1_ESM.pdf]

```
>scaffold-347-Fug.TARs.001
MSLEDGDGYISPFNTSCRLMSDAWKSTLTSAALVCLAPLTVVLNLVVVVSISHFRHFQTNTNIILLSMAVSDLLVGLAVMPLMIVTLDSCRCISS
FICFLFDLLSFVLTSASVGNMVLISGDRYVAICYPLRYSSIKPSTVKICVSVCWITAIIYSLILLKDNLLNINFSSSCNKKCPLYMNYILYIADI
IITFYVPLTVIIVLYARVFVVAVTQARAMRSQVSTAGSKPVSSMKSELRAARTLGIVILFFFICFFPYYISSLIGQHMNIEASTGQILLFFSNST
INPIIYAFFYPWFRKTVKLFLSCKFCKL
>scaffold-347-Fug.TARs.002
MSLEDGDGYISPFNTSCRLMSDAWKSTLTSAALACLAPLTVVLNLVVVVSISHFRHLQTTTNLILLSMAVSDLLVGLAMMPLMIVTLDSCQCISS
FICFLSHLLSFVLTSASIGNMVLISVDRYVAICYPLRYSSIKPSTVKICVSVCWISSIIYNLILLKDNLLNIDFSSSCNKKCPLFINYILYIADI
IITFYGPLTVIIVLYTRVFVVAVTQARAMRSQVSTTGSKAVSAMKSELRAARTLGIVILFFFICFFPYFISSIIGQDTSTEASTGQIFLFYCNSA
INPIIYVFIYPWFRKTAKLLLSCKFSKL
>scaffold_5473-FugTARs.003
MSLEDGDGYISPFNTSCRLMSDAWKSTLTSAALVCLPPLTVVLNLVVVVSISHFRHFQTTTNLLLLSMAVSDLLMGLAMIPLMIVTLDSCRCISS
FICFFFHLLSYILTSASVGNMVLISVDRYVAICYPLRYSSIKPNTVKICVSVCWISSIIYSLILLKDNLLHLDSSSCYKKCPLLLNDILSIVDII
ITFYGPLTVIIVLYARVFVVAVTQARAMRSQVSTTGSKTVSSMKSELRAARTLGIIILFFLICFFPYYISTLILQESNEDLTGYILLFYCNSTIN
PIVYAFFYPWFRNSVKLIVSCKFCKR
>scaffold_7591-Fug.TARs.004
MSLEDGDGSISPFNTSCRLMSDAWKSTLISAALVCLAPLTVVLNLVVVVSISHFRHFQTTTNIILLSMAVSDLLVGLAMMPLMIITLDFCWCINS
FLCFFFHLLGFVLTSASVGNMVLISVDRYVAICYPLRYSSIKPNTVKICVSVCWTSSIIYNIILLKDNLLHLDSSSCYKKCPLLLNDILAIVDVV
ITFYVPLTVIIVLYARVFVVAVTQARAMRSQVSTTGSKTESAMKSELRAARTLGIVILFFLMCFFPYYISSLIGQGMNIEVSTVQLLLFYCNSTI
NPIIYAFFYPWFRKTVKLLLNCKFCKL
>scaffold_2971-Fug.TARs.005
MSLEDGDGYISPFNTSCRLMSDAWKSTLISAALACLAPLTVVLNLVVVVSISHFRHFQTTTNIILLSMAVSDLLVGLAVMPLMIVTLDFCWCISS
FICFLFDLLRFVLTCASIGNMVLISVDRYVAICYPLRYSSIKPSTVKICVSVCWISSIIYNLILLKDNLLNIDFSSSCIKKCPLFINYIVFIADI
IITFYVPLTVIIVLYTRVFVVAVTQARAMRSQVSTTGSKAVNAMKSEMRAARTLGIVILFFFICFFPPYISSLIGQGMNIEVSTGQLLLFCSNST
INPIIYAFFYPWFRKTVKLLLNCKFCKL
>scaffold_347-Fug.TARs.006
MSLEDGDGYISPFNTSCRLMSDVWKSTLISAALACLAPLTVVLNLVVVVSISHFRHFQTTTNLLLLSMAVSDLLVGLAVMPFMIVTLDSCRCISS
FICFLSQLLSFVLTSASVGNMVLISVDRYVAICCPLRYSSITPNTVKICVSVCWTSSIIYNLILLKDNLLHLDFSSSCHKKCPLYKNYILGITDI
IITFYGPLTVIIVLYARVFVVAVTQARAMRSQVSTAGSKTVSSMKSELRAARTLGIIILFFLICFFPYYISALILQESNEDLTGYILLFYCNSTI
NPIVYAFFYPWFRNSVKLIVSCKFCKR
>scaffold_347-Fug.TARs.007
MTLMSLEDGDGYISPFNTSCRLMSDAWKSTLISAVLACLAPLTVVLNLVVVVSISHFRHFQTTTNLILLSMAVSDLLVGLAVMPLMIVTLDSCRC
VSSFLCFLFDLFCYILTSASVGNMVLISVDRYVAICYPLRYSSIKPSTVKICVSVCWVIAIIYNLILLKDNLLHINFSSSCNKKCPLYMNYILSI
ADIIITFYGPLTVIIVLYARVFVVAVTQARAMRSQVSTAGSKTVSAMKSELRAARTLGVIILFFLICFFPYYISSLIGQDLSDEASAGQTLLFFS
NSAINPIIYAFFYPWFRKTVKLLLSCKFL
>scaffold_3984-Fug.TARs.008
LNSCCTSEVITSQQHCDTSDVTGRRRWLHLSIQHLLQFQTTTNIILLSMAVSDLLVGLAVMPLMIVTLDFCWCISSFICFLFDLLRFVLTCASIG
NMVLISVDRYVAICYPLRYSSIKPSTVKICVSVCWISSIIYNLILLKDNLLNIDFSSSCIKKCPLFINYIVFIADIIITFYVPLTVIIVLYTRVF
VVAVTQARAMRSQVSTTGSKAVNAMKSEMRAARTLGIVILFFFICFFPPYISSLIGQGMNIEVSTGQLLLFCSNSTINPIIYAFFYPWFRKTVKL
LLNCKFCKL
>FUGUENSTRUP6503
MLMVNDTSTVNEIHPCYISHDVTYTFTHIPSLKCVILYIFLGSLSVVTVCGNALVIISIIYFKQLHVPTNYLILSLAVADLLVGVLVFPFSMAFT
VTSCWYHEDLACKLREVFDVILSTASILNLCCISIDRYYAVCHPLAYKSKINNCIIGVMILVSWGVAALTGICIVYVGRVDGKCEESCLLDALIS
NTLSCIFAFYIPVIVMLHIYLRIFFAAQKQAKQIQNTKSGANVSKMEKKATKTLAIVVGVFLLCWTPYFLCIIFQPLIHEITPISVIEAFNWLAL
SNSTLNPFIYAFFYSWFRSAFRTIISGKIFKINTKIHLDTQNKHT
>FUGUENSTRUP6504
MLMVNDTSTVNEIHPCYISHDVTYTFTHIPSLKCVILYIFLGSLSVVTVCGNALVIISIIYFKQLHVPTNYLILSLAVADLLVGVLVFPFSMAFT
VTSCWYHEDLACKLREVFDVILSTASILNLCCISIDRYYAVCHPLAYKSKINNCIIGVMILVSWGVAALTGICIVYVGRVDGKCEESCLLDALIS
NTLSCIFAFYIPVIVMLHIYLRIFFAAQKQAKQIQNTKSGANVSKMEKKATKTLAIVVGVFLLCWTPYFLCIIFQPLIHEITPISVIEAFNWLAL
SNSTLNPFIYAFFYSWFRSAFRTIISGFTTTKCSCTSRNVFIYN
>FUGUENSTRUP44411
MVAVSHSSQELDNCNESSSRFGITNVVSPYLLCAIFGLLSVIIVCGNLLVITAILYFRQLHSPTHYLILSLAVADLLVGVLVVPFSAVLIVELAS
TQTFICQVRNSCDMFLSTTSILNLCFISLDRYHAVCQPLKYRMIINVRVTVRMIVVSWTLPVLIGIGVTMNDRNPKQSKTRCLLFQNPSLILVII
GTVVFFYLPAVIILTTYLKILIVAQRQARAIKKAAFLSRLPGEMLNKTQRKATRILAIVMGIFFLCWTPFILCLTFNPLNYNTLTVSVIEFFKWL
AWSNSMLNPFVYAFFYSWFRSAFKMIISGQ
>FUGUENSTRUP11670
MEPRLMVNNTSTVNEIHPCYISHDVTYTFTHIPSLKCVILYIFLGSLSVVTVCGNALVIISIIYFKQLHVPTNYLILSLAVADLLVGVLVFPSSM
AFTVTSCWYHEDLFCKIRGSFDVTLSTASILNLCCISIDRYYAVCHPLAYKSKINNRIISIMILVSWGVAALIGIGIIVAGFNQGKCEGSCLIDT
LISTTLACVFSFYIPVIIMLSIYLKIYLVAQRQAKTIQRTNCRNTKPEAIVSKIERKATKTLATVMGVFLLCWTPYFLCIIFQPLTYKVTPISVI
EALNWLTLSNSMLNPFIYAFFYSWFRCAFKIIISGKIFHS
>FUGUENSTRUP44409
MHHMQMIPESDVFEDNCNESSSRFGITNVVSPYLLCAIFGLLSVIIVCGNLLVITAILYFRQLHSPTHYLILSLAVADLLVGVLVVPFSAVLIVE
LASTQTFICQVRNSCDMFLSTTSILNLCFISLDRYHAVCQPLKYRMIINVRVTVRMIVVSWTLPVLIGIGVTMNDRNPKQSKTRCLLFQNPSLIL
VIIGTVVFFYLPAVIILTTYLKILIVAQRQARAIKKAAFLSRLPGEMLNKTQRKATRILAIVMGIFFLCWTPFILCLTFNPLNYNTLTVSVIEFF
KWLAWSNSMLNPFVYAFFYSWFRSAFKMIISGQIFQGDFMNSKLF
>TARs-MED-2
MEGGDLCFPELNSSCHKQRRSPSFILLASVLLSVFSVLTAVLNLLLIISISHFRQLHTPTNLLLLSLGVADFFVGLLLLFQIPLLDGCWYLGDLM
CVFYYIFMVTTTCASIGTMVLISVDRYVAICYPLHYPVKITPKRAGISVFLCWTCSFLYDILMMKDNFKQPGKFSSCSGDCVVRLSYVSQVVDQV
VSFFLPITVIVVLYARVFAVAVSQGRAMRSHVAAVPRKTKVRVRKSEMKAAGTLGVVVFVFFVCLFPSYSASLSGQETVIDTFSAAFMFFLAFFN
SCLNPIIYAFFYPWFRKSIKLIMTLGLLKADSRDANLIYTGAVETE
>TARs-MED-3
MEGGDLCFPELNSSCHKQRRSPSFILLASVLLSVFSVLTAVLNLLLIISISHFRQLHTPTNLLLLSLGVADFFVGLLLLFQIPLLDGCWYLGDLM
CVFYYIFMVTTTCASIGTMVLISVDRYVAICYPLHYPVKITPKRAGISVFLCWTCSFLYDILMMKDNFKQPGKFSSCSGDCVVRLSYVSQVVDQV
VSFFLPITVIVVLYARVFAVAVSQGRAMRSHVAAVPRKTKVRVRKSEMKAAGTLGVVVFVFFVCLFPSYSASLSGQETVIDTFSAAFMFFLAFFN
SCLNPIIYAFFYPWFRKSIKLIMTLGLLKADSRDANLINSKCASG
>TARs-MED-4
```

MEGGDLCFPELNSSCHKQRRSPSFILLASVLLSVFSVLTAVLNLLLIISISHFRQLHTPTNLLLLSLGVADFFVGLLLLFQIPLLDGCWYLGDLM
CVFYYIFMVTTTCASIGTMVLISVDRYVAICYPLHYPVKITPKRAGISVFLCWTCSFLYDILMMKDNFKQPGKFSSCSGDCVVRLSYVSQVVDQV
VSFFLPITVIVVLYARVFAVAVSQGRAMRSHVAAVPRKTKVRVRKSEMKAAGTLGVVVFVFFVCLFPSYSASLSGQETVIDTFSAAFMFFLAFFN
SCLNPIIYAFFYPWFRKSIKLIMTLGLLKADSRDANLIFSYFD
>chr21–MedTARs.001
MSCIKPVSLSVEAVLVQMVLFFVSFLTVALNLLVIITVSHFRQLHSPTNILLLSLAVSDFLTSLILMPAEIIRNVACWFLGDTACFLYNSLSFVL
TSASVGNMVLISVDRYVAICDPLHYPTKITEKRVKLGVCLCWLCSIFYGTFIVKEDLASQGRPNSCYGECVITVDYIEGTIDLVVSFIVPVFVIV
VLYMRVFVVAVSHARAMRSHVTAVTLQPPTTQTVRKSELKAARTLGVLIAVFLMCFCPYYCISLAADNLLNNSFASYSVFLFYLNSTFNPVVYAL
FYPWFRKAVKLIVTLQILQPDSSDANVS*
>chr21–MedTARs.002
MTTAVDDQHEPGVTDTGKTPNRMLCLVYIREREEGGDSTTSSPIAPSHLVLGATWWSLEHRVKASVADPACILKPLHVPSRPWSHIAMDFITGLP
SSRGKTVKMTAAYGYQPSFFPHQEQAASFSGPAAFVRRCCCFAESFRHRLLRHQERFTAVANCRRTAAPEYKPDPVPPPAPYIVVLSALFGSSSS
PGGKPTLYWTKAMFVNILLPVISVITVVLNLLVIISISHFRQLHSPTNILLLSLAVSDFLVGLLLLPIEIYRNTSCWFLGEIMCAICYYLTCYIP
FISIGNIVFISIDRYVAICYPLHYPTRITAAKVKMGVSLCWLWYAFYSTFYIKDHIIHLIARNVSCLGQCSVALDYATGFVDLILTFIFPVTVIV
FLYTRVFVVAVIQARSMRSQVSFVSAQHPGKVKAKRSELKAARTLGVLVFTYLSCFCPFYMYSLIENNLTSTSYAYLLFVVAYFNSCINPVIYTF
SSLWFRKAVKAIVTLQILKPGSCETNIL
>chr21–MedTARs.003
MEIKDNVDFCFPQLLNISCRKPKLHWTKAFFLDTVLSIISIVTVVLNLLVIISVSHFRQLHSPTNIILLSLAVSDFLVGLLLLPLEIHRNTSCWF
LGETICAVYYYLACQIPFVSIGNIILISVDRYVAICHPLHYPTRITAVRVKLCVFLCWLWYGLYSIFYIKDQIIHLIEPDNNVSCLGQCSIIFSY
VTGVVDLILTFILPVTAIVVLYMRVFVVAVIQARSMRSHVTFVSVQHPGKVKAKRSELKAARTLGVLVFTYLSCFCPAYCFLLVEIDFSSSYLLV
ALFYFNSCLNPVIYTLFYPWFRKAVKLILTLQILKPDSCVTNML
>chr21–MedTARs.004
MEIKDEDEFCFPQLPNISCTKPRLHWTEAFFLDTVLSVISIITVVLNLLVIIAVSHFRQLHSPTNILLLSLAVSDFIVGLLLLPLEIYRNTTCWY
LGEIICAVYYYLGCYIPYTSIGNIILISADRYVAICHPLHYPTRITAVRVKLCVSLCWLWYGLYSIFYIKDQIIHLIEPDNNVSCLGQCSIILSY
VAGVVDLLSTFIIPVTAIVVLYMRVFVVALIQARSMRSHVTFVSVQHPGKVKAKKSELKAARTLGVLVFTYLLCFCPAYCFSLVGIYVPSAYLFI
ILFYFNSCLNPVIYTLFYPWFRKAVKLIITLQILKHDSCETN
>chr21–MedTARs.005
MQTQAKEAELCFPHMLNSSCRRSGRPPAESMFMYIILCSMSVLTVALNLLVIISISHFKQLQTPTNLLLLSLAVSDFFVGLLVVFQILLIDGCWF
LGDVMCVAYYIVDYVVTSASVETVVLISVDCYVAICEPLRYSMKVTKRRVCISVSGCWIFSFLCVFLVMKNNLQQPGSHNSCQGECVVVFDQFSG
LVDVLFFFVGPVSVVLVLYIRVFVVAVSQVRAMRTCAVHGSARVTLKKSELKSARTLGLVGLAFLICLCPYFSVTQTGQEAQFQASSFAFVLFYF
NSLLNPLIYALFYPWFRKSAKLIITLQVLRAGSSQINVE*
>chr21–MedTARs.006
MEIQDTGEFCFPQLLNSSCRKLTIQWTKAMFLNTVLSIISVITVVLNLLVIISVSHFRQLHSPTNIILLSLAVSDFLVGLLLLPLEIYRNTTCWY
LGEIMCAVYYYFACSIPYTSIGNIILISADRYVAICHPLHYPTRITAVRVKLCVFLCWLWYGLYSIFYIRDQIIHLIKPDKDVSCLGQCSIIFSY
VTGVADLILTFIIPVTAIVVLYMRVFVVAVIQARSMRSHVTFVSVQHPSKVKAKRSELKAARTLGVLVFTFLLCFCPLYCYTFIDINLIDTYNQY
LLTGLFYFNSCLNPVIYTLFYPWFRKAVKLIITLQILKPDSCETNML
>chr21–MedTARs.007
MEGGDLCFPELNSSCHKQRRSPSFILLASVLLSVFSVLTAVLNLLLIISISHFRQLHTPTNLLLLSLGVADFFVGLLLLFQIPLLDGCWYLGDLM
CVFYYIFMVTTTCASIGTMVLISVDRYVAICYPLHYPVKITPKRAGISVFLCWTCSFLYDILMMKDNFKQPGKFSSCSGDCVVRLSYVSQVVDQV
VSFFLPITVIVVLYARVFAVAVSQGRAMRSHVAAVPRKTKVRVRKSEMKAAGTLGVVVFVFFVCLFPSYSASLSGQETVIDTFSAAFMFFLAFFN
SCLNPIIYAFFYPWFRKSIKLIMTLGLLKADSRDANLM
>chr21–MedTARs.008
MMETLNTDELCFPHLLNASCRKPPQRPAENVFIFMVLCLLSVLTVTLNLLVIISVSHYRQLHTPTNLLLLSLAVSDFLVGFFVMPIRILLTEGCW
VLGTFMCGLFNFASFILTSVSVGHMVLISADRYVAICDPLRYPTKVTLRRVQVCVCLCWAASAFYNGVILQVALTDPDNNNHCLGECVLLVDYLT
GAFDVVITFFGPIIVIVVLYMSVFVVAVSHARAMRSHIISLQSSAHLSVKKSELKAAAALGVVVFMFLVCFCPYFLPSVTGQDLDSSHFSSIFGI
WLFYANSCLNPVIYAFFYPWFRKSLKSIVTLQILHPNSCDVLLL*
>chr21–MedTARs.009
MEIQDTGEFCFPQLLNSSCRKSTIQSTKAMFLNTVLSIISVITVVLNLLVIISVSHFRQLHSPTNIILLSLAVSDFLVGLLLLPLEIYRNTTCWY
LGEIMCAVYYYLGCYIPYISISNIILISIDRYVAICHPLQYPTRITAVRVKLCVFLCWLWYGLYSIFYIKEQIINLIEPGNNVSCLGQCSLFLSY
VIGVADLILTFIIPVTAIVVLYMRVFVVAVIQARSMRSHVTFVSVQHPSKVKAKRSELKAARTLGVLVITYLSCFCPLYCYTFIYINLTDLSNAY
LFIVLFYFNSCLNPVIYTLFYPWFRKAVKLIITLQILKPDSCVTNIL*
>chr21–MedTARs.010
MEMQDEIELCFPQLLNSSCRKPKLHWTKAVLLNILMSCLSLTTVALNLLVIISVSHFRQLHTPTNIFLLSLAVSDFLVGLLLMPFEILRNTACWL
LGDIACSLYLFVVSLLLCVSNGNIVLISVDRYLAICYPLNYSTRITTAKAKRYIFLCWFLSSLSSVFYVRDQLIQPGKSNSCFGGCELAIDYVAG
IVDLILNFIFSVTLIVVLYTQVFVVAVSQARAMCSRVTATSPQHSAKPKAKRSELKAARTLGILVFVYLLCFCPYYGYSLVQVKITSSSFAFFVV
FLFFSNSCFNPVIYALFYRWFRKAIKLILTLQILQPGSCETNML*
>chr21–MedTARs.011
MEIQYAAELCFQQLPNISCRKPRLHWTKAAFFNILLSIISIITVILNLLVIIAVSHFRQLHTPTNILLLSLAVSDFLVGLLLLPLDIYRSTSCWF
LGEIMCAFNFFLACQIPFVSVGNMILISVDRYVAICHPLHYPTRITAVRAKIFVFLCWFWYGLYSLFYIRDQITDLTQPFRKDSCLGECAYIISY
ATGVVDLIFTLIFPVTVIVFLYMRVFMVAVIQARSMRSHVTFVSVQHSGKVRAKRSELKAARTLGVIVFVYLLAFCPYYCYSLVDINLTESVFAY
FMIGFVYFNSCLNPMIYTLFYPWFRKAIKLIITLQILKPNSCETNMLKRDCGLTEAT*
>chr21–MedTARs.012
MELQLGVELCFPPLSNSSCRKPRSALSEAVFLRFLLFSLSLITVALNLLVIIAVSHFRQLHTPTNILLLSLAVSDFLVGLLLMPGEILRNTACWF
LGDLMCSLYNYVSCIITSTSVGDMVLISVDRYVAICDPLHYPSRVTERRVKVCVCLCWLCSALYNSGIVMSDLTQPGRYNSCHGECVIAVIYVVG
SLDLVLTFIVPVFVIVVLYMRVFVVAVSQARAMRSHVTAVTLQLSVAATSKKSELKAARTLGVLMLVFLTCFCPYYCVSLAGDELISDSSASYSL
FLFYLNSTLNPLIYAMFYPWFRKAVSLIVTLQILHPSSSEVNIH*
>Scaf691–MedTARs.013
METLNTAELCFPHLLNASCRKPPQRPAENVFIFMVLCLLSVLTVTLNLLVIISVSHFRQLHTPTNLLLLSLAVSDFLIGFFVMPIRIRITEGCWV
LGTFMCGLYTFTAFILTSVSVGHMVLISADRYVAICDPLRYPTKVTLRRVQVCVCLCWATSAFYNGVILKDFLEDPDRYNLCLGECVLFIDYITG
AFDVVITFFGPIIVIVVLYMRVFVVAVSQARAMRSHIVSVQSSAHRSVKKSELKAAAALGVVVFMFLVCFCPYFYPNIAGLAYKSSDFLRVFGTW
LFHSNSCLNPVIYAFFYPWFRKSLKSIFTLQILQPDSSX
>Scaf691–MedTARs.014
MMETLNTDELCFPHLLNASCRKPPQRPAENVFIFMVLCLLSVLTVTLNLLVIISVSHFRQLHTPTNLLLLSLAVSDFLVGFFVMPIRFFLTEGCW
VLGTFMCGLCTFTAFILTSVSVGHMVLISADRYVAICDPLRYPTKVTLRRVQVCVCLCWAASAFYNGVILQDALRDPDKYNLCLGECVVLIDYIS
GVFDVVITFFGPIIVIVVLYMSVFVVAVSHARAMRSHIVSVQSSAHLSIKKSELKAAAALGVVVFMFLVCFCPYFYPNIAGLAYKSSDFLAVFVT
LLFYCNSCLNPVIYAFFYPWFRKSLKSIVTLQILQPDSSDANIL*

>Scaf2246-MedTARs.015
MEIQDTGEFCFPQLLNSSCRKLTIQWTKAMFLNTVLSIISVITVVLNLLVIISVSHFRQLHSPTNIILLSLAVSDFLVGLLLLPLEIYRNTSCWY
LGAIMCAVYYYLGCQIPFVSIGNIILISVDRYVAICHPLHYPTRITAVRVKLCVFLCWLWYGLYSIFYIRDQIIHLIKPDKDVSCLGQCSVIFSQ
VTGAADLILTFIIPVTAIVVLYMRVFVVAVIQARSMRSHVTFVSVQHPSKVKAKRSELKAARTLGVLVFTYLLCFCPLYCYTFIDINLIDTSNHY
LLIGLFYFNSCLNPVIYTLFYPWFRKAVKLIITLQILKPDSCVTNIL*
>Scaf3620-MedTARs.017
MSILDRTDLCFPQFPNNSCKKPAFSLSETVSLHFLLSCISLLTTALNLIIIIAVSHFRQLHTPTNILLLSLAVSDFLVGLLAMPAEIMRRTSCWF
LGDSLCFLYNYVSYIITSASVGVMVLISADRFVAICDPLHYPSRVTERRAKVCVCLCWLISALYNIILVKDELIQLGMHNSCYGECVIIVDSITG
TIDTVLTFIVPVFVIVVLYMRVFVVAVSQARAMRSHVTAVTLQLSVAATTKKSELKAARTLGVLVIVFLLCFCPYFCVSVAGSDLLKRFSEFVAF
LFFFNSTINPLIYALFYPWFRKAVKLIITLQILEPGSCETNIL*
>Scaf4535-MedTARs.018
LNTDELCFPHLLNASCRKPPQRPAENVFIFMVLCLLSVLTVTLNLLVIISVSHFMFQGSRNNYQFKHFNCCFCVYRQLHTPTNLLLLSLAVSDFL
IGFFVMPIRILLTEGCWVLGTFMCGLFNFASFTLTSVSVGHMVLISADRYVAICDPLRYPTKVTLRRVQVCVCLCWAASVFYNGVILKDFLEDPD
KYNLCLGECLLLIDYITGAFDVVITFFGPIIVIVVLYMRVFVVAVSQARAMRSHIVSVQSSAHLSVKKSELKAAAALGVVVFLFLVCLCPYFYPN
IVGLTFKSSDFLAVLGTWLFHSNSCLNPVIYAFFYPWFRKSLKSIVTLQILQPDSSDANIL
>chr24-MedTAR.019
MVKDKALNITYSVTDLCYEFDHFSNKLTSSPSVICVILYFFLALLSVITVFGNLLVIISIAYFRQLHTPTHSLILSLAVADLLVGIVVFPLSMAF
SLSSCLFYDGLLCKLRQGFDITFSTCSIMNLCCISVDRYFAVCQPLMYRTKINQRVVVVMIVVSWGVSVLVGIGVIIAGFNNSRCEDYCLINVFI
VSITGPVLSFYFPVIIMLCIYLKIFLVAQRQARSIQMAAKCAAAVSKMERKSTKTLATVLGVFLCCWTPFFLCITFLPFTSSPIPVPVIEAMSWL
ALSNSTLNPFIYAFYYRWFRSAFKMIVSGKILQKNFAHTTTVQ
>chr24-MedTAR.020
MLTRTSSTKCSLLYMFLGSLSVVTVCGNLLVIIAIIYFKQLHTPSNYLILSLAVTDLLVGLVVLPISIVFALSSCLYDRGIFCKIRDSFDVVLST
TSLFNLCCISVDRYYAVCQPLTYRVRMSTRVVAVMITMSWGISIFVAIGFVVAELKQEKCKDDCFSDVVLEKVMAPVFSFYLPVLIMIYIYLRIF
FVAQRQARSIQNTNSAATVSHMERKATKTLAIVMGVFLMCWLPFFLCFSLQVLGGVSVPLYETLNWFTLCNSMLNPFIYGFFYSWFRSAFKMIIS
GKIFQWNCSNFKLQ
>chr24-MedTAR.021
MEQGNKTQTLPLNTCYKLASSVLTEATSTACMLLSLFLSTASTVTVCGNLLVITSVVYFRQLHTPTNALILSLAVADLLVGAVVLPLTMKFSVST
CLYNRSVFCLIRSSLDMILGSCSILNLCCISVDRYYAVCQPLTYRFKINNHVIGIMILISWFLPALFAVGFQLGFNRANCVQRCLSFALVTQFIS
FVFLFYLPLILMLCLYLKIFLVAQKQANSLQKHTKSRENANKLEKKATKTLAVIMGVFLLCWLPSFFCITIVSFSRSSDTGPLIEILNWLALSNS
LLNPFIYAFFYSWFRSAFLLIISGKIFFTDLTNVTLQ
>chr24-MedTAR.022
MPIQPVVFTAELHQTSRERQTDSAETSNIDTEFAINGNKMISYIHPCYNLQNGTYVFTSNPSFACVLLYILLGSLSIVTSCGNLLVITSVIYFKQ
LHTPTNYLILSLAVADLLVGVLVFPFSMAVTVSSCMYHEDLFCKIRDSFDITLCTASILNLCCISLERYYAVCQPLTYRTSITAQVTAIMILVSW
GISVFIGITITVAGFSQGSCEENCSSEVVVANTLGPVFSFYLPAIIMLCIYFKILLVAQKQVNSIKNASSSKSGAEVSKMERKATKTLAIVMGVF
LLCLTPYFICVVFQPLFVTPPQIPVIETLNWLTLSNSMLNPLIYAFFYSWFRSAFRIIMSGKIFQRYSANTQLMMNLFENFN
>chr24-MedTAR.023
MANNAAETSQLKGRKWLSVVVNLSPDDFFSPSDRGHRGGLKADPPSGFTSAFGPSYSRERVDSTEMTSSDRKDNFIGLYVLSYIHPCYNLQNSTY
KFTSNPSFACVLLYILLGSLSIVTSCGNLLVITSVIYFKQLHTPTNYLILSLAVADLLVGVLVFPVSMAVTVSSCMYHEDLFCKIRDSFDITLCT
ASILNLCCISIDRYYAVCQPLTYRTSITAQVTAIMILVSWGISVFIGITITVAGFSQGSCEENCSSEVVVANTLGPVFSFYLPAIIMLCIYFKIL
LVAQKQVNSIQNTSSSKSGAEVSKMERKATKTLAIVMGVFLLCLTPYFICVVFQPLFVTPPQIPVIETLNWVALLNSMLNPLIYAFFYSWFRSAF
RIIMSGKIFQSYSASGQLF
>chr24-MedTAR.024
MTSSDRKDNFIGLYVLSYIHPCYNLQNSTYVFTSSPSFACVLLYILLASLSIVTSCGNLLVITSVIYFKQLHTPTNYLILSLAVADLLVGVLVFP
FSMAVPVSSCMYRQGIFCKIRDSFDITLCTASILNLCCISIDRYYAVCQPLTYRTSITAQVTAIMILVSWGISVFIGITIIVVGFSQGWCEENCS
IDVVVANSLGPVFSFYLPAIIMLCIYFKILLVAQKQVNSIQNTSSSKSEVSKMERKATKTLAIVMGVFLLCFTPCFICVVFQPLFVTPPQIPVIE
TLNWLALSNSMLNPLIYAFFYSWFRSAFRIIMSGKIFQSYSASAQLF
>chr2-MedTAR.025
MDDSGGGSSLCFPNLNSSCRRLLLPSFKTALLYVLLAFVSLLTVTLNLLVIISISHFRQLHTPTNALLLSLAGSDLVVGLLVMPIEGVRYLETCW
LLGRLMCTLTPFLSYCLLSGSLGHMVLISVDRYLAICQPLLYPSRVTLSRVVFLICLCWGCSALYNGCILIGHLQRPHRVHTCHGECVVVISHTS
GTVDLFFSLVGPCSVMLVLYARVFVAAVSQVSRLQTVPSVSMVKRSEWKAARTLGIVIAVFLMCFCPYYYPSLAGEDTTTKLSYYAVLSWVMLLN
SCMNPLIYALFYPWFRKTTRLIFTLKILQLRSSETKIL*
>StkTAR-2
MDGMEGAQLCFPQLLNSSCRGLLRPRTESVLLYTLLSSMAVLTVLLNLLVIISISHFAQLHTPTNLLLLSLAISDLLVGLLVMPSEMVSVIETCW
LLGDLLCTLSTMMGSTLVSASVGNMVLISIDRYVAICYPLQYPIQITRSRISVLYNGLILKDRLRQPDRDNSCDGECKVVINNVSGAIDLVFTFF
APCSVIVVLYVRVFTVAVSQARVGSHFTAVGAVKITAKKSERKAARTLGMVILVFLISFCPFYYPALAGQEISNNASSWVIVSWLLFFNSCLNPL
IYALFYPWFRKAVKLIVTLKILEQNSSQENII
>StkTARs-5
MESVGTELCFPQLINTSCRKQIRPHLEAMLIYILLSSISLFTVALNLLVIISISHFKQLHTPTNLLLLSLAISDFFMGLNMIFQIRMMDGCWLFG
DLMCSLLLILASIIFLASVGTMVLISVDRYVAICYPLHYFTKIKTKRVQVCVCLCWMFAALIYSALLRNNLQHPGRYNSCIGECVIDMNYIASLL
DLIVSFIVPITVIVILYIRIFVVAVYQARAMRPHIAVVTMKVTVKKSEMKAARNLGVVVVVFLICVCPYYCFVLTSQNNVYTTSSVTILIWLLNC
NSSLNPLIYATLYPWFRKSIKVIVTLEILKPGSYRTNML
>StkTARs-12
METLVGTELCFPQLINTSCRKPIRPHLEAMLIYMLFSSISLLTVALNLLVIISISHFKQLQTPTNLLLLSLAFSGLFVGIFLIFQIMLIDGCWLL
GDVMCSLWLILSSFITSSSIGTMALISVDRYVAICYPMHYFTKVKPKRVQVCVCLCWMFAALFNSVLLKDTLQHPGRYNSCIGVCVIEINYIASL
LDLIVSFLFPITVIIILYVRIFVVSMKVTVKKSEMKAARNLGVVVVVFLICFCPYCCFVLTSQNNLYTASSVIIVIWLLYFNSCLNPLIYAVLYP
WFRKSIKVI
>StkTARs-15
FRIYTCCRTHHQCTVLLNFSCRKAVNPHFVFMLTYILLSSISLLTVTLNLLVIVSISHFKQLHTPTNLLLLSLAVSDFFVGLILLFQTMLIDGCW
FLGDLMCILYIFLNYIVTSSSVGTMVLISVDRYVAICEPLHYSNKVTQKRVNICVCLCWTCSAFLLILLLRDNLKKTGRYTSCIGECVIVVNYIA
ELADLFLTFIGPVTVIIVLYMRVFVVAVTQARAMRPHVAASPLKFSMTITAKKSEMKAARTLGVVIVVFLLCVCPYYCFALSGHDSLISLSSVNI
LICVFYFNSCLNPLIYAFFYPWFRKSMKLIVTLKIL
>stkTAR_001
MNVSEGRLLCFPGLNSSCTRLPKPRSEAALLYALLACVSLLTVALNLLVIVSIAHFRQLHNPTNTLLLSLAVSDLVVGLLVMPVEGLRYTETCWL
LGELMCALAPYVSYSLLSASVGNMVLISIDRYMAICDPLLYASKITPNRVKASVCVCWSCSILYNGCILMGHLGQPDRFSSCYGECVVVISHVAG
TIDLFLSFLGPCTVMVVLYMRVFVVAVSQLRVIRLQIVAVKVTPSAKRSERKAATTLGIVIAVFLMCFCPYYYPSLAGEDTSTSLSYYALVSWIM
LLNSCVNPLIYALFYPWFRKAIKLIITLRILQPHSQEYKLL

>stkTAR_002
MEPVLCYESRNGSCLRTIYSLPIQITVYMILGVIVVLTVFGNLLVTVSIAYFKQLHTPTNYLISSLAVCDLLLGLLVMFPSMVQCVESCWYFGDV
FCKVYMSSDVMLCTTSILNLLFISIDRHYAICQPLRYREKISVNVVLIMILLSWTISGLIGFGMIFLKLNFWGIEEFYSNHIVCEGECILFQTGL
SSIMSSVLSFYIPGIILLSVYLKIFLVARRQYSIQIASRTCSEGISNTSQKKATKTLAIIMGAFLSFWTPFFVCNIVDPFISYSTPPALFKTLVW
LGYFNSTVNPLIYAFFYSWFRKALKLFTSGIMFKADISETTLFTLFLGD*
>stkTARs_004
METLVRTELCFPQLINTSCRKPIRPHLEAMLIYMLLSSISLLTVALNLLVIISISHFRQLQTPTNLLLLSLAVSDFFVGLFMIFQIIITDGCWLF
GDLMCSLWLILSSIIMTSSVGTMVLISVDRYVAICYPLHYFTKVKPKRVQVCVCLCWMFAASFYSLLMKNNLQHPGRYNSCIGECFIYMNYIANV
IDLIVTFIFPITVIVILYIRIFGVAVYQARAMRSHIAVVTTKVTVKKSEMKAARNLGVVVVVFLICVCPYFCFVLTSQNNVYTASSVTIVIWLLY
CNSCLNPLIYATLYPWFRKSIKVIVTLQILKSGSYQTN
>stkTARs_005
METLVGTELCFPQLINTSCRKPIRPHLEAMLIYILLSSISLLTVALNLLVIISISHFRQLQTPTNLLLLSLAVSDFFVGLFMIFQIMIIDGCWLL
GDLMCSLWLILLSIIITSSIGTMVLISVDRYVAICYPLHYFTKVKPRRVQVCVCLCWMFAALIYSLLMKNNLQHPGRYNSCIGECVIDMNYIASL
LDLIVTFIFPITVVVILYIRIFAVVVYQARAMRSHIAVVTTKVAVKKSEMKAARNLGVVVVVFMICICPYYCLVLTSQINVYTTSSLTIVIWLTQ
FNSCLNPLIYAILYPWFRKSIRLIVTLQILKSGSYRTN
>stkTARs_006
METLVGTELCFPQLINTSCRKQIRPHLEAMLIYIVLSSISLLTVALNLLVIISISHFRQLQTPTNLLLLSLAVSDFFVGLFMIFQIMIIDGCWLF
SDLMCSLWLVLLSILFLTSIGTMVLISVDRYVAICYPLHYFTKVKPKRVQVCVCLCWMFAASFTGLLLKNNLQHPGRYKSCIGECVYDMNYIANV
FDLIISFLFPITVIVILYIRIFVVAVYQARAMRSHIAVVTKKVTVKKSEMKAARNLGVVVVVFLICVCPYYCFVLTSPYNVYTTASVTIVIWLSN
CNSCLNPLIYAILYPWFRKSIKVIVTLQILKSGSYRTNML*
>stkTARs_008
MRSLEEDELCFPHLLNSSCRKAMRPHFVSMLTYILLSSISLLTVTLNLLVIISISHFRQLHTPTNLLLLSLAVSDFFVGLIVFFQIVLIEGCWFL
GDLMCILYIVLDYIVTSASVGTMVLISVDRYVAICEPLHYSNKVTQKRVKICVCLCWTCSAFLQTLLLKDNLEEPGRYNSCFGECVIVINYIAEL
ADLFFTFICPVTVIIVLYMRVFVVAVTQARVMRSHVAVLPHKFSMNITAKKSEMKAARTLGVVIVVFLLCVCPYYCVLLSGQDTVFNVSSAAFVM
CVFSFNSCLNPLIYAFFYPWFRKSLKLIVTLEILQPGSCERNLL*
>stkTARs_009
METLVGTELCFPQLINTSCRKTIRPHLKAMLIYIVLSSISLLTVALNLLVIISISHFRQLQTPTNLLLLSLSVSDFFVGLFMIFQIMITDGCWLL
GDLMCSLWLILASIITSSSIGTMVLISVDRYVAICYPLHYFTKVKPKTVQVCVCLCWMFAASFTGLLMKDNLQHPGRYKSCIGECVIDVNYIANV
FDLIVTFIFPITVIVILYIRIFGVAVYQARAMRSHIAVVTTKVTVKKSEMKAARNLGVVVVVFLLCVCPYYCFVLTSQNNLYTISSATFVIWLFY
CNSCLNPLIYAILYPWFRKSIRLIVTLQILKSGSYRTNVL*
>stkTARs_010
METLVGTELCFPQLINTSCRKTILPHLEAMLIYIVLSSISLLTVALNLLVIISISHFRQLQTPTNLILLSLAVSDFFMGLIMIFQIMIIEGCWLF
SDLMCSLWLILASIITSSSIGTMVLISVDRYVAICYPLHYFTNVKPKTVQVCVCLCWMFAALYNSLLLKNNLQHPGRYNSCIGECVFDMSYIANV
FDLIVTFVFPITVIVILYMRIFGVAVYQARAMRSHIAVVTIKVTVKKSEMKAARNLGVVVVVFLICACPYYCFVLTSQNNVYTASSVTFVIWLFN
FNSCLNPLIYAILYPWFRKSIKVIVTLQILKSGSYRTNSIYENIWGGCVSGSCHAVSHCSCDNKSNR*
>stkTARs_011
METLVGTELCFPQLINTSCRKPIRPHLEAMLIYILLSSISLLTLALNLLVIISISHFRQLQTPTNLLLLSLAVSDFFVGLLMIFRIMMIDGCWLL
GDLMCSLWLILSSIIISSSVGTMVLISVDRYVAICYPLRYFTKVKPERVQVCVCLCWMFAALFNSVLLKNNLQHPGRYNSCIGECVFEMNYIAYL
IDMTVSVLFPITVIVILYTRIFGVAVYQARAMRSHIAVVTTKITVKSELKAARNLGVVVVVFLICICPFYCFALTSQTNVYTATSVTFVIWLFHF
NSCLNPLIYAILYPWFRKSIRLIVTLQILKPGSYRTNML*
>stkTARs_012
METLVGTELCFPQLINTSCRKTIRPHLEAMLIYIVLSSISLLTVALNLLVIISISHFRQLHTPTNLLLLSLAVSDFFVGLFMIFQIMIIDGCWLF
SDLMCSLWLILLSILFLTSIGTMVLISVDRYVAICYPLHYFTKVKPKTVQVCVCLCWMFAASYTGLLLKNNLQHPGRYNSCIGECVYDMNYIANV
FDLIISFLFPITVIVILYIRIFGVAVYQARAMRSHIAVVTTKVTVKKSEMKAARNLGVVVVVFLLCVCPYYCFVLTSPYNVYTTASVTIVIWLSN
CNSCLNPLIYAILYPWFRKSIKVIVTLQILKPGSYRTNML*
>stkTARs_013
METLVGTELCFPQLINTSCRKTILPHLEAMLIYIVLSSISLLTVALNLLVIISISHFRQLQTPTNLLLLSLAVSDFFMGLNMIFQITITDGCWLF
GDLMCSLWFILSSIIISSSVGTMVLISVDRYVAICYPLHYFTKVKPKRVQVCVCLCWMFAALIYSLLLKNNLQHPGRYNSCIGECVIDVNYIASL
LDLIVTFIFPITVIVILSIRIFGVAVYQARAMRSHIAVVTKKVTVKKSEMKAARNLGVVVVVFLICVCPYYCFVLTSQNNVYTASSVIIVIWLFN
CNSCLNPLIYAILYPWFRKSIKVIVTLQILKSGSYRTNML*
>stkTARs_014
METLVGTELCFPQLINTSCRKTILPHLEAMLIYIVLSSISLLTVALNLLVIISISHFRQLQTPTNLLLLSLAVSDFFMGLNMIFQITITDGCWLF
GDLMCSLWFILSSIIISSSVGTMVLISVDRYVAICYPLHYFTKVKPKRVQVCVCLCWMFAALIYSLLLKNNLQHPGRYNSCIGECVIDVNYIASL
LDLIVTFIFPITVIVILSIRIFGVAVYQARAMRSHIAVVTKKVTVKKSEMKAARNLGVVVVVFLICVCPYYCFVLTSQNNVYTASSVIIVIWLFN
CNSCLNPLIYAILYPWFRKSIKVIVTLQILKSGSYRTNML*
>stkTARs_015
METLVGTDLCFPQLINTSCRKTIRPQLEEILIYVLLSSISLLTVALNLLVIISISHFRQLQTPPNLLLRSLAVSDLFVGLFMIFQIMIIEGCWLF
GDLMCSLWLILSSIVVLSSVGTMVLISVDRYVAICYPLHYFTKVKPERVQVCVCLCWMFAALIYSLLLKNNLQHPGRYNSCIGECVFDVNYIVNL
LDLIVTFIFPITVIIILYIKIFVVAVYHARAMRSHIAVVTTKVTVKKSELKAARNLGVVVVVFLICICPYYCFALTSQNNLYTASSVTIVVWLFH
FNSCLNPLIYAILYPWFRKSIKVIVTLQILKHGSYQANML*
>stkTARs_016
METLVGTELCFPQLINTSCRKTIRPHLEAMLIYILLSSISLLTVALNLLVIISISHFRQLQTPTNLFLLSLAVSDFFMGLNMIFQIMIIEGCWLF
SDLMCSLWLILSSIIISSSVGTMVLISVDRYVAICYPLHYFTKVKPKTVQVCVCLCWMFAASFTGLLLKNNLQHPGRYNSCIGECVFDMNYIAYV
FDLIVTFIFPITVVVILYIRIFAVAVYQARAMRSHIAVVTLKVTVKKSEMKAARNLGVVVVVFLICICPYYCFVLTSQNNLYTISSITIVIWLFQ
CNSCFNPLIYAILYPWFRKSIRLIVTLQILKPGSYRTNML*
>stkTARs_017
METLVGTELCFPQLINTSCRKPIRPHLEAMLIYIVLSSISLLTLALNLLVIISISHFRQLQTPTNLLLLSLAVSDFFMGLNMIFQIMIIEGCWLF
SDLMCSLWLILSSIIISSSVGTMVLISVDRYVAICYPLHYFTKVKPKAVQVCVCLCWMFAALIYSLLMKNNLQHPGRYNSCIGECVIDMNYIAYV
FDLIVTFIVPITVIVILYIRIFAVVVYQARAMRSHIVVVTTKVTVKKSEMKAARNLGVVVVLFMICVCPYYCFVLTSQNNLYTISSATIVIWLFQ
CNSCFNPLIYAILYPWFRKSISLIVTLQILKPGSYRINML*
>stkTARs_018
MKSLEEDELCFPHLLNSSCRKAMHPHFVSMLTYILLSSISLLTVTLNLLVIISISHFRQLHTPANLLLLSLAVSDFFVGFIVFFQIVLIEGCWFL
GDLMCILYIVLDYIVTSASVGTMVLISVDRYVAICEPLHYSNKVTQKRVQICVCLCWTCSAFLQTLLLKDNLEEPGRYNSCFGECVVVINYIAGL
ADLFFTFIGPVTVIIVLYMRVFVVAVTQARVMRSHVAVLPHKFSVNITAKKSEMKAARTLGVIIVVFLLCVCPYYCVLLSAQDTLFNVSSAAFVM
CVFYFNSCLNPLIYAFFYPWFRKSMKLIVTLEILQPGSCERNLL*
>stkTARs_019

MKSLEDDELCFPHLLNSSCRKVVRPHFVSIITYILLSSISLLTVTLNLLVIISISHFRQLHTPTNLLLLSLAFSDFFVGLIMFFQVVLTEGCWFL
GDLICTLYQYLAYIITSSSVGTMVLISVDRYVAICEPLHYSNKVTQKRVQISVCLCWTCSAFLHSVLLKDNLEEPGTYNSCFGECVVVINYIAGL
ADLFFTFIGPVTVIIVLYMRVFVVAVTQARIMRSHVAVLPLKVSMNITAKKSEMKAARTLGVVIVVFLLCLCPYYCVALSDQDTLFNVSSATIVT
CVFYFNSCLNPLIYAFFYPWFRRSMKLIVTLEILQPGSCERNLL*
>stkTARs_020
METLVGTELCFPQLINTSCRKTIRPHLEAMLIYIVLSSISLLTVALNLLVIISISHFRQLHTPTNLLLLSLAVSDFFVGLFMIFRIIIIDGCWLL
GDLMCSLWLIISSTIISSSIGTMVLISVDRYVAICYPLHYFIKVKPRRVQVCVCLCWMFAALYNSELMKDNLQHPGRYNSCIGECIIDIDYIANL
LDLIISFIFPITLIVILYIRIFGVAVYQARAMRSHIAVVTTKETVTKSEMKAARNLGVVVVVFLICAFPYYCFVLTSQNNVYTASSVTIVIWLFY
LNSCLNPLIYATLYPWFRKSIKVIVTLQILKPGSSRANML*
>stkTARs_021
MKSLEDELCFPHINSSCRKAVRPHFVSMFTYILLSSISLLTVTLNLLVIISISHFRQLHTSTNLLLLSLGVSDFFVGLIVFFQILLIDGCWFLGD
LVCILYLYVAYVITSASVGTMVLISVDRYVAICEPLHYSNKVTQKRVKICVCLCWTCSAFLHSVLLKDNLEEPGRYNSCFGECVIVINYIAGRAD
LFFTFIGPVTVIIVLYMRVFVVAVTQARVMRSHVAVLPLKVSMNITAKKSEMKAARTLGVVIVVFLLCLCPYYCVALSGQDTLLDASSATIVICV
FYFNSCLNPLIYAFFYPWFRRSMKLIVTLEILQPGSCERNLL*
>stkTARs_022
MKSLVEDELCFPHLNSSCRKVVRPHFVSMFTYILLSSISLLTVTLNLLVIISISHFRQLHTPTNLLLLSLAVSDLFVGLIVFFQILLIDGCWFLG
DLVCILYLYVAYVITSASVGTMMLISVDRYVAICEPLHYSNKVTQKRVTICVCLCWTCSAIYSTLLLKDNLEEPGRYNSCFGECVIVINYIAGLA
DLFFTFIGPVTVIIVLYMRVFVVAVTQARVMRSHVAVLPHKFSVNKTAKKSELKAARTLGVVIVVFLLCFCPYYCVSLSGQDTYLDASSATIVIC
VFYFNSCLNPLIYAFFYPWFRRSMKLIVTLEILQPGSCERNLL*
>stkTARs_023
METLPGDYLCFPQLNSSCKKPAHPHTEAMLVYVLLSSISLITVVLNVLVIISISHFRQLHSPTNLLILSLAVSDLLVGLLLMPVEIIYIEACWFL
GDILCTTYYLVDYVITSASVANMVLISVDRYIAICEPLHYPTKVTKRRVQNCVCFCWIWSAIFRIFLLYDHLEKPGSSNSCLGECVVIINYAAGV
ADLVFTFIIPILCIIVLYLRVFVVALSQARAMRSRVAASTTRRSGATTVKKTEMKAARTLGLVILVFLFCFCPYYYPALAGKDTSIDASSAAFEI
WLAHFNSCLNPVIYAFFYPWFRKSIQLILTLQILKPGSCDANVL*
>stkTARs_024
MKSLEDDELCFPHINSSCRKVVRPHFVSMLTYILLSSISLLTVTLNLMVIISISHFRQLLILLIISLLFSYLLRQLHTPTNLLLLSLAVSDFFVG
LIVFFQIVLIEDCWFLGDHMCILYTFLDYIITSASVGTMVLISVDRYVAICEPLHYSNKVTQKRVTICVCLCWTGSAIYSTLPLKDNLTEPGRNN
SCFGECVIVINYIAGLADLFLIFIGPVTVIIVLYMRVFVVAVTQARVMRSHVAVLPHKFSMNITAKKSEMKAARTLGVVIVVFLLCVCPYYCVAL
SDQDTLINVSSGTIVVCVFYFNSCLNPLIYAFFYPWFRRSMKLIVTLEILQPGSCERNLL*
>StkTARs_025
MKSLEEDELCFPHLNSSCRKAMRPHFVSMLTYILLSSISLLTVTLNLMVIISISHFRQLLQTMINLLISYLLRQLHTPTNLLLLSLAVSDFFVGL
FVFFQIVLIEGCWFLGDLICILYSFLDFTITSSSVGTMVLISVDRYVAICEPLHYSNKVTQKRVKICVCLCWTCSAFLQTLLLKDNLEEPGRYNS
CFGECVIVINYIAGRANLFLTFICPVTVIIVLYMRVFVVAVTQARVMRSHVAVLPHKFSMNITAKKSEMKAARTLGVVIVVFLLCLCPYYCVSLS
GQDTLINASSATIVICLFYFNSCLNPLIYAFFYPWFKKSMNLIVTLEILQPGSCERNLL*
>StkTARs_026
MKSLEEDELCFPHLFNSSCRKAMRPHFVSMLTYILLSSISLLTVTLNLLVIISISHFRQLHTPTNLLLLSLAVSDFFVGFIVFFQIVVIEGCWFL
GDLMCILYIFLDYIITSASVGTMVLISVDRYVAICEPLHYSNKVTQKRVKISVCLCWTCSAFLQTLLLKDNLEEPGRYNSCFGECVVVINYIAGL
ADLFFTFIGPVTVIIVLYMRVFVVAVTQARVMRSHVAVLPHKFSMNITAKKSEMKAARTLGVVIVVFLLCVCPYYCVLLSAQDTLFNISSAAFVT
CVFYFNSCLNPLIYAFFYPWFTKSMKLIVTLEILQPGSCERNLL*
>StkTARs_027
METLVATELCFPQLINTSCRKTIRPHLEAMLIYILLSSISLLTVALNLLVIISISHFRQLQSPTNLLLLSLAVSDFFVGLLMIFQIMMIDGCWLL
GDLMCSLWFILTIIVFSSSVGTMVLISVDRYVAICYPLHYFAKVKPESVKVCVCLCWMFAALYNSLMIKDNLRHPGRYNSCIGECVIDIEYITVL
FDLIVTLIFPITVIIILYIRIFGVAVYQARAMRSHIADVTLKVTVKKSEMKAARNLGVVVVVFMICICPYYCFVLTSRNNMYTASSVTFVIWLLY
CNSCLNPLIYAILYPWFRKSIKVIVTLQILKPGSRRTNML*
>StkTARs_028
MAEHASQLCFPQLLNVSCRKPSVLWNDAVLSLALMSVSALTVALNLVVIISVSLFRSEFWCFWPVDVRACYFKDAFLFPTRHLHTPTNILIVSLA
VSDLFVGLLVMPVEILFRVSCWFLGDLVCALFNYVFFIITSVSVGTMVLISLDRYLSICEPLHYATRVTVKRVRCSVCTCWLCSVCYSGVLASDG
VTPSGRYSSCHGECLIVIDYVTVTVDLVVTFGVPVSVIVVLYMRVFAVAASQARSLRSHVLVVTHQLPVGQRAKKSELKAARTLGVLVVLFLICF
CPYYIVSLVGYSQLSNLYMSFVLYLFYLNSCLNPLIYALFYPWFRKAVKRIVTLQTGS*
>StkTARs_029
METLVGTELCFPQLINTSCRKTIRPHLEAMLIYILLSSISLLTVALNLLVIISISHFRQLHTPTNLLLLSLAVSDFFMGLNMIFQIIMIDGCWLF
GDLMCSFWLILSSILFLASVGTMVLISVDRYVAICYPLHYFTKIKTKRVQVCVCLCWMFAALIYSLLLKNNLQHPGRYNSCIGECVIDMNYIASL
LDLIVTFIFPITVIVILYIRIFVVAVYQARAMRSHIAVVTMKATVKKPEMKAARNLGVVVVVFLICVCPYYCFVLTSKSNVYTTSSVTIVLWLFN
CNSCLNPLIYATLYPWFRKSIKVIVTLEILKPGSYRTNML*
>StkTARs_030
MESVGTELCFPQLINTSCRKTIRPHLEAMLIYILLSSISLFTVALNLLVIISISHFRQLHTPTNLLLLSLAVSDFFMGLNMIFQIIMIDGCWLLG
DLMCSLLLILASVITMSSIGTMVLISVDRYVAICYPLHYFTKVKPESVQVCVCLCWMFAALFNSALLKNNLQHPGRYNSCIGECVIDMNYIASLL
DLIVTFIFPITVIVILYIRIFVVAVYQARAMRSHIAVVTMKATVKKPEMKAARNLGVVVVVFLVCVCPLYCIVLTSQNTVYTTSSVTILIWLFNC
NSCLNPLIYATLYPWFRKSIKVIVTLEILKPGSYRTNML*
>StkTARs_031
METLVGTELCFPQLLNTSCRKTILPHLEEMLIYILLSSISLLTIALNLLVIISISHFRQLQTPTNLLLLSLAVSDFFMGLNTIFQIIMIDGCWLL
GDLMCSFWLILSAILFLASIGTMVLISVDRYVAICYPLHYFTKIKTKRVQVCVCLCWMFAASLNSLLLKNNLQHPGRYNSCIGECVIDINYITSL
LDLIVTFIFPITVIVILYIRIFVVAVYQARAMRSHIAVVTTKATVKKPEMKAARNLGVVVVVFLICVCPYYCFVLTSQNNVYTTSSLTIVIWLFN
CNSCLNPLIYATLYPWFRKSIKVIATLEILKPGSCRTNML*
>StkTAR_033
MEPEETDNGFNAVSDLHPCYESDNGTYSFTSNFSISCVSLYVFLGGLSVVTICGNLLVIISVSYFKQLHVATNFLILSLAVADLLVGVVVFPFSM
VFTVTSCWHHEGLFCKVRGGFDVTLSTASILHLCCISIDRYYAVCQPLSYKSKVNDRVTGMMILMSWAVPLLIGICIIIAGFNQGKCEESCLIDA
LISTTLACIFSFYIPVIIMLSIYLKIFLVAKRQAISIQITTGLTKKSGTTVSKRERKATKTLAIVLGVFLLCWAPYFLCMIFQPITYNVTPIAVI
ETLNWLTLSNSMLNPFIYAFFYSWFRSAFKMIISGKVFWGNLENSKLL*
>StkTAR_034
MEPEETDNGFNAVSNLHPCYESDNGTYSFTSNFSISCVSLYVFLGGLSVVTICGNLLVIISVSYFKQLHVATNFLILSLAVADLLVGVVVFPFSM
VFTVTSCWHHEGLFCKVRGGFDVTLSTASILHLCCISIDRYYAVCQPLSYKSKVNDRVTGMMILMSWAVPLLIGICIIIASFNQEKCEESCLTDA
LISTTLACIFSFYIPVIIILSTYLKIFLVAKRQAISIQNTTGLTKKSGTTVSKRERKATKTLAIVLGFFLLCWAPYFLCVPFNPFSKNSVPTPVI
ETLNWLTLSNSMLNPFIYAFFYSWFRSAFKMILCGKVFWGNFANSKLL*
>StkTAR_035
MEPEETDNGFNAVSDLHPCYESDNGTYSFTSNFSISCVSLYVFLGGLSVVTICGNLLVIISVSYFKQLHVATNFLILSLAVADLLVGVVVFPFSM

VFTVTSCWHHEGLFCKVRGGFDVTLSTASILHLCCISIDRYYAVCQPLSYKSKVNDRVTGMMILMSWAVPLLIGICIIIAGFNQGKCEESCLIDA
LISTTLACIFSFYIPVIIMLSIYLKIFLVAKRQAISIQITTGLTKKSGTTVSKRERKATKTLAIVLGVFLLCWAPYFLCMIFQPITYNVTPISVI
ETLNWLALSNSMLNPFIYAFFYSWFRSAFKIIICGKVFWGNFANSKLL*
>StkTAR_036
MKLDMEINKTNVLTEIHPCYEIDVSYVLAINPSAECALFHIFLGLLSAITVCGNLLVIIAIIYFKQLQNSTNFLILSLAVADLLVGVLVFPLTMQ
FSITLCLYDEDIFCKVRSTFDVLLCTCSILNLCCISVDRYYAVCQPLTYRAKINNHVVAIMILVSWGASIVIGISFIIIGIYQEKCAAKCAIVAL
MANILGLIFSFYLPTIVMLFIYLKIFLVAQRQARRIQNTTSQRTKGGGTVKMERKATKTLAIVMGLFLIAWLPFFLCTTVLSFSRVNVPLPLIEL
LNWFALSNSTLNPFIYAFFYSWFRSAFRIIISGKIFQGNLKNTNLH*
>StkTARs_037
MKSLEEDEICFPHLLNFSCRKAVNPHFVFMLTYILLSSISLLTVTLNLLVIISISHFRQLHTPTNLLLLSLAVSDFFVGLILLFQTMLIDGCWFL
GDFMCILYIFLNYIVVSSSVGTMVLISVDRYVAICEPLHYSNKVTQKRVNICVCLCWTCSAFLLILLLKDNLKKNGRYTSCIGECVIVVNYIAEL
ADFFLTFIGPVTVIIVLYMRVFVVAVTQARAMRPHVAASPLKFSMTITAKKSEMKAARTLGVVIVVFLLCVCPYYCFALSGHDSLISLSSVNILI
CVFYFNSCLNPLIYAFFYPWFRKSMKLIVTLKILQPGSCERNLL*
>StkTARs_038
METLVGTELCFPQLINTSCRKPIRPHLEAMLIYILLSSISLLTLALNLLVIISISHFRQLHTPTNLILLSLAVSDFFVGLFVIFQIMIIEGCWLL
GDLMCSLWLILSSIIMSSSIGTMVLISVDRYVAICYPLHYFTKVKPKRIRVCVCLCWMFAALFNSLLLKNNLQHPGRYNSCIGECVIEMNYIANV
FDLILSFFFPITVIVILYIRIFGVVVYQARAMRPHIAVVTMKVAVKKSEMKAARNLGVVVVVFLICVCPYYCFTLTSQNNLYTSLSVTILVWLFQ
FNSCLNPLIYATLYPWFRKSIKVIVTLQILKPGSYRTNML*
>StkTARs_039
METLVGTELCFPQLVNTSCRKTIHPHIAAILIYILLSSISLLTVALNLLVIISISHFRQLQTPTNLILLSLSVSDFFIGLFMIFQIMLIDGCWLL
GDLMCSLWLILSSTIFLSSVGTMVLISVDRYVAICYPLHYFTKVKPKRVQVCVCLCWMFAALIYSVLLKNNLQHPGRYNSCIGECVFDMNYIASL
FDLFVTFIFPITVIIILYIRIFCVAVYQARAMRSHIAVVTTKVTVKKSEMKAARNLGVVVVVFMISVCPYYCFVLTSQNNVYTSSSVTIVIWFFQ
FNSCLNPLIYATLYPWFRKSIKVIVTLQILKPGSYRTNVL*
>StkTARs_040
METLVGTELCFPQLMNTSCRKTIRPHLEAMLIYIVLSSISLLTVALNLLVIISISHFRQLQTPTNLLLLSLAVSDFFVGLFMIFQIMLIDGCWLF
SDLMCSLWLVLSSIIMSSSIGTMVLISVDRYVAICYPLHYFTIVKPKRVQVCVCLCWMFAALIYSLLLKNNLQHPGRYNSCIGECVIDVNYIASL
LDLIVTFIFPITVIVILYIRIFVVVVYQARAMRSHIAVVTTKVTVKKSEMKAARNLGVVVAVFMICICPYYCFVLTSQINVYTASSVTIVIWLTN
SNSCFNPLIYAILYPWFRKSIRLIVTLQILKPGSYRTNML*
>StkTARs_041
MKSLEEDEICFPHLLNFSCRKAVNPHFVFMLTYILLSSISLLTVTLNLLVIVSISHFRQLHTPTNLLLLSLAVSDFFVGLILLFQTMLIDGCWFL
GDLMCILYIFLNYIVTSSSVGTMVLISVDRYVAICEPLHYSNKVTQKRVNICVCLCWTCSAFLLILLLRDNLKKTGRYTSCIGECVIVVNYIAEL
ADLFLTFIGPVTVIIVLYMRVFVVAVTQARAMRPHVAASPLKFSMTITAKKSEMKAARTLGVVIVVFLLCVCPYYCFALSGHDSLISLSSVNILI
CVFYFNSCLNPLIYAFFYPWFRKSMKLIVTLKILQPGSCERNLL*
>StkTARs_042
MKSLEKDELCFPHFLNSSCRKALHPHFASMLTYMLLSSISLLTVTLNLLVIISIYHFRQLHTPTNLLLLSLAVSDFFVGLLMVFQIVLIDGCWFL
GDLMCIFYTFLDFIITSASVGTMVLISVDRYVAICEPLHYFNKVTQKRVKICVCLCWTWSAILHSVLFKDNLEEAGRYNSCIGECVIVINYIAGR
ANLFLTFIGPVTVIIVLYMRVFVVAVTQARAMRSHVAALPLKIAMTKTAKKSEMKAARTVGVIVVVFLVCLCPYYCVSLSEQDTLLSASSATIVV
RVFYFNSCLNPLIYAFFYPWFRKSMKLIVTLKILQPGSCERNLL*
>StkTARs_043
MKSLEEDEICFPHLLNFSCRKAVNPHFVFMLTYILLSSISLLTVTLNLLVIVSISHFRQLHTPTNLLLLSLAVSDFFVGLILFFQIMLIDGCWFL
GDLMCTLYIVLDYIVVSSSVGTMVLISVDRYVAICEPLHYSNKVTQKRVNICVCLCWTCSAFIMILMLQDHLKNRGRYTSCIGECVIVLNYVAEL
ADLFLTFIGPVTVIIVLYMRVFVVAVTQARAMRPHVAALPLKFSMNTTAKKSEMKAARTLGVVIVVFLLCVCPYYCFALLGQDSLINFSSANIVI
CLFYFNSCLNPLIYAFYYPWFRKSMKLIVTLKILQPGSCERNLL*
>StkTARs_044
MKSLEKDELCFPHLLNTSCRKAMRPHFASMLTYILLSSISLLTVTLNLLVIISIHHFRQLHTPTNLLLLSLAVSDFFVGLLMVFQIMLIDGCWFL
GDLMCTLYIVLDYIVVSSSVGTMVLISVDRYVAICEPLHYTNKVTQKRVKICVCLCWTCSAFVLILMLQDHLKRTDDISCIGECVIVVSYVAELA
QFFLIFIGPVTVIIVLYMRVFVVAVTQARAMRPHVAALPLKFSMTITAKKSEMKAARTLGVVIVVFLLCVCPYYCFALSGQDNLVNFSSVNILIC
VFYFNSCLNPLIYAFFYPWFRKSIKLIVTLKILQPGSCERNLL*
>StkTARs_045
METLVGTELCFPQLLNTSCRKKIRPHLETMLIYILLSSISLLTVALNLLVIISISHFRQLQTPTNLLLLSLAVSDFFVGLIMIFQIMIIDGCWLL
GDLMCSLWLILLSIITSSSVGTMVLISVDRYVAICYPLHYFTVVKPVRIQVCVCLCWMFAVLFNGLLLKDNLQHPGRYNSCIGECVFEINHIANL
FDLIVSFIFPITAIVILYIRIFGVAVYQARAMRSHIAVVTMKVNAKKSEMKAARNLGVVVVVFLICVLPYYCSALKSQNNLYTALSVTIVIWFFH
FNSCLNPLIYATLYPWFRKSIKVIVTLQILKPGSYQSNML*
>StkTARs_046
METLVGTELCFPQLINTSCRKPIRPHLEAMLIYILLSSISLFTVALNLLVIISISHFRQLHTPTNLLLLSLAVSDFFVGLFMIFQIMLIDGCWLL
GDLMCSLWSILSSIITSSSVGTMVLISGERYVAICYPLHYFIKVKQERVQVCVCLCWMFAALINSLLLKNNLQHPGRYKSCIGECTVDMNYIVNL
FDLIVTFIFPITVIVILYIRIFGVAVYQARAMRSHIAVVIMKVTAKKSEMKAARNLGVVVVVFLICVCPYYCVVLTSQNNLDTSLSVTIVICFFQ
FNSCLNPLIYATLYPWFRKSIKVIVTLQILKPGSYRTNML*
>StkTARs_047
METLVGTELCFPQLINTSCRKPIRLHLEAMLIYILLSSISLLTVALNLLVIISISHFRQLQTPPNLLLLSLAVSDFFVGLFMIFQIMMIDGCWLL
GDLMCSLWLILSSIIISSSVGTMVLISVDRYVAICYPLHYFTKVKPKRVKICVCLCWMFAALIYSLLLKNNLQHPGRYNSCIGECTVDINYIVNL
FDLIVTFIFPITVIVILHIRIFGVVVDQARAMRSHIAVVTRKVTVKKSEMKAARNLGVVVVVFLICICPYFCFTLKSQNNLYTATSVTIVMWLAQ
FNSCLNPLIYAILYPWFRKSIKVIVTLEILKPGSYRTNML*
>StkTARs_048
METLVGTELCFPQLINTSCRKTIHPHLEATLIYILLSSISLLTVVLNLLVIISISHFRQLQTPTNLLLLSLAVSDFFMGLFMIFQTMIEGCWLLG
DVMCYLWLILSSVVFLSSIETMVLISVDRYVAICYPLHYFTKVKRKSVQVCVCLCWMFAALINSVLLKNNLQHPGRYNSCIGQCVIDRNYIASIF
DLIVTFIFPITVIIILYMRIFVVAVYQARAMRSHIAVVTLKATVKKSEMKAARNLGVLVVVFLICICPYYCFVLTSQNNVYPTSSVTIVIWLSNC
NSCLNPLIYAILYPCFRKSIKVIVTLQILKSGSYRTNML*
>StkTARs_049
METLVGTELCFPQLINTSCRKTIHPHLEATLIYILLSSISLLTVVLNLLVIISISHFRQLQTPTNLLLLSLAVSDFFMGLFMIFQTMIEGCWLLG
DVMCYLWLILSSVVFLSSIETMVLISVDRYVAICYPLHYFTKVKRKSVQVCVCLCWMFAALINSVLLKNNLQHPGRYNSCIGQCVIDRNYIASIF
DLIVTFIFPITVIIILYMRIFVVAVYQARAMRSHIAVVTLKATVKKSEMKAARNLGVLVVVFLICICPYYCFVLTSQNNVYPTSSVTIVIWLSNC
NSCLNPLIYAILYPCFRKSIKVIVTLQILKSGSYRTNML*
>DanTAR_19l-001
MAYETEHQETQYCFPNFNSSCIKGKRSRHEYIIMYVFFSLLSAWTVFLNLLVIISISHFKKLHTPTNMIILSLAVNDLLIGLIVMPIQAIRLIET
CWYFGETFCGFYLILIGLIFSASLSNLVLIAVDRYVAVCHPLLYPQKITITNMLKSICLNWLYYSAYNTSFVVNNGSFDSSHRTEVCYGKCSVMM

SFSWIITDLFMSFIFPCLIMITLYSRIFYVVHQQVKVINSLMKGGKRVTESSVKRKSESKAALTLGIIVTIYLLFYIPYYICILFVNSSTTITVL
AWAVCANSGVNPLIYALYYSSFKKTVKHIFTLKIFQPASFLLCIFK
>DanTAR_20r-001
NKLLTGGDSLMAYETEDLETQYCFPDINSSCVKERRSSHRFVIIYLFVSLLSAWTVFLNLLVIISISHFKKLHTPTNMIILSLAVTDLLVGLVMP
VEAIRMIETCWYFGDTFCGLHLLFVSLLIAASLSNLVLIAVDRYVAVCHPLLYPQKITITKTLMSICLSWVCSSAYNTALLINNGYFGTSHRTDV
CYGECLFMSSFNWVVTDLFLSFIFPCLLIISLYLRIFYVVHQQVKVINSLMKGGKCVTEGLVKRKSESKAALTLGIIVLVYLLCYIPYFICSITV
NSSTTINGLLFAVCANSGLNPLIYALFYPWFKKTAKLILTLKIFHSASSLINIFTE
>DanTAR_18b-001
NKHLTGGDSLMAYETEDQETQYCFPEINSSCVKGKRSKHEYNIMYVFFSLLSAWTVFLNLLVIISISHFKKLHTPTNMIILSLAVADLLVGLVVM
PVEAIKLTETCWYFGDTLCDLFMIIMGLTVSASICNLVIIAVDRYVAVCHPLIYPQKITTTKTLISISLCWFCSLTYNAGYLISGRYFEINQSPG
TCYGKCFLVISFSWVFTDLLFSFLLPCTLIITLYLRIFYVAHQQVKIINSMMKGGNLEASVKRKSESKAALTLGIIVTVYLLCYIPFYILYMLSV
TGTTIISSTTMTFLIWTLYINSSVNPLVYALFYSWFKISVKHILTCKIFSP
>DanTAR_19t-001
NKLLTGGDSLMAYETEDQETQYCFPDINSSCVKEKRSIQEYNIMYVFFSLLSAWTVFLNLLVIISISHFKKLHTPTNMIILSLAVNDLLIGLILM
PVEAIRLIETCWYFGETFCVLYLILIGLIFSATLSCLVLIAVDRYVAVCHPLLYPQKITITNMLMSICLSWVCYSAYNTAFAFNNGYLNSSHRRD
VCYGQCFIMMTFSWTVTDLSMCFIFPCVIMITLYSRIFYVVHQQVKVINSLMKGGKCETEGSVKRKSESKAALTLGIIVSVYLLCYIPYYICTLL
VSSSTTITVLMWTVHANSGLNPLVYAIFYPWFKKTAKLILTLKIFQPASSLINIFTEN
>DanTAR_20g-001
NKLLTGGDSLMAYETEDQETQYCFPDINSSCVKGRRSSNGYIIIYVFVSLLSAWTVFLNLLVIISISHFKKLHTPTNMIILSLAVNDLLVGVVMP
VEAIRMTETCWYFGDTFCGLYLFFVSLLLAASLGNLLLIAVDRYVAVCHPLLYTQKITITKMLTSICLCWACFSTYCTTFVIDNRNFDTSHRTDV
CYGECLVMISLSWLVIDLFMSFIFPCTLIILLYLRIFYVVHQQVKVINSLMKRGKCVTEGSVKRKSESKAALTLGIIVSVYLLCWIPYCICSLIV
NSSTALNVIIWTVYANSGVNPLVYALFYPWFKKTGKLILTLNILQPASSVINIFTQN
>DanTAR_19b-001
NKLLTGGDSLMAYETEDQETQYCFPDINSSCVKEQHSTNGYIIYLFVSFLSAWTVFLNLLVIISISHFKKLHTPTNMIVLSLAVADLLIGLIVMP
VEAVKLIETCWYFGDTFCGFTLILIGVIPSASLGNLVLIAVDRYAAVCHPLLYPQKITMANMLMSICLSWVYYSTYITTLVIYNRYFDTSHRTDE
CYGQCLVMMSFSWILTDLFVCFVFPCVLIITLYLRIFYVVHQQVKVINSLMKGGKCVTEGSVKRKSESKAALTLGIIVSVYLLCWIPYYICTLTV
NSSTVLSVLTWVLYANSGLNPLVYALFYPWFKKTVKHILTMTICQLASSLVNIFTEHE
>DanTAR_18j-001
NKLLTGGDSLMAYETEDTQYCFPDINSSCVKGQRTRLEYNIMYVFVSLLSAWTVFLNLLVIISISHFKKLHTPTNLLILSLAVADLLVGLVVIPI
EGIKQIETCWYFGDTYCGLYVMTVRLLLSTSLINLVLIAVDRYMAVCHSLLYPQTITTTKTLISIFLCWFCCSVYIISSVISNGYFDMSNKTLCF
GQCIVLITPAWRFIDLICSFLFPCTVIITAYLRIFYVVHKQVKVINSQINGVKCVMEVSVKRKSESKAAQTLGIIVTAYLLCWIPFYICSLTKTT
AISATTMTFLTWTFYINSGLNPIVYALFYSWFKISVKHIVTFRIFQPASSLMNILTE
>DanTAR_16a-001
MKDKNLEIQFCFPNNNLSCFKEIKPQTDYVVLYIFIFLASVVTVFLNLLVIISISHFRQLHTPTNLLILSLAVADLIVGLIVIPFMGIRFIDSCW
YYGETFCSVFLFITFAVVSGSLGNLVLISLDRYIAVSDPLRYTVKITTDRIVICIVVNWLCSSIYSFIFLYNSVFYPETQNGCYGDCRVSFKFEH
FVTDLLVTFVAPSCVIMSIYVKIFCVAKHQAKVVNSVTGVSKSQRKAGKTLGIVVMVFFMCWIPYYIVILIEGNELTESVEFNVTCWIVYMNSCM
NPLIYSLFYKWFRISAKHILTLNILKLSSEYFNLFPE
>DanTAR_18d-001
NKLLTGEDSLMAYETEDQETQYCFPDINSSCVKEQRSIQEYIIMYVFFSLLSAWTVFLNLLVIISISHFKKLHTPTNMIILSLAMADLFTGLTVM
PVEAIKLIETCWYFGEKFCDIFMIIVGLFGSTSFSNIVLISVDRYLAVCHPLIYPHKFTRSKALISICVCWLFALIYNLGFLLTGKYFQSSERTD
MCYGKCNLVIISSSWAFTDLFVSFLLPCTLILILYLRIFYVAQQQVKVINSLMKGGKCVKEGLVKRKSENKAAFTLGIVVTVYLLCFIPYYILSV
TGVCSATMTYLLWTLYVNCGLNPLIYALFYRWFKISVKHILTLKILQPASSLLDIFTD
>DanTAR_19q-001
NKLLTGVDSLMAYETEDQETQYCFPDINSSCVKGKRSIQEYIIMYVFFSLLSVWTVFLNLLVIISISHFKKLHTPTNMIILSLAVADLLIGLIVM
PIKAIRQVETCWYFGDTFCGLNSILIGAIFSATLSNLVLIAVDRYVAVCHPLLYPQKITMAKMLMFICLCWIYYSAYNTAVVMNNRYFDTSNRTD
MCYGDCSVIMSFGWILTDLFMSFILPCLIMISLYLRIFYVVHQQVKVINSLMKGGKCVTEGSVKRKSESKAALTLGIIVLVYLLCYIPYYICALS
VNSSTTITVLEWTVYTNSGLNPLVYALFYHWFKKTAKLILTLKIFHPESSLINVFTENE
>DanTAR_14i-201
MCEDYSCPERSVSLSVYVILYVAAAAVALLTVCGNLLVIISVSHFKQLHTPANILILSLAASDLLVGVFVMPFQLTLLIESCWIAGPVMCAVLKF
VNFQATSVSVHTVALIAVDRFLALSFPFFYSEKISPTVICIAALLNWLFSLFYNFRLLYVNGNFTDIVCPGVCVYVVDEISSFVDLLIVFVMPCT
LIIILYTHVFVIAKKHATAIRAVQVHNSTESSKNKISDKSERKAAMLLGILVFVFLLCLLPYYTFLVISYSIVDFIFFRDVGVIIFFLNSTINPI
IYALFYPWFKKSLRIIFTFKVFQRDSSLMNVQSY
>DanTAR_14j-201
MCEDYSCPERSVSLSVYVILYVAAAAVALLTVCGNLLVIISVSHFKQLHTPANILILSLAASDLLVGVFVMPLLFTLLIESCWIAGPVMCAVLKF
VNFQATSVSVHTVSLIAVDRFLALSFPFFYSEKISLTVICIAALSNWLFSLFYNFTVLYINRHFTGFMCPGVCFFVIDEVSSLIDLLIVFVMPCT
LIIILYTHVFVIAKKHATAIRALQVHNSTESSKNKISDKSERKAAMLLGILVFVFLLCLLPYYITSLVISYTTVDLLHVRDVAVIFFFVNSTINP
IIYALFYPWFQKCLKLIFTLKVFERDSSLMNVQCY
>DanTAR_19p-001
NKLLTGGDSLMAYETEHQDTQYCFPNINSSCVKGKRSIQEYNIMYVLFSLLSAWTVFLNLLVIISISHFKKLHTPTNMIILSLAVTDLLIGLIVM
PIQAIRLIETCWYFGDTLCGINLIIIGVIFSASFSNLVLIAVDRYVAVCHPLLYPQKITMAKMLMCICLSWVYYSAYNTGLVINNGYFDTSHRTD
VCYGDCSVIMSFSWLVTDLCLSFIFPCVIMITLYLRIFYVVHQQVKVINSLMKGGKCVTKGSVKRKSESKAALTLGIIVLVYLLCYIPYYICTLS
VNSSTTITVLEWTVYTNSGLNPLVYALFYHWFKKTVKLILTLKIFHPESSLINVFTVHEL
>DanTAR_19u-001
NKLLTGGDSLMAYETEDQETQYCFPDINSSCVKGKRSRHEYIIMYVFFSLLSAWTVFLNLLVIISISHFKKLHTPTNMIILSLAVNDLLIGLFVI
PIQAIKLTETCWYFGDIFCVLYLILIGLIFSASLSNLVLIAVDRYVAVCHPLLYPQKITITNMLKSICLSWVCYSAYNTAFVVNNGYFDSSHRTD
MCNGRCSVMVSFSWTVIDLFMCFIFPCLIMITLYLRIFYVVHQQVKVINSLMKGGKRVTESSAKRKSESKAALTLGIIVTIYLLCYIPYYICSLF
VISSTTITVLSWAVHANSGLNPLVYALFYSSFKKTVKHIFTLKIFQPVSFLFRIFK
>DanTAR_20b1-001
NKLLTGGDSLMAYVTEDQETQYCFPNINSSCVKKKHSRNGYHIIYVFVSLLSAWTVFLNLLVIISISHFKKLHTPTNMIILSLTVNDLFIGLIMP
VEAIRLIETCWYFGDTFCGLHILFISLLVSASFSNFVLIALDRYVAVCHPLQYPQKITITKTLMSICLSWLCLSAYITAYVFNNGYFDSSHRTDV
CYGQCSVMISFSWSVTDLIISFIFPCVLIITLYLRIFYVVHQQVKVINSLMKGGKCVTEGSVKRKSESKAALTLGIIVSVYLFCYIPYYICSLTV
NATTIINVLLLLVYANSGLNPLVYALFYPWFKRTAKVILTLKIFQSASSLINIFTQN
>DanTAR_19n-001
NKLLTGGDSVMAYETEDQKTQYCFPNINSSCVKEKRSIREYIIMYLFSSLLSVWIVFLNLLVIISISHFKMLHTPTNMLILSLAVADLLIGLILM
PLQAIKQIETCWYFGETFCGIHLIIIGLIFSATFSNLVLIAVDRYVAVCHPLLYPRKITTAKMLMSILLCWLYYSAYNTGLVINNGYADTSYRTD
MCYGKCTVIMSFSWLVTDLFMSFIFPCLIMITLYLRIFYVVHQQVKVINSLMKGGKCVTEGSVKRKSESKAALTLGIIVTVYLLCYIPYYICALS

VNSVTAINVLGWIVYANSGVNPLVYALFYPWFKKTAKIILTLKIFEPASSLINIFK
>DanTAR_19n-201
YETEDQKTQYCFPNINSSCVKEKRSIREYIIMYLFSSLLSVWIVFLNLLVIISISHFKMLHTPTNMLILSLAVADLLIGLILMPLQAIKQIETCW
YFGETFCGIHLIIIGLIFSATFSNLVLIAVDRYVAVCHPLLYPRKITTAKMLMSILLCWLYYSAYNTGLVINNGYADTSYRTDMCYGKCTVIMSF
SWLVTDLFMSFIFPCLIMITLYLRIFYVVHQQVKVINSLMKGGKCVTEGSVKRKSESKAALTLGIIVTVYLLCYIPYYICALSVNSVTAINVLGW
IVYANSGVNPLVYALFYPWFKKTAKIILTLKIFEPASSLINIFNPIHR
>DanTAR_19a-001
NKLLTGGDLLMAYETEDQETQYCFPDINSSCVKGRHSSHGYIIIYLFVSLLSVWTVFLNLLVIISISHFKKLHTPTNMIILSLAVNDLIIGLFVI
PIQAIKLIETCWYFGDTFCGLYLFLIFELLSASLSNLVLIAVDRYVAVCHPLLYPQKITITKTLMSICLSWVCYSAYNTALIINNRYSETSHRTD
ECYGRCFIVMSFSWLVADLVMSFIFPCLIMITLYLMIFYVVHQQVKVMNSLMKGGKCVTEGSVKRKSESKAALTLGIIVTIYLLCYIPYYICSLV
VNSSTAIHVMSWTVYVNSGMNPLVYALFYPWFKKTAKIIFTLKIFQPASSLINIFTE
>DanTAR_18c-001
NKLLTGGDSLMAYETEDQETQYCFPNINSSCVKERRSKHEYNIMYVFFSLLSAWTVFLNLLVIISISHFKKLHTPTNMIILSLAVADLLVGLVVM
PVEAIKLTETCWYFGDTLCDLFMIIMGLTVSASLCNLVIIAVDRYVAVCHPLIYPQKITSTKILISISLCWFCSLTYNAGYFISGRYFKINQSPG
TCYGKCFLVISFSWVFTDLLFSFLLPCTLIITLYLRIFYVVHQQVKIINSMMKGGNLEASVKRKSESKAALTLGIIVTVYLLCYIPFYILCMLSV
TGTTIISSTTMTFLVWTLYINSSVNPLVYGLFYSWFKISVKHILTCKIFSP
>DanTAR_16f-001
NTNNETIQYCFPNNNLSCTRNVKPGAEYIFVYIFISTVSVLTVFLNLLVIISIAHFHQLHTPTNVLIFSLAMSDLIVGLILMPVLGLKLIEPCWY
FGEIFCSIFPFILYVVTTASLGNLIIISVDRYIAVNDPLRYTTRVDTNRAVYSIVVNWVFSSIYSLLIMYEAILYPEKNHTCIGECIFFIKLEYI
ITDALVCFVTPCCLIIYLYLKICFIAKHQAEHINALTDRKVKSEKKAAKSLGVVVMIYLLCWIPYYIAALTLGQDTNDSLVINIMYWILCMNSLM
NPLIYAMYYKWFRISTKYILTLKILEPSSQYFNLFME
>DanTAR_19j-001
NKLLTEGDSLMAYETEDQETQYCFPNNNSSCIKEQRSTHEYNIMYVFFLLLSAWTVFLNLLVIISISHFKKLHTPTNMIILSLAVADLFVGLIVI
PIEAIKQIEKCWYFGDTFCGLNVILTAVLLSASLSNLVLIAVERYVAVCHPLLYPQKITITNILLSICLSWVYYSAYNTVLVVNNGYFGTLHRNK
VCYGECLVIISFSWMLTDLFMSFIFTCIIMITLYLRIFYVVHQQVKVINSLMKGGKCVTEGSVKRKSESKAALTLGIIVLVYLLCYIPYCICSVT
VNSSTTINVLMWILYANSGLNPLVYAIFYPWFKKTVKHILTLKIFEPASSLINVFIEDE
>DanTAR_20i-001
NKLLTGGDSLMPYETEDQETQYCFPNINSSCVKEQRSSHGYIIIYVFVSLLSAWTVFLNLLVIISISHFKKLHTPTNMIILSLAVNDLLIGLAMP
MEAIRMIQTCWYFGDTFCGIYILFVSILLAASLGNLLLIAVDRYVAVCHPLFYPRKTTMAKILITICLNFACLSAYNTAFVINNGYFDTSHRTDV
CYGQCSVMIRFSWIVTDLFMSFIFPCTLIMMFYLRIFYVVHQQVKVINSLMKGGKCVTEGSGKRKSESKAALTLGIIVSVYMLCWIPYYICSLTV
NSYTAINVLIFGVHASSGLNPLVYALFYPWFKKTAKLILTLMIFQPASSLINVFTHE
>DanTAR_19s-001
NKLLTGGDSLMAYETEDQETQYCFPNINSSCVKGRRYRHEYIIMYVFFSLLSAWTVFLNLLVIISISHFKKLHTPTNMIILFLAVNDLLIGLIVL
PVEAIRLIERCWYFGETFCFLYLILIVLIFSASLSYLVLIAVDRYVAVCHPLLYPQKITITNMLKSICLSWVCYSAYNIAFVINNRNLNSSQKTD
VCYGQCLIMMSFSWTVIDLSICFIFPCLIMITLYLRIFYVVHQQVKVINSLMKGGKCETEGSVKRKSESKAALTLGIIVSVYLLCYIPYYICTLL
VSSSTTINVLMWNVHANSGLNPLVYAIFYPWFKKTAKLILTLKIFQPASSLINIFTENE
>DanTAR_19c-001
NKLLTGGDSLMAYETEHQETQYCFPDINSSCVKEKHFSHGYNILYIFVSLLSVWTVILNLLVIISISHFKKLHTPTNMIILSLAVTDLLIGLIVM
PVEAVKLIDKCWYFGDTFCGLTLILIWVIPSASLSNLVLIAVDRYMAVCHPLLYPQKITMANTLMSICLSWVYYSAYNTALLFDNGYFDTSHRTD
VCYGDCSVMMSFSWRVTDLFMCFIFPCVMIITLYLRIFYAVHQQVKVINSLMKGGKCVLKGSVKRKSESKAALTLGIIVTVYLLCWIPYYICTLT
VNSSTVISVLTWIVYANSGLNPLVYALFYPWFKKTVKLILTLKIFHLASSLVNIFTHHE
>DanTAR_20c1-001
NKLLTGGDSLMAYETEDQETQYCFPDINSSCVKEQRSSHGYIITYVFVSLLSAWTVFLNLLVIISISHFKKLHTPTNMIILSLAVNDLLVGLVMP
IEAIRLIETCWYFGDTFCGLYLLFVSLLLATSIGNLVLIAVDRYVAVCHPLLYPQKITITKTLMSICLSWACSSAYIIAILINNSYFDTSHRKDK
CYGECLLMITFSWFVTDLFMSFIFPCTLIMMLYLRIFYVVHQQVKVINSLMKHGKCLKEGSVKRKSESKAALTLGIIVSVYLLCYIPYCICSLTV
NSVTAMNVLIWIVYSNSGVNPLVYALFYPWFKKTAKHILTLKIFQLTSSLFNVFTEH
>DanTAR_20a-001
NKLLTGGDSLMAYETEDQETQYCFPNINSSCVKEQHSSHGYIIIHLFVSLLSAWTVFLNLLVIISISHFKKLHTPTNMIILSLAVNDLFIGIAMP
VEAIRLIETCWYFGDTFCVLYLFFVALLLSASLGNLVLIAVDRYVAVCHPLLYTQKTTIAKIFCLSICLSCVCFSAYFTALVNVNNQDFDTSQRT
DVCYGQCLVMITFSWIVIDLFVSFIFPCTLIMMFYLRIFYVVHQQVKVINSLMKGGKCVTEGSVKRKSESKAALTLGIIVTVYLLCYIPYYICSL
TVNSSTAINVLVWLVYSNSGMNPLVYAIFYPWFKKTAKLILTLKIFQPASSLINIFTE
>DanTAR_20m-001
NKLLTGGDSLMAYETEDQETQYCFPDINSSCVKEQRLRNRFKITYLLVSLLSAWTVFLNLLVIISISHFKKLHTPTNMIILSLAVTDLLIGFIMP
IEAIRLNDKCWYFGQIFCGLYLLFVSLLLSTSLGNLVLIAVDRYVAVCHPLLYPQKITTAKMLMSICLNWICISAYNTTLVVNNGHFDTSHRIDE
CHGECLFMISFSWIVIDLCISLIFPCTLIMVLYLRIFYVVHQQVKVINSLMKGDKCVTEGSVKKKSEIKAALTLGVMVSVYLLCYIPYYICSLTV
TSSTTLNVLIWTLHANSGLNPLVYALFYRWFKKTAKLILNLKIFLPASSLINIFSQHE
>DanTAR_20h-001
NKLLTGGDSLMAFETEDQETQYCFPDINSSCVKRRSLSHGYITIYVFVSLLSAWTVFLNLLVIISISHFKKLHTPTNMIILSLAVNDLFIGLAMP
VEAIRLIETCWYFGDTFCGLYLLFVSILLSASVGNLLLIAVDRYVAVCHPLLYPQKITITNMLMSICLNWFCLSAYCITFVIKNRDFDTPHRTAY
CYGECFLMISFSWIITDLFMSFIFPCTLIMMFYLRIFYVVHQQVKVINSLMKRDKCVKEGSVKRKSESKAALTLGIIVTVYLLCYIPYCICSLTV
NSSTALNVIVWFLYANSGVNPLVYALFYTWFKKTAKLILTLNIFQPASSLINIFTQN
>DanTAR_18i-001
NKLLTGGDSLMAYETEDQETQYCFPNINSSCVKGKRSKHEYNIFYIFVLLMSAWTVFLNLLVIISISHFKKLHTPTNLLILSLAVADLFVGLVVI
PTEGIKQIETCWYFGDTFCGLFVIIVRLLLSASLSNLVLIAVDRYIAVCYSLQYPQRITKTKTVISICLCWFSCSVYNIWYVIGNGYFSMSDKTL
CYGQCMVVITPTWRFIDLIFSFLFPCTVIITAYVRIFYVVHKQVKVINSQIKGVKCVMEVSIKRKSESKAALTLGIIVTAYLLCWIPFYICSLTK
ITTITSTTMTLLVWTFHLNSGLNPIVYALFYRWFKISVKHIVTLRIFQPASSLMDIFTD
>DanTAR_19e-001
NKLLTGGDSLMAYETEDQETQYCFPNINTSCVKENRSKHEYNVLYVFFSLLSAWTVFLNLLVIISISHFKKLHTPTNMIILSLAVNDLLIGLFVM
PVEAVRLIDKCWYFGDTFCGFTLILIGVIPSASLSNLVLIAVDRYAAVCYPLLYPQKISMTNTLMSICLSWVYYFAYITALVIYNRYLDTSHRTD
ECYGECTIMMSFSWIITDLFMCFIFPCTIMITLYLRIFYVVHQQVKVMNSLMKGGKCVTEGLVKRKSESKAALTLGIIVSVYLLCWIPYYICSLT
VISSTAINVLIWAVFANSGVNPLVYALFYPWFKKTAKLILTLKIFQPASSLINIFTELE
>DanTAR_20o-001
SKLLTGGDSLMAYETEDQETQYCFPDINSSCVKEKHSRHGYITTYLFVSLLSAWTVFLNLLVIISISHFKKLHTPTNMIILSLAVTDLLVGLVMP
VEAIRLIETCWYFGDTFCGLYIFFVSLLLSASLGNLVLIAVDRYVAVCHPLLYPQKITITKTLMSICLSWAGLTAYIITLVINNSYFDSSHRTDV
CYGQCLYMISYSWILTELFMSFIFPCVLIITLYLRIFYVVHQQVKVINSLMKGGKCVTEGSVKRKSESKAALTLGIIVSVYLLCWIPYFIYSLTV
NSSTTINGLLFAVFANSGLNPLVYALFYPWFKKTAKLILTLKIVHSASSLINIFTEH

>DanTAR_20a1-001
NKLLTDRDSLMAYETEDQDTQYCFPDINSSCVKGKRFSHGYIIIYLFVSLLSAWTVCLNLLVIISISHFKKLHTPTNMIILSLAVNDLFIGLVMP
VEAIRLIETCWYFGDTFCAIHVLFVSFLVSASLSNLVLIAVDRYVAVCHPLQYPQKITITKTLMSICLSWVCLSAYITAFVIYNGYFDYSHRTDV
CYGQCSVMMNLSWTVTDLFMSFIFPCLIIITLYLRIFYVVHQQVKVINSLMKGGKCVTEGLVKKKSESKAALTLGIIVTVYMFCYIPYYLYVLTV
TSSTTINVLLLLLYANSGLNPLVYALFYPWFKRTAKLILTLKIFQSASSLINIFTQH
>DanTAR_17a-001
NKLLTGAESLMAYETEETQYCYPAFNSSCVKGKRSNYESNILYVFFSLLSAWTVFLNLLVIFSISHFKKLHTPTNMIILSLSVADLLVGLIVMPI
EATELIETCWYFGDTLCNLFLILMGLLTSTSLCSLVLIAVDRYVAVCHPLMYPQTITMNRTLIIVCFSWFFSSAYNIILSITHTSDGCYGECNVQ
LTFEWKATDLVLSFLLPCTVIITLHLRIFYVVHQQVKVINSQMKSEKCVMEGSVKRKSESKAALILGIIVAIHLICFITYYILTLTDSMAIPPTV
LSCLICFLYINSSLDPLLYALFYTWFRKSIKHILTLKIFQPESSQVNILTNH
>DanTAR_16g-201
AEFIFVYIFISTVSVLTVFLNLLVIISIAHFHQLHSPTNVLIFSLAMADLIVGLILMPVHGLKLIEPCWYFGEIFCSIFPFIFYVVVTASLGNLI
IISVDRYIAVNDPLRYTTRVDTNRAVFSIVVNWVFSSIYSFLIFKFESWLADLSNFTSCLKKNHTCIGECIFFIKLEYIITDALVCFVTPCCLII
YLYLKICFIAKHQAEHINALTDRKVKSEKKAAKSLGVVVMIYLLCWIPYYIAALTLGQDTNDSLVINIMYWILCMNSLMNPLIY
>DanTAR_20f-001
NKLLTGGDSLMAYETEDQETQYCFPAINSSCVKERSASHGYIIIYVFVSLLSAWTVFLNLLVIISISHFKKLHTPTNMIILSLAVNDLFIGLIIP
IEAIRQIETCWYFGDTFCGLYLLFVSLLLAASLGNLVIIAVDRYVAVCHPLLYPQKITITNMLKSICLSWVCFSAYCITFIINNGSFDTSHRTDV
CYGQCLMIISFSCLIIDLFMSFIFPCLLIITLYLRIFYVVHQQVKVINSLMKAGKCVTEGSVKRKSESKAALTLGIIVSVFLLCWIPYYICSLTV
NSYTAINVLIFGVHASSGLNPLVYALFYPWFKKTAKLILTLKIFQPASSLINIFLQ
>DanTAR_20z-001
KLLTGGDSLMAYETEDQETQYCFPDINSSCVKGRSASHGYIIIYVFVSLLSAWTVFLNLLVIISISHFKKLHTPTNMIILSLSVADLLVGFIMPV
EAIRLTETCWYFGDTFCSLYLLYIAVLLSASLSNFVLIALDRYVAVCHPLLYPQKITITKTLMSICLSWACFSAYNITFVIYNGYFDTLYKTDVC
YGQCSFIMSFHWIVTDLFMSFIFPCFLIITLYLRIFYVVHQQVKVINSLMKAGKCVTEGSVKRKSESKAALTLGIIVSVYLLCWIPYYICTLTVS
SSTIINVLTWVVYANSGLNPLVYALFYPSFKKTFKHILTLKILHSASSLINIFTE
>DanTAR_20z-201
LHASSLSPIFTAPLMNIKLKVCSAWTVFLNLLVIISISHFKKLHTPTNMIILSLSVADLLVGFIMPVEAIRLTETCWYFGDTFCSLYLLYIAVLL
SASLSNFVLIALDRYVAVCHPLLYPQKITITKTLMSICLSWACFSAYNITFVIYNGYFDTLYKTDVCYGQCSFIMSFHWIVTDLFMSFIFPCFLI
ITLYLRIFYVVHQQVKVINSLMKAGKCVTEGSVKRKSESKAALTLGIIVSVYLLCWIPYYICTLTVSSSTIINVLTWVVYANSGLNPLVYALFYP
SFKKTFKHIL
>DanTAR_20u-001
NKLLTGGDSLMAYETEDQETQYCFPDINSSCVKRRSSSHGYIIIYVFLSLLSAWTVFLNLLVIISISHFKKLHTPTNMIILSLAVTDLLIGLIMP
VEAIRLIETCWYFGDTLCELYLLFVDLLISTSLSNLVLIAVDRYVAVCHPLLYPQKITITKTLMSICLSWVCLSAYIIAIAINNRYFDTSHRTDV
CYGECLAIISFSWTVTDLFMSFIFPCTVIIILYLRIFYVVHQQVKVINSLMKGGKCVTEGSVKRKSESKAALTLGIIVTVYLLCWIPYYICSLTV
MTSTALNALLWVVYASSGLNPLVYALFYPWFKKTAQLILTLKIFQTASSLINIFTQ
>DanTAR_19h-001
NKLLTGGDSLMDYETEDQETQYCFPDINSSCVKYQRSSHEYNIMYVFFYFLSAWTVFLNLLVIISISHFKKLHTPTNMIILSLAVNDLLVGLIVM
PIQAIRLIETCWYFGDTFCGISLILSGVIISASLTNLVLIAVDRYVAVCHPLLYPQKITITNMLLIICLSWIYYSGYNTVLIIYNGYFETSERTD
ACDGDCLVMIRFSWIVTDLFISFIFPCVIIITLYLRIFYVVHQQVKVINSLMKGGKCVTEGSVKRKSESKAALTLGIIVSVYLLSWIPYCICSVT
VNSSTLITILSWTVYTNSGLNPLVYAIFYPWFKKTVKVILTLKIFDPASSLVNIFMEHD
>DanTAR_19g-001
TEGDSLMAYETEDQETQYCFPDINSSCVKKQRSSHEYNFMYLFFPLLSAWTVFLNLLVIISISHFKKLHTPTNMIILSLSVADLLIGLVMPIEAI
RLIETCWYFGDTFCGFNLIIIGAILSASVSNLVLIAVDRYVAVCYPLLYPQKITISNMLISICLSWVYYSAYNTVLIINNGYFDTSYRTDMCYGQ
CSAMMSFTWILTDLFVCFTFPCVIIITLYLRIFYVVHQQVKVINSLMKGGKCVTDGSVKRKSESKAALTLGIIVSVYMLCWIPYYICALSVNSST
VLSVLTWVVYANSGLNPLVYALFYPWFKKTVKVILTLKIFEPASSLVNIFIENE
>DanTAR_18g-001
NKLLTGGDSLMAYETEDQETQYCFPNINSSCVKERRSSHGYIIYLFVSLLSAWTVFLNLLVIISISHFKKLHTPTNMIILSLAVNDLLIGLAMPI
EAFRLVDTCWYFGELFCGLFLIVMALLSSASLSNLVLIAVDRYVAVCHSLLYPQKITMTNVFVSICLCWSFSLTYCTVFVVNNTYLDTSSRKRVC
YGECILHFSFTYVIIDEIYSFLLPCVVMITVYLRIFCVAIKQVKVINSLMKGGKCVKEGSVKRKSEHKAALTLGIVVTVYLLCYIPYYLISLTGV
YSKTITYLLWTLYVNSGLNPLIYALFYRWFKTSVKHILTLKILQPASSLLDIFT
>DanTAR_19k-001
NKLLTGGDSLMAYETEDQENQYCFPDINSSCLKEKRSSHGYNIIYLCVSLQSAWTVFLNLLVIISISHFKKLHTPTNMIILSLAVADLLLGVVMP
VEAIRLIEMCWYFGDIFCGLYLLFISFLISASLSNLILIAVDRYMAVCHPLQYPQKITISKTLMSICLSWVYSTVYNSTLVISNGHFDTSPRTDV
CYGQCSFDMSFSWIAVDMFMSLIFPCMLIITLYLRIFYVVHKQVMVINSLMKGGKCVVRGSVKRKSESKAALILGIIVSVYLLCYIPYCICSLTV
KSSTAIHSLIWAVYSNSGLNPLIYALFYPWFKKTAKLILTLKIFQPASSLVNIFTQHQL
>DanTAR_20j-001
NKLLTGGDSLMAYETEHQETQYCFPDINSSCIKERRSSHGYIIIYVFVSLLSAWTVFLNLLVIISISHFKKLHTPTNMIILSLAVTDLLIGLVMP
VEAIRLIDKCWYFGNTFCGLYMLFVSLLVTVSLGNLVLIAVDRYVAVCHPLLYPQRITITKTLVSICLNWVCFSAYCTALIINNSFFDTQTDVCY
GQCLVMVSFGWIATDLFISFIFPCLLIISLYLRIFYVVHQQVKVINSLMKGGKCVTEGSVKRKSESKAALTLGIIVSVFLLCYIPYYICSLTVNS
STALNVLVWVLYANSGLNPLVYALFYSWFKKTAKLIITLKILHPASSLINFVSQH
>DanTAR_19i-001
NKPLTGEYSLMDYETEDQETQYCFPDINSSCVKEQRSIHEYNIMYVFFSLLSAWTVFLNLLVIISISHFKKLHTPTNMIILSLSLNDLLVGLIVM
PIQAIRLIETCWYFGHTFCGISLILIGVIISASLSNLVLIAVDRYVAVCHPLLYPQKITISNMLMSICLSWVYYSVYNTALIIYNGYFETSERTD
VCYGDCLVMISFSWIVTDLFISFFSPCVIMITLYVRIFYVVHQQVKVINSLINGGRCVMESSVKRKSESKAALTLGIIVSVYLLSWIPYCICSVT
VNSSTAITVLSWTVYANSGLNPLVYAIFYPWFKKTVKNILTLKIFEPASSLVNIFMEHD
>DanTAR_20p-001
NKLLTGGDSLMAYETEDQETQYCFPDINSSCVKGKNSSHGYIVIYLFVSLLSAWTVFLNLLVIISISHFKKLHTPTNMIILSLAVNDLLIGLFMP
VEAIRLTETCWYFGDTFCGLHLLFVSLLLAASLSNLVLIAVDRYVAVCHPLLYPQKITMTKTLMSICLNWVCLSAYIIALLTNYGYFDTSLKTDV
CYGECLFMSSFSLVMTDLFMSFIFPCFLIITLYLRIFYVVHQQVKVINSLMKVGKCVMEGSVKRKSESKAALTLGIIVSVYLLCWIPYFICSLTV
NSSATINSLLFAVYANSGLNPLVYALFYPWFKKTAKLILTLKIFHSASSLINIFTEH
>DanTAR_19d-001
NKLLTGGDSLMAYETEHQETQYCFPDINSSCVKEQRSTHGYIIICVFVSLLSVWTVFLNLLVIISISHFKKLHTSTNMIILSLAVNDLLIGLIVM
PVEAVRLIDKCWYFGETVCGFILILIWVIPSASLSNLVLIAVDRYVAVCHPLLYPQKITMTNTLMSIGLSWLCYSAYISALIIYNRYFDNSHRTD
VCYGQCLIMMSFSWIVTDLFMCFIFPCLIMITLYLRIFYVVHQQVKIINSLMKRGKCVTEGSVKRKSESKAALTLGIIVLVYLLCWIPYYICTLT
VNYSTAVSILTWIVYANSGMNPLVYALFYPWFKKTTKLILTLKIFQLASSLINIFTEHE
>DanTAR_20n-001
NKLLTGGDSLMAYETEDQETQYCFPDINSSCVKEKHSSNGYIIIYLFASLLSAWTVILNLLVIISISHFKKLHTPTNMIILSLAVNDLFIGLVMP

VDAIRVIETCWYFGDTFCGLYLLFISLLLPASLGNLVLIAVDRYVAVCHPLLYPQKISISKAFMSICLNWVCLSAYNTAFIINNGFLDTSQRTDV
CYGQCSFTVSFSWMLTDLFVSFIFPCMLIITLYLRIFYAVHQQVKVINSLMKGGKCVMKSSVKRKSERKAALTLGIIVSVYLLCYIPYCICSLTG
VSASTKNVLMWILYANSGVNPLVYALFYPWFKKTAKLILTLEIFQSVSSLINIFTEN
>DanTAR_18e-001
KLLTGGDSLMAFETEDQETQYCFPDINSSCVKGKRSRHEYNIMYVFFSLLSAWTVFLNLLVIISISHFKKLHTPTNMIILSLAVNDLLIGLIIMP
LEAIKLIETCWYFGETFCGLFMIIMGLLGSTSLSNLVLIAVDRYVAVCHPLLYPQKITMSKTLVTICLCWLFSSAYCSVFVVSNKYFDSSNRTDV
CYGMCTLNLSFTYIIVDLIYSFLLPCVVMITVYLRIFYVAIKQVKVINFLMKGRRESSVKRKSEHKAALTLGIVVTVYLLCFIPYYLLSVMGVSS
GTLTYLLWTLYANCVVNPLIYALFYRWFKKSVKHILTFKILEPASSLLDIFTD
>DanTAR_10a-001
MDDHINISQTESWEKPLLCYEFSNRSCQKFVYPLDTRILLYMLFSISSIITIIGNLLVIITVVHFRQLHTPTNYLILSLAVADLLVGGVVMPPSM
LRSIETCWYLGDLFCKIHSSLDVTLCTASILNLCIISLDRYYAICHPFQYHSKMTSLATLIMIIICWTVSAVLGFGMIFMELNILGVEDFYYENI
RCDGGCFVFQSKTGGTVFSLICFYIPAFVMLGVYLKILHEAQRQVQAIQSVNSELKKEGKATKTLAIIMGVFLTFWIPFFLCNLIDPLIGYSVPS
LVFDLFLWVGYYNSTCNPIVYAFFYSWFRHAFRVILSKRVFQTNSSRTILM
>DanTAR_15-001
MEFQEHHCFSHWNTSCSLDFHLSYVNVFLFLYISAISVLTVCGNLLVIVSITVFKQLHTPTNLLILSLAISDLLVGICLMPVESIRSINSCFYMG
KSHCHIFHSIMSIVGSASLMNIMLVAIDRYFAVCNPLLYMSKMTIRKALICVCLGWTVSICYNLVPVNLGNSDPTGAVVVCLRECAVAVSNSWGP
ADLIVSFIAPCIMMLILYTRILTVALKQAKAISNMTKKNGSQPSRKSEVKATKTLSTIIFVYFICWIPWYVIMLNMEQFNKLPVSISAFLCLFYT
NSCINPFIYAISYPWFKRSVKLVVSLRILQTATNQLNLFAEDC
>DanTAR_14a-001
MCEDYSCPERSVSLSVYVILYVAAAAVALLTVCGNLLVIISVSHFKQLHTPANILILSLAASDLLVGVFVMPFHLSWLIESCWISGPVMCSVFNF
VTFQATSASVHTVALIAVDRFLALSFPFFYSEKISLTVICTAALLNWLFSLIYNFTLLYINGNFADSMCPGQCLYIVDGVSSFIDLLIVFVMPCT
LIIILYTHVFVIAKKHATAIRALQVHNSTESSKNKISDKSERKAALALGILVFVFLLCLLPYYIFSLTNFLRDDSFSNVINSVLILFYLNSSINP
LMYALLYPWFKRSLKIITSFKVFNKDSSLINVISQ
>DanTAR_14f-001
MNLTAVNQTDMCEDYSCPERSVSLSVYVILYVAAAAVALLTVCGNLLVIISVSHFKQLHTPANILILSLAASDLLVGVFVIPLHLSWVIESCWIS
GPVVCLVFKLVNYQATSVSVHTVALIAVDRFLALSFPFFYSEKISLTVVCTAALLNWLFSLIYNFTLFYVNENFTVVVCPGVCFHHLDGISSLID
LLIVFVMPCTLIIILYTHVFVIAKKHATAIRALQVHNSTESSKNKISDKSERKAAMLLGILVFVFLLCLLPYYITFLVIPYGSIIYMYVINVAVI
FFFLNSTINPIIYALFYSWFKKSLKLIFTFKVFHKDSSLMNVM
>DanTAR_14g-001
MNLTAVNQTDMCEDYSCPERSVSLSVYVILYVAAAAVALLTVCGNLLVMISVSHFKQLHTPANILILSLAASDLLVGVFVIPLHLSLLIESCWIS
GPVMCSIFKFVNFQPTSVSVHTVALIAVDRFLALSFPFFYSEKISLNVVCTAALLNWLFSLIYNFTLFYINGNFTDSVCPGECVYNIDGTSSIID
LLIVFVMPCTLIIILYTHVFVIAKKHATAIRALQVHNSTESSKNKISDKSERKAAMLLGILVFKALPVFLLCLLPYYITFLVIPYGSVINFVRDV
AVIFFFLNSTINPIIYALFYSWFKKSLKLIFTFKVFHKDSSLMNVM
>DanTAR_14l-001
MCEDFSCPERSVSLSVYVILYVAAAAVALLTVCGNLLVIISVSHFKQLHTPANILILSLAASDFLTGVFVMPFHLSWLIESCWISGPVMCSVFNF
VSFQATSVSVHTVALIAVDRFLALSFPFLYSDKISPTVICITTLFNWLFSFLYNFTLLYINGNFVDSVCLGVCVYNIDGISSIIDLLIVFLMPCT
LIIILYTHVFVIAKKHATAIRALQVHNSTESSKNKISDKSERKAAMLLGILVFVFLLCLLPYYITSLVIPYSTANLYHVRSVATIFFFLNSTINP
IIYALFYPWFQKSLKLIFTLKAFNKDSSLMNVM
>DanTAR_14l-203
MCEDFSCPERSVSLSVYVILYVAAAAVALLTVCGNLLVIISVSHFKQLHTPANILILSLAASDFLTGVFVMPFHLSWLIESCWISGPVMCSVFNF
VSFQATSVSVHTVALIAVDRFLALSFPFLYSDKISPTVICITTLFNWLFSFLYNFTLLYINGNFVDSVCLGVCVYNIDGISSIIDLLIVFLMPCT
LIIILYTHVFVIAKKHATAIRALQVHNSTESSKNKISDKSERKAAMLLGILVFVFLLCLLPYYITSLVIPYSTANLYHVRSVATIFFFLNSTINP
IIYALFYPWFQKSLKLIFTLKAFNKDSSLMNVIQNY
>DanTAR_14l-204
MCEDFSCPERSVSLSVYVILYVAAAAVALLTVCGNLLVIISVSHFKQLHTPANILILSLAASDFLTGVFVMPFHLSWLIESCWISGPVMCSVFNF
VSFQATSVSVHTVALIAVDRFLALSFPFLYSDKISPTVICITTLFNWLFSFLYNFTLLYINGNFVDSVCLGVCVYNIDGISSIIDLLIVFLMPCT
LIIILYTHVFVIAKKHATAIRALQVHNSTESSKNKISDKSERKAAMLLGILVFVFLLCLLPYYITSLVIPYSTANLYHVRSVATIFFFLNSTINP
IIYALFYPWFQKSLKLIFTLKAFNKDSSLMNVIECF
>DanTAR_14e-001
MNLTAVNQTDMCEDFSCPERSVSLSVYVILYVAAAAVALLTVCGNLLVIISVSHFKQLQTPANILILSLAASDLLVGVFVIPLHLSWVIESCWTS
GSVMCAVLKVVNFQATSVSVHTVALIAVDRFLALGFPFFYSEKISLTVNCIATLVNWLFSLLYNFTLLYINGNFTDVVCPGVCIAIIDGISSIVD
LLIVFLMPCILIIILYTHVFVIAKKHATAIRALQVHNSTESSKNKISDKSERKAAMLLGILVFVFLLCLLPYYITSLVIPYISENLFHIRDVTVM
MFFLNSTINPIIYALFYPWFQKSLKLIFTFKVFNKDSSLMNVM
>DanTAR_2
MAANETDVYAENVFLCFPLRLDSCLSAQRLTTVKVAMYILMLLIIFLTVFGNLLVVVSISYFKHLQSPTHLIVQSLAACDCLLGSLVMPYSMVRS
IEGCWYLGDVICKVHSSLDMTLSISSKMHLSLISVDRYWAICDPLMYRTRVTNYSITVFIMCAWLFAFVYSFSIVFSEVNLIGLEVLVLQISCLG
SCVLLFNKPSALICSILTFFLPGAIMSSLYVKIFRVASKHAKVLSERVSVLEIKSQTYAQRERKAAKIVAIVVGAFLLCWLPFFVATALDPFLDF
WTPADVFDALVWFGYFNSTCNPLIYGFFYPRFQKAFKILMSTVMCGSSSSRTLAFG
>DanTAR_20q-001
NKLLTGGDSLMAYETEDQETQYCFPDINSSCVKGRRSSLGYNIIYVFVSLLSAWTVFLNLLVIISISHFKKLHTPTNMIILSLAVNDLLIGLFMP
VEAIRLIETCWYFGDTFCGLHLFFVSLLLAASLSNLVLIAVDRYVAVCHPLLYPQKITITKTLLSICLSWVCLSAYIITLSTNNGYFDTSLKTDV
CYGQCLVMMSFSWIVTDLFMSFIFPCTLIITLYLRIFYVVHQQVKVINSLMKGGKCVTEGLVKRKSESKAALTLGIIVSVYLLCYIPYFICSLTV
NSSTTINGLLFAVCANSGLNPLIYALFYTWFKKTAKLILTLKIVHSASSLINIFTEH
>DanTAR_20x-001
GGDSLMAYETEDQETQYCFPDINSSCVKGRHSSHGYIAYLLVSLLSAWTVFLNLLVIISISHFKKLHTPTNMIILSLAVNDLFIGLIMPIEAVRL
IETCWYFGDTFCNLYLIFIVILLSASLSNLVLIAVDRYVAVCHPLLYPQKITITKTFMCICMCWVCFSAYCTALVINNSYFDPSHRTGVCYGQCL
VLMSFSWTVTDLFVGFIFPCALIITLYLRIFYVVHLQVKVINSLMKGGKSVTENLLKRKSESKAALTLGFIVTVYLLCWIPFYICSLTVYSSTAM
NVLIWAVHSNSGLNPLVYALFYPWFKKTGKLILTLKIFQPASSFI
>DanTAR_20c-001
KLLTGGDSLMAYETEDQETQYCFPDINSSCVKRRSSSHGYNIIYVFVSLLSAWTVFLNLLVIISISHFKKLHTPTNMIILSLAVTDLLIGLVMPV
EAIRLIETCWYFGDTFCGLYLLFVSILLSASLGNLVLLAVDRYVAVCHPLQYPQKITTTKTLMSICLSWLCLSAYNTAIVINNGYFDSSRRTDVC
YGRCLIMVSFSWIVTDLCMSCIFPCTLIIMLYLRIFFVVHQQVKVINSLITGGKCVTEGSVQRKSESKAALTLGIIVSVYLLCYIPYYICSLTEI
PSTAMNVLIWAVHSNSGLNPLVYALFYRWFKKTAKLILTLEIFQPASSWINIFTEN
>DanTAR_20c-201
KLLTGGDSLMAYETEDQETQYCFPDINSSCVKRRSSSHGYNIIYVFVSLLSAWTVFLNLLVIISISHFKKLHTPTNMIILSLAVTDLLIGLVMPV
EAIRLIETCWYFGDTFCGLYLLFVSILLSASLGNLVLLAVDRYVAVCHPLQYPQKITTTKTLMSICLSWLCLSAYNTAIVINNGYFDSSRRTDVC

YGRCLIMVSFSWIVTDLCMSCIFPCTLIIMLYLRIFFVVHQQVKVINSLITGGKCVTEGSVQRKSESKAALTLGIIVSVYLLCYIPYYICSLTEI
PSTAMNVLIWAVHSNSGLNPLVYALFYRWFKKTAKLILTLEIFQPASSWINIFTEKQ
>DanTAR_20d1-001
NKLLTGGDSLMAYETEDQETQYCFPDINSSCVKGKRFRHGYITYLFVSLLSAWTVFLNLLVIISIFYFKKLHTPTNMIILSLSVNDLFIGLIMPL
EAIRLTETCWYFGDTFCAIYMLFISLLVSASLSNLVFISVDRYVAVCHPLQYPQKITITKTLMSICLNWVCYSAYIITFVNNIGYFDSSHRTDVC
YGQCSVMMSFSWIVTDLFVSFIFPCVLIISLYLRIFYVVHQQVKVMNSLMKGGKCVMKSSVKRKSESKAALTLGIIVTVYLLCWIPYYICSLTVI
SSTTINVLLLLVYANSGLNPLIYALFYPWFKKSAKLILTLKIF
>DanTAR_19r-001
NKLLTGGDSLMAYETEHQETQYCFPNINSSCVKGRRYRHEYIIMYVFFSLLSAWTVFLNLLVIISISHFKKLHTPTNMIILFLAVNDLLIGLIVL
PVEAIRLIERCWYFGETFCFLYLILIVLIFSASLSYLVLIAVDRYVAVCHPLLYPQKITITNMLKSICLSWVCYSAYNIAFVINNRNLNSSQKTD
VCYGQCLIMMSFSWTIIDLSICFIFPCLIMITLYLRIFYVVHQQVKVINSLMKGAKCETEGSVKRKSESKAALTLGIIVSVYLLCYIPYYICTLL
VSSSTTINVLMWNVHANSGLNPLVYAIFYPWFKKTAKLILTLKIFQPASSLINIFTENE
>DanTAR_12b-001
MTSNETQTDNILLCYPLLSNSCPKLHRLAVVQVGLYVCLLLMILTTVFGNLLIIISISHFKHLQSPTHLIVQSLAACDCLMGSLVMPYSMVRSVE
GCWYLGDVVCKVHSSLDMTFCMSSLLHLGLISVDRYWAICDPLRYRLRVTNTTVTVFIIVIWLFSFIYNFSIVFSGITAVGLEMLILQTYCVGSC
IVLFNKEWAVYPFLTFFITGAIMSSLYMKIFHVAQKHAKVMSERVTGGLKSQSSAQRERKAAKTLAIVMGVFMFCWLPYCAFTALYPFFTFLNSA
EVFDVLFWFAYFNSSCNPVIYGFFYPCFQKAFKILISTYIFGIRNANTSTYE
>DanTAR_1b-001
MDLCYEAMNGSCWKYVRPHAIHVPMLIAIMLIISMTFIGNLLVIISIGHFRQLHTPTNQLILSLALCDFLIGLFVMPLSAVRSMQGCWYFGEFLC
KLHTCIDITLSTSSIFHLVSVSAERFCAVCGPLRYRSCFGLSTVLLMISISWLIPGIFAYVMTFLEINIHGGKDFYDAHVRCVGGCHVFFSHGPA
VFTSVFSFYIPGFIIVVIYSRIYMVARNQERSIRLQLNQLRRVYPSRDVQLQTRKATVTIAIVVGAFLVCWTPFFLCNILNPFIGYATPPMLIDV
LMWFGYANSTLNPFIYAFMHSWCRKAVRIIVTGEIFKNNSSRKDLYT
>DanTAR_2b
MDLSSQEYDPSQFCFPAVNNSCLKGTHHVSTQTVVYLILASAMTVTVLGNSVVIISIAHFKQLQTPTNILVMSLALADLLLGLVVMPFSMIRSVD
GCWYYGETFCLLHTGFDLFLTSVSIFHLIFIAVDRHQAVCFPLQYPTRITIPVAWVMVMISWSMAAFYSYGVVYSKANLEGLEEYIASVYCMGGC
TLYFNALWSVLDTLLTFFLPCSVMVGLYARIFVVAKKHIKSITEANQNENENVFKNPRRSERKAAKTLGIVVGAFILCWLPFFINSLVDPYINFS
TPYALFDAFGWLGYTNSTLNPIIYGLFYPWFRKTLSLIVTLRIFEPNSSDINLFTV
>DanTAR_14d-001
MNLTAVNQTDMCEDYSCPERSVSLSVYVILYVAAAAVALLTVCGNLLVIISVSHFKQLHTPANILILSLAASDFLTGVFVIPLHLSLVIESCWTS
GSVMCLVLKVVDFQATSVSVHTVSLIAVDRFLALSFPFFYSEKISLTVVCTATLLNWLFSFIYNFTLLYVNGNFTDVVCPAVCFAITDEISSIID
LLIVFVMPCTLIIILYTHVFVIAKKHATAIRALQVHNSTESSKNKISDKSERKAAMLLGILVFVFLLCLLPYYITALAIPYNSENTLQIRDVAGI
FFFLNSTINPIIYALFYSWFQKSIKLIFTFKVFHKDSSLMNVM
>DanTAR_13a-001
MDLSSQEYDPSQFCFPAVNNSCLKGTHHVSTQTVVYLILASAMTVTILGNSVVIISIAHFKQLQTPTNILVMSLALADLLLGLVVMPFSMIRSVD
GCWYYGETFCMLHSTFDLFLTSVSILHLVCIAVDRHQAVCYPLQYPTRITISVAWVMVMISWTLIAIYSYGLVYSKANVEGLEEYIESIYCMGHC
SLLFSKLWSVLDTFITFFFPSCIMVGLYIRIFVVANKHARVITEANPNENENVFKSSRRSERKAAKTLGIVVGAFIICWLPFFINSLMDPYINFS
TPVALFDAFVWLGYINSTINPIIYGFFYPWFRKTLYLIITRRIFEPNSSDINVFTV
>DanTAR_20t-001
NKLETDSLMANETEDQETQYCFPDINSSCVKGLLSNHGYIITYVFASLLSAWTVFLNLLVIISISHFKKLHTPTNMIILSLAVTDLFIGVVMPVE
AIRLIETCWYFGDTFCALYLLFVELLLSASLSNLVLIAVDRYVAVCHPLLYPQKITITKTLMSICLSWAWLLAYIVAPVINSRHFYTSHGTDECY
GECLVLISFSWTVTDLFMSFIFPCTVIIMLYSRIFFVVHQQVKVINSLMKGGKCVTEGSVKRKSESKAALTLGIIVTVYLLCWIPYYICSLTVIS
STIINVLIWFLYVNSGLNPLVYALFYPWFKKTAKLILTLKIFQPASSLVNIFKDH
>DanTAR_20t-201
MANETEDQETQYCFPDINSSCVKGLLSNHGYIITYVFASLLSAWTVFLNLLVIISISHFKKLHTPTNMIILSLAVTDLFIGVVMPVEAIRLIETC
WYFGDTFCALYLLFVELLLSASLSNLVLIAVDRYVAVCHPLLYPQKITITKTLMSICLSWAWLLAYIVAPVINSRHFYTSHGTDECYGECLVLIS
FSWTVTDLFMSFIFPCTVIIMLYSRIFFVVHQQVKVINSLMKGGKCVTEGSVKRKSESKAALTLGIIVTVYLLCWIPYYICSLTVISSTIINVLI
WFLYVNSGLNPLVYALFYPWFKKTAKLILTLKIFQPASSLVNIFKDH
>DanTAR_20w-001
DSLMAYEIEDQETQYCFPDINSSCVKEKRSSQGYIIIYLFASLLSAWTVFLNLLVIISISHFKKLHTPTNMIILSLAVNDLLIGVVMPIEAIRQT
ETCWYFGNIFCGLYLLFISLLMSASLSNLVLIAVDRYVAVCHPLLYPQKITITKTLMIICLSWVWLSAYSIALLINNGYFDTSHRSDECYGECLA
IISFSWIITDLFMSFMFPCPLIMMLYLRIFYVVHQQVKVINSLMKGGKCVTEGSVKRKSESKAALTLGIIVAVYMLCWIPYYICSLIVISSTAMI
VIIWAVYANSALNPLVYALFYPWFKKTAKLIVTLKILQPASSLSNICTQ
>DanTAR_20w-201
TEEPFFLQQSDSDTVYQWQKTQPVKNKYINSSCVKEKRSSQGYIIIYLFASLLSAWTVFLNLLVIISISHFKKLHTPTNMIILSLAVNDLLIGVV
MPIEAIRQTETCWYFGNIFCGLYLLFISLLMSASLSNLVLIAVDRYVAVCHPLLYPQKITITKTLMIICLSWVWLSAYSIALLINNGYFDTSHRS
DECYGECLAIISFSWIITDLFMSFMFPCPLIMMLYLRIFYVVHQQVKVINSLMKGGKCVTEGSVKRKSESKAALTLGIIVAVYMLCWIPYYICSL
IVISSTAMIVIIWAVYANSALNPLVYAL
>DanTAR_18h-001
NKLLTGGDSLMAFETEDQETQYCFPNINSSCVKEKRSRHEYNIIYLFLSLLSAWTVFLNLLVIISISHFKKLHTPTNMIILSLAVVDLLTGLIGI
PVEGIKQIETCWYFGDTFCGLYVITVRLLLSASLSNIVLIAVDRYVAVCHPLLYPQKITTTKTLISICVCWFCSSVYSIVYVINNGFFNISQKSM
CYGQCTVMFSPGWKTADLICSFLIPCTLILTVYLRIFYIVHWQVKVINSLMKGGKRVTEGSVRRKSESKAALTLGIIVTVYLFCWIPFYICSLTK
MTAITTTTMTFLTWTMFVSCGINPLVYALFYKWFKISVKCIITLRILQPASSLMDIF
>DanTAR_18a-001
NKLLTGGDSLMAYETEDQETQYCFPDINSSCVKENRSKHEYNIMYVFFSLLSAWTVFLNLLVIISISHFKKLHTPTNMIILSLAVNDLLLGLIVM
PVDAVKLIETCWYFGDILCDLYMIIMGLLLSASLSNLILIAVDRYVAICHPLLYPQKITMTRTLISIFVCWFVCLTNNIAFLVSSRHFDILQKTN
MCHGQCTLIVSFSWTFTDLFLSFLVPCTLIITLYLRIFYVAYQQVKVINAIIKGGKCAVEGSVKRKSERKAALTLGIVVTVYLLCYIPYYILSVI
GTTVISSTMKILIWSLYINSCLNPLIYALFYRWFKISVKCVLTLKILEPASSLLDIFKDN
>DanTAR_18f-001
KLLTGGDSLMAYETEDQETQYCFPDINSSCVKGKRSRYEYNIMYMFFSLLSAWAVFLNLLVIISISHFKKLHTPTNMIILSLAVNDLLVGLIVMP
VEAVKLIETCWYFGETLCGLFMIIMGLIGTTSLSNLVLIAVDRYVAVCHPLIYPQKITMTKTLLSICLCWLFSSTYSTVFVVNNKYFEISNRTDV
CYGMCTLNLSFTYIIVDLIYSFLLPCVVMITVYLRIFYVAFKQVKVINSLLKGSRESSVKRKSEHKAALTLGIVVTVYLICFIPYSLSSVMGVSS
GTLTYLLWTVYVNCVVNPLIYALFYCWFKISVKHILTLKILEPASSLLDIFTD
>DanTAR_19m-001
NKLLTGGDSLMAYETEDQETQYCFPNINSSCVKEKRSIREYIIMYLFSSLLSVWIVFLNLLVIISISHFKKLHTPTNMIILSLAVADLLIGLIVM
PLQAIKQIETCWYFGESFCGINLILIGLIFSASFSNLVLIAVDRYVAVCHPLLYPRKITTAKMLMSILLSWLYYSGYNTGLVINNGYADTSYRTD
MCYGECTIIMSFGWILTDMFMFFIFPCLIMITFYVRIFYVVHQQVKVMNSLMKGGKCVTEGSVKRKSESKAALTLGIIVTVYLLCYIPYYICTLS

VNSVTAINVLGWIVYANSGVNPLVYALFYPWFKKTAKIILTLKIFEPASSLINIFK
>DanTAR_20b-001
NKHLTGGDSLMAYETEDQETQYCFPDINSSCLKENRSSDEYNIIYLLVSLLSAWTVFLNLLVIISISHFKKLHTPTNMIILSLAVNDLLIGLVMP
IEAFRLIETCWYFGDTFCGLYLFFVTLLLSASLGSLVLIAVDRYVAVCHPLLYTQKITMTKTFCLSICLSWVCFSAYLTGLININNQDFDTSHRT
DVCYGQCIIMITFSWILTDLFVSFIFPCTLIFTLYLRIFYVVHQQVKVINSLMKGGKCVTEGSVERKSESKAALTLGIIVLVYLLCYIPYYICSL
TVNSSTALNVLIWIVYSNSGMNPLVYALFYPWFKKTVKLILTLKIFQQASSLINIFTE
>DanTAR_32-001
NKLLTGGDSLMAYETEDQETQYCFPDINSSCVKRQRSSHEYNVMYVLFSLLSAWTVFLNLLVIISISHFKKLHTPTNMIILSLAVTDMFVGLIVM
PVKAIKLIERCWYFGDTFCGLFIIIIGVIFSASLTNLVLIAVDRYVAVCHPLLYPQKITMAKMLMSICLSWLYYSAYNTALIINNGYFDTSNRTD
VCYGECPVMVSFSWIQTDLFMSFIFPCIIMITLYSRIFFAVHQQVKVINSLMKRDKCVTEGSVMRKSESKAALTLGIIVSVYLLCYIPYYICSLV
VNSSTALNVLTWTVYANSGLNPLVYALFYPWFKKTAKLILTLKIFQPASSLINIFTEN
>DanTAR_11-001
MMNQSHQWSAETFSLCFESINDSCVKTVYSPVFRAPLYLLFIIAIILIVFGNLWVICTISFFQQLHTPTNYLILSMAVSDLLLGSFVMPPSMLRS
LETCWYFGDFFCKFHSATDFTLCNASVLHLVFISIDRYYAVCQPFHYQSRMTTRVSVFMILISWSFSAFFGFGIIFSELKIEKKRTEELHVACKG
GCLALHGREIGVTYSLVFYFLPMFIIVSLYSRVFIIALKHVRVINSAASSLSATKMDLKATKTLAIIIGVFMSCWTPYFMCNIIDPIVNHTIPAL
LYEVLMWVAYLNAVFNPLVYAFFYSWFRDKSKLLLEKLYKLC
>DanTAR_11-201
MNQSHQWSAETFSLCFESINDSCVKTVYSPVFRAPLYLLFIIAIILIVFGNLWVICTISFFQQLHTPTNYLILSMAVSDLLLGSFVMPPSMLRSL
ETCWYFGDFFCKFHSATDFTLCNASVLHLVFISIDRYYAVCQPFHYQSRMTTRVSVFMILISWSFSAFFGFGIIFSELKRTEELHVACKGGCLAL
HGREIGVTYSLVFYFLPMFIIVSLYSRVFIIALKHVRVINSAASSLSATKMDLKATKTLAIIIGVFMSCWTPYFMCNIIDPIVNHTIPALLYEVL
MWVAYLNAVFNPLVYAFFYSWFRDKSKLLLEKLYKLCMSSFDLLNAFN
>DanTAR_10c-001
MDIRINFNQSEILEKPFLCYEFSNRSCQKFVHTLETRILFYILLSASSIITIIGNLLVIITVVHFRQLHTPTNYLILSLAVADLLVGGVVMPPSM
LRSIETCWYLGDLFCKIHSSLDVTLCTASILNLCIISLDRYYAICHPFQYHSKMTSLATLVMIIICWTVSAVLGFGMIFMELNILGVEDFYYENI
KCDGGCTLFQSKTIAIVYSLICFYIPALVILCVYLKILHEAQRQVQAIQSVNSELKKEGKANKTLAIIIGVFLTLWVPFFLCNLIDPFIGYSVPP
LLFDLFYWIGYYNSTCNPIVYAFFYSWFRHAFRVILSKAIFQTNSSRTVLL
>DanTAR_13b-001
MDLSSQEYDPSQFCFPAVNNSCLKGTHHVSTQTVVYLVLASAMTVTVLGNSVVIISIAHFKQLQTPTNILVMSLALADLLLGLVVMPFSMIRSVD
GCWYYGETFCLLHSTFDLFLTSVSILHLVCIAVDRHQAVCYPLQYPTRITISVAWVMVMISWAVIAIFSYSLMYSKANMEGLEEYIESIYCMGHC
SLLMNKLWSVLDTFITFFFPSFVMVGLYIRIFVVAKKHARVITEANPNENENLFKSSRRSERKAAKTLGIVVGAFIMCWLPFFINSTLDAYINFS
TPVALFDALVWLGYVNSTINPIIYGFFYPWFRKTLSLIVSLKIFEPNSSDINVFTV
>DanTAR_13d-001
MDLSSQEYDPSQFCFPAVNNSCLKGTHHVSTQTVVYLILASAMTVTILGNSVVIISIAHFKQLQTTTNILVMSLALADLLLGLVVMPFSMIRSVD
GCWYYGETFCMLHSSFDLFLTSVSIFHLIFIAVDRHQAVCFPLQYPTMITIPVAWVMVMISWSMAALYSYGLVYSKANMEGLDEYIQSMYCVGGC
TLYFNALWSVLDTLITFFLPCSVMIGLYARIFVVAKKHARIINEANQNEIDGTFKSSRRSEQKAAKTLGIVVGAFIMCWLPFFINSLMDPYINFS
TPVALFEAFVWLGYINSTMNPIIYGFFYPWFRKTLYLIITRRIFEPNSSDINIFTL
>DanTAR_12i-001
MTSNETDVYAENMFLCFPHLPNSCPKSQRFVVVKLAMYVCLLLMILTTVFGNLLIIISISHFKHLQSPTHLIVRSLAASDCLLGSLVMPYSMVRS
VEGCWYLGDVVCKVHSSLDMTFCISSLLHLGLVSIDRYWAICDPLRYRIRVTNTTVTLYTIFIWLFSFLYSFVIVFSGVNRIGLETFIMQVYCVG
SCVLFFNKEWGLICPVLTFFIPGAIMSSLYMKIFHVARKHAKVMSERVTGGLKSQSSAQRERKAAKTLAIVMGVFLLCWTPIFIASIIDSFLNFV
TPAGVFDALIWFGYFNSTCNPLIYGFFYSCFQKAFKILLSSLFLGFGNSSTLTFE
>DanTAR_12e-001
MMSLNETQTENILLCYPLLSNSCPKLHRLAVVQVGLYVFLLLMILTTVFGNLLIIISISHFKHLQSPTHLIVRSLAACDCLLGLLVMPYSMVRSV
EGCWYLGDVVCKVHSSLDMTFCISSLLHLGLISVDRYWAICDPLRYRLRVTNITVTVFIVFIWLFSFVYSFYVVFSGVNTVGLETFIMQVYCVGS
CVLYFNKQWGLICPILVFFLPGAIMSSLYMKIFHVARKHAKVMSERVTGGLKSQSSAQRERKAAKILAIVMGVFLFCWLPFFTVNALDPFFNFFT
PADIFDAVIWFAYLNSTCNPLIYGFFYPCFQKAFKILIFTYICGVRNLDTFA
>DanTAR_12e-201
LNETQTENILLCYPLLSNSCPKLHRLAVVQVGLYVFLLLMILTTVFGNLLIIISISHFKHLQSPTHLIVRSLAACDCLLGLLVMPYSMVRSVEGC
WYLGDVVCKVHSSLDMTFCISSLLHLGLISVDRYWAICDPLRYRLRVTNITVTVFIVFIWLFSFVYSFYVVFSGVNTVGLETFIMQVYCVGSCVL
YFNKQWGLICPILVFFLPGAIMSSLYMKIFHVARKHAKVMSERVTGGLKSQSSAQRERKAAKILAIVMGVFLFCWLPFFTVNALDPFFNFFTPAD
IFDAVIWFAYLNSTCNPLIYGFFYPCFQKAFKILIFTYICGVRNLDTFAFA
>DanTAR_12c-001
MTSNETQTDNVHLCFPLLPNSCSKLHHFVVVKVAMYVCLLLMILTTVFGNLLIIISISHFKHLQSPTHLIVRSLAASDCLLGSLVMPYSMVRSVE
GCWYLGDVVCKVHSSLDMTFCISSLLHLGLISVDRYWAICDPLRYRLRVTNTTVTVFTVFIWLFAFVYSFSVVFSGIAAVGLEMLILQTYCVGSC
VLFFNKEWGLICPILTFFLPGAIMSFLYMKIFHVARKHAKVMSERVTGGLKSQSSAQRERKAAKTLAIVMGVFMFCWLPYFTVTILGPFFNFATP
ADVFDALVWFAYLNSTCNPLIYGFFYPCFQKAFHILISTYICGVRNSDTLIYE
>DanTAR_12j-001
MTSNDTDVHSENVLLCYPLLSDSCPRTQRLTALTVTMYAFMVLMILTTVFGNLLIIISISHFKQLQSPTHLIVRSLAASDCLLGSLVMPYSMVRS
VEGCWYLGDVVCKVHSSLDMTFSISSLIHLSLVSVDRYWAICDPLRYRMRVTNNTVIVFTVFTWLFSFVYSFSVVFSGVNRIGLETFIMQVYCVG
SCVLFFNKQWGLICSLLTFFLPCTIMSSLYMKIFHVARKHAKVMSERVTGGLKSQSSAQREGKAAKTLAIVMGVFYLCWLPFFTATAVDPFLNFS
TPVHVFDALVWFGYFNSTCNPLIYGFFYPRFQKAFNILISTYICGSSDSHTLIPE
>DanTAR_14h-001
MCEDYSCPERSVSLSVYVILYVAAAAVALLTVCGNLLVIISVSHFKQLHTPANILILSLAASDLLVGVFVIPLYLSWIIESCWTSGLLMCSIFKF
VNFQATSVSVHTVSLIAVDRFLALSFPFFYSEKISLNVNCIAALLNWLFSLIYNVTLLYVNGNFTDVVCPGVCVSVVGVVSSIVDLLLVFVMPCI
LIIILYTHVFVIAKKHATAIRALQVHNSTESSKNKISDKSERKAAMLLGILVFVFLLCLLPYYITSIVVSYSTVDLFHVRDVAVIFFLLNSTTNP
IIYALFYPWFQKSLKLIFTFKVFQRDSSLMNVQSH
>DanTAR_14h-201
MCEDYSCPERSVSLSVYVILYVAAAAVALLTVCGNLLVIISVSHFKQLHTPANILILSLAASDLLVGVFVIPLYLSWIIESCWTSGLLMCSIFKF
VNFQATSVSVHTVSLIAVDRFLALSFPFFYSEKISLNVNCIAALLNWLFSLIYNVTLLYVNGNFTDVVCPGVCVSVVGVVSSIVDLLLVFVMPCI
LIIILYTHVFVIAKKHATAIRALQVHNSTESSKNKISDKSERKAAMLLGILVFVFLLCLLPYYITSIVVSYSTVDLFHVRDVAVIFFLLNSTTNP
IIYALFYPWFQKSLKLIFTFKVFQRDSSLMNVQIY
>DanTAR_10b-001
MDIRINISHNGIWDKPFLCYEFSNRSCQKFVYPLETRIFLYILFSASSIITIIGNLLVIITVVHFRQLHTPTNYLILSLAVADLLVGGVVMPPSM
LRSIETCWYLGDLFCKIHSSLDVTLCTASILNLCIISLDRYYAICHPFQYHSKMTSLATLVMIIICWTVSAVLGFGMIFMELNILGVEDFYYENV
DCNGRCLVFQSREVAVFMSLACFYIPAFVMLCVYLKILHEAQRQVQAIQSVNSELKKEGKATKTLAIIVGVFLTFWIPFFLCNLIDPFIGYSVPP
LLFDLFLWVGYYNSTCNPIVYAFFYSWFRHAFRVILSGGIFQSNSSRTILL

>DanTAR_10b-201
KPFLCYEFSNRSCQKFVYPLETRIFLYILFSASSIITIIGNLLVIITVVHFRQLHTPTNYLILSLAVADLLVGGVVMPPSMLRSIETCWYLGDLF
CKIHSSLDVTLCTASILNLCIISLDRYYAICHPFQYHSKMTSLATLVMIIICWTVSAVLGFGMIFMELNILGVEDFYYENVDCNGRCLVFQSREV
AVFMSLACFYIPAFVMLCVYLKILHEAQRQVQAIQSVNSELKKEGKATKTLAIIVGVFLTFWIPFFLCNLIDPFIGYSVPPLLFDLFLWVGYYNS
TCNPIVYAFFYSWFRHAFRVILSGGIFQSNSSRTILLKIYLKLSV
>DanTAR_10b-202
KPFLCYEFSNRSCQKFVYPLETRIFLYILFSASSIITIIGNLLVIITVVHFRQLHTPTNYLILSLAVADLLVGGVVMPPSMLRSIETCWYLGDLF
CKIHSSLDVTLCTASILNLCIISLDRYYAICHPFQYHSKMTSLATLVMIIICWTVSAVLGFGMIFMELNILGVEDFYYENVDCNGRCLVFQSREV
AVFMSLACFYIPAFVMLCVYLKILHEAQRQVQAIQSVNSELKKEGKATKTLAIIVGVFLTFWIPFFLCNLIDPFIGYSVPPLLFDLFLWVGYYNS
TCNPIVYAFFYSWFRHAFRVILSGGIFQSNSSRTILLLQN
>DanTAR_14b-001
MNLTAVNQTDMCEDYSCPERSVSLSVYVILYVAAAAVALLTVCGNLLVIISVSHFKQLHTPANILILSLAASDFLTGVFVIPLHLSLVIESCWTS
RSVMCLVFKVVNFQATSVSVHTVSLIAVDRFLALSFPFFYSEKISLTVVCTATLLNWLFSFIYNFTLLYVNGNFTDVVCPAVCFAITDGISSIID
LLIVFVMPCILIIILYTHVFVIAKKHATAIRALQVHNSTESSKNKISDKSERKAAMLLGILVFVFLLCLLPYYITALAIPYNSENTLQIRDVAVI
FFFLNSTINPIIYALFYSWFQKSIKLIFTFKVFHKDSSLMNVM
>DanTAR_10d-001
MDIRINISHNGIWDKPFLCYEFSNRSCQKFVYSLETRILFYILLSASSIITIIGNLLVIITVVHFRQLHTPTNYLILSLAVADLLVGGVVMPPSM
LRSIETCWYLGDLFCKIHSSLDVTLCTASILNLCIISLDRYYAICHPFQYHSKMTSLATLVMIIICWTVSAVLGFGMIFMELNILGVEDFYYENI
KCDGGCTLFQSKTGGTVFSLICFYIPALVILFVYLKILHEAQRQVQAIQSVNSELKKEGKANKTLAIIIGVFLTLWVPFFLCNLIDPFIGYSVPP
LLFDLFYWIGYYNSTCNPIVYAFFYSWFRHAFRVILSKAIFQTNSSRTVLL
>DanTAR_12h-001
MILNDTDIYSENVLLCYPLLPDSCPRTQRLPALKVAMYAVMVLMILTTVFGNLLVIISISHFKQLQSPTHLIVQSLAACDCLLGSLVMPYSMVRS
VEGCWYLGTVVCKVHSSLDMTFSISSILHLSLIAIDRFWAISDPLRYKMRVTNTTVAGFITFTWLFSFVYSFSVVFTGVNNVGLEELILQISCFG
GCVLFFNKEWGLICALFVFLIPGTIMSSLYMSIFNVVKRHAKVMSEKVSAAATAGSHFQTSSHRERKAAKTLAIVMGVFYLCWLPFFTATAVDPF
LNFSTPGDVFDALVWFGYFNSTCNPLIYGFFYPRFQKAFKILISTYICGSSDSHTLTFE
>DanTAR_12a-001
MIKKKIIQDVMTSNQTQTENILLCYPLLSNSCPKLHRLAVVQVGLYVFLLLMILTTVFGNLMIIISISHFKHLQSPTHLIVQSLAACDCLMGSLV
MPYSMVRSVEGCWYLGDVVCKVHFSFDVTFCISSLLHLCLISVDRYLAICDPLRYKIRVTNTTMTVFIIFIWLFSVVYSFSIVFSGITAVGLEML
ILQTYCVGSCVLFFNKEWAVYPFLTFFITGAIMSSLYMKIFHVARKHAKVMSERVKGGLKSQRSAQRERKAAKTLAIVMGVFMFCWLPYCAFTAL
YPFFTFLNSAEVFDVLFWFAYFNSSCNPLIYGFFYPCFQKAFKILIFLWSQKCKHFSF
>DanTAR_10-001
MDLSNSTATQFGLVLCFESINNSCVRYIYPSEIQLMLYLFFSAIALTTVCGNLLVIIAILHFKQLHTPTNYLILSLAVADLLIGGVVMPPSMLRS
IQTCWYLGDLFCKIHSSVDIMLCIASLLHISFISIDRYYAVCHPLQYRSKITPPVSFTMISLSWGLAAFVGFAMVFLQLNIMGSEDFYFDNVACN
GACIIFQTPAASITSSFLAFGLPAVIAVSIYLKILLVARRQSKSIQSVSKKNSLSVNKSEGKATKTLAIIMGVFFISISPFFFCNLVDPFIDYTI
SPAVFDVLLWIGYFNSLCNPFVYAFFYRWFRKALRIILLAKIFVVNSSQLDLMGRHE
>DanTAR_10-201
MDLSNSTATQFGLVLCFESINNSCVRYIYPSEIQLMLYLFFSAIALTTVCGNLLVIIAILHFKQLHTPTNYLILSLAVADLLIGGVVMPPSMLRS
IQTCWYLGDLFCKIHSSVDIMLCIASLLHISFISIDRYYAVCHPLQYRSKITPPVSFTMISLSWGLAAFVGFAMVFLQLNIMGSEDFYFDNVACN
GACIIFQTPAASITSSFLAFGLPAVIAVSIYLKILLVARRQSKSIQSVSKKNSLSVNKSEGKATKTLAIIMGVFFISISPFFFCNLVDPFIDYTI
SPAVFDVLLWIGYFNSLCNPFVYAFFYRWFRKALRIILLAKIFVVNSSQLDLMGRHE
>DanTAR_12g-001
MTSNESENIQLCYPLLSNSCPKLHRLAVVQVGLYICLLLMILTTVFGNLLIIISISHFKHLQSPTHLIVRSLAACDCLLGSLVMPNSMVRSVEGC
WYLGDVVCKVHSSLDMTLCISSLLHLGLISVDRYLAICDPLRYRIRVTNTTVTVFTVFIWLFSVVYSFYVKFSGITAVGLEMLILQTYCVGRCVV
FFNKQWGLICPVLAFFLPGAIMSSLYMKIFHVARKQAKVISERVTGGLKSQSSAQRERKAAKTLAIVMGVFLFCWMPFFTLTALDPFFNFLSSAD
VFDALFWFAYLNSACNPLIYGFFYPCFQKAFKILIFTYICGVKKANTLTFE
>DanTAR_12g-201
MQPSTQHESENIQLCYPLLSNSCPKLHRLAVVQVGLYICLLLMILTTVFGNLLIIISISHFKHLQSPTHLIVRSLAACDCLLGSLVMPNSMVRSV
EGCWYLGDVVCKVHSSLDMTLCISSLLHLGLISVDRYLAICDPLRYRIRVTNTTVTVFTVFIWLFSVVYSFYVKFSGITAVGLEMLILQTYCVGR
CVVFFNKQWGLICPVLAFFLPGAIMSSLYMKIFHVARKQAKVISERVTGGLKSQSSAQRERKAAKTLAIVMGVFLFCWMPFFTLTALDPFFNFLS
SADVFDALFWFAYLNSACNPLIYGFFYPCFQKAFKILIFTYICGVKKANTLT
>DanTAR_19o-001
NKLLTGGDSLMAYETEHQETQYCFPNINSSCVKEKRSIQEYIIMYVFFSLLSAWTVFLNLLVIISISHFKKLHTPTNMIILSLAVADLLIGLIVM
PIQAIRLIETCWYFGDTFCGLSLIIISVIFSATLSCLVLIAVDRYVAVCHPLLYPQKITMAKMLMSICLCWLYYSAYNTALVIDNGYFDTSYRTD
MCYGECSVMLSFSWILTDMFMSFIFPCLIMITLYLRIFYVVHQQVKVINSLMKGGKCVMEGSVKRKSESKAALTLGIIVLVYLLCWIPYYICSLT
VNSFTTITVLSWTVYANSGLNPLVYALFYPWFKKTTKLILTQKIFQPASSLINIFTQHE
>DanTAR_19f-001
NKLLTGGDSLMAYETEDQETQYCFPDINTSCVKEKHSKHEYNILYVFFSLLSAWTVFLNLLVIISISHFKKLHTPNVADLFIGLFVMPVKAIRLF
YKCWYFRETFCGFTLILIGVFPSASLSNLVLIAVDRFVAVCHPLLYPQKITITNMLISICLSWLYYCAYITALVINNRYFDTSHRTELCYGECSI
MMSFSWMLTNLFMSLILPCVIIITLYLRIFYVVHRQVKVINSLMKRGKCVMKSSVKRKSESKAALTLGIIVFVYLLCWIPYYICALSVNSSTAIN
VLSWAVFTNSGLNPLIYALFYPWFKKTAKLILTLKIFKPASSLINIFTELE
>DanTAR_20l-001
NKLLTGGDSLMAYETEDQETQYCFPAINSSCVKGRHSSHGYIAYLLVSLLSAWTVFLNLLVIISISHFKKLHTPTNMIILSLAVTDLLVGLLMPV
EAIRLIETCWYFGDTFCGLYLLFVALLLSASLGNLLLIAVDRYVALCHPLLYPQKITITKTLMSICLCWFCISVYNTALVINNGNLDTLNRKDEC
YGECFLMIRFSWIVIDLCVSLFFPCTVIIILYSRIFCVVHRQVKVINSLMKGGKCVMKSSMKRKSESKAALTLGIIVSVYLLCYIPYCICSLTVN
SLTVLNVLIWLLYASSGLNPLVYALFYSWFKKTAKIILTLHIFQPASSLINIF
>DanTAR_2-01
MKPSNETQTENILLCYPLLSNSCPKLHRLAVVQVGLYVFLLLMILTTVFGNLLIIISISHFKHLQSPTHLIVRSLAACDCLLGSLVMPYSMVRSV
EGCWYLGDVVCKVHSSLDMTFCISSLLHLGLISVDRYWAICDPLRYRLRVTNTTVTVFIVFIWLFSFVYSFYVVFSGVNTVGLETFIMQVYCVGN
CVLYFNKQWGLICPILTFFLPGTIMSSLYMKIFHVARKHAKVMSERVTGGLKSQSSAQRERKAAKILAIVMGVFLFCWLPFFTVNALDPFFNFFT
PADIFDAVIWFAYLNSTCNPLIYGLFYPCFQKAFKILISTYLCGVRNLDTFA
>DanTAR_2-02
LKSRIRQIMKPSNETQTENILLCYPLLSNSCPKLHRLAVVQVGLYVFLLLMILTTVFGNLLIIISISHFKHLQSPTHLIVRSLAACDCLLGSLVM
PYSMVRSVEGCWYLGDVVCKVHSSLDMTFCISSLLHLGLISVDRYWAICDPLRYRLRVTNTTVTVFIVFIWLFSFVYSFYVVFSGVNTVGLETFI
MQVYCVGNCVLYFNKQWGLICPILTFFLPGTIMSSLYMKIFHVARKHAKVMSERVTGGLKSQSSAQRERKAAKILAIVMGVFLFCWLPFFTVNAL
DPFFNFFTPADIFDAVIWFAYLNSTCNPLIYGLFYPCFQKAFKILI
>DanTAR_2-03

```
NETQTENILLCYPLLSNSCPKLHRLAVVQVGLYVFLLLMILTTVFGNLLIIISISHFKHLQSPTHLIVRSLAACDCLLGSLVMPYSMVRSVEGCW
YLGDVVCKVHSSLDMTFCISSLLHLGLISVDRYWAICDPLRYRLRVTNTTVTVFIVFIWLFSFVYSFYVVFSGVNTVGLETFIMQVYCVGNCVLY
FNKQWGLICPILTFFLPGTIMSSLYMKIFHVARKHAKVMSERVTGGLKSQSSAQRERKAAKILAIVMGVFLFCWLPFFTVNALDPFFNFFTPADI
FDAVIWFAYLNSTCNPLIYGLFYPCFQKAFKILISTYLCGVRNLDTFAFH
>DanTAR_13e-001
MDLSAQEYDASQFCFPAVNNSCLKGTHHVSTQTVVYLVLASAMTVTILGNSVVIISIAHFKQLQTPTNILVMSLALADLLLGLVVMPFSMIRSVD
GCWYYGETFCLLHSSFDMFLTSVSIFHLIFIAVDRHQAVCFPLQYPTMITIPVAWVMVIISWSMAAFYSYGLVYSKANVEGLEEYIESIYCMGGC
TLLFNALWGAIDTLVAFFLPCFVMIGLYARIFMIAKKHARKLGEANQHDNENLFKSSRRSERKAAKTLGIVVGAFVICWLPFFINSMMDPYINFS
TPGVLFEAFVWLGYMNSAINPIIYGLFYPWFRKTLYLIITLRMFEPNSSDINVFTV
```
