# Supplementary material for: Identification and characterization of cichlid TAAR genes and comparison with other teleost TAAR repertoires
Source: BMC Genomics. 2015 Apr 23;16(1):335. doi: 10.1186/s12864-015-1478-4 (PMC4415300; doi:10.1186/s12864-015-1478-4)
Supplement: Additional file 3: — TAAR gene sequence alignment. TAAR gene sequences were aligned with their cognate genome sequences, with MultiAlin [53], to identify the position of the genes on each contig and the positions of the 2 exons of spliced genes. [file 12864_2015_1478_MOESM3_ESM.pdf]

```
             1014110150    10160     10170     10180     10190     10200     10210     10220     10230     10240     10250     10260     10270
             |---------+---------+---------+---------+---------+---------+---------+---------+---------+---------+---------+---------+---------|
contig025313 CGGGGGGGGGGGGGGACAGGGGACTACTTAGCAGACAGTGATGAAAAGGTTGACTTGGGAGTTGGAAGGGGGGAGTGTCATCAGAGATAAATAAGCTGTACAATGTCTCACAGGGTCAGCAGGTTGCTTG
  BriTAR.A002
    Consensus ..................................................................................................................................

             1027110280    10290     10300     10310     10320     10330     10340     10350     10360     10370     10380     10390     10400
             |---------+---------+---------+---------+---------+---------+---------+---------+---------+---------+---------+---------+---------|
contig025313 AACTCAGCAGGTCCTCCTTTCTTTATACTGGAGGATGGACAGCCAGGACAGAATTGAACTCTGCTACCCTGAGCTTGCGAATTTGTCTTGCAGGGGTTTAACACGTCCTCAGCCTGAGGCTGTCCTCCTC
  BriTAR.A002                                   ATGGACAGCCAGGACAGAATTGAACTCTGCTACCCTGAGCTTGCGAATTTGTCTTGCAGGGGTTTAACACGTCCTCAGCCTGAGGCTGTCCTCCTC
    Consensus ..................................ATGGACAGCCAGGACAGAATTGAACTCTGCTACCCTGAGCTTGCGAATTTGTCTTGCAGGGGTTTAACACGTCCTCAGCCTGAGGCTGTCCTCCTC

             1040110410    10420     10430     10440     10450     10460     10470     10480     10490     10500     10510     10520     10530
             |---------+---------+---------+---------+---------+---------+---------+---------+---------+---------+---------+---------+---------|
contig025313 TATACACTGTTCTTGTCCATCTCTGTGCTAACTGTAGCTCTGAATGTGCTGGTTATCATATCCATCTCTCATTTTAGGCAGCTCCACATGCCCACCAATGTCCTCCTGCTCTCTCTGGCCATCTCAGACC
  BriTAR.A002 TATACACTGTTCTTGTCCATCTCTGTGCTAACTGTAGCTCTGAATGTGCTGGTTATCATATCCATCTCTCATTTTAGGCAGCTCCACATGCCCACCAATGTCCTCCTGCTCTCTCTGGCCATCTCAGACC
    Consensus TATACACTGTTCTTGTCCATCTCTGTGCTAACTGTAGCTCTGAATGTGCTGGTTATCATATCCATCTCTCATTTTAGGCAGCTCCACATGCCCACCAATGTCCTCCTGCTCTCTCTGGCCATCTCAGACC

             1053110540    10550     10560     10570     10580     10590     10600     10610     10620     10630     10640     10650     10660
             |---------+---------+---------+---------+---------+---------+---------+---------+---------+---------+---------+---------+---------|
contig025313 TCTTGGTCGGGCTGCTTGTGATGCCGGTGGAAACAATACGGTTTATAGAAACCTGCTGGCTGCTGGGAGATCTCATGTGTGCTTTTTCTTACATTATCGGCTTCACTCTCACCTCAGCCTCTGTGGGCAA
  BriTAR.A002 TCTTGGTCGGGCTGCTTGTGATGCCGGTGGAAACAATACGGTTTATAGAAACCTGCTGGCTGCTGGGAGATCTCATGTGTGCTTTTTCTTACATTATCGGCTTCACTCTCACCTCAGCCTCTGTGGGCAA
    Consensus TCTTGGTCGGGCTGCTTGTGATGCCGGTGGAAACAATACGGTTTATAGAAACCTGCTGGCTGCTGGGAGATCTCATGTGTGCTTTTTCTTACATTATCGGCTTCACTCTCACCTCAGCCTCTGTGGGCAA

             1066110670    10680     10690     10700     10710     10720     10730     10740     10750     10760     10770     10780     10790
             |---------+---------+---------+---------+---------+---------+---------+---------+---------+---------+---------+---------+---------|
contig025313 CATGGTGCTCATATCAATTGATCGCTACGTAGCTATTTGTTATCCTCTGCAGTACCCCACTAAAATCACTCACAGCAGAGCGGAGCTGTCTGTGACTCTGTGCTGGGCCTGTTCGCTCCTCTACAACGGG
  BriTAR.A002 CATGGTGCTCATATCAATTGATCGCTACGTAGCTATTTGTTATCCTCTGCAGTACCCCACTAAAATCACTCACAGCAGAGCGGAGCTGTCTGTGACTCTGTGCTGGGCCTGTTCGCTCCTCTACAACGGG
    Consensus CATGGTGCTCATATCAATTGATCGCTACGTAGCTATTTGTTATCCTCTGCAGTACCCCACTAAAATCACTCACAGCAGAGCGGAGCTGTCTGTGACTCTGTGCTGGGCCTGTTCGCTCCTCTACAACGGG

             1079110800    10810     10820     10830     10840     10850     10860     10870     10880     10890     10900     10910     10920
             |---------+---------+---------+---------+---------+---------+---------+---------+---------+---------+---------+---------+---------|
contig025313 CTGATCTTAAAGGAGCATCTCAGGCAGCCAGACAGGCATAACACCTGTCACGGTCAGTGTTTGGTGGTGATTAACTATGTCTCTGGAGCCATCGACCTTGTCTTTACTTTCATCGGCCCTTGCTCAGTCA
  BriTAR.A002 CTGATCTTAAAGGAGCATCTCAGGCAGCCAGACAGGCATAACACCTGTCACGGTCAGTGTTTGGTGGTGATTAACTATGTCTCTGGAGCCATCGACCTTGTCTTTACTTTCATCGGCCCTTGCTCAGTCA
    Consensus CTGATCTTAAAGGAGCATCTCAGGCAGCCAGACAGGCATAACACCTGTCACGGTCAGTGTTTGGTGGTGATTAACTATGTCTCTGGAGCCATCGACCTTGTCTTTACTTTCATCGGCCCTTGCTCAGTCA

             1092110930    10940     10950     10960     10970     10980     10990     11000     11010     11020     11030     11040     11050
             |---------+---------+---------+---------+---------+---------+---------+---------+---------+---------+---------+---------+---------|
contig025313 TTATCATTCTGTACATGAGAGTATTTGTTGTAGCGGTGTCTCAGGCTCATGCCATGCGGTCTCATATTACAGCTGCTGCAGCTGGTAGAGTAAAAATCACCGCAAAGAAATCAGAGAAAAAGGCAGCCAG
  BriTAR.A002 TTATCATTCTGTACATGAGAGTATTTGTTGTAGCGGTGTCTCAGGCTCATGCCATGCGGTCTCATATTACAGCTGCTGCAGCTGGTAGAGTAAAAATCACCGCAAAGAAATCAGAGAAAAAGGCAGCCAG
    Consensus TTATCATTCTGTACATGAGAGTATTTGTTGTAGCGGTGTCTCAGGCTCATGCCATGCGGTCTCATATTACAGCTGCTGCAGCTGGTAGAGTAAAAATCACCGCAAAGAAATCAGAGAAAAAGGCAGCCAG

             1105111060    11070     11080     11090     11100     11110     11120     11130     11140     11150     11160     11170     11180
             |---------+---------+---------+---------+---------+---------+---------+---------+---------+---------+---------+---------+---------|
contig025313 GACTCTTGGCGTTGTGATATTTGTGTTTTTGATGAGTTTCTGTCCGTATTACTATCCCTCTCTTGCAGGGCAGGACATTTCAAACAGTGCTTCATCTTGGGCTATTGTTTCCTGGATGCTGTACTTCAAT
  BriTAR.A002 GACTCTTGGCGTTGTGATATTTGTGTTTTTGATGAGTTTCTGTCCGTATTACTATCCCTCTCTTGCAGGGCAGGACATTTCAAACAGTGCTTCATCTTGGGCTATTGTTTCCTGGATGCTGTACTTCAAT
    Consensus GACTCTTGGCGTTGTGATATTTGTGTTTTTGATGAGTTTCTGTCCGTATTACTATCCCTCTCTTGCAGGGCAGGACATTTCAAACAGTGCTTCATCTTGGGCTATTGTTTCCTGGATGCTGTACTTCAAT

             1118111190    11200     11210     11220     11230     11240     11250     11260     11270     11280     11290     11300     11310
             |---------+---------+---------+---------+---------+---------+---------+---------+---------+---------+---------+---------+---------|
contig025313 TCTTGTCTGAATCCACTGATATATGCTTTTTTCTACCCGTGGTTTAGAAAAGCTATCTGGTTCATTGTCTCCCTGAAGATACTAGAGAAGGGCTCCTCTCAGGCAAATATACTTTAAAATAGCAAATTTA
  BriTAR.A002 TCTTGTCTGAATCCACTGATATATGCTTTTTTCTACCCGTGGTTTAGAAAAGCTATCTGGTTCATTGTCTCCCTGAAGATACTAGAGAAGGGCTCCTCTCAGGCAAATATACTTTAA
    Consensus TCTTGTCTGAATCCACTGATATATGCTTTTTTCTACCCGTGGTTTAGAAAAGCTATCTGGTTCATTGTCTCCCTGAAGATACTAGAGAAGGGCTCCTCTCAGGCAAATATACTTTAA.............

             1131111320    11330     11340     11350     11360     11370     11380     11390     11400     11410     11420     11430     11440
             |---------+---------+---------+---------+---------+---------+---------+---------+---------+---------+---------+---------+---------|
contig025313 TCCTGAACCAAATCAGTCTGTATGAGAGAATGAAACTGCTGATTATTATATGGGGCAGTAGCATGATGTTTTTTAATTATAGACTTATTCAATGAATCAAAGACCATCTGTCCCAAAGTCTGATTTCATA
  BriTAR.A002
    Consensus ..................................................................................................................................

             1144111450    11460     11470     11480     11490     11500     11510     11520     11530     11540     11550     11560     11570
```

```
  Consensus  ..................................................................................................................................

             2471   2480      2490      2500      2510      2520      2530      2540      2550      2560      2570      2580      2590      2600
             |--------+---------+---------+---------+---------+---------+---------+---------+---------+---------+---------+---------+---------|
contig029633 GAGTTGAGGTTGAGGAAGAGGTGATGAAAACAAAGACAAAAAGTCTCATCATTCATGTTTAAAAAAAGAAAAAGAAAAAAATGACGGTGTGTGTTTGAAGAAGATAAATGCAGACTCTCGAGCACAGTTG
  BriTAR.A003
  Consensus  ..................................................................................................................................

             2601   2610      2620      2630      2640      2650      2660      2670      2680      2690      2700      2710      2720      2730
             |--------+---------+---------+---------+---------+---------+---------+---------+---------+---------+---------+---------+---------|
contig029633 CAGGTCTTCCCTTTCTCCCCGATGGACGGCACTGGGGGTCCTCCCCTCTGCTTCCCTAACCTCAACTCCTCCTGCAGGCGGCTGCTGCGACCCACCTCCCAGGCCGCTCTTCTCTACACTCTGCTGGCTT
  BriTAR.A003                     ATGGACGGCACTGGGGGTCCTCCCCTCTGCTTCCCTAACCTCAACTCCTCCTGCAGGCGGCTGCTGCGACCCACCTCCCAGGCCGCTCTTCTCTACACTCTGCTGGCTT
  Consensus  .....................ATGGACGGCACTGGGGGTCCTCCCCTCTGCTTCCCTAACCTCAACTCCTCCTGCAGGCGGCTGCTGCGACCCACCTCCCAGGCCGCTCTTCTCTACACTCTGCTGGCTT

             2731   2740      2750      2760      2770      2780      2790      2800      2810      2820      2830      2840      2850      2860
             |--------+---------+---------+---------+---------+---------+---------+---------+---------+---------+---------+---------+---------|
contig029633 CAGTCTCACTGCTCACTGTGGTGCTCAAACTGCTTGTGGTCGTCTCCATCTCCCACTTCAAGCAGCTCCACACCCCGACCAACGCCCTGCTCCTGTCCCTGGCCATGTCCGACCTGCTGTTGGGGTTTCT
  BriTAR.A003 CAGTCTCACTGCTCACTGTGGTGCTCAAACTGCTTGTGGTCGTCTCCATCTCCCACTTCAAGCAGCTCCACACCCCGACCAACGCCCTGCTCCTGTCCCTGGCCATGTCCGACCTGCTGTTGGGGTTTCT
  Consensus  CAGTCTCACTGCTCACTGTGGTGCTCAAACTGCTTGTGGTCGTCTCCATCTCCCACTTCAAGCAGCTCCACACCCCGACCAACGCCCTGCTCCTGTCCCTGGCCATGTCCGACCTGCTGTTGGGGTTTCT

             2861   2870      2880      2890      2900      2910      2920      2930      2940      2950      2960      2970      2980      2990
             |--------+---------+---------+---------+---------+---------+---------+---------+---------+---------+---------+---------+---------|
contig029633 GGTGATGCCCATCGAGGGCCTGTGCTACATCGAGACGTGCTGGCTGCTGGGGAGGCTGATGTGTGCTCTCAGTCCTTATTTGTCTTACTGCCTTCTCTCTTTTTCTCTGGGCAGCATGGTGCTCATATCT
  BriTAR.A003 GGTGATGCCCATCGAGGGCCTGTGCTACATCGAGACGTGCTGGCTGCTGGGGAGGCTGATGTGTGCTCTCAGTCCTTATTTGTCTTACTGCCTTCTCTCTTTTTCTCTGGGCAGCATGGTGCTCATATCT
  Consensus  GGTGATGCCCATCGAGGGCCTGTGCTACATCGAGACGTGCTGGCTGCTGGGGAGGCTGATGTGTGCTCTCAGTCCTTATTTGTCTTACTGCCTTCTCTCTTTTTCTCTGGGCAGCATGGTGCTCATATCT

             2991   3000      3010      3020      3030      3040      3050      3060      3070      3080      3090      3100      3110      3120
             |--------+---------+---------+---------+---------+---------+---------+---------+---------+---------+---------+---------+---------|
contig029633 GTAGATCGCTATATAGCCATCTGTGACCCTCTGCTCTATTCCTCAAAGATCACAGTGAACAGAGTGAAGCTTTCAGTCTGTGTCTGCTGGGCCTGCTCTCTTCTCTACAATGGCTGCATTCTCATGGGGC
  BriTAR.A003 GTAGATCGCTATATAGCCATCTGTGACCCTCTGCTCTATTCCTCAAAGATCACAGTGAACAGAGTGAAGCTTTCAGTCTGTGTCTGCTGGGCCTGCTCTCTTCTCTACAATGGCTGCATTCTCATGGGGC
  Consensus  GTAGATCGCTATATAGCCATCTGTGACCCTCTGCTCTATTCCTCAAAGATCACAGTGAACAGAGTGAAGCTTTCAGTCTGTGTCTGCTGGGCCTGCTCTCTTCTCTACAATGGCTGCATTCTCATGGGGC

             3121   3130      3140      3150      3160      3170      3180      3190      3200      3210      3220      3230      3240      3250
             |--------+---------+---------+---------+---------+---------+---------+---------+---------+---------+---------+---------+---------|
contig029633 ACATAGGGTGGCCAGACAGGTTCAGCTCCTGTCACGGGGAGTGTGTGGTGTTCATCAGCCGAACTTCAGGAACAGTAGATTTCTTTTTGTCATTTCTTGGCCCATGTGCCGTAATGTTTGTTATGTACAT
  BriTAR.A003 ACATAGGGTGGCCAGACAGGTTCAGCTCCTGTCACGGGGAGTGTGTGGTGTTCATCAGCCGAACTTCAGGAACAGTAGATTTCTTTTTGTCATTTCTTGGCCCATGTGCCGTAATGTTTGTTATGTACAT
  Consensus  ACATAGGGTGGCCAGACAGGTTCAGCTCCTGTCACGGGGAGTGTGTGGTGTTCATCAGCCGAACTTCAGGAACAGTAGATTTCTTTTTGTCATTTCTTGGCCCATGTGCCGTAATGTTTGTTATGTACAT

             3251   3260      3270      3280      3290      3300      3310      3320      3330      3340      3350      3360      3370      3380
             |--------+---------+---------+---------+---------+---------+---------+---------+---------+---------+---------+---------+---------|
contig029633 GAGGGTGTTTGTGGTTGCTGTTTCTCAGGTGCGTGCCATTCAGTCGCAGGCAGCTGTGAGAGCAGTTCCAGCTGCTAAAAAATCAGAGTTGAAGGCAGCCAGGACACTCGGGATTTTGATAGCTGTGTTT
  BriTAR.A003 GAGGGTGTTTGTGGTTGCTGTTTCTCAGGTGCGTGCCATTCAGTCGCAGGCAGCTGTGAGAGCAGTTCCAGCTGCTAAAAAATCAGAGTTGAAGGCAGCCAGGACACTCGGGATTTTGATAGCTGTGTTT
  Consensus  GAGGGTGTTTGTGGTTGCTGTTTCTCAGGTGCGTGCCATTCAGTCGCAGGCAGCTGTGAGAGCAGTTCCAGCTGCTAAAAAATCAGAGTTGAAGGCAGCCAGGACACTCGGGATTTTGATAGCTGTGTTT

             3381   3390      3400      3410      3420      3430      3440      3450      3460      3470      3480      3490      3500      3510
             |--------+---------+---------+---------+---------+---------+---------+---------+---------+---------+---------+---------+---------|
contig029633 GTAATGTGCTTCTGCCCTTATTATTATCCTTCCCTTGCAGGTGTGGACACCTTTCTGAGCTTGCCTTCTTACGCCATGGTGTGTTGGATCATGCTAATAAATGCTTGTGTGAACCCTGTGATTTATGTTC
  BriTAR.A003 GTAATGTGCTTCTGCCCTTATTATTATCCTTCCCTTGCAGGTGTGGACACCTTTCTGAGCTTGCCTTCTTACGCCATGGTGTGTTGGATCATGCTAATAAATGCTTGTGTGAACCCTGTGATTTATGTTC
  Consensus  GTAATGTGCTTCTGCCCTTATTATTATCCTTCCCTTGCAGGTGTGGACACCTTTCTGAGCTTGCCTTCTTACGCCATGGTGTGTTGGATCATGCTAATAAATGCTTGTGTGAACCCTGTGATTTATGTTC

             3511   3520      3530      3540      3550      3560      3570      3580      3590      3600      3610      3620      3630      3640
             |--------+---------+---------+---------+---------+---------+---------+---------+---------+---------+---------+---------+---------|
contig029633 TGTTTTACCCCTGGTTTAGAAGAGCATTAAGATTTATTGTCACCCTCAGAATACTGCAGCCTCACTCCAGAGATGTCAAAATCCTGTAGGCAGCGCAATACAACTTAATCATTTTTCAATTTTCATGATA
  BriTAR.A003 TGTTTTACCCCTGGTTTAGAAGAGCATTAAGATTTATTGTCACCCTCAGAATACTGCAGCCTCACTCCAGAGATGTCAAAATCCTGTAG
  Consensus  TGTTTTACCCCTGGTTTAGAAGAGCATTAAGATTTATTGTCACCCTCAGAATACTGCAGCCTCACTCCAGAGATGTCAAAATCCTGTAG........................................

             3641   3650      3660      3670      3680      3690      3700      3710      3720      3730      3740      3750      3760      3770
             |--------+---------+---------+---------+---------+---------+---------+---------+---------+---------+---------+---------+---------|
contig029633 AATTTTCACCATTAAATGTGGGTGTTCAACTGCCCAGCATCACTTTGTGTTTAGTACATAATGAGCAGCAGCGCAGACCCGCGCAGGTAGTGACCCTGCATGCAGATCTCCTGTGCACCTGAAAATTGCT
  BriTAR.A003
  Consensus  ..................................................................................................................................
```

```
             4291 4300      4310      4320      4330      4340      4350      4360      4370      4380      4390      4400      4410      4420
             |--------+---------+---------+---------+---------+---------+---------+---------+---------+---------+---------+---------+---------|
contig084868 AGGAGTTTAGAGAAGGTGTGAGGATCAGTTATATTCTGCTCATAGTGCTGCCAGAGCTGCAGTTCTCTGCAGGTGTGTGGAGATGGAGACCCAGGACGAAGCAGAGCTCTGCTTTCCACAACTCTTCAAC
BriTARs.A014                                                                                   ATGGAGACCCAGGACGAAGCAGAGCTCTGCTTTCCACAACTCTTCAAC
   Consensus ..................................................................................ATGGAGACCCAGGACGAAGCAGAGCTCTGCTTTCCACAACTCTTCAAC

             4421 4430      4440      4450      4460      4470      4480      4490      4500      4510      4520      4530      4540      4550
             |--------+---------+---------+---------+---------+---------+---------+---------+---------+---------+---------+---------+---------|
contig084868 ACCTCCTGCAAGAAGCCTAAAACATCTCTGTCTCAAGTTTTGCTCCCTTACGTTGTGGTGTTTTCAGTCTCTCTGCTAACAGTGGCTCTCAACCTTCTCGTCATTGTCTCAGTCTCCCACTTCAGGCAGA
BriTARs.A014 ACCTCCTGCAAGAAGCCTAAAACATCTCTGTCTCAAGTTTTGCTCCCTTACGTTGTGGTGTTTTCAGTCTCTCTGCTAACAGTGGCTCTCAACCTT----------------------------------
   Consensus ACCTCCTGCAAGAAGCCTAAAACATCTCTGTCTCAAGTTTTGCTCCCTTACGTTGTGGTGTTTTCAGTCTCTCTGCTAACAGTGGCTCTCAACCTT..................................

             4551 4560      4570      4580      4590      4600      4610      4620      4630      4640      4650      4660      4670      4680
             |--------+---------+---------+---------+---------+---------+---------+---------+---------+---------+---------+---------+---------|
contig084868 GATTTACTTTTTTAAACTTCTGCTGTGGCTACTACTACTACAACTTACTACTAATTACAACTAAATTTAAGTTAATTTATATCTTATGGGTAGACTATTATCGGACAATAATGTGCAAATCTATATATTC
BriTARs.A014 ----------------------------------------------------------------------------------------------------------------------------------
   Consensus ..................................................................................................................................

             4681 4690      4700      4710      4720      4730      4740      4750      4760      4770      4780      4790      4800      4810
             |--------+---------+---------+---------+---------+---------+---------+---------+---------+---------+---------+---------+---------|
contig084868 TATCTTCTACCCTTTCAACTGTCTACACTTTCGGATAGGACAGCTGTAATAATTTTGTTTGTTTAGATTGTTTTAGTATAGAAATAATGACTTGTTGATATTTCTCTCTCTTCTTTCAGACAGCTCCACA
BriTARs.A014 ---------------------------------------------------------------------------------------------GTTGATATTTCTCTCTCTTCTTTCAGACAGCTCCACA
   Consensus .............................................................................................GTTGATATTTCTCTCTCTTCTTTCAGACAGCTCCACA

             4811 4820      4830      4840      4850      4860      4870      4880      4890      4900      4910      4920      4930      4940
             |--------+---------+---------+---------+---------+---------+---------+---------+---------+---------+---------+---------+---------|
contig084868 CACCCACTAACAGCCTCCTCCTCTCTCTGGCTGTCTCAGACTTTCTCATTGGTCTGTTGATGATGCCGGCAAAAATCCTAGGAGACACAGCTTGTTGGTTTCTTGGTCAGCTCACATGTTCTCTGTATAG
BriTARs.A014 CACCCACTAACAGCCTCCTCCTCTCTCTGGCTGTCTCAGACTTTCTCATTGGTCTGTTGATGATGCCGGCAAAAATCCTAGGAGACACAGCTTGTTGGTTTCTTGGTCAGCTCACATGTTCTCTGTATAG
   Consensus CACCCACTAACAGCCTCCTCCTCTCTCTGGCTGTCTCAGACTTTCTCATTGGTCTGTTGATGATGCCGGCAAAAATCCTAGGAGACACAGCTTGTTGGTTTCTTGGTCAGCTCACATGTTCTCTGTATAG

             4941 4950      4960      4970      4980      4990      5000      5010      5020      5030      5040      5050      5060      5070
             |--------+---------+---------+---------+---------+---------+---------+---------+---------+---------+---------+---------+---------|
contig084868 TTATATATGCTTCATCATTACCTCTGCCTCAGTGGGCATTATGGTGCTGATATCAGTCGACCGCTATGTGGCTATTTGTGACCCTCTGCATTACCCCACCAGAATCACAGAGAGAAGAGTGAAACTCTGC
BriTARs.A014 TTATATATGCTTCATCATTACCTCTGCCTCAGTGGGCATTATGGTGCTGATATCAGTCGACCGCTATGTGGCTATTTGTGACCCTCTGCATTACCCCACCAGAATCACAGAGAGAAGAGTGAAACTCTGC
   Consensus TTATATATGCTTCATCATTACCTCTGCCTCAGTGGGCATTATGGTGCTGATATCAGTCGACCGCTATGTGGCTATTTGTGACCCTCTGCATTACCCCACCAGAATCACAGAGAGAAGAGTGAAACTCTGC

             5071 5080      5090      5100      5110      5120      5130      5140      5150      5160      5170      5180      5190      5200
             |--------+---------+---------+---------+---------+---------+---------+---------+---------+---------+---------+---------+---------|
contig084868 GTCTGTCTGTGTTGGCTCTGCTCTGTTATTTACAACATACTATTTATAAAGAACGACCTGCTTCAAGGAGAACGACATACTTCCTGTTATGGAGAATGTGTATTTGTCATAGACTACATTGTAGGAACCA
BriTARs.A014 GTCTGTCTGTGTTGGCTCTGCTCTGTTATTTACAACATACTATTTATAAAGAACGACCTGCTTCAAGGAGAACGACATACTTCCTGTTATGGAGAATGTGTATTTGTCATAGACTACATTGTAGGAACCA
   Consensus GTCTGTCTGTGTTGGCTCTGCTCTGTTATTTACAACATACTATTTATAAAGAACGACCTGCTTCAAGGAGAACGACATACTTCCTGTTATGGAGAATGTGTATTTGTCATAGACTACATTGTAGGAACCA

             5201 5210      5220      5230      5240      5250      5260      5270      5280      5290      5300      5310      5320      5330
             |--------+---------+---------+---------+---------+---------+---------+---------+---------+---------+---------+---------+---------|
contig084868 CTGATATTGTTTTAACTTTTATTGCTCCAGTTACTGTCATCGTTGTTCTGTATATGAGAGTATTTGTGGTGGCTGTGTCTCAGGCCCGTGCCATGCGCTCTCATGTTACAGCTGTCACACTGCAGCTCTC
BriTARs.A014 CTGATATTGTTTTAACTTTTATTGCTCCAGTTACTGTCATCGTTGTTCTGTATATGAGAGTATTTGTGGTGGCTGTGTCTCAGGCCCGTGCCATGCGCTCTCATGTTACAGCTGTCACACTGCAGCTCTC
   Consensus CTGATATTGTTTTAACTTTTATTGCTCCAGTTACTGTCATCGTTGTTCTGTATATGAGAGTATTTGTGGTGGCTGTGTCTCAGGCCCGTGCCATGCGCTCTCATGTTACAGCTGTCACACTGCAGCTCTC

             5331 5340      5350      5360      5370      5380      5390      5400      5410      5420      5430      5440      5450      5460
             |--------+---------+---------+---------+---------+---------+---------+---------+---------+---------+---------+---------+---------|
contig084868 AGTGACTCTAACAGCAAAGAAATCAGAGATAAAAGCAGCCAGGACTCTGGGTGTTCTTGTTCTTGTGTTTCTATTAGGTTTCTGCCCATATTACATTGTTTCACTTTTAGGAAACGAGTTGTTCAACAGC
BriTARs.A014 AGTGACTCTAACAGCAAAGAAATCAGAGATAAAAGCAGCCAGGACTCTGGGTGTTCTTGTTCTTGTGTTTCTATTAGGTTTCTGCCCATATTACATTGTTTCACTTTTAGGAAACGAGTTGTTCAACAGC
   Consensus AGTGACTCTAACAGCAAAGAAATCAGAGATAAAAGCAGCCAGGACTCTGGGTGTTCTTGTTCTTGTGTTTCTATTAGGTTTCTGCCCATATTACATTGTTTCACTTTTAGGAAACGAGTTGTTCAACAGC

             5461 5470      5480      5490      5500      5510      5520      5530      5540      5550      5560      5570      5580      5590
             |--------+---------+---------+---------+---------+---------+---------+---------+---------+---------+---------+---------+---------|
contig084868 TCATCTGCATCCATTGCGATTTATCTGTATTATTTTAACTCCTGTCTAAATCCTTTGATTTACGCTATGTTCTACCCCTGGTTTAGAAAAGCTGTGAAATTCGTTGTCACTCTACAGATACTGCAGCCTG
BriTARs.A014 TCATCTGCATCCATTGCGATTTATCTGTATTATTTTAACTCCTGTCTAAATCCTTTGATTTACGCTATGTTCTACCCCTGGTTTAGAAAAGCTGTGAAATTCGTTGTCACTCTACAGATACTGCAGCCTG
   Consensus TCATCTGCATCCATTGCGATTTATCTGTATTATTTTAACTCCTGTCTAAATCCTTTGATTTACGCTATGTTCTACCCCTGGTTTAGAAAAGCTGTGAAATTCGTTGTCACTCTACAGATACTGCAGCCTG

             5591 5600      5610      5620      5630      5640      5650      5660      5670      5680      5690      5700      5710      5720
             |--------+---------+---------+---------+---------+---------+---------+---------+---------+---------+---------+---------+---------|
contig084868 GCTCCTGTGAGGTCAGCATACTGTAGAAGAAAAGAGATCTTTCCAAAAAGATGTTTATGATTAAAACTGAACAACCAGCTGTTAAAACCCTTTCTGTCTCTTTAAGAGAACAAGGAGAATAAGGTGTATTG
BriTARs.A014 GCTCCTGTGAGGTCAGCATACTGTAG
   Consensus GCTCCTGTGAGGTCAGCATACTGTAG........................................................................................................
```

```
              1040110410    10420     10430     10440     10450     10460     10470     10480     10490     10500     10510     10520     10530
              |--------+---------+---------+---------+---------+---------+---------+---------+---------+---------+---------+---------+---------|
contig084876  ACATTGAGAAAAAGACGAGGGGCGGAGTGAGAAGGGGTGAGTTATATAACTAATTCCGTTTGTAATGCTACATAGAGCTACAGGTCTGTCTGAAAATGGATACACAGGACGTGGCAGAGCTCTGTTTTCC
BriTARs.A015                                                                                                 ATGGATACACAGGACGTGGCAGAGCTCTGTTTTCC
   Consensus  ...............................................................................................ATGGATACACAGGACGTGGCAGAGCTCTGTTTTCC

              1053110540    10550     10560     10570     10580     10590     10600     10610     10620     10630     10640     10650     10660
              |--------+---------+---------+---------+---------+---------+---------+---------+---------+---------+---------+---------+---------|
contig084876  ACAACTCTTCAACACCTCCTGCAAGAAACCTATAACTCCTCTGTCAGAATTTGTGTTCCTTCATGTTGTGTTGTCCTCCATCTCGCTGCTAACTGTGACTCTCAACCTGCTCGTCATCATCTCAGTCTCC
BriTARs.A015  ACAACTCTTCAACACCTCCTGCAAGAAACCTATAACTCCTCTGTCAGAATTTGTGTTCCTTCATGTTGTGTTGTCCTCCATCTCGCTGCTAACTGTGACTCTCAACCTGCTCGTCATCATCTCAGTCTCC
   Consensus  ACAACTCTTCAACACCTCCTGCAAGAAACCTATAACTCCTCTGTCAGAATTTGTGTTCCTTCATGTTGTGTTGTCCTCCATCTCGCTGCTAACTGTGACTCTCAACCTGCTCGTCATCATCTCAGTCTCC

              1066110670    10680     10690     10700     10710     10720     10730     10740     10750     10760     10770     10780     10790
              |--------+---------+---------+---------+---------+---------+---------+---------+---------+---------+---------+---------+---------|
contig084876  CACTACAGGCAGAGTTGTTGTTGTTTTTTTAATAGCTGTGTGTAGCTTATACATTCTGTGAGCTAATTGACTTAAGATGTTTTTGTGACCTCAGTGGTGTATACAATTAAACCATCTATATAGAAGTGGT
BriTARs.A015  CACTACAGGCAG----------------------------------------------------------------------------------------------------------------------
   Consensus  CACTACAGGCAG..........................................................................................................................

              1079110800    10810     10820     10830     10840     10850     10860     10870     10880     10890     10900     10910     10920
              |--------+---------+---------+---------+---------+---------+---------+---------+---------+---------+---------+---------+---------|
contig084876  CGATTAGAAATGATCAAAACTGTTTTTCTCTTACCAAGAGCTAATGTTTTATTGTTTGTTGGGTTGCTTTAATTTAGCGATTTGAGGACTGTTAATTTAATAATTTTTGTGTCTTCTTGCAGGCAGCTTC
BriTARs.A015  ------------------------------------------------------------------------------------------------------------------------------CTTC
   Consensus  ..............................................................................................................................CTTC

              1092110930    10940     10950     10960     10970     10980     10990     11000     11010     11020     11030     11040     11050
              |--------+---------+---------+---------+---------+---------+---------+---------+---------+---------+---------+---------+---------|
contig084876  ATACACCCACTAACATCCTCCTCCTCTCTCTGGCTGTCTCAGACTTTCTTGTTGGTCTCCTGTTGATGCCTGGAGAAATCCTCCGAAATACAGCCTGCTGGTTTCTCGGTGACCTCACCTGTTTTATGTA
BriTARs.A015  ATACACCCACTAACATCCTCCTCCTCTCTCTGGCTGTCTCAGACTTTCTTGTTGGTCTCCTGTTGATGCCTGGAGAAATCCTCCGAAATACAGCCTGCTGGTTTCTCGGTGACCTCACCTGTTTTATGTA
   Consensus  ATACACCCACTAACATCCTCCTCCTCTCTCTGGCTGTCTCAGACTTTCTTGTTGGTCTCCTGTTGATGCCTGGAGAAATCCTCCGAAATACAGCCTGCTGGTTTCTCGGTGACCTCACCTGTTTTATGTA

              1105111060    11070     11080     11090     11100     11110     11120     11130     11140     11150     11160     11170     11180
              |--------+---------+---------+---------+---------+---------+---------+---------+---------+---------+---------+---------+---------|
contig084876  CGATTATATGTCTTTAATTGTTACCTCTACCTCAGTGGGAGACATGGTGTTAATATCAATTGACCGCTATGTGGCTCTTTGTGACCCTCTGCATTACCCCACCAGAATCACAGACAGAAGAGTGAAACTC
BriTARs.A015  CGATTATATGTCTTTAATTGTTACCTCTACCTCAGTGGGAGACATGGTGTTAATATCAATTGACCGCTATGTGGCTCTTTGTGACCCTCTGCATTACCCCACCAGAATCACAGACAGAAGAGTGAAACTC
   Consensus  CGATTATATGTCTTTAATTGTTACCTCTACCTCAGTGGGAGACATGGTGTTAATATCAATTGACCGCTATGTGGCTCTTTGTGACCCTCTGCATTACCCCACCAGAATCACAGACAGAAGAGTGAAACTC

              1118111190    11200     11210     11220     11230     11240     11250     11260     11270     11280     11290     11300     11310
              |--------+---------+---------+---------+---------+---------+---------+---------+---------+---------+---------+---------+---------|
contig084876  TCTGTCTGTCTGTGTTGGCTCTCTTCTGTTTTCTATAGCAGCCTGTTTGTAAAGGATGATCTAACTCATTCAGGGAAGCATAATTCCTGCTATGGAGAATGTACAATTGTTGTTGACTTAATTACAGGAA
BriTARs.A015  TCTGTCTGTCTGTGTTGGCTCTCTTCTGTTTTCTATAGCAGCCTGTTTGTAAAGGATGATCTAACTCATTCAGGGAAGCATAATTCCTGCTATGGAGAATGTACAATTGTTGTTGACTTAATTACAGGAA
   Consensus  TCTGTCTGTCTGTGTTGGCTCTCTTCTGTTTTCTATAGCAGCCTGTTTGTAAAGGATGATCTAACTCATTCAGGGAAGCATAATTCCTGCTATGGAGAATGTACAATTGTTGTTGACTTAATTACAGGAA

              1131111320    11330     11340     11350     11360     11370     11380     11390     11400     11410     11420     11430     11440
              |--------+---------+---------+---------+---------+---------+---------+---------+---------+---------+---------+---------+---------|
contig084876  CGATTGACCTTCTTTTAACCTTTTTTGTTCCAGTTACTGTCATTGTAGTTCTGTATCTGAGAGTATTTGTGGTGGCTGTGTCTCAGGCTCGCGCCATGCGCTCTCATGTTACAGCTGCTGCTCTGCAGCT
BriTARs.A015  CGATTGACCTTCTTTTAACCTTTTTTGTTCCAGTTACTGTCATTGTAGTTCTGTATCTGAGAGTATTTGTGGTGGCTGTGTCTCAGGCTCGCGCCATGCGCTCTCATGTTACAGCTGCTGCTCTGCAGCT
   Consensus  CGATTGACCTTCTTTTAACCTTTTTTGTTCCAGTTACTGTCATTGTAGTTCTGTATCTGAGAGTATTTGTGGTGGCTGTGTCTCAGGCTCGCGCCATGCGCTCTCATGTTACAGCTGCTGCTCTGCAGCT

              1144111450    11460     11470     11480     11490     11500     11510     11520     11530     11540     11550     11560     11570
              |--------+---------+---------+---------+---------+---------+---------+---------+---------+---------+---------+---------+---------|
contig084876  TTCAGTGACTCTAACAACAAAGAGATCAGAGTTAAAAGCAGCCAGGACTCTGGGTGTTCTTGTAGTTGTGTTTCTACTGTGTTTCTGCCCTTATTATTGTGTTACTCTTGCCAGGGACGACCTACTCAAT
BriTARs.A015  TTCAGTGACTCTAACAACAAAGAGATCAGAGTTAAAAGCAGCCAGGACTCTGGGTGTTCTTGTAGTTGTGTTTCTACTGTGTTTCTGCCCTTATTATTGTGTTACTCTTGCCAGGGACGACCTACTCAAT
   Consensus  TTCAGTGACTCTAACAACAAAGAGATCAGAGTTAAAAGCAGCCAGGACTCTGGGTGTTCTTGTAGTTGTGTTTCTACTGTGTTTCTGCCCTTATTATTGTGTTACTCTTGCCAGGGACGACCTACTCAAT

              1157111580    11590     11600     11610     11620     11630     11640     11650     11660     11670     11680     11690     11700
              |--------+---------+---------+---------+---------+---------+---------+---------+---------+---------+---------+---------+---------|
contig084876  AGTTCATCTGTATCCTTTTTGCTCTATCTGTTCTATTTTAACTCATGTCTAAACCCTTTGATCTATGCACTGCTCTACCCCTGGTTTAGAAAAGCTGTGAAACTCATAATCTCTTTACACATACTGCAGC
BriTARs.A015  AGTTCATCTGTATCCTTTTTGCTCTATCTGTTCTATTTTAACTCATGTCTAAACCCTTTGATCTATGCACTGCTCTACCCCTGGTTTAGAAAAGCTGTGAAACTCATAATCTCTTTACACATACTGCAGC
   Consensus  AGTTCATCTGTATCCTTTTTGCTCTATCTGTTCTATTTTAACTCATGTCTAAACCCTTTGATCTATGCACTGCTCTACCCCTGGTTTAGAAAAGCTGTGAAACTCATAATCTCTTTACACATACTGCAGC

              1170111710    11720     11730     11740     11750     11760     11770     11780     11790     11800     11810     11820     11830
              |--------+---------+---------+---------+---------+---------+---------+---------+---------+---------+---------+---------+---------|
contig084876  CTGGCTCCTGTGAGATTAGCATTCTGTAAAAAAAATAAAATAAACAAATAAAAGGAACACTGAACAATTGTTGAGTTATTTTGTTGCCCTACTGGTTGGCTACTTTTGGGCTTCCATTTGTGTTATTCTG
BriTARs.A015  CTGGCTCCTGTGAGATTAGCATTCTGTAA
   Consensus  CTGGCTCCTGTGAGATTAGCATTCTGTAA.....................................................................................................

              1183111840    11850     11860     11870     11880     11890     11900     11910     11920     11930     11940     11950     11960
              |--------+---------+---------+---------+---------+---------+---------+---------+---------+---------+---------+---------+---------|
contig084876  TGGCACTGGAAATGCTGCTGTGTCAGTATCACTGTGTCATCGATGACAGTAACTTGTTGGAAAAGAAGGATGGAAAATATTTTTCATGTTGGCACTATTAAATGCTGCTACCTGTCATCTGAACTCACCC
BriTARs.A015
   Consensus  ..................................................................................................................................
```

```
             3901  3910      3920      3930      3940      3950      3960      3970      3980      3990      4000      4010      4020      4030
             |--------+---------+---------+---------+---------+---------+---------+---------+---------+---------+---------+---------+---------|
contig084880 AGAGGTTGTGTGCACAAAAAGAGGGAGGATATCAGTCTCCAGGGAGCACAACAGTGGATGGGAGGTATGGTAGAAGGAGATAAATTTGTTGTATGACAGAAAGTTCGGATTAGACAGCTGCAGGAACCTG
BriTARs.A016
   Consensus ..................................................................................................................................

             4031  4040      4050      4060      4070      4080      4090      4100      4110      4120      4130      4140      4150      4160
             |--------+---------+---------+---------+---------+---------+---------+---------+---------+---------+---------+---------+---------|
contig084880 ATGATGGAGATCCAGATGCATCCAGAGGCAGAGCTCTGTTTTCCAGAGCTACTGAACAGTTCCTGCAGGAAGCCAACACTTCACTGGTCCAAAACGGTGCTCCTGAACGTTGGGCTTTCGTCCATCTCTC
BriTARs.A016    ATGGAGATCCAGATGCATCCAGAGGCAGAGCTCTGTTTTCCAGAGCTACTGAACAGTTCCTGCAGGAAGCCAACACTTCACTGGTCCAAAACGGTGCTCCTGAACGTTGGGCTTTCGTCCATCTCTC
   Consensus ...ATGGAGATCCAGATGCATCCAGAGGCAGAGCTCTGTTTTCCAGAGCTACTGAACAGTTCCTGCAGGAAGCCAACACTTCACTGGTCCAAAACGGTGCTCCTGAACGTTGGGCTTTCGTCCATCTCTC

             4161  4170      4180      4190      4200      4210      4220      4230      4240      4250      4260      4270      4280      4290
             |--------+---------+---------+---------+---------+---------+---------+---------+---------+---------+---------+---------+---------|
contig084880 TGATCACTGCTGCTCTTAATCTGTTCATCATCATCTCGGTCTCCCACTTCAGGCAGAGATTAACTTCTCAGATGAAACTAAAGTTCAACTTTTAGAAAATCAAGTTTAGTAATTAGTTAGTAAATTAGTT
BriTARs.A016 TGATCACTGCTGCTCTTAATCTGTTCATCATCATCTCGGTCTCCCACTTCAGGCAG--------------------------------------------------------------------------
   Consensus TGATCACTGCTGCTCTTAATCTGTTCATCATCATCTCGGTCTCCCACTTCAGGCAG..........................................................................

             4291  4300      4310      4320      4330      4340      4350      4360      4370      4380      4390      4400      4410      4420
             |--------+---------+---------+---------+---------+---------+---------+---------+---------+---------+---------+---------+---------|
contig084880 AAGTAATTAATGAATGTGTGTACTTCATCATGACTGTTTTAGTTGCTTAATTGCACCATGCCTCTAATGGAACATCTTCATTGAAGATATTTTGACATGACTAATGTGTAATTTATGATTTTCTGTCTCC
BriTARs.A016 ----------------------------------------------------------------------------------------------------------------------------------
   Consensus ..................................................................................................................................

             4421  4430      4440      4450      4460      4470      4480      4490      4500      4510      4520      4530      4540      4550
             |--------+---------+---------+---------+---------+---------+---------+---------+---------+---------+---------+---------+---------|
contig084880 CTGCAGGCAGCTCCACACTCCCAGTAACATCATCATCCTCTCTCTGGCTGTCTCTGACTTTTTTGTCTGTTTTTTGTTGATGCCGGTAGAAATCTTCAAAAACACAGCCTGCTGGGTATTTGGTGATCTC
BriTARs.A016 ----------CTCCACACTCCCAGTAACATCATCATCCTCTCTCTGGCTGTCTCTGACTTTTTTGTCTGTTTTTTGTTGATGCCGGTAGAAATCTTCAAAAACACAGCCTGCTGGGTATTTGGTGATCTC
   Consensus ..........CTCCACACTCCCAGTAACATCATCATCCTCTCTCTGGCTGTCTCTGACTTTTTTGTCTGTTTTTTGTTGATGCCGGTAGAAATCTTCAAAAACACAGCCTGCTGGGTATTTGGTGATCTC

             4551  4560      4570      4580      4590      4600      4610      4620      4630      4640      4650      4660      4670      4680
             |--------+---------+---------+---------+---------+---------+---------+---------+---------+---------+---------+---------+---------|
contig084880 ATGTGTTCACTTTATACTTATCTGAGCTGCATTCTTATCAATGCTTCGTTTGAAATGATTATTCTCGTATCAATTGACCGCTATGTGGCTATTTGTGACCCTCTGCATTACCCCACCAGAATCACTGTGC
BriTARs.A016 ATGTGTTCACTTTATACTTATCTGAGCTGCATTCTTATCAATGCTTCGTTTGAAATGATTATTCTCGTATCAATTGACCGCTATGTGGCTATTTGTGACCCTCTGCATTACCCCACCAGAATCACTGTGC
   Consensus ATGTGTTCACTTTATACTTATCTGAGCTGCATTCTTATCAATGCTTCGTTTGAAATGATTATTCTCGTATCAATTGACCGCTATGTGGCTATTTGTGACCCTCTGCATTACCCCACCAGAATCACTGTGC

             4681  4690      4700      4710      4720      4730      4740      4750      4760      4770      4780      4790      4800      4810
             |--------+---------+---------+---------+---------+---------+---------+---------+---------+---------+---------+---------+---------|
contig084880 CGAGAGTCAAACTCTGTGTTTTTCTGTGTTGGTTTTATGCTATTTTGTACAACATCATCTATACAAAGCATGCCCTGATAAACCCAGGCAGGTATGGTTCCTGCTATGGAGAGTGTGTTTTTGTTGTTGA
BriTARs.A016 CGAGAGTCAAACTCTGTGTTTTTCTGTGTTGGTTTTATGCTATTTTGTACAACATCATCTATACAAAGCATGCCCTGATAAACCCAGGCAGGTATGGTTCCTGCTATGGAGAGTGTGTTTTTGTTGTTGA
   Consensus CGAGAGTCAAACTCTGTGTTTTTCTGTGTTGGTTTTATGCTATTTTGTACAACATCATCTATACAAAGCATGCCCTGATAAACCCAGGCAGGTATGGTTCCTGCTATGGAGAGTGTGTTTTTGTTGTTGA

             4811  4820      4830      4840      4850      4860      4870      4880      4890      4900      4910      4920      4930      4940
             |--------+---------+---------+---------+---------+---------+---------+---------+---------+---------+---------+---------+---------|
contig084880 TGATATTATTGGAATTGTTGACTTTGTTGTATCTCTTATAGTTCCAGTTACGATCATCGTAGTTCTGTATACGAGAGTGTTTGTGGTGGTTGTGTCTCAGGCTCGTGCCATGCGCTCTCATGTTACAGCT
BriTARs.A016 TGATATTATTGGAATTGTTGACTTTGTTGTATCTCTTATAGTTCCAGTTACGATCATCGTAGTTCTGTATACGAGAGTGTTTGTGGTGGTTGTGTCTCAGGCTCGTGCCATGCGCTCTCATGTTACAGCT
   Consensus TGATATTATTGGAATTGTTGACTTTGTTGTATCTCTTATAGTTCCAGTTACGATCATCGTAGTTCTGTATACGAGAGTGTTTGTGGTGGTTGTGTCTCAGGCTCGTGCCATGCGCTCTCATGTTACAGCT

             4941  4950      4960      4970      4980      4990      5000      5010      5020      5030      5040      5050      5060      5070
             |--------+---------+---------+---------+---------+---------+---------+---------+---------+---------+---------+---------+---------|
contig084880 GTTACACTTCAGCGTCCACTGAATCAAGCAAACAAATCTGAGCTGAAAGCAGCCAGGAACCTTGGGATTCTTGTAGTAGTTTTTCTGGCGTGCTACTGTCCATTTTACTGCTACTTTTTTCTTGCAGGCA
BriTARs.A016 GTTACACTTCAGCGTCCACTGAATCAAGCAAACAAATCTGAGCTGAAAGCAGCCAGGAACCTTGGGATTCTTGTAGTAGTTTTTCTGGCGTGCTACTGTCCATTTTACTGCTACTTTTTTCTTGCAGGCA
   Consensus GTTACACTTCAGCGTCCACTGAATCAAGCAAACAAATCTGAGCTGAAAGCAGCCAGGAACCTTGGGATTCTTGTAGTAGTTTTTCTGGCGTGCTACTGTCCATTTTACTGCTACTTTTTTCTTGCAGGCA

             5071  5080      5090      5100      5110      5120      5130      5140      5150      5160      5170      5180      5190      5200
             |--------+---------+---------+---------+---------+---------+---------+---------+---------+---------+---------+---------+---------|
contig084880 ATGAAGTTAATGCTTCTTCTGCATCTTCTATCCTCATTGTTTATTATTTTAACTCTTGCCTAAACCCATTAATGTATGCACTGTTTTACCCCTGGTTTAGAAATGCTGTTAAACTTATCATCACTCTGCA
BriTARs.A016 ATGAAGTTAATGCTTCTTCTGCATCTTCTATCCTCATTGTTTATTATTTTAACTCTTGCCTAAACCCATTAATGTATGCACTGTTTTACCCCTGGTTTAGAAATGCTGTTAAACTTATCATCACTCTGCA
   Consensus ATGAAGTTAATGCTTCTTCTGCATCTTCTATCCTCATTGTTTATTATTTTAACTCTTGCCTAAACCCATTAATGTATGCACTGTTTTACCCCTGGTTTAGAAATGCTGTTAAACTTATCATCACTCTGCA

             5201  5210      5220      5230      5240      5250      5260      5270      5280      5290      5300      5310      5320      5330
             |--------+---------+---------+---------+---------+---------+---------+---------+---------+---------+---------+---------+---------|
contig084880 GATATTAAAGGCTAACAGCAGTGAGATCAACATACTACAGAGATAATGCGGAGTGACTTGACTAACATCTGCTATATAAAGATTTCAAATCCAGTAAATGAATGTGTCCATAGTCTGGAAATATAAAACT
BriTARs.A016 GATATTAAAGGCTAACAGCAGTGAGATCAACATACTACAGAGATAA
   Consensus GATATTAAAGGCTAACAGCAGTGAGATCAACATACTACAGAGATAA....................................................................................

             5331  5340      5350      5360      5370      5380      5390      5400      5410      5420      5430      5440      5450      5460
             |--------+---------+---------+---------+---------+---------+---------+---------+---------+---------+---------+---------+---------|
contig084880 GCTGTCTTCTATGTATTGAAATGTGTATGAATTCACTACAATAAAATTTGTTTAAATGTTAACATTTAAGTTAACTTGAACATGAAGAGCATTCAGCTTGAGCCACTTTCTATTTTATAACTAATAATGG
BriTARs.A016
   Consensus ..................................................................................................................................
```

```
             1171   1180      1190      1200      1210      1220      1230      1240      1250      1260      1270      1280      1290      1300
             |--------+---------+---------+---------+---------+---------+---------+---------+---------+---------+---------+---------+---------|
contig084886 GATAAATCCCCTATGTGACAAGAAGCTCTGATCAGAGAGCTGCAGGAGCTGCAGCTGTATGATGATGGAGATGCAGAAAGAAGCCGAGCTCTGTTTCCCACAACTCATCAACAGTTCCTGCAGGAAGCCA
BriTARs.A017                                                                       ATGCAGAAAGAAGCCGAGCTCTGTTTCCCACAACTCATCAACAGTTCCTGCAGGAAGCCA
   Consensus ......................................................................ATGCAGAAAGAAGCCGAGCTCTGTTTCCCACAACTCATCAACAGTTCCTGCAGGAAGCCA

             1301   1310      1320      1330      1340      1350      1360      1370      1380      1390      1400      1410      1420      1430
             |--------+---------+---------+---------+---------+---------+---------+---------+---------+---------+---------+---------+---------|
contig084886 ACACTTCACTGGTCCAAAGCTGTGCTGCTGAACATTGTGCTGTCCTGCATCTCTCTGCTCACTGCTGCTCTAAACCTTCTCGTCATCATCTCAATCTCTTACTTCAGGCAGAGATTAACTTTTCAGCTGA
BriTARs.A017 ACACTTCACTGGTCCAAAGCTGTGCTGCTGAACATTGTGCTGTCCTGCATCTCTCTGCTCACTGCTGCTCTAAACCTTCTCGTCATCATCTCAATCTCTTACTTCAGGCAG-------------------
   Consensus ACACTTCACTGGTCCAAAGCTGTGCTGCTGAACATTGTGCTGTCCTGCATCTCTCTGCTCACTGCTGCTCTAAACCTTCTCGTCATCATCTCAATCTCTTACTTCAGGCAG...................

             1431   1440      1450      1460      1470      1480      1490      1500      1510      1520      1530      1540      1550      1560
             |--------+---------+---------+---------+---------+---------+---------+---------+---------+---------+---------+---------+---------|
contig084886 AATTGAAGTTTAAGTCTTAAAAACTTTTAAAATAATTATTTAGTGATGAATGACATGAATATGGCACATCATTGGTGTATTTTAACCACACTGTAATGTTTCATTTGCTTGGCCTTACTGATGGAACATT
BriTARs.A017 ----------------------------------------------------------------------------------------------------------------------------------
   Consensus ..................................................................................................................................

             1561   1570      1580      1590      1600      1610      1620      1630      1640      1650      1660      1670      1680      1690
             |--------+---------+---------+---------+---------+---------+---------+---------+---------+---------+---------+---------+---------|
contig084886 TTTACTAATGGAATTAGTGAAGACATTTTTACATTATTAATGTGTAATATATTCTTTTTTCTCTCTCCTTAGGCAGCTTCACACACCCACTAACATCCTCCTCCTTTCTCTGGCTGTCTCAGACTTTCTC
BriTARs.A017 ----------------------------------------------------------------------------CTTCACACACCCACTAACATCCTCCTCCTTTCTCTGGCTGTCTCAGACTTTCTC
   Consensus ............................................................................CTTCACACACCCACTAACATCCTCCTCCTTTCTCTGGCTGTCTCAGACTTTCTC

             1691   1700      1710      1720      1730      1740      1750      1760      1770      1780      1790      1800      1810      1820
             |--------+---------+---------+---------+---------+---------+---------+---------+---------+---------+---------+---------+---------|
contig084886 ACTGGTCTCCTTATGCTGCCACTAGAAATAATTAGAAACACGGCCTGTTGGGTACTTGGTGATCTTATGTGTTCTGTTTATACTTGTCTGACAACCGGTCTTTTCTGTGTTTCAACTTGGAACGTAGTTC
BriTARs.A017 ACTGGTCTCCTTATGCTGCCACTAGAAATAATTAGAAACACGGCCTGTTGGGTACTTGGTGATCTTATGTGTTCTGTTTATACTTGTCTGACAACCGGTCTTTTCTGTGTTTCAACTTGGAACGTAGTTC
   Consensus ACTGGTCTCCTTATGCTGCCACTAGAAATAATTAGAAACACGGCCTGTTGGGTACTTGGTGATCTTATGTGTTCTGTTTATACTTGTCTGACAACCGGTCTTTTCTGTGTTTCAACTTGGAACGTAGTTC

             1821   1830      1840      1850      1860      1870      1880      1890      1900      1910      1920      1930      1940      1950
             |--------+---------+---------+---------+---------+---------+---------+---------+---------+---------+---------+---------+---------|
contig084886 TCATTTCAGTTGACCGCTATGTAGCTATTTGTCACCCTCTTCATTACCCCACCAGAATTACCGTGGCAAGAGTCAAACTCTGTGTTTGTCTGTGTTGGTTTTATTCAACCTTCTACGGCAGTCTTTTAAC
BriTARs.A017 TCATTTCAGTTGACCGCTATGTAGCTATTTGTCACCCTCTTCATTACCCCACCAGAATTACCGTGGCAAGAGTCAAACTCTGTGTTTGTCTGTGTTGGTTTTATTCAACCTTCTACGGCAGTCTTTTAAC
   Consensus TCATTTCAGTTGACCGCTATGTAGCTATTTGTCACCCTCTTCATTACCCCACCAGAATTACCGTGGCAAGAGTCAAACTCTGTGTTTGTCTGTGTTGGTTTTATTCAACCTTCTACGGCAGTCTTTTAAC

             1951   1960      1970      1980      1990      2000      2010      2020      2030      2040      2050      2060      2070      2080
             |--------+---------+---------+---------+---------+---------+---------+---------+---------+---------+---------+---------+---------|
contig084886 AAAGGATGTTCTAATTAAGCCGGCCAGGTATAATTCCTGTTATGGAGAGTGTGTGTTTGTCATTAATGATATTGCAGGGTTTATTGATCTTGTGTTATCTTTTATTGTTCCAGTTTCTGTCATCATAGTT
BriTARs.A017 AAAGGATGTTCTAATTAAGCCGGCCAGGTATAATTCCTGTTATGGAGAGTGTGTGTTTGTCATTAATGATATTGCAGGGTTTATTGATCTTGTGTTATCTTTTATTGTTCCAGTTTCTGTCATCATAGTT
   Consensus AAAGGATGTTCTAATTAAGCCGGCCAGGTATAATTCCTGTTATGGAGAGTGTGTGTTTGTCATTAATGATATTGCAGGGTTTATTGATCTTGTGTTATCTTTTATTGTTCCAGTTTCTGTCATCATAGTT

             2081   2090      2100      2110      2120      2130      2140      2150      2160      2170      2180      2190      2200      2210
             |--------+---------+---------+---------+---------+---------+---------+---------+---------+---------+---------+---------+---------|
contig084886 CTGTATATGAGAGTGTTTGTGGTGGCTGTGTCTCAAGCTCGTGCCATGCGCTCTCGTGTTACAGCTGTCACACTTCAGCGTTCAAGCAATCAAGCAAAGAAATCTGAACTGAAAGCAGCCAGGACTCTTG
BriTARs.A017 CTGTATATGAGAGTGTTTGTGGTGGCTGTGTCTCAAGCTCGTGCCATGCGCTCTCGTGTTACAGCTGTCACACTTCAGCGTTCAAGCAATCAAGCAAAGAAATCTGAACTGAAAGCAGCCAGGACTCTTG
   Consensus CTGTATATGAGAGTGTTTGTGGTGGCTGTGTCTCAAGCTCGTGCCATGCGCTCTCGTGTTACAGCTGTCACACTTCAGCGTTCAAGCAATCAAGCAAAGAAATCTGAACTGAAAGCAGCCAGGACTCTTG

             2211   2220      2230      2240      2250      2260      2270      2280      2290      2300      2310      2320      2330      2340
             |--------+---------+---------+---------+---------+---------+---------+---------+---------+---------+---------+---------+---------|
contig084886 GAGTTCTTGTAGTTGGGTTTCTTGCATGTTACCTTCCATTTTACTGTTACTCTCTTATTGAAGTAAATGTGATCAATGACTCATCTACATTTTTTCTGATCATTGTGTTGTACTTTAACTCGTGTCTAAA
BriTARs.A017 GAGTTCTTGTAGTTGGGTTTCTTGCATGTTACCTTCCATTTTACTGTTACTCTCTTATTGAAGTAAATGTGATCAATGACTCATCTACATTTTTTCTGATCATTGTGTTGTACTTTAACTCGTGTCTAAA
   Consensus GAGTTCTTGTAGTTGGGTTTCTTGCATGTTACCTTCCATTTTACTGTTACTCTCTTATTGAAGTAAATGTGATCAATGACTCATCTACATTTTTTCTGATCATTGTGTTGTACTTTAACTCGTGTCTAAA

             2341   2350      2360      2370      2380      2390      2400      2410      2420      2430      2440      2450      2460      2470
             |--------+---------+---------+---------+---------+---------+---------+---------+---------+---------+---------+---------+---------|
contig084886 CCCACTGATGTATGCCCTTTTTTACTCTTGGTTTAGAAATGCTGTTAAACTTATTATCACTCTGCAGATTTTTAAGCATGACACTAGTGAAGCAAACATGTTTTAAAAAGATAAGGAAACCCACTCTACT
BriTARs.A017 CCCACTGATGTATGCCCTTTTTTACTCTTGGTTTAGAAATGCTGTTAAACTTATTATCACTCTGCAGATTTTTAAGCATGACACTAGTGAAGCAAACATGTTTTAA
   Consensus CCCACTGATGTATGCCCTTTTTTACTCTTGGTTTAGAAATGCTGTTAAACTTATTATCACTCTGCAGATTTTTAAGCATGACACTAGTGAAGCAAACATGTTTTAA........................
```

```
             521    530       540       550       560       570       580       590       600       610       620       630       640       650
             |--------+---------+---------+---------+---------+---------+---------+---------+---------+---------+---------+---------+---------|
contig084887 AAAGGAAGAAGAGAAAAGGAAGGTCATCATGTTTTAGACTGAAAGAGAATGGTGGGAGGAGTGAGATAAATTAGCTGTATGAGAAGAGTTTCTAATTACAGAGCTGCAGGTGTCTGATGATGGAGATACC
BriTARs.A018                                                                                                                        ATGGAGATACC
   Consensus .......................................................................................................................ATGGAGATACC

             651    660       670       680       690       700       710       720       730       740       750       760       770       780
             |--------+---------+---------+---------+---------+---------+---------+---------+---------+---------+---------+---------+---------|
contig084887 GAAAGGAGTCGAGCTCTGTTTCCCACAACTCCTCAACAGCTCCTGCAGGAAGCCGACACTTCACTGGTCCAAAGCTGTGCTCCTGAACATTGTGCTCTCATGTATCTCTCTGCTCACTGCTGCTCTAAAC
BriTARs.A018 GAAAGGAGTCGAGCTCTGTTTCCCACAACTCCTCAACAGCTCCTGCAGGAAGCCGACACTTCACTGGTCCAAAGCTGTGCTCCTGAACATTGTGCTCTCATGTATCTCTCTGCTCACTGCTGCTCTAAAC
   Consensus GAAAGGAGTCGAGCTCTGTTTCCCACAACTCCTCAACAGCTCCTGCAGGAAGCCGACACTTCACTGGTCCAAAGCTGTGCTCCTGAACATTGTGCTCTCATGTATCTCTCTGCTCACTGCTGCTCTAAAC

             781    790       800       810       820       830       840       850       860       870       880       890       900       910
             |--------+---------+---------+---------+---------+---------+---------+---------+---------+---------+---------+---------+---------|
contig084887 CTTCTCGTCATCATCTCAGTCTCCCACTTCAGGCAGAGATTAACTTTTCAACTGAAATTACAGTTTAAGTTTTAGATGTGTGAAAACTTCCGTTTATATTATATGACTTAGTTATTAAAGAATGAAGTGA
BriTARs.A018 CTTCTCGTCATCATCTCAGTCTCCCACTTCAGGCAG----------------------------------------------------------------------------------------------
   Consensus CTTCTCGTCATCATCTCAGTCTCCCACTTCAGGCAG..............................................................................................

             911    920       930       940       950       960       970       980       990       1000      1010      1020      1030      1040
             |--------+---------+---------+---------+---------+---------+---------+---------+---------+---------+---------+---------+---------|
contig084887 ACTTTTCTTGGTGTATTTTAACCATGGTCATAATGTCTTATTTGCTTGAATTGAATCATGCCTTATTAGTGCAGCATTTTCACTGAAGACATGTTGACATTAACAATGTGCATTATATTCTTTTCTGTCT
BriTARs.A018 ----------------------------------------------------------------------------------------------------------------------------------
   Consensus ..................................................................................................................................

             1041  1050      1060      1070      1080      1090      1100      1110      1120      1130      1140      1150      1160      1170
             |--------+---------+---------+---------+---------+---------+---------+---------+---------+---------+---------+---------+---------|
contig084887 CCCTGCAGGCAGCTGCACACACCCAGTAATATCCTCCTCCTCTCTCTGGCCGTCTCAGACTTTTTTGTGGGTCTCCTGTTGTTGCCTTTAGAAATTTTTAGAAAGACAAGCTGCTGGGTACTTGGTGATC
BriTARs.A018 ------------CTGCACACACCCAGTAATATCCTCCTCCTCTCTCTGGCCGTCTCAGACTTTTTTGTGGGTCTCCTGTTGTTGCCTTTAGAAATTTTTAGAAAGACAAGCTGCTGGGTACTTGGTGATC
   Consensus ............CTGCACACACCCAGTAATATCCTCCTCCTCTCTCTGGCCGTCTCAGACTTTTTTGTGGGTCTCCTGTTGTTGCCTTTAGAAATTTTTAGAAAGACAAGCTGCTGGGTACTTGGTGATC

             1171  1180      1190      1200      1210      1220      1230      1240      1250      1260      1270      1280      1290      1300
             |--------+---------+---------+---------+---------+---------+---------+---------+---------+---------+---------+---------+---------|
contig084887 GTATGTGTTCTGCTTATTGGTATTTGACCAGCAACATTATCTGTGCTTCAATAGGGAACATTGTTCTAATATCAGTTGACCGCTATGTGGCTATTTGTGACCCTCTGCATTATCCCAGCAGAATTACTTT
BriTARs.A018 GTATGTGTTCTGCTTATTGGTATTTGACCAGCAACATTATCTGTGCTTCAATAGGGAACATTGTTCTAATATCAGTTGACCGCTATGTGGCTATTTGTGACCCTCTGCATTATCCCAGCAGAATTACTTT
   Consensus GTATGTGTTCTGCTTATTGGTATTTGACCAGCAACATTATCTGTGCTTCAATAGGGAACATTGTTCTAATATCAGTTGACCGCTATGTGGCTATTTGTGACCCTCTGCATTATCCCAGCAGAATTACTTT

             1301  1310      1320      1330      1340      1350      1360      1370      1380      1390      1400      1410      1420      1430
             |--------+---------+---------+---------+---------+---------+---------+---------+---------+---------+---------+---------+---------|
contig084887 GGCGAAAGTCAAACTCAGTGTTTGTCTGTGTTGGTTTTATGCTTTTTTCTACAGCAATCTTTATACAAAGGATATCATGATTGAACCAAGCAGGTATAATTCTTGCTTTGGAGAGTGTGTATTTTTTAGC
BriTARs.A018 GGCGAAAGTCAAACTCAGTGTTTGTCTGTGTTGGTTTTATGCTTTTTTCTACAGCAATCTTTATACAAAGGATATCATGATTGAACCAAGCAGGTATAATTCTTGCTTTGGAGAGTGTGTATTTTTTAGC
   Consensus GGCGAAAGTCAAACTCAGTGTTTGTCTGTGTTGGTTTTATGCTTTTTTCTACAGCAATCTTTATACAAAGGATATCATGATTGAACCAAGCAGGTATAATTCTTGCTTTGGAGAGTGTGTATTTTTTAGC

             1431  1440      1450      1460      1470      1480      1490      1500      1510      1520      1530      1540      1550      1560
             |--------+---------+---------+---------+---------+---------+---------+---------+---------+---------+---------+---------+---------|
contig084887 AGCAATATTGCTATTGTTGTTGACCTTATTTTATTCTTTTTTGTTCCAGTAACTGTTATCATAGTCTTGTATATGAGAGTATTTGTGGTGGCTGTGTCTCAAGCTCGTGCCATGCGCTCTCATGTTACAG
BriTARs.A018 AGCAATATTGCTATTGTTGTTGACCTTATTTTATTCTTTTTTGTTCCAGTAACTGTTATCATAGTCTTGTATATGAGAGTATTTGTGGTGGCTGTGTCTCAAGCTCGTGCCATGCGCTCTCATGTTACAG
   Consensus AGCAATATTGCTATTGTTGTTGACCTTATTTTATTCTTTTTTGTTCCAGTAACTGTTATCATAGTCTTGTATATGAGAGTATTTGTGGTGGCTGTGTCTCAAGCTCGTGCCATGCGCTCTCATGTTACAG

             1561  1570      1580      1590      1600      1610      1620      1630      1640      1650      1660      1670      1680      1690
             |--------+---------+---------+---------+---------+---------+---------+---------+---------+---------+---------+---------+---------|
contig084887 CTCTCACACTTCAGCGTTCACTGAATCAAACAAACAAATCTGAGCTGAAAGCAGCCAGGACTCTGGGGATTCTTGTAGTTGTGTTTCTAGCATGCTTCTCTCCACTCTACTGCTACTCTCTTGTTGATGA
BriTARs.A018 CTCTCACACTTCAGCGTTCACTGAATCAAACAAACAAATCTGAGCTGAAAGCAGCCAGGACTCTGGGGATTCTTGTAGTTGTGTTTCTAGCATGCTTCTCTCCACTCTACTGCTACTCTCTTGTTGATGA
   Consensus CTCTCACACTTCAGCGTTCACTGAATCAAACAAACAAATCTGAGCTGAAAGCAGCCAGGACTCTGGGGATTCTTGTAGTTGTGTTTCTAGCATGCTTCTCTCCACTCTACTGCTACTCTCTTGTTGATGA

             1691  1700      1710      1720      1730      1740      1750      1760      1770      1780      1790      1800      1810      1820
             |--------+---------+---------+---------+---------+---------+---------+---------+---------+---------+---------+---------+---------|
contig084887 AAATGCAATCATTGATCCAGCTGCATCTTTTGTGGTCATTATCTTTTACATTAACTCTTGTCTAAACCCTTTGATCTATGCCTTGTTTTACCCCTGGTTTAGAAACGCTGTTAAACTTATCATCACGTTG
BriTARs.A018 AAATGCAATCATTGATCCAGCTGCATCTTTTGTGGTCATTATCTTTTACATTAACTCTTGTCTAAACCCTTTGATCTATGCCTTGTTTTACCCCTGGTTTAGAAACGCTGTTAAACTTATCATCACGTTG
   Consensus AAATGCAATCATTGATCCAGCTGCATCTTTTGTGGTCATTATCTTTTACATTAACTCTTGTCTAAACCCTTTGATCTATGCCTTGTTTTACCCCTGGTTTAGAAACGCTGTTAAACTTATCATCACGTTG

             1821  1830      1840      1850      1860      1870      1880      1890      1900      1910      1920      1930      1940      1950
             |--------+---------+---------+---------+---------+---------+---------+---------+---------+---------+---------+---------+---------|
contig084887 GAGATCTTCAAGTATGACACAAGTGGTGCCAACATACTGTAAAAAGAGTATGAAACCTGCTCGTGCAATGGTTAGCACTGACACACCACAGCAAGAAAGCCCTGGATTTGAATCCAGCTGGTGCATAGTT
BriTARs.A018 GAGATCTTCAAGTATGACACAAGTGGTGCCAACATACTGTAA
   Consensus GAGATCTTCAAGTATGACACAAGTGGTGCCAACATACTGTAA........................................................................................
```

```
            1431     1440      1450      1460      1470      1480      1490      1500      1510      1520      1530      1540      1550      1560
            |--------+---------+---------+---------+---------+---------+---------+---------+---------+---------+---------+---------+---------|
contig086337 GATAAAGATATATGCTGTATGACACAGAATATGATTAGCAGCTCGCCTCACTGTAATGATGGAGGAAACTGAACTCTGCTTTCCAAAACTCCTTAATATCTCCTGCAGGAGGCCAAAGCGTCCTCACTTT
BriTARs.A019                                                           ATGGAGGAAACTGAACTCTGCTTTCCAAAACTCCTTAATATCTCCTGCAGGAGGCCAAAGCGTCCTCACTTT
   Consensus ..........................................................ATGGAGGAAACTGAACTCTGCTTTCCAAAACTCCTTAATATCTCCTGCAGGAGGCCAAAGCGTCCTCACTTT

            1561     1570      1580      1590      1600      1610      1620      1630      1640      1650      1660      1670      1680      1690
            |--------+---------+---------+---------+---------+---------+---------+---------+---------+---------+---------+---------+---------|
contig086337 GAGATCATGCTGACTTACATTCTGCTCTCCTTCATTTCTTTGCTTACTGTGATTCTTAACCTGCTGGTCATTATCTCCATCTCACACTTCAGGTATGAAAATGTTTCATTATCTCCAAATATTGAATAAT
BriTARs.A019 GAGATCATGCTGACTTACATTCTGCTCTCCTTCATTTCTTTGCTTACTGTGATTCTTAACCTGCTGGTCATTATCTCCATCTCACACTTCAGG-------------------------------------
   Consensus GAGATCATGCTGACTTACATTCTGCTCTCCTTCATTTCTTTGCTTACTGTGATTCTTAACCTGCTGGTCATTATCTCCATCTCACACTTCAGG.....................................

            1691     1700      1710      1720      1730      1740      1750      1760      1770      1780      1790      1800      1810      1820
            |--------+---------+---------+---------+---------+---------+---------+---------+---------+---------+---------+---------+---------|
contig086337 TATTTTAATAGTTTAGTTAATTGTGCTGTGTAGCAACATAGCAGTAATAGTAATTAAAACCATAATATGATCTGTACCTACAAGAAAGAGTAGACCTTTTTTTAATGAAGTGCATTTCTATTTCAGTTTT
BriTARs.A019 ----------------------------------------------------------------------------------------------------------------------------------
   Consensus ..................................................................................................................................

            1821     1830      1840      1850      1860      1870      1880      1890      1900      1910      1920      1930      1940      1950
            |--------+---------+---------+---------+---------+---------+---------+---------+---------+---------+---------+---------+---------|
contig086337 TGTTGTATGTGCATCCACATACATTTTTGATGCAGGAAACTAAATATGATGCTCCTTTTTCTCTCCAGGCAGCTCCACACCCCCACCAACTTCCTCCTTCTCTCTCTGGCTGTCGCTGATTTCTTTGTAG
BriTARs.A019 ---------------------------------------------------------------------CAGCTCCACACCCCCACCAACTTCCTCCTTCTCTCTCTGGCTGTCGCTGATTTCTTTGTAG
   Consensus .....................................................................CAGCTCCACACCCCCACCAACTTCCTCCTTCTCTCTCTGGCTGTCGCTGATTTCTTTGTAG

            1951     1960      1970      1980      1990      2000      2010      2020      2030      2040      2050      2060      2070      2080
            |--------+---------+---------+---------+---------+---------+---------+---------+---------+---------+---------+---------+---------|
contig086337 GTTTCCTTATGTTCTTTCAAATAGTGCTCATCGATGGATGCTGGTTCCTCGGTGACATCATGTGCACTCTGTATCAGTATCTAGCATTCATTATTACTTCAGCCTCAATAGGAACCATGGTGATCATATC
BriTARs.A019 GTTTCCTTATGTTCTTTCAAATAGTGCTCATCGATGGATGCTGGTTCCTCGGTGACATCATGTGCACTCTGTATCAGTATCTAGCATTCATTATTACTTCAGCCTCAATAGGAACCATGGTGATCATATC
   Consensus GTTTCCTTATGTTCTTTCAAATAGTGCTCATCGATGGATGCTGGTTCCTCGGTGACATCATGTGCACTCTGTATCAGTATCTAGCATTCATTATTACTTCAGCCTCAATAGGAACCATGGTGATCATATC

            2081     2090      2100      2110      2120      2130      2140      2150      2160      2170      2180      2190      2200      2210
            |--------+---------+---------+---------+---------+---------+---------+---------+---------+---------+---------+---------+---------|
contig086337 TGCTGATAGGTATTTGGCTATTTGTTACCCTCTGCATTACTCCACCAAAATCACACAACAAAGAATTAATATATGTATAAGTTTGTGTTGGTTTTTTTCTGTAATCTTTCAGAGTTTGATTGTGAAGGAT
BriTARs.A019 TGCTGATAGGTATTTGGCTATTTGTTACCCTCTGCATTACTCCACCAAAATCACACAACAAAGAATTAATATATGTATAAGTTTGTGTTGGTTTTTTTCTGTAATCTTTCAGAGTTTGATTGTGAAGGAT
   Consensus TGCTGATAGGTATTTGGCTATTTGTTACCCTCTGCATTACTCCACCAAAATCACACAACAAAGAATTAATATATGTATAAGTTTGTGTTGGTTTTTTTCTGTAATCTTTCAGAGTTTGATTGTGAAGGAT

            2211     2220      2230      2240      2250      2260      2270      2280      2290      2300      2310      2320      2330      2340
            |--------+---------+---------+---------+---------+---------+---------+---------+---------+---------+---------+---------+---------|
contig086337 AACTTGAAACAACCAGGAAAGTATAACTCCTGCATTGGAGAGTGTGTCTTTGTCGTTAACTACATTGCTGGGATTTTTGATCTTTTGTTTTCCTTCATTGTTCCCATTACTGTGATTGTAGTTCTGTATC
BriTARs.A019 AACTTGAAACAACCAGGAAAGTATAACTCCTGCATTGGAGAGTGTGTCTTTGTCGTTAACTACATTGCTGGGATTTTTGATCTTTTGTTTTCCTTCATTGTTCCCATTACTGTGATTGTAGTTCTGTATC
   Consensus AACTTGAAACAACCAGGAAAGTATAACTCCTGCATTGGAGAGTGTGTCTTTGTCGTTAACTACATTGCTGGGATTTTTGATCTTTTGTTTTCCTTCATTGTTCCCATTACTGTGATTGTAGTTCTGTATC

            2341     2350      2360      2370      2380      2390      2400      2410      2420      2430      2440      2450      2460      2470
            |--------+---------+---------+---------+---------+---------+---------+---------+---------+---------+---------+---------+---------|
contig086337 TGAGAGTGTTTGTGGTGGCTGTGACTCAGGCTCGTGCCATGAGGTGTCAACTTGCAGTCACTCACCAGCGATCAGTTACAGTAACTGTTATGAAATCGGAGCTGAAAGCAGCCCGTACTCTTGGTGTTGT
BriTARs.A019 TGAGAGTGTTTGTGGTGGCTGTGACTCAGGCTCGTGCCATGAGGTGTCAACTTGCAGTCACTCACCAGCGATCAGTTACAGTAACTGTTATGAAATCGGAGCTGAAAGCAGCCCGTACTCTTGGTGTTGT
   Consensus TGAGAGTGTTTGTGGTGGCTGTGACTCAGGCTCGTGCCATGAGGTGTCAACTTGCAGTCACTCACCAGCGATCAGTTACAGTAACTGTTATGAAATCGGAGCTGAAAGCAGCCCGTACTCTTGGTGTTGT

            2471     2480      2490      2500      2510      2520      2530      2540      2550      2560      2570      2580      2590      2600
            |--------+---------+---------+---------+---------+---------+---------+---------+---------+---------+---------+---------+---------|
contig086337 TGTAGTTGTGTTTCTTATATGTATGTGCCCATATTACTGCGTTGCTCTCACAGGGCAAGATAACTTCCTAAATGCTTCATCTGCTGCCTTTGTAATATGTTTGGTGTACTTTAACTCTTGCCTAAACCCT
BriTARs.A019 TGTAGTTGTGTTTCTTATATGTATGTGCCCATATTACTGCGTTGCTCTCACAGGGCAAGATAACTTCCTAAATGCTTCATCTGCTGCCTTTGTAATATGTTTGGTGTACTTTAACTCTTGCCTAAACCCT
   Consensus TGTAGTTGTGTTTCTTATATGTATGTGCCCATATTACTGCGTTGCTCTCACAGGGCAAGATAACTTCCTAAATGCTTCATCTGCTGCCTTTGTAATATGTTTGGTGTACTTTAACTCTTGCCTAAACCCT

            2601     2610      2620      2630      2640      2650      2660      2670      2680      2690      2700      2710      2720      2730
            |--------+---------+---------+---------+---------+---------+---------+---------+---------+---------+---------+---------+---------|
contig086337 ATCATTTATGTCTTTTTTTATCCCTGGTTCAGAAAGTCAATCAAACTTATTGCTACTCTTCAAATACTGCAGCCTGACTCCTGTGAGACTAACATGCATTAAGAACACTTTTTAAAACCGTACCCATAAA
BriTARs.A019 ATCATTTATGTCTTTTTTTATCCCTGGTTCAGAAAGTCAATCAAACTTATTGCTACTCTTCAAATACTGCAGCCTGACTCCTGTGAGACTAACATGCATTAA
   Consensus ATCATTTATGTCTTTTTTTATCCCTGGTTCAGAAAGTCAATCAAACTTATTGCTACTCTTCAAATACTGCAGCCTGACTCCTGTGAGACTAACATGCATTAA............................
```

```
             5331 5340      5350      5360      5370      5380      5390      5400      5410      5420      5430      5440      5450      5460
             |--------+---------+---------+---------+---------+---------+---------+---------+---------+---------+---------+---------+---------|
contig086344 GGAAAAAATATCAGAGAATGAGTTACGTCAGAAGCCAAATGAGGGAGGAAGGGTGGTGATACAAAAAAGAGGCTCAGTCACAAAGCTGTTTGTGCTGAACAGCCACTGCTTTCTTTCTCTAACAATGGAA
BriTARs.A020                                                                                                                             ATGGAA
   Consensus ............................................................................................................................ATGGAA

             5461 5470      5480      5490      5500      5510      5520      5530      5540      5550      5560      5570      5580      5590
             |--------+---------+---------+---------+---------+---------+---------+---------+---------+---------+---------+---------+---------|
contig086344 GAAACTGAACTCTGCTTTCCACAACTATTCAACTCCTCTTGTGTGCGGCAAAAGCATTCACAAATTGAGGCTGTGTTTCTTTACACTCCACTGTCTTCCATCTCTGTGCTTACTACAATTCTGAACCTGC
BriTARs.A020 GAAACTGAACTCTGCTTTCCACAACTATTCAACTCCTCTTGTGTGCGGCAAAAGCATTCACAAATTGAGGCTGTGTTTCTTTACACTCCACTGTCTTCCATCTCTGTGCTTACTACAATTCTGAACCTGC
   Consensus GAAACTGAACTCTGCTTTCCACAACTATTCAACTCCTCTTGTGTGCGGCAAAAGCATTCACAAATTGAGGCTGTGTTTCTTTACACTCCACTGTCTTCCATCTCTGTGCTTACTACAATTCTGAACCTGC

             5591 5600      5610      5620      5630      5640      5650      5660      5670      5680      5690      5700      5710      5720
             |--------+---------+---------+---------+---------+---------+---------+---------+---------+---------+---------+---------+---------|
contig086344 TTGTGATCATCTCCATCGCTCACTTCAAGTACTGACATATTTGAATACTTAACAAGTTATAGAAAAATAATGTTTTTTGTGAATTTAATTAGAAAATGTGTTGCTTTGTTCTATATGTTACAGTGGAGTT
BriTARs.A020 TTGTGATCATCTCCATCGCTCACTTCAAG-----------------------------------------------------------------------------------------------------
   Consensus TTGTGATCATCTCCATCGCTCACTTCAAG.....................................................................................................

             5721 5730      5740      5750      5760      5770      5780      5790      5800      5810      5820      5830      5840      5850
             |--------+---------+---------+---------+---------+---------+---------+---------+---------+---------+---------+---------+---------|
contig086344 GCAATTACTGCTGTAGAACAACCTATGATGCTCCTATTTTAATCCAGGCAGCTGCACACCCCAACCAACCTCCTCCTCCTCTCTCTGGCCGTCTCAGATTTCTTCGTGGGCCTCATCATGGCTTGTCAGA
BriTARs.A020 ------------------------------------------------CAGCTGCACACCCCAACCAACCTCCTCCTCCTCTCTCTGGCCGTCTCAGATTTCTTCGTGGGCCTCATCATGGCTTGTCAGA
   Consensus ................................................CAGCTGCACACCCCAACCAACCTCCTCCTCCTCTCTCTGGCCGTCTCAGATTTCTTCGTGGGCCTCATCATGGCTTGTCAGA

             5851 5860      5870      5880      5890      5900      5910      5920      5930      5940      5950      5960      5970      5980
             |--------+---------+---------+---------+---------+---------+---------+---------+---------+---------+---------+---------+---------|
contig086344 TTAGTCTCCTAGATGGCTGCTGGTTTCTTGGTGACCACATGTGTGCTCTGTATACCACTTTAGATTACATTGTTACTTCTGCTTCAGTAGGAACTATGGTATTCATATCAGCTGACCGCTATGTAGCCAT
BriTARs.A020 TTAGTCTCCTAGATGGCTGCTGGTTTCTTGGTGACCACATGTGTGCTCTGTATACCACTTTAGATTACATTGTTACTTCTGCTTCAGTAGGAACTATGGTATTCATATCAGCTGACCGCTATGTAGCCAT
   Consensus TTAGTCTCCTAGATGGCTGCTGGTTTCTTGGTGACCACATGTGTGCTCTGTATACCACTTTAGATTACATTGTTACTTCTGCTTCAGTAGGAACTATGGTATTCATATCAGCTGACCGCTATGTAGCCAT

             5981 5990      6000      6010      6020      6030      6040      6050      6060      6070      6080      6090      6100      6110
             |--------+---------+---------+---------+---------+---------+---------+---------+---------+---------+---------+---------+---------|
contig086344 TTGTGACCCTCTGCATTATCCCACCGAAATTACTATAAAAAGAATCTCAGTCTGTATTTGTACTTGCTGGGCTTGTTCAATTCTGTATAACAGTCTTATCATGAAGGATAATTTCAAGCAGCCAGGGAGG
BriTARs.A020 TTGTGACCCTCTGCATTATCCCACCGAAATTACTATAAAAAGAATCTCAGTCTGTATTTGTACTTGCTGGGCTTGTTCAATTCTGTATAACAGTCTTATCATGAAGGATAATTTCAAGCAGCCAGGGAGG
   Consensus TTGTGACCCTCTGCATTATCCCACCGAAATTACTATAAAAAGAATCTCAGTCTGTATTTGTACTTGCTGGGCTTGTTCAATTCTGTATAACAGTCTTATCATGAAGGATAATTTCAAGCAGCCAGGGAGG

             6111 6120      6130      6140      6150      6160      6170      6180      6190      6200      6210      6220      6230      6240
             |--------+---------+---------+---------+---------+---------+---------+---------+---------+---------+---------+---------+---------|
contig086344 TATAATTCTTGTTCTGGTGATTGTGCAGTTGTCATTGATTACTTTGTGGGAATTTTTGACTTTGTTTTGACCTTTGTCGGCCCTGTGATTGTCATCATAGTTCTGTATCTAAGAGTATTTGTGGTGGCTG
BriTARs.A020 TATAATTCTTGTTCTGGTGATTGTGCAGTTGTCATTGATTACTTTGTGGGAATTTTTGACTTTGTTTTGACCTTTGTCGGCCCTGTGATTGTCATCATAGTTCTGTATCTAAGAGTATTTGTGGTGGCTG
   Consensus TATAATTCTTGTTCTGGTGATTGTGCAGTTGTCATTGATTACTTTGTGGGAATTTTTGACTTTGTTTTGACCTTTGTCGGCCCTGTGATTGTCATCATAGTTCTGTATCTAAGAGTATTTGTGGTGGCTG

             6241 6250      6260      6270      6280      6290      6300      6310      6320      6330      6340      6350      6360      6370
             |--------+---------+---------+---------+---------+---------+---------+---------+---------+---------+---------+---------+---------|
contig086344 TGTCTCAGGCTCGGGCAATGCGCTCTCATATCACTGCTCTCAGACTCCAGGGGTCAGAGACAGTGCATGCTAAGAAATCTGAGCTGAAAGCTGCCAGGACTCTCGGTGTACTTGTTATTGCCTTTCTGAT
BriTARs.A020 TGTCTCAGGCTCGGGCAATGCGCTCTCATATCACTGCTCTCAGACTCCAGGGGTCAGAGACAGTGCATGCTAAGAAATCTGAGCTGAAAGCTGCCAGGACTCTCGGTGTACTTGTTATTGCCTTTCTGAT
   Consensus TGTCTCAGGCTCGGGCAATGCGCTCTCATATCACTGCTCTCAGACTCCAGGGGTCAGAGACAGTGCATGCTAAGAAATCTGAGCTGAAAGCTGCCAGGACTCTCGGTGTACTTGTTATTGCCTTTCTGAT

             6371 6380      6390      6400      6410      6420      6430      6440      6450      6460      6470      6480      6490      6500
             |--------+---------+---------+---------+---------+---------+---------+---------+---------+---------+---------+---------+---------|
contig086344 ATGTCTTTTCCCATTTTTCTGTTCCTCAATGGTAGGCCAGAATAGTTTCTTTGATATTAGATCTGTACCTTTTGAGCGCTTGCTGTTCTACTTTAACTCTTGTCTGAACCCATTAATCTACACTTTTTGC
BriTARs.A020 ATGTCTTTTCCCATTTTTCTGTTCCTCAATGGTAGGCCAGAATAGTTTCTTTGATATTAGATCTGTACCTTTTGAGCGCTTGCTGTTCTACTTTAACTCTTGTCTGAACCCATTAATCTACACTTTTTGC
   Consensus ATGTCTTTTCCCATTTTTCTGTTCCTCAATGGTAGGCCAGAATAGTTTCTTTGATATTAGATCTGTACCTTTTGAGCGCTTGCTGTTCTACTTTAACTCTTGTCTGAACCCATTAATCTACACTTTTTGC

             6501 6510      6520      6530      6540      6550      6560      6570      6580      6590      6600      6610      6620      6630
             |--------+---------+---------+---------+---------+---------+---------+---------+---------+---------+---------+---------+---------|
contig086344 TACCCCTGGTTTCTGAAATCTATCAAGCTCATTGTGACATTTAAGATATTTAGGCATGGTTCCAGTGAGGCCAGTATACTATAGGGAAAAGTGATTTTAGTAATGAGTAAGGCATACAGTCTTTGACACT
BriTARs.A020 TACCCCTGGTTTCTGAAATCTATCAAGCTCATTGTGACATTTAAGATATTTAGGCATGGTTCCAGTGAGGCCAGTATACTATAG
   Consensus TACCCCTGGTTTCTGAAATCTATCAAGCTCATTGTGACATTTAAGATATTTAGGCATGGTTCCAGTGAGGCCAGTATACTATAG..............................................
```

```
             2081 2090      2100      2110      2120      2130      2140      2150      2160      2170      2180      2190      2200      2210
             |--------+---------+---------+---------+---------+---------+---------+---------+---------+---------+---------+---------+---------|
contig086351 ACGTTATAACATTTGAGTTTGTTAGAAAGGTGTCAGGGGCTCATTAGCACTTCTAACTGATGATGGCAGAAACTGAACTCTGCTTTCCAAATCTCAACTCCTCCTGCAGTAGAGTAAAGCGTTCTCCCTC
BriTARs.A021                                                               ATGGCAGAAACTGAACTCTGCTTTCCAAATCTCAACTCCTCCTGCAGTAGAGTAAAGCGTTCTCCCTC
   Consensus ..............................................................ATGGCAGAAACTGAACTCTGCTTTCCAAATCTCAACTCCTCCTGCAGTAGAGTAAAGCGTTCTCCCTC

             2211 2220      2230      2240      2250      2260      2270      2280      2290      2300      2310      2320      2330      2340
             |--------+---------+---------+---------+---------+---------+---------+---------+---------+---------+---------+---------+---------|
contig086351 GGAGTCAGTGCTTATTTATGTGATTCTAACCATCATTTCTCTTCTTACTGTGGCTCTAAACCTGCTGGTCATCATCTCCATCTCCCACTTCAAGTAGATACTTAATACATTTATAAGTCTAGCAGTTGTA
BriTARs.A021 GGAGTCAGTGCTTATTTATGTGATTCTAACCATCATTTCTCTTCTTACTGTGGCTCTAAACCTGCTGGTCATCATCTCCATCTCCCACTTCAAG------------------------------------
   Consensus GGAGTCAGTGCTTATTTATGTGATTCTAACCATCATTTCTCTTCTTACTGTGGCTCTAAACCTGCTGGTCATCATCTCCATCTCCCACTTCAAG....................................

             2341 2350      2360      2370      2380      2390      2400      2410      2420      2430      2440      2450      2460      2470
             |--------+---------+---------+---------+---------+---------+---------+---------+---------+---------+---------+---------+---------|
contig086351 CATGAGAAAATGACAGACATAAAATAAGTTAAACAGAACAACTTTTTCACAATGTTTTGATGTGGATGATCGGCTGGAATAATTGATGCTACAAATAATATAGATTTTTCTATCTCTTATCTCTCCAGGC
BriTARs.A021 ---------------------------------------------------------------------------------------------------------------------------------C
   Consensus .................................................................................................................................C

             2471 2480      2490      2500      2510      2520      2530      2540      2550      2560      2570      2580      2590      2600
             |--------+---------+---------+---------+---------+---------+---------+---------+---------+---------+---------+---------+---------|
contig086351 AGCTCCACACCCCTACAAACTACCTCCTCCTCTCCCTGGCAGTCTCTGATTTCTTTGTGGGTTTCAATATGTGCTTTCAAAGTATGCTCATAGATGGTTGTTGGTATCTTGGTGACCTCATGTGTGTCTT
BriTARs.A021 AGCTCCACACCCCTACAAACTACCTCCTCCTCTCCCTGGCAGTCTCTGATTTCTTTGTGGGTTTCAATATGTGCTTTCAAAGTATGCTCATAGATGGTTGTTGGTATCTTGGTGACCTCATGTGTGTCTT
   Consensus AGCTCCACACCCCTACAAACTACCTCCTCCTCTCCCTGGCAGTCTCTGATTTCTTTGTGGGTTTCAATATGTGCTTTCAAAGTATGCTCATAGATGGTTGTTGGTATCTTGGTGACCTCATGTGTGTCTT

             2601 2610      2620      2630      2640      2650      2660      2670      2680      2690      2700      2710      2720      2730
             |--------+---------+---------+---------+---------+---------+---------+---------+---------+---------+---------+---------+---------|
contig086351 GTATTATGTTTTCGAGATAGTTGTTACTTCTGCCTCAGTAGGAACCATGGTGCTCATTTCAGTTGACCGTTATGTGGCCATTTGTGATCCTCTTCATTATCCCACCAAAGTCACTCCAAAAAGAGTTCAG
BriTARs.A021 GTATTATGTTTTCGAGATAGTTGTTACTTCTGCCTCAGTAGGAACCATGGTGCTCATTTCAGTTGACCGTTATGTGGCCATTTGTGATCCTCTTCATTATCCCACCAAAGTCACTCCAAAAAGAGTTCAG
   Consensus GTATTATGTTTTCGAGATAGTTGTTACTTCTGCCTCAGTAGGAACCATGGTGCTCATTTCAGTTGACCGTTATGTGGCCATTTGTGATCCTCTTCATTATCCCACCAAAGTCACTCCAAAAAGAGTTCAG

             2731 2740      2750      2760      2770      2780      2790      2800      2810      2820      2830      2840      2850      2860
             |--------+---------+---------+---------+---------+---------+---------+---------+---------+---------+---------+---------+---------|
contig086351 ACCTGTGTTTTAATATGTTGGATTTGCTCGCTCCTAGTAGTCGGTGTGCTATTGAAGGATAACCTGGATAAACCAGGTAGGTTTAATTCCTGCTTTGGAGAGTGTGTGATTTATATTGACTTTGCAATAC
BriTARs.A021 ACCTGTGTTTTAATATGTTGGATTTGCTCGCTCCTAGTAGTCGGTGTGCTATTGAAGGATAACCTGGATAAACCAGGTAGGTTTAATTCCTGCTTTGGAGAGTGTGTGATTTATATTGACTTTGCAATAC
   Consensus ACCTGTGTTTTAATATGTTGGATTTGCTCGCTCCTAGTAGTCGGTGTGCTATTGAAGGATAACCTGGATAAACCAGGTAGGTTTAATTCCTGCTTTGGAGAGTGTGTGATTTATATTGACTTTGCAATAC

             2861 2870      2880      2890      2900      2910      2920      2930      2940      2950      2960      2970      2980      2990
             |--------+---------+---------+---------+---------+---------+---------+---------+---------+---------+---------+---------+---------|
contig086351 AAGTTACCGATCTTATTTTTACAATCCTCCTTCCCATTACTGTCATCGTAATTTTGTATGTAAGAGTATTTGCTGTGGCCGTGTTTCAGATTCGGGCCATGCAACATCATGTTGCAGCTGGCACACAGAG
BriTARs.A021 AAGTTACCGATCTTATTTTTACAATCCTCCTTCCCATTACTGTCATCGTAATTTTGTATGTAAGAGTATTTGCTGTGGCCGTGTTTCAGATTCGGGCCATGCAACATCATGTTGCAGCTGGCACACAGAG
   Consensus AAGTTACCGATCTTATTTTTACAATCCTCCTTCCCATTACTGTCATCGTAATTTTGTATGTAAGAGTATTTGCTGTGGCCGTGTTTCAGATTCGGGCCATGCAACATCATGTTGCAGCTGGCACACAGAG

             2991 3000      3010      3020      3030      3040      3050      3060      3070      3080      3090      3100      3110      3120
             |--------+---------+---------+---------+---------+---------+---------+---------+---------+---------+---------+---------+---------|
contig086351 GGGGAAAGTAAGTCCAAAAAAATCTGAACTGAAAGCAGCTAGGACTCTTGGCATTGTTATTGTTGCATTTCTGATATGCCTATTCCCATATTACTGTGTAATACTCTCAGGCCAGGACACACTGCTTGAT
BriTARs.A021 GGGGAAAGTAAGTCCAAAAAAATCTGAACTGAAAGCAGCTAGGACTCTTGGCATTGTTATTGTTGCATTTCTGATATGCCTATTCCCATATTACTGTGTAATACTCTCAGGCCAGGACACACTGCTTGAT
   Consensus GGGGAAAGTAAGTCCAAAAAAATCTGAACTGAAAGCAGCTAGGACTCTTGGCATTGTTATTGTTGCATTTCTGATATGCCTATTCCCATATTACTGTGTAATACTCTCAGGCCAGGACACACTGCTTGAT

             3121 3130      3140      3150      3160      3170      3180      3190      3200      3210      3220      3230      3240      3250
             |--------+---------+---------+---------+---------+---------+---------+---------+---------+---------+---------+---------+---------|
contig086351 ATTTTATCCCTTGCCTTTTTTTTATGTTTGTTTTATTTTAATTCCTTTCTCAACCCTATCATCTATGCCCTTTTCTACCCATGGTTTAGAAAATCTGTTAAACTTATTGTTACATTTCAAATAGTCAAAT
BriTARs.A021 ATTTTATCCCTTGCCTTTTTTTTATGTTTGTTTTATTTTAATTCCTTTCTCAACCCTATCATCTATGCCCTTTTCTACCCATGGTTTAGAAAATCTGTTAAACTTATTGTTACATTTCAAATAGTCAAAT
   Consensus ATTTTATCCCTTGCCTTTTTTTTATGTTTGTTTTATTTTAATTCCTTTCTCAACCCTATCATCTATGCCCTTTTCTACCCATGGTTTAGAAAATCTGTTAAACTTATTGTTACATTTCAAATAGTCAAAT

             3251 3260      3270      3280      3290      3300      3310      3320      3330      3340      3350      3360      3370      3380
             |--------+---------+---------+---------+---------+---------+---------+---------+---------+---------+---------+---------+---------|
contig086351 CTGGCTCCAGTGATGCCAGCATGCTGTGACAAAATAAGCCCTTCAAGTCAAGCCAAATAAATAAAATAATATTTCCATATTTCAAATTGCAAATATATATTATATTATATATAAACTGCAATAGAAAAGA
BriTARs.A021 CTGGCTCCAGTGATGCCAGCATGCTGTGA
   Consensus CTGGCTCCAGTGATGCCAGCATGCTGTGA.....................................................................................................
```

```
            4551 4560      4570      4580      4590      4600      4610      4620      4630      4640      4650      4660      4670      4680
            |--------+---------+---------+---------+---------+---------+---------+---------+---------+---------+---------+---------+---------|
contig082565 CTGAAAGAAAGAAGGGGGAGGAGTTGAGAGGATGTGGTGTGGGGATCAGTTATATTACTAATTCTAGTCATAACTCTGCCAGAGCTGGAGCTCTGTGCAGGTCTGTGGAGATGGATACTCAGGATGGAGC
BriTARs.A022                                                                                                               ATGGATACTCAGGATGGAGC
   Consensus ..............................................................................................................ATGGATACTCAGGATGGAGC

            4681 4690      4700      4710      4720      4730      4740      4750      4760      4770      4780      4790      4800      4810
            |--------+---------+---------+---------+---------+---------+---------+---------+---------+---------+---------+---------+---------|
contig082565 AGAGCTCTGCTTTCCACAACTCTTCAACATCTCCTGCAAGAAACCTACAACTCCTCTGTCTCAAGTTTTGCTCATTTATACTGTTGTGTCCTCAATGTCTCTGCTGACTGTGACTCTCAACCTGCTCGTC
BriTARs.A022 AGAGCTCTGCTTTCCACAACTCTTCAACATCTCCTGCAAGAAACCTACAACTCCTCTGTCTCAAGTTTTGCTCATTTATACTGTTGTGTCCTCAATGTCTCTGCTGACTGTGACTCTCAACCTGCTCGTC
   Consensus AGAGCTCTGCTTTCCACAACTCTTCAACATCTCCTGCAAGAAACCTACAACTCCTCTGTCTCAAGTTTTGCTCATTTATACTGTTGTGTCCTCAATGTCTCTGCTGACTGTGACTCTCAACCTGCTCGTC

            4811 4820      4830      4840      4850      4860      4870      4880      4890      4900      4910      4920      4930      4940
            |--------+---------+---------+---------+---------+---------+---------+---------+---------+---------+---------+---------+---------|
contig082565 ATCATTGCAGTCTCCCACTTCAGGCAGAGTTTCATATTTTTTACCTGCTGCTGTGGCCTTACAAGTCTATACCTCATTTGTTATACCTGTTTGAATAGTTTCTTTCTGTTGACTTTTTAAAAAATTATAT
BriTARs.A022 ATCATTGCAGTCTCCCACTTCAGGCAG-------------------------------------------------------------------------------------------------------
   Consensus ATCATTGCAGTCTCCCACTTCAGGCAG.......................................................................................................

            4941 4950      4960      4970      4980      4990      5000      5010      5020      5030      5040      5050      5060      5070
            |--------+---------+---------+---------+---------+---------+---------+---------+---------+---------+---------+---------+---------|
contig082565 CTTCCTGTCTTTCTAGGCAGCTCCACACACCCACTAACATCCTGCTCCTTTCTCTGGCTGTCACTGACTTTCTCGTTGGTCTTCTGTTTATGCCTGGAGAAATCCTGCGAAATACAGCGTGTTGGTTTCT
BriTARs.A022 --------------------CTCCACACACCCACTAACATCCTGCTCCTTTCTCTGGCTGTCACTGACTTTCTCGTTGGTCTTCTGTTTATGCCTGGAGAAATCCTGCGAAATACAGCGTGTTGGTTTCT
   Consensus ....................CTCCACACACCCACTAACATCCTGCTCCTTTCTCTGGCTGTCACTGACTTTCTCGTTGGTCTTCTGTTTATGCCTGGAGAAATCCTGCGAAATACAGCGTGTTGGTTTCT

            5071 5080      5090      5100      5110      5120      5130      5140      5150      5160      5170      5180      5190      5200
            |--------+---------+---------+---------+---------+---------+---------+---------+---------+---------+---------+---------+---------|
contig082565 TGGTCAGCTCACATGTTCACTGTATAATTATGCATCCTACATCATTGCCTCTGCCTCAGTGGGCAACATGGTGCTGATATCAGTCGACCGCTATGTGGCTATTTGTGACCCTCTGCATTACCCCACCAGA
BriTARs.A022 TGGTCAGCTCACATGTTCACTGTATAATTATGCATCCTACATCATTGCCTCTGCCTCAGTGGGCAACATGGTGCTGATATCAGTCGACCGCTATGTGGCTATTTGTGACCCTCTGCATTACCCCACCAGA
   Consensus TGGTCAGCTCACATGTTCACTGTATAATTATGCATCCTACATCATTGCCTCTGCCTCAGTGGGCAACATGGTGCTGATATCAGTCGACCGCTATGTGGCTATTTGTGACCCTCTGCATTACCCCACCAGA

            5201 5210      5220      5230      5240      5250      5260      5270      5280      5290      5300      5310      5320      5330
            |--------+---------+---------+---------+---------+---------+---------+---------+---------+---------+---------+---------+---------|
contig082565 ATCACAGAGAGAAGAGTGAAACTCTGCGTCTGTCTGTGTTGGCTCTGCTCTGTTTTCTACAGCTATGTGATTTTAATAGATGATCTAAGTCAACCAGGCAAGCATAAATCTTGCTATGGAAAATGTATAA
BriTARs.A022 ATCACAGAGAGAAGAGTGAAACTCTGCGTCTGTCTGTGTTGGCTCTGCTCTGTTTTCTACAGCTATGTGATTTTAATAGATGATCTAAGTCAACCAGGCAAGCATAAATCTTGCTATGGAAAATGTATAA
   Consensus ATCACAGAGAGAAGAGTGAAACTCTGCGTCTGTCTGTGTTGGCTCTGCTCTGTTTTCTACAGCTATGTGATTTTAATAGATGATCTAAGTCAACCAGGCAAGCATAAATCTTGCTATGGAAAATGTATAA

            5331 5340      5350      5360      5370      5380      5390      5400      5410      5420      5430      5440      5450      5460
            |--------+---------+---------+---------+---------+---------+---------+---------+---------+---------+---------+---------+---------|
contig082565 TTTTCATTGAATTCATTGCAGGATTTGTTGACCTTGTTTTAGCTTTTATTATTCCACTTACTGTCATCATTGTTCTGTATATGAGAGTGTTTGTGGTGGCTGTGTCTCAGGCCCGTGCCATGCGCTCTCA
BriTARs.A022 TTTTCATTGAATTCATTGCAGGATTTGTTGACCTTGTTTTAGCTTTTATTATTCCACTTACTGTCATCATTGTTCTGTATATGAGAGTGTTTGTGGTGGCTGTGTCTCAGGCCCGTGCCATGCGCTCTCA
   Consensus TTTTCATTGAATTCATTGCAGGATTTGTTGACCTTGTTTTAGCTTTTATTATTCCACTTACTGTCATCATTGTTCTGTATATGAGAGTGTTTGTGGTGGCTGTGTCTCAGGCCCGTGCCATGCGCTCTCA

            5461 5470      5480      5490      5500      5510      5520      5530      5540      5550      5560      5570      5580      5590
            |--------+---------+---------+---------+---------+---------+---------+---------+---------+---------+---------+---------+---------|
contig082565 GGTTACAGCTGTCACACTGCAGCTCTCAGTGACTCTAACAGCAAAGAAATCAGAGTTAAAAGCAGCCAGGACTCTGGGTGTTCTTGTTCTTGTGTTTCTATTATGTTTCTGCTCATATTACATTATTTCA
BriTARs.A022 GGTTACAGCTGTCACACTGCAGCTCTCAGTGACTCTAACAGCAAAGAAATCAGAGTTAAAAGCAGCCAGGACTCTGGGTGTTCTTGTTCTTGTGTTTCTATTATGTTTCTGCTCATATTACATTATTTCA
   Consensus GGTTACAGCTGTCACACTGCAGCTCTCAGTGACTCTAACAGCAAAGAAATCAGAGTTAAAAGCAGCCAGGACTCTGGGTGTTCTTGTTCTTGTGTTTCTATTATGTTTCTGCTCATATTACATTATTTCA

            5591 5600      5610      5620      5630      5640      5650      5660      5670      5680      5690      5700      5710      5720
            |--------+---------+---------+---------+---------+---------+---------+---------+---------+---------+---------+---------+---------|
contig082565 CTTTTTGGAAACGAGTTGCTCAATAGCTCATCTGCATCGATTGTGATCTATCTGTACTATTTTAACTCCTGTCTAAACCCTTTGATTTATGCTATGTTCTACCCCTGGTTTAGAAAAGCTGTGAAACTAA
BriTARs.A022 CTTTTTGGAAACGAGTTGCTCAATAGCTCATCTGCATCGATTGTGATCTATCTGTACTATTTTAACTCCTGTCTAAACCCTTTGATTTATGCTATGTTCTACCCCTGGTTTAGAAAAGCTGTGAAACTAA
   Consensus CTTTTTGGAAACGAGTTGCTCAATAGCTCATCTGCATCGATTGTGATCTATCTGTACTATTTTAACTCCTGTCTAAACCCTTTGATTTATGCTATGTTCTACCCCTGGTTTAGAAAAGCTGTGAAACTAA

            5721 5730      5740      5750      5760      5770      5780      5790      5800      5810      5820      5830      5840      5850
            |--------+---------+---------+---------+---------+---------+---------+---------+---------+---------+---------+---------+---------|
contig082565 TTGTGACTCTACAGATACTGCAGCCTGGCTCCTGTGAGGTCAGCATACTGTAGAAAAAAGAAATCTTTCCTTAATGTACCAAATGGTATACCAAATTACTTTCTCTATCAATAATGTACTACATTAATTT
BriTARs.A022 TTGTGACTCTACAGATACTGCAGCCTGGCTCCTGTGAGGTCAGCATACTGTAG
   Consensus TTGTGACTCTACAGATACTGCAGCCTGGCTCCTGTGAGGTCAGCATACTGTAG.............................................................................
```

```
             5071 5080      5090      5100      5110      5120      5130      5140      5150      5160      5170      5180      5190      5200
contig035253 TTAACTCTGTTTGGACTCTCACTCTTCTTAGCAATCAGTAGTTGACCTGCACTGAAGGACAATCTACCTCAAGTCAAGTCTCAGGTTAGTGCAGCAGAAAAAATCATATTGTTGGTTTAAGCTGCTTAAT
BriTAR.B025
Consensus    ..................................................................................................................................

             5201 5210      5220      5230      5240      5250      5260      5270      5280      5290      5300      5310      5320      5330
contig035253 TATGGAACAAAAGGTTAGTATGAACATGACCAACATTGTAAATGATCTACATCCCTGTTATGTAATACAAGACTCCAAATACATGCTGACAAACAGCCCTTCCATTATATGTGTATTCTTGTATGCATTC
BriTAR.B025                           ATGACCAACATTGTAAATGATCTACATCCCTGTTATGTAATACAAGACTCCAAATACATGCTGACAAACAGCCCTTCCATTATATGTGTATTCTTGTATGCATTC
Consensus    .........................ATGACCAACATTGTAAATGATCTACATCCCTGTTATGTAATACAAGACTCCAAATACATGCTGACAAACAGCCCTTCCATTATATGTGTATTCTTGTATGCATTC

             5331 5340      5350      5360      5370      5380      5390      5400      5410      5420      5430      5440      5450      5460
contig035253 CTTGCATTATTGTCTGTTATAACAATATGTGGAAATCTCCTTGTAATAATTTCTGTGATTTACTTCAAACAGCTCCATACTCCTACTAATTATCTCATCCTCTCTCTTGCTGTGGCTGACCTGCTCGTTG
BriTAR.B025  CTTGCATTATTGTCTGTTATAACAATATGTGGAAATCTCCTTGTAATAATTTCTGTGATTTACTTCAAACAGCTCCATACTCCTACTAATTATCTCATCCTCTCTCTTGCTGTGGCTGACCTGCTCGTTG
Consensus    CTTGCATTATTGTCTGTTATAACAATATGTGGAAATCTCCTTGTAATAATTTCTGTGATTTACTTCAAACAGCTCCATACTCCTACTAATTATCTCATCCTCTCTCTTGCTGTGGCTGACCTGCTCGTTG

             5461 5470      5480      5490      5500      5510      5520      5530      5540      5550      5560      5570      5580      5590
contig035253 GGATTATAGCATTTCCTCTCAGCATGGCATTCTCTCTCAGCTCATGTCTTTATCATGAGGGTTTGTTTTGCAAAGTGCGAGGGACCTTTGATATTTCACTGAGCACATGTTCCATCCTGAACCTCTGCTG
BriTAR.B025  GGATTATAGCATTTCCTCTCAGCATGGCATTCTCTCTCAGCTCATGTCTTTATCATGAGGGTTTGTTTTGCAAAGTGCGAGGGACCTTTGATATTTCACTGAGCACATGTTCCATCCTGAACCTCTGCTG
Consensus    GGATTATAGCATTTCCTCTCAGCATGGCATTCTCTCTCAGCTCATGTCTTTATCATGAGGGTTTGTTTTGCAAAGTGCGAGGGACCTTTGATATTTCACTGAGCACATGTTCCATCCTGAACCTCTGCTG

             5591 5600      5610      5620      5630      5640      5650      5660      5670      5680      5690      5700      5710      5720
contig035253 TATTTCTATTGACAGATACCATGCTGTTTGCCAGCCTCTGACATATCAAACTAAAATCAGCCCTCGTGTTGTTGTGTTCATGATCCTGATGAGCTGGGGGGTTTCTGGGATAATTGGAATTAGTGTCACC
BriTAR.B025  TATTTCTATTGACAGATACCATGCTGTTTGCCAGCCTCTGACATATCAAACTAAAATCAGCCCTCGTGTTGTTGTGTTCATGATCCTGATGAGCTGGGGGGTTTCTGGGATAATTGGAATTAGTGTCACC
Consensus    TATTTCTATTGACAGATACCATGCTGTTTGCCAGCCTCTGACATATCAAACTAAAATCAGCCCTCGTGTTGTTGTGTTCATGATCCTGATGAGCTGGGGGGTTTCTGGGATAATTGGAATTAGTGTCACC

             5721 5730      5740      5750      5760      5770      5780      5790      5800      5810      5820      5830      5840      5850
contig035253 ATTGCTGGATTTAATAATGAAAAATGTGAGGAATCATGTTTGATTGATGTTCTAATAGAAAGCACTGTTGGACCCATGTTATCATTTTACCTGCCAGTGACCATGATGCTTTTTATCTACCTAAAGATTT
BriTAR.B025  ATTGCTGGATTTAATAATGAAAAATGTGAGGAATCATGTTTGATTGATGTTCTAATAGAAAGCACTGTTGGACCCATGTTATCATTTTACCTGCCAGTGACCATGATGCTTTTTATCTACCTAAAGATTT
Consensus    ATTGCTGGATTTAATAATGAAAAATGTGAGGAATCATGTTTGATTGATGTTCTAATAGAAAGCACTGTTGGACCCATGTTATCATTTTACCTGCCAGTGACCATGATGCTTTTTATCTACCTAAAGATTT

             5851 5860      5870      5880      5890      5900      5910      5920      5930      5940      5950      5960      5970      5980
contig035253 TCTTTGTTGCTTTGAGGCAAGCAAGACGCATCCAGAACACAACAAAGTGTGGACAAACTGCCAGTATCATGGAGAGAAAGGCGACCAAAACTCTGGCTATTGTTCTGGGAGTTTTTATATTTTGCTGGAG
BriTAR.B025  TCTTTGTTGCTTTGAGGCAAGCAAGACGCATCCAGAACACAACAAAGTGTGGACAAACTGCCAGTATCATGGAGAGAAAGGCGACCAAAACTCTGGCTATTGTTCTGGGAGTTTTTATATTTTGCTGGAG
Consensus    TCTTTGTTGCTTTGAGGCAAGCAAGACGCATCCAGAACACAACAAAGTGTGGACAAACTGCCAGTATCATGGAGAGAAAGGCGACCAAAACTCTGGCTATTGTTCTGGGAGTTTTTATATTTTGCTGGAG

             5981 5990      6000      6010      6020      6030      6040      6050      6060      6070      6080      6090      6100      6110
contig035253 TCCTTTTTTTCTTTCCATCACATTCCCACCATTTACCAGTGATTCTGTACCAGTTCCTGTAATTGAAACTCTTAACTGGCTTACATTGGCAAATTCAATGCTAAATCCATTTATTTATGCTTTCTTTTAT
BriTAR.B025  TCCTTTTTTTCTTTCCATCACATTCCCACCATTTACCAGTGATTCTGTACCAGTTCCTGTAATTGAAACTCTTAACTGGCTTACATTGGCAAATTCAATGCTAAATCCATTTATTTATGCTTTCTTTTAT
Consensus    TCCTTTTTTTCTTTCCATCACATTCCCACCATTTACCAGTGATTCTGTACCAGTTCCTGTAATTGAAACTCTTAACTGGCTTACATTGGCAAATTCAATGCTAAATCCATTTATTTATGCTTTCTTTTAT

             6111 6120      6130      6140      6150      6160      6170      6180      6190      6200      6210      6220      6230      6240
contig035253 AGCTGGTTCAGGTCAGCCATCAGAATGATTATTTGTGGGAAAATATTCCAAGGGGATTTTGCCAACACAAATATGACTTGATAAAATATACACATATTAATTAAAATTTCACAAATCTTACAGTAAAAAC
BriTAR.B025  AGCTGGTTCAGGTCAGCCATCAGAATGATTATTTGTGGGAAAATATTCCAAGGGGATTTTGCCAACACAAATATGACTTGA
Consensus    AGCTGGTTCAGGTCAGCCATCAGAATGATTATTTGTGGGAAAATATTCCAAGGGGATTTTGCCAACACAAATATGACTTGA.................................................

             6241 6250      6260      6270      6280      6290      6300      6310      6320      6330      6340      6350      6360      6370
contig035253 AAACATAAAACAGCCACTGAACCCTACCTGAAGCATCACACACATAATGTCTCAATAGCGACTGAAAGAAATAATCTGAGTAGCAAATACGTTTTGTACACATGTACAAACACATTGTTTTATTGTTCAA
BriTAR.B025
Consensus    ..................................................................................................................................
```

```
    Consensus  ......................................................................................................................................

               2861 2870      2880      2890      2900      2910      2920      2930      2940      2950      2960      2970      2980      2990
               |--------+---------+---------+---------+---------+---------+---------+---------+---------+---------+---------+---------+---------|
contig040586   TGGTAAATAGTTCAACCTTTTCCTTTTGACTGCATGCACATGCATGCACCGCTCCTCTCCGATGGATAGCTCTGGAGGTCCTCCCCTCTGCTTCCCCAACCTCAACTCCTCCTGCAGGCGGCTGCTGCGA
 ZebTAR.A001                                                                ATGGATAGCTCTGGAGGTCCTCCCCTCTGCTTCCCCAACCTCAACTCCTCCTGCAGGCGGCTGCTGCGA
    Consensus  .............................................................ATGGATAGCTCTGGAGGTCCTCCCCTCTGCTTCCCCAACCTCAACTCCTCCTGCAGGCGGCTGCTGCGA

               2991 3000      3010      3020      3030      3040      3050      3060      3070      3080      3090      3100      3110      3120
               |--------+---------+---------+---------+---------+---------+---------+---------+---------+---------+---------+---------+---------|
contig040586   CCCACCTCCCAGACCGTTGTTCTCTACACTCTGCTGGCCTCCATCTCCCTGCTCACTGTGGTGCTCAACCTGCTTGTCGTCATCTCCATCTCCCACTTCAGACAGCTTAACACCTCGACCAACACCCTGC
 ZebTAR.A001   CCCACCTCCCAGACCGTTGTTCTCTACACTCTGCTGGCCTCCATCTCCCTGCTCACTGTGGTGCTCAACCTGCTTGTCGTCATCTCCATCTCCCACTTCAGACAGCTTAACACCTCGACCAACACCCTGC
    Consensus  CCCACCTCCCAGACCGTTGTTCTCTACACTCTGCTGGCCTCCATCTCCCTGCTCACTGTGGTGCTCAACCTGCTTGTCGTCATCTCCATCTCCCACTTCAGACAGCTTAACACCTCGACCAACACCCTGC

               3121 3130      3140      3150      3160      3170      3180      3190      3200      3210      3220      3230      3240      3250
               |--------+---------+---------+---------+---------+---------+---------+---------+---------+---------+---------+---------+---------|
contig040586   TCCTTTCCCTGGCTGTGTCTGACCTGCTGGTGGGGTTGCTGGTGATGCCCATCGAGGGCCTGGACTACATCGAGACGTGCTGGCTGCTGGGGAGGCTGATGTGTGCTCTCAGTCCTTATTTGTCTTACTG
 ZebTAR.A001   TCCTTTCCCTGGCTGTGTCTGACCTGCTGGTGGGGTTGCTGGTGATGCCCATCGAGGGCCTGGACTACATCGAGACGTGCTGGCTGCTGGGGAGGCTGATGTGTGCTCTCAGTCCTTATTTGTCTTACTG
    Consensus  TCCTTTCCCTGGCTGTGTCTGACCTGCTGGTGGGGTTGCTGGTGATGCCCATCGAGGGCCTGGACTACATCGAGACGTGCTGGCTGCTGGGGAGGCTGATGTGTGCTCTCAGTCCTTATTTGTCTTACTG

               3251 3260      3270      3280      3290      3300      3310      3320      3330      3340      3350      3360      3370      3380
               |--------+---------+---------+---------+---------+---------+---------+---------+---------+---------+---------+---------+---------|
contig040586   CCTCATCTCTGTCTCTCTGGACAGCATGGTGCTCATATCTGTAGATCGCTATATAGCCATCTGTGACCCTCTGCTCTATTCCTCAAAGATCACAGTGAACAGAGTGAAGCTTTCAGTCTGTGTCTGCTGG
 ZebTAR.A001   CCTCATCTCTGTCTCTCTGGACAGCATGGTGCTCATATCTGTAGATCGCTATATAGCCATCTGTGACCCTCTGCTCTATTCCTCAAAGATCACAGTGAACAGAGTGAAGCTTTCAGTCTGTGTCTGCTGG
    Consensus  CCTCATCTCTGTCTCTCTGGACAGCATGGTGCTCATATCTGTAGATCGCTATATAGCCATCTGTGACCCTCTGCTCTATTCCTCAAAGATCACAGTGAACAGAGTGAAGCTTTCAGTCTGTGTCTGCTGG

               3381 3390      3400      3410      3420      3430      3440      3450      3460      3470      3480      3490      3500      3510
               |--------+---------+---------+---------+---------+---------+---------+---------+---------+---------+---------+---------+---------|
contig040586   GCCTGCTCTCTTCTCTACAATGGCTGCATTCTCATGGAGCACATAGGGTGGCCAGACAGGTTCAGCTCCTGTCACGGGGAGTGTGTGGTGTTCATCAGCCGCGCTTTGGGTACAATAGATCTCTTTTTCT
 ZebTAR.A001   GCCTGCTCTCTTCTCTACAATGGCTGCATTCTCATGGAGCACATAGGGTGGCCAGACAGGTTCAGCTCCTGTCACGGGGAGTGTGTGGTGTTCATCAGCCGCGCTTTGGGTACAATAGATCTCTTTTTCT
    Consensus  GCCTGCTCTCTTCTCTACAATGGCTGCATTCTCATGGAGCACATAGGGTGGCCAGACAGGTTCAGCTCCTGTCACGGGGAGTGTGTGGTGTTCATCAGCCGCGCTTTGGGTACAATAGATCTCTTTTTCT

               3511 3520      3530      3540      3550      3560      3570      3580      3590      3600      3610      3620      3630      3640
               |--------+---------+---------+---------+---------+---------+---------+---------+---------+---------+---------+---------+---------|
contig040586   CATTTCTCGGCCCATGTGCCTTAATGTTTGTTCTGTACATGAGGGTCTTTGTGGTTGCTGTTTCTCAGGTACGTATCATTCGGTCGCAGGCAGCTGTGAGAGCAGCTCCAGCTGCTAAAAAATCAGAGCT
 ZebTAR.A001   CATTTCTCGGCCCATGTGCCTTAATGTTTGTTCTGTACATGAGGGTCTTTGTGGTTGCTGTTTCTCAGGTACGTATCATTCGGTCGCAGGCAGCTGTGAGAGCAGCTCCAGCTGCTAAAAAATCAGAGCT
    Consensus  CATTTCTCGGCCCATGTGCCTTAATGTTTGTTCTGTACATGAGGGTCTTTGTGGTTGCTGTTTCTCAGGTACGTATCATTCGGTCGCAGGCAGCTGTGAGAGCAGCTCCAGCTGCTAAAAAATCAGAGCT

               3641 3650      3660      3670      3680      3690      3700      3710      3720      3730      3740      3750      3760      3770
               |--------+---------+---------+---------+---------+---------+---------+---------+---------+---------+---------+---------+---------|
contig040586   GAAGGCAGCCAGGACACTCGGGATTTTGATAGCTGTGTTTCTAATGTGCTTCTGCCCTTATTATTATCCTTCCTTTGCAGGTGACGACACCTCTATGAGCTTACCTTATTACGCTCTATTCTCGTGGATA
 ZebTAR.A001   GAAGGCAGCCAGGACACTCGGGATTTTGATAGCTGTGTTTCTAATGTGCTTCTGCCCTTATTATTATCCTTCCTTTGCAGGTGACGACACCTCTATGAGCTTACCTTATTACGCTCTATTCTCGTGGATA
    Consensus  GAAGGCAGCCAGGACACTCGGGATTTTGATAGCTGTGTTTCTAATGTGCTTCTGCCCTTATTATTATCCTTCCTTTGCAGGTGACGACACCTCTATGAGCTTACCTTATTACGCTCTATTCTCGTGGATA

               3771 3780      3790      3800      3810      3820      3830      3840      3850      3860      3870      3880      3890      3900
               |--------+---------+---------+---------+---------+---------+---------+---------+---------+---------+---------+---------+---------|
contig040586   ATGCTGACAAATTCTTGTGTGAACCCTGTGATTTATGCTCTGTTCTACCCCTGGTTTAGAAGAGCTATCAGACTCATTGTCACCCTCAGAATACTGCAGCCTCACTCCAGAGATGTCAAGATCCTGTAGG
 ZebTAR.A001   ATGCTGACAAATTCTTGTGTGAACCCTGTGATTTATGCTCTGTTCTACCCCTGGTTTAGAAGAGCTATCAGACTCATTGTCACCCTCAGAATACTGCAGCCTCACTCCAGAGATGTCAAGATCCTGTAG
    Consensus  ATGCTGACAAATTCTTGTGTGAACCCTGTGATTTATGCTCTGTTCTACCCCTGGTTTAGAAGAGCTATCAGACTCATTGTCACCCTCAGAATACTGCAGCCTCACTCCAGAGATGTCAAGATCCTGTAG.

               3901 3910      3920      3930      3940      3950      3960      3970      3980      3990      4000      4010      4020      4030
               |--------+---------+---------+---------+---------+---------+---------+---------+---------+---------+---------+---------+---------|
contig040586   CAGCAGTAAAACAAACCAGTCATTAGTCTTTTCTTAAAAGAATAATCAAAAACAACTATGCCACTGCTCCACAGAGATCCTTCACAGTGGCCTCACGATCAAACAATGTTCACCACACTTGCGTCTATTG
 ZebTAR.A001
    Consensus  ..................................................................................................................................
```

```
  Consensus  ..................................................................................................................................

             6111 6120      6130      6140      6150      6160      6170      6180      6190      6200      6210      6220      6230      6240
             |--------+---------+---------+---------+---------+---------+---------+---------+---------+---------+---------+---------+---------|
contig040586 GTGATGAAAACAAAGACAAGAAGTCTCATCATTCATGTTTAAAAAAAGAAAAAGAAAAAAATGACGGTGTGTGTTTGAAGAAGATAAATGAAGACTCTCGAGCACAGTTGCAGGTCTTCCCTTTCTCCCC
 ZebTAR.A002
  Consensus  ..................................................................................................................................

             6241 6250      6260      6270      6280      6290      6300      6310      6320      6330      6340      6350      6360      6370
             |--------+---------+---------+---------+---------+---------+---------+---------+---------+---------+---------+---------+---------|
contig040586 GATGGACGGCACTGGAGGTCCTCCCCTCTGCTTCCCTAACCTCAACTCCTCCTGCAGGCGGCTGCTGCGACCCACCTCCCAGGCCGCTCTTCTCTACACTCTGCTGGCTTCAGTCTCACTGCTTACTGTG
 ZebTAR.A002  ATGGACGGCACTGGAGGTCCTCCCCTCTGCTTCCCTAACCTCAACTCCTCCTGCAGGCGGCTGCTGCGACCCACCTCCCAGGCCGCTCTTCTCTACACTCTGCTGGCTTCAGTCTCACTGCTTACTGTG
  Consensus  .ATGGACGGCACTGGAGGTCCTCCCCTCTGCTTCCCTAACCTCAACTCCTCCTGCAGGCGGCTGCTGCGACCCACCTCCCAGGCCGCTCTTCTCTACACTCTGCTGGCTTCAGTCTCACTGCTTACTGTG

             6371 6380      6390      6400      6410      6420      6430      6440      6450      6460      6470      6480      6490      6500
             |--------+---------+---------+---------+---------+---------+---------+---------+---------+---------+---------+---------+---------|
contig040586 GTGCTCAACCTGCTTGTGGTCGTCTCCATCTCCCACTTCAAGCAGCTCCACACCCCGACCAACGCCCTGCTCCTGTCCCTGGCCATGTCTGACCTGCTGGTGGGGTTGCTGGTGATGCCCATCGAGGGCC
 ZebTAR.A002 GTGCTCAACCTGCTTGTGGTCGTCTCCATCTCCCACTTCAAGCAGCTCCACACCCCGACCAACGCCCTGCTCCTGTCCCTGGCCATGTCTGACCTGCTGGTGGGGTTGCTGGTGATGCCCATCGAGGGCC
  Consensus  GTGCTCAACCTGCTTGTGGTCGTCTCCATCTCCCACTTCAAGCAGCTCCACACCCCGACCAACGCCCTGCTCCTGTCCCTGGCCATGTCTGACCTGCTGGTGGGGTTGCTGGTGATGCCCATCGAGGGCC

             6501 6510      6520      6530      6540      6550      6560      6570      6580      6590      6600      6610      6620      6630
             |--------+---------+---------+---------+---------+---------+---------+---------+---------+---------+---------+---------+---------|
contig040586 TGCGCTACATCGAGACGTGCTGGCTGCTGGGGAGGCTGATGTGTGCTCTCAGTCCTTATTTGTCTTACTGCCTTATCTCTGTCTCTCTGGGCAGCATGGTGCTCATATCTGTAGATCGCTATATAGCCAT
 ZebTAR.A002 TGCGCTACATCGAGACGTGCTGGCTGCTGGGGAGGCTGATGTGTGCTCTCAGTCCTTATTTGTCTTACTGCCTTATCTCTGTCTCTCTGGGCAGCATGGTGCTCATATCTGTAGATCGCTATATAGCCAT
  Consensus  TGCGCTACATCGAGACGTGCTGGCTGCTGGGGAGGCTGATGTGTGCTCTCAGTCCTTATTTGTCTTACTGCCTTATCTCTGTCTCTCTGGGCAGCATGGTGCTCATATCTGTAGATCGCTATATAGCCAT

             6631 6640      6650      6660      6670      6680      6690      6700      6710      6720      6730      6740      6750      6760
             |--------+---------+---------+---------+---------+---------+---------+---------+---------+---------+---------+---------+---------|
contig040586 CTGTGACCCTCTGCTCTATTCCTCAAAGATCACAGTGAACAGAGTGAAGCTTTCAGTCTGTGTCTGCTGGGCCTGCTCTCTTCTCTACAATGGCTGCATTCTCATGGAGCACATAGGGTGGCCAGACAGG
 ZebTAR.A002 CTGTGACCCTCTGCTCTATTCCTCAAAGATCACAGTGAACAGAGTGAAGCTTTCAGTCTGTGTCTGCTGGGCCTGCTCTCTTCTCTACAATGGCTGCATTCTCATGGAGCACATAGGGTGGCCAGACAGG
  Consensus  CTGTGACCCTCTGCTCTATTCCTCAAAGATCACAGTGAACAGAGTGAAGCTTTCAGTCTGTGTCTGCTGGGCCTGCTCTCTTCTCTACAATGGCTGCATTCTCATGGAGCACATAGGGTGGCCAGACAGG

             6761 6770      6780      6790      6800      6810      6820      6830      6840      6850      6860      6870      6880      6890
             |--------+---------+---------+---------+---------+---------+---------+---------+---------+---------+---------+---------+---------|
contig040586 TTCAGCTCCTGTCACGGGGAGTGTGTTGTGTTCATCAGCCAAACTTCAGGAACAGTAGATTTCTTTTTGTCATTTCTTGGCCCCTGTGGCGTAATGTTTGTTCTGTACATGAGGGTGTTTGTGGTTGCTG
 ZebTAR.A002 TTCAGCTCCTGTCACGGGGAGTGTGTTGTGTTCATCAGCCAAACTTCAGGAACAGTAGATTTCTTTTTGTCATTTCTTGGCCCCTGTGGCGTAATGTTTGTTCTGTACATGAGGGTGTTTGTGGTTGCTG
  Consensus  TTCAGCTCCTGTCACGGGGAGTGTGTTGTGTTCATCAGCCAAACTTCAGGAACAGTAGATTTCTTTTTGTCATTTCTTGGCCCCTGTGGCGTAATGTTTGTTCTGTACATGAGGGTGTTTGTGGTTGCTG

             6891 6900      6910      6920      6930      6940      6950      6960      6970      6980      6990      7000      7010      7020
             |--------+---------+---------+---------+---------+---------+---------+---------+---------+---------+---------+---------+---------|
contig040586 TTTCTCAGGTGCGTGCCATTCAGTCGCAGGCAGCTGTGAGAGCAGCTCCAGCTGCTAAAAAATCAGAGCTGAAGGCAGCCAGGACACTCGGGATTTTGATAGCTGTGTTTGTAATGTGCTTCTGCCCTTA
 ZebTAR.A002 TTTCTCAGGTGCGTGCCATTCAGTCGCAGGCAGCTGTGAGAGCAGCTCCAGCTGCTAAAAAATCAGAGCTGAAGGCAGCCAGGACACTCGGGATTTTGATAGCTGTGTTTGTAATGTGCTTCTGCCCTTA
  Consensus  TTTCTCAGGTGCGTGCCATTCAGTCGCAGGCAGCTGTGAGAGCAGCTCCAGCTGCTAAAAAATCAGAGCTGAAGGCAGCCAGGACACTCGGGATTTTGATAGCTGTGTTTGTAATGTGCTTCTGCCCTTA

             7021 7030      7040      7050      7060      7070      7080      7090      7100      7110      7120      7130      7140      7150
             |--------+---------+---------+---------+---------+---------+---------+---------+---------+---------+---------+---------+---------|
contig040586 TTATTATCCTTCCCTTGCAGGTGTGGACACCTTTCTGAGCTTGCCTTCGTACGCCATGGTGTGTTGGATCATGCTAATAAATGCTTGTGTGAACCCTGTGATTTATGTTCTGTTTTACCCCTGGTTTAGA
 ZebTAR.A002 TTATTATCCTTCCCTTGCAGGTGTGGACACCTTTCTGAGCTTGCCTTCGTACGCCATGGTGTGTTGGATCATGCTAATAAATGCTTGTGTGAACCCTGTGATTTATGTTCTGTTTTACCCCTGGTTTAGA
  Consensus  TTATTATCCTTCCCTTGCAGGTGTGGACACCTTTCTGAGCTTGCCTTCGTACGCCATGGTGTGTTGGATCATGCTAATAAATGCTTGTGTGAACCCTGTGATTTATGTTCTGTTTTACCCCTGGTTTAGA

             7151 7160      7170      7180      7190      7200      7210      7220      7230      7240      7250      7260      7270      7280
             |--------+---------+---------+---------+---------+---------+---------+---------+---------+---------+---------+---------+---------|
contig040586 AGAGCTATCAGATTCATTGTCAGCCTCAGAATACTGCAGCCTCACTCCAGAGACGTCAAAATCCTGTAGGCAGCGCAATACAACTTTCACCTAAATTTTCACCTTTAAATGTGGGTGTTCATCTGCCCAG
 ZebTAR.A002 AGAGCTATCAGATTCATTGTCAGCCTCAGAATACTGCAGCCTCACTCCAGAGACGTCAAAATCCTGTAG
  Consensus  AGAGCTATCAGATTCATTGTCAGCCTCAGAATACTGCAGCCTCACTCCAGAGACGTCAAAATCCTGTAG.............................................................
```

```
             1443114440     14450     14460     14470     14480     14490     14500     14510     14520     14530     14540     14550     14560
             |--------+---------+---------+---------+---------+---------+---------+---------+---------+---------+---------+---------+---------|
contig003909 TCAGAGATAAATAAGCTGTACAATGTCTCACAGGGTCAGCAGGTTGCTTGAACTCAGCAGGTCCTCCTTTCTTTATACTGGATGATGGACAGGCAGGACAGAATTGAACTCTGCTACCCTGAGCTTGCGA
 ZebTAR.A003                                                                                     ATGGACAGGCAGGACAGAATTGAACTCTGCTACCCTGAGCTTGCGA
   Consensus ....................................................................................ATGGACAGGCAGGACAGAATTGAACTCTGCTACCCTGAGCTTGCGA

             1456114570     14580     14590     14600     14610     14620     14630     14640     14650     14660     14670     14680     14690
             |--------+---------+---------+---------+---------+---------+---------+---------+---------+---------+---------+---------+---------|
contig003909 ATCTGTCTTGCAGGGGCTTAACACGTCCTCAGCCTGAGGCTGTCCTCCTCTATACACTGTTCTTGTCTATCTCTGTGCTAACTGTAGCTCTGAATGTGCTGGTTATCATATCCATCTCTCATTTCAGGCA
 ZebTAR.A003 ATCTGTCTTGCAGGGGCTTAACACGTCCTCAGCCTGAGGCTGTCCTCCTCTATACACTGTTCTTGTCTATCTCTGTGCTAACTGTAGCTCTGAATGTGCTGGTTATCATATCCATCTCTCATTTCAGGCA
   Consensus ATCTGTCTTGCAGGGGCTTAACACGTCCTCAGCCTGAGGCTGTCCTCCTCTATACACTGTTCTTGTCTATCTCTGTGCTAACTGTAGCTCTGAATGTGCTGGTTATCATATCCATCTCTCATTTCAGGCA

             1469114700     14710     14720     14730     14740     14750     14760     14770     14780     14790     14800     14810     14820
             |--------+---------+---------+---------+---------+---------+---------+---------+---------+---------+---------+---------+---------|
contig003909 GCTCCACATGCCCACCAATGTCCTCCTGCTCTCTCTGGCCATCTCAGACCTCTTGGTCGGGCTGCTTGTGATGCCGGTGGAAGCAATGCGGTTTATAGAAACCTGCTGGCTGCTGGGAGATCTCATGTGT
 ZebTAR.A003 GCTCCACATGCCCACCAATGTCCTCCTGCTCTCTCTGGCCATCTCAGACCTCTTGGTCGGGCTGCTTGTGATGCCGGTGGAAGCAATGCGGTTTATAGAAACCTGCTGGCTGCTGGGAGATCTCATGTGT
   Consensus GCTCCACATGCCCACCAATGTCCTCCTGCTCTCTCTGGCCATCTCAGACCTCTTGGTCGGGCTGCTTGTGATGCCGGTGGAAGCAATGCGGTTTATAGAAACCTGCTGGCTGCTGGGAGATCTCATGTGT

             1482114830     14840     14850     14860     14870     14880     14890     14900     14910     14920     14930     14940     14950
             |--------+---------+---------+---------+---------+---------+---------+---------+---------+---------+---------+---------+---------|
contig003909 GCTTTTACTTACATTATCGGTTTCACTCTCACCTCAGCCTCTGTGGGCAACATGGTGCTCATATCAATTGATCGCTACGTAGCTATTTGTTATCCTCTGCAGTACCCCACTAAAATAACTCACAGCAGAG
 ZebTAR.A003 GCTTTTACTTACATTATCGGTTTCACTCTCACCTCAGCCTCTGTGGGCAACATGGTGCTCATATCAATTGATCGCTACGTAGCTATTTGTTATCCTCTGCAGTACCCCACTAAAATAACTCACAGCAGAG
   Consensus GCTTTTACTTACATTATCGGTTTCACTCTCACCTCAGCCTCTGTGGGCAACATGGTGCTCATATCAATTGATCGCTACGTAGCTATTTGTTATCCTCTGCAGTACCCCACTAAAATAACTCACAGCAGAG

             1495114960     14970     14980     14990     15000     15010     15020     15030     15040     15050     15060     15070     15080
             |--------+---------+---------+---------+---------+---------+---------+---------+---------+---------+---------+---------+---------|
contig003909 TGGAGCTGTCTGTGACTCTGTGCTGGGCCTGTTCGCTCCTCTACAACGGGATGATCTTAAAGGAGCATCTCAGGCAGCCAAACAGGCATAACACCTGTCACGGTCAGTGTTTGGTGGTGATTAACTATGT
 ZebTAR.A003 TGGAGCTGTCTGTGACTCTGTGCTGGGCCTGTTCGCTCCTCTACAACGGGATGATCTTAAAGGAGCATCTCAGGCAGCCAAACAGGCATAACACCTGTCACGGTCAGTGTTTGGTGGTGATTAACTATGT
   Consensus TGGAGCTGTCTGTGACTCTGTGCTGGGCCTGTTCGCTCCTCTACAACGGGATGATCTTAAAGGAGCATCTCAGGCAGCCAAACAGGCATAACACCTGTCACGGTCAGTGTTTGGTGGTGATTAACTATGT

             1508115090     15100     15110     15120     15130     15140     15150     15160     15170     15180     15190     15200     15210
             |--------+---------+---------+---------+---------+---------+---------+---------+---------+---------+---------+---------+---------|
contig003909 CTCTGGAGCAATCGACCTTGTCTTTACTTTCATCGGCCCTTGCTCAGTCATTATCATTCTGTACATGAGAGTATTTGTTGTAGCGGTGTCTCAGGCTCATGCCATGCGGTCTCATATTACAGCTGCTGCA
 ZebTAR.A003 CTCTGGAGCAATCGACCTTGTCTTTACTTTCATCGGCCCTTGCTCAGTCATTATCATTCTGTACATGAGAGTATTTGTTGTAGCGGTGTCTCAGGCTCATGCCATGCGGTCTCATATTACAGCTGCTGCA
   Consensus CTCTGGAGCAATCGACCTTGTCTTTACTTTCATCGGCCCTTGCTCAGTCATTATCATTCTGTACATGAGAGTATTTGTTGTAGCGGTGTCTCAGGCTCATGCCATGCGGTCTCATATTACAGCTGCTGCA

             1521115220     15230     15240     15250     15260     15270     15280     15290     15300     15310     15320     15330     15340
             |--------+---------+---------+---------+---------+---------+---------+---------+---------+---------+---------+---------+---------|
contig003909 GCTGGTACAGTAAAAATCACCGCAAAGAAATCAGAGAAAAAGGCAGCCAGGACTCTTGGCGTTGTGATATTTGTGTTTTTGATGAGTTTCTGTCCGTATTACTATCCCTCTCTTGCAGGGCAGGACATTT
 ZebTAR.A003 GCTGGTACAGTAAAAATCACCGCAAAGAAATCAGAGAAAAAGGCAGCCAGGACTCTTGGCGTTGTGATATTTGTGTTTTTGATGAGTTTCTGTCCGTATTACTATCCCTCTCTTGCAGGGCAGGACATTT
   Consensus GCTGGTACAGTAAAAATCACCGCAAAGAAATCAGAGAAAAAGGCAGCCAGGACTCTTGGCGTTGTGATATTTGTGTTTTTGATGAGTTTCTGTCCGTATTACTATCCCTCTCTTGCAGGGCAGGACATTT

             1534115350     15360     15370     15380     15390     15400     15410     15420     15430     15440     15450     15460     15470
             |--------+---------+---------+---------+---------+---------+---------+---------+---------+---------+---------+---------+---------|
contig003909 CAAACAGTGCTTCATCTTGGGCTATTGTTTCCTGGATGCTGTACTTCAATTCTTGTCTGAATCCACTGATATATGCTTTTTTCTACCCGTGGTTTAGAAAAGCTATCTGGTTCATTGTCTCCCTGAAGAT
 ZebTAR.A003 CAAACAGTGCTTCATCTTGGGCTATTGTTTCCTGGATGCTGTACTTCAATTCTTGTCTGAATCCACTGATATATGCTTTTTTCTACCCGTGGTTTAGAAAAGCTATCTGGTTCATTGTCTCCCTGAAGAT
   Consensus CAAACAGTGCTTCATCTTGGGCTATTGTTTCCTGGATGCTGTACTTCAATTCTTGTCTGAATCCACTGATATATGCTTTTTTCTACCCGTGGTTTAGAAAAGCTATCTGGTTCATTGTCTCCCTGAAGAT

             1547115480     15490     15500     15510     15520     15530     15540     15550     15560     15570     15580     15590     15600
             |--------+---------+---------+---------+---------+---------+---------+---------+---------+---------+---------+---------+---------|
contig003909 ACTAGAGAAGGGCTCCTCTCAGGCAAATATACTTTAAAATAGCAAATTTATCCTGAACCAAATCAGTCTGTATGAGAGAATGAAACTGCTGATTATTATATGTGGCAGTAGCATAATGGTTTTTAATTAT
 ZebTAR.A003 ACTAGAGAAGGGCTCCTCTCAGGCAAATATACTTTAA
   Consensus ACTAGAGAAGGGCTCCTCTCAGGCAAATATACTTTAA.............................................................................................
```

```
             2081   2090      2100      2110      2120      2130      2140      2150      2160      2170      2180      2190      2200      2210
contig066890 CCAGAGCTGGAGCTCTCTGCAGATCTGTGGAGATGGATACTCAGGATGGAGAAGAGCTCTGCTTTCCACAACTCTTCAACATCTCCTGCAAGAAGCCTACAACTCCTCTGTCTCAATTGTTGCTTCCTTA
ZebTARs.A014                                 ATGGATACTCAGGATGGAGAAGAGCTCTGCTTTCCACAACTCTTCAACATCTCCTGCAAGAAGCCTACAACTCCTCTGTCTCAATTGTTGCTTCCTTA
Consensus    ................................ATGGATACTCAGGATGGAGAAGAGCTCTGCTTTCCACAACTCTTCAACATCTCCTGCAAGAAGCCTACAACTCCTCTGTCTCAATTGTTGCTTCCTTA

             2211   2220      2230      2240      2250      2260      2270      2280      2290      2300      2310      2320      2330      2340
contig066890 TATTGTTGTGTTCTCAATGTCTCTGCTGACTGTGATTCTCAACCTGCTCGTCATCATCGCAGTCTCCCACTTCAGGCAGAGTTTCACTTTTGTAACTGTTATTGTAGCTTTACAAGTCAGAGTCAGCTTA
ZebTARs.A014 TATTGTTGTGTTCTCAATGTCTCTGCTGACTGTGATTCTCAACCTGCTCGTCATCATCGCAGTCTCCCACTTCAG-------------------------------------------------------
Consensus    TATTGTTGTGTTCTCAATGTCTCTGCTGACTGTGATTCTCAACCTGCTCGTCATCATCGCAGTCTCCCACTTCAG.......................................................

             2341   2350      2360      2370      2380      2390      2400      2410      2420      2430      2440      2450      2460      2470
contig066890 AGTAAATACATGATTTTACTCATATGTGCACTAGACTTACATTTGAACTGTTAGACTATTTTTATATAATGATCTGTATATATTAGATGTTAATATTAGATGACAGACTTAGAGAGTTTAGATATTCATT
ZebTARs.A014 ----------------------------------------------------------------------------------------------------------------------------------
Consensus    ..................................................................................................................................

             2471   2480      2490      2500      2510      2520      2530      2540      2550      2560      2570      2580      2590      2600
contig066890 ATATGAGGTACTTCATACCATATTCCTTCTCCTACCAATAATCTTTACATTCAGTCTGCTACAACTTATCTTCTGTCTACACTTGAACATACTACACCTATAACTGAATTATTTTCAGATGTTTCAGATG
ZebTARs.A014 ----------------------------------------------------------------------------------------------------------------------------------
Consensus    ..................................................................................................................................

             2601   2610      2620      2630      2640      2650      2660      2670      2680      2690      2700      2710      2720      2730
contig066890 ATTTCTCTCTCTTCTTTCAGACAGCTCCACACACCCACTAACTTCCTCCTCCTCTCTCTGGCTGTGTCAGACTTTTTCATTGGTCTGTTGTTGATGCCTGCAAATATCATCCGAAAGAAGGTTTGTTGGT
ZebTARs.A014 --------------------ACAGCTCCACACACCCACTAACTTCCTCCTCCTCTCTCTGGCTGTGTCAGACTTTTTCATTGGTCTGTTGTTGATGCCTGCAAATATCATCCGAAAGAAGGTTTGTTGGT
Consensus    ....................ACAGCTCCACACACCCACTAACTTCCTCCTCCTCTCTCTGGCTGTGTCAGACTTTTTCATTGGTCTGTTGTTGATGCCTGCAAATATCATCCGAAAGAAGGTTTGTTGGT

             2731   2740      2750      2760      2770      2780      2790      2800      2810      2820      2830      2840      2850      2860
contig066890 TTCTTGGTCAGCTTGCATGTTTAGTGTTTACTTATGTATCCTTCATTATTACCTCTGCCTCAGTGGGCATCATGGTGCTGATATCAGTCGACAGGTATGCGGCTATTTGTGACCCTCTGCATTACCCCAC
ZebTARs.A014 TTCTTGGTCAGCTTGCATGTTTAGTGTTTACTTATGTATCCTTCATTATTACCTCTGCCTCAGTGGGCATCATGGTGCTGATATCAGTCGACAGGTATGCGGCTATTTGTGACCCTCTGCATTACCCCAC
Consensus    TTCTTGGTCAGCTTGCATGTTTAGTGTTTACTTATGTATCCTTCATTATTACCTCTGCCTCAGTGGGCATCATGGTGCTGATATCAGTCGACAGGTATGCGGCTATTTGTGACCCTCTGCATTACCCCAC

             2861   2870      2880      2890      2900      2910      2920      2930      2940      2950      2960      2970      2980      2990
contig066890 CAGAATAACTCGTGAAAGAGTGAAACTCTGTGTCTGTCTGTGTTGGCTCTGCTCTGTTATCTACAACATTTTGCTCATAAAGGATGAACTGGTTCAACCAGGGCAACATAATTCCTGCTATGGAGAGTGT
ZebTARs.A014 CAGAATAACTCGTGAAAGAGTGAAACTCTGTGTCTGTCTGTGTTGGCTCTGCTCTGTTATCTACAACATTTTGCTCATAAAGGATGAACTGGTTCAACCAGGGCAACATAATTCCTGCTATGGAGAGTGT
Consensus    CAGAATAACTCGTGAAAGAGTGAAACTCTGTGTCTGTCTGTGTTGGCTCTGCTCTGTTATCTACAACATTTTGCTCATAAAGGATGAACTGGTTCAACCAGGGCAACATAATTCCTGCTATGGAGAGTGT

             2991   3000      3010      3020      3030      3040      3050      3060      3070      3080      3090      3100      3110      3120
contig066890 AAAATTCTCGTAGACTACATTGCAGGAACCACTGATATTGTTTTAACCTTTATTGCTCCAATTACTGTCATCATAGTTCTGTATATGAGAGTGTTTGTGGTGGCTGTGTCTCAGGCCCGTACCATGCGCT
ZebTARs.A014 AAAATTCTCGTAGACTACATTGCAGGAACCACTGATATTGTTTTAACCTTTATTGCTCCAATTACTGTCATCATAGTTCTGTATATGAGAGTGTTTGTGGTGGCTGTGTCTCAGGCCCGTACCATGCGCT
Consensus    AAAATTCTCGTAGACTACATTGCAGGAACCACTGATATTGTTTTAACCTTTATTGCTCCAATTACTGTCATCATAGTTCTGTATATGAGAGTGTTTGTGGTGGCTGTGTCTCAGGCCCGTACCATGCGCT

             3121   3130      3140      3150      3160      3170      3180      3190      3200      3210      3220      3230      3240      3250
contig066890 CTCAGGTTACAGCTGTCACACTACAGCTCTCAGTGACTCTAACAACAAAGAAATCAGAGTTAAAAGCAGCCAGGACTCTGGGTGTTCTTGTTCTTGTGTTTCTAATATGTTTCTGTCCTCATTATTGTAT
ZebTARs.A014 CTCAGGTTACAGCTGTCACACTACAGCTCTCAGTGACTCTAACAACAAAGAAATCAGAGTTAAAAGCAGCCAGGACTCTGGGTGTTCTTGTTCTTGTGTTTCTAATATGTTTCTGTCCTCATTATTGTAT
Consensus    CTCAGGTTACAGCTGTCACACTACAGCTCTCAGTGACTCTAACAACAAAGAAATCAGAGTTAAAAGCAGCCAGGACTCTGGGTGTTCTTGTTCTTGTGTTTCTAATATGTTTCTGTCCTCATTATTGTAT

             3251   3260      3270      3280      3290      3300      3310      3320      3330      3340      3350      3360      3370      3380
contig066890 TTTTGTTTTTGGAGGAAAGGTGCTTAATAGCTCATCTGCAACTATTGTGATCTATCTGTATTATTTTAACTCCTGTCTAAATCCTTTGATTTATGCTATGTTCTACCCCTGGTTTAGAAAAGCTGTGAAA
ZebTARs.A014 TTTTGTTTTTGGAGGAAAGGTGCTTAATAGCTCATCTGCAACTATTGTGATCTATCTGTATTATTTTAACTCCTGTCTAAATCCTTTGATTTATGCTATGTTCTACCCCTGGTTTAGAAAAGCTGTGAAA
Consensus    TTTTGTTTTTGGAGGAAAGGTGCTTAATAGCTCATCTGCAACTATTGTGATCTATCTGTATTATTTTAACTCCTGTCTAAATCCTTTGATTTATGCTATGTTCTACCCCTGGTTTAGAAAAGCTGTGAAA

             3381   3390      3400      3410      3420      3430      3440      3450      3460      3470      3480      3490      3500      3510
contig066890 CTAATTGTCACACTACAGATACTGCAGCCTGGCTCCTGTGAGGTCAGCATACTGTAGAAAAAAGAAATCTCCTTTAAAAATATTTTTACGGGCAACACTAAACAAGCAGAAGATAAAATCTTTTCTGTCA
ZebTARs.A014 CTAATTGTCACACTACAGATACTGCAGCCTGGCTCCTGTGAGGTCAGCATACTGTAG
Consensus    CTAATTGTCACACTACAGATACTGCAGCCTGGCTCCTGTGAGGTCAGCATACTGTAG.........................................................................
```

```
             781      790       800       810       820       830       840       850       860       870       880       890       900       910
             |--------+---------+---------+---------+---------+---------+---------+---------+---------+---------+---------+---------+---------|
contig066691 AGACTGAAAGAGATTGGTGGGAGGAGTGAGATAAATTAGCTGTATGAGAAGAATTTCTAATTAGAGAGCTGCAGGTGTCTGATGATGGAGATACCGAAAGGAGTCGAGCTCTGTTTCCCACAACTCCTCA
ZebTARs.A015                                                                                     ATGGAGATACCGAAAGGAGTCGAGCTCTGTTTCCCACAACTCCTCA
   Consensus ....................................................................................ATGGAGATACCGAAAGGAGTCGAGCTCTGTTTCCCACAACTCCTCA

             911      920       930       940       950       960       970       980       990       1000      1010      1020      1030      1040
             |--------+---------+---------+---------+---------+---------+---------+---------+---------+---------+---------+---------+---------|
contig066691 ACAGCTCCTGCAGGAAGCCGACACTTCACTGGTCCAAAGCTGTGCTCCTGAACATTGTGCTCTCATGTATCTCTCTGCTCACTGCTGCTCTAAACCTTCTCGTCATCATCTCAGTCTCCCACTTCAGGCA
ZebTARs.A015 ACAGCTCCTGCAGGAAGCCGACACTTCACTGGTCCAAAGCTGTGCTCCTGAACATTGTGCTCTCATGTATCTCTCTGCTCACTGCTGCTCTAAACCTTCTCGTCATCATCTCAGTCTCCCACTTCAGGCA
   Consensus ACAGCTCCTGCAGGAAGCCGACACTTCACTGGTCCAAAGCTGTGCTCCTGAACATTGTGCTCTCATGTATCTCTCTGCTCACTGCTGCTCTAAACCTTCTCGTCATCATCTCAGTCTCCCACTTCAGGCA

             1041     1050      1060      1070      1080      1090      1100      1110      1120      1130      1140      1150      1160      1170
             |--------+---------+---------+---------+---------+---------+---------+---------+---------+---------+---------+---------+---------|
contig066691 GAGATTAACTTTTCAACTGAAACTACAGTTTAAGTTTTAGATGTGTGAAAGCTTCCATTTACATTATATGACTTAGTGATTAAAGAATGAATTTTAACCATGGTCATAATGTCTTATTTGCTTGAATTGA
ZebTARs.A015 G---------------------------------------------------------------------------------------------------------------------------------
   Consensus G.................................................................................................................................

             1171     1180      1190      1200      1210      1220      1230      1240      1250      1260      1270      1280      1290      1300
             |--------+---------+---------+---------+---------+---------+---------+---------+---------+---------+---------+---------+---------|
contig066691 ATCATGCCTTAGTAGTGCAGCATTTTCACTGAAGACATGTTGACATTAACGATGTGCATTATATTCTTTTCTGTCTCCCTGCAGGCAGCTGCACACACCCAGTAATATCCTCCTCCTCTCTCTGGCCGTC
ZebTARs.A015 ----------------------------------------------------------------------------------------CTGCACACACCCAGTAATATCCTCCTCCTCTCTCTGGCCGTC
   Consensus ........................................................................................CTGCACACACCCAGTAATATCCTCCTCCTCTCTCTGGCCGTC

             1301     1310      1320      1330      1340      1350      1360      1370      1380      1390      1400      1410      1420      1430
             |--------+---------+---------+---------+---------+---------+---------+---------+---------+---------+---------+---------+---------|
contig066691 TCAGACTTTTTTGTGGGTCTCCTGTTGTTGCCTTTGGAAATTTTTAGAAACACAAGCTGCTGGGTACTTGGTGATCGTATGTGTTCTGCTTATTGGTATTTGACCAGCAACATTATCTGTGCTTCAATAG
ZebTARs.A015 TCAGACTTTTTTGTGGGTCTCCTGTTGTTGCCTTTGGAAATTTTTAGAAACACAAGCTGCTGGGTACTTGGTGATCGTATGTGTTCTGCTTATTGGTATTTGACCAGCAACATTATCTGTGCTTCAATAG
   Consensus TCAGACTTTTTTGTGGGTCTCCTGTTGTTGCCTTTGGAAATTTTTAGAAACACAAGCTGCTGGGTACTTGGTGATCGTATGTGTTCTGCTTATTGGTATTTGACCAGCAACATTATCTGTGCTTCAATAG

             1431     1440      1450      1460      1470      1480      1490      1500      1510      1520      1530      1540      1550      1560
             |--------+---------+---------+---------+---------+---------+---------+---------+---------+---------+---------+---------+---------|
contig066691 GGAACATTGTTCTAATATCAGTTGACCGCTATGTGGCTATTTGTGACCCTCTGCATTATCCCAGCAGAATTACTTTGGCGAAAGTCAAACTCAGTGTTTGTCTCTGTTGGTTTTATGCTTTTTTCTACAG
ZebTARs.A015 GGAACATTGTTCTAATATCAGTTGACCGCTATGTGGCTATTTGTGACCCTCTGCATTATCCCAGCAGAATTACTTTGGCGAAAGTCAAACTCAGTGTTTGTCTCTGTTGGTTTTATGCTTTTTTCTACAG
   Consensus GGAACATTGTTCTAATATCAGTTGACCGCTATGTGGCTATTTGTGACCCTCTGCATTATCCCAGCAGAATTACTTTGGCGAAAGTCAAACTCAGTGTTTGTCTCTGTTGGTTTTATGCTTTTTTCTACAG

             1561     1570      1580      1590      1600      1610      1620      1630      1640      1650      1660      1670      1680      1690
             |--------+---------+---------+---------+---------+---------+---------+---------+---------+---------+---------+---------+---------|
contig066691 CAATCTTTATACAAAGAATATCATGATTGAACCAGGCAGGTATAATTCCTGCTTTGGAGAGTGTGTATTTTTTAGCAGTAATATTGCTATTGTTGCTGACCTTATTTTATTCTTTTTTGTTCCAGTAACT
ZebTARs.A015 CAATCTTTATACAAAGAATATCATGATTGAACCAGGCAGGTATAATTCCTGCTTTGGAGAGTGTGTATTTTTTAGCAGTAATATTGCTATTGTTGCTGACCTTATTTTATTCTTTTTTGTTCCAGTAACT
   Consensus CAATCTTTATACAAAGAATATCATGATTGAACCAGGCAGGTATAATTCCTGCTTTGGAGAGTGTGTATTTTTTAGCAGTAATATTGCTATTGTTGCTGACCTTATTTTATTCTTTTTTGTTCCAGTAACT

             1691     1700      1710      1720      1730      1740      1750      1760      1770      1780      1790      1800      1810      1820
             |--------+---------+---------+---------+---------+---------+---------+---------+---------+---------+---------+---------+---------|
contig066691 GTTATCATAGCCTTGTATATGAGAGTATTTGTGGTGGCTGTGTCTCAAGCTCGTGCCATGCGCTCTCATGTTACATTGGTCACACTTCAGCGTTCACTGAATCAAACAAACAAATCTGAGCTGAAAGCAG
ZebTARs.A015 GTTATCATAGCCTTGTATATGAGAGTATTTGTGGTGGCTGTGTCTCAAGCTCGTGCCATGCGCTCTCATGTTACATTGGTCACACTTCAGCGTTCACTGAATCAAACAAACAAATCTGAGCTGAAAGCAG
   Consensus GTTATCATAGCCTTGTATATGAGAGTATTTGTGGTGGCTGTGTCTCAAGCTCGTGCCATGCGCTCTCATGTTACATTGGTCACACTTCAGCGTTCACTGAATCAAACAAACAAATCTGAGCTGAAAGCAG

             1821     1830      1840      1850      1860      1870      1880      1890      1900      1910      1920      1930      1940      1950
             |--------+---------+---------+---------+---------+---------+---------+---------+---------+---------+---------+---------+---------|
contig066691 CCAGGACTCTGGGGATTCTTGTAGTTGTGTTTCTAGCATGCTTCTCTCCACTCTACTGCTACTCTCTTGTTGATGAAAATGCAATCAATAATCCAGCTGCATCTTTTGCGGTCATTATCTTTTACATTAA
ZebTARs.A015 CCAGGACTCTGGGGATTCTTGTAGTTGTGTTTCTAGCATGCTTCTCTCCACTCTACTGCTACTCTCTTGTTGATGAAAATGCAATCAATAATCCAGCTGCATCTTTTGCGGTCATTATCTTTTACATTAA
   Consensus CCAGGACTCTGGGGATTCTTGTAGTTGTGTTTCTAGCATGCTTCTCTCCACTCTACTGCTACTCTCTTGTTGATGAAAATGCAATCAATAATCCAGCTGCATCTTTTGCGGTCATTATCTTTTACATTAA

             1951     1960      1970      1980      1990      2000      2010      2020      2030      2040      2050      2060      2070      2080
             |--------+---------+---------+---------+---------+---------+---------+---------+---------+---------+---------+---------+---------|
contig066691 CTCTTGTCTAAACCCTTTGATCTATGCCTTGTTTTACCCCTGGTTTAGAAATGCTGTTAAACTTATCATCACGTTGGAGATCTTCAAGTATGACACAAGTGGTGCCAACATACTGTAAAAAGAGTACAAA
ZebTARs.A015 CTCTTGTCTAAACCCTTTGATCTATGCCTTGTTTTACCCCTGGTTTAGAAATGCTGTTAAACTTATCATCACGTTGGAGATCTTCAAGTATGACACAAGTGGTGCCAACATACTGTAA
   Consensus CTCTTGTCTAAACCCTTTGATCTATGCCTTGTTTTACCCCTGGTTTAGAAATGCTGTTAAACTTATCATCACGTTGGAGATCTTCAAGTATGACACAAGTGGTGCCAACATACTGTAA............
```

```
           781  790       800       810       820       830       840       850       860       870       880       890       900       910
contig066285 AGGGAAAGCAGAAAGAGATAAATTAGTTCTATGAGAAGTCTTGGTCAAAGAGCGGTGGGAGGCGTAGCTGTTTGGTGATGGAGAAAGGAGCCGAGCTCTGCTTTCCACATCTCTTCAACAATTCCTGCAT
ZebTARs.A016                                                                              ATGGAGAAAGGAGCCGAGCTCTGCTTTCCACATCTCTTCAACAATTCCTGCAT
   Consensus .............................................................................ATGGAGAAAGGAGCCGAGCTCTGCTTTCCACATCTCTTCAACAATTCCTGCAT

           911  920       930       940       950       960       970       980       990       1000      1010      1020      1030      1040
contig066285 CAAGCCGACACTTCACTGGTCCAAAGCCGTGCTCCTGAACATTGTGCTGTCGTGCATCTCTTTGATCACTGCTGGTCTCAATCTTCTTGTTATCATCTCAGTCTCCCATTTCAGGCAGAGATTGACTTAA
ZebTARs.A016 CAAGCCGACACTTCACTGGTCCAAAGCCGTGCTCCTGAACATTGTGCTGTCGTGCATCTCTTTGATCACTGCTGGTCTCAATCTTCTTGTTATCATCTCAGTCTCCCATTTCAGGCAG------------
   Consensus CAAGCCGACACTTCACTGGTCCAAAGCCGTGCTCCTGAACATTGTGCTGTCGTGCATCTCTTTGATCACTGCTGGTCTCAATCTTCTTGTTATCATCTCAGTCTCCCATTTCAGGCAG............

           1041 1050      1060      1070      1080      1090      1100      1110      1120      1130      1140      1150      1160      1170
contig066285 ACTTTAGCTTAAATTTTATAAACTTTGAGAGTATGATTCATGTCATATGATTTGTTGATGTTCGAATGAAGTGAATCTGACATATTCTCCATGTATTTTAAACAAACTGTTGTGTAATTCATTATCAATG
ZebTARs.A016 ----------------------------------------------------------------------------------------------------------------------------------
   Consensus ..................................................................................................................................

           1171 1180      1190      1200      1210      1220      1230      1240      1250      1260      1270      1280      1290      1300
contig066285 AATCATTTTCTTACTTAAGACATGAAAAGATTAATGATTATTATTTTCTCTTCTGTGTCCCTGCAGGCAGCTCCACACACCCAGTAACATCCTCCTCCTCTCTCTGGCTGTGTCAGACTTTCTTGTTGGT
ZebTARs.A016 ----------------------------------------------------------------------CTCCACACACCCAGTAACATCCTCCTCCTCTCTCTGGCTGTGTCAGACTTTCTTGTTGGT
   Consensus ......................................................................CTCCACACACCCAGTAACATCCTCCTCCTCTCTCTGGCTGTGTCAGACTTTCTTGTTGGT

           1301 1310      1320      1330      1340      1350      1360      1370      1380      1390      1400      1410      1420      1430
contig066285 CTCCTGTTAATTCCAGCTGAGATTTTTAGAATCACAGTTTGCTGGGTATTTGGTGAATCCATGTGTTCACTTTATACATATCTAGGCTACATTGTTGTAACATCTTCAATAAGTAACATAGTTCTCATAT
ZebTARs.A016 CTCCTGTTAATTCCAGCTGAGATTTTTAGAATCACAGTTTGCTGGGTATTTGGTGAATCCATGTGTTCACTTTATACATATCTAGGCTACATTGTTGTAACATCTTCAATAAGTAACATAGTTCTCATAT
   Consensus CTCCTGTTAATTCCAGCTGAGATTTTTAGAATCACAGTTTGCTGGGTATTTGGTGAATCCATGTGTTCACTTTATACATATCTAGGCTACATTGTTGTAACATCTTCAATAAGTAACATAGTTCTCATAT

           1431 1440      1450      1460      1470      1480      1490      1500      1510      1520      1530      1540      1550      1560
contig066285 CAGTTGATCGCTATGTGGCAATTTGTGATCCTCTGCATTACCCCAGTAGAATTTCTGTGGCAAAAATAAGTCTTAGTGTTTGTATGTGTTGGTTTTATTCAGCTTTCTACAGCACTCTTTGCACAAAGAA
ZebTARs.A016 CAGTTGATCGCTATGTGGCAATTTGTGATCCTCTGCATTACCCCAGTAGAATTTCTGTGGCAAAAATAAGTCTTAGTGTTTGTATGTGTTGGTTTTATTCAGCTTTCTACAGCACTCTTTGCACAAAGAA
   Consensus CAGTTGATCGCTATGTGGCAATTTGTGATCCTCTGCATTACCCCAGTAGAATTTCTGTGGCAAAAATAAGTCTTAGTGTTTGTATGTGTTGGTTTTATTCAGCTTTCTACAGCACTCTTTGCACAAAGAA

           1561 1570      1580      1590      1600      1610      1620      1630      1640      1650      1660      1670      1680      1690
contig066285 TATCCTGATTGAACCAGGCAGGTATAATTCCTGTTATGGAGAGTGTGTGTTTATCACCAGTGATATTGCTGGCATAATTGACCTTGTTTTATCTTTTATCGTACCAGTTTCCATCATCATAGTTCTATAT
ZebTARs.A016 TATCCTGATTGAACCAGGCAGGTATAATTCCTGTTATGGAGAGTGTGTGTTTATCACCAGTGATATTGCTGGCATAATTGACCTTGTTTTATCTTTTATCGTACCAGTTTCCATCATCATAGTTCTATAT
   Consensus TATCCTGATTGAACCAGGCAGGTATAATTCCTGTTATGGAGAGTGTGTGTTTATCACCAGTGATATTGCTGGCATAATTGACCTTGTTTTATCTTTTATCGTACCAGTTTCCATCATCATAGTTCTATAT

           1691 1700      1710      1720      1730      1740      1750      1760      1770      1780      1790      1800      1810      1820
contig066285 ATGAGAGTATTTGTGGTGGCTGTGTCTCAGGCTCGTGCCATGCACTCTCATGTTACAGCTACACTTCAGCGTTCACTGAATCAAACAAACAAATCTGAGCTGAAAGCAGCCAGGACTCTGGGGGTTCTTG
ZebTARs.A016 ATGAGAGTATTTGTGGTGGCTGTGTCTCAGGCTCGTGCCATGCACTCTCATGTTACAGCTACACTTCAGCGTTCACTGAATCAAACAAACAAATCTGAGCTGAAAGCAGCCAGGACTCTGGGGGTTCTTG
   Consensus ATGAGAGTATTTGTGGTGGCTGTGTCTCAGGCTCGTGCCATGCACTCTCATGTTACAGCTACACTTCAGCGTTCACTGAATCAAACAAACAAATCTGAGCTGAAAGCAGCCAGGACTCTGGGGGTTCTTG

           1821 1830      1840      1850      1860      1870      1880      1890      1900      1910      1920      1930      1940      1950
contig066285 TAGTTGTGTTTCTGGCCAGTTTCTGTCCATTTTATTGTTACTTTCTTGTTGTAGAAGACAAAGTCAGTGATTCATCTGCCTCTATTGTGGTCATCGTCTATTATTTTAACTCTTGTCTAAACCCACTAAT
ZebTARs.A016 TAGTTGTGTTTCTGGCCAGTTTCTGTCCATTTTATTGTTACTTTCTTGTTGTAGAAGACAAAGTCAGTGATTCATCTGCCTCTATTGTGGTCATCGTCTATTATTTTAACTCTTGTCTAAACCCACTAAT
   Consensus TAGTTGTGTTTCTGGCCAGTTTCTGTCCATTTTATTGTTACTTTCTTGTTGTAGAAGACAAAGTCAGTGATTCATCTGCCTCTATTGTGGTCATCGTCTATTATTTTAACTCTTGTCTAAACCCACTAAT

           1951 1960      1970      1980      1990      2000      2010      2020      2030      2040      2050      2060      2070      2080
contig066285 TTATGCCCTGTTTTACCCTTGGTTTAGAAATGCTGTTAAAGTCATCATCACTTTTCAGATATTCAAGCATGACAGCAGTGAGGTCAACGTAATATAGAAATACAATGAAACCTGTTCCAGTAAGATGTTC
ZebTARs.A016 TTATGCCCTGTTTTACCCTTGGTTTAGAAATGCTGTTAAAGTCATCATCACTTTTCAGATATTCAAGCATGACAGCAGTGAGGTCAACGTAATATAG
   Consensus TTATGCCCTGTTTTACCCTTGGTTTAGAAATGCTGTTAAAGTCATCATCACTTTTCAGATATTCAAGCATGACAGCAGTGAGGTCAACGTAATATAG.................................
```

```
             1        10        20        30        40        50        60        70        80        90        100       110       120       130
contig066056 GTTGCGTCAGAAGGCAAATGTGGGAGGAAGGGTGGTGATACAAAAAAGGGGCTCAGTCACAAAGCTGTTTGTGCTGAACAGCTGTCTTTCTCTAACAATGGAAGAAACTGAACTCTGCTTTCCACAACTC
ZebTARs.A017                                                                                                  ATGGAAGAAACTGAACTCTGCTTTCCACAACTC
Consensus    .................................................................................................ATGGAAGAAACTGAACTCTGCTTTCCACAACTC

             131      140       150       160       170       180       190       200       210       220       230       240       250       260
contig066056 CTCAACTCCTCTTGTGTGCGGCAAAAGCGCCCACAAATTGAGGCTGTGTGTATTTACACCCTGCTATCATCTATCTCTCTGCTTACTGCAGTACTGAACCTACTTGTCATCATCTCCATCGCTCACTTCA
ZebTARs.A017 CTCAACTCCTCTTGTGTGCGGCAAAAGCGCCCACAAATTGAGGCTGTGTGTATTTACACCCTGCTATCATCTATCTCTCTGCTTACTGCAGTACTGAACCTACTTGTCATCATCTCCATCGCTCACTTCA
Consensus    CTCAACTCCTCTTGTGTGCGGCAAAAGCGCCCACAAATTGAGGCTGTGTGTATTTACACCCTGCTATCATCTATCTCTCTGCTTACTGCAGTACTGAACCTACTTGTCATCATCTCCATCGCTCACTTCA

             261      270       280       290       300       310       320       330       340       350       360       370       380       390
contig066056 AGTACTGACCTATTTAAATTAGAGACTTTTTTTTAGTGTTAAAAAATCATTTTTGCTTGTGAAGTTTCTTTTTATATGATGCTTTTGTCTTTTCCTCATAGTTCTAATGTTTTAACTGTTATACTAGCGA
ZebTARs.A017 AG--------------------------------------------------------------------------------------------------------------------------------
Consensus    AG................................................................................................................................

             391      400       410       420       430       440       450       460       470       480       490       500       510       520
contig066056 TGCTATTACCAATGTAATGCTTTATTTTAATCCAGGCAGCTGCACACCCCAACCAACCTCCTCCTCCTCTCTCTGGCCGTCTCAGATTTCTTCGTGGGCCTCATCATGGCTTTTGAGATTAGCCTCCTAG
ZebTARs.A017 ------------------------------------CAGCTGCACACCCCAACCAACCTCCTCCTCCTCTCTCTGGCCGTCTCAGATTTCTTCGTGGGCCTCATCATGGCTTTTGAGATTAGCCTCCTAG
Consensus    ....................................CAGCTGCACACCCCAACCAACCTCCTCCTCCTCTCTCTGGCCGTCTCAGATTTCTTCGTGGGCCTCATCATGGCTTTTGAGATTAGCCTCCTAG

             521      530       540       550       560       570       580       590       600       610       620       630       640       650
contig066056 ATGGCTGCTGGTTTCTTGGTGACCACATGTGTGCTCTGTATAGCAGTTTAGATTACATTGTTACTTCTGCTTCAGTAGGAACTATGGTACTCATATCAGCTGACCGCTATGTAGCCATTTGCGACCCTCT
ZebTARs.A017 ATGGCTGCTGGTTTCTTGGTGACCACATGTGTGCTCTGTATAGCAGTTTAGATTACATTGTTACTTCTGCTTCAGTAGGAACTATGGTACTCATATCAGCTGACCGCTATGTAGCCATTTGCGACCCTCT
Consensus    ATGGCTGCTGGTTTCTTGGTGACCACATGTGTGCTCTGTATAGCAGTTTAGATTACATTGTTACTTCTGCTTCAGTAGGAACTATGGTACTCATATCAGCTGACCGCTATGTAGCCATTTGCGACCCTCT

             651      660       670       680       690       700       710       720       730       740       750       760       770       780
contig066056 GCATTATCCCACCAAAATTACTATAAAAAGAGTCTCAGTCTCTATTTGTACTTGCTGGGCTTGTTCAATTCTGTATAACAGTCTTATCATGAAGGATAATTTCAAGCAGCCAGGGAGGTATAATTCTTGT
ZebTARs.A017 GCATTATCCCACCAAAATTACTATAAAAAGAGTCTCAGTCTCTATTTGTACTTGCTGGGCTTGTTCAATTCTGTATAACAGTCTTATCATGAAGGATAATTTCAAGCAGCCAGGGAGGTATAATTCTTGT
Consensus    GCATTATCCCACCAAAATTACTATAAAAAGAGTCTCAGTCTCTATTTGTACTTGCTGGGCTTGTTCAATTCTGTATAACAGTCTTATCATGAAGGATAATTTCAAGCAGCCAGGGAGGTATAATTCTTGT

             781      790       800       810       820       830       840       850       860       870       880       890       900       910
contig066056 TCTGGTGATTGTGTAGTTGTCATTGATTACTTTGTGGGAATTTTTGACTTTGTTTTGACCTTTGTCGGCCCTGTGATTGTCATCATAGTTCTGTATCTAAGAGTATTTGTGGTGGCTGTGTCTCAGGCTC
ZebTARs.A017 TCTGGTGATTGTGTAGTTGTCATTGATTACTTTGTGGGAATTTTTGACTTTGTTTTGACCTTTGTCGGCCCTGTGATTGTCATCATAGTTCTGTATCTAAGAGTATTTGTGGTGGCTGTGTCTCAGGCTC
Consensus    TCTGGTGATTGTGTAGTTGTCATTGATTACTTTGTGGGAATTTTTGACTTTGTTTTGACCTTTGTCGGCCCTGTGATTGTCATCATAGTTCTGTATCTAAGAGTATTTGTGGTGGCTGTGTCTCAGGCTC

             911      920       930       940       950       960       970       980       990       1000      1010      1020      1030      1040
contig066056 GGGCAATGCGCTCTCATATCACTGCTCTCAGACTCCAGGGGTCAGAGACGGTGCATGCTAAGAAATCTGAGCTGAAAGCTGCCAGGACTCTCGGTGTACTTGTTATTGCCTTTCTGATATGTCTTTTCCC
ZebTARs.A017 GGGCAATGCGCTCTCATATCACTGCTCTCAGACTCCAGGGGTCAGAGACGGTGCATGCTAAGAAATCTGAGCTGAAAGCTGCCAGGACTCTCGGTGTACTTGTTATTGCCTTTCTGATATGTCTTTTCCC
Consensus    GGGCAATGCGCTCTCATATCACTGCTCTCAGACTCCAGGGGTCAGAGACGGTGCATGCTAAGAAATCTGAGCTGAAAGCTGCCAGGACTCTCGGTGTACTTGTTATTGCCTTTCTGATATGTCTTTTCCC

             1041 1050      1060      1070      1080      1090      1100      1110      1120      1130      1140      1150      1160      1170
contig066056 ATTTTTCTGTTCCTCAATGGTAGGCCAGAATAGTTTCTTTGATATTAGATCTGTACCTTTTGAGCTCTTGCTGTTCTATTTTAACTCTTGTCTGAACCCATTAATCTACACTTTTTGCTACCCCTGGTTT
ZebTARs.A017 ATTTTTCTGTTCCTCAATGGTAGGCCAGAATAGTTTCTTTGATATTAGATCTGTACCTTTTGAGCTCTTGCTGTTCTATTTTAACTCTTGTCTGAACCCATTAATCTACACTTTTTGCTACCCCTGGTTT
Consensus    ATTTTTCTGTTCCTCAATGGTAGGCCAGAATAGTTTCTTTGATATTAGATCTGTACCTTTTGAGCTCTTGCTGTTCTATTTTAACTCTTGTCTGAACCCATTAATCTACACTTTTTGCTACCCCTGGTTT

             1171 1180      1190      1200      1210      1220      1230      1240      1250      1260      1270      1280      1290      1300
contig066056 CTGAAATCTATCAAGCTCATTGTGACATTTAAGATATTTAGGCATGGCTCCAGTGAGGCCAGTATACTATAGGGAAAAGTGATGTTATCACTGACACGGTGAAAAAAGATAAATTATGATTAATTTACAC
ZebTARs.A017 CTGAAATCTATCAAGCTCATTGTGACATTTAAGATATTTAGGCATGGCTCCAGTGAGGCCAGTATACTATAG
Consensus    CTGAAATCTATCAAGCTCATTGTGACATTTAAGATATTTAGGCATGGCTCCAGTGAGGCCAGTATACTATAG..........................................................

             1301 1310      1320      1330      1340      1350      1360      1370      1380      1390      1400      1410      1420      1430
contig066056 TAAGTACAATTCCAGAAGTCTGATAAAATGGATTTGTTTACATTGTTTCCATTATTAATGTACTAGTCCTCTGGTCCACAATGACAAAATTCTGTGTTTTGTCCATAATTTAAATGATTGTAAATCCATC
ZebTARs.A017
Consensus    ..................................................................................................................................

             1431 1440      1450      1460      1470      1480      1490      1500      1510      1520      1530      1540      1550      1560
contig066056 AAAATTAAATTGGAATGCACAAACAATTTCTGAGAATTGTACATGGTACTCTAATTTATCTGCAGCCTACAGATGAGACAAAAAATGTTGGTCTTAGTAAATAAATACATATATGAATAGTCATTCTTAT
ZebTARs.A017
Consensus    ..................................................................................................................................

             1561 1570      1582
contig066056 TCTACCAGTGAAATTCACACAT
ZebTARs.A017
Consensus    ......................
```

```
             261      270       280       290       300       310       320       330       340       350       360       370       380       390
             |--------+---------+---------+---------+---------+---------+---------+---------+---------+---------+---------+---------+---------|
contig062677 TGCGACCAGAGCTGCAGTTCTTCACTAGTCTGTGACGATGGGCACACAGGAAAAATCTGAGCTCTGCTTTCCACAACTCTTCAACATGTCCTGCAAAAAGCCTGCCATTGCTCGGTCCAAAGCTGTTTTC
ZebTARs.A018                                      ATGGGCACACAGGAAAAATCTGAGCTCTGCTTTCCACAACTCTTCAACATGTCCTGCAAAAAGCCTGCCATTGCTCGGTCCAAAGCTGTTTTC
   Consensus .....................................ATGGGCACACAGGAAAAATCTGAGCTCTGCTTTCCACAACTCTTCAACATGTCCTGCAAAAAGCCTGCCATTGCTCGGTCCAAAGCTGTTTTC

             391      400       410       420       430       440       450       460       470       480       490       500       510       520
             |--------+---------+---------+---------+---------+---------+---------+---------+---------+---------+---------+---------+---------|
contig062677 CTTCATATTGTATTGTCCTCAGTTTCTTTTCTGACAGTGGCTCTCAACTTGCTTGTCATCATCTCAGTCTCCCACTTCAGGCAGAAACAACGTTTTTGTTTTACTAGCTACACTATTCTAGTGTTTCAAG
ZebTARs.A018 CTTCATATTGTATTGTCCTCAGTTTCTTTTCTGACAGTGGCTCTCAACTTGCTTGTCATCATCTCAGTCTCCCACTTCAGGCAG----------------------------------------------
   Consensus CTTCATATTGTATTGTCCTCAGTTTCTTTTCTGACAGTGGCTCTCAACTTGCTTGTCATCATCTCAGTCTCCCACTTCAGGCAG..............................................

             521      530       540       550       560       570       580       590       600       610       620       630       640       650
             |--------+---------+---------+---------+---------+---------+---------+---------+---------+---------+---------+---------+---------|
contig062677 TATTTGAGCTAGTAATTAGTAATTAGCTAGTAATAGATTCTTCATATGTACAGATTTATACTTGAGAATATTTACATTTCATCATTTTTACTTTGGCACCAGAAAACATACTATACTCATTGTCTTATAA
ZebTARs.A018 ----------------------------------------------------------------------------------------------------------------------------------
   Consensus ..................................................................................................................................

             651      660       670       680       690       700       710       720       730       740       750       760       770       780
             |--------+---------+---------+---------+---------+---------+---------+---------+---------+---------+---------+---------+---------|
contig062677 GTAATACTATGTTCTGAATCAGTTTGACGTTGATAATATGCTGCTGTGTGACACTTAATAGCTCTTTGGCTTGTTTCATTATAGAGGTTTGAGGAATTGTTATTTTTCTGTCTCCTTGCAGGCAGCTTCA
ZebTARs.A018 -----------------------------------------------------------------------------------------------------------------------------CTTCA
   Consensus .............................................................................................................................CTTCA

             781      790       800       810       820       830       840       850       860       870       880       890       900       910
             |--------+---------+---------+---------+---------+---------+---------+---------+---------+---------+---------+---------+---------|
contig062677 CACACCCACTAACATCCTGCTCCTCTCTCTGGCTGTCTCAGACTTTCTTGTTGGCCTCCTATTGTCACCTGCAGAAATCCTCCGAAGTACAGCCTGTTGGTTTCTTGGTCAGCCCACATGTTTAATGTAT
ZebTARs.A018 CACACCCACTAACATCCTGCTCCTCTCTCTGGCTGTCTCAGACTTTCTTGTTGGCCTCCTATTGTCACCTGCAGAAATCCTCCGAAGTACAGCCTGTTGGTTTCTTGGTCAGCCCACATGTTTAATGTAT
   Consensus CACACCCACTAACATCCTGCTCCTCTCTCTGGCTGTCTCAGACTTTCTTGTTGGCCTCCTATTGTCACCTGCAGAAATCCTCCGAAGTACAGCCTGTTGGTTTCTTGGTCAGCCCACATGTTTAATGTAT

             911      920       930       940       950       960       970       980       990       1000      1010      1020      1030      1040
             |--------+---------+---------+---------+---------+---------+---------+---------+---------+---------+---------+---------+---------|
contig062677 ATTTTTGTGTCCTTGACTGTTACCTCTGCCTCAGTGGGCATTATGGTGCTGATATCAGCTGACCGCTATGTGGCTATTTGTGACCCTTTGAATTACCCCATCAGGATCACTGACAGAAGAGTTCAACTCT
ZebTARs.A018 ATTTTTGTGTCCTTGACTGTTACCTCTGCCTCAGTGGGCATTATGGTGCTGATATCAGCTGACCGCTATGTGGCTATTTGTGACCCTTTGAATTACCCCATCAGGATCACTGACAGAAGAGTTCAACTCT
   Consensus ATTTTTGTGTCCTTGACTGTTACCTCTGCCTCAGTGGGCATTATGGTGCTGATATCAGCTGACCGCTATGTGGCTATTTGTGACCCTTTGAATTACCCCATCAGGATCACTGACAGAAGAGTTCAACTCT

             1041 1050      1060      1070      1080      1090      1100      1110      1120      1130      1140      1150      1160      1170
             |--------+---------+---------+---------+---------+---------+---------+---------+---------+---------+---------+---------+---------|
contig062677 GTGTTTGTCTCTGTTGGCTCTGCTCCATCACCTTCAGCTGTTTCCTTGTAAGAGATGACCTGCATCAAAGAAGAAAACAAAATTCCTGCTATGGAAAATGTGTCGTTGTTGTTCAATACATTGCAGGAGT
ZebTARs.A018 GTGTTTGTCTCTGTTGGCTCTGCTCCATCACCTTCAGCTGTTTCCTTGTAAGAGATGACCTGCATCAAAGAAGAAAACAAAATTCCTGCTATGGAAAATGTGTCGTTGTTGTTCAATACATTGCAGGAGT
   Consensus GTGTTTGTCTCTGTTGGCTCTGCTCCATCACCTTCAGCTGTTTCCTTGTAAGAGATGACCTGCATCAAAGAAGAAAACAAAATTCCTGCTATGGAAAATGTGTCGTTGTTGTTCAATACATTGCAGGAGT

             1171 1180      1190      1200      1210      1220      1230      1240      1250      1260      1270      1280      1290      1300
             |--------+---------+---------+---------+---------+---------+---------+---------+---------+---------+---------+---------+---------|
contig062677 TGTTGACCTCATTTTAACCTTCATTGTACCAGTTACTGTCATCATAGTTCTGTATATGAGAGTATTTGTGGTGGCTGTGTCTCAGGCCCGCTCTATGCGCTCTCATGCTACAGCTGTCAGAAATCACCTT
ZebTARs.A018 TGTTGACCTCATTTTAACCTTCATTGTACCAGTTACTGTCATCATAGTTCTGTATATGAGAGTATTTGTGGTGGCTGTGTCTCAGGCCCGCTCTATGCGCTCTCATGCTACAGCTGTCAGAAATCACCTT
   Consensus TGTTGACCTCATTTTAACCTTCATTGTACCAGTTACTGTCATCATAGTTCTGTATATGAGAGTATTTGTGGTGGCTGTGTCTCAGGCCCGCTCTATGCGCTCTCATGCTACAGCTGTCAGAAATCACCTT

             1301 1310      1320      1330      1340      1350      1360      1370      1380      1390      1400      1410      1420      1430
             |--------+---------+---------+---------+---------+---------+---------+---------+---------+---------+---------+---------+---------|
contig062677 CCAATGACGCTAACAAAAAAGTCTGAACTAAAAGCAGCCAGGACTCTGGGTGTTCTTGTTCTTGTGTTTCTAATGTGTTTCTGCCCTTATTACTGTGTTTCTCTTGTAGGAGAAGAATTCATCAATAGTT
ZebTARs.A018 CCAATGACGCTAACAAAAAAGTCTGAACTAAAAGCAGCCAGGACTCTGGGTGTTCTTGTTCTTGTGTTTCTAATGTGTTTCTGCCCTTATTACTGTGTTTCTCTTGTAGGAGAAGAATTCATCAATAGTT
   Consensus CCAATGACGCTAACAAAAAAGTCTGAACTAAAAGCAGCCAGGACTCTGGGTGTTCTTGTTCTTGTGTTTCTAATGTGTTTCTGCCCTTATTACTGTGTTTCTCTTGTAGGAGAAGAATTCATCAATAGTT

             1431 1440      1450      1460      1470      1480      1490      1500      1510      1520      1530      1540      1550      1560
             |--------+---------+---------+---------+---------+---------+---------+---------+---------+---------+---------+---------+---------|
contig062677 CATCTGCTTCTTTTGTGGCCTATCTGTTTGGTTTAAACTCATGTCTAAACCCTTTGATCTATGCTATGTTCTACCCCTGGTTTAGAAAAGCTGTGAAACTAGTTGTCACTCTACAGATACTGCAGCCTGG
ZebTARs.A018 CATCTGCTTCTTTTGTGGCCTATCTGTTTGGTTTAAACTCATGTCTAAACCCTTTGATCTATGCTATGTTCTACCCCTGGTTTAGAAAAGCTGTGAAACTAGTTGTCACTCTACAGATACTGCAGCCTGG
   Consensus CATCTGCTTCTTTTGTGGCCTATCTGTTTGGTTTAAACTCATGTCTAAACCCTTTGATCTATGCTATGTTCTACCCCTGGTTTAGAAAAGCTGTGAAACTAGTTGTCACTCTACAGATACTGCAGCCTGG

             1561 1570      1580      1590      1600      1610      1620      1630      1640      1650      1660      1670      1680      1690
             |--------+---------+---------+---------+---------+---------+---------+---------+---------+---------+---------+---------+---------|
contig062677 CTCCAGTGAGGTAAGTATTTTGTAAACAGATCAAAACATTTAAACAGTTGTTTTCAGGTTCAGCTCGTAATAAAAAATCTTAATCTTGTTGACTTCATTTTATAACAATAAACTGTTTGCTTATTTTTGA
ZebTARs.A018 CTCCAGTGAGGTAAGTATTTTGTAA
   Consensus CTCCAGTGAGGTAAGTATTTTGTAA.........................................................................................................
```

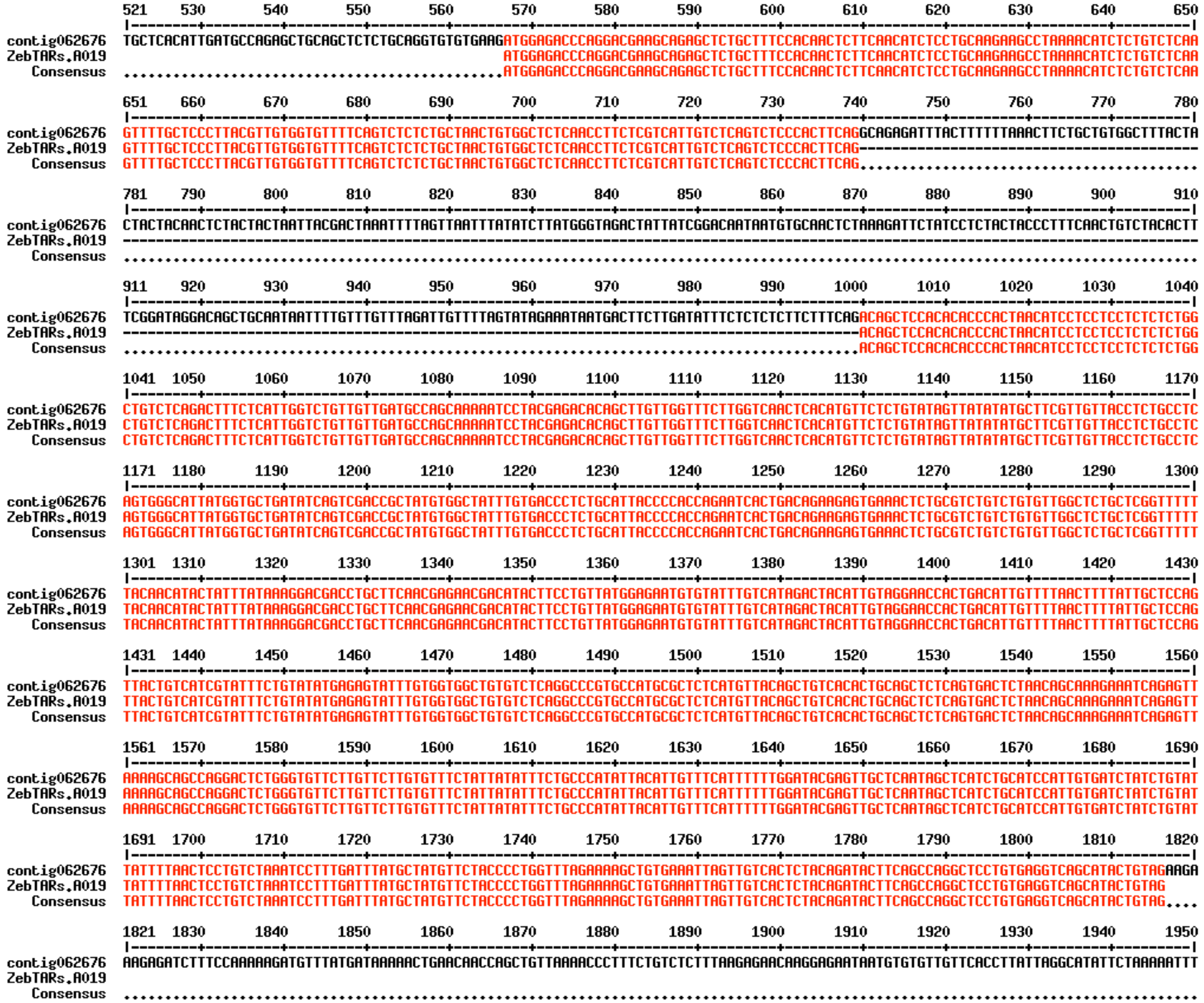
contig062676
ZebTARs.A019
Consensus

```
             391     400       410       420       430       440       450       460       470       480       490       500       510       520
             |--------+---------+---------+---------+---------+---------+---------+---------+---------+---------+---------+---------+---------|
contig061417 AGCGAATTCTACAGAGCATGAATGAGAGGAGGAGACAGATCAGACGCAGAGATAAATCTGCTGTATAATAATGAGATCAGGTCAGAGAGCTGCAGAGGTTGTAGTTGTCTGATGATGGAGATACAGAAAG
ZebTARs.A020                                                                                                                   ATGGAGATACAGAAAG
   Consensus .................................................................................................................ATGGAGATACAGAAAG

             521     530       540       550       560       570       580       590       600       610       620       630       640       650
             |--------+---------+---------+---------+---------+---------+---------+---------+---------+---------+---------+---------+---------|
contig061417 GAGCTGAGCTCTGTTTTCCACAACTCCTCAACAGCTCCTGCAGGAAGCCGACGCTTCGCTGGTCCAAAGCTGTGCTCCTGAACATTGTCCTGTCCTGCATCTCTCTGCTCACTGCTGCTCTAAACCTTCT
ZebTARs.A020 GAGCTGAGCTCTGTTTTCCACAACTCCTCAACAGCTCCTGCAGGAAGCCGACGCTTCGCTGGTCCAAAGCTGTGCTCCTGAACATTGTCCTGTCCTGCATCTCTCTGCTCACTGCTGCTCTAAACCTTCT
   Consensus GAGCTGAGCTCTGTTTTCCACAACTCCTCAACAGCTCCTGCAGGAAGCCGACGCTTCGCTGGTCCAAAGCTGTGCTCCTGAACATTGTCCTGTCCTGCATCTCTCTGCTCACTGCTGCTCTAAACCTTCT

             651     660       670       680       690       700       710       720       730       740       750       760       770       780
             |--------+---------+---------+---------+---------+---------+---------+---------+---------+---------+---------+---------+---------|
contig061417 CGTGATCATCTCAGTCTCCTACTTCAGGCACATATAATGTTTCTAAAACTTAAACTGTATCTTCATTTAAAAAGTTTAACACCATGATTTATTATCTAAATTGAAATTAATTTGGCACATTCTTGGTCAA
ZebTARs.A020 CGTGATCATCTCAGTCTCCTACTTCAGG------------------------------------------------------------------------------------------------------
   Consensus CGTGATCATCTCAGTCTCCTACTTCAGG......................................................................................................

             781     790       800       810       820       830       840       850       860       870       880       890       900       910
             |--------+---------+---------+---------+---------+---------+---------+---------+---------+---------+---------+---------+---------|
contig061417 TTTAAACCACAATATTTAATTTTTATACACCTTAATGTCTTTGTCTTACAGTCTTAATAATGGACCTTTTTACATTTTAAAATTACTAATATATTCTTTTTTGTTCTATTTCAGGAAGCTTCACACACCC
ZebTARs.A020 -------------------------------------------------------------------------------------------------------------------AAGCTTCACACACCC
   Consensus ...................................................................................................................AAGCTTCACACACCC

             911     920       930       940       950       960       970       980       990       1000      1010      1020      1030      1040
             |--------+---------+---------+---------+---------+---------+---------+---------+---------+---------+---------+---------+---------|
contig061417 AGCAACATCCTCCTGCTCTCTCTTGCTGTCTCAGACTTTCTCATGGGTCTCTTGCTGATGCCAGCAGAAATCCTTAGAAGCATGACGTGCTGGGTACTTGGTGATCTTATGTGTTCTGTTTATTTTTTTC
ZebTARs.A020 AGCAACATCCTCCTGCTCTCTCTTGCTGTCTCAGACTTTCTCATGGGTCTCTTGCTGATGCCAGCAGAAATCCTTAGAAGCATGACGTGCTGGGTACTTGGTGATCTTATGTGTTCTGTTTATTTTTTTC
   Consensus AGCAACATCCTCCTGCTCTCTCTTGCTGTCTCAGACTTTCTCATGGGTCTCTTGCTGATGCCAGCAGAAATCCTTAGAAGCATGACGTGCTGGGTACTTGGTGATCTTATGTGTTCTGTTTATTTTTTTC

             1041   1050      1060      1070      1080      1090      1100      1110      1120      1130      1140      1150      1160      1170
             |--------+---------+---------+---------+---------+---------+---------+---------+---------+---------+---------+---------+---------|
contig061417 TGACTGTAAACATTACCTGTGCTTCAATAGGGAACATAGTTCTTATATCGATTGACCGTTATGTGGCTATTTGTGACCCTCTGCATTACTCCACGAGAATTACTGTGGCAAGAGTCAAACTCAGTGTTTG
ZebTARs.A020 TGACTGTAAACATTACCTGTGCTTCAATAGGGAACATAGTTCTTATATCGATTGACCGTTATGTGGCTATTTGTGACCCTCTGCATTACTCCACGAGAATTACTGTGGCAAGAGTCAAACTCAGTGTTTG
   Consensus TGACTGTAAACATTACCTGTGCTTCAATAGGGAACATAGTTCTTATATCGATTGACCGTTATGTGGCTATTTGTGACCCTCTGCATTACTCCACGAGAATTACTGTGGCAAGAGTCAAACTCAGTGTTTG

             1171   1180      1190      1200      1210      1220      1230      1240      1250      1260      1270      1280      1290      1300
             |--------+---------+---------+---------+---------+---------+---------+---------+---------+---------+---------+---------+---------|
contig061417 TCTGTGTTGGTTTTATTCAACTTTCTACTGCAGTCTTTATACGCAAGAAATGCTGATTGAACCAGGCAGGTATAATTCCTGTTATGGTGAGTGTGTGCTTGTCATCAGTGATTTTGCTGGAATGGTTGAC
ZebTARs.A020 TCTGTGTTGGTTTTATTCAACTTTCTACTGCAGTCTTTATACGCAAGAAATGCTGATTGAACCAGGCAGGTATAATTCCTGTTATGGTGAGTGTGTGCTTGTCATCAGTGATTTTGCTGGAATGGTTGAC
   Consensus TCTGTGTTGGTTTTATTCAACTTTCTACTGCAGTCTTTATACGCAAGAAATGCTGATTGAACCAGGCAGGTATAATTCCTGTTATGGTGAGTGTGTGCTTGTCATCAGTGATTTTGCTGGAATGGTTGAC

             1301   1310      1320      1330      1340      1350      1360      1370      1380      1390      1400      1410      1420      1430
             |--------+---------+---------+---------+---------+---------+---------+---------+---------+---------+---------+---------+---------|
contig061417 CTTATTTTATTTTTTATTCTTCCACTTTCTGTCATCATAGTTCTGTATACAAGAGTATTTGTGGTGGCTGTGTCTCAGGCTCGTGCCATGCGCTCTCATGTTACGGCTGTCACACTTCAGCGTTCATTGA
ZebTARs.A020 CTTATTTTATTTTTTATTCTTCCACTTTCTGTCATCATAGTTCTGTATACAAGAGTATTTGTGGTGGCTGTGTCTCAGGCTCGTGCCATGCGCTCTCATGTTACGGCTGTCACACTTCAGCGTTCATTGA
   Consensus CTTATTTTATTTTTTATTCTTCCACTTTCTGTCATCATAGTTCTGTATACAAGAGTATTTGTGGTGGCTGTGTCTCAGGCTCGTGCCATGCGCTCTCATGTTACGGCTGTCACACTTCAGCGTTCATTGA

             1431   1440      1450      1460      1470      1480      1490      1500      1510      1520      1530      1540      1550      1560
             |--------+---------+---------+---------+---------+---------+---------+---------+---------+---------+---------+---------+---------|
contig061417 ATCAAACAAACAAATCTGAGCTGAAAGCAGCCAGGACTCTGGGGGTTCTTGTAGTTGTGTTTCTGTCATGCTACTGTCCATTTTATTGCTACTCTCTCACTGATAAAAATGCTGTCAATGATCCAGCTAC
ZebTARs.A020 ATCAAACAAACAAATCTGAGCTGAAAGCAGCCAGGACTCTGGGGGTTCTTGTAGTTGTGTTTCTGTCATGCTACTGTCCATTTTATTGCTACTCTCTCACTGATAAAAATGCTGTCAATGATCCAGCTAC
   Consensus ATCAAACAAACAAATCTGAGCTGAAAGCAGCCAGGACTCTGGGGGTTCTTGTAGTTGTGTTTCTGTCATGCTACTGTCCATTTTATTGCTACTCTCTCACTGATAAAAATGCTGTCAATGATCCAGCTAC

             1561   1570      1580      1590      1600      1610      1620      1630      1640      1650      1660      1670      1680      1690
             |--------+---------+---------+---------+---------+---------+---------+---------+---------+---------+---------+---------+---------|
contig061417 ATCTTCTGTGATCTTTATCTTTTATTTTAACTCTTGTATAAACCCTTTGATCTATGCCCTGTTTTACCCCTGGTTTAGAAATGCTGTTAAACTTATTATCACATTGCAGATATTCAAGCATAACTCCTGT
ZebTARs.A020 ATCTTCTGTGATCTTTATCTTTTATTTTAACTCTTGTATAAACCCTTTGATCTATGCCCTGTTTTACCCCTGGTTTAGAAATGCTGTTAAACTTATTATCACATTGCAGATATTCAAGCATAACTCCTGT
   Consensus ATCTTCTGTGATCTTTATCTTTTATTTTAACTCTTGTATAAACCCTTTGATCTATGCCCTGTTTTACCCCTGGTTTAGAAATGCTGTTAAACTTATTATCACATTGCAGATATTCAAGCATAACTCCTGT

             1691   1700      1710      1720      1730      1740      1750      1760      1770      1780      1790      1800      1810      1820
             |--------+---------+---------+---------+---------+---------+---------+---------+---------+---------+---------+---------+---------|
contig061417 GAGGCCAACATACTATAAAAAGACTATGAACCCTGCTCCAGTAAACTGCTTTAAATCAGGAAATGGGCCCATGTGGTAATCTACTGCATTAATTACACAGGGGGAAAAAAGCATTTTTGACCACTTCAAT
ZebTARs.A020 GAGGCCAACATACTATAA
   Consensus GAGGCCAACATACTATAA................................................................................................................
```

```
             1171  1180      1190      1200      1210      1220      1230      1240      1250      1260      1270      1280      1290      1300
             |--------+---------+---------+---------+---------+---------+---------+---------+---------+---------+---------+---------+---------|
contig061410 CACAGTGAGGGATGGAGCACATAGATATCCAACTGACATTGAGAAAAAGACGAGGGGCGGAGTGAGAAGGGGTGAGTTATATAACTAATTCAGTTTGTAATGCTACATAGAGCTACAGGTCTCTCTGAAA
ZebTARs.A021
   Consensus ..................................................................................................................................

             1301  1310      1320      1330      1340      1350      1360      1370      1380      1390      1400      1410      1420      1430
             |--------+---------+---------+---------+---------+---------+---------+---------+---------+---------+---------+---------+---------|
contig061410 ATGGATACACAGGATGTGGCAGAGCTCTGTTTTCCACAACTCTTCAACACCTCCTGCAAGAAACCTCCTCTGTCAGAATTTGTGTTCCTTCATGTTGTGTTGTCCTCCATCTCGCTGCTAACTGTGACTC
ZebTARs.A021 ATGGATACACAGGATGTGGCAGAGCTCTGTTTTCCACAACTCTTCAACACCTCCTGCAAGAAACCTCCTCTGTCAGAATTTGTGTTCCTTCATGTTGTGTTGTCCTCCATCTCGCTGCTAACTGTGACTC
   Consensus ATGGATACACAGGATGTGGCAGAGCTCTGTTTTCCACAACTCTTCAACACCTCCTGCAAGAAACCTCCTCTGTCAGAATTTGTGTTCCTTCATGTTGTGTTGTCCTCCATCTCGCTGCTAACTGTGACTC

             1431  1440      1450      1460      1470      1480      1490      1500      1510      1520      1530      1540      1550      1560
             |--------+---------+---------+---------+---------+---------+---------+---------+---------+---------+---------+---------+---------|
contig061410 TCAACCTGCTCATCATCATCTCAGTCTCCCACTACAGGCAGAGTTGTTGTTATTTTTTTAATAGCTGTGTGTAGCTTATACATTCTGTGAGCTAATTGACTTAAGATGTTTTTGTGACCTCAGTGGTGTA
ZebTARs.A021 TCAACCTGCTCATCATCATCTCAGTCTCCCACTACAGGCAG-----------------------------------------------------------------------------------------
   Consensus TCAACCTGCTCATCATCATCTCAGTCTCCCACTACAGGCAG.........................................................................................

             1561  1570      1580      1590      1600      1610      1620      1630      1640      1650      1660      1670      1680      1690
             |--------+---------+---------+---------+---------+---------+---------+---------+---------+---------+---------+---------+---------|
contig061410 TACAATTAAACTATCTGATATAGAAGTGGTCGACAAGAAATGATCAAAACTGTTTTTCTCTTACCAAGAGCTAATGTTTTATTGTTTGTTGGGTTGCTTTAATTTAGCGATTTGAGGACTGTTAATTTAA
ZebTARs.A021 ----------------------------------------------------------------------------------------------------------------------------------
   Consensus ..................................................................................................................................

             1691  1700      1710      1720      1730      1740      1750      1760      1770      1780      1790      1800      1810      1820
             |--------+---------+---------+---------+---------+---------+---------+---------+---------+---------+---------+---------+---------|
contig061410 TAATTTTTGTGTCTTCTTGCAGGCAGCTCCACACACCCACTAACATCCTCCTCCTCTCTCTGGCTGTCTCAGACTTTCTTGTTGGTCTCCTGTTGATGCCTGGAGAAATCCTCCGAAATACAGCCTGCTG
ZebTARs.A021 --------------------------CTCCACACACCCACTAACATCCTCCTCCTCTCTCTGGCTGTCTCAGACTTTCTTGTTGGTCTCCTGTTGATGCCTGGAGAAATCCTCCGAAATACAGCCTGCTG
   Consensus ..........................CTCCACACACCCACTAACATCCTCCTCCTCTCTCTGGCTGTCTCAGACTTTCTTGTTGGTCTCCTGTTGATGCCTGGAGAAATCCTCCGAAATACAGCCTGCTG

             1821  1830      1840      1850      1860      1870      1880      1890      1900      1910      1920      1930      1940      1950
             |--------+---------+---------+---------+---------+---------+---------+---------+---------+---------+---------+---------+---------|
contig061410 GTTTCTCGGTGACCTCACCTGTTCTATGTACAATTATATGTCTTTAATTGTTACCTCTACCTCAGTGGGAGACATGGTGTTAATATCAATTGACCGCTATGTGGCTATTTGTGACCCTCTGCATTACCCC
ZebTARs.A021 GTTTCTCGGTGACCTCACCTGTTCTATGTACAATTATATGTCTTTAATTGTTACCTCTACCTCAGTGGGAGACATGGTGTTAATATCAATTGACCGCTATGTGGCTATTTGTGACCCTCTGCATTACCCC
   Consensus GTTTCTCGGTGACCTCACCTGTTCTATGTACAATTATATGTCTTTAATTGTTACCTCTACCTCAGTGGGAGACATGGTGTTAATATCAATTGACCGCTATGTGGCTATTTGTGACCCTCTGCATTACCCC

             1951  1960      1970      1980      1990      2000      2010      2020      2030      2040      2050      2060      2070      2080
             |--------+---------+---------+---------+---------+---------+---------+---------+---------+---------+---------+---------+---------|
contig061410 ACCAGAATCACAGACAGAAGAGTGAAACTCTGTGTTTGTCTGTGTTGGCTCTGCTCTGTTTTCTATAGCAGCCTGTTTGTAAAGGATGATCTAACTCAACCAGGGAAGCATAATTCCTGCTATGGAGAAT
ZebTARs.A021 ACCAGAATCACAGACAGAAGAGTGAAACTCTGTGTTTGTCTGTGTTGGCTCTGCTCTGTTTTCTATAGCAGCCTGTTTGTAAAGGATGATCTAACTCAACCAGGGAAGCATAATTCCTGCTATGGAGAAT
   Consensus ACCAGAATCACAGACAGAAGAGTGAAACTCTGTGTTTGTCTGTGTTGGCTCTGCTCTGTTTTCTATAGCAGCCTGTTTGTAAAGGATGATCTAACTCAACCAGGGAAGCATAATTCCTGCTATGGAGAAT

             2081  2090      2100      2110      2120      2130      2140      2150      2160      2170      2180      2190      2200      2210
             |--------+---------+---------+---------+---------+---------+---------+---------+---------+---------+---------+---------+---------|
contig061410 GTACAATTGATGTTGACTTAATTACAGGAACGATTGACCTTCTTTTAACCTTTTTTGTTCCAGTTACTGTCATTGTAGTTCTGTATCTGAGAGTATTTGTGGTGGCTGTGTCTCAGGCTCGAGCCATGCG
ZebTARs.A021 GTACAATTGATGTTGACTTAATTACAGGAACGATTGACCTTCTTTTAACCTTTTTTGTTCCAGTTACTGTCATTGTAGTTCTGTATCTGAGAGTATTTGTGGTGGCTGTGTCTCAGGCTCGAGCCATGCG
   Consensus GTACAATTGATGTTGACTTAATTACAGGAACGATTGACCTTCTTTTAACCTTTTTTGTTCCAGTTACTGTCATTGTAGTTCTGTATCTGAGAGTATTTGTGGTGGCTGTGTCTCAGGCTCGAGCCATGCG

             2211  2220      2230      2240      2250      2260      2270      2280      2290      2300      2310      2320      2330      2340
             |--------+---------+---------+---------+---------+---------+---------+---------+---------+---------+---------+---------+---------|
contig061410 CTCTCATGTTACAGTTGCTGCTCTGCAGCTTTCAGTGACTCTAACAACAAAGAAATCAGAGTTAAAAGCAGCCAGGACTCTGGGTGTTCTTGTAGTTGTGTTTCTACTGTGTTTCTGCCCTTATTATTGT
ZebTARs.A021 CTCTCATGTTACAGTTGCTGCTCTGCAGCTTTCAGTGACTCTAACAACAAAGAAATCAGAGTTAAAAGCAGCCAGGACTCTGGGTGTTCTTGTAGTTGTGTTTCTACTGTGTTTCTGCCCTTATTATTGT
   Consensus CTCTCATGTTACAGTTGCTGCTCTGCAGCTTTCAGTGACTCTAACAACAAAGAAATCAGAGTTAAAAGCAGCCAGGACTCTGGGTGTTCTTGTAGTTGTGTTTCTACTGTGTTTCTGCCCTTATTATTGT

             2341  2350      2360      2370      2380      2390      2400      2410      2420      2430      2440      2450      2460      2470
             |--------+---------+---------+---------+---------+---------+---------+---------+---------+---------+---------+---------+---------|
contig061410 GTTACTCTTGCCAGGGACGACCTACTCAATAGTTCATCTGTATCCTTTTTGCTCTATCTGTTCTATTTTAACTCATGTCTAAACCCTTTGATCTATGCACTGCTCTACCCCTGGTTTAGAAAAGCTGTGA
ZebTARs.A021 GTTACTCTTGCCAGGGACGACCTACTCAATAGTTCATCTGTATCCTTTTTGCTCTATCTGTTCTATTTTAACTCATGTCTAAACCCTTTGATCTATGCACTGCTCTACCCCTGGTTTAGAAAAGCTGTGA
   Consensus GTTACTCTTGCCAGGGACGACCTACTCAATAGTTCATCTGTATCCTTTTTGCTCTATCTGTTCTATTTTAACTCATGTCTAAACCCTTTGATCTATGCACTGCTCTACCCCTGGTTTAGAAAAGCTGTGA

             2471  2480      2490      2500      2510      2520      2530      2540      2550      2560      2570      2580      2590      2600
             |--------+---------+---------+---------+---------+---------+---------+---------+---------+---------+---------+---------+---------|
contig061410 AACTCATAATCTCTTTACACATACTGCAGCCTGGCTCCTGTGAGATTAGCATTCTGTAAAAAAAACAAAACAAAAAAAAAAAAACAAATAAAAGGAACACTGAACAATTGTTGAGTTATTTTGTTGCCCTA
ZebTARs.A021 AACTCATAATCTCTTTACACATACTGCAGCCTGGCTCCTGTGAGATTAGCATTCTGTAA
   Consensus AACTCATAATCTCTTTACACATACTGCAGCCTGGCTCCTGTGAGATTAGCATTCTGTAA.......................................................................
```

```
               4291    4300      4310      4320      4330      4340      4350      4360      4370      4380      4390      4400      4410      4420
               |--------+---------+---------+---------+---------+---------+---------+---------+---------+---------+---------+---------+---------|
contig059768   TAGTCAGAAAGGTGCCAGTGTTCAGCAGCTTTCATCTTCTTTTCCCTTTCTGCTCATGGAGAACGTGTATGGACAACAATTCTGCTTTCCACAGCTCCTCAACACCTCCTGTAGGAAGCCGCTGCAACAT
ZebTARs.A022                                                          ATGGAGAACGTGTATGGACAACAATTCTGCTTTCCACAGCTCCTCAACACCTCCTGTAGGAAGCCGCTGCAACAT
   Consensus   .......................................................ATGGAGAACGTGTATGGACAACAATTCTGCTTTCCACAGCTCCTCAACACCTCCTGTAGGAAGCCGCTGCAACAT

               4421    4430      4440      4450      4460      4470      4480      4490      4500      4510      4520      4530      4540      4550
               |--------+---------+---------+---------+---------+---------+---------+---------+---------+---------+---------+---------+---------|
contig059768   CACAATCAGACCATTTTTCTCTACATTCTGCTTTGCTGCATCTCTCTGCTCACAGTGACTCTCAACGTCCTGGTCATCATCTCTATCTCCCACTTCAGGTGTTAAAGTAAACTTTACTGTGATATATTGT
ZebTARs.A022   CACAATCAGACCATTTTTCTCTACATTCTGCTTTGCTGCATCTCTCTGCTCACAGTGACTCTCAACGTCCTGGTCATCATCTCTATCTCCCACTTCAGG-------------------------------
   Consensus   CACAATCAGACCATTTTTCTCTACATTCTGCTTTGCTGCATCTCTCTGCTCACAGTGACTCTCAACGTCCTGGTCATCATCTCTATCTCCCACTTCAGG...............................

               4551    4560      4570      4580      4590      4600      4610      4620      4630      4640      4650      4660      4670      4680
               |--------+---------+---------+---------+---------+---------+---------+---------+---------+---------+---------+---------+---------|
contig059768   GTTTGACTTGAGCATACTTGGATTAGCTACAGAGTACTTCACATTTACTAAACTGTGATAGCTCTCTTTTACAAAGGAACAGCTGATGTGATAAATGTTTGTACTATTTAACTGAAATGATTTTATATTC
ZebTARs.A022   ----------------------------------------------------------------------------------------------------------------------------------
   Consensus   ..................................................................................................................................

               4681    4690      4700      4710      4720      4730      4740      4750      4760      4770      4780      4790      4800      4810
               |--------+---------+---------+---------+---------+---------+---------+---------+---------+---------+---------+---------+---------|
contig059768   TGACCTCCTCCTCTCAGAATGATGTGGAAACGAGAGGTTTGCCCTACTTTGTTGCACTGGATTGTTTATTGTATGTTGTTTTACTTTCAGAACTGGTGCATTACTGTAGCCTTCCACCTGCTGCAGGTTT
ZebTARs.A022   ----------------------------------------------------------------------------------------------------------------------------------
   Consensus   ..................................................................................................................................

               4811    4820      4830      4840      4850      4860      4870      4880      4890      4900      4910      4920      4930      4940
               |--------+---------+---------+---------+---------+---------+---------+---------+---------+---------+---------+---------+---------|
contig059768   ATTAATATTATGTTACGTCTTTCTTTCTGTTTTTTTCTTCTCAGGCAGCTTCACAACCAAACCAACCTCTTCCTCCTCTCTCTCGCTGTCTCAGACCTCCTTGTTGGCCTCCTGCTGATGCCGCCTCGAA
ZebTARs.A022   ---------------------------------------------CAGCTTCACAACCAAACCAACCTCTTCCTCCTCTCTCTCGCTGTCTCAGACCTCCTTGTTGGCCTCCTGCTGATGCCGCCTCGAA
   Consensus   .............................................CAGCTTCACAACCAAACCAACCTCTTCCTCCTCTCTCTCGCTGTCTCAGACCTCCTTGTTGGCCTCCTGCTGATGCCGCCTCGAA

               4941    4950      4960      4970      4980      4990      5000      5010      5020      5030      5040      5050      5060      5070
               |--------+---------+---------+---------+---------+---------+---------+---------+---------+---------+---------+---------+---------|
contig059768   TCCTCCTTTTAGGGGGCTGCTGGTTTTTGGGCACCTTCATGTGTGGACTGTTTTACTATGCCTCTTTTGTTCTCACATCTGCCTCAGTAGGAAACATGGTGCTTATTTCATTTGACCGGTATGTAGCTAT
ZebTARs.A022   TCCTCCTTTTAGGGGGCTGCTGGTTTTTGGGCACCTTCATGTGTGGACTGTTTTACTATGCCTCTTTTGTTCTCACATCTGCCTCAGTAGGAAACATGGTGCTTATTTCATTTGACCGGTATGTAGCTAT
   Consensus   TCCTCCTTTTAGGGGGCTGCTGGTTTTTGGGCACCTTCATGTGTGGACTGTTTTACTATGCCTCTTTTGTTCTCACATCTGCCTCAGTAGGAAACATGGTGCTTATTTCATTTGACCGGTATGTAGCTAT

               5071    5080      5090      5100      5110      5120      5130      5140      5150      5160      5170      5180      5190      5200
               |--------+---------+---------+---------+---------+---------+---------+---------+---------+---------+---------+---------+---------|
contig059768   TTGTGACCCCTTGTCCTACCCCACCACAGTTACTGAGAGAAAAGTCCAAATCAGTGTCTGTCTGTGTTGGGCCTGTTCACTGCTGTATAACGGCACAATACTAAACAACTTTCTGAAGCAGCCAGACAGA
ZebTARs.A022   TTGTGACCCCTTGTCCTACCCCACCACAGTTACTGAGAGAAAAGTCCAAATCAGTGTCTGTCTGTGTTGGGCCTGTTCACTGCTGTATAACGGCACAATACTAAACAACTTTCTGAAGCAGCCAGACAGA
   Consensus   TTGTGACCCCTTGTCCTACCCCACCACAGTTACTGAGAGAAAAGTCCAAATCAGTGTCTGTCTGTGTTGGGCCTGTTCACTGCTGTATAACGGCACAATACTAAACAACTTTCTGAAGCAGCCAGACAGA

               5201    5210      5220      5230      5240      5250      5260      5270      5280      5290      5300      5310      5320      5330
               |--------+---------+---------+---------+---------+---------+---------+---------+---------+---------+---------+---------+---------|
contig059768   TATAATTCTTGTGACGGGGAATGCATAGTTGTAATTAATTTCATAACTGGTGCTTTTGATGTCGTAGTTACCTTCATCGGCCCCACTGCTGTGATCATATTTCTGTACATGAGAGTATTTCTGGTGGCTG
ZebTARs.A022   TATAATTCTTGTGACGGGGAATGCATAGTTGTAATTAATTTCATAACTGGTGCTTTTGATGTCGTAGTTACCTTCATCGGCCCCACTGCTGTGATCATATTTCTGTACATGAGAGTATTTCTGGTGGCTG
   Consensus   TATAATTCTTGTGACGGGGAATGCATAGTTGTAATTAATTTCATAACTGGTGCTTTTGATGTCGTAGTTACCTTCATCGGCCCCACTGCTGTGATCATATTTCTGTACATGAGAGTATTTCTGGTGGCTG

               5331    5340      5350      5360      5370      5380      5390      5400      5410      5420      5430      5440      5450      5460
               |--------+---------+---------+---------+---------+---------+---------+---------+---------+---------+---------+---------+---------|
contig059768   TGTCTCAGGCTCAGGCTATGAGATCTCATGTTGCCTTTGTTACTTCAAAGGGTTCAGTTCACGTTGCCATTAAGAAGTCTGAGAGAAAAGCAGCTACAACTATTGGTGTTGTTGTGGCTGTGTTTCTGAT
ZebTARs.A022   TGTCTCAGGCTCAGGCTATGAGATCTCATGTTGCCTTTGTTACTTCAAAGGGTTCAGTTCACGTTGCCATTAAGAAGTCTGAGAGAAAAGCAGCTACAACTATTGGTGTTGTTGTGGCTGTGTTTCTGAT
   Consensus   TGTCTCAGGCTCAGGCTATGAGATCTCATGTTGCCTTTGTTACTTCAAAGGGTTCAGTTCACGTTGCCATTAAGAAGTCTGAGAGAAAAGCAGCTACAACTATTGGTGTTGTTGTGGCTGTGTTTCTGAT

               5461    5470      5480      5490      5500      5510      5520      5530      5540      5550      5560      5570      5580      5590
               |--------+---------+---------+---------+---------+---------+---------+---------+---------+---------+---------+---------+---------|
contig059768   GTGTTTCTGTCCTTACTTTTATCCCTCTCTCGCAGGGCAGGACACATCAACCAGTGTGGAATTTTCAGTTTTTGGGGTCTGGTTACTTTATTGTAATTCCTGTCTAAATCCACTTATCTATGCGTTTTTC
ZebTARs.A022   GTGTTTCTGTCCTTACTTTTATCCCTCTCTCGCAGGGCAGGACACATCAACCAGTGTGGAATTTTCAGTTTTTGGGGTCTGGTTACTTTATTGTAATTCCTGTCTAAATCCACTTATCTATGCGTTTTTC
   Consensus   GTGTTTCTGTCCTTACTTTTATCCCTCTCTCGCAGGGCAGGACACATCAACCAGTGTGGAATTTTCAGTTTTTGGGGTCTGGTTACTTTATTGTAATTCCTGTCTAAATCCACTTATCTATGCGTTTTTC

               5591    5600      5610      5620      5630      5640      5650      5660      5670      5680      5690      5700      5710      5720
               |--------+---------+---------+---------+---------+---------+---------+---------+---------+---------+---------+---------+---------|
contig059768   TATCCCTGGTTTAGGAAGACTGTTAAACTGATAGTTACATTGCAGATTTTGCAGCCTGACTCTTGTGATGCCAACATACTGTAGAATTAAAATCCTATCACTCCTCAGTTAGTCAAATACACTGTAAAGG
ZebTARs.A022   TATCCCTGGTTTAGGAAGACTGTTAAACTGATAGTTACATTGCAGATTTTGCAGCCTGACTCTTGTGATGCCAACATACTGTAG
   Consensus   TATCCCTGGTTTAGGAAGACTGTTAAACTGATAGTTACATTGCAGATTTTGCAGCCTGACTCTTGTGATGCCAACATACTGTAG..............................................
```

```
               651    660       670       680       690       700       710       720       730       740       750       760       770       780
               |--------+---------+---------+---------+---------+---------+---------+---------+---------+---------+---------+---------+---------|
contig053139   TGGCATTTAATAAAAAGGGGAGGACAAAGATATATGCTGTATGACACAGAATATGATTAGCAGCTCGCCTCATTGTGATGATGGAGGAACCTGAACTCTGCTTTCCAAAACTCCTTAATATCTCCTGCAT
ZebTARs.A023                                                                                   ATGGAGGAACCTGAACTCTGCTTTCCAAAACTCCTTAATATCTCCTGCAT
   Consensus   ................................................................................ATGGAGGAACCTGAACTCTGCTTTCCAAAACTCCTTAATATCTCCTGCAT

               781    790       800       810       820       830       840       850       860       870       880       890       900       910
               |--------+---------+---------+---------+---------+---------+---------+---------+---------+---------+---------+---------+---------|
contig053139   CAGGCCAAAGCGTCCTCACTTTGAGATCATGCTGACTTACATTTTGCTCTCCTTCATTTCTTTGCTTACTGTGATTCTTAACCTGCTGGTCATTATCTCCATCTCACACTTCAGGTATGAAAATGTTTCA
ZebTARs.A023   CAGGCCAAAGCGTCCTCACTTTGAGATCATGCTGACTTACATTTTGCTCTCCTTCATTTCTTTGCTTACTGTGATTCTTAACCTGCTGGTCATTATCTCCATCTCACACTTCAGG---------------
   Consensus   CAGGCCAAAGCGTCCTCACTTTGAGATCATGCTGACTTACATTTTGCTCTCCTTCATTTCTTTGCTTACTGTGATTCTTAACCTGCTGGTCATTATCTCCATCTCACACTTCAGG...............

               911    920       930       940       950       960       970       980       990      1000      1010      1020      1030      1040
               |--------+---------+---------+---------+---------+---------+---------+---------+---------+---------+---------+---------+---------|
contig053139   TTATCTCCAAATATTGAATAATTATTTTAATAGTTTAGTTAATTGTGCTGTGTAGCAACATAGCAGTAATAGTAATTAAAGCCATAATATGATCTGTACCTACAAGTAAGAGTAGACCTTTTTTTAATGA
ZebTARs.A023   ----------------------------------------------------------------------------------------------------------------------------------
   Consensus   ..................................................................................................................................

               1041  1050      1060      1070      1080      1090      1100      1110      1120      1130      1140      1150      1160      1170
               |--------+---------+---------+---------+---------+---------+---------+---------+---------+---------+---------+---------+---------|
contig053139   AGTGCATTTCTATTTCAGTTTTCCTTGTATGTGCATCCACATGCACTTTTGATGCAGGAAACTAAATATGATGCTCCTTTTTCTCTCCAGGCAGCTCCACACCCCCACCAACTTCCTCCTTCTCTCTCTG
ZebTARs.A023   -------------------------------------------------------------------------------------------CAGCTCCACACCCCCACCAACTTCCTCCTTCTCTCTCTG
   Consensus   ...........................................................................................CAGCTCCACACCCCCACCAACTTCCTCCTTCTCTCTCTG

               1171  1180      1190      1200      1210      1220      1230      1240      1250      1260      1270      1280      1290      1300
               |--------+---------+---------+---------+---------+---------+---------+---------+---------+---------+---------+---------+---------|
contig053139   GCTGTCGCTGATTTCTTTGTAGGTCTCCTTATGTTCTTTCAAATAGTGCTCATCGATGGATGCTGGTTCCTCGGTGACATCATGTGCACTCTGTATCAGTATCTAGCATTCATTATTACTTCAGCCTCAG
ZebTARs.A023   GCTGTCGCTGATTTCTTTGTAGGTCTCCTTATGTTCTTTCAAATAGTGCTCATCGATGGATGCTGGTTCCTCGGTGACATCATGTGCACTCTGTATCAGTATCTAGCATTCATTATTACTTCAGCCTCAG
   Consensus   GCTGTCGCTGATTTCTTTGTAGGTCTCCTTATGTTCTTTCAAATAGTGCTCATCGATGGATGCTGGTTCCTCGGTGACATCATGTGCACTCTGTATCAGTATCTAGCATTCATTATTACTTCAGCCTCAG

               1301  1310      1320      1330      1340      1350      1360      1370      1380      1390      1400      1410      1420      1430
               |--------+---------+---------+---------+---------+---------+---------+---------+---------+---------+---------+---------+---------|
contig053139   TAGGAACCATGGTGATCATATCTGCTGATAGGTATTTGGCTATTTGTTACCCTCTGCATTACTCCACCCAAATCACACAACAAAGAGTTAATATATGTATAAGTTTGTGTTGGTTTTTTTCTGTGATCTT
ZebTARs.A023   TAGGAACCATGGTGATCATATCTGCTGATAGGTATTTGGCTATTTGTTACCCTCTGCATTACTCCACCCAAATCACACAACAAAGAGTTAATATATGTATAAGTTTGTGTTGGTTTTTTTCTGTGATCTT
   Consensus   TAGGAACCATGGTGATCATATCTGCTGATAGGTATTTGGCTATTTGTTACCCTCTGCATTACTCCACCCAAATCACACAACAAAGAGTTAATATATGTATAAGTTTGTGTTGGTTTTTTTCTGTGATCTT

               1431  1440      1450      1460      1470      1480      1490      1500      1510      1520      1530      1540      1550      1560
               |--------+---------+---------+---------+---------+---------+---------+---------+---------+---------+---------+---------+---------|
contig053139   TCAGAGTTTGATTGTGAAGGATAACTTGAAACAACCAGGAAAGTATAACTCCTGCATTGGAGAGTGTGTCTTTGTCGTTAACTACATTGCTGGGCTTTTTGATCTTTTGTTTTCCTTCATTGTTCCCATT
ZebTARs.A023   TCAGAGTTTGATTGTGAAGGATAACTTGAAACAACCAGGAAAGTATAACTCCTGCATTGGAGAGTGTGTCTTTGTCGTTAACTACATTGCTGGGCTTTTTGATCTTTTGTTTTCCTTCATTGTTCCCATT
   Consensus   TCAGAGTTTGATTGTGAAGGATAACTTGAAACAACCAGGAAAGTATAACTCCTGCATTGGAGAGTGTGTCTTTGTCGTTAACTACATTGCTGGGCTTTTTGATCTTTTGTTTTCCTTCATTGTTCCCATT

               1561  1570      1580      1590      1600      1610      1620      1630      1640      1650      1660      1670      1680      1690
               |--------+---------+---------+---------+---------+---------+---------+---------+---------+---------+---------+---------+---------|
contig053139   ACTGTGATTGTAGTTCTGTATCTGAGAGTGTTTGTGGTGGCTGTGTCTCAGGCTCGTGCCATGAGGTGTCAACTTGCAGTCACTCACCAGCGATCAGTAACAGTAACTGTTACGAAATCGGAGCTGAAAG
ZebTARs.A023   ACTGTGATTGTAGTTCTGTATCTGAGAGTGTTTGTGGTGGCTGTGTCTCAGGCTCGTGCCATGAGGTGTCAACTTGCAGTCACTCACCAGCGATCAGTAACAGTAACTGTTACGAAATCGGAGCTGAAAG
   Consensus   ACTGTGATTGTAGTTCTGTATCTGAGAGTGTTTGTGGTGGCTGTGTCTCAGGCTCGTGCCATGAGGTGTCAACTTGCAGTCACTCACCAGCGATCAGTAACAGTAACTGTTACGAAATCGGAGCTGAAAG

               1691  1700      1710      1720      1730      1740      1750      1760      1770      1780      1790      1800      1810      1820
               |--------+---------+---------+---------+---------+---------+---------+---------+---------+---------+---------+---------+---------|
contig053139   CAGCCCGTACTCTTGGTGTTGTTGTAGTTGTGTTTCTTATATGTATGTGCCCATATTACTGCGTTGCTCTCACAGGCCAAGATAACTTCCTAAATGCTTCATCTGCTGCCTTTGTAATTTGTTTGGTGTA
ZebTARs.A023   CAGCCCGTACTCTTGGTGTTGTTGTAGTTGTGTTTCTTATATGTATGTGCCCATATTACTGCGTTGCTCTCACAGGCCAAGATAACTTCCTAAATGCTTCATCTGCTGCCTTTGTAATTTGTTTGGTGTA
   Consensus   CAGCCCGTACTCTTGGTGTTGTTGTAGTTGTGTTTCTTATATGTATGTGCCCATATTACTGCGTTGCTCTCACAGGCCAAGATAACTTCCTAAATGCTTCATCTGCTGCCTTTGTAATTTGTTTGGTGTA

               1821  1830      1840      1850      1860      1870      1880      1890      1900      1910      1920      1930      1940      1950
               |--------+---------+---------+---------+---------+---------+---------+---------+---------+---------+---------+---------+---------|
contig053139   CTTTAACTCTTGCCTAAACCCTATCATTTATGTCTTTTTTTATCCCTGGTTCAGAAAGTCAATCAAACTTATTGCTACTCTTCAAATACTGCAGCCTGACTCCCGTGAGACTAACATGCATTAAGAACAC
ZebTARs.A023   CTTTAACTCTTGCCTAAACCCTATCATTTATGTCTTTTTTTATCCCTGGTTCAGAAAGTCAATCAAACTTATTGCTACTCTTCAAATACTGCAGCCTGACTCCCGTGAGACTAACATGCATTAA
   Consensus   CTTTAACTCTTGCCTAAACCCTATCATTTATGTCTTTTTTTATCCCTGGTTCAGAAAGTCAATCAAACTTATTGCTACTCTTCAAATACTGCAGCCTGACTCCCGTGAGACTAACATGCATTAA......

               1951  1960      1970      1980      1990      2000      2010      2020      2030      2040      2050      2060      2070      2080
               |--------+---------+---------+---------+---------+---------+---------+---------+---------+---------+---------+---------+---------|
contig053139   TTTTTAAAACCGTACCCATAAACAGGACAATGTACTTAGTCACAATGAAGACTTGAAAGTTCAGATAAAGCAATATGTTAGCAAACATATTTATATATGTATCTGCTTGCACATGAGTACTTGCAATTAC
ZebTARs.A023
   Consensus   ..................................................................................................................................
```

```
             3511  3520      3530      3540      3550      3560      3570      3580      3590      3600      3610      3620      3630      3640
             |--------+---------+---------+---------+---------+---------+---------+---------+---------+---------+---------+---------+---------|
contig030471 GTGTGTCGTGTGTGTATGTCTCTGGGTGGTGGTGTAATAAATCAGGTCTGTGAGAAAACATTCTGATCAGTGAGCTGCAGCTGTCTGATGATGGAGATTCAGAAAGCAGCTGAGCTTTGTTTTCCACAAC
ZebTARs.A024                                                                                           ATGGAGATTCAGAAAGCAGCTGAGCTTTGTTTTCCACAAC
   Consensus ..........................................................................................ATGGAGATTCAGAAAGCAGCTGAGCTTTGTTTTCCACAAC

             3641  3650      3660      3670      3680      3690      3700      3710      3720      3730      3740      3750      3760      3770
             |--------+---------+---------+---------+---------+---------+---------+---------+---------+---------+---------+---------+---------|
contig030471 TCCTCAACAGTTCCTGCAGGAAGCCGACACTTCATTGGTCCAAAGCTGTGCTCCTGAACATTGTGCTGTCCTGCATCTCTCTGATCACTGCTGCTCTAAACCTCCTGGTCATCATCTCAGTCTCCCACTT
ZebTARs.A024 TCCTCAACAGTTCCTGCAGGAAGCCGACACTTCATTGGTCCAAAGCTGTGCTCCTGAACATTGTGCTGTCCTGCATCTCTCTGATCACTGCTGCTCTAAACCTCCTGGTCATCATCTCAGTCTCCCACTT
   Consensus TCCTCAACAGTTCCTGCAGGAAGCCGACACTTCATTGGTCCAAAGCTGTGCTCCTGAACATTGTGCTGTCCTGCATCTCTCTGATCACTGCTGCTCTAAACCTCCTGGTCATCATCTCAGTCTCCCACTT

             3771  3780      3790      3800      3810      3820      3830      3840      3850      3860      3870      3880      3890      3900
             |--------+---------+---------+---------+---------+---------+---------+---------+---------+---------+---------+---------+---------|
contig030471 CAGGCAGAGATACATTTCTCTGCTGAAACTATACTTTAAAATTTAGAAACTTAAATTTCAGAAAATTATGATTTATGGCATTTGATTTTATGATTAAGATTGAGATGATTGAAGTGGTGTATTTTAACCA
ZebTARs.A024 CAGG------------------------------------------------------------------------------------------------------------------------------
   Consensus CAGG..............................................................................................................................

             3901  3910      3920      3930      3940      3950      3960      3970      3980      3990      4000      4010      4020      4030
             |--------+---------+---------+---------+---------+---------+---------+---------+---------+---------+---------+---------+---------|
contig030471 GCTATTAAGTTTTATATACCTAGACTGAATCTTACTACATACTTTAACTAATGAAAATCTAATTTAAGCCATTTAAAAAAATATTAATGTCCATTATATTCTTTTCTGTTTCCCTGCAGGAAGCTTCATA
ZebTARs.A024 ------------------------------------------------------------------------------------------------------------------------AAGCTTCATA
   Consensus ........................................................................................................................AAGCTTCATA

             4031  4040      4050      4060      4070      4080      4090      4100      4110      4120      4130      4140      4150      4160
             |--------+---------+---------+---------+---------+---------+---------+---------+---------+---------+---------+---------+---------|
contig030471 CACCCAGTAACATCCTGCTCCTCTCTCTGGCCGTCTCAGACTTTCTTGTGGGTCTTCTGCTGATGCCATTAGAGATCTTTAGAAACACAGCTTGCTGGGTACTTGGTGATCGTATGTGTTCTGCTTATTG
ZebTARs.A024 CACCCAGTAACATCCTGCTCCTCTCTCTGGCCGTCTCAGACTTTCTTGTGGGTCTTCTGCTGATGCCATTAGAGATCTTTAGAAACACAGCTTGCTGGGTACTTGGTGATCGTATGTGTTCTGCTTATTG
   Consensus CACCCAGTAACATCCTGCTCCTCTCTCTGGCCGTCTCAGACTTTCTTGTGGGTCTTCTGCTGATGCCATTAGAGATCTTTAGAAACACAGCTTGCTGGGTACTTGGTGATCGTATGTGTTCTGCTTATTG

             4161  4170      4180      4190      4200      4210      4220      4230      4240      4250      4260      4270      4280      4290
             |--------+---------+---------+---------+---------+---------+---------+---------+---------+---------+---------+---------+---------|
contig030471 GTATTTGACCATCAACATTATCTGTGCTTCAATAGGGAACATTGTTCTAATATCAGTTGACCGCTATGTGGCTATTTGTGACCCTCTGCATTATCCCAGCAGAATTACTTTGGCGAAAGTCAAACTCAGT
ZebTARs.A024 GTATTTGACCATCAACATTATCTGTGCTTCAATAGGGAACATTGTTCTAATATCAGTTGACCGCTATGTGGCTATTTGTGACCCTCTGCATTATCCCAGCAGAATTACTTTGGCGAAAGTCAAACTCAGT
   Consensus GTATTTGACCATCAACATTATCTGTGCTTCAATAGGGAACATTGTTCTAATATCAGTTGACCGCTATGTGGCTATTTGTGACCCTCTGCATTATCCCAGCAGAATTACTTTGGCGAAAGTCAAACTCAGT

             4291  4300      4310      4320      4330      4340      4350      4360      4370      4380      4390      4400      4410      4420
             |--------+---------+---------+---------+---------+---------+---------+---------+---------+---------+---------+---------+---------|
contig030471 GTTTGTCTCTGTTGGTTTTATGCTTTTTTCTACAGCAATCTTTATACAAAGAATATCATGATTGAACCAGGCAGGTATAATTCTTGCTTTGGAGAGTGTGTATTTTTTAGCAGCAATATTGCTATTGTTG
ZebTARs.A024 GTTTGTCTCTGTTGGTTTTATGCTTTTTTCTACAGCAATCTTTATACAAAGAATATCATGATTGAACCAGGCAGGTATAATTCTTGCTTTGGAGAGTGTGTATTTTTTAGCAGCAATATTGCTATTGTTG
   Consensus GTTTGTCTCTGTTGGTTTTATGCTTTTTTCTACAGCAATCTTTATACAAAGAATATCATGATTGAACCAGGCAGGTATAATTCTTGCTTTGGAGAGTGTGTATTTTTTAGCAGCAATATTGCTATTGTTG

             4421  4430      4440      4450      4460      4470      4480      4490      4500      4510      4520      4530      4540      4550
             |--------+---------+---------+---------+---------+---------+---------+---------+---------+---------+---------+---------+---------|
contig030471 CTGACCTTATTTTATTCTTTTTTGTTCCAGTAACTGTTATCATAGCCTTGTATATGAGAGTATTTGTGGTGGCTGTGTCTCAGGCTCGTGCCATGCGCTCTCATGTTACATCTGTCACACTTCAGTGTTC
ZebTARs.A024 CTGACCTTATTTTATTCTTTTTTGTTCCAGTAACTGTTATCATAGCCTTGTATATGAGAGTATTTGTGGTGGCTGTGTCTCAGGCTCGTGCCATGCGCTCTCATGTTACATCTGTCACACTTCAGTGTTC
   Consensus CTGACCTTATTTTATTCTTTTTTGTTCCAGTAACTGTTATCATAGCCTTGTATATGAGAGTATTTGTGGTGGCTGTGTCTCAGGCTCGTGCCATGCGCTCTCATGTTACATCTGTCACACTTCAGTGTTC

             4551  4560      4570      4580      4590      4600      4610      4620      4630      4640      4650      4660      4670      4680
             |--------+---------+---------+---------+---------+---------+---------+---------+---------+---------+---------+---------+---------|
contig030471 ACTGAATCAAGCAAACAAATCTGAGCTGAAAGCAGCCAGGACTCTGGGGGTTCTTGTAGTTGTATTTTTGGCATGCTACTGTCCATTTTACTGCTACGCTCTGGTTGATAAAAATGTGGTCAATGATTCA
ZebTARs.A024 ACTGAATCAAGCAAACAAATCTGAGCTGAAAGCAGCCAGGACTCTGGGGGTTCTTGTAGTTGTATTTTTGGCATGCTACTGTCCATTTTACTGCTACGCTCTGGTTGATAAAAATGTGGTCAATGATTCA
   Consensus ACTGAATCAAGCAAACAAATCTGAGCTGAAAGCAGCCAGGACTCTGGGGGTTCTTGTAGTTGTATTTTTGGCATGCTACTGTCCATTTTACTGCTACGCTCTGGTTGATAAAAATGTGGTCAATGATTCA

             4681  4690      4700      4710      4720      4730      4740      4750      4760      4770      4780      4790      4800      4810
             |--------+---------+---------+---------+---------+---------+---------+---------+---------+---------+---------+---------+---------|
contig030471 TCTGCATCTTTTGTGGTTCTTGTCTTTTATTTTAACTCTTGTTTAAACCCACTGATCTATGCTCTGTTTTACCCCTGGTTTAGAAAAGCTATTAGATGTGTTATCACTCTGCTGATATTTAAACATGACA
ZebTARs.A024 TCTGCATCTTTTGTGGTTCTTGTCTTTTATTTTAACTCTTGTTTAAACCCACTGATCTATGCTCTGTTTTACCCCTGGTTTAGAAAAGCTATTAGATGTGTTATCACTCTGCTGATATTTAAACATGACA
   Consensus TCTGCATCTTTTGTGGTTCTTGTCTTTTATTTTAACTCTTGTTTAAACCCACTGATCTATGCTCTGTTTTACCCCTGGTTTAGAAAAGCTATTAGATGTGTTATCACTCTGCTGATATTTAAACATGACA

             4811  4820      4830      4840      4850      4860      4870      4880      4890      4900      4910      4920      4930      4940
             |--------+---------+---------+---------+---------+---------+---------+---------+---------+---------+---------+---------+---------|
contig030471 GCAGTGAGGTCAACATAATATAGAAATACAATGAAAGCTGCTCCACTAAGATTTTAAACCAGTAAATTGATACAGTTCAGTCTGGAAATGCATCAAAAACAAATTGATAAGGAAATTCATAATTCTTAAG
ZebTARs.A024 GCAGTGAGGTCAACATAATATAG
   Consensus GCAGTGAGGTCAACATAATATAG...........................................................................................................
```

```
             1821 1830      1840      1850      1860      1870      1880      1890      1900      1910      1920      1930      1940      1950
contig030464 GATCAGAGAGCTGCAGCTGTATGATTTTGGAGATGCAGAAAGAAGCCGAGCTTTGTTTTCCACAACTCCTCAACAGCTCCTGCAGGAAGCCAACATTTCACTGGTCCAAAGCTGTGCTGCTGAACACTGT
ZebTARs.A025                                 ATGCAGAAAGAAGCCGAGCTTTGTTTTCCACAACTCCTCAACAGCTCCTGCAGGAAGCCAACATTTCACTGGTCCAAAGCTGTGCTGCTGAACACTGT
   Consensus ................................ATGCAGAAAGAAGCCGAGCTTTGTTTTCCACAACTCCTCAACAGCTCCTGCAGGAAGCCAACATTTCACTGGTCCAAAGCTGTGCTGCTGAACACTGT

             1951 1960      1970      1980      1990      2000      2010      2020      2030      2040      2050      2060      2070      2080
contig030464 GCTGTGCTGCATCTCTCTGCTCACTGCTGCTCTAAACCTTCTCGTCATCATCTCAGTCTCTTACTTCAGGCAGAAATTAAGTTCTCAGCTGAAATTGTAGTTTAAGTCTTAAAAACCAATGGAATTAGTG
ZebTARs.A025 GCTGTGCTGCATCTCTCTGCTCACTGCTGCTCTAAACCTTCTCGTCATCATCTCAGTCTCTTACTTCAGGCAG---------------------------------------------------------
   Consensus GCTGTGCTGCATCTCTCTGCTCACTGCTGCTCTAAACCTTCTCGTCATCATCTCAGTCTCTTACTTCAGGCAG.........................................................

             2081 2090      2100      2110      2120      2130      2140      2150      2160      2170      2180      2190      2200      2210
contig030464 AAGACATTTTTACATTATTAATGTGTAATATATTCTTTTTTTCTCTCTCCTTAGGCAGCTTCACACACCCACTAACATCCTCCTCCTTTCTCTGGCTGTCTCAGACTTTCTCACTGGTCTCCTTATGCTG
ZebTARs.A025 ----------------------------------------------------------CTTCACACACCCACTAACATCCTCCTCCTTTCTCTGGCTGTCTCAGACTTTCTCACTGGTCTCCTTATGCTG
   Consensus ..........................................................CTTCACACACCCACTAACATCCTCCTCCTTTCTCTGGCTGTCTCAGACTTTCTCACTGGTCTCCTTATGCTG

             2211 2220      2230      2240      2250      2260      2270      2280      2290      2300      2310      2320      2330      2340
contig030464 CCACTAGAAATAATTAGAAACACGGCCTGCTGGGTACTTGGTGATCTTATGTGTTCTGTTTATACTTATCTGACAGCCGGTCTTTTCTGTGCTTCAACTTGGCACATAGTTCTCATTTCAGTTGACCGCT
ZebTARs.A025 CCACTAGAAATAATTAGAAACACGGCCTGCTGGGTACTTGGTGATCTTATGTGTTCTGTTTATACTTATCTGACAGCCGGTCTTTTCTGTGCTTCAACTTGGCACATAGTTCTCATTTCAGTTGACCGCT
   Consensus CCACTAGAAATAATTAGAAACACGGCCTGCTGGGTACTTGGTGATCTTATGTGTTCTGTTTATACTTATCTGACAGCCGGTCTTTTCTGTGCTTCAACTTGGCACATAGTTCTCATTTCAGTTGACCGCT

             2341 2350      2360      2370      2380      2390      2400      2410      2420      2430      2440      2450      2460      2470
contig030464 ATGTAGCTATTTGTCACCCTCTTCATTACCCCACCAGAATTACCGTGGCAAGAGTCAAACTCTGTGTTTGTTTGTGTTGGTTTTGTTCAACCTTCTACGGCAGTCTTTTAACAAAGGATGTTCTAATTAA
ZebTARs.A025 ATGTAGCTATTTGTCACCCTCTTCATTACCCCACCAGAATTACCGTGGCAAGAGTCAAACTCTGTGTTTGTTTGTGTTGGTTTTGTTCAACCTTCTACGGCAGTCTTTTAACAAAGGATGTTCTAATTAA
   Consensus ATGTAGCTATTTGTCACCCTCTTCATTACCCCACCAGAATTACCGTGGCAAGAGTCAAACTCTGTGTTTGTTTGTGTTGGTTTTGTTCAACCTTCTACGGCAGTCTTTTAACAAAGGATGTTCTAATTAA

             2471 2480      2490      2500      2510      2520      2530      2540      2550      2560      2570      2580      2590      2600
contig030464 GCCGGCCAGGTATAATTCCTGTTATGGAGAGTGTGTGTTTGTCATTAATGATATTGCAGGGTTTATTGATCTTGTGTTATCTTTTATTTTTCCAGTATCTGTTATCATAGTTCTTTATATGAGAGTGTTT
ZebTARs.A025 GCCGGCCAGGTATAATTCCTGTTATGGAGAGTGTGTGTTTGTCATTAATGATATTGCAGGGTTTATTGATCTTGTGTTATCTTTTATTTTTCCAGTATCTGTTATCATAGTTCTTTATATGAGAGTGTTT
   Consensus GCCGGCCAGGTATAATTCCTGTTATGGAGAGTGTGTGTTTGTCATTAATGATATTGCAGGGTTTATTGATCTTGTGTTATCTTTTATTTTTCCAGTATCTGTTATCATAGTTCTTTATATGAGAGTGTTT

             2601 2610      2620      2630      2640      2650      2660      2670      2680      2690      2700      2710      2720      2730
contig030464 GTGGTGGCTGTGTCTCAAGCTCGTTCCATGCGCTCTCATGTTACAGCTGTCACACTTCAGCGTTCAAGCAATCAAGCAAACAAATCTGAACTGAAAGCAGCCAGGAACCTTGGAGTTCTTGTAGTTGTGT
ZebTARs.A025 GTGGTGGCTGTGTCTCAAGCTCGTTCCATGCGCTCTCATGTTACAGCTGTCACACTTCAGCGTTCAAGCAATCAAGCAAACAAATCTGAACTGAAAGCAGCCAGGAACCTTGGAGTTCTTGTAGTTGTGT
   Consensus GTGGTGGCTGTGTCTCAAGCTCGTTCCATGCGCTCTCATGTTACAGCTGTCACACTTCAGCGTTCAAGCAATCAAGCAAACAAATCTGAACTGAAAGCAGCCAGGAACCTTGGAGTTCTTGTAGTTGTGT

             2731 2740      2750      2760      2770      2780      2790      2800      2810      2820      2830      2840      2850      2860
contig030464 TTCTCGCATGTTACCTTCCGTTTTACTGTTACTCTCTTATTGAAGTAAATGTGATCAATGACTCATCTACATTTTTTCTGATCATTGTGTTTTACTTTAACTCGTGTCTAAACCCACTGATGTATGCCCT
ZebTARs.A025 TTCTCGCATGTTACCTTCCGTTTTACTGTTACTCTCTTATTGAAGTAAATGTGATCAATGACTCATCTACATTTTTTCTGATCATTGTGTTTTACTTTAACTCGTGTCTAAACCCACTGATGTATGCCCT
   Consensus TTCTCGCATGTTACCTTCCGTTTTACTGTTACTCTCTTATTGAAGTAAATGTGATCAATGACTCATCTACATTTTTTCTGATCATTGTGTTTTACTTTAACTCGTGTCTAAACCCACTGATGTATGCCCT

             2861 2870      2880      2890      2900      2910      2920      2930      2940      2950      2960      2970      2980      2990
contig030464 TTTTTACTCTTGGTTTAGAAATGCTGTTAAACTTATTATCACTCTGCAGATTTTTAAGCATGACACTAGTGAAGCAAACATGTTTTAAAAAGATAAGGAAATCCACTCTACTAACAATTGAAATCAATAA
ZebTARs.A025 TTTTTACTCTTGGTTTAGAAATGCTGTTAAACTTATTATCACTCTGCAGATTTTTAAGCATGACACTAGTGAAGCAAACATGTTTTAA
   Consensus TTTTTACTCTTGGTTTAGAAATGCTGTTAAACTTATTATCACTCTGCAGATTTTTAAGCATGACACTAGTGAAGCAAACATGTTTTAA..........................................
```

```
             6501 6510      6520      6530      6540      6550      6560      6570      6580      6590      6600      6610      6620      6630
             |--------+---------+---------+---------+---------+---------+---------+---------+---------+---------+---------+---------+---------|
contig030445 CAGAGCTGGAGCTCTCTGCAGGTCTGTGGAGATGGATACTCAGGATGGAGCAGAGCTCTGCTTTCCACAACTCTTCAACATCTCCTGCAAGAAACCTACAACTCCTCTGTCTCAAGTTTTGCTCCTTTAT
ZebTARs.A026                                ATGGATACTCAGGATGGAGCAGAGCTCTGCTTTCCACAACTCTTCAACATCTCCTGCAAGAAACCTACAACTCCTCTGTCTCAAGTTTTGCTCCTTTAT
   Consensus ...............................ATGGATACTCAGGATGGAGCAGAGCTCTGCTTTCCACAACTCTTCAACATCTCCTGCAAGAAACCTACAACTCCTCTGTCTCAAGTTTTGCTCCTTTAT

             6631 6640      6650      6660      6670      6680      6690      6700      6710      6720      6730      6740      6750      6760
             |--------+---------+---------+---------+---------+---------+---------+---------+---------+---------+---------+---------+---------|
contig030445 ATTGTTGTGTCCTCAATGTCTCTGCTGACTGTGACTCTCAACCTGCTCGTCATCATTGCAGTCTCCCACTTCAGGCAGAGTTTCATATTTTTTACCTGCTGCTGTGGCTTTACAAGTGTATACCTGATTT
ZebTARs.A026 ATTGTTGTGTCCTCAATGTCTCTGCTGACTGTGACTCTCAACCTGCTCGTCATCATTGCAGTCTCCCACTTCAGGCAG----------------------------------------------------
   Consensus ATTGTTGTGTCCTCAATGTCTCTGCTGACTGTGACTCTCAACCTGCTCGTCATCATTGCAGTCTCCCACTTCAGGCAG....................................................

             6761 6770      6780      6790      6800      6810      6820      6830      6840      6850      6860      6870      6880      6890
             |--------+---------+---------+---------+---------+---------+---------+---------+---------+---------+---------+---------+---------|
contig030445 GTTATATCTGTTTGAATACTTTCTTTCTGTTGACTTTTTTTTAAAATTATATTTTCCTATCTTTCTAGGCAGCTCCACACACCCACTAACATCCTGCTCCTTTCTCTGGCTGTCACTGACTTTCTCGTTG
ZebTARs.A026 ------------------------------------------------------------------------CTCCACACACCCACTAACATCCTGCTCCTTTCTCTGGCTGTCACTGACTTTCTCGTTG
   Consensus ........................................................................CTCCACACACCCACTAACATCCTGCTCCTTTCTCTGGCTGTCACTGACTTTCTCGTTG

             6891 6900      6910      6920      6930      6940      6950      6960      6970      6980      6990      7000      7010      7020
             |--------+---------+---------+---------+---------+---------+---------+---------+---------+---------+---------+---------+---------|
contig030445 GTCTTCTGTTTATGCCTGGAGAAATCCTGCGAAATACAGCGTGTTGGTTTCTTGGTCAGCTCACATGTTCTCTGTATAATTATGTATCCTACATCATTACCTCTGCCTCAGTGGGCAACATGGTGCTGAT
ZebTARs.A026 GTCTTCTGTTTATGCCTGGAGAAATCCTGCGAAATACAGCGTGTTGGTTTCTTGGTCAGCTCACATGTTCTCTGTATAATTATGTATCCTACATCATTACCTCTGCCTCAGTGGGCAACATGGTGCTGAT
   Consensus GTCTTCTGTTTATGCCTGGAGAAATCCTGCGAAATACAGCGTGTTGGTTTCTTGGTCAGCTCACATGTTCTCTGTATAATTATGTATCCTACATCATTACCTCTGCCTCAGTGGGCAACATGGTGCTGAT

             7021 7030      7040      7050      7060      7070      7080      7090      7100      7110      7120      7130      7140      7150
             |--------+---------+---------+---------+---------+---------+---------+---------+---------+---------+---------+---------+---------|
contig030445 ATCAGTCGACCGCTATGTGGCTATTTGTGACCCTCTGCATTACCCCACCAGAATCACAGAGAGAAGAGTGAAACTTTGTGTTTGTCTGTGTTGGCTCTGCTCTGTTTTCTACTGTTATGTGATTTTAATA
ZebTARs.A026 ATCAGTCGACCGCTATGTGGCTATTTGTGACCCTCTGCATTACCCCACCAGAATCACAGAGAGAAGAGTGAAACTTTGTGTTTGTCTGTGTTGGCTCTGCTCTGTTTTCTACTGTTATGTGATTTTAATA
   Consensus ATCAGTCGACCGCTATGTGGCTATTTGTGACCCTCTGCATTACCCCACCAGAATCACAGAGAGAAGAGTGAAACTTTGTGTTTGTCTGTGTTGGCTCTGCTCTGTTTTCTACTGTTATGTGATTTTAATA

             7151 7160      7170      7180      7190      7200      7210      7220      7230      7240      7250      7260      7270      7280
             |--------+---------+---------+---------+---------+---------+---------+---------+---------+---------+---------+---------+---------|
contig030445 GATGATCTAAGTCAACCAGGCAAGCATAATTCTTGCTATGGAAGATGTATAATTATCATTGAATTTATTGCAGGATTTGTTGACCTTTTTTTATCCTTTATTATTCCACTTACTGTCATCATAGTTCTGT
ZebTARs.A026 GATGATCTAAGTCAACCAGGCAAGCATAATTCTTGCTATGGAAGATGTATAATTATCATTGAATTTATTGCAGGATTTGTTGACCTTTTTTTATCCTTTATTATTCCACTTACTGTCATCATAGTTCTGT
   Consensus GATGATCTAAGTCAACCAGGCAAGCATAATTCTTGCTATGGAAGATGTATAATTATCATTGAATTTATTGCAGGATTTGTTGACCTTTTTTTATCCTTTATTATTCCACTTACTGTCATCATAGTTCTGT

             7281 7290      7300      7310      7320      7330      7340      7350      7360      7370      7380      7390      7400      7410
             |--------+---------+---------+---------+---------+---------+---------+---------+---------+---------+---------+---------+---------|
contig030445 ACATGAGAGTGTTTGTGGTGGCTGTGTCTCAGGCCCGTGCCATGCGCTCTCAGGTTACAGCTGTCACACTGCAGCTCTCAGTGACTCTAACAGCAAAGAAATCAGAGTTAAAAGCAGCCAGGACTCTGGG
ZebTARs.A026 ACATGAGAGTGTTTGTGGTGGCTGTGTCTCAGGCCCGTGCCATGCGCTCTCAGGTTACAGCTGTCACACTGCAGCTCTCAGTGACTCTAACAGCAAAGAAATCAGAGTTAAAAGCAGCCAGGACTCTGGG
   Consensus ACATGAGAGTGTTTGTGGTGGCTGTGTCTCAGGCCCGTGCCATGCGCTCTCAGGTTACAGCTGTCACACTGCAGCTCTCAGTGACTCTAACAGCAAAGAAATCAGAGTTAAAAGCAGCCAGGACTCTGGG

             7411 7420      7430      7440      7450      7460      7470      7480      7490      7500      7510      7520      7530      7540
             |--------+---------+---------+---------+---------+---------+---------+---------+---------+---------+---------+---------+---------|
contig030445 TGTTCTTGTTCTTGTGTTTCTATTATGTTTCTGTTCATATTACTTTATTTCACTTTTTGGAAACGATTTGCTCAATAGCTCATCTGCATCGATTGTGATCTATCTGTACTATTTTAACTCCTGTCTAAAC
ZebTARs.A026 TGTTCTTGTTCTTGTGTTTCTATTATGTTTCTGTTCATATTACTTTATTTCACTTTTTGGAAACGATTTGCTCAATAGCTCATCTGCATCGATTGTGATCTATCTGTACTATTTTAACTCCTGTCTAAAC
   Consensus TGTTCTTGTTCTTGTGTTTCTATTATGTTTCTGTTCATATTACTTTATTTCACTTTTTGGAAACGATTTGCTCAATAGCTCATCTGCATCGATTGTGATCTATCTGTACTATTTTAACTCCTGTCTAAAC

             7541 7550      7560      7570      7580      7590      7600      7610      7620      7630      7640      7650      7660      7670
             |--------+---------+---------+---------+---------+---------+---------+---------+---------+---------+---------+---------+---------|
contig030445 CCTTTGATTTATGCTATGTTCTACCCCTGGTTTAGAAAAGCTGTGAAACTAATTGTGACTCTACAGATACTGCAGCCTGGCTCCTGTGAGGTCAGCGTACTGTAGAAAAAAGAAATCTTTCCTTAATGTA
ZebTARs.A026 CCTTTGATTTATGCTATGTTCTACCCCTGGTTTAGAAAAGCTGTGAAACTAATTGTGACTCTACAGATACTGCAGCCTGGCTCCTGTGAGGTCAGCGTACTGTAG
   Consensus CCTTTGATTTATGCTATGTTCTACCCCTGGTTTAGAAAAGCTGTGAAACTAATTGTGACTCTACAGATACTGCAGCCTGGCTCCTGTGAGGTCAGCGTACTGTAG.........................

             7671 7680      7690      7700      7710      7720      7730      7740      7750      7760      7770      7780      7790      7800
             |--------+---------+---------+---------+---------+---------+---------+---------+---------+---------+---------+---------+---------|
contig030445 CCAAATGGTATATCAAATTACTTTCTCTATCAATAATGTACTATATTAATTTTACGACATTTAACAAGTAATATGCCATACATTACATTTCTTGAGTAATAACCCCAACACTGGTTGTCAAATAGTGCTG
ZebTARs.A026
   Consensus ..................................................................................................................................

             7801 7810      7820      7830      7840      7850      7860      7870      7880      7890      7900      7910      7920      7930
             |--------+---------+---------+---------+---------+---------+---------+---------+---------+---------+---------+---------+---------|
contig030445 GTTTCAATTATAAAGGCCTAGAAAAGGGTTAACAACCCACTGGCAACCTCCTGTCACTAGCAACAAAATGTGCATTCTGATACTGTTTGCCGAGTGGTTGCTGGCTATCACTGGGTCAGACTGGTTGAAA
ZebTARs.A026
   Consensus ..................................................................................................................................

             7931 7940      7950      7960      7970      7980      7990      8000      8010      8020      8030      8040      8050      8060
             |--------+---------+---------+---------+---------+---------+---------+---------+---------+---------+---------+---------+---------|
contig030445 ACAGTGTCACAAAAAACCTCGACATGATTGTTTTAATCACCAAAATGTTGCTGTGGCTACTCAGAGGGAAGATTCCAGGTTTTCAAGTTTGCCTTGTTACCAGGCAGCTGCAAACCAGTCTTTAGTGTGA
ZebTARs.A026
   Consensus ..................................................................................................................................

             8061 8070      8080 8085
             |--------+---------+----|
contig030445 TGACTCCTGTGTGGAGTGTATCTTG
ZebTARs.A026
   Consensus .........................
```

```
             2601  2610      2620      2630      2640      2650      2660      2670      2680      2690      2700      2710      2720      2730
             |--------+---------+---------+---------+---------+---------+---------+---------+---------+---------+---------+---------+---------|
contig066330 GAGGTGTGGAAGAAGGAGGGGAGGATGAATATAAAGACGGCAGACACATTTTAGTTCAGTTTGCCGTTCTTCCAGCAGTGATGGAGGAAGCTGAACTCTGCTTTCAACAACTCTTTAACACCTCCTGCAT
ZebTARs.A027                                                                                 ATGGAGGAAGCTGAACTCTGCTTTCAACAACTCTTTAACACCTCCTGCAT
   Consensus ................................................................................ATGGAGGAAGCTGAACTCTGCTTTCAACAACTCTTTAACACCTCCTGCAT

             2731  2740      2750      2760      2770      2780      2790      2800      2810      2820      2830      2840      2850      2860
             |--------+---------+---------+---------+---------+---------+---------+---------+---------+---------+---------+---------+---------|
contig066330 GAGGCCCAGGCGTCCACACTTTGAGATCATGCTGACTTATATTTTGCTGTCCTTCATTTCTCTGCTTACTGTGATTCTTAACCTGTTGGTCATCATCTCTGTCTCACATTTCAGGTAGAAAGATATCAAA
ZebTARs.A027 GAGGCCCAGGCGTCCACACTTTGAGATCATGCTGACTTATATTTTGCTGTCCTTCATTTCTCTGCTTACTGTGATTCTTAACCTGTTGGTCATCATCTCTGTCTCACATTTCAGG---------------
   Consensus GAGGCCCAGGCGTCCACACTTTGAGATCATGCTGACTTATATTTTGCTGTCCTTCATTTCTCTGCTTACTGTGATTCTTAACCTGTTGGTCATCATCTCTGTCTCACATTTCAGG...............

             2861  2870      2880      2890      2900      2910      2920      2930      2940      2950      2960      2970      2980      2990
             |--------+---------+---------+---------+---------+---------+---------+---------+---------+---------+---------+---------+---------|
contig066330 CTGAATTGATCTCAAGCAGTAAGAAAGACATTTCTTTTCACAGATAGTATTTCTTTTCTTTTTAAGCCTCTGCACTATATACTTAAATTTGAATAGCACTTGATAGAGCAAACTAAATATAAAATATTGT
ZebTARs.A027 ----------------------------------------------------------------------------------------------------------------------------------
   Consensus ..................................................................................................................................

             2991  3000      3010      3020      3030      3040      3050      3060      3070      3080      3090      3100      3110      3120
             |--------+---------+---------+---------+---------+---------+---------+---------+---------+---------+---------+---------+---------|
contig066330 GTTTTCTCTCCCTATTCAGGCAGCTCCACACCCCCACCAACCTCCTGCTCCTTTCTCTGGCTGTCGCTGATTTCTATGTGGGCCTCCTCTTGTTCTTCCAAATTGTGCTCATAGATGGCTGCTGGTTCCT
ZebTARs.A027 --------------------CAGCTCCACACCCCCACCAACCTCCTGCTCCTTTCTCTGGCTGTCGCTGATTTCTATGTGGGCCTCCTCTTGTTCTTCCAAATTGTGCTCATAGATGGCTGCTGGTTCCT
   Consensus ....................CAGCTCCACACCCCCACCAACCTCCTGCTCCTTTCTCTGGCTGTCGCTGATTTCTATGTGGGCCTCCTCTTGTTCTTCCAAATTGTGCTCATAGATGGCTGCTGGTTCCT

             3121  3130      3140      3150      3160      3170      3180      3190      3200      3210      3220      3230      3240      3250
             |--------+---------+---------+---------+---------+---------+---------+---------+---------+---------+---------+---------+---------|
contig066330 CGGTGACATCATGTGCACTCTGTATCAATACCTAGCATATGTCATCACCTCGGCCTCCATAGGAACCATGGTGATCATATCTGTTGATCGATATTTGGCTATTTGTTACCCTCTGCATTACTCCACCAAG
ZebTARs.A027 CGGTGACATCATGTGCACTCTGTATCAATACCTAGCATATGTCATCACCTCGGCCTCCATAGGAACCATGGTGATCATATCTGTTGATCGATATTTGGCTATTTGTTACCCTCTGCATTACTCCACCAAG
   Consensus CGGTGACATCATGTGCACTCTGTATCAATACCTAGCATATGTCATCACCTCGGCCTCCATAGGAACCATGGTGATCATATCTGTTGATCGATATTTGGCTATTTGTTACCCTCTGCATTACTCCACCAAG

             3251  3260      3270      3280      3290      3300      3310      3320      3330      3340      3350      3360      3370      3380
             |--------+---------+---------+---------+---------+---------+---------+---------+---------+---------+---------+---------+---------|
contig066330 ATCACACAACAAAGAGTTAAAATTGTTGTCTGTTTGTGTTGGATCTGTTCTGTCATCTTTCAAAGTCTGATTCTGATGGATAACCTGGAGCAACCAGGCAGGTATAACTCTTGCATTGGAGAGTGTGTCT
ZebTARs.A027 ATCACACAACAAAGAGTTAAAATTGTTGTCTGTTTGTGTTGGATCTGTTCTGTCATCTTTCAAAGTCTGATTCTGATGGATAACCTGGAGCAACCAGGCAGGTATAACTCTTGCATTGGAGAGTGTGTCT
   Consensus ATCACACAACAAAGAGTTAAAATTGTTGTCTGTTTGTGTTGGATCTGTTCTGTCATCTTTCAAAGTCTGATTCTGATGGATAACCTGGAGCAACCAGGCAGGTATAACTCTTGCATTGGAGAGTGTGTCT

             3381  3390      3400      3410      3420      3430      3440      3450      3460      3470      3480      3490      3500      3510
             |--------+---------+---------+---------+---------+---------+---------+---------+---------+---------+---------+---------+---------|
contig066330 TTGTCATTAATTACATCGCAGGACTTGTTGATGTTACTTTTTCCTTTATTGTTCCCTTTACTGTGATTGTAGTTTTGTATCTGAGAGTGTTTGTGGTGGCTGTGTCTCAGGCTCGTGCCATGAGGTCTCA
ZebTARs.A027 TTGTCATTAATTACATCGCAGGACTTGTTGATGTTACTTTTTCCTTTATTGTTCCCTTTACTGTGATTGTAGTTTTGTATCTGAGAGTGTTTGTGGTGGCTGTGTCTCAGGCTCGTGCCATGAGGTCTCA
   Consensus TTGTCATTAATTACATCGCAGGACTTGTTGATGTTACTTTTTCCTTTATTGTTCCCTTTACTGTGATTGTAGTTTTGTATCTGAGAGTGTTTGTGGTGGCTGTGTCTCAGGCTCGTGCCATGAGGTCTCA

             3511  3520      3530      3540      3550      3560      3570      3580      3590      3600      3610      3620      3630      3640
             |--------+---------+---------+---------+---------+---------+---------+---------+---------+---------+---------+---------+---------|
contig066330 GCTTGCAGTCACTCACCAGCGATCAGTAACAGTAACTGCAAAGAAATCGGAGCTGAAAGCAGCCTGGACTCTTGGTATTGTTGTTGTTGTGTTTCTCATATGTATGTGTCCATATTACTGTGTGGCTCTC
ZebTARs.A027 GCTTGCAGTCACTCACCAGCGATCAGTAACAGTAACTGCAAAGAAATCGGAGCTGAAAGCAGCCTGGACTCTTGGTATTGTTGTTGTTGTGTTTCTCATATGTATGTGTCCATATTACTGTGTGGCTCTC
   Consensus GCTTGCAGTCACTCACCAGCGATCAGTAACAGTAACTGCAAAGAAATCGGAGCTGAAAGCAGCCTGGACTCTTGGTATTGTTGTTGTTGTGTTTCTCATATGTATGTGTCCATATTACTGTGTGGCTCTC

             3641  3650      3660      3670      3680      3690      3700      3710      3720      3730      3740      3750      3760      3770
             |--------+---------+---------+---------+---------+---------+---------+---------+---------+---------+---------+---------+---------|
contig066330 ACAGGCCAAGACAGCTTGCCAAGTGCTTCATCACTGACATTTGTTCTATGCTTAGCCTACTTTAACTCATGTCTGAACCCCATTATATATGTCTTTTTCTACCCCTGGTTCAGAAAATCTATAAAAGTCA
ZebTARs.A027 ACAGGCCAAGACAGCTTGCCAAGTGCTTCATCACTGACATTTGTTCTATGCTTAGCCTACTTTAACTCATGTCTGAACCCCATTATATATGTCTTTTTCTACCCCTGGTTCAGAAAATCTATAAAAGTCA
   Consensus ACAGGCCAAGACAGCTTGCCAAGTGCTTCATCACTGACATTTGTTCTATGCTTAGCCTACTTTAACTCATGTCTGAACCCCATTATATATGTCTTTTTCTACCCCTGGTTCAGAAAATCTATAAAAGTCA

             3771  3780      3790      3800      3810      3820      3830      3840      3850      3860      3870      3880      3890      3900
             |--------+---------+---------+---------+---------+---------+---------+---------+---------+---------+---------+---------+---------|
contig066330 TTGTTACTCTTCAGATACTGCAGCCTGACTCTTCTAAAGTCACCATGCGTTAGACCAAGATTTTGAAACTCTTCATCACACACAAACAAACACACACACACACACACACACATGTGTGCACACATGTGCG
ZebTARs.A027 TTGTTACTCTTCAGATACTGCAGCCTGACTCTTCTAAAGTCACCATGCGTTAG
   Consensus TTGTTACTCTTCAGATACTGCAGCCTGACTCTTCTAAAGTCACCATGCGTTAG.............................................................................
```

```
             391      400        410        420        430        440        450        460        470        480        490        500        510        520
             |--------+----------+----------+----------+----------+----------+----------+----------+----------+----------+----------+----------+---------|
contig053145 GATTGACTGGTACTGATATTTCCTATTCTATAGGTGCAACACCAGCAACAACAACAGAGGTGTGGAAGAAGGAGGGGAGGATGAATATAAAGACGGCAGACACATTTTAGTTCAGTTTGCCGTTCTTCCA
ZebTARs.A028
   Consensus ..................................................................................................................................

             521      530        540        550        560        570        580        590        600        610        620        630        640        650
             |--------+----------+----------+----------+----------+----------+----------+----------+----------+----------+----------+----------+---------|
contig053145 GCAGTGATGGAGGAAGCTGAACTCTGCTTTCAACAACTCTTTAACACCTCCTGCATGAGGCCCAGGCGTCCACACTTTGAGATCATGCTGACTTATATTTTGCTGTCCTTCATTTCTCTGCTTACTGTGA
ZebTARs.A028       ATGGAGGAAGCTGAACTCTGCTTTCAACAACTCTTTAACACCTCCTGCATGAGGCCCAGGCGTCCACACTTTGAGATCATGCTGACTTATATTTTGCTGTCCTTCATTTCTCTGCTTACTGTGA
   Consensus ......ATGGAGGAAGCTGAACTCTGCTTTCAACAACTCTTTAACACCTCCTGCATGAGGCCCAGGCGTCCACACTTTGAGATCATGCTGACTTATATTTTGCTGTCCTTCATTTCTCTGCTTACTGTGA

             651      660        670        680        690        700        710        720        730        740        750        760        770        780
             |--------+----------+----------+----------+----------+----------+----------+----------+----------+----------+----------+----------+---------|
contig053145 TTCTTAACCTGTTGGTCATCATCTCTGTCTCACATTTCAGGTAGAAAGATATCAAACTGAATTGATCTCAAGCAGTAAGAAAGACATTTCTTTTCACAGATAGTATTTCTTTTCTTTTTAAGCCTCTGCA
ZebTARs.A028 TTCTTAACCTGTTGGTCATCATCTCTGTCTCACATTTCAGG-----------------------------------------------------------------------------------------
   Consensus TTCTTAACCTGTTGGTCATCATCTCTGTCTCACATTTCAGG.........................................................................................

             781      790        800        810        820        830        840        850        860        870        880        890        900        910
             |--------+----------+----------+----------+----------+----------+----------+----------+----------+----------+----------+----------+---------|
contig053145 CTATATACTTAAATTTGAATAGCACTTGATAGAGCAAACTAAATATAAAATATTGTGTTTTCTCTCCCTATTCAGGCAGCTCCACACCCCCACCAACCTCCTGCTCCTTTCTCTGGCTGTCGCTGATTTC
ZebTARs.A028 ----------------------------------------------------------------------------CAGCTCCACACCCCCACCAACCTCCTGCTCCTTTCTCTGGCTGTCGCTGATTTC
   Consensus ............................................................................CAGCTCCACACCCCCACCAACCTCCTGCTCCTTTCTCTGGCTGTCGCTGATTTC

             911      920        930        940        950        960        970        980        990        1000       1010       1020       1030       1040
             |--------+----------+----------+----------+----------+----------+----------+----------+----------+----------+----------+----------+---------|
contig053145 TATGTGGGCCTCCTCTTGTTCTTCCAAATTGTGCTCATAGATGGCTGCTGGTTCCTCGGTGACATCATGTGCACTCTGTATCAATACCTAGCATATGTCATCACCTCGGCCTCCATAGGAACCATGGTGA
ZebTARs.A028 TATGTGGGCCTCCTCTTGTTCTTCCAAATTGTGCTCATAGATGGCTGCTGGTTCCTCGGTGACATCATGTGCACTCTGTATCAATACCTAGCATATGTCATCACCTCGGCCTCCATAGGAACCATGGTGA
   Consensus TATGTGGGCCTCCTCTTGTTCTTCCAAATTGTGCTCATAGATGGCTGCTGGTTCCTCGGTGACATCATGTGCACTCTGTATCAATACCTAGCATATGTCATCACCTCGGCCTCCATAGGAACCATGGTGA

             1041     1050       1060       1070       1080       1090       1100       1110       1120       1130       1140       1150       1160       1170
             |--------+----------+----------+----------+----------+----------+----------+----------+----------+----------+----------+----------+---------|
contig053145 TCATATCTGTTGATCGATATTTGGCTATTTGTTACCCTCTGCATTACTCCACCAAGATCACACAACAAAGAGTTAAAATTGTTGTCTGTTTGTGTTGGATCTGTTCTGTCATCTTTCAAAGTCTGATTCT
ZebTARs.A028 TCATATCTGTTGATCGATATTTGGCTATTTGTTACCCTCTGCATTACTCCACCAAGATCACACAACAAAGAGTTAAAATTGTTGTCTGTTTGTGTTGGATCTGTTCTGTCATCTTTCAAAGTCTGATTCT
   Consensus TCATATCTGTTGATCGATATTTGGCTATTTGTTACCCTCTGCATTACTCCACCAAGATCACACAACAAAGAGTTAAAATTGTTGTCTGTTTGTGTTGGATCTGTTCTGTCATCTTTCAAAGTCTGATTCT

             1171     1180       1190       1200       1210       1220       1230       1240       1250       1260       1270       1280       1290       1300
             |--------+----------+----------+----------+----------+----------+----------+----------+----------+----------+----------+----------+---------|
contig053145 GATGGATAACCTGGAGCAACCAGGCAGGTATAACTCTTGCATTGGAGAGTGTGTCTTTGTCATTAATTACATCGCAGGACTTGTTGATGTTACTTTTTCCTTTATTGTTCCCTTTACTGTGATTGTAGTT
ZebTARs.A028 GATGGATAACCTGGAGCAACCAGGCAGGTATAACTCTTGCATTGGAGAGTGTGTCTTTGTCATTAATTACATCGCAGGACTTGTTGATGTTACTTTTTCCTTTATTGTTCCCTTTACTGTGATTGTAGTT
   Consensus GATGGATAACCTGGAGCAACCAGGCAGGTATAACTCTTGCATTGGAGAGTGTGTCTTTGTCATTAATTACATCGCAGGACTTGTTGATGTTACTTTTTCCTTTATTGTTCCCTTTACTGTGATTGTAGTT

             1301     1310       1320       1330       1340       1350       1360       1370       1380       1390       1400       1410       1420       1430
             |--------+----------+----------+----------+----------+----------+----------+----------+----------+----------+----------+----------+---------|
contig053145 TTGTATCTGAGAGTGTTTGTGGTGGCTGTGTCTCAGGCTCGTGCCATGAGGTCTCAGCTTGCAGTCACTCACCAGCGATCAGTAACAGTAACTGCAAAGAAATCGGAGCTGAAAGCAGCCTGGACTCTTG
ZebTARs.A028 TTGTATCTGAGAGTGTTTGTGGTGGCTGTGTCTCAGGCTCGTGCCATGAGGTCTCAGCTTGCAGTCACTCACCAGCGATCAGTAACAGTAACTGCAAAGAAATCGGAGCTGAAAGCAGCCTGGACTCTTG
   Consensus TTGTATCTGAGAGTGTTTGTGGTGGCTGTGTCTCAGGCTCGTGCCATGAGGTCTCAGCTTGCAGTCACTCACCAGCGATCAGTAACAGTAACTGCAAAGAAATCGGAGCTGAAAGCAGCCTGGACTCTTG

             1431     1440       1450       1460       1470       1480       1490       1500       1510       1520       1530       1540       1550       1560
             |--------+----------+----------+----------+----------+----------+----------+----------+----------+----------+----------+----------+---------|
contig053145 GTATTGTTGTTGTTGTGTTTCTCATATGTATGTGTCCATATTACTGTGTGGCTCTCACAGGCCAAGATAACTTGCCAAGTGCTTCATCACTGACATTTGTTCTATGTTTGGTCTACTTTAACTCATGTCT
ZebTARs.A028 GTATTGTTGTTGTTGTGTTTCTCATATGTATGTGTCCATATTACTGTGTGGCTCTCACAGGCCAAGATAACTTGCCAAGTGCTTCATCACTGACATTTGTTCTATGTTTGGTCTACTTTAACTCATGTCT
   Consensus GTATTGTTGTTGTTGTGTTTCTCATATGTATGTGTCCATATTACTGTGTGGCTCTCACAGGCCAAGATAACTTGCCAAGTGCTTCATCACTGACATTTGTTCTATGTTTGGTCTACTTTAACTCATGTCT

             1561     1570       1580       1590       1600       1610       1620       1630       1640       1650       1660       1670       1680       1690
             |--------+----------+----------+----------+----------+----------+----------+----------+----------+----------+----------+----------+---------|
contig053145 GAACCCCATTATATATGTCTTTTTCTACCCTTGGTTCAGAAAATCCATAAAAGTCATTGTTACTCTTCAGATACTGCAGCCTGACTCCTGCCAGGCCACCGTGCTTTAGAGGACCGTTTCTCTCTCTCTC
ZebTARs.A028 GAACCCCATTATATATGTCTTTTTCTACCCTTGGTTCAGAAAATCCATAAAAGTCATTGTTACTCTTCAGATACTGCAGCCTGACTCCTGCCAGGCCACCGTGCTTTAG
   Consensus GAACCCCATTATATATGTCTTTTTCTACCCTTGGTTCAGAAAATCCATAAAAGTCATTGTTACTCTTCAGATACTGCAGCCTGACTCCTGCCAGGCCACCGTGCTTTAG.....................

             1691     1700       1710       1720       1730       1740       1750       1760       1770       1780       1790       1800       1810       1820
             |--------+----------+----------+----------+----------+----------+----------+----------+----------+----------+----------+----------+---------|
contig053145 TCTCTCTCTCTCTCTCTCTCTCTCTCTCACACACACACACACATACAATTAAGGAATTATACATTCACAGTGTGATCCCAGTCTGCTCAAGCCTGCATGCTAAATATCCTTGGGCAGGTTGCCCTCCGAT
ZebTARs.A028
   Consensus ..................................................................................................................................
```

```
              2211 2220      2230      2240      2250      2260      2270      2280      2290      2300      2310      2320      2330      2340
              |--------+---------+---------+---------+---------+---------+---------+---------+---------+---------+---------+---------+---------|
 contig030440 AGACACTGAGAGAATGATAAGGGGAGGAGTTTAAAGAAGGTGTGGGGATATTACTAATTCTGGTCACAATGCTGCAGCTCTCTGCAGCTCTGTGAAGATGGATACCCAGAGCAGAGCAGAGCTCTGCTTT
 ZebTARs.A029                                                                                                  ATGGATACCCAGAGCAGAGCAGAGCTCTGCTTT
    Consensus .................................................................................................ATGGATACCCAGAGCAGAGCAGAGCTCTGCTTT

              2341 2350      2360      2370      2380      2390      2400      2410      2420      2430      2440      2450      2460      2470
              |--------+---------+---------+---------+---------+---------+---------+---------+---------+---------+---------+---------+---------|
 contig030440 CCACAACTCTTCAACACCTCCTGCAAGAAGCCTACAACTCCTCTATCACAAGTTTTGCTCTTTCACATTCTGATGCCATCAATGTCTCTGCTGACTGTGACTCTCAACCTGCTTGTCATCATCGCAGTCT
 ZebTARs.A029 CCACAACTCTTCAACACCTCCTGCAAGAAGCCTACAACTCCTCTATCACAAGTTTTGCTCTTTCACATTCTGATGCCATCAATGTCTCTGCTGACTGTGACTCTCAACCTGCTTGTCATCATCGCAGTCT
    Consensus CCACAACTCTTCAACACCTCCTGCAAGAAGCCTACAACTCCTCTATCACAAGTTTTGCTCTTTCACATTCTGATGCCATCAATGTCTCTGCTGACTGTGACTCTCAACCTGCTTGTCATCATCGCAGTCT

              2471 2480      2490      2500      2510      2520      2530      2540      2550      2560      2570      2580      2590      2600
              |--------+---------+---------+---------+---------+---------+---------+---------+---------+---------+---------+---------+---------|
 contig030440 CCCACTTCAGGCAGAGTTTCACTTTTTTTTACCTGCTGCTGTGGCTTTACAAGTCTATACCTGATTTGTAATACCTGTTTGAATAGTTTCTTTCTGTTGATTTTTTTAAAATTATATTTTCCTGTCTTTC
 ZebTARs.A029 CCCACTTCAGGCAG--------------------------------------------------------------------------------------------------------------------
    Consensus CCCACTTCAGGCAG....................................................................................................................

              2601 2610      2620      2630      2640      2650      2660      2670      2680      2690      2700      2710      2720      2730
              |--------+---------+---------+---------+---------+---------+---------+---------+---------+---------+---------+---------+---------|
 contig030440 TAGGCAGCTCCACACACCCACTAACATCCTACTCCTCTCTCTGGCTGCCGCTGACTTTCTCATTGGCCTTCTGTTTATGCCTGGAGAAATCCTGCGAAATACAGTGTGTTGGTTTCTTGGTCAGCTCACA
 ZebTARs.A029 -------CTCCACACACCCACTAACATCCTACTCCTCTCTCTGGCTGCCGCTGACTTTCTCATTGGCCTTCTGTTTATGCCTGGAGAAATCCTGCGAAATACAGTGTGTTGGTTTCTTGGTCAGCTCACA
    Consensus .......CTCCACACACCCACTAACATCCTACTCCTCTCTCTGGCTGCCGCTGACTTTCTCATTGGCCTTCTGTTTATGCCTGGAGAAATCCTGCGAAATACAGTGTGTTGGTTTCTTGGTCAGCTCACA

              2731 2740      2750      2760      2770      2780      2790      2800      2810      2820      2830      2840      2850      2860
              |--------+---------+---------+---------+---------+---------+---------+---------+---------+---------+---------+---------+---------|
 contig030440 TGTTCTCTGTATAATTATATATCCTTCGTTGTTACCTCTGCCTCAGTGGGCGATATGGTGCTGATATCAGTCGACCGCTATGTGGCTATTTGTGACCCTCTGCATTACCCCACCAGAATCACAGAGAGAA
 ZebTARs.A029 TGTTCTCTGTATAATTATATATCCTTCGTTGTTACCTCTGCCTCAGTGGGCGATATGGTGCTGATATCAGTCGACCGCTATGTGGCTATTTGTGACCCTCTGCATTACCCCACCAGAATCACAGAGAGAA
    Consensus TGTTCTCTGTATAATTATATATCCTTCGTTGTTACCTCTGCCTCAGTGGGCGATATGGTGCTGATATCAGTCGACCGCTATGTGGCTATTTGTGACCCTCTGCATTACCCCACCAGAATCACAGAGAGAA

              2861 2870      2880      2890      2900      2910      2920      2930      2940      2950      2960      2970      2980      2990
              |--------+---------+---------+---------+---------+---------+---------+---------+---------+---------+---------+---------+---------|
 contig030440 GAGTGAAACTCTGTGTTTGTCTGTGTTGGCTCTGCTCGGTTTTCTACTGCTATATGATTGTAAGAGATGATCTAAGTCAACCAGGAAAGCATAATTCTTGCTTTGGAGAATGTATAATTTTTGTTGAATT
 ZebTARs.A029 GAGTGAAACTCTGTGTTTGTCTGTGTTGGCTCTGCTCGGTTTTCTACTGCTATATGATTGTAAGAGATGATCTAAGTCAACCAGGAAAGCATAATTCTTGCTTTGGAGAATGTATAATTTTTGTTGAATT
    Consensus GAGTGAAACTCTGTGTTTGTCTGTGTTGGCTCTGCTCGGTTTTCTACTGCTATATGATTGTAAGAGATGATCTAAGTCAACCAGGAAAGCATAATTCTTGCTTTGGAGAATGTATAATTTTTGTTGAATT

              2991 3000      3010      3020      3030      3040      3050      3060      3070      3080      3090      3100      3110      3120
              |--------+---------+---------+---------+---------+---------+---------+---------+---------+---------+---------+---------+---------|
 contig030440 CATTGCAGGATTTGTTGACCTTGTTTTATCCTTTATAATTCCAGTTACTGTGATTGTATTTCTGTATATGAGAGTGTTTGTGGTGGCTGTGTCTCAGGCCCGTGCCATGCGCTCTCAGGTTACAGCTGTC
 ZebTARs.A029 CATTGCAGGATTTGTTGACCTTGTTTTATCCTTTATAATTCCAGTTACTGTGATTGTATTTCTGTATATGAGAGTGTTTGTGGTGGCTGTGTCTCAGGCCCGTGCCATGCGCTCTCAGGTTACAGCTGTC
    Consensus CATTGCAGGATTTGTTGACCTTGTTTTATCCTTTATAATTCCAGTTACTGTGATTGTATTTCTGTATATGAGAGTGTTTGTGGTGGCTGTGTCTCAGGCCCGTGCCATGCGCTCTCAGGTTACAGCTGTC

              3121 3130      3140      3150      3160      3170      3180      3190      3200      3210      3220      3230      3240      3250
              |--------+---------+---------+---------+---------+---------+---------+---------+---------+---------+---------+---------+---------|
 contig030440 ACACTGCAGCTCTCAGTGACTCTAACAGCAACTAAATCAGAGTTAAAAGCAGCCAGGACTCTGGGTGTTCTTGTTCTTGTGTTTCTAATATGTTTCTGCCCTTATTACATTGTTTCGCTTTTTGGAGACG
 ZebTARs.A029 ACACTGCAGCTCTCAGTGACTCTAACAGCAACTAAATCAGAGTTAAAAGCAGCCAGGACTCTGGGTGTTCTTGTTCTTGTGTTTCTAATATGTTTCTGCCCTTATTACATTGTTTCGCTTTTTGGAGACG
    Consensus ACACTGCAGCTCTCAGTGACTCTAACAGCAACTAAATCAGAGTTAAAAGCAGCCAGGACTCTGGGTGTTCTTGTTCTTGTGTTTCTAATATGTTTCTGCCCTTATTACATTGTTTCGCTTTTTGGAGACG

              3251 3260      3270      3280      3290      3300      3310      3320      3330      3340      3350      3360      3370      3380
              |--------+---------+---------+---------+---------+---------+---------+---------+---------+---------+---------+---------+---------|
 contig030440 AGTTTCTCAATAGTGCCTCTGCATCTATTGTATTCTATTTCTTTCTTTTAAACTCCTGTCTAAACCCTTTGATTTATGCTATGTTCTACCCCTGGTTTAGAAAAGCTGTGAAACTAATTATGACTCTACA
 ZebTARs.A029 AGTTTCTCAATAGTGCCTCTGCATCTATTGTATTCTATTTCTTTCTTTTAAACTCCTGTCTAAACCCTTTGATTTATGCTATGTTCTACCCCTGGTTTAGAAAAGCTGTGAAACTAATTATGACTCTACA
    Consensus AGTTTCTCAATAGTGCCTCTGCATCTATTGTATTCTATTTCTTTCTTTTAAACTCCTGTCTAAACCCTTTGATTTATGCTATGTTCTACCCCTGGTTTAGAAAAGCTGTGAAACTAATTATGACTCTACA

              3381 3390      3400      3410      3420      3430      3440      3450      3460      3470      3480      3490      3500      3510
              |--------+---------+---------+---------+---------+---------+---------+---------+---------+---------+---------+---------+---------|
 contig030440 GATACTGCAGCCTGGCTCCTGTGAAGTCAGCATACTGTAGAAGGAAGAAATCTGTTCTAAAAAGATTACCATAATCAAAACTAAGTTTAAATTCGGTTCTGGATTTAACAGGGAGAAGATGAAGAGAAGT
 ZebTARs.A029 GATACTGCAGCCTGGCTCCTGTGAAGTCAGCATACTGTAG
    Consensus GATACTGCAGCCTGGCTCCTGTGAAGTCAGCATACTGTAG..........................................................................................

              3511 3520      3530      3540      3550      3560      3570      3580      3590      3600      3610      3620      3630      3640
              |--------+---------+---------+---------+---------+---------+---------+---------+---------+---------+---------+---------+---------|
 contig030440 CAGTATGGGAGAAATATACTCTCTCTTTCTAGTCCCTGTCAGTACTCTTGCTGCAGCATTTTGGATTGACTTTCAGGGATTTTTTTTTTTAGGACATCCTGACAATAATGAATTGGTAAATGGCCTGCAT
 ZebTARs.A029
    Consensus ..................................................................................................................................

              3641 3650      3660      3670      3680      3690      3700      3710      3720      3730      3740      3750      3760      3770
              |--------+---------+---------+---------+---------+---------+---------+---------+---------+---------+---------+---------+---------|
 contig030440 TTGTATAGCGCTTTTCTAGTCCATAGGACCCCAAAGCGCTTTACACTACATTCAGTCATTCACCCATTCACACACACATTGACACACTGGCGATAGCAAGCTACATTGTAGCCACAGCTGCCCTGGGGCG
 ZebTARs.A029
    Consensus ..................................................................................................................................

              3771 3780      3790
              |--------+---------|
 contig030440 CACTGACAGGCGAGGCTGCC
 ZebTARs.A029
    Consensus
```

```
            1951 1960      1970      1980      1990      2000      2010      2020      2030      2040      2050      2060      2070      2080
contig033536 TTGAATGCTGAAAATTGAAACTTGTTCCAGGGCTGTGACAGGGAGTCATGGAGTTATTCAATGTGACTGTTAATACTGTGAGCTTCCTTCTCTGCGATTCACAAAAGAATAAATTATGTGTGTTATTATA
ZebTAR.B029                                                 ATGGAGTTATTCAATGTGACTGTTAATACTGTGAGCTTCCTTCTCTGCGATTCACAAAAGAATAAATTATGTGTGTTATTATA
Consensus    ...............................................ATGGAGTTATTCAATGTGACTGTTAATACTGTGAGCTTCCTTCTCTGCGATTCACAAAAGAATAAATTATGTGTGTTATTATA

            2081 2090      2100      2110      2120      2130      2140      2150      2160      2170      2180      2190      2200      2210
contig033536 TGTTGTCCTCAGCTCATTTATGCTTCTTACAATATGTGGAAATCTTCTTGTGATAATTTCTATAATTTACTTTAGATATCTCCACACTCCTACAAACTACCTTATCCTCTCTATGGCTGTGGCTGATCTA
ZebTAR.B029  TGTTGTCCTCAGCTCATTTATGCTTCTTACAATATGTGGAAATCTTCTTGTGATAATTTCTATAATTTACTTTAGATATCTCCACACTCCTACAAACTACCTTATCCTCTCTATGGCTGTGGCTGATCTA
Consensus    TGTTGTCCTCAGCTCATTTATGCTTCTTACAATATGTGGAAATCTTCTTGTGATAATTTCTATAATTTACTTTAGATATCTCCACACTCCTACAAACTACCTTATCCTCTCTATGGCTGTGGCTGATCTA

            2211 2220      2230      2240      2250      2260      2270      2280      2290      2300      2310      2320      2330      2340
contig033536 CTAATTGGTGCTTTAATATTTCCTTTGAGCATGACAGTGTCTCTAAAGCCATGTTTGTATATATACAGTTTACTGTGCAACTTAAGAAGCACAATGGATGTAACAATGGGTGTATCTTCCTTATTAAATT
ZebTAR.B029  CTAATTGGTGCTTTAATATTTCCTTTGAGCATGACAGTGTCTCTAAAGCCATGTTTGTATATATACAGTTTACTGTGCAACTTAAGAAGCACAATGGATGTAACAATGGGTGTATCTTCCTTATTAAATT
Consensus    CTAATTGGTGCTTTAATATTTCCTTTGAGCATGACAGTGTCTCTAAAGCCATGTTTGTATATATACAGTTTACTGTGCAACTTAAGAAGCACAATGGATGTAACAATGGGTGTATCTTCCTTATTAAATT

            2341 2350      2360      2370      2380      2390      2400      2410      2420      2430      2440      2450      2460      2470
contig033536 TGTGCTGTATTTCTGTTGATCGATATTATGCTGTTTGCCACCCTCTGATATATAAAACTAAAATAACTGATTGTGTTGCCATGAAGATGGGCCTTGGAAGTTGGGCTGTTGCTATCTTGTGTGGAATTTT
ZebTAR.B029  TGTGCTGTATTTCTGTTGATCGATATTATGCTGTTTGCCACCCTCTGATATATAAAACTAAAATAACTGATTGTGTTGCCATGAAGATGGGCCTTGGAAGTTGGGCTGTTGCTATCTTGTGTGGAATTTT
Consensus    TGTGCTGTATTTCTGTTGATCGATATTATGCTGTTTGCCACCCTCTGATATATAAAACTAAAATAACTGATTGTGTTGCCATGAAGATGGGCCTTGGAAGTTGGGCTGTTGCTATCTTGTGTGGAATTTT

            2471 2480      2490      2500      2510      2520      2530      2540      2550      2560      2570      2580      2590      2600
contig033536 TGTCTTCCTGCTGTTTTTTATTTTAGACGAATGTGACACAAGTTGCGTTTTCGCTCTCATTGCCGCATCAGTGGTAGTATATTATATCCCAACAATAGTTTTACTTTTCATGTACACCAAAATTCTTGTT
ZebTAR.B029  TGTCTTCCTGCTGTTTTTTATTTTAGACGAATGTGACACAAGTTGCGTTTTCGCTCTCATTGCCGCATCAGTGGTAGTATATTATATCCCAACAATAGTTTTACTTTTCATGTACACCAAAATTCTTGTT
Consensus    TGTCTTCCTGCTGTTTTTTATTTTAGACGAATGTGACACAAGTTGCGTTTTCGCTCTCATTGCCGCATCAGTGGTAGTATATTATATCCCAACAATAGTTTTACTTTTCATGTACACCAAAATTCTTGTT

            2601 2610      2620      2630      2640      2650      2660      2670      2680      2690      2700      2710      2720      2730
contig033536 GTTGCACTGAGGCAGGCACGCAGCATCCATAATACAATTTCTCAGAACACAAAGTCTAAAGCAGTTTCCAGTACGGAGAGAAAGGCCACAAAGACATTAACCATAGTTATTGGAATATTTTTAATTTGTT
ZebTAR.B029  GTTGCACTGAGGCAGGCACGCAGCATCCATAATACAATTTCTCAGAACACAAAGTCTAAAGCAGTTTCCAGTACGGAGAGAAAGGCCACAAAGACATTAACCATAGTTATTGGAATATTTTTAATTTGTT
Consensus    GTTGCACTGAGGCAGGCACGCAGCATCCATAATACAATTTCTCAGAACACAAAGTCTAAAGCAGTTTCCAGTACGGAGAGAAAGGCCACAAAGACATTAACCATAGTTATTGGAATATTTTTAATTTGTT

            2731 2740      2750      2760      2770      2780      2790      2800      2810      2820      2830      2840      2850      2860
contig033536 GGGTTCCTCTTTTTCTTAGTTATTCATTTGTTCCTCTGGATAGTTTTATACTTTATGTGCTTCTTGAACCATTTAACTGGTTTGCAATTTCTAACTCAATGCTCAATCCCTTTATTTATGCTTTCTTTTA
ZebTAR.B029  GGGTTCCTCTTTTTCTTAGTTATTCATTTGTTCCTCTGGATAGTTTTATACTTTATGTGCTTCTTGAACCATTTAACTGGTTTGCAATTTCTAACTCAATGCTCAATCCCTTTATTTATGCTTTCTTTTA
Consensus    GGGTTCCTCTTTTTCTTAGTTATTCATTTGTTCCTCTGGATAGTTTTATACTTTATGTGCTTCTTGAACCATTTAACTGGTTTGCAATTTCTAACTCAATGCTCAATCCCTTTATTTATGCTTTCTTTTA

            2861 2870      2880      2890      2900      2910      2920      2930      2940      2950      2960      2970      2980      2990
contig033536 CACTTGGTTTAGAAGAGCTTTTAAAATGATAATTTCTGGAAAAATATTTCAAGGTGATGTTACTAACATAAAACTACACTGACAGCTATATATATATTATGAATTATATCAATAAGAGTAAATATCACCA
ZebTAR.B029  CACTTGGTTTAGAAGAGCTTTTAAAATGATAATTTCTGGAAAAATATTTCAAGGTGATGTTACTAACATAAAACTACACTGA
Consensus    CACTTGGTTTAGAAGAGCTTTTAAAATGATAATTTCTGGAAAAATATTTCAAGGTGATGTTACTAACATAAAACTACACTGA................................................
```

```
            1196111970    11980     11990     12000     12010     12020     12030     12040     12050     12060     12070     12080     12090
            |---------+---------+---------+---------+---------+---------+---------+---------+---------+---------+---------+---------+---------|
contig032272 ATCAGAGATAAATAAGCTGTACAATGTCTCACAGGGTCAGCAGGTTGCTTGAACTCAGCAGGTCCTCCTTTCTTTATACTGGATGATGGACAGGCAGGACAGAATTGAACTCTGCTACCCTGAGCTTGCG
 NyeTAR.A004                                                                                      ATGGACAGGCAGGACAGAATTGAACTCTGCTACCCTGAGCTTGCG
   Consensus .....................................................................................ATGGACAGGCAGGACAGAATTGAACTCTGCTACCCTGAGCTTGCG

            1209112100    12110     12120     12130     12140     12150     12160     12170     12180     12190     12200     12210     12220
            |---------+---------+---------+---------+---------+---------+---------+---------+---------+---------+---------+---------+---------|
contig032272 AATCTGTCTTGCAGGGTTTTAACACGTCCTCAGCCTGAGGCTGTCCTCCTCTATACACTGTTCTTGTCTATCTCTGTGCTAACTGTAGCTCTGAATGTGCTGGTTATCATATCCATCTCTCATTTCAGGC
 NyeTAR.A004 AATCTGTCTTGCAGGGTTTTAACACGTCCTCAGCCTGAGGCTGTCCTCCTCTATACACTGTTCTTGTCTATCTCTGTGCTAACTGTAGCTCTGAATGTGCTGGTTATCATATCCATCTCTCATTTCAGGC
   Consensus AATCTGTCTTGCAGGGTTTTAACACGTCCTCAGCCTGAGGCTGTCCTCCTCTATACACTGTTCTTGTCTATCTCTGTGCTAACTGTAGCTCTGAATGTGCTGGTTATCATATCCATCTCTCATTTCAGGC

            1222112230    12240     12250     12260     12270     12280     12290     12300     12310     12320     12330     12340     12350
            |---------+---------+---------+---------+---------+---------+---------+---------+---------+---------+---------+---------+---------|
contig032272 AGCTCCACATGCCCACCAATGTCCTCCTGCTCTCTCTGGCCATCTCAGACCTCTTGGTCGGGCTGCTTGTGATGCCGGTGGAAGCAATGCGGTTTATAGAAACCTGCTGGCTGCTGGGAGATCTCATGTG
 NyeTAR.A004 AGCTCCACATGCCCACCAATGTCCTCCTGCTCTCTCTGGCCATCTCAGACCTCTTGGTCGGGCTGCTTGTGATGCCGGTGGAAGCAATGCGGTTTATAGAAACCTGCTGGCTGCTGGGAGATCTCATGTG
   Consensus AGCTCCACATGCCCACCAATGTCCTCCTGCTCTCTCTGGCCATCTCAGACCTCTTGGTCGGGCTGCTTGTGATGCCGGTGGAAGCAATGCGGTTTATAGAAACCTGCTGGCTGCTGGGAGATCTCATGTG

            1235112360    12370     12380     12390     12400     12410     12420     12430     12440     12450     12460     12470     12480
            |---------+---------+---------+---------+---------+---------+---------+---------+---------+---------+---------+---------+---------|
contig032272 TGCTTTTACTTACATTATCGGTTTCACTCTCACCTCAGCCTCTGTGGGCAACATGGTGCTCATATCAATTGATCGCTACGTAGCTATTTGTTATCCTCTGCAGTACCCCACTAAAATCACTCACAGCAGA
 NyeTAR.A004 TGCTTTTACTTACATTATCGGTTTCACTCTCACCTCAGCCTCTGTGGGCAACATGGTGCTCATATCAATTGATCGCTACGTAGCTATTTGTTATCCTCTGCAGTACCCCACTAAAATCACTCACAGCAGA
   Consensus TGCTTTTACTTACATTATCGGTTTCACTCTCACCTCAGCCTCTGTGGGCAACATGGTGCTCATATCAATTGATCGCTACGTAGCTATTTGTTATCCTCTGCAGTACCCCACTAAAATCACTCACAGCAGA

            1248112490    12500     12510     12520     12530     12540     12550     12560     12570     12580     12590     12600     12610
            |---------+---------+---------+---------+---------+---------+---------+---------+---------+---------+---------+---------+---------|
contig032272 GCGGAGCTGTCTGTGACTCTGTGCTGGGCCTGTTCGCTCCTCTACAACGGGATGATCTTAAAGGAGCATCTCAGGCAGCCAGACAGGCATAACACCTGTCACGGTCAGTGTTTGGTGGTGATTAACTATG
 NyeTAR.A004 GCGGAGCTGTCTGTGACTCTGTGCTGGGCCTGTTCGCTCCTCTACAACGGGATGATCTTAAAGGAGCATCTCAGGCAGCCAGACAGGCATAACACCTGTCACGGTCAGTGTTTGGTGGTGATTAACTATG
   Consensus GCGGAGCTGTCTGTGACTCTGTGCTGGGCCTGTTCGCTCCTCTACAACGGGATGATCTTAAAGGAGCATCTCAGGCAGCCAGACAGGCATAACACCTGTCACGGTCAGTGTTTGGTGGTGATTAACTATG

            1261112620    12630     12640     12650     12660     12670     12680     12690     12700     12710     12720     12730     12740
            |---------+---------+---------+---------+---------+---------+---------+---------+---------+---------+---------+---------+---------|
contig032272 TCCCTGGAGCCATCGACCTTGTCTTTACTTTTATCGGCCCTTGCTCAGTAATTATCATTCTGTACATGAGAGTATTTGTTGTAGCGGTGTCTCAGGCTCATGCCATGCGGTCTCATATTACAGCTGCTGC
 NyeTAR.A004 TCCCTGGAGCCATCGACCTTGTCTTTACTTTTATCGGCCCTTGCTCAGTAATTATCATTCTGTACATGAGAGTATTTGTTGTAGCGGTGTCTCAGGCTCATGCCATGCGGTCTCATATTACAGCTGCTGC
   Consensus TCCCTGGAGCCATCGACCTTGTCTTTACTTTTATCGGCCCTTGCTCAGTAATTATCATTCTGTACATGAGAGTATTTGTTGTAGCGGTGTCTCAGGCTCATGCCATGCGGTCTCATATTACAGCTGCTGC

            1274112750    12760     12770     12780     12790     12800     12810     12820     12830     12840     12850     12860     12870
            |---------+---------+---------+---------+---------+---------+---------+---------+---------+---------+---------+---------+---------|
contig032272 AGCTGGTACAGTAAAAATCACCGCAAAGAAATCAGAGAAAAAGGCAGCCAGGACTCTTGGCGTTGTGATATTTGTGTTTTTGATGAGTTTCTGTCCGTATTACTATCCCTCTCTTGCAGGGCAGGACATT
 NyeTAR.A004 AGCTGGTACAGTAAAAATCACCGCAAAGAAATCAGAGAAAAAGGCAGCCAGGACTCTTGGCGTTGTGATATTTGTGTTTTTGATGAGTTTCTGTCCGTATTACTATCCCTCTCTTGCAGGGCAGGACATT
   Consensus AGCTGGTACAGTAAAAATCACCGCAAAGAAATCAGAGAAAAAGGCAGCCAGGACTCTTGGCGTTGTGATATTTGTGTTTTTGATGAGTTTCTGTCCGTATTACTATCCCTCTCTTGCAGGGCAGGACATT

            1287112880    12890     12900     12910     12920     12930     12940     12950     12960     12970     12980     12990     13000
            |---------+---------+---------+---------+---------+---------+---------+---------+---------+---------+---------+---------+---------|
contig032272 TCAAACAGTGCTTCATCTTGGGCTATTGTTTCCTGGATGCTGTACTTCAATTCTTGTCTGAATCCACTGATATATGCTTTTTTCTACCCGTGGTTTAGAAAAGCTATCTGGTTCATTGTCTCCCTGAAGA
 NyeTAR.A004 TCAAACAGTGCTTCATCTTGGGCTATTGTTTCCTGGATGCTGTACTTCAATTCTTGTCTGAATCCACTGATATATGCTTTTTTCTACCCGTGGTTTAGAAAAGCTATCTGGTTCATTGTCTCCCTGAAGA
   Consensus TCAAACAGTGCTTCATCTTGGGCTATTGTTTCCTGGATGCTGTACTTCAATTCTTGTCTGAATCCACTGATATATGCTTTTTTCTACCCGTGGTTTAGAAAAGCTATCTGGTTCATTGTCTCCCTGAAGA

            1300113010    13020     13030     13040     13050     13060     13070     13080     13090     13100     13110     13120     13130
            |---------+---------+---------+---------+---------+---------+---------+---------+---------+---------+---------+---------+---------|
contig032272 TACTAGAGAAGGGCTCCTCTCAGGCAAATATACTTTAAAATAGCAAATTTATCCTGAACCAAATCAGTCTGTATGAGAGAATGAAACTGCTGATTATTCTATGTGGCAGTAGCATAATGGTTTTTAATTA
 NyeTAR.A004 TACTAGAGAAGGGCTCCTCTCAGGCAAATATACTTTAA
   Consensus TACTAGAGAAGGGCTCCTCTCAGGCAAATATACTTTAA............................................................................................
```

```
  NyeTAR.A005
    Consensus  ..................................................................................................................................

               911      920       930       940       950       960       970       980       990       1000      1010      1020      1030      1040
               |--------+---------+---------+---------+---------+---------+---------+---------+---------+---------+---------+---------+---------|
  contig038663 CCTTTGACTGCATGCACATGCATGCACCGCTCCTCTCCGATGGATAGCTCTGGAGGTCCTCCCCTCTGCTTCCCCAACCTCAACTCCTCCTGCAGGCGCCTGCTGCGACCCACCTCCCAGACCGTTGTTC
   NyeTAR.A005                                        ATGGATAGCTCTGGAGGTCCTCCCCTCTGCTTCCCCAACCTCAACTCCTCCTGCAGGCGCCTGCTGCGACCCACCTCCCAGACCGTTGTTC
     Consensus .......................................ATGGATAGCTCTGGAGGTCCTCCCCTCTGCTTCCCCAACCTCAACTCCTCCTGCAGGCGCCTGCTGCGACCCACCTCCCAGACCGTTGTTC

               1041     1050      1060      1070      1080      1090      1100      1110      1120      1130      1140      1150      1160      1170
               |--------+---------+---------+---------+---------+---------+---------+---------+---------+---------+---------+---------+---------|
  contig038663 TCTACACTCTGCTGGCCTCCATCTCCCTGCTCACTGTGGTGCTCAACCTGCTTGTCGTCATCTCCATCTCCCACTTCAGACAGCTTAACACCTCGACCAACACCCTGCTCCTTTCCCTGGCTGTGTCTGA
   NyeTAR.A005 TCTACACTCTGCTGGCCTCCATCTCCCTGCTCACTGTGGTGCTCAACCTGCTTGTCGTCATCTCCATCTCCCACTTCAGACAGCTTAACACCTCGACCAACACCCTGCTCCTTTCCCTGGCTGTGTCTGA
     Consensus TCTACACTCTGCTGGCCTCCATCTCCCTGCTCACTGTGGTGCTCAACCTGCTTGTCGTCATCTCCATCTCCCACTTCAGACAGCTTAACACCTCGACCAACACCCTGCTCCTTTCCCTGGCTGTGTCTGA

               1171     1180      1190      1200      1210      1220      1230      1240      1250      1260      1270      1280      1290      1300
               |--------+---------+---------+---------+---------+---------+---------+---------+---------+---------+---------+---------+---------|
  contig038663 CCTGCTGGTGGGGTTGCTGGTGATGCCCATCGAGGGCCTGTACTACATCGAGACGTGCTGGCTGCTGGGGAGGCTGATGTGTGCTCTCAGTCCTTATTTGTCTTACTGCCTTATCTCTGTCTCTCTGGGC
   NyeTAR.A005 CCTGCTGGTGGGGTTGCTGGTGATGCCCATCGAGGGCCTGTACTACATCGAGACGTGCTGGCTGCTGGGGAGGCTGATGTGTGCTCTCAGTCCTTATTTGTCTTACTGCCTTATCTCTGTCTCTCTGGGC
     Consensus CCTGCTGGTGGGGTTGCTGGTGATGCCCATCGAGGGCCTGTACTACATCGAGACGTGCTGGCTGCTGGGGAGGCTGATGTGTGCTCTCAGTCCTTATTTGTCTTACTGCCTTATCTCTGTCTCTCTGGGC

               1301     1310      1320      1330      1340      1350      1360      1370      1380      1390      1400      1410      1420      1430
               |--------+---------+---------+---------+---------+---------+---------+---------+---------+---------+---------+---------+---------|
  contig038663 AGCATGGTGCTCATATCTGTAGATCGCTATATAGCCATCTGTGACCCTCTGCTCTATTCCTCAAAGATCACAGTGAACAGAGTGAAGCTTTCAGTCTGTGTCTGCTGGGCCTGCTCTCTTCTCTACAATG
   NyeTAR.A005 AGCATGGTGCTCATATCTGTAGATCGCTATATAGCCATCTGTGACCCTCTGCTCTATTCCTCAAAGATCACAGTGAACAGAGTGAAGCTTTCAGTCTGTGTCTGCTGGGCCTGCTCTCTTCTCTACAATG
     Consensus AGCATGGTGCTCATATCTGTAGATCGCTATATAGCCATCTGTGACCCTCTGCTCTATTCCTCAAAGATCACAGTGAACAGAGTGAAGCTTTCAGTCTGTGTCTGCTGGGCCTGCTCTCTTCTCTACAATG

               1431     1440      1450      1460      1470      1480      1490      1500      1510      1520      1530      1540      1550      1560
               |--------+---------+---------+---------+---------+---------+---------+---------+---------+---------+---------+---------+---------|
  contig038663 GCTGCATTCTCATGGAGCACATAGGGTGGCCAGACAGGTTCGGCTCCTGTCACGGGGAGTGTGTGGTGTTCATCAGCCGCGCTTTGGGTACAATAGATCTCTTTTTCTCATTTCTCGGCCCATGTGCCTT
   NyeTAR.A005 GCTGCATTCTCATGGAGCACATAGGGTGGCCAGACAGGTTCGGCTCCTGTCACGGGGAGTGTGTGGTGTTCATCAGCCGCGCTTTGGGTACAATAGATCTCTTTTTCTCATTTCTCGGCCCATGTGCCTT
     Consensus GCTGCATTCTCATGGAGCACATAGGGTGGCCAGACAGGTTCGGCTCCTGTCACGGGGAGTGTGTGGTGTTCATCAGCCGCGCTTTGGGTACAATAGATCTCTTTTTCTCATTTCTCGGCCCATGTGCCTT

               1561     1570      1580      1590      1600      1610      1620      1630      1640      1650      1660      1670      1680      1690
               |--------+---------+---------+---------+---------+---------+---------+---------+---------+---------+---------+---------+---------|
  contig038663 AATGTTTGTTCTGTACATGAGGGTCTTTGTGGTTGCTGTTTCTCAGGTACGTATCATTCGGTCGCAGGCAGCTGTGAGAGCAGCTCCAGCTGCTAAAAAATCAGAGCTGAAGGCAGCCAGGACACTCGGG
   NyeTAR.A005 AATGTTTGTTCTGTACATGAGGGTCTTTGTGGTTGCTGTTTCTCAGGTACGTATCATTCGGTCGCAGGCAGCTGTGAGAGCAGCTCCAGCTGCTAAAAAATCAGAGCTGAAGGCAGCCAGGACACTCGGG
     Consensus AATGTTTGTTCTGTACATGAGGGTCTTTGTGGTTGCTGTTTCTCAGGTACGTATCATTCGGTCGCAGGCAGCTGTGAGAGCAGCTCCAGCTGCTAAAAAATCAGAGCTGAAGGCAGCCAGGACACTCGGG

               1691     1700      1710      1720      1730      1740      1750      1760      1770      1780      1790      1800      1810      1820
               |--------+---------+---------+---------+---------+---------+---------+---------+---------+---------+---------+---------+---------|
  contig038663 ATTTTGATAGCTGTGTTTGTAATGTGCTTCTGCCCTTATTATTATCCTTCCTTTGCAGGTGACGACACCTCTATGAGCTTACCTTATTACGCTCTATTCTCGTGGATAATGCTGACAAATTCTTGTGTGA
   NyeTAR.A005 ATTTTGATAGCTGTGTTTGTAATGTGCTTCTGCCCTTATTATTATCCTTCCTTTGCAGGTGACGACACCTCTATGAGCTTACCTTATTACGCTCTATTCTCGTGGATAATGCTGACAAATTCTTGTGTGA
     Consensus ATTTTGATAGCTGTGTTTGTAATGTGCTTCTGCCCTTATTATTATCCTTCCTTTGCAGGTGACGACACCTCTATGAGCTTACCTTATTACGCTCTATTCTCGTGGATAATGCTGACAAATTCTTGTGTGA

               1821     1830      1840      1850      1860      1870      1880      1890      1900      1910      1920      1930      1940      1950
               |--------+---------+---------+---------+---------+---------+---------+---------+---------+---------+---------+---------+---------|
  contig038663 ACCCTGTGATTTATGCTCTGTTCTACCCCTGGTTTAAAAGAGCTATCAGACTCATTGTCACCCTCAGAATACTGCAGCCTCACTCCAGAGATGTCAAGATCCTGTAGGCAGCAGTAAAACAAACCAGTCA
   NyeTAR.A005 ACCCTGTGATTTATGCTCTGTTCTACCCCTGGTTTAAAAGAGCTATCAGACTCATTGTCACCCTCAGAATACTGCAGCCTCACTCCAGAGATGTCAAGATCCTGTAG
     Consensus ACCCTGTGATTTATGCTCTGTTCTACCCCTGGTTTAAAAGAGCTATCAGACTCATTGTCACCCTCAGAATACTGCAGCCTCACTCCAGAGATGTCAAGATCCTGTAG.......................
```

```
             2081  2090      2100      2110      2120      2130      2140      2150      2160      2170      2180      2190      2200      2210
             |--------+---------+---------+---------+---------+---------+---------+---------+---------+---------+---------+---------+---------|
contig035375 ATAAGGGGAGGAGTTTAAAGAAGGTGTGGGGATATTACTAATTCTGGTCACAATGCTGCAGCTCTCTGCAGGTCTGTGAAGATGGATACCCAGAGCAGAGCAGATCTCTGCTTTCCACAACTCTTCAACA
NyeTARs.A013                                                                                  ATGGATACCCAGAGCAGAGCAGATCTCTGCTTTCCACAACTCTTCAACA
   Consensus .................................................................................ATGGATACCCAGAGCAGAGCAGATCTCTGCTTTCCACAACTCTTCAACA

             2211  2220      2230      2240      2250      2260      2270      2280      2290      2300      2310      2320      2330      2340
             |--------+---------+---------+---------+---------+---------+---------+---------+---------+---------+---------+---------+---------|
contig035375 CCTCCTGCAAGAAGCCTACAACTCCTCTATCACAAGTTTTGCTCTTTCACATTCTGATGCCATCAATGTGTCTGCTAACTGTGACTCTCAACCTGCTCGTCATCATCGCAATCTCCCACTTCAGGCAGAG
NyeTARs.A013 CCTCCTGCAAGAAGCCTACAACTCCTCTATCACAAGTTTTGCTCTTTCACATTCTGATGCCATCAATGTGTCTGCTAACTGTGACTCTCAACCTGCTCGTCATCATCGCAATCTCCCACTTCAGGCAG--
   Consensus CCTCCTGCAAGAAGCCTACAACTCCTCTATCACAAGTTTTGCTCTTTCACATTCTGATGCCATCAATGTGTCTGCTAACTGTGACTCTCAACCTGCTCGTCATCATCGCAATCTCCCACTTCAGGCAG..

             2341  2350      2360      2370      2380      2390      2400      2410      2420      2430      2440      2450      2460      2470
             |--------+---------+---------+---------+---------+---------+---------+---------+---------+---------+---------+---------+---------|
contig035375 TTTCACTTTTTTTTACCTGCTGCTGTGGCTTTACAAGTCTATACCTGATTTGTAATACCTGTTTGAATAGTTTCTTTCTGTTGATTTTTTAAAATTATATTTTCCTGTCTTTCTAGGCAGCTCCACTCAC
NyeTARs.A013 ------------------------------------------------------------------------------------------------------------------------CTCCACTCAC
   Consensus ........................................................................................................................CTCCACTCAC

             2471  2480      2490      2500      2510      2520      2530      2540      2550      2560      2570      2580      2590      2600
             |--------+---------+---------+---------+---------+---------+---------+---------+---------+---------+---------+---------+---------|
contig035375 ACACTAACATCCTGCTCCTCTCTCTGGCTGCCGCTGACTTTCTCATTGGCCTTCTGTTTATGCCTGGAGAAATCCTGCGAAATACAGTGTGTTGGTTTCTTGGTCAGCTCACATGTTCTCTGTATAATTA
NyeTARs.A013 ACACTAACATCCTGCTCCTCTCTCTGGCTGCCGCTGACTTTCTCATTGGCCTTCTGTTTATGCCTGGAGAAATCCTGCGAAATACAGTGTGTTGGTTTCTTGGTCAGCTCACATGTTCTCTGTATAATTA
   Consensus ACACTAACATCCTGCTCCTCTCTCTGGCTGCCGCTGACTTTCTCATTGGCCTTCTGTTTATGCCTGGAGAAATCCTGCGAAATACAGTGTGTTGGTTTCTTGGTCAGCTCACATGTTCTCTGTATAATTA

             2601  2610      2620      2630      2640      2650      2660      2670      2680      2690      2700      2710      2720      2730
             |--------+---------+---------+---------+---------+---------+---------+---------+---------+---------+---------+---------+---------|
contig035375 TATATCCTTCGTTGTTACCTCTGCCTCAGTGGGCATCATGGTGCTGATATCAGTCGACCGCTATGTGGCTATTTGTGACCCTCTGCATTACCCCACCAGAATCACAGAGAGAAGAGTGAAACTCTGTGTT
NyeTARs.A013 TATATCCTTCGTTGTTACCTCTGCCTCAGTGGGCATCATGGTGCTGATATCAGTCGACCGCTATGTGGCTATTTGTGACCCTCTGCATTACCCCACCAGAATCACAGAGAGAAGAGTGAAACTCTGTGTT
   Consensus TATATCCTTCGTTGTTACCTCTGCCTCAGTGGGCATCATGGTGCTGATATCAGTCGACCGCTATGTGGCTATTTGTGACCCTCTGCATTACCCCACCAGAATCACAGAGAGAAGAGTGAAACTCTGTGTT

             2731  2740      2750      2760      2770      2780      2790      2800      2810      2820      2830      2840      2850      2860
             |--------+---------+---------+---------+---------+---------+---------+---------+---------+---------+---------+---------+---------|
contig035375 TGTCTGTGTTGGCTCTGCTCGGTTTTCTACTGCTATATGATTGTAAGAGATGATCTAAGTCAACCAGGAAAGCATAATTCTTGCTTTGGAGAATGTATAATTTTTGTTGAATTCATTGCAGGATTTGTTG
NyeTARs.A013 TGTCTGTGTTGGCTCTGCTCGGTTTTCTACTGCTATATGATTGTAAGAGATGATCTAAGTCAACCAGGAAAGCATAATTCTTGCTTTGGAGAATGTATAATTTTTGTTGAATTCATTGCAGGATTTGTTG
   Consensus TGTCTGTGTTGGCTCTGCTCGGTTTTCTACTGCTATATGATTGTAAGAGATGATCTAAGTCAACCAGGAAAGCATAATTCTTGCTTTGGAGAATGTATAATTTTTGTTGAATTCATTGCAGGATTTGTTG

             2861  2870      2880      2890      2900      2910      2920      2930      2940      2950      2960      2970      2980      2990
             |--------+---------+---------+---------+---------+---------+---------+---------+---------+---------+---------+---------+---------|
contig035375 ACCTTGTTTTATCCTTTATAATTCCAGTTACTGTGATTGTATTTCTGTATATGAGAGTGTTTGTGGTGGCTGTGTCTCAGGCCCGTGCCATGCGCTCTCAGGTTACAGCTGTCACACTGCAGCTCTCAGT
NyeTARs.A013 ACCTTGTTTTATCCTTTATAATTCCAGTTACTGTGATTGTATTTCTGTATATGAGAGTGTTTGTGGTGGCTGTGTCTCAGGCCCGTGCCATGCGCTCTCAGGTTACAGCTGTCACACTGCAGCTCTCAGT
   Consensus ACCTTGTTTTATCCTTTATAATTCCAGTTACTGTGATTGTATTTCTGTATATGAGAGTGTTTGTGGTGGCTGTGTCTCAGGCCCGTGCCATGCGCTCTCAGGTTACAGCTGTCACACTGCAGCTCTCAGT

             2991  3000      3010      3020      3030      3040      3050      3060      3070      3080      3090      3100      3110      3120
             |--------+---------+---------+---------+---------+---------+---------+---------+---------+---------+---------+---------+---------|
contig035375 GACTCTAACAGCAAAGAAATCAGAGTTAAAAGCAGCCAGGACTCTGGGTGTTCTTGTTCTTGTGTTTCTAATATGTTTCTGCCCATATTACATTGTTTCGCTTTTTGGAGACGAGTTTCTCAATAGTGCC
NyeTARs.A013 GACTCTAACAGCAAAGAAATCAGAGTTAAAAGCAGCCAGGACTCTGGGTGTTCTTGTTCTTGTGTTTCTAATATGTTTCTGCCCATATTACATTGTTTCGCTTTTTGGAGACGAGTTTCTCAATAGTGCC
   Consensus GACTCTAACAGCAAAGAAATCAGAGTTAAAAGCAGCCAGGACTCTGGGTGTTCTTGTTCTTGTGTTTCTAATATGTTTCTGCCCATATTACATTGTTTCGCTTTTTGGAGACGAGTTTCTCAATAGTGCC

             3121  3130      3140      3150      3160      3170      3180      3190      3200      3210      3220      3230      3240      3250
             |--------+---------+---------+---------+---------+---------+---------+---------+---------+---------+---------+---------+---------|
contig035375 TCTGCATCTATTGTATTCTATTTCTTTCTTTTAAACTCATGTCTAAACCCTTTGATCTATGCTATGTTCTACCCCTGGTTTAGAAAAGCTGTGAAACTAATTATGACTCTACAAATACTGCAGCCTGGCT
NyeTARs.A013 TCTGCATCTATTGTATTCTATTTCTTTCTTTTAAACTCATGTCTAAACCCTTTGATCTATGCTATGTTCTACCCCTGGTTTAGAAAAGCTGTGAAACTAATTATGACTCTACAAATACTGCAGCCTGGCT
   Consensus TCTGCATCTATTGTATTCTATTTCTTTCTTTTAAACTCATGTCTAAACCCTTTGATCTATGCTATGTTCTACCCCTGGTTTAGAAAAGCTGTGAAACTAATTATGACTCTACAAATACTGCAGCCTGGCT

             3251  3260      3270      3280      3290      3300      3310      3320      3330      3340      3350      3360      3370      3380
             |--------+---------+---------+---------+---------+---------+---------+---------+---------+---------+---------+---------+---------|
contig035375 CCTGTGAAGTCAGCATACTGTAGAAGGAAGAAATCTGTTCTAAAAAGATTACCATAATCAAAACTAAGTTTAAATTCGGTTCTGGATTTAACAGGGAGAAAATGAAGAGAAGTCAGTATGGGAGAAATAT
NyeTARs.A013 CCTGTGAAGTCAGCATACTGTAG
   Consensus CCTGTGAAGTCAGCATACTGTAG...........................................................................................................
```

```
             1821 1830      1840      1850      1860      1870      1880      1890      1900      1910      1920      1930      1940      1950
             |--------+---------+---------+---------+---------+---------+---------+---------+---------+---------+---------+---------+---------|
contig035376 AAGAAGAGGGGAGGAGTTTAGAGAATGTGGTGTTGGGATCAGTTATATTACTAATTCTAGTCATAACTCTGCCAGAGCTGGAGCTCTCTGCAGGTCTGTGGAGATGGATACTCAGGATGGAGCAGAGCTC
NyeTARs.A014                                                                                                         ATGGATACTCAGGATGGAGCAGAGCTC
   Consensus .......................................................................................................ATGGATACTCAGGATGGAGCAGAGCTC

             1951 1960      1970      1980      1990      2000      2010      2020      2030      2040      2050      2060      2070      2080
             |--------+---------+---------+---------+---------+---------+---------+---------+---------+---------+---------+---------+---------|
contig035376 TGCTTTCCACAACTCTTCAACATCTCCTGCAAGAAACCTACAACTCCTCGGTCTCAAGTTTTGCTCCTTTATATTGTTGTGTCCTCAATGTCTTTGCTGACTGTGACTCTCAACCTGCTCGTCATCATTA
NyeTARs.A014 TGCTTTCCACAACTCTTCAACATCTCCTGCAAGAAACCTACAACTCCTCGGTCTCAAGTTTTGCTCCTTTATATTGTTGTGTCCTCAATGTCTTTGCTGACTGTGACTCTCAACCTGCTCGTCATCATTA
   Consensus TGCTTTCCACAACTCTTCAACATCTCCTGCAAGAAACCTACAACTCCTCGGTCTCAAGTTTTGCTCCTTTATATTGTTGTGTCCTCAATGTCTTTGCTGACTGTGACTCTCAACCTGCTCGTCATCATTA

             2081 2090      2100      2110      2120      2130      2140      2150      2160      2170      2180      2190      2200      2210
             |--------+---------+---------+---------+---------+---------+---------+---------+---------+---------+---------+---------+---------|
contig035376 CAGTCTCCCACTTCAGGCAGAGTTTCATATTTTTTACCTGCTGCTGTGGCTTTACAAGTTTATACCTGATTTGTTATATCTGTTTGAATACTTTCTTTCTGTTGACTTTTTTTTAAATTATATTTTCCT
NyeTARs.A014 CAGTCTCCCACTTCAGGCAG--------------------------------------------------------------------------------------------------------------
   Consensus CAGTCTCCCACTTCAGGCAG..............................................................................................................

             2211 2220      2230      2240      2250      2260      2270      2280      2290      2300      2310      2320      2330      2340
             |--------+---------+---------+---------+---------+---------+---------+---------+---------+---------+---------+---------+---------|
contig035376 ATCTTTCTAGGCAGCTCCACACACCCACTAACATCCTGCTCCTTTCTCTGGCTGTCACTGACTTTCTCGTTGGTCTTCTGTTTATGCCTGGAGAAATCCTGCGAAATACAGCGTGTTGGTTTCTTGGTCA
NyeTARs.A014 --------------CTCCACACACCCACTAACATCCTGCTCCTTTCTCTGGCTGTCACTGACTTTCTCGTTGGTCTTCTGTTTATGCCTGGAGAAATCCTGCGAAATACAGCGTGTTGGTTTCTTGGTCA
   Consensus ..............CTCCACACACCCACTAACATCCTGCTCCTTTCTCTGGCTGTCACTGACTTTCTCGTTGGTCTTCTGTTTATGCCTGGAGAAATCCTGCGAAATACAGCGTGTTGGTTTCTTGGTCA

             2341 2350      2360      2370      2380      2390      2400      2410      2420      2430      2440      2450      2460      2470
             |--------+---------+---------+---------+---------+---------+---------+---------+---------+---------+---------+---------+---------|
contig035376 GCTCACATGTTCATTGTATAATTATGTATCCTACATCATTGCCTCTGCCTCAGTGGGCAACATGGTGCTGATATCAGTCGACCGCTATGTGGCTATTTGTGACCCTCTGCATTACCCCACCAGAATCACA
NyeTARs.A014 GCTCACATGTTCATTGTATAATTATGTATCCTACATCATTGCCTCTGCCTCAGTGGGCAACATGGTGCTGATATCAGTCGACCGCTATGTGGCTATTTGTGACCCTCTGCATTACCCCACCAGAATCACA
   Consensus GCTCACATGTTCATTGTATAATTATGTATCCTACATCATTGCCTCTGCCTCAGTGGGCAACATGGTGCTGATATCAGTCGACCGCTATGTGGCTATTTGTGACCCTCTGCATTACCCCACCAGAATCACA

             2471 2480      2490      2500      2510      2520      2530      2540      2550      2560      2570      2580      2590      2600
             |--------+---------+---------+---------+---------+---------+---------+---------+---------+---------+---------+---------+---------|
contig035376 GAGAGAAGAGTGAAACTCTGTGTCTGTCTGTGTTGGCTCTGCTCTGTTTTCTACAGCTATGTGATTTTAATAGATGATCTAAGTCAACCAGGCAAGCATAAATCTTGCTATGGAAGATGTATAATTTTCA
NyeTARs.A014 GAGAGAAGAGTGAAACTCTGTGTCTGTCTGTGTTGGCTCTGCTCTGTTTTCTACAGCTATGTGATTTTAATAGATGATCTAAGTCAACCAGGCAAGCATAAATCTTGCTATGGAAGATGTATAATTTTCA
   Consensus GAGAGAAGAGTGAAACTCTGTGTCTGTCTGTGTTGGCTCTGCTCTGTTTTCTACAGCTATGTGATTTTAATAGATGATCTAAGTCAACCAGGCAAGCATAAATCTTGCTATGGAAGATGTATAATTTTCA

             2601 2610      2620      2630      2640      2650      2660      2670      2680      2690      2700      2710      2720      2730
             |--------+---------+---------+---------+---------+---------+---------+---------+---------+---------+---------+---------+---------|
contig035376 TTGAATTTATTGCAGGATTTGTTGACCTTGTTTTAGCCTTTATTATTCCACTTACTGTCATCATAGTTCTGTATATGAGAGTGTTTGTGGTGGCTGTGTCTCAGGCCCGTGCCATGCGCTCTCAGGTTAC
NyeTARs.A014 TTGAATTTATTGCAGGATTTGTTGACCTTGTTTTAGCCTTTATTATTCCACTTACTGTCATCATAGTTCTGTATATGAGAGTGTTTGTGGTGGCTGTGTCTCAGGCCCGTGCCATGCGCTCTCAGGTTAC
   Consensus TTGAATTTATTGCAGGATTTGTTGACCTTGTTTTAGCCTTTATTATTCCACTTACTGTCATCATAGTTCTGTATATGAGAGTGTTTGTGGTGGCTGTGTCTCAGGCCCGTGCCATGCGCTCTCAGGTTAC

             2731 2740      2750      2760      2770      2780      2790      2800      2810      2820      2830      2840      2850      2860
             |--------+---------+---------+---------+---------+---------+---------+---------+---------+---------+---------+---------+---------|
contig035376 AGCTGTCACACTGCAGCTCTCAGTGACTCTAACAGCAACTAAATCAGAGTTAAAAGCAGCCAGGACTCTGGGTGTTCTTGTTCTTGTGTTTCTATTATGTTTCTGCTCATATTACATTATTTCACTTTTT
NyeTARs.A014 AGCTGTCACACTGCAGCTCTCAGTGACTCTAACAGCAACTAAATCAGAGTTAAAAGCAGCCAGGACTCTGGGTGTTCTTGTTCTTGTGTTTCTATTATGTTTCTGCTCATATTACATTATTTCACTTTTT
   Consensus AGCTGTCACACTGCAGCTCTCAGTGACTCTAACAGCAACTAAATCAGAGTTAAAAGCAGCCAGGACTCTGGGTGTTCTTGTTCTTGTGTTTCTATTATGTTTCTGCTCATATTACATTATTTCACTTTTT

             2861 2870      2880      2890      2900      2910      2920      2930      2940      2950      2960      2970      2980      2990
             |--------+---------+---------+---------+---------+---------+---------+---------+---------+---------+---------+---------+---------|
contig035376 GGAAACGAGTTGCTCAATAGCTCATCTGCATCGATTGTGATCTATCTGTACTATTTTAACTCCTGTCTAAACCCTTTGATTTATGCTATGTTCTACCCCTGGTTTAGAAAAGCAGTGAAACTAATTGTGA
NyeTARs.A014 GGAAACGAGTTGCTCAATAGCTCATCTGCATCGATTGTGATCTATCTGTACTATTTTAACTCCTGTCTAAACCCTTTGATTTATGCTATGTTCTACCCCTGGTTTAGAAAAGCAGTGAAACTAATTGTGA
   Consensus GGAAACGAGTTGCTCAATAGCTCATCTGCATCGATTGTGATCTATCTGTACTATTTTAACTCCTGTCTAAACCCTTTGATTTATGCTATGTTCTACCCCTGGTTTAGAAAAGCAGTGAAACTAATTGTGA

             2991 3000      3010      3020      3030      3040      3050      3060      3070      3080      3090      3100      3110      3120
             |--------+---------+---------+---------+---------+---------+---------+---------+---------+---------+---------+---------+---------|
contig035376 CTCTACAGATACTGCAGCCTGGCTCCTGTGAGATCAGCATAATGTAGAAAAAAGAAATCTTTCCTTAATGTACCAAATGGTATACCAAATTACTTTCTCTATCAATAATGTACTATATTAATTTTAAGAC
NyeTARs.A014 CTCTACAGATACTGCAGCCTGGCTCCTGTGAGATCAGCATAATGTAG
   Consensus CTCTACAGATACTGCAGCCTGGCTCCTGTGAGATCAGCATAATGTAG...................................................................................
```

```
            3381 3390      3400      3410      3420      3430      3440      3450      3460      3470      3480      3490      3500      3510
contig035381 AACTCTGCCAGAGCTGGAGCTCTCTGCAGGTCTGTGGAGATGGATACTCAGGATGGAGCAGAGCTCTGCTTTCCACAACTCTTCAACATCTCCTGCAAGAAACCTACAACTCCTCTGTCTCAAGTTTTGC
NyeTARs.A015                                        ATGGATACTCAGGATGGAGCAGAGCTCTGCTTTCCACAACTCTTCAACATCTCCTGCAAGAAACCTACAACTCCTCTGTCTCAAGTTTTGC
   Consensus .......................................ATGGATACTCAGGATGGAGCAGAGCTCTGCTTTCCACAACTCTTCAACATCTCCTGCAAGAAACCTACAACTCCTCTGTCTCAAGTTTTGC

            3511 3520      3530      3540      3550      3560      3570      3580      3590      3600      3610      3620      3630      3640
contig035381 TCCTTTATATTGTTGTGTCCTCAATGTCTCTGCTGACTGTGACTCTCAACCTGCTCGTCATCATTGCAGTCTCCCACTTCAGGCAGAGTTTCATATTTTTTACCTGCTGCTGTGGCTTTACAAGTGTATA
NyeTARs.A015 TCCTTTATATTGTTGTGTCCTCAATGTCTCTGCTGACTGTGACTCTCAACCTGCTCGTCATCATTGCAGTCTCCCACTTCAGGCAG--------------------------------------------
   Consensus TCCTTTATATTGTTGTGTCCTCAATGTCTCTGCTGACTGTGACTCTCAACCTGCTCGTCATCATTGCAGTCTCCCACTTCAGGCAG............................................

            3641 3650      3660      3670      3680      3690      3700      3710      3720      3730      3740      3750      3760      3770
contig035381 CCTGATTTGTTATATCTGTTTGAATACTTTCTTTCTGTTGACTTTTTTTTTTAAATTATATTTTCCTGTCTTTCTAGGCAGCTCCACTCACCCACTAACATCCTGCTCCTTTCTCTGGCTGTCGCTGACT
NyeTARs.A015 ---------------------------------------------------------------------------------CTCCACTCACCCACTAACATCCTGCTCCTTTCTCTGGCTGTCGCTGACT
   Consensus .................................................................................CTCCACTCACCCACTAACATCCTGCTCCTTTCTCTGGCTGTCGCTGACT

            3771 3780      3790      3800      3810      3820      3830      3840      3850      3860      3870      3880      3890      3900
contig035381 TTCTCGTTGGCCTTCTGTTTATGCCTGGAGAAATCCTGCGAAATACAGCGTGTTGGTTTCTTGGTCAGCTCACATGTTCTCTGTATAATTATGTATCCTACATCATTACCTCTGCCTCAGTGGGCAACAT
NyeTARs.A015 TTCTCGTTGGCCTTCTGTTTATGCCTGGAGAAATCCTGCGAAATACAGCGTGTTGGTTTCTTGGTCAGCTCACATGTTCTCTGTATAATTATGTATCCTACATCATTACCTCTGCCTCAGTGGGCAACAT
   Consensus TTCTCGTTGGCCTTCTGTTTATGCCTGGAGAAATCCTGCGAAATACAGCGTGTTGGTTTCTTGGTCAGCTCACATGTTCTCTGTATAATTATGTATCCTACATCATTACCTCTGCCTCAGTGGGCAACAT

            3901 3910      3920      3930      3940      3950      3960      3970      3980      3990      4000      4010      4020      4030
contig035381 GGTGCTGATATCAGTCGACCGCTATGTGGCTATTTGTGACCCTCTGCATTACCCCACCAGAATCACAGAGAGAAGAGTGAAACTTTGTGTTTGTCTGTGTTGGCTCTGCTCTGTTTTCTACTGTTATGTG
NyeTARs.A015 GGTGCTGATATCAGTCGACCGCTATGTGGCTATTTGTGACCCTCTGCATTACCCCACCAGAATCACAGAGAGAAGAGTGAAACTTTGTGTTTGTCTGTGTTGGCTCTGCTCTGTTTTCTACTGTTATGTG
   Consensus GGTGCTGATATCAGTCGACCGCTATGTGGCTATTTGTGACCCTCTGCATTACCCCACCAGAATCACAGAGAGAAGAGTGAAACTTTGTGTTTGTCTGTGTTGGCTCTGCTCTGTTTTCTACTGTTATGTG

            4031 4040      4050      4060      4070      4080      4090      4100      4110      4120      4130      4140      4150      4160
contig035381 ATTTTAATAGATGATCTAAGTCAACCAGGCAAGCATAATTCTTGCTATGGAAAATGTATAATTATCATTGAATTCATTGCAGGATTTGTTGACCTTGTTTTATCCTTTATTATTCCACTTACTGTCATCA
NyeTARs.A015 ATTTTAATAGATGATCTAAGTCAACCAGGCAAGCATAATTCTTGCTATGGAAAATGTATAATTATCATTGAATTCATTGCAGGATTTGTTGACCTTGTTTTATCCTTTATTATTCCACTTACTGTCATCA
   Consensus ATTTTAATAGATGATCTAAGTCAACCAGGCAAGCATAATTCTTGCTATGGAAAATGTATAATTATCATTGAATTCATTGCAGGATTTGTTGACCTTGTTTTATCCTTTATTATTCCACTTACTGTCATCA

            4161 4170      4180      4190      4200      4210      4220      4230      4240      4250      4260      4270      4280      4290
contig035381 TAGTTCTGTACATGAGAGTGTTTGTGGTGGCTGTGTCTCAGGCCCGTGCCATGCGCTCTCAGGTTACAGCTGTCACACTGCAGCTCTCAGTGACTCTAACAGCAAAGAAATCAGAGTTAAAAGCAGCCAA
NyeTARs.A015 TAGTTCTGTACATGAGAGTGTTTGTGGTGGCTGTGTCTCAGGCCCGTGCCATGCGCTCTCAGGTTACAGCTGTCACACTGCAGCTCTCAGTGACTCTAACAGCAAAGAAATCAGAGTTAAAAGCAGCCAA
   Consensus TAGTTCTGTACATGAGAGTGTTTGTGGTGGCTGTGTCTCAGGCCCGTGCCATGCGCTCTCAGGTTACAGCTGTCACACTGCAGCTCTCAGTGACTCTAACAGCAAAGAAATCAGAGTTAAAAGCAGCCAA

            4291 4300      4310      4320      4330      4340      4350      4360      4370      4380      4390      4400      4410      4420
contig035381 AACTCTGGGTGTTCTTGTTCTTGTGTTTCTATTATGTTTCTGCTCATATTTCATTGTTTCACTTTTTGGAAACGAGTTGCTCAATAGCTCATCTGCATCGATTGTGATCTATCTGTACTATTTTAACTCC
NyeTARs.A015 AACTCTGGGTGTTCTTGTTCTTGTGTTTCTATTATGTTTCTGCTCATATTTCATTGTTTCACTTTTTGGAAACGAGTTGCTCAATAGCTCATCTGCATCGATTGTGATCTATCTGTACTATTTTAACTCC
   Consensus AACTCTGGGTGTTCTTGTTCTTGTGTTTCTATTATGTTTCTGCTCATATTTCATTGTTTCACTTTTTGGAAACGAGTTGCTCAATAGCTCATCTGCATCGATTGTGATCTATCTGTACTATTTTAACTCC

            4421 4430      4440      4450      4460      4470      4480      4490      4500      4510      4520      4530      4540      4550
contig035381 TGTCTAAACCCTTTGATTTATGCTATGTTCTACCCCTGGTTTAGAAAAGCAGTGAAACTAATTGTGACCCTACAGATACTGCAGCCTGGCTCCTGTGAGGTCATCATACTGTAGAAAAAAGAAATCTTTC
NyeTARs.A015 TGTCTAAACCCTTTGATTTATGCTATGTTCTACCCCTGGTTTAGAAAAGCAGTGAAACTAATTGTGACCCTACAGATACTGCAGCCTGGCTCCTGTGAGGTCATCATACTGTAG
   Consensus TGTCTAAACCCTTTGATTTATGCTATGTTCTACCCCTGGTTTAGAAAAGCAGTGAAACTAATTGTGACCCTACAGATACTGCAGCCTGGCTCCTGTGAGGTCATCATACTGTAG................
```

```
             2211 2220 2230 2240 2250 2260 2270 2280 2290 2300 2310 2320 2330 2340
contig046011 AGGAGGGATGGGACTGGTGGGTGAAGGACGAAATAGATAAATGAGCTCTGTGAGGAAAAGCTTTGATGAGATGCAGCAGCCTGATGATGGAGATACAGGAAGGACTAGAGCTCTGTTTTCCACAACTCCT
NyeTARs.A016                                                                                       ATGGAGATACAGGAAGGACTAGAGCTCTGTTTTCCACAACTCCT
Consensus    ......................................................................................ATGGAGATACAGGAAGGACTAGAGCTCTGTTTTCCACAACTCCT

             2341 2350 2360 2370 2380 2390 2400 2410 2420 2430 2440 2450 2460 2470
contig046011 CAACGATTCCTGCAGAAAGCCAACACTTCACTATTCCACAACTATGCTGCTGTATATTGTGATGTCATGCATCTCTCTGATCACTGCTGCTCTATCACTGCTGCTCTAAACCTTCTGGCCATCATCTCAG
NyeTARs.A016 CAACGATTCCTGCAGAAAGCCAACACTTCACTATTCCACAACTATGCTGCTGTATATTGTGATGTCATGC-----------------TGCTCTATCACTGCTGCTCTAAACCTTCTGGCCATCATCTCAG
Consensus    CAACGATTCCTGCAGAAAGCCAACACTTCACTATTCCACAACTATGCTGCTGTATATTGTGATGTCATGC.................TGCTCTATCACTGCTGCTCTAAACCTTCTGGCCATCATCTCAG

             2471 2480 2490 2500 2510 2520 2530 2540 2550 2560 2570 2580 2590 2600
contig046011 TCTCCCACTTCAGGCAGAGACCAACTTTTAAATGAACTTACATTAAGGTTTAGATATATGAAAAGTGAGATTTGTATCATTGGAATTAGCAAAGAATACATTAAGTGAAACTAATAAATGTCTTTGGCTC
NyeTARs.A016 TCTCCCACTTCAG---------------------------------------------------------------------------------------------------------------------
Consensus    TCTCCCACTTCAG.....................................................................................................................

             2601 2610 2620 2630 2640 2650 2660 2670 2680 2690 2700 2710 2720 2730
contig046011 AACCATGACTCTTAAGTTGTGTAAGGTTCACCGTGTCTTAATAAGGAAACAATTTCACTAAATACATTTTGACATAATTAACATATGACATAATCTCAACTATCTTATCACTATCATTTCGCTGCCCCAC
NyeTARs.A016 -------------------------------------------------------------------------------------------------AACTATCTTATCACTATCATTTCGCTGCCCCAC
Consensus    .................................................................................................AACTATCTTATCACTATCATTTCGCTGCCCCAC

             2731 2740 2750 2760 2770 2780 2790 2800 2810 2820 2830 2840 2850 2860
contig046011 CTTGGGCATAGATAAACCTCAGCTGAAAATTTTAAAAAGCACCACCTGCTGGATACTTGATGATTTTAATTGTTTTCTTTATTGGTATCTGACTGGCAACATTACCTGTGCTTCAATAGGGAACATAGTT
NyeTARs.A016 CTTGGGCATAGATAAACCTCAGCTGAAAATTTTAAAAAGCACCACCTGCTGGATACTTGATGATTTTAATTGTTTTCTTTATTGGTATCTGACTGGCAACATTACCTGTGCTTCAATAGGGAACATAGTT
Consensus    CTTGGGCATAGATAAACCTCAGCTGAAAATTTTAAAAAGCACCACCTGCTGGATACTTGATGATTTTAATTGTTTTCTTTATTGGTATCTGACTGGCAACATTACCTGTGCTTCAATAGGGAACATAGTT

             2861 2870 2880 2890 2900 2910 2920 2930 2940 2950 2960 2970 2980 2990
contig046011 CTAATATCAGTCGACCGCTATGTGGCTATTTGTGACCCTTTACATTACTCCACCAGAATTACTGTGGCAAAAGTTAAACTCAGTGTTTGTCTGTGTTGGTTTTATGCTATTTCCAGCATTAGTCTTTATG
NyeTARs.A016 CTAATATCAGTCGACCGCTATGTGGCTATTTGTGACCCTTTACATTACTCCACCAGAATTACTGTGGCAAAAGTTAAACTCAGTGTTTGTCTGTGTTGGTTTTATGCTATTTCCAGCATTAGTCTTTATG
Consensus    CTAATATCAGTCGACCGCTATGTGGCTATTTGTGACCCTTTACATTACTCCACCAGAATTACTGTGGCAAAAGTTAAACTCAGTGTTTGTCTGTGTTGGTTTTATGCTATTTCCAGCATTAGTCTTTATG

             2991 3000 3010 3020 3030 3040 3050 3060 3070 3080 3090 3100 3110 3120
contig046011 CAAAAGACTACCTGATTGAACCAGGCAGGTATAATTCCTGTTATGGAGAGTGTGTCATTAATGATATTGCAAGGACTATTGACCTTGTTTTATCCTTTATATTTCCAATTACTGCCATCATAGTTCTGTA
NyeTARs.A016 CAAAAGACTACCTGATTGAACCAGGCAGGTATAATTCCTGTTATGGAGAGTGTGTCATTAATGATATTGCAAGGACTATTGACCTTGTTTTATCCTTTATATTTCCAATTACTGCCATCATAGTTCTGTA
Consensus    CAAAAGACTACCTGATTGAACCAGGCAGGTATAATTCCTGTTATGGAGAGTGTGTCATTAATGATATTGCAAGGACTATTGACCTTGTTTTATCCTTTATATTTCCAATTACTGCCATCATAGTTCTGTA

             3121 3130 3140 3150 3160 3170 3180 3190 3200 3210 3220 3230 3240 3250
contig046011 TATGAGAGTATTTGTGGTGGCTGTGTCTCAGGCTCATGCCATGCGCTCTCAGGTTACAGCTGTCACAATTCATTGTTCTCTGAATCAAGCAAACAAATCAGAGTTGAAAGCAGCCAGGACACTTGGAGTT
NyeTARs.A016 TATGAGAGTATTTGTGGTGGCTGTGTCTCAGGCTCATGCCATGCGCTCTCAGGTTACAGCTGTCACAATTCATTGTTCTCTGAATCAAGCAAACAAATCAGAGTTGAAAGCAGCCAGGACACTTGGAGTT
Consensus    TATGAGAGTATTTGTGGTGGCTGTGTCTCAGGCTCATGCCATGCGCTCTCAGGTTACAGCTGTCACAATTCATTGTTCTCTGAATCAAGCAAACAAATCAGAGTTGAAAGCAGCCAGGACACTTGGAGTT

             3251 3260 3270 3280 3290 3300 3310 3320 3330 3340 3350 3360 3370 3380
contig046011 CTTGTAGTTGTGTTTCTACTGTGTTTCTGCCCATATTACTGTGTCTCTCTTATAAGGGAGGACTTTAGCTCAGTAGCTCCTGTAATGTTTGCATGCATCAAACATTAATGATGACACCTTCAGTATTTCT
NyeTARs.A016 CTTGTAGTTGTGTTTCTACTGTGTTTCTGCCCATATTACTGTGTCTCTCTTATAAGGGAGGACTTTAGCTCAGTAGCTCCT-------------------------------------------------
Consensus    CTTGTAGTTGTGTTTCTACTGTGTTTCTGCCCATATTACTGTGTCTCTCTTATAAGGGAGGACTTTAGCTCAGTAGCTCCT.................................................

             3381 3390 3400 3410 3420 3430 3440 3450 3460 3470 3480 3490 3500 3510
contig046011 CAGTTCCATTCTTTAATTCGCAGGCTTGCAGCAGCAACACAGTCTGCAGCGCTAACTTGTTCATCTACCGTTTTTCATTGTCCCTCTTTTCTATCCTCTCCTTCCGCCTAACATTCGACCTTTCAAAATA
NyeTARs.A016 ----------------------------------------------------------------------------------------------------------------------------------
Consensus    ..................................................................................................................................

             3511 3520 3530 3540 3550 3560 3570 3580 3590 3600 3610 3620 3630 3640
contig046011 AAATCTCTGTAAACATCACAGCGCTCATTGTGATCAGTCTGTTCTTTTCAAACTCCTGTTTAAACCCAGTGATCTATGCCTTGTTCTATCCTTTTTTTCATAAAAGCTTTGAAACTAATTGTGACTTTAC
NyeTARs.A016 --------------------------ATTGTGATCAGTCTGTTCTTTTCAAACTCCTGTTTAAACCCAGTGATCTATGCCTTGTTCTATCCT----------AAAGCTTTGAAACTAATTGTGACTTTAC
Consensus    ..........................ATTGTGATCAGTCTGTTCTTTTCAAACTCCTGTTTAAACCCAGTGATCTATGCCTTGTTCTATCCT..........AAAGCTTTGAAACTAATTGTGACTTTAC

             3641 3650 3660 3670 3680 3690 3700 3710 3720 3730 3740 3750 3760 3770
contig046011 AGATACTGCATTTGTTGTAGAATATGTAAACAGTAATTCATTGTGACAGGAATCACACCCACTTTTACGCTTTTCACAAACTTTATTTCTTATTGTTACTGTTTACACACTGGTTGCAGCTTTGCAGCTC
NyeTARs.A016 AGATACTGCATTTGTTGTAG
Consensus    AGATACTGCATTTGTTGTAG..............................................................................................................
```

```
             521      530       540       550       560       570       580       590       600       610       620       630       640       650
             |--------+---------+---------+---------+---------+---------+---------+---------+---------+---------+---------+---------+---------|
contig046007 GCGGCTGTCTGATGGTGGAGATGCAGAAAGAAAACAAGTTGTGTTTTCCACAACTCCTCAACAGCTCCTGCAGGAAGCCAACACTTCACTGGTCCAAAGATGTGCTCCTGAACACTGTGCTGTCCTGCAT
NyeTARs.A017                     ATGCAGAAAGAAAACAAGTTGTGTTTTCCACAACTCCTCAACAGCTCCTGCAGGAAGCCAACACTTCACTGGTCCAAAGATGTGCTCCTGAACACTGTGCTGTCCTGCAT
   Consensus ....................ATGCAGAAAGAAAACAAGTTGTGTTTTCCACAACTCCTCAACAGCTCCTGCAGGAAGCCAACACTTCACTGGTCCAAAGATGTGCTCCTGAACACTGTGCTGTCCTGCAT

             651      660       670       680       690       700       710       720       730       740       750       760       770       780
             |--------+---------+---------+---------+---------+---------+---------+---------+---------+---------+---------+---------+---------|
contig046007 CTCTCTGCTCACCGCTGCTCTAAACATTCTCGTCATCATCTCAGTCTCTTACTTCAGGCAGAGATTAATTTTAAATATATGACAACTTTAATGTACATTATATCACTTAAGTGAATTTTGCTTGATGTAT
NyeTARs.A017 CTCTCTGCTCACCGCTGCTCTAAACATTCTCGTCATCATCTCAGTCTCTTACTTCAGG------------------------------------------------------------------------
   Consensus CTCTCTGCTCACCGCTGCTCTAAACATTCTCGTCATCATCTCAGTCTCTTACTTCAGG........................................................................

             781      790       800       810       820       830       840       850       860       870       880       890       900       910
             |--------+---------+---------+---------+---------+---------+---------+---------+---------+---------+---------+---------+---------|
contig046007 TTTAACCATGATTGTAATGTCCTGTTTGCCTAGACTGAATCAAGCCTTACTAATGCAGCATTTTCAATGAAGACAGTTTGACATTAATAATGTGCATTAAATTCTTTTGTGTCTCTGCAGGAAGCTTCAC
NyeTARs.A017 -------------------------------------------------------------------------------------------------------------------------AAGCTTCAC
   Consensus .........................................................................................................................AAGCTTCAC

             911      920       930       940       950       960       970       980       990       1000      1010      1020      1030      1040
             |--------+---------+---------+---------+---------+---------+---------+---------+---------+---------+---------+---------+---------|
contig046007 ACACCCAGTAATATCCTTCTCCTTTCTCTGGCTGTCTCAGACTTTCTTGTCGGTCTCCTGTTGATGCCATTAGGAATCCTTAGAAACACAGGCTGCTGGGTACTTGGTGATATAATTTGTTCTCTTTATT
NyeTARs.A017 ACACCCAGTAATATCCTTCTCCTTTCTCTGGCTGTCTCAGACTTTCTTGTCGGTCTCCTGTTGATGCCATTAGGAATCCTTAGAAACACAGGCTGCTGGGTACTTGGTGATATAATTTGTTCTCTTTATT
   Consensus ACACCCAGTAATATCCTTCTCCTTTCTCTGGCTGTCTCAGACTTTCTTGTCGGTCTCCTGTTGATGCCATTAGGAATCCTTAGAAACACAGGCTGCTGGGTACTTGGTGATATAATTTGTTCTCTTTATT

             1041     1050      1060      1070      1080      1090      1100      1110      1120      1130      1140      1150      1160      1170
             |--------+---------+---------+---------+---------+---------+---------+---------+---------+---------+---------+---------+---------|
contig046007 GGTATTTAACCAGCAACATTGTCTGTGCTTCAATAGGGAACATTGTCCTAATATCAGTTGACCGTTATGTGGCTATTTGTGACCCTCTGCATTATCCCAGCAGAATTACTTTGGCGAAAGTCAAACTCAG
NyeTARs.A017 GGTATTTAACCAGCAACATTGTCTGTGCTTCAATAGGGAACATTGTCCTAATATCAGTTGACCGTTATGTGGCTATTTGTGACCCTCTGCATTATCCCAGCAGAATTACTTTGGCGAAAGTCAAACTCAG
   Consensus GGTATTTAACCAGCAACATTGTCTGTGCTTCAATAGGGAACATTGTCCTAATATCAGTTGACCGTTATGTGGCTATTTGTGACCCTCTGCATTATCCCAGCAGAATTACTTTGGCGAAAGTCAAACTCAG

             1171     1180      1190      1200      1210      1220      1230      1240      1250      1260      1270      1280      1290      1300
             |--------+---------+---------+---------+---------+---------+---------+---------+---------+---------+---------+---------+---------|
contig046007 TGTTTGTCTGTGTTGGTTTTATGCTTTTTTCTACGTCAGTCTTTATACAAAGGATATCCTACTTGAACCAGGCAGGGATAATTCCTGCTACGGAGAGTGTGTATTTGTCATCAATGATATTGCTGTTGTT
NyeTARs.A017 TGTTTGTCTGTGTTGGTTTTATGCTTTTTTCTACGTCAGTCTTTATACAAAGGATATCCTACTTGAACCAGGCAGGGATAATTCCTGCTACGGAGAGTGTGTATTTGTCATCAATGATATTGCTGTTGTT
   Consensus TGTTTGTCTGTGTTGGTTTTATGCTTTTTTCTACGTCAGTCTTTATACAAAGGATATCCTACTTGAACCAGGCAGGGATAATTCCTGCTACGGAGAGTGTGTATTTGTCATCAATGATATTGCTGTTGTT

             1301     1310      1320      1330      1340      1350      1360      1370      1380      1390      1400      1410      1420      1430
             |--------+---------+---------+---------+---------+---------+---------+---------+---------+---------+---------+---------+---------|
contig046007 GTTGACCTTGTTTTTTCCTTTATTGTTCCGGTTTCTGTCATTGTAGTTCTGTATATCAGAGTATTTGTGGCAGCTGTGTCTCAGGCTCGTGCCATGCGCTCTCATGTGACATCTGTCACACTGCAGCGCT
NyeTARs.A017 GTTGACCTTGTTTTTTCCTTTATTGTTCCGGTTTCTGTCATTGTAGTTCTGTATATCAGAGTATTTGTGGCAGCTGTGTCTCAGGCTCGTGCCATGCGCTCTCATGTGACATCTGTCACACTGCAGCGCT
   Consensus GTTGACCTTGTTTTTTCCTTTATTGTTCCGGTTTCTGTCATTGTAGTTCTGTATATCAGAGTATTTGTGGCAGCTGTGTCTCAGGCTCGTGCCATGCGCTCTCATGTGACATCTGTCACACTGCAGCGCT

             1431     1440      1450      1460      1470      1480      1490      1500      1510      1520      1530      1540      1550      1560
             |--------+---------+---------+---------+---------+---------+---------+---------+---------+---------+---------+---------+---------|
contig046007 CACTGAATCAAACAAACAAATCTGAGCTGAAAGCAGCCAGGACTCTTGGAATTCTTGTAGTTGTGTTTCTGGCATGCTTCTGTCCACTCTACTACTTTTCTCTTGTTGATGAAAATGCAATCAATGATCC
NyeTARs.A017 CACTGAATCAAACAAACAAATCTGAGCTGAAAGCAGCCAGGACTCTTGGAATTCTTGTAGTTGTGTTTCTGGCATGCTTCTGTCCACTCTACTACTTTTCTCTTGTTGATGAAAATGCAATCAATGATCC
   Consensus CACTGAATCAAACAAACAAATCTGAGCTGAAAGCAGCCAGGACTCTTGGAATTCTTGTAGTTGTGTTTCTGGCATGCTTCTGTCCACTCTACTACTTTTCTCTTGTTGATGAAAATGCAATCAATGATCC

             1561     1570      1580      1590      1600      1610      1620      1630      1640      1650      1660      1670      1680      1690
             |--------+---------+---------+---------+---------+---------+---------+---------+---------+---------+---------+---------+---------|
contig046007 AGCTGCATCTTTTGTGGTCATTATATTTTACTTTAACTCGTGTCTAAATCCTTTGATCTATGCCTTGTTTTACCCCTGGTTTAGAAATGCTGTTAAGCTTATCATCACTCTGCGGATTTTCAAGTATAAC
NyeTARs.A017 AGCTGCATCTTTTGTGGTCATTATATTTTACTTTAACTCGTGTCTAAATCCTTTGATCTATGCCTTGTTTTACCCCTGGTTTAGAAATGCTGTTAAGCTTATCATCACTCTGCGGATTTTCAAGTATAAC
   Consensus AGCTGCATCTTTTGTGGTCATTATATTTTACTTTAACTCGTGTCTAAATCCTTTGATCTATGCCTTGTTTTACCCCTGGTTTAGAAATGCTGTTAAGCTTATCATCACTCTGCGGATTTTCAAGTATAAC

             1691     1700      1710      1720      1730      1740      1750      1760      1770      1780      1790      1800      1810      1820
             |--------+---------+---------+---------+---------+---------+---------+---------+---------+---------+---------+---------+---------|
contig046007 ATGAGTGAGGCCAACATACTATAAAAAGAGCATGAAACTTGCTCTACTAAGATATAAAATCAGTTGTGCAATGTTTTGCGCTGTCACCCCACAGCAAGAAAACCCCGGGGTCTTTCTGTGTTTAGTTTCT
NyeTARs.A017 ATGAGTGAGGCCAACATACTATAA
   Consensus ATGAGTGAGGCCAACATACTATAA..........................................................................................................
```

```
           1041 1050      1060      1070      1080      1090      1100      1110      1120      1130      1140      1150      1160      1170
           |--------+---------+---------+---------+---------+---------+---------+---------+---------+---------+---------+---------+---------|
contig060105 AGGAAAGGTGGTGATACAAAAAAGGGGCTCAGTCACAAAGCTGTTTGTGCTGAACAGCTGTCTTTCTCTAACAATGGAAGAAACTGAACTCTGCTTTCCACAACTATTCAACTCCTCTTGTGTGCGGCAA
NyeTARs.A018                                                                          ATGGAAGAAACTGAACTCTGCTTTCCACAACTATTCAACTCCTCTTGTGTGCGGCAA
   Consensus .........................................................................ATGGAAGAAACTGAACTCTGCTTTCCACAACTATTCAACTCCTCTTGTGTGCGGCAA

           1171 1180      1190      1200      1210      1220      1230      1240      1250      1260      1270      1280      1290      1300
           |--------+---------+---------+---------+---------+---------+---------+---------+---------+---------+---------+---------+---------|
contig060105 AAGCATTCACAAATTGAGGCTGTGTTTATTTACACTCCACTCTCTTCCATCTCTGTGCTTACTACAATTCTGAACCTGCTTGTGATCATCTCCATCGCTCACTTCAAGTACTGACATATTTGAATACTTA
NyeTARs.A018 AAGCATTCACAAATTGAGGCTGTGTTTATTTACACTCCACTCTCTTCCATCTCTGTGCTTACTACAATTCTGAACCTGCTTGTGATCATCTCCATCGCTCACTTCAAG----------------------
   Consensus AAGCATTCACAAATTGAGGCTGTGTTTATTTACACTCCACTCTCTTCCATCTCTGTGCTTACTACAATTCTGAACCTGCTTGTGATCATCTCCATCGCTCACTTCAAG......................

           1301 1310      1320      1330      1340      1350      1360      1370      1380      1390      1400      1410      1420      1430
           |--------+---------+---------+---------+---------+---------+---------+---------+---------+---------+---------+---------+---------|
contig060105 ACAAGTTATCGAAAAATAATGTTTTTTGTGAATTTAATTAGAAAATGTGTTGCTTTGTTCTATATGTTACAGTGGAGTTGCAATTACTGCTGTAGAACAACCTTTGATGCTCCTATTTTAATCCAGGCAG
NyeTARs.A018 -------------------------------------------------------------------------------------------------------------------------------CAG
   Consensus ...............................................................................................................................CAG

           1431 1440      1450      1460      1470      1480      1490      1500      1510      1520      1530      1540      1550      1560
           |--------+---------+---------+---------+---------+---------+---------+---------+---------+---------+---------+---------+---------|
contig060105 CTGCACACCCCAACCAACCTCCTCCTCCTCCTCTCTCTGGCCGTCTCAGATTTCTTCGTGGGCCTCATCATGGCTTGTCAGATTAGCCTCCTAGATGGCTGCTGGTTTCTTGGTGACCACATGTGTGCTC
NyeTARs.A018 CTGCACACCCCAACCAACCTCCTCCTCCTCCTCTCTCTGGCCGTCTCAGATTTCTTCGTGGGCCTCATCATGGCTTGTCAGATTAGCCTCCTAGATGGCTGCTGGTTTCTTGGTGACCACATGTGTGCTC
   Consensus CTGCACACCCCAACCAACCTCCTCCTCCTCCTCTCTCTGGCCGTCTCAGATTTCTTCGTGGGCCTCATCATGGCTTGTCAGATTAGCCTCCTAGATGGCTGCTGGTTTCTTGGTGACCACATGTGTGCTC

           1561 1570      1580      1590      1600      1610      1620      1630      1640      1650      1660      1670      1680      1690
           |--------+---------+---------+---------+---------+---------+---------+---------+---------+---------+---------+---------+---------|
contig060105 TGTATAGCAGTTTAGATTACATTGTTACTTCTGCTTCAGTAGGAACTATGGTACTCATATCAGCTGACCGCTATGTAGCCATTTGTGACCCTCTGCATTATCCCACCAAAATTACTATAAAAAGAGTCTC
NyeTARs.A018 TGTATAGCAGTTTAGATTACATTGTTACTTCTGCTTCAGTAGGAACTATGGTACTCATATCAGCTGACCGCTATGTAGCCATTTGTGACCCTCTGCATTATCCCACCAAAATTACTATAAAAAGAGTCTC
   Consensus TGTATAGCAGTTTAGATTACATTGTTACTTCTGCTTCAGTAGGAACTATGGTACTCATATCAGCTGACCGCTATGTAGCCATTTGTGACCCTCTGCATTATCCCACCAAAATTACTATAAAAAGAGTCTC

           1691 1700      1710      1720      1730      1740      1750      1760      1770      1780      1790      1800      1810      1820
           |--------+---------+---------+---------+---------+---------+---------+---------+---------+---------+---------+---------+---------|
contig060105 AGTCTCTATTTGTACTTGCTGGGCTTGTTCAATTCTGTATAACAGTCTTATCATGAAGGATAATTTCAAGCAGCCAGGGAGGTATAATTCTTGTTCTGGTGATTGTGTAGTTGTCATTGACTACTTTGTG
NyeTARs.A018 AGTCTCTATTTGTACTTGCTGGGCTTGTTCAATTCTGTATAACAGTCTTATCATGAAGGATAATTTCAAGCAGCCAGGGAGGTATAATTCTTGTTCTGGTGATTGTGTAGTTGTCATTGACTACTTTGTG
   Consensus AGTCTCTATTTGTACTTGCTGGGCTTGTTCAATTCTGTATAACAGTCTTATCATGAAGGATAATTTCAAGCAGCCAGGGAGGTATAATTCTTGTTCTGGTGATTGTGTAGTTGTCATTGACTACTTTGTG

           1821 1830      1840      1850      1860      1870      1880      1890      1900      1910      1920      1930      1940      1950
           |--------+---------+---------+---------+---------+---------+---------+---------+---------+---------+---------+---------+---------|
contig060105 GGAATTTTTGACTTTGTTTTGACCTTTGTCGGCCCTGTGATTGTCATCATAGTTCTGTATCTAAGAGTATTTGTGGTGGCTGTGTCTCAGGCTCGGGCAATGCGCTCTCATATCACTGCTCTCAGACTCC
NyeTARs.A018 GGAATTTTTGACTTTGTTTTGACCTTTGTCGGCCCTGTGATTGTCATCATAGTTCTGTATCTAAGAGTATTTGTGGTGGCTGTGTCTCAGGCTCGGGCAATGCGCTCTCATATCACTGCTCTCAGACTCC
   Consensus GGAATTTTTGACTTTGTTTTGACCTTTGTCGGCCCTGTGATTGTCATCATAGTTCTGTATCTAAGAGTATTTGTGGTGGCTGTGTCTCAGGCTCGGGCAATGCGCTCTCATATCACTGCTCTCAGACTCC

           1951 1960      1970      1980      1990      2000      2010      2020      2030      2040      2050      2060      2070      2080
           |--------+---------+---------+---------+---------+---------+---------+---------+---------+---------+---------+---------+---------|
contig060105 AGGGGTCAGAGACGGTGCATGCTAAGAAATCTGAGCTGAAAGCTGCCAGGACTCTCGGTGTACTTGTTATTGCCTTTCTGATATGTCTTTTCCCATTTTTCTGTTCCTCAATGGTAGGCCAGAATAGTTT
NyeTARs.A018 AGGGGTCAGAGACGGTGCATGCTAAGAAATCTGAGCTGAAAGCTGCCAGGACTCTCGGTGTACTTGTTATTGCCTTTCTGATATGTCTTTTCCCATTTTTCTGTTCCTCAATGGTAGGCCAGAATAGTTT
   Consensus AGGGGTCAGAGACGGTGCATGCTAAGAAATCTGAGCTGAAAGCTGCCAGGACTCTCGGTGTACTTGTTATTGCCTTTCTGATATGTCTTTTCCCATTTTTCTGTTCCTCAATGGTAGGCCAGAATAGTTT

           2081 2090      2100      2110      2120      2130      2140      2150      2160      2170      2180      2190      2200      2210
           |--------+---------+---------+---------+---------+---------+---------+---------+---------+---------+---------+---------+---------|
contig060105 CTTTGATATTAGATCTGTACCTTTTGAGCGCTTGCTGTTCTATTTTAACTCTTGTCTGAACCCATTAATCTATACTTTTTGCTACCCCTGGTTTCTGAAATCTATCAAGCTCATTGTGACATTTAAGATA
NyeTARs.A018 CTTTGATATTAGATCTGTACCTTTTGAGCGCTTGCTGTTCTATTTTAACTCTTGTCTGAACCCATTAATCTATACTTTTTGCTACCCCTGGTTTCTGAAATCTATCAAGCTCATTGTGACATTTAAGATA
   Consensus CTTTGATATTAGATCTGTACCTTTTGAGCGCTTGCTGTTCTATTTTAACTCTTGTCTGAACCCATTAATCTATACTTTTTGCTACCCCTGGTTTCTGAAATCTATCAAGCTCATTGTGACATTTAAGATA

           2211 2220      2230      2240      2250      2260      2270      2280      2290      2300      2310      2320      2330      2340
           |--------+---------+---------+---------+---------+---------+---------+---------+---------+---------+---------+---------+---------|
contig060105 TTTAGGCATGGCTCCAGTGAGGCCAGTGTACTATAGGGAAAAGTGATTTTAGTGATGAGCAAGGCATACAGTCTTTGACACTGAAAAACTGATAAATGATTATTCATTTCAAGTGTGATAAAACATCTGC
NyeTARs.A018 TTTAGGCATGGCTCCAGTGAGGCCAGTGTACTATAG
   Consensus TTTAGGCATGGCTCCAGTGAGGCCAGTGTACTATAG..............................................................................................
```

```
            1041    1050      1060      1070      1080      1090      1100      1110      1120      1130      1140      1150      1160      1170
            |--------+---------+---------+---------+---------+---------+---------+---------+---------+---------+---------+---------+---------|
contig046010 GATAAATCCCCTATGTGACAAGAAGCTCTGATCAGAGAGCTGCAGCTGTATGATTTTGGAGATGCAGAAAGAAGCCGAGCTTTGTTTTCCACAACTCCTCAACAGCTCCTGCAGGAAGCCAACATTTCAC
NyeTARs.A019                                                              ATGCAGAAAGAAGCCGAGCTTTGTTTTCCACAACTCCTCAACAGCTCCTGCAGGAAGCCAACATTTCAC
   Consensus .............................................................ATGCAGAAAGAAGCCGAGCTTTGTTTTCCACAACTCCTCAACAGCTCCTGCAGGAAGCCAACATTTCAC

            1171    1180      1190      1200      1210      1220      1230      1240      1250      1260      1270      1280      1290      1300
            |--------+---------+---------+---------+---------+---------+---------+---------+---------+---------+---------+---------+---------|
contig046010 TGGTCCAAAGCTGTGCTGCTGAACATTGTGCTGTGCTGCATCTCTCTGCTCACTGCTGCTCTAAACCTTCTCGTCATCATCTCAGTCTCTTACTTCAGGCAGAAATTAACTTCTCAGCTGAAATTGTAGT
NyeTARs.A019 TGGTCCAAAGCTGTGCTGCTGAACATTGTGCTGTGCTGCATCTCTCTGCTCACTGCTGCTCTAAACCTTCTCGTCATCATCTCAGTCTCTTACTTCAGGCAG----------------------------
   Consensus TGGTCCAAAGCTGTGCTGCTGAACATTGTGCTGTGCTGCATCTCTCTGCTCACTGCTGCTCTAAACCTTCTCGTCATCATCTCAGTCTCTTACTTCAGGCAG............................

            1301    1310      1320      1330      1340      1350      1360      1370      1380      1390      1400      1410      1420      1430
            |--------+---------+---------+---------+---------+---------+---------+---------+---------+---------+---------+---------+---------|
contig046010 TTAAGTCTTAAAAACCAATGGAATTAGTGAAGACATTTTTACATTATTAATGTGTAATATATTCTTTTTTTTTCTCTCCTTAGGCAGCTTCACACACCCACTAACATCCTCCTCCTTTCTCTGGCTGTCT
NyeTARs.A019 ---------------------------------------------------------------------------------------CTTCACACACCCACTAACATCCTCCTCCTTTCTCTGGCTGTCT
   Consensus .......................................................................................CTTCACACACCCACTAACATCCTCCTCCTTTCTCTGGCTGTCT

            1431    1440      1450      1460      1470      1480      1490      1500      1510      1520      1530      1540      1550      1560
            |--------+---------+---------+---------+---------+---------+---------+---------+---------+---------+---------+---------+---------|
contig046010 CAGACTTTCTCACTGGTCTCCTTATGCTGCCACTAGAAATAATTAGAAACACGGCCTGCTGGGTACTTGGTGATCTTATGTGTTCTGTTTATACTTATCTGACAGCCGGTCTTTTCTGTGCTTCAACTTG
NyeTARs.A019 CAGACTTTCTCACTGGTCTCCTTATGCTGCCACTAGAAATAATTAGAAACACGGCCTGCTGGGTACTTGGTGATCTTATGTGTTCTGTTTATACTTATCTGACAGCCGGTCTTTTCTGTGCTTCAACTTG
   Consensus CAGACTTTCTCACTGGTCTCCTTATGCTGCCACTAGAAATAATTAGAAACACGGCCTGCTGGGTACTTGGTGATCTTATGTGTTCTGTTTATACTTATCTGACAGCCGGTCTTTTCTGTGCTTCAACTTG

            1561    1570      1580      1590      1600      1610      1620      1630      1640      1650      1660      1670      1680      1690
            |--------+---------+---------+---------+---------+---------+---------+---------+---------+---------+---------+---------+---------|
contig046010 GAACATAGTTCTCATTTCAGTCGACCGCTATGTAGCTATTTGTCACCCTCTTCATTACCCCACCAGAATTACCGTGGCAAGAGTCAAACTCTGTGTTTGTTTGTGTTGGTTTTGTTCAACCTTCTACGGC
NyeTARs.A019 GAACATAGTTCTCATTTCAGTCGACCGCTATGTAGCTATTTGTCACCCTCTTCATTACCCCACCAGAATTACCGTGGCAAGAGTCAAACTCTGTGTTTGTTTGTGTTGGTTTTGTTCAACCTTCTACGGC
   Consensus GAACATAGTTCTCATTTCAGTCGACCGCTATGTAGCTATTTGTCACCCTCTTCATTACCCCACCAGAATTACCGTGGCAAGAGTCAAACTCTGTGTTTGTTTGTGTTGGTTTTGTTCAACCTTCTACGGC

            1691    1700      1710      1720      1730      1740      1750      1760      1770      1780      1790      1800      1810      1820
            |--------+---------+---------+---------+---------+---------+---------+---------+---------+---------+---------+---------+---------|
contig046010 AGTCTTTTAACAAAGGATGTTCTAATTAAGCCGGCCAGGTATAATTCCTGTTATGGAGAGTGTGTGTTTGTCATTAATGATATTGCAGGGTTTATTGATCTTGTGTTATCTTTTATTTTTCCAGTATCTG
NyeTARs.A019 AGTCTTTTAACAAAGGATGTTCTAATTAAGCCGGCCAGGTATAATTCCTGTTATGGAGAGTGTGTGTTTGTCATTAATGATATTGCAGGGTTTATTGATCTTGTGTTATCTTTTATTTTTCCAGTATCTG
   Consensus AGTCTTTTAACAAAGGATGTTCTAATTAAGCCGGCCAGGTATAATTCCTGTTATGGAGAGTGTGTGTTTGTCATTAATGATATTGCAGGGTTTATTGATCTTGTGTTATCTTTTATTTTTCCAGTATCTG

            1821    1830      1840      1850      1860      1870      1880      1890      1900      1910      1920      1930      1940      1950
            |--------+---------+---------+---------+---------+---------+---------+---------+---------+---------+---------+---------+---------|
contig046010 TTATCATAGTTCTTTATATGAGAGTGTTTGTGGTGGCTGTGTCTCAAGCTCGTTCCATGCGCTCTCATGTTACAGCTGTCGCACTTCAGCGTTCAAGCAATCAAGCAAACAAATCTGAGCTGAAAGCAGC
NyeTARs.A019 TTATCATAGTTCTTTATATGAGAGTGTTTGTGGTGGCTGTGTCTCAAGCTCGTTCCATGCGCTCTCATGTTACAGCTGTCGCACTTCAGCGTTCAAGCAATCAAGCAAACAAATCTGAGCTGAAAGCAGC
   Consensus TTATCATAGTTCTTTATATGAGAGTGTTTGTGGTGGCTGTGTCTCAAGCTCGTTCCATGCGCTCTCATGTTACAGCTGTCGCACTTCAGCGTTCAAGCAATCAAGCAAACAAATCTGAGCTGAAAGCAGC

            1951    1960      1970      1980      1990      2000      2010      2020      2030      2040      2050      2060      2070      2080
            |--------+---------+---------+---------+---------+---------+---------+---------+---------+---------+---------+---------+---------|
contig046010 CAGGAACCTTGGAGTTCTTGTAGTTGTGTTTCTCGCATGTTACCTTCCGTTTTACTGTTACTCTCTTATTGAAGTAAATGTGATCAATGACTCATCTACATTTTTTCTGATCATTGTGTTTTACTTTAAC
NyeTARs.A019 CAGGAACCTTGGAGTTCTTGTAGTTGTGTTTCTCGCATGTTACCTTCCGTTTTACTGTTACTCTCTTATTGAAGTAAATGTGATCAATGACTCATCTACATTTTTTCTGATCATTGTGTTTTACTTTAAC
   Consensus CAGGAACCTTGGAGTTCTTGTAGTTGTGTTTCTCGCATGTTACCTTCCGTTTTACTGTTACTCTCTTATTGAAGTAAATGTGATCAATGACTCATCTACATTTTTTCTGATCATTGTGTTTTACTTTAAC

            2081    2090      2100      2110      2120      2130      2140      2150      2160      2170      2180      2190      2200      2210
            |--------+---------+---------+---------+---------+---------+---------+---------+---------+---------+---------+---------+---------|
contig046010 TCGTGTCTAAACCCACTGATGTATGCCCTTTTTTACTCTTGGTTTAGAAATGCTGTTAAACTTATTATCACTCTGCAGATTTTTAAGCATGACACTAGTGAAGCAAACATGTTTTAAAAAGATAAGGAAA
NyeTARs.A019 TCGTGTCTAAACCCACTGATGTATGCCCTTTTTTACTCTTGGTTTAGAAATGCTGTTAAACTTATTATCACTCTGCAGATTTTTAAGCATGACACTAGTGAAGCAAACATGTTTTAA
   Consensus TCGTGTCTAAACCCACTGATGTATGCCCTTTTTTACTCTTGGTTTAGAAATGCTGTTAAACTTATTATCACTCTGCAGATTTTTAAGCATGACACTAGTGAAGCAAACATGTTTTAA.............
```

```
             4551    4560      4570      4580      4590      4600      4610      4620      4630      4640      4650      4660      4670      4680
             |--------+---------+---------+---------+---------+---------+---------+---------+---------+---------+---------+---------+---------|
contig057515 ACAAAAAAGGGGCTCAGTCACAAAGCTGTTTGTGCTGAACAGCTGTCTTTCTCTAACAATGGAAGAAACTGAACTCTGCTTTCCACAACTCCTCAACTCCTCTTGTGTGCGGCAAAAGCGCCCACAAATT
NyeTARsP.A020                                                          ATGGAAGAAACTGAACTCTGCTTTCCACAACTCCTCAACTCCTCTTGTGTGCGGCAAAAGCGCCCACAAATT
   Consensus ..........................................................ATGGAAGAAACTGAACTCTGCTTTCCACAACTCCTCAACTCCTCTTGTGTGCGGCAAAAGCGCCCACAAATT

             4681    4690      4700      4710      4720      4730      4740      4750      4760      4770      4780      4790      4800      4810
             |--------+---------+---------+---------+---------+---------+---------+---------+---------+---------+---------+---------+---------|
contig057515 GAGGCTGTGTGTATTTACACCCTGCTATCATCTATCTCTCTGCTTACTGCAGTACTGAACCTACTTGTCATCATCTCCATCGCTCACTTCAAGTACTGACCTATTTAAATTAGTGACTTTTTTTCAGTGT
NyeTARsP.A020 GAGGCTGTGTGTATTTACACCCTGCTATCATCTATCTCTCTGCTTACTGCAGTACTGAACCTACTTGTCATCATCTCCATCGCTCACTTCAAG-------------------------------------
   Consensus GAGGCTGTGTGTATTTACACCCTGCTATCATCTATCTCTCTGCTTACTGCAGTACTGAACCTACTTGTCATCATCTCCATCGCTCACTTCAAG.....................................

             4811    4820      4830      4840      4850      4860      4870      4880      4890      4900      4910      4920      4930      4940
             |--------+---------+---------+---------+---------+---------+---------+---------+---------+---------+---------+---------+---------|
contig057515 TAAAAAATCATATTTGCTTGTGAAGTTTCTTTTTATATGATGCTTTTGTCTTTTCCTCATAGTTCTAATGTTTTAACTGTTATACTAGCGATGCTATTACCAATGTAATGCTTTATTTTAATCCAGGCAG
NyeTARsP.A020 -------------------------------------------------------------------------------------------------------------------------------CAG
   Consensus ...............................................................................................................................CAG

             4941    4950      4960      4970      4980      4990      5000      5010      5020      5030      5040      5050      5060      5070
             |--------+---------+---------+---------+---------+---------+---------+---------+---------+---------+---------+---------+---------|
contig057515 CTGCACACCCCAACCAACCTCCTCCTCCTCTCTCTGGCCGTCTCAGATTTCTTCGTGGGCCTCATCATGGCTTGACAGATTAGCCTCCTAGATGGCTGCTGGTTTCTTGGTGACCACATGTGTGCTCTGT
NyeTARsP.A020 CTGCACACCCCAACCAACCTCCTCCTCCTCTCTCTGGCCGTCTCAGATTTCTTCGTGGGCCTCATCATGGCTTGACAGATTAGCCTCCTAGATGGCTGCTGGTTTCTTGGTGACCACATGTGTGCTCTGT
   Consensus CTGCACACCCCAACCAACCTCCTCCTCCTCTCTCTGGCCGTCTCAGATTTCTTCGTGGGCCTCATCATGGCTTGACAGATTAGCCTCCTAGATGGCTGCTGGTTTCTTGGTGACCACATGTGTGCTCTGT

             5071    5080      5090      5100      5110      5120      5130      5140      5150      5160      5170      5180      5190      5200
             |--------+---------+---------+---------+---------+---------+---------+---------+---------+---------+---------+---------+---------|
contig057515 ATAGCAGTTTAGATTACATTGTTACTTCTGCTTCAGTAGGAACTATGGTACTCATATCAGCTGACCGCTATGTAGCCATTTGTGACCCTCTGCATTATCCCACCAAAATTACTATAAAAAGAGTCTCAGT
NyeTARsP.A020 ATAGCAGTTTAGATTACATTGTTACTTCTGCTTCAGTAGGAACTATGGTACTCATATCAGCTGACCGCTATGTAGCCATTTGTGACCCTCTGCATTATCCCACCAAAATTACTATAAAAAGAGTCTCAGT
   Consensus ATAGCAGTTTAGATTACATTGTTACTTCTGCTTCAGTAGGAACTATGGTACTCATATCAGCTGACCGCTATGTAGCCATTTGTGACCCTCTGCATTATCCCACCAAAATTACTATAAAAAGAGTCTCAGT

             5201    5210      5220      5230      5240      5250      5260      5270      5280      5290      5300      5310      5320      5330
             |--------+---------+---------+---------+---------+---------+---------+---------+---------+---------+---------+---------+---------|
contig057515 CTCTATTTGTACTTGCTGGGCTTGTTCAATTCTGTATAACAGTCTTATCATGAAGGATAATTTCAAGCAGCCAGGGAGGTATAATTCTTGTTCTGGTGATTGTGTAGTTGTCATTGACTACTTTGTGGGA
NyeTARsP.A020 CTCTATTTGTACTTGCTGGGCTTGTTCAATTCTGTATAACAGTCTTATCATGAAGGATAATTTCAAGCAGCCAGGGAGGTATAATTCTTGTTCTGGTGATTGTGTAGTTGTCATTGACTACTTTGTGGGA
   Consensus CTCTATTTGTACTTGCTGGGCTTGTTCAATTCTGTATAACAGTCTTATCATGAAGGATAATTTCAAGCAGCCAGGGAGGTATAATTCTTGTTCTGGTGATTGTGTAGTTGTCATTGACTACTTTGTGGGA

             5331    5340      5350      5360      5370      5380      5390      5400      5410      5420      5430      5440      5450      5460
             |--------+---------+---------+---------+---------+---------+---------+---------+---------+---------+---------+---------+---------|
contig057515 ATTTTTGACTTTGTTTTGACCTTTGTCGGCCCTGTGATTGTCATCATAGTTCTGTATCTAAGAGTATTTGTGGTGGCTGTGTCTCAGGCTCGGGCAATGCGCTCTCATATCACTGCTCTCAGACTCCAGG
NyeTARsP.A020 ATTTTTGACTTTGTTTTGACCTTTGTCGGCCCTGTGATTGTCATCATAGTTCTGTATCTAAGAGTATTTGTGGTGGCTGTGTCTCAGGCTCGGGCAATGCGCTCTCATATCACTGCTCTCAGACTCCAGG
   Consensus ATTTTTGACTTTGTTTTGACCTTTGTCGGCCCTGTGATTGTCATCATAGTTCTGTATCTAAGAGTATTTGTGGTGGCTGTGTCTCAGGCTCGGGCAATGCGCTCTCATATCACTGCTCTCAGACTCCAGG

             5461    5470      5480      5490      5500      5510      5520      5530      5540      5550      5560      5570      5580      5590
             |--------+---------+---------+---------+---------+---------+---------+---------+---------+---------+---------+---------+---------|
contig057515 GGTCAGAGACGGTGCATGCTAAGAAATCTGAGCTGAAAGCTGCCAGGACTCTCGGTGTACTTGTTATTGCCTTTCTGATATGTCTTTTCCCATTTTTCTGTTCCTCAATGGTAGGCCAGAATAGTTTCTT
NyeTARsP.A020 GGTCAGAGACGGTGCATGCTAAGAAATCTGAGCTGAAAGCTGCCAGGACTCTCGGTGTACTTGTTATTGCCTTTCTGATATGTCTTTTCCCATTTTTCTGTTCCTCAATGGTAGGCCAGAATAGTTTCTT
   Consensus GGTCAGAGACGGTGCATGCTAAGAAATCTGAGCTGAAAGCTGCCAGGACTCTCGGTGTACTTGTTATTGCCTTTCTGATATGTCTTTTCCCATTTTTCTGTTCCTCAATGGTAGGCCAGAATAGTTTCTT

             5591    5600      5610      5620      5630      5640      5650      5660      5670      5680      5690      5700      5710      5720
             |--------+---------+---------+---------+---------+---------+---------+---------+---------+---------+---------+---------+---------|
contig057515 TGATATTAGATCTGTACCTTTTGAGCTCTTGCTGTTCTATTTTAACTCTTGTCTGAACCCATTAATCTACACTTTTTGCTACCCCTGGTTTCTGAAATCTATCAAGCTCATTGTGACATTTAAGATATTT
NyeTARsP.A020 TGATATTAGATCTGTACCTTTTGAGCTCTTGCTGTTCTATTTTAACTCTTGTCTGAACCCATTAATCTACACTTTTTGCTACCCCTGGTTTCTGAAATCTATCAAGCTCATTGTGACATTTAAGATATTT
   Consensus TGATATTAGATCTGTACCTTTTGAGCTCTTGCTGTTCTATTTTAACTCTTGTCTGAACCCATTAATCTACACTTTTTGCTACCCCTGGTTTCTGAAATCTATCAAGCTCATTGTGACATTTAAGATATTT

             5721    5730      5740      5750      5760      5770      5780      5790      5800      5810      5820      5830      5840      5850
             |--------+---------+---------+---------+---------+---------+---------+---------+---------+---------+---------+---------+---------|
contig057515 AGGCATGGCTCCAGTGAGGCCAGTATACTATAGGGAAAAGTGATGTTATCACTGACACGGTGAAAAAAGATAAATTATGATTAATTTACACTAAGTACAATTCCAGAAGTCTGATAAAATGGATTTGTTT
NyeTARsP.A020 AGGCATGGCTCCAGTGAGGCCAGTATACTATAG
   Consensus AGGCATGGCTCCAGTGAGGCCAGTATACTATAG.................................................................................................
```

```
             261  270      280      290      300      310      320      330      340      350      360      370      380      390
             |--------+---------+---------+---------+---------+---------+---------+---------+---------+---------+---------+---------+---------|
contig046013 ATTGGAGAGCTGCAGGTGTCTGATGATGGAGATACCGAAAGGAGTTGAGCTTTGTTTCCCACAACTCCTCAACAGCTCCTGCAGGAAGCCGACACTTCACTGGTCCAAAGCTGTGCTCCTGAACACTGTG
NyeTARs.A021                          ATGGAGATACCGAAAGGAGTTGAGCTTTGTTTCCCACAACTCCTCAACAGCTCCTGCAGGAAGCCGACACTTCACTGGTCCAAAGCTGTGCTCCTGAACACTGTG
   Consensus .........................ATGGAGATACCGAAAGGAGTTGAGCTTTGTTTCCCACAACTCCTCAACAGCTCCTGCAGGAAGCCGACACTTCACTGGTCCAAAGCTGTGCTCCTGAACACTGTG

             391  400      410      420      430      440      450      460      470      480      490      500      510      520
             |--------+---------+---------+---------+---------+---------+---------+---------+---------+---------+---------+---------+---------|
contig046013 CTGTGCTGCATCTCTCTGCTCACTGCTGCTCTAAACCTTCTCGTCATCATCTCAGTCTCCCACTTCAGGCAGAGATTAACTTTTCAACTGAAACTACAGTTTAAGTTTTAGATGTGTGAAAACTTCCGTT
NyeTARs.A021 CTGTGCTGCATCTCTCTGCTCACTGCTGCTCTAAACCTTCTCGTCATCATCTCAGTCTCCCACTTCAGGCAG----------------------------------------------------------
   Consensus CTGTGCTGCATCTCTCTGCTCACTGCTGCTCTAAACCTTCTCGTCATCATCTCAGTCTCCCACTTCAGGCAG..........................................................

             521  530      540      550      560      570      580      590      600      610      620      630      640      650
             |--------+---------+---------+---------+---------+---------+---------+---------+---------+---------+---------+---------+---------|
contig046013 TACATTATATGACTTAGTGATTAAAGAATGAAGTGAACTTTTCTTGGTGTATTTTAACCATGGTCATAATGTCTTATTTGCTTGAATTGAATCATGCCTTAGTAGTGCAGCATTTTCACTGAAGACATGT
NyeTARs.A021 ----------------------------------------------------------------------------------------------------------------------------------
   Consensus ..................................................................................................................................

             651  660      670      680      690      700      710      720      730      740      750      760      770      780
             |--------+---------+---------+---------+---------+---------+---------+---------+---------+---------+---------+---------+---------|
contig046013 TGACATTAACGATGTGCATTATATTCTTTTCTGTTTCCCTGCAGGCAGCTGCACACACCCAGTAATGTCCTCCTCCTCTCTCTGGCCGTCTCAGACTTTTTTGTGGGTCTCCTGTTGTTGCCTTTAGAAA
NyeTARs.A021 ------------------------------------------------CTGCACACACCCAGTAATGTCCTCCTCCTCTCTCTGGCCGTCTCAGACTTTTTTGTGGGTCTCCTGTTGTTGCCTTTAGAAA
   Consensus ................................................CTGCACACACCCAGTAATGTCCTCCTCCTCTCTCTGGCCGTCTCAGACTTTTTTGTGGGTCTCCTGTTGTTGCCTTTAGAAA

             781  790      800      810      820      830      840      850      860      870      880      890      900      910
             |--------+---------+---------+---------+---------+---------+---------+---------+---------+---------+---------+---------+---------|
contig046013 TTTTTAGAAACACAAGCTGCTGGGTACTTGGTGATCGTATGTGTTCTGCTTATTGGTATTTGACCAGCAACATTGTCTGTGCTTCAATAGGGAACATTGTTCTAATATCAGTTGACCGCTATGTGGCTAT
NyeTARs.A021 TTTTTAGAAACACAAGCTGCTGGGTACTTGGTGATCGTATGTGTTCTGCTTATTGGTATTTGACCAGCAACATTGTCTGTGCTTCAATAGGGAACATTGTTCTAATATCAGTTGACCGCTATGTGGCTAT
   Consensus TTTTTAGAAACACAAGCTGCTGGGTACTTGGTGATCGTATGTGTTCTGCTTATTGGTATTTGACCAGCAACATTGTCTGTGCTTCAATAGGGAACATTGTTCTAATATCAGTTGACCGCTATGTGGCTAT

             911  920      930      940      950      960      970      980      990     1000     1010     1020     1030     1040
             |--------+---------+---------+---------+---------+---------+---------+---------+---------+---------+---------+---------+---------|
contig046013 TTGTGACCCTCTGCATTATCCCACCAGAATTACTTTGGCAAACGTCAAACTCAGTGTTTGTCTGTGTTGGTTTTATGCTTTTTTCTACAGCAATCTTTATACAAAGGATATCATGATTGAACCAGGCAGG
NyeTARs.A021 TTGTGACCCTCTGCATTATCCCACCAGAATTACTTTGGCAAACGTCAAACTCAGTGTTTGTCTGTGTTGGTTTTATGCTTTTTTCTACAGCAATCTTTATACAAAGGATATCATGATTGAACCAGGCAGG
   Consensus TTGTGACCCTCTGCATTATCCCACCAGAATTACTTTGGCAAACGTCAAACTCAGTGTTTGTCTGTGTTGGTTTTATGCTTTTTTCTACAGCAATCTTTATACAAAGGATATCATGATTGAACCAGGCAGG

             1041 1050     1060     1070     1080     1090     1100     1110     1120     1130     1140     1150     1160     1170
             |--------+---------+---------+---------+---------+---------+---------+---------+---------+---------+---------+---------+---------|
contig046013 TATAATTCTTGCTTTGGAGAGTGTGTATTTTTTGGCAGCAATATTGCTATTGTTGCTGACCTTATTTTATTCTTTTTTGTTCCAGTAACTGTTATCATAGTCTTGTATATGAGAGTATTTGTGGTGGCTG
NyeTARs.A021 TATAATTCTTGCTTTGGAGAGTGTGTATTTTTTGGCAGCAATATTGCTATTGTTGCTGACCTTATTTTATTCTTTTTTGTTCCAGTAACTGTTATCATAGTCTTGTATATGAGAGTATTTGTGGTGGCTG
   Consensus TATAATTCTTGCTTTGGAGAGTGTGTATTTTTTGGCAGCAATATTGCTATTGTTGCTGACCTTATTTTATTCTTTTTTGTTCCAGTAACTGTTATCATAGTCTTGTATATGAGAGTATTTGTGGTGGCTG

             1171 1180     1190     1200     1210     1220     1230     1240     1250     1260     1270     1280     1290     1300
             |--------+---------+---------+---------+---------+---------+---------+---------+---------+---------+---------+---------+---------|
contig046013 TGTCTCAGGCTCGTGCCATGCGCTCTCATGTTACATTGGTCACACTTCAGCGTTCACTGAATCAAACAAACAAATCTGAGCTGAAAGCAGCCAGGACTCTGGGGATTCTTGTAGTTGTGTTTCTAGCATG
NyeTARs.A021 TGTCTCAGGCTCGTGCCATGCGCTCTCATGTTACATTGGTCACACTTCAGCGTTCACTGAATCAAACAAACAAATCTGAGCTGAAAGCAGCCAGGACTCTGGGGATTCTTGTAGTTGTGTTTCTAGCATG
   Consensus TGTCTCAGGCTCGTGCCATGCGCTCTCATGTTACATTGGTCACACTTCAGCGTTCACTGAATCAAACAAACAAATCTGAGCTGAAAGCAGCCAGGACTCTGGGGATTCTTGTAGTTGTGTTTCTAGCATG

             1301 1310     1320     1330     1340     1350     1360     1370     1380     1390     1400     1410     1420     1430
             |--------+---------+---------+---------+---------+---------+---------+---------+---------+---------+---------+---------+---------|
contig046013 CTTCTCTCCACTCTACTGCTACTCTCTTGTTGATGAAAATGCAATCAATGATCCAGCTGCATCTTTTGCAGTCATTATCTTTTACATTAACTCCTGTCTAAACCCTTTGATCTATGCCTTGTTTTACCCC
NyeTARs.A021 CTTCTCTCCACTCTACTGCTACTCTCTTGTTGATGAAAATGCAATCAATGATCCAGCTGCATCTTTTGCAGTCATTATCTTTTACATTAACTCCTGTCTAAACCCTTTGATCTATGCCTTGTTTTACCCC
   Consensus CTTCTCTCCACTCTACTGCTACTCTCTTGTTGATGAAAATGCAATCAATGATCCAGCTGCATCTTTTGCAGTCATTATCTTTTACATTAACTCCTGTCTAAACCCTTTGATCTATGCCTTGTTTTACCCC

             1431 1440     1450     1460     1470     1480     1490     1500     1510     1520     1530     1540     1550     1560
             |--------+---------+---------+---------+---------+---------+---------+---------+---------+---------+---------+---------+---------|
contig046013 TGGTTTAGAAATGCTGTTAAACTTATCATCACGTTGGAGATCTTCAAGTATGACACAAGTGGTGCCAACATACTGTAAAAAGAGTACGAAACATGCTCGTGCAATGGTTAGCACTGACACACCACAGCAA
NyeTARs.A021 TGGTTTAGAAATGCTGTTAAACTTATCATCACGTTGGAGATCTTCAAGTATGACACAAGTGGTGCCAACATACTGTAA
   Consensus TGGTTTAGAAATGCTGTTAAACTTATCATCACGTTGGAGATCTTCAAGTATGACACAAGTGGTGCCAACATACTGTAA....................................................
```

```
             131     140     150     160     170     180     190     200     210     220     230     240     250     260
             |--------+---------+---------+---------+---------+---------+---------+---------+---------+---------+---------+---------+---------|
contig062039 TAAACAGACTGTGTCTGCTGATCCTCTTGTCCTCATCTAGATGATGGAGAACAAGGACCGTATATGCTTTCCAGAAGTCTTCAACTCTTCCTGCAGGAGACCAGCATTTCACTGGTCTGAAGCTGTGCCC
NyeTARs.A022                                         ATGATGGAGAACAAGGACCGTATATGCTTTCCAGAAGTCTTCAACTCTTCCTGCAGGAGACCAGCATTTCACTGGTCTGAAGCTGTGCCC
   Consensus ........................................ATGATGGAGAACAAGGACCGTATATGCTTTCCAGAAGTCTTCAACTCTTCCTGCAGGAGACCAGCATTTCACTGGTCTGAAGCTGTGCCC

             261     270     280     290     300     310     320     330     340     350     360     370     380     390
             |--------+---------+---------+---------+---------+---------+---------+---------+---------+---------+---------+---------+---------|
contig062039 CTGAATACCCTGCTGTGCTCCATCTCTGTAATCACCATAGCTCTGAACCTCCTTGTCATCATCTCAGTCTCCTACTTCAGGCAGATTCTATTTTCTATTAGATTTAATTTGGGAAGTTTTCAAAATATGA
NyeTARs.A022 CTGAATACCCTGCTGTGCTCCATCTCTGTAATCACCATAGCTCTGAACCTCCTTGTCATCATCTCAGTCTCCTACTTCAGGCAG----------------------------------------------
   Consensus CTGAATACCCTGCTGTGCTCCATCTCTGTAATCACCATAGCTCTGAACCTCCTTGTCATCATCTCAGTCTCCTACTTCAGGCAG..............................................

             391     400     410     420     430     440     450     460     470     480     490     500     510     520
             |--------+---------+---------+---------+---------+---------+---------+---------+---------+---------+---------+---------+---------|
contig062039 TCGTGCAAAACCTGATGTACAGAAAACCACCACTACACCTTTCTCCAAAACAAGTTATTTTAATTGTTTTACAAAGTTAAATTTAACATAATGTTATGTGGCACTAACATAAAGTTTCTAATTTTAAGGG
NyeTARs.A022 ----------------------------------------------------------------------------------------------------------------------------------
   Consensus ..................................................................................................................................

             521     530     540     550     560     570     580     590     600     610     620     630     640     650
             |--------+---------+---------+---------+---------+---------+---------+---------+---------+---------+---------+---------+---------|
contig062039 AAGATTTTTTTGGTAATGGTGCTTTTTTGACTTATTCCAGGCAGCTCCACACACCCACTAACATCCTCCTCCTCTCTTTGGCTGTGTCAGACTTTCTTGTGGGGCTCCTGTTGCTGCCTGGAGAAATCTT
NyeTARs.A022 --------------------------------------------CTCCACACACCCACTAACATCCTCCTCCTCTCTTTGGCTGTGTCAGACTTTCTTGTGGGGCTCCTGTTGCTGCCTGGAGAAATCTT
   Consensus ............................................CTCCACACACCCACTAACATCCTCCTCCTCTCTTTGGCTGTGTCAGACTTTCTTGTGGGGCTCCTGTTGCTGCCTGGAGAAATCTT

             651     660     670     680     690     700     710     720     730     740     750     760     770     780
             |--------+---------+---------+---------+---------+---------+---------+---------+---------+---------+---------+---------+---------|
contig062039 CCTAGGAATAGCCTGCTGGGCTTTCGGTAACCTTTTGTGTTCTCTCTTTAATTATGTGTCTTTCATCATTACCTCGGCTTCAGTGGGAAACATGGTTCTCATATCAGTTGACCGCTATGTGGCGATCTGT
NyeTARs.A022 CCTAGGAATAGCCTGCTGGGCTTTCGGTAACCTTTTGTGTTCTCTCTTTAATTATGTGTCTTTCATCATTACCTCGGCTTCAGTGGGAAACATGGTTCTCATATCAGTTGACCGCTATGTGGCGATCTGT
   Consensus CCTAGGAATAGCCTGCTGGGCTTTCGGTAACCTTTTGTGTTCTCTCTTTAATTATGTGTCTTTCATCATTACCTCGGCTTCAGTGGGAAACATGGTTCTCATATCAGTTGACCGCTATGTGGCGATCTGT

             781     790     800     810     820     830     840     850     860     870     880     890     900     910
             |--------+---------+---------+---------+---------+---------+---------+---------+---------+---------+---------+---------+---------|
contig062039 TATCCTCTGCATTACTCTACCAGAATTACTGTGACAAGAGTTAAACGTTCTGTTTGTTTGTGTTGGCTCTGTTGTGTTTTGTACAGTAGTGTCCTTTTAAAAGATGAGCTGATTCAACCAGGAAGGCATA
NyeTARs.A022 TATCCTCTGCATTACTCTACCAGAATTACTGTGACAAGAGTTAAACGTTCTGTTTGTTTGTGTTGGCTCTGTTGTGTTTTGTACAGTAGTGTCCTTTTAAAAGATGAGCTGATTCAACCAGGAAGGCATA
   Consensus TATCCTCTGCATTACTCTACCAGAATTACTGTGACAAGAGTTAAACGTTCTGTTTGTTTGTGTTGGCTCTGTTGTGTTTTGTACAGTAGTGTCCTTTTAAAAGATGAGCTGATTCAACCAGGAAGGCATA

             911     920     930     940     950     960     970     980     990     1000    1010    1020    1030    1040
             |--------+---------+---------+---------+---------+---------+---------+---------+---------+---------+---------+---------+---------|
contig062039 ATTCCTGCTATGGAGAATGTGTTTTTGTCATCAACTTTATTGCAGGAACTGTTGATCTTTTGTTAACCTTTATTGTTCCTATTTCTGTTATTGTAGTTCTGTATATAAGAGTATTTGCAGTGGCTGTGTC
NyeTARs.A022 ATTCCTGCTATGGAGAATGTGTTTTTGTCATCAACTTTATTGCAGGAACTGTTGATCTTTTGTTAACCTTTATTGTTCCTATTTCTGTTATTGTAGTTCTGTATATAAGAGTATTTGCAGTGGCTGTGTC
   Consensus ATTCCTGCTATGGAGAATGTGTTTTTGTCATCAACTTTATTGCAGGAACTGTTGATCTTTTGTTAACCTTTATTGTTCCTATTTCTGTTATTGTAGTTCTGTATATAAGAGTATTTGCAGTGGCTGTGTC

             1041  1050     1060     1070     1080     1090     1100     1110     1120     1130     1140     1150     1160     1170
             |--------+---------+---------+---------+---------+---------+---------+---------+---------+---------+---------+---------+---------|
contig062039 TCAGGCCCGTGCCATGCGCTCTCAGCTTTCAGTGAATCTGACAACAAAGAAATCTGAGTTGAAAGCAGGAAGGACTCTTGGTGTCCTAATAGTTGTGTTTCTAATGTGTTTCTGTCCATACTATTGCGTC
NyeTARs.A022 TCAGGCCCGTGCCATGCGCTCTCAGCTTTCAGTGAATCTGACAACAAAGAAATCTGAGTTGAAAGCAGGAAGGACTCTTGGTGTCCTAATAGTTGTGTTTCTAATGTGTTTCTGTCCATACTATTGCGTC
   Consensus TCAGGCCCGTGCCATGCGCTCTCAGCTTTCAGTGAATCTGACAACAAAGAAATCTGAGTTGAAAGCAGGAAGGACTCTTGGTGTCCTAATAGTTGTGTTTCTAATGTGTTTCTGTCCATACTATTGCGTC

             1171  1180     1190     1200     1210     1220     1230     1240     1250     1260     1270     1280     1290     1300
             |--------+---------+---------+---------+---------+---------+---------+---------+---------+---------+---------+---------+---------|
contig062039 TCTCTTGCAGGGGAAGACTCACTCAGTGAGTCATCCACATCAATTGTCCGTTATCTCTTCTATTTTAACTCCTGTTTGAACCCTCTGATCTATGCACTATTTTACCCCTGGTTTAGAAAAGCTATTAAAC
NyeTARs.A022 TCTCTTGCAGGGGAAGACTCACTCAGTGAGTCATCCACATCAATTGTCCGTTATCTCTTCTATTTTAACTCCTGTTTGAACCCTCTGATCTATGCACTATTTTACCCCTGGTTTAGAAAAGCTATTAAAC
   Consensus TCTCTTGCAGGGGAAGACTCACTCAGTGAGTCATCCACATCAATTGTCCGTTATCTCTTCTATTTTAACTCCTGTTTGAACCCTCTGATCTATGCACTATTTTACCCCTGGTTTAGAAAAGCTATTAAAC

             1301  1310     1320     1330     1340     1350     1360     1370     1380     1390     1400     1410     1420     1430
             |--------+---------+---------+---------+---------+---------+---------+---------+---------+---------+---------+---------+---------|
contig062039 ACATTGTCATGCTTCAGATATTTCACACTGGCTCCCATGAGGACAACATACTATAGAGAGGACCTAAATGAGTGAATAAGTGAAATCTGCTGAACATATATTTTTATCCTTAACCCGTATAATGTTATGC
NyeTARs.A022 ACATTGTCATGCTTCAGATATTTCACACTGGCTCCCATGAGGACAACATACTATAG
   Consensus ACATTGTCATGCTTCAGATATTTCACACTGGCTCCCATGAGGACAACATACTATAG..........................................................................
```

```
              2081     2090      2100      2110      2120      2130      2140      2150      2160      2170      2180      2190      2200      2210
              |--------+---------+---------+---------+---------+---------+---------+---------+---------+---------+---------+---------+---------|
contig035377  AGGATGTGGCATGGGTTTTAGTTATATTACTAATTCTGGTCATAACTCAGCCAGAGCTGGAGCTCTCTGCAGGTCTGTGGAGATGGATACTCAGGATGGAGCAGAGCTCTGCTTTCCACAACTCTTCAAC
NyeTARs.A023                                                                                    ATGGATACTCAGGATGGAGCAGAGCTCTGCTTTCCACAACTCTTCAAC
   Consensus  ..................................................................................ATGGATACTCAGGATGGAGCAGAGCTCTGCTTTCCACAACTCTTCAAC

              2211     2220      2230      2240      2250      2260      2270      2280      2290      2300      2310      2320      2330      2340
              |--------+---------+---------+---------+---------+---------+---------+---------+---------+---------+---------+---------+---------|
contig035377  ATCTCCTGCAAGAAGCCTACAACTCCTCTGTCTCAATTGTTGCTTCCTTATATTGTTGTGTCCTCAATGTCTCTGCTGACTGTGACTCTCAACCTGCTCGTCATCATCGCAGTCTCCCACTTCAGGCAGA
NyeTARs.A023  ATCTCCTGCAAGAAGCCTACAACTCCTCTGTCTCAATTGTTGCTTCCTTATATTGTTGTGTCCTCAATGTCTCTGCTGACTGTGACTCTCAACCTGCTCGTCATCATCGCAGTCTCCCACTTCAG-----
   Consensus  ATCTCCTGCAAGAAGCCTACAACTCCTCTGTCTCAATTGTTGCTTCCTTATATTGTTGTGTCCTCAATGTCTCTGCTGACTGTGACTCTCAACCTGCTCGTCATCATCGCAGTCTCCCACTTCAG.....

              2341     2350      2360      2370      2380      2390      2400      2410      2420      2430      2440      2450      2460      2470
              |--------+---------+---------+---------+---------+---------+---------+---------+---------+---------+---------+---------+---------|
contig035377  GTTTCACTTTTGTAACTGTTATTGTAGCTTTACAAGTCAGAGTCAGCTTAAGTAAATACATGATTTTACTCATATGTGCACTAGACTTACATTTGAACTGTTAGACTATTTTTATATAATGATCTGTATA
NyeTARs.A023  ----------------------------------------------------------------------------------------------------------------------------------
   Consensus  ..................................................................................................................................

              2471     2480      2490      2500      2510      2520      2530      2540      2550      2560      2570      2580      2590      2600
              |--------+---------+---------+---------+---------+---------+---------+---------+---------+---------+---------+---------+---------|
contig035377  TATTAGATGTCAATATTAGATGACAGACTTAGAGACTTTAGATATTCTTTATATGAGGTACTTCATACCATATTCCTTCTCCTACCAATAATCTTTACATTCAGTCTGCTACAACTTATCGTCTGTCTAC
NyeTARs.A023  ----------------------------------------------------------------------------------------------------------------------------------
   Consensus  ..................................................................................................................................

              2601     2610      2620      2630      2640      2650      2660      2670      2680      2690      2700      2710      2720      2730
              |--------+---------+---------+---------+---------+---------+---------+---------+---------+---------+---------+---------+---------|
contig035377  ACTTGAACAAACTACACCTATAACTGAATTATTTTCAGATGTTTCAGATGATTTCTCTCTCTTCTTTCAGACAGCTCAACACACCCACTAACATCCTCCTCCTCTCTCTGGCTGTGTCAGACTTTTTCAT
NyeTARs.A023  ----------------------------------------------------------------------ACAGCTCAACACACCCACTAACATCCTCCTCCTCTCTCTGGCTGTGTCAGACTTTTTCAT
   Consensus  ......................................................................ACAGCTCAACACACCCACTAACATCCTCCTCCTCTCTCTGGCTGTGTCAGACTTTTTCAT

              2731     2740      2750      2760      2770      2780      2790      2800      2810      2820      2830      2840      2850      2860
              |--------+---------+---------+---------+---------+---------+---------+---------+---------+---------+---------+---------+---------|
contig035377  TGGTCTGTTGTTGATGCCTGCAAATATCATCCGAAAGAAGGTTTGTTGGTTTCTTGGTCAGCTTGCATGTTTAGTGTTTACTTATGTATCCTTCATTATTACCTCTGCCTCAGTGGGCATCATGGTGCTG
NyeTARs.A023  TGGTCTGTTGTTGATGCCTGCAAATATCATCCGAAAGAAGGTTTGTTGGTTTCTTGGTCAGCTTGCATGTTTAGTGTTTACTTATGTATCCTTCATTATTACCTCTGCCTCAGTGGGCATCATGGTGCTG
   Consensus  TGGTCTGTTGTTGATGCCTGCAAATATCATCCGAAAGAAGGTTTGTTGGTTTCTTGGTCAGCTTGCATGTTTAGTGTTTACTTATGTATCCTTCATTATTACCTCTGCCTCAGTGGGCATCATGGTGCTG

              2861     2870      2880      2890      2900      2910      2920      2930      2940      2950      2960      2970      2980      2990
              |--------+---------+---------+---------+---------+---------+---------+---------+---------+---------+---------+---------+---------|
contig035377  ATATCAATCGACAGGTATGCGGCTATTTGTGACCCTCTGCATTACCCCACCAGAATAACTCGTGAAAGAGTGAAACTCTGTGTCTGTCTGTGTTGGCTCTGCTCGGTTTTTTACAACATACTATTTATAA
NyeTARs.A023  ATATCAATCGACAGGTATGCGGCTATTTGTGACCCTCTGCATTACCCCACCAGAATAACTCGTGAAAGAGTGAAACTCTGTGTCTGTCTGTGTTGGCTCTGCTCGGTTTTTTACAACATACTATTTATAA
   Consensus  ATATCAATCGACAGGTATGCGGCTATTTGTGACCCTCTGCATTACCCCACCAGAATAACTCGTGAAAGAGTGAAACTCTGTGTCTGTCTGTGTTGGCTCTGCTCGGTTTTTTACAACATACTATTTATAA

              2991     3000      3010      3020      3030      3040      3050      3060      3070      3080      3090      3100      3110      3120
              |--------+---------+---------+---------+---------+---------+---------+---------+---------+---------+---------+---------+---------|
contig035377  AGAACGACCTGCTTCAACGAGAACGACATACTTCCTGTTATGGAGAGTGTAAAATTCTCGTAGACTACATTGCAGGAACCACTGATATTGTTTTAACCTTTATTGCTCCAGTTACTGTCATCATAGTTCT
NyeTARs.A023  AGAACGACCTGCTTCAACGAGAACGACATACTTCCTGTTATGGAGAGTGTAAAATTCTCGTAGACTACATTGCAGGAACCACTGATATTGTTTTAACCTTTATTGCTCCAGTTACTGTCATCATAGTTCT
   Consensus  AGAACGACCTGCTTCAACGAGAACGACATACTTCCTGTTATGGAGAGTGTAAAATTCTCGTAGACTACATTGCAGGAACCACTGATATTGTTTTAACCTTTATTGCTCCAGTTACTGTCATCATAGTTCT

              3121     3130      3140      3150      3160      3170      3180      3190      3200      3210      3220      3230      3240      3250
              |--------+---------+---------+---------+---------+---------+---------+---------+---------+---------+---------+---------+---------|
contig035377  GTATATGAGAGTATTTGTGGTGGCTGTGTCTCAGGCCCGTGCCATGCGCTCTCAGGTTACAGCTGTCACACTACAGCTCTCAGTGACTCTAACAACAAAGAAATCAGAGTTAAAAGCAGCCAGGACACTG
NyeTARs.A023  GTATATGAGAGTATTTGTGGTGGCTGTGTCTCAGGCCCGTGCCATGCGCTCTCAGGTTACAGCTGTCACACTACAGCTCTCAGTGACTCTAACAACAAAGAAATCAGAGTTAAAAGCAGCCAGGACACTG
   Consensus  GTATATGAGAGTATTTGTGGTGGCTGTGTCTCAGGCCCGTGCCATGCGCTCTCAGGTTACAGCTGTCACACTACAGCTCTCAGTGACTCTAACAACAAAGAAATCAGAGTTAAAAGCAGCCAGGACACTG

              3251     3260      3270      3280      3290      3300      3310      3320      3330      3340      3350      3360      3370      3380
              |--------+---------+---------+---------+---------+---------+---------+---------+---------+---------+---------+---------+---------|
contig035377  GGTGTTCTTGTTCTTGTGTTTCTAATATGTTTCTGCCCTCATTATTGTATTTTTGTTTTTGGAGGAAAGGTGCTTAATAGCTCATCTGCAACTATTGTGATCTATCTGTATTATTTTAACTCCTGTCTAA
NyeTARs.A023  GGTGTTCTTGTTCTTGTGTTTCTAATATGTTTCTGCCCTCATTATTGTATTTTTGTTTTTGGAGGAAAGGTGCTTAATAGCTCATCTGCAACTATTGTGATCTATCTGTATTATTTTAACTCCTGTCTAA
   Consensus  GGTGTTCTTGTTCTTGTGTTTCTAATATGTTTCTGCCCTCATTATTGTATTTTTGTTTTTGGAGGAAAGGTGCTTAATAGCTCATCTGCAACTATTGTGATCTATCTGTATTATTTTAACTCCTGTCTAA

              3381     3390      3400      3410      3420      3430      3440      3450      3460      3470      3480      3490      3500      3510
              |--------+---------+---------+---------+---------+---------+---------+---------+---------+---------+---------+---------+---------|
contig035377  ATCCCTTGATTTATGCTATGTTCTACCCCTGGTTTAGAAAAGCTGTGAAACTAATTGTGACACTACAGATACTGCAGCCTGGCTCCTGTGAAGTCAGCATACTGTAGAAAAAAGAAATCTCCTTTAAAAA
NyeTARs.A023  ATCCCTTGATTTATGCTATGTTCTACCCCTGGTTTAGAAAAGCTGTGAAACTAATTGTGACACTACAGATACTGCAGCCTGGCTCCTGTGAAGTCAGCATACTGTAG
   Consensus  ATCCCTTGATTTATGCTATGTTCTACCCCTGGTTTAGAAAAGCTGTGAAACTAATTGTGACACTACAGATACTGCAGCCTGGCTCCTGTGAAGTCAGCATACTGTAG.......................
```

```
             1171 1180      1190      1200      1210      1220      1230      1240      1250      1260      1270      1280      1290      1300
             |--------+---------+---------+---------+---------+---------+---------+---------+---------+---------+---------+---------+---------|
contig046014 GGAGGGACATTTTTGTGTGTGTCGTGTGTGTATGTCTCTGGGTGGTGGTGTAATAAATCAGGTCTGTGAGAAAACATTCTGATCAGTGAGCTGCAGCTGTCTGATGATGGAGATTCAGAAAGCAGCTGAG
NyeTARs.A024                                                                                                           ATGGAGATTCAGAAAGCAGCTGAG
   Consensus ..........................................................................................................ATGGAGATTCAGAAAGCAGCTGAG

             1301 1310      1320      1330      1340      1350      1360      1370      1380      1390      1400      1410      1420      1430
             |--------+---------+---------+---------+---------+---------+---------+---------+---------+---------+---------+---------+---------|
contig046014 CTTTGTTTTCCACAACTCCTCAACAGTTCCTGCAGGAAGCCGACACTTCACTGGTCCAAAGCTGTGCTCCTGAACATTGTGCTGTCCTGCATCTCTCTGATCACTGCTGCTCTAAACCTCCTGGTCATCA
NyeTARs.A024 CTTTGTTTTCCACAACTCCTCAACAGTTCCTGCAGGAAGCCGACACTTCACTGGTCCAAAGCTGTGCTCCTGAACATTGTGCTGTCCTGCATCTCTCTGATCACTGCTGCTCTAAACCTCCTGGTCATCA
   Consensus CTTTGTTTTCCACAACTCCTCAACAGTTCCTGCAGGAAGCCGACACTTCACTGGTCCAAAGCTGTGCTCCTGAACATTGTGCTGTCCTGCATCTCTCTGATCACTGCTGCTCTAAACCTCCTGGTCATCA

             1431 1440      1450      1460      1470      1480      1490      1500      1510      1520      1530      1540      1550      1560
             |--------+---------+---------+---------+---------+---------+---------+---------+---------+---------+---------+---------+---------|
contig046014 TCTCAGTCTCCCACTTCAGGCAGAGAAACATTTCACTGCTGAAACTATACTTTAAAATTTAGAAACTTAAATTTCAGAAAATTATGATTTATGGCATTTGATTTTATGATTAAGATTGAGATGATTGAAG
NyeTARs.A024 TCTCAGTCTCCCACTTCAGGCAG-----------------------------------------------------------------------------------------------------------
   Consensus TCTCAGTCTCCCACTTCAGGCAG...........................................................................................................

             1561 1570      1580      1590      1600      1610      1620      1630      1640      1650      1660      1670      1680      1690
             |--------+---------+---------+---------+---------+---------+---------+---------+---------+---------+---------+---------+---------|
contig046014 TGGTGTATTTTAACCAGCTATTAAGTTTTATATACCTAGACTGAATCTTACTACATACTTTAACTAATGAAAATCTAATTTAAGCCATTTAAAAAAATATTAATGTCCATTATATTCTTTTCTGTTTCCC
NyeTARs.A024 ----------------------------------------------------------------------------------------------------------------------------------
   Consensus ..................................................................................................................................

             1691 1700      1710      1720      1730      1740      1750      1760      1770      1780      1790      1800      1810      1820
             |--------+---------+---------+---------+---------+---------+---------+---------+---------+---------+---------+---------+---------|
contig046014 TGCAGGCAGCTGCACACACCCAGTAACATCCTGCTCGTCTCTCTGGCTGTCTCAGACTTTCTTGTGGGTCTTCTGCTGATGCCATTAGAGATCTTTAGAAACACAGCTTGCTGGGTACTTGGTGATGTAA
NyeTARs.A024 ---------CTGCACACACCCAGTAACATCCTGCTCGTCTCTCTGGCTGTCTCAGACTTTCTTGTGGGTCTTCTGCTGATGCCATTAGAGATCTTTAGAAACACAGCTTGCTGGGTACTTGGTGATGTAA
   Consensus .........CTGCACACACCCAGTAACATCCTGCTCGTCTCTCTGGCTGTCTCAGACTTTCTTGTGGGTCTTCTGCTGATGCCATTAGAGATCTTTAGAAACACAGCTTGCTGGGTACTTGGTGATGTAA

             1821 1830      1840      1850      1860      1870      1880      1890      1900      1910      1920      1930      1940      1950
             |--------+---------+---------+---------+---------+---------+---------+---------+---------+---------+---------+---------+---------|
contig046014 TGTGTTCTGTTTATTGGTACCTGACCAGCAACATTACCTGTGCTTCAATAGGGAACATAGTTCTAATATCAGTTGACCGTTATGTGGCGATTTGTGATCCTCTGCATTACCCCACTAGAATTACTTTGGT
NyeTARs.A024 TGTGTTCTGTTTATTGGTACCTGACCAGCAACATTACCTGTGCTTCAATAGGGAACATAGTTCTAATATCAGTTGACCGTTATGTGGCGATTTGTGATCCTCTGCATTACCCCACTAGAATTACTTTGGT
   Consensus TGTGTTCTGTTTATTGGTACCTGACCAGCAACATTACCTGTGCTTCAATAGGGAACATAGTTCTAATATCAGTTGACCGTTATGTGGCGATTTGTGATCCTCTGCATTACCCCACTAGAATTACTTTGGT

             1951 1960      1970      1980      1990      2000      2010      2020      2030      2040      2050      2060      2070      2080
             |--------+---------+---------+---------+---------+---------+---------+---------+---------+---------+---------+---------+---------|
contig046014 GAAAGTCAAACTCAGTGTTTGTCTGTGTTGGTTTTTTTCTATTTTCTACTGGAGTCTTTATATGAAAGATATCCTAGTTGAACCAGGCAGGTATAATTCCTGCTATGGAGAGTGTGTGCTTATTATTAAT
NyeTARs.A024 GAAAGTCAAACTCAGTGTTTGTCTGTGTTGGTTTTTTTCTATTTTCTACTGGAGTCTTTATATGAAAGATATCCTAGTTGAACCAGGCAGGTATAATTCCTGCTATGGAGAGTGTGTGCTTATTATTAAT
   Consensus GAAAGTCAAACTCAGTGTTTGTCTGTGTTGGTTTTTTTCTATTTTCTACTGGAGTCTTTATATGAAAGATATCCTAGTTGAACCAGGCAGGTATAATTCCTGCTATGGAGAGTGTGTGCTTATTATTAAT

             2081 2090      2100      2110      2120      2130      2140      2150      2160      2170      2180      2190      2200      2210
             |--------+---------+---------+---------+---------+---------+---------+---------+---------+---------+---------+---------+---------|
contig046014 GACATTGCAGGGATTGTTGACCTTGCTTTGTCCTTTATAGTTCCAGTTACTGTTATCATAGTTTTGTATATGAGAGTATTTGTGGTGGCTGTGTCTCAGGCTCGTGCCATGCGCTCTCATGTTACATTTG
NyeTARs.A024 GACATTGCAGGGATTGTTGACCTTGCTTTGTCCTTTATAGTTCCAGTTACTGTTATCATAGTTTTGTATATGAGAGTATTTGTGGTGGCTGTGTCTCAGGCTCGTGCCATGCGCTCTCATGTTACATTTG
   Consensus GACATTGCAGGGATTGTTGACCTTGCTTTGTCCTTTATAGTTCCAGTTACTGTTATCATAGTTTTGTATATGAGAGTATTTGTGGTGGCTGTGTCTCAGGCTCGTGCCATGCGCTCTCATGTTACATTTG

             2211 2220      2230      2240      2250      2260      2270      2280      2290      2300      2310      2320      2330      2340
             |--------+---------+---------+---------+---------+---------+---------+---------+---------+---------+---------+---------+---------|
contig046014 TCACACTTCAGTGTTCATTGAATCAAGCAAACAAATCTGAGCTGAAAGCAGCCAGGACTCTGGGGGTTCTTGTAGTTGTATTTTTGGCATGCTACTGCCCATTTTACTGCTACTCTCTGGTTGATAAAAA
NyeTARs.A024 TCACACTTCAGTGTTCATTGAATCAAGCAAACAAATCTGAGCTGAAAGCAGCCAGGACTCTGGGGGTTCTTGTAGTTGTATTTTTGGCATGCTACTGCCCATTTTACTGCTACTCTCTGGTTGATAAAAA
   Consensus TCACACTTCAGTGTTCATTGAATCAAGCAAACAAATCTGAGCTGAAAGCAGCCAGGACTCTGGGGGTTCTTGTAGTTGTATTTTTGGCATGCTACTGCCCATTTTACTGCTACTCTCTGGTTGATAAAAA

             2341 2350      2360      2370      2380      2390      2400      2410      2420      2430      2440      2450      2460      2470
             |--------+---------+---------+---------+---------+---------+---------+---------+---------+---------+---------+---------+---------|
contig046014 TGTGGTCAATGATTCATCTGCATCTTTTGTGGTTCTTGTCTTTTATTTTAACTCTTGTTTAAACCCATTGATCTATGCTCTGTTTTACCCCTGGTTTAGAAAAGCTATTAGATGTGTTATCACTCTGCTG
NyeTARs.A024 TGTGGTCAATGATTCATCTGCATCTTTTGTGGTTCTTGTCTTTTATTTTAACTCTTGTTTAAACCCATTGATCTATGCTCTGTTTTACCCCTGGTTTAGAAAAGCTATTAGATGTGTTATCACTCTGCTG
   Consensus TGTGGTCAATGATTCATCTGCATCTTTTGTGGTTCTTGTCTTTTATTTTAACTCTTGTTTAAACCCATTGATCTATGCTCTGTTTTACCCCTGGTTTAGAAAAGCTATTAGATGTGTTATCACTCTGCTG

             2471 2480      2490      2500      2510      2520      2530      2540      2550      2560      2570      2580      2590      2600
             |--------+---------+---------+---------+---------+---------+---------+---------+---------+---------+---------+---------+---------|
contig046014 ATATTTAAGCATGACAGCAGTGAGGTCAACGTAATATAGAAATACAATGAAAGCTGCTCCACTAAGATTTTAAACCAGTAAATTGATACAGTTCAGTCTGGAAATGCATCAAAAACAAATTGATAAGGAA
NyeTARs.A024 ATATTTAAGCATGACAGCAGTGAGGTCAACGTAATATAG
   Consensus ATATTTAAGCATGACAGCAGTGAGGTCAACGTAATATAG...........................................................................................
```

```
              7281  7290      7300      7310      7320      7330      7340      7350      7360      7370      7380      7390      7400      7410
              |--------+---------+---------+---------+---------+---------+---------+---------+---------+---------+---------+---------+---------|
contig058002  GCAAGCTATCTCTTCCTTTTTACTAATGATGGAAATGTTTGAAGAAACTCAACTCTGCTTTCCACAGCTTCTTAACTTCTCCTGCAGGAAGCCCATACGACCTCACTCAGTATCTATACTCATTTATATT
NyeTARs.A025                              ATGGAAATGTTTGAAGAAACTCAACTCTGCTTTCCACAGCTTCTTAACTTCTCCTGCAGGAAGCCCATACGACCTCACTCAGTATCTATACTCATTTATATT
   Consensus  ............................ATGGAAATGTTTGAAGAAACTCAACTCTGCTTTCCACAGCTTCTTAACTTCTCCTGCAGGAAGCCCATACGACCTCACTCAGTATCTATACTCATTTATATT

              7411  7420      7430      7440      7450      7460      7470      7480      7490      7500      7510      7520      7530      7540
              |--------+---------+---------+---------+---------+---------+---------+---------+---------+---------+---------+---------+---------|
contig058002  ACATTGTCCTCCATCTCTTTACTGACTGTGACTCTTAATCTGCTGGTCATCATCTCCATCTCACATTTCAAGTAATAATTTTGTCATAGATAAGGTTATCAGGTTTTGTGGATCAGTAAAATGAAGATAA
NyeTARs.A025  ACATTGTCCTCCATCTCTTTACTGACTGTGACTCTTAATCTGCTGGTCATCATCTCCATCTCACATTTCAAG----------------------------------------------------------
   Consensus  ACATTGTCCTCCATCTCTTTACTGACTGTGACTCTTAATCTGCTGGTCATCATCTCCATCTCACATTTCAAG..........................................................

              7541  7550      7560      7570      7580      7590      7600      7610      7620      7630      7640      7650      7660      7670
              |--------+---------+---------+---------+---------+---------+---------+---------+---------+---------+---------+---------+---------|
contig058002  CATATTAGAGTATAAGTGAAATGTGTGTCGGTAGTAATGTCTGTTTTTCTCATATATATATATATATCTGTATAAAATTGTACTTGTTGCTACAAATAATAATAATAATAACTATGTCGCTGTCTTTTGT
NyeTARs.A025  ----------------------------------------------------------------------------------------------------------------------------------
   Consensus  ..................................................................................................................................

              7671  7680      7690      7700      7710      7720      7730      7740      7750      7760      7770      7780      7790      7800
              |--------+---------+---------+---------+---------+---------+---------+---------+---------+---------+---------+---------+---------|
contig058002  CTTCAGGAAGCTGCACACCCCCACCAATCTCCTCCTCCTATCTTTGGCTGTCTCTGATTGCCTGGTGGGTCTCCTGATTTTATTTCAAATTATGATTATAGATGGTTGCTGGTTTCTTGGTGAATTCATG
NyeTARs.A025  -------AAGCTGCACACCCCCACCAATCTCCTCCTCCTATCTTTGGCTGTCTCTGATTGCCTGGTGGGTCTCCTGATTTTATTTCAAATTATGATTATAGATGGTTGCTGGTTTCTTGGTGAATTCATG
   Consensus  .......AAGCTGCACACCCCCACCAATCTCCTCCTCCTATCTTTGGCTGTCTCTGATTGCCTGGTGGGTCTCCTGATTTTATTTCAAATTATGATTATAGATGGTTGCTGGTTTCTTGGTGAATTCATG

              7801  7810      7820      7830      7840      7850      7860      7870      7880      7890      7900      7910      7920      7930
              |--------+---------+---------+---------+---------+---------+---------+---------+---------+---------+---------+---------+---------|
contig058002  TGTAGTATGTATTTTCTCTTGGACTACATTATTACCTCTGCCTCAGTAGGAACCATGGTTCTCATATCAATTGACCGTTATGTGGCTATTTGTTACCCTCTCCATTACTCTACCAAAGTCACCCCAAAAA
NyeTARs.A025  TGTAGTATGTATTTTCTCTTGGACTACATTATTACCTCTGCCTCAGTAGGAACCATGGTTCTCATATCAATTGACCGTTATGTGGCTATTTGTTACCCTCTCCATTACTCTACCAAAGTCACCCCAAAAA
   Consensus  TGTAGTATGTATTTTCTCTTGGACTACATTATTACCTCTGCCTCAGTAGGAACCATGGTTCTCATATCAATTGACCGTTATGTGGCTATTTGTTACCCTCTCCATTACTCTACCAAAGTCACCCCAAAAA

              7931  7940      7950      7960      7970      7980      7990      8000      8010      8020      8030      8040      8050      8060
              |--------+---------+---------+---------+---------+---------+---------+---------+---------+---------+---------+---------+---------|
contig058002  GAACAAAAGCATGTGTTTATCTCTGTTGGATTTGTTCTTCAGTCGACCAATGTCTCCTGCTAAAAGACAACCTGGTGCAACCTGGCAGGTATAATTCCTGCTATGGGGAGTGTGTGGTTGTTGTTGGCCA
NyeTARs.A025  GAACAAAAGCATGTGTTTATCTCTGTTGGATTTGTTCTTCAGTCGACCAATGTCTCCTGCTAAAAGACAACCTGGTGCAACCTGGCAGGTATAATTCCTGCTATGGGGAGTGTGTGGTTGTTGTTGGCCA
   Consensus  GAACAAAAGCATGTGTTTATCTCTGTTGGATTTGTTCTTCAGTCGACCAATGTCTCCTGCTAAAAGACAACCTGGTGCAACCTGGCAGGTATAATTCCTGCTATGGGGAGTGTGTGGTTGTTGTTGGCCA

              8061  8070      8080      8090      8100      8110      8120      8130      8140      8150      8160      8170      8180      8190
              |--------+---------+---------+---------+---------+---------+---------+---------+---------+---------+---------+---------+---------|
contig058002  TGCTTTTGGAGTTGCAGATCTATTGCTGTCCATTATTGGTCCTGTCACTGTGATTGTAGTTCTTTATCTAAACGTATTTGTTGTGGCTATGACTCAAGCTCGTGCCCTGCGGTCACATATTGCAGCTCTG
NyeTARs.A025  TGCTTTTGGAGTTGCAGATCTATTGCTGTCCATTATTGGTCCTGTCACTGTGATTGTAGTTCTTTATCTAAACGTATTTGTTGTGGCTATGACTCAAGCTCGTGCCCTGCGGTCACATATTGCAGCTCTG
   Consensus  TGCTTTTGGAGTTGCAGATCTATTGCTGTCCATTATTGGTCCTGTCACTGTGATTGTAGTTCTTTATCTAAACGTATTTGTTGTGGCTATGACTCAAGCTCGTGCCCTGCGGTCACATATTGCAGCTCTG

              8191  8200      8210      8220      8230      8240      8250      8260      8270      8280      8290      8300      8310      8320
              |--------+---------+---------+---------+---------+---------+---------+---------+---------+---------+---------+---------+---------|
contig058002  ACACACGAGAGGTCAGTAAGTACAAATGTTAAGAAATCTGAAATGAAAGCAGTAAGGACCATCAGTGTTCTTATCATTGTGTTTCTAATATGTCTCTGCCCATATTACTGTGTCACACTCTCAGGTCAAG
NyeTARs.A025  ACACACGAGAGGTCAGTAAGTACAAATGTTAAGAAATCTGAAATGAAAGCAGTAAGGACCATCAGTGTTCTTATCATTGTGTTTCTAATATGTCTCTGCCCATATTACTGTGTCACACTCTCAGGTCAAG
   Consensus  ACACACGAGAGGTCAGTAAGTACAAATGTTAAGAAATCTGAAATGAAAGCAGTAAGGACCATCAGTGTTCTTATCATTGTGTTTCTAATATGTCTCTGCCCATATTACTGTGTCACACTCTCAGGTCAAG

              8321  8330      8340      8350      8360      8370      8380      8390      8400      8410      8420      8430      8440      8450
              |--------+---------+---------+---------+---------+---------+---------+---------+---------+---------+---------+---------+---------|
contig058002  ATGCCATGCTCAGTGCTTCATCTGTTGCATTTGTAATGTGTTTGTTCTATTTAAACTCCTGTCTAAACCCTCTAATCTATGCTTTATTTTACCCCTGGTTCAGAAAATCTGTTAAACAAATTGTTACACT
NyeTARs.A025  ATGCCATGCTCAGTGCTTCATCTGTTGCATTTGTAATGTGTTTGTTCTATTTAAACTCCTGTCTAAACCCTCTAATCTATGCTTTATTTTACCCCTGGTTCAGAAAATCTGTTAAACAAATTGTTACACT
   Consensus  ATGCCATGCTCAGTGCTTCATCTGTTGCATTTGTAATGTGTTTGTTCTATTTAAACTCCTGTCTAAACCCTCTAATCTATGCTTTATTTTACCCCTGGTTCAGAAAATCTGTTAAACAAATTGTTACACT

              8451  8460      8470      8480      8490      8500      8510      8520      8530      8540      8550      8560      8570      8580
              |--------+---------+---------+---------+---------+---------+---------+---------+---------+---------+---------+---------+---------|
contig058002  TAAAATATTGAAGTCTGGCTCCTGTGACACCAATATCATGTACACAGAATGAAAGAAATGACATACAAACAAAATGTTTACCAACTAAAAAAAGGGACAGTCAATTCAAACATACGCTTGGTGTAACTTA
NyeTARs.A025  TAAAATATTGAAGTCTGGCTCCTGTGACACCAATATCATGTACACAGAATGA
   Consensus  TAAAATATTGAAGTCTGGCTCCTGTGACACCAATATCATGTACACAGAATGA...............................................................................
```

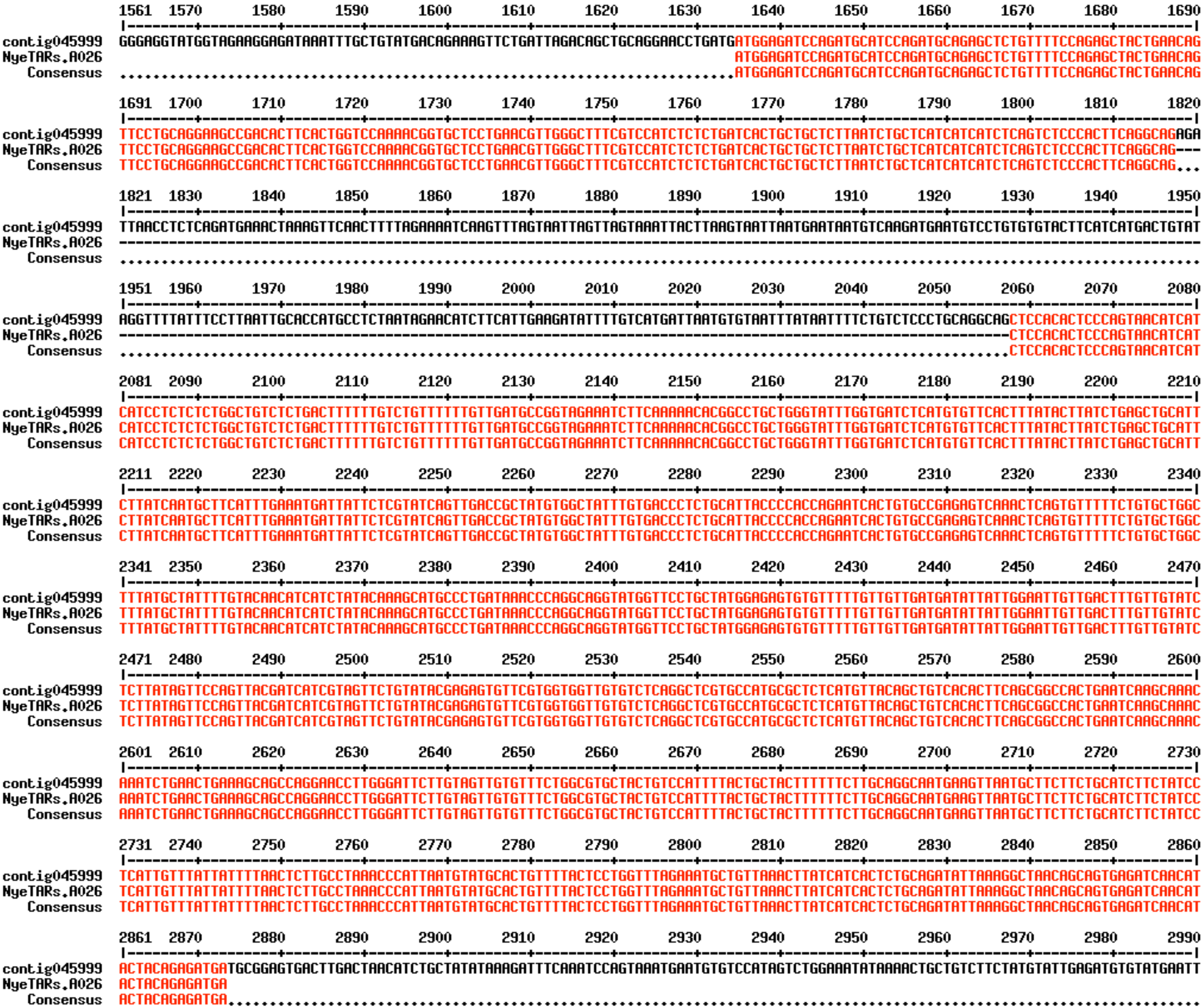
contig045999
NyeTARs.A026
Consensus

```
             2861  2870      2880      2890      2900      2910      2920      2930      2940      2950      2960      2970      2980      2990
             |---------+---------+---------+---------+---------+---------+---------+---------+---------+---------+---------+---------+---------|
contig060292 AGCACTTCTAACTGATGATGGCAGAAACTGAACTCTGCTTTCCAAATCTCAACTCCTCCTGCAGTAGAGTAAAGCGTTCTCCCTCGGACTCAGTGCTTATTTATGTAATCCTAACCATCATTTCTCTTCT
NyeTARs.A027                  ATGGCAGAAACTGAACTCTGCTTTCCAAATCTCAACTCCTCCTGCAGTAGAGTAAAGCGTTCTCCCTCGGACTCAGTGCTTATTTATGTAATCCTAACCATCATTTCTCTTCT
   Consensus .................ATGGCAGAAACTGAACTCTGCTTTCCAAATCTCAACTCCTCCTGCAGTAGAGTAAAGCGTTCTCCCTCGGACTCAGTGCTTATTTATGTAATCCTAACCATCATTTCTCTTCT

             2991  3000      3010      3020      3030      3040      3050      3060      3070      3080      3090      3100      3110      3120
             |---------+---------+---------+---------+---------+---------+---------+---------+---------+---------+---------+---------+---------|
contig060292 TACTGTGGCTCTAAACCTGCTGGTCATCATCTCCATCTCCCACTTCAAGTAGATACTTAATACATTTATAAGTCTAGCAGTTGTACAGGAGAAAATGACTGACATAAAATAAGTTAAACAGAACAACTTT
NyeTARs.A027 TACTGTGGCTCTAAACCTGCTGGTCATCATCTCCATCTCCCACTTCAAG-----------------------------------------------------------------------------------
   Consensus TACTGTGGCTCTAAACCTGCTGGTCATCATCTCCATCTCCCACTTCAAG...................................................................................

             3121  3130      3140      3150      3160      3170      3180      3190      3200      3210      3220      3230      3240      3250
             |---------+---------+---------+---------+---------+---------+---------+---------+---------+---------+---------+---------+---------|
contig060292 TTGACAATGTTTTGATGTGGATAATCGGCTGGAATAATTGATGCTACAAATAATATATATTTTTCTATCTCCTATCTCTCCAGGCAGCTCCACACTCCTACAAACCACCTCCTCCTCTCCCTGGCAGTCT
NyeTARs.A027 ------------------------------------------------------------------------------------CAGCTCCACACTCCTACAAACCACCTCCTCCTCTCCCTGGCAGTCT
   Consensus ....................................................................................CAGCTCCACACTCCTACAAACCACCTCCTCCTCTCCCTGGCAGTCT

             3251  3260      3270      3280      3290      3300      3310      3320      3330      3340      3350      3360      3370      3380
             |---------+---------+---------+---------+---------+---------+---------+---------+---------+---------+---------+---------+---------|
contig060292 CTGATTTCTTTGTGGGTCTCAATATGTGCTTTCAAAGTATGCTCATAGATGGTTGTTGGTATCTTGGTGACCTCATGTGTGTCTTGTATTATGTTTTCGACATAGTTGCTACTTCTGCCTCAGTAGGAAC
NyeTARs.A027 CTGATTTCTTTGTGGGTCTCAATATGTGCTTTCAAAGTATGCTCATAGATGGTTGTTGGTATCTTGGTGACCTCATGTGTGTCTTGTATTATGTTTTCGACATAGTTGCTACTTCTGCCTCAGTAGGAAC
   Consensus CTGATTTCTTTGTGGGTCTCAATATGTGCTTTCAAAGTATGCTCATAGATGGTTGTTGGTATCTTGGTGACCTCATGTGTGTCTTGTATTATGTTTTCGACATAGTTGCTACTTCTGCCTCAGTAGGAAC

             3381  3390      3400      3410      3420      3430      3440      3450      3460      3470      3480      3490      3500      3510
             |---------+---------+---------+---------+---------+---------+---------+---------+---------+---------+---------+---------+---------|
contig060292 CATGGTGCTCATTTCAGTTGACCGTTATGTGGCCATTTGTGATCCTCTTCATTATCCCACCAAAGTCACTCCAAAAAGAGTTCAGACCTGTGTCTTAATGTGTTGGATTTGCTCGCTCCTAGTAGTCGGT
NyeTARs.A027 CATGGTGCTCATTTCAGTTGACCGTTATGTGGCCATTTGTGATCCTCTTCATTATCCCACCAAAGTCACTCCAAAAAGAGTTCAGACCTGTGTCTTAATGTGTTGGATTTGCTCGCTCCTAGTAGTCGGT
   Consensus CATGGTGCTCATTTCAGTTGACCGTTATGTGGCCATTTGTGATCCTCTTCATTATCCCACCAAAGTCACTCCAAAAAGAGTTCAGACCTGTGTCTTAATGTGTTGGATTTGCTCGCTCCTAGTAGTCGGT

             3511  3520      3530      3540      3550      3560      3570      3580      3590      3600      3610      3620      3630      3640
             |---------+---------+---------+---------+---------+---------+---------+---------+---------+---------+---------+---------+---------|
contig060292 GTGCTCTTGAAGGATAACCTGGATAAACCAGGTAGATTTAATTCCTGCTTTGGAGAGTGTGTGATTTATGTTGACTTTGCAATACAAGTTACCGATCTTATTTTGACAATCCTCCTTCCCATTACTGTCA
NyeTARs.A027 GTGCTCTTGAAGGATAACCTGGATAAACCAGGTAGATTTAATTCCTGCTTTGGAGAGTGTGTGATTTATGTTGACTTTGCAATACAAGTTACCGATCTTATTTTGACAATCCTCCTTCCCATTACTGTCA
   Consensus GTGCTCTTGAAGGATAACCTGGATAAACCAGGTAGATTTAATTCCTGCTTTGGAGAGTGTGTGATTTATGTTGACTTTGCAATACAAGTTACCGATCTTATTTTGACAATCCTCCTTCCCATTACTGTCA

             3641  3650      3660      3670      3680      3690      3700      3710      3720      3730      3740      3750      3760      3770
             |---------+---------+---------+---------+---------+---------+---------+---------+---------+---------+---------+---------+---------|
contig060292 TCGTAATTTTGTATGTAAGAGTATTTGCCGTGGCCGTGTTTCAGATTCGGGCCATGCAACCTCATGTTGCAGCTGTCACACAGAGGGGGAAAGTAAGTCCAAAAAAATCTGAACTGAAAGCAGCTAGGAC
NyeTARs.A027 TCGTAATTTTGTATGTAAGAGTATTTGCCGTGGCCGTGTTTCAGATTCGGGCCATGCAACCTCATGTTGCAGCTGTCACACAGAGGGGGAAAGTAAGTCCAAAAAAATCTGAACTGAAAGCAGCTAGGAC
   Consensus TCGTAATTTTGTATGTAAGAGTATTTGCCGTGGCCGTGTTTCAGATTCGGGCCATGCAACCTCATGTTGCAGCTGTCACACAGAGGGGGAAAGTAAGTCCAAAAAAATCTGAACTGAAAGCAGCTAGGAC

             3771  3780      3790      3800      3810      3820      3830      3840      3850      3860      3870      3880      3890      3900
             |---------+---------+---------+---------+---------+---------+---------+---------+---------+---------+---------+---------+---------|
contig060292 TCTTGGCATTGTTATTGTTGCATTTCTGATATGCCTATTCCCATATTACAGTGTGATACTCTCAGGCCAGGACACACTGCTTGATATTTTATCCGTTACCTTTTTTTTTATGTTTGTTTTATTTTAATTCC
NyeTARs.A027 TCTTGGCATTGTTATTGTTGCATTTCTGATATGCCTATTCCCATATTACAGTGTGATACTCTCAGGCCAGGACACACTGCTTGATATTTTATCCGTTACCTTTTTTTTTATGTTTGTTTTATTTTAATTCC
   Consensus TCTTGGCATTGTTATTGTTGCATTTCTGATATGCCTATTCCCATATTACAGTGTGATACTCTCAGGCCAGGACACACTGCTTGATATTTTATCCGTTACCTTTTTTTTTATGTTTGTTTTATTTTAATTCC

             3901  3910      3920      3930      3940      3950      3960      3970      3980      3990      4000      4010      4020      4030
             |---------+---------+---------+---------+---------+---------+---------+---------+---------+---------+---------+---------+---------|
contig060292 TGTCTCAACCCTATCATCTATGCCTTTTTCTACCCATGGTTTAGAAAATCTGTTAAACTTATTGTTACATTTCAAATAGTCAAGTCTGGCTCCAGTGATGCCAGCATGCTGTGACTGAAATAAGCCCTTC
NyeTARs.A027 TGTCTCAACCCTATCATCTATGCCTTTTTCTACCCATGGTTTAGAAAATCTGTTAAACTTATTGTTACATTTCAAATAGTCAAGTCTGGCTCCAGTGATGCCAGCATGCTGTGA
   Consensus TGTCTCAACCCTATCATCTATGCCTTTTTCTACCCATGGTTTAGAAAATCTGTTAAACTTATTGTTACATTTCAAATAGTCAAGTCTGGCTCCAGTGATGCCAGCATGCTGTGA...............

[illegible]
```

```
             8971 8980      8990      9000      9010      9020      9030      9040      9050      9060      9070      9080      9090      9100
             |--------+---------+---------+---------+---------+---------+---------+---------+---------+---------+---------+---------+---------|
contig042499 ACAGCAACATGGGAAATATGTGGAAGGAGGGACCTTTTTTAATGAAGAGAACATGGGAGGAGTAAAAGAACAAGATAAACCTAATCAGCTCACCTCCTGTGATGATGATGGAGGGGGTTGAATTCTGCTT
NyeTARs.A028                                                                                                            ATGGAGGGGGTTGAATTCTGCTT
   Consensus ...........................................................................................................ATGGAGGGGGTTGAATTCTGCTT

             9101 9110      9120      9130      9140      9150      9160      9170      9180      9190      9200      9210      9220      9230
             |--------+---------+---------+---------+---------+---------+---------+---------+---------+---------+---------+---------+---------|
contig042499 TCCACATCTGCTCAACTCCTCTTGCAGAAAGGCTCTGCATCCTGTCTCAGTTTCCACGCTCATTTACATGATAATATCTTCTATCTCTGTGCTCACTGCAACTCTTAACCTGCTGGTCATCATCTCCATC
NyeTARs.A028 TCCACATCTGCTCAACTCCTCTTGCAGAAAGGCTCTGCATCCTGTCTCAGTTTCCACGCTCATTTACATGATAATATCTTCTATCTCTGTGCTCACTGCAACTCTTAACCTGCTGGTCATCATCTCCATC
   Consensus TCCACATCTGCTCAACTCCTCTTGCAGAAAGGCTCTGCATCCTGTCTCAGTTTCCACGCTCATTTACATGATAATATCTTCTATCTCTGTGCTCACTGCAACTCTTAACCTGCTGGTCATCATCTCCATC

             9231 9240      9250      9260      9270      9280      9290      9300      9310      9320      9330      9340      9350      9360
             |--------+---------+---------+---------+---------+---------+---------+---------+---------+---------+---------+---------+---------|
contig042499 TCCCACTTCAAGTAGCCACATATTTTATAAAGTTAAAAGATTAATAGCTTTTTTTAAGGAAAAACTTAAAAAAACAACAACTATGAATTTATGCTGCAAATAACAAGTTCTTTTTTTCTCTAGGCAGCTC
NyeTARs.A028 TCCCACTTCAAG----------------------------------------------------------------------------------------------------------------CAGCTC
   Consensus TCCCACTTCAAG................................................................................................................CAGCTC

             9361 9370      9380      9390      9400      9410      9420      9430      9440      9450      9460      9470      9480      9490
             |--------+---------+---------+---------+---------+---------+---------+---------+---------+---------+---------+---------+---------|
contig042499 CACAATCCCACCAACTTCCTCCTCCTCTCTCTGGCTGTCTCCGATTTCTTTGTGGGTCTCTATTTGCTCTTTTACATAATGTTTATAGACGGCTGCTGGTATTTTGGTGACTTCATGTGTATTCTGTATT
NyeTARs.A028 CACAATCCCACCAACTTCCTCCTCCTCTCTCTGGCTGTCTCCGATTTCTTTGTGGGTCTCTATTTGCTCTTTTACATAATGTTTATAGACGGCTGCTGGTATTTTGGTGACTTCATGTGTATTCTGTATT
   Consensus CACAATCCCACCAACTTCCTCCTCCTCTCTCTGGCTGTCTCCGATTTCTTTGTGGGTCTCTATTTGCTCTTTTACATAATGTTTATAGACGGCTGCTGGTATTTTGGTGACTTCATGTGTATTCTGTATT

             9491 9500      9510      9520      9530      9540      9550      9560      9570      9580      9590      9600      9610      9620
             |--------+---------+---------+---------+---------+---------+---------+---------+---------+---------+---------+---------+---------|
contig042499 ATGTTATTGCCACAATTAACACCTCTTCCTCAATAGGAACCATGGTACTGATATCAGTTGACCGTTATGTGGCCATTTGTGATCCTCTGCATTATCCCACCAAAGTCACTGCAAAAAGAGTTCAGATCTG
NyeTARs.A028 ATGTTATTGCCACAATTAACACCTCTTCCTCAATAGGAACCATGGTACTGATATCAGTTGACCGTTATGTGGCCATTTGTGATCCTCTGCATTATCCCACCAAAGTCACTGCAAAAAGAGTTCAGATCTG
   Consensus ATGTTATTGCCACAATTAACACCTCTTCCTCAATAGGAACCATGGTACTGATATCAGTTGACCGTTATGTGGCCATTTGTGATCCTCTGCATTATCCCACCAAAGTCACTGCAAAAAGAGTTCAGATCTG

             9621 9630      9640      9650      9660      9670      9680      9690      9700      9710      9720      9730      9740      9750
             |--------+---------+---------+---------+---------+---------+---------+---------+---------+---------+---------+---------+---------|
contig042499 TGTTTCACTGTGTTGGAGCTTTTCTGCCCTTGCTGGTAGTTTCCTCTTAAAGGACAACTTGAAACAGCAAAGCAGGTTTAATTCTTGTGTGGGAGAGTGTGTTGTCCATATTAACTTTATTGAATATGTT
NyeTARs.A028 TGTTTCACTGTGTTGGAGCTTTTCTGCCCTTGCTGGTAGTTTCCTCTTAAAGGACAACTTGAAACAGCAAAGCAGGTTTAATTCTTGTGTGGGAGAGTGTGTTGTCCATATTAACTTTATTGAATATGTT
   Consensus TGTTTCACTGTGTTGGAGCTTTTCTGCCCTTGCTGGTAGTTTCCTCTTAAAGGACAACTTGAAACAGCAAAGCAGGTTTAATTCTTGTGTGGGAGAGTGTGTTGTCCATATTAACTTTATTGAATATGTT

             9751 9760      9770      9780      9790      9800      9810      9820      9830      9840      9850      9860      9870      9880
             |--------+---------+---------+---------+---------+---------+---------+---------+---------+---------+---------+---------+---------|
contig042499 GCAGATTTTGCTTTGAACTTCCTACTTCCTATTACTGTCATTATAGTTCTGTATTTGAGAATATTTGTAGTGGTTGTGTCTCAGGTTCGGGCCATGCGGAGTCATACTGCAGGTGTCACATACCAGTGTT
NyeTARs.A028 GCAGATTTTGCTTTGAACTTCCTACTTCCTATTACTGTCATTATAGTTCTGTATTTGAGAATATTTGTAGTGGTTGTGTCTCAGGTTCGGGCCATGCGGAGTCATACTGCAGGTGTCACATACCAGTGTT
   Consensus GCAGATTTTGCTTTGAACTTCCTACTTCCTATTACTGTCATTATAGTTCTGTATTTGAGAATATTTGTAGTGGTTGTGTCTCAGGTTCGGGCCATGCGGAGTCATACTGCAGGTGTCACATACCAGTGTT

             9881 9890      9900      9910      9920      9930      9940      9950      9960      9970      9980      9990     10000     10010
             |--------+---------+---------+---------+---------+---------+---------+---------+---------+---------+---------+---------+---------|
contig042499 CAAGGAAGGGAAATCCAAAGAAATCAGAGATGAAAGCAGCCAGGACTCTTGGTATTGTTGTAATTGCATTCTTGTCATGCGCCTTACCATTTTATTGTGTCACACTCACAGGCCAGAATGCCTTCCTCAA
NyeTARs.A028 CAAGGAAGGGAAATCCAAAGAAATCAGAGATGAAAGCAGCCAGGACTCTTGGTATTGTTGTAATTGCATTCTTGTCATGCGCCTTACCATTTTATTGTGTCACACTCACAGGCCAGAATGCCTTCCTCAA
   Consensus CAAGGAAGGGAAATCCAAAGAAATCAGAGATGAAAGCAGCCAGGACTCTTGGTATTGTTGTAATTGCATTCTTGTCATGCGCCTTACCATTTTATTGTGTCACACTCACAGGCCAGAATGCCTTCCTCAA

             1001110020     10030     10040     10050     10060     10070     10080     10090     10100     10110     10120     10130     10140
             |--------+---------+---------+---------+---------+---------+---------+---------+---------+---------+---------+---------+---------|
contig042499 CGGATCATCTTCTGCATTTGTTTTGTGTCTTTTCTATTTTAATTCGTGCCTCAACCCTATCATCTATGCCTTATTCTACCCCTGGTTTAGAAAATCTGTTAAGCTTATTGTTACATTTCAAATACTGAAG
NyeTARs.A028 CGGATCATCTTCTGCATTTGTTTTGTGTCTTTTCTATTTTAATTCGTGCCTCAACCCTATCATCTATGCCTTATTCTACCCCTGGTTTAGAAAATCTGTTAAGCTTATTGTTACATTTCAAATACTGAAG
   Consensus CGGATCATCTTCTGCATTTGTTTTGTGTCTTTTCTATTTTAATTCGTGCCTCAACCCTATCATCTATGCCTTATTCTACCCCTGGTTTAGAAAATCTGTTAAGCTTATTGTTACATTTCAAATACTGAAG

             1014110150     10160     10170     10180     10190     10200     10210     10220     10230     10240     10250     10260     10270
             |--------+---------+---------+---------+---------+---------+---------+---------+---------+---------+---------+---------+---------|
contig042499 TCTGGCTCCAGGAATGCCAACATAGTCAAAGTGACAGAATAGTGACTAAAACGAGGTCTCTACTGTTTTAGCAGCAGTTTACGAGTAAGTCAGCCCAAACAAGCAGTGTTATATTTTCAGATTGCAACAT
NyeTARs.A028 TCTGGCTCCAGGAATGCCAACATAGTCAAAGTGACAGAATAG
   Consensus TCTGGCTCCAGGAATGCCAACATAGTCAAAGTGACAGAATAG........................................................................................

             1027110280     10290     10300     10310     10320     10330     10340     10350     10360     10370     10380     10390     10400
```

```
            781   790       800       810       820       830       840       850       860       870       880       890       900       910
            |---------+---------+---------+---------+---------+---------+---------+---------+---------+---------+---------+---------+---------|
contig056200 CAACAAACAATGGGGGAAAGAAAGGGTGGAGTTGAGAGAAGGAGTATCACCTACTTATGAAATTAATTTTTGTTCATAATGCTGCGACCAGAGCTGTAGTTCTTCACTAGTCTGTGACGATGGGCACACA
NyeTARs.A029                                                                                                                          ATGGGCACACA
   Consensus ........................................................................................................................ATGGGCACACA

            911   920       930       940       950       960       970       980       990      1000      1010      1020      1030      1040
            |---------+---------+---------+---------+---------+---------+---------+---------+---------+---------+---------+---------+---------|
contig056200 GGAAAAATCTGAGCTCTGCTTTCCACAACTCTTCAACATGTCCTGCAAAAAGCCTGCCATTGCTCGGTCCAAAGCTGTGTTCCTTCATATTGTATTGTCCTCAGTTTCTTTTCTGACGGTGGCTCTCAAC
NyeTARs.A029 GGAAAAATCTGAGCTCTGCTTTCCACAACTCTTCAACATGTCCTGCAAAAAGCCTGCCATTGCTCGGTCCAAAGCTGTGTTCCTTCATATTGTATTGTCCTCAGTTTCTTTTCTGACGGTGGCTCTCAAC
   Consensus GGAAAAATCTGAGCTCTGCTTTCCACAACTCTTCAACATGTCCTGCAAAAAGCCTGCCATTGCTCGGTCCAAAGCTGTGTTCCTTCATATTGTATTGTCCTCAGTTTCTTTTCTGACGGTGGCTCTCAAC

            1041  1050      1060      1070      1080      1090      1100      1110      1120      1130      1140      1150      1160      1170
            |---------+---------+---------+---------+---------+---------+---------+---------+---------+---------+---------+---------+---------|
contig056200 TTGCTTGTCATCATCTCAGTCTCCCACTTCAGGCAGAAACAACGTTTTTGTTTTACTAGCTACACTATTCTAGTGTTTCAAGTATTTGAGCTAATAATACAAGATTCTTCATATGTACAGATTTATACTT
NyeTARs.A029 TTGCTTGTCATCATCTCAGTCTCCCACTTCAGGCAG----------------------------------------------------------------------------------------------
   Consensus TTGCTTGTCATCATCTCAGTCTCCCACTTCAGGCAG..............................................................................................

            1171  1180      1190      1200      1210      1220      1230      1240      1250      1260      1270      1280      1290      1300
            |---------+---------+---------+---------+---------+---------+---------+---------+---------+---------+---------+---------+---------|
contig056200 GAGAATATTTACATTTCATCATTTTTACTTTGGCACCAGAAAACATACTATACTCATTGTCTTATAAGTAATACTATGTTCTGAATCAGTTTGATGTTGATAATATGCTGCTGTGTGACACTTAATAGCT
NyeTARs.A029 ----------------------------------------------------------------------------------------------------------------------------------
   Consensus ..................................................................................................................................

            1301  1310      1320      1330      1340      1350      1360      1370      1380      1390      1400      1410      1420      1430
            |---------+---------+---------+---------+---------+---------+---------+---------+---------+---------+---------+---------+---------|
contig056200 CTTTGGCTTGTTTCATTATAGAGGTTTGAGGAATTGTTATTTTTCTGTCTCCTTGCAGGCAGCTTCACACACCCACTAACATCCTGCTCCTCTCTCTGGCTGTCTCAGACTTTCTTGTTGGCCTCCTATT
NyeTARs.A029 --------------------------------------------------------------CTTCACACACCCACTAACATCCTGCTCCTCTCTCTGGCTGTCTCAGACTTTCTTGTTGGCCTCCTATT
   Consensus ..............................................................CTTCACACACCCACTAACATCCTGCTCCTCTCTCTGGCTGTCTCAGACTTTCTTGTTGGCCTCCTATT

            1431  1440      1450      1460      1470      1480      1490      1500      1510      1520      1530      1540      1550      1560
            |---------+---------+---------+---------+---------+---------+---------+---------+---------+---------+---------+---------+---------|
contig056200 GTCACCTGCAGAAATCCTCCGAAGTACAGCCTGTTGGTTTCTTGGTCAGCCCACATGTTTAATGTATATTTTTGTGTCCTTGACTGTTACCTCTGCCTCAGTGGGCATTATGGTGCTGATATCAGCTGAC
NyeTARs.A029 GTCACCTGCAGAAATCCTCCGAAGTACAGCCTGTTGGTTTCTTGGTCAGCCCACATGTTTAATGTATATTTTTGTGTCCTTGACTGTTACCTCTGCCTCAGTGGGCATTATGGTGCTGATATCAGCTGAC
   Consensus GTCACCTGCAGAAATCCTCCGAAGTACAGCCTGTTGGTTTCTTGGTCAGCCCACATGTTTAATGTATATTTTTGTGTCCTTGACTGTTACCTCTGCCTCAGTGGGCATTATGGTGCTGATATCAGCTGAC

            1561  1570      1580      1590      1600      1610      1620      1630      1640      1650      1660      1670      1680      1690
            |---------+---------+---------+---------+---------+---------+---------+---------+---------+---------+---------+---------+---------|
contig056200 CGCTATGTGGCTATTTGTGACCCTTTGAATTACCCCATCAGGATCACTGACAGAAGAGTTCAACTCTGTGTTTGTCTGTGTTGGCTCTGCTCCATCGCCTTCAGCTGTTTCCTTGTAAGAGATGACCTGC
NyeTARs.A029 CGCTATGTGGCTATTTGTGACCCTTTGAATTACCCCATCAGGATCACTGACAGAAGAGTTCAACTCTGTGTTTGTCTGTGTTGGCTCTGCTCCATCGCCTTCAGCTGTTTCCTTGTAAGAGATGACCTGC
   Consensus CGCTATGTGGCTATTTGTGACCCTTTGAATTACCCCATCAGGATCACTGACAGAAGAGTTCAACTCTGTGTTTGTCTGTGTTGGCTCTGCTCCATCGCCTTCAGCTGTTTCCTTGTAAGAGATGACCTGC

            1691  1700      1710      1720      1730      1740      1750      1760      1770      1780      1790      1800      1810      1820
            |---------+---------+---------+---------+---------+---------+---------+---------+---------+---------+---------+---------+---------|
contig056200 ATCAAAAAAGAAAACAAAATTCCTGCTATGGAAAATGTGTCGTTGTTGTTCAATACATTGCAGGAGTTGTTGACCTCATTTTAACCTTCATTGTACCAGTTACTGTCATCATAGTTCTGTATATGAGAGT
NyeTARs.A029 ATCAAAAAAGAAAACAAAATTCCTGCTATGGAAAATGTGTCGTTGTTGTTCAATACATTGCAGGAGTTGTTGACCTCATTTTAACCTTCATTGTACCAGTTACTGTCATCATAGTTCTGTATATGAGAGT
   Consensus ATCAAAAAAGAAAACAAAATTCCTGCTATGGAAAATGTGTCGTTGTTGTTCAATACATTGCAGGAGTTGTTGACCTCATTTTAACCTTCATTGTACCAGTTACTGTCATCATAGTTCTGTATATGAGAGT

            1821  1830      1840      1850      1860      1870      1880      1890      1900      1910      1920      1930      1940      1950
            |---------+---------+---------+---------+---------+---------+---------+---------+---------+---------+---------+---------+---------|
contig056200 ATTTGTGGTGGCTGTGTCTCAGGCCCGCTCTATGCGCTCTCATGCTACAGCTGTCAGAAATCACCTTCCAATGACGCTAACAAAAAAGTCTGAACTAAAAGCAGCCAGGACTCTGGGTGTTCTTGTTCTT
NyeTARs.A029 ATTTGTGGTGGCTGTGTCTCAGGCCCGCTCTATGCGCTCTCATGCTACAGCTGTCAGAAATCACCTTCCAATGACGCTAACAAAAAAGTCTGAACTAAAAGCAGCCAGGACTCTGGGTGTTCTTGTTCTT
   Consensus ATTTGTGGTGGCTGTGTCTCAGGCCCGCTCTATGCGCTCTCATGCTACAGCTGTCAGAAATCACCTTCCAATGACGCTAACAAAAAAGTCTGAACTAAAAGCAGCCAGGACTCTGGGTGTTCTTGTTCTT

            1951  1960      1970      1980      1990      2000      2010      2020      2030      2040      2050      2060      2070      2080
            |---------+---------+---------+---------+---------+---------+---------+---------+---------+---------+---------+---------+---------|
contig056200 GTGTTTCTAATGTGTTTCTGCCCTTATTACTGTGTTTCTCTTGTAGGAGAAGAATTCATCAATAGTTCATCTGCTTCTTTTGTGGCCTATCTGTTTGGTTTAAACTCATGTCTAAACCCTTTGATCTATG
NyeTARs.A029 GTGTTTCTAATGTGTTTCTGCCCTTATTACTGTGTTTCTCTTGTAGGAGAAGAATTCATCAATAGTTCATCTGCTTCTTTTGTGGCCTATCTGTTTGGTTTAAACTCATGTCTAAACCCTTTGATCTATG
   Consensus GTGTTTCTAATGTGTTTCTGCCCTTATTACTGTGTTTCTCTTGTAGGAGAAGAATTCATCAATAGTTCATCTGCTTCTTTTGTGGCCTATCTGTTTGGTTTAAACTCATGTCTAAACCCTTTGATCTATG

            2081  2090      2100      2110      2120      2130      2140      2150      2160      2170      2180      2190      2200      2210
            |---------+---------+---------+---------+---------+---------+---------+---------+---------+---------+---------+---------+---------|
contig056200 CTATGTTCTACCCCTGGTTTAGAAAAGCTGTGAAACTAGTTGTCACTCTACAGATACTGCAGCCTGGCTCCAGTGAGGTAAGTATTTTGTAAACAGATCAAAACATTTAAAAAATAGTTGTTTTCAGGTT
NyeTARs.A029 CTATGTTCTACCCCTGGTTTAGAAAAGCTGTGAAACTAGTTGTCACTCTACAGATACTGCAGCCTGGCTCCAGTGAGGTAAGTATTTTGTAA
   Consensus CTATGTTCTACCCCTGGTTTAGAAAAGCTGTGAAACTAGTTGTCACTCTACAGATACTGCAGCCTGGCTCCAGTGAGGTAAGTATTTTGTAA.....................................

            [illegible]
```

```
           1041 1050      1060      1070      1080      1090      1100      1110      1120      1130      1140      1150      1160      1170
           |--------+---------+---------+---------+---------+---------+---------+---------+---------+---------+---------+---------+---------|
contig052987 TCATGGAGTTATTCAATGTGACTGTTAATACTGTGAGCTTCCTTCTCTGCGATTCACAAAAGAACAAATTATGTGTGTTATTATATGTTGTCCTCAGCTCATTTATGCTTCTTACAATATGTGGAAATCT
 NyeTAR.B030   ATGGAGTTATTCAATGTGACTGTTAATACTGTGAGCTTCCTTCTCTGCGATTCACAAAAGAACAAATTATGTGTGTTATTATATGTTGTCCTCAGCTCATTTATGCTTCTTACAATATGTGGAAATCT
   Consensus ..ATGGAGTTATTCAATGTGACTGTTAATACTGTGAGCTTCCTTCTCTGCGATTCACAAAAGAACAAATTATGTGTGTTATTATATGTTGTCCTCAGCTCATTTATGCTTCTTACAATATGTGGAAATCT

           1171 1180      1190      1200      1210      1220      1230      1240      1250      1260      1270      1280      1290      1300
           |--------+---------+---------+---------+---------+---------+---------+---------+---------+---------+---------+---------+---------|
contig052987 TCTTGTGATAATTTCTATAATTTACTTTAGATATCTCCACACTCCTACAAACTACCTTATCCTCTCTATGGCTGTGGCTGATCTACTAATTGGTGCTTTAATATTTCCTTTGAGCATGACAGTGTCTCTA
 NyeTAR.B030 TCTTGTGATAATTTCTATAATTTACTTTAGATATCTCCACACTCCTACAAACTACCTTATCCTCTCTATGGCTGTGGCTGATCTACTAATTGGTGCTTTAATATTTCCTTTGAGCATGACAGTGTCTCTA
   Consensus TCTTGTGATAATTTCTATAATTTACTTTAGATATCTCCACACTCCTACAAACTACCTTATCCTCTCTATGGCTGTGGCTGATCTACTAATTGGTGCTTTAATATTTCCTTTGAGCATGACAGTGTCTCTA

           1301 1310      1320      1330      1340      1350      1360      1370      1380      1390      1400      1410      1420      1430
           |--------+---------+---------+---------+---------+---------+---------+---------+---------+---------+---------+---------+---------|
contig052987 AAGCCATGTTTGTATATATACAGTTTACTGTGCAACTTAAGAAGCACAATGGATGTAACAATGGGTGTATCTTCCTTATTAAATTTGTGCTGTATTTCTGTTGATCGATATTATGCTGTTTGCCACCCTC
 NyeTAR.B030 AAGCCATGTTTGTATATATACAGTTTACTGTGCAACTTAAGAAGCACAATGGATGTAACAATGGGTGTATCTTCCTTATTAAATTTGTGCTGTATTTCTGTTGATCGATATTATGCTGTTTGCCACCCTC
   Consensus AAGCCATGTTTGTATATATACAGTTTACTGTGCAACTTAAGAAGCACAATGGATGTAACAATGGGTGTATCTTCCTTATTAAATTTGTGCTGTATTTCTGTTGATCGATATTATGCTGTTTGCCACCCTC

           1431 1440      1450      1460      1470      1480      1490      1500      1510      1520      1530      1540      1550      1560
           |--------+---------+---------+---------+---------+---------+---------+---------+---------+---------+---------+---------+---------|
contig052987 TGATATATAAAACTAAAATAACTGATTGTGTTGCCATGAAGATGGGCCTTGGAAGTTGGGCTGTTGCTATCTTGTGTGGAATTTTTGTCTTCCTGCTGTTTTTTATTTTAGACGAATGTGACACAAGTTG
 NyeTAR.B030 TGATATATAAAACTAAAATAACTGATTGTGTTGCCATGAAGATGGGCCTTGGAAGTTGGGCTGTTGCTATCTTGTGTGGAATTTTTGTCTTCCTGCTGTTTTTTATTTTAGACGAATGTGACACAAGTTG
   Consensus TGATATATAAAACTAAAATAACTGATTGTGTTGCCATGAAGATGGGCCTTGGAAGTTGGGCTGTTGCTATCTTGTGTGGAATTTTTGTCTTCCTGCTGTTTTTTATTTTAGACGAATGTGACACAAGTTG

           1561 1570      1580      1590      1600      1610      1620      1630      1640      1650      1660      1670      1680      1690
           |--------+---------+---------+---------+---------+---------+---------+---------+---------+---------+---------+---------+---------|
contig052987 CGTTTTCGCTCTCATTGCCGCATCAGTGGTAGTATATTATATCCCAACAATAGTTTTACTTTTCATGTACACCAAAATTCTTGTTGTTGCACTGAGGCAGGCACGCAGCATCCATAATACAATTTCTCAG
 NyeTAR.B030 CGTTTTCGCTCTCATTGCCGCATCAGTGGTAGTATATTATATCCCAACAATAGTTTTACTTTTCATGTACACCAAAATTCTTGTTGTTGCACTGAGGCAGGCACGCAGCATCCATAATACAATTTCTCAG
   Consensus CGTTTTCGCTCTCATTGCCGCATCAGTGGTAGTATATTATATCCCAACAATAGTTTTACTTTTCATGTACACCAAAATTCTTGTTGTTGCACTGAGGCAGGCACGCAGCATCCATAATACAATTTCTCAG

           1691 1700      1710      1720      1730      1740      1750      1760      1770      1780      1790      1800      1810      1820
           |--------+---------+---------+---------+---------+---------+---------+---------+---------+---------+---------+---------+---------|
contig052987 AACACAAAGTCTAAAGCAGTTTCCAGTACGGAGAGAAAGGCCACAAAGACATTAACCATAGTTATTGGAATATTTTTAATTTTTTGGGTTCCTCTTTTTCTTAGTTATTCATTTGTTCCTCTGGATAGTT
 NyeTAR.B030 AACACAAAGTCTAAAGCAGTTTCCAGTACGGAGAGAAAGGCCACAAAGACATTAACCATAGTTATTGGAATATTTTTAATTTTTTGGGTTCCTCTTTTTCTTAGTTATTCATTTGTTCCTCTGGATAGTT
   Consensus AACACAAAGTCTAAAGCAGTTTCCAGTACGGAGAGAAAGGCCACAAAGACATTAACCATAGTTATTGGAATATTTTTAATTTTTTGGGTTCCTCTTTTTCTTAGTTATTCATTTGTTCCTCTGGATAGTT

           1821 1830      1840      1850      1860      1870      1880      1890      1900      1910      1920      1930      1940      1950
           |--------+---------+---------+---------+---------+---------+---------+---------+---------+---------+---------+---------+---------|
contig052987 TTATACTTTATGTGCTTCTTGAACCATTTAACTGGTTTGCAATTTCTAACTCAATGCTCAATCCCTTTATTTATGCTTTCTTTTACACTTGGTTTAGAAGAGCTTTTAAAATGATAATTTCTGGAAAAAT
 NyeTAR.B030 TTATACTTTATGTGCTTCTTGAACCATTTAACTGGTTTGCAATTTCTAACTCAATGCTCAATCCCTTTATTTATGCTTTCTTTTACACTTGGTTTAGAAGAGCTTTTAAAATGATAATTTCTGGAAAAAT
   Consensus TTATACTTTATGTGCTTCTTGAACCATTTAACTGGTTTGCAATTTCTAACTCAATGCTCAATCCCTTTATTTATGCTTTCTTTTACACTTGGTTTAGAAGAGCTTTTAAAATGATAATTTCTGGAAAAAT

           1951 1960      1970      1980      1990      2000      2010      2020      2030      2040      2050      2060      2070      2080
           |--------+---------+---------+---------+---------+---------+---------+---------+---------+---------+---------+---------+---------|
contig052987 ATTTCAAGGTGATGTTACTAACATAAAACTACACTGACAGCTATATATATATTATGAATTATATCAATAAGAGTAAATATCACCACAGTTGATCTTCTCTATGTTGTTCATTACAGTTTCAACTATGTAA
 NyeTAR.B030 ATTTCAAGGTGATGTTACTAACATAAAACTACACTGA
   Consensus ATTTCAAGGTGATGTTACTAACATAAAACTACACTGA.............................................................................................
```

```
             1691  1700      1710      1720      1730      1740      1750      1760      1770      1780      1790      1800      1810      1820
             |--------+---------+---------+---------+---------+---------+---------+---------+---------+---------+---------+---------+---------|
contig045302 GTAAAAATTACAGGGTGGTGTTAGAAGAGGATAAAGGGACAACCTGGCAAATAGTTCAACCTCTTCCCTTTGACTGCATGCACATGCATGCACCGCTCCTCTCCGATGGATAGCTCTGGAGGTCCTCCCC
 BurTAR.A001                                                                                                          ATGGATAGCTCTGGAGGTCCTCCCC
   Consensus .........................................................................................................ATGGATAGCTCTGGAGGTCCTCCCC

             1821  1830      1840      1850      1860      1870      1880      1890      1900      1910      1920      1930      1940      1950
             |--------+---------+---------+---------+---------+---------+---------+---------+---------+---------+---------+---------+---------|
contig045302 TCTGCTTCCCCAACCTCAACTCCTCCTGCAGGCGGCTGCTGCGACCCACCTCCCAGACCGTTGTTCTCTACACTCTACTGGCCTCCATCTCCCTGCTCACTGTGGTGCTCAACCTGCTTGTCGTCATCTC
 BurTAR.A001 TCTGCTTCCCCAACCTCAACTCCTCCTGCAGGCGGCTGCTGCGACCCACCTCCCAGACCGTTGTTCTCTACACTCTACTGGCCTCCATCTCCCTGCTCACTGTGGTGCTCAACCTGCTTGTCGTCATCTC
   Consensus TCTGCTTCCCCAACCTCAACTCCTCCTGCAGGCGGCTGCTGCGACCCACCTCCCAGACCGTTGTTCTCTACACTCTACTGGCCTCCATCTCCCTGCTCACTGTGGTGCTCAACCTGCTTGTCGTCATCTC

             1951  1960      1970      1980      1990      2000      2010      2020      2030      2040      2050      2060      2070      2080
             |--------+---------+---------+---------+---------+---------+---------+---------+---------+---------+---------+---------+---------|
contig045302 CATCTCCCACTTCAGACAGCTTAACACCTCGACCAACACCCTGCTCCTTTCCCTGGCTGTGTCTGACCTGCTGGTGGGGCTGCTGGTGATGCCCATCGAGGGCCTGTACTACATCGAAACGTGCTGGCTG
 BurTAR.A001 CATCTCCCACTTCAGACAGCTTAACACCTCGACCAACACCCTGCTCCTTTCCCTGGCTGTGTCTGACCTGCTGGTGGGGCTGCTGGTGATGCCCATCGAGGGCCTGTACTACATCGAAACGTGCTGGCTG
   Consensus CATCTCCCACTTCAGACAGCTTAACACCTCGACCAACACCCTGCTCCTTTCCCTGGCTGTGTCTGACCTGCTGGTGGGGCTGCTGGTGATGCCCATCGAGGGCCTGTACTACATCGAAACGTGCTGGCTG

             2081  2090      2100      2110      2120      2130      2140      2150      2160      2170      2180      2190      2200      2210
             |--------+---------+---------+---------+---------+---------+---------+---------+---------+---------+---------+---------+---------|
contig045302 CTGGGGAGGCTGATGTGTGCTCTCAGTCCTTATTTGTCTTACTGCCTCATCTCTGTCTCTCTGGACAGCATGGTGCTCATATCTGTAGATCGCTATATAGCCATCTGTGACCCTCTGCTCTATTCCTCAA
 BurTAR.A001 CTGGGGAGGCTGATGTGTGCTCTCAGTCCTTATTTGTCTTACTGCCTCATCTCTGTCTCTCTGGACAGCATGGTGCTCATATCTGTAGATCGCTATATAGCCATCTGTGACCCTCTGCTCTATTCCTCAA
   Consensus CTGGGGAGGCTGATGTGTGCTCTCAGTCCTTATTTGTCTTACTGCCTCATCTCTGTCTCTCTGGACAGCATGGTGCTCATATCTGTAGATCGCTATATAGCCATCTGTGACCCTCTGCTCTATTCCTCAA

             2211  2220      2230      2240      2250      2260      2270      2280      2290      2300      2310      2320      2330      2340
             |--------+---------+---------+---------+---------+---------+---------+---------+---------+---------+---------+---------+---------|
contig045302 AGATCACAGTGAACAGAGTGAAGCTTTCAGTCTGTGTCTGCTGGGCCTGCTCTCTTCTCTACAATGGCTGCATTCTCATGGAGCACATAGGGTGGCCAGACAGGTTCAGCTCCTGTCACGGGGAGTGTGT
 BurTAR.A001 AGATCACAGTGAACAGAGTGAAGCTTTCAGTCTGTGTCTGCTGGGCCTGCTCTCTTCTCTACAATGGCTGCATTCTCATGGAGCACATAGGGTGGCCAGACAGGTTCAGCTCCTGTCACGGGGAGTGTGT
   Consensus AGATCACAGTGAACAGAGTGAAGCTTTCAGTCTGTGTCTGCTGGGCCTGCTCTCTTCTCTACAATGGCTGCATTCTCATGGAGCACATAGGGTGGCCAGACAGGTTCAGCTCCTGTCACGGGGAGTGTGT

             2341  2350      2360      2370      2380      2390      2400      2410      2420      2430      2440      2450      2460      2470
             |--------+---------+---------+---------+---------+---------+---------+---------+---------+---------+---------+---------+---------|
contig045302 TGTGTTCATCAGCCGTGCTTTGGGTACAATAGATCTCTTTTTCTCATTTCTCGGCCCATGTGCTTTAATGTTTGTTCTGTACATGAGGGTCTTTGTGGTTGCTGTTTCTCAGGTACGTATCATTCGGTCG
 BurTAR.A001 TGTGTTCATCAGCCGTGCTTTGGGTACAATAGATCTCTTTTTCTCATTTCTCGGCCCATGTGCTTTAATGTTTGTTCTGTACATGAGGGTCTTTGTGGTTGCTGTTTCTCAGGTACGTATCATTCGGTCG
   Consensus TGTGTTCATCAGCCGTGCTTTGGGTACAATAGATCTCTTTTTCTCATTTCTCGGCCCATGTGCTTTAATGTTTGTTCTGTACATGAGGGTCTTTGTGGTTGCTGTTTCTCAGGTACGTATCATTCGGTCG

             2471  2480      2490      2500      2510      2520      2530      2540      2550      2560      2570      2580      2590      2600
             |--------+---------+---------+---------+---------+---------+---------+---------+---------+---------+---------+---------+---------|
contig045302 CAGGCAGCTGTGAGAGCACCTCCAGCTGCTAAAAAATCAGAGCTGAAGGCAGCCAGGACACTCGGGATTTTGATAGCTGTGTTTCTAATGTGCTTCTGCCCTTATTATTATCCTTCCTTTGCAGGTGACG
 BurTAR.A001 CAGGCAGCTGTGAGAGCACCTCCAGCTGCTAAAAAATCAGAGCTGAAGGCAGCCAGGACACTCGGGATTTTGATAGCTGTGTTTCTAATGTGCTTCTGCCCTTATTATTATCCTTCCTTTGCAGGTGACG
   Consensus CAGGCAGCTGTGAGAGCACCTCCAGCTGCTAAAAAATCAGAGCTGAAGGCAGCCAGGACACTCGGGATTTTGATAGCTGTGTTTCTAATGTGCTTCTGCCCTTATTATTATCCTTCCTTTGCAGGTGACG

             2601  2610      2620      2630      2640      2650      2660      2670      2680      2690      2700      2710      2720      2730
             |--------+---------+---------+---------+---------+---------+---------+---------+---------+---------+---------+---------+---------|
contig045302 ACACCTCTATGAACTTACCTTATTACGCTCTATTCTCGTGGATAATGCTGACAAATTCTTGTGTGAACCCTGTGATTTATGCTCTGTTCTACCCCTGGTTTAGAAGAGCTATCAGACTCATTGTCACCCT
 BurTAR.A001 ACACCTCTATGAACTTACCTTATTACGCTCTATTCTCGTGGATAATGCTGACAAATTCTTGTGTGAACCCTGTGATTTATGCTCTGTTCTACCCCTGGTTTAGAAGAGCTATCAGACTCATTGTCACCCT
   Consensus ACACCTCTATGAACTTACCTTATTACGCTCTATTCTCGTGGATAATGCTGACAAATTCTTGTGTGAACCCTGTGATTTATGCTCTGTTCTACCCCTGGTTTAGAAGAGCTATCAGACTCATTGTCACCCT

             2731  2740      2750      2760      2770      2780      2790      2800      2810      2820      2830      2840      2850      2860
             |--------+---------+---------+---------+---------+---------+---------+---------+---------+---------+---------+---------+---------|
contig045302 CAGAATACTGCAGCCTCACTCCAGAGATGTCAAGATCCTGTAGGCAGCAGTAAAACAAACCAGTCATTAGTCTTTTCTTAAAAGAATAATCAAAAAACAACTATGCCACTGCTCCACAGAGATCCTTCAC
 BurTAR.A001 CAGAATACTGCAGCCTCACTCCAGAGATGTCAAGATCCTGTAG
   Consensus CAGAATACTGCAGCCTCACTCCAGAGATGTCAAGATCCTGTAG.......................................................................................
```

```
 Consensus  ..................................................................................................................................

            8451  8460      8470      8480      8490      8500      8510      8520      8530      8540      8550      8560      8570      8580
            |--------+---------+---------+---------+---------+---------+---------+---------+---------+---------+---------+---------+---------|
contig020038  GGTTGACTCTGGAGTTGGAAGGGGGGAGTGTCATCAGAGATAAATAAGCTGTACAATGTCTCACAGGGTCAGCAGGTCCTCCTTTCTTTATACTGGATGATGGACAGGCAGGACAGAATTGAACTCTGCT
  BurTAR.A002                                                                                                     ATGGACAGGCAGGACAGAATTGAACTCTGCT
    Consensus  ...................................................................................................ATGGACAGGCAGGACAGAATTGAACTCTGCT

            8581  8590      8600      8610      8620      8630      8640      8650      8660      8670      8680      8690      8700      8710
            |--------+---------+---------+---------+---------+---------+---------+---------+---------+---------+---------+---------+---------|
contig020038  ACCCTGAGCTTGCGAATCTGTCTTGCAGGGGTTTAACACGTCCTCAGCCTGAGGCTTTCCTCCTCTATACACTGTTCTTGTCTATCTCTGTGCTAACTGTAGCTCTGAATGTGCTGGTTATCATATCCAT
  BurTAR.A002  ACCCTGAGCTTGCGAATCTGTCTTGCAGGGGTTTAACACGTCCTCAGCCTGAGGCTTTCCTCCTCTATACACTGTTCTTGTCTATCTCTGTGCTAACTGTAGCTCTGAATGTGCTGGTTATCATATCCAT
    Consensus  ACCCTGAGCTTGCGAATCTGTCTTGCAGGGGTTTAACACGTCCTCAGCCTGAGGCTTTCCTCCTCTATACACTGTTCTTGTCTATCTCTGTGCTAACTGTAGCTCTGAATGTGCTGGTTATCATATCCAT

            8711  8720      8730      8740      8750      8760      8770      8780      8790      8800      8810      8820      8830      8840
            |--------+---------+---------+---------+---------+---------+---------+---------+---------+---------+---------+---------+---------|
contig020038  CTCTCATTTCAGGCAGCTCCACATGCCCACCAATGTCCTCCTGCTCTCTCTGGCCATCTCAGACCTCTTGGTCGGGCTGCTTGTGATGCCGGTGGAAGCAATGCGGTTTATAGAAACCTGCTGGATGCTG
  BurTAR.A002  CTCTCATTTCAGGCAGCTCCACATGCCCACCAATGTCCTCCTGCTCTCTCTGGCCATCTCAGACCTCTTGGTCGGGCTGCTTGTGATGCCGGTGGAAGCAATGCGGTTTATAGAAACCTGCTGGATGCTG
    Consensus  CTCTCATTTCAGGCAGCTCCACATGCCCACCAATGTCCTCCTGCTCTCTCTGGCCATCTCAGACCTCTTGGTCGGGCTGCTTGTGATGCCGGTGGAAGCAATGCGGTTTATAGAAACCTGCTGGATGCTG

            8841  8850      8860      8870      8880      8890      8900      8910      8920      8930      8940      8950      8960      8970
            |--------+---------+---------+---------+---------+---------+---------+---------+---------+---------+---------+---------+---------|
contig020038  GGAGATCTCATGTGTGCTTTTACTTACATTATCGGTTTCACTCTCACCTCAGCCTCTGTGGGCAACATGGTGCTCATATCAATTGATCGCTACGTAGCTATTTGTTATCCTCTGCAGTACCCCACTAAAA
  BurTAR.A002  GGAGATCTCATGTGTGCTTTTACTTACATTATCGGTTTCACTCTCACCTCAGCCTCTGTGGGCAACATGGTGCTCATATCAATTGATCGCTACGTAGCTATTTGTTATCCTCTGCAGTACCCCACTAAAA
    Consensus  GGAGATCTCATGTGTGCTTTTACTTACATTATCGGTTTCACTCTCACCTCAGCCTCTGTGGGCAACATGGTGCTCATATCAATTGATCGCTACGTAGCTATTTGTTATCCTCTGCAGTACCCCACTAAAA

            8971  8980      8990      9000      9010      9020      9030      9040      9050      9060      9070      9080      9090      9100
            |--------+---------+---------+---------+---------+---------+---------+---------+---------+---------+---------+---------+---------|
contig020038  TCACTCACAGCAGAGCGGAGCTGTCTGTGACTCTGTGCTGGGCCTGTTCGCTCCTCTACAACGGGATGATCTTAAAGGAGCATCTCAGGCAGCCAGACAGGCATAACACCTGTCACGGTCAGTGTTTGGT
  BurTAR.A002  TCACTCACAGCAGAGCGGAGCTGTCTGTGACTCTGTGCTGGGCCTGTTCGCTCCTCTACAACGGGATGATCTTAAAGGAGCATCTCAGGCAGCCAGACAGGCATAACACCTGTCACGGTCAGTGTTTGGT
    Consensus  TCACTCACAGCAGAGCGGAGCTGTCTGTGACTCTGTGCTGGGCCTGTTCGCTCCTCTACAACGGGATGATCTTAAAGGAGCATCTCAGGCAGCCAGACAGGCATAACACCTGTCACGGTCAGTGTTTGGT

            9101  9110      9120      9130      9140      9150      9160      9170      9180      9190      9200      9210      9220      9230
            |--------+---------+---------+---------+---------+---------+---------+---------+---------+---------+---------+---------+---------|
contig020038  GGTGATTAACTATGTCTCTGGAGCCATCGACCTTGTCTTTACTTTCATCGGCCCTTGCTCAGTCATTATCATTCTGTACATGAGAGTATTTGTTGTAGCGCTGTCTCAGGCTCATGCCATGCGGTCTCAT
  BurTAR.A002  GGTGATTAACTATGTCTCTGGAGCCATCGACCTTGTCTTTACTTTCATCGGCCCTTGCTCAGTCATTATCATTCTGTACATGAGAGTATTTGTTGTAGCGCTGTCTCAGGCTCATGCCATGCGGTCTCAT
    Consensus  GGTGATTAACTATGTCTCTGGAGCCATCGACCTTGTCTTTACTTTCATCGGCCCTTGCTCAGTCATTATCATTCTGTACATGAGAGTATTTGTTGTAGCGCTGTCTCAGGCTCATGCCATGCGGTCTCAT

            9231  9240      9250      9260      9270      9280      9290      9300      9310      9320      9330      9340      9350      9360
            |--------+---------+---------+---------+---------+---------+---------+---------+---------+---------+---------+---------+---------|
contig020038  ATTACAGCTGCTGCAGCTGGTACAGTAAAAATCACCGCAAAGAAATCAGAGAAAAAGGCAGCCAGGACTCTTGGCGTTGTGATATTTGTGTTTTTGATGAGTTTCTGTCCGTATTACTATCCCTCTCTTG
  BurTAR.A002  ATTACAGCTGCTGCAGCTGGTACAGTAAAAATCACCGCAAAGAAATCAGAGAAAAAGGCAGCCAGGACTCTTGGCGTTGTGATATTTGTGTTTTTGATGAGTTTCTGTCCGTATTACTATCCCTCTCTTG
    Consensus  ATTACAGCTGCTGCAGCTGGTACAGTAAAAATCACCGCAAAGAAATCAGAGAAAAAGGCAGCCAGGACTCTTGGCGTTGTGATATTTGTGTTTTTGATGAGTTTCTGTCCGTATTACTATCCCTCTCTTG

            9361  9370      9380      9390      9400      9410      9420      9430      9440      9450      9460      9470      9480      9490
            |--------+---------+---------+---------+---------+---------+---------+---------+---------+---------+---------+---------+---------|
contig020038  CAGGGCAGGACATTTCAAACAGTGCTTCATCTTGGGCTATTGTTTCCTGGATGCTGTACTTCAATTCTTGTCTGAATCCACTGATATATGCTTTTTTCTACCCGTGGTTTAGAAAAGCTATCAGGTTCAT
  BurTAR.A002  CAGGGCAGGACATTTCAAACAGTGCTTCATCTTGGGCTATTGTTTCCTGGATGCTGTACTTCAATTCTTGTCTGAATCCACTGATATATGCTTTTTTCTACCCGTGGTTTAGAAAAGCTATCAGGTTCAT
    Consensus  CAGGGCAGGACATTTCAAACAGTGCTTCATCTTGGGCTATTGTTTCCTGGATGCTGTACTTCAATTCTTGTCTGAATCCACTGATATATGCTTTTTTCTACCCGTGGTTTAGAAAAGCTATCAGGTTCAT

            9491  9500      9510      9520      9530      9540      9550      9560      9570      9580      9590      9600      9610      9620
            |--------+---------+---------+---------+---------+---------+---------+---------+---------+---------+---------+---------+---------|
contig020038  TGTCTCCCTGAAGATACTAGAGAAGGGCTCCTCTCAGGCAAATATACTTTAAAATAGCAAATTTATCCTGAACCAAATCAGTCTGTATGAGAGAATGAAACTGCTGATTATTATATGTGGCAGTAGCATG
  BurTAR.A002  TGTCTCCCTGAAGATACTAGAGAAGGGCTCCTCTCAGGCAAATATACTTTAA
    Consensus  TGTCTCCCTGAAGATACTAGAGAAGGGCTCCTCTCAGGCAAATATACTTTAA..............................................................................

            9621  9630      9640      9650      9660      9670      9680      9690      9700      9710      9720      9730      9740      9750
            |--------+---------+---------+---------+---------+---------+---------+---------+---------+---------+---------+---------+---------|
```

```
            2731     2740      2750      2760      2770      2780      2790      2800      2810      2820      2830      2840      2850      2860
            |--------+---------+---------+---------+---------+---------+---------+---------+---------+---------+---------+---------+---------|
contig061977 AGTTATATAACTAATTCTGTTTGTAATGCTACATAGAGCTACAGGTCTCTCTGAAAATGGATACACAGGATGTGGCAGAGCTCTGTTTTCCACAACTCTTCAACACCTCCTGCAAGAAACCTCTGTCAGA
BurTARs.A012                                                         ATGGATACACAGGATGTGGCAGAGCTCTGTTTTCCACAACTCTTCAACACCTCCTGCAAGAAACCTCTGTCAGA
   Consensus ........................................................ATGGATACACAGGATGTGGCAGAGCTCTGTTTTCCACAACTCTTCAACACCTCCTGCAAGAAACCTCTGTCAGA

            2861     2870      2880      2890      2900      2910      2920      2930      2940      2950      2960      2970      2980      2990
            |--------+---------+---------+---------+---------+---------+---------+---------+---------+---------+---------+---------+---------|
contig061977 ATTTGTGTTCCTTCATGTTGTGTTGTCCTCCATCTCGCTGCTAACTGTGACTCTCAACCTGCTCGTCATCATCTCAGTCTCCCACTACAGGCAGAGTTGTTGTTATTTTTCTAATAGCTGTGTGTAGCTT
BurTARs.A012 ATTTGTGTTCCTTCATGTTGTGTTGTCCTCCATCTCGCTGCTAACTGTGACTCTCAACCTGCTCGTCATCATCTCAGTCTCCCACTACAGGCAG------------------------------------
   Consensus ATTTGTGTTCCTTCATGTTGTGTTGTCCTCCATCTCGCTGCTAACTGTGACTCTCAACCTGCTCGTCATCATCTCAGTCTCCCACTACAGGCAG....................................

            2991     3000      3010      3020      3030      3040      3050      3060      3070      3080      3090      3100      3110      3120
            |--------+---------+---------+---------+---------+---------+---------+---------+---------+---------+---------+---------+---------|
contig061977 AAACATTCTGTGACCTAATTGACTTAAGATGTTTTTGTGACCTCAGTGGTGTATATAATCAAACTATCTGATATAGAAGTGGTCGATTAGAAATGATCAAAACTGTTTTTCTCTTACCAAGAGCTAATGT
BurTARs.A012 ----------------------------------------------------------------------------------------------------------------------------------
   Consensus ..................................................................................................................................

            3121     3130      3140      3150      3160      3170      3180      3190      3200      3210      3220      3230      3240      3250
            |--------+---------+---------+---------+---------+---------+---------+---------+---------+---------+---------+---------+---------|
contig061977 TTTATTGTTTGTTGGGTTGCTTTAATTTAGCGATTTGAGGACTGTTAATTTAATAATTTTTGTGTCTTCTTGCAGGCAGCTCCACACACCCACTAACATCCTCCTCCTCTCTCTGGCTGTCTCAGACTTT
BurTARs.A012 -------------------------------------------------------------------------------CTCCACACACCCACTAACATCCTCCTCCTCTCTCTGGCTGTCTCAGACTTT
   Consensus ...............................................................................CTCCACACACCCACTAACATCCTCCTCCTCTCTCTGGCTGTCTCAGACTTT

            3251     3260      3270      3280      3290      3300      3310      3320      3330      3340      3350      3360      3370      3380
            |--------+---------+---------+---------+---------+---------+---------+---------+---------+---------+---------+---------+---------|
contig061977 CTTGTTGGTCTCCTATTGATGCCTGGAGAAATCCTCCGAAATACAGCCTGCTGGTTTCTCGGTGACCTCACCTGTTCTATGTACAATTATATGTCTTTAATTGTTACCTCTACCTCAGTGGGAGACATGG
BurTARs.A012 CTTGTTGGTCTCCTATTGATGCCTGGAGAAATCCTCCGAAATACAGCCTGCTGGTTTCTCGGTGACCTCACCTGTTCTATGTACAATTATATGTCTTTAATTGTTACCTCTACCTCAGTGGGAGACATGG
   Consensus CTTGTTGGTCTCCTATTGATGCCTGGAGAAATCCTCCGAAATACAGCCTGCTGGTTTCTCGGTGACCTCACCTGTTCTATGTACAATTATATGTCTTTAATTGTTACCTCTACCTCAGTGGGAGACATGG

            3381     3390      3400      3410      3420      3430      3440      3450      3460      3470      3480      3490      3500      3510
            |--------+---------+---------+---------+---------+---------+---------+---------+---------+---------+---------+---------+---------|
contig061977 TGTTAATATCAATTGACCGCTATGTGGCTATTTGTGACCCTCTGCATTACCCCACCAGAATCACAGACAGAAGAGTGAAACTCTGTGTTTGTCTGTGTTGGCTCTGCTCTGTTTTCTATAGCAGCCTGTT
BurTARs.A012 TGTTAATATCAATTGACCGCTATGTGGCTATTTGTGACCCTCTGCATTACCCCACCAGAATCACAGACAGAAGAGTGAAACTCTGTGTTTGTCTGTGTTGGCTCTGCTCTGTTTTCTATAGCAGCCTGTT
   Consensus TGTTAATATCAATTGACCGCTATGTGGCTATTTGTGACCCTCTGCATTACCCCACCAGAATCACAGACAGAAGAGTGAAACTCTGTGTTTGTCTGTGTTGGCTCTGCTCTGTTTTCTATAGCAGCCTGTT

            3511     3520      3530      3540      3550      3560      3570      3580      3590      3600      3610      3620      3630      3640
            |--------+---------+---------+---------+---------+---------+---------+---------+---------+---------+---------+---------+---------|
contig061977 TGTAAAGGATGATCTAACTCAACCAGGGAAGCATAATTCCTGCTATGGAGAATGTACAATTGTTGTTGACTTAATTACAGGAACGATTGACCTTCTTTTAACCTTTTTTGTTCCAGTTACTGTCATTGTA
BurTARs.A012 TGTAAAGGATGATCTAACTCAACCAGGGAAGCATAATTCCTGCTATGGAGAATGTACAATTGTTGTTGACTTAATTACAGGAACGATTGACCTTCTTTTAACCTTTTTTGTTCCAGTTACTGTCATTGTA
   Consensus TGTAAAGGATGATCTAACTCAACCAGGGAAGCATAATTCCTGCTATGGAGAATGTACAATTGTTGTTGACTTAATTACAGGAACGATTGACCTTCTTTTAACCTTTTTTGTTCCAGTTACTGTCATTGTA

            3641     3650      3660      3670      3680      3690      3700      3710      3720      3730      3740      3750      3760      3770
            |--------+---------+---------+---------+---------+---------+---------+---------+---------+---------+---------+---------+---------|
contig061977 GTTCTGTATCTGAGAGTATTTGTGGTGGCTGTGTCTCAGGCTCGCGCCATGCGCTCTCATGTTACAGTTGCTGCTCTGCAGCTTTCAGTGACTCTAACAACAAAGAAATCAGAGTTAAAAGCAGCCAGGA
BurTARs.A012 GTTCTGTATCTGAGAGTATTTGTGGTGGCTGTGTCTCAGGCTCGCGCCATGCGCTCTCATGTTACAGTTGCTGCTCTGCAGCTTTCAGTGACTCTAACAACAAAGAAATCAGAGTTAAAAGCAGCCAGGA
   Consensus GTTCTGTATCTGAGAGTATTTGTGGTGGCTGTGTCTCAGGCTCGCGCCATGCGCTCTCATGTTACAGTTGCTGCTCTGCAGCTTTCAGTGACTCTAACAACAAAGAAATCAGAGTTAAAAGCAGCCAGGA

            3771     3780      3790      3800      3810      3820      3830      3840      3850      3860      3870      3880      3890      3900
            |--------+---------+---------+---------+---------+---------+---------+---------+---------+---------+---------+---------+---------|
contig061977 CTCTGGGTGTTCTTGTAGTTGTGTTTCTACTGTGTTTCTGCCCTTATTATTGTGTTACTCTTGCCAGGGACGACCTACTCAATAGTTCATCTGTATCCTTTTTGCTCTATCTGTTCTATTTTAACTCATG
BurTARs.A012 CTCTGGGTGTTCTTGTAGTTGTGTTTCTACTGTGTTTCTGCCCTTATTATTGTGTTACTCTTGCCAGGGACGACCTACTCAATAGTTCATCTGTATCCTTTTTGCTCTATCTGTTCTATTTTAACTCATG
   Consensus CTCTGGGTGTTCTTGTAGTTGTGTTTCTACTGTGTTTCTGCCCTTATTATTGTGTTACTCTTGCCAGGGACGACCTACTCAATAGTTCATCTGTATCCTTTTTGCTCTATCTGTTCTATTTTAACTCATG

            3901     3910      3920      3930      3940      3950      3960      3970      3980      3990      4000      4010      4020      4030
            |--------+---------+---------+---------+---------+---------+---------+---------+---------+---------+---------+---------+---------|
contig061977 TCTAAACCCTTTGATCTATGCACTGCTCTACCCCTGGTTTAGAAAAGCTGTGAAACTCATAATCTCTTTACACATACTGCAGCCTGGTTCCTGTGAGATTAGCATTCTGTAAAAAAAAACAAACAAAAAA
BurTARs.A012 TCTAAACCCTTTGATCTATGCACTGCTCTACCCCTGGTTTAGAAAAGCTGTGAAACTCATAATCTCTTTACACATACTGCAGCCTGGTTCCTGTGAGATTAGCATTCTGTAA
   Consensus TCTAAACCCTTTGATCTATGCACTGCTCTACCCCTGGTTTAGAAAAGCTGTGAAACTCATAATCTCTTTACACATACTGCAGCCTGGTTCCTGTGAGATTAGCATTCTGTAA..................
```

```
              911     920       930       940       950       960       970       980       990       1000      1010      1020      1030      1040
              |--------+---------+---------+---------+---------+---------+---------+---------+---------+---------+---------+---------+--------|
contig061433  GGATGGGAGGTATGGTAGAAGGAGATAAATTTGCTGTATGACAGAAAGTTCTGTCCAGACAGCTGCAGGAACCTGATGATGGAGATCCAGATGCATCCAGAGGCAGAGCTCTGTTTTCCAGAGCTACCGA
BurTARs.A013                                                                                ATGGAGATCCAGATGCATCCAGAGGCAGAGCTCTGTTTTCCAGAGCTACCGA
   Consensus  ..............................................................................ATGGAGATCCAGATGCATCCAGAGGCAGAGCTCTGTTTTCCAGAGCTACCGA

              1041    1050      1060      1070      1080      1090      1100      1110      1120      1130      1140      1150      1160      1170
              |--------+---------+---------+---------+---------+---------+---------+---------+---------+---------+---------+---------+--------|
contig061433  ACAGTTCCTGCAGGAAGCCGACACTTCACTGGTCCAAAACGGTGCTCCTGAACGTTGGGCTTTCGTCCATCTCTGTGATCACTGCTGCTCTTAATCTGCTCATCATCATCTCAGTCTACCACTTCAGGCA
BurTARs.A013  ACAGTTCCTGCAGGAAGCCGACACTTCACTGGTCCAAAACGGTGCTCCTGAACGTTGGGCTTTCGTCCATCTCTGTGATCACTGCTGCTCTTAATCTGCTCATCATCATCTCAGTCTACCACTTCAGGCA
   Consensus  ACAGTTCCTGCAGGAAGCCGACACTTCACTGGTCCAAAACGGTGCTCCTGAACGTTGGGCTTTCGTCCATCTCTGTGATCACTGCTGCTCTTAATCTGCTCATCATCATCTCAGTCTACCACTTCAGGCA

              1171    1180      1190      1200      1210      1220      1230      1240      1250      1260      1270      1280      1290      1300
              |--------+---------+---------+---------+---------+---------+---------+---------+---------+---------+---------+---------+--------|
contig061433  GAGATTAACCTTTCAGATGAAACTAAAGTTCAACTTTTAGAAAATAATGTTACATTGACATCATATTCTTCAGTAATGTTTGAATTATGTCAAGATGAATGTCCTGTGTGTACCTAATCATAACTGTATA
BurTARs.A013  G---------------------------------------------------------------------------------------------------------------------------------
   Consensus  G.................................................................................................................................

              1301    1310      1320      1330      1340      1350      1360      1370      1380      1390      1400      1410      1420      1430
              |--------+---------+---------+---------+---------+---------+---------+---------+---------+---------+---------+---------+--------|
contig061433  GTTTTATTTCCTTAATTGCACCATGCCTCAGCAATGGAACATTTCCATTGAAGACATTTGACATGATTAATGGGTAATTTGTAATTTTCTGTCTCCCTGCAGGCAGCTCCACACTCCCAGTAACATCCTC
BurTARs.A013  ----------------------------------------------------------------------------------------------------------CTCCACACTCCCAGTAACATCCTC
   Consensus  ..........................................................................................................CTCCACACTCCCAGTAACATCCTC

              1431    1440      1450      1460      1470      1480      1490      1500      1510      1520      1530      1540      1550      1560
              |--------+---------+---------+---------+---------+---------+---------+---------+---------+---------+---------+---------+--------|
contig061433  CTCCTCTCTCTGGCTGTCTCTGACTTTTTTGTGGGTTTTCTGTTGCTGCCGGTAGAAATCTTCAAAAACACGGCCTGCTGGGTATTTGGTGATCTCATGTGTTCACTTTATATTTATCTGAGCGGCATTC
BurTARs.A013  CTCCTCTCTCTGGCTGTCTCTGACTTTTTTGTGGGTTTTCTGTTGCTGCCGGTAGAAATCTTCAAAAACACGGCCTGCTGGGTATTTGGTGATCTCATGTGTTCACTTTATATTTATCTGAGCGGCATTC
   Consensus  CTCCTCTCTCTGGCTGTCTCTGACTTTTTTGTGGGTTTTCTGTTGCTGCCGGTAGAAATCTTCAAAAACACGGCCTGCTGGGTATTTGGTGATCTCATGTGTTCACTTTATATTTATCTGAGCGGCATTC

              1561    1570      1580      1590      1600      1610      1620      1630      1640      1650      1660      1670      1680      1690
              |--------+---------+---------+---------+---------+---------+---------+---------+---------+---------+---------+---------+--------|
contig061433  TTATGAATGCTTCAATTGAAATTATTGTTTTTATATCAGTTGACCGCTATGTGGCTATTTGTGACCCTCTGCATTACCCCACCAGAATCACTGTGACACGAGTCAAACTCAGTGTTTGTCTGTGTTGGTT
BurTARs.A013  TTATGAATGCTTCAATTGAAATTATTGTTTTTATATCAGTTGACCGCTATGTGGCTATTTGTGACCCTCTGCATTACCCCACCAGAATCACTGTGACACGAGTCAAACTCAGTGTTTGTCTGTGTTGGTT
   Consensus  TTATGAATGCTTCAATTGAAATTATTGTTTTTATATCAGTTGACCGCTATGTGGCTATTTGTGACCCTCTGCATTACCCCACCAGAATCACTGTGACACGAGTCAAACTCAGTGTTTGTCTGTGTTGGTT

              1691    1700      1710      1720      1730      1740      1750      1760      1770      1780      1790      1800      1810      1820
              |--------+---------+---------+---------+---------+---------+---------+---------+---------+---------+---------+---------+--------|
contig061433  TTATGCTATTTTCTACATGAGTCTTTACACAAAGGATGTCTTGATAAAGCCAGGCAGGTATGCTTCCTGCTATGGAGAGTGTGTGTTTTTTATTGAAGATATAACTGGAACTGTTGACATTGTTTTATGT
BurTARs.A013  TTATGCTATTTTCTACATGAGTCTTTACACAAAGGATGTCTTGATAAAGCCAGGCAGGTATGCTTCCTGCTATGGAGAGTGTGTGTTTTTTATTGAAGATATAACTGGAACTGTTGACATTGTTTTATGT
   Consensus  TTATGCTATTTTCTACATGAGTCTTTACACAAAGGATGTCTTGATAAAGCCAGGCAGGTATGCTTCCTGCTATGGAGAGTGTGTGTTTTTTATTGAAGATATAACTGGAACTGTTGACATTGTTTTATGT

              1821    1830      1840      1850      1860      1870      1880      1890      1900      1910      1920      1930      1940      1950
              |--------+---------+---------+---------+---------+---------+---------+---------+---------+---------+---------+---------+--------|
contig061433  TTTTTTGTTCCAGTTATTATCATCATTGTTCTGTATACAAGAGTATTTGTAGTGGCAGTGTCTCAGGCCCGTGCCATGCGCTCTCATGTTACAGCTGTCACACTTCAGCGTTCATTGAATCAATCAAACA
BurTARs.A013  TTTTTTGTTCCAGTTATTATCATCATTGTTCTGTATACAAGAGTATTTGTAGTGGCAGTGTCTCAGGCCCGTGCCATGCGCTCTCATGTTACAGCTGTCACACTTCAGCGTTCATTGAATCAATCAAACA
   Consensus  TTTTTTGTTCCAGTTATTATCATCATTGTTCTGTATACAAGAGTATTTGTAGTGGCAGTGTCTCAGGCCCGTGCCATGCGCTCTCATGTTACAGCTGTCACACTTCAGCGTTCATTGAATCAATCAAACA

              1951    1960      1970      1980      1990      2000      2010      2020      2030      2040      2050      2060      2070      2080
              |--------+---------+---------+---------+---------+---------+---------+---------+---------+---------+---------+---------+--------|
contig061433  AATCTGAGCTGAAAGCAGCCAGGAACCTTGGAGTTCTTGTAATTGTGTTCCTGGCAAGTATTTGCCCATTTTACTTCTACTCTCTTGTTGACGGCAATGTGGTCAAAGCTTCATCTGCGACTTTTTTGAT
BurTARs.A013  AATCTGAGCTGAAAGCAGCCAGGAACCTTGGAGTTCTTGTAATTGTGTTCCTGGCAAGTATTTGCCCATTTTACTTCTACTCTCTTGTTGACGGCAATGTGGTCAAAGCTTCATCTGCGACTTTTTTGAT
   Consensus  AATCTGAGCTGAAAGCAGCCAGGAACCTTGGAGTTCTTGTAATTGTGTTCCTGGCAAGTATTTGCCCATTTTACTTCTACTCTCTTGTTGACGGCAATGTGGTCAAAGCTTCATCTGCGACTTTTTTGAT

              2081    2090      2100      2110      2120      2130      2140      2150      2160      2170      2180      2190      2200      2210
              |--------+---------+---------+---------+---------+---------+---------+---------+---------+---------+---------+---------+--------|
contig061433  CATTGTTTATTTTAACGCTTGTTTAAACCCACTGATCTATACCCTGTTTTACCCCTGGTTTAGAAATGCTGTTAAACTTATTATCACCCTGCAGATATTCAAGCATAACAGTAGTGAAGCCAACATACTA
BurTARs.A013  CATTGTTTATTTTAACGCTTGTTTAAACCCACTGATCTATACCCTGTTTTACCCCTGGTTTAGAAATGCTGTTAAACTTATTATCACCCTGCAGATATTCAAGCATAACAGTAGTGAAGCCAACATACTA
   Consensus  CATTGTTTATTTTAACGCTTGTTTAAACCCACTGATCTATACCCTGTTTTACCCCTGGTTTAGAAATGCTGTTAAACTTATTATCACCCTGCAGATATTCAAGCATAACAGTAGTGAAGCCAACATACTA

              2211    2220      2230      2240      2250      2260      2270      2280      2290      2300      2310      2320      2330      2340
              |--------+---------+---------+---------+---------+---------+---------+---------+---------+---------+---------+---------+--------|
contig061433  TAGAGACAATGTGGAGGAACCTGACTGAAATCTGCAGAAAGATTTTTTTTCAGTAAATTGATGTGTCCAAAATCTGGAAATATAAAATTGTTTTTTAAAATGATGAACATATAGATGTGTCAGTTTCCAG
BurTARs.A013  TAG
   Consensus  TAG...............................................................................................................................
```

```
             2471  2480  2490  2500  2510  2520  2530  2540  2550  2560  2570  2580  2590  2600
contig061091 TGGTGGGAGGAGTGAGATAAATTAGCTGTATGAGAAGAATTTCTAATTAGAGAGCTGCAGGTGTCTGATGATGGAGATACCGAAAGGAGTCGAGCTCTGTTTCCCACAACTCCTCAACAGTTCCTGCAGG
BurTARs.A014                                                                         ATGGAGATACCGAAAGGAGTCGAGCTCTGTTTCCCACAACTCCTCAACAGTTCCTGCAGG
Consensus    ........................................................................ATGGAGATACCGAAAGGAGTCGAGCTCTGTTTCCCACAACTCCTCAACAGTTCCTGCAGG

             2601  2610  2620  2630  2640  2650  2660  2670  2680  2690  2700  2710  2720  2730
contig061091 AAGCCGACACTTCACTGGTCCAAAGCTGTGCTCCTGAACATTGTGCTCTCATGTATCTCTCTGCTCACTGCTGCTCTAAACCTTCTCGTCATCATCTCAGTCTCCCACTTCAGGCAGAGATTAACTTTTC
BurTARs.A014 AAGCCGACACTTCACTGGTCCAAAGCTGTGCTCCTGAACATTGTGCTCTCATGTATCTCTCTGCTCACTGCTGCTCTAAACCTTCTCGTCATCATCTCAGTCTCCCACTTCAGGCAG-------------
Consensus    AAGCCGACACTTCACTGGTCCAAAGCTGTGCTCCTGAACATTGTGCTCTCATGTATCTCTCTGCTCACTGCTGCTCTAAACCTTCTCGTCATCATCTCAGTCTCCCACTTCAGGCAG.............

             2731  2740  2750  2760  2770  2780  2790  2800  2810  2820  2830  2840  2850  2860
contig061091 AACTGAAACTACAGTTTAAGTTTTAGATGTGTGAAAACTTCCGTTTACATTATATGACTTAGTGATTAAAGAATGAATTTTAACCATGGTCATAATGTCTTATTTGCTTGAATTGAATCATGCCTTAGTA
BurTARs.A014 ----------------------------------------------------------------------------------------------------------------------------------
Consensus    ..................................................................................................................................

             2861  2870  2880  2890  2900  2910  2920  2930  2940  2950  2960  2970  2980  2990
contig061091 GTGCAGCATTTTCACTGAAGACATGTTGACATTAACAATGTGCATTATATTCTTTTCTGTCTCCCTGCAGGCAGCTGCACACACCCAGTAACATCCTCCTCCTCTCTCTGGCCGTCTCAGACTTTTTTGT
BurTARs.A014 --------------------------------------------------------------------------CTGCACACACCCAGTAACATCCTCCTCCTCTCTCTGGCCGTCTCAGACTTTTTTGT
Consensus    ..........................................................................CTGCACACACCCAGTAACATCCTCCTCCTCTCTCTGGCCGTCTCAGACTTTTTTGT

             2991  3000  3010  3020  3030  3040  3050  3060  3070  3080  3090  3100  3110  3120
contig061091 GGGTCTCCTGTTGTTGCCTTTAGAAATTTTTAGAAACACAAGCTGCTGGGTACTTGGTGATCGTATGTGTTCTGCTTATTGGTATTTGACCAGCAACATTATCTGTGCTTCAATAGGGAACATTGTTCTA
BurTARs.A014 GGGTCTCCTGTTGTTGCCTTTAGAAATTTTTAGAAACACAAGCTGCTGGGTACTTGGTGATCGTATGTGTTCTGCTTATTGGTATTTGACCAGCAACATTATCTGTGCTTCAATAGGGAACATTGTTCTA
Consensus    GGGTCTCCTGTTGTTGCCTTTAGAAATTTTTAGAAACACAAGCTGCTGGGTACTTGGTGATCGTATGTGTTCTGCTTATTGGTATTTGACCAGCAACATTATCTGTGCTTCAATAGGGAACATTGTTCTA

             3121  3130  3140  3150  3160  3170  3180  3190  3200  3210  3220  3230  3240  3250
contig061091 ATATCAGTTGACCGCTATGTGGCTATTTGTGACCCTCTGCATTACCCCAGCAGAATTACTTTGGCGAAAGTCAAACTCAGTGTTTGTCTCTGTTGGTTTTATGCTTTTTTCTACAGCAATCTTTATACAA
BurTARs.A014 ATATCAGTTGACCGCTATGTGGCTATTTGTGACCCTCTGCATTACCCCAGCAGAATTACTTTGGCGAAAGTCAAACTCAGTGTTTGTCTCTGTTGGTTTTATGCTTTTTTCTACAGCAATCTTTATACAA
Consensus    ATATCAGTTGACCGCTATGTGGCTATTTGTGACCCTCTGCATTACCCCAGCAGAATTACTTTGGCGAAAGTCAAACTCAGTGTTTGTCTCTGTTGGTTTTATGCTTTTTTCTACAGCAATCTTTATACAA

             3251  3260  3270  3280  3290  3300  3310  3320  3330  3340  3350  3360  3370  3380
contig061091 AGAATATCATGATTGAACCAGGCAGGTATAATTCTTGCTATGGAGAGTGTGTATTTTTTAGCAGCAATATTGCTATTGTTGCTGACCTTATTTTATTCTTTTTTGTTCCAGTAACTGTTATCATAGTCTT
BurTARs.A014 AGAATATCATGATTGAACCAGGCAGGTATAATTCTTGCTATGGAGAGTGTGTATTTTTTAGCAGCAATATTGCTATTGTTGCTGACCTTATTTTATTCTTTTTTGTTCCAGTAACTGTTATCATAGTCTT
Consensus    AGAATATCATGATTGAACCAGGCAGGTATAATTCTTGCTATGGAGAGTGTGTATTTTTTAGCAGCAATATTGCTATTGTTGCTGACCTTATTTTATTCTTTTTTGTTCCAGTAACTGTTATCATAGTCTT

             3381  3390  3400  3410  3420  3430  3440  3450  3460  3470  3480  3490  3500  3510
contig061091 GTATATGAGAGTATTTGTGGTGGCTGTGTCTCAAGCTCGTGCCATGCGCTCTCATGTTACATTGGTCACACTTCAGCGTTCACTGAATCAAACAAACAAATCTGAGCTGAAAGCAGCCAGGACTCTGGGG
BurTARs.A014 GTATATGAGAGTATTTGTGGTGGCTGTGTCTCAAGCTCGTGCCATGCGCTCTCATGTTACATTGGTCACACTTCAGCGTTCACTGAATCAAACAAACAAATCTGAGCTGAAAGCAGCCAGGACTCTGGGG
Consensus    GTATATGAGAGTATTTGTGGTGGCTGTGTCTCAAGCTCGTGCCATGCGCTCTCATGTTACATTGGTCACACTTCAGCGTTCACTGAATCAAACAAACAAATCTGAGCTGAAAGCAGCCAGGACTCTGGGG

             3511  3520  3530  3540  3550  3560  3570  3580  3590  3600  3610  3620  3630  3640
contig061091 ATTCTTGTAGTTGTGTTTCTAGCATGCTTCTCTCCACTCTACTGCTACTCTCTTGTTGATGAAAATGCAATCAATAATCCAGCTGCATCTTTTGCGGTCATTATCTTTTACATTAACTCTTGTCTAAACC
BurTARs.A014 ATTCTTGTAGTTGTGTTTCTAGCATGCTTCTCTCCACTCTACTGCTACTCTCTTGTTGATGAAAATGCAATCAATAATCCAGCTGCATCTTTTGCGGTCATTATCTTTTACATTAACTCTTGTCTAAACC
Consensus    ATTCTTGTAGTTGTGTTTCTAGCATGCTTCTCTCCACTCTACTGCTACTCTCTTGTTGATGAAAATGCAATCAATAATCCAGCTGCATCTTTTGCGGTCATTATCTTTTACATTAACTCTTGTCTAAACC

             3641  3650  3660  3670  3680  3690  3700  3710  3720  3730  3740  3750  3760  3770
contig061091 CTTTGATCTATGCCTTGTTTTACCCCTGGTTTAGAAATGCTGTTAAACTTATCATCACGTTGGAGATCTTCAAGTATGACACAAGTGGTGCCAACATACTGTAAAAAGAGTACGAAACCTGCTTGTGCAA
BurTARs.A014 CTTTGATCTATGCCTTGTTTTACCCCTGGTTTAGAAATGCTGTTAAACTTATCATCACGTTGGAGATCTTCAAGTATGACACAAGTGGTGCCAACATACTGTAA
Consensus    CTTTGATCTATGCCTTGTTTTACCCCTGGTTTAGAAATGCTGTTAAACTTATCATCACGTTGGAGATCTTCAAGTATGACACAAGTGGTGCCAACATACTGTAA..........................
```

```
             1301 1310      1320      1330      1340      1350      1360      1370      1380      1390      1400      1410      1420      1430
             |--------+---------+---------+---------+---------+---------+---------+---------+---------+---------+---------+---------+---------|
contig060707 TTGTGCTGAACAGCTGTCTTTCTCTAACAATGGAAGAAACTGAACTCTGCTTTCCACAACTATTCAACTCCTCTTGTGTGCGGCAAAAGCATTCACAAATTGAGGCTGTGTTTATTTACACTCCACTCTC
BurTARs.A015                              ATGGAAGAAACTGAACTCTGCTTTCCACAACTATTCAACTCCTCTTGTGTGCGGCAAAAGCATTCACAAATTGAGGCTGTGTTTATTTACACTCCACTCTC
   Consensus .............................ATGGAAGAAACTGAACTCTGCTTTCCACAACTATTCAACTCCTCTTGTGTGCGGCAAAAGCATTCACAAATTGAGGCTGTGTTTATTTACACTCCACTCTC

             1431 1440      1450      1460      1470      1480      1490      1500      1510      1520      1530      1540      1550      1560
             |--------+---------+---------+---------+---------+---------+---------+---------+---------+---------+---------+---------+---------|
contig060707 TTCCATCTCTGTGCTTACTACAATTCTGAACCTGCTTGTGATCATCTCCATTGCTCACTTCAAGTACTGACATATTTGAATACTTAACAAGTTATAGAAAAATAATGTTTTTTGTGAATTTAATTAGAAA
BurTARs.A015 TTCCATCTCTGTGCTTACTACAATTCTGAACCTGCTTGTGATCATCTCCATTGCTCACTTCAAG------------------------------------------------------------------
   Consensus TTCCATCTCTGTGCTTACTACAATTCTGAACCTGCTTGTGATCATCTCCATTGCTCACTTCAAG..................................................................

             1561 1570      1580      1590      1600      1610      1620      1630      1640      1650      1660      1670      1680      1690
             |--------+---------+---------+---------+---------+---------+---------+---------+---------+---------+---------+---------+---------|
contig060707 ATGTGTTGCTTTGTTCTATATGTTACAGTGGAGTTGCAATTACTGCTGTAGAACAACCTTTGATGCTCCTATTTTAATCCAGGCAGCTGCACACCCCAACCAACCTCCTCCTCCTCTCTCTGGCCGTCTC
BurTARs.A015 -----------------------------------------------------------------------------------CAGCTGCACACCCCAACCAACCTCCTCCTCCTCTCTCTGGCCGTCTC
   Consensus ...................................................................................CAGCTGCACACCCCAACCAACCTCCTCCTCCTCTCTCTGGCCGTCTC

             1691 1700      1710      1720      1730      1740      1750      1760      1770      1780      1790      1800      1810      1820
             |--------+---------+---------+---------+---------+---------+---------+---------+---------+---------+---------+---------+---------|
contig060707 AGATTTCTTCGTGGGCCTCATCATGGCTTGTCAGATTAGCCTCCTAGATGGCTGCTGGTTTCTTGGTGACCACATGTGTGCTCTGTATAGCAGTTTAGATTACATTGTTACTTCTGCTTCAGTAGGAACT
BurTARs.A015 AGATTTCTTCGTGGGCCTCATCATGGCTTGTCAGATTAGCCTCCTAGATGGCTGCTGGTTTCTTGGTGACCACATGTGTGCTCTGTATAGCAGTTTAGATTACATTGTTACTTCTGCTTCAGTAGGAACT
   Consensus AGATTTCTTCGTGGGCCTCATCATGGCTTGTCAGATTAGCCTCCTAGATGGCTGCTGGTTTCTTGGTGACCACATGTGTGCTCTGTATAGCAGTTTAGATTACATTGTTACTTCTGCTTCAGTAGGAACT

             1821 1830      1840      1850      1860      1870      1880      1890      1900      1910      1920      1930      1940      1950
             |--------+---------+---------+---------+---------+---------+---------+---------+---------+---------+---------+---------+---------|
contig060707 ATGGTACTCATATCAGCTGACCGCTATGTAGCCATTTGTGACCCTCTGCATTATCCCACCAAAATTACTATGAAAAGAGTCTCAGTCTCTATTTGTACTTGCTGGGCTTGTTCAATTCTGTATAACAGTC
BurTARs.A015 ATGGTACTCATATCAGCTGACCGCTATGTAGCCATTTGTGACCCTCTGCATTATCCCACCAAAATTACTATGAAAAGAGTCTCAGTCTCTATTTGTACTTGCTGGGCTTGTTCAATTCTGTATAACAGTC
   Consensus ATGGTACTCATATCAGCTGACCGCTATGTAGCCATTTGTGACCCTCTGCATTATCCCACCAAAATTACTATGAAAAGAGTCTCAGTCTCTATTTGTACTTGCTGGGCTTGTTCAATTCTGTATAACAGTC

             1951 1960      1970      1980      1990      2000      2010      2020      2030      2040      2050      2060      2070      2080
             |--------+---------+---------+---------+---------+---------+---------+---------+---------+---------+---------+---------+---------|
contig060707 TTATCATGAAGGATAATTTCAAGCAGCCAGGGAGGTATAATTCTTGTTCTGGTGATTGTGTAGTTGTCATTGATTACTTTGTGGGAATTTTTGACTTTGTTTTGACCTTTGTCGGCCCTGTCATTGTCAT
BurTARs.A015 TTATCATGAAGGATAATTTCAAGCAGCCAGGGAGGTATAATTCTTGTTCTGGTGATTGTGTAGTTGTCATTGATTACTTTGTGGGAATTTTTGACTTTGTTTTGACCTTTGTCGGCCCTGTCATTGTCAT
   Consensus TTATCATGAAGGATAATTTCAAGCAGCCAGGGAGGTATAATTCTTGTTCTGGTGATTGTGTAGTTGTCATTGATTACTTTGTGGGAATTTTTGACTTTGTTTTGACCTTTGTCGGCCCTGTCATTGTCAT

             2081 2090      2100      2110      2120      2130      2140      2150      2160      2170      2180      2190      2200      2210
             |--------+---------+---------+---------+---------+---------+---------+---------+---------+---------+---------+---------+---------|
contig060707 CATAGTTCTGTATCTAAGAGTATTTGTGGTGGCTGTGTCTCAGGCTCGGGCAATGCGCTCTCATATCACTGCTCTCAGACTCCAGGGGTCAGAGACGGTGCATGCTAAGAAATCTGAGCTGAAAGCTGCC
BurTARs.A015 CATAGTTCTGTATCTAAGAGTATTTGTGGTGGCTGTGTCTCAGGCTCGGGCAATGCGCTCTCATATCACTGCTCTCAGACTCCAGGGGTCAGAGACGGTGCATGCTAAGAAATCTGAGCTGAAAGCTGCC
   Consensus CATAGTTCTGTATCTAAGAGTATTTGTGGTGGCTGTGTCTCAGGCTCGGGCAATGCGCTCTCATATCACTGCTCTCAGACTCCAGGGGTCAGAGACGGTGCATGCTAAGAAATCTGAGCTGAAAGCTGCC

             2211 2220      2230      2240      2250      2260      2270      2280      2290      2300      2310      2320      2330      2340
             |--------+---------+---------+---------+---------+---------+---------+---------+---------+---------+---------+---------+---------|
contig060707 AGGACTCTCGGTGTACTTGTTATTGCCTTTCTGATATGTCTTTTCCCATTTTTCTGTTCCTCAATGGTAGGCCAGAATAGTTTCTTTGATATTAGATCTGTACCTTTTGAGCTCTTGCTGTTCTATTTTA
BurTARs.A015 AGGACTCTCGGTGTACTTGTTATTGCCTTTCTGATATGTCTTTTCCCATTTTTCTGTTCCTCAATGGTAGGCCAGAATAGTTTCTTTGATATTAGATCTGTACCTTTTGAGCTCTTGCTGTTCTATTTTA
   Consensus AGGACTCTCGGTGTACTTGTTATTGCCTTTCTGATATGTCTTTTCCCATTTTTCTGTTCCTCAATGGTAGGCCAGAATAGTTTCTTTGATATTAGATCTGTACCTTTTGAGCTCTTGCTGTTCTATTTTA

             2341 2350      2360      2370      2380      2390      2400      2410      2420      2430      2440      2450      2460      2470
             |--------+---------+---------+---------+---------+---------+---------+---------+---------+---------+---------+---------+---------|
contig060707 ACTCTTGTCTGAACCCATTAATCTACACTTTTTGCTACCCCTGGTTTCTGAAATCTATCAAGCTCATTGTGACATTTAAGATATTTAGGCATGGCTCCAGTGAGGCCAGTATACTATAGGGAAAAGTGAT
BurTARs.A015 ACTCTTGTCTGAACCCATTAATCTACACTTTTTGCTACCCCTGGTTTCTGAAATCTATCAAGCTCATTGTGACATTTAAGATATTTAGGCATGGCTCCAGTGAGGCCAGTATACTATAG
   Consensus ACTCTTGTCTGAACCCATTAATCTACACTTTTTGCTACCCCTGGTTTCTGAAATCTATCAAGCTCATTGTGACATTTAAGATATTTAGGCATGGCTCCAGTGAGGCCAGTATACTATAG...........

             [illegible]
```

```
             1691  1700      1710      1720      1730      1740      1750      1760      1770      1780      1790      1800      1810      1820
             |--------+---------+---------+---------+---------+---------+---------+---------+---------+---------+---------+---------+---------|
contig059673 AAGCTGGAACCTCCTTTATCTAATGATGAAAACCTTTGAAGAAGCTGAACTCTGCTTTCCACAACTCCTCAACTCCTCCTGCAGGAAGACCATGCGTCCTTATACATTTTCTATCCTTATTTATATTACC
BurTARs.A016                          ATGAAAACCTTTGAAGAAGCTGAACTCTGCTTTCCACAACTCCTCAACTCCTCCTGCAGGAAGACCATGCGTCCTTATACATTTTCTATCCTTATTTATATTACC
   Consensus .........................ATGAAAACCTTTGAAGAAGCTGAACTCTGCTTTCCACAACTCCTCAACTCCTCCTGCAGGAAGACCATGCGTCCTTATACATTTTCTATCCTTATTTATATTACC

             1821  1830      1840      1850      1860      1870      1880      1890      1900      1910      1920      1930      1940      1950
             |--------+---------+---------+---------+---------+---------+---------+---------+---------+---------+---------+---------+---------|
contig059673 CTGTCCTCCATTTCTTTGCTCACTGTGACTCTTAATCTGCTGGTCATCATCTCCATCTCACATTTCAAGTAATAATTTTGTTAGACATATGCAGACAGTAAGATTTTATGGCTAAGAGATGAAAATGATA
BurTARs.A016 CTGTCCTCCATTTCTTTGCTCAC-----------------------------------------------------------------------------------------------------------
   Consensus CTGTCCTCCATTTCTTTGCTCAC...........................................................................................................

             1951  1960      1970      1980      1990      2000      2010      2020      2030      2040      2050      2060      2070      2080
             |--------+---------+---------+---------+---------+---------+---------+---------+---------+---------+---------+---------+---------|
contig059673 TGATATCCTATCTGAGTGAGTCTGAGCTAAATGCTTTTTCCTTTTATCTGTATAAAATTGCACTTGTTGCTACAAATAATAATAATAATAACTATGTCGCTGTCTTTTGTCTTCAGGAAGCTGCACACCC
BurTARs.A016 --------------------------------------------------------------------------------------------------------------------GAAGCTGCACACCC
   Consensus ....................................................................................................................GAAGCTGCACACCC

             2081  2090      2100      2110      2120      2130      2140      2150      2160      2170      2180      2190      2200      2210
             |--------+---------+---------+---------+---------+---------+---------+---------+---------+---------+---------+---------+---------|
contig059673 CCACCAATCTCCTCCTCCTATCTTTGGCTGTCTCTGATTGCCTGGTGGGTCTCCTGATTTTATTTCAAATTATGATTATAGATGGTTGCTGGTTTCTTGGTGAATTCATGTGTAGTATGTATTTTCTCTT
BurTARs.A016 CCACCAATCTCCTCCTCCTATCTTTGGCTGTCTCTGATTGCCTGGTGGGTCTCCTGATTTTATTTCAAATTATGATTATAGATGGTTGCTGGTTTCTTGGTGAATTCATGTGTAGTATGTATTTTCTCTT
   Consensus CCACCAATCTCCTCCTCCTATCTTTGGCTGTCTCTGATTGCCTGGTGGGTCTCCTGATTTTATTTCAAATTATGATTATAGATGGTTGCTGGTTTCTTGGTGAATTCATGTGTAGTATGTATTTTCTCTT

             2211  2220      2230      2240      2250      2260      2270      2280      2290      2300      2310      2320      2330      2340
             |--------+---------+---------+---------+---------+---------+---------+---------+---------+---------+---------+---------+---------|
contig059673 GGACTACATTATTACCTCTGCATCAATAGGAACCATGGTTCTCATATCAATTGACCGTTATGTGGCTATTTGTTACCCTCTCCATTACTCTACCAAAGTCACCCCAAAAAGAACAAAAGCATGTGTTTAT
BurTARs.A016 GGACTACATTATTACCTCTGCATCAATAGGAACCATGGTTCTCATATCAATTGACCGTTATGTGGCTATTTGTTACCCTCTCCATTACTCTACCAAAGTCACCCCAAAAAGAACAAAAGCATGTGTTTAT
   Consensus GGACTACATTATTACCTCTGCATCAATAGGAACCATGGTTCTCATATCAATTGACCGTTATGTGGCTATTTGTTACCCTCTCCATTACTCTACCAAAGTCACCCCAAAAAGAACAAAAGCATGTGTTTAT

             2341  2350      2360      2370      2380      2390      2400      2410      2420      2430      2440      2450      2460      2470
             |--------+---------+---------+---------+---------+---------+---------+---------+---------+---------+---------+---------+---------|
contig059673 CTCTGTTGGATTTGTTCTTCAGTCTTCCAATGTCTCCTGCTAAAAGACAACCTGGTGCAACCTGGCAGGTATAATTCCTGCTATGGGGAGTGTGTGGTTGTTGTTGGCCATGCTTTTGGAGTTGCAGATC
BurTARs.A016 CTCTGTTGGATTTGTTCTTCAGTCTTCCAATGTCTCCTGCTAAAAGACAACCTGGTGCAACCTGGCAGGTATAATTCCTGCTATGGGGAGTGTGTGGTTGTTGTTGGCCATGCTTTTGGAGTTGCAGATC
   Consensus CTCTGTTGGATTTGTTCTTCAGTCTTCCAATGTCTCCTGCTAAAAGACAACCTGGTGCAACCTGGCAGGTATAATTCCTGCTATGGGGAGTGTGTGGTTGTTGTTGGCCATGCTTTTGGAGTTGCAGATC

             2471  2480      2490      2500      2510      2520      2530      2540      2550      2560      2570      2580      2590      2600
             |--------+---------+---------+---------+---------+---------+---------+---------+---------+---------+---------+---------+---------|
contig059673 TATTGCTGTCCATTATTGGTCCTGTCACTGTGATTGTAGTTCTTTATCTAAACGTATTTGTTGTGGCTATGACTCAAGCTCGTGCCCTGCGGTCACATATTGCAGCTCTGACACACGAGAGGTCAGTAAG
BurTARs.A016 TATTGCTGTCCATTATTGGTCCTGTCACTGTGATTGTAGTTCTTTATCTAAACGTATTTGTTGTGGCTATGACTCAAGCTCGTGCCCTGCGGTCACATATTGCAGCTCTGACACACGAGAGGTCAGTAAG
   Consensus TATTGCTGTCCATTATTGGTCCTGTCACTGTGATTGTAGTTCTTTATCTAAACGTATTTGTTGTGGCTATGACTCAAGCTCGTGCCCTGCGGTCACATATTGCAGCTCTGACACACGAGAGGTCAGTAAG

             2601  2610      2620      2630      2640      2650      2660      2670      2680      2690      2700      2710      2720      2730
             |--------+---------+---------+---------+---------+---------+---------+---------+---------+---------+---------+---------+---------|
contig059673 TACAAATGTTAAGAAATCTGAAATGAAAGCAGTAAGGACCATCAGTGTTCTTATCATTGTGTTTCTAATATGTCTCTGCCCATACTTTGGTGTCACACTCTCAGGTCAAGATGCCATGCTCAGTGCTTCA
BurTARs.A016 TACAAATGTTAAGAAATCTGAAATGAAAGCAGTAAGGACCATCAGTGTTCTTATCATTGTGTTTCTAATATGTCTCTGCCCATACTTTGGTGTCACACTCTCAGGTCAAGATGCCATGCTCAGTGCTTCA
   Consensus TACAAATGTTAAGAAATCTGAAATGAAAGCAGTAAGGACCATCAGTGTTCTTATCATTGTGTTTCTAATATGTCTCTGCCCATACTTTGGTGTCACACTCTCAGGTCAAGATGCCATGCTCAGTGCTTCA

             2731  2740      2750      2760      2770      2780      2790      2800      2810      2820      2830      2840      2850      2860
             |--------+---------+---------+---------+---------+---------+---------+---------+---------+---------+---------+---------+---------|
contig059673 TCTGTTGCATTTGTAATGTGTTTGTTCTATTTAAACTCCTGTCTAAACCCTCTAATCTATGCTTTATTTTACCCCTGGTTCAGAAAATCTGTTAAACAAATTGTTACACTTAAAATATTGAAGTCTGGCT
BurTARs.A016 TCTGTTGCATTTGTAATGTGTTTGTTCTATTTAAACTCCTGTCTAAACCCTCTAATCTATGCTTTATTTTACCCCTGGTTCAGAAAATCTGTTAAACAAATTGTTACACTTAAAATATTGAAGTCTGGCT
   Consensus TCTGTTGCATTTGTAATGTGTTTGTTCTATTTAAACTCCTGTCTAAACCCTCTAATCTATGCTTTATTTTACCCCTGGTTCAGAAAATCTGTTAAACAAATTGTTACACTTAAAATATTGAAGTCTGGCT

             2861  2870      2880      2890      2900      2910      2920      2930      2940      2950      2960      2970      2980      2990
             |--------+---------+---------+---------+---------+---------+---------+---------+---------+---------+---------+---------+---------|
contig059673 CCTGTGACACCAATATCATGTAAACAGAATGAAAGACTGAGAATGAAAGCCCCATTGTTTTTTTGTTGTTGTTTTTTTATGTTTTCTATTACAAAAAAATTACTAACAGAGAAGTAACACCCATAAGTAA
BurTARs.A016 CCTGTGACACCAATATCATGTAA
   Consensus CCTGTGACACCAATATCATGTAA...........................................................................................................
```

```
             6241 6250      6260      6270      6280      6290      6300      6310      6320      6330      6340      6350      6360      6370
             |--------+---------+---------+---------+---------+---------+---------+---------+---------+---------+---------+---------+---------|
contig057301 AATAAACACAGACAGCAACATGGGAAATATGTGGAAGGAGGGACCTTTTTTAATGAAGAGAACATGGGAGGAGTAAAAGAACAAGATAAACCTAATCAGCTCACCTCCTGTGATGATGATGGAGGGGGTT
BurTARs.A017                                                                                                                       ATGGAGGGGGTT
   Consensus ......................................................................................................................ATGGAGGGGGTT

             6371 6380      6390      6400      6410      6420      6430      6440      6450      6460      6470      6480      6490      6500
             |--------+---------+---------+---------+---------+---------+---------+---------+---------+---------+---------+---------+---------|
contig057301 GAATTCTGCTTTCCACATCTGCTCAACTCCTCTTGCAGAAAGGCTCTGCATCCTGTCTCAGTTTCCACGCTCATTTACATGATAATATCTTCTATCTCTGTGCTCACTGCAACTCTTAACCTGCTGGTCA
BurTARs.A017 GAATTCTGCTTTCCACATCTGCTCAACTCCTCTTGCAGAAAGGCTCTGCATCCTGTCTCAGTTTCCACGCTCATTTACATGATAATATCTTCTATCTCTGTGCTCACTGCAACTCTTAACCTGCTGGTCA
   Consensus GAATTCTGCTTTCCACATCTGCTCAACTCCTCTTGCAGAAAGGCTCTGCATCCTGTCTCAGTTTCCACGCTCATTTACATGATAATATCTTCTATCTCTGTGCTCACTGCAACTCTTAACCTGCTGGTCA

             6501 6510      6520      6530      6540      6550      6560      6570      6580      6590      6600      6610      6620      6630
             |--------+---------+---------+---------+---------+---------+---------+---------+---------+---------+---------+---------+---------|
contig057301 TCATCTCCATCTCCCACTTCAAGTAGCCACATATTTTATAAAGTTAAAAGATTAATAGCTTTTTTTAAGGAAAAACTTAAAAAAAAATAACAACTATGAATTTATGCTGCAAATAACAAGTTCTTTTTT
BurTARs.A017 TCATCTCCATCTCCCACTTCAAG-----------------------------------------------------------------------------------------------------------
   Consensus TCATCTCCATCTCCCACTTCAAG...........................................................................................................

             6631 6640      6650      6660      6670      6680      6690      6700      6710      6720      6730      6740      6750      6760
             |--------+---------+---------+---------+---------+---------+---------+---------+---------+---------+---------+---------+---------|
contig057301 CTTTTTTTTCCTCTAGGCAGCTCCATAATCCCACCAACTTCCTCCTCCTCTCTCTGGCTGTCTCCGATTTCTTTGTGGGTCTCTATTTGCTCTTTTACATAATGTTTATAGACGGCTGCTGGTATTTTGG
BurTARs.A017 -----------------CAGCTCCATAATCCCACCAACTTCCTCCTCCTCTCTCTGGCTGTCTCCGATTTCTTTGTGGGTCTCTATTTGCTCTTTTACATAATGTTTATAGACGGCTGCTGGTATTTTGG
   Consensus .................CAGCTCCATAATCCCACCAACTTCCTCCTCCTCTCTCTGGCTGTCTCCGATTTCTTTGTGGGTCTCTATTTGCTCTTTTACATAATGTTTATAGACGGCTGCTGGTATTTTGG

             6761 6770      6780      6790      6800      6810      6820      6830      6840      6850      6860      6870      6880      6890
             |--------+---------+---------+---------+---------+---------+---------+---------+---------+---------+---------+---------+---------|
contig057301 TGAATTCATGTGTATTCTGTATTATGTTATTGGCACAATTAACACCTCTTCCTCAATAGGAACCATGGTACTGATATCAGTTGACCGTTATGTGGCCATTTGTGATCCTCTGCATTATCCCATCAAAGTC
BurTARs.A017 TGAATTCATGTGTATTCTGTATTATGTTATTGGCACAATTAACACCTCTTCCTCAATAGGAACCATGGTACTGATATCAGTTGACCGTTATGTGGCCATTTGTGATCCTCTGCATTATCCCATCAAAGTC
   Consensus TGAATTCATGTGTATTCTGTATTATGTTATTGGCACAATTAACACCTCTTCCTCAATAGGAACCATGGTACTGATATCAGTTGACCGTTATGTGGCCATTTGTGATCCTCTGCATTATCCCATCAAAGTC

             6891 6900      6910      6920      6930      6940      6950      6960      6970      6980      6990      7000      7010      7020
             |--------+---------+---------+---------+---------+---------+---------+---------+---------+---------+---------+---------+---------|
contig057301 ACTGCAAAAAGAGTTCAGATCTGTGTTTCACTGTGTTGGAGCTTTTCTGCCCTTGCTGGTAGTTTCCTCTTAAAGGACAACTTGAAACAGCAAAGCAGGTTTAATTCTTGTGTGGGAGAGTGTGTTGTCC
BurTARs.A017 ACTGCAAAAAGAGTTCAGATCTGTGTTTCACTGTGTTGGAGCTTTTCTGCCCTTGCTGGTAGTTTCCTCTTAAAGGACAACTTGAAACAGCAAAGCAGGTTTAATTCTTGTGTGGGAGAGTGTGTTGTCC
   Consensus ACTGCAAAAAGAGTTCAGATCTGTGTTTCACTGTGTTGGAGCTTTTCTGCCCTTGCTGGTAGTTTCCTCTTAAAGGACAACTTGAAACAGCAAAGCAGGTTTAATTCTTGTGTGGGAGAGTGTGTTGTCC

             7021 7030      7040      7050      7060      7070      7080      7090      7100      7110      7120      7130      7140      7150
             |--------+---------+---------+---------+---------+---------+---------+---------+---------+---------+---------+---------+---------|
contig057301 ATATTAACTTTATTGAATATGTTGCAGATCTTGCTTTGAACTTCCTACTTCCTATTACTGTCATCATAGTTCTGTATTTGAGAATATTTGTAGTGGTTGTGTCTCAGGTTCGGGCCATGCGGACTCATAC
BurTARs.A017 ATATTAACTTTATTGAATATGTTGCAGATCTTGCTTTGAACTTCCTACTTCCTATTACTGTCATCATAGTTCTGTATTTGAGAATATTTGTAGTGGTTGTGTCTCAGGTTCGGGCCATGCGGACTCATAC
   Consensus ATATTAACTTTATTGAATATGTTGCAGATCTTGCTTTGAACTTCCTACTTCCTATTACTGTCATCATAGTTCTGTATTTGAGAATATTTGTAGTGGTTGTGTCTCAGGTTCGGGCCATGCGGACTCATAC

             7151 7160      7170      7180      7190      7200      7210      7220      7230      7240      7250      7260      7270      7280
             |--------+---------+---------+---------+---------+---------+---------+---------+---------+---------+---------+---------+---------|
contig057301 TGCAGGTGTCACATACCAGTGTTCAAGGAAGGGAAATCCAAAGAAATCAGAGATGAAAGCAGCCAGGACTCTTGGTATTGTTGTAATTGCATTCTTGTCATGCGCCTTACCATTTTATTGTGTCACACTC
BurTARs.A017 TGCAGGTGTCACATACCAGTGTTCAAGGAAGGGAAATCCAAAGAAATCAGAGATGAAAGCAGCCAGGACTCTTGGTATTGTTGTAATTGCATTCTTGTCATGCGCCTTACCATTTTATTGTGTCACACTC
   Consensus TGCAGGTGTCACATACCAGTGTTCAAGGAAGGGAAATCCAAAGAAATCAGAGATGAAAGCAGCCAGGACTCTTGGTATTGTTGTAATTGCATTCTTGTCATGCGCCTTACCATTTTATTGTGTCACACTC

             7281 7290      7300      7310      7320      7330      7340      7350      7360      7370      7380      7390      7400      7410
             |--------+---------+---------+---------+---------+---------+---------+---------+---------+---------+---------+---------+---------|
contig057301 ACAGGCCAGAATGCCTTCCTCAACGGATCATCTTCTGCATTTGTTTTGTGTCTTTTCTATTTTAATTCGTGCCTCAACCCTATCATCTATGCCTTATTCTACCCCTGGTTTAGAAAATCTATTAAGCTTA
BurTARs.A017 ACAGGCCAGAATGCCTTCCTCAACGGATCATCTTCTGCATTTGTTTTGTGTCTTTTCTATTTTAATTCGTGCCTCAACCCTATCATCTATGCCTTATTCTACCCCTGGTTTAGAAAATCTATTAAGCTTA
   Consensus ACAGGCCAGAATGCCTTCCTCAACGGATCATCTTCTGCATTTGTTTTGTGTCTTTTCTATTTTAATTCGTGCCTCAACCCTATCATCTATGCCTTATTCTACCCCTGGTTTAGAAAATCTATTAAGCTTA

             7411 7420      7430      7440      7450      7460      7470      7480      7490      7500      7510      7520      7530      7540
             |--------+---------+---------+---------+---------+---------+---------+---------+---------+---------+---------+---------+---------|
contig057301 TTGTTACATTTCAAATACTGAAGTCTGGCTCCAGGAATGCCAACATAGTCAAAGTGACATAATAGTGACTAAAACGAGGTCTCTACTGTTTTAGCAGCAGTTTACGAGTAAGTCAGCCCAAACAAGCAGT
BurTARs.A017 TTGTTACATTTCAAATACTGAAGTCTGGCTCCAGGAATGCCAACATAGTCAAAGTGACATAA
   Consensus TTGTTACATTTCAAATACTGAAGTCTGGCTCCAGGAATGCCAACATAGTCAAAGTGACATAA....................................................................

             7541 7550      7560      7570      7580      7590      7600      7610      7620      7630      7640      7650      7660      7670
```

```
             2081 2090      2100      2110      2120      2130      2140      2150      2160      2170      2180      2190      2200      2210
             |--------+---------+---------+---------+---------+---------+---------+---------+---------+---------+---------+---------+---------|
contig057145 TAATGGAGATACAGAAAGTGCCCGAGCTTTGTTTTCCACAATTTCTCAACAATTCCTGCAGAAAGCCGACACTTCATTGGTCGAAAGCCGTGCTCCTGAACATTGTGTTCTCCTGTATCTCTCTGCTCAC
BurTARs.A018   ATGGAGATACAGAAAGTGCCCGAGCTTTGTTTTCCACAATTTCTCAACAATTCCTGCAGAAAGCCGACACTTCATTGGTCGAAAGCCGTGCTCCTGAACATTGTGTTCTCCTGTATCTCTCTGCTCAC
   Consensus ..ATGGAGATACAGAAAGTGCCCGAGCTTTGTTTTCCACAATTTCTCAACAATTCCTGCAGAAAGCCGACACTTCATTGGTCGAAAGCCGTGCTCCTGAACATTGTGTTCTCCTGTATCTCTCTGCTCAC

             2211 2220      2230      2240      2250      2260      2270      2280      2290      2300      2310      2320      2330      2340
             |--------+---------+---------+---------+---------+---------+---------+---------+---------+---------+---------+---------+---------|
contig057145 TGCTGCTCTAAACCTTCTCGTCATCATTTCAGTCTCCCACTTCAGGCACAGATGATTTTTTTTATTGACAATAATGAAATGCAACTGATAAATATCTCTGTTTTTAATTTTTACTTTCGTTTTGTTAGAT
BurTARs.A018 TGCTGCTCTAAACCTTCTCGTCATCATTTCAGTCTCCCACTTCAGG------------------------------------------------------------------------------------
   Consensus TGCTGCTCTAAACCTTCTCGTCATCATTTCAGTCTCCCACTTCAGG....................................................................................

             2341 2350      2360      2370      2380      2390      2400      2410      2420      2430      2440      2450      2460      2470
             |--------+---------+---------+---------+---------+---------+---------+---------+---------+---------+---------+---------+---------|
contig057145 AAATAAATATCTTTGTCTCAGTTATCACTGTTAAGTTGAATAGGCTTCTTCATGTCTTAATAATGAAACATTTTCACTAAGATTGTTTTAACATGATTGACAAAACATTTTCAGTGCAGCTGTCAGCAGT
BurTARs.A018 ----------------------------------------------------------------------------------------------------------------------------------
   Consensus ..................................................................................................................................

             2471 2480      2490      2500      2510      2520      2530      2540      2550      2560      2570      2580      2590      2600
             |--------+---------+---------+---------+---------+---------+---------+---------+---------+---------+---------+---------+---------|
contig057145 CTTATTTTCATCCTAATCTTACACTTCAGACACTTCAAACTGAAATTGCTTAGTGATGAGTGAGTGGAATCAATTTTGCATGTTCTTGGTGTATTTTAATCACAACTGAAATATTTTATTTGCCTCGATT
BurTARs.A018 ----------------------------------------------------------------------------------------------------------------------------------
   Consensus ..................................................................................................................................

             2601 2610      2620      2630      2640      2650      2660      2670      2680      2690      2700      2710      2720      2730
             |--------+---------+---------+---------+---------+---------+---------+---------+---------+---------+---------+---------+---------|
contig057145 GAATCATGCCTTATTAATAGGACTTTTTCACTGATGGAATTTTTACTGAAGACATTTTGAAATGATGAATTACTTCTTTTTTTTTCTCTTTGTAGGAAACTTCACACACCAAGTAACATCGTCCTCCTCT
BurTARs.A018 ------------------------------------------------------------------------------------------------AAACTTCACACACCAAGTAACATCGTCCTCCTCT
   Consensus ................................................................................................AAACTTCACACACCAAGTAACATCGTCCTCCTCT

             2731 2740      2750      2760      2770      2780      2790      2800      2810      2820      2830      2840      2850      2860
             |--------+---------+---------+---------+---------+---------+---------+---------+---------+---------+---------+---------+---------|
contig057145 CTCTGGCTGTCTCAGACTTTCTTGTGGGTCTTCTGTTGATGCCAGCAGAAATATTAAGAAGCACGACGTGCTGGGTACTTGGTGACATTATGTGTTCTGTTTATTGGTATCTGACCGGCAACATTATCTG
BurTARs.A018 CTCTGGCTGTCTCAGACTTTCTTGTGGGTCTTCTGTTGATGCCAGCAGAAATATTAAGAAGCACGACGTGCTGGGTACTTGGTGACATTATGTGTTCTGTTTATTGGTATCTGACCGGCAACATTATCTG
   Consensus CTCTGGCTGTCTCAGACTTTCTTGTGGGTCTTCTGTTGATGCCAGCAGAAATATTAAGAAGCACGACGTGCTGGGTACTTGGTGACATTATGTGTTCTGTTTATTGGTATCTGACCGGCAACATTATCTG

             2861 2870      2880      2890      2900      2910      2920      2930      2940      2950      2960      2970      2980      2990
             |--------+---------+---------+---------+---------+---------+---------+---------+---------+---------+---------+---------+---------|
contig057145 TGCTTCAATAGGGAACATAGTTCTAATATCAGTTGATCGTTATGTGGCTATTTGTGACCCCTTGCATTACCGCACCAGAATTACTGTGAAACGAACCAAACTCAGTGTGTGCTTATGTTGGTTTTATGCT
BurTARs.A018 TGCTTCAATAGGGAACATAGTTCTAATATCAGTTGATCGTTATGTGGCTATTTGTGACCCCTTGCATTACCGCACCAGAATTACTGTGAAACGAACCAAACTCAGTGTGTGCTTATGTTGGTTTTATGCT
   Consensus TGCTTCAATAGGGAACATAGTTCTAATATCAGTTGATCGTTATGTGGCTATTTGTGACCCCTTGCATTACCGCACCAGAATTACTGTGAAACGAACCAAACTCAGTGTGTGCTTATGTTGGTTTTATGCT

             2991 3000      3010      3020      3030      3040      3050      3060      3070      3080      3090      3100      3110      3120
             |--------+---------+---------+---------+---------+---------+---------+---------+---------+---------+---------+---------+---------|
contig057145 ATTTTCTACTGGAGTCTTTATATAAAGAATATCCTGATTGAACCGGGCAGGTATAATTCCTGCTACGGAGAGTGTATGTTTGTCAGCAGTGATATTGCAGGGATTATTGACCTTTTTTTATCTTTTATTG
BurTARs.A018 ATTTTCTACTGGAGTCTTTATATAAAGAATATCCTGATTGAACCGGGCAGGTATAATTCCTGCTACGGAGAGTGTATGTTTGTCAGCAGTGATATTGCAGGGATTATTGACCTTTTTTTATCTTTTATTG
   Consensus ATTTTCTACTGGAGTCTTTATATAAAGAATATCCTGATTGAACCGGGCAGGTATAATTCCTGCTACGGAGAGTGTATGTTTGTCAGCAGTGATATTGCAGGGATTATTGACCTTTTTTTATCTTTTATTG

             3121 3130      3140      3150      3160      3170      3180      3190      3200      3210      3220      3230      3240      3250
             |--------+---------+---------+---------+---------+---------+---------+---------+---------+---------+---------+---------+---------|
contig057145 TTCCACTTTCTGTCATCATAGTTCTTTATATGAGAGTATTTGTGGCGGCTGTGTCTCAAGCTCGTGCCATGCACTCTCATGTTACATCTGTCACACTTCAGCATTCAAGCAATCAAGCAAAGAAATCTGA
BurTARs.A018 TTCCACTTTCTGTCATCATAGTTCTTTATATGAGAGTATTTGTGGCGGCTGTGTCTCAAGCTCGTGCCATGCACTCTCATGTTACATCTGTCACACTTCAGCATTCAAGCAATCAAGCAAAGAAATCTGA
   Consensus TTCCACTTTCTGTCATCATAGTTCTTTATATGAGAGTATTTGTGGCGGCTGTGTCTCAAGCTCGTGCCATGCACTCTCATGTTACATCTGTCACACTTCAGCATTCAAGCAATCAAGCAAAGAAATCTGA

             3251 3260      3270      3280      3290      3300      3310      3320      3330      3340      3350      3360      3370      3380
             |--------+---------+---------+---------+---------+---------+---------+---------+---------+---------+---------+---------+---------|
contig057145 ACTGAAAGCAGCCAGGACTCTGGGGGTTCTTGTAGTTGTGTTTCTGTCATGTTACTCTCCATTTTACTGCTATTATCTCGCTGAAGAAAATGTGGTCAATGATCCATCTGCATCTACTGTTATCATTATT
BurTARs.A018 ACTGAAAGCAGCCAGGACTCTGGGGGTTCTTGTAGTTGTGTTTCTGTCATGTTACTCTCCATTTTACTGCTATTATCTCGCTGAAGAAAATGTGGTCAATGATCCATCTGCATCTACTGTTATCATTATT
   Consensus ACTGAAAGCAGCCAGGACTCTGGGGGTTCTTGTAGTTGTGTTTCTGTCATGTTACTCTCCATTTTACTGCTATTATCTCGCTGAAGAAAATGTGGTCAATGATCCATCTGCATCTACTGTTATCATTATT

             3381 3390      3400      3410      3420      3430      3440      3450      3460      3470      3480      3490      3500      3510
             |--------+---------+---------+---------+---------+---------+---------+---------+---------+---------+---------+---------+---------|
contig057145 TTTTACATTAACTCTTGTCTAAACCCTTTGATCTATGCCTTGTTTTACCCCTGGTTTAGAAATGCTGTTAAACTTATTGTCACTCTGCAGATCTTCAAGCATGGCACCTGTGAGGCCAACATACTATAAA
BurTARs.A018 TTTTACATTAACTCTTGTCTAAACCCTTTGATCTATGCCTTGTTTTACCCCTGGTTTAGAAATGCTGTTAAACTTATTGTCACTCTGCAGATCTTCAAGCATGGCACCTGTGAGGCCAACATACTATAA
   Consensus TTTTACATTAACTCTTGTCTAAACCCTTTGATCTATGCCTTGTTTTACCCCTGGTTTAGAAATGCTGTTAAACTTATTGTCACTCTGCAGATCTTCAAGCATGGCACCTGTGAGGCCAACATACTATAA.
```

```
   Consensus ..................................................................................................................................

             2991  3000      3010      3020      3030      3040      3050      3060      3070      3080      3090      3100      3110      3120
             |--------+---------+---------+---------+---------+---------+---------+---------+---------+---------+---------+---------+---------|
contig056023 ACAGCTGCAGGAACCTGATGATGGAGATTCAGATGCATCCAGAGGCAGGGCTCTGTTTTCCAGAGCTACTGAACAGTTCCTGCAGGAAGCCGACACTTCACTGGTCCAAAACGGTGCTCCTGAACGTTGG
BurTARs.A019                     ATGGAGATTCAGATGCATCCAGAGGCAGGGCTCTGTTTTCCAGAGCTACTGAACAGTTCCTGCAGGAAGCCGACACTTCACTGGTCCAAAACGGTGCTCCTGAACGTTGG
   Consensus ....................ATGGAGATTCAGATGCATCCAGAGGCAGGGCTCTGTTTTCCAGAGCTACTGAACAGTTCCTGCAGGAAGCCGACACTTCACTGGTCCAAAACGGTGCTCCTGAACGTTGG

             3121  3130      3140      3150      3160      3170      3180      3190      3200      3210      3220      3230      3240      3250
             |--------+---------+---------+---------+---------+---------+---------+---------+---------+---------+---------+---------+---------|
contig056023 GCTTTCGTCCATCTCTCTGATCACTGCTGCTCTTAATCTGTTCATCATCATCTCAGTCTCCCACTTCAGGCAGAGATTAACTTCTCAGATGAAACTAAAGTTCAACTTTTAGAAAATCAAGTTTAGTAAT
BurTARs.A019 GCTTTCGTCCATCTCTCTGATCACTGCTGCTCTTAATCTGTTCATCATCATCTCAGTCTCCCACTTCAGGCAG---------------------------------------------------------
   Consensus GCTTTCGTCCATCTCTCTGATCACTGCTGCTCTTAATCTGTTCATCATCATCTCAGTCTCCCACTTCAGGCAG.........................................................

             3251  3260      3270      3280      3290      3300      3310      3320      3330      3340      3350      3360      3370      3380
             |--------+---------+---------+---------+---------+---------+---------+---------+---------+---------+---------+---------+---------|
contig056023 TAGTTAGTAAATTAGTTAAGTAATTAATGAATAATGTCAAGATGAATGTCCTGTGTGTACTTCATCATGACTGTATAGGTTTTATTTCCTTAATTGCACCATGCCTCTAATAGAACATCTTCATTGAAGA
BurTARs.A019 ----------------------------------------------------------------------------------------------------------------------------------
   Consensus ..................................................................................................................................

             3381  3390      3400      3410      3420      3430      3440      3450      3460      3470      3480      3490      3500      3510
             |--------+---------+---------+---------+---------+---------+---------+---------+---------+---------+---------+---------+---------|
contig056023 TATTTTGACATGACTAATGTGTAATTTATAATTTTCTGTCTCCCTGCAGGCAGCTCCACACTCCCAGTAACATCATCATCCTCTCTCTGGCTGTCTCTGACTTTTTTGTCTGTTTTTTGTTGATGCCGGT
BurTARs.A019 -----------------------------------------------------CTCCACACTCCCAGTAACATCATCATCCTCTCTCTGGCTGTCTCTGACTTTTTTGTCTGTTTTTTGTTGATGCCGGT
   Consensus .....................................................CTCCACACTCCCAGTAACATCATCATCCTCTCTCTGGCTGTCTCTGACTTTTTTGTCTGTTTTTTGTTGATGCCGGT

             3511  3520      3530      3540      3550      3560      3570      3580      3590      3600      3610      3620      3630      3640
             |--------+---------+---------+---------+---------+---------+---------+---------+---------+---------+---------+---------+---------|
contig056023 AGAAATCTTCAAAAACACGGCCTGCTGGGTATTTGGTGATCTCATGTGTTCACTTTATACTTATCTGAGCTGCATTCTTATCAATGCTTCATTTGAAATGATTATTCTCGTATCAGTTGACCGCTATGTG
BurTARs.A019 AGAAATCTTCAAAAACACGGCCTGCTGGGTATTTGGTGATCTCATGTGTTCACTTTATACTTATCTGAGCTGCATTCTTATCAATGCTTCATTTGAAATGATTATTCTCGTATCAGTTGACCGCTATGTG
   Consensus AGAAATCTTCAAAAACACGGCCTGCTGGGTATTTGGTGATCTCATGTGTTCACTTTATACTTATCTGAGCTGCATTCTTATCAATGCTTCATTTGAAATGATTATTCTCGTATCAGTTGACCGCTATGTG

             3641  3650      3660      3670      3680      3690      3700      3710      3720      3730      3740      3750      3760      3770
             |--------+---------+---------+---------+---------+---------+---------+---------+---------+---------+---------+---------+---------|
contig056023 GCTATTTGTGACCCTCTGCATTACCCCACCAGAATCACTGTGCCGAGAGTCAAACTCAGTGTTTGTCTGTGCTGGCTTTATGCTATTTTGTACAACATCATCTATACAAAGCATGCCCTGATAAACCCAG
BurTARs.A019 GCTATTTGTGACCCTCTGCATTACCCCACCAGAATCACTGTGCCGAGAGTCAAACTCAGTGTTTGTCTGTGCTGGCTTTATGCTATTTTGTACAACATCATCTATACAAAGCATGCCCTGATAAACCCAG
   Consensus GCTATTTGTGACCCTCTGCATTACCCCACCAGAATCACTGTGCCGAGAGTCAAACTCAGTGTTTGTCTGTGCTGGCTTTATGCTATTTTGTACAACATCATCTATACAAAGCATGCCCTGATAAACCCAG

             3771  3780      3790      3800      3810      3820      3830      3840      3850      3860      3870      3880      3890      3900
             |--------+---------+---------+---------+---------+---------+---------+---------+---------+---------+---------+---------+---------|
contig056023 GCAGGTATGGTTCCTGCTATGGAGAGTGTGTTTTTGTTGTTGATGATATTATTGGAATTGTTGACTTTGTTGTATCTCTTATAGTTCCAGTTACGATCATCGTAGTTCTGTATACGAGAGTGTTTGTGGT
BurTARs.A019 GCAGGTATGGTTCCTGCTATGGAGAGTGTGTTTTTGTTGTTGATGATATTATTGGAATTGTTGACTTTGTTGTATCTCTTATAGTTCCAGTTACGATCATCGTAGTTCTGTATACGAGAGTGTTTGTGGT
   Consensus GCAGGTATGGTTCCTGCTATGGAGAGTGTGTTTTTGTTGTTGATGATATTATTGGAATTGTTGACTTTGTTGTATCTCTTATAGTTCCAGTTACGATCATCGTAGTTCTGTATACGAGAGTGTTTGTGGT

             3901  3910      3920      3930      3940      3950      3960      3970      3980      3990      4000      4010      4020      4030
             |--------+---------+---------+---------+---------+---------+---------+---------+---------+---------+---------+---------+---------|
contig056023 GGTTGTGTCTCAGGCTCGTGCCATGCGCTCTCATGTTACAGCTGTCACACTTCAGCGGCCACTGAATCAAGCAAACAAATCTGAGCTGAAAGCAGCCAGGAACCTTGGGATTCTTGTAGTTGTGTTTCTG
BurTARs.A019 GGTTGTGTCTCAGGCTCGTGCCATGCGCTCTCATGTTACAGCTGTCACACTTCAGCGGCCACTGAATCAAGCAAACAAATCTGAGCTGAAAGCAGCCAGGAACCTTGGGATTCTTGTAGTTGTGTTTCTG
   Consensus GGTTGTGTCTCAGGCTCGTGCCATGCGCTCTCATGTTACAGCTGTCACACTTCAGCGGCCACTGAATCAAGCAAACAAATCTGAGCTGAAAGCAGCCAGGAACCTTGGGATTCTTGTAGTTGTGTTTCTG

             4031  4040      4050      4060      4070      4080      4090      4100      4110      4120      4130      4140      4150      4160
             |--------+---------+---------+---------+---------+---------+---------+---------+---------+---------+---------+---------+---------|
contig056023 GCGTGCTACTGTCCATTTTACTGCTACTTTTTTCTTGCAGGCAATGAAGTTAATGCTTCTTCTGCATCTTCTATCCTCATTGTTTATTATTTTAACTCTTGCCTAAACCCATTAATGTATGCACTGTTTT
BurTARs.A019 GCGTGCTACTGTCCATTTTACTGCTACTTTTTTCTTGCAGGCAATGAAGTTAATGCTTCTTCTGCATCTTCTATCCTCATTGTTTATTATTTTAACTCTTGCCTAAACCCATTAATGTATGCACTGTTTT
   Consensus GCGTGCTACTGTCCATTTTACTGCTACTTTTTTCTTGCAGGCAATGAAGTTAATGCTTCTTCTGCATCTTCTATCCTCATTGTTTATTATTTTAACTCTTGCCTAAACCCATTAATGTATGCACTGTTTT

             4161  4170      4180      4190      4200      4210      4220      4230      4240      4250      4260      4270      4280      4290
             |--------+---------+---------+---------+---------+---------+---------+---------+---------+---------+---------+---------+---------|
contig056023 ACTCCTGGTTTAGAAATGCTGTTAAACTTATCATCACTCTGCAGATATTAAAGGCTAACAGCAGTGAGATCAACATACTACAGAGATAATGCAGAGTGACTTGACTAACATCTGCTATATAAAGATTTCA
BurTARs.A019 ACTCCTGGTTTAGAAATGCTGTTAAACTTATCATCACTCTGCAGATATTAAAGGCTAACAGCAGTGAGATCAACATACTACAGAGATAA
   Consensus ACTCCTGGTTTAGAAATGCTGTTAAACTTATCATCACTCTGCAGATATTAAAGGCTAACAGCAGTGAGATCAACATACTACAGAGATAA.........................................
```

```
             1041  1050      1060      1070      1080      1090      1100      1110      1120      1130      1140      1150      1160      1170
             |--------+---------+---------+---------+---------+---------+---------+---------+---------+---------+---------+---------+---------|
contig056021 GTCAGAGAGCTGCAGAGGTTGTAGTTGTCTGATGATGGAGATACAGAAAGGAGCTGAGCTCTGTTTTCCACAACTCCTCAACATTTCCTGCAGGAAGCCGACGCTTCACTGGTCCAAAGCTGTGCTCCTG
BurTARs.A020                                   ATGGAGATACAGAAAGGAGCTGAGCTCTGTTTTCCACAACTCCTCAACATTTCCTGCAGGAAGCCGACGCTTCACTGGTCCAAAGCTGTGCTCCTG
   Consensus .................................ATGGAGATACAGAAAGGAGCTGAGCTCTGTTTTCCACAACTCCTCAACATTTCCTGCAGGAAGCCGACGCTTCACTGGTCCAAAGCTGTGCTCCTG

             1171  1180      1190      1200      1210      1220      1230      1240      1250      1260      1270      1280      1290      1300
             |--------+---------+---------+---------+---------+---------+---------+---------+---------+---------+---------+---------+---------|
contig056021 AACATTGTCCTGTCCTGCATCTCTCTGCTCACTGCTGCTCTAAACCTTCTCGTGATCATCTCAGTCTCCTACTTCAGGCACATATAATGTTTCTAAAACTTAAACTGTATCTTCATTTAAAAAGTTTAAC
BurTARs.A020 AACATTGTCCTGTCCTGCATCTCTCTGCTCACTGCTGCTCTAAACCTTCTCGTGATCATCTCAGTCTCCTACTTCAGG--------------------------------------------------
   Consensus AACATTGTCCTGTCCTGCATCTCTCTGCTCACTGCTGCTCTAAACCTTCTCGTGATCATCTCAGTCTCCTACTTCAGG..................................................

             1301  1310      1320      1330      1340      1350      1360      1370      1380      1390      1400      1410      1420      1430
             |--------+---------+---------+---------+---------+---------+---------+---------+---------+---------+---------+---------+---------|
contig056021 ACCATGATTTATTATCTAAATTGAAATTAATTTGGCACATTCTTGGTCAATTTAAACCACAATATTTAATTTTTATACACCTTAATGTCTTTGTCTTACAGTCTTAATAATGGACCTTTTTACATTTTAA
BurTARs.A020 ----------------------------------------------------------------------------------------------------------------------------------
   Consensus ..................................................................................................................................

             1431  1440      1450      1460      1470      1480      1490      1500      1510      1520      1530      1540      1550      1560
             |--------+---------+---------+---------+---------+---------+---------+---------+---------+---------+---------+---------+---------|
contig056021 AATTACTAATATATTCTTTTTTGTTCTATTTCAGGAAGCTTCACACACCCAGCAACATCCTCCTACTCTCTCTTGCTGTCTCAGACTTTCTCATGGGTCTCTTGCTGATGCCAGCAGAAATCCTTAGAAG
BurTARs.A020 -----------------------------------AAGCTTCACACACCCAGCAACATCCTCCTACTCTCTCTTGCTGTCTCAGACTTTCTCATGGGTCTCTTGCTGATGCCAGCAGAAATCCTTAGAAG
   Consensus ...................................AAGCTTCACACACCCAGCAACATCCTCCTACTCTCTCTTGCTGTCTCAGACTTTCTCATGGGTCTCTTGCTGATGCCAGCAGAAATCCTTAGAAG

             1561  1570      1580      1590      1600      1610      1620      1630      1640      1650      1660      1670      1680      1690
             |--------+---------+---------+---------+---------+---------+---------+---------+---------+---------+---------+---------+---------|
contig056021 CATGACGTGCTGGGTACTTGGTGATCTTATGTGTTCTGTTTATTTTTTTCTGACTGTAAACCTTACCTGTGCTTCAATAGGGAACATAGTTCTTATATCGATTGACCGTTATGTGGCTATTTGTGACCCT
BurTARs.A020 CATGACGTGCTGGGTACTTGGTGATCTTATGTGTTCTGTTTATTTTTTTCTGACTGTAAACCTTACCTGTGCTTCAATAGGGAACATAGTTCTTATATCGATTGACCGTTATGTGGCTATTTGTGACCCT
   Consensus CATGACGTGCTGGGTACTTGGTGATCTTATGTGTTCTGTTTATTTTTTTCTGACTGTAAACCTTACCTGTGCTTCAATAGGGAACATAGTTCTTATATCGATTGACCGTTATGTGGCTATTTGTGACCCT

             1691  1700      1710      1720      1730      1740      1750      1760      1770      1780      1790      1800      1810      1820
             |--------+---------+---------+---------+---------+---------+---------+---------+---------+---------+---------+---------+---------|
contig056021 CTGCATTACTCCACAAGAATTACTGTGGCAAGAGTCAAACTCAGTGTTTGTCTATGTTGGTTTTATTCAACTTTCTACTGCAGTCTTTATACGCAAGAAATGCTGATTGAACCAGGCAGGTATAATTCCT
BurTARs.A020 CTGCATTACTCCACAAGAATTACTGTGGCAAGAGTCAAACTCAGTGTTTGTCTATGTTGGTTTTATTCAACTTTCTACTGCAGTCTTTATACGCAAGAAATGCTGATTGAACCAGGCAGGTATAATTCCT
   Consensus CTGCATTACTCCACAAGAATTACTGTGGCAAGAGTCAAACTCAGTGTTTGTCTATGTTGGTTTTATTCAACTTTCTACTGCAGTCTTTATACGCAAGAAATGCTGATTGAACCAGGCAGGTATAATTCCT

             1821  1830      1840      1850      1860      1870      1880      1890      1900      1910      1920      1930      1940      1950
             |--------+---------+---------+---------+---------+---------+---------+---------+---------+---------+---------+---------+---------|
contig056021 GTTATGGTGAGTGTGTGCTTGTCATCAGTGATTTTGCTGGAATGGTTGACCTTATTTTATTTTTTTATTCTTCCAGTTATTATCATCATAGTTCTGTATACAAGAGTATTTGTGGTGGCTGTGTCTCAGGC
BurTARs.A020 GTTATGGTGAGTGTGTGCTTGTCATCAGTGATTTTGCTGGAATGGTTGACCTTATTTTATTTTTTTATTCTTCCAGTTATTATCATCATAGTTCTGTATACAAGAGTATTTGTGGTGGCTGTGTCTCAGGC
   Consensus GTTATGGTGAGTGTGTGCTTGTCATCAGTGATTTTGCTGGAATGGTTGACCTTATTTTATTTTTTTATTCTTCCAGTTATTATCATCATAGTTCTGTATACAAGAGTATTTGTGGTGGCTGTGTCTCAGGC

             1951  1960      1970      1980      1990      2000      2010      2020      2030      2040      2050      2060      2070      2080
             |--------+---------+---------+---------+---------+---------+---------+---------+---------+---------+---------+---------+---------|
contig056021 TCGTGCCATGCGCTCTCATGTTACGGCTGTCACACTTCAGCGTTCATTGAATCAAACAAACAAATCTGAGCTAAAAGCAGCCAGGACTCTGGGGGTTCTTGTAGTTGTGTTTCTGTCATGCTACTGTCCA
BurTARs.A020 TCGTGCCATGCGCTCTCATGTTACGGCTGTCACACTTCAGCGTTCATTGAATCAAACAAACAAATCTGAGCTAAAAGCAGCCAGGACTCTGGGGGTTCTTGTAGTTGTGTTTCTGTCATGCTACTGTCCA
   Consensus TCGTGCCATGCGCTCTCATGTTACGGCTGTCACACTTCAGCGTTCATTGAATCAAACAAACAAATCTGAGCTAAAAGCAGCCAGGACTCTGGGGGTTCTTGTAGTTGTGTTTCTGTCATGCTACTGTCCA

             2081  2090      2100      2110      2120      2130      2140      2150      2160      2170      2180      2190      2200      2210
             |--------+---------+---------+---------+---------+---------+---------+---------+---------+---------+---------+---------+---------|
contig056021 TTTTATTGCTACTCTCTCACTGATAAAAATGCTGTCAATGATCCAGCTGCATCTTTTGTGATCTTTATCTTTTATTTTAACTCTTGTATAAACCCTTTGATGTATGCCCTGTTTTACCCCTGGTTTAGAA
BurTARs.A020 TTTTATTGCTACTCTCTCACTGATAAAAATGCTGTCAATGATCCAGCTGCATCTTTTGTGATCTTTATCTTTTATTTTAACTCTTGTATAAACCCTTTGATGTATGCCCTGTTTTACCCCTGGTTTAGAA
   Consensus TTTTATTGCTACTCTCTCACTGATAAAAATGCTGTCAATGATCCAGCTGCATCTTTTGTGATCTTTATCTTTTATTTTAACTCTTGTATAAACCCTTTGATGTATGCCCTGTTTTACCCCTGGTTTAGAA

             2211  2220      2230      2240      2250      2260      2270      2280      2290      2300      2310      2320      2330      2340
             |--------+---------+---------+---------+---------+---------+---------+---------+---------+---------+---------+---------+---------|
contig056021 ATGCTGTTAAACTTATTATCACATTGCAGATATTCAAGCATAACTCCTGTGAGGCCAACATACTATAAAAAGACTATGAACCCTGCTCCAGTAAACTGCTTTAAATCAGGAAATGGGCCCATGTGGTAAT
BurTARs.A020 ATGCTGTTAAACTTATTATCACATTGCAGATATTCAAGCATAACTCCTGTGAGGCCAACATACTATAA
   Consensus ATGCTGTTAAACTTATTATCACATTGCAGATATTCAAGCATAACTCCTGTGAGGCCAACATACTATAA............................................................
```

```
             5721 5730      5740      5750      5760      5770      5780      5790      5800      5810      5820      5830      5840      5850
             |--------+---------+---------+---------+---------+---------+---------+---------+---------+---------+---------+---------+---------|
contig056020 TCTTGAAAACTGAAAGAGCTTGGTGAGAAGGTGGAGATTAATCAGCTTTATAAGAAAAAGTTCTGATCAGAGAGCTGCGGCTGTCTGATGATGGAGATACAGAAAGAAAACAAGTTGTGTTTTCCACAAC
BurTARs.A021                                                                                           ATGGAGATACAGAAAGAAAACAAGTTGTGTTTTCCACAAC
   Consensus ..........................................................................................ATGGAGATACAGAAAGAAAACAAGTTGTGTTTTCCACAAC

             5851 5860      5870      5880      5890      5900      5910      5920      5930      5940      5950      5960      5970      5980
             |--------+---------+---------+---------+---------+---------+---------+---------+---------+---------+---------+---------+---------|
contig056020 TCCTCAACAGCTCCTGCAGGAAGCCAACACTTCACTGGTCCAAAGATGTGCTCCTGAACACTGTGCTGTCCTGCATCTCTCTGCTCACCGCTGCTCTAAACCTTCTCGTCATCATCTCAGTCTCTTACTT
BurTARs.A021 TCCTCAACAGCTCCTGCAGGAAGCCAACACTTCACTGGTCCAAAGATGTGCTCCTGAACACTGTGCTGTCCTGCATCTCTCTGCTCACCGCTGCTCTAAACCTTCTCGTCATCATCTCAGTCTCTTACTT
   Consensus TCCTCAACAGCTCCTGCAGGAAGCCAACACTTCACTGGTCCAAAGATGTGCTCCTGAACACTGTGCTGTCCTGCATCTCTCTGCTCACCGCTGCTCTAAACCTTCTCGTCATCATCTCAGTCTCTTACTT

             5981 5990      6000      6010      6020      6030      6040      6050      6060      6070      6080      6090      6100      6110
             |--------+---------+---------+---------+---------+---------+---------+---------+---------+---------+---------+---------+---------|
contig056020 CAGGCAGAGATTAATTTTAAATATATGACAACTTTAATGTACATTATATCACTTAAGTGAATTTTGCTTGATGTATTTTAACCATGATTGTAATGTCCTGTTTGCCTAGACTGAATCAAGCCTTACTAAT
BurTARs.A021 CAGG------------------------------------------------------------------------------------------------------------------------------
   Consensus CAGG..............................................................................................................................

             6111 6120      6130      6140      6150      6160      6170      6180      6190      6200      6210      6220      6230      6240
             |--------+---------+---------+---------+---------+---------+---------+---------+---------+---------+---------+---------+---------|
contig056020 GCAGCATTTTCAATGAAGACAGTTTGACATTAATAATGTGCATTAAATTCTTTTGTGTCTCTGCAGGAAGCTTCACACACCCAGTAATATCCTCCTCCTTTCTCTGGCTGTCTCAGACTTTCTTGTCGGT
BurTARs.A021 -------------------------------------------------------------------AAGCTTCACACACCCAGTAATATCCTCCTCCTTTCTCTGGCTGTCTCAGACTTTCTTGTCGGT
   Consensus ...................................................................AAGCTTCACACACCCAGTAATATCCTCCTCCTTTCTCTGGCTGTCTCAGACTTTCTTGTCGGT

             6241 6250      6260      6270      6280      6290      6300      6310      6320      6330      6340      6350      6360      6370
             |--------+---------+---------+---------+---------+---------+---------+---------+---------+---------+---------+---------+---------|
contig056020 CTCCTGTTGATGCCATTAGGAATCCTTAGAAACACAGGCTGCTGGGTACTTGGTGATATAATTTGTTCTCTTTATTGGTATTTAACCAGCAACATTGTCTGTGCTTCAATAGGGAACATTGTCCTAATAT
BurTARs.A021 CTCCTGTTGATGCCATTAGGAATCCTTAGAAACACAGGCTGCTGGGTACTTGGTGATATAATTTGTTCTCTTTATTGGTATTTAACCAGCAACATTGTCTGTGCTTCAATAGGGAACATTGTCCTAATAT
   Consensus CTCCTGTTGATGCCATTAGGAATCCTTAGAAACACAGGCTGCTGGGTACTTGGTGATATAATTTGTTCTCTTTATTGGTATTTAACCAGCAACATTGTCTGTGCTTCAATAGGGAACATTGTCCTAATAT

             6371 6380      6390      6400      6410      6420      6430      6440      6450      6460      6470      6480      6490      6500
             |--------+---------+---------+---------+---------+---------+---------+---------+---------+---------+---------+---------+---------|
contig056020 CAGTTGACCGCTATGTGGCTATTTGTGACCCTCTGCATTATCCCAGCAGAATTACTTTGGCGAAAGTCAAACTCAGTGTTTGTCTGTGTTGGTTTTATGCTTTTTTCTACGTCAGTCTTTATACAAAGGA
BurTARs.A021 CAGTTGACCGCTATGTGGCTATTTGTGACCCTCTGCATTATCCCAGCAGAATTACTTTGGCGAAAGTCAAACTCAGTGTTTGTCTGTGTTGGTTTTATGCTTTTTTCTACGTCAGTCTTTATACAAAGGA
   Consensus CAGTTGACCGCTATGTGGCTATTTGTGACCCTCTGCATTATCCCAGCAGAATTACTTTGGCGAAAGTCAAACTCAGTGTTTGTCTGTGTTGGTTTTATGCTTTTTTCTACGTCAGTCTTTATACAAAGGA

             6501 6510      6520      6530      6540      6550      6560      6570      6580      6590      6600      6610      6620      6630
             |--------+---------+---------+---------+---------+---------+---------+---------+---------+---------+---------+---------+---------|
contig056020 TATCCTAATTGAACCAGGCAGGGATAATTCCTGCTACGGAGAGTGTGCATTTGTCATCAATGATATTGCTGTTGTTGTTGACCTTGTTTTTTCCTTTATTGTTCCGGTTTCTGTCATTGTAGTTCTGTAT
BurTARs.A021 TATCCTAATTGAACCAGGCAGGGATAATTCCTGCTACGGAGAGTGTGCATTTGTCATCAATGATATTGCTGTTGTTGTTGACCTTGTTTTTTCCTTTATTGTTCCGGTTTCTGTCATTGTAGTTCTGTAT
   Consensus TATCCTAATTGAACCAGGCAGGGATAATTCCTGCTACGGAGAGTGTGCATTTGTCATCAATGATATTGCTGTTGTTGTTGACCTTGTTTTTTCCTTTATTGTTCCGGTTTCTGTCATTGTAGTTCTGTAT

             6631 6640      6650      6660      6670      6680      6690      6700      6710      6720      6730      6740      6750      6760
             |--------+---------+---------+---------+---------+---------+---------+---------+---------+---------+---------+---------+---------|
contig056020 ATCAGAGTATTTGTGGCAGCTGTGTCTCAGGCTCGTGCCATGCGCTCTCATGTGACATCTGTCACACTGCAGCGCTCACTGAATCAAACAAACAAATCTGAGCTGAAAGCAGCCAGGACTCTTGGAATTC
BurTARs.A021 ATCAGAGTATTTGTGGCAGCTGTGTCTCAGGCTCGTGCCATGCGCTCTCATGTGACATCTGTCACACTGCAGCGCTCACTGAATCAAACAAACAAATCTGAGCTGAAAGCAGCCAGGACTCTTGGAATTC
   Consensus ATCAGAGTATTTGTGGCAGCTGTGTCTCAGGCTCGTGCCATGCGCTCTCATGTGACATCTGTCACACTGCAGCGCTCACTGAATCAAACAAACAAATCTGAGCTGAAAGCAGCCAGGACTCTTGGAATTC

             6761 6770      6780      6790      6800      6810      6820      6830      6840      6850      6860      6870      6880      6890
             |--------+---------+---------+---------+---------+---------+---------+---------+---------+---------+---------+---------+---------|
contig056020 TTGTAGTTGTGTTTCTGGCATGCTTCTGTCCACTCTACTACTTTTCTCTTGTTGATGAAAATGCAATCAATGATCCAGCTGCATCTTTTGTGGTCATTATATTTTACTTTAACTCGTGTCTAAATCCTTT
BurTARs.A021 TTGTAGTTGTGTTTCTGGCATGCTTCTGTCCACTCTACTACTTTTCTCTTGTTGATGAAAATGCAATCAATGATCCAGCTGCATCTTTTGTGGTCATTATATTTTACTTTAACTCGTGTCTAAATCCTTT
   Consensus TTGTAGTTGTGTTTCTGGCATGCTTCTGTCCACTCTACTACTTTTCTCTTGTTGATGAAAATGCAATCAATGATCCAGCTGCATCTTTTGTGGTCATTATATTTTACTTTAACTCGTGTCTAAATCCTTT

             6891 6900      6910      6920      6930      6940      6950      6960      6970      6980      6990      7000      7010      7020
             |--------+---------+---------+---------+---------+---------+---------+---------+---------+---------+---------+---------+---------|
contig056020 GATCTATGCCTTCTTTTACCCCTGGTTTAGAAATGCTGTTAAGCTTATCATCACTCTGCAGATTTTCAAGTATAATATGAGTGAGGCCAACATACTATAAAAAGAGTATGAAACTTGCTCTACTAAGATA
BurTARs.A021 GATCTATGCCTTCTTTTACCCCTGGTTTAGAAATGCTGTTAAGCTTATCATCACTCTGCAGATTTTCAAGTATAATATGAGTGAGGCCAACATACTATAA
   Consensus GATCTATGCCTTCTTTTACCCCTGGTTTAGAAATGCTGTTAAGCTTATCATCACTCTGCAGATTTTCAAGTATAATATGAGTGAGGCCAACATACTATAA..............................
```

```
             1951 1960      1970      1980      1990      2000      2010      2020      2030      2040      2050      2060      2070      2080
             |--------+---------+---------+---------+---------+---------+---------+---------+---------+---------+---------+---------+---------|
contig055697 GAGGACAAAGATATATGCTGTATGACACAGAATATGATTAGCAGCTCGCCTCACTGTGATGATGGAGGAACCTGAACTCTGCTTTCCAAAACTCCTTAATATCTCTTGCAGCAGGCCAAAGCGTCCTCAC
BurTARs.A022                                                           ATGATGGAGGAACCTGAACTCTGCTTTCCAAAACTCCTTAATATCTCTTGCAGCAGGCCAAAGCGTCCTCAC
   Consensus ..........................................................ATGATGGAGGAACCTGAACTCTGCTTTCCAAAACTCCTTAATATCTCTTGCAGCAGGCCAAAGCGTCCTCAC

             2081 2090      2100      2110      2120      2130      2140      2150      2160      2170      2180      2190      2200      2210
             |--------+---------+---------+---------+---------+---------+---------+---------+---------+---------+---------+---------+---------|
contig055697 TTTGAGATCATGCTGACTTACATTCTGCTCTCCTTCATTTCTTTGCTTACTGTGATTCTTAACCTGCTGGTCATTATCTCCATCTCACACTTCAGGTATGAAAATGTTTCATTATCTCCAAATATTGAAT
BurTARs.A022 TTTGAGATCATGCTGACTTACATTCTGCTCTCCTTCATTTCTTTGCTTACTGTGATTCTTAACCTGCTGGTCATTATCTCCATCTCACACTTCAGG----------------------------------
   Consensus TTTGAGATCATGCTGACTTACATTCTGCTCTCCTTCATTTCTTTGCTTACTGTGATTCTTAACCTGCTGGTCATTATCTCCATCTCACACTTCAGG..................................

             2211 2220      2230      2240      2250      2260      2270      2280      2290      2300      2310      2320      2330      2340
             |--------+---------+---------+---------+---------+---------+---------+---------+---------+---------+---------+---------+---------|
contig055697 AATTATTTTAATAGTTTAGTTAATTGTGCTGTGTAGCAACATAGCAGTAATAGTAATTAAAGCCATAATATGATCTGTACCTACAAGTAAGAGTAGACCTTTTTTTAATGAAGTGCATTTCTATTTCAGT
BurTARs.A022 ----------------------------------------------------------------------------------------------------------------------------------
   Consensus ..................................................................................................................................

             2341 2350      2360      2370      2380      2390      2400      2410      2420      2430      2440      2450      2460      2470
             |--------+---------+---------+---------+---------+---------+---------+---------+---------+---------+---------+---------+---------|
contig055697 TTTCGTTGTATGTGCATCCACATGCATTTTTGATGAAGGAAACTAAATATGATGCTCCTTTTTCTCTCCAGGCAGCTCCACACCCCCACCAACTTCCTCCTTCTCTCTCTGGCTGTCGCTGATTTCTTTG
BurTARs.A022 ------------------------------------------------------------------------CAGCTCCACACCCCCACCAACTTCCTCCTTCTCTCTCTGGCTGTCGCTGATTTCTTTG
   Consensus ........................................................................CAGCTCCACACCCCCACCAACTTCCTCCTTCTCTCTCTGGCTGTCGCTGATTTCTTTG

             2471 2480      2490      2500      2510      2520      2530      2540      2550      2560      2570      2580      2590      2600
             |--------+---------+---------+---------+---------+---------+---------+---------+---------+---------+---------+---------+---------|
contig055697 TAGGTCTCCTTATGTTCTTTCAAATAGTGCTCATCGATGGATGCTGGTTCCTCGGTGACATCATGTGCACTCTGTATCAGTATCTAGCATTCATTATTACTTCAGCCTCAGTAGGAACCATGGTGATCAT
BurTARs.A022 TAGGTCTCCTTATGTTCTTTCAAATAGTGCTCATCGATGGATGCTGGTTCCTCGGTGACATCATGTGCACTCTGTATCAGTATCTAGCATTCATTATTACTTCAGCCTCAGTAGGAACCATGGTGATCAT
   Consensus TAGGTCTCCTTATGTTCTTTCAAATAGTGCTCATCGATGGATGCTGGTTCCTCGGTGACATCATGTGCACTCTGTATCAGTATCTAGCATTCATTATTACTTCAGCCTCAGTAGGAACCATGGTGATCAT

             2601 2610      2620      2630      2640      2650      2660      2670      2680      2690      2700      2710      2720      2730
             |--------+---------+---------+---------+---------+---------+---------+---------+---------+---------+---------+---------+---------|
contig055697 ATCTGCTGATAGGTATTTGGCTATTTGTTACCCTCTGCATTACTCCACCCAAATAACACAACAAAGAGTTAATATATGTATAAGTTTGTGTTGGTTTTTTTCTGTGATCTTTCAGAGTTTGATTGTGAAG
BurTARs.A022 ATCTGCTGATAGGTATTTGGCTATTTGTTACCCTCTGCATTACTCCACCCAAATAACACAACAAAGAGTTAATATATGTATAAGTTTGTGTTGGTTTTTTTCTGTGATCTTTCAGAGTTTGATTGTGAAG
   Consensus ATCTGCTGATAGGTATTTGGCTATTTGTTACCCTCTGCATTACTCCACCCAAATAACACAACAAAGAGTTAATATATGTATAAGTTTGTGTTGGTTTTTTTCTGTGATCTTTCAGAGTTTGATTGTGAAG

             2731 2740      2750      2760      2770      2780      2790      2800      2810      2820      2830      2840      2850      2860
             |--------+---------+---------+---------+---------+---------+---------+---------+---------+---------+---------+---------+---------|
contig055697 GATAACTTGAAACAACCAGGAAAGTATAACTCCTGCATTGGAGAGTGTGTCTTTGTCGTTAACTACATTGCTGGGCTTTTTGATCTTTTGTTTTCCTTCATTGTTCCCATTACTGTGATTGTAGTTCTGT
BurTARs.A022 GATAACTTGAAACAACCAGGAAAGTATAACTCCTGCATTGGAGAGTGTGTCTTTGTCGTTAACTACATTGCTGGGCTTTTTGATCTTTTGTTTTCCTTCATTGTTCCCATTACTGTGATTGTAGTTCTGT
   Consensus GATAACTTGAAACAACCAGGAAAGTATAACTCCTGCATTGGAGAGTGTGTCTTTGTCGTTAACTACATTGCTGGGCTTTTTGATCTTTTGTTTTCCTTCATTGTTCCCATTACTGTGATTGTAGTTCTGT

             2861 2870      2880      2890      2900      2910      2920      2930      2940      2950      2960      2970      2980      2990
             |--------+---------+---------+---------+---------+---------+---------+---------+---------+---------+---------+---------+---------|
contig055697 ATCTGAGAGTGTTTGTGGTGGCTGTGTCTCAGGCTCGTGCCATGAGGTGTCAACTTGCAGTCACTCACCAGCGATCAGTAACAGTAACTGTTACGAAATCGGAGCTGAAAGCAGCCCGTACTCTTGGTGT
BurTARs.A022 ATCTGAGAGTGTTTGTGGTGGCTGTGTCTCAGGCTCGTGCCATGAGGTGTCAACTTGCAGTCACTCACCAGCGATCAGTAACAGTAACTGTTACGAAATCGGAGCTGAAAGCAGCCCGTACTCTTGGTGT
   Consensus ATCTGAGAGTGTTTGTGGTGGCTGTGTCTCAGGCTCGTGCCATGAGGTGTCAACTTGCAGTCACTCACCAGCGATCAGTAACAGTAACTGTTACGAAATCGGAGCTGAAAGCAGCCCGTACTCTTGGTGT

             2991 3000      3010      3020      3030      3040      3050      3060      3070      3080      3090      3100      3110      3120
             |--------+---------+---------+---------+---------+---------+---------+---------+---------+---------+---------+---------+---------|
contig055697 TGTTGTAGTTGTGTTTCTTATATGTATGTGCCCATATTACTGCGTTGCTCTCACAGGCCAAGATAACTTCCTAAATGCTTCATCTGCTGCCTTTGTAATTTGTTTGGTGTACTTTAACTCTTGCCTAAAC
BurTARs.A022 TGTTGTAGTTGTGTTTCTTATATGTATGTGCCCATATTACTGCGTTGCTCTCACAGGCCAAGATAACTTCCTAAATGCTTCATCTGCTGCCTTTGTAATTTGTTTGGTGTACTTTAACTCTTGCCTAAAC
   Consensus TGTTGTAGTTGTGTTTCTTATATGTATGTGCCCATATTACTGCGTTGCTCTCACAGGCCAAGATAACTTCCTAAATGCTTCATCTGCTGCCTTTGTAATTTGTTTGGTGTACTTTAACTCTTGCCTAAAC

             3121 3130      3140      3150      3160      3170      3180      3190      3200      3210      3220      3230      3240      3250
             |--------+---------+---------+---------+---------+---------+---------+---------+---------+---------+---------+---------+---------|
contig055697 CCTATCATTTATGTCTTTTTTTATCCCTGGTTCAGAAAGTCAATCAAACTTATTGCTACTCTTCAAATACTGCAGCCTGACTCCCGTGAGACTAACATGCATTAAGAACACTTTTTAAAACCGTACCCAT
BurTARs.A022 CCTATCATTTATGTCTTTTTTTATCCCTGGTTCAGAAAGTCAATCAAACTTATTGCTACTCTTCAAATACTGCAGCCTGACTCCCGTGAGACTAACATGCATTAA
   Consensus CCTATCATTTATGTCTTTTTTTATCCCTGGTTCAGAAAGTCAATCAAACTTATTGCTACTCTTCAAATACTGCAGCCTGACTCCCGTGAGACTAACATGCATTAA.........................
```

```
             9231 9240      9250      9260      9270      9280      9290      9300      9310      9320      9330      9340      9350      9360
             |--------+---------+---------+---------+---------+---------+---------+---------+---------+---------+---------+---------+---------|
contig054630 ACGTCACATTACACGGATAGCTTTAGTCAGAAAGGTGCCAGTGTTCAGCAGCTTTCATCTTCTTTTCCCTTTCTGCTCATGGAGAACGTGTATGGACAACAATTCTGCTTTCCACAGCTCCCCAACACCT
 BurTARs.A023                                                                               ATGGAGAACGTGTATGGACAACAATTCTGCTTTCCACAGCTCCCCAACACCT
    Consensus ..............................................................................ATGGAGAACGTGTATGGACAACAATTCTGCTTTCCACAGCTCCCCAACACCT

             9361 9370      9380      9390      9400      9410      9420      9430      9440      9450      9460      9470      9480      9490
             |--------+---------+---------+---------+---------+---------+---------+---------+---------+---------+---------+---------+---------|
contig054630 CCTGTAGGAAGCCGCTGCAACATCACAATCAGACCATTTTTCTCTACATTCTGCTTTGCTGCATCTCTCTGCTCACAGTGACTCTCAACGTCCTGGTCATCATCTCTATCTCCCACTTCAGGTGTTAAAG
 BurTARs.A023 CCTGTAGGAAGCCGCTGCAACATCACAATCAGACCATTTTTCTCTACATTCTGCTTTGCTGCATCTCTCTGCTCACAGTGACTCTCAACGTCCTGGTCATCATCTCTATCTCCCACTTCAGG--------
    Consensus CCTGTAGGAAGCCGCTGCAACATCACAATCAGACCATTTTTCTCTACATTCTGCTTTGCTGCATCTCTCTGCTCACAGTGACTCTCAACGTCCTGGTCATCATCTCTATCTCCCACTTCAGG........

             9491 9500      9510      9520      9530      9540      9550      9560      9570      9580      9590      9600      9610      9620
             |--------+---------+---------+---------+---------+---------+---------+---------+---------+---------+---------+---------+---------|
contig054630 TAAACTTTACTGTGATATATTGTGTTTGACTTGAGCATACTTGGATTAGCTACAGAGTACTCTTCACATTTGGCCTTTACTAAACTGTAATAGCTCTCTTTTAGAAAGGAACAGCTGATGTGATAAATGT
 BurTARs.A023 ----------------------------------------------------------------------------------------------------------------------------------
    Consensus ..................................................................................................................................

             9621 9630      9640      9650      9660      9670      9680      9690      9700      9710      9720      9730      9740      9750
             |--------+---------+---------+---------+---------+---------+---------+---------+---------+---------+---------+---------+---------|
contig054630 TTGTACTATTTAACTGAAATGATTTCATATTCTGACCTCCTCCTCTCAGAATGATGTGGAAACGAGAGGTTTGCCCTACTTTGTTGCACTGGATTGTTTATTGTATGTTGTTTTACTTTCAGAACTGGTG
 BurTARs.A023 ----------------------------------------------------------------------------------------------------------------------------------
    Consensus ..................................................................................................................................

             9751 9760      9770      9780      9790      9800      9810      9820      9830      9840      9850      9860      9870      9880
             |--------+---------+---------+---------+---------+---------+---------+---------+---------+---------+---------+---------+---------|
contig054630 CATTACTGTAGCATTCCACCTGCTGCAGGTTTATTAATATTATGTTACGTCTTTCTTTCTGTTTTTTTCTTCTCAGGCAGCTTCACAACCAAACCAACCTCTTCCTCCTCTCTCTCGCTGTCTCAGACCT
 BurTARs.A023 -----------------------------------------------------------------------------CAGCTTCACAACCAAACCAACCTCTTCCTCCTCTCTCTCGCTGTCTCAGACCT
    Consensus .............................................................................CAGCTTCACAACCAAACCAACCTCTTCCTCCTCTCTCTCGCTGTCTCAGACCT

             9881 9890      9900      9910      9920      9930      9940      9950      9960      9970      9980      9990      10000     10010
             |--------+---------+---------+---------+---------+---------+---------+---------+---------+---------+---------+---------+---------|
contig054630 CCTTGTTGGCCTCCTGCTGATGCCGCCTCGAATCCTCCTTTTAGGGGGCTGCTGGTTTTTGGGCACCTTCATGTGTGGACTGTTTTACTATGCCTCTTTTGTTCTCACATCTGCCTCAGTAGGAAACATG
 BurTARs.A023 CCTTGTTGGCCTCCTGCTGATGCCGCCTCGAATCCTCCTTTTAGGGGGCTGCTGGTTTTTGGGCACCTTCATGTGTGGACTGTTTTACTATGCCTCTTTTGTTCTCACATCTGCCTCAGTAGGAAACATG
    Consensus CCTTGTTGGCCTCCTGCTGATGCCGCCTCGAATCCTCCTTTTAGGGGGCTGCTGGTTTTTGGGCACCTTCATGTGTGGACTGTTTTACTATGCCTCTTTTGTTCTCACATCTGCCTCAGTAGGAAACATG

             1001110020     10030     10040     10050     10060     10070     10080     10090     10100     10110     10120     10130     10140
             |--------+---------+---------+---------+---------+---------+---------+---------+---------+---------+---------+---------+---------|
contig054630 GTGCTTATTTCATTTGACCGGTATGTAGCTATTTGTGACCCCTTGTCCTACCCCACTGCAGTTACTGAGAGAAAAGTCCAAATCAGTGTCTGTCTGTGTTGGGCCTGTTCACTGCTGTATAACGGCACAA
 BurTARs.A023 GTGCTTATTTCATTTGACCGGTATGTAGCTATTTGTGACCCCTTGTCCTACCCCACTGCAGTTACTGAGAGAAAAGTCCAAATCAGTGTCTGTCTGTGTTGGGCCTGTTCACTGCTGTATAACGGCACAA
    Consensus GTGCTTATTTCATTTGACCGGTATGTAGCTATTTGTGACCCCTTGTCCTACCCCACTGCAGTTACTGAGAGAAAAGTCCAAATCAGTGTCTGTCTGTGTTGGGCCTGTTCACTGCTGTATAACGGCACAA

             1014110150     10160     10170     10180     10190     10200     10210     10220     10230     10240     10250     10260     10270
             |--------+---------+---------+---------+---------+---------+---------+---------+---------+---------+---------+---------+---------|
contig054630 TACTAAACAACTTTCTGAAGCAGCCAGACAGATATAATTCTTGTGACGGGGAATGCATAGTTGTAATTAATTTCATAACTGGTGCTTTTGATGTCGTAGCTACCTTCATCGGCCCCACTGCTGTGATTAT
 BurTARs.A023 TACTAAACAACTTTCTGAAGCAGCCAGACAGATATAATTCTTGTGACGGGGAATGCATAGTTGTAATTAATTTCATAACTGGTGCTTTTGATGTCGTAGCTACCTTCATCGGCCCCACTGCTGTGATTAT
    Consensus TACTAAACAACTTTCTGAAGCAGCCAGACAGATATAATTCTTGTGACGGGGAATGCATAGTTGTAATTAATTTCATAACTGGTGCTTTTGATGTCGTAGCTACCTTCATCGGCCCCACTGCTGTGATTAT

             1027110280     10290     10300     10310     10320     10330     10340     10350     10360     10370     10380     10390     10400
             |--------+---------+---------+---------+---------+---------+---------+---------+---------+---------+---------+---------+---------|
contig054630 ATTTCTGTACATGAGAGTATTTCTGGTGGCTGTGTCTCAGGCTCAGGCTATGAGATCTCATGTTGCCTTTGTTACTTCAAAGGGTTCAGTTCACGTTGCCATTAAGAAGTCTGAGAGAAAAGCAGCTACA
 BurTARs.A023 ATTTCTGTACATGAGAGTATTTCTGGTGGCTGTGTCTCAGGCTCAGGCTATGAGATCTCATGTTGCCTTTGTTACTTCAAAGGGTTCAGTTCACGTTGCCATTAAGAAGTCTGAGAGAAAAGCAGCTACA
    Consensus ATTTCTGTACATGAGAGTATTTCTGGTGGCTGTGTCTCAGGCTCAGGCTATGAGATCTCATGTTGCCTTTGTTACTTCAAAGGGTTCAGTTCACGTTGCCATTAAGAAGTCTGAGAGAAAAGCAGCTACA

             1040110410     10420     10430     10440     10450     10460     10470     10480     10490     10500     10510     10520     10530
             |--------+---------+---------+---------+---------+---------+---------+---------+---------+---------+---------+---------+---------|
contig054630 ACTATTGGTGTTGTTGTGGCTGTGTTTCTGATGTGTTTCTGTCCTTACTTTTATCCCTCTCTCGCAGGGCAGGACACATCAACCAGTGTGGAATTTTCAGTTTTTGGGGTCTGGTTACTTTATTGTAATT
 BurTARs.A023 ACTATTGGTGTTGTTGTGGCTGTGTTTCTGATGTGTTTCTGTCCTTACTTTTATCCCTCTCTCGCAGGGCAGGACACATCAACCAGTGTGGAATTTTCAGTTTTTGGGGTCTGGTTACTTTATTGTAATT
    Consensus ACTATTGGTGTTGTTGTGGCTGTGTTTCTGATGTGTTTCTGTCCTTACTTTTATCCCTCTCTCGCAGGGCAGGACACATCAACCAGTGTGGAATTTTCAGTTTTTGGGGTCTGGTTACTTTATTGTAATT

             1053110540     10550     10560     10570     10580     10590     10600     10610     10620     10630     10640     10650     10660
             |--------+---------+---------+---------+---------+---------+---------+---------+---------+---------+---------+---------+---------|
contig054630 CCTGTCTAAATCCACTTATCTATGCGTTTTTCTATCCCTGGTTTAGGAAGACTGTTAAACTGATAGTTACATTGCAGATTTTGCAGCCTGACTCTTGTGATGCCAACATACTGTAGAATTCAAATCCTAT
 BurTARs.A023 CCTGTCTAAATCCACTTATCTATGCGTTTTTCTATCCCTGGTTTAGGAAGACTGTTAAACTGATAGTTACATTGCAGATTTTGCAGCCTGACTCTTGTGATGCCAACATACTGTAG
    Consensus CCTGTCTAAATCCACTTATCTATGCGTTTTTCTATCCCTGGTTTAGGAAGACTGTTAAACTGATAGTTACATTGCAGATTTTGCAGCCTGACTCTTGTGATGCCAACATACTGTAG..............

             1066110670     10680     10690     10700     10710     10720     10730     10740     10750     10760     10770     10780     10790
```

```
            4681  4690      4700      4710      4720      4730      4740      4750      4760      4770      4780      4790      4800      4810
            |--------+---------+---------+---------+---------+---------+---------+---------+---------+---------+---------+---------+---------|
contig049540 TGTTCATAATGCTGCAACCAGAGCTGCAGTTCTTCACTAGTCTGTGACGATGGGCACACAGGAAAAATCTGAGCTCTGCTTTCCACAACTCTTCAACATGTCCTGCAAAAAGCCTGCCATTGCTCGGTCC
BurTARs.A024                                                   ATGGGCACACAGGAAAAATCTGAGCTCTGCTTTCCACAACTCTTCAACATGTCCTGCAAAAAGCCTGCCATTGCTCGGTCC
   Consensus ..................................................ATGGGCACACAGGAAAAATCTGAGCTCTGCTTTCCACAACTCTTCAACATGTCCTGCAAAAAGCCTGCCATTGCTCGGTCC

            4811  4820      4830      4840      4850      4860      4870      4880      4890      4900      4910      4920      4930      4940
            |--------+---------+---------+---------+---------+---------+---------+---------+---------+---------+---------+---------+---------|
contig049540 AAAGCTGTGTTCCTTCATATTGTATTGTCCTCAGTTTCTTTTCTGACGGTGGCTCTCAACTTGCTTGTCATCATCTCAGTCTCCCACTTCAGGCAGAAACAACGTTTTTGTTTTACTAGCTACACTATTC
BurTARs.A024 AAAGCTGTGTTCCTTCATATTGTATTGTCCTCAGTTTCTTTTCTGACGGTGGCTCTCAACTTGCTTGTCATCATCTCAGTCTCCCACTTCAGGCAG----------------------------------
   Consensus AAAGCTGTGTTCCTTCATATTGTATTGTCCTCAGTTTCTTTTCTGACGGTGGCTCTCAACTTGCTTGTCATCATCTCAGTCTCCCACTTCAGGCAG..................................

            4941  4950      4960      4970      4980      4990      5000      5010      5020      5030      5040      5050      5060      5070
            |--------+---------+---------+---------+---------+---------+---------+---------+---------+---------+---------+---------+---------|
contig049540 TAGTGTTTCAAGTATTTGAGCTAGTAATACAAGATTCTTCATATGTACAGATTTATACTTGAGAATATTTACATTTCATCATTTTAACTTTGGCACCAGAAAACATACTATACTCATTGTCTTATAAGTA
BurTARs.A024 ----------------------------------------------------------------------------------------------------------------------------------
   Consensus ..................................................................................................................................

            5071  5080      5090      5100      5110      5120      5130      5140      5150      5160      5170      5180      5190      5200
            |--------+---------+---------+---------+---------+---------+---------+---------+---------+---------+---------+---------+---------|
contig049540 ATACTATGTTCTGAATCAGTTTGACGTTGATAATATGCTGCTGTGTGACACTTAATAGCTCTTTGGCTTGTTTCATTATAGAGGTTTGAGGAATTGTTATTTTTCTGTCTCCTTGCAGGCAGCTTCACAC
BurTARs.A024 --------------------------------------------------------------------------------------------------------------------------CTTCACAC
   Consensus ..........................................................................................................................CTTCACAC

            5201  5210      5220      5230      5240      5250      5260      5270      5280      5290      5300      5310      5320      5330
            |--------+---------+---------+---------+---------+---------+---------+---------+---------+---------+---------+---------+---------|
contig049540 ACCCACTAACATCCTGCTCCTCTCTCTGGCTGTCTCAGACTTTCTTGTTGGCCTCCTATTGTCACCTGCAGAAATCCTCCGAAGTACAGCCTGTTGGTTTCTTGGTCAGCCCACATGTTTAATGTATATT
BurTARs.A024 ACCCACTAACATCCTGCTCCTCTCTCTGGCTGTCTCAGACTTTCTTGTTGGCCTCCTATTGTCACCTGCAGAAATCCTCCGAAGTACAGCCTGTTGGTTTCTTGGTCAGCCCACATGTTTAATGTATATT
   Consensus ACCCACTAACATCCTGCTCCTCTCTCTGGCTGTCTCAGACTTTCTTGTTGGCCTCCTATTGTCACCTGCAGAAATCCTCCGAAGTACAGCCTGTTGGTTTCTTGGTCAGCCCACATGTTTAATGTATATT

            5331  5340      5350      5360      5370      5380      5390      5400      5410      5420      5430      5440      5450      5460
            |--------+---------+---------+---------+---------+---------+---------+---------+---------+---------+---------+---------+---------|
contig049540 TTTGTGTCCTTGACTGTTACCTCTGCCTCAGTGGGCATTATGGTGCTGATATCAGCTGACCGCTATGTAGCTATTTGTGACCCTTTGAATTACCCCATCAGGATCACTGACAGAAGAGTGAAACTCTGTG
BurTARs.A024 TTTGTGTCCTTGACTGTTACCTCTGCCTCAGTGGGCATTATGGTGCTGATATCAGCTGACCGCTATGTAGCTATTTGTGACCCTTTGAATTACCCCATCAGGATCACTGACAGAAGAGTGAAACTCTGTG
   Consensus TTTGTGTCCTTGACTGTTACCTCTGCCTCAGTGGGCATTATGGTGCTGATATCAGCTGACCGCTATGTAGCTATTTGTGACCCTTTGAATTACCCCATCAGGATCACTGACAGAAGAGTGAAACTCTGTG

            5461  5470      5480      5490      5500      5510      5520      5530      5540      5550      5560      5570      5580      5590
            |--------+---------+---------+---------+---------+---------+---------+---------+---------+---------+---------+---------+---------|
contig049540 TTTGTCTGTGTTGGCTCTGCTCCATCGCCTTCAGCTGTTTCCTTGTAAGAGATGACCTGCATCAAAAAAGAAAACAAAATTCCTGCTATGGAAAATGTGTCGTTGTTGTTCAATACATTGCAGGAGTTGT
BurTARs.A024 TTTGTCTGTGTTGGCTCTGCTCCATCGCCTTCAGCTGTTTCCTTGTAAGAGATGACCTGCATCAAAAAAGAAAACAAAATTCCTGCTATGGAAAATGTGTCGTTGTTGTTCAATACATTGCAGGAGTTGT
   Consensus TTTGTCTGTGTTGGCTCTGCTCCATCGCCTTCAGCTGTTTCCTTGTAAGAGATGACCTGCATCAAAAAAGAAAACAAAATTCCTGCTATGGAAAATGTGTCGTTGTTGTTCAATACATTGCAGGAGTTGT

            5591  5600      5610      5620      5630      5640      5650      5660      5670      5680      5690      5700      5710      5720
            |--------+---------+---------+---------+---------+---------+---------+---------+---------+---------+---------+---------+---------|
contig049540 TGACCTCATTTTAACCTTCATTGTACCAGTTACTGTCATCATAGTTCTGTATATGAGAGTATTTGTGGTGGCTGTGTCTCAGGCCCGCTCTATGCGCTCTCATGCTACAGCTGTCAGAAATCACCTTCCA
BurTARs.A024 TGACCTCATTTTAACCTTCATTGTACCAGTTACTGTCATCATAGTTCTGTATATGAGAGTATTTGTGGTGGCTGTGTCTCAGGCCCGCTCTATGCGCTCTCATGCTACAGCTGTCAGAAATCACCTTCCA
   Consensus TGACCTCATTTTAACCTTCATTGTACCAGTTACTGTCATCATAGTTCTGTATATGAGAGTATTTGTGGTGGCTGTGTCTCAGGCCCGCTCTATGCGCTCTCATGCTACAGCTGTCAGAAATCACCTTCCA

            5721  5730      5740      5750      5760      5770      5780      5790      5800      5810      5820      5830      5840      5850
            |--------+---------+---------+---------+---------+---------+---------+---------+---------+---------+---------+---------+---------|
contig049540 ATGACGCTAACAAAAAAGTCTGAACTAAAAGCAGCCAGGACTCTGGGTGTTCTTGTTCTTGTGTTTCTAATGTGTTTCTGCCCTTATTACTGTGTTTCTCTTGTAGGAGAAGAATTCATCAATAGTTCAT
BurTARs.A024 ATGACGCTAACAAAAAAGTCTGAACTAAAAGCAGCCAGGACTCTGGGTGTTCTTGTTCTTGTGTTTCTAATGTGTTTCTGCCCTTATTACTGTGTTTCTCTTGTAGGAGAAGAATTCATCAATAGTTCAT
   Consensus ATGACGCTAACAAAAAAGTCTGAACTAAAAGCAGCCAGGACTCTGGGTGTTCTTGTTCTTGTGTTTCTAATGTGTTTCTGCCCTTATTACTGTGTTTCTCTTGTAGGAGAAGAATTCATCAATAGTTCAT

            5851  5860      5870      5880      5890      5900      5910      5920      5930      5940      5950      5960      5970      5980
            |--------+---------+---------+---------+---------+---------+---------+---------+---------+---------+---------+---------+---------|
contig049540 CTGCTTCTTTTGTGGCCTATCTGTTTGGTTTAAACTCATGTCTAAACCCTTTGATCTATGCTATGTTCTACCCCTGGTTTAGAAAAGCTGTGAAACTAGTTGTCACTCTACAGATACTGCAGCCTGGCTC
BurTARs.A024 CTGCTTCTTTTGTGGCCTATCTGTTTGGTTTAAACTCATGTCTAAACCCTTTGATCTATGCTATGTTCTACCCCTGGTTTAGAAAAGCTGTGAAACTAGTTGTCACTCTACAGATACTGCAGCCTGGCTC
   Consensus CTGCTTCTTTTGTGGCCTATCTGTTTGGTTTAAACTCATGTCTAAACCCTTTGATCTATGCTATGTTCTACCCCTGGTTTAGAAAAGCTGTGAAACTAGTTGTCACTCTACAGATACTGCAGCCTGGCTC

            5981  5990      6000      6010      6020      6030      6040      6050      6060      6070      6080      6090      6100      6110
            |--------+---------+---------+---------+---------+---------+---------+---------+---------+---------+---------+---------+---------|
contig049540 CAGTGAGGTAAGTATTTTGTAAACAGATCAAAACATGTCAAAAATAGTTGTTTTCAGAAAAAAATAGTTGTTTTCAGCTTCAGCTCGTAATAAAAAAATCTTAATCTTGTTGACTTCATTTTATAACAATA
BurTARs.A024 CAGTGAGGTAAGTATTTTGTAA
   Consensus CAGTGAGGTAAGTATTTTGTAA....................................................................................................
```

```
             2731 2740      2750      2760      2770      2780      2790      2800      2810      2820      2830      2840      2850      2860
             |--------+---------+---------+---------+---------+---------+---------+---------+---------+---------+---------+---------+---------|
contig049534 AAAGAGGTAATGAGGAGGAGGGAAAGCAGAAAGAGATAAATTAGTTCTATGAGAAGTCTTGGTCAAAGAGCGGTGGGAGGCGTAGCTGTTTGGTGATGGAGAAAGGAGTCGAGCTCTGCTTTCCACAACT
BurTARs.A025                                                                                                ATGGAGAAAGGAGTCGAGCTCTGCTTTCCACAACT
   Consensus ...............................................................................................ATGGAGAAAGGAGTCGAGCTCTGCTTTCCACAACT

             2861 2870      2880      2890      2900      2910      2920      2930      2940      2950      2960      2970      2980      2990
             |--------+---------+---------+---------+---------+---------+---------+---------+---------+---------+---------+---------+---------|
contig049534 CCTCAACAATTCCTGCATCAAGCCGACACTTCACTGGTCCAAAGCCGTGCTCCTGAACATTGTGCTGTCGTGCATCTCTTTGATCACTGCTGGTCTCAATCTTCTTGTTATCATCTCAGTCTCCCATTTC
BurTARs.A025 CCTCAACAATTCCTGCATCAAGCCGACACTTCACTGGTCCAAAGCCGTGCTCCTGAACATTGTGCTGTCGTGCATCTCTTTGATCACTGCTGGTCTCAATCTTCTTGTTATCATCTCAGTCTCCCATTTC
   Consensus CCTCAACAATTCCTGCATCAAGCCGACACTTCACTGGTCCAAAGCCGTGCTCCTGAACATTGTGCTGTCGTGCATCTCTTTGATCACTGCTGGTCTCAATCTTCTTGTTATCATCTCAGTCTCCCATTTC

             2991 3000      3010      3020      3030      3040      3050      3060      3070      3080      3090      3100      3110      3120
             |--------+---------+---------+---------+---------+---------+---------+---------+---------+---------+---------+---------+---------|
contig049534 AGGCAGAGATTGACTTCAACTTTAGCTTAAATTTTATAAACTTTGAGAGTATGATTTATGTCATATGATTTGTTGATGGACGAATGAAGTGAATCTGACATATTCTCCATGTATTTTAACCAAACTGTAT
BurTARs.A025 AGGCAG----------------------------------------------------------------------------------------------------------------------------
   Consensus AGGCAG............................................................................................................................

             3121 3130      3140      3150      3160      3170      3180      3190      3200      3210      3220      3230      3240      3250
             |--------+---------+---------+---------+---------+---------+---------+---------+---------+---------+---------+---------+---------|
contig049534 TGTGTAATTCATTATCAATGAATCATTTTCTTACTTTGACACTTTGAAAAGATTAATGATTATTATTTTCTCTTCTGTGTCCCTGCAGGCAGCTGCACACACCCAGTAACATCCTCCTCCTCTCTCTGGC
BurTARs.A025 --------------------------------------------------------------------------------------------CTGCACACACCCAGTAACATCCTCCTCCTCTCTCTGGC
   Consensus ............................................................................................CTGCACACACCCAGTAACATCCTCCTCCTCTCTCTGGC

             3251 3260      3270      3280      3290      3300      3310      3320      3330      3340      3350      3360      3370      3380
             |--------+---------+---------+---------+---------+---------+---------+---------+---------+---------+---------+---------+---------|
contig049534 TGTGTCAGACTTTCTTGTTGGTCTCCTGTTAATTCCAGCTGAGATTTTTAGAATCACAGTCTGCTGGGTATTTGGTGAATCCATGTGTTCACTTTATACATATCTAGGCTACATTGTTGTAACATCTTCA
BurTARs.A025 TGTGTCAGACTTTCTTGTTGGTCTCCTGTTAATTCCAGCTGAGATTTTTAGAATCACAGTCTGCTGGGTATTTGGTGAATCCATGTGTTCACTTTATACATATCTAGGCTACATTGTTGTAACATCTTCA
   Consensus TGTGTCAGACTTTCTTGTTGGTCTCCTGTTAATTCCAGCTGAGATTTTTAGAATCACAGTCTGCTGGGTATTTGGTGAATCCATGTGTTCACTTTATACATATCTAGGCTACATTGTTGTAACATCTTCA

             3381 3390      3400      3410      3420      3430      3440      3450      3460      3470      3480      3490      3500      3510
             |--------+---------+---------+---------+---------+---------+---------+---------+---------+---------+---------+---------+---------|
contig049534 ATAAGTAACATAGTTCTCATATCAGTTGATCGCTATGTGGCAATTTGTGATCCTCTGCATTACCCCAGTAGAATTTCTGTGGCAAAAATCAGACTTAGTGTTTGTATGTGTTGGTTTTATTCGGCTTTCT
BurTARs.A025 ATAAGTAACATAGTTCTCATATCAGTTGATCGCTATGTGGCAATTTGTGATCCTCTGCATTACCCCAGTAGAATTTCTGTGGCAAAAATCAGACTTAGTGTTTGTATGTGTTGGTTTTATTCGGCTTTCT
   Consensus ATAAGTAACATAGTTCTCATATCAGTTGATCGCTATGTGGCAATTTGTGATCCTCTGCATTACCCCAGTAGAATTTCTGTGGCAAAAATCAGACTTAGTGTTTGTATGTGTTGGTTTTATTCGGCTTTCT

             3511 3520      3530      3540      3550      3560      3570      3580      3590      3600      3610      3620      3630      3640
             |--------+---------+---------+---------+---------+---------+---------+---------+---------+---------+---------+---------+---------|
contig049534 ACAGCACTCTTTGTACAAAGAATATCCTGATTGAACCAGGCAGGTATAATTCCTGTTATGGAGAGTGTGTCTTTATCACCAGTGATATTGCTGGCATAATTGACCTTGTTTTATCTTTTATCGTACCAGT
BurTARs.A025 ACAGCACTCTTTGTACAAAGAATATCCTGATTGAACCAGGCAGGTATAATTCCTGTTATGGAGAGTGTGTCTTTATCACCAGTGATATTGCTGGCATAATTGACCTTGTTTTATCTTTTATCGTACCAGT
   Consensus ACAGCACTCTTTGTACAAAGAATATCCTGATTGAACCAGGCAGGTATAATTCCTGTTATGGAGAGTGTGTCTTTATCACCAGTGATATTGCTGGCATAATTGACCTTGTTTTATCTTTTATCGTACCAGT

             3641 3650      3660      3670      3680      3690      3700      3710      3720      3730      3740      3750      3760      3770
             |--------+---------+---------+---------+---------+---------+---------+---------+---------+---------+---------+---------+---------|
contig049534 TTCCATCATCATAGTTCTATATATGAGAGTATTTGTGGTGGCTGTGTCTCAGGCTCGTGCCATGCACTCTCATGTTACAGCTACACTTCAGCGTTCACTGAATCAAACAAACAAATCTGAGCTGAAAGCA
BurTARs.A025 TTCCATCATCATAGTTCTATATATGAGAGTATTTGTGGTGGCTGTGTCTCAGGCTCGTGCCATGCACTCTCATGTTACAGCTACACTTCAGCGTTCACTGAATCAAACAAACAAATCTGAGCTGAAAGCA
   Consensus TTCCATCATCATAGTTCTATATATGAGAGTATTTGTGGTGGCTGTGTCTCAGGCTCGTGCCATGCACTCTCATGTTACAGCTACACTTCAGCGTTCACTGAATCAAACAAACAAATCTGAGCTGAAAGCA

             3771 3780      3790      3800      3810      3820      3830      3840      3850      3860      3870      3880      3890      3900
             |--------+---------+---------+---------+---------+---------+---------+---------+---------+---------+---------+---------+---------|
contig049534 GCCAGGACCCTGGGGGTTCTTGTAGTTGTGTTTCTGGCCAGTTTCTGTCCATTTTATTGTTACTTTCTTGTTGTAGAAGACATAGTCAGTGATTCATCTGCCTCTATTGTGGTCATCGTCTATTATTTTA
BurTARs.A025 GCCAGGACCCTGGGGGTTCTTGTAGTTGTGTTTCTGGCCAGTTTCTGTCCATTTTATTGTTACTTTCTTGTTGTAGAAGACATAGTCAGTGATTCATCTGCCTCTATTGTGGTCATCGTCTATTATTTTA
   Consensus GCCAGGACCCTGGGGGTTCTTGTAGTTGTGTTTCTGGCCAGTTTCTGTCCATTTTATTGTTACTTTCTTGTTGTAGAAGACATAGTCAGTGATTCATCTGCCTCTATTGTGGTCATCGTCTATTATTTTA

             3901 3910      3920      3930      3940      3950      3960      3970      3980      3990      4000      4010      4020      4030
             |--------+---------+---------+---------+---------+---------+---------+---------+---------+---------+---------+---------+---------|
contig049534 ACTCTTGTCTAAACCCACTAATTTATGCCCTGTTTTACCCTTGGTTTAGAAATGCTGTTAAAGTCATCATCACTTTTCAGATATTCAAGCGTGACAGCAGTGAGGCCAACGTAATATAGAAATACAATGA
BurTARs.A025 ACTCTTGTCTAAACCCACTAATTTATGCCCTGTTTTACCCTTGGTTTAGAAATGCTGTTAAAGTCATCATCACTTTTCAGATATTCAAGCGTGACAGCAGTGAGGCCAACGTAATATAG
   Consensus ACTCTTGTCTAAACCCACTAATTTATGCCCTGTTTTACCCTTGGTTTAGAAATGCTGTTAAAGTCATCATCACTTTTCAGATATTCAAGCGTGACAGCAGTGAGGCCAACGTAATATAG...........
```

```
           3641 3650      3660      3670      3680      3690      3700      3710      3720      3730      3740      3750      3760      3770
           |---------+---------+---------+---------+---------+---------+---------+---------+---------+---------+---------+---------+---------|
contig041024 GTGAGCTGCAGCTGTCTGATGATGGAGATTCAGAAAGCAGCTGAGCTTTGTTTTCCACAACTCCTCAACAGTTCCTGCAGGAAGCCAACACTTCATTGGTCCAAAGCTGTGCTCCTGAACATTGTGCTGT
BurTARs.A026                      ATGGAGATTCAGAAAGCAGCTGAGCTTTGTTTTCCACAACTCCTCAACAGTTCCTGCAGGAAGCCAACACTTCATTGGTCCAAAGCTGTGCTCCTGAACATTGTGCTGT
   Consensus .....................ATGGAGATTCAGAAAGCAGCTGAGCTTTGTTTTCCACAACTCCTCAACAGTTCCTGCAGGAAGCCAACACTTCATTGGTCCAAAGCTGTGCTCCTGAACATTGTGCTGT

           3771 3780      3790      3800      3810      3820      3830      3840      3850      3860      3870      3880      3890      3900
           |---------+---------+---------+---------+---------+---------+---------+---------+---------+---------+---------+---------+---------|
contig041024 CCTGCATCTCTCTGATCACTGCTGCTCTAAACCTCCTGGTCATCATCTCAGTCTCCCACTTCAGGCAGAGATACATTTCTCTGCTGAAACTATACTTTAAAAATTAGAAACTTAATTTTCAGAAAATTAT
BurTARs.A026 CCTGCATCTCTCTGATCACTGCTGCTCTAAACCTCCTGGTCATCATCTCAGTCTCCCACTTCAGG-----------------------------------------------------------------
   Consensus CCTGCATCTCTCTGATCACTGCTGCTCTAAACCTCCTGGTCATCATCTCAGTCTCCCACTTCAGG.................................................................

           3901 3910      3920      3930      3940      3950      3960      3970      3980      3990      4000      4010      4020      4030
           |---------+---------+---------+---------+---------+---------+---------+---------+---------+---------+---------+---------+---------|
contig041024 GATTTATGGCATTTGATTTTATGATTAAGATTGAAATGATTGAAGTGGTGTATTTTAACCAGCTATTGTTTTATATACCTAGACTGAATCTTACTACATACTTTAACTAATGAAAATCTAATTTAAGCCA
BurTARs.A026 ----------------------------------------------------------------------------------------------------------------------------------
   Consensus ..................................................................................................................................

           4031 4040      4050      4060      4070      4080      4090      4100      4110      4120      4130      4140      4150      4160
           |---------+---------+---------+---------+---------+---------+---------+---------+---------+---------+---------+---------+---------|
contig041024 TTTAAAAAAATATTAATGTCCATTATATTCTTTTCTGTTTCCCTGCAGGAAGCTTCATACACCCAGTAACATCCTGCTCCTCTCTCTGGCCGTCTCAGACTTTCTTGTGGGTCTTCTGCTGATGCCATTA
BurTARs.A026 -------------------------------------------------AAGCTTCATACACCCAGTAACATCCTGCTCCTCTCTCTGGCCGTCTCAGACTTTCTTGTGGGTCTTCTGCTGATGCCATTA
   Consensus .................................................AAGCTTCATACACCCAGTAACATCCTGCTCCTCTCTCTGGCCGTCTCAGACTTTCTTGTGGGTCTTCTGCTGATGCCATTA

           4161 4170      4180      4190      4200      4210      4220      4230      4240      4250      4260      4270      4280      4290
           |---------+---------+---------+---------+---------+---------+---------+---------+---------+---------+---------+---------+---------|
contig041024 GAGATCTTTAGAAACACAGCTTGCTGGGTACTTGGTGATGTAATGTGTTCTGTTTATTGGTACCTGACCAACAACATTACCTGTGCTTCAATAGGGAACATAGTTCTAATATCAGTTGACCGTTATGTGG
BurTARs.A026 GAGATCTTTAGAAACACAGCTTGCTGGGTACTTGGTGATGTAATGTGTTCTGTTTATTGGTACCTGACCAACAACATTACCTGTGCTTCAATAGGGAACATAGTTCTAATATCAGTTGACCGTTATGTGG
   Consensus GAGATCTTTAGAAACACAGCTTGCTGGGTACTTGGTGATGTAATGTGTTCTGTTTATTGGTACCTGACCAACAACATTACCTGTGCTTCAATAGGGAACATAGTTCTAATATCAGTTGACCGTTATGTGG

           4291 4300      4310      4320      4330      4340      4350      4360      4370      4380      4390      4400      4410      4420
           |---------+---------+---------+---------+---------+---------+---------+---------+---------+---------+---------+---------+---------|
contig041024 CTATTTGTGACCCTCTGCATTACCCCACTAGAATTACTTTGGCGAAAGTCAAACTCAGTGTTTGTCTGTGTTGGTTTTTTTCTATTTTCTACTGGAGTCTTTATTTGAAAGATATCCTAGTTGAACCAGG
BurTARs.A026 CTATTTGTGACCCTCTGCATTACCCCACTAGAATTACTTTGGCGAAAGTCAAACTCAGTGTTTGTCTGTGTTGGTTTTTTTCTATTTTCTACTGGAGTCTTTATTTGAAAGATATCCTAGTTGAACCAGG
   Consensus CTATTTGTGACCCTCTGCATTACCCCACTAGAATTACTTTGGCGAAAGTCAAACTCAGTGTTTGTCTGTGTTGGTTTTTTTCTATTTTCTACTGGAGTCTTTATTTGAAAGATATCCTAGTTGAACCAGG

           4421 4430      4440      4450      4460      4470      4480      4490      4500      4510      4520      4530      4540      4550
           |---------+---------+---------+---------+---------+---------+---------+---------+---------+---------+---------+---------+---------|
contig041024 CAGGTATAATTCCTGCTATGGAGAGTGTGTGCTTATTATTAATGACATTGCAGGGATTGTTGACCTTGCTTTGTCCTTTATAGTTCCAGTTACTGTTATCATAGTCTTGTATATGAGAGTATTTGTGGTG
BurTARs.A026 CAGGTATAATTCCTGCTATGGAGAGTGTGTGCTTATTATTAATGACATTGCAGGGATTGTTGACCTTGCTTTGTCCTTTATAGTTCCAGTTACTGTTATCATAGTCTTGTATATGAGAGTATTTGTGGTG
   Consensus CAGGTATAATTCCTGCTATGGAGAGTGTGTGCTTATTATTAATGACATTGCAGGGATTGTTGACCTTGCTTTGTCCTTTATAGTTCCAGTTACTGTTATCATAGTCTTGTATATGAGAGTATTTGTGGTG

           4551 4560      4570      4580      4590      4600      4610      4620      4630      4640      4650      4660      4670      4680
           |---------+---------+---------+---------+---------+---------+---------+---------+---------+---------+---------+---------+---------|
contig041024 GCTGTGTCTCAGGCTCGTGCCATGCGCTCTCATGTTACAGCTGTCACACTTCAGTGTTCACTGAATCAAGCAAACAAATCTGAGCTGAAAGCAGCCAGGACTCTGGGGGTTCTTGTAGTTGTATTTTTGG
BurTARs.A026 GCTGTGTCTCAGGCTCGTGCCATGCGCTCTCATGTTACAGCTGTCACACTTCAGTGTTCACTGAATCAAGCAAACAAATCTGAGCTGAAAGCAGCCAGGACTCTGGGGGTTCTTGTAGTTGTATTTTTGG
   Consensus GCTGTGTCTCAGGCTCGTGCCATGCGCTCTCATGTTACAGCTGTCACACTTCAGTGTTCACTGAATCAAGCAAACAAATCTGAGCTGAAAGCAGCCAGGACTCTGGGGGTTCTTGTAGTTGTATTTTTGG

           4681 4690      4700      4710      4720      4730      4740      4750      4760      4770      4780      4790      4800      4810
           |---------+---------+---------+---------+---------+---------+---------+---------+---------+---------+---------+---------+---------|
contig041024 CATGCTACTGCCCATTTTACTGCTACTCTCTGGTTGATAAAAAATGTGGTCAATGATTCATCTGCATCTTTTGTGGTTCTTGTCTTTTATTTTAACTCTTGTTTAAACCCACTGATCTATGCTCTGTTTTA
BurTARs.A026 CATGCTACTGCCCATTTTACTGCTACTCTCTGGTTGATAAAAAATGTGGTCAATGATTCATCTGCATCTTTTGTGGTTCTTGTCTTTTATTTTAACTCTTGTTTAAACCCACTGATCTATGCTCTGTTTTA
   Consensus CATGCTACTGCCCATTTTACTGCTACTCTCTGGTTGATAAAAAATGTGGTCAATGATTCATCTGCATCTTTTGTGGTTCTTGTCTTTTATTTTAACTCTTGTTTAAACCCACTGATCTATGCTCTGTTTTA

           4811 4820      4830      4840      4850      4860      4870      4880      4890      4900      4910      4920      4930      4940
           |---------+---------+---------+---------+---------+---------+---------+---------+---------+---------+---------+---------+---------|
contig041024 CCCCTGGTTTAGAAAAGCTATTAGATGTGTTATCACTCTGCTGATATTTAAGCATGACAGCAGTGAGGTCAACGTAATATAGAAATACAATGAAAGCTGCTCCACTAAGATTTTAAACCAGTAAATTGAT
BurTARs.A026 CCCCTGGTTTAGAAAAGCTATTAGATGTGTTATCACTCTGCTGATATTTAAGCATGACAGCAGTGAGGTCAACGTAATATAG
   Consensus CCCCTGGTTTAGAAAAGCTATTAGATGTGTTATCACTCTGCTGATATTTAAGCATGACAGCAGTGAGGTCAACGTAATATAG................................................
```

```
      Consensus ..................................................................................................................................

                 5331 5340      5350      5360      5370      5380      5390      5400      5410      5420      5430      5440      5450      5460
                 |--------+---------+---------+---------+---------+---------+---------+---------+---------+---------+---------+---------+---------|
    contig034854 AGGAGTTTAAAGAAGGTGTGGGGATATTACTAATTCTGGTCACAATGCTGCAGCTCTCTGCAGGTCTGTGAAGATGGATACCCAGGGCGGAGCAGAGCTCTGCTTTCCACAACTCTTCAACACCTCCTGC
     BurTARs.A027                                                                          ATGGATACCCAGGGCGGAGCAGAGCTCTGCTTTCCACAACTCTTCAACACCTCCTGC
       Consensus .........................................................................ATGGATACCCAGGGCGGAGCAGAGCTCTGCTTTCCACAACTCTTCAACACCTCCTGC

                 5461 5470      5480      5490      5500      5510      5520      5530      5540      5550      5560      5570      5580      5590
                 |--------+---------+---------+---------+---------+---------+---------+---------+---------+---------+---------+---------+---------|
    contig034854 AAGAAGCCTACAACTCCTCTATCACAAGTTTTGCTCTTTCACATTCTGATGCCATCAATGTCTCTGCTAACTGTGACTCTCAACCTGCTTGTCATCATTGCAATCTCCCACTTCAGGCAGAGTATCACTT
     BurTARs.A027 AAGAAGCCTACAACTCCTCTATCACAAGTTTTGCTCTTTCACATTCTGATGCCATCAATGTCTCTGCTAACTGTGACTCTCAACCTGCTTGTCATCATTGCAATCTCCCACTTCAGGCAG----------
       Consensus AAGAAGCCTACAACTCCTCTATCACAAGTTTTGCTCTTTCACATTCTGATGCCATCAATGTCTCTGCTAACTGTGACTCTCAACCTGCTTGTCATCATTGCAATCTCCCACTTCAGGCAG..........

                 5591 5600      5610      5620      5630      5640      5650      5660      5670      5680      5690      5700      5710      5720
                 |--------+---------+---------+---------+---------+---------+---------+---------+---------+---------+---------+---------+---------|
    contig034854 TTTTTACCTGCTGAGTCTATATACCTGATTTGTAATACCTGTTTGAATAGTTTCTTTCTGTTGATTTTTTTAAATTATATTTTCCTGTCTTTCTAGGCAGCTCCACTCACACACTAACATCCTGCTCCTC
     BurTARs.A027 ----------------------------------------------------------------------------------------------------CTCCACTCACACACTAACATCCTGCTCCTC
       Consensus ....................................................................................................CTCCACTCACACACTAACATCCTGCTCCTC

                 5721 5730      5740      5750      5760      5770      5780      5790      5800      5810      5820      5830      5840      5850
                 |--------+---------+---------+---------+---------+---------+---------+---------+---------+---------+---------+---------+---------|
    contig034854 TCTCTGGCTGCCGCTGACTTTCTCAATGGCCTTCTGTTTATGCCTGGAGAAATCCTGCGAAATACAGCGTGTTGGTTTCTTGGTCAGCTCACATGTTCATTGTATAATTATGTATCCTACATCATTGCCT
     BurTARs.A027 TCTCTGGCTGCCGCTGACTTTCTCAATGGCCTTCTGTTTATGCCTGGAGAAATCCTGCGAAATACAGCGTGTTGGTTTCTTGGTCAGCTCACATGTTCATTGTATAATTATGTATCCTACATCATTGCCT
       Consensus TCTCTGGCTGCCGCTGACTTTCTCAATGGCCTTCTGTTTATGCCTGGAGAAATCCTGCGAAATACAGCGTGTTGGTTTCTTGGTCAGCTCACATGTTCATTGTATAATTATGTATCCTACATCATTGCCT

                 5851 5860      5870      5880      5890      5900      5910      5920      5930      5940      5950      5960      5970      5980
                 |--------+---------+---------+---------+---------+---------+---------+---------+---------+---------+---------+---------+---------|
    contig034854 CTGCCTCAGTGGGCAACATGGTGCTGATATCAGTCGACCGCTATGTGGCTATTTGTGACCCTCTGCATTACCCCACCAGAATCACAGAGGGAAAAGTGAAACTTTGTGTTTGTCTGTGTTGGCTCTGCTC
     BurTARs.A027 CTGCCTCAGTGGGCAACATGGTGCTGATATCAGTCGACCGCTATGTGGCTATTTGTGACCCTCTGCATTACCCCACCAGAATCACAGAGGGAAAAGTGAAACTTTGTGTTTGTCTGTGTTGGCTCTGCTC
       Consensus CTGCCTCAGTGGGCAACATGGTGCTGATATCAGTCGACCGCTATGTGGCTATTTGTGACCCTCTGCATTACCCCACCAGAATCACAGAGGGAAAAGTGAAACTTTGTGTTTGTCTGTGTTGGCTCTGCTC

                 5981 5990      6000      6010      6020      6030      6040      6050      6060      6070      6080      6090      6100      6110
                 |--------+---------+---------+---------+---------+---------+---------+---------+---------+---------+---------+---------+---------|
    contig034854 TGTTTTGTACAGCTATGTGATTTTAATAGATGATCTAAGTCAACCAGGCAAGCATAAATCTTGCTATGGAAAATGTATAATTTTCATTGAATTTATTGCAGGATTTGTTGACCTTTTTATATCCTTTATT
     BurTARs.A027 TGTTTTGTACAGCTATGTGATTTTAATAGATGATCTAAGTCAACCAGGCAAGCATAAATCTTGCTATGGAAAATGTATAATTTTCATTGAATTTATTGCAGGATTTGTTGACCTTTTTATATCCTTTATT
       Consensus TGTTTTGTACAGCTATGTGATTTTAATAGATGATCTAAGTCAACCAGGCAAGCATAAATCTTGCTATGGAAAATGTATAATTTTCATTGAATTTATTGCAGGATTTGTTGACCTTTTTATATCCTTTATT

                 6111 6120      6130      6140      6150      6160      6170      6180      6190      6200      6210      6220      6230      6240
                 |--------+---------+---------+---------+---------+---------+---------+---------+---------+---------+---------+---------+---------|
    contig034854 ATTCCACTTACTGTCATCATTGTTCTGTATATGAGAGTGTTTGTGGTGGCTGTGTCTCAGGCCCGTGCCATGCGCTCTCAGGTTACAACTGTCACACTGCAGCTCTCAGTGACTCTAACAGCAACTAAAT
     BurTARs.A027 ATTCCACTTACTGTCATCATTGTTCTGTATATGAGAGTGTTTGTGGTGGCTGTGTCTCAGGCCCGTGCCATGCGCTCTCAGGTTACAACTGTCACACTGCAGCTCTCAGTGACTCTAACAGCAACTAAAT
       Consensus ATTCCACTTACTGTCATCATTGTTCTGTATATGAGAGTGTTTGTGGTGGCTGTGTCTCAGGCCCGTGCCATGCGCTCTCAGGTTACAACTGTCACACTGCAGCTCTCAGTGACTCTAACAGCAACTAAAT

                 6241 6250      6260      6270      6280      6290      6300      6310      6320      6330      6340      6350      6360      6370
                 |--------+---------+---------+---------+---------+---------+---------+---------+---------+---------+---------+---------+---------|
    contig034854 CAGAGTTAAAAGCAGCCAGGACACTGGGTGTTCTTGTTCTTGTGTTTCTATTATGTTTCTGCCCATATTACATTGTTTCACTTTTTGGAGACGAGGTTCTCAATAGTGCCTCTGAATCTATTGCATTCTA
     BurTARs.A027 CAGAGTTAAAAGCAGCCAGGACACTGGGTGTTCTTGTTCTTGTGTTTCTATTATGTTTCTGCCCATATTACATTGTTTCACTTTTTGGAGACGAGGTTCTCAATAGTGCCTCTGAATCTATTGCATTCTA
       Consensus CAGAGTTAAAAGCAGCCAGGACACTGGGTGTTCTTGTTCTTGTGTTTCTATTATGTTTCTGCCCATATTACATTGTTTCACTTTTTGGAGACGAGGTTCTCAATAGTGCCTCTGAATCTATTGCATTCTA

                 6371 6380      6390      6400      6410      6420      6430      6440      6450      6460      6470      6480      6490      6500
                 |--------+---------+---------+---------+---------+---------+---------+---------+---------+---------+---------+---------+---------|
    contig034854 TTTCTTTCTTTTAAACTCATGTCTAAACCCTTTGATCTATGCTATGTTCTACCCCTGGTTTAGAAAAGCTGTGAAACTAATTGTGACTCTACAGATACTGCAGCCTGGCTCCTGTGAAGTCAGCATACTG
     BurTARs.A027 TTTCTTTCTTTTAAACTCATGTCTAAACCCTTTGATCTATGCTATGTTCTACCCCTGGTTTAGAAAAGCTGTGAAACTAATTGTGACTCTACAGATACTGCAGCCTGGCTCCTGTGAAGTCAGCATACTG
       Consensus TTTCTTTCTTTTAAACTCATGTCTAAACCCTTTGATCTATGCTATGTTCTACCCCTGGTTTAGAAAAGCTGTGAAACTAATTGTGACTCTACAGATACTGCAGCCTGGCTCCTGTGAAGTCAGCATACTG

                 6501 6510      6520      6530      6540      6550      6560      6570      6580      6590      6600      6610      6620      6630
                 |--------+---------+---------+---------+---------+---------+---------+---------+---------+---------+---------+---------+---------|
    contig034854 TAGAAGGAAGAAATCTGTTCTAAAAAGATTACCATAATCAAAACTAAGTTTAAATTTGGTTCTGGATTTAACAGGGAGAAAATGAAGAGAAGTCAGTATGGGAGAAATATACTCTCGTTCTAGTCACT
     BurTARs.A027 TAG
       Consensus TAG...............................................................................................................................
```

```
             3251 3260      3270      3280      3290      3300      3310      3320      3330      3340      3350      3360      3370      3380
             |--------+---------+---------+---------+---------+---------+---------+---------+---------+---------+---------+---------+---------|
contig057148 GGAGAGGTGAAGGGGGTGTGGAGGTGTGGCGAGATAAATCCCCTATGTGACAAGCAGCTCTGATCAGAGAGCTGCAGGAGCTGCAGCTGTATGATTTTGGAGATGCAGAAAGAAGCCGAGCTTTGTTTTC
BurTARs.A028                                                                                                       ATGCAGAAAGAAGCCGAGCTTTGTTTTC
   Consensus ......................................................................................................ATGCAGAAAGAAGCCGAGCTTTGTTTTC

             3381 3390      3400      3410      3420      3430      3440      3450      3460      3470      3480      3490      3500      3510
             |--------+---------+---------+---------+---------+---------+---------+---------+---------+---------+---------+---------+---------|
contig057148 CACAACTCCTCAACAGCTCCTGCAGGAAGCCAACATTTCACTGGTCCAAAGCTGTGCTGCTGAACATTGTGCTGTGCTGCATCTCTCTGCTCACTGCTGCTCTAAACCTTCTCGTCATCATCTCAGTCTC
BurTARs.A028 CACAACTCCTCAACAGCTCCTGCAGGAAGCCAACATTTCACTGGTCCAAAGCTGTGCTGCTGAACATTGTGCTGTGCTGCATCTCTCTGCTCACTGCTGCTCTAAACCTTCTCGTCATCATCTCAGTCTC
   Consensus CACAACTCCTCAACAGCTCCTGCAGGAAGCCAACATTTCACTGGTCCAAAGCTGTGCTGCTGAACATTGTGCTGTGCTGCATCTCTCTGCTCACTGCTGCTCTAAACCTTCTCGTCATCATCTCAGTCTC

             3511 3520      3530      3540      3550      3560      3570      3580      3590      3600      3610      3620      3630      3640
             |--------+---------+---------+---------+---------+---------+---------+---------+---------+---------+---------+---------+---------|
contig057148 TTACTTCAGGCAGAAATTAACTTCTCAGCTGAAATTGTAGTTTAAGTCTTAAAAACCAATGGAATTAGTGAAGACATTTTTACATTATTAATGTGTAATATATTCTTTTTTTTCTCTCCTTAGGCAGCTT
BurTARs.A028 TTACTTCAGGCAG------------------------------------------------------------------------------------------------------------------CTT
   Consensus TTACTTCAGGCAG..................................................................................................................CTT

             3641 3650      3660      3670      3680      3690      3700      3710      3720      3730      3740      3750      3760      3770
             |--------+---------+---------+---------+---------+---------+---------+---------+---------+---------+---------+---------+---------|
contig057148 CACACACCCACTAATATCCTCCTCCTTTCTCTGGCTGTCTCAGACTTTCTCACTGGTCTCCTTATGCTGCCACTAGAAATAATTAGAAACACGGCCTGCTGGGTACTTGGTGATCTTATGTGTTCTGTTT
BurTARs.A028 CACACACCCACTAATATCCTCCTCCTTTCTCTGGCTGTCTCAGACTTTCTCACTGGTCTCCTTATGCTGCCACTAGAAATAATTAGAAACACGGCCTGCTGGGTACTTGGTGATCTTATGTGTTCTGTTT
   Consensus CACACACCCACTAATATCCTCCTCCTTTCTCTGGCTGTCTCAGACTTTCTCACTGGTCTCCTTATGCTGCCACTAGAAATAATTAGAAACACGGCCTGCTGGGTACTTGGTGATCTTATGTGTTCTGTTT

             3771 3780      3790      3800      3810      3820      3830      3840      3850      3860      3870      3880      3890      3900
             |--------+---------+---------+---------+---------+---------+---------+---------+---------+---------+---------+---------+---------|
contig057148 ATACTTATCTGACAGCCGGTCTTTTCTGTGCTTCAACTTGGAACATAGTTCTCATTTCAGTTGACCGCTATGTAGCTATTTGTCACCCTCTTCATTACCCCACCAGAATTACCGTGGCAAGAGTCAAACT
BurTARs.A028 ATACTTATCTGACAGCCGGTCTTTTCTGTGCTTCAACTTGGAACATAGTTCTCATTTCAGTTGACCGCTATGTAGCTATTTGTCACCCTCTTCATTACCCCACCAGAATTACCGTGGCAAGAGTCAAACT
   Consensus ATACTTATCTGACAGCCGGTCTTTTCTGTGCTTCAACTTGGAACATAGTTCTCATTTCAGTTGACCGCTATGTAGCTATTTGTCACCCTCTTCATTACCCCACCAGAATTACCGTGGCAAGAGTCAAACT

             3901 3910      3920      3930      3940      3950      3960      3970      3980      3990      4000      4010      4020      4030
             |--------+---------+---------+---------+---------+---------+---------+---------+---------+---------+---------+---------+---------|
contig057148 CTGTGTTTGTTTGTGTTGGTTTTGTTCAACCTTCTACGGCAGTCTTTTAACAAAGGATGTTCTAATTAAGCCGGCCAGGTATAATTCCTGTTATGGTGAGTGTGTGTTTGTCATTAATGATATTGCAGGG
BurTARs.A028 CTGTGTTTGTTTGTGTTGGTTTTGTTCAACCTTCTACGGCAGTCTTTTAACAAAGGATGTTCTAATTAAGCCGGCCAGGTATAATTCCTGTTATGGTGAGTGTGTGTTTGTCATTAATGATATTGCAGGG
   Consensus CTGTGTTTGTTTGTGTTGGTTTTGTTCAACCTTCTACGGCAGTCTTTTAACAAAGGATGTTCTAATTAAGCCGGCCAGGTATAATTCCTGTTATGGTGAGTGTGTGTTTGTCATTAATGATATTGCAGGG

             4031 4040      4050      4060      4070      4080      4090      4100      4110      4120      4130      4140      4150      4160
             |--------+---------+---------+---------+---------+---------+---------+---------+---------+---------+---------+---------+---------|
contig057148 TTTATTGATCTTGTGTTATCTTTTATTTTTCCAGTATCTGTTATCATAGTTCTTTATATGAGAGTGTTTGTGGTGGCTGTGTCTCAAGCTCGTGCCATGCGCTCTCATGTTACAGCTGTCGCACTTCAGC
BurTARs.A028 TTTATTGATCTTGTGTTATCTTTTATTTTTCCAGTATCTGTTATCATAGTTCTTTATATGAGAGTGTTTGTGGTGGCTGTGTCTCAAGCTCGTGCCATGCGCTCTCATGTTACAGCTGTCGCACTTCAGC
   Consensus TTTATTGATCTTGTGTTATCTTTTATTTTTCCAGTATCTGTTATCATAGTTCTTTATATGAGAGTGTTTGTGGTGGCTGTGTCTCAAGCTCGTGCCATGCGCTCTCATGTTACAGCTGTCGCACTTCAGC

             4161 4170      4180      4190      4200      4210      4220      4230      4240      4250      4260      4270      4280      4290
             |--------+---------+---------+---------+---------+---------+---------+---------+---------+---------+---------+---------+---------|
contig057148 GTTCAAGCAATCAAGCAAACAAATCTGAACTGAAAGCAGCCAGGAACCTTGGAGTTCTTGTAGTTGTGTTTCTACTGTGTTACCTTCCGTTTTACTGCTACTCTCTTATTGAAGTAAATGTGATCAATGA
BurTARs.A028 GTTCAAGCAATCAAGCAAACAAATCTGAACTGAAAGCAGCCAGGAACCTTGGAGTTCTTGTAGTTGTGTTTCTACTGTGTTACCTTCCGTTTTACTGCTACTCTCTTATTGAAGTAAATGTGATCAATGA
   Consensus GTTCAAGCAATCAAGCAAACAAATCTGAACTGAAAGCAGCCAGGAACCTTGGAGTTCTTGTAGTTGTGTTTCTACTGTGTTACCTTCCGTTTTACTGCTACTCTCTTATTGAAGTAAATGTGATCAATGA

             4291 4300      4310      4320      4330      4340      4350      4360      4370      4380      4390      4400      4410      4420
             |--------+---------+---------+---------+---------+---------+---------+---------+---------+---------+---------+---------+---------|
contig057148 CTCATCTACATTTTTTCTGATCATTGTGTTTTACTTTAACTCGTGTCTAAACCCACTGATGTATGCCCTTTTTTACTCTTGGTTTAGAAATGCTGTTAAACTTATTATCACTCTGCAGATTTTTAAGCAT
BurTARs.A028 CTCATCTACATTTTTTCTGATCATTGTGTTTTACTTTAACTCGTGTCTAAACCCACTGATGTATGCCCTTTTTTACTCTTGGTTTAGAAATGCTGTTAAACTTATTATCACTCTGCAGATTTTTAAGCAT
   Consensus CTCATCTACATTTTTTCTGATCATTGTGTTTTACTTTAACTCGTGTCTAAACCCACTGATGTATGCCCTTTTTTACTCTTGGTTTAGAAATGCTGTTAAACTTATTATCACTCTGCAGATTTTTAAGCAT

             4421 4430      4440      4450      4460      4470      4480      4490      4500      4510      4520      4530      4540      4550
             |--------+---------+---------+---------+---------+---------+---------+---------+---------+---------+---------+---------+---------|
contig057148 GACACTACTGAAGCAAACATGTTTTAAAAAGATAAGGAAATCCACTCTACTAACAATTGAAATCAATAAATCAATAAATTCAGAAATCTGAAAATGAAAATTTTGACCTTGTCACAAATGAGGAAATATA
BurTARs.A028 GACACTACTGAAGCAAACATGTTTTAA
   Consensus GACACTACTGAAGCAAACATGTTTTAA.......................................................................................................
```

```
             4681 4690      4700      4710      4720      4730      4740      4750      4760      4770      4780      4790      4800      4810
             |--------+---------+---------+---------+---------+---------+---------+---------+---------+---------+---------+---------+---------|
contig059766 AGCAGTGATGGAGGAAACTGAACTCTGCTTTCAACAACTCTTTAACACCTCCTGCATGAGGCCCAGGCGTCCACACTTTGAGATCATGCTGACTTATATTTTGCTGTCCTTCATTTCTCTGCTTACTGTG
BurTARs.A029        ATGGAGGAAACTGAACTCTGCTTTCAACAACTCTTTAACACCTCCTGCATGAGGCCCAGGCGTCCACACTTTGAGATCATGCTGACTTATATTTTGCTGTCCTTCATTTCTCTGCTTACTGTG
   Consensus .......ATGGAGGAAACTGAACTCTGCTTTCAACAACTCTTTAACACCTCCTGCATGAGGCCCAGGCGTCCACACTTTGAGATCATGCTGACTTATATTTTGCTGTCCTTCATTTCTCTGCTTACTGTG

             4811 4820      4830      4840      4850      4860      4870      4880      4890      4900      4910      4920      4930      4940
             |--------+---------+---------+---------+---------+---------+---------+---------+---------+---------+---------+---------+---------|
contig059766 ATTCTTAACCTGTTGGTCATCATCTCCGTCTCACATTTCAGGTAGAAAGATATCAAACTGAATTGATCTCAAGCAGTAAGAAAGACATTTGTTTTCACAGATAGTATTTCTTTTCTTTTTAAGCCTCTGC
BurTARs.A029 ATTCTTAACCTGTTGGTCATCATCTCCGTCTCACATTTCAGG----------------------------------------------------------------------------------------
   Consensus ATTCTTAACCTGTTGGTCATCATCTCCGTCTCACATTTCAGG........................................................................................

             4941 4950      4960      4970      4980      4990      5000      5010      5020      5030      5040      5050      5060      5070
             |--------+---------+---------+---------+---------+---------+---------+---------+---------+---------+---------+---------+---------|
contig059766 ACTATATACTTAAATTTGAATAGCACTTGATAGAGCAAACTAAATATAAAATATTGTGTTTTCTCTCCCTATTCAGGCAGCTCCACACCCCCACCAACCTCCTGCTCCTTTCTCTGGCTGTCGCTGATTT
BurTARs.A029 -----------------------------------------------------------------------------CAGCTCCACACCCCCACCAACCTCCTGCTCCTTTCTCTGGCTGTCGCTGATTT
   Consensus .............................................................................CAGCTCCACACCCCCACCAACCTCCTGCTCCTTTCTCTGGCTGTCGCTGATTT

             5071 5080      5090      5100      5110      5120      5130      5140      5150      5160      5170      5180      5190      5200
             |--------+---------+---------+---------+---------+---------+---------+---------+---------+---------+---------+---------+---------|
contig059766 CTATGTGGGCCTCCTCTTGTTCTTCCAAATTGTGCTCATAGATGGCTGCTGGTTCCTCGGTGACATCATGTGCACTCTGTATCAATACCTAGCATATGTCATCACCTCGGCCTCCATAGGAACCATGGTG
BurTARs.A029 CTATGTGGGCCTCCTCTTGTTCTTCCAAATTGTGCTCATAGATGGCTGCTGGTTCCTCGGTGACATCATGTGCACTCTGTATCAATACCTAGCATATGTCATCACCTCGGCCTCCATAGGAACCATGGTG
   Consensus CTATGTGGGCCTCCTCTTGTTCTTCCAAATTGTGCTCATAGATGGCTGCTGGTTCCTCGGTGACATCATGTGCACTCTGTATCAATACCTAGCATATGTCATCACCTCGGCCTCCATAGGAACCATGGTG

             5201 5210      5220      5230      5240      5250      5260      5270      5280      5290      5300      5310      5320      5330
             |--------+---------+---------+---------+---------+---------+---------+---------+---------+---------+---------+---------+---------|
contig059766 ATCATATCTGTTGATCGATATTTGGCTATTTGTTACCCTCTGCATTACTCCACCAAGATCACACAACAAAGAGTTAAAATTGTTGTCTGTTTGTGTTGGATCTGTTCTGTCATCTTTCAAAGTCTGATTC
BurTARs.A029 ATCATATCTGTTGATCGATATTTGGCTATTTGTTACCCTCTGCATTACTCCACCAAGATCACACAACAAAGAGTTAAAATTGTTGTCTGTTTGTGTTGGATCTGTTCTGTCATCTTTCAAAGTCTGATTC
   Consensus ATCATATCTGTTGATCGATATTTGGCTATTTGTTACCCTCTGCATTACTCCACCAAGATCACACAACAAAGAGTTAAAATTGTTGTCTGTTTGTGTTGGATCTGTTCTGTCATCTTTCAAAGTCTGATTC

             5331 5340      5350      5360      5370      5380      5390      5400      5410      5420      5430      5440      5450      5460
             |--------+---------+---------+---------+---------+---------+---------+---------+---------+---------+---------+---------+---------|
contig059766 TGATGGATAACCTGGAGCAACCAGGCAGGTATAACTCTTGCATTGGAGAGTGTGTCTTTGTCATTAATTACATCGCAGGACTTGTTGATGTTACTTTTTCCTTTATTGTTCCCTTTACTGTGATTGTAGT
BurTARs.A029 TGATGGATAACCTGGAGCAACCAGGCAGGTATAACTCTTGCATTGGAGAGTGTGTCTTTGTCATTAATTACATCGCAGGACTTGTTGATGTTACTTTTTCCTTTATTGTTCCCTTTACTGTGATTGTAGT
   Consensus TGATGGATAACCTGGAGCAACCAGGCAGGTATAACTCTTGCATTGGAGAGTGTGTCTTTGTCATTAATTACATCGCAGGACTTGTTGATGTTACTTTTTCCTTTATTGTTCCCTTTACTGTGATTGTAGT

             5461 5470      5480      5490      5500      5510      5520      5530      5540      5550      5560      5570      5580      5590
             |--------+---------+---------+---------+---------+---------+---------+---------+---------+---------+---------+---------+---------|
contig059766 TTTGTATCTGAGAGTGTTTGTGGTGGCTGTGTCTCAGGCTCGTGCCATGAGGTCTCAGCTTGCAGTCACTCACCAGCGATCAGTAACAGTAACTGCAAAGAAATCGGAGCTGAAAGCAGCCTGGACTCTT
BurTARs.A029 TTTGTATCTGAGAGTGTTTGTGGTGGCTGTGTCTCAGGCTCGTGCCATGAGGTCTCAGCTTGCAGTCACTCACCAGCGATCAGTAACAGTAACTGCAAAGAAATCGGAGCTGAAAGCAGCCTGGACTCTT
   Consensus TTTGTATCTGAGAGTGTTTGTGGTGGCTGTGTCTCAGGCTCGTGCCATGAGGTCTCAGCTTGCAGTCACTCACCAGCGATCAGTAACAGTAACTGCAAAGAAATCGGAGCTGAAAGCAGCCTGGACTCTT

             5591 5600      5610      5620      5630      5640      5650      5660      5670      5680      5690      5700      5710      5720
             |--------+---------+---------+---------+---------+---------+---------+---------+---------+---------+---------+---------+---------|
contig059766 GGTATTGTTGTTTTTGTGTTTCTCATATGTATGTGTCCATATTACTGTGTGGCTCTCACAGGCCAAGATAACTTGCCAAGTGCTTCATCACTGACATTTGTTCTATGTTTGGTCTACTTTAACTCATGTC
BurTARs.A029 GGTATTGTTGTTTTTGTGTTTCTCATATGTATGTGTCCATATTACTGTGTGGCTCTCACAGGCCAAGATAACTTGCCAAGTGCTTCATCACTGACATTTGTTCTATGTTTGGTCTACTTTAACTCATGTC
   Consensus GGTATTGTTGTTTTTGTGTTTCTCATATGTATGTGTCCATATTACTGTGTGGCTCTCACAGGCCAAGATAACTTGCCAAGTGCTTCATCACTGACATTTGTTCTATGTTTGGTCTACTTTAACTCATGTC

             5721 5730      5740      5750      5760      5770      5780      5790      5800      5810      5820      5830      5840      5850
             |--------+---------+---------+---------+---------+---------+---------+---------+---------+---------+---------+---------+---------|
contig059766 TGAACCCCATTATATATGTCTTTTTCTACCCTTGGTTCAGAAAATCCATAAAAGTCATTGTTACTCTTCAGATACTGCAGCCTGACTCCTGCCAGGCCACCGTGCTTTAGAGGACCGTTTCTCTCTCTCT
BurTARs.A029 TGAACCCCATTATATATGTCTTTTTCTACCCTTGGTTCAGAAAATCCATAAAAGTCATTGTTACTCTTCAGATACTGCAGCCTGACTCCTGCCAGGCCACCGTGCTTTAG
   Consensus TGAACCCCATTATATATGTCTTTTTCTACCCTTGGTTCAGAAAATCCATAAAAGTCATTGTTACTCTTCAGATACTGCAGCCTGACTCCTGCCAGGCCACCGTGCTTTAG....................
```

```
           1301 1310      1320      1330      1340      1350      1360      1370      1380      1390      1400      1410      1420      1430
           |--------+---------+---------+---------+---------+---------+---------+---------+---------+---------+---------+---------+---------|
contig065494 AGTTCAGTTTGCCGTTCTTCCAGCAGTGATGGAGGAAACTGAACTCTGCTTTCAACAACTCTTTAACACCTCCTGCATGAGGCCCAGGCGTCCACACTTTGAGATCATGCTGACTTATATTTTGCTGTCC
BurTARs.A030                             ATGGAGGAAACTGAACTCTGCTTTCAACAACTCTTTAACACCTCCTGCATGAGGCCCAGGCGTCCACACTTTGAGATCATGCTGACTTATATTTTGCTGTCC
   Consensus ............................ATGGAGGAAACTGAACTCTGCTTTCAACAACTCTTTAACACCTCCTGCATGAGGCCCAGGCGTCCACACTTTGAGATCATGCTGACTTATATTTTGCTGTCC

           1431 1440      1450      1460      1470      1480      1490      1500      1510      1520      1530      1540      1550      1560
           |--------+---------+---------+---------+---------+---------+---------+---------+---------+---------+---------+---------+---------|
contig065494 TTCATTTCTCTGCTTACTGTGATTCTTAACCTGTTGGTCATCATCTCCGTCTCACATTTCAGGTAGAAAGATATCAAACTGAATTGATCTCAAGCAGTAAGAAAGAAATTTGTTTTCACAGATAGTATTT
BurTARs.A030 TTCATTTCTCTGCTTACTGTGATTCTTAACCTGTTGGTCATCATCTCCGTCTCACATTTCAGG-------------------------------------------------------------------
   Consensus TTCATTTCTCTGCTTACTGTGATTCTTAACCTGTTGGTCATCATCTCCGTCTCACATTTCAGG...................................................................

           1561 1570      1580      1590      1600      1610      1620      1630      1640      1650      1660      1670      1680      1690
           |--------+---------+---------+---------+---------+---------+---------+---------+---------+---------+---------+---------+---------|
contig065494 CTTTTCTTTTTAAGCCTCTGCACTATATACTTAAATTTGAATAGCACTTGATAGAGCAAACTAAATATAAAATATTGTGTTTTCTCTCCCTATTCAGGCAGCTCCACACCCCCACCAACCTCCTGCTCCT
BurTARs.A030 --------------------------------------------------------------------------------------------------CAGCTCCACACCCCCACCAACCTCCTGCTCCT
   Consensus ..................................................................................................CAGCTCCACACCCCCACCAACCTCCTGCTCCT

           1691 1700      1710      1720      1730      1740      1750      1760      1770      1780      1790      1800      1810      1820
           |--------+---------+---------+---------+---------+---------+---------+---------+---------+---------+---------+---------+---------|
contig065494 TTCTCTGGCTGTCGCTGATTTCTATGTGGGCCTCCTCTTGTTCTTCCAAATTGTGCTCATAGATGGCTGCTGGTTCCTCGGTGACATCATGTGCACTCTGTATCAATACCTAGCATATGTCATCACCTCG
BurTARs.A030 TTCTCTGGCTGTCGCTGATTTCTATGTGGGCCTCCTCTTGTTCTTCCAAATTGTGCTCATAGATGGCTGCTGGTTCCTCGGTGACATCATGTGCACTCTGTATCAATACCTAGCATATGTCATCACCTCG
   Consensus TTCTCTGGCTGTCGCTGATTTCTATGTGGGCCTCCTCTTGTTCTTCCAAATTGTGCTCATAGATGGCTGCTGGTTCCTCGGTGACATCATGTGCACTCTGTATCAATACCTAGCATATGTCATCACCTCG

           1821 1830      1840      1850      1860      1870      1880      1890      1900      1910      1920      1930      1940      1950
           |--------+---------+---------+---------+---------+---------+---------+---------+---------+---------+---------+---------+---------|
contig065494 GCCTCCATAGGAACCATGGTGATCATATCTGTTGATCGATATTTGGCTATTTGTTACCCTCTGCATTACTCCACCAAGATCACACAACAAAGAGTTAAAATTGTTGTCTGTTTGTGTTGGATCTGTTCTG
BurTARs.A030 GCCTCCATAGGAACCATGGTGATCATATCTGTTGATCGATATTTGGCTATTTGTTACCCTCTGCATTACTCCACCAAGATCACACAACAAAGAGTTAAAATTGTTGTCTGTTTGTGTTGGATCTGTTCTG
   Consensus GCCTCCATAGGAACCATGGTGATCATATCTGTTGATCGATATTTGGCTATTTGTTACCCTCTGCATTACTCCACCAAGATCACACAACAAAGAGTTAAAATTGTTGTCTGTTTGTGTTGGATCTGTTCTG

           1951 1960      1970      1980      1990      2000      2010      2020      2030      2040      2050      2060      2070      2080
           |--------+---------+---------+---------+---------+---------+---------+---------+---------+---------+---------+---------+---------|
contig065494 TCATCTTTCAAAGTCTGATTCTGATGGATAACCTGGAGCAACCAGGCAGGTATAACTCTTGCATTGGAGAGTGTGTCTTTGTCATTAATTACATCGCAGGACTTGTTGATGTTACTTTTTCCTTTATTGT
BurTARs.A030 TCATCTTTCAAAGTCTGATTCTGATGGATAACCTGGAGCAACCAGGCAGGTATAACTCTTGCATTGGAGAGTGTGTCTTTGTCATTAATTACATCGCAGGACTTGTTGATGTTACTTTTTCCTTTATTGT
   Consensus TCATCTTTCAAAGTCTGATTCTGATGGATAACCTGGAGCAACCAGGCAGGTATAACTCTTGCATTGGAGAGTGTGTCTTTGTCATTAATTACATCGCAGGACTTGTTGATGTTACTTTTTCCTTTATTGT

           2081 2090      2100      2110      2120      2130      2140      2150      2160      2170      2180      2190      2200      2210
           |--------+---------+---------+---------+---------+---------+---------+---------+---------+---------+---------+---------+---------|
contig065494 TCCCTTTACTGTGATTGTAGTTTTGTATCTGAGAGTGTTTGTGGTGGCTGTGTCTCAGGCTCGTGCCATGAGGTCTCAGCTTGCAGTCACTCACCAGCGATCAGTAACAGTAACTGCAAAGAAATCGGAG
BurTARs.A030 TCCCTTTACTGTGATTGTAGTTTTGTATCTGAGAGTGTTTGTGGTGGCTGTGTCTCAGGCTCGTGCCATGAGGTCTCAGCTTGCAGTCACTCACCAGCGATCAGTAACAGTAACTGCAAAGAAATCGGAG
   Consensus TCCCTTTACTGTGATTGTAGTTTTGTATCTGAGAGTGTTTGTGGTGGCTGTGTCTCAGGCTCGTGCCATGAGGTCTCAGCTTGCAGTCACTCACCAGCGATCAGTAACAGTAACTGCAAAGAAATCGGAG

           2211 2220      2230      2240      2250      2260      2270      2280      2290      2300      2310      2320      2330      2340
           |--------+---------+---------+---------+---------+---------+---------+---------+---------+---------+---------+---------+---------|
contig065494 CTGAAAGCAGCCTGGACTCTTGGTATTGTTGTTGTTGTGTTTCTCATATGTATGTGTCCATATTACTGTGTGGCTCTCACAGGCCAAGACAGCTTGCCAAGTGCTTCATCACTGACATTTGTTCTATGCT
BurTARs.A030 CTGAAAGCAGCCTGGACTCTTGGTATTGTTGTTGTTGTGTTTCTCATATGTATGTGTCCATATTACTGTGTGGCTCTCACAGGCCAAGACAGCTTGCCAAGTGCTTCATCACTGACATTTGTTCTATGCT
   Consensus CTGAAAGCAGCCTGGACTCTTGGTATTGTTGTTGTTGTGTTTCTCATATGTATGTGTCCATATTACTGTGTGGCTCTCACAGGCCAAGACAGCTTGCCAAGTGCTTCATCACTGACATTTGTTCTATGCT

           2341 2350      2360      2370      2380      2390      2400      2410      2420      2430      2440      2450      2460      2470
           |--------+---------+---------+---------+---------+---------+---------+---------+---------+---------+---------+---------+---------|
contig065494 TAGCCTACTTTAACTCATGTCTGAACCCCATTATATATGTCTTTTTCTACCCCTGGTTCAGAAAATCTATAAAAGTCATTGTTACTCTTCAGATACTGCAGCCTGACTCTTCTAAAGTCACCATGCGTTA
BurTARs.A030 TAGCCTACTTTAACTCATGTCTGAACCCCATTATATATGTCTTTTTCTACCCCTGGTTCAGAAAATCTATAAAAGTCATTGTTACTCTTCAGATACTGCAGCCTGACTCTTCTAAAGTCACCATGCGTTA
   Consensus TAGCCTACTTTAACTCATGTCTGAACCCCATTATATATGTCTTTTTCTACCCCTGGTTCAGAAAATCTATAAAAGTCATTGTTACTCTTCAGATACTGCAGCCTGACTCTTCTAAAGTCACCATGCGTTA

           2471 2480      2490      2500      2510      2520      2530      2540      2550      2560      2570      2580      2590      2600
           |--------+---------+---------+---------+---------+---------+---------+---------+---------+---------+---------+---------+---------|
contig065494 GACCAAGATTTTGAAACTCTTCATCACACACAAACAAACACACACACACACACACACACACACACACACACACACACACACACACACAAATAAATAAACTACTCACAACTAATAAAGTACTCATTGGT
BurTARs.A030 G
   Consensus G.................................................................................................................................
```

```
             651  660       670       680       690       700       710       720       730       740       750       760       770       780
             |--------+---------+---------+---------+---------+---------+---------+---------+---------+---------+---------+---------+---------|
contig057305 CTTTAATCATCAAGTAAGAAAGAATCTTAGAAGAGGGACAACGTTATAACATTTGAGTTTGTTAGAAAGGTGTCAGGGGCTCATAAGCACTTCTAACTGATGATGGCAGAAACTGAACTCTGCTTTCCAA
BurTARs.A031                                                                                                       ATGGCAGAAACTGAACTCTGCTTTCCAA
   Consensus ......................................................................................................ATGGCAGAAACTGAACTCTGCTTTCCAA

             781  790       800       810       820       830       840       850       860       870       880       890       900       910
             |--------+---------+---------+---------+---------+---------+---------+---------+---------+---------+---------+---------+---------|
contig057305 ATCTCAACTCCTCCTGCAGTAGAGTAAAGCGTTCTCCCTCGGACTCAGTGCTTATTTATGTAATCCTAACCATCATTTCTCTTCTTACTGTGGCTCTAAACCTTCTGGTCATCATCTCCATCTCCCACTT
BurTARs.A031 ATCTCAACTCCTCCTGCAGTAGAGTAAAGCGTTCTCCCTCGGACTCAGTGCTTATTTATGTAATCCTAACCATCATTTCTCTTCTTACTGTGGCTCTAAACCTTCTGGTCATCATCTCCATCTCCCACTT
   Consensus ATCTCAACTCCTCCTGCAGTAGAGTAAAGCGTTCTCCCTCGGACTCAGTGCTTATTTATGTAATCCTAACCATCATTTCTCTTCTTACTGTGGCTCTAAACCTTCTGGTCATCATCTCCATCTCCCACTT

             911  920       930       940       950       960       970       980       990       1000      1010      1020      1030      1040
             |--------+---------+---------+---------+---------+---------+---------+---------+---------+---------+---------+---------+---------|
contig057305 CAAGTAGATACTTAATACATTTATAAGTCTAGCAGTTGTACAGGAGAAAATGACTAACATAAAATAAGTTAAACAGAACAACTTTTTGACAATGTTTTGATGTGGATGATCGGCTGGAATAATTGATGCT
BurTARs.A031 CAAG------------------------------------------------------------------------------------------------------------------------------
   Consensus CAAG..............................................................................................................................

             1041 1050      1060      1070      1080      1090      1100      1110      1120      1130      1140      1150      1160      1170
             |--------+---------+---------+---------+---------+---------+---------+---------+---------+---------+---------+---------+---------|
contig057305 ACAAATAATATATATTTTTCTATCTCTTATCTATCCAGGCAGCTCCACACTCCTACAAACCACCTCCTCCTCTCCCTGGCAGTCTCTGATTTCTTTGTGGGTCTCAATATGTGCTTTCAAAGTATGCTCG
BurTARs.A031 ---------------------------------------CAGCTCCACACTCCTACAAACCACCTCCTCCTCTCCCTGGCAGTCTCTGATTTCTTTGTGGGTCTCAATATGTGCTTTCAAAGTATGCTCG
   Consensus .......................................CAGCTCCACACTCCTACAAACCACCTCCTCCTCTCCCTGGCAGTCTCTGATTTCTTTGTGGGTCTCAATATGTGCTTTCAAAGTATGCTCG

             1171 1180      1190      1200      1210      1220      1230      1240      1250      1260      1270      1280      1290      1300
             |--------+---------+---------+---------+---------+---------+---------+---------+---------+---------+---------+---------+---------|
contig057305 TAGATGGTTGTTGGTATCTTGGTGACCTCATGTGTGTCTTGTATTATGTTTTCGACATAGTTGTTACTTCTGCCTCAGTAGGAACCATGGTGCTCATTTCAGTTGACCGTTATGTGGCCATTTGTGATCC
BurTARs.A031 TAGATGGTTGTTGGTATCTTGGTGACCTCATGTGTGTCTTGTATTATGTTTTCGACATAGTTGTTACTTCTGCCTCAGTAGGAACCATGGTGCTCATTTCAGTTGACCGTTATGTGGCCATTTGTGATCC
   Consensus TAGATGGTTGTTGGTATCTTGGTGACCTCATGTGTGTCTTGTATTATGTTTTCGACATAGTTGTTACTTCTGCCTCAGTAGGAACCATGGTGCTCATTTCAGTTGACCGTTATGTGGCCATTTGTGATCC

             1301 1310      1320      1330      1340      1350      1360      1370      1380      1390      1400      1410      1420      1430
             |--------+---------+---------+---------+---------+---------+---------+---------+---------+---------+---------+---------+---------|
contig057305 TCTTCATTATCCCACCAAAGTCACTCCAAAAAGAGTTCAGACCTGTGTCTTAATGTGTTGGATTTGCTCGCTCCTAGTAGTCGGTGTGCTCTTGAAGGATAACCTGGATAAACCAGGTAGATTTAATTCC
BurTARs.A031 TCTTCATTATCCCACCAAAGTCACTCCAAAAAGAGTTCAGACCTGTGTCTTAATGTGTTGGATTTGCTCGCTCCTAGTAGTCGGTGTGCTCTTGAAGGATAACCTGGATAAACCAGGTAGATTTAATTCC
   Consensus TCTTCATTATCCCACCAAAGTCACTCCAAAAAGAGTTCAGACCTGTGTCTTAATGTGTTGGATTTGCTCGCTCCTAGTAGTCGGTGTGCTCTTGAAGGATAACCTGGATAAACCAGGTAGATTTAATTCC

             1431 1440      1450      1460      1470      1480      1490      1500      1510      1520      1530      1540      1550      1560
             |--------+---------+---------+---------+---------+---------+---------+---------+---------+---------+---------+---------+---------|
contig057305 TGCTTTGGAGAGTGTGTGATTTATGTTGACTTTGCAATACAAGTTACCGATCTTATTTTGACAATCCTCCTTCCCATTACTGTCATCGTAATTTTGTATGTAAGAGTATTTGCCGTGGCCGTGTTTCAGA
BurTARs.A031 TGCTTTGGAGAGTGTGTGATTTATGTTGACTTTGCAATACAAGTTACCGATCTTATTTTGACAATCCTCCTTCCCATTACTGTCATCGTAATTTTGTATGTAAGAGTATTTGCCGTGGCCGTGTTTCAGA
   Consensus TGCTTTGGAGAGTGTGTGATTTATGTTGACTTTGCAATACAAGTTACCGATCTTATTTTGACAATCCTCCTTCCCATTACTGTCATCGTAATTTTGTATGTAAGAGTATTTGCCGTGGCCGTGTTTCAGA

             1561 1570      1580      1590      1600      1610      1620      1630      1640      1650      1660      1670      1680      1690
             |--------+---------+---------+---------+---------+---------+---------+---------+---------+---------+---------+---------+---------|
contig057305 TTCGGGCCATGCAACCTCATGTTCCAGCTGTCACACAGAGGGGGAAAGTAAGTCCAAAAAAATCTGAACTGAAAGCAGCTAGGACTCTTGGCATTGTTATTGTTGCATTTCTGATATGCCTATTCCCATA
BurTARs.A031 TTCGGGCCATGCAACCTCATGTTCCAGCTGTCACACAGAGGGGGAAAGTAAGTCCAAAAAAATCTGAACTGAAAGCAGCTAGGACTCTTGGCATTGTTATTGTTGCATTTCTGATATGCCTATTCCCATA
   Consensus TTCGGGCCATGCAACCTCATGTTCCAGCTGTCACACAGAGGGGGAAAGTAAGTCCAAAAAAATCTGAACTGAAAGCAGCTAGGACTCTTGGCATTGTTATTGTTGCATTTCTGATATGCCTATTCCCATA

             1691 1700      1710      1720      1730      1740      1750      1760      1770      1780      1790      1800      1810      1820
             |--------+---------+---------+---------+---------+---------+---------+---------+---------+---------+---------+---------+---------|
contig057305 TTACAGTGTAATACTCTCAGGCCAGGACACACTGCTTGATATTTTATCCGTTACCTTTTTTTTATGTTTGTTTTATTTTAATTCCTGTCTCAACCCTATCATCTATGCCTTTTTCTACCCATGGTTTAGA
BurTARs.A031 TTACAGTGTAATACTCTCAGGCCAGGACACACTGCTTGATATTTTATCCGTTACCTTTTTTTTATGTTTGTTTTATTTTAATTCCTGTCTCAACCCTATCATCTATGCCTTTTTCTACCCATGGTTTAGA
   Consensus TTACAGTGTAATACTCTCAGGCCAGGACACACTGCTTGATATTTTATCCGTTACCTTTTTTTTATGTTTGTTTTATTTTAATTCCTGTCTCAACCCTATCATCTATGCCTTTTTCTACCCATGGTTTAGA

             1821 1830      1840      1850      1860      1870      1880      1890      1900      1910      1920      1930      1940      1950
             |--------+---------+---------+---------+---------+---------+---------+---------+---------+---------+---------+---------+---------|
contig057305 AAATCTGTTAAACTTATTGTTACATTTCAAATAGTCAAGTCTGGCTCCAGTGATGCCAGCATGCTGTGACTGAAATAAGCCCTTCAAGTCAAGCCAAATAAATAAAATAATATATTTCCATATTTCAAATTG
BurTARs.A031 AAATCTGTTAAACTTATTGTTACATTTCAAATAGTCAAGTCTGGCTCCAGTGATGCCAGCATGCTGTGA
   Consensus AAATCTGTTAAACTTATTGTTACATTTCAAATAGTCAAGTCTGGCTCCAGTGATGCCAGCATGCTGTGA.............................................................
```

```
              ...................................................................................................................................

              4941 4950      4960      4970      4980      4990      5000      5010      5020      5030      5040      5050      5060      5070
              |--------+---------+---------+---------+---------+---------+---------+---------+---------+---------+---------+---------+---------|
contig006087  ACAACCTAAAGAATGCATTGTTTGTTTGTTTGTTCATGAGAACAGAGAAAGAAATGTTTTTTGAATGCTGAAAATTGAAACTTGTTCCAGGGCTGTGACAGGGAGTCATGGAGTTATTCAATGTGACTG
  BurTAR.B032                                                                                                                ATGGAGTTATTCAATGTGACTG
    Consensus  ...........................................................................................................ATGGAGTTATTCAATGTGACTG

              5071 5080      5090      5100      5110      5120      5130      5140      5150      5160      5170      5180      5190      5200
              |--------+---------+---------+---------+---------+---------+---------+---------+---------+---------+---------+---------+---------|
contig006087  TTAATACTGTGAGCTTCCTTCTCTGCGATTCACAAAAGAACAAATTATGTGTGTTATTATATGTTGTCCTCAGCTCATTTATGCTTCTTACAATATGTGGAAATCTTCTTGTGATAATTTCTATAATTTA
  BurTAR.B032 TTAATACTGTGAGCTTCCTTCTCTGCGATTCACAAAAGAACAAATTATGTGTGTTATTATATGTTGTCCTCAGCTCATTTATGCTTCTTACAATATGTGGAAATCTTCTTGTGATAATTTCTATAATTTA
    Consensus TTAATACTGTGAGCTTCCTTCTCTGCGATTCACAAAAGAACAAATTATGTGTGTTATTATATGTTGTCCTCAGCTCATTTATGCTTCTTACAATATGTGGAAATCTTCTTGTGATAATTTCTATAATTTA

              5201 5210      5220      5230      5240      5250      5260      5270      5280      5290      5300      5310      5320      5330
              |--------+---------+---------+---------+---------+---------+---------+---------+---------+---------+---------+---------+---------|
contig006087  CTTTAGATATCTCCACACTCCTACAAACTACCTTATCCTCTCTATGGCTGTGGCTGATCTACTAATTGGTGCTTTAATATTTCCTTTGAGCATGACAGTGTCTCTAAAGCCATGTTTGTATATATACAGT
  BurTAR.B032 CTTTAGATATCTCCACACTCCTACAAACTACCTTATCCTCTCTATGGCTGTGGCTGATCTACTAATTGGTGCTTTAATATTTCCTTTGAGCATGACAGTGTCTCTAAAGCCATGTTTGTATATATACAGT
    Consensus CTTTAGATATCTCCACACTCCTACAAACTACCTTATCCTCTCTATGGCTGTGGCTGATCTACTAATTGGTGCTTTAATATTTCCTTTGAGCATGACAGTGTCTCTAAAGCCATGTTTGTATATATACAGT

              5331 5340      5350      5360      5370      5380      5390      5400      5410      5420      5430      5440      5450      5460
              |--------+---------+---------+---------+---------+---------+---------+---------+---------+---------+---------+---------+---------|
contig006087  TTACTGTGCAACTTAAGAAGCACAATGGATGTAACAATGGGTGTATCTTCCTTATTAAATTTGTGCTGTATTTCTGTTGATCGATATTATGCTGTTTGCCACCCTCTGATATATAAAACTAAAATAACTG
  BurTAR.B032 TTACTGTGCAACTTAAGAAGCACAATGGATGTAACAATGGGTGTATCTTCCTTATTAAATTTGTGCTGTATTTCTGTTGATCGATATTATGCTGTTTGCCACCCTCTGATATATAAAACTAAAATAACTG
    Consensus TTACTGTGCAACTTAAGAAGCACAATGGATGTAACAATGGGTGTATCTTCCTTATTAAATTTGTGCTGTATTTCTGTTGATCGATATTATGCTGTTTGCCACCCTCTGATATATAAAACTAAAATAACTG

              5461 5470      5480      5490      5500      5510      5520      5530      5540      5550      5560      5570      5580      5590
              |--------+---------+---------+---------+---------+---------+---------+---------+---------+---------+---------+---------+---------|
contig006087  ATTGTGTTGCCATGAAGATGGGCCTTGGAAGTTGGGCTGTTGCTATCTTGTGTGGAATTTTTGTCTTCCTGCTGTTTTTTATTTTAGACGAATGTGACACAAGTTGCCTTTTCGCTCTCATTGCCGCATC
  BurTAR.B032 ATTGTGTTGCCATGAAGATGGGCCTTGGAAGTTGGGCTGTTGCTATCTTGTGTGGAATTTTTGTCTTCCTGCTGTTTTTTATTTTAGACGAATGTGACACAAGTTGCCTTTTCGCTCTCATTGCCGCATC
    Consensus ATTGTGTTGCCATGAAGATGGGCCTTGGAAGTTGGGCTGTTGCTATCTTGTGTGGAATTTTTGTCTTCCTGCTGTTTTTTATTTTAGACGAATGTGACACAAGTTGCCTTTTCGCTCTCATTGCCGCATC

              5591 5600      5610      5620      5630      5640      5650      5660      5670      5680      5690      5700      5710      5720
              |--------+---------+---------+---------+---------+---------+---------+---------+---------+---------+---------+---------+---------|
contig006087  AGTGGTAGTATATTATATCCCAACAATAGTTTTACTTTTCATGTACACCAAAATTCTTGTTGTTGCACTGAGGCAGGCACGCAGCATCCATAATACAATTTCTCAGAACACAAAGTCTAAAGCAGTTTCC
  BurTAR.B032 AGTGGTAGTATATTATATCCCAACAATAGTTTTACTTTTCATGTACACCAAAATTCTTGTTGTTGCACTGAGGCAGGCACGCAGCATCCATAATACAATTTCTCAGAACACAAAGTCTAAAGCAGTTTCC
    Consensus AGTGGTAGTATATTATATCCCAACAATAGTTTTACTTTTCATGTACACCAAAATTCTTGTTGTTGCACTGAGGCAGGCACGCAGCATCCATAATACAATTTCTCAGAACACAAAGTCTAAAGCAGTTTCC

              5721 5730      5740      5750      5760      5770      5780      5790      5800      5810      5820      5830      5840      5850
              |--------+---------+---------+---------+---------+---------+---------+---------+---------+---------+---------+---------+---------|
contig006087  AGTACGGAGAGAAAGGCCACAAAGACATTAACCATAGTTATTGGAATATTTTTAATTTTTTGGGTTCCTCTTTTTCTTAGTTATTCATTTGTTCCTCTGGATAGTTTTATACTTTATGTGCTTCTTGAAC
  BurTAR.B032 AGTACGGAGAGAAAGGCCACAAAGACATTAACCATAGTTATTGGAATATTTTTAATTTTTTGGGTTCCTCTTTTTCTTAGTTATTCATTTGTTCCTCTGGATAGTTTTATACTTTATGTGCTTCTTGAAC
    Consensus AGTACGGAGAGAAAGGCCACAAAGACATTAACCATAGTTATTGGAATATTTTTAATTTTTTGGGTTCCTCTTTTTCTTAGTTATTCATTTGTTCCTCTGGATAGTTTTATACTTTATGTGCTTCTTGAAC

              5851 5860      5870      5880      5890      5900      5910      5920      5930      5940      5950      5960      5970      5980
              |--------+---------+---------+---------+---------+---------+---------+---------+---------+---------+---------+---------+---------|
contig006087  CATTTAACTGGTTTGCAATTTCTAACTCAATGCTCAATCCCTTTATTTATGCTTTCTTTTACACTTGGTTTAGAAGAGCTTTTAAAATGATAATTTCTGGAAAAATATTTCAAGGTGATGTTACTAACAT
  BurTAR.B032 CATTTAACTGGTTTGCAATTTCTAACTCAATGCTCAATCCCTTTATTTATGCTTTCTTTTACACTTGGTTTAGAAGAGCTTTTAAAATGATAATTTCTGGAAAAATATTTCAAGGTGATGTTACTAACAT
    Consensus CATTTAACTGGTTTGCAATTTCTAACTCAATGCTCAATCCCTTTATTTATGCTTTCTTTTACACTTGGTTTAGAAGAGCTTTTAAAATGATAATTTCTGGAAAAATATTTCAAGGTGATGTTACTAACAT

              5981 5990      6000      6010      6020      6030      6040      6050      6060      6070      6080      6090      6100      6110
              |--------+---------+---------+---------+---------+---------+---------+---------+---------+---------+---------+---------+---------|
contig006087  AAAACTACACTGACAGCTATATATATATTATGAATTATATCAATAAGAGTAAATATCACCACAGTTGATCTTCTCTATGTTGTTCATTACAGTTTCAACTATGTAAGTCAACTGTCACTATGCACAGACT
  BurTAR.B032 AAAACTACACTGA
    Consensus AAAACTACACTGA.....................................................................................................................
```

```
             1561 1570      1580      1590      1600      1610      1620      1630      1640      1650      1660      1670      1680      1690
             |---------+---------+---------+---------+---------+---------+---------+---------+---------+---------+---------+---------+---------|
contig039639 TGTCGAACACAGTTGCAGGTCTTCCCTTTCTCTCCAATGGACGGCTCTGGAGGTCCTCCCCTCTGCTTCCCCAACCTCAACTCCTCCTGCAGGCGGCTGCTGCGACCCACCTCCCAGGCCGCTCTTCTCT
 TilTAR.A001                                     ATGGACGGCTCTGGAGGTCCTCCCCTCTGCTTCCCCAACCTCAACTCCTCCTGCAGGCGGCTGCTGCGACCCACCTCCCAGGCCGCTCTTCTCT
   Consensus ....................................ATGGACGGCTCTGGAGGTCCTCCCCTCTGCTTCCCCAACCTCAACTCCTCCTGCAGGCGGCTGCTGCGACCCACCTCCCAGGCCGCTCTTCTCT

             1691 1700      1710      1720      1730      1740      1750      1760      1770      1780      1790      1800      1810      1820
             |---------+---------+---------+---------+---------+---------+---------+---------+---------+---------+---------+---------+---------|
contig039639 ACACCCTGATGGCTTCAGTCTCACTGCTCACTGTGGTGCTCAACCTGCTCGTGGTTGTCTCCATCTCCCACTTCAAGCAGCTCCACACTCCGACCAACACCCTGCTCCTGTCCCTGGCCATGTCCGACCT
 TilTAR.A001 ACACCCTGATGGCTTCAGTCTCACTGCTCACTGTGGTGCTCAACCTGCTCGTGGTTGTCTCCATCTCCCACTTCAAGCAGCTCCACACTCCGACCAACACCCTGCTCCTGTCCCTGGCCATGTCCGACCT
   Consensus ACACCCTGATGGCTTCAGTCTCACTGCTCACTGTGGTGCTCAACCTGCTCGTGGTTGTCTCCATCTCCCACTTCAAGCAGCTCCACACTCCGACCAACACCCTGCTCCTGTCCCTGGCCATGTCCGACCT

             1821 1830      1840      1850      1860      1870      1880      1890      1900      1910      1920      1930      1940      1950
             |---------+---------+---------+---------+---------+---------+---------+---------+---------+---------+---------+---------+---------|
contig039639 GCTGGTGGGGCTGCTGGTGATGCCCATCGAGGGCCTGCGCTACATCGAGACGTGCTGGCTGCTGGGGAGGCTGATGTGTGCTCTCAGTCCTTATTTGTCTTACTGCCTCATCTCTGTCTCTCTGGGCAGC
 TilTAR.A001 GCTGGTGGGGCTGCTGGTGATGCCCATCGAGGGCCTGCGCTACATCGAGACGTGCTGGCTGCTGGGGAGGCTGATGTGTGCTCTCAGTCCTTATTTGTCTTACTGCCTCATCTCTGTCTCTCTGGGCAGC
   Consensus GCTGGTGGGGCTGCTGGTGATGCCCATCGAGGGCCTGCGCTACATCGAGACGTGCTGGCTGCTGGGGAGGCTGATGTGTGCTCTCAGTCCTTATTTGTCTTACTGCCTCATCTCTGTCTCTCTGGGCAGC

             1951 1960      1970      1980      1990      2000      2010      2020      2030      2040      2050      2060      2070      2080
             |---------+---------+---------+---------+---------+---------+---------+---------+---------+---------+---------+---------+---------|
contig039639 ATGGTGCTCATATCTGTAGATCGCTATATAGCCATCTGTGACCCTCTGCTCTATTCCTCAAAGATCACAGTGAACAGAGTGAAGCTTTCAGTCTGTGTCTGCTGGGCCTGCTCTCTTCTCTACAATGGCT
 TilTAR.A001 ATGGTGCTCATATCTGTAGATCGCTATATAGCCATCTGTGACCCTCTGCTCTATTCCTCAAAGATCACAGTGAACAGAGTGAAGCTTTCAGTCTGTGTCTGCTGGGCCTGCTCTCTTCTCTACAATGGCT
   Consensus ATGGTGCTCATATCTGTAGATCGCTATATAGCCATCTGTGACCCTCTGCTCTATTCCTCAAAGATCACAGTGAACAGAGTGAAGCTTTCAGTCTGTGTCTGCTGGGCCTGCTCTCTTCTCTACAATGGCT

             2081 2090      2100      2110      2120      2130      2140      2150      2160      2170      2180      2190      2200      2210
             |---------+---------+---------+---------+---------+---------+---------+---------+---------+---------+---------+---------+---------|
contig039639 GCATTCTCATGGAACACTTAGGGTGGCCGGACAGGTTCAGCTCCTGTCACGGGGAGTGTGTTGTGTTCATCAGCCGAACTTCAGGAACAGTAGATTTCTTTTTGTCATTTGTCGGCCCCTGTGGCGTAAT
 TilTAR.A001 GCATTCTCATGGAACACTTAGGGTGGCCGGACAGGTTCAGCTCCTGTCACGGGGAGTGTGTTGTGTTCATCAGCCGAACTTCAGGAACAGTAGATTTCTTTTTGTCATTTGTCGGCCCCTGTGGCGTAAT
   Consensus GCATTCTCATGGAACACTTAGGGTGGCCGGACAGGTTCAGCTCCTGTCACGGGGAGTGTGTTGTGTTCATCAGCCGAACTTCAGGAACAGTAGATTTCTTTTTGTCATTTGTCGGCCCCTGTGGCGTAAT

             2211 2220      2230      2240      2250      2260      2270      2280      2290      2300      2310      2320      2330      2340
             |---------+---------+---------+---------+---------+---------+---------+---------+---------+---------+---------+---------+---------|
contig039639 GTTTGTTCTGTACATGAGGGTGTTTGTGGTTGCTGTTTCTCAGGTGCGTGCCATTCAGTCGCAGGTGGCTGTGAGAGCAGCTCCAGCTGCTAAAAAATCAGAGTTGAAGGCAGCCAGGACACTCGGGATT
 TilTAR.A001 GTTTGTTCTGTACATGAGGGTGTTTGTGGTTGCTGTTTCTCAGGTGCGTGCCATTCAGTCGCAGGTGGCTGTGAGAGCAGCTCCAGCTGCTAAAAAATCAGAGTTGAAGGCAGCCAGGACACTCGGGATT
   Consensus GTTTGTTCTGTACATGAGGGTGTTTGTGGTTGCTGTTTCTCAGGTGCGTGCCATTCAGTCGCAGGTGGCTGTGAGAGCAGCTCCAGCTGCTAAAAAATCAGAGTTGAAGGCAGCCAGGACACTCGGGATT

             2341 2350      2360      2370      2380      2390      2400      2410      2420      2430      2440      2450      2460      2470
             |---------+---------+---------+---------+---------+---------+---------+---------+---------+---------+---------+---------+---------|
contig039639 GTGATAGTTGTTTTTCTAATGTGCTCCTGCCCTTATTATTATCCTTCCTTTGCAGGTGACGACACTTCTATGAGCTTGCCTTATTACACCTTGCTGTTTTGGATGGTGCTGACAAACTCTTGTGTGAACC
 TilTAR.A001 GTGATAGTTGTTTTTCTAATGTGCTCCTGCCCTTATTATTATCCTTCCTTTGCAGGTGACGACACTTCTATGAGCTTGCCTTATTACACCTTGCTGTTTTGGATGGTGCTGACAAACTCTTGTGTGAACC
   Consensus GTGATAGTTGTTTTTCTAATGTGCTCCTGCCCTTATTATTATCCTTCCTTTGCAGGTGACGACACTTCTATGAGCTTGCCTTATTACACCTTGCTGTTTTGGATGGTGCTGACAAACTCTTGTGTGAACC

             2471 2480      2490      2500      2510      2520      2530      2540      2550      2560      2570      2580      2590      2600
             |---------+---------+---------+---------+---------+---------+---------+---------+---------+---------+---------+---------+---------|
contig039639 CTGTGATTTATGTACTGTTCTACCTCTGGTTTAGAAGAGCTATCAGATTCATCGTCACCCTCAGAATACTGCAGCCTCACTCCAGAGAGGTCAACATCCTGTAGGCAGCACTGAAACAAACCAGTCATTA
 TilTAR.A001 CTGTGATTTATGTACTGTTCTACCTCTGGTTTAGAAGAGCTATCAGATTCATCGTCACCCTCAGAATACTGCAGCCTCACTCCAGAGAGGTCAACATCCTGTAG
   Consensus CTGTGATTTATGTACTGTTCTACCTCTGGTTTAGAAGAGCTATCAGATTCATCGTCACCCTCAGAATACTGCAGCCTCACTCCAGAGAGGTCAACATCCTGTAG..........................

             [illegible]
```

```
              4551 4560      4570      4580      4590      4600      4610      4620      4630      4640      4650      4660      4670      4680
              |--------+---------+---------+---------+---------+---------+---------+---------+---------+---------+---------+---------+---------|
contig039640  TGGGCGTGTGTTTGAAGAAGATAAATGAAGACTCTCAAGCATAGTTGCAGGTCTTCCCTTTCTCCACGATGGACGGCTCTGGAGGTCCTCCCCTCTGCTTCCCCAACCTCAACTCCTCCTGCAGGCGGCT
TilTAR.A002                                                                        ATGGACGGCTCTGGAGGTCCTCCCCTCTGCTTCCCCAACCTCAACTCCTCCTGCAGGCGGCT
Consensus     .....................................................................ATGGACGGCTCTGGAGGTCCTCCCCTCTGCTTCCCCAACCTCAACTCCTCCTGCAGGCGGCT

              4681 4690      4700      4710      4720      4730      4740      4750      4760      4770      4780      4790      4800      4810
              |--------+---------+---------+---------+---------+---------+---------+---------+---------+---------+---------+---------+---------|
contig039640  GCTGCGACCCACCTCCCAGGCCGCTCTTCTCTACACCCTGCTGGCTTCAGTCTCACTGCTCACTGTGGTGCTCAACCTGCTCGTGGTCATCTCCATCTCCCACTTCAAGCAGCTCCACACCCCGACCAAT
TilTAR.A002   GCTGCGACCCACCTCCCAGGCCGCTCTTCTCTACACCCTGCTGGCTTCAGTCTCACTGCTCACTGTGGTGCTCAACCTGCTCGTGGTCATCTCCATCTCCCACTTCAAGCAGCTCCACACCCCGACCAAT
Consensus     GCTGCGACCCACCTCCCAGGCCGCTCTTCTCTACACCCTGCTGGCTTCAGTCTCACTGCTCACTGTGGTGCTCAACCTGCTCGTGGTCATCTCCATCTCCCACTTCAAGCAGCTCCACACCCCGACCAAT

              4811 4820      4830      4840      4850      4860      4870      4880      4890      4900      4910      4920      4930      4940
              |--------+---------+---------+---------+---------+---------+---------+---------+---------+---------+---------+---------+---------|
contig039640  GCCCTGCTCCTGTCCCTGGCCGTGTCCGACCTGCTTTTGGGGTTGCTGGTGATGCCCATTGAGGGCCTGCGCTACATCGAGACGTGCTGGCTGCTGGGGAGGCTGATGTGTGCTCTCAGTCCTTATTTGT
TilTAR.A002   GCCCTGCTCCTGTCCCTGGCCGTGTCCGACCTGCTTTTGGGGTTGCTGGTGATGCCCATTGAGGGCCTGCGCTACATCGAGACGTGCTGGCTGCTGGGGAGGCTGATGTGTGCTCTCAGTCCTTATTTGT
Consensus     GCCCTGCTCCTGTCCCTGGCCGTGTCCGACCTGCTTTTGGGGTTGCTGGTGATGCCCATTGAGGGCCTGCGCTACATCGAGACGTGCTGGCTGCTGGGGAGGCTGATGTGTGCTCTCAGTCCTTATTTGT

              4941 4950      4960      4970      4980      4990      5000      5010      5020      5030      5040      5050      5060      5070
              |--------+---------+---------+---------+---------+---------+---------+---------+---------+---------+---------+---------+---------|
contig039640  CTTACTGCTTCCTCTCTTTTTCTCTGGACAGCATGGTGCTCATATCTGTAGATCGCTATATAGCCATCTGTGACCCTCTGCTCTATTCCTCAAAGATCACAGTGAACAGAGTGAAGCTTTCAGTCTGTTT
TilTAR.A002   CTTACTGCTTCCTCTCTTTTTCTCTGGACAGCATGGTGCTCATATCTGTAGATCGCTATATAGCCATCTGTGACCCTCTGCTCTATTCCTCAAAGATCACAGTGAACAGAGTGAAGCTTTCAGTCTGTTT
Consensus     CTTACTGCTTCCTCTCTTTTTCTCTGGACAGCATGGTGCTCATATCTGTAGATCGCTATATAGCCATCTGTGACCCTCTGCTCTATTCCTCAAAGATCACAGTGAACAGAGTGAAGCTTTCAGTCTGTTT

              5071 5080      5090      5100      5110      5120      5130      5140      5150      5160      5170      5180      5190      5200
              |--------+---------+---------+---------+---------+---------+---------+---------+---------+---------+---------+---------+---------|
contig039640  CTGCTGGGTCTGCTCTCTTCTCTACAATGGCTGCATTCTCATGGAACACTTAGGGTCGCCGGACAGGTTCAGCTCCTGTCACGGGGAGTGTGTTGTGTTCATCAGCTACACTTCAGGCACAGTAGATCTC
TilTAR.A002   CTGCTGGGTCTGCTCTCTTCTCTACAATGGCTGCATTCTCATGGAACACTTAGGGTCGCCGGACAGGTTCAGCTCCTGTCACGGGGAGTGTGTTGTGTTCATCAGCTACACTTCAGGCACAGTAGATCTC
Consensus     CTGCTGGGTCTGCTCTCTTCTCTACAATGGCTGCATTCTCATGGAACACTTAGGGTCGCCGGACAGGTTCAGCTCCTGTCACGGGGAGTGTGTTGTGTTCATCAGCTACACTTCAGGCACAGTAGATCTC

              5201 5210      5220      5230      5240      5250      5260      5270      5280      5290      5300      5310      5320      5330
              |--------+---------+---------+---------+---------+---------+---------+---------+---------+---------+---------+---------+---------|
contig039640  TTTTTGTCATTTGTTGCCCCCTTTGCTGTAATGTTTGTTCTGTACATGAGGGTGTTTGTGGTTGCTGTTTCTCAGGTGCGTGCCATTCAGTCACAGGTGGCTGTGAGAGCAGCTCCAGCTGCTAAAACAT
TilTAR.A002   TTTTTGTCATTTGTTGCCCCCTTTGCTGTAATGTTTGTTCTGTACATGAGGGTGTTTGTGGTTGCTGTTTCTCAGGTGCGTGCCATTCAGTCACAGGTGGCTGTGAGAGCAGCTCCAGCTGCTAAAACAT
Consensus     TTTTTGTCATTTGTTGCCCCCTTTGCTGTAATGTTTGTTCTGTACATGAGGGTGTTTGTGGTTGCTGTTTCTCAGGTGCGTGCCATTCAGTCACAGGTGGCTGTGAGAGCAGCTCCAGCTGCTAAAACAT

              5331 5340      5350      5360      5370      5380      5390      5400      5410      5420      5430      5440      5450      5460
              |--------+---------+---------+---------+---------+---------+---------+---------+---------+---------+---------+---------+---------|
contig039640  CAGAGTTGAAGGCAGCCAGGACACTCGGGATTTTGATAGCTGTGTTTCTAATGTGCTTCTGCCCTTATTATTATCCTTCCTTTACAGGTGATGACACCTCTACAAGCTTGCCTTATTACGCTGTGCTGTT
TilTAR.A002   CAGAGTTGAAGGCAGCCAGGACACTCGGGATTTTGATAGCTGTGTTTCTAATGTGCTTCTGCCCTTATTATTATCCTTCCTTTACAGGTGATGACACCTCTACAAGCTTGCCTTATTACGCTGTGCTGTT
Consensus     CAGAGTTGAAGGCAGCCAGGACACTCGGGATTTTGATAGCTGTGTTTCTAATGTGCTTCTGCCCTTATTATTATCCTTCCTTTACAGGTGATGACACCTCTACAAGCTTGCCTTATTACGCTGTGCTGTT

              5461 5470      5480      5490      5500      5510      5520      5530      5540      5550      5560      5570      5580      5590
              |--------+---------+---------+---------+---------+---------+---------+---------+---------+---------+---------+---------+---------|
contig039640  TTGGATCATGCTAATAAATGCTTGTGTGAACCCTGTGATTTATGTTCTGTTTTACCCCTGGTTTAGAAGAGCTATCAGATTCATCGTCACCCTCAGAATACTGCAGCCTCACTCCAGAGAGGTCAACATC
TilTAR.A002   TTGGATCATGCTAATAAATGCTTGTGTGAACCCTGTGATTTATGTTCTGTTTTACCCCTGGTTTAGAAGAGCTATCAGATTCATCGTCACCCTCAGAATACTGCAGCCTCACTCCAGAGAGGTCAACATC
Consensus     TTGGATCATGCTAATAAATGCTTGTGTGAACCCTGTGATTTATGTTCTGTTTTACCCCTGGTTTAGAAGAGCTATCAGATTCATCGTCACCCTCAGAATACTGCAGCCTCACTCCAGAGAGGTCAACATC

              5591 5600      5610      5620      5630      5640      5650      5660      5670      5680      5690      5700      5710      5720
              |--------+---------+---------+---------+---------+---------+---------+---------+---------+---------+---------+---------+---------|
contig039640  CTGTAGGCAGCGCAATACAACTTAATCATTTTTCAACTTTCATGATAAATTTTCACCTTTAAATGTGGGTGTACATCTGCCCAGCATCACCTTTTGTTCAGTACATAATGAGCAGCAGCGCAGACCCGCG
TilTAR.A002   CTGTAG
Consensus     CTGTAG............................................................................................................................
```

```
             2861  2870          2880          2890          2900          2910          2920          2930          2940          2950          2960          2970          2980          2990
             |--------+---------+---------+---------+---------+---------+---------+---------+---------+---------+---------+---------+---------|
contig007518 CTTCAGTCAGAAGGCTGTCAGAGCTCAAGATCTTCTCCTCTCTTTGTTGCAATGAAAACCATTGACAGAGCTGAACTGTGCTTTTCACAACTCAACTCCTCCTGCAGAAAGACCATGCATCCTCGTTCGC
TilTARs.A019                                                    ATGAAAACCATTGACAGAGCTGAACTGTGCTTTTCACAACTCAACTCCTCCTGCAGAAAGACCATGCATCCTCGTTCGC
   Consensus ...................................................ATGAAAACCATTGACAGAGCTGAACTGTGCTTTTCACAACTCAACTCCTCCTGCAGAAAGACCATGCATCCTCGTTCGC

             2991  3000          3010          3020          3030          3040          3050          3060          3070          3080          3090          3100          3110          3120
             |--------+---------+---------+---------+---------+---------+---------+---------+---------+---------+---------+---------+---------|
contig007518 TTTCTGTTCTCTTTTATGTCATACTGTCCTCCATTTCTGTGCTCACTGTGGCTCTTAATCTACTGGTGATCATCTCTATTTCCCACTTCAAGTAATCAGATTTCCTTTTATCACTTGAAAAAAGGAAAGC
TilTARs.A019 TTTCTGTTCTCTTTTATGTCATACTGTCCTCCATTTCTGTGCTCACTGTGGCTCTTAATCTACTGGTGATCATCTCTATTTCCCACTTCA----------------------------------------
   Consensus TTTCTGTTCTCTTTTATGTCATACTGTCCTCCATTTCTGTGCTCACTGTGGCTCTTAATCTACTGGTGATCATCTCTATTTCCCACTTCA........................................

             3121  3130          3140          3150          3160          3170          3180          3190          3200          3210          3220          3230          3240          3250
             |--------+---------+---------+---------+---------+---------+---------+---------+---------+---------+---------+---------+---------|
contig007518 AAAGCCGCACTGTATTTGAATAAACCTATGCTTTTGTACGCTGAAATGACAAAAGATTTTGCTCTTTTACCTCTCCAGGCAGCTCCACACCCCGACCAACCTCCTCCTCTTCTCTCTGGCTGTCTCTGAT
TilTARs.A019 -----------------------------------------------------------------------------GGCAGCTCCACACCCCGACCAACCTCCTCCTCTTCTCTCTGGCTGTCTCTGAT
   Consensus .............................................................................GGCAGCTCCACACCCCGACCAACCTCCTCCTCTTCTCTCTGGCTGTCTCTGAT

             3251  3260          3270          3280          3290          3300          3310          3320          3330          3340          3350          3360          3370          3380
             |--------+---------+---------+---------+---------+---------+---------+---------+---------+---------+---------+---------+---------|
contig007518 TGTTTTGTGGGCCTTCTCACAGTCTTTCAAATACTGGTCATAGACGGCTGCTGGTATCTTGGTGAACAGTTGTGTATTTTGTATTATATTTTCGATTATGTTGTTACTAATGCCTCAATAGGAACCATGG
TilTARs.A019 TGTTTTGTGGGCCTTCTCACAGTCTTTCAAATACTGGTCATAGACGGCTGCTGGTATCTTGGTGAACAGTTGTGTATTTTGTATTATATTTTCGATTATGTTGTTACTAATGCCTCAATAGGAACCATGG
   Consensus TGTTTTGTGGGCCTTCTCACAGTCTTTCAAATACTGGTCATAGACGGCTGCTGGTATCTTGGTGAACAGTTGTGTATTTTGTATTATATTTTCGATTATGTTGTTACTAATGCCTCAATAGGAACCATGG

             3381  3390          3400          3410          3420          3430          3440          3450          3460          3470          3480          3490          3500          3510
             |--------+---------+---------+---------+---------+---------+---------+---------+---------+---------+---------+---------+---------|
contig007518 TGCTCATATCAGTTGACCGCTATGTGGCTATTTGCGACCCCCTTTATTACCCAAGCAAAGTCACTCTAAAAAGAGTAAGGACTTCTGTTTCATTATGTTGGATTTTTTCTCTCTTCTGTGTCTTTGTGCT
TilTARs.A019 TGCTCATATCAGTTGACCGCTATGTGGCTATTTGCGACCCCCTTTATTACCCAAGCAAAGTCACTCTAAAAAGAGTAAGGACTTCTGTTTCATTATGTTGGATTTTTTCTCTCTTCTGTGTCTTTGTGCT
   Consensus TGCTCATATCAGTTGACCGCTATGTGGCTATTTGCGACCCCCTTTATTACCCAAGCAAAGTCACTCTAAAAAGAGTAAGGACTTCTGTTTCATTATGTTGGATTTTTTCTCTCTTCTGTGTCTTTGTGCT

             3511  3520          3530          3540          3550          3560          3570          3580          3590          3600          3610          3620          3630          3640
             |--------+---------+---------+---------+---------+---------+---------+---------+---------+---------+---------+---------+---------|
contig007518 GTTGAAAGATAATCTGGGACAACCAGGTAGTTATAATTCCTGCTCTGGTGAGTGTGTGGTTCTTGTTTATTATGTTACTGGAATTGTAGATATTTTATTGTCATTTATTGGTCCTGTCATTGTTATCATA
TilTARs.A019 GTTGAAAGATAATCTGGGACAACCAGGTAGTTATAATTCCTGCTCTGGTGAGTGTGTGGTTCTTGTTTATTATGTTACTGGAATTGTAGATATTTTATTGTCATTTATTGGTCCTGTCATTGTTATCATA
   Consensus GTTGAAAGATAATCTGGGACAACCAGGTAGTTATAATTCCTGCTCTGGTGAGTGTGTGGTTCTTGTTTATTATGTTACTGGAATTGTAGATATTTTATTGTCATTTATTGGTCCTGTCATTGTTATCATA

             3641  3650          3660          3670          3680          3690          3700          3710          3720          3730          3740          3750          3760          3770
             |--------+---------+---------+---------+---------+---------+---------+---------+---------+---------+---------+---------+---------|
contig007518 GCTCTGTATCTGAAAGTATTTATGGTGGTTGTTACTCAGGCTCGTTCCATGAGGTCTCGTATTGAAGCTGTCGCTGTGCAGGGTTCTGTAACAGTAACTATTAAGAAATCTGAAATCAAAGCTGCCAGAG
TilTARs.A019 GCTCTGTATCTGAAAGTATTTATGGTGGTTGTTACTCAGGCTCGTTCCATGAGGTCTCGTATTGAAGCTGTCGCTGTGCAGGGTTCTGTAACAGTAACTATTAAGAAATCTGAAATCAAAGCTGCCAGAG
   Consensus GCTCTGTATCTGAAAGTATTTATGGTGGTTGTTACTCAGGCTCGTTCCATGAGGTCTCGTATTGAAGCTGTCGCTGTGCAGGGTTCTGTAACAGTAACTATTAAGAAATCTGAAATCAAAGCTGCCAGAG

             3771  3780          3790          3800          3810          3820          3830          3840          3850          3860          3870          3880          3890          3900
             |--------+---------+---------+---------+---------+---------+---------+---------+---------+---------+---------+---------+---------|
contig007518 TTCTCGGAATTGTTGTAGTTGTATTTCTAGCATGTCTTTTACCTTATTTTTGTATTGGACTTACAGGTGACAAGACCCTACTCAATGCTTCAACTGCGACTGTTTTTATAACTCTCTTCTATTTTAATTC
TilTARs.A019 TTCTCGGAATTGTTGTAGTTGTATTTCTAGCATGTCTTTTACCTTATTTTTGTATTGGACTTACAGGTGACAAGACCCTACTCAATGCTTCAACTGCGACTGTTTTTATAACTCTCTTCTATTTTAATTC
   Consensus TTCTCGGAATTGTTGTAGTTGTATTTCTAGCATGTCTTTTACCTTATTTTTGTATTGGACTTACAGGTGACAAGACCCTACTCAATGCTTCAACTGCGACTGTTTTTATAACTCTCTTCTATTTTAATTC

             3901  3910          3920          3930          3940          3950          3960          3970          3980          3990          4000          4010          4020          4030
             |--------+---------+---------+---------+---------+---------+---------+---------+---------+---------+---------+---------+---------|
contig007518 CTGTGTAAACCCTCTGGTTTATGCCTTTTTCTATCCCTGGTTCAGGAGAACTATTAAACTAATAGTAACACTTAAGATACTGAATTCTCGCTCCTGTAAGGCCAATTTACTAATTTCATGAAACGTTAAA
TilTARs.A019 CTGTGTAAACCCTCTGGTTTATGCCTTTTTCTATCCCTGGTTCAGGAGAACTATTAAACTAATAGTAACACTTAAGATACTGAATTCTCGCTCCTGTAAGGCCAATTTACTAATTTCATGA
   Consensus CTGTGTAAACCCTCTGGTTTATGCCTTTTTCTATCCCTGGTTCAGGAGAACTATTAAACTAATAGTAACACTTAAGATACTGAATTCTCGCTCCTGTAAGGCCAATTTACTAATTTCATGA.........

             4031  4040          4050          4060          4070          4080          4090          4100          4110          4120          4130          4140          4150          4160
             |--------+---------+---------+---------+---------+---------+---------+---------+---------+---------+---------+---------+---------|
contig007518 TAAGCCTACCCAACTTTTGATGTCTATTGTCAGCTTGCTTGTATTTCACATTAAATGCAATTTATTGATGTACTTGAACAAAATTTATACCATAAACTTAAACCGACAAAGATGTGTCTGAAAACATGGT
TilTARs.A019
   Consensus ..................................................................................................................................
```

```
             2211 2220      2230      2240      2250      2260      2270      2280      2290      2300      2310      2320      2330      2340
contig007512 CGCCTCACTGTGATGATGGAGGAAACTGAACTCTGCTTTCCAAAACTTCTTAATATCTCCTGCAGGAGGCCAAAGCGTCCTCACTTTGAGATCATGCTGACTTACATTCTGCTCTCCTTCATTTCTTTGC
TilTARs.A024             ATGATGGAGGAAACTGAACTCTGCTTTCCAAAACTTCTTAATATCTCCTGCAGGAGGCCAAAGCGTCCTCACTTTGAGATCATGCTGACTTACATTCTGCTCTCCTTCATTTCTTTGC
   Consensus ............ATGATGGAGGAAACTGAACTCTGCTTTCCAAAACTTCTTAATATCTCCTGCAGGAGGCCAAAGCGTCCTCACTTTGAGATCATGCTGACTTACATTCTGCTCTCCTTCATTTCTTTGC

             2341 2350      2360      2370      2380      2390      2400      2410      2420      2430      2440      2450      2460      2470
contig007512 TTACTGTGATTCTTAACCTGCTGGTCATTATCTCCATCTCACACTTCAGGTATGAAAATGTTTCATTATCTCCAAATATTGAATAATTATTTTAATAGTTTAATTAATTGTGCTGTGTAGCAACATAGCA
TilTARs.A024 TTACTGTGATTCTTAACCTGCTGGTCATTATCTCCATCTCACACTTCAGG--------------------------------------------------------------------------------
   Consensus TTACTGTGATTCTTAACCTGCTGGTCATTATCTCCATCTCACACTTCAGG................................................................................

             2471 2480      2490      2500      2510      2520      2530      2540      2550      2560      2570      2580      2590      2600
contig007512 AATAGTAATTAAAACCATAACATGATCTGTACCTACAAGTAAGAGTAGACCTTTTTTTTAATGAAGTGCATTTCTATTTCAGTTTTTGTTGTATGTGCATCCACATACATTTTGGATGCAGGAGACTAAA
TilTARs.A024 ----------------------------------------------------------------------------------------------------------------------------------
   Consensus ..................................................................................................................................

             2601 2610      2620      2630      2640      2650      2660      2670      2680      2690      2700      2710      2720      2730
contig007512 TATGATGCACCTCTTTCTCCCCAGGCAGCTCCACACCCCCACCAACTTCCTCCTTCTCTCTCTGGCTGTCGCTGATTTCTTTGTAGGTCTCCTTATGTTCTTTCAAATAGTGCTCATCGATGGATGCTGG
TilTARs.A024 -------------------------CAGCTCCACACCCCCACCAACTTCCTCCTTCTCTCTCTGGCTGTCGCTGATTTCTTTGTAGGTCTCCTTATGTTCTTTCAAATAGTGCTCATCGATGGATGCTGG
   Consensus .........................CAGCTCCACACCCCCACCAACTTCCTCCTTCTCTCTCTGGCTGTCGCTGATTTCTTTGTAGGTCTCCTTATGTTCTTTCAAATAGTGCTCATCGATGGATGCTGG

             2731 2740      2750      2760      2770      2780      2790      2800      2810      2820      2830      2840      2850      2860
contig007512 TTCCTCGGTGACATCATGTGCACTCTGTATCAGTATCTAGCATTCATTATTACTTCAGCCTCAATAGGAACCATGGTGATCATATCTGCTGATAGGTATTTGGCTATTTGTTACCCTCTGCATTACTCCA
TilTARs.A024 TTCCTCGGTGACATCATGTGCACTCTGTATCAGTATCTAGCATTCATTATTACTTCAGCCTCAATAGGAACCATGGTGATCATATCTGCTGATAGGTATTTGGCTATTTGTTACCCTCTGCATTACTCCA
   Consensus TTCCTCGGTGACATCATGTGCACTCTGTATCAGTATCTAGCATTCATTATTACTTCAGCCTCAATAGGAACCATGGTGATCATATCTGCTGATAGGTATTTGGCTATTTGTTACCCTCTGCATTACTCCA

             2861 2870      2880      2890      2900      2910      2920      2930      2940      2950      2960      2970      2980      2990
contig007512 CCAAAATCACACAACAAAGAGTTAAAATCTGTATATGTTTATGTTGGGTTTTTTCTGTGATCTTTCAGAGTTTGATTGTGAAGGATAACTTGAAACAACCAGGAAAGTATAACTCCTGCATTGGAGAGTG
TilTARs.A024 CCAAAATCACACAACAAAGAGTTAAAATCTGTATATGTTTATGTTGGGTTTTTTCTGTGATCTTTCAGAGTTTGATTGTGAAGGATAACTTGAAACAACCAGGAAAGTATAACTCCTGCATTGGAGAGTG
   Consensus CCAAAATCACACAACAAAGAGTTAAAATCTGTATATGTTTATGTTGGGTTTTTTCTGTGATCTTTCAGAGTTTGATTGTGAAGGATAACTTGAAACAACCAGGAAAGTATAACTCCTGCATTGGAGAGTG

             2991 3000      3010      3020      3030      3040      3050      3060      3070      3080      3090      3100      3110      3120
contig007512 TGTCTTTGTTGTTAACTACATTGCTGGGCTTTTTGATCTTTTGTTTTCCTTCATTGTTCCCATTACTGTGATTATAGTTCTGTATCTGAGAGTGTTTGCGGTGGCTGTGTCTCAGGCTCATGCCATGAGG
TilTARs.A024 TGTCTTTGTTGTTAACTACATTGCTGGGCTTTTTGATCTTTTGTTTTCCTTCATTGTTCCCATTACTGTGATTATAGTTCTGTATCTGAGAGTGTTTGCGGTGGCTGTGTCTCAGGCTCATGCCATGAGG
   Consensus TGTCTTTGTTGTTAACTACATTGCTGGGCTTTTTGATCTTTTGTTTTCCTTCATTGTTCCCATTACTGTGATTATAGTTCTGTATCTGAGAGTGTTTGCGGTGGCTGTGTCTCAGGCTCATGCCATGAGG

             3121 3130      3140      3150      3160      3170      3180      3190      3200      3210      3220      3230      3240      3250
contig007512 TGTCAACATGCAGTCACTCACCAGCGAGCAGTTACAGTAACTGTTACGAAATCGGAGCTGAAAGCAGCCCGTACTCTTGGTGTTGTTGTAGTTGTGTTTCTTATATGTATGTGCCCATATTACTGCGTTG
TilTARs.A024 TGTCAACATGCAGTCACTCACCAGCGAGCAGTTACAGTAACTGTTACGAAATCGGAGCTGAAAGCAGCCCGTACTCTTGGTGTTGTTGTAGTTGTGTTTCTTATATGTATGTGCCCATATTACTGCGTTG
   Consensus TGTCAACATGCAGTCACTCACCAGCGAGCAGTTACAGTAACTGTTACGAAATCGGAGCTGAAAGCAGCCCGTACTCTTGGTGTTGTTGTAGTTGTGTTTCTTATATGTATGTGCCCATATTACTGCGTTG

             3251 3260      3270      3280      3290      3300      3310      3320      3330      3340      3350      3360      3370      3380
contig007512 CTCTCACAGGCCAAGATAACTTCCTAAATGCTTCATCTGCTGCCTTTGTAATATGTTTGGTGTATTTTAACTCTTGCGTAAACCCTATCATTTATGTCTTTTTTTATCCCTGGTTCAGAAAGTCAATCAA
TilTARs.A024 CTCTCACAGGCCAAGATAACTTCCTAAATGCTTCATCTGCTGCCTTTGTAATATGTTTGGTGTATTTTAACTCTTGCGTAAACCCTATCATTTATGTCTTTTTTTATCCCTGGTTCAGAAAGTCAATCAA
   Consensus CTCTCACAGGCCAAGATAACTTCCTAAATGCTTCATCTGCTGCCTTTGTAATATGTTTGGTGTATTTTAACTCTTGCGTAAACCCTATCATTTATGTCTTTTTTTATCCCTGGTTCAGAAAGTCAATCAA

             3381 3390      3400      3410      3420      3430      3440      3450      3460      3470      3480      3490      3500      3510
contig007512 ACTTATTGCTACTCTTCAAATACTGCAGCCTGACTCCCATGAGACTAACATGCATTAAGAACACTTTTTAAAACCGTACCCATAAACAGGACAATGTACTTAGTCACACTGAAGACTTGTTGAATGACAG
TilTARs.A024 ACTTATTGCTACTCTTCAAATACTGCAGCCTGACTCCCATGAGACTAACATGCATTAA
   Consensus ACTTATTGCTACTCTTCAAATACTGCAGCCTGACTCCCATGAGACTAACATGCATTAA........................................................................
```

```
            1001110020     10030     10040     10050     10060     10070     10080     10090     10100     10110     10120     10130     10140
            |--------+---------+---------+---------+---------+---------+---------+---------+---------+---------+---------+---------+---------|
contig007512 ATCTCTTCCTTTTTACTAGTGATGGAAATGTTTGAAGAAACTGAACTCTGCTTTCCACAGCTTCTTAACTTCTCCTGCAGGAAGCCCATGCAACCTCACTCAGTATCTATACTCATTTATATTGCATTGT
TilTARs.A025                      ATGGAAATGTTTGAAGAAACTGAACTCTGCTTTCCACAGCTTCTTAACTTCTCCTGCAGGAAGCCCATGCAACCTCACTCAGTATCTATACTCATTTATATTGCATTGT
   Consensus .....................ATGGAAATGTTTGAAGAAACTGAACTCTGCTTTCCACAGCTTCTTAACTTCTCCTGCAGGAAGCCCATGCAACCTCACTCAGTATCTATACTCATTTATATTGCATTGT

            1014110150     10160     10170     10180     10190     10200     10210     10220     10230     10240     10250     10260     10270
            |--------+---------+---------+---------+---------+---------+---------+---------+---------+---------+---------+---------+---------|
contig007512 CCTCCATCTCTTTACTGACTGTGACTCTTAATCTGCTGGTCATCATCTCCATCTCACATTTCAAGTAATAATTTTGTCATAGATAAGGGTACCAGGTTTTGTGGATCAGTAAAATGAAGATAACATATTA
TilTARs.A025 CCTCCATCTCTTTACTGACTGTGACTCTTAATCTGCTGGTCATCATCTCCATCTCACATTTCAAG-----------------------------------------------------------------
   Consensus CCTCCATCTCTTTACTGACTGTGACTCTTAATCTGCTGGTCATCATCTCCATCTCACATTTCAAG.................................................................

            1027110280     10290     10300     10310     10320     10330     10340     10350     10360     10370     10380     10390     10400
            |--------+---------+---------+---------+---------+---------+---------+---------+---------+---------+---------+---------+---------|
contig007512 GAGTATAAGTGAAATGTGTGTCGGTAGTAATGTCTGTTTTTGTCTTACACATATATCTGTATAAAATTGTACTTGTTGCTACAAATAATAATAATAATAACTGTGTCGCTGTCTTTTGTCTTCAGGAAGC
TilTARs.A025 ------------------------------------------------------------------------------------------------------------------------------AAGC
   Consensus ..............................................................................................................................AAGC

            1040110410     10420     10430     10440     10450     10460     10470     10480     10490     10500     10510     10520     10530
            |--------+---------+---------+---------+---------+---------+---------+---------+---------+---------+---------+---------+---------|
contig007512 TGCACACCCCCACCAATCTCCTCCTCCTCTCTCTGGCTGTCTCTGATTGCCTGGTGGGTCTCCTGATGTTATTTCAAATTATGATTATAGATGGTTGCTGGTTCCTTGGTGAATTCATGTGTAGTATGTA
TilTARs.A025 TGCACACCCCCACCAATCTCCTCCTCCTCTCTCTGGCTGTCTCTGATTGCCTGGTGGGTCTCCTGATGTTATTTCAAATTATGATTATAGATGGTTGCTGGTTCCTTGGTGAATTCATGTGTAGTATGTA
   Consensus TGCACACCCCCACCAATCTCCTCCTCCTCTCTCTGGCTGTCTCTGATTGCCTGGTGGGTCTCCTGATGTTATTTCAAATTATGATTATAGATGGTTGCTGGTTCCTTGGTGAATTCATGTGTAGTATGTA

            1053110540     10550     10560     10570     10580     10590     10600     10610     10620     10630     10640     10650     10660
            |--------+---------+---------+---------+---------+---------+---------+---------+---------+---------+---------+---------+---------|
contig007512 TTTTCTCTTGGACTACATTATTACCTCTGCCTCAGTAGGAACCATGGTTCTCATATCAATTGATCGTTATGTGGCCATTTGTTACCCTCTCCATTACTCTACCAAAGTCACCCCAAAAAGAACAAAAGCA
TilTARs.A025 TTTTCTCTTGGACTACATTATTACCTCTGCCTCAGTAGGAACCATGGTTCTCATATCAATTGATCGTTATGTGGCCATTTGTTACCCTCTCCATTACTCTACCAAAGTCACCCCAAAAAGAACAAAAGCA
   Consensus TTTTCTCTTGGACTACATTATTACCTCTGCCTCAGTAGGAACCATGGTTCTCATATCAATTGATCGTTATGTGGCCATTTGTTACCCTCTCCATTACTCTACCAAAGTCACCCCAAAAAGAACAAAAGCA

            1066110670     10680     10690     10700     10710     10720     10730     10740     10750     10760     10770     10780     10790
            |--------+---------+---------+---------+---------+---------+---------+---------+---------+---------+---------+---------+---------|
contig007512 TGTGTTTATCTGTGTTGGATTTGTTCTTCAGTCTTCCAGTGTCTTGTGCTAAAAGACAACCTGGTGCAACCTGGCAGGTATAATTCCTGCTATGGGGAGTGTGTGGTTGTTGTTGGCCATGCTTTTGGAG
TilTARs.A025 TGTGTTTATCTGTGTTGGATTTGTTCTTCAGTCTTCCAGTGTCTTGTGCTAAAAGACAACCTGGTGCAACCTGGCAGGTATAATTCCTGCTATGGGGAGTGTGTGGTTGTTGTTGGCCATGCTTTTGGAG
   Consensus TGTGTTTATCTGTGTTGGATTTGTTCTTCAGTCTTCCAGTGTCTTGTGCTAAAAGACAACCTGGTGCAACCTGGCAGGTATAATTCCTGCTATGGGGAGTGTGTGGTTGTTGTTGGCCATGCTTTTGGAG

            1079110800     10810     10820     10830     10840     10850     10860     10870     10880     10890     10900     10910     10920
            |--------+---------+---------+---------+---------+---------+---------+---------+---------+---------+---------+---------+---------|
contig007512 TTGCAGATCTATTGTTGTCCTTCATTGGTCCTGTCATTGTGATTGTAGTTCTTTATCTAAACGTATTTGTTGTGGCTATGACTCAAGCTCGTGCCCTGCGGTCACATATTGCAGCTCTGACACACGAGGG
TilTARs.A025 TTGCAGATCTATTGTTGTCCTTCATTGGTCCTGTCATTGTGATTGTAGTTCTTTATCTAAACGTATTTGTTGTGGCTATGACTCAAGCTCGTGCCCTGCGGTCACATATTGCAGCTCTGACACACGAGGG
   Consensus TTGCAGATCTATTGTTGTCCTTCATTGGTCCTGTCATTGTGATTGTAGTTCTTTATCTAAACGTATTTGTTGTGGCTATGACTCAAGCTCGTGCCCTGCGGTCACATATTGCAGCTCTGACACACGAGGG

            1092110930     10940     10950     10960     10970     10980     10990     11000     11010     11020     11030     11040     11050
            |--------+---------+---------+---------+---------+---------+---------+---------+---------+---------+---------+---------+---------|
contig007512 GTCAGTAAGTACAAATGTTAAGAAATCTGAAATGAAAGCAGTGAGGACCATCAGTGTTGTTATCATTGTGTTTCTAATATGTCTCTGCCCATATTTTTGTGTCACACTCTCAGGTCAAGATGCCATGCTC
TilTARs.A025 GTCAGTAAGTACAAATGTTAAGAAATCTGAAATGAAAGCAGTGAGGACCATCAGTGTTGTTATCATTGTGTTTCTAATATGTCTCTGCCCATATTTTTGTGTCACACTCTCAGGTCAAGATGCCATGCTC
   Consensus GTCAGTAAGTACAAATGTTAAGAAATCTGAAATGAAAGCAGTGAGGACCATCAGTGTTGTTATCATTGTGTTTCTAATATGTCTCTGCCCATATTTTTGTGTCACACTCTCAGGTCAAGATGCCATGCTC

            1105111060     11070     11080     11090     11100     11110     11120     11130     11140     11150     11160     11170     11180
            |--------+---------+---------+---------+---------+---------+---------+---------+---------+---------+---------+---------+---------|
contig007512 AGTGCTTCATCTGTTGCATTTGTAATGTGTTTGTTCTATTTAAACTCCTGTCTAAACCCTCTAATTTATGCTTTATTTTACCCCTGGTTCAGAAAATCTGTTAAACAAATTGTTACACTTAAAATATTGA
TilTARs.A025 AGTGCTTCATCTGTTGCATTTGTAATGTGTTTGTTCTATTTAAACTCCTGTCTAAACCCTCTAATTTATGCTTTATTTTACCCCTGGTTCAGAAAATCTGTTAAACAAATTGTTACACTTAAAATATTGA
   Consensus AGTGCTTCATCTGTTGCATTTGTAATGTGTTTGTTCTATTTAAACTCCTGTCTAAACCCTCTAATTTATGCTTTATTTTACCCCTGGTTCAGAAAATCTGTTAAACAAATTGTTACACTTAAAATATTGA

            1118111190     11200     11210     11220     11230     11240     11250     11260     11270     11280     11290     11300     11310
            |--------+---------+---------+---------+---------+---------+---------+---------+---------+---------+---------+---------+---------|
contig007512 AGTCTGGCTCCTGTGACACCAATATCATGTAAACAGAATGAAAGCACTCACATACAAACAGAATGTTTACCAACTAAAAAAAGGGAGAGTCAATTCAAACATACGCTTGGTGTAACTGAAATATGTAATG
TilTARs.A025 AGTCTGGCTCCTGTGACACCAATATCATGTAA
   Consensus AGTCTGGCTCCTGTGACACCAATATCATGTAA..................................................................................................
```

```
             3381 3390      3400      3410      3420      3430      3440      3450      3460      3470      3480      3490      3500      3510
contig007524 GAAAATAAATCAGGTACACAACAAAGACCTTCAGGCAGAAAGCTGGAACCTCCTTTTTCTAATGATGAAAACCTTTGAAGAAACTGAACTCTGCTTTCCACAACTCCTCAACTCCTCCTGCAGGAAGACC
TilTARs.A026                                                                 ATGAAAACCTTTGAAGAAACTGAACTCTGCTTTCCACAACTCCTCAACTCCTCCTGCAGGAAGACC
   Consensus ................................................................ATGAAAACCTTTGAAGAAACTGAACTCTGCTTTCCACAACTCCTCAACTCCTCCTGCAGGAAGACC

             3511 3520      3530      3540      3550      3560      3570      3580      3590      3600      3610      3620      3630      3640
contig007524 ATGCGTCCTTATACATTATCTATACTTATTTATATTACCCTGTCCTCCATTTCGTTGATCACTGTGACTCTTAATCTGCTGGTCATCATCTCCATCTCACATTTCGAGTAATAATTTTGTTAGACATATT
TilTARs.A026 ATGCGTCCTTATACATTATCTATACTTATTTATATTACCCTGTCCTCCATTTCGTTGATCACTGTGACTCTTAATCTGCTGGTCATCATCTCCATCTCACATTTCGAG----------------------
   Consensus ATGCGTCCTTATACATTATCTATACTTATTTATATTACCCTGTCCTCCATTTCGTTGATCACTGTGACTCTTAATCTGCTGGTCATCATCTCCATCTCACATTTCGAG......................

             3641 3650      3660      3670      3680      3690      3700      3710      3720      3730      3740      3750      3760      3770
contig007524 CAGACAGTAAGATTTTATGGATAAAAGATGAAAATGATATGATATCCTATCTGAGTGAGTCTGAGTGAAATGCTTTTTCCTTTTATCTGTATAAAATTGTACTTGTTGCTACAAATAATAATAATAATAA
TilTARs.A026 ----------------------------------------------------------------------------------------------------------------------------------
   Consensus ..................................................................................................................................

             3771 3780      3790      3800      3810      3820      3830      3840      3850      3860      3870      3880      3890      3900
contig007524 CTGTGTCGCTGTCTTTTGTCTTCAGGAAGCTGCACACCCCCACCAATCTCCTCCTCCTCTCTCTGGCTGTCTCTGATTGCCTGGTGGGTCTCCTGATGTTATTTCAAATTATGATTATAGATGGTTGCTG
TilTARs.A026 --------------------------AAGCTGCACACCCCCACCAATCTCCTCCTCCTCTCTCTGGCTGTCTCTGATTGCCTGGTGGGTCTCCTGATGTTATTTCAAATTATGATTATAGATGGTTGCTG
   Consensus ..........................AAGCTGCACACCCCCACCAATCTCCTCCTCCTCTCTCTGGCTGTCTCTGATTGCCTGGTGGGTCTCCTGATGTTATTTCAAATTATGATTATAGATGGTTGCTG

             3901 3910      3920      3930      3940      3950      3960      3970      3980      3990      4000      4010      4020      4030
contig007524 GTTTCTTGGTGAATTCATGTGTAGTATGTATTTTCTCTTGGACTACATTATTACCTCTGCATCAATAGGAACCATGGTTCTCATATCAATTGACCGTTATGTGGCTATTTGTTACCCTCTCCATTACTCT
TilTARs.A026 GTTTCTTGGTGAATTCATGTGTAGTATGTATTTTCTCTTGGACTACATTATTACCTCTGCATCAATAGGAACCATGGTTCTCATATCAATTGACCGTTATGTGGCTATTTGTTACCCTCTCCATTACTCT
   Consensus GTTTCTTGGTGAATTCATGTGTAGTATGTATTTTCTCTTGGACTACATTATTACCTCTGCATCAATAGGAACCATGGTTCTCATATCAATTGACCGTTATGTGGCTATTTGTTACCCTCTCCATTACTCT

             4031 4040      4050      4060      4070      4080      4090      4100      4110      4120      4130      4140      4150      4160
contig007524 ACCAAAGTCACCCCAAAAAGAACAAAAGCATGTGTTTATCTGTGTTGGATTTGTTCTTCAGTCTTCCAGTGTCTTGTGCTAAAAGACAACCTGGTGCAACCTGGCAGGTATAATTCCTGCTATGGGGAGT
TilTARs.A026 ACCAAAGTCACCCCAAAAAGAACAAAAGCATGTGTTTATCTGTGTTGGATTTGTTCTTCAGTCTTCCAGTGTCTTGTGCTAAAAGACAACCTGGTGCAACCTGGCAGGTATAATTCCTGCTATGGGGAGT
   Consensus ACCAAAGTCACCCCAAAAAGAACAAAAGCATGTGTTTATCTGTGTTGGATTTGTTCTTCAGTCTTCCAGTGTCTTGTGCTAAAAGACAACCTGGTGCAACCTGGCAGGTATAATTCCTGCTATGGGGAGT

             4161 4170      4180      4190      4200      4210      4220      4230      4240      4250      4260      4270      4280      4290
contig007524 GTGTGGTTGTTGTTGGCCATGCTTTTGGAGTTGCAGATCTATTGTTGTCCTTCATTGGTCCTGTCATTGTGATTGTAGTTCTTTATCTAAACGTATTTGTTGTGGCTATGACTCAAGCTCGTGCCCTGCG
TilTARs.A026 GTGTGGTTGTTGTTGGCCATGCTTTTGGAGTTGCAGATCTATTGTTGTCCTTCATTGGTCCTGTCATTGTGATTGTAGTTCTTTATCTAAACGTATTTGTTGTGGCTATGACTCAAGCTCGTGCCCTGCG
   Consensus GTGTGGTTGTTGTTGGCCATGCTTTTGGAGTTGCAGATCTATTGTTGTCCTTCATTGGTCCTGTCATTGTGATTGTAGTTCTTTATCTAAACGTATTTGTTGTGGCTATGACTCAAGCTCGTGCCCTGCG

             4291 4300      4310      4320      4330      4340      4350      4360      4370      4380      4390      4400      4410      4420
contig007524 GTCACATATTGCAGCTCTGACACACGAGGGGTCAGTAAGTACAAATGTTAAGAAATCTGAAATGAAAGCAGTGAGGACCATCAGTGTTGTTATCATTGTGTTTCTAATATGTCTCTGCCCATATTTTTGT
TilTARs.A026 GTCACATATTGCAGCTCTGACACACGAGGGGTCAGTAAGTACAAATGTTAAGAAATCTGAAATGAAAGCAGTGAGGACCATCAGTGTTGTTATCATTGTGTTTCTAATATGTCTCTGCCCATATTTTTGT
   Consensus GTCACATATTGCAGCTCTGACACACGAGGGGTCAGTAAGTACAAATGTTAAGAAATCTGAAATGAAAGCAGTGAGGACCATCAGTGTTGTTATCATTGTGTTTCTAATATGTCTCTGCCCATATTTTTGT

             4421 4430      4440      4450      4460      4470      4480      4490      4500      4510      4520      4530      4540      4550
contig007524 GTCACACTCTCAGGTCAAGATGCCATGCTCAGTGCTTCATCTGTTGCATTTGTAATGTGTTTGTTCTATTTAAACTCCTGTCTAAACCCTCTAATTTATGCTTTATTTTACCCCTGGTTCAGAAAATCTG
TilTARs.A026 GTCACACTCTCAGGTCAAGATGCCATGCTCAGTGCTTCATCTGTTGCATTTGTAATGTGTTTGTTCTATTTAAACTCCTGTCTAAACCCTCTAATTTATGCTTTATTTTACCCCTGGTTCAGAAAATCTG
   Consensus GTCACACTCTCAGGTCAAGATGCCATGCTCAGTGCTTCATCTGTTGCATTTGTAATGTGTTTGTTCTATTTAAACTCCTGTCTAAACCCTCTAATTTATGCTTTATTTTACCCCTGGTTCAGAAAATCTG

             4551 4560      4570      4580      4590      4600      4610      4620      4630      4640      4650      4660      4670      4680
contig007524 TTAAACAAATTGTTACACTTAAAATATTGAAGTCTGGCTCCTGTGACACCAATATCATGTAAACAGAATGACTTGCAATCGTCATGTATGAGTGCTGTGTCCTATTATTTATTTTATTCATAACTATAAA
TilTARs.A026 TTAAACAAATTGTTACACTTAAAATATTGAAGTCTGGCTCCTGTGACACCAATATCATGTAA
   Consensus TTAAACAAATTGTTACACTTAAAATATTGAAGTCTGGCTCCTGTGACACCAATATCATGTAA....................................................................
```

```
             2731  2740       2750       2760       2770       2780       2790       2800       2810       2820       2830       2840       2850       2860
             |--------+---------+---------+---------+---------+---------+---------+---------+---------+---------+---------+---------+---------|
contig022324 GGATATCATTCTCCAGGGAGCCCAAGAGTGGATGGGAGGTATGGTAGAAGGAGATAAATTTGCTGTATGACAGAAAGTTCAGTTCAGACAGCTGCAGGAACCTGACGATGGAAATCCAGATGCATCTAGA
TilTARs.A027                                                                                                            ATGGAAATCCAGATGCATCTAGA
   Consensus ...........................................................................................................ATGGAAATCCAGATGCATCTAGA

             2861  2870       2880       2890       2900       2910       2920       2930       2940       2950       2960       2970       2980       2990
             |--------+---------+---------+---------+---------+---------+---------+---------+---------+---------+---------+---------+---------|
contig022324 GGCAGAGTTCTGTTTTCCAGAGCTACTGAACAGTTCCTGCAGGAAGCCGACACTTCACTGGTCCAAAACGGTGCTCCTGAACGTTGGGCTTTCGTTCATCTCTGTGATCACTGCTGCTCTTAATCTGCTC
TilTARs.A027 GGCAGAGTTCTGTTTTCCAGAGCTACTGAACAGTTCCTGCAGGAAGCCGACACTTCACTGGTCCAAAACGGTGCTCCTGAACGTTGGGCTTTCGTTCATCTCTGTGATCACTGCTGCTCTTAATCTGCTC
   Consensus GGCAGAGTTCTGTTTTCCAGAGCTACTGAACAGTTCCTGCAGGAAGCCGACACTTCACTGGTCCAAAACGGTGCTCCTGAACGTTGGGCTTTCGTTCATCTCTGTGATCACTGCTGCTCTTAATCTGCTC

             2991  3000       3010       3020       3030       3040       3050       3060       3070       3080       3090       3100       3110       3120
             |--------+---------+---------+---------+---------+---------+---------+---------+---------+---------+---------+---------+---------|
contig022324 ATCATCATCTCAGTCTCCCACTTCAGGCAGAGATTAACCTCTCAGATGAAACTAAAGTTCAACTTTTAGAAAACAAAGTTACATTGACATCATATTCTTCAGTAATGTTTGAATTATGTCAAGATGAATG
TilTARs.A027 ATCATCATCTCAGTCTCCCACTTCAGGCAG----------------------------------------------------------------------------------------------------
   Consensus ATCATCATCTCAGTCTCCCACTTCAGGCAG....................................................................................................

             3121  3130       3140       3150       3160       3170       3180       3190       3200       3210       3220       3230       3240       3250
             |--------+---------+---------+---------+---------+---------+---------+---------+---------+---------+---------+---------+---------|
contig022324 TCCAGTGTGTACTTAATCATAACTGTATAGTTGTATTTACTTAATTGCACAATGCCTCAGCAATGGAACATCTTCACTGAAGACATTTGACATGATTAATGTGTAATTTATAATTTTCTGTCTCCCTGCA
TilTARs.A027 ----------------------------------------------------------------------------------------------------------------------------------
   Consensus ..................................................................................................................................

             3251  3260       3270       3280       3290       3300       3310       3320       3330       3340       3350       3360       3370       3380
             |--------+---------+---------+---------+---------+---------+---------+---------+---------+---------+---------+---------+---------|
contig022324 GGCAGCTCCACACTCCCAGTAACATCCTCCTACTCTCTCTGGCTGTCTCTGACTTTTTTGTGGGTTTTCTGTTGATGCCGGTAGAAATCTTCCGAAGCACGGCCTGCTGGGTATTTGGTGATCTCATGTG
TilTARs.A027 -----CTCCACACTCCCAGTAACATCCTCCTACTCTCTCTGGCTGTCTCTGACTTTTTTGTGGGTTTTCTGTTGATGCCGGTAGAAATCTTCCGAAGCACGGCCTGCTGGGTATTTGGTGATCTCATGTG
   Consensus .....CTCCACACTCCCAGTAACATCCTCCTACTCTCTCTGGCTGTCTCTGACTTTTTTGTGGGTTTTCTGTTGATGCCGGTAGAAATCTTCCGAAGCACGGCCTGCTGGGTATTTGGTGATCTCATGTG

             3381  3390       3400       3410       3420       3430       3440       3450       3460       3470       3480       3490       3500       3510
             |--------+---------+---------+---------+---------+---------+---------+---------+---------+---------+---------+---------+---------|
contig022324 CTCACTTTATATTTATCTGAGCGGCATTCTTATGAACGCTTCTTTTGAAATTATTGTTTTTATATCAGTTGACCGCTATGTGGCTATCTGTGACCCTCTGCATTACCCCACCAGAATCACTGTGGCAAGA
TilTARs.A027 CTCACTTTATATTTATCTGAGCGGCATTCTTATGAACGCTTCTTTTGAAATTATTGTTTTTATATCAGTTGACCGCTATGTGGCTATCTGTGACCCTCTGCATTACCCCACCAGAATCACTGTGGCAAGA
   Consensus CTCACTTTATATTTATCTGAGCGGCATTCTTATGAACGCTTCTTTTGAAATTATTGTTTTTATATCAGTTGACCGCTATGTGGCTATCTGTGACCCTCTGCATTACCCCACCAGAATCACTGTGGCAAGA

             3511  3520       3530       3540       3550       3560       3570       3580       3590       3600       3610       3620       3630       3640
             |--------+---------+---------+---------+---------+---------+---------+---------+---------+---------+---------+---------+---------|
contig022324 GTCAAACTTAGTGTTTGTCTGTGTTGGTTTTATGCTATTTTCTACATGAGTCTTTACACAAAGGATGTCTTGATAAAGCCAGGCAGGTATGCTTCCTGCTATGGAGAGTGTGTGTTTGTTATTGAAGATA
TilTARs.A027 GTCAAACTTAGTGTTTGTCTGTGTTGGTTTTATGCTATTTTCTACATGAGTCTTTACACAAAGGATGTCTTGATAAAGCCAGGCAGGTATGCTTCCTGCTATGGAGAGTGTGTGTTTGTTATTGAAGATA
   Consensus GTCAAACTTAGTGTTTGTCTGTGTTGGTTTTATGCTATTTTCTACATGAGTCTTTACACAAAGGATGTCTTGATAAAGCCAGGCAGGTATGCTTCCTGCTATGGAGAGTGTGTGTTTGTTATTGAAGATA

             3641  3650       3660       3670       3680       3690       3700       3710       3720       3730       3740       3750       3760       3770
             |--------+---------+---------+---------+---------+---------+---------+---------+---------+---------+---------+---------+---------|
contig022324 TAACTGGCACTGTTGACCTTGTTTTATGTTTTTTTGTTCCAGTTATTATCATCATTGTTCTGTATACAAGAGTATTTGTGGTGGCTGTGTCTCAGGCTCGTGCCATGCGCTCTCATGTTACAGCTGTCAC
TilTARs.A027 TAACTGGCACTGTTGACCTTGTTTTATGTTTTTTTGTTCCAGTTATTATCATCATTGTTCTGTATACAAGAGTATTTGTGGTGGCTGTGTCTCAGGCTCGTGCCATGCGCTCTCATGTTACAGCTGTCAC
   Consensus TAACTGGCACTGTTGACCTTGTTTTATGTTTTTTTGTTCCAGTTATTATCATCATTGTTCTGTATACAAGAGTATTTGTGGTGGCTGTGTCTCAGGCTCGTGCCATGCGCTCTCATGTTACAGCTGTCAC

             3771  3780       3790       3800       3810       3820       3830       3840       3850       3860       3870       3880       3890       3900
             |--------+---------+---------+---------+---------+---------+---------+---------+---------+---------+---------+---------+---------|
contig022324 ACTTGAGCGTTCACTGAATCAGACAAACAAATCTGAGTTGAAAGCAGCCAGGAACCTTGGAGTTCTTGTAATTGTGTTTCTAGCAAGTATTTGCCCATTTTACTTCTACTCTCTTGTTGATGGCAGTGTG
TilTARs.A027 ACTTGAGCGTTCACTGAATCAGACAAACAAATCTGAGTTGAAAGCAGCCAGGAACCTTGGAGTTCTTGTAATTGTGTTTCTAGCAAGTATTTGCCCATTTTACTTCTACTCTCTTGTTGATGGCAGTGTG
   Consensus ACTTGAGCGTTCACTGAATCAGACAAACAAATCTGAGTTGAAAGCAGCCAGGAACCTTGGAGTTCTTGTAATTGTGTTTCTAGCAAGTATTTGCCCATTTTACTTCTACTCTCTTGTTGATGGCAGTGTG

             3901  3910       3920       3930       3940       3950       3960       3970       3980       3990       4000       4010       4020       4030
             |--------+---------+---------+---------+---------+---------+---------+---------+---------+---------+---------+---------+---------|
contig022324 CTCAATGCTTCATCTGCAACTTTTTTGATCATTGTTTATTTTAACTCTTTTTTAAACCCATTGATCTATACCCTGTTTTACCCCTGGTTTAGAAATGCTGTTAAACTCATCATCACTCTGCAGATATTCA
TilTARs.A027 CTCAATGCTTCATCTGCAACTTTTTTGATCATTGTTTATTTTAACTCTTTTTTAAACCCATTGATCTATACCCTGTTTTACCCCTGGTTTAGAAATGCTGTTAAACTCATCATCACTCTGCAGATATTCA
   Consensus CTCAATGCTTCATCTGCAACTTTTTTGATCATTGTTTATTTTAACTCTTTTTTAAACCCATTGATCTATACCCTGTTTTACCCCTGGTTTAGAAATGCTGTTAAACTCATCATCACTCTGCAGATATTCA

             4031  4040       4050       4060       4070       4080       4090       4100       4110       4120       4130       4140       4150       4160
             |--------+---------+---------+---------+---------+---------+---------+---------+---------+---------+---------+---------+---------|
contig022324 AGCATAACAGTAGTGAAGCCAACATACTATAGAGACAATGTGGAGGAACTTGACTGAAATCTGCAGAAAGATTTTTTTCAGTAAATTGATGTGTCCAAAATCTGGAAATATAAAAATGTTTTTTAATATG
TilTARs.A027 AGCATAACAGTAGTGAAGCCAACATACTATAG
   Consensus AGCATAACAGTAGTGAAGCCAACATACTATAG..................................................................................................

             4161  4170       4180       4190       4200       4210       4220       4230       4240       4250       4260       4270       4280       4290
```

```
            1         10        20        30        40        50        60        70        80        90        100       110       120       130
contig022330 CAGTGGTTGTAGTTGTCTGATGATGGAGATACAGAAAGAAGCTGAGCTCTGTTTTCCACAACTCCTCAACATTTCCTGCAGGAAGCCGACACTTCACTGGTCCAAAGCTGTGCTCCTGAACATTGTGCTG
TilTARs.A028                       ATGGAGATACAGAAAGAAGCTGAGCTCTGTTTTCCACAACTCCTCAACATTTCCTGCAGGAAGCCGACACTTCACTGGTCCAAAGCTGTGCTCCTGAACATTGTGCTG
   Consensus ......................ATGGAGATACAGAAAGAAGCTGAGCTCTGTTTTCCACAACTCCTCAACATTTCCTGCAGGAAGCCGACACTTCACTGGTCCAAAGCTGTGCTCCTGAACATTGTGCTG

            131       140       150       160       170       180       190       200       210       220       230       240       250       260
contig022330 TCCTGCATCTCTCTGCTGACTGCTACTCTAAACCTTCTCGTGATTATCTCAGTCTCCTACTTCAGGCACAGATAATGTTTCTAAAACTTAAACTGTATTTTCATTTAAAAAATTTAACACCATGATATAT
TilTARs.A028 TCCTGCATCTCTCTGCTGACTGCTACTCTAAACCTTCTCGTGATTATCTCAGTCTCCTACTTCAGG----------------------------------------------------------------
   Consensus TCCTGCATCTCTCTGCTGACTGCTACTCTAAACCTTCTCGTGATTATCTCAGTCTCCTACTTCAGG................................................................

            261       270       280       290       300       310       320       330       340       350       360       370       380       390
contig022330 TATTTAAATTGAAATGAATTTGGCACATTCTTGGTCAATTTAAACCACAATATTTAATTTTTATACACCTGGAGTGAGTCTTAATAATGGACCTCTTTACATTTTAAAATTACTAATATATTCTTTTTTG
TilTARs.A028 ----------------------------------------------------------------------------------------------------------------------------------
   Consensus ..................................................................................................................................

            391       400       410       420       430       440       450       460       470       480       490       500       510       520
contig022330 TTTTATTTCAGGAAGCTTCACACACCCAGCAACATCCTCCTACTCTCTCTTGCTGTCTCAGACTTTCTCATGGGTCTCTTGCTGATACCAGCAGAAATCCTTAGAAGCATGACCTGCTGGGTACTTGGTG
TilTARs.A028 ------------AAGCTTCACACACCCAGCAACATCCTCCTACTCTCTCTTGCTGTCTCAGACTTTCTCATGGGTCTCTTGCTGATACCAGCAGAAATCCTTAGAAGCATGACCTGCTGGGTACTTGGTG
   Consensus ............AAGCTTCACACACCCAGCAACATCCTCCTACTCTCTCTTGCTGTCTCAGACTTTCTCATGGGTCTCTTGCTGATACCAGCAGAAATCCTTAGAAGCATGACCTGCTGGGTACTTGGTG

            521       530       540       550       560       570       580       590       600       610       620       630       640       650
contig022330 ATCTCATGTGTTCTGTTTATTTTTTTCTGACTGTAAACATTACCTGTGCTTCAATAGGGAACATAGTTCTTATATCGATTGACCGTCATGTGGCTATTTGTGACCCTCTGCATTACTCCACCAGAATTAC
TilTARs.A028 ATCTCATGTGTTCTGTTTATTTTTTTCTGACTGTAAACATTACCTGTGCTTCAATAGGGAACATAGTTCTTATATCGATTGACCGTCATGTGGCTATTTGTGACCCTCTGCATTACTCCACCAGAATTAC
   Consensus ATCTCATGTGTTCTGTTTATTTTTTTCTGACTGTAAACATTACCTGTGCTTCAATAGGGAACATAGTTCTTATATCGATTGACCGTCATGTGGCTATTTGTGACCCTCTGCATTACTCCACCAGAATTAC

            651       660       670       680       690       700       710       720       730       740       750       760       770       780
contig022330 TGTGCCTAGAGTCAAACTCAGTGTTTGTCTGTGTTGGTTTTATTCAACTTTCTACAGCAGTCTTAATACACAAGAAATGCTGATTGTACCAGGCAGGTATAATTCCTGTTATGGAGAATGTGTGCTTGTC
TilTARs.A028 TGTGCCTAGAGTCAAACTCAGTGTTTGTCTGTGTTGGTTTTATTCAACTTTCTACAGCAGTCTTAATACACAAGAAATGCTGATTGTACCAGGCAGGTATAATTCCTGTTATGGAGAATGTGTGCTTGTC
   Consensus TGTGCCTAGAGTCAAACTCAGTGTTTGTCTGTGTTGGTTTTATTCAACTTTCTACAGCAGTCTTAATACACAAGAAATGCTGATTGTACCAGGCAGGTATAATTCCTGTTATGGAGAATGTGTGCTTGTC

            781       790       800       810       820       830       840       850       860       870       880       890       900       910
contig022330 ATCAGTGATTTTGCTGGAACTCTTGACCTCATTTTATTTTTTATTCTTCCAGTTACTGTCATCATAGTTCTGTATATGAGAGTATTTGTGGTGGCTGTGTCTCAGGCTCGTGCCATGCGCTCTCATGTTA
TilTARs.A028 ATCAGTGATTTTGCTGGAACTCTTGACCTCATTTTATTTTTTATTCTTCCAGTTACTGTCATCATAGTTCTGTATATGAGAGTATTTGTGGTGGCTGTGTCTCAGGCTCGTGCCATGCGCTCTCATGTTA
   Consensus ATCAGTGATTTTGCTGGAACTCTTGACCTCATTTTATTTTTTATTCTTCCAGTTACTGTCATCATAGTTCTGTATATGAGAGTATTTGTGGTGGCTGTGTCTCAGGCTCGTGCCATGCGCTCTCATGTTA

            911       920       930       940       950       960       970       980       990       1000      1010      1020      1030      1040
contig022330 CAGCTGTCACACTTCAGCGTTCACCCAATCAAACAAACAAATCTGAGCTGAAAGCAGCCAGGACTCTTGGGGTTCTGGTAGTTGTGTTTCTGGCATGCTACTGTCCATTTTATTGCTACTCTCTCGCTGA
TilTARs.A028 CAGCTGTCACACTTCAGCGTTCACCCAATCAAACAAACAAATCTGAGCTGAAAGCAGCCAGGACTCTTGGGGTTCTGGTAGTTGTGTTTCTGGCATGCTACTGTCCATTTTATTGCTACTCTCTCGCTGA
   Consensus CAGCTGTCACACTTCAGCGTTCACCCAATCAAACAAACAAATCTGAGCTGAAAGCAGCCAGGACTCTTGGGGTTCTGGTAGTTGTGTTTCTGGCATGCTACTGTCCATTTTATTGCTACTCTCTCGCTGA

            1041 1050       1060      1070      1080      1090      1100      1110      1120      1130      1140      1150      1160      1170
contig022330 TGAAAATGCTGTCAATGATCCAGCAGCGTCTTTTGTGGTCTTTGTCTTTTACTTTAACTCTTGTATAAACCCTTTGATGTATGCCCTGTTTTACCCCTGGTTTAGAAATGCTGTTAAACTTATTATCACG
TilTARs.A028 TGAAAATGCTGTCAATGATCCAGCAGCGTCTTTTGTGGTCTTTGTCTTTTACTTTAACTCTTGTATAAACCCTTTGATGTATGCCCTGTTTTACCCCTGGTTTAGAAATGCTGTTAAACTTATTATCACG
   Consensus TGAAAATGCTGTCAATGATCCAGCAGCGTCTTTTGTGGTCTTTGTCTTTTACTTTAACTCTTGTATAAACCCTTTGATGTATGCCCTGTTTTACCCCTGGTTTAGAAATGCTGTTAAACTTATTATCACG

            1171 1180       1190      1200      1210      1220      1230      1240      1250      1260      1270      1280      1290      1300
contig022330 TTGCAGATATTCAAGCATAACACCTGTGAGGCCAACATACTATAAAAAGACTATGAACCCTGCTGCAGTAAATTGCTTTAACTCAGCAAATGGACCCATGTGGTAATCTACTGCATTACATACAAACAGA
TilTARs.A028 TTGCAGATATTCAAGCATAACACCTGTGAGGCCAACATACTATAA
   Consensus TTGCAGATATTCAAGCATAACACCTGTGAGGCCAACATACTATAA.....................................................................................

            1301 1310       1320      1330      1340      1350      1360      1370      1380      1390      1400      1410      1420      1430
contig022330 GGGAAAAAACAGCATATGTGACCAGGGCTGGACAGGGACAAAAAATCGGCCCGGGCATTTTGACTAGAGACCGGCCCACCAGGTATTATGGTAAAAACCATAAAGCCTTTGAATGAAAACAAACGTTGTT
TilTARs.A028
   Consensus ..................................................................................................................................
```

```
            911     920       930       940       950       960       970       980       990       1000      1010      1020      1030      1040
            |---------+---------+---------+---------+---------+---------+---------+---------+---------+---------+---------+---------+---------|
contig022334 GTCTGATGATGGAGATACAGAAAGAAGCTGAGCTCTGTTTTCCACAACTCCTCAACATTTCCTGCAGGAAGCCGACACTTCACTGGTCCAAAGCTGTGCTCCTGAACATTGTGCTGTCCTGCATCTCTCT
TilTARs.A029         ATGGAGATACAGAAAGAAGCTGAGCTCTGTTTTCCACAACTCCTCAACATTTCCTGCAGGAAGCCGACACTTCACTGGTCCAAAGCTGTGCTCCTGAACATTGTGCTGTCCTGCATCTCTCT
   Consensus ........ATGGAGATACAGAAAGAAGCTGAGCTCTGTTTTCCACAACTCCTCAACATTTCCTGCAGGAAGCCGACACTTCACTGGTCCAAAGCTGTGCTCCTGAACATTGTGCTGTCCTGCATCTCTCT

            1041    1050      1060      1070      1080      1090      1100      1110      1120      1130      1140      1150      1160      1170
            |---------+---------+---------+---------+---------+---------+---------+---------+---------+---------+---------+---------+---------|
contig022334 GCTGACTGCTACTCTAAACCTTCTCGTGATTATCTCAGTCTCCTACTTCAGGCACAGATAATGTTTCTAAAACTTAAACTGTATTTTCATTTAAAAAATTTAACACCATGATATATTATTTAAATTGAAA
TilTARs.A029 GCTGACTGCTACTCTAAACCTTCTCGTGATTATCTCAGTCTCCTACTTCAGG------------------------------------------------------------------------------
   Consensus GCTGACTGCTACTCTAAACCTTCTCGTGATTATCTCAGTCTCCTACTTCAGG..............................................................................

            1171    1180      1190      1200      1210      1220      1230      1240      1250      1260      1270      1280      1290      1300
            |---------+---------+---------+---------+---------+---------+---------+---------+---------+---------+---------+---------+---------|
contig022334 TGAATTTGGCACATTCTTGGTCAATTTAAACCACAATATTTAATTTTTATACACCTGGAGTGAGTCTTAATAATGGACCTCTTTACATTTTAAAATTACTAATATATTCTTTTTTGTTTTATTTCAGGAA
TilTARs.A029 --------------------------------------------------------------------------------------------------------------------------------AA
   Consensus ................................................................................................................................AA

            1301    1310      1320      1330      1340      1350      1360      1370      1380      1390      1400      1410      1420      1430
            |---------+---------+---------+---------+---------+---------+---------+---------+---------+---------+---------+---------+---------|
contig022334 GCTTCACACACCCAGCAACATCCTCCTACTCTCTCTTGCTGTCTCAGACTTTCTCATGGGTCTCTTGCTGATACCAGCAGAAATCCTTAGAAGCATGACCTGCTGGGTACTTGGTGATCTCATGTGTTCT
TilTARs.A029 GCTTCACACACCCAGCAACATCCTCCTACTCTCTCTTGCTGTCTCAGACTTTCTCATGGGTCTCTTGCTGATACCAGCAGAAATCCTTAGAAGCATGACCTGCTGGGTACTTGGTGATCTCATGTGTTCT
   Consensus GCTTCACACACCCAGCAACATCCTCCTACTCTCTCTTGCTGTCTCAGACTTTCTCATGGGTCTCTTGCTGATACCAGCAGAAATCCTTAGAAGCATGACCTGCTGGGTACTTGGTGATCTCATGTGTTCT

            1431    1440      1450      1460      1470      1480      1490      1500      1510      1520      1530      1540      1550      1560
            |---------+---------+---------+---------+---------+---------+---------+---------+---------+---------+---------+---------+---------|
contig022334 GTTTATTTTTTTCTGACTGTAAACATTACCTGTGCTTCAATAGGGAACATAGTTCTTATATCGATTGACCGTCATGTGGCTATTTGTGACCCTCTGCATTACCCCACCAGAATGACTGTGCCAAAAATCA
TilTARs.A029 GTTTATTTTTTTCTGACTGTAAACATTACCTGTGCTTCAATAGGGAACATAGTTCTTATATCGATTGACCGTCATGTGGCTATTTGTGACCCTCTGCATTACCCCACCAGAATGACTGTGCCAAAAATCA
   Consensus GTTTATTTTTTTCTGACTGTAAACATTACCTGTGCTTCAATAGGGAACATAGTTCTTATATCGATTGACCGTCATGTGGCTATTTGTGACCCTCTGCATTACCCCACCAGAATGACTGTGCCAAAAATCA

            1561    1570      1580      1590      1600      1610      1620      1630      1640      1650      1660      1670      1680      1690
            |---------+---------+---------+---------+---------+---------+---------+---------+---------+---------+---------+---------+---------|
contig022334 AACTCAGTGTTTGTCTGTGTTGGTTTTATTCAACTTTCTACAGCAGTCTTAACACAAAAAAAATCCTGATTGAACCAGGCAGGTATAATTCCTGCTATGGAGAATGTGTGCTTGTCAGCAGTAATATTGC
TilTARs.A029 AACTCAGTGTTTGTCTGTGTTGGTTTTATTCAACTTTCTACAGCAGTCTTAACACAAAAAAAATCCTGATTGAACCAGGCAGGTATAATTCCTGCTATGGAGAATGTGTGCTTGTCAGCAGTAATATTGC
   Consensus AACTCAGTGTTTGTCTGTGTTGGTTTTATTCAACTTTCTACAGCAGTCTTAACACAAAAAAAATCCTGATTGAACCAGGCAGGTATAATTCCTGCTATGGAGAATGTGTGCTTGTCAGCAGTAATATTGC

            1691    1700      1710      1720      1730      1740      1750      1760      1770      1780      1790      1800      1810      1820
            |---------+---------+---------+---------+---------+---------+---------+---------+---------+---------+---------+---------+---------|
contig022334 TGGAACTCTTGACCTCATTTTATTTTTTATTCTTCCAGTTACTGTCATCATAGTTCTGTATATGAGAGTATTTGTGGTGGCTGTGTCTCAGGCTCGTGCCATGCGCTCTCATGTTACAGCTGTCACACTT
TilTARs.A029 TGGAACTCTTGACCTCATTTTATTTTTTATTCTTCCAGTTACTGTCATCATAGTTCTGTATATGAGAGTATTTGTGGTGGCTGTGTCTCAGGCTCGTGCCATGCGCTCTCATGTTACAGCTGTCACACTT
   Consensus TGGAACTCTTGACCTCATTTTATTTTTTATTCTTCCAGTTACTGTCATCATAGTTCTGTATATGAGAGTATTTGTGGTGGCTGTGTCTCAGGCTCGTGCCATGCGCTCTCATGTTACAGCTGTCACACTT

            1821    1830      1840      1850      1860      1870      1880      1890      1900      1910      1920      1930      1940      1950
            |---------+---------+---------+---------+---------+---------+---------+---------+---------+---------+---------+---------+---------|
contig022334 CAGCGTTCACCCAATCAAACAAACAAATCTGAGCTGAAAGCAGCCAGGACTCTTGGGGTTCTGGTAGTTGTGTTTCTGGCATGCTACTGTCCATTTTATTGCTACTCTCTCGCTGATGAAAATGCTGTCA
TilTARs.A029 CAGCGTTCACCCAATCAAACAAACAAATCTGAGCTGAAAGCAGCCAGGACTCTTGGGGTTCTGGTAGTTGTGTTTCTGGCATGCTACTGTCCATTTTATTGCTACTCTCTCGCTGATGAAAATGCTGTCA
   Consensus CAGCGTTCACCCAATCAAACAAACAAATCTGAGCTGAAAGCAGCCAGGACTCTTGGGGTTCTGGTAGTTGTGTTTCTGGCATGCTACTGTCCATTTTATTGCTACTCTCTCGCTGATGAAAATGCTGTCA

            1951    1960      1970      1980      1990      2000      2010      2020      2030      2040      2050      2060      2070      2080
            |---------+---------+---------+---------+---------+---------+---------+---------+---------+---------+---------+---------+---------|
contig022334 ATGATCCAGCAGCGTCTTTTGTGGTCTTTGTCTTTTACTTTAACTCTTGTATAAACCCTTTGATGTATGCCCTGTTTTACCCCTGGTTTAGAAATGCTGTTAAACTTATTATCACGTTGCAGATATTCAA
TilTARs.A029 ATGATCCAGCAGCGTCTTTTGTGGTCTTTGTCTTTTACTTTAACTCTTGTATAAACCCTTTGATGTATGCCCTGTTTTACCCCTGGTTTAGAAATGCTGTTAAACTTATTATCACGTTGCAGATATTCAA
   Consensus ATGATCCAGCAGCGTCTTTTGTGGTCTTTGTCTTTTACTTTAACTCTTGTATAAACCCTTTGATGTATGCCCTGTTTTACCCCTGGTTTAGAAATGCTGTTAAACTTATTATCACGTTGCAGATATTCAA

            2081    2090      2100      2110      2120      2130      2140      2150      2160      2170      2180      2190      2200      2210
            |---------+---------+---------+---------+---------+---------+---------+---------+---------+---------+---------+---------+---------|
contig022334 GCATAACACCTGTGAGGCCAACATACTATAAAAAGACTATGAACCCTGCTGCAGTAAATTGCTTTAACTCAGCAAATGGACCCATGTGGTAATCTACTGCATTACATACAAACAGAAGAAATTTTAGTTC
TilTARs.A029 GCATAACACCTGTGAGGCCAACATACTATAA
   Consensus GCATAACACCTGTGAGGCCAACATACTATAA...................................................................................................

            2211    2220      2230      2240      2250      2260      2270      2280      2290      2300      2310      2320      2330      2340
            |---------+---------+---------+---------+---------+---------+---------+---------+---------+---------+---------+---------+---------|
contig022334 AATTCAATAAACACTAACATTCAGATTACTTACCTTATGCCATTTTCTCATGAAAGAAATTTCAAATTTATATTTATATTCATTTATAATATATTATTTATAATTATGGCTATGTTCAGACACTGAGACT
TilTARs.A029
   Consensus ..................................................................................................................................

            [illegible]
```

```
              8191  8200      8210      8220      8230      8240      8250      8260      8270      8280      8290      8300      8310      8320
              |--------+---------+---------+---------+---------+---------+---------+---------+---------+---------+---------+---------+---------|
contig022334  TCCATTTGATTCATGTGCTGATTATTAACAGCTCTGCAAGGCCGACATCTGATCAGTCAGCTGCTGATGGAGATACAAAATGAAGCTGAGCTTTGCTTTCCAGAACTCCTCAACAGTTCCTGCAGGAGGA
TilTARs.A030                                                                      ATGGAGATACAAAATGAAGCTGAGCTTTGCTTTCCAGAACTCCTCAACAGTTCCTGCAGGAGGA
   Consensus  ..................................................................ATGGAGATACAAAATGAAGCTGAGCTTTGCTTTCCAGAACTCCTCAACAGTTCCTGCAGGAGGA

              8321  8330      8340      8350      8360      8370      8380      8390      8400      8410      8420      8430      8440      8450
              |--------+---------+---------+---------+---------+---------+---------+---------+---------+---------+---------+---------+---------|
contig022334  CGACACTTGTCCACTGGTCCAAAGCTGTACTGCTGTACATTGTGCTGTTCTGCATTTCTCTGATCACTGCTGCTCTAAACCTTCTGGTCATCATCTCAGTCTCCCACTTCAGGCATGAATTAACCGTTCA
TilTARs.A030  CGACACTTGTCCACTGGTCCAAAGCTGTACTGCTGTACATTGTGCTGTTCTGCATTTCTCTGATCACTGCTGCTCTAAACCTTCTGGTCATCATCTCAGTCTCCCACTTCAGGCA---------------
   Consensus  CGACACTTGTCCACTGGTCCAAAGCTGTACTGCTGTACATTGTGCTGTTCTGCATTTCTCTGATCACTGCTGCTCTAAACCTTCTGGTCATCATCTCAGTCTCCCACTTCAGGCA...............

              8451  8460      8470      8480      8490      8500      8510      8520      8530      8540      8550      8560      8570      8580
              |--------+---------+---------+---------+---------+---------+---------+---------+---------+---------+---------+---------+---------|
contig022334  TTTAAAACTACAGCTTTAAATTCCTACATATGTGAAAATTTAGATCAGTGGAATTAGCTAGAAAGAAGTGCAATAGATAGTTATAACTGAGCGAGATATATTTAAGTTGATAAAACCTAATCATGTTTTA
TilTARs.A030  ----------------------------------------------------------------------------------------------------------------------------------
   Consensus  ..................................................................................................................................

              8581  8590      8600      8610      8620      8630      8640      8650      8660      8670      8680      8690      8700      8710
              |--------+---------+---------+---------+---------+---------+---------+---------+---------+---------+---------+---------+---------|
contig022334  ATATTGAAATATTTTCACTAAAGACATCTTGTTATGATTTACACATGACATTAACACTGCTGCTTTCAACTATCATCAAAGTTACCAACATCAGACATCAGATTAATTTCTTTGCTGTAATTACAGTTTT
TilTARs.A030  ----------------------------------------------------------------------------------------------------------------------------------
   Consensus  ..................................................................................................................................

              8711  8720      8730      8740      8750      8760      8770      8780      8790      8800      8810      8820      8830      8840
              |--------+---------+---------+---------+---------+---------+---------+---------+---------+---------+---------+---------+---------|
contig022334  CAAAACTTTAAGACAATGATTTGGTGAATCTGGCACATTCTTAGGATATTTAAAGCATGATTATGTTTTGAATATTGGAATTTTAAATAAAATAGTTTGACATTAATAATGTGCATTATATATTTTTTGT
TilTARs.A030  ----------------------------------------------------------------------------------------------------------------------------------
   Consensus  ..................................................................................................................................

              8841  8850      8860      8870      8880      8890      8900      8910      8920      8930      8940      8950      8960      8970
              |--------+---------+---------+---------+---------+---------+---------+---------+---------+---------+---------+---------+---------|
contig022334  TTCTCTGCAGGCAGCTTCACACACCCACTAACATCCTCCTCCTCTCTCTGGCTGTTTCAGACTTTCTTGTGGGCCTCCTATTGATGCCATTAGAAATTGTTAGAGGCATGACCTGCTGGGTACTTGGTGA
TilTARs.A030  -------------GCTTCACACACCCACTAACATCCTCCTCCTCTCTCTGGCTGTTTCAGACTTTCTTGTGGGCCTCCTATTGATGCCATTAGAAATTGTTAGAGGCATGACCTGCTGGGTACTTGGTGA
   Consensus  .............GCTTCACACACCCACTAACATCCTCCTCCTCTCTCTGGCTGTTTCAGACTTTCTTGTGGGCCTCCTATTGATGCCATTAGAAATTGTTAGAGGCATGACCTGCTGGGTACTTGGTGA

              8971  8980      8990      9000      9010      9020      9030      9040      9050      9060      9070      9080      9090      9100
              |--------+---------+---------+---------+---------+---------+---------+---------+---------+---------+---------+---------+---------|
contig022334  TCTTATGTGTTCTGTTTATATTTTCCTTACTGGCAACGTTACCTGTGCTTCAATAGGGAACATAGTTCTAATATCAGTTGACCGTTATGTGGCTATTTGTTACCCTCTGCATTATCCCACCAGAATTACT
TilTARs.A030  TCTTATGTGTTCTGTTTATATTTTCCTTACTGGCAACGTTACCTGTGCTTCAATAGGGAACATAGTTCTAATATCAGTTGACCGTTATGTGGCTATTTGTTACCCTCTGCATTATCCCACCAGAATTACT
   Consensus  TCTTATGTGTTCTGTTTATATTTTCCTTACTGGCAACGTTACCTGTGCTTCAATAGGGAACATAGTTCTAATATCAGTTGACCGTTATGTGGCTATTTGTTACCCTCTGCATTATCCCACCAGAATTACT

              9101  9110      9120      9130      9140      9150      9160      9170      9180      9190      9200      9210      9220      9230
              |--------+---------+---------+---------+---------+---------+---------+---------+---------+---------+---------+---------+---------|
contig022334  GTGCCTAGAGTCAAACTAAGTGTTTGTGTTGGTTTTATGCCATTTTCTACATCAGTCTTTATGCAAAAGACTCTGAACCAGGCAGGTATAGTTCTTGCTACGGAGAGTGTGTCCTGGTTGTCAGTGTTGT
TilTARs.A030  GTGCCTAGAGTCAAACTAAGTGTTTGTGTTGGTTTTATGCCATTTT-----------------------------------------ATAGTTCTTGCTACGGAGAGTGTGTCCTGGTTGTCAGTGTTGT
   Consensus  GTGCCTAGAGTCAAACTAAGTGTTTGTGTTGGTTTTATGCCATTTT.........................................ATAGTTCTTGCTACGGAGAGTGTGTCCTGGTTGTCAGTGTTGT

              9231  9240      9250      9260      9270      9280      9290      9300      9310      9320      9330      9340      9350      9360
              |--------+---------+---------+---------+---------+---------+---------+---------+---------+---------+---------+---------+---------|
contig022334  TGTAGGGATTGTTGACCTTGTTTTATCCTATTTTGTTCCAGTTACTGTCATCATAGTTCTGTATATGAGAGTGTTTGTGGTGGCTGTGTCTCAGGCTCGCGCCATGCGCTCTCATGTTACAACTGTCGCA
TilTARs.A030  TGTAGGGATTGTTGACCTTGTTTTATCCTATTTTGTTCCAGTTACTGTCATCATAGTTCTGTATATGAGAGTGTTTGTGGTGGCTGTGTCTCAGGCTCGCGCCATGCGCTCTCATGTTACAACTGTCGCA
   Consensus  TGTAGGGATTGTTGACCTTGTTTTATCCTATTTTGTTCCAGTTACTGTCATCATAGTTCTGTATATGAGAGTGTTTGTGGTGGCTGTGTCTCAGGCTCGCGCCATGCGCTCTCATGTTACAACTGTCGCA

              9361  9370      9380      9390      9400      9410      9420      9430      9440      9450      9460      9470      9480      9490
              |--------+---------+---------+---------+---------+---------+---------+---------+---------+---------+---------+---------+---------|
contig022334  CTTCAGCGTTCATTGAATCAAACTAACAAATCTGAGTTGAAAGCAGCCAGGACTCTGGGGATTCTTGTAGTTGTGTTTCTACTGTGCTCTTGCCCATACTACTGCATCTCATTTGCAAGGGATAACTATA
TilTARs.A030  CTTCAGCGTTCATTGAATCAAACTAACAAATCTGAGTTGAAAGCAGCCAGGACTCTGGGGATTCTTGTAGTTGTGTTTCTACTGTGCTCTTGCCCATACTACTGCATCTCATTTGCAAGGGATAACTATA
   Consensus  CTTCAGCGTTCATTGAATCAAACTAACAAATCTGAGTTGAAAGCAGCCAGGACTCTGGGGATTCTTGTAGTTGTGTTTCTACTGTGCTCTTGCCCATACTACTGCATCTCATTTGCAAGGGATAACTATA

              9491  9500      9510      9520      9530      9540      9550      9560      9570      9580      9590      9600      9610      9620
              |--------+---------+---------+---------+---------+---------+---------+---------+---------+---------+---------+---------+---------|
contig022334  GCTCAGTACCATCTATTGTGTTTGGTCTGTTTTTCTTAAACTCCTCTCTAAATCCTCTGATCTATGCAATGTTCTACCCTTGGTTTAGAAAAACTGTTACACTAATTGTCACTCTACAGATCCTGCAGAC
TilTARs.A030  GCTCAGTACCATCTATTGTGTTTGGTCTGTTTTTCTTAAACTCCTCTCTAAATCCTCTGATCTATGCAATGTTCTACCCTTGGTTTAGAAAAACTGTTACACTAATTGTCACTCTACAGATCCTGCAGAC
   Consensus  GCTCAGTACCATCTATTGTGTTTGGTCTGTTTTTCTTAAACTCCTCTCTAAATCCTCTGATCTATGCAATGTTCTACCCTTGGTTTAGAAAAACTGTTACACTAATTGTCACTCTACAGATCCTGCAGAC

              9621  9630      9640      9650      9660      9670      9680      9690      9700      9710      9720      9730      9740      9750
              |--------+---------+---------+---------+---------+---------+---------+---------+---------+---------+---------+---------+---------|
contig022334  TATTTCTTGTGAGGTCAATATACTGTAGAACAGGGGTGTCCAACTCCAGGCCTTTAGGGCCAGTGTCCTGCAGGTTTTAGATCTCACCCTGGGTCAACACCTGAATAAAAAGATTAGTTCATTACCAGGC
TilTARs.A030  TATTTCTTGTGAGGTCAATATACTGTAG
   Consensus  TATTTCTTGTGAGGTCAATATACTGTAG......................................................................................................
```

```
   Consensus  ..................................................................................................................................

              1691 1700      1710      1720      1730      1740      1750      1760      1770      1780      1790      1800      1810      1820
              |--------+---------+---------+---------+---------+---------+---------+---------+---------+---------+---------+---------+---------|
contig022337  ATATTTTAAAGAGATTGGTGAGAAGAGAAAGAAAAATTAGCTTTATAAGAAAAAGTTTTGATTCAAGAGCTGCTGCCTGATGATGGAGATAGAGAAAGGAGCCGAGCTCTGTTTTCCGCAACTCCTCAAC
TilTARs.A031                                                                                 ATGATGGAGATAGAGAAAGGAGCCGAGCTCTGTTTTCCGCAACTCCTCAAC
   Consensus  ...............................................................................ATGATGGAGATAGAGAAAGGAGCCGAGCTCTGTTTTCCGCAACTCCTCAAC

              1821 1830      1840      1850      1860      1870      1880      1890      1900      1910      1920      1930      1940      1950
              |--------+---------+---------+---------+---------+---------+---------+---------+---------+---------+---------+---------+---------|
contig022337  AGTTCCTGCAGGAAGCCGACACTTCACTGGTCCAGAGCTTTGCTCCTGAACATTGTGCTGTCCTGCATCTCTCTGATCACTGCAGCTCTAAACCTTCTTGTCATCATCTCAGTCTCTTACTTCAGGTAAT
TilTARs.A031  AGTTCCTGCAGGAAGCCGACACTTCACTGGTCCAGAGCTTTGCTCCTGAACATTGTGCTGTCCTGCATCTCTCTGATCACTGCAGCTCTAAACCTTCTTGTCATCATCTCAGTCTCTTACTTCAGG----
   Consensus  AGTTCCTGCAGGAAGCCGACACTTCACTGGTCCAGAGCTTTGCTCCTGAACATTGTGCTGTCCTGCATCTCTCTGATCACTGCAGCTCTAAACCTTCTTGTCATCATCTCAGTCTCTTACTTCAGG....

              1951 1960      1970      1980      1990      2000      2010      2020      2030      2040      2050      2060      2070      2080
              |--------+---------+---------+---------+---------+---------+---------+---------+---------+---------+---------+---------+---------|
contig022337  GACTTTTTTAACTGCAGGTAAGTATTGGTACTTTATTTAATTATCTAACAGGGTCAGTACATCTTAACACTGTATATAGGACTTAGCTACAGCTACTTTTGAGAGTGTGTCCCTAAGACAGAGTACATTA
TilTARs.A031  ----------------------------------------------------------------------------------------------------------------------------------
   Consensus  ..................................................................................................................................

              2081 2090      2100      2110      2120      2130      2140      2150      2160      2170      2180      2190      2200      2210
              |--------+---------+---------+---------+---------+---------+---------+---------+---------+---------+---------+---------+---------|
contig022337  AAACAAATATATGATGACAGGAGACCCAATTCAATGATCACAGATGTGCTTTTAAGTCATAATTTTGCCTTTGGATTGAAAAACTATATTATCTGGTAATGTTTTTATTTCTGGTGGTAAAGTGTTCTAA
TilTARs.A031  ----------------------------------------------------------------------------------------------------------------------------------
   Consensus  ..................................................................................................................................

              2211 2220      2230      2240      2250      2260      2270      2280      2290      2300      2310      2320      2330      2340
              |--------+---------+---------+---------+---------+---------+---------+---------+---------+---------+---------+---------+---------|
contig022337  ACCTGGCGGCCTTTAACTGTAAAAGAGGACAAACCAAATCAGTTTCTACATTTTGGCAGCTTAAAATCCCATAGTAGCATTCTAGCAATTACATTTAAAAGTCAACTTTAATAAAGAAACTTTTATGAAA
TilTARs.A031  ----------------------------------------------------------------------------------------------------------------------------------
   Consensus  ..................................................................................................................................

              2341 2350      2360      2370      2380      2390      2400      2410      2420      2430      2440      2450      2460      2470
              |--------+---------+---------+---------+---------+---------+---------+---------+---------+---------+---------+---------+---------|
contig022337  TTCTCAAAGCTTAACATTAGTTAACACAAGTTTTATATATATAAAAACTGATTGACGTTAAGTTTTTTATGTACAAAGACTGAATCATGCCTACTTTAACATTTTAACTGAAAACCTTTTGAAATGCTTT
TilTARs.A031  ----------------------------------------------------------------------------------------------------------------------------------
   Consensus  ..................................................................................................................................

              2471 2480      2490      2500      2510      2520      2530      2540      2550      2560      2570      2580      2590      2600
              |--------+---------+---------+---------+---------+---------+---------+---------+---------+---------+---------+---------+---------|
contig022337  TTTTTTCTGTTTCCATCCAGGCAGCTCCACACGCCTAGCAACATTGTCCTACTCTCTCTGGCTGTTTCAGACTTTCTAATTGGTCTCCTCAAGATGCCATTTGAAATCATTAGAAACACAGGCTGCTGGG
TilTARs.A031  ---------------------CAGCTCCACACGCCTAGCAACATTGTCCTACTCTCTCTGGCTGTTTCAGACTTTCTAATTGGTCTCCTCAAGATGCCATTTGAAATCATTAGAAACACAGGCTGCTGGG
   Consensus  .....................CAGCTCCACACGCCTAGCAACATTGTCCTACTCTCTCTGGCTGTTTCAGACTTTCTAATTGGTCTCCTCAAGATGCCATTTGAAATCATTAGAAACACAGGCTGCTGGG

              2601 2610      2620      2630      2640      2650      2660      2670      2680      2690      2700      2710      2720      2730
              |--------+---------+---------+---------+---------+---------+---------+---------+---------+---------+---------+---------+---------|
contig022337  TACTTGGTGATCTTATGTGTTCTGTTTATATTTTTCTGACCATCAGTCTTCTCTGTGCTTCAATACTTAACATAGTGCTAATATCAATTGACCGTTATGTGGCTATTTGTGACCCTCTGCACTATCCCAC
TilTARs.A031  TACTTGGTGATCTTATGTGTTCTGTTTATATTTTTCTGACCATCAGTCTTCTCTGTGCTTCAATACTTAACATAGTGCTAATATCAATTGACCGTTATGTGGCTATTTGTGACCCTCTGCACTATCCCAC
   Consensus  TACTTGGTGATCTTATGTGTTCTGTTTATATTTTTCTGACCATCAGTCTTCTCTGTGCTTCAATACTTAACATAGTGCTAATATCAATTGACCGTTATGTGGCTATTTGTGACCCTCTGCACTATCCCAC

              2731 2740      2750      2760      2770      2780      2790      2800      2810      2820      2830      2840      2850      2860
              |--------+---------+---------+---------+---------+---------+---------+---------+---------+---------+---------+---------+---------|
contig022337  CAGACTGACGGTGGCAAGAGTCAAACTCAGTGTTTGTCTGTGTTGGTTTTATTCTGCTTTCTACAGCAGTCTTTATGCAAAGAATGTCCTGATTGAACCAGGCAGGTATAATTTCTGCTTTGGAGAGTGT
TilTARs.A031  CAGACTGACGGTGGCAAGAGTCAAACTCAGTGTTTGTCTGTGTTGGTTTTATTCTGCTTTCTACAGCAGTCTTTATGCAAAGAATGTCCTGATTGAACCAGGCAGGTATAATTTCTGCTTTGGAGAGTGT
   Consensus  CAGACTGACGGTGGCAAGAGTCAAACTCAGTGTTTGTCTGTGTTGGTTTTATTCTGCTTTCTACAGCAGTCTTTATGCAAAGAATGTCCTGATTGAACCAGGCAGGTATAATTTCTGCTTTGGAGAGTGT

              2861 2870      2880      2890      2900      2910      2920      2930      2940      2950      2960      2970      2980      2990
              |--------+---------+---------+---------+---------+---------+---------+---------+---------+---------+---------+---------+---------|
contig022337  GTGTTTTTCAGCAGTAATATTGCTGTTGTTGTTGACCTTGTTTTATTCTTTATTGTTCCAGTTTCTGTCATCATAGTTCTGTATATGAGAGTATTTGTGGTGGCTGTGTCTCAGGCTCGTGCCATGCGCT
TilTARs.A031  GTGTTTTTCAGCAGTAATATTGCTGTTGTTGTTGACCTTGTTTTATTCTTTATTGTTCCAGTTTCTGTCATCATAGTTCTGTATATGAGAGTATTTGTGGTGGCTGTGTCTCAGGCTCGTGCCATGCGCT
   Consensus  GTGTTTTTCAGCAGTAATATTGCTGTTGTTGTTGACCTTGTTTTATTCTTTATTGTTCCAGTTTCTGTCATCATAGTTCTGTATATGAGAGTATTTGTGGTGGCTGTGTCTCAGGCTCGTGCCATGCGCT

              2991 3000      3010      3020      3030      3040      3050      3060      3070      3080      3090      3100      3110      3120
              |--------+---------+---------+---------+---------+---------+---------+---------+---------+---------+---------+---------+---------|
contig022337  CTCATGTTACATCTGTCACACTTCAGCGTTCAGCAAATCAAGCAAACAAATCTGAGCTGAAAGCAGCCAGGACTCTTGGGGTTCTTGTAGTTGTGTTTCTGGCAACCTTTTGTCCATTCTACTGCTACTC
TilTARs.A031  CTCATGTTACATCTGTCACACTTCAGCGTTCAGCAAATCAAGCAAACAAATCTGAGCTGAAAGCAGCCAGGACTCTTGGGGTTCTTGTAGTTGTGTTTCTGGCAACCTTTTGTCCATTCTACTGCTACTC
   Consensus  CTCATGTTACATCTGTCACACTTCAGCGTTCAGCAAATCAAGCAAACAAATCTGAGCTGAAAGCAGCCAGGACTCTTGGGGTTCTTGTAGTTGTGTTTCTGGCAACCTTTTGTCCATTCTACTGCTACTC

              3121 3130      3140      3150      3160      3170      3180      3190      3200      3210      3220      3230      3240      3250
              |--------+---------+---------+---------+---------+---------+---------+---------+---------+---------+---------+---------+---------|
contig022337  TCTTGTTGAGGAAAATGCTCTCAATGACCCATCGACATCTACTTTGATCACAGTTTTTTACTTATCTAACTCTTGTCTAAACCCTTTGATCTATGCCTTGTTTCACCCGTGGTTTAGAAATGCTGTTAAA
TilTARs.A031  TCTTGTTGAGGAAAATGCTCTCAATGACCCATCGACATCTACTTTGATCACAGTTTTTTACTTATCTAACTCTTGTCTAAACCCTTTGATCTATGCCTTGTTTCACCCGTGGTTTAGAAATGCTGTTAAA
   Consensus  TCTTGTTGAGGAAAATGCTCTCAATGACCCATCGACATCTACTTTGATCACAGTTTTTTACTTATCTAACTCTTGTCTAAACCCTTTGATCTATGCCTTGTTTCACCCGTGGTTTAGAAATGCTGTTAAA

              3251 3260      3270      3280      3290      3300      3310      3320      3330      3340      3350      3360      3370      3380
              |--------+---------+---------+---------+---------+---------+---------+---------+---------+---------+---------+---------+---------|
contig022337  CTCATCATCACTTTGCAGATCTTCAAGTGTGACACAAGTGAGGCCAACATACTGTAAAAAGATAAAGAAACTCAATACACTAAGAATTTAAATAAATAAATACACACATCTAAGAATGGAAATGGAAAAA
TilTARs.A031  CTCATCATCACTTTGCAGATCTTCAAGTGTGACACAAGTGAGGCCAACATACTGTAA
   Consensus  CTCATCATCACTTTGCAGATCTTCAAGTGTGACACAAGTGAGGCCAACATACTGTAA.........................................................................

              3381 3390      3400      3410      3420      3430      3440      3450      3460      3470      3480      3490      3500      3510
```

```
             2731 2740      2750      2760      2770      2780      2790      2800      2810      2820      2830      2840      2850      2860
             |--------+---------+---------+---------+---------+---------+---------+---------+---------+---------+---------+---------+---------|
contig022341 AAAAAAAGAGCAGAAATGGAAGGATGGGGAGGTGGAGGTGGAGGTGGGGTTGGTGGTAGAGATAAGGAGAGCTCTATAAGGGAAAGCTTCGTTCAGAGAGCTGCAGCAGTTGCAGTTGTCTTATTATGGA
TilTARs.A032                                                                                                                              ATGGA
   Consensus .............................................................................................................................ATGGA

             2861 2870      2880      2890      2900      2910      2920      2930      2940      2950      2960      2970      2980      2990
             |--------+---------+---------+---------+---------+---------+---------+---------+---------+---------+---------+---------+---------|
contig022341 GATACAGAAAGGAGTCGAGCTCTGTTTTCCACAACTCCTCAACACTTCCTGTAGGAAGCCGGCACTTCACTGGTCCAAAGCTGTGCTCCTGAACATTGTGCTGTCATGTGTCTCTCTGATCACTGCTGCT
TilTARs.A032 GATACAGAAAGGAGTCGAGCTCTGTTTTCCACAACTCCTCAACACTTCCTGTAGGAAGCCGGCACTTCACTGGTCCAAAGCTGTGCTCCTGAACATTGTGCTGTCATGTGTCTCTCTGATCACTGCTGCT
   Consensus GATACAGAAAGGAGTCGAGCTCTGTTTTCCACAACTCCTCAACACTTCCTGTAGGAAGCCGGCACTTCACTGGTCCAAAGCTGTGCTCCTGAACATTGTGCTGTCATGTGTCTCTCTGATCACTGCTGCT

             2991 3000      3010      3020      3030      3040      3050      3060      3070      3080      3090      3100      3110      3120
             |--------+---------+---------+---------+---------+---------+---------+---------+---------+---------+---------+---------+---------|
contig022341 CTAAACCTTCTGGTCATCATCTCAGTCTCTTACTTCAGGCACAAATTTAATATTTTAAACTAAACTGGATTTTAAAGTTTAGAAACACGGTGATTGAATGATAAGTGCAATGAATGTGGTACATAATTGA
TilTARs.A032 CTAAACCTTCTGGTCATCATCTCAGTCTCTTACTTCAGG-------------------------------------------------------------------------------------------
   Consensus CTAAACCTTCTGGTCATCATCTCAGTCTCTTACTTCAGG...........................................................................................

             3121 3130      3140      3150      3160      3170      3180      3190      3200      3210      3220      3230      3240      3250
             |--------+---------+---------+---------+---------+---------+---------+---------+---------+---------+---------+---------+---------|
contig022341 TGTACTTTAGCTATGACTGTAATGTTTTATGTGGCTAGACAGAATCATGTCTTTTAAAAGGAAATCTTTTACTAATGGAATTTTCAATAAGGATATATTGAAATGATTATTGTTGAATATATATATATTT
TilTARs.A032 ----------------------------------------------------------------------------------------------------------------------------------
   Consensus ..................................................................................................................................

             3251 3260      3270      3280      3290      3300      3310      3320      3330      3340      3350      3360      3370      3380
             |--------+---------+---------+---------+---------+---------+---------+---------+---------+---------+---------+---------+---------|
contig022341 TTTTTTTGTCTCTCTCTGCAGGAAGCTTCACACACCGACCAACATCCTCCTCCTTTCTCTGGCTGTCTCAGACTTTCTTGTGGGTCTCTTGCTGATGCCATTTGAAATCTTTAGGAACATGGCCTGCTGG
TilTARs.A032 ----------------------AAGCTTCACACACCGACCAACATCCTCCTCCTTTCTCTGGCTGTCTCAGACTTTCTTGTGGGTCTCTTGCTGATGCCATTTGAAATCTTTAGGAACATGGCCTGCTGG
   Consensus ......................AAGCTTCACACACCGACCAACATCCTCCTCCTTTCTCTGGCTGTCTCAGACTTTCTTGTGGGTCTCTTGCTGATGCCATTTGAAATCTTTAGGAACATGGCCTGCTGG

             3381 3390      3400      3410      3420      3430      3440      3450      3460      3470      3480      3490      3500      3510
             |--------+---------+---------+---------+---------+---------+---------+---------+---------+---------+---------+---------+---------|
contig022341 GTACTTGGTGATCTTATGTGTTCTGTTTCTTGGTATCTGACTGGTAACATTGCCTTTGCCTCAACAGGGAACATAGTTCTCATATCAATTGACCGTTATGTGGCTATTTGTCACCCTCTGCATTACCCCA
TilTARs.A032 GTACTTGGTGATCTTATGTGTTCTGTTTCTTGGTATCTGACTGGTAACATTGCCTTTGCCTCAACAGGGAACATAGTTCTCATATCAATTGACCGTTATGTGGCTATTTGTCACCCTCTGCATTACCCCA
   Consensus GTACTTGGTGATCTTATGTGTTCTGTTTCTTGGTATCTGACTGGTAACATTGCCTTTGCCTCAACAGGGAACATAGTTCTCATATCAATTGACCGTTATGTGGCTATTTGTCACCCTCTGCATTACCCCA

             3511 3520      3530      3540      3550      3560      3570      3580      3590      3600      3610      3620      3630      3640
             |--------+---------+---------+---------+---------+---------+---------+---------+---------+---------+---------+---------+---------|
contig022341 CCAGAATTACTGTGGCAAGAGTCAAACTTAGTGTTTCTCTGTTTTGGTTATTTGCCATTTTCTACAGCAGTCTCTATATGAAGGATGTACTGATAAATCCAGGCAGGTATAATTCCTGCTATGGAGAGTG
TilTARs.A032 CCAGAATTACTGTGGCAAGAGTCAAACTTAGTGTTTCTCTGTTTTGGTTATTTGCCATTTTCTACAGCAGTCTCTATATGAAGGATGTACTGATAAATCCAGGCAGGTATAATTCCTGCTATGGAGAGTG
   Consensus CCAGAATTACTGTGGCAAGAGTCAAACTTAGTGTTTCTCTGTTTTGGTTATTTGCCATTTTCTACAGCAGTCTCTATATGAAGGATGTACTGATAAATCCAGGCAGGTATAATTCCTGCTATGGAGAGTG

             3641 3650      3660      3670      3680      3690      3700      3710      3720      3730      3740      3750      3760      3770
             |--------+---------+---------+---------+---------+---------+---------+---------+---------+---------+---------+---------+---------|
contig022341 TGTGTTTATCATTAATGATATTGCAGGGATTTTTGACATGGTTTTATCTTTTTTTGTTCCAGTTCCAGTTATCGTAATTCTGTATATGAGAGTGTTTGTAGTGGCTGTGTCTCAGGCTCGTGCCATGCGC
TilTARs.A032 TGTGTTTATCATTAATGATATTGCAGGGATTTTTGACATGGTTTTATCTTTTTTTGTTCCAGTTCCAGTTATCGTAATTCTGTATATGAGAGTGTTTGTAGTGGCTGTGTCTCAGGCTCGTGCCATGCGC
   Consensus TGTGTTTATCATTAATGATATTGCAGGGATTTTTGACATGGTTTTATCTTTTTTTGTTCCAGTTCCAGTTATCGTAATTCTGTATATGAGAGTGTTTGTAGTGGCTGTGTCTCAGGCTCGTGCCATGCGC

             3771 3780      3790      3800      3810      3820      3830      3840      3850      3860      3870      3880      3890      3900
             |--------+---------+---------+---------+---------+---------+---------+---------+---------+---------+---------+---------+---------|
contig022341 TCTCATGTTACAGCTGTCACACTGCAGCGTTCATTGAATCAAACAAACAAATCTGAGTTGAAAGCAGCCAGGACTCTTGGAATTCTTGTAGTCGTGTTTCTAATATGCTACTGTCCATTCTTCTGCTTTT
TilTARs.A032 TCTCATGTTACAGCTGTCACACTGCAGCGTTCATTGAATCAAACAAACAAATCTGAGTTGAAAGCAGCCAGGACTCTTGGAATTCTTGTAGTCGTGTTTCTAATATGCTACTGTCCATTCTTCTGCTTTT
   Consensus TCTCATGTTACAGCTGTCACACTGCAGCGTTCATTGAATCAAACAAACAAATCTGAGTTGAAAGCAGCCAGGACTCTTGGAATTCTTGTAGTCGTGTTTCTAATATGCTACTGTCCATTCTTCTGCTTTT

             3901 3910      3920      3930      3940      3950      3960      3970      3980      3990      4000      4010      4020      4030
             |--------+---------+---------+---------+---------+---------+---------+---------+---------+---------+---------+---------+---------|
contig022341 CTTTTGGTGTAGAAGACATGGTTAATAATCCATCTACATTTTTTGTGATCATGGTCTTTTACTTTAATTCTTGTCTAAACCCTTTGATCTATGCCCTGTTTTACCCCTGGTTTAGAAATGCTGTAAAACT
TilTARs.A032 CTTTTGGTGTAGAAGACATGGTTAATAATCCATCTACATTTTTTGTGATCATGGTCTTTTACTTTAATTCTTGTCTAAACCCTTTGATCTATGCCCTGTTTTACCCCTGGTTTAGAAATGCTGTAAAACT
   Consensus CTTTTGGTGTAGAAGACATGGTTAATAATCCATCTACATTTTTTGTGATCATGGTCTTTTACTTTAATTCTTGTCTAAACCCTTTGATCTATGCCCTGTTTTACCCCTGGTTTAGAAATGCTGTAAAACT

             4031 4040      4050      4060      4070      4080      4090      4100      4110      4120      4130      4140      4150      4160
             |--------+---------+---------+---------+---------+---------+---------+---------+---------+---------+---------+---------+---------|
contig022341 TATCATCACACTGCAGATTTTCAAACATGAAACTAGTGAAGCCAACATACTGTAATAAGACTAGTTAGAGAAAACCATGCTGCCATAATATAGTAAACCTGTAACTTCATGAATCTATTATCTACCCTAG
TilTARs.A032 TATCATCACACTGCAGATTTTCAAACATGAAACTAGTGAAGCCAACATACTGTAA
   Consensus TATCATCACACTGCAGATTTTCAAACATGAAACTAGTGAAGCCAACATACTGTAA...........................................................................
```

```
           1691 1700      1710      1720      1730      1740      1750      1760      1770      1780      1790      1800      1810      1820
contig022343  TTAGCTTTATGAGAAAAAGTGTTTATTCAAGAGTTGCTGCCTGATGATGGAGATAGAGAAAGGAGCTGAGCTCTGTTTTCCGCAACTCCTCAACAGTTCCTGCAGGAAGCCGACACTTCACTGGTCCAGA
TilTARs.A033                                                ATGGAGATAGAGAAAGGAGCTGAGCTCTGTTTTCCGCAACTCCTCAACAGTTCCTGCAGGAAGCCGACACTTCACTGGTCCAGA
   Consensus  ..............................................ATGGAGATAGAGAAAGGAGCTGAGCTCTGTTTTCCGCAACTCCTCAACAGTTCCTGCAGGAAGCCGACACTTCACTGGTCCAGA

           1821 1830      1840      1850      1860      1870      1880      1890      1900      1910      1920      1930      1940      1950
contig022343  GCTTTGCTTCTGTACATTGTGCTGTCCTGCATCTCTCTGATCACTGCAGCTCTAAACCTTCTTGTCATCATCTCAGTCTCTTACTTCAGGTAGTGACTTTTTTAACTGCAGATAAGTATTGGTATTTTAT
TilTARs.A033  GCTTTGCTTCTGTACATTGTGCTGTCCTGCATCTCTCTGATCACTGCAGCTCTAAACCTTCTTGTCATCATCTCAGTCTCTTACTTCAGG----------------------------------------
   Consensus  GCTTTGCTTCTGTACATTGTGCTGTCCTGCATCTCTCTGATCACTGCAGCTCTAAACCTTCTTGTCATCATCTCAGTCTCTTACTTCAGG........................................

           1951 1960      1970      1980      1990      2000      2010      2020      2030      2040      2050      2060      2070      2080
contig022343  TGAATTATATAACAGGGTCAGTACATCTTAACACTGCTGCATTTACACAAAAGGGAAATAATGCAATGTATATAGGACTTAGCTACAGCTACTTTTCAGAGCATGTCCCTGGTTTGACTTTTAAGACAGA
TilTARs.A033  ----------------------------------------------------------------------------------------------------------------------------------
   Consensus  ..................................................................................................................................

           2081 2090      2100      2110      2120      2130      2140      2150      2160      2170      2180      2190      2200      2210
contig022343  GTACATTAAAACAAATATTTGATCAGACAGTTAGGAAACAGCAACAATGATAATAATAGAAGCAAGAGCAACTATTGCAAAAAATAGAAACATTTAAGAATGATCATTAATAATGACAGAAGACCCAATT
TilTARs.A033  ----------------------------------------------------------------------------------------------------------------------------------
   Consensus  ..................................................................................................................................

           2211 2220      2230      2240      2250      2260      2270      2280      2290      2300      2310      2320      2330      2340
contig022343  CAGTGATCACAGATGTGCTTTTAAGTCATAATTTTGCCTTTGGATTGAAAAACTATATTATCTGTTAATGTTTTTATTTCTAGTGGTAAAGTGTTCTAAACCTGGCGGCCTTTAACTGTAAAAGAGGACG
TilTARs.A033  ----------------------------------------------------------------------------------------------------------------------------------
   Consensus  ..................................................................................................................................

           2341 2350      2360      2370      2380      2390      2400      2410      2420      2430      2440      2450      2460      2470
contig022343  GACCAAATCAGTTTCTACATTTTGGCAGCTTAAAATCCCATAGTAGCATTCTAGCAATTACATTTAAAAGTCAACTTTAATAAAGAAACTTTTATGAATTTTTTTTTTTATGAAAAGCTTAACATTAGTT
TilTARs.A033  ----------------------------------------------------------------------------------------------------------------------------------
   Consensus  ..................................................................................................................................

           2471 2480      2490      2500      2510      2520      2530      2540      2550      2560      2570      2580      2590      2600
contig022343  AACACAAGTTATATATATATATATATGTATATATATATATATATATATATATATATATATATATAAACTTTGATTGACGTTAATTTTTAATGTACCTAGACTGAATCATGCCTACTTTAACATTTTAA
TilTARs.A033  ----------------------------------------------------------------------------------------------------------------------------------
   Consensus  ..................................................................................................................................

           2601 2610      2620      2630      2640      2650      2660      2670      2680      2690      2700      2710      2720      2730
contig022343  CCTTTTGAAATGCTTTTTTTTTCTGTTTCCATCCAGGCAGCTCCACACACCAAGTAACATCGTCCTACTCTCTCTGGCTGTTTCAGACTTTCTAATTGGTCTACTTATGATGCCATTTGAAATCATTAGA
TilTARs.A033  -------------------------------------CAGCTCCACACACCAAGTAACATCGTCCTACTCTCTCTGGCTGTTTCAGACTTTCTAATTGGTCTACTTATGATGCCATTTGAAATCATTAGA
   Consensus  .....................................CAGCTCCACACACCAAGTAACATCGTCCTACTCTCTCTGGCTGTTTCAGACTTTCTAATTGGTCTACTTATGATGCCATTTGAAATCATTAGA

           2731 2740      2750      2760      2770      2780      2790      2800      2810      2820      2830      2840      2850      2860
contig022343  AACACAGGCTGCTGGGTACTTGGTGATCTTATGTGTTCTGTTTATATTTTTCTGACCATCAGTCTTGTCTGTGCTTCAATACTTAACATAGTGCTAATATCAATTGACCGCTATGTGGCTATTTGTGACC
TilTARs.A033  AACACAGGCTGCTGGGTACTTGGTGATCTTATGTGTTCTGTTTATATTTTTCTGACCATCAGTCTTGTCTGTGCTTCAATACTTAACATAGTGCTAATATCAATTGACCGCTATGTGGCTATTTGTGACC
   Consensus  AACACAGGCTGCTGGGTACTTGGTGATCTTATGTGTTCTGTTTATATTTTTCTGACCATCAGTCTTGTCTGTGCTTCAATACTTAACATAGTGCTAATATCAATTGACCGCTATGTGGCTATTTGTGACC

           2861 2870      2880      2890      2900      2910      2920      2930      2940      2950      2960      2970      2980      2990
contig022343  CTCTGCACTATCCCACCAGACTGACGGTGGCAAGAGTCAAACTCAGTGTTTGTCTGTGTTGGTTTTATTCTACTTTCTACAGCAGTCTTTACGCGAAGAATGTCCTGATTGAACCAGGCAGGTATAATTT
TilTARs.A033  CTCTGCACTATCCCACCAGACTGACGGTGGCAAGAGTCAAACTCAGTGTTTGTCTGTGTTGGTTTTATTCTACTTTCTACAGCAGTCTTTACGCGAAGAATGTCCTGATTGAACCAGGCAGGTATAATTT
   Consensus  CTCTGCACTATCCCACCAGACTGACGGTGGCAAGAGTCAAACTCAGTGTTTGTCTGTGTTGGTTTTATTCTACTTTCTACAGCAGTCTTTACGCGAAGAATGTCCTGATTGAACCAGGCAGGTATAATTT

           2991 3000      3010      3020      3030      3040      3050      3060      3070      3080      3090      3100      3110      3120
contig022343  CTGCTTTGGAGAGTGTGTGTTTTTCAGCAGTAATATTGCTGTTGTTGTTGACCTTGTTTTATTCTTTATTGTTCCAGTTTCTGTCATTGTAGTTCTGTATATGAGAGTATTTGTGGTGGCTGTGTCTCAG
TilTARs.A033  CTGCTTTGGAGAGTGTGTGTTTTTCAGCAGTAATATTGCTGTTGTTGTTGACCTTGTTTTATTCTTTATTGTTCCAGTTTCTGTCATTGTAGTTCTGTATATGAGAGTATTTGTGGTGGCTGTGTCTCAG
   Consensus  CTGCTTTGGAGAGTGTGTGTTTTTCAGCAGTAATATTGCTGTTGTTGTTGACCTTGTTTTATTCTTTATTGTTCCAGTTTCTGTCATTGTAGTTCTGTATATGAGAGTATTTGTGGTGGCTGTGTCTCAG

           3121 3130      3140      3150      3160      3170      3180      3190      3200      3210      3220      3230      3240      3250
contig022343  GCTCATGCCATGCGCTCTCATGTTACATCTGTCACACTTCAGCGTTCAGCAAATCAAGCAAACAAATCTGAGTTGAAAGCAGCCAGGACTCTTGGGGTTCTTGTAGTTGTGTTTCTGGCAACCTTTTGTC
TilTARs.A033  GCTCATGCCATGCGCTCTCATGTTACATCTGTCACACTTCAGCGTTCAGCAAATCAAGCAAACAAATCTGAGTTGAAAGCAGCCAGGACTCTTGGGGTTCTTGTAGTTGTGTTTCTGGCAACCTTTTGTC
   Consensus  GCTCATGCCATGCGCTCTCATGTTACATCTGTCACACTTCAGCGTTCAGCAAATCAAGCAAACAAATCTGAGTTGAAAGCAGCCAGGACTCTTGGGGTTCTTGTAGTTGTGTTTCTGGCAACCTTTTGTC

           3251 3260      3270      3280      3290      3300      3310      3320      3330      3340      3350      3360      3370      3380
contig022343  CATATTACTGCTACTCTCTTGTTGATGAAAATGCTGTCAATGATCCCTCTGCATCTTTTGTGATCATTATCTTTTACATTAACTCTTGTCTAAACCCATTGATCTACGCCTTGTTTTACCCCTGGTTTAG
TilTARs.A033  CATATTACTGCTACTCTCTTGTTGATGAAAATGCTGTCAATGATCCCTCTGCATCTTTTGTGATCATTATCTTTTACATTAACTCTTGTCTAAACCCATTGATCTACGCCTTGTTTTACCCCTGGTTTAG
   Consensus  CATATTACTGCTACTCTCTTGTTGATGAAAATGCTGTCAATGATCCCTCTGCATCTTTTGTGATCATTATCTTTTACATTAACTCTTGTCTAAACCCATTGATCTACGCCTTGTTTTACCCCTGGTTTAG

           3381 3390      3400      3410      3420      3430      3440      3450      3460      3470      3480      3490      3500      3510
contig022343  AAATGCTGTTAAACTCATCATCACTTTGCAGATCTTCAAGTGTGACACAAGTGAGGCCAACATACTATAAAAAGATAAAGAAGCTCATGACACTAAGAATTTAAATTAATAAATAACTACACACAGTAAT
TilTARs.A033  AAATGCTGTTAAACTCATCATCACTTTGCAGATCTTCAAGTGTGACACAAGTGAGGCCAACATACTATAA
   Consensus  AAATGCTGTTAAACTCATCATCACTTTGCAGATCTTCAAGTGTGACACAAGTGAGGCCAACATACTATAA............................................................

           3511 3520      3530      3540      3550      3560      3570      3580      3590      3600      3610      3620      3630      3640
```

```
             1561      1570      1580      1590      1600      1610      1620      1630      1640      1650      1660      1670      1680      1690
contig022345 GTCAATGTGTGTGTGTGTGTGTGTGTGTGTGAGGGGAGGGGAGGGGGGGCAATATATCAGCTCTGTGACAAAACCTTTAGAGAGCTGCATCTAACTGATTATGGAGATGCAGAAAGGAACTGAGCTCTGT
TilTARs.A034                                                                                                     ATGGAGATGCAGAAAGGAACTGAGCTCTGT
   Consensus ....................................................................................................ATGGAGATGCAGAAAGGAACTGAGCTCTGT

             1691      1700      1710      1720      1730      1740      1750      1760      1770      1780      1790      1800      1810      1820
contig022345 TTTCCACAGCTCCTCAACAATTCCTGCAGGAAGCCGATACGTCACTGGTCGAAAGCTGTTCTTCTGTACATTGTGCTGTTCTGCATCTCTCTGATCACTGCTGCTCTAAACCTACTGGTCATCATCTCCA
TilTARs.A034 TTTCCACAGCTCCTCAACAATTCCTGCAGGAAGCCGATACGTCACTGGTCGAAAGCTGTTCTTCTGTACATTGTGCTGTTCTGCATCTCTCTGATCACTGCTGCTCTAAACCTACTGGTCATCATCTCCA
   Consensus TTTCCACAGCTCCTCAACAATTCCTGCAGGAAGCCGATACGTCACTGGTCGAAAGCTGTTCTTCTGTACATTGTGCTGTTCTGCATCTCTCTGATCACTGCTGCTCTAAACCTACTGGTCATCATCTCCA

             1821      1830      1840      1850      1860      1870      1880      1890      1900      1910      1920      1930      1940      1950
contig022345 TCTCTCACTTCAAGTAAAGATTAACTTTTCAACTGAAACTTTTCAACTGAAATTTTAAGATTTACAACAGTGGCAAAAAATGAATGAAGTGAAATAGATAAATATCTTTGGCTTGAACATGACTGTTATG
TilTARs.A034 TCTCTCACTTCAAG--------------------------------------------------------------------------------------------------------------------
   Consensus TCTCTCACTTCAAG....................................................................................................................

             1951      1960      1970      1980      1990      2000      2010      2020      2030      2040      2050      2060      2070      2080
contig022345 TTGACTAAGCTTTTAACATGATTCACTCATGACATGATCACTGCTGCTTTCAACTGTCTTGTCTCAATTGTCAATCTCAGTCTTTCATTTCAGACAGAAATAAACTTCTCATATGAAATTACAGTTTAAC
TilTARs.A034 ----------------------------------------------------------------------------------------------------------------------------------
   Consensus ..................................................................................................................................

             2081      2090      2100      2110      2120      2130      2140      2150      2160      2170      2180      2190      2200      2210
contig022345 TTTTAGAAATTTTAACACATTCGAAGATGTTAATAAACTGATTTGACTTTATTAACGTTTAATCTATTTTTCCATTTCCCTGCAGGCAGCTTCACACACCCAGTAACATCCTCCTCCTCTCTCTGGCTGT
TilTARs.A034 ----------------------------------------------------------------------------------------CAGCTTCACACACCCAGTAACATCCTCCTCCTCTCTCTGGCTGT
   Consensus ........................................................................................CAGCTTCACACACCCAGTAACATCCTCCTCCTCTCTCTGGCTGT

             2211      2220      2230      2240      2250      2260      2270      2280      2290      2300      2310      2320      2330      2340
contig022345 GTCAGACTTTCTTGTGGGTCTTCTGCTGATGCCATTAGAAATCTTTAGAAGCACGGCCTGCTGGGTACTTGGTGATCTTATGTGTTCTGTTTATATTTATATGACTGTAAACATTACCTGTGCTTCAATA
TilTARs.A034 GTCAGACTTTCTTGTGGGTCTTCTGCTGATGCCATTAGAAATCTTTAGAAGCACGGCCTGCTGGGTACTTGGTGATCTTATGTGTTCTGTTTATATTTATATGACTGTAAACATTACCTGTGCTTCAATA
   Consensus GTCAGACTTTCTTGTGGGTCTTCTGCTGATGCCATTAGAAATCTTTAGAAGCACGGCCTGCTGGGTACTTGGTGATCTTATGTGTTCTGTTTATATTTATATGACTGTAAACATTACCTGTGCTTCAATA

             2341      2350      2360      2370      2380      2390      2400      2410      2420      2430      2440      2450      2460      2470
contig022345 GGGAACATAGTTCTTATATCAGTTGACCGTTATGTAGCTATTTGTGACCCTCTGCATTACCCAACCAGAATTACTGTAGCGAGAATCAAACTCAGCATTTGTTTCTGTTGGTTTTATACTATTTTCTACA
TilTARs.A034 GGGAACATAGTTCTTATATCAGTTGACCGTTATGTAGCTATTTGTGACCCTCTGCATTACCCAACCAGAATTACTGTAGCGAGAATCAAACTCAGCATTTGTTTCTGTTGGTTTTATACTATTTTCTACA
   Consensus GGGAACATAGTTCTTATATCAGTTGACCGTTATGTAGCTATTTGTGACCCTCTGCATTACCCAACCAGAATTACTGTAGCGAGAATCAAACTCAGCATTTGTTTCTGTTGGTTTTATACTATTTTCTACA

             2471      2480      2490      2500      2510      2520      2530      2540      2550      2560      2570      2580      2590      2600
contig022345 GCAGTCTTTATACAAAGGATATCCTGATTGAACCGGGCAGGTATAACTCCTGCTGCGGAGAGTGTGTGTTGGTCATCAATGATATTGTAGGGATTGTTGACCTTGTCTTAACTTTTATTGTTCCAGTTGC
TilTARs.A034 GCAGTCTTTATACAAAGGATATCCTGATTGAACCGGGCAGGTATAACTCCTGCTGCGGAGAGTGTGTGTTGGTCATCAATGATATTGTAGGGATTGTTGACCTTGTCTTAACTTTTATTGTTCCAGTTGC
   Consensus GCAGTCTTTATACAAAGGATATCCTGATTGAACCGGGCAGGTATAACTCCTGCTGCGGAGAGTGTGTGTTGGTCATCAATGATATTGTAGGGATTGTTGACCTTGTCTTAACTTTTATTGTTCCAGTTGC

             2601      2610      2620      2630      2640      2650      2660      2670      2680      2690      2700      2710      2720      2730
contig022345 TGTCATTATAGTTCTGTATATGAGAGTGTTTGTGGCAGCTGTGTCTCAGGCTCGTGCCATGCGCTCTCATGTTACAGCTGTCACATTGCAGCGTCCACTGAATCAAACAAACAAATCTGAGTTGAAAGCA
TilTARs.A034 TGTCATTATAGTTCTGTATATGAGAGTGTTTGTGGCAGCTGTGTCTCAGGCTCGTGCCATGCGCTCTCATGTTACAGCTGTCACATTGCAGCGTCCACTGAATCAAACAAACAAATCTGAGTTGAAAGCA
   Consensus TGTCATTATAGTTCTGTATATGAGAGTGTTTGTGGCAGCTGTGTCTCAGGCTCGTGCCATGCGCTCTCATGTTACAGCTGTCACATTGCAGCGTCCACTGAATCAAACAAACAAATCTGAGTTGAAAGCA

             2731      2740      2750      2760      2770      2780      2790      2800      2810      2820      2830      2840      2850      2860
contig022345 GCCAGGACTCTTGGAGTTCTTGTAGTTGTGTTTCTGGCATGCTACTTTCCATTCTACTGCTATGCCTCTGTTGTAGAAGACATGGTTAATAATCCATCTGCATTGTTTGTGTTCATGGTCTTTTATTTTA
TilTARs.A034 GCCAGGACTCTTGGAGTTCTTGTAGTTGTGTTTCTGGCATGCTACTTTCCATTCTACTGCTATGCCTCTGTTGTAGAAGACATGGTTAATAATCCATCTGCATTGTTTGTGTTCATGGTCTTTTATTTTA
   Consensus GCCAGGACTCTTGGAGTTCTTGTAGTTGTGTTTCTGGCATGCTACTTTCCATTCTACTGCTATGCCTCTGTTGTAGAAGACATGGTTAATAATCCATCTGCATTGTTTGTGTTCATGGTCTTTTATTTTA

             2861      2870      2880      2890      2900      2910      2920      2930      2940      2950      2960      2970      2980      2990
contig022345 ACTCTTGCCTAAACCCTTTGATCTATGCCCTGTTTTACCCCTGGTTTAGAAATGCTGTAAAACTTATTGTCACATTGCAGTTCTTAAAGCATGGCACCTGTGAAGCCAACATATTATAGGAACATTTTCT
TilTARs.A034 ACTCTTGCCTAAACCCTTTGATCTATGCCCTGTTTTACCCCTGGTTTAGAAATGCTGTAAAACTTATTGTCACATTGCAGTTCTTAAAGCATGGCACCTGTGAAGCCAACATATTATAG
   Consensus ACTCTTGCCTAAACCCTTTGATCTATGCCCTGTTTTACCCCTGGTTTAGAAATGCTGTAAAACTTATTGTCACATTGCAGTTCTTAAAGCATGGCACCTGTGAAGCCAACATATTATAG...........
```

```
            4031 4040      4050      4060      4070      4080      4090      4100      4110      4120      4130      4140      4150      4160
            |--------+---------+---------+---------+---------+---------+---------+---------+---------+---------+---------+---------+---------|
contig022349 AGAGCTGCAGCTGCCTGATGATGGAGATACAGAAAGGAGCCGAGCTCTGTTTTCCACAGCTCCTCAACAGTTCCTGCAGGAACCCAACACTTCACTGGTCCAGATCTTTGCTCCTGAACATTGTGCTGTC
 TilTARs.A035                     ATGGAGATACAGAAAGGAGCCGAGCTCTGTTTTCCACAGCTCCTCAACAGTTCCTGCAGGAACCCAACACTTCACTGGTCCAGATCTTTGCTCCTGAACATTGTGCTGTC
    Consensus ....................ATGGAGATACAGAAAGGAGCCGAGCTCTGTTTTCCACAGCTCCTCAACAGTTCCTGCAGGAACCCAACACTTCACTGGTCCAGATCTTTGCTCCTGAACATTGTGCTGTC

            4161 4170      4180      4190      4200      4210      4220      4230      4240      4250      4260      4270      4280      4290
            |--------+---------+---------+---------+---------+---------+---------+---------+---------+---------+---------+---------+---------|
contig022349 CTGCTTCTCTCCGATCACTGCTGCTCTAAACCTTCTGGTCATCATCTCAGTCTCCCACTTCAGGCAGAGATTAACCTCTTAGTTTAAATTGCAAAGTGTTAGAACTGTTAAAATAATAACTAACTGATTA
 TilTARs.A035 CTGCTTCTCTCCGATCACTGCTGCTCTAAACCTTCTGGTCATCATCTCAGTCTCCCACTTCAGGCAG---------------------------------------------------------------
    Consensus CTGCTTCTCTCCGATCACTGCTGCTCTAAACCTTCTGGTCATCATCTCAGTCTCCCACTTCAGGCAG...............................................................

            4291 4300      4310      4320      4330      4340      4350      4360      4370      4380      4390      4400      4410      4420
            |--------+---------+---------+---------+---------+---------+---------+---------+---------+---------+---------+---------+---------|
contig022349 ATGAAGTAAATATTTCACATAATTGGTGTATTTTAACCATGTCTAAATGTGTTATTTTCCCAGACTGAATCATTAATTTCTTATGGAACTTTTTCACTCATGGAATTTTCACTAAAGACTGAATTTTGCA
 TilTARs.A035 ----------------------------------------------------------------------------------------------------------------------------------
    Consensus ..................................................................................................................................

            4421 4430      4440      4450      4460      4470      4480      4490      4500      4510      4520      4530      4540      4550
            |--------+---------+---------+---------+---------+---------+---------+---------+---------+---------+---------+---------+---------|
contig022349 TTGCTGATGTGTAATATTTTATTTTCTGTTTCTCTCCAGGCAGCTGCACACACCCACTAACATCCTGCTCCTCTCTCTGGCTGTCTCAGACTTTTTTGTGGGTCTCCTTTTGATGCCATTAGAAATTTTT
 TilTARs.A035 -------------------------------------------CTGCACACACCCACTAACATCCTGCTCCTCTCTCTGGCTGTCTCAGACTTTTTTGTGGGTCTCCTTTTGATGCCATTAGAAATTTTT
    Consensus ...........................................CTGCACACACCCACTAACATCCTGCTCCTCTCTCTGGCTGTCTCAGACTTTTTTGTGGGTCTCCTTTTGATGCCATTAGAAATTTTT

            4551 4560      4570      4580      4590      4600      4610      4620      4630      4640      4650      4660      4670      4680
            |--------+---------+---------+---------+---------+---------+---------+---------+---------+---------+---------+---------+---------|
contig022349 AGAAACACAACTTGCTGGATACTTGGTGACATTATGTGTTCTGTTTATTGGTATCTAAACTTCAGTTTCATCTGTGTTACAATAGGGAACATACTTTTAATATCAGTTGACCGCTATGTAGCAATTTGTT
 TilTARs.A035 AGAAACACAACTTGCTGGATACTTGGTGACATTATGTGTTCTGTTTATTGGTATCTAAACTTCAGTTTCATCTGTGTTACAATAGGGAACATACTTTTAATATCAGTTGACCGCTATGTAGCAATTTGTT
    Consensus AGAAACACAACTTGCTGGATACTTGGTGACATTATGTGTTCTGTTTATTGGTATCTAAACTTCAGTTTCATCTGTGTTACAATAGGGAACATACTTTTAATATCAGTTGACCGCTATGTAGCAATTTGTT

            4681 4690      4700      4710      4720      4730      4740      4750      4760      4770      4780      4790      4800      4810
            |--------+---------+---------+---------+---------+---------+---------+---------+---------+---------+---------+---------+---------|
contig022349 ACCCTCTGCATTATCCCAACAGAATTACTGTGGCGAGAGTGAAACGCAGTGTTTGTCTGTGTTGGTTTTATGCTATTTTCTACTGTAGTTTTTATACAAAGGACATCCTGATTCAACCAGGCAGGTATAA
 TilTARs.A035 ACCCTCTGCATTATCCCAACAGAATTACTGTGGCGAGAGTGAAACGCAGTGTTTGTCTGTGTTGGTTTTATGCTATTTTCTACTGTAGTTTTTATACAAAGGACATCCTGATTCAACCAGGCAGGTATAA
    Consensus ACCCTCTGCATTATCCCAACAGAATTACTGTGGCGAGAGTGAAACGCAGTGTTTGTCTGTGTTGGTTTTATGCTATTTTCTACTGTAGTTTTTATACAAAGGACATCCTGATTCAACCAGGCAGGTATAA

            4811 4820      4830      4840      4850      4860      4870      4880      4890      4900      4910      4920      4930      4940
            |--------+---------+---------+---------+---------+---------+---------+---------+---------+---------+---------+---------+---------|
contig022349 TTCCTGCTATGGAGAGTGTGTGTTTTTCATTGATGATATGGCAGGGATTATTGACCTTGTTTTATTTTTTTTAATTCCGGTAATGGTCATCATAGTTCTTTATACGAGAGTATTTGTGGTGGCTGTGTCC
 TilTARs.A035 TTCCTGCTATGGAGAGTGTGTGTTTTTCATTGATGATATGGCAGGGATTATTGACCTTGTTTTATTTTTTTTAATTCCGGTAATGGTCATCATAGTTCTTTATACGAGAGTATTTGTGGTGGCTGTGTCC
    Consensus TTCCTGCTATGGAGAGTGTGTGTTTTTCATTGATGATATGGCAGGGATTATTGACCTTGTTTTATTTTTTTTAATTCCGGTAATGGTCATCATAGTTCTTTATACGAGAGTATTTGTGGTGGCTGTGTCC

            4941 4950      4960      4970      4980      4990      5000      5010      5020      5030      5040      5050      5060      5070
            |--------+---------+---------+---------+---------+---------+---------+---------+---------+---------+---------+---------+---------|
contig022349 CAGGCTCGCGCCATGCGCTCTCATGTTACAGCTGTCACACTTCAGCGTTCAGCAAATCAAGCAAACAAATCTGAGCTGAAAGCAGCCAGGACTCTGGGGGTTCTTGTAGTTGTGTTTCTTTTATGCTACT
 TilTARs.A035 CAGGCTCGCGCCATGCGCTCTCATGTTACAGCTGTCACACTTCAGCGTTCAGCAAATCAAGCAAACAAATCTGAGCTGAAAGCAGCCAGGACTCTGGGGGTTCTTGTAGTTGTGTTTCTTTTATGCTACT
    Consensus CAGGCTCGCGCCATGCGCTCTCATGTTACAGCTGTCACACTTCAGCGTTCAGCAAATCAAGCAAACAAATCTGAGCTGAAAGCAGCCAGGACTCTGGGGGTTCTTGTAGTTGTGTTTCTTTTATGCTACT

            5071 5080      5090      5100      5110      5120      5130      5140      5150      5160      5170      5180      5190      5200
            |--------+---------+---------+---------+---------+---------+---------+---------+---------+---------+---------+---------+---------|
contig022349 GTCCATCTTATTTCTTTACCTTTTTTGCAGAAGGCATGCTCAACGATCCAGCAACTGTTTTCTTTATAACTGTCATTTATTTTAACTCTTGTCTAAACCCTTTGATCTATGCCCTGTTTTACTCCTGGTT
 TilTARs.A035 GTCCATCTTATTTCTTTACCTTTTTTGCAGAAGGCATGCTCAACGATCCAGCAACTGTTTTCTTTATAACTGTCATTTATTTTAACTCTTGTCTAAACCCTTTGATCTATGCCCTGTTTTACTCCTGGTT
    Consensus GTCCATCTTATTTCTTTACCTTTTTTGCAGAAGGCATGCTCAACGATCCAGCAACTGTTTTCTTTATAACTGTCATTTATTTTAACTCTTGTCTAAACCCTTTGATCTATGCCCTGTTTTACTCCTGGTT

            5201 5210      5220      5230      5240      5250      5260      5270      5280      5290      5300      5310      5320      5330
            |--------+---------+---------+---------+---------+---------+---------+---------+---------+---------+---------+---------+---------|
contig022349 TAGAAATGCTGTTAAACGTATCATCACTCTCCAGATCGTTACACATGACACAAATGAAGCCAACATATTATGAAAACACTATGAAACATGTTTTACTAAGATTTAAAGTCAGTAAATTGCTTCATCTGAA
 TilTARs.A035 TAGAAATGCTGTTAAACGTATCATCACTCTCCAGATCGTTACACATGACACAAATGAAGCCAACATATTATGA
    Consensus TAGAAATGCTGTTAAACGTATCATCACTCTCCAGATCGTTACACATGACACAAATGAAGCCAACATATTATGA.........................................................
```

```
             4551  4560      4570      4580      4590      4600      4610      4620      4630      4640      4650      4660      4670      4680
             |--------+---------+---------+---------+---------+---------+---------+---------+---------+---------+---------+---------+---------|
contig022353 TCTGATGATGGAGATACCGAAAGGAGTCGAGCTCTGTTTCCCACAACTCCTCAACAGTTCCTGCAGGAAGCCGACACTTCACTGGTCCAAAGCTGTGCTCCTGAACATTGTGCTCTCATGTATCTCTCTG
TilTARs.A036        ATGGAGATACCGAAAGGAGTCGAGCTCTGTTTCCCACAACTCCTCAACAGTTCCTGCAGGAAGCCGACACTTCACTGGTCCAAAGCTGTGCTCCTGAACATTGTGCTCTCATGTATCTCTCTG
   Consensus .......ATGGAGATACCGAAAGGAGTCGAGCTCTGTTTCCCACAACTCCTCAACAGTTCCTGCAGGAAGCCGACACTTCACTGGTCCAAAGCTGTGCTCCTGAACATTGTGCTCTCATGTATCTCTCTG

             4681  4690      4700      4710      4720      4730      4740      4750      4760      4770      4780      4790      4800      4810
             |--------+---------+---------+---------+---------+---------+---------+---------+---------+---------+---------+---------+---------|
contig022353 CTCACTGCTGCTCTAAACCTTCTTGTCATCATCTCCGTCTCCCACTTCAGGCAGAGTTTAACTTTTCAACTGAAACTACAGTTTAAGTTTTAGAAGTTTTTGAATTGAATCATGCCTTATTAGTGCAGAA
TilTARs.A036 CTCACTGCTGCTCTAAACCTTCTTGTCATCATCTCCGTCTCCCACTTCAGGCAG----------------------------------------------------------------------------
   Consensus CTCACTGCTGCTCTAAACCTTCTTGTCATCATCTCCGTCTCCCACTTCAGGCAG............................................................................

             4811  4820      4830      4840      4850      4860      4870      4880      4890      4900      4910      4920      4930      4940
             |--------+---------+---------+---------+---------+---------+---------+---------+---------+---------+---------+---------+---------|
contig022353 TTTTCACTGAAGACATATTGACATTAACAATGCGCATTATATTCTTTTCTGTCTCCCTGCAGGCAGCTGCACACACCCAGTAACATCCTCCTCCTCTCTCTGGCTGTCTCAGACTTTTTTGTGGGTCTCC
TilTARs.A036 ------------------------------------------------------------------CTGCACACACCCAGTAACATCCTCCTCCTCTCTCTGGCTGTCTCAGACTTTTTTGTGGGTCTCC
   Consensus ..................................................................CTGCACACACCCAGTAACATCCTCCTCCTCTCTCTGGCTGTCTCAGACTTTTTTGTGGGTCTCC

             4941  4950      4960      4970      4980      4990      5000      5010      5020      5030      5040      5050      5060      5070
             |--------+---------+---------+---------+---------+---------+---------+---------+---------+---------+---------+---------+---------|
contig022353 TGTTGTTGCCTTTAGAAATCTTTAGAAACACAAGCTGCTGGGTACTTGGTGATCTTATGTGTTCTGCTTATTGGTATTTGACCAGCAACATTATCTGTGCTTCAATAGGGAACATTGTTCTAATATCAGT
TilTARs.A036 TGTTGTTGCCTTTAGAAATCTTTAGAAACACAAGCTGCTGGGTACTTGGTGATCTTATGTGTTCTGCTTATTGGTATTTGACCAGCAACATTATCTGTGCTTCAATAGGGAACATTGTTCTAATATCAGT
   Consensus TGTTGTTGCCTTTAGAAATCTTTAGAAACACAAGCTGCTGGGTACTTGGTGATCTTATGTGTTCTGCTTATTGGTATTTGACCAGCAACATTATCTGTGCTTCAATAGGGAACATTGTTCTAATATCAGT

             5071  5080      5090      5100      5110      5120      5130      5140      5150      5160      5170      5180      5190      5200
             |--------+---------+---------+---------+---------+---------+---------+---------+---------+---------+---------+---------+---------|
contig022353 TGACCGCTATGTGGCTATTTGTGACCCTCTGCATTACCCCACCAGAATTACTTTGCCGAAAGTCAAACTCGGTGTTTGTCTGTGTTGGTTTTATGCTATTTTCTACAGCAGTCTTTATACAAAGGATGTC
TilTARs.A036 TGACCGCTATGTGGCTATTTGTGACCCTCTGCATTACCCCACCAGAATTACTTTGCCGAAAGTCAAACTCGGTGTTTGTCTGTGTTGGTTTTATGCTATTTTCTACAGCAGTCTTTATACAAAGGATGTC
   Consensus TGACCGCTATGTGGCTATTTGTGACCCTCTGCATTACCCCACCAGAATTACTTTGCCGAAAGTCAAACTCGGTGTTTGTCTGTGTTGGTTTTATGCTATTTTCTACAGCAGTCTTTATACAAAGGATGTC

             5201  5210      5220      5230      5240      5250      5260      5270      5280      5290      5300      5310      5320      5330
             |--------+---------+---------+---------+---------+---------+---------+---------+---------+---------+---------+---------+---------|
contig022353 ATGATTGAACCAGGCAGGTATAATTCTTGCTTTGGAGAGTGTGTATTTTTCAGCAGCAATATTGCTATTGTTGTTGACCTTATTTTATCCTTTTTTGGTCCAGTTACTGTTATCATAGTCTTGTATATGA
TilTARs.A036 ATGATTGAACCAGGCAGGTATAATTCTTGCTTTGGAGAGTGTGTATTTTTCAGCAGCAATATTGCTATTGTTGTTGACCTTATTTTATCCTTTTTTGGTCCAGTTACTGTTATCATAGTCTTGTATATGA
   Consensus ATGATTGAACCAGGCAGGTATAATTCTTGCTTTGGAGAGTGTGTATTTTTCAGCAGCAATATTGCTATTGTTGTTGACCTTATTTTATCCTTTTTTGGTCCAGTTACTGTTATCATAGTCTTGTATATGA

             5331  5340      5350      5360      5370      5380      5390      5400      5410      5420      5430      5440      5450      5460
             |--------+---------+---------+---------+---------+---------+---------+---------+---------+---------+---------+---------+---------|
contig022353 GAGTATTTGTGGTGGCTGTGTCTCAGGCTCGTGCCATGCGCTCTCATGTTACATCTGTCACACTTCAGCGTCCACTGAATCAGACAAACAAATCTGAGCTGAAAGCAGCCAGGAGTCTTGGGGTTCTTGT
TilTARs.A036 GAGTATTTGTGGTGGCTGTGTCTCAGGCTCGTGCCATGCGCTCTCATGTTACATCTGTCACACTTCAGCGTCCACTGAATCAGACAAACAAATCTGAGCTGAAAGCAGCCAGGAGTCTTGGGGTTCTTGT
   Consensus GAGTATTTGTGGTGGCTGTGTCTCAGGCTCGTGCCATGCGCTCTCATGTTACATCTGTCACACTTCAGCGTCCACTGAATCAGACAAACAAATCTGAGCTGAAAGCAGCCAGGAGTCTTGGGGTTCTTGT

             5461  5470      5480      5490      5500      5510      5520      5530      5540      5550      5560      5570      5580      5590
             |--------+---------+---------+---------+---------+---------+---------+---------+---------+---------+---------+---------+---------|
contig022353 AGTTGTATTTCTAGCATGCTTCTGTCCACTCTACTGCTACTCTCTTGTTGATGAAAATGCTGTCAATGATCCCTCTGCATCTTTTGTGGTCATTATCTTTTACATTAACTCTTGTCTAAACCCATTGATC
TilTARs.A036 AGTTGTATTTCTAGCATGCTTCTGTCCACTCTACTGCTACTCTCTTGTTGATGAAAATGCTGTCAATGATCCCTCTGCATCTTTTGTGGTCATTATCTTTTACATTAACTCTTGTCTAAACCCATTGATC
   Consensus AGTTGTATTTCTAGCATGCTTCTGTCCACTCTACTGCTACTCTCTTGTTGATGAAAATGCTGTCAATGATCCCTCTGCATCTTTTGTGGTCATTATCTTTTACATTAACTCTTGTCTAAACCCATTGATC

             5591  5600      5610      5620      5630      5640      5650      5660      5670      5680      5690      5700      5710      5720
             |--------+---------+---------+---------+---------+---------+---------+---------+---------+---------+---------+---------+---------|
contig022353 TACGCCTTGTTTTACCCCTGGTTTAGAAATGCTGTTAAACTTATCATCAGTTTGGAGATCTTCAAGTATGACACCAGTGGGGCCAACATACTATAAAAAGAGTATGAAACCTGCTCTACTAAGACATAAA
TilTARs.A036 TACGCCTTGTTTTACCCCTGGTTTAGAAATGCTGTTAAACTTATCATCAGTTTGGAGATCTTCAAGTATGACACCAGTGGGGCCAACATACTATAA
   Consensus TACGCCTTGTTTTACCCCTGGTTTAGAAATGCTGTTAAACTTATCATCAGTTTGGAGATCTTCAAGTATGACACCAGTGGGGCCAACATACTATAA..................................
```

```
Consensus    ..................................................................................................................................

             2341 2350      2360      2370      2380      2390      2400      2410      2420      2430      2440      2450      2460      2470
             |--------+---------+---------+---------+---------+---------+---------+---------+---------+---------+---------+---------+---------|
contig022354 GGAGAGAGCGAGTTTGGAAAAAAAAGAAAGTGCCAAAGGTTGTGTCAACAGAAAGAGGAGGGATGGGACTGGTGGGTGAGGGACAAAATAGATAAATGAGCTCTGTGAGGAAAAGCTTTGATGAGATGATG
TilTARs.A037                                                                                                                                ATG
   Consensus ...............................................................................................................................ATG

             2471 2480      2490      2500      2510      2520      2530      2540      2550      2560      2570      2580      2590      2600
             |--------+---------+---------+---------+---------+---------+---------+---------+---------+---------+---------+---------+---------|
contig022354 GAGATACAGGAAGGACCAGAGCTCTGTTTTCCACAACTCCTCAACGATTCCTGCAGAAAGCAAACACTTCACTATTCCAAAACTATGCTGCTGTATATTGTGATGTCATGCATCTCTCTTATCACTACTG
TilTARs.A037 GAGATACAGGAAGGACCAGAGCTCTGTTTTCCACAACTCCTCAACGATTCCTGCAGAAAGCAAACACTTCACTATTCCAAAACTATGCTGCTGTATATTGTGATGTCATGCATCTCTCTTATCACTACTG
   Consensus GAGATACAGGAAGGACCAGAGCTCTGTTTTCCACAACTCCTCAACGATTCCTGCAGAAAGCAAACACTTCACTATTCCAAAACTATGCTGCTGTATATTGTGATGTCATGCATCTCTCTTATCACTACTG

             2601 2610      2620      2630      2640      2650      2660      2670      2680      2690      2700      2710      2720      2730
             |--------+---------+---------+---------+---------+---------+---------+---------+---------+---------+---------+---------+---------|
contig022354 CTCTAAACCTTCTGGTCATCATCTCAGTCTCCCACTTCAGGCAGAGACCAACTTTGAATTTAAATTACATTAAGGTTTAGATATATGAAAAGTGAGATTTGCATCATTGGAATTAGCAAAGAATAAATTA
TilTARs.A037 CTCTAAACCTTCTGGTCATCATCTCAGTCTCCCACTTCAGGCAG--------------------------------------------------------------------------------------
   Consensus CTCTAAACCTTCTGGTCATCATCTCAGTCTCCCACTTCAGGCAG......................................................................................

             2731 2740      2750      2760      2770      2780      2790      2800      2810      2820      2830      2840      2850      2860
             |--------+---------+---------+---------+---------+---------+---------+---------+---------+---------+---------+---------+---------|
contig022354 AGTGAAACTAATAAATATCTTTGGCTCAACCATGACTGTTAAGTTGTCTAAGGTTCATCGTGTCTTAATAAGGAAACAATTTCACTAAATACATTTTGACACGATTAACACATGACATGATCTCAACTAT
TilTARs.A037 ----------------------------------------------------------------------------------------------------------------------------------
   Consensus ..................................................................................................................................

             2861 2870      2880      2890      2900      2910      2920      2930      2940      2950      2960      2970      2980      2990
             |--------+---------+---------+---------+---------+---------+---------+---------+---------+---------+---------+---------+---------|
contig022354 CTTATCACTATCATTTTGCTGCCCCACCTTGGGCATAGATAAACCTCAGCTGAAACTACAGTCTTTGGACTGTAATTTAATTTAGTGATGGTTAAGTTAGTTTGGCACATTCTGTGTATTTTAAACAAGC
TilTARs.A037 ----------------------------------------------------------------------------------------------------------------------------------
   Consensus ..................................................................................................................................

             2991 3000      3010      3020      3030      3040      3050      3060      3070      3080      3090      3100      3110      3120
             |--------+---------+---------+---------+---------+---------+---------+---------+---------+---------+---------+---------+---------|
contig022354 TGTGCCTTATTTGCCTAAATTGAATCATGTCTTACTAATGAATCATTTCTACAAATGGAATTTTCACTAAAGACAATGTAACATTATTAATGTGCATTATTTATTTTTTCTGTTACGCTGCAGGCAGCTT
TilTARs.A037 -------------------------------------------------------------------------------------------------------------------------------CTT
   Consensus ...............................................................................................................................CTT

             3121 3130      3140      3150      3160      3170      3180      3190      3200      3210      3220      3230      3240      3250
             |--------+---------+---------+---------+---------+---------+---------+---------+---------+---------+---------+---------+---------|
contig022354 CACACACCCAGCAACATCCTCCTCCTCTCTCTGGCCGTTTCAGACTTTCTCGTGGGTCTCCTGTTGATGCCGTTAGAAATTTTAAGAAGCACCACCTGCTGGGTACTAGGTGATTTTATGTGTTTTCTTT
TilTARs.A037 CACACACCCAGCAACATCCTCCTCCTCTCTCTGGCCGTTTCAGACTTTCTCGTGGGTCTCCTGTTGATGCCGTTAGAAATTTTAAGAAGCACCACCTGCTGGGTACTAGGTGATTTTATGTGTTTTCTTT
   Consensus CACACACCCAGCAACATCCTCCTCCTCTCTCTGGCCGTTTCAGACTTTCTCGTGGGTCTCCTGTTGATGCCGTTAGAAATTTTAAGAAGCACCACCTGCTGGGTACTAGGTGATTTTATGTGTTTTCTTT

             3251 3260      3270      3280      3290      3300      3310      3320      3330      3340      3350      3360      3370      3380
             |--------+---------+---------+---------+---------+---------+---------+---------+---------+---------+---------+---------+---------|
contig022354 ATTGGTATCTGACTGGCAACATTACCTGTGTTTCAATAGGTAACATAGTTCTCATATCAGTTGACCGCTATGTGGCTATTTGTGACCCTTTACATTACTCCACCAGAATTACTGTGGCAAAAGTTAAACT
TilTARs.A037 ATTGGTATCTGACTGGCAACATTACCTGTGTTTCAATAGGTAACATAGTTCTCATATCAGTTGACCGCTATGTGGCTATTTGTGACCCTTTACATTACTCCACCAGAATTACTGTGGCAAAAGTTAAACT
   Consensus ATTGGTATCTGACTGGCAACATTACCTGTGTTTCAATAGGTAACATAGTTCTCATATCAGTTGACCGCTATGTGGCTATTTGTGACCCTTTACATTACTCCACCAGAATTACTGTGGCAAAAGTTAAACT

             3381 3390      3400      3410      3420      3430      3440      3450      3460      3470      3480      3490      3500      3510
             |--------+---------+---------+---------+---------+---------+---------+---------+---------+---------+---------+---------+---------|
contig022354 CAGTGTTTGTCTGTGTTGGTTTTATGCTATTTTTAGCATCAGTCTTTATGCAAAAGACTACCTGATTGAATCAGGCAGGTATAATTCCTGTTATGGAGAGTGTGCATTCGTCATTAATGATATTGCAAGG
TilTARs.A037 CAGTGTTTGTCTGTGTTGGTTTTATGCTATTTTTAGCATCAGTCTTTATGCAAAAGACTACCTGATTGAATCAGGCAGGTATAATTCCTGTTATGGAGAGTGTGCATTCGTCATTAATGATATTGCAAGG
   Consensus CAGTGTTTGTCTGTGTTGGTTTTATGCTATTTTTAGCATCAGTCTTTATGCAAAAGACTACCTGATTGAATCAGGCAGGTATAATTCCTGTTATGGAGAGTGTGCATTCGTCATTAATGATATTGCAAGG

             3511 3520      3530      3540      3550      3560      3570      3580      3590      3600      3610      3620      3630      3640
             |--------+---------+---------+---------+---------+---------+---------+---------+---------+---------+---------+---------+---------|
contig022354 ACTATCGACCTTGTTTTATCCTTTATATTTCCAATTACTGTCATCATAGTTCTGTATATGAGAGTATTTGTGGTGGCTGTGTCTCAGGCTCGTGCCATGCGCTCTCATGTTACAGCTGTCACACTTCAGT
TilTARs.A037 ACTATCGACCTTGTTTTATCCTTTATATTTCCAATTACTGTCATCATAGTTCTGTATATGAGAGTATTTGTGGTGGCTGTGTCTCAGGCTCGTGCCATGCGCTCTCATGTTACAGCTGTCACACTTCAGT
   Consensus ACTATCGACCTTGTTTTATCCTTTATATTTCCAATTACTGTCATCATAGTTCTGTATATGAGAGTATTTGTGGTGGCTGTGTCTCAGGCTCGTGCCATGCGCTCTCATGTTACAGCTGTCACACTTCAGT

             3641 3650      3660      3670      3680      3690      3700      3710      3720      3730      3740      3750      3760      3770
             |--------+---------+---------+---------+---------+---------+---------+---------+---------+---------+---------+---------+---------|
contig022354 GTTCACTGAATCAAGCAAACAAATCAGAGTTGAAAGCAGCCAGGACTCTTGGGATTCTTGTAGTTGTGTTTCTACTGTGTTTCTGCCCATATTACTGTGTCTCTCTTATAAGGGAGGACTTTAGCTCAGT
TilTARs.A037 GTTCACTGAATCAAGCAAACAAATCAGAGTTGAAAGCAGCCAGGACTCTTGGGATTCTTGTAGTTGTGTTTCTACTGTGTTTCTGCCCATATTACTGTGTCTCTCTTATAAGGGAGGACTTTAGCTCAGT
   Consensus GTTCACTGAATCAAGCAAACAAATCAGAGTTGAAAGCAGCCAGGACTCTTGGGATTCTTGTAGTTGTGTTTCTACTGTGTTTCTGCCCATATTACTGTGTCTCTCTTATAAGGGAGGACTTTAGCTCAGT

             3771 3780      3790      3800      3810      3820      3830      3840      3850      3860      3870      3880      3890      3900
             |--------+---------+---------+---------+---------+---------+---------+---------+---------+---------+---------+---------+---------|
contig022354 AGCGCCCATTGTGATCAGTCTGTTCTTTTCAAACTCCTGTTTAAACCCAGTGATCTATGCCTTGTTCTATCCTTGGTTTAGAAAAGCTTTGAAACTAATTGTCACTTTACAGATACTGCATAGTGGCTCC
TilTARs.A037 AGCGCCCATTGTGATCAGTCTGTTCTTTTCAAACTCCTGTTTAAACCCAGTGATCTATGCCTTGTTCTATCCTTGGTTTAGAAAAGCTTTGAAACTAATTGTCACTTTACAGATACTGCATAGTGGCTCC
   Consensus AGCGCCCATTGTGATCAGTCTGTTCTTTTCAAACTCCTGTTTAAACCCAGTGATCTATGCCTTGTTCTATCCTTGGTTTAGAAAAGCTTTGAAACTAATTGTCACTTTACAGATACTGCATAGTGGCTCC

             3901 3910      3920      3930      3940      3950      3960      3970      3980      3990      4000      4010      4020      4030
             |--------+---------+---------+---------+---------+---------+---------+---------+---------+---------+---------+---------+---------|
contig022354 TGTGAGGTCAGTATACTGTAGACATGAAGAACTCTAGAAATGCAGAAAATAGTTTTAATGTTCATAACTGAGCAATTTGCAATCATCCCTTGCTTTTGTTGTAGAATATGTGGACAGTAATTCATTGTGA
TilTARs.A037 TGTGAGGTCAGTATACTGTAG
   Consensus TGTGAGGTCAGTATACTGTAG.............................................................................................................

[illegible]
```

```
              9491 9500      9510      9520      9530      9540      9550      9560      9570      9580      9590      9600      9610      9620
              |--------+---------+---------+---------+---------+---------+---------+---------+---------+---------+---------+---------+---------|
contig022354  TGTATGATGATGGAGATGCAGAAAGGAGCCGAGCTGTGTTTTCCACAACTCCTCAACAGTTCCTGCAGGAAGCCAACACTTCACTGGTCCAAAGCTGTGCTCCTGAACATTGTGCTGTCCTGCATCTCTC
TilTARs.A038           ATGGAGATGCAGAAAGGAGCCGAGCTGTGTTTTCCACAACTCCTCAACAGTTCCTGCAGGAAGCCAACACTTCACTGGTCCAAAGCTGTGCTCCTGAACATTGTGCTGTCCTGCATCTCTC
   Consensus  .........ATGGAGATGCAGAAAGGAGCCGAGCTGTGTTTTCCACAACTCCTCAACAGTTCCTGCAGGAAGCCAACACTTCACTGGTCCAAAGCTGTGCTCCTGAACATTGTGCTGTCCTGCATCTCTC

              9621 9630      9640      9650      9660      9670      9680      9690      9700      9710      9720      9730      9740      9750
              |--------+---------+---------+---------+---------+---------+---------+---------+---------+---------+---------+---------+---------|
contig022354  TGCTCACTGCTGCTCTAAACCTTCTCGTCATCATCTCAGTCTCTTACTTCAGGCAGAGATTAATTTCTCAGCTGAAATTGTAGTTTAAGTCTTAGAAACTAGTTTAGTTATTATTTATTATTATTTAGTG
TilTARs.A038  TGCTCACTGCTGCTCTAAACCTTCTCGTCATCATCTCAGTCTCTTACTTCAGGCAG--------------------------------------------------------------------------
   Consensus  TGCTCACTGCTGCTCTAAACCTTCTCGTCATCATCTCAGTCTCTTACTTCAGGCAG..........................................................................

              9751 9760      9770      9780      9790      9800      9810      9820      9830      9840      9850      9860      9870      9880
              |--------+---------+---------+---------+---------+---------+---------+---------+---------+---------+---------+---------+---------|
contig022354  ATGAATGACATGAATATTGCACATTATTGGTGTATTTTAACCACACTGTAATGTTTTATTTGCTTGGCCTTACTAATGGAACATTTTTACTAATGGAATTAGTGAAGACATTTTTACATTATTAATGTGT
TilTARs.A038  ----------------------------------------------------------------------------------------------------------------------------------
   Consensus  ..................................................................................................................................

              9881 9890      9900      9910      9920      9930      9940      9950      9960      9970      9980      9990      10000     10010
              |--------+---------+---------+---------+---------+---------+---------+---------+---------+---------+---------+---------+---------|
contig022354  AATATATTCTTTTTTCTCTCTCCTTAGGCAGCTTCACACACCCACTAACATCCTCCTTCTTTCTCTGGCTGTCTCAGACTTTCTCACTGGTCTCCTTATGCTGCCACTAGAAATAATTAGAAACACGGCC
TilTARs.A038  -------------------------------CTTCACACACCCACTAACATCCTCCTTCTTTCTCTGGCTGTCTCAGACTTTCTCACTGGTCTCCTTATGCTGCCACTAGAAATAATTAGAAACACGGCC
   Consensus  ...............................CTTCACACACCCACTAACATCCTCCTTCTTTCTCTGGCTGTCTCAGACTTTCTCACTGGTCTCCTTATGCTGCCACTAGAAATAATTAGAAACACGGCC

              1001110020     10030     10040     10050     10060     10070     10080     10090     10100     10110     10120     10130     10140
              |--------+---------+---------+---------+---------+---------+---------+---------+---------+---------+---------+---------+---------|
contig022354  TGCTGGGTACTTGGTGATCTTATGTGTTCTATTTATACTTATCTGACAGCTGGTCTTTTCTGTGCTTCAACTGGGAACATAGTTCTCATTTCAGTTGACCGCTATGTGGCTATTTGTCACCCTCTGCATT
TilTARs.A038  TGCTGGGTACTTGGTGATCTTATGTGTTCTATTTATACTTATCTGACAGCTGGTCTTTTCTGTGCTTCAACTGGGAACATAGTTCTCATTTCAGTTGACCGCTATGTGGCTATTTGTCACCCTCTGCATT
   Consensus  TGCTGGGTACTTGGTGATCTTATGTGTTCTATTTATACTTATCTGACAGCTGGTCTTTTCTGTGCTTCAACTGGGAACATAGTTCTCATTTCAGTTGACCGCTATGTGGCTATTTGTCACCCTCTGCATT

              1014110150     10160     10170     10180     10190     10200     10210     10220     10230     10240     10250     10260     10270
              |--------+---------+---------+---------+---------+---------+---------+---------+---------+---------+---------+---------+---------|
contig022354  ACCCCACCAGAATTACTGTGGCAAGAGTCAAACTCTGTGTTTGTTTGTGTTGGTTTTGTTCAACTTTCTACAGCAGTCTTTTAACAAAGGATGTTCTAATTAAGCCGGCCAGGTATAATTCCTGTTATGG
TilTARs.A038  ACCCCACCAGAATTACTGTGGCAAGAGTCAAACTCTGTGTTTGTTTGTGTTGGTTTTGTTCAACTTTCTACAGCAGTCTTTTAACAAAGGATGTTCTAATTAAGCCGGCCAGGTATAATTCCTGTTATGG
   Consensus  ACCCCACCAGAATTACTGTGGCAAGAGTCAAACTCTGTGTTTGTTTGTGTTGGTTTTGTTCAACTTTCTACAGCAGTCTTTTAACAAAGGATGTTCTAATTAAGCCGGCCAGGTATAATTCCTGTTATGG

              1027110280     10290     10300     10310     10320     10330     10340     10350     10360     10370     10380     10390     10400
              |--------+---------+---------+---------+---------+---------+---------+---------+---------+---------+---------+---------+---------|
contig022354  AGAGTGTGTGTTTGTCATTAATGATATTGCAGGGTTTATTGATCTTGTGTTATCTTTTATTGTTCCAGTATCTGTTATCATAGTTCTTTATATGAGAGTATTTGTGGTGGCTGTGTCTCAGGCTCGTGCC
TilTARs.A038  AGAGTGTGTGTTTGTCATTAATGATATTGCAGGGTTTATTGATCTTGTGTTATCTTTTATTGTTCCAGTATCTGTTATCATAGTTCTTTATATGAGAGTATTTGTGGTGGCTGTGTCTCAGGCTCGTGCC
   Consensus  AGAGTGTGTGTTTGTCATTAATGATATTGCAGGGTTTATTGATCTTGTGTTATCTTTTATTGTTCCAGTATCTGTTATCATAGTTCTTTATATGAGAGTATTTGTGGTGGCTGTGTCTCAGGCTCGTGCC

              1040110410     10420     10430     10440     10450     10460     10470     10480     10490     10500     10510     10520     10530
              |--------+---------+---------+---------+---------+---------+---------+---------+---------+---------+---------+---------+---------|
contig022354  ATGCGCTCTCATGTTACAGCTGTCACACTTCATCATTCAAGCAATCAAGCAAACAAATCTGAACTGAAAGCAGCCAGGACTCTTGGAGTTCTTGTAGTTGTGTTTCTCGCATGTTACCTTCCATTTTACT
TilTARs.A038  ATGCGCTCTCATGTTACAGCTGTCACACTTCATCATTCAAGCAATCAAGCAAACAAATCTGAACTGAAAGCAGCCAGGACTCTTGGAGTTCTTGTAGTTGTGTTTCTCGCATGTTACCTTCCATTTTACT
   Consensus  ATGCGCTCTCATGTTACAGCTGTCACACTTCATCATTCAAGCAATCAAGCAAACAAATCTGAACTGAAAGCAGCCAGGACTCTTGGAGTTCTTGTAGTTGTGTTTCTCGCATGTTACCTTCCATTTTACT

              1053110540     10550     10560     10570     10580     10590     10600     10610     10620     10630     10640     10650     10660
              |--------+---------+---------+---------+---------+---------+---------+---------+---------+---------+---------+---------+---------|
contig022354  GTTACTCTCTTATTGAAGTAAATGTGATCAATGACTCATCTACATTTTTTCTGATCATTGTGTTTTACTTTAACTCGTGTCTAAACCCACTGATCTATGCCCTTTTTTACTCTTGGTTTAGAAATGCTGT
TilTARs.A038  GTTACTCTCTTATTGAAGTAAATGTGATCAATGACTCATCTACATTTTTTCTGATCATTGTGTTTTACTTTAACTCGTGTCTAAACCCACTGATCTATGCCCTTTTTTACTCTTGGTTTAGAAATGCTGT
   Consensus  GTTACTCTCTTATTGAAGTAAATGTGATCAATGACTCATCTACATTTTTTCTGATCATTGTGTTTTACTTTAACTCGTGTCTAAACCCACTGATCTATGCCCTTTTTTACTCTTGGTTTAGAAATGCTGT

              1066110670     10680     10690     10700     10710     10720     10730     10740     10750     10760     10770     10780     10790
              |--------+---------+---------+---------+---------+---------+---------+---------+---------+---------+---------+---------+---------|
contig022354  TAAACTTATCATCACTCTGCAGATTTTTAAGCATGACACTAGTGAAGCAAACATGTTTTAAAAAGATAAGGAAACCCACTATACTAAGAATTTAAATCAATAAATCGATAAATTCAGTAATCTGAAAATG
TilTARs.A038  TAAACTTATCATCACTCTGCAGATTTTTAAGCATGACACTAGTGAAGCAAACATGTTTTAA
   Consensus  TAAACTTATCATCACTCTGCAGATTTTTAAGCATGACACTAGTGAAGCAAACATGTTTTAA.....................................................................
```

```
              5461  5470      5480      5490      5500      5510      5520      5530      5540      5550      5560      5570      5580      5590
              |--------+---------+---------+---------+---------+---------+---------+---------+---------+---------+---------+---------+---------|
contig022355  AAAGAGATTGGTGAGAAGGGGGAGATAAATCAGCTTTATGAGAAAAAGTTCTGATTAGAGCTGCGGCTGTCTGATGATGGAGATACAGAAAGAAAACAAACTGTGTTTTCCGCAACTCCTCAACAGTT
TilTARs.A039                                                                              ATGGAGATACAGAAAGAAAACAAACTGTGTTTTCCGCAACTCCTCAACAGTT
   Consensus  ............................................................................ATGGAGATACAGAAAGAAAACAAACTGTGTTTTCCGCAACTCCTCAACAGTT

              5591  5600      5610      5620      5630      5640      5650      5660      5670      5680      5690      5700      5710      5720
              |--------+---------+---------+---------+---------+---------+---------+---------+---------+---------+---------+---------+---------|
contig022355  CGTGCAGGAAGCCAACACTTCACTGGTCCAAAGCTGTGCTCCTGAACACTGTGCTGTCCTGCATCTCTCTGCTCACTGCTACTCTAAACCTTCTCGTCATCATCTCAGTCTCTTACTTCAGGCAGAGATT
TilTARs.A039  CGTGCAGGAAGCCAACACTTCACTGGTCCAAAGCTGTGCTCCTGAACACTGTGCTGTCCTGCATCTCTCTGCTCACTGCTACTCTAAACCTTCTCGTCATCATCTCAGTCTCTTACTTCAGG--------
   Consensus  CGTGCAGGAAGCCAACACTTCACTGGTCCAAAGCTGTGCTCCTGAACACTGTGCTGTCCTGCATCTCTCTGCTCACTGCTACTCTAAACCTTCTCGTCATCATCTCAGTCTCTTACTTCAGG........

              5721  5730      5740      5750      5760      5770      5780      5790      5800      5810      5820      5830      5840      5850
              |--------+---------+---------+---------+---------+---------+---------+---------+---------+---------+---------+---------+---------|
contig022355  AACTTTAAATATATGACAACTTTAATGTACATTATATCACTTAAGTGAATTTTGCTTGATGTATTTTAACCATGATTTTAATTTCCTGTTTGCCTAGACTGAATCAAGCCTTACTAATGCAGCATTTTCA
TilTARs.A039  ----------------------------------------------------------------------------------------------------------------------------------
   Consensus  ..................................................................................................................................

              5851  5860      5870      5880      5890      5900      5910      5920      5930      5940      5950      5960      5970      5980
              |--------+---------+---------+---------+---------+---------+---------+---------+---------+---------+---------+---------+---------|
contig022355  GTGAAGACAGTTTGACATTAATAATGTGCATTATATTCTTTTGTGTCTCTGCAGGAAGCTTCACACACCCAGTAATATCCTCCTCCTCTCTCTGGCTGTCTCAGACTTTCTTGTCGGTGTCCTGTTGATG
TilTARs.A039  -------------------------------------------------------AAGCTTCACACACCCAGTAATATCCTCCTCCTCTCTCTGGCTGTCTCAGACTTTCTTGTCGGTGTCCTGTTGATG
   Consensus  .......................................................AAGCTTCACACACCCAGTAATATCCTCCTCCTCTCTCTGGCTGTCTCAGACTTTCTTGTCGGTGTCCTGTTGATG

              5981  5990      6000      6010      6020      6030      6040      6050      6060      6070      6080      6090      6100      6110
              |--------+---------+---------+---------+---------+---------+---------+---------+---------+---------+---------+---------+---------|
contig022355  CCATTAGGAATCCTTAGAAACACAAGCTGCTGGGTACTTGGTGATATAATTTGTTCTCTTTATTGGTATTTAACCAGCAACATTGTCTGTGCTTCAATAGGGAACATAGTTCTCATATCAGTTGACCGCT
TilTARs.A039  CCATTAGGAATCCTTAGAAACACAAGCTGCTGGGTACTTGGTGATATAATTTGTTCTCTTTATTGGTATTTAACCAGCAACATTGTCTGTGCTTCAATAGGGAACATAGTTCTCATATCAGTTGACCGCT
   Consensus  CCATTAGGAATCCTTAGAAACACAAGCTGCTGGGTACTTGGTGATATAATTTGTTCTCTTTATTGGTATTTAACCAGCAACATTGTCTGTGCTTCAATAGGGAACATAGTTCTCATATCAGTTGACCGCT

              6111  6120      6130      6140      6150      6160      6170      6180      6190      6200      6210      6220      6230      6240
              |--------+---------+---------+---------+---------+---------+---------+---------+---------+---------+---------+---------+---------|
contig022355  ATGTGGCTATTTGTGACCCTCTGCATTATCCCAGCAGAATTACTTTGGTGAAAGTCAAACTCAGTGTTTGTCTGTGTTGGTTTTATGCTATTTTCTACTGCAGTCTTTATACAAAGGATATCCTAATTGA
TilTARs.A039  ATGTGGCTATTTGTGACCCTCTGCATTATCCCAGCAGAATTACTTTGGTGAAAGTCAAACTCAGTGTTTGTCTGTGTTGGTTTTATGCTATTTTCTACTGCAGTCTTTATACAAAGGATATCCTAATTGA
   Consensus  ATGTGGCTATTTGTGACCCTCTGCATTATCCCAGCAGAATTACTTTGGTGAAAGTCAAACTCAGTGTTTGTCTGTGTTGGTTTTATGCTATTTTCTACTGCAGTCTTTATACAAAGGATATCCTAATTGA

              6241  6250      6260      6270      6280      6290      6300      6310      6320      6330      6340      6350      6360      6370
              |--------+---------+---------+---------+---------+---------+---------+---------+---------+---------+---------+---------+---------|
contig022355  ACCGGGCAGGGATAATTCCTGCTACGGAGAGTGTGTATTTGTGATCAATGATATTGCTGTTGTTGTTGACCTTGTTTTTTCCTTTATTGTTCCAGTTTCTGTCATTGTAGTTCTGTATATGAGAGTATTT
TilTARs.A039  ACCGGGCAGGGATAATTCCTGCTACGGAGAGTGTGTATTTGTGATCAATGATATTGCTGTTGTTGTTGACCTTGTTTTTTCCTTTATTGTTCCAGTTTCTGTCATTGTAGTTCTGTATATGAGAGTATTT
   Consensus  ACCGGGCAGGGATAATTCCTGCTACGGAGAGTGTGTATTTGTGATCAATGATATTGCTGTTGTTGTTGACCTTGTTTTTTCCTTTATTGTTCCAGTTTCTGTCATTGTAGTTCTGTATATGAGAGTATTT

              6371  6380      6390      6400      6410      6420      6430      6440      6450      6460      6470      6480      6490      6500
              |--------+---------+---------+---------+---------+---------+---------+---------+---------+---------+---------+---------+---------|
contig022355  GTGGCAGCTGTGTCTCAGGCTCGTGCCATGCGCTCTCATGTTACATCTGTCACACTGCAGCGCTCACTGAATCAGACAAACAAATCTGAGCTGAAAGCAGCCAGGACTCTTGGAGTTCTTGTAGTTGTGT
TilTARs.A039  GTGGCAGCTGTGTCTCAGGCTCGTGCCATGCGCTCTCATGTTACATCTGTCACACTGCAGCGCTCACTGAATCAGACAAACAAATCTGAGCTGAAAGCAGCCAGGACTCTTGGAGTTCTTGTAGTTGTGT
   Consensus  GTGGCAGCTGTGTCTCAGGCTCGTGCCATGCGCTCTCATGTTACATCTGTCACACTGCAGCGCTCACTGAATCAGACAAACAAATCTGAGCTGAAAGCAGCCAGGACTCTTGGAGTTCTTGTAGTTGTGT

              6501  6510      6520      6530      6540      6550      6560      6570      6580      6590      6600      6610      6620      6630
              |--------+---------+---------+---------+---------+---------+---------+---------+---------+---------+---------+---------+---------|
contig022355  TTCTTGCATGCTTCTGTCCACTCTACTACTTTTCTCTTGTTGATGAAAATGCAATCAATGATCCATCTGCATCTTTTGTGGTCATTATATTTTACTTTAACTCTTGTATAAACCCTTTGATCTATGCCTT
TilTARs.A039  TTCTTGCATGCTTCTGTCCACTCTACTACTTTTCTCTTGTTGATGAAAATGCAATCAATGATCCATCTGCATCTTTTGTGGTCATTATATTTTACTTTAACTCTTGTATAAACCCTTTGATCTATGCCTT
   Consensus  TTCTTGCATGCTTCTGTCCACTCTACTACTTTTCTCTTGTTGATGAAAATGCAATCAATGATCCATCTGCATCTTTTGTGGTCATTATATTTTACTTTAACTCTTGTATAAACCCTTTGATCTATGCCTT

              6631  6640      6650      6660      6670      6680      6690      6700      6710      6720      6730      6740      6750      6760
              |--------+---------+---------+---------+---------+---------+---------+---------+---------+---------+---------+---------+---------|
contig022355  GTTTTACCCCTGGTTTAGAAATGCTGTTAAGCTTATCATCACTCTGCAGATTTTCAAGTATAACATAAGTGAGGCCAACATACTATAAAAAGAGTATGAAACCTGCTCTACTAAGATATAAAATCAGTTG
TilTARs.A039  GTTTTACCCCTGGTTTAGAAATGCTGTTAAGCTTATCATCACTCTGCAGATTTTCAAGTATAACATAAGTGAGGCCAACATACTATAA
   Consensus  GTTTTACCCCTGGTTTAGAAATGCTGTTAAGCTTATCATCACTCTGCAGATTTTCAAGTATAACATAAGTGAGGCCAACATACTATAA..........................................
```

```
             2861  2870      2880      2890      2900      2910      2920      2930      2940      2950      2960      2970      2980      2990
             |--------+---------+---------+---------+---------+---------+---------+---------+---------+---------+---------+---------+---------|
contig022356 CTGGACCTGCCTAATGGAGATACAGAAAGGAGCTGAGCTTTGTTTTCCACAATTTCTCAACAATTCCTGCAGGAAGCCGACACTACGTTCGTCGAAAGCTGTGCTCCTGAACATTGTGTTCTCCTGTATC
TilTARs.A040              ATGGAGATACAGAAAGGAGCTGAGCTTTGTTTTCCACAATTTCTCAACAATTCCTGCAGGAAGCCGACACTACGTTCGTCGAAAGCTGTGCTCCTGAACATTGTGTTCTCCTGTATC
   Consensus .............ATGGAGATACAGAAAGGAGCTGAGCTTTGTTTTCCACAATTTCTCAACAATTCCTGCAGGAAGCCGACACTACGTTCGTCGAAAGCTGTGCTCCTGAACATTGTGTTCTCCTGTATC

             2991  3000      3010      3020      3030      3040      3050      3060      3070      3080      3090      3100      3110      3120
             |--------+---------+---------+---------+---------+---------+---------+---------+---------+---------+---------+---------+---------|
contig022356 TCTCTGCTCACTGCTGCTCTAAACCTTCTCGTCATCATCTCAGTCTCTTACTTCAGGCACAGATGAATTTTTTTTATTGACGCTAATGAAATGCAACTGATAAATATATCTTTTTTTAATTTTCACTTTA
TilTARs.A040 TCTCTGCTCACTGCTGCTCTAAACCTTCTCGTCATCATCTCAGTCTCTTACTTCAGG-------------------------------------------------------------------------
   Consensus TCTCTGCTCACTGCTGCTCTAAACCTTCTCGTCATCATCTCAGTCTCTTACTTCAGG.........................................................................

             3121  3130      3140      3150      3160      3170      3180      3190      3200      3210      3220      3230      3240      3250
             |--------+---------+---------+---------+---------+---------+---------+---------+---------+---------+---------+---------+---------|
contig022356 GTTTTGCTAGATAAATAAATATCTTTGTCTCAGTTATCACTGTTAAGTTGAATAGGCTTCTTCATGCCTTAATAATGAAACATTTTCACAAAGTTGGTTTTAACATGATTGACAAAACATTTTCAATCCT
TilTARs.A040 ----------------------------------------------------------------------------------------------------------------------------------
   Consensus ..................................................................................................................................

             3251  3260      3270      3280      3290      3300      3310      3320      3330      3340      3350      3360      3370      3380
             |--------+---------+---------+---------+---------+---------+---------+---------+---------+---------+---------+---------+---------|
contig022356 GCTGTCAGCTGTCTCATTTTCATCCTAGTCTTGCGCTTCAAAGCTGAAATTGCAGTTTTGGTAATTTAGTGATGAGTGGAATCAATTTGGCATGTTCTTGGTGTATTTTAATCATGACTGAAATATTTTA
TilTARs.A040 ----------------------------------------------------------------------------------------------------------------------------------
   Consensus ..................................................................................................................................

             3381  3390      3400      3410      3420      3430      3440      3450      3460      3470      3480      3490      3500      3510
             |--------+---------+---------+---------+---------+---------+---------+---------+---------+---------+---------+---------+---------|
contig022356 TTCGCCTCGATTGAATCAAGCCTTATTAATAGGACTTTTTCACTAATGGAATTTTCACTGAAGACATTTTGAAATGATGAATTACTTTATTTTTTCGTCTCTCTGCAGGAAACTTCACACACCAAGTAAC
TilTARs.A040 -------------------------------------------------------------------------------------------------------------AAACTTCACACACCAAGTAAC
   Consensus .............................................................................................................AAACTTCACACACCAAGTAAC

             3511  3520      3530      3540      3550      3560      3570      3580      3590      3600      3610      3620      3630      3640
             |--------+---------+---------+---------+---------+---------+---------+---------+---------+---------+---------+---------+---------|
contig022356 ATCCTCCTCCTCTCTCTGGCTGTCTCAGACTTTCTTGTGGGTCTTCTGTTGATGCCTTTAGAAATATTTAGAAGCACGACCTGCTGGGTACTTGGTGACGTTATGTGTTCTGTTTATTGGTATCTGACCG
TilTARs.A040 ATCCTCCTCCTCTCTCTGGCTGTCTCAGACTTTCTTGTGGGTCTTCTGTTGATGCCTTTAGAAATATTTAGAAGCACGACCTGCTGGGTACTTGGTGACGTTATGTGTTCTGTTTATTGGTATCTGACCG
   Consensus ATCCTCCTCCTCTCTCTGGCTGTCTCAGACTTTCTTGTGGGTCTTCTGTTGATGCCTTTAGAAATATTTAGAAGCACGACCTGCTGGGTACTTGGTGACGTTATGTGTTCTGTTTATTGGTATCTGACCG

             3641  3650      3660      3670      3680      3690      3700      3710      3720      3730      3740      3750      3760      3770
             |--------+---------+---------+---------+---------+---------+---------+---------+---------+---------+---------+---------+---------|
contig022356 GCAACATTACCTGTGCTTCAATAGGGAACGTGGTTCTAATATCAGTTGATCGTTATGTGGCTATTTGTGACCCTTTGTATTACCGCACCAGAATTACTGTGGAAAGAACCAAACTAAGTGTGTGCTTATG
TilTARs.A040 GCAACATTACCTGTGCTTCAATAGGGAACGTGGTTCTAATATCAGTTGATCGTTATGTGGCTATTTGTGACCCTTTGTATTACCGCACCAGAATTACTGTGGAAAGAACCAAACTAAGTGTGTGCTTATG
   Consensus GCAACATTACCTGTGCTTCAATAGGGAACGTGGTTCTAATATCAGTTGATCGTTATGTGGCTATTTGTGACCCTTTGTATTACCGCACCAGAATTACTGTGGAAAGAACCAAACTAAGTGTGTGCTTATG

             3771  3780      3790      3800      3810      3820      3830      3840      3850      3860      3870      3880      3890      3900
             |--------+---------+---------+---------+---------+---------+---------+---------+---------+---------+---------+---------+---------|
contig022356 TTGGTTTTATGCTATTTTCTACTGGAGTCTTTATATAAAGAATATCCTGATTGAACCGGGCAGGTATAATTCCTGTTACGGAGAGTGTATGTTTGTAAGCAGTGATATTGCAGGGATTATTGACCTTGTT
TilTARs.A040 TTGGTTTTATGCTATTTTCTACTGGAGTCTTTATATAAAGAATATCCTGATTGAACCGGGCAGGTATAATTCCTGTTACGGAGAGTGTATGTTTGTAAGCAGTGATATTGCAGGGATTATTGACCTTGTT
   Consensus TTGGTTTTATGCTATTTTCTACTGGAGTCTTTATATAAAGAATATCCTGATTGAACCGGGCAGGTATAATTCCTGTTACGGAGAGTGTATGTTTGTAAGCAGTGATATTGCAGGGATTATTGACCTTGTT

             3901  3910      3920      3930      3940      3950      3960      3970      3980      3990      4000      4010      4020      4030
             |--------+---------+---------+---------+---------+---------+---------+---------+---------+---------+---------+---------+---------|
contig022356 TTATCTTTTATTGTTCCAGTTTCCGTCATCATAGTTCTTTATATGAGAGTATTTGTGGTGGCTGTGTCTCAGGCTCGTGCCATGCGCTCTCATGTTACAGCTGTCACACTTCAGCATTCAAACAATCAAG
TilTARs.A040 TTATCTTTTATTGTTCCAGTTTCCGTCATCATAGTTCTTTATATGAGAGTATTTGTGGTGGCTGTGTCTCAGGCTCGTGCCATGCGCTCTCATGTTACAGCTGTCACACTTCAGCATTCAAACAATCAAG
   Consensus TTATCTTTTATTGTTCCAGTTTCCGTCATCATAGTTCTTTATATGAGAGTATTTGTGGTGGCTGTGTCTCAGGCTCGTGCCATGCGCTCTCATGTTACAGCTGTCACACTTCAGCATTCAAACAATCAAG

             4031  4040      4050      4060      4070      4080      4090      4100      4110      4120      4130      4140      4150      4160
             |--------+---------+---------+---------+---------+---------+---------+---------+---------+---------+---------+---------+---------|
contig022356 CAAACAAATCTGAGCTGAAAGCAGCCAGGACTCTTGGGGTTCTTGTAGCTGTGTTTCTGTCATGTTACTCTCCATTTTACTGCTACTATCTTGCTGAAGAAAATGTGGTCAATGATCCATCTGCATCTAC
TilTARs.A040 CAAACAAATCTGAGCTGAAAGCAGCCAGGACTCTTGGGGTTCTTGTAGCTGTGTTTCTGTCATGTTACTCTCCATTTTACTGCTACTATCTTGCTGAAGAAAATGTGGTCAATGATCCATCTGCATCTAC
   Consensus CAAACAAATCTGAGCTGAAAGCAGCCAGGACTCTTGGGGTTCTTGTAGCTGTGTTTCTGTCATGTTACTCTCCATTTTACTGCTACTATCTTGCTGAAGAAAATGTGGTCAATGATCCATCTGCATCTAC

             4161  4170      4180      4190      4200      4210      4220      4230      4240      4250      4260      4270      4280      4290
             |--------+---------+---------+---------+---------+---------+---------+---------+---------+---------+---------+---------+---------|
contig022356 TGTTATCATTATTTTTTACTTTAACTCTTGTATAAACCCTTTGATATATGCGTTGTTTTACCCCTGGTTTAGAAATGCTGTTAAACTTATTATCACTCTGCAGATCTTCAAGCATAACACCTGTGAGGCC
TilTARs.A040 TGTTATCATTATTTTTTACTTTAACTCTTGTATAAACCCTTTGATATATGCGTTGTTTTACCCCTGGTTTAGAAATGCTGTTAAACTTATTATCACTCTGCAGATCTTCAAGCATAACACCTGTGAGGCC
   Consensus TGTTATCATTATTTTTTACTTTAACTCTTGTATAAACCCTTTGATATATGCGTTGTTTTACCCCTGGTTTAGAAATGCTGTTAAACTTATTATCACTCTGCAGATCTTCAAGCATAACACCTGTGAGGCC

             4291  4300      4310      4320      4330      4340      4350      4360      4370      4380      4390      4400      4410      4420
             |--------+---------+---------+---------+---------+---------+---------+---------+---------+---------+---------+---------+---------|
contig022356 AACATACTATAAAAGACTCTGAACTCTTATAATTGAGTGATTTGTATATATTAGTGTTATTTCTAAAATTTGTAAGCTCTATAACATATTGTATTATTTAAAAAGTTTAGTCTGTTACTGTTACTGTCCT
TilTARs.A040 AACATACTATAA
   Consensus AACATACTATAA......................................................................................................................

             4421  4430      4440      4450      4460      4470      4480      4490      4500      4510      4520      4530      4540      4550
             |--------+---------+---------+---------+---------+---------+---------+---------+---------+---------+---------+---------+---------|
[illegible]
```

```
              6241     6250      6260      6270      6280      6290      6300      6310      6320      6330      6340      6350      6360      6370
              |--------+---------+---------+---------+---------+---------+---------+---------+---------+---------+---------+---------+---------|
contig022357  CTGTCTGATAATGGTGATACAGAAAGGAGCTGAGCTCTGCTTTCCACAACTCCTCAACAGTTCATGCAGGAAGCCAACACTTCACTGGTCCAAAGCTGTGTTTCTGAACATTATGCTGCCCTGCCTCTCT
TilTARs.A041            ATGGTGATACAGAAAGGAGCTGAGCTCTGCTTTCCACAACTCCTCAACAGTTCATGCAGGAAGCCAACACTTCACTGGTCCAAAGCTGTGTTTCTGAACATTATGCTGCCCTGCCTCTCT
   Consensus  ..........ATGGTGATACAGAAAGGAGCTGAGCTCTGCTTTCCACAACTCCTCAACAGTTCATGCAGGAAGCCAACACTTCACTGGTCCAAAGCTGTGTTTCTGAACATTATGCTGCCCTGCCTCTCT

              6371     6380      6390      6400      6410      6420      6430      6440      6450      6460      6470      6480      6490      6500
              |--------+---------+---------+---------+---------+---------+---------+---------+---------+---------+---------+---------+---------|
contig022357  CTGACCACTGCTGCTCTAAACCTTTTGGTCATCATCTCAGTCTCCCACTTCAGGCAGAGATTAACTTTTCAACTGAAACTACAGTTAAATTTTAGATATTTGACTTTTAAACTCACTAATCTTAACATAT
TilTARs.A041  CTGACCACTGCTGCTCTAAACCTTTTGGTCATCATCTCAGTCTCCCACTTCAGGCA--------------------------------------------------------------------------
   Consensus  CTGACCACTGCTGCTCTAAACCTTTTGGTCATCATCTCAGTCTCCCACTTCAGGCA..........................................................................

              6501     6510      6520      6530      6540      6550      6560      6570      6580      6590      6600      6610      6620      6630
              |--------+---------+---------+---------+---------+---------+---------+---------+---------+---------+---------+---------+---------|
contig022357  GAAATCTATGAAACATTTGGCACATTCTTGGTGTATTATTTGCCTAGACTAAATAACGGAATGGAATATTTTCATTAATTTATTTTTCATAGAAGACATTTTGACATTATTAATGTGTAATATATTTTTT
TilTARs.A041  ----------------------------------------------------------------------------------------------------------------------------------
   Consensus  ..................................................................................................................................

              6631     6640      6650      6660      6670      6680      6690      6700      6710      6720      6730      6740      6750      6760
              |--------+---------+---------+---------+---------+---------+---------+---------+---------+---------+---------+---------+---------|
contig022357  TCTTTTGTCTTTCTGCAGGCAACTTCATACACCCACTAACATCCTCCTTCTGTCTCTGGCTGTCTCAGACTTTCTCACGGGTTTCCTGATTTTGCCATTAGAAATAATTAGAAACATGGCCTGCTGGGTA
TilTARs.A041  ---------------------ACTTCATACACCCACTAACATCCTCCTTCTGTCTCTGGCTGTCTCAGACTTTCTCACGGGTTTCCTGATTTTGCCATTAGAAATAATTAGAAACATGGCCTGCTGGGTA
   Consensus  .....................ACTTCATACACCCACTAACATCCTCCTTCTGTCTCTGGCTGTCTCAGACTTTCTCACGGGTTTCCTGATTTTGCCATTAGAAATAATTAGAAACATGGCCTGCTGGGTA

              6761     6770      6780      6790      6800      6810      6820      6830      6840      6850      6860      6870      6880      6890
              |--------+---------+---------+---------+---------+---------+---------+---------+---------+---------+---------+---------+---------|
contig022357  CTTGGTGATCTCATGTGTTCTGTTTATACTTATTTGATCACCAGTCTTTTCTGTGCTTCAACTGGGATCATAGTTCTAATATCAGTTGACCGCTATGTGGCTATTTGTGACCCTCTGCATTACCCCACCA
TilTARs.A041  CTTGGTGATCTCATGTGTTCTGTTTATACTTATTTGATCACCAGTCTTTTCTGTGCTTCAACTGGGATCATAGTTCTAATATCAGTTGACCGCTATGTGGCTATTTGTGACCCTCTGCATTACCCCACCA
   Consensus  CTTGGTGATCTCATGTGTTCTGTTTATACTTATTTGATCACCAGTCTTTTCTGTGCTTCAACTGGGATCATAGTTCTAATATCAGTTGACCGCTATGTGGCTATTTGTGACCCTCTGCATTACCCCACCA

              6891     6900      6910      6920      6930      6940      6950      6960      6970      6980      6990      7000      7010      7020
              |--------+---------+---------+---------+---------+---------+---------+---------+---------+---------+---------+---------+---------|
contig022357  GAATGACTGTGGTGAGATCCAAGCTTAGTGTGTGCCTATGCTGGTTTTATTCGACTTTCTACAGCAGCCTTCAAACAAAGGATGTTCTAATTAATCCAGGCAGGTATAATTCCTGCGACGGAGAGTGTGT
TilTARs.A041  GAATGACTGTGGTGAGATCCAAGCTTAGTGTGTGCCTATGCTGGTTTTATTCGACTTTCTACAGCAGCCTTCAAACAAAGGATGTTCTAATTAATCCAGGCAGGTATAATTCCTGCGACGGAGAGTGTGT
   Consensus  GAATGACTGTGGTGAGATCCAAGCTTAGTGTGTGCCTATGCTGGTTTTATTCGACTTTCTACAGCAGCCTTCAAACAAAGGATGTTCTAATTAATCCAGGCAGGTATAATTCCTGCGACGGAGAGTGTGT

              7021     7030      7040      7050      7060      7070      7080      7090      7100      7110      7120      7130      7140      7150
              |--------+---------+---------+---------+---------+---------+---------+---------+---------+---------+---------+---------+---------|
contig022357  GTTTCTCATCAGTAATATTGCAGGGACCATTGACCTTGTTTTATCTTTTATTTTTCCAGTTTCTGTCATCATAATTTTGTATATGAGAGTATTTGTGGTGGCTGTGTCTCAGGCTCGTGCCACGCGCTCT
TilTARs.A041  GTTTCTCATCAGTAATATTGCAGGGACCATTGACCTTGTTTTATCTTTTATTTTTCCAGTTTCTGTCATCATAATTTTGTATATGAGAGTATTTGTGGTGGCTGTGTCTCAGGCTCGTGCCACGCGCTCT
   Consensus  GTTTCTCATCAGTAATATTGCAGGGACCATTGACCTTGTTTTATCTTTTATTTTTCCAGTTTCTGTCATCATAATTTTGTATATGAGAGTATTTGTGGTGGCTGTGTCTCAGGCTCGTGCCACGCGCTCT

              7151     7160      7170      7180      7190      7200      7210      7220      7230      7240      7250      7260      7270      7280
              |--------+---------+---------+---------+---------+---------+---------+---------+---------+---------+---------+---------+---------|
contig022357  CATGTTACAGCTGTCACACTTCAGAGTTCACTGAATCAAACAAACAAATCTGAGTTGAAAGCGGCCAGGACTCTGGGGATTCTTGTAGTTGTGTTTCTTGCATGCTACTGTCCATTTTACTGCTATTTTC
TilTARs.A041  CATGTTACAGCTGTCACACTTCAGAGTTCACTGAATCAAACAAACAAATCTGAGTTGAAAGCGGCCAGGACTCTGGGGATTCTTGTAGTTGTGTTTCTTGCATGCTACTGTCCATTTTACTGCTATTTTC
   Consensus  CATGTTACAGCTGTCACACTTCAGAGTTCACTGAATCAAACAAACAAATCTGAGTTGAAAGCGGCCAGGACTCTGGGGATTCTTGTAGTTGTGTTTCTTGCATGCTACTGTCCATTTTACTGCTATTTTC

              7281     7290      7300      7310      7320      7330      7340      7350      7360      7370      7380      7390      7400      7410
              |--------+---------+---------+---------+---------+---------+---------+---------+---------+---------+---------+---------+---------|
contig022357  TTGTAAAAGAAAATGTGGTCAATGATTCAGCTACATTTTTTGTGATCATTTTATATCATGTTAACTCTTGTTTGAATCCATTAATCTATGCCTTGTTTTACCCCTGGTTTAGAAATGCTGTTAAACTTAT
TilTARs.A041  TTGTAAAAGAAAATGTGGTCAATGATTCAGCTACATTTTTTGTGATCATTTTATATCATGTTAACTCTTGTTTGAATCCATTAATCTATGCCTTGTTTTACCCCTGGTTTAGAAATGCTGTTAAACTTAT
   Consensus  TTGTAAAAGAAAATGTGGTCAATGATTCAGCTACATTTTTTGTGATCATTTTATATCATGTTAACTCTTGTTTGAATCCATTAATCTATGCCTTGTTTTACCCCTGGTTTAGAAATGCTGTTAAACTTAT

              7411     7420      7430      7440      7450      7460      7470      7480      7490      7500      7510      7520      7530      7540
              |--------+---------+---------+---------+---------+---------+---------+---------+---------+---------+---------+---------+---------|
contig022357  CATCACTTTTCAGATATTCACGCATGACACCAGTGGGGCCAACATACTATAAAAACTCCATAAAATTTGTTGCACTAAGATTTGAAATCAGGAAATTGGCACATCTAATAATGTGGAAATGTAAAATTGC
TilTARs.A041  CATCACTTTTCAGATATTCACGCATGACACCAGTGGGGCCAACATACTATAA
   Consensus  CATCACTTTTCAGATATTCACGCATGACACCAGTGGGGCCAACATACTATAA..............................................................................

              7541     7550      7560      7570      7580      7590      7600      7610      7620      7630      7640      7650      7660      7670
```

```
             1171 1180      1190      1200      1210      1220      1230      1240      1250      1260      1270      1280      1290      1300
             |--------+---------+---------+---------+---------+---------+---------+---------+---------+---------+---------+---------+---------|
contig022362 TTGAAAATGTGAAAACAAAAGGCGGTCTCAAAAGAAAGAGTGGGGGGTGGGGGGCAAATATAAAAGTTCTTTGAGTAAAAGCTCTCATCAGAAAGCTGCAGGATATGTAGTTTTGTGATAATGGAGATAC
TilTARs.A042                                                                                                                         ATGGAGATAC
   Consensus ........................................................................................................................ATGGAGATAC

             1301 1310      1320      1330      1340      1350      1360      1370      1380      1390      1400      1410      1420      1430
             |--------+---------+---------+---------+---------+---------+---------+---------+---------+---------+---------+---------+---------|
contig022362 AGAAGGGAGCCGAGCTTTGTTTTCAACAACTACTCAACAATTCCTGCAGGAAGCTGACACTTAACTGGTCCAAAACTGTGCTTCTGTACATTGTGCTGTTTTGCATCTCTCTGATCACTGTTGCTCTGAA
TilTARs.A042 AGAAGGGAGCCGAGCTTTGTTTTCAACAACTACTCAACAATTCCTGCAGGAAGCTGACACTTAACTGGTCCAAAACTGTGCTTCTGTACATTGTGCTGTTTTGCATCTCTCTGATCACTGTTGCTCTGAA
   Consensus AGAAGGGAGCCGAGCTTTGTTTTCAACAACTACTCAACAATTCCTGCAGGAAGCTGACACTTAACTGGTCCAAAACTGTGCTTCTGTACATTGTGCTGTTTTGCATCTCTCTGATCACTGTTGCTCTGAA

             1431 1440      1450      1460      1470      1480      1490      1500      1510      1520      1530      1540      1550      1560
             |--------+---------+---------+---------+---------+---------+---------+---------+---------+---------+---------+---------+---------|
contig022362 CCTCCTGGTCATCATCTCGGTCTCCCACTTCAGGCAGAGATTAACTTTTCTGTTGAAACTGCGGTTTAAGTGTTAGATATGTGAAAAAGTAAATTTTCATCAATGAATAAATGAAGTGCAAGAGATGTGT
TilTARs.A042 CCTCCTGGTCATCATCTCGGTCTCCCACTTCAGGCAG---------------------------------------------------------------------------------------------
   Consensus CCTCCTGGTCATCATCTCGGTCTCCCACTTCAGGCAG.............................................................................................

             1561 1570      1580      1590      1600      1610      1620      1630      1640      1650      1660      1670      1680      1690
             |--------+---------+---------+---------+---------+---------+---------+---------+---------+---------+---------+---------+---------|
contig022362 TCTAACTAAGGAAACATTTTCACTAAAGACATTTCGGCATGATTACATGTACATGACATAGACACTGGTCCTCTCAACTGTCTTATCTTTTTCAAAATCTCTCCTTTGAACTTTTGAGCTGAAATCACAG
TilTARs.A042 ----------------------------------------------------------------------------------------------------------------------------------
   Consensus ..................................................................................................................................

             1691 1700      1710      1720      1730      1740      1750      1760      1770      1780      1790      1800      1810      1820
             |--------+---------+---------+---------+---------+---------+---------+---------+---------+---------+---------+---------+---------|
contig022362 TTTACATTTTAGAAGGTTCAAAGCATGTTAAATGAAATTAATTTGGTGCATTCTTTCTGTATTTTAACCACTGTATTTTTTCATTTTCTTAGACTGAATCATGTCTTACTAATATAAACATTTTTACTAA
TilTARs.A042 ----------------------------------------------------------------------------------------------------------------------------------
   Consensus ..................................................................................................................................

             1821 1830      1840      1850      1860      1870      1880      1890      1900      1910      1920      1930      1940      1950
             |--------+---------+---------+---------+---------+---------+---------+---------+---------+---------+---------+---------+---------|
contig022362 TAGAATTTTCACTAAAGATATTTTGATATTCTTAATTTGTATTATATTCTTTTCTCTCTTGTTGCAGGCAGCTTCACACACCCACTAACATCCTGCTTCTCTCTCTGGCTGTCTCAGACTTTCTTGTTGG
TilTARs.A042 -----------------------------------------------------------------------CTTCACACACCCACTAACATCCTGCTTCTCTCTCTGGCTGTCTCAGACTTTCTTGTTGG
   Consensus .......................................................................CTTCACACACCCACTAACATCCTGCTTCTCTCTCTGGCTGTCTCAGACTTTCTTGTTGG

             1951 1960      1970      1980      1990      2000      2010      2020      2030      2040      2050      2060      2070      2080
             |--------+---------+---------+---------+---------+---------+---------+---------+---------+---------+---------+---------+---------|
contig022362 TCTCCTGTTGATACCTTTAGAAATCCTTAGAAGCACAACCTGCTGGGTATTTGGTGATGTTATATGTTCTTTTTATTTTTATCTGACTGGCAACATTACCTGTGCTTCAACAGGAATCATAGTTCTAATA
TilTARs.A042 TCTCCTGTTGATACCTTTAGAAATCCTTAGAAGCACAACCTGCTGGGTATTTGGTGATGTTATATGTTCTTTTTATTTTTATCTGACTGGCAACATTACCTGTGCTTCAACAGGAATCATAGTTCTAATA
   Consensus TCTCCTGTTGATACCTTTAGAAATCCTTAGAAGCACAACCTGCTGGGTATTTGGTGATGTTATATGTTCTTTTTATTTTTATCTGACTGGCAACATTACCTGTGCTTCAACAGGAATCATAGTTCTAATA

             2081 2090      2100      2110      2120      2130      2140      2150      2160      2170      2180      2190      2200      2210
             |--------+---------+---------+---------+---------+---------+---------+---------+---------+---------+---------+---------+---------|
contig022362 TCAGTTGACCGCTATGTGGCTATTTGTGACCCTCTGCATTACCCCACCAGAATGACTGTGGCGAGGGTGAAACTCAGTGTTTGTCTGTGTTGGTTTTATGCTATTTTCTATGTCACTCTTTACCTAAAAG
TilTARs.A042 TCAGTTGACCGCTATGTGGCTATTTGTGACCCTCTGCATTACCCCACCAGAATGACTGTGGCGAGGGTGAAACTCAGTGTTTGTCTGTGTTGGTTTTATGCTATTTTCTATGTCACTCTTTACCTAAAAG
   Consensus TCAGTTGACCGCTATGTGGCTATTTGTGACCCTCTGCATTACCCCACCAGAATGACTGTGGCGAGGGTGAAACTCAGTGTTTGTCTGTGTTGGTTTTATGCTATTTTCTATGTCACTCTTTACCTAAAAG

             2211 2220      2230      2240      2250      2260      2270      2280      2290      2300      2310      2320      2330      2340
             |--------+---------+---------+---------+---------+---------+---------+---------+---------+---------+---------+---------+---------|
contig022362 ACTTCCTGAAGCCAGGCAGGTATAACTCCTGCTATGGAGAGTGTATCCTTGTCATCAATGATACTGCAGGAACTGCTGACCTTGTTTTATCTTTTTTTGTTCCCATCACTGTCATTATAGTTCTGTATAT
TilTARs.A042 ACTTCCTGAAGCCAGGCAGGTATAACTCCTGCTATGGAGAGTGTATCCTTGTCATCAATGATACTGCAGGAACTGCTGACCTTGTTTTATCTTTTTTTGTTCCCATCACTGTCATTATAGTTCTGTATAT
   Consensus ACTTCCTGAAGCCAGGCAGGTATAACTCCTGCTATGGAGAGTGTATCCTTGTCATCAATGATACTGCAGGAACTGCTGACCTTGTTTTATCTTTTTTTGTTCCCATCACTGTCATTATAGTTCTGTATAT

             2341 2350      2360      2370      2380      2390      2400      2410      2420      2430      2440      2450      2460      2470
             |--------+---------+---------+---------+---------+---------+---------+---------+---------+---------+---------+---------+---------|
contig022362 GAGAATATTTGTGGTGGCTGTGTCTCAGGCTCGTGCAATGCGCTCTCATGTTACAGCAGTTGCAAAATCCAAATCTGAGTTGAAAGCAGCCAGGACTCTGGGTGTTCTAGTAGTTGTGTTTCTTGCAAGC
TilTARs.A042 GAGAATATTTGTGGTGGCTGTGTCTCAGGCTCGTGCAATGCGCTCTCATGTTACAGCAGTTGCAAAATCCAAATCTGAGTTGAAAGCAGCCAGGACTCTGGGTGTTCTAGTAGTTGTGTTTCTTGCAAGC
   Consensus GAGAATATTTGTGGTGGCTGTGTCTCAGGCTCGTGCAATGCGCTCTCATGTTACAGCAGTTGCAAAATCCAAATCTGAGTTGAAAGCAGCCAGGACTCTGGGTGTTCTAGTAGTTGTGTTTCTTGCAAGC

             2471 2480      2490      2500      2510      2520      2530      2540      2550      2560      2570      2580      2590      2600
             |--------+---------+---------+---------+---------+---------+---------+---------+---------+---------+---------+---------+---------|
contig022362 TACTGTCCATTTTACTGTTACACCCTCGTTGAAGAAAATGTGATCAGTGATTCATCTGCATTTTTTGTGGTCATTGCCTTTTATTTGAACTCTTTTCTAAACCCAATGATCTATGCCTTGTTTTATCCAT
TilTARs.A042 TACTGTCCATTTTACTGTTACACCCTCGTTGAAGAAAATGTGATCAGTGATTCATCTGCATTTTTTGTGGTCATTGCCTTTTATTTGAACTCTTTTCTAAACCCAATGATCTATGCCTTGTTTTATCCAT
   Consensus TACTGTCCATTTTACTGTTACACCCTCGTTGAAGAAAATGTGATCAGTGATTCATCTGCATTTTTTGTGGTCATTGCCTTTTATTTGAACTCTTTTCTAAACCCAATGATCTATGCCTTGTTTTATCCAT

             2601 2610      2620      2630      2640      2650      2660      2670      2680      2690      2700      2710      2720      2730
             |--------+---------+---------+---------+---------+---------+---------+---------+---------+---------+---------+---------+---------|
contig022362 GGTTTAAAAAATCTGTTAACCTTATCATCACTCTGCAGATCTTTAAGCGTCACAGCAGTGATTCTAACATATTATAAACAACAAACATTATAAGTCAGTACATCATACATCTAGTAATCTGGAGAAATGA
TilTARs.A042 GGTTTAAAAAATCTGTTAACCTTATCATCACTCTGCAGATCTTTAAGCGTCACAGCAGTGATTCTAACATATTATAA
   Consensus GGTTTAAAAAATCTGTTAACCTTATCATCACTCTGCAGATCTTTAAGCGTCACAGCAGTGATTCTAACATATTATAA.....................................................
```

```
             2081  2090      2100      2110      2120      2130      2140      2150      2160      2170      2180      2190      2200      2210
             |--------+---------+---------+---------+---------+---------+---------+---------+---------+---------+---------+---------+---------|
contig022363 ATATAACAGTTCTCTGAGTCAAAGCTCTCATCAGAGAGCTGCAGGATCTGCAGTTATGTGATAATGGAGATACAGAAAGGAGCCGAACTTTGTTTTCAACAACTACTCAACAATTCCTGCAGGAAGCCAA
TilTARs.A043                                                                ATGGAGATACAGAAAGGAGCCGAACTTTGTTTTCAACAACTACTCAACAATTCCTGCAGGAAGCCAA
   Consensus ...............................................................ATGGAGATACAGAAAGGAGCCGAACTTTGTTTTCAACAACTACTCAACAATTCCTGCAGGAAGCCAA

             2211  2220      2230      2240      2250      2260      2270      2280      2290      2300      2310      2320      2330      2340
             |--------+---------+---------+---------+---------+---------+---------+---------+---------+---------+---------+---------+---------|
contig022363 CACTTCACTGGTCCAAAGCTGTACTTCTATACATTGTGCTGTTCTGCATCTCTCTGATCACTGTTGCTCTGAACCTCCTGGTCATCATCTCAGTCTCCCACTTCAGGCAGAGATTAGTTTTTCAGTTGAA
TilTARs.A043 CACTTCACTGGTCCAAAGCTGTACTTCTATACATTGTGCTGTTCTGCATCTCTCTGATCACTGTTGCTCTGAACCTCCTGGTCATCATCTCAGTCTCCCACTTCAGGCAG--------------------
   Consensus CACTTCACTGGTCCAAAGCTGTACTTCTATACATTGTGCTGTTCTGCATCTCTCTGATCACTGTTGCTCTGAACCTCCTGGTCATCATCTCAGTCTCCCACTTCAGGCAG....................

             2341  2350      2360      2370      2380      2390      2400      2410      2420      2430      2440      2450      2460      2470
             |--------+---------+---------+---------+---------+---------+---------+---------+---------+---------+---------+---------+---------|
contig022363 ACTGCGATTTAGGTTTTAGATATGTGAAAAAGTAAATTTTCATCAATGAATGGATGAAGTGCAAGAGATAAATATCTTCAGCTTAACAATGGCTGTTGACTAAACTTAATCATGTTTTAACTAAGGACAC
TilTARs.A043 ----------------------------------------------------------------------------------------------------------------------------------
   Consensus ..................................................................................................................................

             2471  2480      2490      2500      2510      2520      2530      2540      2550      2560      2570      2580      2590      2600
             |--------+---------+---------+---------+---------+---------+---------+---------+---------+---------+---------+---------+---------|
contig022363 ATTTTCACTAAAGACATTTTAGCATGATTAGTACATGACATAACCACGGGTCCTCTCAGCTGTCTTATCTTCTTTAAAATCTCTCTCTTCCTCAGATGAACTTTTGAGCTGAAATCACTGTTTAAATTTT
TilTARs.A043 ----------------------------------------------------------------------------------------------------------------------------------
   Consensus ..................................................................................................................................

             2601  2610      2620      2630      2640      2650      2660      2670      2680      2690      2700      2710      2720      2730
             |--------+---------+---------+---------+---------+---------+---------+---------+---------+---------+---------+---------+---------|
contig022363 AGAAAGTTCAGTGCATCAATTTTGTATTTGTGTATTATATTTGCATTTGTGTAGAATTTTCACTAAAGATATTTTGATATTCTTAATTTGTCTTATATTCTTTTCTCTCTTGTTGCAGGCAGCTTCACAC
TilTARs.A043 --------------------------------------------------------------------------------------------------------------------------CTTCACAC
   Consensus ..........................................................................................................................CTTCACAC

             2731  2740      2750      2760      2770      2780      2790      2800      2810      2820      2830      2840      2850      2860
             |--------+---------+---------+---------+---------+---------+---------+---------+---------+---------+---------+---------+---------|
contig022363 ACCCACTAACATCCTCCTCCTCTCTCTGGCTGTCTCAGACTTTCTTGTTGGCCTCCTGTTGATACCTTTAGAAATCCTTAGAAGCACAACCTGCTGGGTATTTGGTGATGTTATGTGTTCTGTTTATTGG
TilTARs.A043 ACCCACTAACATCCTCCTCCTCTCTCTGGCTGTCTCAGACTTTCTTGTTGGCCTCCTGTTGATACCTTTAGAAATCCTTAGAAGCACAACCTGCTGGGTATTTGGTGATGTTATGTGTTCTGTTTATTGG
   Consensus ACCCACTAACATCCTCCTCCTCTCTCTGGCTGTCTCAGACTTTCTTGTTGGCCTCCTGTTGATACCTTTAGAAATCCTTAGAAGCACAACCTGCTGGGTATTTGGTGATGTTATGTGTTCTGTTTATTGG

             2861  2870      2880      2890      2900      2910      2920      2930      2940      2950      2960      2970      2980      2990
             |--------+---------+---------+---------+---------+---------+---------+---------+---------+---------+---------+---------+---------|
contig022363 TATCTGACTGGCAACATTACTTGTGCTTCAATAGGGAACATAGTTCTAATATCAGTTGACCGCTATGTGGCTATTTGTGACCCTCTGCATTACCCCACTAGAATGACTGTGGTGAGAATCAAACACTGGA
TilTARs.A043 TATCTGACTGGCAACATTACTTGTGCTTCAATAGGGAACATAGTTCTAATATCAGTTGACCGCTATGTGGCTATTTGTGACCCTCTGCATTACCCCACTAGAATGACTGTGGTGAGAATCAAACACTGGA
   Consensus TATCTGACTGGCAACATTACTTGTGCTTCAATAGGGAACATAGTTCTAATATCAGTTGACCGCTATGTGGCTATTTGTGACCCTCTGCATTACCCCACTAGAATGACTGTGGTGAGAATCAAACACTGGA

             2991  3000      3010      3020      3030      3040      3050      3060      3070      3080      3090      3100      3110      3120
             |--------+---------+---------+---------+---------+---------+---------+---------+---------+---------+---------+---------+---------|
contig022363 TTTGTTTGTGTTGGTTTTATGCTATTTTCTATGACACTTTATACCTAAAAGACTTACTGATTAAACCAGGCAGGTATAATTCCTGCTATGGAGAGTGTGTACTTGTCATCAATGATATTGCAGGAACTGC
TilTARs.A043 TTTGTTTGTGTTGGTTTTATGCTATTTTCTATGACACTTTATACCTAAAAGACTTACTGATTAAACCAGGCAGGTATAATTCCTGCTATGGAGAGTGTGTACTTGTCATCAATGATATTGCAGGAACTGC
   Consensus TTTGTTTGTGTTGGTTTTATGCTATTTTCTATGACACTTTATACCTAAAAGACTTACTGATTAAACCAGGCAGGTATAATTCCTGCTATGGAGAGTGTGTACTTGTCATCAATGATATTGCAGGAACTGC

             3121  3130      3140      3150      3160      3170      3180      3190      3200      3210      3220      3230      3240      3250
             |--------+---------+---------+---------+---------+---------+---------+---------+---------+---------+---------+---------+---------|
contig022363 TGACCTTGTTGGTGGTTCCTTTGGTTCTTACCACCGTACATCGCCATGGTAACGTATACTACAAACCTAATCTACTCCAGAGCAGGTTCTAAGATCCAGATTAGAGATCAACAGGAATACTGCTGACCAA
TilTARs.A043 TGACCTTGTTGGTGGTTCCTTTGGTTCTTACCACCGTACATCGCCATGGTAA
   Consensus TGACCTTGTTGGTGGTTCCTTTGGTTCTTACCACCGTACATCGCCATGGTAA..............................................................................

             3251  3260      3270      3280      3290      3300      3310      3320      3330      3340      3350      3360      3370      3380
```

```
             6891 6900      6910      6920      6930      6940      6950      6960      6970      6980      6990      7000      7010      7020
             |--------+---------+---------+---------+---------+---------+---------+---------+---------+---------+---------+---------+---------|
contig022363 GGCTCTTCAAGCAAAAGCTCTTAACTGAGAGCTGCAGGATTTGCAGCCAAGTGATGATGGAGATAAAGAAAGGAGACGAGCTTTGTTTTGTAGAGCTCAACAGTTCCTGCAAGAAGCCAACACTTCACTG
TilTARs.A044                                                         ATGGAGATAAAGAAAGGAGACGAGCTTTGTTTTGTAGAGCTCAACAGTTCCTGCAAGAAGCCAACACTTCACTG
   Consensus ........................................................ATGGAGATAAAGAAAGGAGACGAGCTTTGTTTTGTAGAGCTCAACAGTTCCTGCAAGAAGCCAACACTTCACTG

             7021 7030      7040      7050      7060      7070      7080      7090      7100      7110      7120      7130      7140      7150
             |--------+---------+---------+---------+---------+---------+---------+---------+---------+---------+---------+---------+---------|
contig022363 GTCCAAAGCTGTGCTCCTGAACATTGTGCTGTCCTGTATCTCTTTGATCACTGCTGTTCTGAACCTTCTCGTCATCATCTCAGTCTCTCACTTCAGGCACAGATCAATTCCTCAGCTGAAATTAGAGTTT
TilTARs.A044 GTCCAAAGCTGTGCTCCTGAACATTGTGCTGTCCTGTATCTCTTTGATCACTGCTGTTCTGAACCTTCTCGTCATCATCTCAGTCTCTCACTTCAGGCA-------------------------------
   Consensus GTCCAAAGCTGTGCTCCTGAACATTGTGCTGTCCTGTATCTCTTTGATCACTGCTGTTCTGAACCTTCTCGTCATCATCTCAGTCTCTCACTTCAGGCA...............................

             7151 7160      7170      7180      7190      7200      7210      7220      7230      7240      7250      7260      7270      7280
             |--------+---------+---------+---------+---------+---------+---------+---------+---------+---------+---------+---------+---------|
contig022363 AAGTTTTCAAAACTTTAACACTATGCTTTAATAAATTAATGGAGTGAATCTAGCACATTGTTGGTGTAGTTTAACTATGATGTTTTATTTGCCTTGCTTCAGTGCCTTACTAATGAAATTATTGGAGACA
TilTARs.A044 ----------------------------------------------------------------------------------------------------------------------------------
   Consensus ..................................................................................................................................

             7281 7290      7300      7310      7320      7330      7340      7350      7360      7370      7380      7390      7400      7410
             |--------+---------+---------+---------+---------+---------+---------+---------+---------+---------+---------+---------+---------|
contig022363 TTTTGACATTATTAATCTGCATTATCTTCTTTTCTCTCTTGTTGCAGGCAGCTTCACACACCCACTAACATCCTTCTCCTCTCTCTGGCTGTCTCAGACTTTCTTGTTGGTTTCCTGTTGATGCCATTAG
TilTARs.A044 --------------------------------------------------GCTTCACACACCCACTAACATCCTTCTCCTCTCTCTGGCTGTCTCAGACTTTCTTGTTGGTTTCCTGTTGATGCCATTAG
   Consensus ..................................................GCTTCACACACCCACTAACATCCTTCTCCTCTCTCTGGCTGTCTCAGACTTTCTTGTTGGTTTCCTGTTGATGCCATTAG

             7411 7420      7430      7440      7450      7460      7470      7480      7490      7500      7510      7520      7530      7540
             |--------+---------+---------+---------+---------+---------+---------+---------+---------+---------+---------+---------+---------|
contig022363 AAATCTTTAGAAGCACAACCTGCTGGGTGTTTGGTGATGTTATGTGTTCTGTTTATTGGTATCTGACTGGCAACATTACCTGTGCTTCAATAGGGAACATAGTTCTAATATCAGTTGACCGCTATGTGGC
TilTARs.A044 AAATCTTTAGAAGCACAACCTGCTGGGTGTTTGGTGATGTTATGTGTTCTGTTTATTGGTATCTGACTGGCAACATTACCTGTGCTTCAATAGGGAACATAGTTCTAATATCAGTTGACCGCTATGTGGC
   Consensus AAATCTTTAGAAGCACAACCTGCTGGGTGTTTGGTGATGTTATGTGTTCTGTTTATTGGTATCTGACTGGCAACATTACCTGTGCTTCAATAGGGAACATAGTTCTAATATCAGTTGACCGCTATGTGGC

             7541 7550      7560      7570      7580      7590      7600      7610      7620      7630      7640      7650      7660      7670
             |--------+---------+---------+---------+---------+---------+---------+---------+---------+---------+---------+---------+---------|
contig022363 TATTTGTGACCCTCTGCATTACCCCACTAGAATGACTGTGGTGAGAATCAAACACTGGATTTGTTTGTGTTGGTTTTATGCTATTTTCTATGACACTTTATACCTAAAAGACTTACTGATTAAACCAGGC
TilTARs.A044 TATTTGTGACCCTCTGCATTACCCCACTAGAATGACTGTGGTGAGAATCAAACACTGGATTTGTTTGTGTTGGTTTTATGCTATTTTCTATGACACTTTATACCTAAAAGACTTACTGATTAAACCAGGC
   Consensus TATTTGTGACCCTCTGCATTACCCCACTAGAATGACTGTGGTGAGAATCAAACACTGGATTTGTTTGTGTTGGTTTTATGCTATTTTCTATGACACTTTATACCTAAAAGACTTACTGATTAAACCAGGC

             7671 7680      7690      7700      7710      7720      7730      7740      7750      7760      7770      7780      7790      7800
             |--------+---------+---------+---------+---------+---------+---------+---------+---------+---------+---------+---------+---------|
contig022363 AGGTATAATTCCTGCTATGGAGAGTGTGTCCTTGTCATCAATGATATTGCAGGAACTGCTGACCTTGTTTTATCGTTTTGTGTTCCAATTTCTGTTATCATAGTTCTGTATATGAGAGTATTTTTGGTGG
TilTARs.A044 AGGTATAATTCCTGCTATGGAGAGTGTGTCCTTGTCATCAATGATATTGCAGGAACTGCTGACCTTGTTTTATCGTTTTGTGTTCCAATTTCTGTTATCATAGTTCTGTATATGAGAGTATTTTTGGTGG
   Consensus AGGTATAATTCCTGCTATGGAGAGTGTGTCCTTGTCATCAATGATATTGCAGGAACTGCTGACCTTGTTTTATCGTTTTGTGTTCCAATTTCTGTTATCATAGTTCTGTATATGAGAGTATTTTTGGTGG

             7801 7810      7820      7830      7840      7850      7860      7870      7880      7890      7900      7910      7920      7930
             |--------+---------+---------+---------+---------+---------+---------+---------+---------+---------+---------+---------+---------|
contig022363 CTGTGTCTCAGGCTCGTGCCATGCGCTCTCATGTAACAACTGTCACACTGCAGCGTTCATTGAATCAAACAAACAAATCTGAGCTGAAAGCAGCCAGGACTCTTGGGATTCTTGTAGTTGTGTTTCTACT
TilTARs.A044 CTGTGTCTCAGGCTCGTGCCATGCGCTCTCATGTAACAACTGTCACACTGCAGCGTTCATTGAATCAAACAAACAAATCTGAGCTGAAAGCAGCCAGGACTCTTGGGATTCTTGTAGTTGTGTTTCTACT
   Consensus CTGTGTCTCAGGCTCGTGCCATGCGCTCTCATGTAACAACTGTCACACTGCAGCGTTCATTGAATCAAACAAACAAATCTGAGCTGAAAGCAGCCAGGACTCTTGGGATTCTTGTAGTTGTGTTTCTACT

             7931 7940      7950      7960      7970      7980      7990      8000      8010      8020      8030      8040      8050      8060
             |--------+---------+---------+---------+---------+---------+---------+---------+---------+---------+---------+---------+---------|
contig022363 TTGTTTCTGCCCGTATTACTGTGTCTCTCTTGTAAGGGACAGAAATAGCTCAGTAGCCCCTAATTTAATGAGTGTGTTTTTTTTAAACTCATGTCTAAACCCGTTGATCTATACTATGTTCTATCCTTGG
TilTARs.A044 TTGTTTCTGCCCGTATTACTGTGTCTCTCTTGTAAGGGACAGAAATAGCTCAGTAGCCCCTAATTTAATGAGTGTGTTTTTTTTAAACTCATGTCTAAACCCGTTGATCTATACTATGTTCTATCCTTGG
   Consensus TTGTTTCTGCCCGTATTACTGTGTCTCTCTTGTAAGGGACAGAAATAGCTCAGTAGCCCCTAATTTAATGAGTGTGTTTTTTTTAAACTCATGTCTAAACCCGTTGATCTATACTATGTTCTATCCTTGG

             8061 8070      8080      8090      8100      8110      8120      8130      8140      8150      8160      8170      8180      8190
             |--------+---------+---------+---------+---------+---------+---------+---------+---------+---------+---------+---------+---------|
contig022363 TTTAGAAAAGCTGTAAAACTAATTGTCACTCTACAGATCCTACAGACTGGCTCCTGTGAGGTCAGTATACTGTAGAAATACTGTTTTAAAAAGGTTTGAAACTTTAAAAATTATATACAAGTAAATGCAG
TilTARs.A044 TTTAGAAAAGCTGTAAAACTAATTGTCACTCTACAGATCCTACAGACTGGCTCCTGTGAGGTCAGTATACTGTAG
   Consensus TTTAGAAAAGCTGTAAAACTAATTGTCACTCTACAGATCCTACAGACTGGCTCCTGTGAGGTCAGTATACTGTAG.......................................................
```

```
             2341 2350      2360      2370      2380      2390      2400      2410      2420      2430      2440      2450      2460      2470
             |--------+---------+---------+---------+---------+---------+---------+---------+---------+---------+---------+---------+---------|
contig022365 GTGAAAGCAGAAAGAGAAGTTATTTCTGTGTGAGGTCTTGGTCAAAAAGCTGTGGGTAGTGCAGCTCTCCAGTGATGGAGAAAGGAGCTGAGCTCTGCTTTCCACAACTCAACAGTTCCTGCAGGAAGCC
TilTARs.A045                                                                          ATGGAGAAAGGAGCTGAGCTCTGCTTTCCACAACTCAACAGTTCCTGCAGGAAGCC
   Consensus ..........................................................................ATGGAGAAAGGAGCTGAGCTCTGCTTTCCACAACTCAACAGTTCCTGCAGGAAGCC

             2471 2480      2490      2500      2510      2520      2530      2540      2550      2560      2570      2580      2590      2600
             |--------+---------+---------+---------+---------+---------+---------+---------+---------+---------+---------+---------+---------|
contig022365 GACACTTCACTGGTCCAAGGCTGTGCTGCTGACCATTGTGCTGTTCTGCATCTCTCTGATCACTGTTACTCTGAACATCTTAGTCATCATCTCAGTCTCCCACTTCAGGCACAGAATAGCTTTTCTACTG
TilTARs.A045 GACACTTCACTGGTCCAAGGCTGTGCTGCTGACCATTGTGCTGTTCTGCATCTCTCTGATCACTGTTACTCTGAACATCTTAGTCATCATCTCAGTCTCCCACTTCAGGCA------------------
   Consensus GACACTTCACTGGTCCAAGGCTGTGCTGCTGACCATTGTGCTGTTCTGCATCTCTCTGATCACTGTTACTCTGAACATCTTAGTCATCATCTCAGTCTCCCACTTCAGGCA..................

             2601 2610      2620      2630      2640      2650      2660      2670      2680      2690      2700      2710      2720      2730
             |--------+---------+---------+---------+---------+---------+---------+---------+---------+---------+---------+---------+---------|
contig022365 AAAGTACAGTTTAAGTTTTAGAAATGTGAAAGCTCATATTTAAATCTATGAATGAATAAAGTGCAAGAGATGACTGTTGACTAAGATTAATCATGTTTTCACTAAAGACATGTTGGAATATTTAAAACAT
TilTARs.A045 ----------------------------------------------------------------------------------------------------------------------------------
   Consensus ..................................................................................................................................

             2731 2740      2750      2760      2770      2780      2790      2800      2810      2820      2830      2840      2850      2860
             |--------+---------+---------+---------+---------+---------+---------+---------+---------+---------+---------+---------+---------|
contig022365 GACATAAACTTTTGAGGTGAAATCACAGTTTAAGTTTTAGTAATTGAAATCCATTCTTTGTGTGTTTTAAACATGATTGTAATGTTTTATTTGCTTAGATTGAATCAAACCTTACCAATATAATTGGTGT
TilTARs.A045 ----------------------------------------------------------------------------------------------------------------------------------
   Consensus ..................................................................................................................................

             2861 2870      2880      2890      2900      2910      2920      2930      2940      2950      2960      2970      2980      2990
             |--------+---------+---------+---------+---------+---------+---------+---------+---------+---------+---------+---------+---------|
contig022365 TTTACCAACAGAATTTTCACTAAAGATATTTTGACATGATTAATTTGTATTATATTCTCTCTTGTTGCAGGCAGCTTCACACACCCAGTAACATCCTCCTCCCTTCTCTGGCTGTCTCAGACTTACTTGT
TilTARs.A045 ------------------------------------------------------------------------GCTTCACACACCCAGTAACATCCTCCTCCCTTCTCTGGCTGTCTCAGACTTACTTGT
   Consensus ........................................................................GCTTCACACACCCAGTAACATCCTCCTCCCTTCTCTGGCTGTCTCAGACTTACTTGT

             2991 3000      3010      3020      3030      3040      3050      3060      3070      3080      3090      3100      3110      3120
             |--------+---------+---------+---------+---------+---------+---------+---------+---------+---------+---------+---------+---------|
contig022365 TGGCCTCCTGTTGATGCCTTTAGAAATCTTTAGAAACACAAGCTGCTGGGTATTTGGAGATCTCATATGTTCGGTCTATTGGTATCTGAGTGGCAACATTACCTCCACTTCAACAGGGAACATAGTTCTT
TilTARs.A045 TGGCCTCCTGTTGATGCCTTTAGAAATCTTTAGAAACACAAGCTGCTGGGTATTTGGAGATCTCATATGTTCGGTCTATTGGTATCTGAGTGGCAACATTACCTCCACTTCAACAGGGAACATAGTTCTT
   Consensus TGGCCTCCTGTTGATGCCTTTAGAAATCTTTAGAAACACAAGCTGCTGGGTATTTGGAGATCTCATATGTTCGGTCTATTGGTATCTGAGTGGCAACATTACCTCCACTTCAACAGGGAACATAGTTCTT

             3121 3130      3140      3150      3160      3170      3180      3190      3200      3210      3220      3230      3240      3250
             |--------+---------+---------+---------+---------+---------+---------+---------+---------+---------+---------+---------+---------|
contig022365 ATATCAGTTGACCGCTATGTGGCTATTTGTGACCCCCTTCATTATCCCACCAGAATGACTGTGGTGAGAGTCAAACTTAGTGTTTGTCTGTGTTGGTTTTATTATATTTTTTACATCAGTTTTTACCTTA
TilTARs.A045 ATATCAGTTGACCGCTATGTGGCTATTTGTGACCCCCTTCATTATCCCACCAGAATGACTGTGGTGAGAGTCAAACTTAGTGTTTGTCTGTGTTGGTTTTATTATATTTTTTACATCAGTTTTTACCTTA
   Consensus ATATCAGTTGACCGCTATGTGGCTATTTGTGACCCCCTTCATTATCCCACCAGAATGACTGTGGTGAGAGTCAAACTTAGTGTTTGTCTGTGTTGGTTTTATTATATTTTTTACATCAGTTTTTACCTTA

             3251 3260      3270      3280      3290      3300      3310      3320      3330      3340      3350      3360      3370      3380
             |--------+---------+---------+---------+---------+---------+---------+---------+---------+---------+---------+---------+---------|
contig022365 AAGACATCCTGATTGAACCAGGCAGGTATAATTCCTGCAAAGGAGAGTGTGTACTTGTCATCAATGATATTGCAGGGATTATTGACCTTGTCATAGCTTTTATTGTTCCAGTTTCTGTAATCATAGTTCT
TilTARs.A045 AAGACATCCTGATTGAACCAGGCAGGTATAATTCCTGCAAAGGAGAGTGTGTACTTGTCATCAATGATATTGCAGGGATTATTGACCTTGTCATAGCTTTTATTGTTCCAGTTTCTGTAATCATAGTTCT
   Consensus AAGACATCCTGATTGAACCAGGCAGGTATAATTCCTGCAAAGGAGAGTGTGTACTTGTCATCAATGATATTGCAGGGATTATTGACCTTGTCATAGCTTTTATTGTTCCAGTTTCTGTAATCATAGTTCT

             3381 3390      3400      3410      3420      3430      3440      3450      3460      3470      3480      3490      3500      3510
             |--------+---------+---------+---------+---------+---------+---------+---------+---------+---------+---------+---------+---------|
contig022365 GTATATGAGAGTATTTGTGGTGGCTGTGTCTCAGGCTCGTGCCATGCGCTCTTGTGTCAGAGCAGTAACACTTCAGCATTCACTGAATCAAGCAAACAAATCTGAGTTGAAAGCAGCCAGGACTCTTGGG
TilTARs.A045 GTATATGAGAGTATTTGTGGTGGCTGTGTCTCAGGCTCGTGCCATGCGCTCTTGTGTCAGAGCAGTAACACTTCAGCATTCACTGAATCAAGCAAACAAATCTGAGTTGAAAGCAGCCAGGACTCTTGGG
   Consensus GTATATGAGAGTATTTGTGGTGGCTGTGTCTCAGGCTCGTGCCATGCGCTCTTGTGTCAGAGCAGTAACACTTCAGCATTCACTGAATCAAGCAAACAAATCTGAGTTGAAAGCAGCCAGGACTCTTGGG

             3511 3520      3530      3540      3550      3560      3570      3580      3590      3600      3610      3620      3630      3640
             |--------+---------+---------+---------+---------+---------+---------+---------+---------+---------+---------+---------+---------|
contig022365 ATTCTTGTAGTTGTGTTTCTTGCAAGCTTCTGTCCATTTTACTGTTACTTTTTTGTTGGAGAAAATGTGGTCAGTGATTCATCTGCATTTTTTGTGATCATTGCCTTTTATTTTAACTCTTTCCTAAACC
TilTARs.A045 ATTCTTGTAGTTGTGTTTCTTGCAAGCTTCTGTCCATTTTACTGTTACTTTTTTGTTGGAGAAAATGTGGTCAGTGATTCATCTGCATTTTTTGTGATCATTGCCTTTTATTTTAACTCTTTCCTAAACC
   Consensus ATTCTTGTAGTTGTGTTTCTTGCAAGCTTCTGTCCATTTTACTGTTACTTTTTTGTTGGAGAAAATGTGGTCAGTGATTCATCTGCATTTTTTGTGATCATTGCCTTTTATTTTAACTCTTTCCTAAACC

             3641 3650      3660      3670      3680      3690      3700      3710      3720      3730      3740      3750      3760      3770
             |--------+---------+---------+---------+---------+---------+---------+---------+---------+---------+---------+---------+---------|
contig022365 CAATGATCTATGCCTTGTTTTATCCTTGGTTTAGAAATGCTGTTAAGCTTATCATCACTCTGCAGATCTTTAAGCGTCACAGCAGTGATACTAACATATTATAAACAACAAAAACAACAAAAATGTAAGA
TilTARs.A045 CAATGATCTATGCCTTGTTTTATCCTTGGTTTAGAAATGCTGTTAAGCTTATCATCACTCTGCAGATCTTTAAGCGTCACAGCAGTGATACTAACATATTATAA
   Consensus CAATGATCTATGCCTTGTTTTATCCTTGGTTTAGAAATGCTGTTAAGCTTATCATCACTCTGCAGATCTTTAAGCGTCACAGCAGTGATACTAACATATTATAA.........................
```

```
             6631 6640      6650      6660      6670      6680      6690      6700      6710      6720      6730      6740      6750      6760
contig022368 GGGGGCGGGAGGAGGGTGAGCAAAAGCTGATATAAAAGTTCTCTGAGTCAAAGCTCTCATCAGAGAGCTGCAGGATCTGCAGTTATGTGATAATGGAGATACAGAAAGGAGCCGAGCTTTGTTTTCAACA
TilTARs.A046                                                                                             ATGGAGATACAGAAAGGAGCCGAGCTTTGTTTTCAACA
   Consensus ............................................................................................ATGGAGATACAGAAAGGAGCCGAGCTTTGTTTTCAACA

             6761 6770      6780      6790      6800      6810      6820      6830      6840      6850      6860      6870      6880      6890
contig022368 ACTACTCAACAATTCCTGCAGGAAGCTGACACTTCACTGGTCCAAGGCTGTGCTTCTGTACATTGTGCTGTTCTCCATCTCTCTGATCACTGTTGCTCTGAACCTCCTGGTCATCATCTCAGTCTCCCAC
TilTARs.A046 ACTACTCAACAATTCCTGCAGGAAGCTGACACTTCACTGGTCCAAGGCTGTGCTTCTGTACATTGTGCTGTTCTCCATCTCTCTGATCACTGTTGCTCTGAACCTCCTGGTCATCATCTCAGTCTCCCAC
   Consensus ACTACTCAACAATTCCTGCAGGAAGCTGACACTTCACTGGTCCAAGGCTGTGCTTCTGTACATTGTGCTGTTCTCCATCTCTCTGATCACTGTTGCTCTGAACCTCCTGGTCATCATCTCAGTCTCCCAC

             6891 6900      6910      6920      6930      6940      6950      6960      6970      6980      6990      7000      7010      7020
contig022368 TTCAGGCAGAGATTAACTTTTCAGTTGAAACTGCGATTTAGGTTTTAGATATGTGAAAACGTAAATTTTCATCAATGGATGGATGAAGTGCAAGAGATAAATATCTTCAGCTTAACAATGGCTGTTGACT
TilTARs.A046 TTCAGGCAG-------------------------------------------------------------------------------------------------------------------------
   Consensus TTCAGGCAG.........................................................................................................................

             7021 7030      7040      7050      7060      7070      7080      7090      7100      7110      7120      7130      7140      7150
contig022368 AAACTTAATCATGTTTTAACTAAGGAAACATTTTCACTAAAGACATTTTGGCATGATTAGTACATGACTCAACTGTCTTAATTTCTTTACAATCTCTCTCTACCAGCACAGATGAACTTTTGAGCTGAAA
TilTARs.A046 ----------------------------------------------------------------------------------------------------------------------------------
   Consensus ..................................................................................................................................

             7151 7160      7170      7180      7190      7200      7210      7220      7230      7240      7250      7260      7270      7280
contig022368 TCACAGTTTACATTTTAGAAGGTTCAGTGCATGAATGAAATTAATTTGGTGCATTCTTCATGTATTTTAACCATTGTACTTTTTTATTATCTTAGATTGAATCAAGTCTTACTAATATAAACATTTTTAC
TilTARs.A046 ----------------------------------------------------------------------------------------------------------------------------------
   Consensus ..................................................................................................................................

             7281 7290      7300      7310      7320      7330      7340      7350      7360      7370      7380      7390      7400      7410
contig022368 TAATAGAATTTTCACTAAAGATATTTTGATATTCTTAATTTGTCTTATATTATTTTCTCTCTTGTTCCAGGCAGCTTCACACACCCACTAACATCCTGCTTCTCTCTCTGGCTGTCTCAGACTTTCTTGT
TilTARs.A046 --------------------------------------------------------------------------CTTCACACACCCACTAACATCCTGCTTCTCTCTCTGGCTGTCTCAGACTTTCTTGT
   Consensus ..........................................................................CTTCACACACCCACTAACATCCTGCTTCTCTCTCTGGCTGTCTCAGACTTTCTTGT

             7411 7420      7430      7440      7450      7460      7470      7480      7490      7500      7510      7520      7530      7540
contig022368 TGGCCTCCTGTTGATACCTTTAGAAATCCTTAGAAGCACAACCTGCTGGGTATTTGGTGATGTTATATGTTCTGTTTATTGGTATCTGACTAGCAACATTACCTGTGCTTCAACAGGAATCATAGTTCTA
TilTARs.A046 TGGCCTCCTGTTGATACCTTTAGAAATCCTTAGAAGCACAACCTGCTGGGTATTTGGTGATGTTATATGTTCTGTTTATTGGTATCTGACTAGCAACATTACCTGTGCTTCAACAGGAATCATAGTTCTA
   Consensus TGGCCTCCTGTTGATACCTTTAGAAATCCTTAGAAGCACAACCTGCTGGGTATTTGGTGATGTTATATGTTCTGTTTATTGGTATCTGACTAGCAACATTACCTGTGCTTCAACAGGAATCATAGTTCTA

             7541 7550      7560      7570      7580      7590      7600      7610      7620      7630      7640      7650      7660      7670
contig022368 ATATCAGTTGACCGCTATGTAGCTATTTGTGACCCTCTGCATTACCCCACTAGAATGACTGTGGCGAGGGTGAAACTCAGTGTTTGTCTGTGTTGGTTTTATGCTATTTTCTACGTCACTCTTTACCTAA
TilTARs.A046 ATATCAGTTGACCGCTATGTAGCTATTTGTGACCCTCTGCATTACCCCACTAGAATGACTGTGGCGAGGGTGAAACTCAGTGTTTGTCTGTGTTGGTTTTATGCTATTTTCTACGTCACTCTTTACCTAA
   Consensus ATATCAGTTGACCGCTATGTAGCTATTTGTGACCCTCTGCATTACCCCACTAGAATGACTGTGGCGAGGGTGAAACTCAGTGTTTGTCTGTGTTGGTTTTATGCTATTTTCTACGTCACTCTTTACCTAA

             7671 7680      7690      7700      7710      7720      7730      7740      7750      7760      7770      7780      7790      7800
contig022368 AAGATTTCCTGATTAAACCAGGCAGTTATAATTCCTGCTATGGAGAGTGTGTCCTTGTCATCAATGATACTGCAGGAACTGCTGACCTTGTTTTATCCTTTTTTGTTCCCATCATTGTTATCACAGTTCT
TilTARs.A046 AAGATTTCCTGATTAAACCAGGCAGTTATAATTCCTGCTATGGAGAGTGTGTCCTTGTCATCAATGATACTGCAGGAACTGCTGACCTTGTTTTATCCTTTTTTGTTCCCATCATTGTTATCACAGTTCT
   Consensus AAGATTTCCTGATTAAACCAGGCAGTTATAATTCCTGCTATGGAGAGTGTGTCCTTGTCATCAATGATACTGCAGGAACTGCTGACCTTGTTTTATCCTTTTTTGTTCCCATCATTGTTATCACAGTTCT

             7801 7810      7820      7830      7840      7850      7860      7870      7880      7890      7900      7910      7920      7930
contig022368 GTATATGAGAGTATTTGTGGTGGCTGTGTCTCAGGCTCGTGCCATGCGCTCATATGTTACAGCTGTCACACTTCAGCGTTCACTGAATCAAACAAACAAATCTGAGTTGAAAGCAGCCAGGACTCTGGGT
TilTARs.A046 GTATATGAGAGTATTTGTGGTGGCTGTGTCTCAGGCTCGTGCCATGCGCTCATATGTTACAGCTGTCACACTTCAGCGTTCACTGAATCAAACAAACAAATCTGAGTTGAAAGCAGCCAGGACTCTGGGT
   Consensus GTATATGAGAGTATTTGTGGTGGCTGTGTCTCAGGCTCGTGCCATGCGCTCATATGTTACAGCTGTCACACTTCAGCGTTCACTGAATCAAACAAACAAATCTGAGTTGAAAGCAGCCAGGACTCTGGGT

             7931 7940      7950      7960      7970      7980      7990      8000      8010      8020      8030      8040      8050      8060
contig022368 GTTCTAGTTGTTGTGTTTCTTGCAAGCTACTGTCCGTTTTACTGTTATACCCTTGTTGAAGAAAATGTGGTCAGTGATTCATCTGCATTTTTTGTGGTCATTGCCTTTTATTTTAACTCTTTTCTAAACC
TilTARs.A046 GTTCTAGTTGTTGTGTTTCTTGCAAGCTACTGTCCGTTTTACTGTTATACCCTTGTTGAAGAAAATGTGGTCAGTGATTCATCTGCATTTTTTGTGGTCATTGCCTTTTATTTTAACTCTTTTCTAAACC
   Consensus GTTCTAGTTGTTGTGTTTCTTGCAAGCTACTGTCCGTTTTACTGTTATACCCTTGTTGAAGAAAATGTGGTCAGTGATTCATCTGCATTTTTTGTGGTCATTGCCTTTTATTTTAACTCTTTTCTAAACC

             8061 8070      8080      8090      8100      8110      8120      8130      8140      8150      8160      8170      8180      8190
contig022368 CAATGATCTATGCCTTCTTTTATCCTTGGTTTAGAAAGTCAGTTAAACTTATCATCACTCTGCAGATCTTTAAGCGTCACAGCAGTGATTCTAACATATTATAAACAACAAACAAAAAAAAACCCTTTTC
TilTARs.A046 CAATGATCTATGCCTTCTTTTATCCTTGGTTTAGAAAGTCAGTTAAACTTATCATCACTCTGCAGATCTTTAAGCGTCACAGCAGTGATTCTAACATATTATAA
   Consensus CAATGATCTATGCCTTCTTTTATCCTTGGTTTAGAAAGTCAGTTAAACTTATCATCACTCTGCAGATCTTTAAGCGTCACAGCAGTGATTCTAACATATTATAA..........................
```

```
             1157111580     11590     11600     11610     11620     11630     11640     11650     11660     11670     11680     11690     11700
             |--------+---------+---------+---------+---------+---------+---------+---------+---------+---------+---------+---------+---------|
contig022368 GAAGGCAGAAAGAGATAAGTTGTTTCTGTGTGAGGTCTTGGTCAAAAAGCTGTGGGTAGTGCAGCTCTCCGGTGATGGAGAAAGGAGCTGAGCTCTGCTTTCCACAACGCCCCAACAGTTCCTGCGGGAA
TilTARs.A047                                                                           ATGGAGAAAGGAGCTGAGCTCTGCTTTCCACAACGCCCCAACAGTTCCTGCGGGAA
   Consensus ..........................................................................ATGGAGAAAGGAGCTGAGCTCTGCTTTCCACAACGCCCCAACAGTTCCTGCGGGAA

             1170111710     11720     11730     11740     11750     11760     11770     11780     11790     11800     11810     11820     11830
             |--------+---------+---------+---------+---------+---------+---------+---------+---------+---------+---------+---------+---------|
contig022368 GCCGACACTTCACTGGTCCAAGGCTGTGCTTCTGTACAGTGTGCTGTTCTGCATCTCTCTGATCACTGTTGCTCTGAACCTCTTGGTCATCATCTCAGTCTCCCACTTCAGGCAGAGATTAACTTTTTAA
TilTARs.A047 GCCGACACTTCACTGGTCCAAGGCTGTGCTTCTGTACAGTGTGCTGTTCTGCATCTCTCTGATCACTGTTGCTCTGAACCTCTTGGTCATCATCTCAGTCTCCCACTTCAGGCAG---------------
   Consensus GCCGACACTTCACTGGTCCAAGGCTGTGCTTCTGTACAGTGTGCTGTTCTGCATCTCTCTGATCACTGTTGCTCTGAACCTCTTGGTCATCATCTCAGTCTCCCACTTCAGGCAG...............

             1183111840     11850     11860     11870     11880     11890     11900     11910     11920     11930     11940     11950     11960
             |--------+---------+---------+---------+---------+---------+---------+---------+---------+---------+---------+---------+---------|
contig022368 CTAAAGCTACAATTTAAGTTGTAGATATTTGAAAGCTCAGATTCACATCTATCAATGAACAAATTGCAAGAGATGACTGCTAATTAAGCTCAATCATGTTTTCATTAAAGACATTTTGACATGATTAAAA
TilTARs.A047 ----------------------------------------------------------------------------------------------------------------------------------
   Consensus ..................................................................................................................................

             1196111970     11980     11990     12000     12010     12020     12030     12040     12050     12060     12070     12080     12090
             |--------+---------+---------+---------+---------+---------+---------+---------+---------+---------+---------+---------+---------|
contig022368 CAAGACATGTAATTTTCAGGTGAAATCACAGTTTGTAATGTTTTGTTTGCTTAGATTCCCACCTTACCAACATAAATCGTTTTACTAATAGAATTTTCACTAAATATATTTTTATATTATTAATTTGTAA
TilTARs.A047 ----------------------------------------------------------------------------------------------------------------------------------
   Consensus ..................................................................................................................................

             1209112100     12110     12120     12130     12140     12150     12160     12170     12180     12190     12200     12210     12220
             |--------+---------+---------+---------+---------+---------+---------+---------+---------+---------+---------+---------+---------|
contig022368 TATATACTTTTCTCTCTTGTTGCAGGCAGCTTCACACACCCAGTAACATCCTCCTCCTCTCTCTGGCTGTCTCAGACTTACTTGTTGGTCTTCTGTTGATGCCATTAGAAATCCTTAGAAGCACAACCTG
TilTARs.A047 -----------------------------CTTCACACACCCAGTAACATCCTCCTCCTCTCTCTGGCTGTCTCAGACTTACTTGTTGGTCTTCTGTTGATGCCATTAGAAATCCTTAGAAGCACAACCTG
   Consensus .............................CTTCACACACCCAGTAACATCCTCCTCCTCTCTCTGGCTGTCTCAGACTTACTTGTTGGTCTTCTGTTGATGCCATTAGAAATCCTTAGAAGCACAACCTG

             1222112230     12240     12250     12260     12270     12280     12290     12300     12310     12320     12330     12340     12350
             |--------+---------+---------+---------+---------+---------+---------+---------+---------+---------+---------+---------+---------|
contig022368 CTGGGTATTTGGTGATGTTATATGTTCTGTTTATTGGTTTCTGAGTGGCAACATTATCTGTGCTTCAATAGGGAACATAGTTCTAATATCAGCTGACCGCTATGTAGCTATTTGTGACCCCCTTCATTAT
TilTARs.A047 CTGGGTATTTGGTGATGTTATATGTTCTGTTTATTGGTTTCTGAGTGGCAACATTATCTGTGCTTCAATAGGGAACATAGTTCTAATATCAGCTGACCGCTATGTAGCTATTTGTGACCCCCTTCATTAT
   Consensus CTGGGTATTTGGTGATGTTATATGTTCTGTTTATTGGTTTCTGAGTGGCAACATTATCTGTGCTTCAATAGGGAACATAGTTCTAATATCAGCTGACCGCTATGTAGCTATTTGTGACCCCCTTCATTAT

             1235112360     12370     12380     12390     12400     12410     12420     12430     12440     12450     12460     12470     12480
             |--------+---------+---------+---------+---------+---------+---------+---------+---------+---------+---------+---------+---------|
contig022368 CCCACTAGAATGACTGTGGTGAGAGTCAAACTTAATGTCAGTCTGTGTTGGTTTTATTATGTTTTTTACATCAGTCTTTACCTTAAAGACATCCTAATTAAACCAGGCAGGTATAATTCCTGCTATGGAG
TilTARs.A047 CCCACTAGAATGACTGTGGTGAGAGTCAAACTTAATGTCAGTCTGTGTTGGTTTTATTATGTTTTTTACATCAGTCTTTACCTTAAAGACATCCTAATTAAACCAGGCAGGTATAATTCCTGCTATGGAG
   Consensus CCCACTAGAATGACTGTGGTGAGAGTCAAACTTAATGTCAGTCTGTGTTGGTTTTATTATGTTTTTTACATCAGTCTTTACCTTAAAGACATCCTAATTAAACCAGGCAGGTATAATTCCTGCTATGGAG

             1248112490     12500     12510     12520     12530     12540     12550     12560     12570     12580     12590     12600     12610
             |--------+---------+---------+---------+---------+---------+---------+---------+---------+---------+---------+---------+---------|
contig022368 AGTGTGTGCCTGTCATCAATGATATTGCAGGGATTATTGACCTTGTCTTAACTTTTATTGTCCCAGTTTCTGTCATCATAGTTCTATATATGAGAGTATTTGTGGCAGCTGTGTCTCAGGCTCGTGCCAT
TilTARs.A047 AGTGTGTGCCTGTCATCAATGATATTGCAGGGATTATTGACCTTGTCTTAACTTTTATTGTCCCAGTTTCTGTCATCATAGTTCTATATATGAGAGTATTTGTGGCAGCTGTGTCTCAGGCTCGTGCCAT
   Consensus AGTGTGTGCCTGTCATCAATGATATTGCAGGGATTATTGACCTTGTCTTAACTTTTATTGTCCCAGTTTCTGTCATCATAGTTCTATATATGAGAGTATTTGTGGCAGCTGTGTCTCAGGCTCGTGCCAT

             1261112620     12630     12640     12650     12660     12670     12680     12690     12700     12710     12720     12730     12740
             |--------+---------+---------+---------+---------+---------+---------+---------+---------+---------+---------+---------+---------|
contig022368 GCGCTCTCATGTTACAGCAATAACATTTCAGCGTTCATTGAATCAAGCAAACAAATCTGAGTTGAAAGCAGCCAGGACTCTGGGTGTTCTTGTAATTGTGTTTCTAGCATGCTACTGTCCTTTTTTCTGT
TilTARs.A047 GCGCTCTCATGTTACAGCAATAACATTTCAGCGTTCATTGAATCAAGCAAACAAATCTGAGTTGAAAGCAGCCAGGACTCTGGGTGTTCTTGTAATTGTGTTTCTAGCATGCTACTGTCCTTTTTTCTGT
   Consensus GCGCTCTCATGTTACAGCAATAACATTTCAGCGTTCATTGAATCAAGCAAACAAATCTGAGTTGAAAGCAGCCAGGACTCTGGGTGTTCTTGTAATTGTGTTTCTAGCATGCTACTGTCCTTTTTTCTGT

             1274112750     12760     12770     12780     12790     12800     12810     12820     12830     12840     12850     12860     12870
             |--------+---------+---------+---------+---------+---------+---------+---------+---------+---------+---------+---------+---------|
contig022368 TATACCCTTGTTGAAGAAAAAGTAGTCAGTGATTCATTTGTGTTTTTTCTGATCATTGCCGTTTATTTTAACTCTTTTCTAAACCCACTAATCTATGCCTTCTTTTATCCTTGGTTTAGAAATGCTGTTA
TilTARs.A047 TATACCCTTGTTGAAGAAAAAGTAGTCAGTGATTCATTTGTGTTTTTTCTGATCATTGCCGTTTATTTTAACTCTTTTCTAAACCCACTAATCTATGCCTTCTTTTATCCTTGGTTTAGAAATGCTGTTA
   Consensus TATACCCTTGTTGAAGAAAAAGTAGTCAGTGATTCATTTGTGTTTTTTCTGATCATTGCCGTTTATTTTAACTCTTTTCTAAACCCACTAATCTATGCCTTCTTTTATCCTTGGTTTAGAAATGCTGTTA

             1287112880     12890     12900     12910     12920     12930     12940     12950     12960     12970     12980     12990     13000
             |--------+---------+---------+---------+---------+---------+---------+---------+---------+---------+---------+---------+---------|
contig022368 AGCTTATCATCACTCTGCAGATCTTTAAGCGTCACAGCAGTGATGCTAACATACTATAAGCAACAAAAACAACAAAAATCAAAGATTTTAAATCAGTAAATTCGTACATTTAGTCATCTGGAAAAGAGAA
TilTARs.A047 AGCTTATCATCACTCTGCAGATCTTTAAGCGTCACAGCAGTGATGCTAACATACTATAA
   Consensus AGCTTATCATCACTCTGCAGATCTTTAAGCGTCACAGCAGTGATGCTAACATACTATAA.......................................................................
```

```
             1561 1570      1580      1590      1600      1610      1620      1630      1640      1650      1660      1670      1680      1690
             |---------+---------+---------+---------+---------+---------+---------+---------+---------+---------+---------+---------+---------|
contig022375 GCCAACTGAAAATGGAAATGCAGAAAGGAACCGAGCTTTGTTTTCCAGATTTCCTCAACACTTCCTGCAGGAAGCCAACACTTCACTGGTCCAAAGCTGTGCTGTTGAACATTGTGCTGTCCTGCATCTC
TilTARs.A048            ATGGAAATGCAGAAAGGAACCGAGCTTTGTTTTCCAGATTTCCTCAACACTTCCTGCAGGAAGCCAACACTTCACTGGTCCAAAGCTGTGCTGTTGAACATTGTGCTGTCCTGCATCTC
   Consensus ...........ATGGAAATGCAGAAAGGAACCGAGCTTTGTTTTCCAGATTTCCTCAACACTTCCTGCAGGAAGCCAACACTTCACTGGTCCAAAGCTGTGCTGTTGAACATTGTGCTGTCCTGCATCTC

             1691 1700      1710      1720      1730      1740      1750      1760      1770      1780      1790      1800      1810      1820
             |---------+---------+---------+---------+---------+---------+---------+---------+---------+---------+---------+---------+---------|
contig022375 TTTGATCACTTCTGCTCTAAACCTTCTTGTCATCATCTCAGTCTCCCACTTCAGGCACAGATCAACTTCTCAGCTGAAATTATAGTTTAAGTTTTCAAGTCTTCAACACTATAATTACGTGATGAATGAA
TilTARs.A048 TTTGATCACTTCTGCTCTAAACCTTCTTGTCATCATCTCAGTCTCCCACTTCAGGCA-------------------------------------------------------------------------
   Consensus TTTGATCACTTCTGCTCTAAACCTTCTTGTCATCATCTCAGTCTCCCACTTCAGGCA.........................................................................

             1821 1830      1840      1850      1860      1870      1880      1890      1900      1910      1920      1930      1940      1950
             |---------+---------+---------+---------+---------+---------+---------+---------+---------+---------+---------+---------+---------|
contig022375 GTGAATCTGGCAGATTCTTGATGTATTTTAACCATGATGTAATGTTTTACTTGCCTAGATTGAATCATCCCTTACTAATGCAATTAGTGAACAACTGTGGCATTATTAATGTGTCTTATATTCTTTTTCT
TilTARs.A048 ----------------------------------------------------------------------------------------------------------------------------------
   Consensus ..................................................................................................................................

             1951 1960      1970      1980      1990      2000      2010      2020      2030      2040      2050      2060      2070      2080
             |---------+---------+---------+---------+---------+---------+---------+---------+---------+---------+---------+---------+---------|
contig022375 TTCTCTCTGCAGGCAGCTTCACACACCCACTAACATCCTGCTCCTCTCTCTGGCTGTCTCAGACTTTCTTGTGGGTCTTCTGTTGATGCCATTAGAAATCTTTAGGAGCACAGCCTGCTGGGTACTTGGT
TilTARs.A048 ---------------GCTTCACACACCCACTAACATCCTGCTCCTCTCTCTGGCTGTCTCAGACTTTCTTGTGGGTCTTCTGTTGATGCCATTAGAAATCTTTAGGAGCACAGCCTGCTGGGTACTTGGT
   Consensus ...............GCTTCACACACCCACTAACATCCTGCTCCTCTCTCTGGCTGTCTCAGACTTTCTTGTGGGTCTTCTGTTGATGCCATTAGAAATCTTTAGGAGCACAGCCTGCTGGGTACTTGGT

             2081 2090      2100      2110      2120      2130      2140      2150      2160      2170      2180      2190      2200      2210
             |---------+---------+---------+---------+---------+---------+---------+---------+---------+---------+---------+---------+---------|
contig022375 GATCTTATGTGTTCTGTTTATTGGTGTCTGACCGGCAACATTATCTGTGCTTCAATAGGGAACATAGTTCTTATATCAATTGACCGCTATGTGGCTATTTGTGACCCTTTGCATTACCCCACCAGAATGA
TilTARs.A048 GATCTTATGTGTTCTGTTTATTGGTGTCTGACCGGCAACATTATCTGTGCTTCAATAGGGAACATAGTTCTTATATCAATTGACCGCTATGTGGCTATTTGTGACCCTTTGCATTACCCCACCAGAATGA
   Consensus GATCTTATGTGTTCTGTTTATTGGTGTCTGACCGGCAACATTATCTGTGCTTCAATAGGGAACATAGTTCTTATATCAATTGACCGCTATGTGGCTATTTGTGACCCTTTGCATTACCCCACCAGAATGA

             2211 2220      2230      2240      2250      2260      2270      2280      2290      2300      2310      2320      2330      2340
             |---------+---------+---------+---------+---------+---------+---------+---------+---------+---------+---------+---------+---------|
contig022375 CTGTGGCAAGAGTCAGAGTCTGTGTTTGTCTGTGTTGGTTTTTTGCTATTTTCTACAGCAGTCTCTATACAACAGACTTCCTAATTGACCCAGGCAGGTATAATTCTTGCTATGGAGAGTGTGTGTTTGT
TilTARs.A048 CTGTGGCAAGAGTCAGAGTCTGTGTTTGTCTGTGTTGGTTTTTTGCTATTTTCTACAGCAGTCTCTATACAACAGACTTCCTAATTGACCCAGGCAGGTATAATTCTTGCTATGGAGAGTGTGTGTTTGT
   Consensus CTGTGGCAAGAGTCAGAGTCTGTGTTTGTCTGTGTTGGTTTTTTGCTATTTTCTACAGCAGTCTCTATACAACAGACTTCCTAATTGACCCAGGCAGGTATAATTCTTGCTATGGAGAGTGTGTGTTTGT

             2341 2350      2360      2370      2380      2390      2400      2410      2420      2430      2440      2450      2460      2470
             |---------+---------+---------+---------+---------+---------+---------+---------+---------+---------+---------+---------+---------|
contig022375 CATCAGTGCCATTGCAGGCATTTTTGACCTTGTTGTATCTTTTTTTGTTTCAGTTACTGTTATCATAGTTCTGTATATGAGAGTATTTGTGGTGGCTGTGTCTCAGGCTCGTGCCATGCGCTCTCATGTA
TilTARs.A048 CATCAGTGCCATTGCAGGCATTTTTGACCTTGTTGTATCTTTTTTTGTTTCAGTTACTGTTATCATAGTTCTGTATATGAGAGTATTTGTGGTGGCTGTGTCTCAGGCTCGTGCCATGCGCTCTCATGTA
   Consensus CATCAGTGCCATTGCAGGCATTTTTGACCTTGTTGTATCTTTTTTTGTTTCAGTTACTGTTATCATAGTTCTGTATATGAGAGTATTTGTGGTGGCTGTGTCTCAGGCTCGTGCCATGCGCTCTCATGTA

             2471 2480      2490      2500      2510      2520      2530      2540      2550      2560      2570      2580      2590      2600
             |---------+---------+---------+---------+---------+---------+---------+---------+---------+---------+---------+---------+---------|
contig022375 ACAACTGTCACACTTCAGCGTTCACTAAACCAAACAAACAAATCTGAGCTGAAAGCAGCCAGGACTCTTGGAATTCTTGTAGTTGTGTTTCTACTTTGTTTCTGCCCGTATTACTGTGTCTCTCTTGTAA
TilTARs.A048 ACAACTGTCACACTTCAGCGTTCACTAAACCAAACAAACAAATCTGAGCTGAAAGCAGCCAGGACTCTTGGAATTCTTGTAGTTGTGTTTCTACTTTGTTTCTGCCCGTATTACTGTGTCTCTCTTGTAA
   Consensus ACAACTGTCACACTTCAGCGTTCACTAAACCAAACAAACAAATCTGAGCTGAAAGCAGCCAGGACTCTTGGAATTCTTGTAGTTGTGTTTCTACTTTGTTTCTGCCCGTATTACTGTGTCTCTCTTGTAA

             2601 2610      2620      2630      2640      2650      2660      2670      2680      2690      2700      2710      2720      2730
             |---------+---------+---------+---------+---------+---------+---------+---------+---------+---------+---------+---------+---------|
contig022375 GAGATGAAAATAGCTCAATTGCCTCCACAGTTATAAGTGTGTTTTTTTTAAACTCCTGTTTAAACCCTTTGATCTATGCTATGTTCTACCCTTGGTTTAGAAAAGCTGTGAAACTAACTGTCACTCTACA
TilTARs.A048 GAGATGAAAATAGCTCAATTGCCTCCACAGTTATAAGTGTGTTTTTTTTAAACTCCTGTTTAAACCCTTTGATCTATGCTATGTTCTACCCTTGGTTTAGAAAAGCTGTGAAACTAACTGTCACTCTACA
   Consensus GAGATGAAAATAGCTCAATTGCCTCCACAGTTATAAGTGTGTTTTTTTTAAACTCCTGTTTAAACCCTTTGATCTATGCTATGTTCTACCCTTGGTTTAGAAAAGCTGTGAAACTAACTGTCACTCTACA

             2731 2740      2750      2760      2770      2780      2790      2800      2810      2820      2830      2840      2850      2860
             |---------+---------+---------+---------+---------+---------+---------+---------+---------+---------+---------+---------+---------|
contig022375 GATCCTCCGGACTGGCTCCTGTGAGGTCAACATACTGTAGAAATACTGTTCTAAAAGGGTTTGAAACTTAAAAGATATACAAGTAAATGGAACCATTCTTTTGGTCTCGGTAAGTAGAGCTTATTGAGCT
TilTARs.A048 GATCCTCCGGACTGGCTCCTGTGAGGTCAACATACTGTAG
   Consensus GATCCTCCGGACTGGCTCCTGTGAGGTCAACATACTGTAG..........................................................................................
```

```
             5071  5080      5090      5100      5110      5120      5130      5140      5150      5160      5170      5180      5190      5200
             |--------+---------+---------+---------+---------+---------+---------+---------+---------+---------+---------+---------+---------|
contig022377 TTGAGAGAAAGAGGTGGGTTATTATTTTTCTGAGTTTAAGAGATTAGTGGGGTGGGGGACAGATATAACAGCTCTGTGAGCAAAGCTCTCCTCAGAGAGCTGCAGGAGCTGTAGCTGCCTAGTGATGGAG
TilTARsp.A049                                                                                                                             ATGGAG
   Consensus ............................................................................................................................ATGGAG

             5201  5210      5220      5230      5240      5250      5260      5270      5280      5290      5300      5310      5320      5330
             |--------+---------+---------+---------+---------+---------+---------+---------+---------+---------+---------+---------+---------|
contig022377 ATACAGAAAGGAGCCGAGCTATGTTTTCCACAACATCTCAACAATTCCTGCATCAAGCCGACACTTCACTGGTCCAAAGCTGTGCTCCTGAACATTGTGCTGTCATGCATCTCTCTGATTACTGCTGTTC
TilTARsp.A049 ATACAGAAAGGAGCCGAGCTATGTTTTCCACAACATCTCAACAATTCCTGCATCAAGCCGACACTTCACTGGTCCAAAGCTGTGCTCCTGAACATTGTGCTGTCATGCATCTCTCTGATTACTGCTGTTC
   Consensus ATACAGAAAGGAGCCGAGCTATGTTTTCCACAACATCTCAACAATTCCTGCATCAAGCCGACACTTCACTGGTCCAAAGCTGTGCTCCTGAACATTGTGCTGTCATGCATCTCTCTGATTACTGCTGTTC

             5331  5340      5350      5360      5370      5380      5390      5400      5410      5420      5430      5440      5450      5460
             |--------+---------+---------+---------+---------+---------+---------+---------+---------+---------+---------+---------+---------|
contig022377 TCAGTCTTCATCTCAGTCTCTCATTTCAGGCAGAGATTAACTTATGAACTGAAACTATAGTTTAAGTTTTAGATATATGAAAACTTCAATTTACATCTAATGACTTAGTGATGAAAGAAAGAAGTGAATC
TilTARsp.A049 TCAGTCTTCATCTCAGTCTCTCATTT--------------------------------------------------------------------------------------------------------
   Consensus TCAGTCTTCATCTCAGTCTCTCATTT........................................................................................................

             5461  5470      5480      5490      5500      5510      5520      5530      5540      5550      5560      5570      5580      5590
             |--------+---------+---------+---------+---------+---------+---------+---------+---------+---------+---------+---------+---------|
contig022377 TGCCACATTCTTGGTGCACTTTAAACATTGCTGTAAAGTTTATTTTTCTAGATGTGTAATATTTGCTGTTTCTATCTCTCTGCAGGCAGCTTCACGCACCCAGTAACATCCTCCTCCTCTCTCTGGCTGT
TilTARsp.A049 -----------------------------------------------------------------------------------AGGCAGCTTCACGCACCCAGTAACATCCTCCTCCTCTCTCTGGCTGT
   Consensus ...................................................................................AGGCAGCTTCACGCACCCAGTAACATCCTCCTCCTCTCTCTGGCTGT

             5591  5600      5610      5620      5630      5640      5650      5660      5670      5680      5690      5700      5710      5720
             |--------+---------+---------+---------+---------+---------+---------+---------+---------+---------+---------+---------+---------|
contig022377 CTCAGTCTTTCTTGTGGGTCTCCTGTTGATGCCATTAGAAATTGCTAGAAACACGGCATGTTGGGTACTTGGTGATCTTATGTGTTCTGTTTATATCCATTTAACTGGCATTCTAATCTGTGCTTCAGTA
TilTARsp.A049 CTCAGTCTTTCTTGTGGGTCTCCTGTTGATGCCATTAGAAATTGCTAGAAACACGGCATGTTGGGTACTTGGTGATCTTATGTGTTCTGTTTATATCCATTTAACTGGCATTCTAATCTGTGCTTCAGTA
   Consensus CTCAGTCTTTCTTGTGGGTCTCCTGTTGATGCCATTAGAAATTGCTAGAAACACGGCATGTTGGGTACTTGGTGATCTTATGTGTTCTGTTTATATCCATTTAACTGGCATTCTAATCTGTGCTTCAGTA

             5721  5730      5740      5750      5760      5770      5780      5790      5800      5810      5820      5830      5840      5850
             |--------+---------+---------+---------+---------+---------+---------+---------+---------+---------+---------+---------+---------|
contig022377 GGGAATATAGTTCTAATATCAATTGACCGCTATGTGGCTATTTGTGACCCTTTACATTATCCCACAAAAATTACATTGGAAAGAGTTAAACTGAGTGTTTGTCTGTCTTGGCTTTTTTCAATTTTCTACA
TilTARsp.A049 GGGAATATAGTTCTAATATCAATTGACCGCTATGTGGCTATTTGTGACCCTTTACATTATCCCACAAAAATTACATTGGAAAGAGTTAAACTGAGTGTTTGTCTGTCTTGGCTTTTTTCAATTTTCTACA
   Consensus GGGAATATAGTTCTAATATCAATTGACCGCTATGTGGCTATTTGTGACCCTTTACATTATCCCACAAAAATTACATTGGAAAGAGTTAAACTGAGTGTTTGTCTGTCTTGGCTTTTTTCAATTTTCTACA

             5851  5860      5870      5880      5890      5900      5910      5920      5930      5940      5950      5960      5970      5980
             |--------+---------+---------+---------+---------+---------+---------+---------+---------+---------+---------+---------+---------|
contig022377 GCAGTCTTTATGCAAAGGATGTCCTGATTGAACCAGGAAAGTATCATTCCTGTTATGGAGAGTGTGTATTCCTTATTAATAATATTGCAGGTATGATTGACCTTGTTTTGACTTTTTTGGTTCCAGTTTC
TilTARsp.A049 GCAGTCTTTATGCAAAGGATGTCCTGATTGAACCAGGAAAGTATCATTCCTGTTATGGAGAGTGTGTATTCCTTATTAATAATATTGCAGGTATGATTGACCTTGTTTTGACTTTTTTGGTTCCAGTTTC
   Consensus GCAGTCTTTATGCAAAGGATGTCCTGATTGAACCAGGAAAGTATCATTCCTGTTATGGAGAGTGTGTATTCCTTATTAATAATATTGCAGGTATGATTGACCTTGTTTTGACTTTTTTGGTTCCAGTTTC

             5981  5990      6000      6010      6020      6030      6040      6050      6060      6070      6080      6090      6100      6110
             |--------+---------+---------+---------+---------+---------+---------+---------+---------+---------+---------+---------+---------|
contig022377 TGTCATCATAGTTCTATAAATGAGAGTATTTGGGGTGGCTGTGTCTCAGGCTCGTGCCATGCGCTCTAATGTTACAGCTGTCACACTTGAGCGTTCACTGAATCAAACAAACAATTCTGAGCTGAAAGCG
TilTARsp.A049 TGTCATCATAGTTCTATAAATGAGAGTATTTGGGGTGGCTGTGTCTCAGGCTCGTGCCATGCGCTCTAATGTTACAGCTGTCACACTTGAGCGTTCACTGAATCAAACAAACAATTCTGAGCTGAAAGCG
   Consensus TGTCATCATAGTTCTATAAATGAGAGTATTTGGGGTGGCTGTGTCTCAGGCTCGTGCCATGCGCTCTAATGTTACAGCTGTCACACTTGAGCGTTCACTGAATCAAACAAACAATTCTGAGCTGAAAGCG

             6111  6120      6130      6140      6150      6160      6170      6180      6190      6200      6210      6220      6230      6240
             |--------+---------+---------+---------+---------+---------+---------+---------+---------+---------+---------+---------+---------|
contig022377 GCCAGGACTCTGGGGGTTCTTGTAGTAGTGTTCCTGGCAAGCTACTGTCCATTTTATTGCTACACTCTTGTTGAAGAAAATGTGGTCAATGATCCATCTGCATCTACTGTGATCATTGTCTTTTACTTTA
TilTARsp.A049 GCCAGGACTCTGGGGGTTCTTGTAGTAGTGTTCCTGGCAAGCTACTGTCCATTTTATTGCTACACTCTTGTTGAAGAAAATGTGGTCAATGATCCATCTGCATCTACTGTGATCATTGTCTTTTACTTTA
   Consensus GCCAGGACTCTGGGGGTTCTTGTAGTAGTGTTCCTGGCAAGCTACTGTCCATTTTATTGCTACACTCTTGTTGAAGAAAATGTGGTCAATGATCCATCTGCATCTACTGTGATCATTGTCTTTTACTTTA

             6241  6250      6260      6270      6280      6290      6300      6310      6320      6330      6340      6350      6360      6370
             |--------+---------+---------+---------+---------+---------+---------+---------+---------+---------+---------+---------+---------|
contig022377 ACTCTTGTCTAAACCCTTTGAGCCATGCCCTGTTTTACCCCTGGTTTAGAAATGCTGTTAAACTCATCATCACTTTTCAGATATTCAAGCATGACAGTAGTGAGGCCAATATACTGTAAAAAAGACTAAC
TilTARsp.A049 ACTCTTGTCTAAACCCTTTGAGCCATGCCCTGTTTTACCCCTGGTTTAGAAATGCTGTTAAACTCATCATCACTTTTCAGATATTCAAGCATGACAGTAGTGAGGCCAATATACTGTAA
   Consensus ACTCTTGTCTAAACCCTTTGAGCCATGCCCTGTTTTACCCCTGGTTTAGAAATGCTGTTAAACTCATCATCACTTTTCAGATATTCAAGCATGACAGTAGTGAGGCCAATATACTGTAA...........

             6371  6380      6390      6400      6410      6420      6430      6440      6450      6460      6470      6480      6490      6500
```

```
            1066110670    10680     10690     10700     10710     10720     10730     10740     10750     10760     10770     10780     10790
            |---------+---------+---------+---------+---------+---------+---------+---------+---------+---------+---------+---------+---------|
contig022377 CTGCCTGGTGATGGAGAAAGGAGCTGAGCTCTGCTTTCCACAACTCCTCAACAGTTCCTGCAGGAAGCCAACACTTCACTGGTCCAAAGCTGTGCTCCTGAACATTGTGCTGTCCTGCATCTCTCTGATC
TilTARs.A050           ATGGAGAAAGGAGCTGAGCTCTGCTTTCCACAACTCCTCAACAGTTCCTGCAGGAAGCCAACACTTCACTGGTCCAAAGCTGTGCTCCTGAACATTGTGCTGTCCTGCATCTCTCTGATC
   Consensus ..........ATGGAGAAAGGAGCTGAGCTCTGCTTTCCACAACTCCTCAACAGTTCCTGCAGGAAGCCAACACTTCACTGGTCCAAAGCTGTGCTCCTGAACATTGTGCTGTCCTGCATCTCTCTGATC

            1079110800    10810     10820     10830     10840     10850     10860     10870     10880     10890     10900     10910     10920
            |---------+---------+---------+---------+---------+---------+---------+---------+---------+---------+---------+---------+---------|
contig022377 ACTGCTGGTCTCAATCTTCTTGTCATCATCTCAGTCTCCCACTTCAGGCAGAGATTGACTTAAACTACAGTTTAAATTTTATAAACTTTAAGAGTATGATCTATGTCATTTGATTTGTTGATGGACGAAT
TilTARs.A050 ACTGCTGGTCTCAATCTTCTTGTCATCATCTCAGTCTCCCACTTCAGGCAG-------------------------------------------------------------------------------
   Consensus ACTGCTGGTCTCAATCTTCTTGTCATCATCTCAGTCTCCCACTTCAGGCAG...............................................................................

            1092110930    10940     10950     10960     10970     10980     10990     11000     11010     11020     11030     11040     11050
            |---------+---------+---------+---------+---------+---------+---------+---------+---------+---------+---------+---------+---------|
contig022377 GAAGTGAATCTGACACATTCTTCATGTATTTTAACCAAACTGTATTGTGTCATTCATTATTAATGAATCATTTTCTTACTTAAGACACTTTGAAAAGAATAATGTCTATTATTTTCTCTTCTGTATCCCT
TilTARs.A050 ----------------------------------------------------------------------------------------------------------------------------------
   Consensus ..................................................................................................................................

            1105111060    11070     11080     11090     11100     11110     11120     11130     11140     11150     11160     11170     11180
            |---------+---------+---------+---------+---------+---------+---------+---------+---------+---------+---------+---------+---------|
contig022377 GCAGGCAGCTCCACACGCCCAGTAACATCCTCCTCCTCTCTCTGGCTGTGTCAGACTTTCTTGTTGGTCTCCTGTTAATTCCAGCTGAGATTTTTAGAATCACAGTCTGCTGGGTATTTGGTGAATCCAT
TilTARs.A050 --------CTCCACACGCCCAGTAACATCCTCCTCCTCTCTCTGGCTGTGTCAGACTTTCTTGTTGGTCTCCTGTTAATTCCAGCTGAGATTTTTAGAATCACAGTCTGCTGGGTATTTGGTGAATCCAT
   Consensus ........CTCCACACGCCCAGTAACATCCTCCTCCTCTCTCTGGCTGTGTCAGACTTTCTTGTTGGTCTCCTGTTAATTCCAGCTGAGATTTTTAGAATCACAGTCTGCTGGGTATTTGGTGAATCCAT

            1118111190    11200     11210     11220     11230     11240     11250     11260     11270     11280     11290     11300     11310
            |---------+---------+---------+---------+---------+---------+---------+---------+---------+---------+---------+---------+---------|
contig022377 GTGTTCACTTTATACATATCTAGGCTACATTGTTGTAACATCTTCAATAAGTAACATAGTTCTCATATCAGTTGATCGCTATGTGGCTATTTGTGATCCTCTGCATTATCCCAGCAGAATTACTGTGGCA
TilTARs.A050 GTGTTCACTTTATACATATCTAGGCTACATTGTTGTAACATCTTCAATAAGTAACATAGTTCTCATATCAGTTGATCGCTATGTGGCTATTTGTGATCCTCTGCATTATCCCAGCAGAATTACTGTGGCA
   Consensus GTGTTCACTTTATACATATCTAGGCTACATTGTTGTAACATCTTCAATAAGTAACATAGTTCTCATATCAGTTGATCGCTATGTGGCTATTTGTGATCCTCTGCATTATCCCAGCAGAATTACTGTGGCA

            1131111320    11330     11340     11350     11360     11370     11380     11390     11400     11410     11420     11430     11440
            |---------+---------+---------+---------+---------+---------+---------+---------+---------+---------+---------+---------+---------|
contig022377 AGAATCAAACTTAGTGTTTGTCTGTGTTGGTTTTATTCGGCTTTCTATAGCACTCTTTGTACAAAGAATATCCTGATTAAACCAGGCAGGTATAATTCCTGTTATGGAGAGTGTGTGTTTATCACCAGTG
TilTARs.A050 AGAATCAAACTTAGTGTTTGTCTGTGTTGGTTTTATTCGGCTTTCTATAGCACTCTTTGTACAAAGAATATCCTGATTAAACCAGGCAGGTATAATTCCTGTTATGGAGAGTGTGTGTTTATCACCAGTG
   Consensus AGAATCAAACTTAGTGTTTGTCTGTGTTGGTTTTATTCGGCTTTCTATAGCACTCTTTGTACAAAGAATATCCTGATTAAACCAGGCAGGTATAATTCCTGTTATGGAGAGTGTGTGTTTATCACCAGTG

            1144111450    11460     11470     11480     11490     11500     11510     11520     11530     11540     11550     11560     11570
            |---------+---------+---------+---------+---------+---------+---------+---------+---------+---------+---------+---------+---------|
contig022377 ATATTGCTGGCATACTTGACCTTGTTTTATCTTTTATCGTACCAGTTTCCATCATCATAGTTCTTTATATTAGAGTATTTGTGGTGGCTGTGTCTCAGGCTCGTGCCATGCGCTCTCATGTTACAGCTGC
TilTARs.A050 ATATTGCTGGCATACTTGACCTTGTTTTATCTTTTATCGTACCAGTTTCCATCATCATAGTTCTTTATATTAGAGTATTTGTGGTGGCTGTGTCTCAGGCTCGTGCCATGCGCTCTCATGTTACAGCTGC
   Consensus ATATTGCTGGCATACTTGACCTTGTTTTATCTTTTATCGTACCAGTTTCCATCATCATAGTTCTTTATATTAGAGTATTTGTGGTGGCTGTGTCTCAGGCTCGTGCCATGCGCTCTCATGTTACAGCTGC

            1157111580    11590     11600     11610     11620     11630     11640     11650     11660     11670     11680     11690     11700
            |---------+---------+---------+---------+---------+---------+---------+---------+---------+---------+---------+---------+---------|
contig022377 CACACTTCAGAGTTCATTGAATCAAACAAACAAATCTGAGTTGAAAGCAGCCAGGACTCTGGGGGTTCTTGTAGTTGTGTTTCTGGCCAGTTTCTGTCCATTTTATTGTTACTTTCTTGTTGTAGAAGAC
TilTARs.A050 CACACTTCAGAGTTCATTGAATCAAACAAACAAATCTGAGTTGAAAGCAGCCAGGACTCTGGGGGTTCTTGTAGTTGTGTTTCTGGCCAGTTTCTGTCCATTTTATTGTTACTTTCTTGTTGTAGAAGAC
   Consensus CACACTTCAGAGTTCATTGAATCAAACAAACAAATCTGAGTTGAAAGCAGCCAGGACTCTGGGGGTTCTTGTAGTTGTGTTTCTGGCCAGTTTCTGTCCATTTTATTGTTACTTTCTTGTTGTAGAAGAC

            1170111710    11720     11730     11740     11750     11760     11770     11780     11790     11800     11810     11820     11830
            |---------+---------+---------+---------+---------+---------+---------+---------+---------+---------+---------+---------+---------|
contig022377 ATAGTCAGTGATTCATCTGCCTCTATTGTGGTCATTGTCTATTATTTTAACTCTTGTCTAAACCCACTAATCTATGCCCTGTTTTACCCTTGGTTTAGAAATGCTGTTAAAGTCATCATCACTTTTCAGA
TilTARs.A050 ATAGTCAGTGATTCATCTGCCTCTATTGTGGTCATTGTCTATTATTTTAACTCTTGTCTAAACCCACTAATCTATGCCCTGTTTTACCCTTGGTTTAGAAATGCTGTTAAAGTCATCATCACTTTTCAGA
   Consensus ATAGTCAGTGATTCATCTGCCTCTATTGTGGTCATTGTCTATTATTTTAACTCTTGTCTAAACCCACTAATCTATGCCCTGTTTTACCCTTGGTTTAGAAATGCTGTTAAAGTCATCATCACTTTTCAGA

            1183111840    11850     11860     11870     11880     11890     11900     11910     11920     11930     11940     11950     11960
            |---------+---------+---------+---------+---------+---------+---------+---------+---------+---------+---------+---------+---------|
contig022377 TATTCAAGCATGACAGCAGTGAGGTCAACATACTATAGAAATATGGTGAAACCTGTTCCAGTAAGATGTTAAACCAGTCAACTGATACATCCAATAGTCTGGAAATACACATTTTTATCACTTTGTTTAA
TilTARs.A050 TATTCAAGCATGACAGCAGTGAGGTCAACATACTATAG
   Consensus TATTCAAGCATGACAGCAGTGAGGTCAACATACTATAG............................................................................................
```

```
              5071   5080   5090   5100   5110   5120   5130   5140   5150   5160   5170   5180   5190   5200
contig022378  GGAGGGGAGAAATGAAGGTCCAGAAACAGACAATTTCATCAGATTTGAAAGCTTAAACAGACTGTGTCTGCTGCTACTCTTGTCCTCATCTAGATGATGGAGAACAAGGACAGTATATGCTTTCCAGAAC
TilTARs.A051                                                                                               ATGATGGAGAACAAGGACAGTATATGCTTTCCAGAAC
   Consensus  .............................................................................................ATGATGGAGAACAAGGACAGTATATGCTTTCCAGAAC

              5201   5210   5220   5230   5240   5250   5260   5270   5280   5290   5300   5310   5320   5330
contig022378  TCTTCAACTCTTCCTGCAGGAGACGAGCACTTCACTGGTCTGAAACTGTGCCCCTAAATATCCTGCTGTGCTCCATCTCTGCGATCACTATAGCTCTGAACCTCCTTGTCATCATCTCAGTCTCCTACTT
TilTARs.A051  TCTTCAACTCTTCCTGCAGGAGACGAGCACTTCACTGGTCTGAAACTGTGCCCCTAAATATCCTGCTGTGCTCCATCTCTGCGATCACTATAGCTCTGAACCTCCTTGTCATCATCTCAGTCTCCTACTT
   Consensus  TCTTCAACTCTTCCTGCAGGAGACGAGCACTTCACTGGTCTGAAACTGTGCCCCTAAATATCCTGCTGTGCTCCATCTCTGCGATCACTATAGCTCTGAACCTCCTTGTCATCATCTCAGTCTCCTACTT

              5331   5340   5350   5360   5370   5380   5390   5400   5410   5420   5430   5440   5450   5460
contig022378  CAGGCAGATTCCATTTTCTATTAGATTTAATTTGTTAAGTTTTGAAATATGATCGTGCAAAACCTGATGTACAGAAAACCACCGCTACACCTTTCTCCAAAACAAGTTATTTTAATTGTTTTACAAAGTT
TilTARs.A051  CAGGCAG---------------------------------------------------------------------------------------------------------------------------
   Consensus  CAGGCAG...........................................................................................................................

              5461   5470   5480   5490   5500   5510   5520   5530   5540   5550   5560   5570   5580   5590
contig022378  TAATTTAACATAATGTTATGGCAAGTTATGTGGGACTAACATAAATTTTAAGGGAAGATTTTTTTGGTAATGGTGCTTTTTTGACTTATTCCAGGCAGCTCCACACACCCACTAACATCCTCCTCCTCTC
TilTARs.A051  --------------------------------------------------------------------------------------------------CTCCACACACCCACTAACATCCTCCTCCTCTC
   Consensus  ..................................................................................................CTCCACACACCCACTAACATCCTCCTCCTCTC

              5591   5600   5610   5620   5630   5640   5650   5660   5670   5680   5690   5700   5710   5720
contig022378  TTTGGCTGTGTCAGACTTTCTTGTGGGGTTCCTGTTGCTGCCTGGAGAAATCTTCCTAGGAATAGCCTGCTGGGCTTTCGGTAACCTTTTGTGTTCTCTCTTTAATTATGTGTCTTTCATCATTACCTCG
TilTARs.A051  TTTGGCTGTGTCAGACTTTCTTGTGGGGTTCCTGTTGCTGCCTGGAGAAATCTTCCTAGGAATAGCCTGCTGGGCTTTCGGTAACCTTTTGTGTTCTCTCTTTAATTATGTGTCTTTCATCATTACCTCG
   Consensus  TTTGGCTGTGTCAGACTTTCTTGTGGGGTTCCTGTTGCTGCCTGGAGAAATCTTCCTAGGAATAGCCTGCTGGGCTTTCGGTAACCTTTTGTGTTCTCTCTTTAATTATGTGTCTTTCATCATTACCTCG

              5721   5730   5740   5750   5760   5770   5780   5790   5800   5810   5820   5830   5840   5850
contig022378  GCTTCAGTGGGAAACATGGTTCTCATATCAGTTGACCGCTATGTGGCGATTTGTTATCCTCTGCATTACTCTACCAGAATTACTGTGACCAGAGTTAAACGCTCTGTTTGTTTGTGTTGGCTCTGTTGTG
TilTARs.A051  GCTTCAGTGGGAAACATGGTTCTCATATCAGTTGACCGCTATGTGGCGATTTGTTATCCTCTGCATTACTCTACCAGAATTACTGTGACCAGAGTTAAACGCTCTGTTTGTTTGTGTTGGCTCTGTTGTG
   Consensus  GCTTCAGTGGGAAACATGGTTCTCATATCAGTTGACCGCTATGTGGCGATTTGTTATCCTCTGCATTACTCTACCAGAATTACTGTGACCAGAGTTAAACGCTCTGTTTGTTTGTGTTGGCTCTGTTGTG

              5851   5860   5870   5880   5890   5900   5910   5920   5930   5940   5950   5960   5970   5980
contig022378  TTTTGTACAGTAGTGCCCTTTTAAAAGATGAGCTGATTCAACCAGGAGGGCATAATTCCTGCTATGGAGAATGTGTTTTTGTCATCAACTTTATTGCAGGAACTGTTGATCTTTTGTTAACCTTTATTGT
TilTARs.A051  TTTTGTACAGTAGTGCCCTTTTAAAAGATGAGCTGATTCAACCAGGAGGGCATAATTCCTGCTATGGAGAATGTGTTTTTGTCATCAACTTTATTGCAGGAACTGTTGATCTTTTGTTAACCTTTATTGT
   Consensus  TTTTGTACAGTAGTGCCCTTTTAAAAGATGAGCTGATTCAACCAGGAGGGCATAATTCCTGCTATGGAGAATGTGTTTTTGTCATCAACTTTATTGCAGGAACTGTTGATCTTTTGTTAACCTTTATTGT

              5981   5990   6000   6010   6020   6030   6040   6050   6060   6070   6080   6090   6100   6110
contig022378  TCCTATTTCTGTTATCGTAGTTCTGTATATGAGAGTATTTGTAGTGGCTGTGTCTCAGGCCCGTGCCATGCGCTCTCATGTTACAGCTGTCACACTGCAGCTTTCAGTGAATCTCACAACAAAGAAATCA
TilTARs.A051  TCCTATTTCTGTTATCGTAGTTCTGTATATGAGAGTATTTGTAGTGGCTGTGTCTCAGGCCCGTGCCATGCGCTCTCATGTTACAGCTGTCACACTGCAGCTTTCAGTGAATCTCACAACAAAGAAATCA
   Consensus  TCCTATTTCTGTTATCGTAGTTCTGTATATGAGAGTATTTGTAGTGGCTGTGTCTCAGGCCCGTGCCATGCGCTCTCATGTTACAGCTGTCACACTGCAGCTTTCAGTGAATCTCACAACAAAGAAATCA

              6111   6120   6130   6140   6150   6160   6170   6180   6190   6200   6210   6220   6230   6240
contig022378  GAGTTGAAAGCAGGAAGGACTCTTGGTGTTCTAATAGTTGTGTTTCTAATGTGTTTCTGTCCATACTATTGCGCTTCTTTTGCAGTGGAAGACTCACTCAGTGAGTCATCCACATCCCTTGTCCGTTATC
TilTARs.A051  GAGTTGAAAGCAGGAAGGACTCTTGGTGTTCTAATAGTTGTGTTTCTAATGTGTTTCTGTCCATACTATTGCGCTTCTTTTGCAGTGGAAGACTCACTCAGTGAGTCATCCACATCCCTTGTCCGTTATC
   Consensus  GAGTTGAAAGCAGGAAGGACTCTTGGTGTTCTAATAGTTGTGTTTCTAATGTGTTTCTGTCCATACTATTGCGCTTCTTTTGCAGTGGAAGACTCACTCAGTGAGTCATCCACATCCCTTGTCCGTTATC

              6241   6250   6260   6270   6280   6290   6300   6310   6320   6330   6340   6350   6360   6370
contig022378  TCTTCTATTTTAACTCCTGTTTGAACCCTGTGATCTATGCACTATTTTACCCCTGGTTTAGAAAAGCTATTAAACACATTGTCATGCTTCAGATATTTCACACTGGCTCCCGTGAGGCCAACATACTATA
TilTARs.A051  TCTTCTATTTTAACTCCTGTTTGAACCCTGTGATCTATGCACTATTTTACCCCTGGTTTAGAAAAGCTATTAAACACATTGTCATGCTTCAGATATTTCACACTGGCTCCCGTGAGGCCAACATACTATA
   Consensus  TCTTCTATTTTAACTCCTGTTTGAACCCTGTGATCTATGCACTATTTTACCCCTGGTTTAGAAAAGCTATTAAACACATTGTCATGCTTCAGATATTTCACACTGGCTCCCGTGAGGCCAACATACTATA

              6371   6380   6390   6400   6410   6420   6430   6440   6450   6460   6470   6480   6490   6500
contig022378  GAGAGGACCTAAATGAGTGAATGAATGATATCTGCGGAACATATATTTTAACCCTTAACCCGTATTATGTTAAGCAAGCTGCCTGGCATTATTTTGTTGTTTTAGCTCTAAAACTGTTGTAAAATCAGCT
TilTARs.A051  G
   Consensus  G.................................................................................................................................
```

```
            7151  7160      7170      7180      7190      7200      7210      7220      7230      7240      7250      7260      7270      7280
            |--------+---------+---------+---------+---------+---------+---------+---------+---------+---------+---------+---------+---------|
contig022379 ACAGAGAGCTACAGACCTCTGCAGGTCTGAGAAAATGGATACACAGGATGTGGCAGAGCTCTGTTTTCCACAACTCTTCAACACCTCCTGCAAGAAACCTATAACTCCTCTGTCAGAATTTGTGTTCCTT
TilTARs.A052                                   ATGGATACACAGGATGTGGCAGAGCTCTGTTTTCCACAACTCTTCAACACCTCCTGCAAGAAACCTATAACTCCTCTGTCAGAATTTGTGTTCCTT
   Consensus ..................................ATGGATACACAGGATGTGGCAGAGCTCTGTTTTCCACAACTCTTCAACACCTCCTGCAAGAAACCTATAACTCCTCTGTCAGAATTTGTGTTCCTT

            7281  7290      7300      7310      7320      7330      7340      7350      7360      7370      7380      7390      7400      7410
            |--------+---------+---------+---------+---------+---------+---------+---------+---------+---------+---------+---------+---------|
contig022379 CATGTTGTGTTGTCCTCCATCTCGCTGCTAACTGTGACTCTCAACCTGCTCGTCATCATCTCAGTCTCCCACTACAGGCAGAGTTGTTGTTATTTTTTTAATAGCTGTGTGTAGCTTATACATTCTGTAA
TilTARs.A052 CATGTTGTGTTGTCCTCCATCTCGCTGCTAACTGTGACTCTCAACCTGCTCGTCATCATCTCAGTCTCCCACTACAGGCAG-------------------------------------------------
   Consensus CATGTTGTGTTGTCCTCCATCTCGCTGCTAACTGTGACTCTCAACCTGCTCGTCATCATCTCAGTCTCCCACTACAGGCAG.................................................

            7411  7420      7430      7440      7450      7460      7470      7480      7490      7500      7510      7520      7530      7540
            |--------+---------+---------+---------+---------+---------+---------+---------+---------+---------+---------+---------+---------|
contig022379 GCTAATTAACTTTTTATTTTTCTGTGACCTCAGTGGTGTTTACAATTAAACCATCTGATATAGAAGTGGTCGATTAGAAATGATCAAAACTGTTTTTCTCTTATCAAGAGCTAATGTTTTATTGTTTGTT
TilTARs.A052 ----------------------------------------------------------------------------------------------------------------------------------
   Consensus ..................................................................................................................................

            7541  7550      7560      7570      7580      7590      7600      7610      7620      7630      7640      7650      7660      7670
            |--------+---------+---------+---------+---------+---------+---------+---------+---------+---------+---------+---------+---------|
contig022379 GGGTTGCTTTAACCCTCTCAGGCTCAAATTAAGTTTTAGTTACAGGAAAAATCTGATATTTGGGCAAAATTATCAAAAAAAACCCCTCATAAAATATGTATGTGGGGTAATCAGGTTGTTAGTTTTTAAC
TilTARs.A052 ----------------------------------------------------------------------------------------------------------------------------------
   Consensus ..................................................................................................................................

            7671  7680      7690      7700      7710      7720      7730      7740      7750      7760      7770      7780      7790      7800
            |--------+---------+---------+---------+---------+---------+---------+---------+---------+---------+---------+---------+---------|
contig022379 TGTTGCAAATCGGCAACGCCTGCCTCGAGAGGGTTAATTTGGCGATTTGAGGACTGTTAATTTAATAATTTTTCTGTCTTCTTGCAGGCAGCTTCACACACCCACTAACATCCTCCTCCTCTCTCTGGCT
TilTARs.A052 -------------------------------------------------------------------------------------------CTTCACACACCCACTAACATCCTCCTCCTCTCTCTGGCT
   Consensus ...........................................................................................CTTCACACACCCACTAACATCCTCCTCCTCTCTCTGGCT

            7801  7810      7820      7830      7840      7850      7860      7870      7880      7890      7900      7910      7920      7930
            |--------+---------+---------+---------+---------+---------+---------+---------+---------+---------+---------+---------+---------|
contig022379 GTCTCAGACTTTCTTGTTGGTCTCCTGTTGATGCCTGGAGAAATCCTCAGAAATACAGCCTGCTGGTTTCTCGGTGACCTCACCTGTTCTATGTACAATTATATGTCTTTCATTGTTACCTCTACCTCAG
TilTARs.A052 GTCTCAGACTTTCTTGTTGGTCTCCTGTTGATGCCTGGAGAAATCCTCAGAAATACAGCCTGCTGGTTTCTCGGTGACCTCACCTGTTCTATGTACAATTATATGTCTTTCATTGTTACCTCTACCTCAG
   Consensus GTCTCAGACTTTCTTGTTGGTCTCCTGTTGATGCCTGGAGAAATCCTCAGAAATACAGCCTGCTGGTTTCTCGGTGACCTCACCTGTTCTATGTACAATTATATGTCTTTCATTGTTACCTCTACCTCAG

            7931  7940      7950      7960      7970      7980      7990      8000      8010      8020      8030      8040      8050      8060
            |--------+---------+---------+---------+---------+---------+---------+---------+---------+---------+---------+---------+---------|
contig022379 TGGGAGACATGGTGCTAATATCAATTGACCGCTATTTGGCTATTTGTGACCCTCTGCATTACCCCACCAGAATCACAGACAGAAGAGTGAAACTCTCTATCTGTCTGTGTTGGCTCTCTTCTGTCTTCTA
TilTARs.A052 TGGGAGACATGGTGCTAATATCAATTGACCGCTATTTGGCTATTTGTGACCCTCTGCATTACCCCACCAGAATCACAGACAGAAGAGTGAAACTCTCTATCTGTCTGTGTTGGCTCTCTTCTGTCTTCTA
   Consensus TGGGAGACATGGTGCTAATATCAATTGACCGCTATTTGGCTATTTGTGACCCTCTGCATTACCCCACCAGAATCACAGACAGAAGAGTGAAACTCTCTATCTGTCTGTGTTGGCTCTCTTCTGTCTTCTA

            8061  8070      8080      8090      8100      8110      8120      8130      8140      8150      8160      8170      8180      8190
            |--------+---------+---------+---------+---------+---------+---------+---------+---------+---------+---------+---------+---------|
contig022379 TAGCAGCCTGTTTGTAAAGGATGATCTAACTCAACCAGGGAAGCATAATTCCTGCTATGGAGAATGTACAATTGTTGTTGACTTAATTACAGGAATTATTGACCTTCTTTTAACCTTTTTTGTTCCAGTT
TilTARs.A052 TAGCAGCCTGTTTGTAAAGGATGATCTAACTCAACCAGGGAAGCATAATTCCTGCTATGGAGAATGTACAATTGTTGTTGACTTAATTACAGGAATTATTGACCTTCTTTTAACCTTTTTTGTTCCAGTT
   Consensus TAGCAGCCTGTTTGTAAAGGATGATCTAACTCAACCAGGGAAGCATAATTCCTGCTATGGAGAATGTACAATTGTTGTTGACTTAATTACAGGAATTATTGACCTTCTTTTAACCTTTTTTGTTCCAGTT

            8191  8200      8210      8220      8230      8240      8250      8260      8270      8280      8290      8300      8310      8320
            |--------+---------+---------+---------+---------+---------+---------+---------+---------+---------+---------+---------+---------|
contig022379 ACTGTCATTGTAGTTCTGTATCTGAGAGTATTTGTGGTGGCTGTGTCTCAGGCTCGTGCCATGCGCTCTCATGTTACAGCTACTGCTCTGCAGCTTTCAGTGACTCTAACAACAAAGAAATCAGAGTTAA
TilTARs.A052 ACTGTCATTGTAGTTCTGTATCTGAGAGTATTTGTGGTGGCTGTGTCTCAGGCTCGTGCCATGCGCTCTCATGTTACAGCTACTGCTCTGCAGCTTTCAGTGACTCTAACAACAAAGAAATCAGAGTTAA
   Consensus ACTGTCATTGTAGTTCTGTATCTGAGAGTATTTGTGGTGGCTGTGTCTCAGGCTCGTGCCATGCGCTCTCATGTTACAGCTACTGCTCTGCAGCTTTCAGTGACTCTAACAACAAAGAAATCAGAGTTAA

            8321  8330      8340      8350      8360      8370      8380      8390      8400      8410      8420      8430      8440      8450
            |--------+---------+---------+---------+---------+---------+---------+---------+---------+---------+---------+---------+---------|
contig022379 AAGCAGCCAGGACTCTGGGTGTTCTTGTAGTTGTGTTTCTACTGTGTTTCTGTCCATATTATTATTGTGTTACTCTTGCCAGGGATGACCCACTCAATAGCTCATCTGTATCCTTTGTGCTCTATCTGTT
TilTARs.A052 AAGCAGCCAGGACTCTGGGTGTTCTTGTAGTTGTGTTTCTACTGTGTTTCTGTCCATATTATTATTGTGTTACTCTTGCCAGGGATGACCCACTCAATAGCTCATCTGTATCCTTTGTGCTCTATCTGTT
   Consensus AAGCAGCCAGGACTCTGGGTGTTCTTGTAGTTGTGTTTCTACTGTGTTTCTGTCCATATTATTATTGTGTTACTCTTGCCAGGGATGACCCACTCAATAGCTCATCTGTATCCTTTGTGCTCTATCTGTT

            8451  8460      8470      8480      8490      8500      8510      8520      8530      8540      8550      8560      8570      8580
            |--------+---------+---------+---------+---------+---------+---------+---------+---------+---------+---------+---------+---------|
contig022379 CTATTTTAACTCCTGTCTAAACCCTTTGATCTATGCACTGTTCTACCCCTGGTTTAGAAAAGCTGTGAAACTCATAATCTCTTTACACATACTGCAGCCTGGCTCCTGTGAGATTAGCATTTTGTAAAAA
TilTARs.A052 CTATTTTAACTCCTGTCTAAACCCTTTGATCTATGCACTGTTCTACCCCTGGTTTAGAAAAGCTGTGAAACTCATAATCTCTTTACACATACTGCAGCCTGGCTCCTGTGAGATTAGCATTTTGTAA
   Consensus CTATTTTAACTCCTGTCTAAACCCTTTGATCTATGCACTGTTCTACCCCTGGTTTAGAAAAGCTGTGAAACTCATAATCTCTTTACACATACTGCAGCCTGGCTCCTGTGAGATTAGCATTTTGTAA...
```

```
             1261112620     12630     12640     12650     12660     12670     12680     12690     12700     12710     12720     12730     12740
             |--------+---------+---------+---------+---------+---------+---------+---------+---------+---------+---------+---------+---------|
contig022382 TTTTTGTTCATAATGCTGCGACCAGAGCTGCAGTTCTTCACTAGTCTGTGACAATGGGCACACAGGAAAAATCTGAGCTCTGCTTTCCACAACTCTTCAACATGTCCTGCAAAAAGCCTGCAATTGCTCG
TilTARs.A053                                                      ATGGGCACACAGGAAAAATCTGAGCTCTGCTTTCCACAACTCTTCAACATGTCCTGCAAAAAGCCTGCAATTGCTCG
   Consensus .....................................................ATGGGCACACAGGAAAAATCTGAGCTCTGCTTTCCACAACTCTTCAACATGTCCTGCAAAAAGCCTGCAATTGCTCG

             1274112750     12760     12770     12780     12790     12800     12810     12820     12830     12840     12850     12860     12870
             |--------+---------+---------+---------+---------+---------+---------+---------+---------+---------+---------+---------+---------|
contig022382 GTCCAAAGCTGTGTTCCTTCATATTGTATTGTCCTCAGTTTCTTTTCTGACGGTGGCTCTCAACTTGCTTGTCATCATCTCAGTCTCCCACTTCAGGTAGAAACAACTTTTTTTTTTTTTTACTAGCTAC
TilTARs.A053 GTCCAAAGCTGTGTTCCTTCATATTGTATTGTCCTCAGTTTCTTTTCTGACGGTGGCTCTCAACTTGCTTGTCATCATCTCAGTCTCCCACTTCAGG---------------------------------
   Consensus GTCCAAAGCTGTGTTCCTTCATATTGTATTGTCCTCAGTTTCTTTTCTGACGGTGGCTCTCAACTTGCTTGTCATCATCTCAGTCTCCCACTTCAGG.................................

             1287112880     12890     12900     12910     12920     12930     12940     12950     12960     12970     12980     12990     13000
             |--------+---------+---------+---------+---------+---------+---------+---------+---------+---------+---------+---------+---------|
contig022382 ACTATTCTAGTATTTCAAATATTTGAGCTAGTAATAAAAGATTCTTCACATGTACAGATTTATACTTGAGAATATTTACATTTCATCAATTTTACTTTGATACCAGAAAACATACTATACTCATTGTCTT
TilTARs.A053 ----------------------------------------------------------------------------------------------------------------------------------
   Consensus ..................................................................................................................................

             1300113010     13020     13030     13040     13050     13060     13070     13080     13090     13100     13110     13120     13130
             |--------+---------+---------+---------+---------+---------+---------+---------+---------+---------+---------+---------+---------|
contig022382 ATAAGTAATACTATGTTCTGAATCAGTTTAACGTTGATAATATGCTGCTGTATGACATTTAATAGCTCTTTGGCTTGTTTCATTATAGAGGTTTGAGGACTTGTTATTTTTCTGTCTCCCTGCAGGCAGC
TilTARs.A053 ------------------------------------------------------------------------------------------------------------------------------CAGC
   Consensus ..............................................................................................................................CAGC

             1313113140     13150     13160     13170     13180     13190     13200     13210     13220     13230     13240     13250     13260
             |--------+---------+---------+---------+---------+---------+---------+---------+---------+---------+---------+---------+---------|
contig022382 TCCACACACCCACTAACATCCTGCTCCTCTCTCTGGCTGTCTCAGACTTTCTTGTTGGCCTCCTATTGTCACCTGCAGAAATCCTCCGAAGTACAGCCTGTTGGTTTCTTGGTCAGCTCACATGTTTAAT
TilTARs.A053 TCCACACACCCACTAACATCCTGCTCCTCTCTCTGGCTGTCTCAGACTTTCTTGTTGGCCTCCTATTGTCACCTGCAGAAATCCTCCGAAGTACAGCCTGTTGGTTTCTTGGTCAGCTCACATGTTTAAT
   Consensus TCCACACACCCACTAACATCCTGCTCCTCTCTCTGGCTGTCTCAGACTTTCTTGTTGGCCTCCTATTGTCACCTGCAGAAATCCTCCGAAGTACAGCCTGTTGGTTTCTTGGTCAGCTCACATGTTTAAT

             1326113270     13280     13290     13300     13310     13320     13330     13340     13350     13360     13370     13380     13390
             |--------+---------+---------+---------+---------+---------+---------+---------+---------+---------+---------+---------+---------|
contig022382 GTATATTTTTGTGTCCTTGACTGTTACCTCTGCCTCAGTGGGCATTATGGTGCTGATATCAGCTGACCGCTATGTGGCTATTTGTGACCCTTTGAATTACCCCATCAGGATCACTGACAGAAGAGTTCAA
TilTARs.A053 GTATATTTTTGTGTCCTTGACTGTTACCTCTGCCTCAGTGGGCATTATGGTGCTGATATCAGCTGACCGCTATGTGGCTATTTGTGACCCTTTGAATTACCCCATCAGGATCACTGACAGAAGAGTTCAA
   Consensus GTATATTTTTGTGTCCTTGACTGTTACCTCTGCCTCAGTGGGCATTATGGTGCTGATATCAGCTGACCGCTATGTGGCTATTTGTGACCCTTTGAATTACCCCATCAGGATCACTGACAGAAGAGTTCAA

             1339113400     13410     13420     13430     13440     13450     13460     13470     13480     13490     13500     13510     13520
             |--------+---------+---------+---------+---------+---------+---------+---------+---------+---------+---------+---------+---------|
contig022382 CTCTGTGTTTGTCTGTGTTGGCTCTGCTCCATTGCCTTCAGCTGTTTCCTTGTAAGAGATGACCTGCATCAAACAAGAAAACAAAATTCCTGCTATGGAAAATGTGTAGTTGTTGTTCAATACATTGCAG
TilTARs.A053 CTCTGTGTTTGTCTGTGTTGGCTCTGCTCCATTGCCTTCAGCTGTTTCCTTGTAAGAGATGACCTGCATCAAACAAGAAAACAAAATTCCTGCTATGGAAAATGTGTAGTTGTTGTTCAATACATTGCAG
   Consensus CTCTGTGTTTGTCTGTGTTGGCTCTGCTCCATTGCCTTCAGCTGTTTCCTTGTAAGAGATGACCTGCATCAAACAAGAAAACAAAATTCCTGCTATGGAAAATGTGTAGTTGTTGTTCAATACATTGCAG

             1352113530     13540     13550     13560     13570     13580     13590     13600     13610     13620     13630     13640     13650
             |--------+---------+---------+---------+---------+---------+---------+---------+---------+---------+---------+---------+---------|
contig022382 GAGTTGTTGACCTCATTTTAACCTTCATTGTACCAGTTAGTGTCATCATAGTTCTGTATATGAGAGTATTTGTGGTGGCTGTGTCTCAGGCCCGTGCTATGCGCTCTCATGTTACAGCTGTCACAAGTCA
TilTARs.A053 GAGTTGTTGACCTCATTTTAACCTTCATTGTACCAGTTAGTGTCATCATAGTTCTGTATATGAGAGTATTTGTGGTGGCTGTGTCTCAGGCCCGTGCTATGCGCTCTCATGTTACAGCTGTCACAAGTCA
   Consensus GAGTTGTTGACCTCATTTTAACCTTCATTGTACCAGTTAGTGTCATCATAGTTCTGTATATGAGAGTATTTGTGGTGGCTGTGTCTCAGGCCCGTGCTATGCGCTCTCATGTTACAGCTGTCACAAGTCA

             1365113660     13670     13680     13690     13700     13710     13720     13730     13740     13750     13760     13770     13780
             |--------+---------+---------+---------+---------+---------+---------+---------+---------+---------+---------+---------+---------|
contig022382 CCTTCCAGTGACTCTAACAAAAAAGTCTGAACTAAAAGCAGCCAGGACTCTGGGTGTTCTTGTTCTTGTGTTTCTAATGTGTTTCTGCCCTTATTACTGTGTTTCTCTTGTAGGAGAAGAATTCATCAAT
TilTARs.A053 CCTTCCAGTGACTCTAACAAAAAAGTCTGAACTAAAAGCAGCCAGGACTCTGGGTGTTCTTGTTCTTGTGTTTCTAATGTGTTTCTGCCCTTATTACTGTGTTTCTCTTGTAGGAGAAGAATTCATCAAT
   Consensus CCTTCCAGTGACTCTAACAAAAAAGTCTGAACTAAAAGCAGCCAGGACTCTGGGTGTTCTTGTTCTTGTGTTTCTAATGTGTTTCTGCCCTTATTACTGTGTTTCTCTTGTAGGAGAAGAATTCATCAAT

             1378113790     13800     13810     13820     13830     13840     13850     13860     13870     13880     13890     13900     13910
             |--------+---------+---------+---------+---------+---------+---------+---------+---------+---------+---------+---------+---------|
contig022382 AGTTCATCTGCTTCTTTTGTGGCCTATCTGTTTGGTTTAAACTCATGTCTAAACCCTTTGATCTATGCTATGTTCTACCCCTGGTTTAGAAAAGCTGTGAAACTAGTTGTCACTCTACAGATACTGCAGC
TilTARs.A053 AGTTCATCTGCTTCTTTTGTGGCCTATCTGTTTGGTTTAAACTCATGTCTAAACCCTTTGATCTATGCTATGTTCTACCCCTGGTTTAGAAAAGCTGTGAAACTAGTTGTCACTCTACAGATACTGCAGC
   Consensus AGTTCATCTGCTTCTTTTGTGGCCTATCTGTTTGGTTTAAACTCATGTCTAAACCCTTTGATCTATGCTATGTTCTACCCCTGGTTTAGAAAAGCTGTGAAACTAGTTGTCACTCTACAGATACTGCAGC

             1391113920     13930     13940     13950     13960     13970     13980     13990     14000     14010     14020     14030     14040
             |--------+---------+---------+---------+---------+---------+---------+---------+---------+---------+---------+---------+---------|
contig022382 CTGGCTCCTGTGAGGTAAGTATTCTGTAAACAGATCAAAGCATTTAAACAATTGTTTTCAGGTTCAGCTTTTAATAAAAAATCTTAATCTTAACCAAAAACAATAAAACAACCTTTTTGTTGACTTCATC
TilTARs.A053 CTGGCTCCTGTGAGGTAAGTATTCTGTAA
   Consensus CTGGCTCCTGTGAGGTAAGTATTCTGTAA.....................................................................................................
```

```
             911     920       930       940       950       960       970       980       990       1000      1010      1020      1030      1040
contig022383 CCAGAGCTGCAGCTCTCTGCAGGTGTTTGAAGATGGAGACCCAGGACGAAGCAGAGCTCTGCTTTCCACAACTCTTCAACATCTCCTGCAAGAAGCCTAAAACATCCCTGTCTCAAGTTTTGCTCCCTTA
TilTARs.A054                                 ATGGAGACCCAGGACGAAGCAGAGCTCTGCTTTCCACAACTCTTCAACATCTCCTGCAAGAAGCCTAAAACATCCCTGTCTCAAGTTTTGCTCCCTTA
   Consensus ................................ATGGAGACCCAGGACGAAGCAGAGCTCTGCTTTCCACAACTCTTCAACATCTCCTGCAAGAAGCCTAAAACATCCCTGTCTCAAGTTTTGCTCCCTTA

             1041    1050      1060      1070      1080      1090      1100      1110      1120      1130      1140      1150      1160      1170
contig022383 CGTTGTGGTGTATTCAGTCTCTCTGCTAACTGTGGCTCTAAACCTGCTCGTCATTGTTTCAGTCTCCCACTTCAGGCAGAGATTCACTTTTTTAAACTTCTGCTGTGGCTTTACTGCTACTACAACTACA
TilTARs.A054 CGTTGTGGTGTATTCAGTCTCTCTGCTAACTGTGGCTCTAAACCTGCTCGTCATTGTTTCAGTCTCCCACTTCAG-------------------------------------------------------
   Consensus CGTTGTGGTGTATTCAGTCTCTCTGCTAACTGTGGCTCTAAACCTGCTCGTCATTGTTTCAGTCTCCCACTTCAG.......................................................

             1171    1180      1190      1200      1210      1220      1230      1240      1250      1260      1270      1280      1290      1300
contig022383 ACTCTACTACTAATTACAACAAAATTTAAGTTAATTTATATCTTATGGGTAGACTATTATCAGACAATAATGTACTCCACTGCTATTACATAATAATAATAATTATGTATCTATAGATTCTATCCTCTAC
TilTARs.A054 ----------------------------------------------------------------------------------------------------------------------------------
   Consensus ..................................................................................................................................

             1301    1310      1320      1330      1340      1350      1360      1370      1380      1390      1400      1410      1420      1430
contig022383 TACCCTTGCAACTGTCTACACTTTAGGATAGGACAGCTGTAATATAATTTTTTGTTTAGATTGTTTCAGTATAGACACATAATAATGACTTGTTGATATTTCTCTCTCTTCTTTCAGACAGCTCCACACA
TilTARs.A054 ---------------------------------------------------------------------------------------------------------------------ACAGCTCCACACA
   Consensus .....................................................................................................................ACAGCTCCACACA

             1431    1440      1450      1460      1470      1480      1490      1500      1510      1520      1530      1540      1550      1560
contig022383 CCCACTAACATCCTCCTCCTCTCTCTGGCTGTCTCAGACTTTCTCATTGGTCTGTTGATGATGCCGGCAAAAATCCTACGAGACACAGCTTGTTGGTTTCTTGGTCAGCTCACATGTTTAATATATAATT
TilTARs.A054 CCCACTAACATCCTCCTCCTCTCTCTGGCTGTCTCAGACTTTCTCATTGGTCTGTTGATGATGCCGGCAAAAATCCTACGAGACACAGCTTGTTGGTTTCTTGGTCAGCTCACATGTTTAATATATAATT
   Consensus CCCACTAACATCCTCCTCCTCTCTCTGGCTGTCTCAGACTTTCTCATTGGTCTGTTGATGATGCCGGCAAAAATCCTACGAGACACAGCTTGTTGGTTTCTTGGTCAGCTCACATGTTTAATATATAATT

             1561    1570      1580      1590      1600      1610      1620      1630      1640      1650      1660      1670      1680      1690
contig022383 ATATATGCTTCATTATTACCTCTGCCTCAGTGGGCATTATGGTGCTGATATCAGTCGACCGCTATGTGGCTATTTGTGACCCTCTGCATTACCCCACCAGAATCACTGAAAAAAGAGTGAAACTTTGCGT
TilTARs.A054 ATATATGCTTCATTATTACCTCTGCCTCAGTGGGCATTATGGTGCTGATATCAGTCGACCGCTATGTGGCTATTTGTGACCCTCTGCATTACCCCACCAGAATCACTGAAAAAAGAGTGAAACTTTGCGT
   Consensus ATATATGCTTCATTATTACCTCTGCCTCAGTGGGCATTATGGTGCTGATATCAGTCGACCGCTATGTGGCTATTTGTGACCCTCTGCATTACCCCACCAGAATCACTGAAAAAAGAGTGAAACTTTGCGT

             1691    1700      1710      1720      1730      1740      1750      1760      1770      1780      1790      1800      1810      1820
contig022383 CTGTCTGTGTTGGCTCTGCTCTGTTTTTTACAACATACTATTTATAAAGGACGACCTGCTTCAACGAGAGCAACATACTTCCTGTTATGGAGAATGTGTATTTGTCATAGACTACATTGTAGGAACCACT
TilTARs.A054 CTGTCTGTGTTGGCTCTGCTCTGTTTTTTACAACATACTATTTATAAAGGACGACCTGCTTCAACGAGAGCAACATACTTCCTGTTATGGAGAATGTGTATTTGTCATAGACTACATTGTAGGAACCACT
   Consensus CTGTCTGTGTTGGCTCTGCTCTGTTTTTTACAACATACTATTTATAAAGGACGACCTGCTTCAACGAGAGCAACATACTTCCTGTTATGGAGAATGTGTATTTGTCATAGACTACATTGTAGGAACCACT

             1821    1830      1840      1850      1860      1870      1880      1890      1900      1910      1920      1930      1940      1950
contig022383 GACATTGTTGTGACTTTTATTGCTCCAGTTACTGTCATTGTAATTTTGTATATGAGAGTATTTGTGGTGGCTGTGTCTCAGGCCCGTGCCATGCGCTCTCATGTTACAGCTGTCACACTGCAGCTCTCAG
TilTARs.A054 GACATTGTTGTGACTTTTATTGCTCCAGTTACTGTCATTGTAATTTTGTATATGAGAGTATTTGTGGTGGCTGTGTCTCAGGCCCGTGCCATGCGCTCTCATGTTACAGCTGTCACACTGCAGCTCTCAG
   Consensus GACATTGTTGTGACTTTTATTGCTCCAGTTACTGTCATTGTAATTTTGTATATGAGAGTATTTGTGGTGGCTGTGTCTCAGGCCCGTGCCATGCGCTCTCATGTTACAGCTGTCACACTGCAGCTCTCAG

             1951    1960      1970      1980      1990      2000      2010      2020      2030      2040      2050      2060      2070      2080
contig022383 TGACTCTAACAGCAAAGAAATCAGAGTTAAAAGCTGCCAGGACTCTGGGTGTTCTTGTTCTTGTGTTTCTATTATGTTTCTGCCCATATTACATTGTTTCACTTTTAGGAAACGAGTTGTTCAACAGCTC
TilTARs.A054 TGACTCTAACAGCAAAGAAATCAGAGTTAAAAGCTGCCAGGACTCTGGGTGTTCTTGTTCTTGTGTTTCTATTATGTTTCTGCCCATATTACATTGTTTCACTTTTAGGAAACGAGTTGTTCAACAGCTC
   Consensus TGACTCTAACAGCAAAGAAATCAGAGTTAAAAGCTGCCAGGACTCTGGGTGTTCTTGTTCTTGTGTTTCTATTATGTTTCTGCCCATATTACATTGTTTCACTTTTAGGAAACGAGTTGTTCAACAGCTC

             2081    2090      2100      2110      2120      2130      2140      2150      2160      2170      2180      2190      2200      2210
contig022383 ATCTGCATCTATTGTGATCTACCTGTATTATTTTAACTCCTGTCTAAATCCTTTGATTTATGCTATGTTCTACCCCTGGTTTAGAAAAGCTGTGAAATTAGTTGTCACTCTACAGATACTGCAGCCTGGC
TilTARs.A054 ATCTGCATCTATTGTGATCTACCTGTATTATTTTAACTCCTGTCTAAATCCTTTGATTTATGCTATGTTCTACCCCTGGTTTAGAAAAGCTGTGAAATTAGTTGTCACTCTACAGATACTGCAGCCTGGC
   Consensus ATCTGCATCTATTGTGATCTACCTGTATTATTTTAACTCCTGTCTAAATCCTTTGATTTATGCTATGTTCTACCCCTGGTTTAGAAAAGCTGTGAAATTAGTTGTCACTCTACAGATACTGCAGCCTGGC

             2211    2220      2230      2240      2250      2260      2270      2280      2290      2300      2310      2320      2330      2340
contig022383 TCCTGTGAGGTCAGCATACTGTAAAGAGATCTTTCCAAAAAGATGTTTATGATAAAAACTGAACAAGCAGATGTTAAAACCCTTTCTGTCTCTTTAAGAGAACAAGGAGAATAAGATGTATTGTTCATCT
TilTARs.A054 TCCTGTGAGGTCAGCATACTGTAA
   Consensus TCCTGTGAGGTCAGCATACTGTAA..........................................................................................................
```

```
TilTARs.A055
   Consensus  ..................................................................................................................................

              781    790       800       810       820       830       840       850       860       870       880       890       900       910
              |--------+---------+---------+---------+---------+---------+---------+---------+---------+---------+---------+---------+---------|
 contig022390 AAGAGGGGAGGAGTTGAGAGGATGTGGTGTGAGGATCAGTTATATTACTAATTCTGGTCACAACTCTGCCAGAGCTGCAGCTCCCTGCAGGTCTGTGGAGATGGATACACAGGAAGGAGCAGAGCTCTGC
 TilTARs.A055                                                                                                     ATGGATACACAGGAAGGAGCAGAGCTCTGC
    Consensus ....................................................................................................ATGGATACACAGGAAGGAGCAGAGCTCTGC

              911    920       930       940       950       960       970       980       990       1000      1010      1020      1030      1040
              |--------+---------+---------+---------+---------+---------+---------+---------+---------+---------+---------+---------+---------|
 contig022390 TTTCCACAACTCTTCAACATCTCCTGCAAGAAGCCTACAACTCCTCTGTCTCAAGTTTTGCTCCTTTATATTGTTTTGTCCTCAATGTCTCTGCTAACTGTGACTCTCAACCTGCTCGTCATCATCGCAG
 TilTARs.A055 TTTCCACAACTCTTCAACATCTCCTGCAAGAAGCCTACAACTCCTCTGTCTCAAGTTTTGCTCCTTTATATTGTTTTGTCCTCAATGTCTCTGCTAACTGTGACTCTCAACCTGCTCGTCATCATCGCAG
    Consensus TTTCCACAACTCTTCAACATCTCCTGCAAGAAGCCTACAACTCCTCTGTCTCAAGTTTTGCTCCTTTATATTGTTTTGTCCTCAATGTCTCTGCTAACTGTGACTCTCAACCTGCTCGTCATCATCGCAG

              1041   1050      1060      1070      1080      1090      1100      1110      1120      1130      1140      1150      1160      1170
              |--------+---------+---------+---------+---------+---------+---------+---------+---------+---------+---------+---------+---------|
 contig022390 TCTCCCACTTCAGGCAGAGTTTCATATTGTTTACCTGCTGCTGTGGCTTTACAAGTCTATACCTGATTTGTTACACCTGTGTGAATAATTTCTTTCTGTTGATTTTTTTAAATTATATTTTCCTGTCTTT
 TilTARs.A055 TCTCCCACTTCAGGC-------------------------------------------------------------------------------------------------------------------
    Consensus TCTCCCACTTCAGGC...................................................................................................................

              1171   1180      1190      1200      1210      1220      1230      1240      1250      1260      1270      1280      1290      1300
              |--------+---------+---------+---------+---------+---------+---------+---------+---------+---------+---------+---------+---------|
 contig022390 CTAGGCAGCTCCACACACCCACTAACATCCTGCTCCTCTCTCTGGCTGTCACTGACTTTCTCGTTGGTCTTCTGTTTATGCCTGGAGAAATCCTGCGAAATACAGCATGCTGGTTTCTTGGTCAGCTCAC
 TilTARs.A055 ------------------------------------------------------------------------------------------------GAAATACAGCATGCTGGTTTCTTGGTCAGCTCAC
    Consensus ................................................................................................GAAATACAGCATGCTGGTTTCTTGGTCAGCTCAC

              1301   1310      1320      1330      1340      1350      1360      1370      1380      1390      1400      1410      1420      1430
              |--------+---------+---------+---------+---------+---------+---------+---------+---------+---------+---------+---------+---------|
 contig022390 ATGTTCTCTGTATAATTATGTATCCTACATCGTTGCCTCTGCCTCAGTGGGCAACATGGTGCTGATATCAGTCGACCGTTATGTGGCTATTTGTGACCCTTTGCATTACCCCTCCAGAATCACTGACAAA
 TilTARs.A055 ATGTTCTCTGTATAATTATGTATCCTACATCGTTGCCTCTGCCTCAGTGGGCAACATGGTGCTGATATCAGTCGACCGTTATGTGGCTATTTGTGACCCTTTGCATTACCCCTCCAGAATCACTGACAAA
    Consensus ATGTTCTCTGTATAATTATGTATCCTACATCGTTGCCTCTGCCTCAGTGGGCAACATGGTGCTGATATCAGTCGACCGTTATGTGGCTATTTGTGACCCTTTGCATTACCCCTCCAGAATCACTGACAAA

              1431   1440      1450      1460      1470      1480      1490      1500      1510      1520      1530      1540      1550      1560
              |--------+---------+---------+---------+---------+---------+---------+---------+---------+---------+---------+---------+---------|
 contig022390 AGAGTGAAACTCTGCGTCTGTCTGTGTTGGCTCTGCTCTGTTTTCTACAGCTATGTGATTTTAATAGATGATCTAAGTCAACCAGGCAAGCATAATTCTTGTTATGGAAAATGTATAATTTTCATTGAAT
 TilTARs.A055 AGAGTGAAACTCTGCGTCTGTCTGTGTTGGCTCTGCTCTGTTTTCTACAGCTATGTGATTTTAATAGATGATCTAAGTCAACCAGGCAAGCATAATTCTTGTTATGGAAAATGTATAATTTTCATTGAAT
    Consensus AGAGTGAAACTCTGCGTCTGTCTGTGTTGGCTCTGCTCTGTTTTCTACAGCTATGTGATTTTAATAGATGATCTAAGTCAACCAGGCAAGCATAATTCTTGTTATGGAAAATGTATAATTTTCATTGAAT

              1561   1570      1580      1590      1600      1610      1620      1630      1640      1650      1660      1670      1680      1690
              |--------+---------+---------+---------+---------+---------+---------+---------+---------+---------+---------+---------+---------|
 contig022390 TCATTGCAGGATTTGTTGACCTTGTTTTATCCTTTATAATTCCACTTACTGTCATCGTAGTTCTGTATATGAGAGTATTTGTGGTGGCTGTGTCTCAGGCCCGTGCCATGCGCTCTCATGTTACAGCTGT
 TilTARs.A055 TCATTGCAGGATTTGTTGACCTTGTTTTATCCTTTATAATTCCACTTACTGTCATCGTAGTTCTGTATATGAGAGTATTTGTGGTGGCTGTGTCTCAGGCCCGTGCCATGCGCTCTCATGTTACAGCTGT
    Consensus TCATTGCAGGATTTGTTGACCTTGTTTTATCCTTTATAATTCCACTTACTGTCATCGTAGTTCTGTATATGAGAGTATTTGTGGTGGCTGTGTCTCAGGCCCGTGCCATGCGCTCTCATGTTACAGCTGT

              1691   1700      1710      1720      1730      1740      1750      1760      1770      1780      1790      1800      1810      1820
              |--------+---------+---------+---------+---------+---------+---------+---------+---------+---------+---------+---------+---------|
 contig022390 CACACTGCAGCTCTCAGTGACTCTAACAGCAAAGAAATCAGAGTTAAAAGCTGCCAGGACTCTGGGTGTTCTTGTTCTTGTGTTTCTAATATGTTTCTGCCCATATTACATTGTTTCGCTTTTTGGAGAC
 TilTARs.A055 CACACTGCAGCTCTCAGTGACTCTAACAGCAAAGAAATCAGAGTTAAAAGCTGCCAGGACTCTGGGTGTTCTTGTTCTTGTGTTTCTAATATGTTTCTGCCCATATTACATTGTTTCGCTTTTTGGAGAC
    Consensus CACACTGCAGCTCTCAGTGACTCTAACAGCAAAGAAATCAGAGTTAAAAGCTGCCAGGACTCTGGGTGTTCTTGTTCTTGTGTTTCTAATATGTTTCTGCCCATATTACATTGTTTCGCTTTTTGGAGAC

              1821   1830      1840      1850      1860      1870      1880      1890      1900      1910      1920      1930      1940      1950
              |--------+---------+---------+---------+---------+---------+---------+---------+---------+---------+---------+---------+---------|
 contig022390 GAGTTTCTAAACAGCTCATCTGCATCCATTGTGATCTATCTGTATTATTTTAACTCCTGTGTAAACCCTTTGATTTATGCTATGTTCTACCCCTGGTTTAGAAAAGCTGTGAAACTAATTGTGACTCTAC
 TilTARs.A055 GAGTTTCTAAACAGCTCATCTGCATCCATTGTGATCTATCTGTATTATTTTAACTCCTGTGTAAACCCTTTGATTTATGCTATGTTCTACCCCTGGTTTAGAAAAGCTGTGAAACTAATTGTGACTCTAC
    Consensus GAGTTTCTAAACAGCTCATCTGCATCCATTGTGATCTATCTGTATTATTTTAACTCCTGTGTAAACCCTTTGATTTATGCTATGTTCTACCCCTGGTTTAGAAAAGCTGTGAAACTAATTGTGACTCTAC

              1951   1960      1970      1980      1990      2000      2010      2020      2030      2040      2050      2060      2070      2080
              |--------+---------+---------+---------+---------+---------+---------+---------+---------+---------+---------+---------+---------|
 contig022390 AGATACTGCAGCCTGACTCCTGTGAGGTCAGCATACTGTAGAAGAAAGAAATCTCTCTTTAATGTACCAAGTGGTTTACCAAATTACTTTCTCTATCAATAATGTACTACATTAATTTTAAGACATTTAA
 TilTARs.A055 AGATACTGCAGCCTGACTCCTGTGAGGTCAGCATACTGTAG
    Consensus AGATACTGCAGCCTGACTCCTGTGAGGTCAGCATACTGTAG.........................................................................................

              2081   2090      2100      2110      2120      2130      2140      2150      2160      2170      2180      2190      2200      2210
              |--------+---------+---------+---------+---------+---------+---------+---------+---------+---------+---------+---------+---------|
 contig022390 CAAGTAATGTGTAATAAATTACATTTCTTGAGTAATAACCCCAGCACTGGTTGTCAAATAGTGCTTAGTACTCTATGGTTTCAATTATACAGGCCTAGAAAAGGGTTGACAACCCACTGGCAACCTCCTG
 TilTARs.A055
    Consensus ..................................................................................................................................
```

```
              3901  3910      3920      3930      3940      3950      3960      3970      3980      3990      4000      4010      4020      4030
contig022390  CTCTGCAGGTCTGTGGAGATGGATACACAGGATGGATTAGAGCTCTGCTTTCCACAACTCTTCAACATCTCCTGCAAGAAGCCTACAACTCCTCTGTCTCAAGTTTTGCTTCCTTATATTGTTGTGTTCT
TilTARs.A056                    ATGGATACACAGGATGGATTAGAGCTCTGCTTTCCACAACTCTTCAACATCTCCTGCAAGAAGCCTACAACTCCTCTGTCTCAAGTTTTGCTTCCTTATATTGTTGTGTTCT
Consensus     ..................ATGGATACACAGGATGGATTAGAGCTCTGCTTTCCACAACTCTTCAACATCTCCTGCAAGAAGCCTACAACTCCTCTGTCTCAAGTTTTGCTTCCTTATATTGTTGTGTTCT

              4031  4040      4050      4060      4070      4080      4090      4100      4110      4120      4130      4140      4150      4160
contig022390  CAATGTCTCTGCTAACTGTGACTCTCAACCTGCTCGTCATCATCGCAGTCTCCCACTTCAGGCAGAGTTTCACTTTTTTAACCGTTACTGTAGTTTTACAAGTCAGAGTCGGTTTGAGTAAATACATGAT
TilTARs.A056  CAATGTCTCTGCTAACTGTGACTCTCAACCTGCTCGTCATCATCGCAGTCTCCCACTTCAG---------------------------------------------------------------------
Consensus     CAATGTCTCTGCTAACTGTGACTCTCAACCTGCTCGTCATCATCGCAGTCTCCCACTTCAG.....................................................................

              4161  4170      4180      4190      4200      4210      4220      4230      4240      4250      4260      4270      4280      4290
contig022390  TTTACTCATATATGGACTAGACTTACATTTTAACTGGTAGACTATTTTTACATAATGATCTGTATATATTAGATGTTAATATAACATTAGATGACAGACTTTATGAGTTTAGATATTCATTATATGAGGT
TilTARs.A056  ----------------------------------------------------------------------------------------------------------------------------------
Consensus     ..................................................................................................................................

              4291  4300      4310      4320      4330      4340      4350      4360      4370      4380      4390      4400      4410      4420
contig022390  ACTTCATACCATATTCCTTCTCCTACCAGTAATCTTTACATTCAGTCTCCTACAACTTATCTTCTGTCTACACTTGAACATGCTAAACCTATAATTGAATTATTTTCAGATTTTTCAAATGATTTTGTTT
TilTARs.A056  ----------------------------------------------------------------------------------------------------------------------------------
Consensus     ..................................................................................................................................

              4421  4430      4440      4450      4460      4470      4480      4490      4500      4510      4520      4530      4540      4550
contig022390  CTCTCTCTTCTTTCAGACAGCTCCACACACCCACTAACATCCTCCTCCTCTCTCTGGCTGTCTCAGACTTTCTCATTGGTTTGTTGTTGATGCCTGCAAATATCATCCGAAAGAAAGTTTGTTGGTTTCT
TilTARs.A056  ----------------ACAGCTCCACACACCCACTAACATCCTCCTCCTCTCTCTGGCTGTCTCAGACTTTCTCATTGGTTTGTTGTTGATGCCTGCAAATATCATCCGAAAGAAAGTTTGTTGGTTTCT
Consensus     ................ACAGCTCCACACACCCACTAACATCCTCCTCCTCTCTCTGGCTGTCTCAGACTTTCTCATTGGTTTGTTGTTGATGCCTGCAAATATCATCCGAAAGAAAGTTTGTTGGTTTCT

              4551  4560      4570      4580      4590      4600      4610      4620      4630      4640      4650      4660      4670      4680
contig022390  TGGTCAGCTCACATGTTTAGTCTATACTTATGTATCCTTCATTATTACCTCTGCCTCAGTGGGCATCATGGTGCTGATATCAGTTGACAGGTATGTGGCTATTTGTGACCCTCTGCATTACCCCACCAGA
TilTARs.A056  TGGTCAGCTCACATGTTTAGTCTATACTTATGTATCCTTCATTATTACCTCTGCCTCAGTGGGCATCATGGTGCTGATATCAGTTGACAGGTATGTGGCTATTTGTGACCCTCTGCATTACCCCACCAGA
Consensus     TGGTCAGCTCACATGTTTAGTCTATACTTATGTATCCTTCATTATTACCTCTGCCTCAGTGGGCATCATGGTGCTGATATCAGTTGACAGGTATGTGGCTATTTGTGACCCTCTGCATTACCCCACCAGA

              4681  4690      4700      4710      4720      4730      4740      4750      4760      4770      4780      4790      4800      4810
contig022390  ATAACTCATGGAAGAGTGAAACTCTGTGTCTGTCTGTGTTGGCTCTGCTCTGTTATCTACAACATTTTGCTCATAAAGGATGAACTGGTTAAACCAGGGCAACATATTTCCTGTTATGGAGAATGTAAAA
TilTARs.A056  ATAACTCATGGAAGAGTGAAACTCTGTGTCTGTCTGTGTTGGCTCTGCTCTGTTATCTACAACATTTTGCTCATAAAGGATGAACTGGTTAAACCAGGGCAACATATTTCCTGTTATGGAGAATGTAAAA
Consensus     ATAACTCATGGAAGAGTGAAACTCTGTGTCTGTCTGTGTTGGCTCTGCTCTGTTATCTACAACATTTTGCTCATAAAGGATGAACTGGTTAAACCAGGGCAACATATTTCCTGTTATGGAGAATGTAAAA

              4811  4820      4830      4840      4850      4860      4870      4880      4890      4900      4910      4920      4930      4940
contig022390  TTCTCGTAGACTACATTGCAGGAACCACTGACATTGTTTTAACCTTTATTATTCCTGTTACTGTCATCATAGTTCTGTACATGAGAGTATTTGTGGTGGCTGTGTCTCAGGCCCGTGCCATGCGCTCTCA
TilTARs.A056  TTCTCGTAGACTACATTGCAGGAACCACTGACATTGTTTTAACCTTTATTATTCCTGTTACTGTCATCATAGTTCTGTACATGAGAGTATTTGTGGTGGCTGTGTCTCAGGCCCGTGCCATGCGCTCTCA
Consensus     TTCTCGTAGACTACATTGCAGGAACCACTGACATTGTTTTAACCTTTATTATTCCTGTTACTGTCATCATAGTTCTGTACATGAGAGTATTTGTGGTGGCTGTGTCTCAGGCCCGTGCCATGCGCTCTCA

              4941  4950      4960      4970      4980      4990      5000      5010      5020      5030      5040      5050      5060      5070
contig022390  TGTTACAGCTGTCACAATGCAGCTCTCAGTGACTCTAACAACAAAGAAATCAGAGTTAAAAGCAGCCAGGACTCTGGGTGTTCTTGTTCTTGTGTTTCTAATATGTTTCTGTCCTCATTATTGTATTTTT
TilTARs.A056  TGTTACAGCTGTCACAATGCAGCTCTCAGTGACTCTAACAACAAAGAAATCAGAGTTAAAAGCAGCCAGGACTCTGGGTGTTCTTGTTCTTGTGTTTCTAATATGTTTCTGTCCTCATTATTGTATTTTT
Consensus     TGTTACAGCTGTCACAATGCAGCTCTCAGTGACTCTAACAACAAAGAAATCAGAGTTAAAAGCAGCCAGGACTCTGGGTGTTCTTGTTCTTGTGTTTCTAATATGTTTCTGTCCTCATTATTGTATTTTT

              5071  5080      5090      5100      5110      5120      5130      5140      5150      5160      5170      5180      5190      5200
contig022390  GTTTTTGGAGGAAAGTTGCTCAGTAGCTCGTCTGCAACCATTGTGATCTATCTGTATTATTTTAACTCCTGTGTAAACCCTTTGATTTATGCTATGTTCTACCCCTGGTTTAGAAAAGCTGTGAAACTAA
TilTARs.A056  GTTTTTGGAGGAAAGTTGCTCAGTAGCTCGTCTGCAACCATTGTGATCTATCTGTATTATTTTAACTCCTGTGTAAACCCTTTGATTTATGCTATGTTCTACCCCTGGTTTAGAAAAGCTGTGAAACTAA
Consensus     GTTTTTGGAGGAAAGTTGCTCAGTAGCTCGTCTGCAACCATTGTGATCTATCTGTATTATTTTAACTCCTGTGTAAACCCTTTGATTTATGCTATGTTCTACCCCTGGTTTAGAAAAGCTGTGAAACTAA

              5201  5210      5220      5230      5240      5250      5260      5270      5280      5290      5300      5310      5320      5330
contig022390  CTGTCACACTACAGATACTGCAGCCTGGCTCCTGTGAGGTCAGCATACTGTAGAAGAAAGAAATCTTTCCTTAATGTAAAGCAAAACTGAACAAGCAGCAGTTAAAATCTTTTCTGTCTCTTTAGGAGAA
TilTARs.A056  CTGTCACACTACAGATACTGCAGCCTGGCTCCTGTGAGGTCAGCATACTGTAG
Consensus     CTGTCACACTACAGATACTGCAGCCTGGCTCCTGTGAGGTCAGCATACTGTAG.............................................................................
```

```
             2081 2090      2100      2110      2120      2130      2140      2150      2160      2170      2180      2190      2200      2210
             |--------+---------+---------+---------+---------+---------+---------+---------+---------+---------+---------+---------+---------|
contig045088 ATGATGGAGGGGGTTGAATTCTGCTTTCCACATCTGCTCAACTCCTCTTGCAGAAAGGCTCTGCGTCCTGTCTCAGTTTCCATGCTCATTTACATGATAATATCTTCTATCTCTGTGCTCACTGCAACTC
TilTARs.A057    ATGGAGGGGGTTGAATTCTGCTTTCCACATCTGCTCAACTCCTCTTGCAGAAAGGCTCTGCGTCCTGTCTCAGTTTCCATGCTCATTTACATGATAATATCTTCTATCTCTGTGCTCACTGCAACTC
   Consensus ...ATGGAGGGGGTTGAATTCTGCTTTCCACATCTGCTCAACTCCTCTTGCAGAAAGGCTCTGCGTCCTGTCTCAGTTTCCATGCTCATTTACATGATAATATCTTCTATCTCTGTGCTCACTGCAACTC

             2211 2220      2230      2240      2250      2260      2270      2280      2290      2300      2310      2320      2330      2340
             |--------+---------+---------+---------+---------+---------+---------+---------+---------+---------+---------+---------+---------|
contig045088 TTAACCTGCTGGTCATCGTCTCCATCTCCCACTTCAAGTAGCCACATATTTTGTAAAGTTAAAAGATTAATAGCTGTTTTTAAGGAAAAACTTCAAAACAAAACAACTATGAATTTATGCTGCAAATAAG
TilTARs.A057 TTAACCTGCTGGTCATCGTCTCCATCTCCCACTTCAAG--------------------------------------------------------------------------------------------
   Consensus TTAACCTGCTGGTCATCGTCTCCATCTCCCACTTCAAG............................................................................................

             2341 2350      2360      2370      2380      2390      2400      2410      2420      2430      2440      2450      2460      2470
             |--------+---------+---------+---------+---------+---------+---------+---------+---------+---------+---------+---------+---------|
contig045088 TGCTTTCTTTCTTATTTCTCTAGGCAGCTCCATACTCCCACCAACTTCCTCCTCCTCTCTCTGGCTGTCTCCGATTTCTTTGTGGGTCTCTATTTGCTCTTTTACATAATGTTTATAGACGGCTGCTGGT
TilTARs.A057 ------------------------CAGCTCCATACTCCCACCAACTTCCTCCTCCTCTCTCTGGCTGTCTCCGATTTCTTTGTGGGTCTCTATTTGCTCTTTTACATAATGTTTATAGACGGCTGCTGGT
   Consensus ........................CAGCTCCATACTCCCACCAACTTCCTCCTCCTCTCTCTGGCTGTCTCCGATTTCTTTGTGGGTCTCTATTTGCTCTTTTACATAATGTTTATAGACGGCTGCTGGT

             2471 2480      2490      2500      2510      2520      2530      2540      2550      2560      2570      2580      2590      2600
             |--------+---------+---------+---------+---------+---------+---------+---------+---------+---------+---------+---------+---------|
contig045088 ATTTTGGTGAATTCATGTGTATTCTGTATTATGTTATTGGCACAATTAACACCTCTTCCTCAATAGGAACCATGGTACTGATATCAGTTGACCGTTATGTGGCCATTTGTGATCCTCTGCATTATCCCAT
TilTARs.A057 ATTTTGGTGAATTCATGTGTATTCTGTATTATGTTATTGGCACAATTAACACCTCTTCCTCAATAGGAACCATGGTACTGATATCAGTTGACCGTTATGTGGCCATTTGTGATCCTCTGCATTATCCCAT
   Consensus ATTTTGGTGAATTCATGTGTATTCTGTATTATGTTATTGGCACAATTAACACCTCTTCCTCAATAGGAACCATGGTACTGATATCAGTTGACCGTTATGTGGCCATTTGTGATCCTCTGCATTATCCCAT

             2601 2610      2620      2630      2640      2650      2660      2670      2680      2690      2700      2710      2720      2730
             |--------+---------+---------+---------+---------+---------+---------+---------+---------+---------+---------+---------+---------|
contig045088 CAAAGTCACTGCAAAAAGAGTTCAGACCTGTGTTTCACTGTGTTGGAGCTTTTCTGCCCTTGCTGGTAGTTACCTCTTAAAGGACAACTTGAAACAGCAAAGCAGGTTTAATTCTTGTGTGGGAGAGTGT
TilTARs.A057 CAAAGTCACTGCAAAAAGAGTTCAGACCTGTGTTTCACTGTGTTGGAGCTTTTCTGCCCTTGCTGGTAGTTACCTCTTAAAGGACAACTTGAAACAGCAAAGCAGGTTTAATTCTTGTGTGGGAGAGTGT
   Consensus CAAAGTCACTGCAAAAAGAGTTCAGACCTGTGTTTCACTGTGTTGGAGCTTTTCTGCCCTTGCTGGTAGTTACCTCTTAAAGGACAACTTGAAACAGCAAAGCAGGTTTAATTCTTGTGTGGGAGAGTGT

             2731 2740      2750      2760      2770      2780      2790      2800      2810      2820      2830      2840      2850      2860
             |--------+---------+---------+---------+---------+---------+---------+---------+---------+---------+---------+---------+---------|
contig045088 GTTGTCCATATTAACTTTATTGAATATGTTGCAGATCTTGCTTTGAACTTCCTACTTCCTATTACTGTCATTATAGTTCTGTATTTGAGAATATTTGTAGTGGTTGTGTCTCAGGTTCGGGCCATGCGGT
TilTARs.A057 GTTGTCCATATTAACTTTATTGAATATGTTGCAGATCTTGCTTTGAACTTCCTACTTCCTATTACTGTCATTATAGTTCTGTATTTGAGAATATTTGTAGTGGTTGTGTCTCAGGTTCGGGCCATGCGGT
   Consensus GTTGTCCATATTAACTTTATTGAATATGTTGCAGATCTTGCTTTGAACTTCCTACTTCCTATTACTGTCATTATAGTTCTGTATTTGAGAATATTTGTAGTGGTTGTGTCTCAGGTTCGGGCCATGCGGT

             2861 2870      2880      2890      2900      2910      2920      2930      2940      2950      2960      2970      2980      2990
             |--------+---------+---------+---------+---------+---------+---------+---------+---------+---------+---------+---------+---------|
contig045088 CTCATACTGCAGGTGTCACATACCAGTGTTCAAGGAAGGGAAATCCAAAGAAATCAGAGATGAAAGCAGCCAGGACTCTTGGTATTGTTGTAATTGCATTCTTGTCATGCACCTTACCATTTTATTGTGT
TilTARs.A057 CTCATACTGCAGGTGTCACATACCAGTGTTCAAGGAAGGGAAATCCAAAGAAATCAGAGATGAAAGCAGCCAGGACTCTTGGTATTGTTGTAATTGCATTCTTGTCATGCACCTTACCATTTTATTGTGT
   Consensus CTCATACTGCAGGTGTCACATACCAGTGTTCAAGGAAGGGAAATCCAAAGAAATCAGAGATGAAAGCAGCCAGGACTCTTGGTATTGTTGTAATTGCATTCTTGTCATGCACCTTACCATTTTATTGTGT

             2991 3000      3010      3020      3030      3040      3050      3060      3070      3080      3090      3100      3110      3120
             |--------+---------+---------+---------+---------+---------+---------+---------+---------+---------+---------+---------+---------|
contig045088 CACACTCACAGGCCAGAATGCCTTCCTCAACGGATCATCTTCTGCATTTGTTCTATGTCTTTTCTATTTTAATTCGTGCCTCAACCCTATCGTCTATGCCTTATTCTACCCCTGGTTTAGAAAATCTATT
TilTARs.A057 CACACTCACAGGCCAGAATGCCTTCCTCAACGGATCATCTTCTGCATTTGTTCTATGTCTTTTCTATTTTAATTCGTGCCTCAACCCTATCGTCTATGCCTTATTCTACCCCTGGTTTAGAAAATCTATT
   Consensus CACACTCACAGGCCAGAATGCCTTCCTCAACGGATCATCTTCTGCATTTGTTCTATGTCTTTTCTATTTTAATTCGTGCCTCAACCCTATCGTCTATGCCTTATTCTACCCCTGGTTTAGAAAATCTATT

             3121 3130      3140      3150      3160      3170      3180      3190      3200      3210      3220      3230      3240      3250
             |--------+---------+---------+---------+---------+---------+---------+---------+---------+---------+---------+---------+---------|
contig045088 AAGTTTATTGTTACATTTCAAATACTGAAGTCTGGCTCCAGGAATGCCAACATAGTCAAAGTGACAGAATAGTGACTAAAATGAGGTCTCTACTGTTTTAGCAGCAGTTTACGAGTAAGTCAGCCCAAAC
TilTARs.A057 AAGTTTATTGTTACATTTCAAATACTGAAGTCTGGCTCCAGGAATGCCAACATAGTCAAAGTGACAGAATAG
   Consensus AAGTTTATTGTTACATTTCAAATACTGAAGTCTGGCTCCAGGAATGCCAACATAGTCAAAGTGACAGAATAG..........................................................
```

```
            1561 1570          1580      1590      1600      1610      1620      1630      1640      1650      1660      1670      1680      1690
            |--------+---------+---------+---------+---------+---------+---------+---------+---------+---------+---------+---------+---------|
contig056134 AGGGAAGGTAAAGGTGTGGGATAAGGTTGGTGTGTGCGTGTGTTGGTAACTATGGGGGTGGGGGGATACTAACTCACCTAAAACATGTTGCACAAACACTGGGTTCAGCAGTTTCACCTACCTGCAGTGA
TilTARs.A058                                                                                                                                  A
   Consensus .................................................................................................................................A

            1691 1700          1710      1720      1730      1740      1750      1760      1770      1780      1790      1800      1810      1820
            |--------+---------+---------+---------+---------+---------+---------+---------+---------+---------+---------+---------+---------|
contig056134 TGGAGGAAACTGAACTCTGCTTTCCAAAACTTCTTAACACCTCCTGCATGAGGCTGAGGCGTCCCCACTTTGAGATCATGCTGACTTATATTCTGCTGTCTTCAATCGCTCTGTTAACTGTGACTCTTAA
TilTARs.A058 TGGAGGAAACTGAACTCTGCTTTCCAAAACTTCTTAACACCTCCTGCATGAGGCTGAGGCGTCCCCACTTTGAGATCATGCTGACTTATATTCTGCTGTCTTCAATCGCTCTGTTAACTGTGACTCTTAA
   Consensus TGGAGGAAACTGAACTCTGCTTTCCAAAACTTCTTAACACCTCCTGCATGAGGCTGAGGCGTCCCCACTTTGAGATCATGCTGACTTATATTCTGCTGTCTTCAATCGCTCTGTTAACTGTGACTCTTAA

            1821 1830          1840      1850      1860      1870      1880      1890      1900      1910      1920      1930      1940      1950
            |--------+---------+---------+---------+---------+---------+---------+---------+---------+---------+---------+---------+---------|
contig056134 TCTGTTTGTCATCATCTCCATCTCACACTTCAGGTAGACACACATTACACTGACTTGATGTTAAAATTGTAAAGATTTTTCCATGTGTTGTTTTTTTTAATGTTATTTCAGCTTTTCCTCTACAATGAAT
TilTARs.A058 TCTGTTTGTCATCATCTCCATCTCACACTTCAGG------------------------------------------------------------------------------------------------
   Consensus TCTGTTTGTCATCATCTCCATCTCACACTTCAGG................................................................................................

            1951 1960          1970      1980      1990      2000      2010      2020      2030      2040      2050      2060      2070      2080
            |--------+---------+---------+---------+---------+---------+---------+---------+---------+---------+---------+---------+---------|
contig056134 GATAACTGAAACATTACACTAGTAAGACGTATTACCATTCTTCCTCTTCAGGCAGCTCCACACCCGCAACAACGTCCTCCTTCTGTCACTCTCCGCAGCTGATTTTGGTGTAGGTCTTGTCATGTTCTTC
TilTARs.A058 ----------------------------------------------------CAGCTCCACACCCGCAACAACGTCCTCCTTCTGTCACTCTCCGCAGCTGATTTTGGTGTAGGTCTTGTCATGTTCTTC
   Consensus ....................................................CAGCTCCACACCCGCAACAACGTCCTCCTTCTGTCACTCTCCGCAGCTGATTTTGGTGTAGGTCTTGTCATGTTCTTC

            2081 2090          2100      2110      2120      2130      2140      2150      2160      2170      2180      2190      2200      2210
            |--------+---------+---------+---------+---------+---------+---------+---------+---------+---------+---------+---------+---------|
contig056134 CAAATTGTGCTCATAGATGGCTGCTGGTTCCTCGGTGACATCATGTGCACTCTGTATCAATACCTAGCATATGTCATCACCTCGGCCTCAATAGGAACCATGGTGATCATATCTGTTGATCGATATTTGG
TilTARs.A058 CAAATTGTGCTCATAGATGGCTGCTGGTTCCTCGGTGACATCATGTGCACTCTGTATCAATACCTAGCATATGTCATCACCTCGGCCTCAATAGGAACCATGGTGATCATATCTGTTGATCGATATTTGG
   Consensus CAAATTGTGCTCATAGATGGCTGCTGGTTCCTCGGTGACATCATGTGCACTCTGTATCAATACCTAGCATATGTCATCACCTCGGCCTCAATAGGAACCATGGTGATCATATCTGTTGATCGATATTTGG

            2211 2220          2230      2240      2250      2260      2270      2280      2290      2300      2310      2320      2330      2340
            |--------+---------+---------+---------+---------+---------+---------+---------+---------+---------+---------+---------+---------|
contig056134 CTATTTGTTACCCTCTACATTACTCCACCAAGATCACACAACAAAGAGTTAAAATTGTTGTCTGTTTGTGTTGGATCTGTTCTGTCATCTTTCAAAGTCTGATTCTGATGGATAACCTGAAGCAACCAGG
TilTARs.A058 CTATTTGTTACCCTCTACATTACTCCACCAAGATCACACAACAAAGAGTTAAAATTGTTGTCTGTTTGTGTTGGATCTGTTCTGTCATCTTTCAAAGTCTGATTCTGATGGATAACCTGAAGCAACCAGG
   Consensus CTATTTGTTACCCTCTACATTACTCCACCAAGATCACACAACAAAGAGTTAAAATTGTTGTCTGTTTGTGTTGGATCTGTTCTGTCATCTTTCAAAGTCTGATTCTGATGGATAACCTGAAGCAACCAGG

            2341 2350          2360      2370      2380      2390      2400      2410      2420      2430      2440      2450      2460      2470
            |--------+---------+---------+---------+---------+---------+---------+---------+---------+---------+---------+---------+---------|
contig056134 CAGGTATAACTCTTGCATTGGAGAGTGTGTCTTTGTCATTAATTACATCGCAGGACTTGTTGATGTTACTTTTTCCTTTATTGTTCCCATTACTGTAATTGTAGTTTTGTATCTGAGAGTGTTTGTGGTG
TilTARs.A058 CAGGTATAACTCTTGCATTGGAGAGTGTGTCTTTGTCATTAATTACATCGCAGGACTTGTTGATGTTACTTTTTCCTTTATTGTTCCCATTACTGTAATTGTAGTTTTGTATCTGAGAGTGTTTGTGGTG
   Consensus CAGGTATAACTCTTGCATTGGAGAGTGTGTCTTTGTCATTAATTACATCGCAGGACTTGTTGATGTTACTTTTTCCTTTATTGTTCCCATTACTGTAATTGTAGTTTTGTATCTGAGAGTGTTTGTGGTG

            2471 2480          2490      2500      2510      2520      2530      2540      2550      2560      2570      2580      2590      2600
            |--------+---------+---------+---------+---------+---------+---------+---------+---------+---------+---------+---------+---------|
contig056134 GCTGTGTCTCAGGCTCATGCCATGAGGTCTCAGCTTGCAGTCACTCACCAGCAATCAGTTACAGTTACTGCAAAGAAATCGGAGCTGAAAGCAGCCTGGACTCTTGGTATTGTTGTCGTTGTGTTTCTCA
TilTARs.A058 GCTGTGTCTCAGGCTCATGCCATGAGGTCTCAGCTTGCAGTCACTCACCAGCAATCAGTTACAGTTACTGCAAAGAAATCGGAGCTGAAAGCAGCCTGGACTCTTGGTATTGTTGTCGTTGTGTTTCTCA
   Consensus GCTGTGTCTCAGGCTCATGCCATGAGGTCTCAGCTTGCAGTCACTCACCAGCAATCAGTTACAGTTACTGCAAAGAAATCGGAGCTGAAAGCAGCCTGGACTCTTGGTATTGTTGTCGTTGTGTTTCTCA

            2601 2610          2620      2630      2640      2650      2660      2670      2680      2690      2700      2710      2720      2730
            |--------+---------+---------+---------+---------+---------+---------+---------+---------+---------+---------+---------+---------|
contig056134 TATGTATGTGTCCATATTACTGTGTGGCTCTCACAGTCAAAGACAATGCACTGAGTGCTTCTTCTGCTGCTTTTGTGATATATTTATTCTTCTTTAACTCGTGTCTGAACCCCATCTTATATGTCTTTTT
TilTARs.A058 TATGTATGTGTCCATATTACTGTGTGGCTCTCACAGTCAAAGACAATGCACTGAGTGCTTCTTCTGCTGCTTTTGTGATATATTTATTCTTCTTTAACTCGTGTCTGAACCCCATCTTATATGTCTTTTT
   Consensus TATGTATGTGTCCATATTACTGTGTGGCTCTCACAGTCAAAGACAATGCACTGAGTGCTTCTTCTGCTGCTTTTGTGATATATTTATTCTTCTTTAACTCGTGTCTGAACCCCATCTTATATGTCTTTTT

            2731 2740          2750      2760      2770      2780      2790      2800      2810      2820      2830      2840      2850      2860
            |--------+---------+---------+---------+---------+---------+---------+---------+---------+---------+---------+---------+---------|
contig056134 TTACTCGTGGTTCAGAAAATCCATAAAAGTCATTGTTACTCTTCAGATACTGCAGCCTGACTCTTCTAAGGCCAACATGCTTTAGAACACAATTTCTCAAAGTTTTGACACATGTATTTAAGGAGCCAGA
TilTARs.A058 TTACTCGTGGTTCAGAAAATCCATAAAAGTCATTGTTACTCTTCAGATACTGCAGCCTGACTCTTCTAAGGCCAACATGCTTTAG
   Consensus TTACTCGTGGTTCAGAAAATCCATAAAAGTCATTGTTACTCTTCAGATACTGCAGCCTGACTCTTCTAAGGCCAACATGCTTTAG.............................................
```

```
             9231 9240      9250      9260      9270      9280      9290      9300      9310      9320      9330      9340      9350      9360
             |--------+---------+---------+---------+---------+---------+---------+---------+---------+---------+---------+---------+---------|
contig022320 CAGCTGTCTGATGATGGAGATTCAGAAAGCAGCTGAGCTTTGTTTTCCACAACTCCTCAACAGTTCCTGCAGGAAGCCGACACTTCATTGGTCCAAAGCTGTGCTCCTGAACATTGTGCTGTTCTGCATC
TilTARs.A059              ATGGAGATTCAGAAAGCAGCTGAGCTTTGTTTTCCACAACTCCTCAACAGTTCCTGCAGGAAGCCGACACTTCATTGGTCCAAAGCTGTGCTCCTGAACATTGTGCTGTTCTGCATC
   Consensus .............ATGGAGATTCAGAAAGCAGCTGAGCTTTGTTTTCCACAACTCCTCAACAGTTCCTGCAGGAAGCCGACACTTCATTGGTCCAAAGCTGTGCTCCTGAACATTGTGCTGTTCTGCATC

             9361 9370      9380      9390      9400      9410      9420      9430      9440      9450      9460      9470      9480      9490
             |--------+---------+---------+---------+---------+---------+---------+---------+---------+---------+---------+---------+---------|
contig022320 TCTGTGATCACTGCTGCTCTAAACCTTCTGGTCATCATCTCAGTCTCCCACTTCAGGCAGAGATACATTTCTCAGCTGAAACTATACTTTTAAATTTTAGAAACTTAAGCTTCAGAAAATTGTGATTTAT
TilTARs.A059 TCTGTGATCACTGCTGCTCTAAACCTTCTGGTCATCATCTCAGTCTCCCACTTCAGG-------------------------------------------------------------------------
   Consensus TCTGTGATCACTGCTGCTCTAAACCTTCTGGTCATCATCTCAGTCTCCCACTTCAGG.........................................................................

             9491 9500      9510      9520      9530      9540      9550      9560      9570      9580      9590      9600      9610      9620
             |--------+---------+---------+---------+---------+---------+---------+---------+---------+---------+---------+---------+---------|
contig022320 GGCATTTGATTTTATGATTAAGATTGAGATGATTGAAGACTCAATCTTACTAAATACTTTAACTAATGAAAATCTCATTTAAGCCATTTAAAAAAAATATCAATGTCCATTATATTCTTTTCTGTTTCCC
TilTARs.A059 ----------------------------------------------------------------------------------------------------------------------------------
   Consensus ..................................................................................................................................

             9621 9630      9640      9650      9660      9670      9680      9690      9700      9710      9720      9730      9740      9750
             |--------+---------+---------+---------+---------+---------+---------+---------+---------+---------+---------+---------+---------|
contig022320 TGCAGGAAGCTTCACACACCCAGTAACATCCTGCTCGTCTCTCTGGCTGTCTCAGACTTTCTTGTGGGTCTTCTGCTGATGCCATTAGAGATCTTTAGAAACACAGCCTGCTGGGTACTTGGTGATGTAA
TilTARs.A059 ------AAGCTTCACACACCCAGTAACATCCTGCTCGTCTCTCTGGCTGTCTCAGACTTTCTTGTGGGTCTTCTGCTGATGCCATTAGAGATCTTTAGAAACACAGCCTGCTGGGTACTTGGTGATGTAA
   Consensus ......AAGCTTCACACACCCAGTAACATCCTGCTCGTCTCTCTGGCTGTCTCAGACTTTCTTGTGGGTCTTCTGCTGATGCCATTAGAGATCTTTAGAAACACAGCCTGCTGGGTACTTGGTGATGTAA

             9751 9760      9770      9780      9790      9800      9810      9820      9830      9840      9850      9860      9870      9880
             |--------+---------+---------+---------+---------+---------+---------+---------+---------+---------+---------+---------+---------|
contig022320 TGTGTTCTGTTTATTGGTACCTGACCAGCAACATTACCTGTGCTTCAATAGGGAACATAGTTCTAATATCAGTTGACCGTTATGTGGCGATTTGTGATCCTCTGCATTACCCCACCAGAATTACTGTGGT
TilTARs.A059 TGTGTTCTGTTTATTGGTACCTGACCAGCAACATTACCTGTGCTTCAATAGGGAACATAGTTCTAATATCAGTTGACCGTTATGTGGCGATTTGTGATCCTCTGCATTACCCCACCAGAATTACTGTGGT
   Consensus TGTGTTCTGTTTATTGGTACCTGACCAGCAACATTACCTGTGCTTCAATAGGGAACATAGTTCTAATATCAGTTGACCGTTATGTGGCGATTTGTGATCCTCTGCATTACCCCACCAGAATTACTGTGGT

             9881 9890      9900      9910      9920      9930      9940      9950      9960      9970      9980      9990     10000     10010
             |--------+---------+---------+---------+---------+---------+---------+---------+---------+---------+---------+---------+---------|
contig022320 CAGAGTCAAGCTCAGTGTGTATTTGTGTTGGTTTTTTTCTATTTTCTACTGGAGTCTTTATATGAAAGATATCCTAGTTGAACCAGGCAGGTATAATTCGTGCTATGGAGAGTGTGTGCTTATTATTAAT
TilTARs.A059 CAGAGTCAAGCTCAGTGTGTATTTGTGTTGGTTTTTTTCTATTTTCTACTGGAGTCTTTATATGAAAGATATCCTAGTTGAACCAGGCAGGTATAATTCGTGCTATGGAGAGTGTGTGCTTATTATTAAT
   Consensus CAGAGTCAAGCTCAGTGTGTATTTGTGTTGGTTTTTTTCTATTTTCTACTGGAGTCTTTATATGAAAGATATCCTAGTTGAACCAGGCAGGTATAATTCGTGCTATGGAGAGTGTGTGCTTATTATTAAT

             1001110020     10030     10040     10050     10060     10070     10080     10090     10100     10110     10120     10130     10140
             |--------+---------+---------+---------+---------+---------+---------+---------+---------+---------+---------+---------+---------|
contig022320 GACATTGCAGGGATTGTTGACCTTGCTTTGTCCTTTATAGTTCCAGTTACTGTTATTATAGTTTTGTATATGAGAGTATTTGTGGTGGCTGTGTCTCAGGCTCGTGCCATGCGCTCTCATGTTACAGCTG
TilTARs.A059 GACATTGCAGGGATTGTTGACCTTGCTTTGTCCTTTATAGTTCCAGTTACTGTTATTATAGTTTTGTATATGAGAGTATTTGTGGTGGCTGTGTCTCAGGCTCGTGCCATGCGCTCTCATGTTACAGCTG
   Consensus GACATTGCAGGGATTGTTGACCTTGCTTTGTCCTTTATAGTTCCAGTTACTGTTATTATAGTTTTGTATATGAGAGTATTTGTGGTGGCTGTGTCTCAGGCTCGTGCCATGCGCTCTCATGTTACAGCTG

             1014110150     10160     10170     10180     10190     10200     10210     10220     10230     10240     10250     10260     10270
             |--------+---------+---------+---------+---------+---------+---------+---------+---------+---------+---------+---------+---------|
contig022320 TCACACTTCAGTGTTCACTGAATCAAGCAAACAAATCTGAGCTGAAAGCAGCCAGGACTCTTGGGGTTCTTGTAGTTGTATTTTTGGCATGCTACTGCCCATTTTACTGCTACTCTCTTGTTGATAAAAA
TilTARs.A059 TCACACTTCAGTGTTCACTGAATCAAGCAAACAAATCTGAGCTGAAAGCAGCCAGGACTCTTGGGGTTCTTGTAGTTGTATTTTTGGCATGCTACTGCCCATTTTACTGCTACTCTCTTGTTGATAAAAA
   Consensus TCACACTTCAGTGTTCACTGAATCAAGCAAACAAATCTGAGCTGAAAGCAGCCAGGACTCTTGGGGTTCTTGTAGTTGTATTTTTGGCATGCTACTGCCCATTTTACTGCTACTCTCTTGTTGATAAAAA

             1027110280     10290     10300     10310     10320     10330     10340     10350     10360     10370     10380     10390     10400
             |--------+---------+---------+---------+---------+---------+---------+---------+---------+---------+---------+---------+---------|
contig022320 TGTGGTCAATGATTCATCTGCATCTTTTGTGGTTCTTGTCTTTTATTTTAACTCATGTTTAAACCCATTGATCTATGCTCTGTTTTACCCCTGGTTTAGAAAAGCTATTAAATGTGTTATCACTCTGCTG
TilTARs.A059 TGTGGTCAATGATTCATCTGCATCTTTTGTGGTTCTTGTCTTTTATTTTAACTCATGTTTAAACCCATTGATCTATGCTCTGTTTTACCCCTGGTTTAGAAAAGCTATTAAATGTGTTATCACTCTGCTG
   Consensus TGTGGTCAATGATTCATCTGCATCTTTTGTGGTTCTTGTCTTTTATTTTAACTCATGTTTAAACCCATTGATCTATGCTCTGTTTTACCCCTGGTTTAGAAAAGCTATTAAATGTGTTATCACTCTGCTG

             1040110410     10420     10430     10440     10450     10460     10470     10480     10490     10500     10510     10520     10530
             |--------+---------+---------+---------+---------+---------+---------+---------+---------+---------+---------+---------+---------|
contig022320 ATATTTAAGCATGACAGCAGTGAGGTCAACGTAATATAGAAATACAATGAAAGCTGCTCCACTAAGATTTTAAATCAGTAAATTGATACAGTTCAGTCTGGAACTGCATCAAAAATAAATTGATAAGGAA
TilTARs.A059 ATATTTAAGCATGACAGCAGTGAGGTCAACGTAATATAG
   Consensus ATATTTAAGCATGACAGCAGTGAGGTCAACGTAATATAG...........................................................................................
```

```
             1469114700    14710     14720     14730     14740     14750     14760     14770     14780     14790     14800     14810     14820
             |---------+---------+---------+---------+---------+---------+---------+---------+---------+---------+---------+---------+---------|
contig037879 GATAACATGCATCCCTGCTATGAATCAGAAAATGCCACTTATACTTTTACAAACAACCCTTCCATTGCCTGTGTTATATTGTATATTTTCCTTGGTTCATTGTCTGTTGTCACAATATTCGGAAACCTTC
  TilTAR.B060       ATGCATCCCTGCTATGAATCAGAAAATGCCACTTATACTTTTACAAACAACCCTTCCATTGCCTGTGTTATATTGTATATTTTCCTTGGTTCATTGTCTGTTGTCACAATATTCGGAAACCTTC
    Consensus ......ATGCATCCCTGCTATGAATCAGAAAATGCCACTTATACTTTTACAAACAACCCTTCCATTGCCTGTGTTATATTGTATATTTTCCTTGGTTCATTGTCTGTTGTCACAATATTCGGAAACCTTC

             1482114830    14840     14850     14860     14870     14880     14890     14900     14910     14920     14930     14940     14950
             |---------+---------+---------+---------+---------+---------+---------+---------+---------+---------+---------+---------+---------|
contig037879 TTGTAACAATTGCCATAATTTACTTCAAACAGCTTCACACGCCTACTAACTACCTCATCCTGTCTCTGGCTGTGGCTGACCTGCTTGTAGGAGTTTTAGTTTTCCCTTTCAGCATGGCCTTCACTGTTAC
  TilTAR.B060 TTGTAACAATTGCCATAATTTACTTCAAACAGCTTCACACGCCTACTAACTACCTCATCCTGTCTCTGGCTGTGGCTGACCTGCTTGTAGGAGTTTTAGTTTTCCCTTTCAGCATGGCCTTCACTGTTAC
    Consensus TTGTAACAATTGCCATAATTTACTTCAAACAGCTTCACACGCCTACTAACTACCTCATCCTGTCTCTGGCTGTGGCTGACCTGCTTGTAGGAGTTTTAGTTTTCCCTTTCAGCATGGCCTTCACTGTTAC

             1495114960    14970     14980     14990     15000     15010     15020     15030     15040     15050     15060     15070     15080
             |---------+---------+---------+---------+---------+---------+---------+---------+---------+---------+---------+---------+---------|
contig037879 TTTGTGTCTGGATCATGAAGATTTATTTTGCAAAATACGCGATAGCTTTGATGTGTCACTATGCACATCTTCTATTCTAAACTTGTGCTGCATTTCCATAGAGAGATATTATGCAGTTTGTCAGCCTCTG
  TilTAR.B060 TTTGTGTCTGGATCATGAAGATTTATTTTGCAAAATACGCGATAGCTTTGATGTGTCACTATGCACATCTTCTATTCTAAACTTGTGCTGCATTTCCATAGAGAGATATTATGCAGTTTGTCAGCCTCTG
    Consensus TTTGTGTCTGGATCATGAAGATTTATTTTGCAAAATACGCGATAGCTTTGATGTGTCACTATGCACATCTTCTATTCTAAACTTGTGCTGCATTTCCATAGAGAGATATTATGCAGTTTGTCAGCCTCTG

             1508115090    15100     15110     15120     15130     15140     15150     15160     15170     15180     15190     15200     15210
             |---------+---------+---------+---------+---------+---------+---------+---------+---------+---------+---------+---------+---------|
contig037879 AATTACAGATCAAAAATAAATACTCAAGTTACAATGTTTATGATACTGGTGAGCTGGGGCATTTCTGTATTAATTGGAATTTGCATTATAATTGCAGGATTCAGCCAAGGTATGTGTGAAGAAGTGTGCT
  TilTAR.B060 AATTACAGATCAAAAATAAATACTCAAGTTACAATGTTTATGATACTGGTGAGCTGGGGCATTTCTGTATTAATTGGAATTTGCATTATAATTGCAGGATTCAGCCAAGGTATGTGTGAAGAAGTGTGCT
    Consensus AATTACAGATCAAAAATAAATACTCAAGTTACAATGTTTATGATACTGGTGAGCTGGGGCATTTCTGTATTAATTGGAATTTGCATTATAATTGCAGGATTCAGCCAAGGTATGTGTGAAGAAGTGTGCT

             1521115220    15230     15240     15250     15260     15270     15280     15290     15300     15310     15320     15330     15340
             |---------+---------+---------+---------+---------+---------+---------+---------+---------+---------+---------+---------+---------|
contig037879 CTGTTGATGTTGTAATTGCAAACACTATGGGACCTGTTTTCTCATTTTACCTTCCAGCAATCATTATGCTTTGTATATACCTAAAGATTTTCCTTGTTGCACAGAAACAAGTAAGCACCATCCAAAATGC
  TilTAR.B060 CTGTTGATGTTGTAATTGCAAACACTATGGGACCTGTTTTCTCATTTTACCTTCCAGCAATCATTATGCTTTGTATATACCTAAAGATTTTCCTTGTTGCACAGAAACAAGTAAGCACCATCCAAAATGC
    Consensus CTGTTGATGTTGTAATTGCAAACACTATGGGACCTGTTTTCTCATTTTACCTTCCAGCAATCATTATGCTTTGTATATACCTAAAGATTTTCCTTGTTGCACAGAAACAAGTAAGCACCATCCAAAATGC

             1534115350    15360     15370     15380     15390     15400     15410     15420     15430     15440     15450     15460     15470
             |---------+---------+---------+---------+---------+---------+---------+---------+---------+---------+---------+---------+---------|
contig037879 AATCTGTACAAAATCTGGAGCAACTATTAGTAAGCTAGAAAGAAAGGCCACAAAAACGCTGTCTATTGTCATGGGAGTGTTTCTTTTATGTTTGAGTCCTTACTTTATTTGTGTTGTCTTTCAGCCTTTA
  TilTAR.B060 AATCTGTACAAAATCTGGAGCAACTATTAGTAAGCTAGAAAGAAAGGCCACAAAAACGCTGTCTATTGTCATGGGAGTGTTTCTTTTATGTTTGAGTCCTTACTTTATTTGTGTTGTCTTTCAGCCTTTA
    Consensus AATCTGTACAAAATCTGGAGCAACTATTAGTAAGCTAGAAAGAAAGGCCACAAAAACGCTGTCTATTGTCATGGGAGTGTTTCTTTTATGTTTGAGTCCTTACTTTATTTGTGTTGTCTTTCAGCCTTTA

             1547115480    15490     15500     15510     15520     15530     15540     15550     15560     15570     15580     15590     15600
             |---------+---------+---------+---------+---------+---------+---------+---------+---------+---------+---------+---------+---------|
contig037879 GCTCATAATCCCCCACCGATTCCTGTGATTGAAACACTTAACTGGCTTACGTTGTCAAATTCAATGTTAAATCCATTAATTTATGCTTTCTTTTATAGCTGGTTTAGGTCAGCTTACAGATTGATTGTTT
  TilTAR.B060 GCTCATAATCCCCCACCGATTCCTGTGATTGAAACACTTAACTGGCTTACGTTGTCAAATTCAATGTTAAATCCATTAATTTATGCTTTCTTTTATAGCTGGTTTAGGTCAGCTTACAGATTGATTGTTT
    Consensus GCTCATAATCCCCCACCGATTCCTGTGATTGAAACACTTAACTGGCTTACGTTGTCAAATTCAATGTTAAATCCATTAATTTATGCTTTCTTTTATAGCTGGTTTAGGTCAGCTTACAGATTGATTGTTT

             1560115610    15620     15630     15640     15650     15660     15670     15680     15690     15700     15710     15720     15730
             |---------+---------+---------+---------+---------+---------+---------+---------+---------+---------+---------+---------+---------|
contig037879 CTGGAACAATACTTCATTGTAATTTGGCTAACTCAAAATTGTTTTAATTTTAACAGCCATGAATGTGTATATGTGTGTAATAGCCATGAACGTGTGCATATGTGTAACAACCATGAATCTGTATATGTGC
  TilTAR.B060 CTGGAACAATACTTCATTGTAATTTGGCTAACTCAAAATTGTTTTAA
    Consensus CTGGAACAATACTTCATTGTAATTTGGCTAACTCAAAATTGTTTTAA...................................................................................
```

```
           1041 1050      1060      1070      1080      1090      1100      1110      1120      1130      1140      1150      1160      1170
           |--------+---------+---------+---------+---------+---------+---------+---------+---------+---------+---------+---------+---------|
contig037889 ATTCCATATTGTTGGTTTGAAACTGTTAAATTATGGAACAAAAAGTTAGTATGAACATGACCATCATTGTAAATGATCTACATCCCTGTTATGTAATACAAGACTCCAAATACATGCTGACAAACAGCCC
  TilTAR.B061                                                         ATGACCATCATTGTAAATGATCTACATCCCTGTTATGTAATACAAGACTCCAAATACATGCTGACAAACAGCCC
    Consensus ........................................................ATGACCATCATTGTAAATGATCTACATCCCTGTTATGTAATACAAGACTCCAAATACATGCTGACAAACAGCCC

           1171 1180      1190      1200      1210      1220      1230      1240      1250      1260      1270      1280      1290      1300
           |--------+---------+---------+---------+---------+---------+---------+---------+---------+---------+---------+---------+---------|
contig037889 TTCCATTATATGTGTATTCTTGTACGCACTCCTTGCATTATTGTCTGTTATAACAATATGTGGAAATCTTCTTGTAATAATTTCTGTGATTTACTTCAAACAGCTCCACACTCCTACTAACTCTCTCATC
  TilTAR.B061 TTCCATTATATGTGTATTCTTGTACGCACTCCTTGCATTATTGTCTGTTATAACAATATGTGGAAATCTTCTTGTAATAATTTCTGTGATTTACTTCAAACAGCTCCACACTCCTACTAACTCTCTCATC
    Consensus TTCCATTATATGTGTATTCTTGTACGCACTCCTTGCATTATTGTCTGTTATAACAATATGTGGAAATCTTCTTGTAATAATTTCTGTGATTTACTTCAAACAGCTCCACACTCCTACTAACTCTCTCATC

           1301 1310      1320      1330      1340      1350      1360      1370      1380      1390      1400      1410      1420      1430
           |--------+---------+---------+---------+---------+---------+---------+---------+---------+---------+---------+---------+---------|
contig037889 CTCTCTCTTGCTGTGGCTGACCTGCTCATTGGGATTATAGCATTTCCTCTCAGCATGGCATTCTCTCTCAGCTCATGTCTTTATCATGAGGGTTTGTTTTGCAAAGTGCGAGGCACCTTTGATATTTCAC
  TilTAR.B061 CTCTCTCTTGCTGTGGCTGACCTGCTCATTGGGATTATAGCATTTCCTCTCAGCATGGCATTCTCTCTCAGCTCATGTCTTTATCATGAGGGTTTGTTTTGCAAAGTGCGAGGCACCTTTGATATTTCAC
    Consensus CTCTCTCTTGCTGTGGCTGACCTGCTCATTGGGATTATAGCATTTCCTCTCAGCATGGCATTCTCTCTCAGCTCATGTCTTTATCATGAGGGTTTGTTTTGCAAAGTGCGAGGCACCTTTGATATTTCAC

           1431 1440      1450      1460      1470      1480      1490      1500      1510      1520      1530      1540      1550      1560
           |--------+---------+---------+---------+---------+---------+---------+---------+---------+---------+---------+---------+---------|
contig037889 TGAGCACATGTTCCATCCTGAATCTCTGCTGTATTTCTATTGACAGATATCATGCTGTTTGCCAGCCTCTGACTTATCAAACTAAAATCAGCCATCGTGCTGTTGTGTTCATGATCCTGATGAGCTGGGG
  TilTAR.B061 TGAGCACATGTTCCATCCTGAATCTCTGCTGTATTTCTATTGACAGATATCATGCTGTTTGCCAGCCTCTGACTTATCAAACTAAAATCAGCCATCGTGCTGTTGTGTTCATGATCCTGATGAGCTGGGG
    Consensus TGAGCACATGTTCCATCCTGAATCTCTGCTGTATTTCTATTGACAGATATCATGCTGTTTGCCAGCCTCTGACTTATCAAACTAAAATCAGCCATCGTGCTGTTGTGTTCATGATCCTGATGAGCTGGGG

           1561 1570      1580      1590      1600      1610      1620      1630      1640      1650      1660      1670      1680      1690
           |--------+---------+---------+---------+---------+---------+---------+---------+---------+---------+---------+---------+---------|
contig037889 GGTTTCTGGGATAATTGGAATTAGTGTCACCATTGCTGGATTAAATAATGAAAAATATGAGGAATCATTTTTGATTGACGTTCTAATGGAAAGCACTGTTGGACCCATGTTATCATTTTACCTGCCAGTG
  TilTAR.B061 GGTTTCTGGGATAATTGGAATTAGTGTCACCATTGCTGGATTAAATAATGAAAAATATGAGGAATCATTTTTGATTGACGTTCTAATGGAAAGCACTGTTGGACCCATGTTATCATTTTACCTGCCAGTG
    Consensus GGTTTCTGGGATAATTGGAATTAGTGTCACCATTGCTGGATTAAATAATGAAAAATATGAGGAATCATTTTTGATTGACGTTCTAATGGAAAGCACTGTTGGACCCATGTTATCATTTTACCTGCCAGTG

           1691 1700      1710      1720      1730      1740      1750      1760      1770      1780      1790      1800      1810      1820
           |--------+---------+---------+---------+---------+---------+---------+---------+---------+---------+---------+---------+---------|
contig037889 ATCATAATGCTTTGTATCTACCTAAAGATTTTCTTTGTTGCACGGAGGCAAGCACGCAGCATCCAGAACACAACAAAGTGTGGACAAACTGCCAGCATGATGGAGAGAAAGGCGACCAAAACTCTGGCTA
  TilTAR.B061 ATCATAATGCTTTGTATCTACCTAAAGATTTTCTTTGTTGCACGGAGGCAAGCACGCAGCATCCAGAACACAACAAAGTGTGGACAAACTGCCAGCATGATGGAGAGAAAGGCGACCAAAACTCTGGCTA
    Consensus ATCATAATGCTTTGTATCTACCTAAAGATTTTCTTTGTTGCACGGAGGCAAGCACGCAGCATCCAGAACACAACAAAGTGTGGACAAACTGCCAGCATGATGGAGAGAAAGGCGACCAAAACTCTGGCTA

           1821 1830      1840      1850      1860      1870      1880      1890      1900      1910      1920      1930      1940      1950
           |--------+---------+---------+---------+---------+---------+---------+---------+---------+---------+---------+---------+---------|
contig037889 TTGTTTTGGGAGTTTTTATATTATGCTGGAGTCCTTTTTTTTCTTTGCATCACATTTCCACCATTTACCAGTGATTACCAGTACCAGTTACAGGTACCAGTTCCTGTAATTGAAACTCTTAACTGGCTTAC
  TilTAR.B061 TTGTTTTGGGAGTTTTTATATTATGCTGGAGTCCTTTTTTTTCTTTGCATCACATTTCCACCATTTACCAGTGATTACCAGTACCAGTTACAGGTACCAGTTCCTGTAATTGAAACTCTTAACTGGCTTAC
    Consensus TTGTTTTGGGAGTTTTTATATTATGCTGGAGTCCTTTTTTTTCTTTGCATCACATTTCCACCATTTACCAGTGATTACCAGTACCAGTTACAGGTACCAGTTCCTGTAATTGAAACTCTTAACTGGCTTAC

           1951 1960      1970      1980      1990      2000      2010      2020      2030      2040      2050      2060      2070      2080
           |--------+---------+---------+---------+---------+---------+---------+---------+---------+---------+---------+---------+---------|
contig037889 ATTGGCAAATTCAATGCTAAATCCATTTATTTATGCTTTCTTTTACAGCTGGTTCAGGTCAGCCATCAGAATGATTATTTGTGGGAAAATATTCCAAGGGGATTTTGCAAACACAAAATTGACTTGAACA
  TilTAR.B061 ATTGGCAAATTCAATGCTAAATCCATTTATTTATGCTTTCTTTTACAGCTGGTTCAGGTCAGCCATCAGAATGATTATTTGTGGGAAAATATTCCAAGGGGATTTTGCAAACACAAAATTGACTTGA
    Consensus ATTGGCAAATTCAATGCTAAATCCATTTATTTATGCTTTCTTTTACAGCTGGTTCAGGTCAGCCATCAGAATGATTATTTGTGGGAAAATATTCCAAGGGGATTTTGCAAACACAAAATTGACTTGA...
```

```
               1404114050    14060     14070     14080     14090     14100     14110     14120     14130     14140     14150     14160     14170
               |---------+---------+---------+---------+---------+---------+---------+---------+---------+---------+---------+---------+---------|
contig002574   GTTGACCTGTACTGAAGGACAATCTACCTCAAGTCAAGTCCCAGGTTAGTGCAGCAGAAAAAATCATATTGTTGGTTTAAACTGTTTAATTATGGAACAAAGGTTAGTATGAACATGACCAACATTGTA
TilTAR.B062                                                                                                                       ATGACCAACATTGTA
Consensus      ...................................................................................................................ATGACCAACATTGTA

               1417114180    14190     14200     14210     14220     14230     14240     14250     14260     14270     14280     14290     14300
               |---------+---------+---------+---------+---------+---------+---------+---------+---------+---------+---------+---------+---------|
contig002574   AATGATCTACATCCCTGTTATGTAATACAAGACTCCAAATACATGCTGACAAACAGCCCTTCCATTATATGTGTATTCTTGTATGCATTCCTTGCATTATTGTCTGTTATAACAATATGTGGAAATCTCC
TilTAR.B062    AATGATCTACATCCCTGTTATGTAATACAAGACTCCAAATACATGCTGACAAACAGCCCTTCCATTATATGTGTATTCTTGTATGCATTCCTTGCATTATTGTCTGTTATAACAATATGTGGAAATCTCC
Consensus      AATGATCTACATCCCTGTTATGTAATACAAGACTCCAAATACATGCTGACAAACAGCCCTTCCATTATATGTGTATTCTTGTATGCATTCCTTGCATTATTGTCTGTTATAACAATATGTGGAAATCTCC

               1430114310    14320     14330     14340     14350     14360     14370     14380     14390     14400     14410     14420     14430
               |---------+---------+---------+---------+---------+---------+---------+---------+---------+---------+---------+---------+---------|
contig002574   TTGTAATAATTTCTGTGATTTACTTCAAACAGCTCCATACTCCTACTAATTCTCTCATCCTCTCTCTTGCTGTGGCTGACCTGCTCGTTGGGATTATAGCATTTCCTCTCAGCATGGCATTCTCTCTCAG
TilTAR.B062    TTGTAATAATTTCTGTGATTTACTTCAAACAGCTCCATACTCCTACTAATTCTCTCATCCTCTCTCTTGCTGTGGCTGACCTGCTCGTTGGGATTATAGCATTTCCTCTCAGCATGGCATTCTCTCTCAG
Consensus      TTGTAATAATTTCTGTGATTTACTTCAAACAGCTCCATACTCCTACTAATTCTCTCATCCTCTCTCTTGCTGTGGCTGACCTGCTCGTTGGGATTATAGCATTTCCTCTCAGCATGGCATTCTCTCTCAG

               1443114440    14450     14460     14470     14480     14490     14500     14510     14520     14530     14540     14550     14560
               |---------+---------+---------+---------+---------+---------+---------+---------+---------+---------+---------+---------+---------|
contig002574   CTCATGTCTTTATCATGAGGGTTTGTTTTGCAAAGTGCGAGGGACCTTTGATATTTCACTGAGCACATGTTCCATCCTGAACCTCTGCTGTATTTCTGTTGACAGATACCATGCTGTTTGCCAGCCTCTG
TilTAR.B062    CTCATGTCTTTATCATGAGGGTTTGTTTTGCAAAGTGCGAGGGACCTTTGATATTTCACTGAGCACATGTTCCATCCTGAACCTCTGCTGTATTTCTGTTGACAGATACCATGCTGTTTGCCAGCCTCTG
Consensus      CTCATGTCTTTATCATGAGGGTTTGTTTTGCAAAGTGCGAGGGACCTTTGATATTTCACTGAGCACATGTTCCATCCTGAACCTCTGCTGTATTTCTGTTGACAGATACCATGCTGTTTGCCAGCCTCTG

               1456114570    14580     14590     14600     14610     14620     14630     14640     14650     14660     14670     14680     14690
               |---------+---------+---------+---------+---------+---------+---------+---------+---------+---------+---------+---------+---------|
contig002574   ACATATCAAACTAAAATCAGCCATCGTGTTGTTGTGTTCATGATCCTGATGAGCTGGGGGGTTTCTGGGATAATTGGAATTAGTGTCACCATTGCTGGATTTAATAATGAAAAATGTGAGGAATCATGTT
TilTAR.B062    ACATATCAAACTAAAATCAGCCATCGTGTTGTTGTGTTCATGATCCTGATGAGCTGGGGGGTTTCTGGGATAATTGGAATTAGTGTCACCATTGCTGGATTTAATAATGAAAAATGTGAGGAATCATGTT
Consensus      ACATATCAAACTAAAATCAGCCATCGTGTTGTTGTGTTCATGATCCTGATGAGCTGGGGGGTTTCTGGGATAATTGGAATTAGTGTCACCATTGCTGGATTTAATAATGAAAAATGTGAGGAATCATGTT

               1469114700    14710     14720     14730     14740     14750     14760     14770     14780     14790     14800     14810     14820
               |---------+---------+---------+---------+---------+---------+---------+---------+---------+---------+---------+---------+---------|
contig002574   TGATTGACGTTCTAATGGAAAGCACTGTTGGACCCATGTTATCATTTTACCTGCCAGTGACCATAATGCTTTGTATCTACCTAAAGATTTTCTTTGTTGCTTTGAAGCAAGCAAGCAGCATCCAGAACAC
TilTAR.B062    TGATTGACGTTCTAATGGAAAGCACTGTTGGACCCATGTTATCATTTTACCTGCCAGTGACCATAATGCTTTGTATCTACCTAAAGATTTTCTTTGTTGCTTTGAAGCAAGCAAGCAGCATCCAGAACAC
Consensus      TGATTGACGTTCTAATGGAAAGCACTGTTGGACCCATGTTATCATTTTACCTGCCAGTGACCATAATGCTTTGTATCTACCTAAAGATTTTCTTTGTTGCTTTGAAGCAAGCAAGCAGCATCCAGAACAC

               1482114830    14840     14850     14860     14870     14880     14890     14900     14910     14920     14930     14940     14950
               |---------+---------+---------+---------+---------+---------+---------+---------+---------+---------+---------+---------+---------|
contig002574   AACAAAGTGTGGACAAACTGCCAGTATCATGGAGAGAAAGGCAACCAAAACTCTGGCTATTGTTTTGGGAGTTTTTATATTTTGCTGGAGTCCTTGTTTTCTTTGCATCACATTCCCACCATTTACCAGT
TilTAR.B062    AACAAAGTGTGGACAAACTGCCAGTATCATGGAGAGAAAGGCAACCAAAACTCTGGCTATTGTTTTGGGAGTTTTTATATTTTGCTGGAGTCCTTGTTTTCTTTGCATCACATTCCCACCATTTACCAGT
Consensus      AACAAAGTGTGGACAAACTGCCAGTATCATGGAGAGAAAGGCAACCAAAACTCTGGCTATTGTTTTGGGAGTTTTTATATTTTGCTGGAGTCCTTGTTTTCTTTGCATCACATTCCCACCATTTACCAGT

               1495114960    14970     14980     14990     15000     15010     15020     15030     15040     15050     15060     15070     15080
               |---------+---------+---------+---------+---------+---------+---------+---------+---------+---------+---------+---------+---------|
contig002574   GATTCTGTACCAGTTCCTGTAATTGAAACTCTTAACTGGCTTGCATTGGCAAATTCAATGCTAAATCCATTTATTTATGCTTTCTTTTATAGGTGGTTCAGGTCAGCCATCAGAATGATTATTTGTGGGA
TilTAR.B062    GATTCTGTACCAGTTCCTGTAATTGAAACTCTTAACTGGCTTGCATTGGCAAATTCAATGCTAAATCCATTTATTTATGCTTTCTTTTATAGGTGGTTCAGGTCAGCCATCAGAATGATTATTTGTGGGA
Consensus      GATTCTGTACCAGTTCCTGTAATTGAAACTCTTAACTGGCTTGCATTGGCAAATTCAATGCTAAATCCATTTATTTATGCTTTCTTTTATAGGTGGTTCAGGTCAGCCATCAGAATGATTATTTGTGGGA

               1508115090    15100     15110     15120     15130     15140     15150     15160     15170     15180     15190     15200     15210
               |---------+---------+---------+---------+---------+---------+---------+---------+---------+---------+---------+---------+---------|
contig002574   AAATATTCCAAAGGGATTTTGCCAACACAAATATGACTTGATAAAATATACACATATTAATTAAAATTTCACAAATCTTACAGTAAAAACAAACATAAAACAGCCACTGAACCCTACCTGAAGCATCACA
TilTAR.B062    AAATATTCCAAAGGGATTTTGCCAACACAAATATGACTTGA
Consensus      AAATATTCCAAAGGGATTTTGCCAACACAAATATGACTTGA.........................................................................................
```

```
             391     400        410        420        430        440        450        460        470        480        490        500        510        520
             |--------+----------+----------+----------+----------+----------+----------+----------+----------+----------+----------+----------+---------|
contig037900 ATGGATGGATGGATGGATGGAATATAACTTATAGGAACCCAGAAACATGGCACCAGATGTCCCTGTCAACCGCACTGATGCTGATGACTTGCACCCTTGTTATAAAATAGATAAGGGCTCCTACATAGTA
  TilTAR.B063                                               ATGGCACCAGATGTCCCTGTCAACCGCACTGATGCTGATGACTTGCACCCTTGTTATAAAATAGATAAGGGCTCCTACATAGTA
    Consensus ..............................................ATGGCACCAGATGTCCCTGTCAACCGCACTGATGCTGATGACTTGCACCCTTGTTATAAAATAGATAAGGGCTCCTACATAGTA

             521     530        540        550        560        570        580        590        600        610        620        630        640        650
             |--------+----------+----------+----------+----------+----------+----------+----------+----------+----------+----------+----------+---------|
contig037900 AAAAGCAACCCTTCCACACTCTGTGTCTTATTGCACATTTTTCTGGGATCATTATCTCTTGTAACTGTGTGTGGAAATCTTCTGGTAATAATCTCCATCATTTATTTCAAACAGCTCCACACTCTCACTA
  TilTAR.B063 AAAAGCAACCCTTCCACACTCTGTGTCTTATTGCACATTTTTCTGGGATCATTATCTCTTGTAACTGTGTGTGGAAATCTTCTGGTAATAATCTCCATCATTTATTTCAAACAGCTCCACACTCTCACTA
    Consensus AAAAGCAACCCTTCCACACTCTGTGTCTTATTGCACATTTTTCTGGGATCATTATCTCTTGTAACTGTGTGTGGAAATCTTCTGGTAATAATCTCCATCATTTATTTCAAACAGCTCCACACTCTCACTA

             651     660        670        680        690        700        710        720        730        740        750        760        770        780
             |--------+----------+----------+----------+----------+----------+----------+----------+----------+----------+----------+----------+---------|
contig037900 ACTCTCTGGTCCTCTCTCTGGCTGTGGCTGACCTGGTTGTTGGAGTTTTAGTGTTTCCTTTGAGCATGAGATTCACTGTTAGTGTGTGTCTGTTTTATCAAGATTTATTCTGCAAAGTACGAGACAGCTT
  TilTAR.B063 ACTCTCTGGTCCTCTCTCTGGCTGTGGCTGACCTGGTTGTTGGAGTTTTAGTGTTTCCTTTGAGCATGAGATTCACTGTTAGTGTGTGTCTGTTTTATCAAGATTTATTCTGCAAAGTACGAGACAGCTT
    Consensus ACTCTCTGGTCCTCTCTCTGGCTGTGGCTGACCTGGTTGTTGGAGTTTTAGTGTTTCCTTTGAGCATGAGATTCACTGTTAGTGTGTGTCTGTTTTATCAAGATTTATTCTGCAAAGTACGAGACAGCTT

             781     790        800        810        820        830        840        850        860        870        880        890        900        910
             |--------+----------+----------+----------+----------+----------+----------+----------+----------+----------+----------+----------+---------|
contig037900 TGATGTAACCTTGAGCACATCTTCCATTTTGAATTTGTGTTGTATTTCCATTGACAGATACTATGCTGTGTGTAGGCCTCTGACATACAGAAGTAAGATTAATGTTCATGTTGTTGTGATCATGATCCTG
  TilTAR.B063 TGATGTAACCTTGAGCACATCTTCCATTTTGAATTTGTGTTGTATTTCCATTGACAGATACTATGCTGTGTGTAGGCCTCTGACATACAGAAGTAAGATTAATGTTCATGTTGTTGTGATCATGATCCTG
    Consensus TGATGTAACCTTGAGCACATCTTCCATTTTGAATTTGTGTTGTATTTCCATTGACAGATACTATGCTGTGTGTAGGCCTCTGACATACAGAAGTAAGATTAATGTTCATGTTGTTGTGATCATGATCCTG

             911     920        930        940        950        960        970        980        990       1000       1010       1020       1030       1040
             |--------+----------+----------+----------+----------+----------+----------+----------+----------+----------+----------+----------+---------|
contig037900 GTAAGCTGGAGTGTTTCTATTTTAGTTGCTGTTGGCTTTCTTATTGCAGGATTAAACCATGAAAAATGCAAAGAAGGCTGGATTTCTGAACTTCTACTTGCAAATATTTTGGGACCTGTGCTCTCATTTT
  TilTAR.B063 GTAAGCTGGAGTGTTTCTATTTTAGTTGCTGTTGGCTTTCTTATTGCAGGATTAAACCATGAAAAATGCAAAGAAGGCTGGATTTCTGAACTTCTACTTGCAAATATTTTGGGACCTGTGCTCTCATTTT
    Consensus GTAAGCTGGAGTGTTTCTATTTTAGTTGCTGTTGGCTTTCTTATTGCAGGATTAAACCATGAAAAATGCAAAGAAGGCTGGATTTCTGAACTTCTACTTGCAAATATTTTGGGACCTGTGCTCTCATTTT

             1041   1050       1060       1070       1080       1090       1100       1110       1120       1130       1140       1150       1160       1170
             |--------+----------+----------+----------+----------+----------+----------+----------+----------+----------+----------+----------+---------|
contig037900 ATCTCCCTGTGATAATAATGCTCTGTATCTACCTGAAGATTTTCCTTGTTGCAGAGAAACATGCTCGCAGCATCCAGAACACAAAGTGTGGTGCCACTGTGGTGAGTAAAATGGAGAAGAAGGCCACTAA
  TilTAR.B063 ATCTCCCTGTGATAATAATGCTCTGTATCTACCTGAAGATTTTCCTTGTTGCAGAGAAACATGCTCGCAGCATCCAGAACACAAAGTGTGGTGCCACTGTGGTGAGTAAAATGGAGAAGAAGGCCACTAA
    Consensus ATCTCCCTGTGATAATAATGCTCTGTATCTACCTGAAGATTTTCCTTGTTGCAGAGAAACATGCTCGCAGCATCCAGAACACAAAGTGTGGTGCCACTGTGGTGAGTAAAATGGAGAAGAAGGCCACTAA

             1171   1180       1190       1200       1210       1220       1230       1240       1250       1260       1270       1280       1290       1300
             |--------+----------+----------+----------+----------+----------+----------+----------+----------+----------+----------+----------+---------|
contig037900 AACTCTGGCAACTGTTATGGGAGTTTTTGTGATGTGTTGGCTGCCTTTCTTTTTCTGTGTCACTCTTCAACTACTGGGTCATATATTAATACCACTTCCAGTGTATGAAACACTTAATTGGCTGGCACTG
  TilTAR.B063 AACTCTGGCAACTGTTATGGGAGTTTTTGTGATGTGTTGGCTGCCTTTCTTTTTCTGTGTCACTCTTCAACTACTGGGTCATATATTAATACCACTTCCAGTGTATGAAACACTTAATTGGCTGGCACTG
    Consensus AACTCTGGCAACTGTTATGGGAGTTTTTGTGATGTGTTGGCTGCCTTTCTTTTTCTGTGTCACTCTTCAACTACTGGGTCATATATTAATACCACTTCCAGTGTATGAAACACTTAATTGGCTGGCACTG

             1301   1310       1320       1330       1340       1350       1360       1370       1380       1390       1400       1410       1420       1430
             |--------+----------+----------+----------+----------+----------+----------+----------+----------+----------+----------+----------+---------|
contig037900 TGTAATTCAATGCTAAATCCCTTTATTTATGCTTTCTTTTAAAGCAGAATGCAAAACTACGTATGTGTTTATACATGCATATATATATATATATATATAAGAATGAAAGAATGTGAAACTGGCTAGGTAC
  TilTAR.B063 TGTAATTCAATGCTAAATCCCTTTATTTATGCTTTCTTTTAA
    Consensus TGTAATTCAATGCTAAATCCCTTTATTTATGCTTTCTTTTAA........................................................................................
```
