# Supplementary material for: Identification and characterization of cichlid TAAR genes and comparison with other teleost TAAR repertoires
Source: BMC Genomics. 2015 Apr 23;16(1):335. doi: 10.1186/s12864-015-1478-4 (PMC4415300; doi:10.1186/s12864-015-1478-4)
Supplement: Additional file 4: — Intron positions within TAAR genes. The nature of the last codon of the first coding exon, and its position and its phase are reported. [file 12864_2015_1478_MOESM4_ESM.pdf]

TAAR names	last codon	codon #	intron phase	Position within 2D
TilTARs.A019	TTC=F	56	0	INTLOOP1
TilTARs.A024	TTC=F	55	0	INTLOOP1
TilTARs.A025	TTC=F	57	0	INTLOOP1
TilTARs.A026	TTC=F	57	0	INTLOOP1
TilTARs.A027	TTC=F	59	0	INTLOOP1
TilTARs.A028	TTC=F	57	0	INTLOOP1
TilTARs.A029	TTC=F	57	0	INTLOOP1
TilTARs.A031	TTC=F	58	0	INTLOOP1
TilTARs.A032	TTC=F	57	0	INTLOOP1
TilTARs.A033	TTC=F	57	0	INTLOOP1
TilTARs.A034	TTC=F	57	0	INTLOOP1
TilTARs.A035	TTC=F	57	0	INTLOOP1
TilTARs.A036	TTC=F	57	0	INTLOOP1
TilTARs.A037	TTC=F	57	0	INTLOOP1
TilTARs.A038	TTC=F	57	0	INTLOOP1
TilTARs.A039	TTC=F	57	0	INTLOOP1
TilTARs.A040	TTC=F	57	0	INTLOOP1
TilTARs.A041	TTC=F	57	0	INTLOOP1
TilTARs.A042	TTC=F	57	0	INTLOOP1
TilTARs.A043	TTC=F	57	0	INTLOOP1
TilTARs.A044	TTC=F	56	0	INTLOOP1
TilTARs.A045	TTC=F	54	0	INTLOOP1
TilTARs.A046	TTC=F	57	0	INTLOOP1
TilTARs.A047	TTC=F	55	0	INTLOOP1
TilTARs.A048	TTC=F	57	0	INTLOOP1
TilTARs.A050	TTC=F	55	0	INTLOOP1
TilTARs.A051	TTC=F	56	0	INTLOOP1
TilTARs.A052	TAC=TYR	57	0	INTLOOP1
TilTARs.A053	TTC=F	57	0	INTLOOP1
TilTARs.A054	TTC=F	57	0	INTLOOP1
TilTARs.A055	TTC=F	57	0	INTLOOP1
TilTARs.A056	TTC=F	57	0	INTLOOP1
TilTARs.A057	TTC=F	54	0	INTLOOP1
TilTARs.A058	TTC=F	54	0	INTLOOP1
TilTARs.A059	TTC=F	57	0	INTLOOP1
ZebTARs.A014	TTC=F	57	0	INTLOOP1
ZebTARs.A015	TTC=F	57	0	INTLOOP1
ZebTARs.A016	TTC=F	55	0	INTLOOP1
ZebTARs.A017	TTC=F	54	0	INTLOOP1
ZebTARs.A018	TTC=F	57	0	INTLOOP1
ZebTARs.A019	TTC=F	57	0	INTLOOP1
ZebTARs.A020	TTC=F	57	0	INTLOOP1
ZebTARs.A021	TAC=TYR	55	0	INTLOOP1
ZebTARs.A022	TTC=F	57	0	INTLOOP1
ZebTARs.A023	TTC=F	54	0	INTLOOP1
ZebTARs.A024	TTC=F	57	0	INTLOOP1
ZebTARs.A025	TTC=F	55	0	INTLOOP1
ZebTARs.A026	TTC=F	57	0	INTLOOP1
ZebTARs.A027	TTC=F	54	0	INTLOOP1

ZebTARs.A028	TTC=F	54	0	INTLOOP1
ZebTARs.A029	TTC=F	57	0	INTLOOP1
BurTARs.A012	TAC=TYR	54	0	INTLOOP1
BurTARs.A013	TTC=F	59	0	INTLOOP1
BurTARs.A014	TTC=F	57	0	INTLOOP1
BurTARs.A015	TTC=F	54	0	INTLOOP1
BurTARs.A016	CTC=LEU	42	0	N-ter
BurTARs.A017	TTC=F	54	0	INTLOOP1
BurTARs.A018	TTC=F	57	0	INTLOOP1
BurTARs.A019	TTC=F	59	0	INTLOOP1
BurTARs.A020	TTC=F	57	0	INTLOOP1
BurTARs.A021	TTC=F	57	0	INTLOOP1
BurTARs.A022	TTC=F	55	0	INTLOOP1
BurTARs.A023	TTC=F	57	0	INTLOOP1
BurTARs.A024	TTC=F	57	0	INTLOOP1
BurTARs.A025	TTC=F	55	0	INTLOOP1
BurTARs.A026	TTC=F	57	0	INTLOOP1
BurTARs.A027	TTC=F	57	0	INTLOOP1
BurTARs.A028	TTC=F	55	0	INTLOOP1
BurTARs.A029	TTC=F	54	0	INTLOOP1
BurTARs.A030	TTC=F	54	0	INTLOOP1
BurTARs.A031	TTC=F	53	0	INTLOOP1
BriTARs.A014	AAC=N	47	0	TM1
BriTARs.A015	TAC=TYR	57	0	INTLOOP1
BriTARs.A016	TTC=F	59	0	INTLOOP1
BriTARs.A017	TTC=F	55	0	INTLOOP1
BriTARs.A018	TTC=F	57	0	INTLOOP1
BriTARs.A019	TTC=F	54	0	INTLOOP1
BriTARs.A020	TTC=F	54	0	INTLOOP1
BriTARs.A021	TTC=F	53	0	INTLOOP1
BriTARs.A022	TTC=F	55	0	INTLOOP1
NyeTARs.A013	TTC=F	57	0	INTLOOP1
NyeTARs.A014	TTC=F	57	0	INTLOOP1
NyeTARs.A015	TTC=F	57	0	INTLOOP1
NyeTARs.A017	TTC=F	55	0	INTLOOP1
NyeTARs.A018	TTC=F	54	0	INTLOOP1
NyeTARs.A019	TTC=F	55	0	INTLOOP1
NyeTARs.A021	TTC=F	57	0	INTLOOP1
NyeTARs.A022	TTC=F	56	0	INTLOOP1
NyeTARs.A023	TTC=F	57	0	INTLOOP1
NyeTARs.A024	TTC=F	57	0	INTLOOP1
NyeTARs.A025	TTC=F	57	0	INTLOOP1
NyeTARs.A026	TTC=F	59	0	INTLOOP1
NyeTARs.A027	TTC=F	53	0	INTLOOP1
NyeTARs.A028	TTC=F	54	0	INTLOOP1
NyeTARs.A029	TTC=F	57	0	INTLOOP1
