# Supplementary material for: Identification and characterization of cichlid TAAR genes and comparison with other teleost TAAR repertoires
Source: BMC Genomics. 2015 Apr 23;16(1):335. doi: 10.1186/s12864-015-1478-4 (PMC4415300; doi:10.1186/s12864-015-1478-4)
Supplement: Additional file 5: — Sequences of the cichlid and tetraodon TAAR genes and their corresponding receptors. Dipeptides at the splice junction are shown in red. [file 12864_2015_1478_MOESM5_ESM.pdf]

Cichlid TAAR gene sequences

>contig063687-BurTARe.A003
ATGGAGACCCAGGACGAAGCAGAGCTCTGCTTTCCACAACTCTTCAACATCTCCTGCAAGAAGCCTAAAACATCTCTGTCTCAAGTTTTGCTCCCTTACGTTGTGGTGT
TTTCAGTCTCTCTGCTAACTGTGGCTCTCAACCTTCTCGTCATTGTCTCAGTCTCCCACTTCAGGCAGCTCCACACACCCACTAACATCCTCCTCCTCTCTCTGGCTGT
CTCTGACTTTCTCATTGGTCTGTTGTTGATGCCGGCAAAAATCCTACGAGACACAGCTTGTTGGTTTCTTGGTCAGCTTGCATGTTTTGTGTGTAGTTATATATGCTTCGT
CATTACCTCTGCCTCAGTGGGCATTATGGTGCTGATATCAGTCGACCGCTATGTGGCTATTTGTGACCCTCTGCATTACCCCACCAGAATCACTGACAGAAGAGTGAAA
CTCTGTGTCTGTCTGTGTTGGCTCTGCTCGGTTTTTTACAACATACTATTTATAAAGGACGACCTGCTTCAACGAGAACGACATACTTCCTGTTATGGAGAATGTGTATTT
GTCATAGACTACATTGTAGGAACCACTGACATTGTTTTAACTTTTATTGCTCCAGTTACTGTCATCGTATTTCTGTATATGAGAGTATTTGTGGTGGCTGTGTCTCAGGCCC
GTGCCATG
>contig059670-BurTARe.A004
ATGGAAGAAACTGAACTCTGCTTTCCACAACTCCTCAACTCCTCTTGTGTGCGGCAAAAGCGCCCACAAATTGAGGCTGTGTGTATTTACACCCTGCTATCATCTATCT
CTCTGCTTACTGCAGTACTGAACCTACTTGTCATCATCTCCATCGCTCACTTCAAGCAGCTGCACACTCCAACCAACCTCCTCCTCCTCTCTCTGGCCGTCTCAGATTT
CTTCGTGGGCCTCATCATGGCTTTTGAGATTAGCCTCCTAGATGGCTGCTGGTTTCTTGGTGACCACATGTGTGCTCTGTATAGCAGTTTAGATTACATTGTTACTTCTG
CTTCAGTAGGAACTATGGTACTCATATCAGCTGACCGCTATGTAGCCATTTGTGACCCTCTGCATTATCCCACCAAAATTACTATGAAAAGAGTCTCAGTCTCTATTTGTAC
TTGCTGGGCTTGTTCAATTCTGTATAACAGTCTTATCATGAAGGATAATTTCAAGCAGCCAGGGAGGTATAATTCTTGTTCTGGTGATTGTGTAGTTGTCATTGATTACTTT
GTGGGAATTTTTGACTTTGTTTTGACCTTTGTCGGCCCTGTCATTGTCATCATAGTTCTGTATCTAAGAGTATTTGTGGTGGCTGTGTCTCAGGCTCGGGCAATGCGCTC
TCATATCACTGCTCTCAGACTCCAGGGGTCAGAGACGGTGCATGCTAAGAAATCTGAGCTGAAAGCTGCCAGGACTCTCGGTGTACTTGTTATTGCCTTTCTGATATGT
CTTTTCCCATTTTTCTGTTCCTCAATGGTAGGCCAGAATAGTTTCTTT
>contig045302-BurTARe.A005
ATGGACGGCACTGGAGGTCCTCCCCTCTGCTTCCCTAACCTCAACTCCTCCTGCAGGCGGCTGCTGCGACCCACCTCCCAGGCCACTCTTCTCTACACTCTACTGGC
TTCAGTCTCACTGCTCACTGTGGTGCTCAACCTGCTTGTGGTCGTCTCCATCTCCCACTTCAAGCAGCTCCACACCCCGACCAACGCCCTGCTCCTGTCCCTGGCCG
TGTCCGACCTGCTGGTGGGGCTGCTGGTGATGCCCATCGAGGGCCTGCGCTACATTGAGACGTGCTGGCTGCTGGGGAGGCTGATGTGTGCTCTCAGTCCTTATTT
GTCTTACTGCCTTATCTCTGTCTCTCTGGGCAGCATGGTGCTCATA
>contig034854-BurTARe.A006
ATGATTTCTCTCTCTTCTTTCAGACAGCTCCACACACCCACTAACATCCTCCTCCTCTCTCTGGCTGTGTCAGACTTTTTCATTGGTCTGTTGTTGATGCCTGCAAATATC
ATCCGAAAGAAGGTTTGTTGGTTTCTTGGTCAGCTTGCATGTTTAGTGTTTACTTATGTATCCTTCATTATTACCTCTGCCTCAGTGGGCATCATGGTGCTGATATCAATC
GACAGGTATGCGGCTATTTGTGACCCTCTGCATTACCCCACCAGAATAACTCGTGAAAGAGTGAAACTCTGTGTCTGTCTGTGTTGGCTCTGCTCTGTTATCTACAACAT
TTTGCTCATAAAGGATGAACTGGTTCAACCAGGGCAACATAATTCCTGTTATGGAGAGTGTAAAATTCTCGTAGACTACATTGCAGGAACCACTGATATTGTTTTAACCTT
TATTGCTCCAGTTACTGTCATCATTGTTCTGTATATGAGAGTATTTGTGGTGGCTGTGTCTCAGGCCCGTGCCATGCGCTCTCAGGTTACAGCTGTCACACTACAGCTCT
CAGTGACTCTAACAACAAAGAAATCAGAGTTAAAAGCAGCCAGGACACTGGGTGTTCTTGTTCTTGTGTTTCTAATATGTTTCTGTCCTCATTATTGTATTTTTGTTTTTG
GAGGAAAGGTGCTTAATAGCTCATCTGCAACTATTGTGATCTATCTGTACTATTTTAACTCCTGTTTAAACCCTTTGATTTATGCTATGTTCTACCCCTGGTTTAGAAAAGCT
GTGAAACTAATTGTCACACTACAGATACTGCAGCCTGGCTCCTGTGAAGTCAGCATACTGtag
>contig061433-BurTARf.A007
ATGGAGATACAAAATAAAGCTGAGCTTTGTTTTCCAGAACTCCTCAACAGTTCCTGCAGGAGGATGACGCTTTACTGGTCCAAAGCTGTACTCCTGTACATTGAGGTGT
TCTGCATCTCTCTGATCACTGCTGCTCTAAACCTTCTGGTCATCATCTCAGTCTCCCACTTAAGGCAT
>contig056023-BurTARf.A008
GTGTCTCCCTCCAGGCAGCTTCGCACACCCACTAACATCCTCCTCCTCTCTCTGGCTGTCTCAGATGTTTTTGTGGGACTCCTGTTACTGCCATTAGAAATATTTAGAA
GTACAACCTGCTGGGTACTTGGTGATCTTATGTGTTTACTTTATATTTATATGACCAGCAACATTGTCTGTGCTTCAATAGGGAACATAGTTCTCATCTCAGTTGACCGTTA
TGTGGCGATTTGTGACCCTCTGCATTACCCCACCAGAATGACTGTGGCGAGAGTCAAATTTAGTGTTTGTCTGTGTTGGTTTTATGCTGTTTTCTACTGCAGTCTCTATA
TGAAGGATATGCTAGTTGAACCAGGCAGGTATAATTCCTGCTATGGAGAGTGTTTGTTTGTCATTAATGATATTGCAGGGATTTTTGACATAGTTTTCTCTTTTTTTGCTCC
AGTTATTATCATCACAGTTGAGTGTTTGTGGTGGCTGTGTCTCAGGCTCGAGCCATGCGGTCTCATGTCACTGAATCAAGCAAACAAATCGGAGCTGAAAGCAGCCAG
GACCCTTGGGATTCCTGTAGTTGTGTTTCTCTCCTCTCTTTGA
>contig060903-BurTARp.A009
ATGATGGAGATACAGAAAGGAGTCGAGCTCTGTTTTCCAAAACTCCTCAACACTTCCTGCAGGAAGCCGGCACATCACTGGTCCAAAGCTGTGCTCCTGAACATTGTG
CTGTCATGTGTCTCTCTGATCACTGCTGCTCTAAACCTTCTGGTCATCATCTCAGTCTCTCACTTCAGGAAGCTTCACACACCTACCAACATCCTCCTCCTCTCTCTGGC
TGTCTCAGACTTTCTTGTGGGTCTCTTGCTGATGCCATTTGACATCTTTAGGAACATGGCCTGCTGGGTACTTGGTGATCCTATGTGTTCTGTTTATTGGTATCTGACTG
GCCTGACTGGCAACATTGCCTTTGCCTCAACAGGGAACATAGTTCTAATATCAATTGACCATTATGTGGCTTTTTGTCCCCCTCTGCATTACCCCACCAGAATTACTGTG
GCAAGAGTCAAACTTAGTGTTTCTCTGTGTTGGTTATTTGCCATTTTCTACAGCAGTCTCTATATGAAGGATGTACTGATAAAGAGGTATAATTCCTGCTATGGAGAGTGT
GTGTTTATCATTGATGATATTGCAGGGATTTTTGACATGGTTTTATCTTTTTTTGTTCCAGTTCCAGTTATCGTAATTCTGTATATGAGAGTGTTTGTAGTGCCTGTGTCTCA
GGCTCGAGCCATGCGCTCTCATGTTACAGCTGTCACACTGCAGCGTTCATTGAATCACACAAACAAATCTGAGTTGAAAGCAGCTGGGACTCTGTGAATTCTTGTAGTT
GTGTTTTTAATATGCTACTGTCCATGTCCATTCTTCTGCTTTTCTTTTGGTGTAGAAGACATGCTACATTGGCTAAACCCAGAGATTGCCTGA
>contig057148-BurTARp.A010
ATGGAGATACACGAAGGACTAGAGCTCTGTTTTCCACAACTCCTCAACGATTCCTGCAGAAAGCCAACACTTGACTATTCCACAACTATGCTGCTGTATATTGTGATGTC
ATGCATCTCTCTGATCACTGCTGCTCTAAACCTTCTGGCCATCATCTCAGTCTCCCACTTCAGGCAGCTTCGCACACCCAGCAACATACTCCTCCTCTCACTGGCTGTT
TCAGACTTTCTCGTGGGTCTCCTGTTGATGCCGTTACGAATTTTAAAAAGCACCACCTGCTGGATACTTGGTGATTTTAATTGTTTTCTTTATTGGTGTCTGACTGGCAAC
ATTACCTGTGCTTCAATAGGGAACATAGTTCTAATATCAGTCGACCGCTATGTGGCTATTTGTGACCCTTTACATTACTCCACCAGAATTACTGTGGCAAAAGTTAAACTCA
GTGTTTGTCTGTGTTGGTTTTATGCTATTTCCAGCATTAGTCTTTATGCAAAAGACTACCTGATTGAACCAGGCAGGTATAATTCCTGTTATGGAGAGTGTGTCATTAATGA
TATTGCAAAGACTATTGACCTTGTTTTATCCTTCATATTTCCAATTACTGTCATCATAGTTCTGTATATGAGAGTATTTGTGGTGGCTGTGTCTCAGGCTCATGCCATGCGC
TCTCAGGTTACAGCTGTCACAATTCAGTGTTCTCTGAATCAAGAAAACAAATCAGAGTTGAAAGCAGCCAGGACACTTGGAGTTCTTGTAGTTGTGTTTCTACTGTGTTT
CTGCCCATATTACTGTGTCTCTCTTATAAGGGAGGAC
>contig049535-BurTARp.A011
ATGATGGAGAACAAGGACAGTATATGCTTTCCAGAAGTCTTCAACTCTTCCTGCAGGAGACCAGCATTTCACTGGTCCGAAGCTGTGCCCCTGAATACCGTGCTGTGC
TCCATCTCTGTAATCACCATAGCGCTGAACCTCCTTGTCATCATCTCAGTCTCCTACTTCAGGCAGATTCTCCACACACCCACTAACATCCTCCTCCTCTCTTTGGCTGT
GTCAGACGTTCTTGTGGGGCTCCTGTTGCTGCCTGGAGAAATCTTCCTAGGAATAGCCTGCTGGGCTTTCGGTAACCTTTTGTGTTCTCTCTTTAATTATGTGTCTTTCA
TCATTACCTCGGCTTCAGTGGGAAACATGGTTCTCATATCAGTTGACCGCTATGTGGCGATCTGTTATCCTCTGCATTACTCTACCAGAATTACTGTGACAAGAGTTAAAC
GTTCTGTTTGTTTGTGTTGGCTCTGTTGTGTTTTGTATAGTAGTGTCCTTTTAAAAGATGAGCTGATTCAACCAGGAAGGCATAATTCCTGCTATGGAGAATGTGTTTTTG
TCATCAACTTTATTGCAGGAACTGTTGATCTTTTGTTAACCTTTATTGTTCCTATTTCTGTTATTGTAGTTCTGTATATAAGAGTATTTGCAGTGGCTGTGTCTCAGGCCCGT
GCCATGCGCTCTCAGCTTTCAGTGAATCTGACAACAAAGAAATCTGAGTTGAAAGCAGGAAGAACTCTTGGTGTTCTAATAGTTGTGTTTCTAATGTGTTTCTGTCAATA
CTATTGCGTCTCTCTTGCAGGGGAAGACTCACTCAGTGAGTCATCCACATCAATTGTCCGTTATCTCTTCTATTTTAACTCCTGTTTGAACCCTCTGATCTATGCACTATT
TTACCCCTGGTTTAGAAAAGCTATTAAACACATTGTCATGCTTCAGATATTTCACAGTGGCTCCCATGAGGACAACATACTA
>contig045302-BurTAR.A001
ATGGATAGCTCTGGAGGTCCTCCCCTCTGCTTCCCCAACCTCAACTCCTCCTGCAGGCGGCTGCTGCGACCCACCTCCCAGACCGTTGTTCTCTACACTCTACTGGC
CTCCATCTCCCTGCTCACTGTGGTGCTCAACCTGCTTGTCGTCATCTCCATCTCCCACTTCAGACAGCTTAACACCTCGACCAACACCCTGCTCCTTTCCCTGGCTGT
GTCTGACCTGCTGGTGGGGCTGCTGGTGATGCCCATCGAGGGCCTGTACTACATCGAAACGTGCTGGCTGCTGGGGAGGCTGATGTGTGCTCTCAGTCCTTATTTGT
CTTACTGCCTCATCTCTGTCTCTCTGGACAGCATGGTGCTCATATCTGTAGATCGCTATATAGCCATCTGTGACCCTCTGCTCTATTCCTCAAAGATCACAGTGAACAGA
GTGAAGCTTTCAGTCTGTGTCTGCTGGGCCTGCTCTCTTCTCTACAATGGCTGCATTCTCATGGAGCACATAGGGTGGCCAGACAGGTTCAGCTCCTGTCACGGGGA
GTGTGTTGTGTTCATCAGCCGTGCTTTGGGTACAATAGATCTCTTTTTCTCATTTCTCGGCCCATGTGCTTTAATGTTTGTTCTGTACATGAGGGTCTTTGTGGTTGCTGT
TTCTCAGGTACGTATCATTCGGTCGCAGGCAGCTGTGAGAGCACCTCCAGCTGCTAAAAAATCAGAGCTGAAGGCAGCCAGGACACTCGGGATTTTGATAGCTGTGTT
TCTAATGTGCTTCTGCCCTTATTATTATCCTTCCTTTGCAGGTGACGACACCTCTATGAACTTACCTTATTACGCTCTATTCTCGTGGATAATGCTGACAAATTCTTGTGTG
AACCCTGTGATTTATGCTCTGTTCTACCCCTGGTTTAGAAGAGCTATCAGACTCATTGTCACCCTCAGAATACTGCAGCCTCACTCCAGAGATGTCAAGATCCTGtag
>contig020038-BurTAR.A002
ATGGACAGGCAGGACAGAATTGAACTCTGCTACCCTGAGCTTGCGAATCTGTCTTGCAGGGGTTTAACACGTCCTCAGCCTGAGGCTTTCCTCCTCTATACACTGTTC
TTGTCTATCTCTGTGCTAACTGTAGCTCTGAATGTGCTGGTTATCATATCCATCTCTCATTTCAGGCAGCTCCACATGCCCACCAATGTCCTCCTGCTCTCTCTGGCCAT
CTCAGACCTCTTGGTCGGGCTGCTTGTGATGCCGGTGGAAGCAATGCGGTTTATAGAAACCTGCTGGATGCTGGGAGATCTCATGTGTGCTTTTACTTACATTATCGGT
TTCACTCTCACCTCAGCCTCTGTGGGCAACATGGTGCTCATATCAATTGATCGCTACGTAGCTATTTGTTATCCTCTGCAGTACCCCACTAAAATCACTCACAGCAGAGC
GGAGCTGTCTGTGACTCTGTGCTGGGCCTGTTCGCTCCTCTACAACGGGATGATCTTAAAGGAGCATCTCAGGCAGCCAGACAGGCATAACACCTGTCACGGTCAGT
GTTTGGTGGTGATTAACTATGTCTCTGGAGCCATCGACCTTGTCTTTACTTTCATCGGCCCTTGCTCAGTCATTATCATTCTGTACATGAGAGTATTTGTTGTAGCGCTGT
CTCAGGCTCATGCCATGCGGTCTCATATTACAGCTGCTGCAGCTGGTACAGTAAAAATCACCGCAAAGAAATCAGAGAAAAAGGCAGCCAGGACTCTTGGCGTTGTGA
TATTTGTGTTTTTGATGAGTTTCTGTCCGTATTACTATCCCTCTCTTGCAGGGCAGGACATTTCAAACAGTGCTTCATCTTGGGCTATTGTTTCCTGGATGCTGTACTTCA
ATTCTTGTCTGAATCCACTGATATATGCTTTTTTCTACCCGTGGTTTAGAAAAGCTATCAGGTTCATTGTCTCCCTGAAGATACTAGAGAAGGGCTCCTCTCAGGCAAATA
TACTTtaa
>contig006087-BurTAR.B032
ATGGAGTTATTCAATGTGACTGTTAATACTGTGAGCTTCCTTCTCTGCGATTCACAAAAGAACAAATTATGTGTGTTATTATATGTTGTCCTCAGCTCATTTATGCTTCTTAC
AATATGTGGAAATCTTCTTGTGATAATTTCTATAATTTACTTTAGATATCTCCACACTCCTACAAACTACCTTATCCTCTCTATGGCTGTGGCTGATCTACTAATTGGTGCTTT
AATATTTCCTTTGAGCATGACAGTGTCTCTAAAGCCATGTTTGTATATATACAGTTTACTGTGCAACTTAAGAAGCACAATGGATGTAACAATGGGTGTATCTTCCTTATTAA
ATTTGTGCTGTATTTCTGTTGATCGATATTATGCTGTTTGCCACCCTCTGATATATAAAACTAAAATAACTGATTGTGTTGCCATGAAGATGGGCCTTGGAAGTTGGGCTGT
TGCTATCTTGTGTGGAATTTTTGTCTTCCTGCTGTTTTTTATTTTAGACGAATGTGACACAAGTTGCCTTTTCGCTCTCATTGCCGCATCAGTGGTAGTATATTATATCCCA
ACAATAGTTTTACTTTTCATGTACACCAAAATTCTTGTTGTTGCACTGAGGCAGGCACGCAGCATCCATAATACAATTTCTCAGAACACAAAGTCTAAAGCAGTTTCCAGT

ACGGAGAGAAAGGCCACAAAGACATTAACCATAGTTATTGGAATATTTTTAATTTTTTGGGTTCCTCTTTTTCTTAGTTATTCATTTGTTCCTCTGGATAGTTTTATACTTTAT
GTGCTTCTTGAACCATTTAACTGGTTTGCAATTTCTAACTCAATGCTCAATCCCTTTATTTATGCTTTCTTTTACACTTGGTTTAGAAGAGCTTTTAAAATGATAATTTCTGG
AAAAATATTTCAAGGTGATGTTACTAACATAAAACTACACtga
>contig061977-BurTARs.A012
ATGGATACACAGGATGTGGCAGAGCTCTGTTTTCCACAACTCTTCAACACCTCCTGCAAGAAACCTCTGTCAGAATTTGTGTTCCTTCATGTTGTGTTGTCCTCCATCTC
GCTGCTAACTGTGACTCTCAACCTGCTCGTCATCATCTCAGTCTCCCACTACAGGCAGCTCCACACACCCACTAACATCCTCCTCCTCTCTCTGGCTGTCTCAGACTTT
CTTGTTGGTCTCCTATTGATGCCTGGAGAAATCCTCCGAAATACAGCCTGCTGGTTTCTCGGTGACCTCACCTGTTCTATGTACAATTATATGTCTTTAATTGTTACCTCTA
CCTCAGTGGGAGACATGGTGTTAATATCAATTGACCGCTATGTGGCTATTTGTGACCCTCTGCATTACCCCACCAGAATCACAGACAGAAGAGTGAAACTCTGTGTTTG
TCTGTGTTGGCTCTGCTCTGTTTTCTATAGCAGCCTGTTTGTAAAGGATGATCTAACTCAACCAGGGAAGCATAATTCCTGCTATGGAGAATGTACAATTGTTGTTGACTT
AATTACAGGAACGATTGACCTTCTTTTAACCTTTTTTGTTCCAGTTACTGTCATTGTAGTTCTGTATCTGAGAGTATTTGTGGTGGCTGTGTCTCAGGCTCGCGCCATGC
GCTCTCATGTTACAGTTGCTGCTCTGCAGCTTTCAGTGACTCTAACAACAAAGAAATCAGAGTTAAAAGCAGCCAGGACTCTGGGTGTTCTTGTAGTTGTGTTTCTACT
GTGTTTCTGCCCTTATTATTGTGTTACTCTTGCCAGGGACGACCTACTCAATAGTTCATCTGTATCCTTTTTGCTCTATCTGTTCTATTTTAACTCATGTCTAAACCCTTTGA
TCTATGCACTGCTCTACCCCTGGTTTAGAAAAGCTGTGAAACTCATAATCTCTTTACACATACTGCAGCCTGGTTCCTGTGAGATTAGCATTCTGtaa
>contig061433-BurTARs.A013
ATGGAGATCCAGATGCATCCAGAGGCAGAGCTCTGTTTTCCAGAGCTACCGAACAGTTCCTGCAGGAAGCCGACACTTCACTGGTCCAAAACGGTGCTCCTGAACGT
TGGGCTTTCGTCCATCTCTGTGATCACTGCTGCTCTTAATCTGCTCATCATCATCTCAGTCTACCACTTCAGGCAGCTCCACACTCCCAGTAACATCCTCCTCCTCTCTC
TGGCTGTCTCTGACTTTTTTGTGGGTTTTCTGTTGCTGCCGGTAGAAATCTTCAAAAACACGGCCTGCTGGGTATTTGGTGATCTCATGTGTTCACTTTATATTTATCTGA
GCGGCATTCTTATGAATGCTTCAATTGAAATTATTGTTTTTATATCAGTTGACCGCTATGTGGCTATTTGTGACCCTCTGCATTACCCCACCAGAATCACTGTGACACGAG
TCAAACTCAGTGTTTGTCTGTGTTGGTTTTATGCTATTTTCTACATGAGTCTTTACACAAAGGATGTCTTGATAAAGCCAGGCAGGTATGCTTCCTGCTATGGAGAGTGTG
TGTTTTTTATTGAAGATATAACTGGAACTGTTGACATTGTTTTATGTTTTTTTGTTCCAGTTATTATCATCATTGTTCTGTATACAAGAGTATTTGTAGTGGCAGTGTCTCAGG
CCCGTGCCATGCGCTCTCATGTTACAGCTGTCACACTTCAGCGTTCATTGAATCAATCAAACAAATCTGAGCTGAAAGCAGCCAGGAACCTTGGAGTTCTTGTAATTGT
GTTCCTGGCAAGTATTTGCCCATTTTACTTCTACTCTCTTGTTGACGGCAATGTGGTCAAAGCTTCATCTGCGACTTTTTTGATCATTGTTTATTTTAACGCTTGTTTAAAC
CCACTGATCTATACCCTGTTTTACCCCTGGTTTAGAAATGCTGTTAAACTTATTATCACCCTGCAGATATTCAAGCATAACAGTAGTGAAGCCAACATACTAtag
>contig061091-BurTARs.A014
ATGGAGATACCGAAAGGAGTCGAGCTCTGTTTCCCACAACTCCTCAACAGTTCCTGCAGGAAGCCGACACTTCACTGGTCCAAAGCTGTGCTCCTGAACATTGTGCT
CTCATGTATCTCTCTGCTCACTGCTGCTCTAAACCTTCTCGTCATCATCTCAGTCTCCCACTTCAGGCAGCTGCACACACCCAGTAACATCCTCCTCCTCTCTCTGGCC
GTCTCAGACTTTTTTGTGGGTCTCCTGTTGTTGCCTTTAGAAATTTTTAGAAACACAAGCTGCTGGGTACTTGGTGATCGTATGTGTTCTGCTTATTGGTATTTGACCAGC
AACATTATCTGTGCTTCAATAGGGAACATTGTTCTAATATCAGTTGACCGCTATGTGGCTATTTGTGACCCTCTGCATTACCCCAGCAGAATTACTTTGGCGAAAGTCAAA
CTCAGTGTTTGTCTCTGTTGGTTTTATGCTTTTTTCTACAGCAATCTTTATACAAAGAATATCATGATTGAACCAGGCAGGTATAATTCTTGCTATGGAGAGTGTGTATTTTT
TAGCAGCAATATTGCTATTGTTGCTGACCTTATTTTATTCTTTTTTGTTCCAGTAACTGTTATCATAGTCTTGTATATGAGAGTATTTGTGGTGGCTGTGTCTCAAGCTCGTG
CCATGCGCTCTCATGTTACATTGGTCACACTTCAGCGTTCACTGAATCAAACAAACAAATCTGAGCTGAAAGCAGCCAGGACTCTGGGGATTCTTGTAGTTGTGTTTCT
AGCATGCTTCTCTCCACTCTACTGCTACTCTCTTGTTGATGAAAATGCAATCAATAATCCAGCTGCATCTTTTGCGGTCATTATCTTTTACATTAACTCTTGTCTAAACCCT
TTGATCTATGCCTTGTTTTACCCCTGGTTTAGAAATGCTGTTAAACTTATCATCACGTTGGAGATCTTCAAGTATGACACAAGTGGTGCCAACATACTGtaa
>contig060707-BurTARs.A015
ATGGAAGAAACTGAACTCTGCTTTCCACAACTATTCAACTCCTCTTGTGTGCGGCAAAAGCATTCACAAATTGAGGCTGTGTTTATTTACACTCCACTCTCTTCCATCTC
TGTGCTTACTACAATTCTGAACCTGCTTGTGATCATCTCCATTGCTCACTTCAAGCAGCTGCACACCCCAACCAACCTCCTCCTCCTCTCTCTGGCCGTCTCAGATTTC
TTCGTGGGCCTCATCATGGCTTGTCAGATTAGCCTCCTAGATGGCTGCTGGTTTCTTGGTGACCACATGTGTGCTCTGTATAGCAGTTTAGATTACATTGTTACTTCTGC
TTCAGTAGGAACTATGGTACTCATATCAGCTGACCGCTATGTAGCCATTTGTGACCCTCTGCATTATCCCACCAAAATTACTATGAAAAGAGTCTCAGTCTCTATTTGTACT
TGCTGGGCTTGTTCAATTCTGTATAACAGTCTTATCATGAAGGATAATTTCAAGCAGCCAGGGAGGTATAATTCTTGTTCTGGTGATTGTGTAGTTGTCATTGATTACTTTG
TGGGAATTTTTGACTTTGTTTTGACCTTTGTCGGCCCTGTCATTGTCATCATAGTTCTGTATCTAAGAGTATTTGTGGTGGCTGTGTCTCAGGCTCGGGCAATGCGCTCT
CATATCACTGCTCTCAGACTCCAGGGGTCAGAGACGGTGCATGCTAAGAAATCTGAGCTGAAAGCTGCCAGGACTCTCGGTGTACTTGTTATTGCCTTTCTGATATGTC
TTTTCCCATTTTTCTGTTCCTCAATGGTAGGCCAGAATAGTTTCTTTGATATTAGATCTGTACCTTTTGAGCTCTTGCTGTTCTATTTTAACTCTTGTCTGAACCCATTAATC
TACACTTTTTGCTACCCCTGGTTTCTGAAATCTATCAAGCTCATTGTGACATTTAAGATATTTAGGCATGGCTCCAGTGAGGCCAGTATACTAtag
>contig059673-BurTARs.A016
ATGAAAACCTTTGAAGAAGCTGAACTCTGCTTTCCACAACTCCTCAACTCCTCCTGCAGGAAGACCATGCGTCCTTATACATTTTCTATCCTTATTTATATTACCCTGTCC
TCCATTTCTTTGCTCACGAAGCTGCACACCCCCACCAATCTCCTCCTCCTATCTTTGGCTGTCTCTGATTGCCTGGTGGGTCTCCTGATTTTATTTCAAATTATGATTATA
GATGGTTGCTGGTTTCTTGGTGAATTCATGTGTAGTATGTATTTTCTCTTGGACTACATTATTACCTCTGCATCAATAGGAACCATGGTTCTCATATCAATTGACCGTTATGT
GGCTATTTGTTACCCTCTCCATTACTCTACCAAAGTCACCCCAAAAAGAACAAAAGCATGTGTTTATCTCTGTTGGATTTGTTCTTCAGTCTTCCAATGTCTCCTGCTAAA
AGACAACCTGGTGCAACCTGGCAGGTATAATTCCTGCTATGGGGAGTGTGTGGTTGTTGTTGGCCATGCTTTTGGAGTTGCAGATCTATTGCTGTCCATTATTGGTCCT
GTCACTGTGATTGTAGTTCTTTATCTAAACGTATTTGTTGTGGCTATGACTCAAGCTCGTGCCCTGCGGTCACATATTGCAGCTCTGACACACGAGAGGTCAGTAAGTAC
AAATGTTAAGAAATCTGAAATGAAAGCAGTAAGGACCATCAGTGTTCTTATCATTGTGTTTCTAATATGTCTCTGCCCATACTTTGGTGTCACACTCTCAGGTCAAGATGC
CATGCTCAGTGCTTCATCTGTTGCATTTGTAATGTGTTTGTTCTATTTAAACTCCTGTCTAAACCCTCTAATCTATGCTTTATTTTACCCCTGGTTCAGAAAATCTGTTAAAC
AAATTGTTACACTTAAAATATTGAAGTCTGGCTCCTGTGACACCAATATCATGtaa
>contig057301-BurTARs.A017
ATGGAGGGGGTTGAATTCTGCTTTCCACATCTGCTCAACTCCTCTTGCAGAAAGGCTCTGCATCCTGTCTCAGTTTCCACGCTCATTTACATGATAATATCTTCTATCTCT
GTGCTCACTGCAACTCTTAACCTGCTGGTCATCATCTCCATCTCCCACTTCAAGCAGCTCCATAATCCCACCAACTTCCTCCTCCTCTCTCTGGCTGTCTCCGATTTCTT
TGTGGGTCTCTATTTGCTCTTTTACATAATGTTTATAGACGGCTGCTGGTATTTTGGTGAATTCATGTGTATTCTGTATTATGTTATTGGCACAATTAACACCTCTTCCTCAA
TAGGAACCATGGTACTGATATCAGTTGACCGTTATGTGGCCATTTGTGATCCTCTGCATTATCCCATCAAAGTCACTGCAAAAAGAGTTCAGATCTGTGTTTCACTGTGTT
GGAGCTTTTCTGCCCTTGCTGGTAGTTTCCTCTTAAAGGACAACTTGAAACAGCAAAGCAGGTTTAATTCTTGTGTGGGAGAGTGTGTTGTCCATATTAACTTTATTGAA
TATGTTGCAGATCTTGCTTTGAACTTCCTACTTCCTATTACTGTCATCATAGTTCTGTATTTGAGAATATTTGTAGTGGTTGTGTCTCAGGTTCGGGCCATGCGGACTCATA
CTGCAGGTGTCACATACCAGTGTTCAAGGAAGGGAAATCCAAAGAAATCAGAGATGAAAGCAGCCAGGACTCTTGGTATTGTTGTAATTGCATTCTTGTCATGCGCCTT
ACCATTTTATTGTGTCACACTCACAGGCCAGAATGCCTTCCTCAACGGATCATCTTCTGCATTTGTTTTGTGTCTTTTCTATTTTAATTCGTGCCTCAACCCTATCATCTAT
GCCTTATTCTACCCCTGGTTTAGAAAATCTATTAAGCTTATTGTTACATTTCAAATACTGAAGTCTGGCTCCAGGAATGCCAACATAGTCAAAGTGACAtaa
>contig057145-BurTARs.A018
ATGGAGATACAGAAAGTGCCCGAGCTTTGTTTTCCACAATTTCTCAACAATTCCTGCAGAAAGCCGACACTTCATTGGTCGAAAGCCGTGCTCCTGAACATTGTGTTCT
CCTGTATCTCTCTGCTCACTGCTGCTCTAAACCTTCTCGTCATCATTTCAGTCTCCCACTTCAGGAAACTTCACACACCAAGTAACATCGTCCTCCTCTCTCTGGCTGTC
TCAGACTTTCTTGTGGGTCTTCTGTTGATGCCAGCAGAAATATTAAGAAGCACGACGTGCTGGGTACTTGGTGACATTATGTGTTCTGTTTATTGGTATCTGACCGGCAA
CATTATCTGTGCTTCAATAGGGAACATAGTTCTAATATCAGTTGATCGTTATGTGGCTATTTGTGACCCCTTGCATTACCGCACCAGAATTACTGTGAAACGAACCAAACT
CAGTGTGTGCTTATGTTGGTTTTATGCTATTTTCTACTGGAGTCTTTATATAAAGAATATCCTGATTGAACCGGGCAGGTATAATTCCTGCTACGGAGAGTGTATGTTTGTC
AGCAGTGATATTGCAGGGATTATTGACCTTTTTTTATCTTTTATTGTTCCACTTTCTGTCATCATAGTTCTTTATATGAGAGTATTTGTGGCGGCTGTGTCTCAAGCTCGTG
CCATGCACTCTCATGTTACATCTGTCACACTTCAGCATTCAAGCAATCAAGCAAAGAAATCTGAACTGAAAGCAGCCAGGACTCTGGGGGTTCTTGTAGTTGTGTTTCT
GTCATGTTACTCTCCATTTTACTGCTATTATCTCGCTGAAGAAAATGTGGTCAATGATCCATCTGCATCTACTGTTATCATTATTTTTTACATTAACTCTTGTCTAAACCCTTT
GATCTATGCCTTGTTTTACCCCTGGTTTAGAAATGCTGTTAAACTTATTGTCACTCTGCAGATCTTCAAGCATGGCACCTGTGAGGCCAACATACTAtaa
>contig056023-BurTARs.A019
ATGGAGATTCAGATGCATCCAGAGGCAGGGCTCTGTTTTCCAGAGCTACTGAACAGTTCCTGCAGGAAGCCGACACTTCACTGGTCCAAAACGGTGCTCCTGAACGT
TGGGCTTTCGTCCATCTCTCTGATCACTGCTGCTCTTAATCTGTTCATCATCATCTCAGTCTCCCACTTCAGGCAGCTCCACACTCCCAGTAACATCATCATCCTCTCTC
TGGCTGTCTCTGACTTTTTTGTCTGTTTTTTGTTGATGCCGGTAGAAATCTTCAAAAACACGGCCTGCTGGGTATTTGGTGATCTCATGTGTTCACTTTATACTTATCTGA
GCTGCATTCTTATCAATGCTTCATTTGAAATGATTATTCTCGTATCAGTTGACCGCTATGTGGCTATTTGTGACCCTCTGCATTACCCCACCAGAATCACTGTGCCGAGAG
TCAAACTCAGTGTTTGTCTGTGCTGGCTTTATGCTATTTTGTACAACATCATCTATACAAAGCATGCCCTGATAAACCCAGGCAGGTATGGTTCCTGCTATGGAGAGTGT
GTTTTTGTTGTTGATGATATTATTGGAATTGTTGACTTTGTTGTATCTCTTATAGTTCCAGTTACGATCATCGTAGTTCTGTATACGAGAGTGTTTGTGGTGGTTGTGTCTC
AGGCTCGTGCCATGCGCTCTCATGTTACAGCTGTCACACTTCAGCGGCCACTGAATCAAGCAAACAAATCTGAGCTGAAAGCAGCCAGGAACCTTGGGATTCTTGTAG
TTGTGTTTCTGGCGTGCTACTGTCCATTTTACTGCTACTTTTTTCTTGCAGGCAATGAAGTTAATGCTTCTTCTGCATCTTCTATCCTCATTGTTTATTATTTTAACTCTTGC
CTAAACCCATTAATGTATGCACTGTTTTACTCCTGGTTTAGAAATGCTGTTAAACTTATCATCACTCTGCAGATATTAAAGGCTAACAGCAGTGAGATCAACATACTAcagag
ataa
>contig056021-BurTARs.A020
ATGGAGATACAGAAAGGAGCTGAGCTCTGTTTTCCACAACTCCTCAACATTTCCTGCAGGAAGCCGACGCTTCACTGGTCCAAAGCTGTGCTCCTGAACATTGTCCTG
TCCTGCATCTCTCTGCTCACTGCTGCTCTAAACCTTCTCGTGATCATCTCAGTCTCCTACTTCAGGAAGCTTCACACACCCAGCAACATCCTCCTACTCTCTCTTGCTGT
CTCAGACTTTCTCATGGGTCTCTTGCTGATGCCAGCAGAAATCCTTAGAAGCATGACGTGCTGGGTACTTGGTGATCTTATGTGTTCTGTTTATTTTTTTCTGACTGTAAA
CCTTACCTGTGCTTCAATAGGGAACATAGTTCTTATATCGATTGACCGTTATGTGGCTATTTGTGACCCTCTGCATTACTCCACAAGAATTACTGTGGCAAGAGTCAAACT
CAGTGTTTGTCTATGTTGGTTTTATTCAACTTTCTACTGCAGTCTTTATACGCAAGAAATGCTGATTGAACCAGGCAGGTATAATTCCTGTTATGGTGAGTGTGTGCTTGT
CATCAGTGATTTTGCTGGAATGGTTGACCTTATTTTATTTTTTATTCTTCCAGTTATTATCATCATAGTTCTGTATACAAGAGTATTTGTGGTGGCTGTGTCTCAGGCTCGTG
CCATGCGCTCTCATGTTACGGCTGTCACACTTCAGCGTTCATTGAATCAAACAAACAAATCTGAGCTAAAAGCAGCCAGGACTCTGGGGGTTCTTGTAGTTGTGTTTCT
GTCATGCTACTGTCCATTTTATTGCTACTCTCTCACTGATAAAAATGCTGTCAATGATCCAGCTGCATCTTTTGTGATCTTTATCTTTTATTTTAACTCTTGTATAAACCCTTT
GATGTATGCCCTGTTTTACCCCTGGTTTAGAAATGCTGTTAAACTTATTATCACATTGCAGATATTCAAGCATAACTCCTGTGAGGCCAACATACTAtaa
>contig056020-BurTARs.A021
ATGGAGATACAGAAAGAAAACAAGTTGTGTTTTCCACAACTCCTCAACAGCTCCTGCAGGAAGCCAACACTTCACTGGTCCAAAGATGTGCTCCTGAACACTGTGCTG
TCCTGCATCTCTCTGCTCACCGCTGCTCTAAACCTTCTCGTCATCATCTCAGTCTCTTACTTCAGGAAGCTTCACACACCCAGTAATATCCTCCTCCTTTCTCTGGCTGT
CTCAGACTTTCTTGTCGGTCTCCTGTTGATGCCATTAGGAATCCTTAGAAACACAGGCTGCTGGGTACTTGGTGATATAATTTGTTCTCTTTATTGGTATTTAACCAGCAA
CATTGTCTGTGCTTCAATAGGGAACATTGTCCTAATATCAGTTGACCGCTATGTGGCTATTTGTGACCCTCTGCATTATCCCAGCAGAATTACTTTGGCGAAAGTCAAACT
CAGTGTTTGTCTGTGTTGGTTTTATGCTTTTTTCTACGTCAGTCTTTATACAAAGGATATCCTAATTGAACCAGGCAGGGATAATTCCTGCTACGGAGAGTGTGCATTTGT

CATCAATGATATTGCTGTTGTTGTTGACCTTGTTTTTTCCTTTATTGTTCCGGTTTCTGTCATTGTAGTTCTGTATATCAGAGTATTTGTGGCAGCTGTGTCTCAGGCTCGT
GCCATGCGCTCTCATGTGACATCTGTCACACTGCAGCGCTCACTGAATCAAACAAACAAATCTGAGCTGAAAGCAGCCAGGACTCTTGGAATTCTTGTAGTTGTGTTTC
TGGCATGCTTCTGTCCACTCTACTACTTTTCTCTTGTTGATGAAAATGCAATCAATGATCCAGCTGCATCTTTTGTGGTCATTATATTTACTTTAACTCGTGTCTAAATCCT
TTGATCTATGCCTTCTTTTACCCCTGGTTTAGAAATGCTGTTAAGCTTATCATCACTCTGCAGATTTTCAAGTATAATatgagtgaggccaacatactataa
>contig055697-BurTARs.A022
ATGATGGAGGAACCTGAACTCTGCTTTCCAAAACTCCTTAATATCTCTTGCAGCAGGCCAAAGCGTCCTCACTTTGAGATCATGCTGACTTACATTCTGCTCTCCTTCAT
TTCTTTGCTTACTGTGATTCTTAACCTGCTGGTCATTATCTCCATCTCACACTTCAGGCAGCTCCACACCCCCACCAACTTCCTCCTTCTCTCTCTGGCTGTCGCTGATT
TCTTTGTAGGTCTCCTTATGTTCTTTCAAATAGTGCTCATCGATGGATGCTGGTTCCTCGGTGACATCATGTGCACTCTGTATCAGTATCTAGCATTCATTATTACTTCAGC
CTCAGTAGGAACCATGGTGATCATATCTGCTGATAGGTATTTGGCTATTTGTTACCCTCTGCATTACTCCACCCAAATAACACAACAAAGAGTTAATATATGTATAAGTTTGT
GTTGGTTTTTTTCTGTGATCTTTCAGAGTTTGATTGTGAAGGATAACTTGAAACAACCAGGAAAGTATAACTCCTGCATTGGAGAGTGTGTCTTTGTCGTTAACTACATTG
CTGGGCTTTTTGATCTTTTGTTTTCCTTCATTGTTCCCATTACTGTGATTGTAGTTCTGTATCTGAGAGTGTTTGTGGTGGCTGTGTCTCAGGCTCGTGCCATGAGGTGT
CAACTTGCAGTCACTCACCAGCGATCAGTAACAGTAACTGTTACGAAATCGGAGCTGAAAGCAGCCCGTACTCTTGGTGTTGTTGTAGTTGTGTTTCTTATATGTATGTG
CCCATATTACTGCGTTGCTCTCACAGGCCAAGATAACTTCCTAAATGCTTCATCTGCTGCCTTTGTAATTTGTTTGGTGTACTTTAACTCTTGCCTAAACCCTATCATTTAT
GTCTTTTTTTATCCCTGGTTCAGAAAGTCAATCAAACTTATTGCTACTCTTCAAATACTGCAGCCTGACTCCCGTGAGACTAACATGcattaa
>contig054630-BurTARs.A023
ATGGAGAACGTGTATGGACAACAATTCTGCTTTCCACAGCTCCCCAACACCTCCTGTAGGAAGCCGCTGCAACATCACAATCAGACCATTTTTCTCTACATTCTGCTTT
GCTGCATCTCTCTGCTCACAGTGACTCTCAACGTCCTGGTCATCATCTCTATCTCCCACTTCAGGCAGCTTCACAACCAAACCAACCTCTTCCTCCTCTCTCTCGCTGT
CTCAGACCTCCTTGTTGGCCTCCTGCTGATGCCGCCTCGAATCCTCCTTTTAGGGGGCTGCTGGTTTTTGGGCACCTTCATGTGTGGACTGTTTTACTATGCCTCTTTT
GTTCTCACATCTGCCTCAGTAGGAAACATGGTGCTTATTTCATTTGACCGGTATGTAGCTATTTGTGACCCCTTGTCCTACCCCACTGCAGTTACTGAGAGAAAAGTCCA
AATCAGTGTCTGTCTGTGTTGGGCCTGTTCACTGCTGTATAACGGCACAATACTAAACAACTTTCTGAAGCAGCCAGACAGATATAATTCTTGTGACGGGGAATGCATAG
TTGTAATTAATTTCATAACTGGTGCTTTTGATGTCGTAGCTACCTTCATCGGCCCCACTGCTGTGATTATATTTCTGTACATGAGAGTATTTCTGGTGGCTGTGTCTCAGG
CTCAGGCTATGAGATCTCATGTTGCCTTTGTTACTTCAAAGGGTTCAGTTCACGTTGCCATTAAGAAGTCTGAGAGAAAAGCAGCTACAACTATTGGTGTTGTTGTGGCT
GTGTTTCTGATGTGTTTCTGTCCTTACTTTTATCCCTCTCTCGCAGGGCAGGACACATCAACCAGTGTGGAATTTTCAGTTTTTGGGGTCTGGTTACTTTATTGTAATTCC
TGTCTAAATCCACTTATCTATGCGTTTTTCTATCCCTGGTTTAGGAAGACTGTTAAACTGATAGTTACATTGCAGATTTTGCAGCCTGACTCTTGTGATGCCAACATACTGta
g
>contig049540-BurTARs.A024
ATGGGCACACAGGAAAAATCTGAGCTCTGCTTTCCACAACTCTTCAACATGTCCTGCAAAAAGCCTGCCATTGCTCGGTCCAAAGCTGTGTTCCTTCATATTGTATTGT
CCTCAGTTTCTTTTCTGACGGTGGCTCTCAACTTGCTTGTCATCATCTCAGTCTCCCACTTCAGGCAGCTTCACACACCCACTAACATCCTGCTCCTCTCTCTGGCTGT
CTCAGACTTTCTTGTTGGCCTCCTATTGTCACCTGCAGAAATCCTCCGAAGTACAGCCTGTTGGTTTCTTGGTCAGCCCACATGTTTAATGTATATTTTTGTGTCCTTGAC
TGTTACCTCTGCCTCAGTGGGCATTATGGTGCTGATATCAGCTGACCGCTATGTAGCTATTTGTGACCCTTTGAATTACCCCATCAGGATCACTGACAGAAGAGTGAAAC
TCTGTGTTTGTCTGTGTTGGCTCTGCTCCATCGCCTTCAGCTGTTTCCTTGTAAGAGATGACCTGCATCAAAAAAGAAAACAAAATTCCTGCTATGGAAAATGTGTCGTT
GTTGTTCAATACATTGCAGGAGTTGTTGACCTCATTTTAACCTTCATTGTACCAGTTACTGTCATCATAGTTCTGTATATGAGAGTATTTGTGGTGGCTGTGTCTCAGGCC
CGCTCTATGCGCTCTCATGCTACAGCTGTCAGAAATCACCTTCCAATGACGCTAACAAAAAAGTCTGAACTAAAAGCAGCCAGGACTCTGGGTGTTCTTGTTCTTGTGT
TTCTAATGTGTTTCTGCCCTTATTACTGTGTTTCTCTTGTAGGAGAAGAATTCATCAATAGTTCATCTGCTTCTTTTGTGGCCTATCTGTTTGGTTTAAACTCATGTCTAAAC
CCTTTGATCTATGCTATGTTCTACCCCTGGTTTAGAAAAGCTGTGAAACTAGTTGTCACTCTACAGATACTGCAGCCTGGCTCCAGTGAGGTAAGTATTTTGtaa
>contig049534-BurTARs.A025
ATGGAGAAAGGAGTCGAGCTCTGCTTTCCACAACTCCTCAACAATTCCTGCATCAAGCCGACACTTCACTGGTCCAAAGCCGTGCTCCTGAACATTGTGCTGTCGTGC
ATCTCTTTGATCACTGCTGGTCTCAATCTTCTTGTTATCATCTCAGTCTCCCATTTCAGGCAGCTGCACACACCCAGTAACATCCTCCTCCTCTCTCTGGCTGTGTCAGA
CTTTCTTGTTGGTCTCCTGTTAATTCCAGCTGAGATTTTTAGAATCACAGTCTGCTGGGTATTTGGTGAATCCATGTGTTCACTTTATACATATCTAGGCTACATTGTTGTA
ACATCTTCAATAAGTAACATAGTTCTCATATCAGTTGATCGCTATGTGGCAATTTGTGATCCTCTGCATTACCCCAGTAGAATTTCTGTGGCAAAAATCAGACTTAGTGTTT
GTATGTGTTGGTTTTATTCGGCTTTCTACAGCACTCTTTGTACAAAGAATATCCTGATTGAACCAGGCAGGTATAATTCCTGTTATGGAGAGTGTGTCTTTATCACCAGTG
ATATTGCTGGCATAATTGACCTTGTTTTATCTTTTATCGTACCAGTTTCCATCATCATAGTTCTATATATGAGAGTATTTGTGGTGGCTGTGTCTCAGGCTCGTGCCATGCA
CTCTCATGTTACAGCTACACTTCAGCGTTCACTGAATCAAACAAACAAATCTGAGCTGAAAGCAGCCAGGACCCTGGGGGTTCTTGTAGTTGTGTTTCTGGCCAGTTTC
TGTCCATTTTATTGTTACTTTCTTGTTGTAGAAGACATAGTCAGTGATTCATCTGCCTCTATTGTGGTCATCGTCTATTATTTTAACTCTTGTCTAAACCCACTAATTTATGCC
CTGTTTTACCCTTGGTTTAGAAATGCTGTTAAAGTCATCATCACTTTTCAGATATTCAAGCGTGACAGCAGTGAGGCCAACGTAATAtag
>contig041024-BurTARs.A026
ATGGAGATTCAGAAAGCAGCTGAGCTTTGTTTTCCACAACTCCTCAACAGTTCCTGCAGGAAGCCAACACTTCATTGGTCCAAAGCTGTGCTCCTGAACATTGTGCTG
TCCTGCATCTCTCTGATCACTGCTGCTCTAAACCTCCTGGTCATCATCTCAGTCTCCCACTTCAGGAAGCTTCATACACCCAGTAACATCCTGCTCCTCTCTCTGGCCG
TCTCAGACTTTCTTGTGGGTCTTCTGCTGATGCCATTAGAGATCTTTAGAAACACAGCTTGCTGGGTACTTGGTGATGTAATGTGTTCTGTTTATTGGTACCTGACCAAC
AACATTACCTGTGCTTCAATAGGGAACATAGTTCTAATATCAGTTGACCGTTATGTGGCTATTTGTGACCCTCTGCATTACCCCACTAGAATTACTTTGGCGAAAGTCAAA
CTCAGTGTTTGTCTGTGTTGGTTTTTTTCTATTTTCTACTGGAGTCTTTATTTGAAAGATATCCTAGTTGAACCAGGCAGGTATAATTCCTGCTATGGAGAGTGTGTGCTTA
TTATTAATGACATTGCAGGGATTGTTGACCTTGCTTTGTCCTTTATAGTTCCAGTTACTGTTATCATAGTCTTGTATATGAGAGTATTTGTGGTGGCTGTGTCTCAGGCTCG
TGCCATGCGCTCTCATGTTACAGCTGTCACACTTCAGTGTTCACTGAATCAAGCAAACAAATCTGAGCTGAAAGCAGCCAGGACTCTGGGGGTTCTTGTAGTTGTATTT
TTGGCATGCTACTGCCCATTTTACTGCTACTCTCTGGTTGATAAAAATGTGGTCAATGATTCATCTGCATCTTTTGTGGTTCTTGTCTTTTATTTTAACTCTTGTTTAAACCC
ACTGATCTATGCTCTGTTTTACCCCTGGTTTAGAAAAGCTATTAGATGTGTTATCACTCTGCTGATATTTAAGCATGACAGCAGTGAGGTCAACGTAATAtag
>contig034854-BurTARs.A027
ATGGATACCCAGGGCGGAGCAGAGCTCTGCTTTCCACAACTCTTCAACACCTCCTGCAAGAAGCCTACAACTCCTCTATCACAAGTTTTGCTCTTTCACATTCTGATGC
CATCAATGTCTCTGCTAACTGTGACTCTCAACCTGCTTGTCATCATTGCAATCTCCCACTTCAGGCAGCTCCACTCACACACTAACATCCTGCTCCTCTCTCTGGCTGC
CGCTGACTTTCTCAATGGCCTTCTGTTTATGCCTGGAGAAATCCTGCGAAATACAGCGTGTTGGTTTCTTGGTCAGCTCACATGTTCATTGTATAATTATGTATCCTACAT
CATTGCCTCTGCCTCAGTGGGCAACATGGTGCTGATATCAGTCGACCGCTATGTGGCTATTTGTGACCCTCTGCATTACCCCACCAGAATCACAGAGGGAAAAGTGAA
ACTTTGTGTTTGTCTGTGTTGGCTCTGCTCTGTTTTGTACAGCTATGTGATTTTAATAGATGATCTAAGTCAACCAGGCAAGCATAAATCTTGCTATGGAAAATGTATAATT
TTCATTGAATTTATTGCAGGATTTGTTGACCTTTTTATATCCTTTATTATTCCACTTACTGTCATCATTGTTCTGTATATGAGAGTGTTTGTGGTGGCTGTGTCTCAGGCCCG
TGCCATGCGCTCTCAGGTTACAACTGTCACACTGCAGCTCTCAGTGACTCTAACAGCAACTAAATCAGAGTTAAAAGCAGCCAGGACACTGGGTGTTCTTGTTCTTGT
GTTTCTATTATGTTTCTGCCCATATTACATTGTTTCACTTTTTGGAGACGAGGTTCTCAATAGTGCCTCTGAATCTATTGCATTCTATTTCTTTCTTTTAAACTCATGTCTAAA
CCCTTTGATCTATGCTATGTTCTACCCCTGGTTTAGAAAAGCTGTGAAACTAATTGTGACTCTACAGATACTGCAGCCTGGCTCCTGTGAAGTCAGCATACTGtag
>contig057148-BurTARs.A028
ATGCAGAAAGAAGCCGAGCTTTGTTTTCCACAACTCCTCAACAGCTCCTGCAGGAAGCCAACATTTCACTGGTCCAAAGCTGTGCTGCTGAACATTGTGCTGTGCTGC
ATCTCTCTGCTCACTGCTGCTCTAAACCTTCTCGTCATCATCTCAGTCTCTTACTTCAGGCAGCTTCACACACCCACTAATATCCTCCTCCTTTCTCTGGCTGTCTCAGA
CTTTCTCACTGGTCTCCTTATGCTGCCACTAGAAATAATTAGAAACACGGCCTGCTGGGTACTTGGTGATCTTATGTGTTCTGTTTATACTTATCTGACAGCCGGTCTTTT
CTGTGCTTCAACTTGGAACATAGTTCTCATTTCAGTTGACCGCTATGTAGCTATTTGTCACCCTCTTCATTACCCCACCAGAATTACCGTGGCAAGAGTCAAACTCTGTG
TTTGTTTGTGTTGGTTTTGTTCAACCTTCTACGGCAGTCTTTTAACAAAGGATGTTCTAATTAAGCCGGCCAGGTATAATTCCTGTTATGGTGAGTGTGTGTTTGTCATTAA
TGATATTGCAGGGTTTATTGATCTTGTGTTATCTTTTATTTTTCCAGTATCTGTTATCATAGTTCTTTATATGAGAGTGTTTGTGGTGGCTGTGTCTCAAGCTCGTGCCATGC
GCTCTCATGTTACAGCTGTCGCACTTCAGCGTTCAAGCAATCAAGCAAACAAATCTGAACTGAAAGCAGCCAGGAACCTTGGAGTTCTTGTAGTTGTGTTTCTACTGTG
TTACCTTCCGTTTTACTGCTACTCTCTTATTGAAGTAAATGTGATCAATGACTCATCTACATTTTTTCTGATCATTGTGTTTTACTTTAACTCGTGTCTAAACCCACTGATGTA
TGCCCTTTTTTACTCTTGGTTTAGAAATGCTGTTAAACTTATTATCACTCTGCAGATTTTTAAGCATGACACTACTGAAGCAAACATGttttaa
>contig059766-BurTARs.A029
ATGGAGGAAACTGAACTCTGCTTTCAACAACTCTTTAACACCTCCTGCATGAGGCCCAGGCGTCCACACTTTGAGATCATGCTGACTTATATTTTGCTGTCCTTCATTTC
TCTGCTTACTGTGATTCTTAACCTGTTGGTCATCATCTCCGTCTCACATTTCAGGCAGCTCCACACCCCCACCAACCTCCTGCTCCTTTCTCTGGCTGTCGCTGATTTCT
ATGTGGGCCTCCTCTTGTTCTTCCAAATTGTGCTCATAGATGGCTGCTGGTTCCTCGGTGACATCATGTGCACTCTGTATCAATACCTAGCATATGTCATCACCTCGGCC
TCCATAGGAACCATGGTGATCATATCTGTTGATCGATATTTGGCTATTTGTTACCCTCTGCATTACTCCACCAAGATCACACAACAAAGAGTTAAAATTGTTGTCTGTTTGT
GTTGGATCTGTTCTGTCATCTTTCAAAGTCTGATTCTGATGGATAACCTGGAGCAACCAGGCAGGTATAACTCTTGCATTGGAGAGTGTGTCTTTGTCATTAATTACATCG
CAGGACTTGTTGATGTTACTTTTTCCTTTATTGTTCCCTTTACTGTGATTGTAGTTTTGTATCTGAGAGTGTTTGTGGTGGCTGTGTCTCAGGCTCGTGCCATGAGGTCT
CAGCTTGCAGTCACTCACCAGCGATCAGTAACAGTAACTGCAAAGAAATCGGAGCTGAAAGCAGCCTGGACTCTTGGTATTGTTGTTTTTGTGTTTCTCATATGTATGTG
TCCATATTACTGTGTGGCTCTCACAGGCCAAGATAACTTGCCAAGTGCTTCATCACTGACATTTGTTCTATGTTTGGTCTACTTTAACTCATGTCTGAACCCCATTATATAT
GTCTTTTTCTACCCTTGGTTCAGAAAATCCATAAAAGTCATTGTTACTCTTCAGATACTGCAGCCTGACTCCTGCCAGGCCACCGTGCTTtag
>contig065494-BurTARs.A030
ATGGAGGAAACTGAACTCTGCTTTCAACAACTCTTTAACACCTCCTGCATGAGGCCCAGGCGTCCACACTTTGAGATCATGCTGACTTATATTTTGCTGTCCTTCATTTC
TCTGCTTACTGTGATTCTTAACCTGTTGGTCATCATCTCCGTCTCACATTTCAGGCAGCTCCACACCCCCACCAACCTCCTGCTCCTTTCTCTGGCTGTCGCTGATTTCT
ATGTGGGCCTCCTCTTGTTCTTCCAAATTGTGCTCATAGATGGCTGCTGGTTCCTCGGTGACATCATGTGCACTCTGTATCAATACCTAGCATATGTCATCACCTCGGCC
TCCATAGGAACCATGGTGATCATATCTGTTGATCGATATTTGGCTATTTGTTACCCTCTGCATTACTCCACCAAGATCACACAACAAAGAGTTAAAATTGTTGTCTGTTTGT
GTTGGATCTGTTCTGTCATCTTTCAAAGTCTGATTCTGATGGATAACCTGGAGCAACCAGGCAGGTATAACTCTTGCATTGGAGAGTGTGTCTTTGTCATTAATTACATCG
CAGGACTTGTTGATGTTACTTTTTCCTTTATTGTTCCCTTTACTGTGATTGTAGTTTTGTATCTGAGAGTGTTTGTGGTGGCTGTGTCTCAGGCTCGTGCCATGAGGTCT
CAGCTTGCAGTCACTCACCAGCGATCAGTAACAGTAACTGCAAAGAAATCGGAGCTGAAAGCAGCCTGGACTCTTGGTATTGTTGTTGTTGTGTTTCTCATATGTATGT
GTCCATATTACTGTGTGGCTCTCACAGGCCAAGACAGCTTGCCAAGTGCTTCATCACTGACATTTGTTCTATGCTTAGCCTACTTTAACTCATGTCTGAACCCCATTATAT
ATGTCTTTTTCTACCCCTGGTTCAGAAAATCTATAAAAGTCATTGTTACTCTTCAGATACTGCAGCCTGACTCTTCTAAAGTCACCATGCGTtag
>contig057305-BurTARs.A031
ATGGCAGAAACTGAACTCTGCTTTCCAAATCTCAACTCCTCCTGCAGTAGAGTAAAGCGTTCTCCCTCGGACTCAGTGCTTATTTATGTAATCCTAACCATCATTTCTCTT
CTTACTGTGGCTCTAAACCTTCTGGTCATCATCTCCATCTCCCACTTCAAGCAGCTCCACACTCCTACAAACCACCTCCTCCTCTCCCTGGCAGTCTCTGATTTCTTTGT
GGGTCTCAATATGTGCTTTCAAAGTATGCTCGTAGATGGTTGTTGGTATCTTGGTGACCTCATGTGTGTCTTGTATTATGTTTTCGACATAGTTGTTACTTCTGCCTCAGTA
GGAACCATGGTGCTCATTTCAGTTGACCGTTATGTGGCCATTTGTGATCCTCTTCATTATCCCACCAAAGTCACTCCAAAAAGAGTTCAGACCTGTGTCTTAATGTGTTG

GATTTGCTCGCTCCTAGTAGTCGGTGTGCTCTTGAAGGATAACCTGGATAAACCAGGTAGATTTAATTCCTGCTTTGGAGAGTGTGTGATTTATGTTGACTTTGCAATAC
AAGTTACCGATCTTATTTTGACAATCCTCCTTCCCATTACTGTCATCGTAATTTTGTATGTAAGAGTATTTGCCGTGGCCGTGTTTCAGATTCGGGCCATGCAACCTCATG
TTCCAGCTGTCACACAGAGGGGGAAAGTAAGTCCAAAAAAATCTGAACTGAAAGCAGCTAGGACTCTTGGCATTGTTATTGTTGCATTTCTGATATGCCTATTCCCATAT
TACAGTGTAATACTCTCAGGCCAGGACACACTGCTTGATATTTTATCCGTTACCTTTTTTTTATGTTTGTTTTATTTTAATTCCTGTCTCAACCCTATCATCTATGCCTTTTTC
TACCCATGGTTTAGAAAATCTGTTAAACTTATTGTTACATTTCAAATAGTCAAGTCTGGCTCCAGTGATGCCAGCATGCTGtga
>contig040586-ZebTAR.A001
ATGGATAGCTCTGGAGGTCCTCCCCTCTGCTTCCCCAACCTCAACTCCTCCTGCAGGCGGCTGCTGCGACCCACCTCCCAGACCGTTGTTCTCTACACTCTGCTGGC
CTCCATCTCCCTGCTCACTGTGGTGCTCAACCTGCTTGTCGTCATCTCCATCTCCCACTTCAGACAGCTTAACACCTCGACCAACACCCTGCTCCTTTCCCTGGCTGT
GTCTGACCTGCTGGTGGGGTTGCTGGTGATGCCCATCGAGGGCCTGGACTACATCGAGACGTGCTGGCTGCTGGGGAGGCTGATGTGTGCTCTCAGTCCTTATTTG
TCTTACTGCCTCATCTCTGTCTCTCTGGACAGCATGGTGCTCATATCTGTAGATCGCTATATAGCCATCTGTGACCCTCTGCTCTATTCCTCAAAGATCACAGTGAACAGA
GTGAAGCTTTCAGTCTGTGTCTGCTGGGCCTGCTCTCTTCTCTACAATGGCTGCATTCTCATGGAGCACATAGGGTGGCCAGACAGGTTCAGCTCCTGTCACGGGGA
GTGTGTGGTGTTCATCAGCCGCGCTTTGGGTACAATAGATCTCTTTTTCTCATTTCTCGGCCCATGTGCCTTAATGTTTGTTCTGTACATGAGGGTCTTTGTGGTTGCTG
TTTCTCAGGTACGTATCATTCGGTCGCAGGCAGCTGTGAGAGCAGCTCCAGCTGCTAAAAAATCAGAGCTGAAGGCAGCCAGGACACTCGGGATTTTGATAGCTGTGT
TTCTAATGTGCTTCTGCCCTTATTATTATCCTTCCTTTGCAGGTGACGACACCTCTATGAGCTTACCTTATTACGCTCTATTCTCGTGGATAATGCTGACAAATTCTTGTGT
GAACCCTGTGATTTATGCTCTGTTCTACCCCTGGTTTAGAAGAGCTATCAGACTCATTGTCACCCTCAGAATACTGCAGCCTCACTCCAGAGATGTCAAGATCCTGtag
>contig040586-ZebTAR.A002
ATGGACGGCACTGGAGGTCCTCCCCTCTGCTTCCCTAACCTCAACTCCTCCTGCAGGCGGCTGCTGCGACCCACCTCCCAGGCCGCTCTTCTCTACACTCTGCTGG
CTTCAGTCTCACTGCTTACTGTGGTGCTCAACCTGCTTGTGGTCGTCTCCATCTCCCACTTCAAGCAGCTCCACACCCCGACCAACGCCCTGCTCCTGTCCCTGGCC
ATGTCTGACCTGCTGGTGGGGTTGCTGGTGATGCCCATCGAGGGCCTGCGCTACATCGAGACGTGCTGGCTGCTGGGGAGGCTGATGTGTGCTCTCAGTCCTTATTT
GTCTTACTGCCTTATCTCTGTCTCTCTGGGCAGCATGGTGCTCATATCTGTAGATCGCTATATAGCCATCTGTGACCCTCTGCTCTATTCCTCAAAGATCACAGTGAACAG
AGTGAAGCTTTCAGTCTGTGTCTGCTGGGCCTGCTCTCTTCTCTACAATGGCTGCATTCTCATGGAGCACATAGGGTGGCCAGACAGGTTCAGCTCCTGTCACGGGG
AGTGTGTTGTGTTCATCAGCCAAACTTCAGGAACAGTAGATTTCTTTTTGTCATTTCTTGGCCCCTGTGGCGTAATGTTTGTTCTGTACATGAGGGTGTTTGTGGTTGCT
GTTTCTCAGGTGCGTGCCATTCAGTCGCAGGCAGCTGTGAGAGCAGCTCCAGCTGCTAAAAAATCAGAGCTGAAGGCAGCCAGGACACTCGGGATTTTGATAGCTGT
GTTTGTAATGTGCTTCTGCCCTTATTATTATCCTTCCCTTGCAGGTGTGGACACCTTTCTGAGCTTGCCTTCGTACGCCATGGTGTGTTGGATCATGCTAATAAATGCTTG
TGTGAACCCTGTGATTTATGTTCTGTTTTACCCCTGGTTTAGAAGAGCTATCAGATTCATTGTCAGCCTCAGAATACTGCAGCCTCACTCCAGAGACGTCAAAATCCTGta
g
>contig033536-ZebTAR.B029
ATGGAGTTATTCAATGTGACTGTTAATACTGTGAGCTTCCTTCTCTGCGATTCACAAAAGAATAAATTATGTGTGTTATTATATGTTGTCCTCAGCTCATTTATGCTTCTTAC
AATATGTGGAAATCTTCTTGTGATAATTTCTATAATTTACTTTAGATATCTCCACACTCCTACAAACTACCTTATCCTCTCTATGGCTGTGGCTGATCTACTAATTGGTGCTTT
AATATTTCCTTTGAGCATGACAGTGTCTCTAAAGCCATGTTTGTATATATACAGTTTACTGTGCAACTTAAGAAGCACAATGGATGTAACAATGGGTGTATCTTCCTTATTAA
ATTTGTGCTGTATTTCTGTTGATCGATATTATGCTGTTTGCCACCCTCTGATATATAAAACTAAAATAACTGATTGTGTTGCCATGAAGATGGGCCTTGGAAGTTGGGCTGT
TGCTATCTTGTGTGGAATTTTTGTCTTCCTGCTGTTTTTTATTTTAGACGAATGTGACACAAGTTGCGTTTTCGCTCTCATTGCCGCATCAGTGGTAGTATATTATATCCCA
ACAATAGTTTTACTTTTCATGTACACCAAAATTCTTGTTGTTGCACTGAGGCAGGCACGCAGCATCCATAATACAATTTCTCAGAACACAAAGTCTAAAGCAGTTTCCAGT
ACGGAGAGAAAGGCCACAAAGACATTAACCATAGTTATTGGAATATTTTTAATTTGTTGGGTTCCTCTTTTTCTTAGTTATTCATTTGTTCCTCTGGATAGTTTTATACTTTAT
GTGCTTCTTGAACCATTTAACTGGTTTGCAATTTCTAACTCAATGCTCAATCCCTTTATTTATGCTTTCTTTTACACTTGGTTTAGAAGAGCTTTTAAAATGATAATTTCTGG
AAAAATATTTCAAGGTGATGTTACTAACATAAAACTAcactga
>contig003909-ZebTAR.A003
ATGGACAGGCAGGACAGAATTGAACTCTGCTACCCTGAGCTTGCGAATCTGTCTTGCAGGGGCTTAACACGTCCTCAGCCTGAGGCTGTCCTCCTCTATACACTGTTC
TTGTCTATCTCTGTGCTAACTGTAGCTCTGAATGTGCTGGTTATCATATCCATCTCTCATTTCAGGCAGCTCCACATGCCCACCAATGTCCTCCTGCTCTCTCTGGCCAT
CTCAGACCTCTTGGTCGGGCTGCTTGTGATGCCGGTGGAAGCAATGCGGTTTATAGAAACCTGCTGGCTGCTGGGAGATCTCATGTGTGCTTTTACTTACATTATCGGT
TTCACTCTCACCTCAGCCTCTGTGGGCAACATGGTGCTCATATCAATTGATCGCTACGTAGCTATTTGTTATCCTCTGCAGTACCCCACTAAAATAACTCACAGCAGAGT
GGAGCTGTCTGTGACTCTGTGCTGGGCCTGTTCGCTCCTCTACAACGGGATGATCTTAAAGGAGCATCTCAGGCAGCCAAACAGGCATAACACCTGTCACGGTCAGT
GTTTGGTGGTGATTAACTATGTCTCTGGAGCAATCGACCTTGTCTTTACTTTCATCGGCCCTTGCTCAGTCATTATCATTCTGTACATGAGAGTATTTGTTGTAGCGGTGT
CTCAGGCTCATGCCATGCGGTCTCATATTACAGCTGCTGCAGCTGGTACAGTAAAAATCACCGCAAAGAAATCAGAGAAAAAGGCAGCCAGGACTCTTGGCGTTGTGA
TATTTGTGTTTTTGATGAGTTTCTGTCCGTATTACTATCCCTCTCTTGCAGGGCAGGACATTTCAAACAGTGCTTCATCTTGGGCTATTGTTTCCTGGATGCTGTACTTCA
ATTCTTGTCTGAATCCACTGATATATGCTTTTTTCTACCCGTGGTTTAGAAAAGCTATCTGGTTCATTGTCTCCCTGAAGATACTAGAGAAGGGCTCCTCTCAGGCAAATA
TACTTtaa
>contig063680-ZebTARe.A004
ATGGAAGAAACTGAACTCTGCTTTCCACAACTATTCAACTCCTCTTGTGTGCGGCAAAAGCATTCACAAATTGAGGCTGTGTTTATTTACACTCCACTGTCTTCCATCTC
TGTGCTTACTACAATTCTGAACCTGCTTGTGATCATCTCCATCGCTCACTTCAAGCAGCTGCACACCCCAACCAACCTCCTCCTCCTCTCTCTGGCCGTCTCAGATTTC
TTCGTGGGCCTCATCATGGCTTTTGAGATTAGCCTCCTAGATGGCTGCTGGTTTCTTGGTGACCACATGTGTGCTCTGTATAGCAGTTTAGATTACATTGCTACTTCTGC
TTCAGTAGGAACTATGGTACTCATATCAGCTGACCGCTATGTAGCCATTTGTGACCCTCTGCATTATCCCACCAAAATCACTATAAAAAGAGTCTCAGTCTCTATTTGTACT
TGCTGGGCTTGTTCAATTCTGTATAACAGTCTTATCATGAAGGATAATTTGAAGCAGCCAGGGAGGTATAATTCTTGTTCTGGTGATTGTGTAGTTGTCATT
>contig061509-ZebTARe.A005
AAAGTCACTGCAAAAAGAGTTCAGATCTGTGTTTCACTGTGTTGGAGCTTTTCTGCCCTTGCTGGTAGTTTCCTCTTAAAGGACAACTTGAAACAGCAAAGCAGGTTTA
ATTCTTGTGTGGGAGAGTGTGTTGTCCATATTAACTTTATTGAATATGTTGCAGATCTTGCTTTGAACTTCCTACTTCCTATTACTGTCATTATAGTTCTGTATTTGAGAATAT
TTGTAGTGGTTGTGTCTCAGGTTCGGGCCATGCGGACTCATACTGCAGGTGTCACATACCAGTGTTCAAGGAAGGGAAATCCAAAGAAATCAGAGATGAAAGCAGCCA
GGACTCTTGGTATTGTTGTAATTGCATTCTTGTCATGCGCCTTACCATTTTATTGTGTCACACTCACAGGCCAGAATGCCTTCCTCAACGGATCATCTTCTGCATTTGTTT
TATGTCTTTTCTATTTTAATTCGTGCCTCAACCCTATCATCTATGCCTTATTCTACCCCTGGTTTAGAAAATCTATTAAGCTTATTGTTACATTTCAAATACTGAAGTCTGGCT
CCAGGAATGCCAACATAGTCAAAGTGACAGAAtag
>contig061414-ZebTARe.A006
ATGAGTCTTTACACAAAGGATGTCTTGATAAAGCCAGGCAGGTATGCTTCCTGCTATGGAGAGTGTGTGTTTTTTATTGAAGATATAACTGGAACTGTTGACATTGTTTTA
TGTTTTTTTGTTCCAGTTATTATCATCATTGTTCTGTATACAAGAGTATTTGTAGTGGCAGTGTCTCAGGCCCGTGCCATGCGCTCTCATGTTACAGCTGTCACACTTCAG
CGTTCATTGAATCAATCAAACAAATCTGAGCTGAAAGCAGCCAGGAACCTTGGAGTTCTTGTAATTGTGTTCCTGGCAAGTATTTGCCCATTTTACTTCTACTCTCTTGTT
GACGGCAATGTGGTCAAAGCTTCATCTGCGACTTTTTTGATCATTGTTTATTTTAACTCTTGTTTAAACCCACTGATCTATACCCTGTTTTACCCCTGGTTTAGAAATGCTG
TTAAACTTATTATCACCCTGCAGATATTCAAGCATAACAGTAGTGAAGCCAACATACTAtag
>contig053136-ZebTARe.A007
GTGATTGTAGTTCTTTATCTAAACGTATTTGTTGTGGCTATGACTCAAGCTCGTGCCCTGCGGTCACATATTGCAGCTCTGACACACGAGAGGTCAGTAAGTACAAATGT
TAAGAAATCTGAAATGAAAGCAGTAAGGACCATCAGTGTTCTTATCATTGTGTTTCTAATATGTCTCTGCCCATACTACGGTGTCACACTCTCAGGTCAAGATGCCATCCT
CAGTGCTTCATCTGTTGCATTTGTAATGTGTTTGTTCTATTTAAACTCCTGTCTAAACCCTCTAATCTATGCTTTATTTTACCCCTGGTTCAGAAAATCTGTTAAACAAATTG
TTACACTTAAAATATTGAAGTCTGGCTCCTGTGACACCAATATCATGTACACAGAATGA
>contig053129-ZebTARe.A008
ATGGCAGAAACTGAACTCTGCTTTCCAAATCTCAACTCCTCCTGCAGTAGAGTAAAGCGTTCTCCCTCGGACTCAGTGCTTATTTATGTAATCCTATCTATCTCTTATCTC
TCCAGGCAGCTCCACACTCCTACAAACCACCTCCTCCTCTCCCTGGCAGTCTCTGATTTCTTTGTGGGTCTCAATATGTGCTTTCAAAGTATGCTCGTAGATGGTTGTT
GGTATCTTGGTGACCTCATGTGTGTCTTGTATTATGTTTTCGACATAGTTGTTACTTCTGCCTCAGTAGGAACCATGGTGCTCATTTCAGTTGACCGTTATGTG
>contig067676-ZebTARep.A010
AACATAGTTCTCATCTCAGTTGACCGTTATGTGGCGATTTGTGACCCTCTGCATTACCCCACCAGAATGACTGTGGCGAGAGTCAAATTTAGTGTTTGTCTGCGTTGGTT
TTATGCTGTTTTCTACTGCAGTCTCTACATGAAGGATATCCTAGTTGAACCAGGCAGGTATAATTCCTGCTATGGAGAGTGTGTGTTTGTCATTAATGATATTGCAGGGATT
TTTGACCTAGTTTTCTCTTTTTTTGCTCCAGTTATTATCATCACAGTTGTTTATATTTGAGTGTTTGTGGTGGCTGTGTCTCAGGCTCGAGCCATGCGGTCTCATGTCACT
GAATCAAGCAAACAAATCGGAGCTGAAAGCAGCCAGGACTCTTGGGATTCCTGTAGTTGTGTTTCTCTCCTCTCTTTGAACTCTTGCAGAGACTCCTGCACTATTTTCC
AGACATACTGCTTGTTTTCATAA
>contig030442-ZebTARfs.A011
AGGCAGCTCCACACACCCACTAACATCCTGCTCCTTTCTCTGGCTGTCACTGACTTTCTCGTTGGTCTTCTGTTTATGCCTGGAGAAATCCTGCGAAATACAGCGTGTT
GGTTTCTTGGTCAGCTCACATGTTCATTGTATAATTATGTATCCTACATCGTTGCCTCTGCCTCAGTGGGCAACATGGTGCTGATATCAGTCGACCGCTATGTGGCTATTT
GTGACCCTCTGCATTACCCCACCAGAATCACAGAGGGAAGAGTGAAACTCTGTGTCTGTCTGTGTTGGCTCTGCTCTGTTTTCTACAGCTATGTGATTTTAATAGATGAT
CTAAGTCAACCAGTCAAGCATAAATCTTGCTATGGAAGATGTATAATTTTCATTGAATTTATTGCAGGATTTGTTGACCTTGTTTTAGCCTTTATTATTCCACTTACTGTCATC
ATTGTTCTGTATATGAGAGTGTTTGTGGTGGCTGTGTCTCAGGCCCGTGCCATGCGCTCTCAGGTTACAGCTGTCACACTGCAGCTCTCAGTGACTCTAACAGCAACT
AAATCAGAGTTAAAAGCAGCCAGGACTCTGGGTGTTCTTGTTCTTGTGTTTCTATTATGTTTCTGCTCATATTACATTATTTCACTTTTTGGAAACGAGTTGCTCAATAGCT
CATCTGCATCGATTGTGATCTATATGTACTATTTTAACTCCTGTCTAAACCCTTTGATTTATGCTATGTTCTACCCCTGGTTTAGAAAAGCAGTGAAACTAATTGTGACTCTA
CAGATACTGCAGCCTGGCTCCTGTGAGGTCAGCATACTGTAG
>contig067676-ZebTARp.A012
ATGTCCTGTGTGTACTTCATCATGACTGTATAGGTTTTATTTCCTTAATTGCACCATGCCTCTAATAGAACATCTTCATTGAAGATATTTTGACATGATTAATGTGTAATTTATA
ATTTTCTGTCTCCCTGCAGGCAGCTCCACACTCCCAGTAACATCATCATCCTCTCTCTGGCTGTCTCTGACTTTTTTGTCTGTTTTTTGTTGATGCCGGTAGAAATCTTC
AAAAACACGGCCTGCTGGGTATTTGGTGATCTCATGTGTTCACTTTATACTTATCTGAGCTGCATTCTTATCAATGCTTCATTTGAAATGATTATTCTCGTATCAGTTGACC
GCTATGTGGCTATTTGTGACCCTCTGCATTACCCCACCAGAATCACTGTGCCGAGAGTCAAACTCAGTGTTTTTCTGTGCTGGCTTTATGCTATTTTGTACAACATCATCT
ATACAAAGCATGCCCTGATAAACCCAGGCAGGTATGGTTCCTGCTATGGAGAGTGTGTTTTTGTTGTTGATGATATTATTGGAATTGTTGACTTTGTTGTATCTCTTATAGT
TCCAGTTACGATCATCGTAGTTCTGTATACGAGAGTGTTTGTGGTGGTTGTGTCTCAGGCTCGTGCCATGCGCTCTCATGTTACAGCTGTCACACTTCAGCAGCCACTG
AATCAAGCAAACAAATCTGAGCTGAAAGCAGCCAGGAACCTTGGGATTCTTGTAGTTGTGTTTCTGGCGTGCTACTGTCCATTTTACTGCTACTTTTTTCTTGCAGGCAA
TGATCCAGCTGCTTCTTCTGCATCTTCTATCCTCATTGTTTATTATTTTAACTCTTGCCTAAACCCATTAATGTATGCACTGTTTTACTCCTGGTTTAGAAATGCTGTTAAAC
TTATCATCACTCTGCAGATATTAAAGGCTAACAGCAGTGAGATCAACATACTACAGAGATAA

>contig030469-ZebTARp.A030
CTGACTGGCAACATTGCCTTTGCCTCAACAGGGAACATAGTTCTAATATCAATTGACCATTATGTGGCTTTTTGTCCCCCTCTGCATTACCCCACCAGAATTACTGTGGC
AAGAGTCAAACTTAGTGTTTCTCTGTGTTGGTTATTTGCCATTTTCTACAGCAGTCTCTATATGAAGGATGTACTGATAAAGAGGTATAATTCCTGCTATGGAGAGTGTGT
GTTTATCATTAATGATATTGCAGGGATTTTTGACACGGTTTTATTTTTTGTTCCAGTTCCAGTTATCGTAATTCTGTATATGAGAGTGTTTGTAGTGGCTGTGTCTCAGGCTT
GAGCCATGCGCTCTCATGTTACAGCTGTCACACTGCAGCGTTCATTGAATCACACAAACAAATCTGAGTTGAAAGCATCTGGGACTCTTGGAATTCTTGTAGTTGTGTT
TTTAATATGCTACTGTCCATTCTTCTGCTTTTCTTTTGGTGTAGAAGACATGGTCAATAATCCATCTACATTTTTTGTGATCATGGTCTTTTACTTTAATTCTTGTCTAAACCC
TTTGATCTATGCCCTGTTTTACCCCTGGTTTAGAAATGCTGTAAAACTGATCATCACACTGCAGATTTTCAAACATGAAACTAGTGAAGCCAACATACTGTAA
>contig030459-ZebTARp.A013
ATGTCCTGTTTGCCTAGACTGAATCAAGCCTTACTAATGCAGCATTTTCAATGAAGACAGTTTGACATTAATAATGTGCATTAAATTCTTTTGTGTCTCTGCAGGAAGCTTC
ACACACCCAGTAATATCCTCCTCCTTTCTCTGGCTGTCTCAGACTTTCTTGTCGGTCTCCTGTTGATGCCATTAGGAATCCTTAGAAACACAGGCTGCTGGGTACTTGG
TGATATAATTTGTTCTCTTTATTGGTATTTAACCAGCAACATTGTCTGTGCTTCAATAGGGAACATTGTCCTAATATCAGTTGACCGCTATGTGGCTATTTGTGACCCTCTG
CATTATCCCAGCAGAATTACTTTGGTGAAAGTCAAACTCAGTGTTTGTCTGTGTTGGTTTTATGATTTTTTCTACATCAGTCTTTATACAAAGGATATCCTAATTGAACCAG
GCAGGGATAATTCCTGCTACGGAGAGTGTGCATTTGTCATCAATGATATTGCTGTTGTTGTTGACCTTGTTTTTTCCTTTATTGTTCCGGTTTCTGTCATTGTAGTTCTGTA
TATCAGAGTATTTGTGGCAGCTGTGTCTCAGGCTCGTGCCATGCGCTCTCATGTGACATCTGTCACACTGCAGCGCTCACTGAATCAAACAAACAAATCTGAGCTGAAA
GCAGCCAGGACTCTTGGAATTCTTGTAGTTGTGTTTCTGGCATGCTTCTGTCCACTCTACTACTTTTCTCTTGTTGATGAAAATATCAATGATCCAGCTGCATCTTTTGTG
GTCATTATATTTTACTTTAACTCGTGTCTAAATCCTTTGATCTATGCCTTCTTTTACCCCTGGTTTAGAAATGCTGTTAAGCTTATCATCACTCTGCAGATTTTCAAGTATAAC
ATGAGTGAGGCCAACATACTATAA
>contig066890-ZebTARs.A014
ATGGATACTCAGGATGGAGAAGAGCTCTGCTTTCCACAACTCTTCAACATCTCCTGCAAGAAGCCTACAACTCCTCTGTCTCAATTGTTGCTTCCTTATATTGTTGTGTT
CTCAATGTCTCTGCTGACTGTGATTCTCAACCTGCTCGTCATCATCGCAGTCTCCCACTTCAGACAGCTCCACACACCCACTAACTTCCTCCTCCTCTCTCTGGCTGTG
TCAGACTTTTTCATTGGTCTGTTGTTGATGCCTGCAAATATCATCCGAAAGAAGGTTTGTTGGTTTCTTGGTCAGCTTGCATGTTTAGTGTTTACTTATGTATCCTTCATTA
TTACCTCTGCCTCAGTGGGCATCATGGTGCTGATATCAGTCGACAGGTATGCGGCTATTTGTGACCCTCTGCATTACCCCACCAGAATAACTCGTGAAAGAGTGAAACT
CTGTGTCTGTCTGTGTTGGCTCTGCTCTGTTATCTACAACATTTTGCTCATAAAGGATGAACTGGTTCAACCAGGGCAACATAATTCCTGCTATGGAGAGTGTAAAATTCT
CGTAGACTACATTGCAGGAACCACTGATATTGTTTTAACCTTTATTGCTCCAATTACTGTCATCATAGTTCTGTATATGAGAGTGTTTGTGGTGGCTGTGTCTCAGGCCCG
TACCATGCGCTCTCAGGTTACAGCTGTCACACTACAGCTCTCAGTGACTCTAACAACAAAGAAATCAGAGTTAAAAGCAGCCAGGACTCTGGGTGTTCTTGTTCTTGTG
TTTCTAATATGTTTCTGTCCTCATTATTGTATTTTTGTTTTTGGAGGAAAGGTGCTTAATAGCTCATCTGCAACTATTGTGATCTATCTGTATTATTTTAACTCCTGTCTAAATC
CTTTGATTTATGCTATGTTCTACCCCTGGTTTAGAAAAGCTGTGAAACTAATTGTCACACTACAGATACTGCAGCCTGGCTCCTGTGAGGTCAGCATACTGtag
>contig066691-ZebTARs.A015
ATGGAGATACCGAAAGGAGTCGAGCTCTGTTTCCCACAACTCCTCAACAGCTCCTGCAGGAAGCCGACACTTCACTGGTCCAAAGCTGTGCTCCTGAACATTGTGCT
CTCATGTATCTCTCTGCTCACTGCTGCTCTAAACCTTCTCGTCATCATCTCAGTCTCCCACTTCAGGCAGCTGCACACACCCAGTAATATCCTCCTCCTCTCTCTGGCC
GTCTCAGACTTTTTTGTGGGTCTCCTGTTGTTGCCTTTGGAAATTTTTAGAAACACAAGCTGCTGGGTACTTGGTGATCGTATGTGTTCTGCTTATTGGTATTTGACCAG
CAACATTATCTGTGCTTCAATAGGGAACATTGTTCTAATATCAGTTGACCGCTATGTGGCTATTTGTGACCCTCTGCATTATCCCAGCAGAATTACTTTGGCGAAAGTCAA
ACTCAGTGTTTGTCTCTGTTGGTTTTATGCTTTTTTCTACAGCAATCTTTATACAAAGAATATCATGATTGAACCAGGCAGGTATAATTCCTGCTTTGGAGAGTGTGTATTT
TTTAGCAGTAATATTGCTATTGTTGCTGACCTTATTTTATTCTTTTTTGTTCCAGTAACTGTTATCATAGCCTTGTATATGAGAGTATTTGTGGTGGCTGTGTCTCAAGCTCG
TGCCATGCGCTCTCATGTTACATTGGTCACACTTCAGCGTTCACTGAATCAAACAAACAAATCTGAGCTGAAAGCAGCCAGGACTCTGGGGATTCTTGTAGTTGTGTTT
CTAGCATGCTTCTCTCCACTCTACTGCTACTCTCTTGTTGATGAAAATGCAATCAATAATCCAGCTGCATCTTTTGCGGTCATTATCTTTTACATTAACTCTTGTCTAAACC
CTTTGATCTATGCCTTGTTTTACCCCTGGTTTAGAAATGCTGTTAAACTTATCATCACGTTGGAGATCTTCAAGTATGACACAAGTGGTGCCAACATACTGtaa
>contig066285-ZebTARs.A016
ATGGAGAAAGGAGCCGAGCTCTGCTTTCCACATCTCTTCAACAATTCCTGCATCAAGCCGACACTTCACTGGTCCAAAGCCGTGCTCCTGAACATTGTGCTGTCGTGC
ATCTCTTTGATCACTGCTGGTCTCAATCTTCTTGTTATCATCTCAGTCTCCCATTTCAGGCAGCTCCACACACCCAGTAACATCCTCCTCCTCTCTCTGGCTGTGTCAGA
CTTTCTTGTTGGTCTCCTGTTAATTCCAGCTGAGATTTTTAGAATCACAGTTTGCTGGGTATTTGGTGAATCCATGTGTTCACTTTATACATATCTAGGCTACATTGTTGTA
ACATCTTCAATAAGTAACATAGTTCTCATATCAGTTGATCGCTATGTGGCAATTTGTGATCCTCTGCATTACCCCAGTAGAATTTCTGTGGCAAAAATAAGTCTTAGTGTTT
GTATGTGTTGGTTTTATTCAGCTTTCTACAGCACTCTTTGCACAAAGAATATCCTGATTGAACCAGGCAGGTATAATTCCTGTTATGGAGAGTGTGTGTTTATCACCAGTG
ATATTGCTGGCATAATTGACCTTGTTTTATCTTTTATCGTACCAGTTTCCATCATCATAGTTCTATATATGAGAGTATTTGTGGTGGCTGTGTCTCAGGCTCGTGCCATGCA
CTCTCATGTTACAGCTACACTTCAGCGTTCACTGAATCAAACAAACAAATCTGAGCTGAAAGCAGCCAGGACTCTGGGGGTTCTTGTAGTTGTGTTTCTGGCCAGTTTC
TGTCCATTTTATTGTTACTTTCTTGTTGTAGAAGACAAAGTCAGTGATTCATCTGCCTCTATTGTGGTCATCGTCTATTATTTTAACTCTTGTCTAAACCCACTAATTTATGC
CCTGTTTTACCCTTGGTTTAGAAATGCTGTTAAAGTCATCATCACTTTTCAGATATTCAAGCATGACAGCAGTGAGGTCAACGTAATAtag
>contig066056-ZebTARs.A017
ATGGAAGAAACTGAACTCTGCTTTCCACAACTCCTCAACTCCTCTTGTGTGCGGCAAAAGCGCCCACAAATTGAGGCTGTGTGTATTTACACCCTGCTATCATCTATCT
CTCTGCTTACTGCAGTACTGAACCTACTTGTCATCATCTCCATCGCTCACTTCAAGCAGCTGCACACCCCAACCAACCTCCTCCTCCTCTCTCTGGCCGTCTCAGATTT
CTTCGTGGGCCTCATCATGGCTTTTGAGATTAGCCTCCTAGATGGCTGCTGGTTTCTTGGTGACCACATGTGTGCTCTGTATAGCAGTTTAGATTACATTGTTACTTCTG
CTTCAGTAGGAACTATGGTACTCATATCAGCTGACCGCTATGTAGCCATTTGCGACCCTCTGCATTATCCCACCAAAATTACTATAAAAAGAGTCTCAGTCTCTATTTGTAC
TTGCTGGGCTTGTTCAATTCTGTATAACAGTCTTATCATGAAGGATAATTTCAAGCAGCCAGGGAGGTATAATTCTTGTTCTGGTGATTGTGTAGTTGTCATTGATTACTTT
GTGGGAATTTTTGACTTTGTTTTGACCTTTGTCGGCCCTGTGATTGTCATCATAGTTCTGTATCTAAGAGTATTTGTGGTGGCTGTGTCTCAGGCTCGGGCAATGCGCTC
TCATATCACTGCTCTCAGACTCCAGGGGTCAGAGACGGTGCATGCTAAGAAATCTGAGCTGAAAGCTGCCAGGACTCTCGGTGTACTTGTTATTGCCTTTCTGATATGT
CTTTTCCCATTTTTCTGTTCCTCAATGGTAGGCCAGAATAGTTTCTTTGATATTAGATCTGTACCTTTTGAGCTCTTGCTGTTCTATTTTAACTCTTGTCTGAACCCATTAAT
CTACACTTTTTGCTACCCCTGGTTTCTGAAATCTATCAAGCTCATTGTGACATTTAAGATATTTAGGCATGGCTCCAGTGAGGCCAGTATACTAtag
>contig062677-ZebTARs.A018
ATGGGCACACAGGAAAAATCTGAGCTCTGCTTTCCACAACTCTTCAACATGTCCTGCAAAAAGCCTGCCATTGCTCGGTCCAAAGCTGTTTTCCTTCATATTGTATTGTC
CTCAGTTTCTTTTCTGACAGTGGCTCTCAACTTGCTTGTCATCATCTCAGTCTCCCACTTCAGGCAGCTTCACACACCCACTAACATCCTGCTCCTCTCTCTGGCTGTC
TCAGACTTTCTTGTTGGCCTCCTATTGTCACCTGCAGAAATCCTCCGAAGTACAGCCTGTTGGTTTCTTGGTCAGCCCACATGTTTAATGTATATTTTTGTGTCCTTGACT
GTTACCTCTGCCTCAGTGGGCATTATGGTGCTGATATCAGCTGACCGCTATGTGGCTATTTGTGACCCTTTGAATTACCCCATCAGGATCACTGACAGAAGAGTTCAACT
CTGTGTTTGTCTCTGTTGGCTCTGCTCCATCACCTTCAGCTGTTTCCTTGTAAGAGATGACCTGCATCAAAGAAGAAAACAAAATTCCTGCTATGGAAAATGTGTCGTTG
TTGTTCAATACATTGCAGGAGTTGTTGACCTCATTTTAACCTTCATTGTACCAGTTACTGTCATCATAGTTCTGTATATGAGAGTATTTGTGGTGGCTGTGTCTCAGGCCC
GCTCTATGCGCTCTCATGCTACAGCTGTCAGAAATCACCTTCCAATGACGCTAACAAAAAAGTCTGAACTAAAAGCAGCCAGGACTCTGGGTGTTCTTGTTCTTGTGTT
TCTAATGTGTTTCTGCCCTTATTACTGTGTTTCTCTTGTAGGAGAAGAATTCATCAATAGTTCATCTGCTTCTTTTGTGGCCTATCTGTTTGGTTTAAACTCATGTCTAAAC
CCTTTGATCTATGCTATGTTCTACCCCTGGTTTAGAAAAGCTGTGAAACTAGTTGTCACTCTACAGATACTGCAGCCTGGCTCCAGTGAGGTAAGTATTTTGtaa
>contig062676-ZebTARs.A019
ATGGAGACCCAGGACGAAGCAGAGCTCTGCTTTCCACAACTCTTCAACATCTCCTGCAAGAAGCCTAAAACATCTCTGTCTCAAGTTTTGCTCCCTTACGTTGTGGTGT
TTTCAGTCTCTCTGCTAACTGTGGCTCTCAACCTTCTCGTCATTGTCTCAGTCTCCCACTTCAGACAGCTCCACACACCCACTAACATCCTCCTCCTCTCTCTGGCTGT
CTCAGACTTTCTCATTGGTCTGTTGTTGATGCCAGCAAAAATCCTACGAGACACAGCTTGTTGGTTTCTTGGTCAACTCACATGTTCTCTGTATAGTTATATATGCTTCGT
TGTTACCTCTGCCTCAGTGGGCATTATGGTGCTGATATCAGTCGACCGCTATGTGGCTATTTGTGACCCTCTGCATTACCCCACCAGAATCACTGACAGAAGAGTGAAA
CTCTGCGTCTGTCTGTGTTGGCTCTGCTCGGTTTTTTACAACATACTATTTATAAAGGACGACCTGCTTCAACGAGAACGACATACTTCCTGTTATGGAGAATGTGTATTT
GTCATAGACTACATTGTAGGAACCACTGACATTGTTTTAACTTTTATTGCTCCAGTTACTGTCATCGTATTTCTGTATATGAGAGTATTTGTGGTGGCTGTGTCTCAGGCCC
GTGCCATGCGCTCTCATGTTACAGCTGTCACACTGCAGCTCTCAGTGACTCTAACAGCAAAGAAATCAGAGTTAAAAGCAGCCAGGACTCTGGGTGTTCTTGTTCTTG
TGTTTCTATTATATTTCTGCCCATATTACATTGTTTCATTTTTTGGATACGAGTTGCTCAATAGCTCATCTGCATCCATTGTGATCTATCTGTATTATTTTAACTCCTGTCTAAA
TCCTTTGATTTATGCTATGTTCTACCCCTGGTTTAGAAAAGCTGTGAAATTAGTTGTCACTCTACAGATACTTCAGCCAGGCTCCTGTGAGGTCAGCATACTGtag
>contig061417-ZebTARs.A020
ATGGAGATACAGAAAGGAGCTGAGCTCTGTTTTCCACAACTCCTCAACAGCTCCTGCAGGAAGCCGACGCTTCGCTGGTCCAAAGCTGTGCTCCTGAACATTGTCCT
GTCCTGCATCTCTCTGCTCACTGCTGCTCTAAACCTTCTCGTGATCATCTCAGTCTCCTACTTCAGGAAGCTTCACACACCCAGCAACATCCTCCTGCTCTCTCTTGCT
GTCTCAGACTTTCTCATGGGTCTCTTGCTGATGCCAGCAGAAATCCTTAGAAGCATGACGTGCTGGGTACTTGGTGATCTTATGTGTTCTGTTTATTTTTTTCTGACTGTA
AACATTACCTGTGCTTCAATAGGGAACATAGTTCTTATATCGATTGACCGTTATGTGGCTATTTGTGACCCTCTGCATTACTCCACGAGAATTACTGTGGCAAGAGTCAAA
CTCAGTGTTTGTCTGTGTTGGTTTTATTCAACTTTCTACTGCAGTCTTTATACGCAAGAAATGCTGATTGAACCAGGCAGGTATAATTCCTGTTATGGTGAGTGTGTGCTT
GTCATCAGTGATTTTGCTGGAATGGTTGACCTTATTTTATTTTTTATTCTTCCACTTTCTGTCATCATAGTTCTGTATACAAGAGTATTTGTGGTGGCTGTGTCTCAGGCTC
GTGCCATGCGCTCTCATGTTACGGCTGTCACACTTCAGCGTTCATTGAATCAAACAAACAAATCTGAGCTGAAAGCAGCCAGGACTCTGGGGGTTCTTGTAGTTGTGT
TTCTGTCATGCTACTGTCCATTTTATTGCTACTCTCTCACTGATAAAAATGCTGTCAATGATCCAGCTACATCTTCTGTGATCTTTATCTTTTATTTTAACTCTTGTATAAACC
CTTTGATCTATGCCCTGTTTTACCCCTGGTTTAGAAATGCTGTTAAACTTATTATCACATTGCAGATATTCAAGCATAACTCCTGTGAGGCCAACATACTAtaa
>contig061410-ZebTARs.A021
ATGGATACACAGGATGTGGCAGAGCTCTGTTTTCCACAACTCTTCAACACCTCCTGCAAGAAACCTCCTCTGTCAGAATTTGTGTTCCTTCATGTTGTGTTGTCCTCCAT
CTCGCTGCTAACTGTGACTCTCAACCTGCTCATCATCATCTCAGTCTCCCACTACAGGCAGCTCCACACACCCACTAACATCCTCCTCCTCTCTCTGGCTGTCTCAGAC
TTTCTTGTTGGTCTCCTGTTGATGCCTGGAGAAATCCTCCGAAATACAGCCTGCTGGTTTCTCGGTGACCTCACCTGTTCTATGTACAATTATATGTCTTTAATTGTTACC
TCTACCTCAGTGGGAGACATGGTGTTAATATCAATTGACCGCTATGTGGCTATTTGTGACCCTCTGCATTACCCCACCAGAATCACAGACAGAAGAGTGAAACTCTGTGT
TTGTCTGTGTTGGCTCTGCTCTGTTTTCTATAGCAGCCTGTTTGTAAAGGATGATCTAACTCAACCAGGGAAGCATAATTCCTGCTATGGAGAATGTACAATTGATGTTGA
CTTAATTACAGGAACGATTGACCTTCTTTTAACCTTTTTTGTTCCAGTTACTGTCATTGTAGTTCTGTATCTGAGAGTATTTGTGGTGGCTGTGTCTCAGGCTCGAGCCAT
GCGCTCTCATGTTACAGTTGCTGCTCTGCAGCTTTCAGTGACTCTAACAACAAAGAAATCAGAGTTAAAAGCAGCCAGGACTCTGGGTGTTCTTGTAGTTGTGTTTCTA
CTGTGTTTCTGCCCTTATTATTGTGTTACTCTTGCCAGGGACGACCTACTCAATAGTTCATCTGTATCCTTTTTGCTCTATCTGTTCTATTTTAACTCATGTCTAAACCCTTT
GATCTATGCACTGCTCTACCCCTGGTTTAGAAAAGCTGTGAAACTCATAATCTCTTTACACATACTGCAGCCTGGCTCCTGTGAGATTAGCATTCTGtaa
>contig059768-ZebTARs.A022
ATGGAGAACGTGTATGGACAACAATTCTGCTTTCCACAGCTCCTCAACACCTCCTGTAGGAAGCCGCTGCAACATCACAATCAGACCATTTTTCTCTACATTCTGCTTTG
CTGCATCTCTCTGCTCACAGTGACTCTCAACGTCCTGGTCATCATCTCTATCTCCCACTTCAGGCAGCTTCACAACCAAACCAACCTCTTCCTCCTCTCTCTCGCTGTC

TCAGACCTCCTTGTTGGCCTCCTGCTGATGCCGCCTCGAATCCTCCTTTTAGGGGGCTGCTGGTTTTTGGGCACCTTCATGTGTGGACTGTTTACTATGCCTCTTTTG
TTCTCACATCTGCCTCAGTAGGAAACATGGTGCTTATTTCATTTGACCGGTATGTAGCTATTTGTGACCCCTTGTCCTACCCCACCACAGTTACTGAGAGAAAAGTCCAA
ATCAGTGTCTGTCTGTGTTGGGCCTGTTCACTGCTGTATAACGGCACAATACTAAACAACTTTCTGAAGCAGCCAGACAGATATAATTCTTGTGACGGGGAATGCATAGT
TGTAATTAATTTCATAACTGGTGCTTTTGATGTCGTAGTTACCTTCATCGGCCCCACTGCTGTGATCATATTTCTGTACATGAGAGTATTTCTGGTGGCTGTGTCTCAGGC
TCAGGCTATGAGATCTCATGTTGCCTTTGTTACTTCAAAGGGTTCAGTTCACGTTGCCATTAAGAAGTCTGAGAGAAAAGCAGCTACAACTATTGGTGTTGTTGTGGCT
GTGTTTCTGATGTGTTTCTGTCCTTACTTTTATCCCTCTCTCGCAGGGCAGGACACATCAACCAGTGTGGAATTTTCAGTTTTTGGGGTCTGGTTACTTTATTGTAATTCC
TGTCTAAATCCACTTATCTATGCGTTTTTCTATCCCTGGTTTAGGAAGACTGTTAAACTGATAGTTACATTGCAGATTTTGCAGCCTGACTCTTGTGATGCCAACATACTGta
g
>contig053139-ZebTARs.A023
ATGGAGGAACCTGAACTCTGCTTTCCAAAACTCCTTAATATCTCCTGCATCAGGCCAAAGCGTCCTCACTTTGAGATCATGCTGACTTACATTTTGCTCTCCTTCATTTCT
TTGCTTACTGTGATTCTTAACCTGCTGGTCATTATCTCCATCTCACACTTCAGGCAGCTCCACACCCCCACCAACTTCCTCCTTCTCTCTCTGGCTGTCGCTGATTTCTT
TGTAGGTCTCCTTATGTTCTTTCAAATAGTGCTCATCGATGGATGCTGGTTCCTCGGTGACATCATGTGCACTCTGTATCAGTATCTAGCATTCATTATTACTTCAGCCTCA
GTAGGAACCATGGTGATCATATCTGCTGATAGGTATTTGGCTATTTGTTACCCTCTGCATTACTCCACCCAAATCACACAACAAAGAGTTAATATATGTATAAGTTTGTGTT
GGTTTTTTTCTGTGATCTTTCAGAGTTTGATTGTGAAGGATAACTTGAAACAACCAGGAAAGTATAACTCCTGCATTGGAGAGTGTGTCTTTGTCGTTAACTACATTGCTG
GGCTTTTTGATCTTTTGTTTTCCTTCATTGTTCCCATTACTGTGATTGTAGTTCTGTATCTGAGAGTGTTTGTGGTGGCTGTGTCTCAGGCTCGTGCCATGAGGTGTCAA
CTTGCAGTCACTCACCAGCGATCAGTAACAGTAACTGTTACGAAATCGGAGCTGAAAGCAGCCCGTACTCTTGGTGTTGTTGTAGTTGTGTTTCTTATATGTATGTGCCC
ATATTACTGCGTTGCTCTCACAGGCCAAGATAACTTCCTAAATGCTTCATCTGCTGCCTTTGTAATTTGTTTGGTGTACTTTAACTCTTGCCTAAACCCTATCATTTATGTCT
TTTTTTATCCCTGGTTCAGAAAGTCAATCAAACTTATTGCTACTCTTCAAATACTGCAGCCTGACTCCCGTGAGACTAACATGCATtaa
>contig030471-ZebTARs.A024
ATGGAGATTCAGAAAGCAGCTGAGCTTTGTTTTCCACAACTCCTCAACAGTTCCTGCAGGAAGCCGACACTTCATTGGTCCAAAGCTGTGCTCCTGAACATTGTGCTG
TCCTGCATCTCTCTGATCACTGCTGCTCTAAACCTCCTGGTCATCATCTCAGTCTCCCACTTCAGGAAGCTTCATACACCCAGTAACATCCTGCTCCTCTCTCTGGCCG
TCTCAGACTTTCTTGTGGGTCTTCTGCTGATGCCATTAGAGATCTTTAGAAACACAGCTTGCTGGGTACTTGGTGATCGTATGTGTTCTGCTTATTGGTATTTGACCATCA
ACATTATCTGTGCTTCAATAGGGAACATTGTTCTAATATCAGTTGACCGCTATGTGGCTATTTGTGACCCTCTGCATTATCCCAGCAGAATTACTTTGGCGAAAGTCAAAC
TCAGTGTTTGTCTCTGTTGGTTTTATGCTTTTTTCTACAGCAATCTTTATACAAAGAATATCATGATTGAACCAGGCAGGTATAATTCTTGCTTTGGAGAGTGTGTATTTTTT
AGCAGCAATATTGCTATTGTTGCTGACCTTATTTTATTCTTTTTTGTTCCAGTAACTGTTATCATAGCCTTGTATATGAGAGTATTTGTGGTGGCTGTGTCTCAGGCTCGTG
CCATGCGCTCTCATGTTACATCTGTCACACTTCAGTGTTCACTGAATCAAGCAAACAAATCTGAGCTGAAAGCAGCCAGGACTCTGGGGGTTCTTGTAGTTGTATTTTT
GGCATGCTACTGTCCATTTTACTGCTACGCTCTGGTTGATAAAAATGTGGTCAATGATTCATCTGCATCTTTTGTGGTTCTTGTCTTTTATTTTAACTCTTGTTTAAACCCA
CTGATCTATGCTCTGTTTTACCCCTGGTTTAGAAAAGCTATTAGATGTGTTATCACTCTGCTGATATTTAAACATGACAGCAGTGAGGTCAACATAATAtag
>contig030464-ZebTARs.A025
ATGCAGAAAGAAGCCGAGCTTTGTTTTCCACAACTCCTCAACAGCTCCTGCAGGAAGCCAACATTTCACTGGTCCAAAGCTGTGCTGCTGAACACTGTGCTGTGCTG
CATCTCTCTGCTCACTGCTGCTCTAAACCTTCTCGTCATCATCTCAGTCTCTTACTTCAGGCAGCTTCACACACCCACTAACATCCTCCTCCTTTCTCTGGCTGTCTCAG
ACTTTCTCACTGGTCTCCTTATGCTGCCACTAGAAATAATTAGAAACACGGCCTGCTGGGTACTTGGTGATCTTATGTGTTCTGTTTATACTTATCTGACAGCCGGTCTTT
TCTGTGCTTCAACTTGGCACATAGTTCTCATTTCAGTTGACCGCTATGTAGCTATTTGTCACCCTCTTCATTACCCCACCAGAATTACCGTGGCAAGAGTCAAACTCTGT
GTTTGTTTGTGTTGGTTTTGTTCAACCTTCTACGGCAGTCTTTTAACAAAGGATGTTCTAATTAAGCCGGCCAGGTATAATTCCTGTTATGGAGAGTGTGTGTTTGTCATT
AATGATATTGCAGGGTTTATTGATCTTGTGTTATCTTTTATTTTTCCAGTATCTGTTATCATAGTTCTTTATATGAGAGTGTTTGTGGTGGCTGTGTCTCAAGCTCGTTCCAT
GCGCTCTCATGTTACAGCTGTCACACTTCAGCGTTCAAGCAATCAAGCAAACAAATCTGAACTGAAAGCAGCCAGGAACCTTGGAGTTCTTGTAGTTGTGTTTCTCGCA
TGTTACCTTCCGTTTTACTGTTACTCTCTTATTGAAGTAAATGTGATCAATGACTCATCTACATTTTTTCTGATCATTGTGTTTTACTTTAACTCGTGTCTAAACCCACTGAT
GTATGCCCTTTTTTACTCTTGGTTTAGAAATGCTGTTAAACTTATTATCACTCTGCAGATTTTTAAGCATGACACTAGTGAAGCAAACATGttttaa
>contig030445-ZebTARs.A026
ATGGATACTCAGGATGGAGCAGAGCTCTGCTTTCCACAACTCTTCAACATCTCCTGCAAGAAACCTACAACTCCTCTGTCTCAAGTTTTGCTCCTTTATATTGTTGTGTC
CTCAATGTCTCTGCTGACTGTGACTCTCAACCTGCTCGTCATCATTGCAGTCTCCCACTTCAGGCAGCTCCACACACCCACTAACATCCTGCTCCTTTCTCTGGCTGTC
ACTGACTTTCTCGTTGGTCTTCTGTTTATGCCTGGAGAAATCCTGCGAAATACAGCGTGTTGGTTTCTTGGTCAGCTCACATGTTCTCTGTATAATTATGTATCCTACATC
ATTACCTCTGCCTCAGTGGGCAACATGGTGCTGATATCAGTCGACCGCTATGTGGCTATTTGTGACCCTCTGCATTACCCCACCAGAATCACAGAGAGAAGAGTGAAAC
TTTGTGTTTGTCTGTGTTGGCTCTGCTCTGTTTTCTACTGTTATGTGATTTTAATAGATGATCTAAGTCAACCAGGCAAGCATAATTCTTGCTATGGAAGATGTATAATTATC
ATTGAATTTATTGCAGGATTTGTTGACCTTTTTTTATCCTTTATTATTCCACTTACTGTCATCATAGTTCTGTACATGAGAGTGTTTGTGGTGGCTGTGTCTCAGGCCCGTG
CCATGCGCTCTCAGGTTACAGCTGTCACACTGCAGCTCTCAGTGACTCTAACAGCAAAGAAATCAGAGTTAAAAGCAGCCAGGACTCTGGGTGTTCTTGTTCTTGTGT
TTCTATTATGTTTCTGTTCATATTACTTTATTTCACTTTTTGGAAACGATTTGCTCAATAGCTCATCTGCATCGATTGTGATCTATCTGTACTATTTTAACTCCTGTCTAAACC
CTTTGATTTATGCTATGTTCTACCCCTGGTTTAGAAAAGCTGTGAAACTAATTGTGACTCTACAGATACTGCAGCCTGGCTCCTGTGAGGTCAGCGTACTGtag
>contig066330-ZebTARs.A027
ATGGAGGAAGCTGAACTCTGCTTTCAACAACTCTTTAACACCTCCTGCATGAGGCCCAGGCGTCCACACTTTGAGATCATGCTGACTTATATTTTGCTGTCCTTCATTTC
TCTGCTTACTGTGATTCTTAACCTGTTGGTCATCATCTCTGTCTCACATTTCAGGCAGCTCCACACCCCCACCAACCTCCTGCTCCTTTCTCTGGCTGTCGCTGATTTCT
ATGTGGGCCTCCTCTTGTTCTTCCAAATTGTGCTCATAGATGGCTGCTGGTTCCTCGGTGACATCATGTGCACTCTGTATCAATACCTAGCATATGTCATCACCTCGGCC
TCCATAGGAACCATGGTGATCATATCTGTTGATCGATATTTGGCTATTTGTTACCCTCTGCATTACTCCACCAAGATCACACAACAAAGAGTTAAAATTGTTGTCTGTTTGT
GTTGGATCTGTTCTGTCATCTTTCAAAGTCTGATTCTGATGGATAACCTGGAGCAACCAGGCAGGTATAACTCTTGCATTGGAGAGTGTGTCTTTGTCATTAATTACATCG
CAGGACTTGTTGATGTTACTTTTTCCTTTATTGTTCCCTTTACTGTGATTGTAGTTTTGTATCTGAGAGTGTTTGTGGTGGCTGTGTCTCAGGCTCGTGCCATGAGGTCT
CAGCTTGCAGTCACTCACCAGCGATCAGTAACAGTAACTGCAAAGAAATCGGAGCTGAAAGCAGCCTGGACTCTTGGTATTGTTGTTGTTGTGTTTCTCATATGTATGT
GTCCATATTACTGTGTGGCTCTCACAGGCCAAGACAGCTTGCCAAGTGCTTCATCACTGACATTTGTTCTATGCTTAGCCTACTTTAACTCATGTCTGAACCCCATTATAT
ATGTCTTTTTCTACCCCTGGTTCAGAAAATCTATAAAAGTCATTGTTACTCTTCAGATACTGCAGCCTGACTCTTCTAAAGTCACCATGcgttag
>contig053145-ZebTARs.A028
ATGGAGGAAGCTGAACTCTGCTTTCAACAACTCTTTAACACCTCCTGCATGAGGCCCAGGCGTCCACACTTTGAGATCATGCTGACTTATATTTTGCTGTCCTTCATTTC
TCTGCTTACTGTGATTCTTAACCTGTTGGTCATCATCTCTGTCTCACATTTCAGGCAGCTCCACACCCCCACCAACCTCCTGCTCCTTTCTCTGGCTGTCGCTGATTTCT
ATGTGGGCCTCCTCTTGTTCTTCCAAATTGTGCTCATAGATGGCTGCTGGTTCCTCGGTGACATCATGTGCACTCTGTATCAATACCTAGCATATGTCATCACCTCGGCC
TCCATAGGAACCATGGTGATCATATCTGTTGATCGATATTTGGCTATTTGTTACCCTCTGCATTACTCCACCAAGATCACACAACAAAGAGTTAAAATTGTTGTCTGTTTGT
GTTGGATCTGTTCTGTCATCTTTCAAAGTCTGATTCTGATGGATAACCTGGAGCAACCAGGCAGGTATAACTCTTGCATTGGAGAGTGTGTCTTTGTCATTAATTACATCG
CAGGACTTGTTGATGTTACTTTTTCCTTTATTGTTCCCTTTACTGTGATTGTAGTTTTGTATCTGAGAGTGTTTGTGGTGGCTGTGTCTCAGGCTCGTGCCATGAGGTCT
CAGCTTGCAGTCACTCACCAGCGATCAGTAACAGTAACTGCAAAGAAATCGGAGCTGAAAGCAGCCTGGACTCTTGGTATTGTTGTTGTTGTGTTTCTCATATGTATGT
GTCCATATTACTGTGTGGCTCTCACAGGCCAAGATAACTTGCCAAGTGCTTCATCACTGACATTTGTTCTATGTTTGGTCTACTTTAACTCATGTCTGAACCCCATTATATA
TGTCTTTTTCTACCCTTGGTTCAGAAAATCCATAAAAGTCATTGTTACTCTTCAGATACTGCAGCCTGACTCCTGCCAGGCCACCGTGCTTtag
>contig030440-ZebTARs.A029
ATGGATACCCAGAGCAGAGCAGAGCTCTGCTTTCCACAACTCTTCAACACCTCCTGCAAGAAGCCTACAACTCCTCTATCACAAGTTTTGCTCTTTCACATTCTGATGC
CATCAATGTCTCTGCTGACTGTGACTCTCAACCTGCTTGTCATCATCGCAGTCTCCCACTTCAGGCAGCTCCACACACCCACTAACATCCTACTCCTCTCTCTGGCTGC
CGCTGACTTTCTCATTGGCCTTCTGTTTATGCCTGGAGAAATCCTGCGAAATACAGTGTGTTGGTTTCTTGGTCAGCTCACATGTTCTCTGTATAATTATATATCCTTCGTT
GTTACCTCTGCCTCAGTGGGCGATATGGTGCTGATATCAGTCGACCGCTATGTGGCTATTTGTGACCCTCTGCATTACCCCACCAGAATCACAGAGAGAAGAGTGAAAC
TCTGTGTTTGTCTGTGTTGGCTCTGCTCGGTTTTCTACTGCTATATGATTGTAAGAGATGATCTAAGTCAACCAGGAAAGCATAATTCTTGCTTTGGAGAATGTATAATTTT
TGTTGAATTCATTGCAGGATTTGTTGACCTTGTTTTATCCTTTATAATTCCAGTTACTGTGATTGTATTTCTGTATATGAGAGTGTTTGTGGTGGCTGTGTCTCAGGCCCGT
GCCATGCGCTCTCAGGTTACAGCTGTCACACTGCAGCTCTCAGTGACTCTAACAGCAACTAAATCAGAGTTAAAAGCAGCCAGGACTCTGGGTGTTCTTGTTCTTGTG
TTTCTAATATGTTTCTGCCCTTATTACATTGTTTCGCTTTTTGGAGACGAGTTTCTCAATAGTGCCTCTGCATCTATTGTATTCTATTTCTTTCTTTTAAACTCCTGTCTAAAC
CCTTTGATTTATGCTATGTTCTACCCCTGGTTTAGAAAAGCTGTGAAACTAATTATGACTCTACAGATACTGCAGCCTGGCTCCTGTGAAGTCAGCATACTGtag
>contig030465-ZebTARep.A009
ATGGAGATACACGAAGGACTAGAGCTCTGTTTTCCACAACTCCTCAACGATTCCTGCAGAAAGCCAACACTTCACTATTCCACAACTATGCTGCTGTATATTGTGATGTC
ATGCATCACTGCTGCTCTAAACCTTCTGGCCATCATCTCAGTCTCCCACTTCAGGCAGCTTCGCACACCCAGCAACATACTCCTCCTCTCACTGGCTGTTTCAGACTTT
CTCGTGGGTCTCCTGTTGATGCCGTTACGAATTTTAAAAAGCACCACCTGCTGGATACTTGGTGATTTTAATTGTTTTCTTTATTGGTATCTGACTGGCAACATTACGTGT
GCTTCAATAGGGAACATAGTTCTAATATCAGTCCGCTATGTGGCTATTTGTGACCCTTTACATTACTCCACCAGAATTACTGTGGCAAAAGTTAAACTC
>contig037879-TilTAR.B060
ATGCATCCCTGCTATGAATCAGAAAATGCCACTTATACTTTTACAAACAACCCTTCCATTGCCTGTGTTATATTGTATATTTTCCTTGGTTCATTGTCTGTTGTCACAATATT
CGGAAACCTTCTTGTAACAATTGCCATAATTTACTTCAAACAGCTTCACACGCCTACTAACTACCTCATCCTGTCTCTGGCTGTGGCTGACCTGCTTGTAGGAGTTTTAG
TTTTCCCTTTCAGCATGGCCTTCACTGTTACTTTGTGTCTGGATCATGAAGATTTATTTTGCAAAATACGCGATAGCTTTGATGTGTCACTATGCACATCTTCTATTCTAAA
CTTGTGCTGCATTTCCATAGAGAGATATTATGCAGTTTGTCAGCCTCTGAATTACAGATCAAAAATAAATACTCAAGTTACAATGTTTATGATACTGGTGAGCTGGGGCATT
TCTGTATTAATTGGAATTTGCATTATAATTGCAGGATTCAGCCAAGGTATGTGTGAAGAAGTGTGCTCTGTTGATGTTGTAATTGCAAACACTATGGGACCTGTTTTCTCAT
TTTACCTTCCAGCAATCATTATGCTTTGTATATACCTAAAGATTTTCCTTGTTGCACAGAAACAAGTAAGCACCATCCAAAATGCAATCTGTACAAAATCTGGAGCAACTAT
TAGTAAGCTAGAAAGAAAGGCCACAAAAACGCTGTCTATTGTCATGGGAGTGTTTCTTTTATGTTTGAGTCCTTACTTTATTTGTGTTGTCTTTCAGCCTTTAGCTCATAAT
CCCCCACCGATTCCTGTGATTGAAACACTTAACTGGCTTACGTTGTCAAATTCAATGTTAAATCCATTAATTTATGCTTTCTTTTATAGCTGGTTTAGGTCAGCTTACAGAT
TGATTGTTTCTGGAACAATACTTCATTGTAATTTGGCTAACTCAAAATTGTTTtaa
>contig037889-TilTAR.B061
ATGACCATCATTGTAAATGATCTACATCCCTGTTATGTAATACAAGACTCCAAATACATGCTGACAAACAGCCCTTCCATTATATGTGTATTCTTGTACGCACTCCTTGCATT
ATTGTCTGTTATAACAATATGTGGAAATCTTCTTGTAATAATTTCTGTGATTTACTTCAAACAGCTCCACACTCCTACTAACTCTCTCATCCTCTCTCTTGCTGTGGCTGAC
CTGCTCATTGGGATTATAGCATTTCCTCTCAGCATGGCATTCTCTCTCAGCTCATGTCTTTATCATGAGGGTTTGTTTTGCAAAGTGCGAGGCACCTTTGATATTTCACTG
AGCACATGTTCCATCCTGAATCTCTGCTGTATTTCTATTGACAGATATCATGCTGTTTGCCAGCCTCTGACTTATCAAACTAAAATCAGCCATCGTGCTGTTGTGTTCATG
ATCCTGATGAGCTGGGGGGTTTCTGGGATAATTGGAATTAGTGTCACCATTGCTGGATTAAATAATGAAAAATATGAGGAATCATTTTTGATTGACGTTCTAATGGAAAGC
ACTGTTGGACCCATGTTATCATTTTACCTGCCAGTGATCATAATGCTTTGTATCTACCTAAAGATTTTCTTTGTTGCACGGAGGCAAGCACGCAGCATCCAGAACACAAC

AAAGTGTGGACAAACTGCCAGCATGATGGAGAGAAAGGCGACCAAAACTCTGGCTATTGTTTTGGGAGTTTTTATATTATGCTGGAGTCCTTTTTTTCTTTGCATCACAT
TTCCACCATTTACCAGTGATTACCAGTACCAGTTACAGGTACCAGTTCCTGTAATTGAAACTCTTAACTGGCTTACATTGGCAAATTCAATGCTAAATCCATTTATTTATGCT
TTCTTTTACAGCTGGTTCAGGTCAGCCATCAGAATGATTATTTGTGGGAAAATATTCCAAGGGGATTTTGCAAACACAAAATTGACTtga
>contig002574-TilTAR.B062
ATGACCAACATTGTAAATGATCTACATCCCTGTTATGTAATACAAGACTCCAAATACATGCTGACAAACAGCCCTTCCATTATATGTGTATTCTTGTATGCATTCCTTGCATT
ATTGTCTGTTATAACAATATGTGGAAATCTCCTTGTAATAATTTCTGTGATTTACTTCAAACAGCTCCATACTCCTACTAATTCTCTCATCCTCTCTCTTGCTGTGGCTGACC
TGCTCGTTGGGATTATAGCATTTCCTCTCAGCATGGCATTCTCTCTCAGCTCATGTCTTTATCATGAGGGTTTGTTTTGCAAAGTGCGAGGGACCTTTGATATTTCACTGA
GCACATGTTCCATCCTGAACCTCTGCTGTATTTCTGTTGACAGATACCATGCTGTTTGCCAGCCTCTGACATATCAAACTAAAATCAGCCATCGTGTTGTTGTGTTCATGA
TCCTGATGAGCTGGGGGGTTTCTGGGATAATTGGAATTAGTGTCACCATTGCTGGATTTAATAATGAAAAATGTGAGGAATCATGTTTGATTGACGTTCTAATGGAAAGC
ACTGTTGGACCCATGTTATCATTTTACCTGCCAGTGACCATAATGCTTTGTATCTACCTAAAGATTTTCTTTGTTGCTTTGAAGCAAGCAAGCAGCATCCAGAACACAACA
AAGTGTGGACAAACTGCCAGTATCATGGAGAGAAAGGCAACCAAAACTCTGGCTATTGTTTTGGGAGTTTTTATATTTTGCTGGAGTCCTTGTTTTCTTTGCATCACATT
CCCACCATTTACCAGTGATTCTGTACCAGTTCCTGTAATTGAAACTCTTAACTGGCTTGCATTGGCAAATTCAATGCTAAATCCATTTATTTATGCTTTCTTTTATAGGTGGT
TCAGGTCAGCCATCAGAATGATTATTTGTGGGAAAATATTCCAAAGGGATTTTGCCAACACAAATATGACTTGA
>contig037900-TilTAR.B063
ATGGCACCAGATGTCCCTGTCAACCGCACTGATGCTGATGACTTGCACCCTTGTTATAAAATAGATAAGGGCTCCTACATAGTAAAAAGCAACCCTTCCACACTCTGTGT
CTTATTGCACATTTTTCTGGGATCATTATCTCTTGTAACTGTGTGTGGAAATCTTCTGGTAATAATCTCCATCATTTATTTCAAACAGCTCCACACTCTCACTAACTCTCTGG
TCCTCTCTCTGGCTGTGGCTGACCTGGTTGTTGGAGTTTTAGTGTTTCCTTTGAGCATGAGATTCACTGTTAGTGTGTGTCTGTTTTATCAAGATTTATTCTGCAAAGTAC
GAGACAGCTTTGATGTAACCTTGAGCACATCTTCCATTTTGAATTTGTGTTGTATTTCCATTGACAGATACTATGCTGTGTGTAGGCCTCTGACATACAGAAGTAAGATTAA
TGTTCATGTTGTTGTGATCATGATCCTGGTAAGCTGGAGTGTTTCTATTTTAGTTGCTGTTGGCTTTCTTATTGCAGGATTAAACCATGAAAAATGCAAAGAAGGCTGGAT
TTCTGAACTTCTACTTGCAAATATTTTGGGACCTGTGCTCTCATTTTATCTCCCTGTGATAATAATGCTCTGTATCTACCTGAAGATTTTCCTTGTTGCAGAGAAACATGCT
CGCAGCATCCAGAACACAAAGTGTGGTGCCACTGTGGTGAGTAAAATGGAGAAGAAGGCCACTAAAACTCTGGCAACTGTTATGGGAGTTTTTGTGATGTGTTGGCTG
CCTTTCTTTTTCTGTGTCACTCTTCAACTACTGGGTCATATATTAATACCACTTCCAGTGTATGAAACACTTAATTGGCTGGCACTGTGTAATTCAATGCTAAATCCCTTTAT
TTATGCTTTCTTTTAA
>contig037890-TilTARe.B064
ATTGTCAATGATTTACATCCATGTTATGTAATACAGGGCTCCAAATACATGCTGACAAACAGCCCTTCCATTATATGTGTATTCTTGTATGCATTCCTTTCAATATTTTCTGTT
ATAACAATATGTGGAAATCTTCTTGTAATAATTTCTGTGATTTACTTCAAACAGCTGCACACTCCTACTAACTCTCTCATCCTCTCTCTTGCTGTGGCTGACCTGCTCGTTG
GGATTATAGTCTTTCCTCTCAGCATGGCATTCTCTCTCAGCTCATGTCTTTATCATGAGGGTTTGTTTTGCAAAGTGCGAGGCACCTTTGATATTTCACTGAGC
>contig039639-TilTAR.A001
ATGGACGGCTCTGGAGGTCCTCCCCTCTGCTTCCCCAACCTCAACTCCTCCTGCAGGCGGCTGCTGCGACCCACCTCCCAGGCCGCTCTTCTCTACACCCTGATGG
CTTCAGTCTCACTGCTCACTGTGGTGCTCAACCTGCTCGTGGTTGTCTCCATCTCCCACTTCAAGCAGCTCCACACTCCGACCAACACCCTGCTCCTGTCCCTGGCC
ATGTCCGACCTGCTGGTGGGGCTGCTGGTGATGCCCATCGAGGGCCTGCGCTACATCGAGACGTGCTGGCTGCTGGGGAGGCTGATGTGTGCTCTCAGTCCTTATT
TGTCTTACTGCCTCATCTCTGTCTCTCTGGGCAGCATGGTGCTCATATCTGTAGATCGCTATATAGCCATCTGTGACCCTCTGCTCTATTCCTCAAAGATCACAGTGAACA
GAGTGAAGCTTTCAGTCTGTGTCTGCTGGGCCTGCTCTCTTCTCTACAATGGCTGCATTCTCATGGAACACTTAGGGTGGCCGGACAGGTTCAGCTCCTGTCACGGG
GAGTGTGTTGTGTTCATCAGCCGAACTTCAGGAACAGTAGATTTCTTTTTGTCATTTGTCGGCCCCTGTGGCGTAATGTTTGTTCTGTACATGAGGGTGTTTGTGGTTG
CTGTTTCTCAGGTGCGTGCCATTCAGTCGCAGGTGGCTGTGAGAGCAGCTCCAGCTGCTAAAAAATCAGAGTTGAAGGCAGCCAGGACACTCGGGATTGTGATAGTT
GTTTTTCTAATGTGCTCCTGCCCTTATTATTATCCTTCCTTTGCAGGTGACGACACTTCTATGAGCTTGCCTTATTACACCTTGCTGTTTTGGATGGTGCTGACAAACTCT
TGTGTGAACCCTGTGATTTATGTACTGTTCTACCTCTGGTTTAGAAGAGCTATCAGATTCATCGTCACCCTCAGAATACTGCAGCCTCACTCCAGAGAGGTCAACATCCT
Gtag
>contig039640-TilTAR.A002
ATGGACGGCTCTGGAGGTCCTCCCCTCTGCTTCCCCAACCTCAACTCCTCCTGCAGGCGGCTGCTGCGACCCACCTCCCAGGCCGCTCTTCTCTACACCCTGCTGG
CTTCAGTCTCACTGCTCACTGTGGTGCTCAACCTGCTCGTGGTCATCTCCATCTCCCACTTCAAGCAGCTCCACACCCCGACCAATGCCCTGCTCCTGTCCCTGGCC
GTGTCCGACCTGCTTTTGGGGTTGCTGGTGATGCCCATTGAGGGCCTGCGCTACATCGAGACGTGCTGGCTGCTGGGGAGGCTGATGTGTGCTCTCAGTCCTTATTT
GTCTTACTGCTTCCTCTCTTTTTCTCTGGACAGCATGGTGCTCATATCTGTAGATCGCTATATAGCCATCTGTGACCCTCTGCTCTATTCCTCAAAGATCACAGTGAACAG
AGTGAAGCTTTCAGTCTGTTTCTGCTGGGTCTGCTCTCTTCTCTACAATGGCTGCATTCTCATGGAACACTTAGGGTCGCCGGACAGGTTCAGCTCCTGTCACGGGGA
GTGTGTTGTGTTCATCAGCTACACTTCAGGCACAGTAGATCTCTTTTTGTCATTTGTTGCCCCCTTTGCTGTAATGTTTGTTCTGTACATGAGGGTGTTTGTGGTTGCTG
TTTCTCAGGTGCGTGCCATTCAGTCACAGGTGGCTGTGAGAGCAGCTCCAGCTGCTAAAACATCAGAGTTGAAGGCAGCCAGGACACTCGGGATTTTGATAGCTGTG
TTTCTAATGTGCTTCTGCCCTTATTATTATCCTTCCTTTACAGGTGATGACACCTCTACAAGCTTGCCTTATTACGCTGTGCTGTTTTGGATCATGCTAATAAATGCTTGTGT
GAACCCTGTGATTTATGTTCTGTTTTACCCCTGGTTTAGAAGAGCTATCAGATTCATCGTCACCCTCAGAATACTGCAGCCTCACTCCAGAGAGGTCAACATCCTGtag
>contig039640-TilTAR.A003
ATGGACGGCTCTGGAGGTCCTCCCCTCTGCTTCCCCAACCTCAACTCCTCCTGCAGGCGGCTGCTGCGACCCACCTCCCAGGCCGCTCTTCTCTACACTCTGCTGG
CTTCAGTCTCACTGCTCACTGTGGTGCTCAACCTGCTCGTGGTCATCTCCATCTCCCACTTCAAGCAGCTCCACACCCCGACCAACGCCCTGCTCCTGTCCCTGGCC
GTGTCCGACCTGCTGGTGGGGCTGCTGGTGATGCCCATCGAGGGCCTGCGCTACATCGAGACGTGCTGGCTGCTGGGGAGGCTGATGTGTGCTCTCAGTCCTTATT
TGTCTTACTGCCTCATCTCTGTCTCTCTGGACAGCATGGTGCTCATATCTGTAGATCGCTATATAGCCATCTGTGACCCTCTGCTCTATTCCTCAAAGATCACAGTGAACA
GAGTGAAGCTTTCAGTCTGTGTCTGCTGGGTCTGCTCTCTTCTCTACAATGGCTGCATTCTCATGGAGCACTTAAGGTGGCCGAAGAGGTTCAGCTCCTGTCATGGGG
AGTGTGTGGTGTTCATCAGCTACACTTCAGGAACAATAGATCTCTTTTTGTCATTTGTCGGCCCCTGTGCCTTAATGTTTGTTCTGTACATGAGGGTGTTTGTGGTTGCT
GTCTCTCAGGTGCGAATCATTCGGTCGCAGGTGGCTGTGAGAGCAGCTCCAGCTGCTAAAAAATCAGAGATGAAGGCAGCCAGGACACTCGGGATTGTGATAGCTGT
GTTTCTAATGTGCTTCTGCCCTTATTATTATCCTTCCCTTGCAGGTGTGGACACCTCTACGAGCTTGCCTTATTATGCTCTATTCTCTTGGATAATGCTGACAAACTCTTGT
GTGAACCCTGTGATTTATGCTCTGTTCTACCCCTGGTTTAGAAGAGCTATCAGACTCATCGTCACCCTCAGAATACTGCAGCCTCACTCCAGAGAGGTCAACATCCTGta
g
>contig039642-TilTAR.A004
ATGGACGGCTCTGGAGGTCCTCCCCTCTGCTTCCCCAACCTCAACTCCTCCTGCAGGCGGCTGCTGCGACCCACCTCCCAGGCCGCCCTTCTCTACACTCTGCTGG
CTTCAGTCTCACTGCTCACTGTGGTGCTCAACCTGCTCGTGGTCGTCTCCATCTCCCACTTCAAGCAGCTCCACACCCCGACCAACGCCCTGCTCCTGTCCCTGGCC
GTGTCCGACCTGCTGGTGGGGCTGCTGGTGATGCCCATCGAGGGCCTGCGCTACATCGAGACGTGCTGGCTGCTGGGGAGGCTGATGTGTGCTCTCAGTCCTTATT
TGTCTTACTGCCTTATCTCTGTCTCTCTGGGCAGCATGGTGCTCATATCTGTAGATCGCTATATAGCCATCTGTGACCCTCTGCTCTATTCCTCAAAGATCACAGTGAACA
GAGTGAAGCTTTCAGTCTGTGTCTGCTGGGCCTGCTCTCTTCTCTACAATGGCTGCATTCTCATGGAACACTTAGGGTGGCCGGACAGGTTAAGCTCCTGTCACGGG
GAGTGTGTTGTGTTCATCAGCCGAACTTCAGGAACAGTAGATTTCTTTTTCTCATTTCTCGGCCCCTGTGGCGTAATGTTTGTTCTGTACATGAGGGTGTTTGTGGTTG
CTGTTTCTCAGGTGCGTGCCATTCAGTCACAGGTGGCTGTGAGAGCAGCTCCAGCTGCTAAAATATCAGAGTTGAAAGCAGCCAGGACACTCGGGATTTTGATAGCTG
TGTTTGTAATGTGCTTCTGCCCTTATTATTATCCTTCCCTTGCAGGTGTGGACACCTTTCTGAGCTTGCCTTCTTACGCCATGGTGTGTTGGATCATGCTTACAAATGCTT
GTGTGAACCCTGTGATTTATGTTCTGTTTTACCCCTGGTTTAGAAGAGCTATCAGATTCATCGTCACCCTCAGAATACTGCAGCCTCACTCCAGAGAGGTCAACATCCTG
tag
>contig023443-TilTAR.A005
ATGATGGACAGCCAGGACAGAACTGAACTCTGCTACCCTGAGCTTGCGAATTTGTCTTGCAGGGGTTTAACACGTCCTCAGCCTGAGGCTGTCCTCCTCTATACACTG
TTCTTGTCCATCTCTGTGCTAACTGTAGCTCTGAATGTGCTGGTTATCATATCCATCTCTCATTTCAGGCAGCTTCACATGCCCACCAATGTCCTCCTGCTCTCTCTTGCT
ATCTCAGACCTCTTGGTCGGGCTGCTTGTGATGCCGGTGGAAACAATGCGGTTTATAGAAACCTGCTGGCTGCTGGGAGATCTCATGTGTGCTTTGTCTTACATTATCG
GCTTCACTCTCACCTCAGCCTCTGTGGGCAACATGGTGCTCATATCAATTGATCGCTACGTAGCTATTTGTTATCCTCTGCAGTACCCCACAAAAATCACTCACAGCAGA
GCGGAGCTGTCTGTGACTCTGTGCTGGGCTTGTTCGCTCCTCTACAACGGGCTGATCTTAAAGGAGCATCTCAGGCAGCCAGACAGGCATAACACCTGTCACAGTCA
GTGTTTGGTGGTGATTAACTATGTCTCTGGAGCCATTGACCTTGTCTTTACTTTCATCGGCCCTTGCTCAGTCATTATCATTCTGTACATGAGAGTATTTGTTGTAGCGGT
GTCTCAGGCTCATGCCATGCGGTCTCATATTACAGCTGCTGCAGTAAGAATTACCGCAAAGAAATCAGAGAAAAAGGCAGCCAGGACTCTTGGTGTTGTGATATTTGTG
TTTTTGCTGAGTTTCTGTCCGTATTACTATCCCTCTCTTGCAGGGCAGGACATTTCAAACAGTGCTTCTTCTTGGGCTATTGTTTCCTGGATGCTGTACTTCAATTCTTGT
CTGAATCCACTGATATATGCTTTGTTCTACCCGTGGTTTAGAAAAGCTATCTGGTTCATTGTCTCCCTGAAGATACTAGAAAAGGGCTCCTCTCAGGCAAATATACTTtaa
>contig022321-TilTARe.A006
TGTGTAATTTATAATTTTCTGTCTCCCTGCAGGCAGCTCCACACTCCCAGTAACATCCTCCTCCTCTCTCTGGCTGTCTCTGACTTTTTTGTCTGTTTTTTGTTGATGCCG
GTAGAAATCTTCAGAAGCACGGCCTGCTGGGTATTTGGTGATCTCATGTGTTCACTTTATATTTATCTGAGCAGCCTTTTTATCAATGCTTCATTTGAAATTATTGTTCTTAT
ATCTGTTGACCGCTATGTGGCTATCTGTGACCCTCTGCATTACCCCACCAGAATGACTGTGGCAAGAGTCAAACTCAGTGTTTGTCTGTGTTGGTTTTATGCTATTTTTT
ACTGCAGTCTTTACACAAAGGATGTCTTGATAAAACCAGGCAGATATGGCTCCTGCTATGGAGAGTGTGTGTTTGTTATTGATGATATAACTGGAACTGTTGACCTTGTTT
TATCTTTTATAGTTCCAGTTACGATCATTATAGTTCTGTATACAAGAGTGTTTGTGGTGGCTGTGTCTCAAGCTCATACCATGCGCTCTCATGTCACAGCAGTCACACTAC
AGCGTTCACTGAATCAAGCAAACAAATCTGAGCTGAAAGCAGCCAGGAACCTTGGGATTCTGGTAGTTGTGTTTCTGTCATGCTACTGTCCATATTATTGCTACTCTCTT
GTTGATGAAAATGCTGTCAATGATCCAGCTGCATCTTTTGTGGTCTTTATCTTTTATTTTAACTCTTGTATAAACCCTTTGATGTATGCCCTGTTGTACCCCTGGTTTAGAAA
TGCTGTTAAACTTATTATCACACTCCAGATATTCAAGCATAACACCTGTGAGGCCAACATAatataa
>contig022322-TilTARe.A007
CTCTCTCTGGCTGTCTCTGACTTTTTTGTGGGTTTTCTGTTGATGCCGGTAGAAATCTTCCGAAGCACGGCCTGCTGGGTATTTGGTGATCTCATGTCCTCACTTTATAT
TTATCTGAGCATCATTCTTATCAATGCTTCATTGGAAATTATTGTTCTTATATCAGTTGACCGCTATGTGGCTATTTGTAACCCTCTGCATTACCCCACCAGAATCACTGTGC
CTAGAGTCAAACTCAGTGTTTGTCTGTGTTGGTTTTATGCTATTTTTTACTGCAGTCTTTACACAAAGCATGTCCTGATAAAACCAGGCAGGTCTGGTTCCTGCTATGGA
GAGTGTGTGTTTGTTATTGATGATATAACTGGAACTGTTGACCTTGTTTTATCTTTTATAGTTCCAGTTACCATCATTGTAGTTCTGTATATGAGAGTATTTGTGGTGGCTGT
GTCTCAGGCTCGTGCCATGCGCTCTCATGTCACAGCAGTCACACTACAGCGTTCACTGAACCAAGCAAACAAATCTGAGCTGAAAGCAGCCAGGAACCTTGGGATTC
TTGTAGTTGTGTTTCTGTCATGCTACTGTCCATTTTATTTCTACTCTCTTGTTGATGAAAATGCTGTCAATGATCCAGCTGCATCTTTTGTGGTCTTTATCTTTTATTTTAACT
CTTGTATAAACCCGTTGATCTATGCCCTGTTGTACCCCTGGTTTAGAAATGCTGTTAAACTTATTATCACACTCCAGATATTCAAGCATAACACCTGTGAGGCCAACATAca
ataa
>contig022368-TilTARe.A008
ATGGAGATACAGAAAGGAGCCGAGCTCTGCTTTCCACAACTCCTCAACAGTTCCTGCAGGAAGCCGACACTTCTCTGGTCCAAAGCTGTGCTCCTGAACATTGTGCT

CTCATGTATCTCTGTGATCACTGCTGCTCTAAACCTTCTCGTCATCGTCTCAGTCTCTTACTTCAGGCAGAGGCAACTTCACACACCCAGTAACATCCTCCTCCTCTCTC
TGGCTGTCTCAGACTTTCTTGTGGGTCTCCTGTTGATGCCTTTAGAAATCTTTAGAAACACAACCTGCTGGGTACTTGGTGATCTAATGTGTTCGGTTTATTGGTATCTG
ACTGGCAACATTACCTGCGCTTCAATAGGGAACATAATTCTAATATCAGTTGACCGTTATGTGGCTATTTGTGACCCTCTGCATTACCCCACTAGGATGACTGTGGCAAG
AGTCAAACTGAGTGTTTGTCTGTGTTGGTTTTTTTCAATTTTCTACATCACTCTCTATACAAAGGATATCCTAACTAAACCAGGCAGGTATAATTCCTGTTACGGAGAGTGT
ATGTTTGTCAGCACTGATATTGCAGGGTTTGTTGACATTGTTTTACGCTTCATTGTTCCAGTTTCTGTCATCATAGTTCTGTATATGAGAGTATTTGTGGTGGCTGTGTCTC
AGGCTCGCGCCATGCGCTCTCATGTCACAGCTGTCACACTTCAGAGGTCACTGACTCAA
>contig022371-TilTARe.A009
ATGGAGATACAGAAAGGAGCCGAGCTCTGCTTTCCACAACTCCTCAACAGTTCCTGCAGGAAGCCGACACTTCTCTGGTCCAAAGCTGTGCTCCTGAACATTGTGCT
CTCATGTATCTCTGTGATCACTGCTGCTCTAAACCTTCTCGTCATCGTCTCAGTCTCTTACTTCAGGCAGAGGCAACTTCACACACCCAGTAACATCCTCCTCCTCTCTC
TGGCTGTCTCAGACTTTCTTGTGGGTCTCCTGTTGATGCCTTTAGAAATCTTTAGAAACACAACCTGCTGGGTACTTGGTGATCTAATGTGTTCGGTTTATTGGTATCTG
ACTGGCAACATTACCTGCGCTTCAATAGGGAACATAATTCTAATATCAGTTGACCGTTATGTGGCTATTTGTGACCCTCTGCATTACCCCACTAGGATGACTGTGGCAAG
AGTCAAACTGAGTGTTTGTCTGTGTTGGTTTTTTTCAATTTTCTACATCACTCTCTATACAAAGGATATCCTAACTAAACCAGGCAGGTATAATTCCTGTTACGGAGAGTGT
ATGTTTGTCAGCACTGATATTGCAGGGTTTGTTGACATTGTTTTACGCTTCATTGTTCCAGTTTCTGTCATCATAGTTCTGTATATGAGAGTATTTGTGGTGGCTGTGTCTC
AGGCTCGCGCCATGCGCTCTCATGTCACAGCTGTCACACTTCAGAGGTCACTGACTCAA
>contig022383-TilTARe.A010
ATGGATAACCAGGGCGGAGCAGAGCTCTGCTTTCCACAACTCTTCAACACCTCCTGCAAAAAGCCTACAACTCCTCTATCACAAGTTTTGTTCCATTATATTGTTGTGTT
CTCAGTGTCACTGCTAACTGCGGCTCTCAACCTCCTCGTCATCATCGCAGTCTCCCATTTCAGGCAGCTCCACACACCCACTAACATCCTCCTCCTCTCTCTGGCTGT
CTCAGACTTTCTCATTGGTCTGTTGATGATGCCGGCAAAAATCCTACGAGACACAGCTTGTTGGTTTCTTGGTCAGCTCACATGTTTAATATATAATTATATATGCTTCATTA
TTACCTCTGCCTCAGTGGGCATTATGGTGCTGATATCAGTCGACCGCTATGTGGCTATTTGTGACCCTCTGCATTACCCCACCAGAATCACTGAAAAAAGAGTGAAACTT
TGCGTCTGTCTGTGTTGGCTCTGCTCTGTTTTTTACAACATACTATTTATAAAGGACGACCTGCTTCAACGAGAGCAACATACTTCCTGTTATGGAGAATGTGTATTTGTC
ATAGACTACATTGTAGGAACCACTGACATTGTTGTGACTTTTATTGCTCCAGTTACTGTCATTGTAATTTTGTATATGAGAGTATTTGTGGTGGCTGTGTCTCAGGCCCGT
GCCATGCGCTCTCATGTTACAGCTGTCACACTGCAGCTCTCAGTGACTCTAACAGCAAAGAAATCAGAGTTAAAAGCTGCCAGGACTCTGGGTGTTCTTGTTCTTGTG
TTTCTATTATGTTTCTGCCCATATTACATTGTTTCACTT
>contig022384-TilTARe.A011
GCTGTCACACTGCAGCTCTCAGTGACTCTAACAGCAAAGAAATCAGAGTTAAAAGCTGCCAGGACTCTGGGTGTTCTTGTTCTTGTGTTTCTATTATGTTTCTGCCCATA
TTACATTGTTTCACTTTTTGGATACAAGTTGCTCAACAGCTCGTCTGCATCCATTGTGATCTATCTGTATTATTTTAACTCCTGTCTAAATCCTTTGATTTATGCTATGTTCTA
CCCCTGGTTTAGAAAAGCTGTGAAGCTAATTGTGACTCTACAAATACTGCAGCCTGGCTCCTGTGAGGTCAACATACTAtag
>contig033907-TilTARe.A012
GTATTTGTGGTGGCTGTGTCTCAGGCCCGTGCCATGCGCTCTCATGTTACAGCTGTCACACTGCAGCTCTCAGTGACTCTAACAGCAAAGAAATCAGAGTTAAAAGCT
GCCAGGACTCTGGGTGTTCTTGTTCTTGTGTTTCTATTATGTTTCTGCCCATATTACATTGTTTCACTTTTAGGAAACGAGTTGTTCAACAGCTCATCTGCATCTATTGTGA
TCTACCTGTATTATTTTAACTCCTGTCTAAATCCTTTGATTTATGCTATGTTCTACCCCTGGTTTAGAAAAGCTGTGAAATTAGTTGTCACTCTACAGATACTGCAGCCTGG
CTCCTGTGAGGTCAGCATACTGTAA
>contig056116-TilTARe.A013
ATTGTTGTCTGTTTGTGTTGGATCTGTTCTGTCATCTTTCAAAGTCTGATTCTGATGGATAACCTGAAGCAACCAGGCAGGTATAACTCTTGCATTGGAGAGTGTGTCTT
TGTCATTAATTACATCGCAGGACTTGTTGATGTTACTTTTTCCTTTATTGTTCCCATTACTGTGATTGTAGTTTTGTATCTGAGAGTGTTTGTGGTGGCTGTGTCTCAGGCT
CGTGCTATGAGGTCTCAGCTTGCAGTCACTCACCAGCGATCAGTTACAGTTACTGCAAAGAAATCGGAGCTGAAAGCAGCCTGGACTCTTGGTATTGTTGTTGTTGTG
TTTCTCATATGTATGTGTCCATATTACTGTGTGGCTCTCACAGGCCAAGACAGCTTGCCAAGTGCTTCATCACTGACATTTGTTCTATGTTTGGTCTACTTTAACTCATGT
CTGAACCCCATTATATATGTCTTTTTCTACCCTTGGTTCAGAAAATCCATAAAAGTCATTGTTACTCTTCAGATACTGCAGCCTGACTCCTGCCAGGCCACTGTGCTTtag
>contig056129-TilTARe.A014
ATTGTTGTCTGTTTGTGTTGGATCTGTTCTGTCATCTTTCAAAGTCTGATTCTGATGGATAACCTGAAGCAACCAGGCAGGTATAACTCTTGCATTGGAGAGTGTGTCTT
TGTCATTAATTACATCGCAGGACTTGTTGATGTTACTTTTTCCTTTATTGTTCCCATTACTGTGATTGTAGTTTTGTATCTGAGAGTGTTTGTGGTGGCTGTGTCTCAGGCT
CGTGCTATGAGGTCTCAGCTTGCAGTCACTCACCAGCGATCAGTTACAGTTACTGCAAAGAAATCGGAGCTGAAAGCAGCCTGGACTCTTGGTATTGTTGTTGTTGTG
TTTCTCATATGTATGTGTCCATATTACTGTGTGGCTCTCACAGGCCAAGACAGCTTGCCAAGTGCTTCATCACTGACATTTGTTCTATGTTTAGCCTACTTTAACTCATGTC
TGAACCCCATTATATATGTCTTTTTCTACCCCTGGTTCAGAAAATCTATAAAAGTCATTGTTACTCTTCAGATACTGCAGCCTGACTCTTCTAAAGCCACCATGCGCtag

>contig072760-TilTARe.A016
GTGCTCATATCTGTAGATCGCTATATAGCCATCTGTGACCCTCTGCTCTATTCCTCAAAGATCACAGTGAACAGAGTGAAGCTTTCAGTCTGTGTCTGCTGGGTCTGCTC
TCTTCTCTACAATGGCTGCATTCTCATGGAGCACTTAGGGTGGCCGGAGAGGTTCAGCTCCTGTCATGGGGAGTGTGTTGTGTTCATCAGCTGCACTTCAGGAACAGT
AGATTTCTTTTTGTCATTTGTCGGCCCCTGTGGCTTAATGTTTGTTCTGTACATGAGGGTGTTTGTGGTTGCTGTTTCTCAGGTGCGTGTCATTCGGTCGCAGGTGGCT
GTGAGAGCAGCTCCAGCTGCTAAAAAATCAGAGTTGAAGGCAGCCAGGACACTCGGGATTGTGATAGTTGTTTTTCTAATGTGCTTCTGCCCTTATTATTATCCTTCCTT
TGCAGGTGTGGACACTTCTATGAGCTTGCCTTATTATGCTTTGTTGTTTTGGATGGTGCTGACAAACTCTTGTGTGAACCCTGTGATTTATGTTCTGTTCTACCTCTGGTT
TAGAAGAGCTATCAGATTCATCGTCACCCTCAGAATACTGCAGCCTCACTCCAGAGAGGTCAACATCCTGTAG
>contig056128-TilTARe.A015
ATGGAGGAAACTGAACTCTGCTTTCAACAACTCTTTAACACCTCCTGCATGAGGCCCAGGCGTCCATACTTTGAGATCATGCTGACTTATATTCTGCTGTCCTTCATTTC
TCTGCTTACTGCAATTCTTAACCTGTTGGTCATCATCTCTGTCTCACATTTCAGGCAGCTCCACACCCCCACCAACCTCCTGCTCCTTTCTCTGGCTGTTGCTGATTTCT
GTGTGGGCCTCCTCTTGTTCTTCCAAATTGTGCTCATAGATGGATGCTGGTTCCTCGGTGACATCATGTGCACTCTGTATCAATACCTAGCATATGTCATCACCTCGGCC
TCAATAGGAACCATGGTGATCATA
>contig056115-TilTARes.A017
ATGGAGGAAACTGAACTCTGCTTTCAACAACTCTTTAACACCTCCTGCATGAGGGCCAGGCGTCCACACTTTGAGATCATGCTAACTTATATTCTGCTGTCTTTCATTTC
TCTGCTTACTGTGATTCTTAACCTGTTGGTCATCATCTCCGTCTCACATTTCAGGCTCTCCCTATTCAGGCAGCTCCACACCCCCACCAACCTCCTGCTCCTTTCTCTGG
CTGTCGCTGATTTCTGTGTGGGCCTCCTCTTGTTCTTCCAAATTGTGCTCATAGATGGATGCTGGTTCCTCGGTGACATCATGTGCACTCTGTATCAATACCTAGCATAT
GTCATCACCTCGGCCTCAATAGGAACCATGGTGATCATA
>contig045078-TilTARf.A018
ATGGAGAACCTGTATGGACAACAATTCTGCTTTCCACAGCTCCTCAACACCTCCTGTAGGAAGCTGCTGCAACATCACAATCAGACCATTTTTCTGTACATTCTGCTTTC
CTGCATCTCTCTGCTCACAGTGACTCTCAACGTCCTGGTCATCGTCTCTATCTCCCACTTCAGGAGGCAGCTTCACAACCAAACCAACCTCTTCCTCCTCTCTCTCGCT
GTCTCAGACTTCCTTGTTGGCCTCCTGCTGATGCCGCCTCGAATCCTCCTTTTAGGGGGCTGCTGGTTTTTGGGCACCTTCACGTGTGGACTGTTTTACTATGCCTCTT
TTGTCCTCACATCTGCCTCAGTAGGAAACATGGTGCTTATTTCATTTGACCGGTATGTAGCTATTTGTGACCCCTTGTCCTACCCCACCAAAGTTACTGAAAGAAAAGTC
CAAATCAGTATCTGCCTGTGTTGGGCCTGTTCACTGCTCTATAACGGCACAATACTGAACAACTTTCTGAAGCAGCCAGACAGATATAATTCTTGTGACGGGGAATGCAT
AGTTGTAATTAATTTCATAACTGGTGCTTTTGATGTCGTAGTTACCTTCATCGGCCCCACTGCTGTGATTATATTTCTGTACATGAGAGTATTTCTGGTGGCTGTGTCTCAG
GCTCAGGCTATGAGATCTCATGTTGCCTTTGTTACTCCAAAGGGTTCAGTTCATGTTGCCATTAAGAAGTCTGAGAGAAAAGCAGCTACAACTATTGGTGTTGTTGTGG
CTGTGTTTCTGATGTGTTTCTGTCCTTATTTTTATCCCTCTCTCGCAGGGCAGGACACATCAACCAGTGTGGAATTTTCAGTTTTTGGGGTCTGGTTACTTTATTGTAATT
CCTGTCTGAATCCACTTATTTATGCTTTTTTCTATCCCTGGTTTAGGAAAACTGTTAAACTGATAGTTACATTGCAGATTTTGCAGCCTGACTCTTGTGATGCCAACATACT
GTAG
>contig007531-TilTARp.A020
ATGGCAGAAACTGAACTCTGCTTTCCAAATCTCAACTCCTCCTGCAGTAGATTAAAGCGCTCTCCCTCAGACTCAGTGCTTATTTATGTAATTCTAACCGCCATTTCTCTT
CTTACTGTGGCTCTAAACCTGCTGGTCATCATCTCCATCTCCCACTTCAGGCAGCTCCACACCCCTACAAACCACCTCCTCCTCTCCCTGGCAGTCTCTGATTTCTTTG
TGGGTCTCAATATGTGCTTTCAAAGTATGCTCATAGATGGTTGTTGGTATCTTGGTGACCTGATGTGTGTGTTGTATTATGTTTGCGACATAGTTGTTACTTCTGCCTCAG
TAGGAACCATGGTGCTCATTTCAGTTGACCGTTATGTGGCCATTTGTGATCCTCTTCATTATCCCACCAAAGTCACTCCAAAAAGAGTTCAGACCTGTGTCTTAATGTGT
TGGATTTGCTCGCTCCTACTTGTCGGTGTGCTATTGAAGGATAACCTGGATAAACCAGGCAGGTTTAATTCCTGCTTTGGAGAGTGTGTGATTTATGTTGACTTTGCAAT
ACAGGTTGCCGATCTTATTTTGACATTCCTCCTTCCCATTACTGTCATTGTAATTTTGTATCTCAGAGTATTTGCCGTGGCCGTGTTTCAGATTCGGACCATGCAACCTCA
TGTTGCAGCTGTCACACAAAGGGGGAAAGTAAGTCCAAAAAAATCTGAACTGAAAGCAGCTAGGACTCTTGGCGTTGTTATTCTTGCATTTCTGATATGCCTATTCCCAT
ATTACTGTGTAATACTTTCAGGCCAGGACACACTGCTTGATATTTTATCAGTTACCTTTTTTTTATGTTTGTTTTATTTTAATTCCTGTCTCAACCCTATCATCTGTGCCTTTT
TCTACCCATGGTTTAGAAAATCTGTTAAACTTATTGTTACATTTCAAATAGTCAAGTCTGGCTCCAGTGATGCCAGCATGCTGTGA
>contig037870-TilTARp.B065
ATGGAGTCATTTAATCTGACTGTTAATACTATGAGCTTCCTTCTCTGCGATTCACAACAGAACAAATTATGTGTGTTATTATATGTTGTCCTCATCTCATTAATGCTTATTACA
ATATGTGGAAATCTTCTTGTGATAATTTCTATCATTTACTTTAGATATCTCCACACTCCTACTAACTACCTTATCCTCTCTCTGGCTGTGGCTGATCTACTAATTGGTGCTTTA
ATATTTCCTTTGAGCATGACATTCTCTCTAAAGCCATGTATGTATACATACAGTTTACTGTGCAACTTAAGAAGCACATTGGATATGACAATAAGTATATCTTCCTTATTGAAC
CTGTGCTGTATTTCTGTTGATCGACATTATGCTGTTTGCCACCCTCTGATATATAAAACTAAAATAACTGATTGTGTTGCCGTGAAGATGGGCCTTGGAAGCTGGGCTGC
TGCAATCTTAATTGGAACTTTTGTCTTCCTGATGTTTTTTTATCCCAGAAGGCTGGACACAAGCTGTGGGATTGCTCTCCTTGCCATAGCTATTGTTTCATATTATATCCCA
ACAATAGTTTTACTGTCCATGTACAGTAAAATTCTTGTTGTTGCACTGAGGCAGGCACGCAGCATTGGTAATAAAACTTGTCAGAACACAAAGTCTAAAGCAGTTGCCAG
CAAGGAGAGAAAGGCCACCAAGACAATGACTGTAGTTATTGGAATATTTTTAATTTTTTGGGTTCCTGTTTTTCTTAGTTATTCATTTTATCCTCTGAATAGTTTTATACTTTA
TGTGCTTCTTGAACCATTTAACTGGTTTTCACTTTCTAACTCAATGCTCAATCCCTTTATTTATGCTTTCTTTTACACTTGGTTTAGAAGAGCTTTTAAAATGATAATTTCTG
GAAAAATATTTCAAGGTGATGCTACTAACAGAAAACTACACTGA
>contig037874-TilTARp.B066
ATGGAGTCATTTAATCTGACTGTTAATACTATGAGCTTCCTTCTCTGCGATTCACAACAGAACAAATTATGTGTGTTATTATATGTTGTCCTCATCTCATTAATGCTTATTACA
ATATGTGGAAATCTTCTTGTGATAATTTCTATCATTTACTTTAGATATCTCCACACTCCTACTAACTACCTTATCCTCTCTCTGGCTGTGGCTGATCTACTAATTGGTGCTTTA
ATATTTCCTTTGAGCATGACATTCTCTCTAAAGCCATGTATGTATATAAAGAATTTCTTTTGCAACTTAAGAAACACTTTTGATATAACAATAAGTGTATCTTCCTTATTAAATT
TGTGCTGTATTTCTGTTGATCGATATTATGCTGTTTGCCACCCTCTGATATATAAAACTAAAATAACTGATTGTGTTGCTGTGAAGATGGGCCTTGGAAGCTGGGCTGTTG
CTATCTTAAGTGGAACTTTTGTCTTCCTGATGTTTTTTATTCCAGACGAATGTGACACAAGTTGCACTTTCACTCTCGTTGTCACATCAATGGTAGGATATTATATCCCAAC
AATAGTTTTACTGTCCATGTACAGCAAAATTCTTGTTGTTGCACTGAGGCAGGCACGCAGCATCCATAATAAAACTTCTCAGAACACAAAGTCTAAAGCAGTTTTCAGTAA

GGAGAGAAAGGCCACAAAGACAATGACCATAGTTATTGGAATATTTTTAATTTTTTGGGTTCCTGTTTTTCTTAGTTATTCATTTTATCCTCTGGATAGTTTTATACTTTATGT
GCTTCTTGAACCATTTAACTGGTTTTCACTTTCTAACTCAATGCTCAATCCCTTTATTTATGCTTTCTTTTACACTTGGTTTAGAAGAGCTTTTAAAATGATCATTTCTGGAA
AAATATTTCAAGGTGATGTTACTAACATAAAACTACACTGA
>contig007518-TilTARs.A019
ATGAAAACCATTGACAGAGCTGAACTGTGCTTTTCACAACTCAACTCCTCCTGCAGAAAGACCATGCATCCTCGTTCGCTTTCTGTTCTCTTTTATGTCATACTGTCCTC
CATTTCTGTGCTCACTGTGGCTCTTAATCTACTGGTGATCATCTCTATTTCCCACTTCAGGCAGCTCCACACCCCGACCAACCTCCTCCTCTTCTCTCTGGCTGTCTCTG
ATTGTTTTGTGGGCCTTCTCACAGTCTTTCAAATACTGGTCATAGACGGCTGCTGGTATCTTGGTGAACAGTTGTGTATTTTGTATTATATTTTCGATTATGTTGTTACTAAT
GCCTCAATAGGAACCATGGTGCTCATATCAGTTGACCGCTATGTGGCTATTTGCGACCCCCTTTATTACCCAAGCAAAGTCACTCTAAAAAGAGTAAGGACTTCTGTTTC
ATTATGTTGGATTTTTTCTCTCTTCTGTGTCTTTGTGCTGTTGAAAGATAATCTGGGACAACCAGGTAGTTATAATTCCTGCTCTGGTGAGTGTGTGGTTCTTGTTTATTAT
GTTACTGGAATTGTAGATATTTTATTGTCATTTATTGGTCCTGTCATTGTTATCATAGCTCTGTATCTGAAAGTATTTATGGTGGTTGTTACTCAGGCTCGTTCCATGAGGTC
TCGTATTGAAGCTGTCGCTGTGCAGGGTTCTGTAACAGTAACTATTAAGAAATCTGAAATCAAAGCTGCCAGAGTTCTCGGAATTGTTGTAGTTGTATTTCTAGCATGTCT
TTTACCTTATTTTTGTATTGGACTTACAGGTGACAAGACCCTACTCAATGCTTCAACTGCGACTGTTTTTATAACTCTCTTCTATTTTAATTCCTGTGTAAACCCTCTGGTTT
ATGCCTTTTTCTATCCCTGGTTCAGGAGAACTATTAAACTAATAGTAACACTTAAGATACTGAATTCTCGCTCCTGTAAGGCCAATTTACTAATTTCATGA
>contig022357-TilTARp.A021
ATGGAGACCCAGGAGGAAGCAGAGCTCTGCTTTCCACAATTCTTCAACACCTCGTGCAAGAAGACTATAACATCCCTGTCTCAAGCTTTGTTCTCTTACATTGTGATGT
TGTTGGTCACTCTGCTCACTGTGGGTCTCAACCTGCTCGTCATCATCTCAGTCTCCCACTTCAGACAGCTCCACACACCCACAAACATCCTCCTCCTCTCTCTAGCTGT
GTCAGACTTTCTCATTGGTGCGGTGTTGATACCTGCAAAAATCATACAAGATACAGCTTGTGTGTTTCTTGATCAGTTCACATGTTTACTCTATACTTATTTGTCCTTCATT
GTTACCTCTGCCTCAGTGGGCATTATGGTGCTGATATCAGTCGACCGCTATGTGGCTATTTGTGACCCTCTGCATTACCCGACCAGAATCACTCAGAGAAGAATGAAAC
TCTGTGTCTGTCTGTGTTGGTTCTCTTCTGTTATCTATGACATTCTGCTCATAAAGGATGAGCTGTTTCAACCAGGCAGATACAATTCCTGCGGAGAATGTATTTTTGTCA
TTGATTACATTGCAGGACCCACTGACATTATTATTATTTTCAGCAGTAATATTGCTGTTGTTGTTGACCTTGTTTTATTTTTTATTCTTCCAGTTACTGTCATCATAGTTCTGT
ATATGAGAGTATTTGTGGTGGCTGTGTCTCAGGCCCGTGCCATGCGCTCTCATGTTACAGCTGTCACACTTCAGAGTTCACTGAATCAGACAAACAAATCTGAACTGAA
AGCAGCCAGGACTCTTGGAGTTCTTGTCATTGTATTTTTCTCATGCTACTGTCCATTTTATGGCTACACTCTTTTTAAAGAAAACCATGTGAATGATCCATGTGTATCTATT
GTGGTCATTATATTTTACTTTAACTCTAGTCTAAACCCTTTGATATATGCTTTGTTTTAGCCTTGGCTTAGAAACACTGTCAAACTTCTCATCACATTGCAGATCTTCAAGTA
TGACACAGGTGAGGTCAACATATTA
>contig007525-TilTARp.A022
ATGGAAGAAACTGAACTCTGCTTTCCACAACTCCTCAACTCATCCTGTGTGCGGCAAAAGCGCCCACAAATTGAGGCTGTGTGTATTTACACCCTGCTATCATCTATCTC
TCTGCTTACTGCAGTACTGAACCTACTTGTCATCATCTCCATCGCCCACTTCAAGCAGCTGCACACCCCAACCAACCTCCTCCTCCTCTCTCTGGCCGTCTCAGATTTC
TTTGTGGGCCTCATCATGGCTTGTCAGATTAGTCTCCTAGATGGCTGCTGGTTTCTTGGTGACCACATGTGTGCTCTGTATAGCAGTTTAGATTACATTGCTACTTCTGC
TTCAGTAGGAACTATGGTACTCATATCAGCTGACCGCTATGTAGCCATTTGTGACCCTCTGCATTATCCCACCAAAATTACTATAAAAAGAGTCTCAGTCTGTATTTGTACT
TGCTGGGCTTGTTCAATTCTGTATAACAGTCTTATCATGAAGGATAATTTCAAGCAGCCAGGGAGGTATAATTCTTGTTCTGGTGATTGTGTATACATTGACTACTTTGTGG
GAATTGTTGACTTTGTTTTGACCTTTGTCGGCCCTGTGACTGTCATCATAGTTCTGTATCTAAGAATATTTGTGGTGGCTGTGTCTCAGGCTTGGGCAATGCACTCTCATA
TCACTGCTCTCAGACTCCACGGGTCAGAGACGGTGCATGCTAAGAAATCTGAGCTGAAAGCTGCCAGGACTCTCGGTGTACTTCTTATTGCCTTTTTAACATGTCTTTT
CCCATTTTACTGTTCCTCAATGGTAGGCCAGAATAGTTTCTTTAATATTAGATCTGTACCTTTTGAGCGCATGCTGTTCTATTTTAACTCTTGTCTGAACCCGTTAATCTACA
CTTTTTGCTACCCCTGGTTTCTGAAATCTATTAAGCTCATTGTGACATTTAAGATATTTAGGCATTGCTCCAGTAAGGCCAGTATACTATAG
>contig022380-TilTARp.A023
ATGGACACACAGGACGTAGCAGAGCTCTGCTTTCCACAACTCTTTAACACCTCCTGCAAGAAGCCTGCGATTTACATTGTGCTGTCGTCAATCTCTTTCTTAACTGTGG
CTCTAAACCTACTAGTCATCATTTCAGTCTCCCACTACAGGCAGATTTTTCTGTCTTCTTTCAGGCAGCTCCACACCTCCACTAATATCCTCCTCTTCTCTCTGGCTGTCT
CAGACTTTCTTGTTGGTGTCTTGGTGATACCAGGAGAAATCCTCAGAAACACAGCCTGCTGGACTGTTGGTCAGCTCACATGTTTACTGTATATTTTTGTGTCCTTGATT
CTTACCTCTGCCTCAGTGGGAATTATGGTGCTGATATCAGTTGAGCGCTATGTAGCTATTTGTGACCTTCTGCATTATCCCACTAGAATCACTCACAGAAGAGTGAAACT
CTGTGTTTGTACACTTCAGCAATCAGTGACTCTAACAGCAAAGAAATCAGAGATGAAAGCAGCCAGAACTCTGGGTGTTCTCATAGTTGTGTTTCTATTATGTTTCTGTC
CATATTATTGTGTCTCTCTCATAGGGGACGACTTTCTCAGTGGCTCATCAGCAGCTATTGTGATATATGTGTTCTACTTTAACTCATGCATAAACTCTGTAATCTATGCTATG
TTCTACTCCTGGTTTAGAAAAGCAGTGAAACACATTGTGACTCTACAGATACTGCGGTCTGGCTCCAGTGAGGTCAACATACTGTAG
>contig007512-TilTARs.A024
ATGATGGAGGAAACTGAACTCTGCTTTCCAAAACTTCTTAATATCTCCTGCAGGAGGCCAAAGCGTCCTCACTTTGAGATCATGCTGACTTACATTCTGCTCTCCTTCAT
TTCTTTGCTTACTGTGATTCTTAACCTGCTGGTCATTATCTCCATCTCACACTTCAGGCAGCTCCACACCCCCACCAACTTCCTCCTTCTCTCTCTGGCTGTCGCTGATT
TCTTTGTAGGTCTCCTTATGTTCTTTCAAATAGTGCTCATCGATGGATGCTGGTTCCTCGGTGACATCATGTGCACTCTGTATCAGTATCTAGCATTCATTATTACTTCAGC
CTCAATAGGAACCATGGTGATCATATCTGCTGATAGGTATTTGGCTATTTGTTACCCTCTGCATTACTCCACCAAAATCACACAACAAAGAGTTAAAATCTGTATATGTTTAT
GTTGGGTTTTTTCTGTGATCTTTCAGAGTTTGATTGTGAAGGATAACTTGAAACAACCAGGAAAGTATAACTCCTGCATTGGAGAGTGTGTCTTTGTTGTTAACTACATTG
CTGGGCTTTTTGATCTTTTGTTTTCCTTCATTGTTCCCATTACTGTGATTATAGTTCTGTATCTGAGAGTGTTTGCGGTGGCTGTGTCTCAGGCTCATGCCATGAGGTGTC
AACATGCAGTCACTCACCAGCGAGCAGTTACAGTAACTGTTACGAAATCGGAGCTGAAAGCAGCCCGTACTCTTGGTGTTGTTGTAGTTGTGTTTCTTATATGTATGTGC
CCATATTACTGCGTTGCTCTCACAGGCCAAGATAACTTCCTAAATGCTTCATCTGCTGCCTTTGTAATATGTTTGGTGTATTTTAACTCTTGCGTAAACCCTATCATTTATGT
CTTTTTTTATCCCTGGTTCAGAAAGTCAATCAAACTTATTGCTACTCTTCAAATACTGCAGCCTGACTCCCATGAGACTAACATGcattaa
>contig007520-TilTARs.A025
ATGGAAATGTTTGAAGAAACTGAACTCTGCTTTCCACAGCTTCTTAACTTCTCCTGCAGGAAGCCCATGCAACCTCACTCAGTATCTATACTCATTTATATTGCATTGTCC
TCCATCTCTTTACTGACTGTGACTCTTAATCTGCTGGTCATCATCTCCATCTCACATTTCAAGAAGCTGCACACCCCCACCAATCTCCTCCTCCTCTCTCTGGCTGTCTC
TGATTGCCTGGTGGGTCTCCTGATGTTATTTCAAATTATGATTATAGATGGTTGCTGGTTCCTTGGTGAATTCATGTGTAGTATGTATTTTCTCTTGGACTACATTATTACCT
CTGCCTCAGTAGGAACCATGGTTCTCATATCAATTGATCGTTATGTGGCCATTTGTTACCCTCTCCATTACTCTACCAAAGTCACCCCAAAAAGAACAAAAGCATGTGTTT
ATCTGTGTTGGATTTGTTCTTCAGTCTTCCAGTGTCTTGTGCTAAAAGACAACCTGGTGCAACCTGGCAGGTATAATTCCTGCTATGGGGAGTGTGTGGTTGTTGTTGG
CCATGCTTTTGGAGTTGCAGATCTATTGTTGTCCTTCATTGGTCCTGTCATTGTGATTGTAGTTCTTTATCTAAACGTATTTGTTGTGGCTATGACTCAAGCTCGTGCCCT
GCGGTCACATATTGCAGCTCTGACACACGAGGGGTCAGTAAGTACAAATGTTAAGAAATCTGAAATGAAAGCAGTGAGGACCATCAGTGTTGTTATCATTGTGTTTCTAA
TATGTCTCTGCCCATATTTTTGTGTCACACTCTCAGGTCAAGATGCCATGCTCAGTGCTTCATCTGTTGCATTTGTAATGTGTTTGTTCTATTTAAACTCCTGTCTAAACCC
TCTAATTTATGCTTTATTTTACCCCTGGTTCAGAAAATCTGTTAAACAAATTGTTACACTTAAAATATTGAAGTCTGGCTCCTGTGACACCAATATCATGtaa
>contig007524-TilTARs.A026
ATGAAAACCTTTGAAGAAACTGAACTCTGCTTTCCACAACTCCTCAACTCCTCCTGCAGGAAGACCATGCGTCCTTATACATTATCTATACTTATTTATATTACCCTGTCCT
CCATTTCGTTGATCACTGTGACTCTTAATCTGCTGGTCATCATCTCCATCTCACATTTCGAGAAGCTGCACACCCCCACCAATCTCCTCCTCCTCTCTCTGGCTGTCTCT
GATTGCCTGGTGGGTCTCCTGATGTTATTTCAAATTATGATTATAGATGGTTGCTGGTTTCTTGGTGAATTCATGTGTAGTATGTATTTTCTCTTGGACTACATTATTACCTC
TGCATCAATAGGAACCATGGTTCTCATATCAATTGACCGTTATGTGGCTATTTGTTACCCTCTCCATTACTCTACCAAAGTCACCCCAAAAAGAACAAAAGCATGTGTTTAT
CTGTGTTGGATTTGTTCTTCAGTCTTCCAGTGTCTTGTGCTAAAAGACAACCTGGTGCAACCTGGCAGGTATAATTCCTGCTATGGGGAGTGTGTGGTTGTTGTTGGCC
ATGCTTTTGGAGTTGCAGATCTATTGTTGTCCTTCATTGGTCCTGTCATTGTGATTGTAGTTCTTTATCTAAACGTATTTGTTGTGGCTATGACTCAAGCTCGTGCCCTGC
GGTCACATATTGCAGCTCTGACACACGAGGGGTCAGTAAGTACAAATGTTAAGAAATCTGAAATGAAAGCAGTGAGGACCATCAGTGTTGTTATCATTGTGTTTCTAATA
TGTCTCTGCCCATATTTTTGTGTCACACTCTCAGGTCAAGATGCCATGCTCAGTGCTTCATCTGTTGCATTTGTAATGTGTTTGTTCTATTTAAACTCCTGTCTAAACCCT
CTAATTTATGCTTTATTTTACCCCTGGTTCAGAAAATCTGTTAAACAAATTGTTACACTTAAAATATTGAAGTCTGGCTCCTGTGACACCAATATCATGtaa
>contig022324-TilTARs.A027
ATGGAAATCCAGATGCATCTAGAGGCAGAGTTCTGTTTTCCAGAGCTACTGAACAGTTCCTGCAGGAAGCCGACACTTCACTGGTCCAAAACGGTGCTCCTGAACGTT
GGGCTTTCGTTCATCTCTGTGATCACTGCTGCTCTTAATCTGCTCATCATCATCTCAGTCTCCCACTTCAGGCAGCTCCACACTCCCAGTAACATCCTCCTACTCTCTCT
GGCTGTCTCTGACTTTTTTGTGGGTTTTCTGTTGATGCCGGTAGAAATCTTCCGAAGCACGGCCTGCTGGGTATTTGGTGATCTCATGTGCTCACTTTATATTTATCTGA
GCGGCATTCTTATGAACGCTTCTTTTGAAATTATTGTTTTTATATCAGTTGACCGCTATGTGGCTATCTGTGACCCTCTGCATTACCCCACCAGAATCACTGTGGCAAGAG
TCAAACTTAGTGTTTGTCTGTGTTGGTTTTATGCTATTTTCTACATGAGTCTTTACACAAAGGATGTCTTGATAAAGCCAGGCAGGTATGCTTCCTGCTATGGAGAGTGTG
TGTTTGTTATTGAAGATATAACTGGCACTGTTGACCTTGTTTTATGTTTTTTTGTTCCAGTTATTATCATCATTGTTCTGTATACAAGAGTATTTGTGGTGGCTGTGTCTCAG
GCTCGTGCCATGCGCTCTCATGTTACAGCTGTCACACTTGAGCGTTCACTGAATCAGACAAACAAATCTGAGTTGAAAGCAGCCAGGAACCTTGGAGTTCTTGTAATT
GTGTTTCTAGCAAGTATTTGCCCATTTTACTTCTACTCTCTTGTTGATGGCAGTGTGCTCAATGCTTCATCTGCAACTTTTTTGATCATTGTTTATTTTAACTCTTTTTTAAA
CCCATTGATCTATACCCTGTTTTACCCCTGGTTTAGAAATGCTGTTAAACTCATCATCACTCTGCAGATATTCAAGCATAACAGTAGTGAAGCCAACATACTAtag
>contig022330-TilTARs.A028
ATGGAGATACAGAAAGAAGCTGAGCTCTGTTTTCCACAACTCCTCAACATTTCCTGCAGGAAGCCGACACTTCACTGGTCCAAAGCTGTGCTCCTGAACATTGTGCTG
TCCTGCATCTCTCTGCTGACTGCTACTCTAAACCTTCTCGTGATTATCTCAGTCTCCTACTTCAGGAAGCTTCACACACCCAGCAACATCCTCCTACTCTCTCTTGCTGT
CTCAGACTTTCTCATGGGTCTCTTGCTGATACCAGCAGAAATCCTTAGAAGCATGACCTGCTGGGTACTTGGTGATCTCATGTGTTCTGTTTATTTTTTTCTGACTGTAAA
CATTACCTGTGCTTCAATAGGGAACATAGTTCTTATATCGATTGACCGTCATGTGGCTATTTGTGACCCTCTGCATTACTCCACCAGAATTACTGTGCCTAGAGTCAAACT
CAGTGTTTGTCTGTGTTGGTTTTATTCAACTTTCTACAGCAGTCTTAATACACAAGAAATGCTGATTGTACCAGGCAGGTATAATTCCTGTTATGGAGAATGTGTGCTTGT
CATCAGTGATTTTGCTGGAACTCTTGACCTCATTTTATTTTTTATTCTTCCAGTTACTGTCATCATAGTTCTGTATATGAGAGTATTTGTGGTGGCTGTGTCTCAGGCTCGT
GCCATGCGCTCTCATGTTACAGCTGTCACACTTCAGCGTTCACCCAATCAAACAAACAAATCTGAGCTGAAAGCAGCCAGGACTCTTGGGGTTCTGGTAGTTGTGTTT
CTGGCATGCTACTGTCCATTTTATTGCTACTCTCTCGCTGATGAAAATGCTGTCAATGATCCAGCAGCGTCTTTTGTGGTCTTTGTCTTTTACTTTAACTCTTGTATAAACC
CTTTGATGTATGCCCTGTTTTACCCCTGGTTTAGAAATGCTGTTAAACTTATTATCACGTTGCAGATATTCAAGCATAACACCTGTGAGGCCAACATACTAtaa
>contig022334-TilTARs.A029
ATGGAGATACAGAAAGAAGCTGAGCTCTGTTTTCCACAACTCCTCAACATTTCCTGCAGGAAGCCGACACTTCACTGGTCCAAAGCTGTGCTCCTGAACATTGTGCTG
TCCTGCATCTCTCTGCTGACTGCTACTCTAAACCTTCTCGTGATTATCTCAGTCTCCTACTTCAGGAAGCTTCACACACCCAGCAACATCCTCCTACTCTCTCTTGCTGT
CTCAGACTTTCTCATGGGTCTCTTGCTGATACCAGCAGAAATCCTTAGAAGCATGACCTGCTGGGTACTTGGTGATCTCATGTGTTCTGTTTATTTTTTTCTGACTGTAAA
CATTACCTGTGCTTCAATAGGGAACATAGTTCTTATATCGATTGACCGTCATGTGGCTATTTGTGACCCTCTGCATTACCCCACCAGAATGACTGTGCCAAAAATCAAACT
CAGTGTTTGTCTGTGTTGGTTTTATTCAACTTTCTACAGCAGTCTTAACACAAAAAAAATCCTGATTGAACCAGGCAGGTATAATTCCTGCTATGGAGAATGTGTGCTTGT
CAGCAGTAATATTGCTGGAACTCTTGACCTCATTTTATTTTTTATTCTTCCAGTTACTGTCATCATAGTTCTGTATATGAGAGTATTTGTGGTGGCTGTGTCTCAGGCTCGT
GCCATGCGCTCTCATGTTACAGCTGTCACACTTCAGCGTTCACCCAATCAAACAAACAAATCTGAGCTGAAAGCAGCCAGGACTCTTGGGGTTCTGGTAGTTGTGTTT

CTGGCATGCTACTGTCCATTTTATTGCTACTCTCTCGCTGATGAAAATGCTGTCAATGATCCAGCAGCGTCTTTTGTGGTCTTTGTCTTTTACTTTAACTCTTGTATAAACC
CTTTGATGTATGCCCTGTTTTACCCCTGGTTTAGAAATGCTGTTAAACTTATTATCACGTTGCAGATATTCAAGCATAACACCTGTGAGGCCAACATACTAtaa
>contig022334-TilTARs.A030
ATGGAGATACAAAATGAAGCTGAGCTTTGCTTTCCAGAACTCCTCAACAGTTCCTGCAGGAGGACGACACTTGTCCACTGGTCCAAAGCTGTACTGCTGTACATTGTG
CTGTTCTGCATTTCTCTGATCACTGCTGCTCTAAACCTTCTGGTCATCATCTCAGTCTCCCACTTCAGGCAGCTTCACACACCCACTAACATCCTCCTCCTCTCTCTGGC
TGTTTCAGACTTTCTTGTGGGCCTCCTATTGATGCCATTAGAAATTGTTAGAGGCATGACCTGCTGGGTACTTGGTGATCTTATGTGTTCTGTTTATATTTTCCTTACTGG
CAACGTTACCTGTGCTTCAATAGGGAACATAGTTCTAATATCAGTTGACCGTTATGTGGCTATTTGTTACCCTCTGCATTATCCCACCAGAATTACTGTGCCTAGAGTCAA
ACTAAGTGTTTGTGTTGGTTTTATGCCATTTTATAGTTCTTGCTACGGAGAGTGTGTCCTGGTTGTCAGTGTTGTTGTAGGGATTGTTGACCTTGTTTTATCCTATTTTGTT
CCAGTTACTGTCATCATAGTTCTGTATATGAGAGTGTTTGTGGTGGCTGTGTCTCAGGCTCGCGCCATGCGCTCTCATGTTACAACTGTCGCACTTCAGCGTTCATTGA
ATCAAACTAACAAATCTGAGTTGAAAGCAGCCAGGACTCTGGGGATTCTTGTAGTTGTGTTTCTACTGTGCTCTTGCCCATACTACTGCATCTCATTTGCAAGGGATAAC
TATAGCTCAGTACCATCTATTGTGTTTGGTCTGTTTTTCTTAAACTCCTCTCTAAATCCTCTGATCTATGCAATGTTCTACCCTTGGTTTAGAAAAACTGTTACACTAATTGT
CACTCTACAGATCCTGCAGACTATTTCTTGTGAGGTCAATATACTGtag
>contig022337-TilTARs.A031
ATGATGGAGATAGAGAAAGGAGCCGAGCTCTGTTTTCCGCAACTCCTCAACAGTTCCTGCAGGAAGCCGACACTTCACTGGTCCAGAGCTTTGCTCCTGAACATTGTG
CTGTCCTGCATCTCTCTGATCACTGCAGCTCTAAACCTTCTTGTCATCATCTCAGTCTCTTACTTCAGGCAGCTCCACACGCCTAGCAACATTGTCCTACTCTCTCTGGC
TGTTTCAGACTTTCTAATTGGTCTCCTCAAGATGCCATTTGAAATCATTAGAAACACAGGCTGCTGGGTACTTGGTGATCTTATGTGTTCTGTTTATATTTTTCTGACCATC
AGTCTTCTCTGTGCTTCAATACTTAACATAGTGCTAATATCAATTGACCGTTATGTGGCTATTTGTGACCCTCTGCACTATCCCACCAGACTGACGGTGGCAAGAGTCAA
ACTCAGTGTTTGTCTGTGTTGGTTTTATTCTGCTTTCTACAGCAGTCTTTATGCAAAGAATGTCCTGATTGAACCAGGCAGGTATAATTTCTGCTTTGGAGAGTGTGTGTT
TTTCAGCAGTAATATTGCTGTTGTTGTTGACCTTGTTTTATTCTTTATTGTTCCAGTTTCTGTCATCATAGTTCTGTATATGAGAGTATTTGTGGTGGCTGTGTCTCAGGCT
CGTGCCATGCGCTCTCATGTTACATCTGTCACACTTCAGCGTTCAGCAAATCAAGCAAACAAATCTGAGCTGAAAGCAGCCAGGACTCTTGGGGTTCTTGTAGTTGTG
TTTCTGGCAACCTTTTGTCCATTCTACTGCTACTCTCTTGTTGAGGAAAATGCTCTCAATGACCCATCGACATCTACTTTGATCACAGTTTTTTACTTATCTAACTCTTGTC
TAAACCCTTTGATCTATGCCTTGTTTCACCCGTGGTTTAGAAATGCTGTTAAACTCATCATCACTTTGCAGATCTTCAAGTGTGACACAAGTGAGGCCAACATACTGtaa
>contig022341-TilTARs.A032
ATGGAGATACAGAAAGGAGTCGAGCTCTGTTTTCCACAACTCCTCAACACTTCCTGTAGGAAGCCGGCACTTCACTGGTCCAAAGCTGTGCTCCTGAACATTGTGCTG
TCATGTGTCTCTCTGATCACTGCTGCTCTAAACCTTCTGGTCATCATCTCAGTCTCTTACTTCAGGAAGCTTCACACACCGACCAACATCCTCCTCCTTTCTCTGGCTGT
CTCAGACTTTCTTGTGGGTCTCTTGCTGATGCCATTTGAAATCTTTAGGAACATGGCCTGCTGGGTACTTGGTGATCTTATGTGTTCTGTTTCTTGGTATCTGACTGGTA
ACATTGCCTTTGCCTCAACAGGGAACATAGTTCTCATATCAATTGACCGTTATGTGGCTATTTGTCACCCTCTGCATTACCCCACCAGAATTACTGTGGCAAGAGTCAAA
CTTAGTGTTTCTCTGTTTTGGTTATTTGCCATTTTCTACAGCAGTCTCTATATGAAGGATGTACTGATAAATCCAGGCAGGTATAATTCCTGCTATGGAGAGTGTGTGTTTA
TCATTAATGATATTGCAGGGATTTTTGACATGGTTTTATCTTTTTTTGTTCCAGTTCCAGTTATCGTAATTCTGTATATGAGAGTGTTTGTAGTGGCTGTGTCTCAGGCTCG
TGCCATGCGCTCTCATGTTACAGCTGTCACACTGCAGCGTTCATTGAATCAAACAAACAAATCTGAGTTGAAAGCAGCCAGGACTCTTGGAATTCTTGTAGTCGTGTTT
CTAATATGCTACTGTCCATTCTTCTGCTTTTCTTTTGGTGTAGAAGACATGGTTAATAATCCATCTACATTTTTTGTGATCATGGTCTTTTACTTTAATTCTTGTCTAAACCCT
TTGATCTATGCCCTGTTTTACCCCTGGTTTAGAAATGCTGTAAAACTTATCATCACACTGCAGATTTTCAAACATGAAACTAGTGAAGCCAACATACTGtaa
>contig022343-TilTARs.A033
ATGGAGATAGAGAAAGGAGCTGAGCTCTGTTTTCCGCAACTCCTCAACAGTTCCTGCAGGAAGCCGACACTTCACTGGTCCAGAGCTTTGCTTCTGTACATTGTGCTG
TCCTGCATCTCTCTGATCACTGCAGCTCTAAACCTTCTTGTCATCATCTCAGTCTCTTACTTCAGGCAGCTCCACACACCAAGTAACATCGTCCTACTCTCTCTGGCTGT
TTCAGACTTTCTAATTGGTCTACTTATGATGCCATTTGAAATCATTAGAAACACAGGCTGCTGGGTACTTGGTGATCTTATGTGTTCTGTTTATATTTTTCTGACCATCAGT
CTTGTCTGTGCTTCAATACTTAACATAGTGCTAATATCAATTGACCGCTATGTGGCTATTTGTGACCCTCTGCACTATCCCACCAGACTGACGGTGGCAAGAGTCAAACT
CAGTGTTTGTCTGTGTTGGTTTTATTCTACTTTCTACAGCAGTCTTTACGCGAAGAATGTCCTGATTGAACCAGGCAGGTATAATTTCTGCTTTGGAGAGTGTGTGTTTTT
CAGCAGTAATATTGCTGTTGTTGTTGACCTTGTTTTATTCTTTATTGTTCCAGTTTCTGTCATTGTAGTTCTGTATATGAGAGTATTTGTGGTGGCTGTGTCTCAGGCTCAT
GCCATGCGCTCTCATGTTACATCTGTCACACTTCAGCGTTCAGCAAATCAAGCAAACAAATCTGAGTTGAAAGCAGCCAGGACTCTTGGGGTTCTTGTAGTTGTGTTTC
TGGCAACCTTTTGTCCATATTACTGCTACTCTCTTGTTGATGAAAATGCTGTCAATGATCCCTCTGCATCTTTTGTGATCATTATCTTTTACATTAACTCTTGTCTAAACCCA
TTGATCTACGCCTTGTTTTACCCCTGGTTTAGAAATGCTGTTAAACTCATCATCACTTTGCAGATCTTCAAGTGTGACACAAGTGAGGCCAACATACTAtaa
>contig022345-TilTARs.A034
ATGGAGATGCAGAAAGGAACTGAGCTCTGTTTTCCACAGCTCCTCAACAATTCCTGCAGGAAGCCGATACGTCACTGGTCGAAAGCTGTTCTTCTGTACATTGTGCTG
TTCTGCATCTCTCTGATCACTGCTGCTCTAAACCTACTGGTCATCATCTCCATCTCTCACTTCAAGCAGCTTCACACACCCAGTAACATCCTCCTCCTCTCTCTGGCTGT
GTCAGACTTTCTTGTGGGTCTTCTGCTGATGCCATTAGAAATCTTTAGAAGCACGGCCTGCTGGGTACTTGGTGATCTTATGTGTTCTGTTTATATTTATATGACTGTAAA
CATTACCTGTGCTTCAATAGGGAACATAGTTCTTATATCAGTTGACCGTTATGTAGCTATTTGTGACCCTCTGCATTACCCAACCAGAATTACTGTAGCGAGAATCAAACTC
AGCATTTGTTTCTGTTGGTTTTATACTATTTTCTACAGCAGTCTTTATACAAAGGATATCCTGATTGAACCGGGCAGGTATAACTCCTGCTGCGGAGAGTGTGTGTTGGTC
ATCAATGATATTGTAGGGATTGTTGACCTTGTCTTAACTTTTATTGTTCCAGTTGCTGTCATTATAGTTCTGTATATGAGAGTGTTTGTGGCAGCTGTGTCTCAGGCTCGTG
CCATGCGCTCTCATGTTACAGCTGTCACATTGCAGCGTCCACTGAATCAAACAAACAAATCTGAGTTGAAAGCAGCCAGGACTCTTGGAGTTCTTGTAGTTGTGTTTCT
GGCATGCTACTTTCCATTCTACTGCTATGCCTCTGTTGTAGAAGACATGGTTAATAATCCATCTGCATTGTTTGTGTTCATGGTCTTTTATTTTAACTCTTGCCTAAACCCTT
TGATCTATGCCCTGTTTTACCCCTGGTTTAGAAATGCTGTAAAACTTATTGTCACATTGCAGTTCTTAAAGCATGGCACCTGTGAAGCCAACATATTAtag
>contig022349-TilTARs.A035
ATGGAGATACAGAAAGGAGCCGAGCTCTGTTTTCCACAGCTCCTCAACAGTTCCTGCAGGAACCCAACACTTCACTGGTCCAGATCTTTGCTCCTGAACATTGTGCTG
TCCTGCTTCTCTCCGATCACTGCTGCTCTAAACCTTCTGGTCATCATCTCAGTCTCCCACTTCAGGCAGCTGCACACACCCACTAACATCCTGCTCCTCTCTCTGGCTG
TCTCAGACTTTTTTGTGGGTCTCCTTTTGATGCCATTAGAAATTTTTAGAAACACAACTTGCTGGATACTTGGTGACATTATGTGTTCTGTTTATTGGTATCTAAACTTCAG
TTTCATCTGTGTTACAATAGGGAACATACTTTTAATATCAGTTGACCGCTATGTAGCAATTTGTTACCCTCTGCATTATCCCAACAGAATTACTGTGGCGAGAGTGAAACG
CAGTGTTTGTCTGTGTTGGTTTTATGCTATTTTCTACTGTAGTTTTTATACAAAGGACATCCTGATTCAACCAGGCAGGTATAATTCCTGCTATGGAGAGTGTGTGTTTTTC
ATTGATGATATGGCAGGGATTATTGACCTTGTTTTATTTTTTTTAATTCCGGTAATGGTCATCATAGTTCTTTATACGAGAGTATTTGTGGTGGCTGTGTCCCAGGCTCGCG
CCATGCGCTCTCATGTTACAGCTGTCACACTTCAGCGTTCAGCAAATCAAGCAAACAAATCTGAGCTGAAAGCAGCCAGGACTCTGGGGGTTCTTGTAGTTGTGTTTC
TTTTATGCTACTGTCCATCTTATTTCTTTACCTTTTTTGCAGAAGGCATGCTCAACGATCCAGCAACTGTTTTCTTTATAACTGTCATTTATTTTAACTCTTGTCTAAACCCTT
TGATCTATGCCCTGTTTTACTCCTGGTTTAGAAATGCTGTTAAACGTATCATCACTCTCCAGATCGTTACACATGACACAAATGAAGCCAACATATTAtga
>contig022353-TilTARs.A036
ATGGAGATACCGAAAGGAGTCGAGCTCTGTTTCCCACAACTCCTCAACAGTTCCTGCAGGAAGCCGACACTTCACTGGTCCAAAGCTGTGCTCCTGAACATTGTGCT
CTCATGTATCTCTCTGCTCACTGCTGCTCTAAACCTTCTTGTCATCATCTCCGTCTCCCACTTCAGGCAGCTGCACACACCCAGTAACATCCTCCTCCTCTCTCTGGCT
GTCTCAGACTTTTTTGTGGGTCTCCTGTTGTTGCCTTTAGAAATCTTTAGAAACACAAGCTGCTGGGTACTTGGTGATCTTATGTGTTCTGCTTATTGGTATTTGACCAGC
AACATTATCTGTGCTTCAATAGGGAACATTGTTCTAATATCAGTTGACCGCTATGTGGCTATTTGTGACCCTCTGCATTACCCCACCAGAATTACTTTGCCGAAAGTCAAA
CTCGGTGTTTGTCTGTGTTGGTTTTATGCTATTTTCTACAGCAGTCTTTATACAAAGGATGTCATGATTGAACCAGGCAGGTATAATTCTTGCTTTGGAGAGTGTGTATTTT
TCAGCAGCAATATTGCTATTGTTGTTGACCTTATTTTATCCTTTTTTGGTCCAGTTACTGTTATCATAGTCTTGTATATGAGAGTATTTGTGGTGGCTGTGTCTCAGGCTCG
TGCCATGCGCTCTCATGTTACATCTGTCACACTTCAGCGTCCACTGAATCAGACAAACAAATCTGAGCTGAAAGCAGCCAGGAGTCTTGGGGTTCTTGTAGTTGTATTT
CTAGCATGCTTCTGTCCACTCTACTGCTACTCTCTTGTTGATGAAAATGCTGTCAATGATCCCTCTGCATCTTTTGTGGTCATTATCTTTTACATTAACTCTTGTCTAAACC
CATTGATCTACGCCTTGTTTTACCCCTGGTTTAGAAATGCTGTTAAACTTATCATCAGTTTGGAGATCTTCAAGTATGACACCagtggggccaacatactataa
>contig022354-TilTARs.A037
ATGGAGATACAGGAAGGACCAGAGCTCTGTTTTCCACAACTCCTCAACGATTCCTGCAGAAAGCAAACACTTCACTATTCCAAAACTATGCTGCTGTATATTGTGATGTC
ATGCATCTCTCTTATCACTACTGCTCTAAACCTTCTGGTCATCATCTCAGTCTCCCACTTCAGGCAGCTTCACACACCCAGCAACATCCTCCTCCTCTCTCTGGCCGTTT
CAGACTTTCTCGTGGGTCTCCTGTTGATGCCGTTAGAAATTTTAAGAAGCACCACCTGCTGGGTACTAGGTGATTTTATGTGTTTTCTTTATTGGTATCTGACTGGCAACA
TTACCTGTGTTTCAATAGGTAACATAGTTCTCATATCAGTTGACCGCTATGTGGCTATTTGTGACCCTTTACATTACTCCACCAGAATTACTGTGGCAAAAGTTAAACTCAG
TGTTTGTCTGTGTTGGTTTTATGCTATTTTTAGCATCAGTCTTTATGCAAAAGACTACCTGATTGAATCAGGCAGGTATAATTCCTGTTATGGAGAGTGTGCATTCGTCATT
AATGATATTGCAAGGACTATCGACCTTGTTTTATCCTTTATATTTCCAATTACTGTCATCATAGTTCTGTATATGAGAGTATTTGTGGTGGCTGTGTCTCAGGCTCGTGCCAT
GCGCTCTCATGTTACAGCTGTCACACTTCAGTGTTCACTGAATCAAGCAAACAAATCAGAGTTGAAAGCAGCCAGGACTCTTGGGATTCTTGTAGTTGTGTTTCTACTG
TGTTTCTGCCCATATTACTGTGTCTCTCTTATAAGGGAGGACTTTAGCTCAGTAGCGCCCATTGTGATCAGTCTGTTCTTTTCAAACTCCTGTTTAAACCCAGTGATCTAT
GCCTTGTTCTATCCTTGGTTTAGAAAAGCTTTGAAACTAATTGTCACTTTACAGATACTGCATAGTGGCTCCTGTGAGGTCAGTATACTGtag
>contig022354-TilTARs.A038
ATGGAGATGCAGAAAGGAGCCGAGCTGTGTTTTCCACAACTCCTCAACAGTTCCTGCAGGAAGCCAACACTTCACTGGTCCAAAGCTGTGCTCCTGAACATTGTGCT
GTCCTGCATCTCTCTGCTCACTGCTGCTCTAAACCTTCTCGTCATCATCTCAGTCTCTTACTTCAGGCAGCTTCACACACCCACTAACATCCTCCTTCTTTCTCTGGCTG
TCTCAGACTTTCTCACTGGTCTCCTTATGCTGCCACTAGAAATAATTAGAAACACGGCCTGCTGGGTACTTGGTGATCTTATGTGTTCTATTTATACTTATCTGACAGCTG
GTCTTTTCTGTGCTTCAACTGGGAACATAGTTCTCATTTCAGTTGACCGCTATGTGGCTATTTGTCACCCTCTGCATTACCCCACCAGAATTACTGTGGCAAGAGTCAAA
CTCTGTGTTTGTTTGTGTTGGTTTTGTTCAACTTTCTACAGCAGTCTTTTAACAAAGGATGTTCTAATTAAGCCGGCCAGGTATAATTCCTGTTATGGAGAGTGTGTGTTT
GTCATTAATGATATTGCAGGGTTTATTGATCTTGTGTTATCTTTTATTGTTCCAGTATCTGTTATCATAGTTCTTTATATGAGAGTATTTGTGGTGGCTGTGTCTCAGGCTCG
TGCCATGCGCTCTCATGTTACAGCTGTCACACTTCATCATTCAAGCAATCAAGCAAACAAATCTGAACTGAAAGCAGCCAGGACTCTTGGAGTTCTTGTAGTTGTGTTT
CTCGCATGTTACCTTCCATTTTACTGTTACTCTCTTATTGAAGTAAATGTGATCAATGACTCATCTACATTTTTTCTGATCATTGTGTTTTACTTTAACTCGTGTCTAAACCCA
CTGATCTATGCCCTTTTTTACTCTTGGTTTAGAAATGCTGTTAAACTTATCATCACTCTGCAGATTTTTAAGCATGACACTAGTGAAGCAAACATGttttaa
>contig022355-TilTARs.A039
ATGGAGATACAGAAAGAAAACAAACTGTGTTTTCCGCAACTCCTCAACAGTTCGTGCAGGAAGCCAACACTTCACTGGTCCAAAGCTGTGCTCCTGAACACTGTGCTG
TCCTGCATCTCTCTGCTCACTGCTACTCTAAACCTTCTCGTCATCATCTCAGTCTCTTACTTCAGGAAGCTTCACACACCCAGTAATATCCTCCTCCTCTCTCTGGCTGT
CTCAGACTTTCTTGTCGGTGTCCTGTTGATGCCATTAGGAATCCTTAGAAACACAAGCTGCTGGGTACTTGGTGATATAATTTGTTCTCTTTATTGGTATTTAACCAGCAA
CATTGTCTGTGCTTCAATAGGGAACATAGTTCTCATATCAGTTGACCGCTATGTGGCTATTTGTGACCCTCTGCATTATCCCAGCAGAATTACTTTGGTGAAAGTCAAACT
CAGTGTTTGTCTGTGTTGGTTTTATGCTATTTTCTACTGCAGTCTTTATACAAAGGATATCCTAATTGAACCGGGCAGGGATAATTCCTGCTACGGAGAGTGTGTATTTGT
GATCAATGATATTGCTGTTGTTGTTGACCTTGTTTTTTCCTTTATTGTTCCAGTTTCTGTCATTGTAGTTCTGTATATGAGAGTATTTGTGGCAGCTGTGTCTCAGGCTCGT
GCCATGCGCTCTCATGTTACATCTGTCACACTGCAGCGCTCACTGAATCAGACAAACAAATCTGAGCTGAAAGCAGCCAGGACTCTTGGAGTTCTTGTAGTTGTGTTTC

TTGCATGCTTCTGTCCACTCTACTACTTTTCTCTTGTTGATGAAAATGCAATCAATGATCCATCTGCATCTTTTGTGGTCATTATATTTTACTTTAACTCTTGTATAAACCCTT
TGATCTATGCCTTGTTTTACCCCTGGTTTAGAAATGCTGTTAAGCTTATCATCACTCTGCAGATTTTCAAGTATAACATAAGTGAGGCCAACATACTAtaa
>contig022356-TilTARs.A040
ATGGAGATACAGAAAGGAGCTGAGCTTTGTTTTCCACAATTTCTCAACAATTCCTGCAGGAAGCCGACACTACGTTCGTCGAAAGCTGTGCTCCTGAACATTGTGTTCT
CCTGTATCTCTCTGCTCACTGCTGCTCTAAACCTTCTCGTCATCATCTCAGTCTCTTACTTCAGGAAACTTCACACACCAAGTAACATCCTCCTCCTCTCTCTGGCTGTC
TCAGACTTTCTTGTGGGTCTTCTGTTGATGCCTTTAGAAATATTTAGAAGCACGACCTGCTGGGTACTTGGTGACGTTATGTGTTCTGTTTATTGGTATCTGACCGGCAA
CATTACCTGTGCTTCAATAGGGAACGTGGTTCTAATATCAGTTGATCGTTATGTGGCTATTTGTGACCCTTTGTATTACCGCACCAGAATTACTGTGGAAAGAACCAAACT
AAGTGTGTGCTTATGTTGGTTTTATGCTATTTTCTACTGGAGTCTTTATATAAAGAATATCCTGATTGAACCGGGCAGGTATAATTCCTGTTACGGAGAGTGTATGTTTGTA
AGCAGTGATATTGCAGGGATTATTGACCTTGTTTTATCTTTTATTGTTCCAGTTTCCGTCATCATAGTTCTTTATATGAGAGTATTTGTGGTGGCTGTGTCTCAGGCTCGTG
CCATGCGCTCTCATGTTACAGCTGTCACACTTCAGCATTCAAACAATCAAGCAAACAAATCTGAGCTGAAAGCAGCCAGGACTCTTGGGGTTCTTGTAGCTGTGTTTCT
GTCATGTTACTCTCCATTTTACTGCTACTATCTTGCTGAAGAAAATGTGGTCAATGATCCATCTGCATCTACTGTTATCATTATTTTTTACTTTAACTCTTGTATAAACCCTTT
GATATATGCGTTGTTTTACCCCTGGTTTAGAAATGCTGTTAAACTTATTATCACTCTGCAGATCTTCAAGCATAACACCTGTGAGGCCAACATACTAtaa
>contig022357-TilTARs.A041
ATGGTGATACAGAAAGGAGCTGAGCTCTGCTTTCCACAACTCCTCAACAGTTCATGCAGGAAGCCAACACTTCACTGGTCCAAAGCTGTGTTTCTGAACATTATGCTGC
CCTGCCTCTCTCTGACCACTGCTGCTCTAAACCTTTTGGTCATCATCTCAGTCTCCCACTTCAGGCAACTTCATACACCCACTAACATCCTCCTTCTGTCTCTGGCTGTC
TCAGACTTTCTCACGGGTTTCCTGATTTTGCCATTAGAAATAATTAGAAACATGGCCTGCTGGGTACTTGGTGATCTCATGTGTTCTGTTTATACTTATTTGATCACCAGTC
TTTTCTGTGCTTCAACTGGGATCATAGTTCTAATATCAGTTGACCGCTATGTGGCTATTTGTGACCCTCTGCATTACCCCACCAGAATGACTGTGGTGAGATCCAAGCTT
AGTGTGTGCCTATGCTGGTTTTATTCGACTTTCTACAGCAGCCTTCAAACAAAGGATGTTCTAATTAATCCAGGCAGGTATAATTCCTGCGACGGAGAGTGTGTGTTTCT
CATCAGTAATATTGCAGGGACCATTGACCTTGTTTTATCTTTTATTTTTCCAGTTTCTGTCATCATAATTTTGTATATGAGAGTATTTGTGGTGGCTGTGTCTCAGGCTCGT
GCCACGCGCTCTCATGTTACAGCTGTCACACTTCAGAGTTCACTGAATCAAACAAACAAATCTGAGTTGAAAGCGGCCAGGACTCTGGGGATTCTTGTAGTTGTGTTT
CTTGCATGCTACTGTCCATTTTACTGCTATTTTCTTGTAAAAGAAAATGTGGTCAATGATTCAGCTACATTTTTTGTGATCATTTTATATCATGTTAACTCTTGTTTGAATCCA
TTAATCTATGCCTTGTTTTACCCCTGGTTTAGAAATGCTGTTAAACTTATCATCACTTTTCAGATATTCACGCATGACACCAGTGGGGCCAACATACTAtaa
>contig022362-TilTARs.A042
ATGGAGATACAGAAGGGAGCCGAGCTTTGTTTTCAACAACTACTCAACAATTCCTGCAGGAAGCTGACACTTAACTGGTCCAAAACTGTGCTTCTGTACATTGTGCTGT
TTTGCATCTCTCTGATCACTGTTGCTCTGAACCTCCTGGTCATCATCTCGGTCTCCCACTTCAGGCAGCTTCACACACCCACTAACATCCTGCTTCTCTCTCTGGCTGT
CTCAGACTTTCTTGTTGGTCTCCTGTTGATACCTTTAGAAATCCTTAGAAGCACAACCTGCTGGGTATTTGGTGATGTTATATGTTCTTTTTATTTTTATCTGACTGGCAAC
ATTACCTGTGCTTCAACAGGAATCATAGTTCTAATATCAGTTGACCGCTATGTGGCTATTTGTGACCCTCTGCATTACCCCACCAGAATGACTGTGGCGAGGGTGAAACT
CAGTGTTTGTCTGTGTTGGTTTTATGCTATTTTCTATGTCACTCTTTACCTAAAAGACTTCCTGAAGCCAGGCAGGTATAACTCCTGCTATGGAGAGTGTATCCTTGTCAT
CAATGATACTGCAGGAACTGCTGACCTTGTTTTATCTTTTTTTGTTCCCATCACTGTCATTATAGTTCTGTATATGAGAATATTTGTGGTGGCTGTGTCTCAGGCTCGTGCA
ATGCGCTCTCATGTTACAGCAGTTGCAAAATCCAAATCTGAGTTGAAAGCAGCCAGGACTCTGGGTGTTCTAGTAGTTGTGTTTCTTGCAAGCTACTGTCCATTTTACTG
TTACACCCTCGTTGAAGAAAATGTGATCAGTGATTCATCTGCATTTTTTGTGGTCATTGCCTTTTATTTGAACTCTTTTCTAAACCCAATGATCTATGCCTTGTTTTATCCAT
GGTTTAAAAAATCTGTTAACCTTATCATCACTCTGCAGATCTTTAAGCGTCACAGCAGTGATTCTAACATATTAtaa
>contig022363-TilTARs.A043
ATGGAGATACAGAAAGGAGCCGAACTTTGTTTTCAACAACTACTCAACAATTCCTGCAGGAAGCCAACACTTCACTGGTCCAAAGCTGTACTTCTATACATTGTGCTGTT
CTGCATCTCTCTGATCACTGTTGCTCTGAACCTCCTGGTCATCATCTCAGTCTCCCACTTCAGGCAGCTTCACACACCCACTAACATCCTCCTCCTCTCTCTGGCTGTC
TCAGACTTTCTTGTTGGCCTCCTGTTGATACCTTTAGAAATCCTTAGAAGCACAACCTGCTGGGTATTTGGTGATGTTATGTGTTCTGTTTATTGGTATCTGACTGGCAAC
ATTACTTGTGCTTCAATAGGGAACATAGTTCTAATATCAGTTGACCGCTATGTGGCTATTTGTGACCCTCTGCATTACCCCACTAGAATGACTGTGGTGAGAATCAAACAC
TGGATTTGTTTGTGTTGGTTTTATGCTATTTTCTATGACACTTTATACCTAAAAGACTTACTGATTAAACCAGGCAGGTATAATTCCTGCTATGGAGAGTGTGTACTTGTCAT
CAATGATATTGCAGGAACTGCTGACCTTGTTGGTGGTTCCTTTGGTTCTTACCACCGTACATCGCCATGGtaa
>contig022363-TilTARs.A044
ATGGAGATAAAGAAAGGAGACGAGCTTTGTTTTGTAGAGCTCAACAGTTCCTGCAAGAAGCCAACACTTCACTGGTCCAAAGCTGTGCTCCTGAACATTGTGCTGTCC
TGTATCTCTTTGATCACTGCTGTTCTGAACCTTCTCGTCATCATCTCAGTCTCTCACTTCAGGCAGCTTCACACACCCACTAACATCCTTCTCCTCTCTCTGGCTGTCTC
AGACTTTCTTGTTGGTTTCCTGTTGATGCCATTAGAAATCTTTAGAAGCACAACCTGCTGGGTGTTTGGTGATGTTATGTGTTCTGTTTATTGGTATCTGACTGGCAACAT
TACCTGTGCTTCAATAGGGAACATAGTTCTAATATCAGTTGACCGCTATGTGGCTATTTGTGACCCTCTGCATTACCCCACTAGAATGACTGTGGTGAGAATCAAACACT
GGATTTGTTTGTGTTGGTTTTATGCTATTTTCTATGACACTTTATACCTAAAAGACTTACTGATTAAACCAGGCAGGTATAATTCCTGCTATGGAGAGTGTGTCCTTGTCAT
CAATGATATTGCAGGAACTGCTGACCTTGTTTTATCGTTTTGTGTTCCAATTTCTGTTATCATAGTTCTGTATATGAGAGTATTTTTGGTGGCTGTGTCTCAGGCTCGTGCC
ATGCGCTCTCATGTAACAACTGTCACACTGCAGCGTTCATTGAATCAAACAAACAAATCTGAGCTGAAAGCAGCCAGGACTCTTGGGATTCTTGTAGTTGTGTTTCTACT
TTGTTTCTGCCCGTATTACTGTGTCTCTCTTGTAAGGGACAGAAATAGCTCAGTAGCCCCTAATTTAATGAGTGTGTTTTTTTTAAACTCATGTCTAAACCCGTTGATCTAT
ACTATGTTCTATCCTTGGTTTAGAAAAGCTGTAAAACTAATTGTCACTCTACAGATCCTACAGACTGGCTCCTGTGAGGTCAGTATACTGtag
>contig022365-TilTARs.A045
ATGGAGAAAGGAGCTGAGCTCTGCTTTCCACAACTCAACAGTTCCTGCAGGAAGCCGACACTTCACTGGTCCAAGGCTGTGCTGCTGACCATTGTGCTGTTCTGCAT
CTCTCTGATCACTGTTACTCTGAACATCTTAGTCATCATCTCAGTCTCCCACTTCAGGCAGCTTCACACACCCAGTAACATCCTCCTCCCTTCTCTGGCTGTCTCAGACT
TACTTGTTGGCCTCCTGTTGATGCCTTTAGAAATCTTTAGAAACACAAGCTGCTGGGTATTTGGAGATCTCATATGTTCGGTCTATTGGTATCTGAGTGGCAACATTACCT
CCACTTCAACAGGGAACATAGTTCTTATATCAGTTGACCGCTATGTGGCTATTTGTGACCCCCTTCATTATCCCACCAGAATGACTGTGGTGAGAGTCAAACTTAGTGTT
TGTCTGTGTTGGTTTTATTATATTTTTTACATCAGTTTTTACCTTAAAGACATCCTGATTGAACCAGGCAGGTATAATTCCTGCAAAGGAGAGTGTGTACTTGTCATCAATG
ATATTGCAGGGATTATTGACCTTGTCATAGCTTTTATTGTTCCAGTTTCTGTAATCATAGTTCTGTATATGAGAGTATTTGTGGTGGCTGTGTCTCAGGCTCGTGCCATGCG
CTCTTGTGTCAGAGCAGTAACACTTCAGCATTCACTGAATCAAGCAAACAAATCTGAGTTGAAAGCAGCCAGGACTCTTGGGATTCTTGTAGTTGTGTTTCTTGCAAGC
TTCTGTCCATTTTACTGTTACTTTTTTGTTGGAGAAAATGTGGTCAGTGATTCATCTGCATTTTTTGTGATCATTGCCTTTTATTTTAACTCTTTCCTAAACCCAATGATCTAT
GCCTTGTTTTATCCTTGGTTTAGAAATGCTGTTAAGCTTATCATCACTCTGCAGATCTTTAAGCGTCACAGCAGTGATACTAACATATTAtaa
>contig022368-TilTARs.A046
ATGGAGATACAGAAAGGAGCCGAGCTTTGTTTTCAACAACTACTCAACAATTCCTGCAGGAAGCTGACACTTCACTGGTCCAAGGCTGTGCTTCTGTACATTGTGCTGT
TCTCCATCTCTCTGATCACTGTTGCTCTGAACCTCCTGGTCATCATCTCAGTCTCCCACTTCAGGCAGCTTCACACACCCACTAACATCCTGCTTCTCTCTCTGGCTGT
CTCAGACTTTCTTGTTGGCCTCCTGTTGATACCTTTAGAAATCCTTAGAAGCACAACCTGCTGGGTATTTGGTGATGTTATATGTTCTGTTTATTGGTATCTGACTAGCAA
CATTACCTGTGCTTCAACAGGAATCATAGTTCTAATATCAGTTGACCGCTATGTAGCTATTTGTGACCCTCTGCATTACCCCACTAGAATGACTGTGGCGAGGGTGAAAC
TCAGTGTTTGTCTGTGTTGGTTTTATGCTATTTTCTACGTCACTCTTTACCTAAAAGATTTCCTGATTAAACCAGGCAGTTATAATTCCTGCTATGGAGAGTGTGTCCTTGT
CATCAATGATACTGCAGGAACTGCTGACCTTGTTTTATCCTTTTTTGTTCCCATCATTGTTATCACAGTTCTGTATATGAGAGTATTTGTGGTGGCTGTGTCTCAGGCTCG
TGCCATGCGCTCATATGTTACAGCTGTCACACTTCAGCGTTCACTGAATCAAACAAACAAATCTGAGTTGAAAGCAGCCAGGACTCTGGGTGTTCTAGTTGTTGTGTTT
CTTGCAAGCTACTGTCCGTTTTACTGTTATACCCTTGTTGAAGAAAATGTGGTCAGTGATTCATCTGCATTTTTTGTGGTCATTGCCTTTTATTTTAACTCTTTTCTAAACC
CAATGATCTATGCCTTCTTTTATCCTTGGTTTAGAAAGTCAGTTAAACTTATCATCACTCTGCAGATCTTTAAGCGTCACAGCAGTGATTCTAACATATTAtaa
>contig022368-TilTARs.A047
ATGGAGAAAGGAGCTGAGCTCTGCTTTCCACAACGCCCCAACAGTTCCTGCGGGAAGCCGACACTTCACTGGTCCAAGGCTGTGCTTCTGTACAGTGTGCTGTTCTG
CATCTCTCTGATCACTGTTGCTCTGAACCTCTTGGTCATCATCTCAGTCTCCCACTTCAGGCAGCTTCACACACCCAGTAACATCCTCCTCCTCTCTCTGGCTGTCTCA
GACTTACTTGTTGGTCTTCTGTTGATGCCATTAGAAATCCTTAGAAGCACAACCTGCTGGGTATTTGGTGATGTTATATGTTCTGTTTATTGGTTTCTGAGTGGCAACATTA
TCTGTGCTTCAATAGGGAACATAGTTCTAATATCAGCTGACCGCTATGTAGCTATTTGTGACCCCCTTCATTATCCCACTAGAATGACTGTGGTGAGAGTCAAACTTAATG
TCAGTCTGTGTTGGTTTTATTATGTTTTTTACATCAGTCTTTACCTTAAAGACATCCTAATTAAACCAGGCAGGTATAATTCCTGCTATGGAGAGTGTGTGCCTGTCATCAA
TGATATTGCAGGGATTATTGACCTTGTCTTAACTTTTATTGTCCCAGTTTCTGTCATCATAGTTCTATATATGAGAGTATTTGTGGCAGCTGTGTCTCAGGCTCGTGCCATG
CGCTCTCATGTTACAGCAATAACATTTCAGCGTTCATTGAATCAAGCAAACAAATCTGAGTTGAAAGCAGCCAGGACTCTGGGTGTTCTTGTAATTGTGTTTCTAGCATG
CTACTGTCCTTTTTTCTGTTATACCCTTGTTGAAGAAAAAGTAGTCAGTGATTCATTTGTGTTTTTTCTGATCATTGCCGTTTATTTTAACTCTTTTCTAAACCCACTAATCTA
TGCCTTCTTTTATCCTTGGTTTAGAAATGCTGTTAAGCTTATCATCACTCTGCAGATCTTTAAGCGTCACAGCAGTGATGCTAACATACTAtaa
>contig022375-TilTARs.A048
ATGGAAATGCAGAAAGGAACCGAGCTTTGTTTTCCAGATTTCCTCAACACTTCCTGCAGGAAGCCAACACTTCACTGGTCCAAAGCTGTGCTGTTGAACATTGTGCTG
TCCTGCATCTCTTTGATCACTTCTGCTCTAAACCTTCTTGTCATCATCTCAGTCTCCCACTTCAGGCAGCTTCACACACCCACTAACATCCTGCTCCTCTCTCTGGCTGT
CTCAGACTTTCTTGTGGGTCTTCTGTTGATGCCATTAGAAATCTTTAGGAGCACAGCCTGCTGGGTACTTGGTGATCTTATGTGTTCTGTTTATTGGTGTCTGACCGGCA
ACATTATCTGTGCTTCAATAGGGAACATAGTTCTTATATCAATTGACCGCTATGTGGCTATTTGTGACCCTTTGCATTACCCCACCAGAATGACTGTGGCAAGAGTCAGAG
TCTGTGTTTGTCTGTGTTGGTTTTTTGCTATTTTCTACAGCAGTCTCTATACAACAGACTTCCTAATTGACCCAGGCAGGTATAATTCTTGCTATGGAGAGTGTGTGTTTG
TCATCAGTGCCATTGCAGGCATTTTTGACCTTGTTGTATCTTTTTTTGTTTCAGTTACTGTTATCATAGTTCTGTATATGAGAGTATTTGTGGTGGCTGTGTCTCAGGCTCG
TGCCATGCGCTCTCATGTAACAACTGTCACACTTCAGCGTTCACTAAACCAAACAAACAAATCTGAGCTGAAAGCAGCCAGGACTCTTGGAATTCTTGTAGTTGTGTTT
CTACTTTGTTTCTGCCCGTATTACTGTGTCTCTCTTGTAAGAGATGAAAATAGCTCAATTGCCTCCACAGTTATAAGTGTGTTTTTTTTAAACTCCTGTTTAAACCCTTTGA
TCTATGCTATGTTCTACCCTTGGTTTAGAAAAGCTGTGAAACTAACTGTCACTCTACAGATCCTCCGGACTGGCTCCTGTGAGGTCAACATACTGtag
>contig022377-TilTARsp.A049
ATGGAGATACAGAAAGGAGCCGAGCTATGTTTTCCACAACATCTCAACAATTCCTGCATCAAGCCGACACTTCACTGGTCCAAAGCTGTGCTCCTGAACATTGTGCTGT
CATGCATCTCTCTGATTACTGCTGTTCTCAGTCTTCATCTCAGTCTCTCATTTAGGCAGCTTCACGCACCCAGTAACATCCTCCTCCTCTCTCTGGCTGTCTCAGTCTTT
CTTGTGGGTCTCCTGTTGATGCCATTAGAAATTGCTAGAAACACGGCATGTTGGGTACTTGGTGATCTTATGTGTTCTGTTTATATCCATTTAACTGGCATTCTAATCTGT
GCTTCAGTAGGGAATATAGTTCTAATATCAATTGACCGCTATGTGGCTATTTGTGACCCTTTACATTATCCCACAAAAATTACATTGGAAAGAGTTAAACTGAGTGTTTGTC
TGTCTTGGCTTTTTTCAATTTTCTACAGCAGTCTTTATGCAAAGGATGTCCTGATTGAACCAGGAAAGTATCATTCCTGTTATGGAGAGTGTGTATTCCTTATTAATAATATT
GCAGGTATGATTGACCTTGTTTTGACTTTTTTGGTTCCAGTTTCTGTCATCATAGTTCTATAAATGAGAGTATTTGGGGTGGCTGTGTCTCAGGCTCGTGCCATGCGCTC
TAATGTTACAGCTGTCACACTTGAGCGTTCACTGAATCAAACAAACAATTCTGAGCTGAAAGCGGCCAGGACTCTGGGGGTTCTTGTAGTAGTGTTCCTGGCAAGCTA
CTGTCCATTTTATTGCTACACTCTTGTTGAAGAAAATGTGGTCAATGATCCATCTGCATCTACTGTGATCATTGTCTTTTACTTTAACTCTTGTCTAAACCCTTTGAGCCAT
GCCCTGTTTTACCCCTGGTTTAGAAATGCTGTTAAACTCATCATCACTTTTCAGATATTCAAGCATGACAGTAGTGAGGCCAATATACTGTAA
>contig022377-TilTARs.A050

ATGGAGAAAGGAGCTGAGCTCTGCTTTCCACAACTCCTCAACAGTTCCTGCAGGAAGCCAACACTTCACTGGTCCAAAGCTGTGCTCCTGAACATTGTGCTGTCCTG
CATCTCTCTGATCACTGCTGGTCTCAATCTTCTTGTCATCATCTCAGTCTCCCACTTCAGGCAGCTCCACACGCCCAGTAACATCCTCCTCCTCTCTCTGGCTGTGTCA
GACTTTCTTGTTGGTCTCCTGTTAATTCCAGCTGAGATTTTTAGAATCACAGTCTGCTGGGTATTTGGTGAATCCATGTGTTCACTTTATACATATCTAGGCTACATTGTTG
TAACATCTTCAATAAGTAACATAGTTCTCATATCAGTTGATCGCTATGTGGCTATTTGTGATCCTCTGCATTATCCCAGCAGAATTACTGTGGCAAGAATCAAACTTAGTGT
TTGTCTGTGTTGGTTTTATTCGGCTTTCTATAGCACTCTTTGTACAAAGAATATCCTGATTAAACCAGGCAGGTATAATTCCTGTTATGGAGAGTGTGTGTTTATCACCAGT
GATATTGCTGGCATACTTGACCTTGTTTTATCTTTTATCGTACCAGTTTCCATCATCATAGTTCTTTATATTAGAGTATTTGTGGTGGCTGTGTCTCAGGCTCGTGCCATGC
GCTCTCATGTTACAGCTGCCACACTTCAGAGTTCATTGAATCAAACAAACAAATCTGAGTTGAAAGCAGCCAGGACTCTGGGGGTTCTTGTAGTTGTGTTTCTGGCCAG
TTTCTGTCCATTTTATTGTTACTTTCTTGTTGTAGAAGACATAGTCAGTGATTCATCTGCCTCTATTGTGGTCATTGTCTATTATTTTAACTCTTGTCTAAACCCACTAATCTA
TGCCCTGTTTTACCCTTGGTTTAGAAATGCTGTTAAAGTCATCATCACTTTTCAGATATTCAAGCATGACAGCAGTGAGGTCAACATACTAtag
>contig022378-TilTARs.A051
ATGATGGAGAACAAGGACAGTATATGCTTTCCAGAACTCTTCAACTCTTCCTGCAGGAGACGAGCACTTCACTGGTCTGAAACTGTGCCCCTAAATATCCTGCTGTGCT
CCATCTCTGCGATCACTATAGCTCTGAACCTCCTTGTCATCATCTCAGTCTCCTACTTCAGGCAGCTCCACACACCCACTAACATCCTCCTCCTCTCTTTGGCTGTGTCA
GACTTTCTTGTGGGGTTCCTGTTGCTGCCTGGAGAAATCTTCCTAGGAATAGCCTGCTGGGCTTTCGGTAACCTTTTGTGTTCTCTCTTTAATTATGTGTCTTTCATCATT
ACCTCGGCTTCAGTGGGAAACATGGTTCTCATATCAGTTGACCGCTATGTGGCGATTTGTTATCCTCTGCATTACTCTACCAGAATTACTGTGACCAGAGTTAAACGCTC
TGTTTGTTTGTGTTGGCTCTGTTGTGTTTTGTACAGTAGTGCCCTTTTAAAAGATGAGCTGATTCAACCAGGAGGGCATAATTCCTGCTATGGAGAATGTGTTTTTGTCAT
CAACTTTATTGCAGGAACTGTTGATCTTTTGTTAACCTTTATTGTTCCTATTTCTGTTATCGTAGTTCTGTATATGAGAGTATTTGTAGTGGCTGTGTCTCAGGCCCGTGCC
ATGCGCTCTCATGTTACAGCTGTCACACTGCAGCTTTCAGTGAATCTCACAACAAAGAAATCAGAGTTGAAAGCAGGAAGGACTCTTGGTGTTCTAATAGTTGTGTTTCT
AATGTGTTTCTGTCCATACTATTGCGCTTCTTTTGCAGTGGAAGACTCACTCAGTGAGTCATCCACATCCCTTGTCCGTTATCTCTTCTATTTTAACTCCTGTTTGAACCC
TGTGATCTATGCACTATTTTACCCCTGGTTTAGAAAAGCTATTAAACACATTGTCATGCTTCAGATATTTCACACTGGCTCCCGTGAGGCCAACATACTAtag
>contig022379-TilTARs.A052
ATGGATACACAGGATGTGGCAGAGCTCTGTTTTCCACAACTCTTCAACACCTCCTGCAAGAAACCTATAACTCCTCTGTCAGAATTTGTGTTCCTTCATGTTGTGTTGTC
CTCCATCTCGCTGCTAACTGTGACTCTCAACCTGCTCGTCATCATCTCAGTCTCCCACTACAGGCAGCTTCACACACCCACTAACATCCTCCTCCTCTCTCTGGCTGTC
TCAGACTTTCTTGTTGGTCTCCTGTTGATGCCTGGAGAAATCCTCAGAAATACAGCCTGCTGGTTTCTCGGTGACCTCACCTGTTCTATGTACAATTATATGTCTTTCATT
GTTACCTCTACCTCAGTGGGAGACATGGTGCTAATATCAATTGACCGCTATTTGGCTATTTGTGACCCTCTGCATTACCCCACCAGAATCACAGACAGAAGAGTGAAACT
CTCTATCTGTCTGTGTTGGCTCTCTTCTGTCTTCTATAGCAGCCTGTTTGTAAAGGATGATCTAACTCAACCAGGGAAGCATAATTCCTGCTATGGAGAATGTACAATTGT
TGTTGACTTAATTACAGGAATTATTGACCTTCTTTTAACCTTTTTTGTTCCAGTTACTGTCATTGTAGTTCTGTATCTGAGAGTATTTGTGGTGGCTGTGTCTCAGGCTCGT
GCCATGCGCTCTCATGTTACAGCTACTGCTCTGCAGCTTTCAGTGACTCTAACAACAAAGAAATCAGAGTTAAAAGCAGCCAGGACTCTGGGTGTTCTTGTAGTTGTGT
TTCTACTGTGTTTCTGTCCATATTATTATTGTGTTACTCTTGCCAGGGATGACCCACTCAATAGCTCATCTGTATCCTTTGTGCTCTATCTGTTCTATTTTAACTCCTGTCTA
AACCCTTTGATCTATGCACTGTTCTACCCCTGGTTTAGAAAAGCTGTGAAACTCATAATCTCTTTACACATACTGCAGCCTGGCTCCTGTGAGATTAGCATTTTGtaa
>contig022382-TilTARs.A053
ATGGGCACACAGGAAAAATCTGAGCTCTGCTTTCCACAACTCTTCAACATGTCCTGCAAAAAGCCTGCAATTGCTCGGTCCAAAGCTGTGTTCCTTCATATTGTATTGTC
CTCAGTTTCTTTTCTGACGGTGGCTCTCAACTTGCTTGTCATCATCTCAGTCTCCCACTTCAGGCAGCTCCACACACCCACTAACATCCTGCTCCTCTCTCTGGCTGTC
TCAGACTTTCTTGTTGGCCTCCTATTGTCACCTGCAGAAATCCTCCGAAGTACAGCCTGTTGGTTTCTTGGTCAGCTCACATGTTTAATGTATATTTTTGTGTCCTTGACT
GTTACCTCTGCCTCAGTGGGCATTATGGTGCTGATATCAGCTGACCGCTATGTGGCTATTTGTGACCCTTTGAATTACCCCATCAGGATCACTGACAGAAGAGTTCAACT
CTGTGTTTGTCTGTGTTGGCTCTGCTCCATTGCCTTCAGCTGTTTCCTTGTAAGAGATGACCTGCATCAAACAAGAAAACAAAATTCCTGCTATGGAAAATGTGTAGTTG
TTGTTCAATACATTGCAGGAGTTGTTGACCTCATTTTAACCTTCATTGTACCAGTTAGTGTCATCATAGTTCTGTATATGAGAGTATTTGTGGTGGCTGTGTCTCAGGCCC
GTGCTATGCGCTCTCATGTTACAGCTGTCACAAGTCACCTTCCAGTGACTCTAACAAAAAAGTCTGAACTAAAAGCAGCCAGGACTCTGGGTGTTCTTGTTCTTGTGTT
TCTAATGTGTTTCTGCCCTTATTACTGTGTTTCTCTTGTAGGAGAAGAATTCATCAATAGTTCATCTGCTTCTTTTGTGGCCTATCTGTTTGGTTTAAACTCATGTCTAAAC
CCTTTGATCTATGCTATGTTCTACCCCTGGTTTAGAAAAGCTGTGAAACTAGTTGTCACTCTACAGATACTGCAGCCTGGCTCCTGTGAGGTAAGTATTCTGTAA
>contig022383-TilTARs.A054
ATGGAGACCCAGGACGAAGCAGAGCTCTGCTTTCCACAACTCTTCAACATCTCCTGCAAGAAGCCTAAAACATCCCTGTCTCAAGTTTTGCTCCCTTACGTTGTGGTGT
ATTCAGTCTCTCTGCTAACTGTGGCTCTAAACCTGCTCGTCATTGTTTCAGTCTCCCACTTCAGACAGCTCCACACACCCACTAACATCCTCCTCCTCTCTCTGGCTGT
CTCAGACTTTCTCATTGGTCTGTTGATGATGCCGGCAAAAATCCTACGAGACACAGCTTGTTGGTTTCTTGGTCAGCTCACATGTTTAATATATAATTATATATGCTTCATTA
TTACCTCTGCCTCAGTGGGCATTATGGTGCTGATATCAGTCGACCGCTATGTGGCTATTTGTGACCCTCTGCATTACCCCACCAGAATCACTGAAAAAAGAGTGAAACTT
TGCGTCTGTCTGTGTTGGCTCTGCTCTGTTTTTTACAACATACTATTTATAAAGGACGACCTGCTTCAACGAGAGCAACATACTTCCTGTTATGGAGAATGTGTATTTGTC
ATAGACTACATTGTAGGAACCACTGACATTGTTGTGACTTTTATTGCTCCAGTTACTGTCATTGTAATTTTGTATATGAGAGTATTTGTGGTGGCTGTGTCTCAGGCCCGT
GCCATGCGCTCTCATGTTACAGCTGTCACACTGCAGCTCTCAGTGACTCTAACAGCAAAGAAATCAGAGTTAAAAGCTGCCAGGACTCTGGGTGTTCTTGTTCTTGTG
TTTCTATTATGTTTCTGCCCATATTACATTGTTTCACTTTTAGGAAACGAGTTGTTCAACAGCTCATCTGCATCTATTGTGATCTACCTGTATTATTTTAACTCCTGTCTAAAT
CCTTTGATTTATGCTATGTTCTACCCCTGGTTTAGAAAAGCTGTGAAATTAGTTGTCACTCTACAGATACTGCAGCCTGGCTCCTGTGAGGTCAGCATACTGtaa
>contig022390-TilTARs.A055
ATGGATACACAGGAAGGAGCAGAGCTCTGCTTTCCACAACTCTTCAACATCTCCTGCAAGAAGCCTACAACTCCTCTGTCTCAAGTTTTGCTCCTTTATATTGTTTTGTC
CTCAATGTCTCTGCTAACTGTGACTCTCAACCTGCTCGTCATCATCGCAGTCTCCCACTTCAGGCGAAATACAGCATGCTGGTTTCTTGGTCAGCTCACATGTTCTCTG
TATAATTATGTATCCTACATCGTTGCCTCTGCCTCAGTGGGCAACATGGTGCTGATATCAGTCGACCGTTATGTGGCTATTTGTGACCCTTTGCATTACCCCTCCAGAATC
ACTGACAAAAGAGTGAAACTCTGCGTCTGTCTGTGTTGGCTCTGCTCTGTTTTCTACAGCTATGTGATTTTAATAGATGATCTAAGTCAACCAGGCAAGCATAATTCTTGT
TATGGAAAATGTATAATTTTCATTGAATTCATTGCAGGATTTGTTGACCTTGTTTTATCCTTTATAATTCCACTTACTGTCATCGTAGTTCTGTATATGAGAGTATTTGTGGTG
GCTGTGTCTCAGGCCCGTGCCATGCGCTCTCATGTTACAGCTGTCACACTGCAGCTCTCAGTGACTCTAACAGCAAAGAAATCAGAGTTAAAAGCTGCCAGGACTCTG
GGTGTTCTTGTTCTTGTGTTTCTAATATGTTTCTGCCCATATTACATTGTTTCGCTTTTTGGAGACGAGTTTCTAAACAGCTCATCTGCATCCATTGTGATCTATCTGTATTA
TTTTAACTCCTGTGTAAACCCTTTGATTTATGCTATGTTCTACCCCTGGTTTAGAAAAGCTGTGAAACTAATTGTGACTCTACAGATACTGCAGCCTGACTCCTGTGAGGT
CAGCATACTGtag
>contig022390-TilTARs.A056
ATGGATACACAGGATGGATTAGAGCTCTGCTTTCCACAACTCTTCAACATCTCCTGCAAGAAGCCTACAACTCCTCTGTCTCAAGTTTTGCTTCCTTATATTGTTGTGTTC
TCAATGTCTCTGCTAACTGTGACTCTCAACCTGCTCGTCATCATCGCAGTCTCCCACTTCAGACAGCTCCACACACCCACTAACATCCTCCTCCTCTCTCTGGCTGTCT
CAGACTTTCTCATTGGTTTGTTGTTGATGCCTGCAAATATCATCCGAAAGAAAGTTTGTTGGTTTCTTGGTCAGCTCACATGTTTAGTCTATACTTATGTATCCTTCATTATT
ACCTCTGCCTCAGTGGGCATCATGGTGCTGATATCAGTTGACAGGTATGTGGCTATTTGTGACCCTCTGCATTACCCCACCAGAATAACTCATGGAAGAGTGAAACTCT
GTGTCTGTCTGTGTTGGCTCTGCTCTGTTATCTACAACATTTTGCTCATAAAGGATGAACTGGTTAAACCAGGGCAACATATTTCCTGTTATGGAGAATGTAAAATTCTCG
TAGACTACATTGCAGGAACCACTGACATTGTTTTAACCTTTATTATTCCTGTTACTGTCATCATAGTTCTGTACATGAGAGTATTTGTGGTGGCTGTGTCTCAGGCCCGTG
CCATGCGCTCTCATGTTACAGCTGTCACAATGCAGCTCTCAGTGACTCTAACAACAAAGAAATCAGAGTTAAAAGCAGCCAGGACTCTGGGTGTTCTTGTTCTTGTGTT
TCTAATATGTTTCTGTCCTCATTATTGTATTTTTGTTTTTGGAGGAAAGTTGCTCAGTAGCTCGTCTGCAACCATTGTGATCTATCTGTATTATTTTAACTCCTGTGTAAACC
CTTTGATTTATGCTATGTTCTACCCCTGGTTTAGAAAAGCTGTGAAACTAACTGTCACACTACAGATACTGCAGCCTGGCTCCTGTGAGGTCAGCATACTGtag
>contig045088-TilTARs.A057
ATGGAGGGGGTTGAATTCTGCTTTCCACATCTGCTCAACTCCTCTTGCAGAAAGGCTCTGCGTCCTGTCTCAGTTTCCATGCTCATTTACATGATAATATCTTCTATCTCT
GTGCTCACTGCAACTCTTAACCTGCTGGTCATCGTCTCCATCTCCCACTTCAAGCAGCTCCATACTCCCACCAACTTCCTCCTCCTCTCTCTGGCTGTCTCCGATTTCT
TTGTGGGTCTCTATTTGCTCTTTTACATAATGTTTATAGACGGCTGCTGGTATTTTGGTGAATTCATGTGTATTCTGTATTATGTTATTGGCACAATTAACACCTCTTCCTCA
ATAGGAACCATGGTACTGATATCAGTTGACCGTTATGTGGCCATTTGTGATCCTCTGCATTATCCCATCAAAGTCACTGCAAAAAGAGTTCAGACCTGTGTTTCACTGTG
TTGGAGCTTTTCTGCCCTTGCTGGTAGTTACCTCTTAAAGGACAACTTGAAACAGCAAAGCAGGTTTAATTCTTGTGTGGGAGAGTGTGTTGTCCATATTAACTTTATTG
AATATGTTGCAGATCTTGCTTTGAACTTCCTACTTCCTATTACTGTCATTATAGTTCTGTATTTGAGAATATTTGTAGTGGTTGTGTCTCAGGTTCGGGCCATGCGGTCTCA
TACTGCAGGTGTCACATACCAGTGTTCAAGGAAGGGAAATCCAAAGAAATCAGAGATGAAAGCAGCCAGGACTCTTGGTATTGTTGTAATTGCATTCTTGTCATGCACC
TTACCATTTTATTGTGTCACACTCACAGGCCAGAATGCCTTCCTCAACGGATCATCTTCTGCATTTGTTCTATGTCTTTTCTATTTTAATTCGTGCCTCAACCCTATCGTCT
ATGCCTTATTCTACCCCTGGTTTAGAAAATCTATTAAGTTTATTGTTACATTTCAAATACTGAAGTCTGGCTCCAGGAATGCCAACATAGTCAAAGTGACAGAAtag
>contig056134-TilTARs.A058
ATGGAGGAAACTGAACTCTGCTTTCCAAAACTTCTTAACACCTCCTGCATGAGGCTGAGGCGTCCCCACTTTGAGATCATGCTGACTTATATTCTGCTGTCTTCAATCG
CTCTGTTAACTGTGACTCTTAATCTGTTTGTCATCATCTCCATCTCACACTTCAGGCAGCTCCACACCCGCAACAACGTCCTCCTTCTGTCACTCTCCGCAGCTGATTTT
GGTGTAGGTCTTGTCATGTTCTTCCAAATTGTGCTCATAGATGGCTGCTGGTTCCTCGGTGACATCATGTGCACTCTGTATCAATACCTAGCATATGTCATCACCTCGGC
CTCAATAGGAACCATGGTGATCATATCTGTTGATCGATATTTGGCTATTTGTTACCCTCTACATTACTCCACCAAGATCACACAACAAAGAGTTAAAATTGTTGTCTGTTTG
TGTTGGATCTGTTCTGTCATCTTTCAAAGTCTGATTCTGATGGATAACCTGAAGCAACCAGGCAGGTATAACTCTTGCATTGGAGAGTGTGTCTTTGTCATTAATTACATC
GCAGGACTTGTTGATGTTACTTTTTCCTTTATTGTTCCCATTACTGTAATTGTAGTTTTGTATCTGAGAGTGTTTGTGGTGGCTGTGTCTCAGGCTCATGCCATGAGGTCT
CAGCTTGCAGTCACTCACCAGCAATCAGTTACAGTTACTGCAAAGAAATCGGAGCTGAAAGCAGCCTGGACTCTTGGTATTGTTGTCGTTGTGTTTCTCATATGTATGT
GTCCATATTACTGTGTGGCTCTCACAGTCAAAGACAATGCACTGAGTGCTTCTTCTGCTGCTTTTGTGATATATTTATTCTTCTTTAACTCGTGTCTGAACCCCATCTTATA
TGTCTTTTTTTACTCGTGGTTCAGAAAATCCATAAAAGTCATTGTTACTCTTCAGATACTGCAGCCTGACTCTTCTAAGGCCAACATGCTTtag
>contig022320-TilTARs.A059
ATGGAGATTCAGAAAGCAGCTGAGCTTTGTTTTCCACAACTCCTCAACAGTTCCTGCAGGAAGCCGACACTTCATTGGTCCAAAGCTGTGCTCCTGAACATTGTGCTG
TTCTGCATCTCTGTGATCACTGCTGCTCTAAACCTTCTGGTCATCATCTCAGTCTCCCACTTCAGGAAGCTTCACACACCCAGTAACATCCTGCTCGTCTCTCTGGCTG
TCTCAGACTTTCTTGTGGGTCTTCTGCTGATGCCATTAGAGATCTTTAGAAACACAGCCTGCTGGGTACTTGGTGATGTAATGTGTTCTGTTTATTGGTACCTGACCAGC
AACATTACCTGTGCTTCAATAGGGAACATAGTTCTAATATCAGTTGACCGTTATGTGGCGATTTGTGATCCTCTGCATTACCCCACCAGAATTACTGTGGTCAGAGTCAAG
CTCAGTGTGTATTTGTGTTGGTTTTTTTCTATTTTCTACTGGAGTCTTTATATGAAAGATATCCTAGTTGAACCAGGCAGGTATAATTCGTGCTATGGAGAGTGTGTGCTTA
TTATTAATGACATTGCAGGGATTGTTGACCTTGCTTTGTCCTTTATAGTTCCAGTTACTGTTATTATAGTTTTGTATATGAGAGTATTTGTGGTGGCTGTGTCTCAGGCTCG
TGCCATGCGCTCTCATGTTACAGCTGTCACACTTCAGTGTTCACTGAATCAAGCAAACAAATCTGAGCTGAAAGCAGCCAGGACTCTTGGGGTTCTTGTAGTTGTATTT
TTGGCATGCTACTGCCCATTTTACTGCTACTCTCTTGTTGATAAAAATGTGGTCAATGATTCATCTGCATCTTTTGTGGTTCTTGTCTTTTATTTTAACTCATGTTTAAACCC
ATTGATCTATGCTCTGTTTTACCCCTGGTTTAGAAAAGCTATTAAATGTGTTATCACTCTGCTGATATTTAAGCATGACAGCAGTGAGGTCAACGTAATAtag
>contig032272-NyeTAR.A004

ATGGACAGGCAGGACAGAATTGAACTCTGCTACCCTGAGCTTGCGAATCTGTCTTGCAGGGTTTTAACACGTCCTCAGCCTGAGGCTGTCCTCCTCTATACACTGTTC
TTGTCTATCTCTGTGCTAACTGTAGCTCTGAATGTGCTGGTTATCATATCCATCTCTCATTTCAGGCAGCTCCACATGCCCACCAATGTCCTCCTGCTCTCTCTGGCCAT
CTCAGACCTCTTGGTCGGGCTGCTTGTGATGCCGGTGGAAGCAATGCGGTTTATAGAAACCTGCTGGCTGCTGGGAGATCTCATGTGTGCTTTTACTTACATTATCGGT
TTCACTCTCACCTCAGCCTCTGTGGGCAACATGGTGCTCATATCAATTGATCGCTACGTAGCTATTTGTTATCCTCTGCAGTACCCCACTAAAATCACTCACAGCAGAGC
GGAGCTGTCTGTGACTCTGTGCTGGGCCTGTTCGCTCCTCTACAACGGGATGATCTTAAAGGAGCATCTCAGGCAGCCAGACAGGCATAACACCTGTCACGGTCAGT
GTTTGGTGGTGATTAACTATGTCCCTGGAGCCATCGACCTTGTCTTTACTTTTATCGGCCCTTGCTCAGTAATTATCATTCTGTACATGAGAGTATTTGTTGTAGCGGTGT
CTCAGGCTCATGCCATGCGGTCTCATATTACAGCTGCTGCAGCTGGTACAGTAAAAATCACCGCAAAGAAATCAGAGAAAAAGGCAGCCAGGACTCTTGGCGTTGTGA
TATTTGTGTTTTTGATGAGTTTCTGTCCGTATTACTATCCCTCTCTTGCAGGGCAGGACATTTCAAACAGTGCTTCATCTTGGGCTATTGTTTCCTGGATGCTGTACTTCA
ATTCTTGTCTGAATCCACTGATATATGCTTTTTTCTACCCGTGGTTTAGAAAAGCTATCTGGTTCATTGTCTCCCTGAAGATACTAGAGAAGGGCTCCTCTCAGGCAAATA
TACTTtaa
>contig038663-NyeTAR.A005
ATGGATAGCTCTGGAGGTCCTCCCCTCTGCTTCCCCAACCTCAACTCCTCCTGCAGGCGCCTGCTGCGACCCACCTCCCAGACCGTTGTTCTCTACACTCTGCTGGC
CTCCATCTCCCTGCTCACTGTGGTGCTCAACCTGCTTGTCGTCATCTCCATCTCCCACTTCAGACAGCTTAACACCTCGACCAACACCCTGCTCCTTTCCCTGGCTGT
GTCTGACCTGCTGGTGGGGTTGCTGGTGATGCCCATCGAGGGCCTGTACTACATCGAGACGTGCTGGCTGCTGGGGAGGCTGATGTGTGCTCTCAGTCCTTATTTGT
CTTACTGCCTTATCTCTGTCTCTCTGGGCAGCATGGTGCTCATATCTGTAGATCGCTATATAGCCATCTGTGACCCTCTGCTCTATTCCTCAAAGATCACAGTGAACAGA
GTGAAGCTTTCAGTCTGTGTCTGCTGGGCCTGCTCTCTTCTCTACAATGGCTGCATTCTCATGGAGCACATAGGGTGGCCAGACAGGTTCGGCTCCTGTCACGGGGA
GTGTGTGGTGTTCATCAGCCGCGCTTTGGGTACAATAGATCTCTTTTTCTCATTTCTCGGCCCATGTGCCTTAATGTTTGTTCTGTACATGAGGGTCTTTGTGGTTGCTG
TTTCTCAGGTACGTATCATTCGGTCGCAGGCAGCTGTGAGAGCAGCTCCAGCTGCTAAAAAATCAGAGCTGAAGGCAGCCAGGACACTCGGGATTTTGATAGCTGTGT
TTGTAATGTGCTTCTGCCCTTATTATTATCCTTCCTTTGCAGGTGACGACACCTCTATGAGCTTACCTTATTACGCTCTATTCTCGTGGATAATGCTGACAAATTCTTGTGT
GAACCCTGTGATTTATGCTCTGTTCTACCCCTGGTTTAAAAGAGCTATCAGACTCATTGTCACCCTCAGAATACTGCAGCCTCACTCCAGAGATGTCAAGATCCTGtag
>contig052987-NyeTAR.B030
ATGGAGTTATTCAATGTGACTGTTAATACTGTGAGCTTCCTTCTCTGCGATTCACAAAAGAACAAATTATGTGTGTTATTATATGTTGTCCTCAGCTCATTTATGCTTCTTAC
AATATGTGGAAATCTTCTTGTGATAATTTCTATAATTTACTTTAGATATCTCCACACTCCTACAAACTACCTTATCCTCTCTATGGCTGTGGCTGATCTACTAATTGGTGCTTT
AATATTTCCTTTGAGCATGACAGTGTCTCTAAAGCCATGTTTGTATATATACAGTTTACTGTGCAACTTAAGAAGCACAATGGATGTAACAATGGGTGTATCTTCCTTATTAA
ATTTGTGCTGTATTTCTGTTGATCGATATTATGCTGTTTGCCACCCTCTGATATATAAAACTAAAATAACTGATTGTGTTGCCATGAAGATGGGCCTTGGAAGTTGGGCTGT
TGCTATCTTGTGTGGAATTTTTGTCTTCCTGCTGTTTTTTATTTTAGACGAATGTGACACAAGTTGCGTTTTCGCTCTCATTGCCGCATCAGTGGTAGTATATTATATCCCA
ACAATAGTTTTACTTTTCATGTACACCAAAATTCTTGTTGTTGCACTGAGGCAGGCACGCAGCATCCATAATACAATTTCTCAGAACACAAAGTCTAAAGCAGTTTCCAGT
ACGGAGAGAAAGGCCACAAAGACATTAACCATAGTTATTGGAATATTTTTAATTTTTTGGGTTCCTCTTTTTCTTAGTTATTCATTTGTTCCTCTGGATAGTTTTATACTTTAT
GTGCTTCTTGAACCATTTAACTGGTTTGCAATTTCTAACTCAATGCTCAATCCCTTTATTTATGCTTTCTTTTACACTTGGTTTAGAAGAGCTTTTAAAATGATAATTTCTGG
AAAAATATTTCAAGGTGATGTTACTAACATAAAACTACACtga
>contig057511-NyeTARe.A001
ATGAAAACCTTTAAAGAAGCTGAACTCTGCTTTCCACAACTCCTCAACTCCTCCTGCAGGAAGACCATGCGTCCTTATACATTTTTCAGGAAGCTGCACACCCCCACCA
ATCTCCTCCTCCTATCTTTGGCTGTCTCTGATTGCCTGGTGGGTCTCCTGATTTTATTTCAAATTATGATTATAGATGGTTGCTGGTTTCTTGGTGAATTCATGTGTAGTAT
GTATTTTCTCTTG
>contig063576-NyeTARf.A002
ATGGAGATACAGAAAGGAGTTGAGCTCTGTTTTCCACAACTCCTCAACACTTCCTGCAGGAAGCCGGCACCTCACTGGTCCAAAGCTGTGCTCCTGAACATTGTGCTG
TCATGTGTCTCTCTGATCACTGCTGCTCTAAACCTTCTGGTCATCATCTCAGTCTCTCACTTCAGGGCTGTCTCAGACTTTCTTGTGGGTCTCTTGCTGATGCCATTTGA
AATCTTTAGGAACATGGCCTGCTGGGTACTTGGTGATCCTATGTGTTCTGTTTATTGGTATCTGACTGGCAGTGTTGGG
>contig066211-NyeTARe.A006
ATGGAGATACAGAAAGTGCCCGCGCTTTGTTTTCCACAATTTCTCAACAATTCCTGCAGAAAGCCGACACTTCATTGGTCGAAAGCCGTGCTGCTGAACATTGTGTTCT
CCTGTATCTCTCTGCTCACTGCTGCTCTAAACCTTCTCGTCATCATTTCAGTCTCCCACTTCAGGAGGAAACTTCACTCACCAAGTAACATCGTCCTCCTCTCTCTGGCT
GTCTCAGACTTTCTTGTGGGTCTTCTGTTGATGCCAGCAGAAATATTAAGAAGCACGACTTGCTGGGTACTTGGTGACATTATGTGTTCTGTTTATTGGTATCTGACCGG
CAACATTATCTGTGCTTCAATAGGGAACATAGTTCTAATATCAGTTGATCGTTATGTGGCTATTTGTGACCCCTTGCATTACCGCACCAGAATTACTGTGAAACGAACCAA
ACTCAATGTGTGCTTATGTTGGTTTTATGCTATTTTCTACTGGAGTCTTTATATAAAGAATATCCTGATTGAA
>contig046002-NyeTARe.A007
CTCTCTCTTGCTGTCTCAGACTTTCTCATGGGTCTCTTGCTGATGCCAGCAGAAATCCTTAGAAGCATGACGTGCTGGGTACTTGGTGATCTTATGTGTTCTGTTAATTT
TTTTCTGACTGTAAACATTACCTGTGCTTCAATAGGGAACATAGTTCTTATATCGATTGACCGTTATGTGGCTATTTGTGACCCTCTACATTACTCCACAAGAATTACTGTG
GCAAGAGTCAAACTCAGTGTTTGTCTGTGTTGGTTTTATTCAACTTTCTACTGCAGTCTTTATACACAAGAAATGCTGATTGAACCAGGCAGGTATAATTCCTGTTATGGT
GAGTGTGTGCTTGTCATCAGTGATTTTGCTGGAATGGTTGACCTTATTTTATTTTTTATTCTTCCAGTTTCTGTCATCATAGTTCTGTATACAAGAGTATTTGTGGTGGCTG
TGTCTCAGGCTCGTGCCATGCGCTCTCATGTTACGGCTGTCACACTTCAGCGTTCATTGAATCAAACAAACAAATCTGAGCTGAAAGCAGCCAGGACTCTGGGGGTTC
TTGTAGTTGTGTTTCTGTCATGCTACTGTCCATTTTATTGCTACACTCTCACTGATAAAAATGCTGTCAATGATCCAGCTGCATCTTTTGTGATCTTTATCTTTTATTTTAACT
CTTGTATAAACCCTTTGATGTATGCCCTGTTTTACCCCTGGTTTAGAAATGCTGTTAAACTTATTATCACATGGCAGATATTCAAGCATAACTCCTGTGAGGCCAACATACT
Ataa
>contig046003-NyeTARe.A008
GTGTGCTTATGTTGGTTTTATGCTATTTTCTACTGGAGTCTTTATATAAAGAATATCCTGATTGAACCGGGCAGGTATAATTCCTGCTACGGAGAGTGTATGTTTGTCAGCA
GTGATATTGCAGGGATTATTGACCTTTTTTTATCTTTTATTGTTCCACTTTCTGTCATCATAGTTCTTTATATGAGAGTATTTGTGGCGGCTGTGTCTCAAGCTCGTGCCAT
GCGCTCTCATGTTACATCTGTCACACTTCAGCATTCAAGCAATCAAGCAAAGAAATCTGAACTGAAAGCAGCCAGGACTCTGGGGGTTCTTGTAGTTGTGTTTCTGTTT
TGTTACTCTCCATTTTACTGCTATTATCTTGCTGAAGAAAATGTGGTCAATGATCCATCTGCATCTACTGTTATCATTATTTTTTACATTAACTCTTGTCTAAACCCTTTGATC
TATGCCTTGTTTTACCCCTGGTTTAGAAATGCTGTTAAACTTATTGTCACTCTGCAGGTCTTCAAGCATAACTCCTGTGAGGCCAACATACTAtaa
>contig051547-NyeTARe.A009
ATGGAGGAAACTGAACTCTGCTTTCAACAACTCTTTAACACCTCCTGCATGAGGCCCAGGCGTCCACACTTTGAGATCATGCTGACTTATATTTTGCTGTCCTTTATTTC
TCTGCTTACTGTGATTCTTAACCTGTTGGTCATCATCTCCCTCTCCCTATTCAGGCAGCTCCACACCCCCACCAACCTCCTGCTCCTTTCTCTGGCTGTCGCTGATTTCT
ATGTGGGCCTCCTCTTGTTCTTCCAAATTGTGCTCATAGATGGCTGCTGGTTCCTCGGTGACATCATGTGCACTCTGTATCAATACATAGCATATGTCATCACCTCGGCC
TCCATAGGAACCATGGTGATCATATCTGTTGATCGATATTTGGCTATTTGTTACCCTCTGCATTACTCCACCAAGATCACAAAACAAAGAGTTAAAATTGTTGTCTGTTTGT
GTTGGATCTGTTCTGTCATCTTTCAAAGTCTGATTCTGATGGATAACCTGGAGCAACCAGGCAGGTATAACTCTTGCATTGGAGAGTGTGTCTTTGTCATTAATTACATCG
CAGGACTTGTTGATGTTACTTTTTCCTTTATTGTTCCCTTTACTGTGATTGTAGTTTTGTATCTGAGAGTGTTTGTGGTGGCTGTGTCTCAGGCTCGTGCCATGAGGTCT
CAGCTTGCAGTCACTCACCAGCGATCAGTAACAGTAACTGCAAAGAAATCGGAGCTGAAAGCAGCCTGGACTCTTGGTATTGTTGTTGTTGTGTTTCTCATATGTATGT
GTCCATATTACTGTGTGGCTCTCACAGGCCAAGACAGCTTGCCAAGTGCTTCATCACTGACATTTGTTCTATGTTTGg
>contig063978-NyeTARe.A010
CTTGCAGTCACTCACCAGCGATCAGTAACAGTAACTGCAAAGAAATCGGAGCTGAAAGCAGCCTGGACTCTTGGTATTGTTGTTGTTGTGTTTCTCATATGTATGTGTC
CATATTACTGTGTGGCTCTCACAGGCCAAGACAGCTTGCCAAGTGCTTCATCACTGACATTTGTTCTATGTTTGGCCTACTTTAACTCATGTCTGAACCCCATTATATATG
TCTTTTTCTACCCCTGGTTCAGAAAATCTATAAAAGTCATTGTTACTCTTCAGATACTGCAGCCTGACTCTTCTGAAGTCACCATGCGTtag
>contig042497-NyeTARf.A011
ATGGAGAACGTGTATGGACAACAATTCTGCTTTCCACAGCTCCTCAACACCTCCTGTAGGAAGCCGCTGCAACATCACAATCAGATTAATATTATGTTACGTCTTTCTTTC
TGTTTTTTTCTTCTCAGGCAGCTTCACAACCAAACCAACCTCTTCCTCCTCTCTCTCGCTGTCTCAGACCTCCTTGTTGGCCTCCTGCTGATGCCGCCTCGAATCCTCC
TTTTAGGGGGCTGCTGGTTTTTGGGCACCTTCATGTGTGGACTGTTTTACTATGCCTCTTTTGTTCTCACATCTGCCTCAGTAGGAAACATGGTGCTTATTTCATTTGAC
CGGTATGTAGCTATTTGTGACCCCTTGTCCTACCCCACCACAGTTACTGAGAGAAAAGTCCAAATCAGTGTCTGTCTGTGTTGGGCCTGTTCACTGCTGTATAACGGCA
CAATACTAAACAACTTCCTGAAGCAGCCAGACAGATATAATTCTTGTGACGGGGAATGCATAGTTGTAATTAATTTCATAACTGGTGCTTTTGATGTCGTAGCTACCTTCAT
CGGCCCCACTGCTGTGATCATATTTCTGTACATGAGAGTATTTCTGGTGGCTGTGTCTCAGGCTCAGGCTATGAGATCTCATGTTGCCTTTGTTACTTCAAAGGGTTCAG
TTCACGTTGCCATTAAGAAGTCTGAGAGAAAAGCAGCTACAACTATTGGTGTTGTTGTGGTTGTGTTTCTGATGTGTTTCTGTCCTTACTTTTATCCCTCTCTCGCAGGG
CAGGACACATCAACCAGTGTGGAATTTTCAGTTTTTGGGGTCTGGTTACTTTATTGTAATTCCTGTCTAAATCCACTTATCTATGCGTTTTTCTATCCCTGGTTTAGGAAGA
CTGTTAAACTGATAGTTACATTGCAGATTTTGCAGCCTGACTCTTGTGATGCCAACATACTG
>contig064861-NyeTARe.A003
AGGCAGCTCCACACACCCAGTAACATCCTCCTCCTCTCTCTGGCTGTGTCAGACTTTCTTGTTGGTCTCCTGTTAATTCCAGCTGAGATTTTTAGAATCACAGTCTGCT
GGGTATTTGGTGAATCCATGTGTTCACTTTATACATATCTAGGCTACATTGTTGTAACATCTTCAATAAGTAACATAGTTCTCATATCAGTTGATCGCTATGTGGCAATTTGT
GATCCTCTGCATTACCCCAATAGAATTTCTGTGGCAAAAATCAGACTTAGTGTTTGTATGTGTTGGTTTTATTCGGCTTTCTACAGCACTCTTTGTACAAAGAATATCCTGA
TTGAACCAGGCAGGTATAATTCCTGTTATGGAGAGTGTGTGTTTATCACCAGTGATATTGCTGGCATAATTGACCTTGTTTTATCTTTTATCGTACCAGTTTCCATCATCATA
GTTCTATATATGAGAGTATTTGTGGTGGCTGTGTCTCAGGCTCGTGCCATGCACTCTCATGTTACAGCTACACTTCAGCGTTCACTGAATCAAACAAACAAATCTGAGTT
GAAAGCAGCCAGGACCCTGGGGGTTCTTGTAGTTGTGTTTCTGGCCAGTTTCTGTCCATTTTATTGTTACTTTCTTGTTGTAGAAGACATAGTCAGTGATTCATCTGCCT
ATATTGTGGTCATCATCTATTATTTTAACTCTTGTCTAAACCCACTAATTTATGCCCTGTTTTACCCTTGGTTTAGAAATGCTGTTAAAGTCATCATCACTTTTCAGATATTCA
AGCGTGACAGCAGTGAGGCCAACGTAATATAG
>contig058003-NyeTARp.A012
ATGATGGAGGAATCTGAACTCTGCTTTCCAAAACTCCTTAATATCTCCTGCAGCAGACCAAAGCGTCCTCACTTTGAGATCATGCTGACTTACATTCTGCTCTCCTTCAT
TTCTTTGCTTACTGTGATTCTTAACCTGCTGGTCATTATCTCCATCTCACACTTCAGGCAGCTCCACACCCCCACCAACTTCCTCCTTCTCTCTCTGGCTGTCGCTGATT
TCTTTGTGGGTCTCCTTATGTTCTTTCAAATAGTGCTCATCGATGGATGCTGGTTCCTCGGTGACATCATGTGCACTCTGTATCAGTATCTAGCATTCATTATTACTTCAGC
CTCAGTAGGAACCATGGTGATCATATCTGCTGATAGGTATTTGGCTATTTGTTACCCTCTGCATTACTCCACCCAAATCACACAACAAAGAGTTAATATATGTATAAGTTTG
TGTTGGTTTTTTTCTGTGATCTTTCAGAGTTTGATTGTGAAGGATAACTTGAAACAACCAGGAAAGTATAACTCCTGCATTGGAGAGTGTGTCTTTGTCGTTAACTACATT
GCTGGGCTTTTTGATCTTTTGTTTTCCTTCATTGTTCCCATTACTGTGATTGTAGTTCTGTATCTGAGAGTGTTTGTGGTGGCTGTGTCTCAGGCTCGTGCCATGAGGTG
TCAACTTGCAGTCACTCACCAGCGATCAGTAACAGTAACTGTTATGAAAGCGGAGCTGAAAGCAGCCCGTACTCTTGGTGTTGTTGTAGTTGTGTTTCTTATATGTATGT
GCCCATATTACTGTGTTGCTCTCACAGGCCAAGACAACTTCCTAAATGCTTCATCTGCTGCCTTTGTAATTTGTTTGGTGTACTTTAACTCTTGCCTAAACCCTATCATTTA

TGTCTTTTTTTATCCCTGGTTCAGAAAGTCAATCAAACTTATTGCTACTCTTTAAATACTGCAGCCTGACTCCCGTGAGACTAACATGCATTAA
>contig038663-NyeTARp.A031
ATGGACGGCACTGGAGGTCCTCCCCTCTGCTTCCCTAACCTCAACTCCTCCTGCAGGCGGCTGCTGCGACCCACCTCCCAGGCCACTCTTCTCTACACTCTACTGGC
TTCAGTCTCACTGCTTACTGTGGTGCTCAACCTGCTTGTGGTCGTCTCCATCTCCCACTTCAAGCAGCTCCACACCCCGACCAACGCCCTGCTCCTGTCCCTGGCCAT
GTCCGACCTGCTTGTGGGGTTGCTGGTGATGCCCATCGAGGGCCTGCGCTACATCGAGACGTGCTGGCTGCGGGGAAGGCTGATGTGTGCTCTCAGTCCTTATTTG
TCTTACTGCCTTATCTCTGTCTCTCTGGGCAGCATGGTGCTCATATCTGTAGATCGCTATATAGCCATCTGTGACCCTCTGCTCTATTCCTCAAAGATCACAGTGAACAGA
GTGAAGCTTTCAGTCTGTGTCTGCTGGGCCTGCTCTCTTCTCTACAATGGCTGCATTCTCATGGAGCACATAGGGTGGCCAGACAGGTTCAGCTCCTGTCACGGGGA
GTGTGTTGTGTTCATCAGCCGAACTTCAGGAACAGTAGATTTCTTTTTGTCATTTCTTGGCCCCTGTGGCGTAATGTTTGTTCTGTACATGAGGGTGTTTGTGGTTGCTG
TTTCTCAGGTACGTGCCATTCAGTCGCAGGCAGCTGTGAGAGCAGCTCCAGCTGCTAAAAAATCAGAGCTGAAGGCAGCCAGGACACTCGGGATTTTGATAGCTGTG
TTTGTAATGTGCTTCTGCCCTTATTATTATCCTTCCCTTACAGGTGTGGACACCTTTCTGAGCTTGCCTTCGTACGCCATGGTGTGTTGGATCATGCTAATAAATGCTTGT
GTGAACCCTGTGATTTATGTTCTGTTTTACCCCTGGTTTAGAAGAGCTATCAGATTCATTGTCAGCCTCAGAATACTGCAGCCTCACTCCAGAGACGTCAAAATCCTGTA
G
>contig035375-NyeTARs.A013
ATGGATACCCAGAGCAGAGCAGATCTCTGCTTTCCACAACTCTTCAACACCTCCTGCAAGAAGCCTACAACTCCTCTATCACAAGTTTTGCTCTTTCACATTCTGATGCC
ATCAATGTGTCTGCTAACTGTGACTCTCAACCTGCTCGTCATCATCGCAATCTCCCACTTCAGGCAGCTCCACTCACACACTAACATCCTGCTCCTCTCTCTGGCTGCC
GCTGACTTTCTCATTGGCCTTCTGTTTATGCCTGGAGAAATCCTGCGAAATACAGTGTGTTGGTTTCTTGGTCAGCTCACATGTTCTCTGTATAATTATATATCCTTCGTTG
TTACCTCTGCCTCAGTGGGCATCATGGTGCTGATATCAGTCGACCGCTATGTGGCTATTTGTGACCCTCTGCATTACCCCACCAGAATCACAGAGAGAAGAGTGAAACT
CTGTGTTTGTCTGTGTTGGCTCTGCTCGGTTTTCTACTGCTATATGATTGTAAGAGATGATCTAAGTCAACCAGGAAAGCATAATTCTTGCTTTGGAGAATGTATAATTTTT
GTTGAATTCATTGCAGGATTTGTTGACCTTGTTTTATCCTTTATAATTCCAGTTACTGTGATTGTATTTCTGTATATGAGAGTGTTTGTGGTGGCTGTGTCTCAGGCCCGTG
CCATGCGCTCTCAGGTTACAGCTGTCACACTGCAGCTCTCAGTGACTCTAACAGCAAAGAAATCAGAGTTAAAAGCAGCCAGGACTCTGGGTGTTCTTGTTCTTGTGT
TTCTAATATGTTTCTGCCCATATTACATTGTTTCGCTTTTTGGAGACGAGTTTCTCAATAGTGCCTCTGCATCTATTGTATTCTATTTCTTTCTTTTAAACTCATGTCTAAACC
CTTTGATCTATGCTATGTTCTACCCCTGGTTTAGAAAAGCTGTGAAACTAATTATGACTCTACAAATACTGCAGCCTGGCTCCTGTGAAGTCAGCATACTGtag
>contig035376-NyeTARs.A014
ATGGATACTCAGGATGGAGCAGAGCTCTGCTTTCCACAACTCTTCAACATCTCCTGCAAGAAACCTACAACTCCTCGGTCTCAAGTTTTGCTCCTTTATATTGTTGTGTC
CTCAATGTCTTTGCTGACTGTGACTCTCAACCTGCTCGTCATCATTACAGTCTCCCACTTCAGGCAGCTCCACACACCCACTAACATCCTGCTCCTTTCTCTGGCTGTC
ACTGACTTTCTCGTTGGTCTTCTGTTTATGCCTGGAGAAATCCTGCGAAATACAGCGTGTTGGTTTCTTGGTCAGCTCACATGTTCATTGTATAATTATGTATCCTACATCA
TTGCCTCTGCCTCAGTGGGCAACATGGTGCTGATATCAGTCGACCGCTATGTGGCTATTTGTGACCCTCTGCATTACCCCACCAGAATCACAGAGAGAAGAGTGAAAC
TCTGTGTCTGTCTGTGTTGGCTCTGCTCTGTTTTCTACAGCTATGTGATTTTAATAGATGATCTAAGTCAACCAGGCAAGCATAAATCTTGCTATGGAAGATGTATAATTTT
CATTGAATTTATTGCAGGATTTGTTGACCTTGTTTTAGCCTTTATTATTCCACTTACTGTCATCATAGTTCTGTATATGAGAGTGTTTGTGGTGGCTGTGTCTCAGGCCCGT
GCCATGCGCTCTCAGGTTACAGCTGTCACACTGCAGCTCTCAGTGACTCTAACAGCAACTAAATCAGAGTTAAAAGCAGCCAGGACTCTGGGTGTTCTTGTTCTTGTG
TTTCTATTATGTTTCTGCTCATATTACATTATTTCACTTTTTGGAAACGAGTTGCTCAATAGCTCATCTGCATCGATTGTGATCTATCTGTACTATTTTAACTCCTGTCTAAAC
CCTTTGATTTATGCTATGTTCTACCCCTGGTTTAGAAAAGCAGTGAAACTAATTGTGACTCTACAGATACTGCAGCCTGGCTCCTGTGAGATCAGCATAATGtag
>contig035381-NyeTARs.A015
ATGGATACTCAGGATGGAGCAGAGCTCTGCTTTCCACAACTCTTCAACATCTCCTGCAAGAAACCTACAACTCCTCTGTCTCAAGTTTTGCTCCTTTATATTGTTGTGTC
CTCAATGTCTCTGCTGACTGTGACTCTCAACCTGCTCGTCATCATTGCAGTCTCCCACTTCAGGCAGCTCCACTCACCCACTAACATCCTGCTCCTTTCTCTGGCTGTC
GCTGACTTTCTCGTTGGCCTTCTGTTTATGCCTGGAGAAATCCTGCGAAATACAGCGTGTTGGTTTCTTGGTCAGCTCACATGTTCTCTGTATAATTATGTATCCTACATC
ATTACCTCTGCCTCAGTGGGCAACATGGTGCTGATATCAGTCGACCGCTATGTGGCTATTTGTGACCCTCTGCATTACCCCACCAGAATCACAGAGAGAAGAGTGAAAC
TTTGTGTTTGTCTGTGTTGGCTCTGCTCTGTTTTCTACTGTTATGTGATTTTAATAGATGATCTAAGTCAACCAGGCAAGCATAATTCTTGCTATGGAAAATGTATAATTATC
ATTGAATTCATTGCAGGATTTGTTGACCTTGTTTTATCCTTTATTATTCCACTTACTGTCATCATAGTTCTGTACATGAGAGTGTTTGTGGTGGCTGTGTCTCAGGCCCGT
GCCATGCGCTCTCAGGTTACAGCTGTCACACTGCAGCTCTCAGTGACTCTAACAGCAAAGAAATCAGAGTTAAAAGCAGCCAAAACTCTGGGTGTTCTTGTTCTTGTG
TTTCTATTATGTTTCTGCTCATATTTCATTGTTTCACTTTTTGGAAACGAGTTGCTCAATAGCTCATCTGCATCGATTGTGATCTATCTGTACTATTTTAACTCCTGTCTAAAC
CCTTTGATTTATGCTATGTTCTACCCCTGGTTTAGAAAAGCAGTGAAACTAATTGTGACCCTACAGATACTGCAGCCTGGCTCCTGTGAGGTCATCATACTGtag
>contig046011-NyeTARs.A016
ATGGAGATACAGGAAGGACTAGAGCTCTGTTTTCCACAACTCCTCAACGATTCCTGCAGAAAGCCAACACTTCACTATTCCACAACTATGCTGCTGTATATTGTGATGTC
ATGCTGCTCTATCACTGCTGCTCTAAACCTTCTGGCCATCATCTCAGTCTCCCACTTCAGAACTATCTTATCACTATCATTTCGCTGCCCCACCTTGGGCATAGATAAACC
TCAGCTGAAAATTTTAAAAAGCACCACCTGCTGGATACTTGATGATTTTAATTGTTTTCTTTATTGGTATCTGACTGGCAACATTACCTGTGCTTCAATAGGGAACATAGTT
CTAATATCAGTCGACCGCTATGTGGCTATTTGTGACCCTTTACATTACTCCACCAGAATTACTGTGGCAAAAGTTAAACTCAGTGTTTGTCTGTGTTGGTTTTATGCTATTT
CCAGCATTAGTCTTTATGCAAAAGACTACCTGATTGAACCAGGCAGGTATAATTCCTGTTATGGAGAGTGTGTCATTAATGATATTGCAAGGACTATTGACCTTGTTTTATC
CTTTATATTTCCAATTACTGCCATCATAGTTCTGTATATGAGAGTATTTGTGGTGGCTGTGTCTCAGGCTCATGCCATGCGCTCTCAGGTTACAGCTGTCACAATTCATTGT
TCTCTGAATCAAGCAAACAAATCAGAGTTGAAAGCAGCCAGGACACTTGGAGTTCTTGTAGTTGTGTTTCTACTGTGTTTCTGCCCATATTACTGTGTCTCTCTTATAAG
GGAGGACTTTAGCTCAGTAGCTCCTATTGTGATCAGTCTGTTCTTTTCAAACTCCTGTTTAAACCCAGTGATCTATGCCTTGTTCTATCCTAAAGCTTTGAAACTAATTGT
GACTTTACAGATACTGCATTTGTTGtag
>contig046007-NyeTARs.A017
ATGCAGAAAGAAAACAAGTTGTGTTTTCCACAACTCCTCAACAGCTCCTGCAGGAAGCCAACACTTCACTGGTCCAAAGATGTGCTCCTGAACACTGTGCTGTCCTGC
ATCTCTCTGCTCACCGCTGCTCTAAACATTCTCGTCATCATCTCAGTCTCTTACTTCAGGAAGCTTCACACACCCAGTAATATCCTTCTCCTTTCTCTGGCTGTCTCAGA
CTTTCTTGTCGGTCTCCTGTTGATGCCATTAGGAATCCTTAGAAACACAGGCTGCTGGGTACTTGGTGATATAATTTGTTCTCTTTATTGGTATTTAACCAGCAACATTGT
CTGTGCTTCAATAGGGAACATTGTCCTAATATCAGTTGACCGTTATGTGGCTATTTGTGACCCTCTGCATTATCCCAGCAGAATTACTTTGGCGAAAGTCAAACTCAGTGT
TTGTCTGTGTTGGTTTTATGCTTTTTTCTACGTCAGTCTTTATACAAAGGATATCCTACTTGAACCAGGCAGGGATAATTCCTGCTACGGAGAGTGTGTATTTGTCATCAAT
GATATTGCTGTTGTTGTTGACCTTGTTTTTTCCTTTATTGTTCCGGTTTCTGTCATTGTAGTTCTGTATATCAGAGTATTTGTGGCAGCTGTGTCTCAGGCTCGTGCCATG
CGCTCTCATGTGACATCTGTCACACTGCAGCGCTCACTGAATCAAACAAACAAATCTGAGCTGAAAGCAGCCAGGACTCTTGGAATTCTTGTAGTTGTGTTTCTGGCAT
GCTTCTGTCCACTCTACTACTTTTCTCTTGTTGATGAAAATGCAATCAATGATCCAGCTGCATCTTTTGTGGTCATTATATTTTACTTTAACTCGTGTCTAAATCCTTTGATC
TATGCCTTGTTTTACCCCTGGTTTAGAAATGCTGTTAAGCTTATCATCACTCTGCGGATTTTCAAGTATAACATGAGTGAGGCCAACATActataa
>contig060105-NyeTARs.A018
ATGGAAGAAACTGAACTCTGCTTTCCACAACTATTCAACTCCTCTTGTGTGCGGCAAAAGCATTCACAAATTGAGGCTGTGTTTATTTACACTCCACTCTCTTCCATCTC
TGTGCTTACTACAATTCTGAACCTGCTTGTGATCATCTCCATCGCTCACTTCAAGCAGCTGCACACCCCAACCAACCTCCTCCTCCTCCTCTCTCTGGCCGTCTCAGAT
TTCTTCGTGGGCCTCATCATGGCTTGTCAGATTAGCCTCCTAGATGGCTGCTGGTTTCTTGGTGACCACATGTGTGCTCTGTATAGCAGTTTAGATTACATTGTTACTTCT
GCTTCAGTAGGAACTATGGTACTCATATCAGCTGACCGCTATGTAGCCATTTGTGACCCTCTGCATTATCCCACCAAAATTACTATAAAAAGAGTCTCAGTCTCTATTTGTA
CTTGCTGGGCTTGTTCAATTCTGTATAACAGTCTTATCATGAAGGATAATTTCAAGCAGCCAGGGAGGTATAATTCTTGTTCTGGTGATTGTGTAGTTGTCATTGACTACT
TTGTGGGAATTTTTGACTTTGTTTTGACCTTTGTCGGCCCTGTGATTGTCATCATAGTTCTGTATCTAAGAGTATTTGTGGTGGCTGTGTCTCAGGCTCGGGCAATGCGC
TCTCATATCACTGCTCTCAGACTCCAGGGGTCAGAGACGGTGCATGCTAAGAAATCTGAGCTGAAAGCTGCCAGGACTCTCGGTGTACTTGTTATTGCCTTTCTGATAT
GTCTTTTCCCATTTTTCTGTTCCTCAATGGTAGGCCAGAATAGTTTCTTTGATATTAGATCTGTACCTTTTGAGCGCTTGCTGTTCTATTTTAACTCTTGTCTGAACCCATTA
ATCTATACTTTTTGCTACCCCTGGTTTCTGAAATCTATCAAGCTCATTGTGACATTTAAGATATTTAGGCATGGCTCCAGTGAGGCCAGTGTACTAtag
>contig046010-NyeTARs.A019
ATGCAGAAAGAAGCCGAGCTTTGTTTTCCACAACTCCTCAACAGCTCCTGCAGGAAGCCAACATTTCACTGGTCCAAAGCTGTGCTGCTGAACATTGTGCTGTGCTGC
ATCTCTCTGCTCACTGCTGCTCTAAACCTTCTCGTCATCATCTCAGTCTCTTACTTCAGGCAGCTTCACACACCCACTAACATCCTCCTCCTTTCTCTGGCTGTCTCAGA
CTTTCTCACTGGTCTCCTTATGCTGCCACTAGAAATAATTAGAAACACGGCCTGCTGGGTACTTGGTGATCTTATGTGTTCTGTTTATACTTATCTGACAGCCGGTCTTTT
CTGTGCTTCAACTTGGAACATAGTTCTCATTTCAGTCGACCGCTATGTAGCTATTTGTCACCCTCTTCATTACCCCACCAGAATTACCGTGGCAAGAGTCAAACTCTGTG
TTTGTTTGTGTTGGTTTTGTTCAACCTTCTACGGCAGTCTTTTAACAAAGGATGTTCTAATTAAGCCGGCCAGGTATAATTCCTGTTATGGAGAGTGTGTGTTTGTCATTA
ATGATATTGCAGGGTTTATTGATCTTGTGTTATCTTTTATTTTTCCAGTATCTGTTATCATAGTTCTTTATATGAGAGTGTTTGTGGTGGCTGTGTCTCAAGCTCGTTCCATG
CGCTCTCATGTTACAGCTGTCGCACTTCAGCGTTCAAGCAATCAAGCAAACAAATCTGAGCTGAAAGCAGCCAGGAACCTTGGAGTTCTTGTAGTTGTGTTTCTCGCAT
GTTACCTTCCGTTTTACTGTTACTCTCTTATTGAAGTAAATGTGATCAATGACTCATCTACATTTTTTCTGATCATTGTGTTTTACTTTAACTCGTGTCTAAACCCACTGATG
TATGCCCTTTTTTACTCTTGGTTTAGAAATGCTGTTAAACTTATTATCACTCTGCAGATTTTTAAGCATGACACTAGTGAAGCAAACATGttttaa
>contig057515-NyeTARsP.A020
ATGGAAGAAACTGAACTCTGCTTTCCACAACTCCTCAACTCCTCTTGTGTGCGGCAAAAGCGCCCACAAATTGAGGCTGTGTGTATTTACACCCTGCTATCATCTATCT
CTCTGCTTACTGCAGTACTGAACCTACTTGTCATCATCTCCATCGCTCACTTCAAGCAGCTGCACACCCCAACCAACCTCCTCCTCCTCTCTCTGGCCGTCTCAGATTT
CTTCGTGGGCCTCATCATGGCTTGACAGATTAGCCTCCTAGATGGCTGCTGGTTTCTTGGTGACCACATGTGTGCTCTGTATAGCAGTTTAGATTACATTGTTACTTCTG
CTTCAGTAGGAACTATGGTACTCATATCAGCTGACCGCTATGTAGCCATTTGTGACCCTCTGCATTATCCCACCAAAATTACTATAAAAAGAGTCTCAGTCTCTATTTGTAC
TTGCTGGGCTTGTTCAATTCTGTATAACAGTCTTATCATGAAGGATAATTTCAAGCAGCCAGGGAGGTATAATTCTTGTTCTGGTGATTGTGTAGTTGTCATTGACTACTTT
GTGGGAATTTTTGACTTTGTTTTGACCTTTGTCGGCCCTGTGATTGTCATCATAGTTCTGTATCTAAGAGTATTTGTGGTGGCTGTGTCTCAGGCTCGGGCAATGCGCTC
TCATATCACTGCTCTCAGACTCCAGGGGTCAGAGACGGTGCATGCTAAGAAATCTGAGCTGAAAGCTGCCAGGACTCTCGGTGTACTTGTTATTGCCTTTCTGATATGT
CTTTTCCCATTTTTCTGTTCCTCAATGGTAGGCCAGAATAGTTTCTTTGATATTAGATCTGTACCTTTTGAGCTCTTGCTGTTCTATTTTAACTCTTGTCTGAACCCATTAAT
CTACACTTTTTGCTACCCCTGGTTTCTGAAATCTATCAAGCTCATTGTGACATTTAAGATATTTAGGCATGGCTCCAGTGAGGCCAGTATACTATAG
>contig046013-NyeTARs.A021
ATGGAGATACCGAAAGGAGTTGAGCTTTGTTTCCCACAACTCCTCAACAGCTCCTGCAGGAAGCCGACACTTCACTGGTCCAAAGCTGTGCTCCTGAACACTGTGCT
GTGCTGCATCTCTCTGCTCACTGCTGCTCTAAACCTTCTCGTCATCATCTCAGTCTCCCACTTCAGGCAGCTGCACACACCCAGTAATGTCCTCCTCCTCTCTCTGGCC
GTCTCAGACTTTTTTGTGGGTCTCCTGTTGTTGCCTTTAGAAATTTTTAGAAACACAAGCTGCTGGGTACTTGGTGATCGTATGTGTTCTGCTTATTGGTATTTGACCAGC
AACATTGTCTGTGCTTCAATAGGGAACATTGTTCTAATATCAGTTGACCGCTATGTGGCTATTTGTGACCCTCTGCATTATCCCACCAGAATTACTTTGGCAAACGTCAAA
CTCAGTGTTTGTCTGTGTTGGTTTTATGCTTTTTTCTACAGCAATCTTTATACAAAGGATATCATGATTGAACCAGGCAGGTATAATTCTTGCTTTGGAGAGTGTGTATTTT
TTGGCAGCAATATTGCTATTGTTGCTGACCTTATTTTATTCTTTTTTGTTCCAGTAACTGTTATCATAGTCTTGTATATGAGAGTATTTGTGGTGGCTGTGTCTCAGGCTCGT
GCCATGCGCTCTCATGTTACATTGGTCACACTTCAGCGTTCACTGAATCAAACAAACAAATCTGAGCTGAAAGCAGCCAGGACTCTGGGGATTCTTGTAGTTGTGTTTC

TAGCATGCTTCTCTCCACTCTACTGCTACTCTCTTGTTGATGAAAATGCAATCAATGATCCAGCTGCATCTTTTGCAGTCATTATCTTTTACATTAACTCCTGTCTAAACCC
TTTGATCTATGCCTTGTTTTACCCCTGGTTTAGAAATGCTGTTAAACTTATCATCACGTTGGAGATCTTCAAGTATGACACAAGTGGTGCCAACATACTGtaa
>contig062039-NyeTARs.A022
ATGATGGAGAACAAGGACCGTATATGCTTTCCAGAAGTCTTCAACTCTTCCTGCAGGAGACCAGCATTTCACTGGTCTGAAGCTGTGCCCCTGAATACCCTGCTGTGC
TCCATCTCTGTAATCACCATAGCTCTGAACCTCCTTGTCATCATCTCAGTCTCCTACTTCAGGCAGCTCCACACACCCACTAACATCCTCCTCCTCTCTTTGGCTGTGTC
AGACTTTCTTGTGGGGCTCCTGTTGCTGCCTGGAGAAATCTTCCTAGGAATAGCCTGCTGGGCTTTCGGTAACCTTTTGTGTTCTCTCTTTAATTATGTGTCTTTCATCA
TTACCTCGGCTTCAGTGGGAAACATGGTTCTCATATCAGTTGACCGCTATGTGGCGATCTGTTATCCTCTGCATTACTCTACCAGAATTACTGTGACAAGAGTTAAACGT
TCTGTTTGTTTGTGTTGGCTCTGTTGTGTTTTGTACAGTAGTGTCCTTTTAAAAGATGAGCTGATTCAACCAGGAAGGCATAATTCCTGCTATGGAGAATGTGTTTTTGTC
ATCAACTTTATTGCAGGAACTGTTGATCTTTTGTTAACCTTTATTGTTCCTATTTCTGTTATTGTAGTTCTGTATATAAGAGTATTTGCAGTGGCTGTGTCTCAGGCCCGTG
CCATGCGCTCTCAGCTTTCAGTGAATCTGACAACAAAGAAATCTGAGTTGAAAGCAGGAAGGACTCTTGGTGTCCTAATAGTTGTGTTTCTAATGTGTTTCTGTCCATAC
TATTGCGTCTCTCTTGCAGGGGAAGACTCACTCAGTGAGTCATCCACATCAATTGTCCGTTATCTCTTCTATTTTAACTCCTGTTTGAACCCTCTGATCTATGCACTATTTT
ACCCCTGGTTTAGAAAAGCTATTAAACACATTGTCATGCTTCAGATATTTCACACTGGCTCCCATGAGGACAACATACTAtag
>contig035377-NyeTARs.A023
ATGGATACTCAGGATGGAGCAGAGCTCTGCTTTCCACAACTCTTCAACATCTCCTGCAAGAAGCCTACAACTCCTCTGTCTCAATTGTTGCTTCCTTATATTGTTGTGTC
CTCAATGTCTCTGCTGACTGTGACTCTCAACCTGCTCGTCATCATCGCAGTCTCCCACTTCAGACAGCTCAACACACCCACTAACATCCTCCTCCTCTCTCTGGCTGTG
TCAGACTTTTTCATTGGTCTGTTGTTGATGCCTGCAAATATCATCCGAAAGAAGGTTTGTTGGTTTCTTGGTCAGCTTGCATGTTTAGTGTTTACTTATGTATCCTTCATTA
TTACCTCTGCCTCAGTGGGCATCATGGTGCTGATATCAATCGACAGGTATGCGGCTATTTGTGACCCTCTGCATTACCCCACCAGAATAACTCGTGAAAGAGTGAAACT
CTGTGTCTGTCTGTGTTGGCTCTGCTCGGTTTTTTACAACATACTATTTATAAAGAACGACCTGCTTCAACGAGAACGACATACTTCCTGTTATGGAGAGTGTAAAATTCT
CGTAGACTACATTGCAGGAACCACTGATATTGTTTTAACCTTTATTGCTCCAGTTACTGTCATCATAGTTCTGTATATGAGAGTATTTGTGGTGGCTGTGTCTCAGGCCCG
TGCCATGCGCTCTCAGGTTACAGCTGTCACACTACAGCTCTCAGTGACTCTAACAACAAAGAAATCAGAGTTAAAAGCAGCCAGGACACTGGGTGTTCTTGTTCTTGT
GTTTCTAATATGTTTCTGCCCTCATTATTGTATTTTTGTTTTTGGAGGAAAGGTGCTTAATAGCTCATCTGCAACTATTGTGATCTATCTGTATTATTTTAACTCCTGTCTAAA
TCCCTTGATTTATGCTATGTTCTACCCCTGGTTTAGAAAAGCTGTGAAACTAATTGTGACACTACAGATACTGCAGCCTGGCTCCTGTGAAGTCAGCATACTGtag
>contig046014-NyeTARs.A024
ATGGAGATTCAGAAAGCAGCTGAGCTTTGTTTTCCACAACTCCTCAACAGTTCCTGCAGGAAGCCGACACTTCACTGGTCCAAAGCTGTGCTCCTGAACATTGTGCTG
TCCTGCATCTCTCTGATCACTGCTGCTCTAAACCTCCTGGTCATCATCTCAGTCTCCCACTTCAGGCAGCTGCACACACCCAGTAACATCCTGCTCGTCTCTCTGGCTG
TCTCAGACTTTCTTGTGGGTCTTCTGCTGATGCCATTAGAGATCTTTAGAAACACAGCTTGCTGGGTACTTGGTGATGTAATGTGTTCTGTTTATTGGTACCTGACCAGC
AACATTACCTGTGCTTCAATAGGGAACATAGTTCTAATATCAGTTGACCGTTATGTGGCGATTTGTGATCCTCTGCATTACCCCACTAGAATTACTTTGGTGAAAGTCAAA
CTCAGTGTTTGTCTGTGTTGGTTTTTTTCTATTTTCTACTGGAGTCTTTATATGAAAGATATCCTAGTTGAACCAGGCAGGTATAATTCCTGCTATGGAGAGTGTGTGCTTA
TTATTAATGACATTGCAGGGATTGTTGACCTTGCTTTGTCCTTTATAGTTCCAGTTACTGTTATCATAGTTTTGTATATGAGAGTATTTGTGGTGGCTGTGTCTCAGGCTCG
TGCCATGCGCTCTCATGTTACATTTGTCACACTTCAGTGTTCATTGAATCAAGCAAACAAATCTGAGCTGAAAGCAGCCAGGACTCTGGGGGTTCTTGTAGTTGTATTTT
TGGCATGCTACTGCCCATTTTACTGCTACTCTCTGGTTGATAAAAATGTGGTCAATGATTCATCTGCATCTTTTGTGGTTCTTGTCTTTTATTTTAACTCTTGTTTAAACCCA
TTGATCTATGCTCTGTTTTACCCCTGGTTTAGAAAAGCTATTAGATGTGTTATCACTCTGCTGATATTTAAGCATGACAGCAGTGAGGTCAACGTAATAtag
>contig058002-NyeTARs.A025
ATGGAAATGTTTGAAGAAACTCAACTCTGCTTTCCACAGCTTCTTAACTTCTCCTGCAGGAAGCCCATACGACCTCACTCAGTATCTATACTCATTTATATTACATTGTCCT
CCATCTCTTTACTGACTGTGACTCTTAATCTGCTGGTCATCATCTCCATCTCACATTTCAAGAAGCTGCACACCCCCACCAATCTCCTCCTCCTATCTTTGGCTGTCTCT
GATTGCCTGGTGGGTCTCCTGATTTTATTTCAAATTATGATTATAGATGGTTGCTGGTTTCTTGGTGAATTCATGTGTAGTATGTATTTTCTCTTGGACTACATTATTACCTC
TGCCTCAGTAGGAACCATGGTTCTCATATCAATTGACCGTTATGTGGCTATTTGTTACCCTCTCCATTACTCTACCAAAGTCACCCCAAAAAGAACAAAAGCATGTGTTTA
TCTCTGTTGGATTTGTTCTTCAGTCGACCAATGTCTCCTGCTAAAAGACAACCTGGTGCAACCTGGCAGGTATAATTCCTGCTATGGGGAGTGTGTGGTTGTTGTTGGC
CATGCTTTTGGAGTTGCAGATCTATTGCTGTCCATTATTGGTCCTGTCACTGTGATTGTAGTTCTTTATCTAAACGTATTTGTTGTGGCTATGACTCAAGCTCGTGCCCTG
CGGTCACATATTGCAGCTCTGACACACGAGAGGTCAGTAAGTACAAATGTTAAGAAATCTGAAATGAAAGCAGTAAGGACCATCAGTGTTCTTATCATTGTGTTTCTAATA
TGTCTCTGCCCATATTACTGTGTCACACTCTCAGGTCAAGATGCCATGCTCAGTGCTTCATCTGTTGCATTTGTAATGTGTTTGTTCTATTTAAACTCCTGTCTAAACCCT
CTAATCTATGCTTTATTTTACCCCTGGTTCAGAAAATCTGTTAAACAAATTGTTACACTTAAAATATTGAAGTCTGGCTCCTGTGACACCAATATCATGtacacagaatga
>contig045999-NyeTARs.A026
ATGGAGATCCAGATGCATCCAGATGCAGAGCTCTGTTTTCCAGAGCTACTGAACAGTTCCTGCAGGAAGCCGACACTTCACTGGTCCAAAACGGTGCTCCTGAACGTT
GGGCTTTCGTCCATCTCTCTGATCACTGCTGCTCTTAATCTGCTCATCATCATCTCAGTCTCCCACTTCAGGCAGCTCCACACTCCCAGTAACATCATCATCCTCTCTCT
GGCTGTCTCTGACTTTTTTGTCTGTTTTTTGTTGATGCCGGTAGAAATCTTCAAAAACACGGCCTGCTGGGTATTTGGTGATCTCATGTGTTCACTTTATACTTATCTGAG
CTGCATTCTTATCAATGCTTCATTTGAAATGATTATTCTCGTATCAGTTGACCGCTATGTGGCTATTTGTGACCCTCTGCATTACCCCACCAGAATCACTGTGCCGAGAGT
CAAACTCAGTGTTTTTCTGTGCTGGCTTTATGCTATTTTGTACAACATCATCTATACAAAGCATGCCCTGATAAACCCAGGCAGGTATGGTTCCTGCTATGGAGAGTGTGT
TTTTGTTGTTGATGATATTATTGGAATTGTTGACTTTGTTGTATCTCTTATAGTTCCAGTTACGATCATCGTAGTTCTGTATACGAGAGTGTTCGTGGTGGTTGTGTCTCAG
GCTCGTGCCATGCGCTCTCATGTTACAGCTGTCACACTTCAGCGGCCACTGAATCAAGCAAACAAATCTGAACTGAAAGCAGCCAGGAACCTTGGGATTCTTGTAGTT
GTGTTTCTGGCGTGCTACTGTCCATTTTACTGCTACTTTTTTCTTGCAGGCAATGAAGTTAATGCTTCTTCTGCATCTTCTATCCTCATTGTTTATTATTTTAACTCTTGCCT
AAACCCATTAATGTATGCACTGTTTTACTCCTGGTTTAGAAATGCTGTTAAACTTATCATCACTCTGCAGATATTAAAGGCTAACAGCAGTGAGATCAACATACTAcagagatg
a
>contig060292-NyeTARs.A027
ATGGCAGAAACTGAACTCTGCTTTCCAAATCTCAACTCCTCCTGCAGTAGAGTAAAGCGTTCTCCCTCGGACTCAGTGCTTATTTATGTAATCCTAACCATCATTTCTCTT
CTTACTGTGGCTCTAAACCTGCTGGTCATCATCTCCATCTCCCACTTCAAGCAGCTCCACACTCCTACAAACCACCTCCTCCTCTCCCTGGCAGTCTCTGATTTCTTTG
TGGGTCTCAATATGTGCTTTCAAAGTATGCTCATAGATGGTTGTTGGTATCTTGGTGACCTCATGTGTGTCTTGTATTATGTTTTCGACATAGTTGCTACTTCTGCCTCAGT
AGGAACCATGGTGCTCATTTCAGTTGACCGTTATGTGGCCATTTGTGATCCTCTTCATTATCCCACCAAAGTCACTCCAAAAAGAGTTCAGACCTGTGTCTTAATGTGTT
GGATTTGCTCGCTCCTAGTAGTCGGTGTGCTCTTGAAGGATAACCTGGATAAACCAGGTAGATTTAATTCCTGCTTTGGAGAGTGTGTGATTTATGTTGACTTTGCAATA
CAAGTTACCGATCTTATTTTGACAATCCTCCTTCCCATTACTGTCATCGTAATTTTGTATGTAAGAGTATTTGCCGTGGCCGTGTTTCAGATTCGGGCCATGCAACCTCAT
GTTGCAGCTGTCACACAGAGGGGGAAAGTAAGTCCAAAAAAATCTGAACTGAAAGCAGCTAGGACTCTTGGCATTGTTATTGTTGCATTTCTGATATGCCTATTCCCATA
TTACAGTGTGATACTCTCAGGCCAGGACACACTGCTTGATATTTTATCCGTTACCTTTTTTTTATGTTTGTTTTATTTTAATTCCTGTCTCAACCCTATCATCTATGCCTTTTT
CTACCCATGGTTTAGAAAATCTGTTAAACTTATTGTTACATTTCAAATAGTCAAGTCTGGCTCCAGTGATGCCAGCATGCTGtga
>contig042499-NyeTARs.A028
ATGGAGGGGGTTGAATTCTGCTTTCCACATCTGCTCAACTCCTCTTGCAGAAAGGCTCTGCATCCTGTCTCAGTTTCCACGCTCATTTACATGATAATATCTTCTATCTCT
GTGCTCACTGCAACTCTTAACCTGCTGGTCATCATCTCCATCTCCCACTTCAAGCAGCTCCACAATCCCACCAACTTCCTCCTCCTCTCTCTGGCTGTCTCCGATTTCT
TTGTGGGTCTCTATTTGCTCTTTTACATAATGTTTATAGACGGCTGCTGGTATTTTGGTGACTTCATGTGTATTCTGTATTATGTTATTGCCACAATTAACACCTCTTCCTCA
ATAGGAACCATGGTACTGATATCAGTTGACCGTTATGTGGCCATTTGTGATCCTCTGCATTATCCCACCAAAGTCACTGCAAAAAGAGTTCAGATCTGTGTTTCACTGTG
TTGGAGCTTTTCTGCCCTTGCTGGTAGTTTCCTCTTAAAGGACAACTTGAAACAGCAAAGCAGGTTTAATTCTTGTGTGGGAGAGTGTGTTGTCCATATTAACTTTATTG
AATATGTTGCAGATTTTGCTTTGAACTTCCTACTTCCTATTACTGTCATTATAGTTCTGTATTTGAGAATATTTGTAGTGGTTGTGTCTCAGGTTCGGGCCATGCGGAGTCA
TACTGCAGGTGTCACATACCAGTGTTCAAGGAAGGGAAATCCAAAGAAATCAGAGATGAAAGCAGCCAGGACTCTTGGTATTGTTGTAATTGCATTCTTGTCATGCGCC
TTACCATTTTATTGTGTCACACTCACAGGCCAGAATGCCTTCCTCAACGGATCATCTTCTGCATTTGTTTTGTGTCTTTTCTATTTTAATTCGTGCCTCAACCCTATCATCTA
TGCCTTATTCTACCCCTGGTTTAGAAAATCTGTTAAGCTTATTGTTACATTTCAAATACTGAAGTCTGGCTCCAGGAATGCCAACATAGTCAAAGTGACAGAAtag
>contig056200-NyeTARs.A029
ATGGGCACACAGGAAAAATCTGAGCTCTGCTTTCCACAACTCTTCAACATGTCCTGCAAAAAGCCTGCCATTGCTCGGTCCAAAGCTGTGTTCCTTCATATTGTATTGT
CCTCAGTTTCTTTTCTGACGGTGGCTCTCAACTTGCTTGTCATCATCTCAGTCTCCCACTTCAGGCAGCTTCACACACCCACTAACATCCTGCTCCTCTCTCTGGCTGT
CTCAGACTTTCTTGTTGGCCTCCTATTGTCACCTGCAGAAATCCTCCGAAGTACAGCCTGTTGGTTTCTTGGTCAGCCCACATGTTTAATGTATATTTTTGTGTCCTTGAC
TGTTACCTCTGCCTCAGTGGGCATTATGGTGCTGATATCAGCTGACCGCTATGTGGCTATTTGTGACCCTTTGAATTACCCCATCAGGATCACTGACAGAAGAGTTCAAC
TCTGTGTTTGTCTGTGTTGGCTCTGCTCCATCGCCTTCAGCTGTTTCCTTGTAAGAGATGACCTGCATCAAAAAAGAAAACAAAATTCCTGCTATGGAAAATGTGTCGTT
GTTGTTCAATACATTGCAGGAGTTGTTGACCTCATTTTAACCTTCATTGTACCAGTTACTGTCATCATAGTTCTGTATATGAGAGTATTTGTGGTGGCTGTGTCTCAGGCC
CGCTCTATGCGCTCTCATGCTACAGCTGTCAGAAATCACCTTCCAATGACGCTAACAAAAAAGTCTGAACTAAAAGCAGCCAGGACTCTGGGTGTTCTTGTTCTTGTGT
TTCTAATGTGTTTCTGCCCTTATTACTGTGTTTCTCTTGTAGGAGAAGAATTCATCAATAGTTCATCTGCTTCTTTTGTGGCCTATCTGTTTGGTTTAAACTCATGTCTAAAC
CCTTTGATCTATGCTATGTTCTACCCCTGGTTTAGAAAAGCTGTGAAACTAGTTGTCACTCTACAGATACTGCAGCCTGGCTCCAGTGAGGTAAGTATTTTGtaa
>contig025313-BriTAR.A002
ATGGACAGCCAGGACAGAATTGAACTCTGCTACCCTGAGCTTGCGAATTTGTCTTGCAGGGGTTTAACACGTCCTCAGCCTGAGGCTGTCCTCCTCTATACACTGTTC
TTGTCCATCTCTGTGCTAACTGTAGCTCTGAATGTGCTGGTTATCATATCCATCTCTCATTTTAGGCAGCTCCACATGCCCACCAATGTCCTCCTGCTCTCTCTGGCCAT
CTCAGACCTCTTGGTCGGGCTGCTTGTGATGCCGGTGGAAACAATACGGTTTATAGAAACCTGCTGGCTGCTGGGAGATCTCATGTGTGCTTTTTCTTACATTATCGGC
TTCACTCTCACCTCAGCCTCTGTGGGCAACATGGTGCTCATATCAATTGATCGCTACGTAGCTATTTGTTATCCTCTGCAGTACCCCACTAAAATCACTCACAGCAGAGC
GGAGCTGTCTGTGACTCTGTGCTGGGCCTGTTCGCTCCTCTACAACGGGCTGATCTTAAAGGAGCATCTCAGGCAGCCAGACAGGCATAACACCTGTCACGGTCAGT
GTTTGGTGGTGATTAACTATGTCTCTGGAGCCATCGACCTTGTCTTTACTTTCATCGGCCCTTGCTCAGTCATTATCATTCTGTACATGAGAGTATTTGTTGTAGCGGTGT
CTCAGGCTCATGCCATGCGGTCTCATATTACAGCTGCTGCAGCTGGTAGAGTAAAAATCACCGCAAAGAAATCAGAGAAAAAGGCAGCCAGGACTCTTGGCGTTGTGA
TATTTGTGTTTTTGATGAGTTTCTGTCCGTATTACTATCCCTCTCTTGCAGGGCAGGACATTTCAAACAGTGCTTCATCTTGGGCTATTGTTTCCTGGATGCTGTACTTCA
ATTCTTGTCTGAATCCACTGATATATGCTTTTTTCTACCCGTGGTTTAGAAAAGCTATCTGGTTCATTGTCTCCCTGAAGATACTAGAGAAGGGCTCCTCTCAGGCAAATA
TACTTTAA
>contig029633-BriTAR.A003
ATGGACGGCACTGGGGGTCCTCCCCTCTGCTTCCCTAACCTCAACTCCTCCTGCAGGCGGCTGCTGCGACCCACCTCCCAGGCCGCTCTTCTCTACACTCTGCTGG
CTTCAGTCTCACTGCTCACTGTGGTGCTCAAACTGCTTGTGGTCGTCTCCATCTCCCACTTCAAGCAGCTCCACACCCCGACCAACGCCCTGCTCCTGTCCCTGGCC
ATGTCCGACCTGCTGTTGGGGTTTCTGGTGATGCCCATCGAGGGCCTGTGCTACATCGAGACGTGCTGGCTGCTGGGGAGGCTGATGTGTGCTCTCAGTCCTTATTT
GTCTTACTGCCTTCTCTCTTTTTCTCTGGGCAGCATGGTGCTCATATCTGTAGATCGCTATATAGCCATCTGTGACCCTCTGCTCTATTCCTCAAAGATCACAGTGAACAG
AGTGAAGCTTTCAGTCTGTGTCTGCTGGGCCTGCTCTCTTCTCTACAATGGCTGCATTCTCATGGGGCACATAGGGTGGCCAGACAGGTTCAGCTCCTGTCACGGGG

AGTGTGTGGTGTTCATCAGCCGAACTTCAGGAACAGTAGATTTCTTTTTGTCATTTCTTGGCCCATGTGCCGTAATGTTTGTTATGTACATGAGGGTGTTTGTGGTTGCT
GTTTCTCAGGTGCGTGCCATTCAGTCGCAGGCAGCTGTGAGAGCAGTTCCAGCTGCTAAAAAATCAGAGTTGAAGGCAGCCAGGACACTCGGGATTTTGATAGCTGT
GTTTGTAATGTGCTTCTGCCCTTATTATTATCCTTCCCTTGCAGGTGTGGACACCTTTCTGAGCTTGCCTTCTTACGCCATGGTGTGTTGGATCATGCTAATAAATGCTTG
TGTGAACCCTGTGATTTATGTTCTGTTTTACCCCTGGTTTAGAAGAGCATTAAGATTTATTGTCACCCTCAGAATACTGCAGCCTCACTCCAGAGATGTCAAAATCCTGtag
>contig035253-BriTAR.B025
ATGACCAACATTGTAAATGATCTACATCCCTGTTATGTAATACAAGACTCCAAATACATGCTGACAAACAGCCCTTCCATTATATGTGTATTCTTGTATGCATTCCTTGCATT
ATTGTCTGTTATAACAATATGTGGAAATCTCCTTGTAATAATTTCTGTGATTTACTTCAAACAGCTCCATACTCCTACTAATTATCTCATCCTCTCTCTCTTGCTGTGGCTGACC
TGCTCGTTGGGATTATAGCATTTCCTCTCAGCATGGCATTCTCTCTCAGCTCATGTCTTTATCATGAGGGTTTGTTTTGCAAAGTGCGAGGGACCTTTGATATTTCACTGA
GCACATGTTCCATCCTGAACCTCTGCTGTATTTCTATTGACAGATACCATGCTGTTTGCCAGCCTCTGACATATCAAACTAAAATCAGCCCTCGTGTTGTTGTGTTCATGA
TCCTGATGAGCTGGGGGGTTTCTGGGATAATTGGAATTAGTGTCACCATTGCTGGATTTAATAATGAAAAATGTGAGGAATCATGTTTGATTGATGTTCTAATAGAAAGCA
CTGTTGGACCCATGTTATCATTTTACCTGCCAGTGACCATGATGCTTTTTATCTACCTAAAGATTTTCTTTGTTGCTTTGAGGCAAGCAAGACGCATCCAGAACACAACAA
AGTGTGGACAAACTGCCAGTATCATGGAGAGAAAGGCGACCAAAACTCTGGCTATTGTTCTGGGAGTTTTTATATTTTGCTGGAGTCCTTTTTTTCTTTCCATCACATTC
CCACCATTTACCAGTGATTCTGTACCAGTTCCTGTAATTGAAACTCTTAACTGGCTTACATTGGCAAATTCAATGCTAAATCCATTTATTTATGCTTTCTTTTATAGCTGGTT
CAGGTCAGCCATCAGAATGATTATTTGTGGGAAAATATTCCAAGGGGATTTTGCCAACACAAATATGACTtga
>contig011696-BriTARe.B026
CACCTTCTCTGCGATTCACAAAAGAACAAATTATGTGTGTTATTATATGTTGTCCTCAGCTCATTTATGCTTATTACAATATGTGGAAATCTTCTTGTGATAATTTCTATAATTT
ACTTTAGATATCTCCACACTCCTACTAACTACCTTATCCTCTCTCTGGCTGTGGCTGATCTACTAGTTGGTGCTTTAATATTTCCTTTGAGCATGACAGTGACTCTAAAGTC
ATGTTTGTATAGATTCAGTTTACTATGCACCTTAAGAAGCGCAATAGATGTGATAATGGGTGTATCTTCCTTATTACATTTGTGCTGTATTTCTGTTGATCGATATTATGCTGT
TTGCCACCCTCTGATATATAAAACTAAAATGACTGATTGTGTTGCCATGAAGATGGGCCTTGGAAGTTGGGCTGGTGCTATCTTGATTGGAATTTTTGCCTTCCTGATGTT
TTTTATTCCAGGCGAATGTGACACAGGTTGCATTTTCACTCTCATTGCATCATCAGTGGTAGCATATTATATCCCAACAATAGTTTTAGTTTCCATGTACATCAAAATTCTTG
TTGTTGCACTGAGGCAGGCACGCAGCATCCATAATACAACTTCTGAGAACACAAAGTCTAAAGCAGTTTCCAGTAAGGAGAGAAAGGCCACAAAGACATTAACCATAGT
TATTGGAATATTTTTAATTTTTTGGGTTCCTCTTTATGTTAGTTATTCATTTGTTCCTCTGGATAGTTTTATACTTTATGAGGTTCTTGAACCATTTAACTGGTTTGCAATTTCT
AACTCAATGCTCAATCCCTTTATTTATGCTTTCTTTTACACTTGGTTTAGAAAAGCTTTTAAAATGATAATTTCTGGAAAA
>contig029635-BriTARe.A005
ATGGACGGCACTGGGGGTCCTCCCCTCTGCTTCCCTAACCTCAACTCCTCCTGCAGGCAGCTGCTGCGACCCACCTCCCAGGCCGCTCTTCTCTACACTCTGCTGG
CCTCAGTCTCACTGCTCACTGTGGTGCTCAACCTTCTCGTGGTCGTCTCCATCTCCCACTTCAAGCAGCTCCACACCCCGACCAACACCCTGCTCCTGTCCCTGGCC
ATGTCCGACCTGCTGTTGGGGTTACTGGTGATGCCCATCGAGGGCCTGCGCTACATCGAGACGTGCTGGCTGCTGGGGAGGCTGATGTGTGCTCTCAGTCCTTATTT
GTCTTACTGCCTCCTCTCTTTTTCTCTGGACAGCATGGTGCTCATATCTGTAGATCGCTATATAGCCATCTGTGACCCTCTGCTCTATTCCTCAAAGATCACAGTGAACAG
AGTGAAGCTTTCA
>contig082566-BriTARe.A006
CTTGTTGATATTTCTCTCTCTTCTTTCAGACAGCTCCACACACCCACTAACATCCTCCTCCTCTCTCTGGCTGTCTCAGACTTTCTCATTGGTCTGTTGATGATGCCGGC
AAAAATCCTACGACACACATCTTGTTGGTTTCTTGGTCAGCTTACATGTTCAATGTATATTTATGCATCCTACATCATTAGCTCTGCCTCAGTGGGCATTATGGTGCTGATA
TCAGTCGACCGCTATGTGGCTATTTGTGACCCTCTGCATTACCCCACCAGAATCACAGAGAGAAGAGTGAAACTCTGCGTCTGTCTGTGTTGGCTCTGCTCTGTTATTT
ACAACATACTATTTATAAAGAACGACCTGCTTCAAGGAGAACGACATACTTCCTGTTATGGAGAATGTGTAATTGTCATAGACTACATTGTAGGAACCACTGACATTGTTTT
AACTTTTATTGCTCCAGTTACTGTCATCGTATTTCTGTATATGAGAGTGTTTGTGGTGGCTGTGTCTCAGGCCCGTGCCATGCGCTCTCATGTTACAGCTGTCACACTGC
AGCTCTCAGTGACTCTAACAGCAAAGAAATCAGAGATAAAAGCAGCCAGGACTCTGGGTGTTCTTGTTCTTGTGTTTCTATTAGGTTTCTGCCCATATTACATTGTTTCA
CTTTTAGGAAACGAGTTGTTCAACAGCTCATCTGCATCCATTGTGATTTATCTGTATTATTTTAACTCCTGTCTAAATCCTTTGATTTACGCTATGTTCTACCCCTGGTTTAG
AAAAGCTGTGAAATTCGTTGTCACTCTACAGATACTGCAGCCTGGCTCCTGTGAGGTCAGCATACTGtag
>contig086341-BriTARe.A007
ATGGCAAAGAGCTTCAGACAGAAAGGTGGTGGAACTCCACAGCAAGCTATCTCTTCCTTTTTACTAATGATGGAAATGTTTGAAGAAACTGAACTCTGCTTTCCACAGC
TTCTTAACTTCTCCTGCAGGAAGCCCATACGACCTCACTTCAGGAAGCTGCACACCCCCACCAATCTCCTCCTCCTCTCTTTGGCTGTCTCTGATTGCCTGGTGGGTC
TCCTGATATTATTTCAAATTATGATTATAGATGGTTGCTGGTTTCTTGGTGAATTCATGTGTAGTATGTATTTTCTCTTGGACTACATTATTACCTCTGCGTCAATAGGAACCA
TGGTTCTCATATCAATTGACCGTTATGTGGCTATTTGTTACCCTCTCCATTACTCTACCAAAGTCACCCCAAAAAGAACAAAAGCATGTGTTTATCTGTGTTGGATTTGTTC
TTCAGTCTTC
>contig086348-BriTARe.A008
ATGGAAGAAACTGAACTCTGCTTTCCACAACTATTCAACTCCTCTTGTGTGCGGCAAAAGCGCCCACAAATTGAGGCTGTGTGTATTTACACCCTGCTATCATCTATCTC
TCTGCTTACTGCAGTACTGAACCTACTTGTCATCATCTCCATCGCTCACTTCAAGCTGCACACCCCAACCAACCTCCTCCTCGTCTCTCTGGCCGTCTCAGATTTCTTC
GTGGGCCTCATCATGGCTTTTGAGATTAGTCTCCTAGATGGCTGCTGGTTTCTTGGTGACCACATGTGTGCTCTGTATAGCAGTTTAGATTACATTGCTACTTCTGCTTC
AGTAGGAACTATGGTACTCATATCAGCTGACCGCTATGTAGCCATTTGTGACCCTCTGCATTATCCCACCAAAATTACTATAAAAAGAATCTCAGTCTGTATTTGTACTTGC
TGGGCTTGTTCAATTCTGTATAACAGTCTTATCATGAAGGATAATTTCAAGCAGCCAGGGAGGTATAATTCTTGTTCTGGTGATTGT
>contig109361-BriTARe.A009
ATGAGAGTATTTGTGGTGGCTGTGTCTCAGGCCCGTGCCATGCGCTCTCAGGTTACAGCTGTCACACTGCAGCTCTCAGTGACTCTAACAGCAAAGAAATCAGAGTTA
AAAGCAGCCAGGACACTGGGTGTTCTTGTTCTTGTGTTTCTAATATGTCTCTGTCCTCATTATTGTATTTTTCTTTTTGGAGGAAAGGTGCTTAATAGCTCATCTGCAACTA
TTGTGATCTATCTGTACTATTTTAACTCCTGTCTAAATCCTTTGATTTATGCTATGTTCTACCCCTGGTTTAGAAAAGCTGTGAAACTAATTGTCACACTACAGATACTGCAG
CCTGGCTCCTGTGAGGTCAGCATACTG
>contig116966-BriTARe.A010
ATGGAGATACAGAAAGGAGCTGAGCTCTGTTTTCCACAACTCCTCAACATTTCTTGCAGGAAGCCGACACTTCACTGGTCCAAAGCTGTGCTCCTGAACATTGTCTTGT
CCTGCATCTCTCTGCTCACTGCTGCTCTAAACCTTCTCGTGATCATCTCAGTCTCCTACTTCAGGAAGCTTCACACACCCAGCAATATCCTCCTACTCTCTCTTGCTGTC
TCAGACTTTCTCATGGGTCTCTTGTTGATGCCAGCAGAAATCCTTAGAAGCATGGCCTGCTGGGTACTTGGTGATCTTATGTGTTCTGTTTATTTTTTTCTGGCTGTAAAC
ATTAGCTGTGCTTCAATAGGGAACATAGTTCTTATATCAATTGACCGTTATGTGGCTATTTGTGACCCTCTGCATTACTCCACGAGAATTACTGTGCCTAGAGTCAAACTC
AGTGTTTGTCTGTGTTGGTTTTATACAACTTTCTACTGCAGTTTTTATACGCAAGAAATGCTGATTGAACCAGGCAGGTATAATTCCTGTTATGGTGAGTGTGTGTTTGTC
ATCAGTGATTTTTTTGGAATGGTTGACCTTATTTTATTTTTTATTCTTCCAGTTTCTGTCATCATAGTTTTGTATATGAGAGTATTTGTGGTGGCTGTGTCTCAGGCTCGTGC
CATGCGCTCTCATGTTACAGCTGTCACACTTCAGCGTTCATTGAATCAAACAAACAAATCTGAGCTGAAAGCAGCCAGGACTCTGGGGGTTCTTGTAGTTGTGTTT
>contig109362-BriTARe.A011
ATGGATACCCAGGGCAGAGCAGATCTCTGCTTTCCACAACTCTTCAACACCTCCTGCAAGAAGCCTACAACTCCTCTATCACAAGTTTTGCTCTTTCACATTCTGATGC
CATCAATGTCTCTGCTAACTGTGACTCTCAACCTGCTCGTCATCATCGCAGTCTCCCACTTCAGGCAGCTCCACTCACCCACTAACATCCTGCTCGTCTCTCTGGCTGT
CGCTGACTTTCTCACTGGTCTTCTGTTTATGCCTGGAGAAATCCTGCGAAATACAGAGTGTTGGTTTCTTGGTCAGCTCACATGTTCTCTGTATAATTATGTATCCTACAT
CATTGCCTCTGCCTCAGTGGGCAACATGGTGCTGATATCAGTGGACCGCTATGTGGCTATTTGTGACCCTCTGCATTACCCCACCAGAATCACAAAGAGAAGAGTGAA
ACTCTGTGTCTGTCTGTGTTGGCTCTGCTCTGTTTTCTACAGCTATGTGATTTTAATAGATGATCTAAGTCAACCAGGAAAGCATAACTCTTGCTTTGGAGAATGTATAATT
TTTGTTGAATTCATTGCAGGATTTGTTGACCTTGTTTTATCCTTTATTATTCCACTTACTGTCATTGTATTTCTGTATATGAGAGTATTTGTGGTGGCTGTGTCTCAGGCCCG
TGCCATGCGCTCTCAGGTTACAGCTGTCACACTGCAGCTCTCAGTGACTCTA
>contig109361-BriTARe.A012
ATGGATACCCAGGGCAGAGCAGATCTCTGCTTTCCACAACTCTTCAACACCTCCTGCAAGAAGCCTACAACTCCTCTATCACAAGTTTTGCTCTTTCACATTCTGATGC
CATCAATGTCTCTGCTAACTGTGACTCTCAACCTGCTCGTCATCATCGCAGTCTCCCACTTCAGGCAGCTCCACTCACCCACTAACATCCTGCTCGTCTCTCTGGCTGT
CGCTGACTTTCTCACTGGTCTTCTGTTTATGCCTGGAGAAATCCTGCGAAATACAGAGTGTTGGTTTCTTGGTCAGCTCACATGTTCTCTGTATAATTATGTATCCTACAT
CATTGCCTCTGCCTCAGTGGGCAACATGGTGCTGATATCAGTGGACCGCTATGTGGCTATTTGTGACCCTCTGCATTACCCCACCAGAATCACAAAGAGAAGAGTGAA
ACTCTGTGTCTGTCTGTGTTGGCTCTGCTCTGTTTTCTACAGCTATGTGATTTTAATAGATGATCTAAGTCAACCAGGAAAGCATAACTCTTGCTTTGGAGAATGTATAATT
TTTGTTGAATTCATTGCAGGATTTGTTGACCTTGTTTTATCCTTTATTATTCCACTTACTGTCATTGTATTTCTGTATATGAGAGTATTTGTGGTGGCTGTGTCTCAGGCCCG
TGCCATGCGCTCTCAGGTTACAGCTGTCACACTGCAGCTCTCAGTGACTCTAACAGCA
>contig086329-BriTARe.A004
ATGGAGGAAACTGAACTCTGCTTTCAACAACTCTTTAACACCTCCTGCATGAGGCCCAGGCGTCCACACTTTGAGATCATGCTGACTTATATTTTGCTGTCCTTCATTTC
TCTACTTACTGTGATTCTTAACCTGTTGGTCATCATCTCCGTCTCACATTTCAGGAGGCCCAGGCGTCCACACTTTGAGATCATGCTGACTTATATTTTGCTGTCCTTCAT
TTCTCTACTTACTGTGATTCTTAACCTGTTGGTCATCATCTCCGTCTCACATTTCAGGCTCCACACCCCCACCAACCTCCTGCTCCTTTCTCTGGCTGTTGCTGATTTAT
GTGTGGGCCTCCTCTTGTTCTTCCAAATTGTGCTCATAGATGGCTGCTGGTTCCTCGGTGACATCATGTGCACCCTGTATCAATACCTAGTATATGTCATCACCTCGGCC
TCAATAGGAACCATGGTGATCATATCTGTTGATCGATATTTGGCTATTTGTTACCCTCTGCATTACTCCACCAAGATCACACAACAAAGAGTTAAAATTGTTGTCTGTTTGT
GTTGGATCTGTTCTGTCATCTTTCAAAGTCTGATTCTGATGGATAACCTGAAGCAA
>contig086354-BriTARe.A001
GTTACATCTTTCTTTGTTTTTCTTCTCAGGCAGCTTCACAACCAAACCAACCTCTTCCTCCTCTCTCTCGCTGTCTCAGACTTCCTTGTTGGCCTCCTGCTGATGCCTCC
TCGAATCCTCCTTTTAGGGGGCTGCTGGGTTTTGGGCACCTTCATGTGTGGACTGTTTTACTATGCCTCTTTTGTTCTCACATCTGCCTCAGTAGGAAACATGGTGCTTA
TTTCATTTGACCGGTATGTAGCTATTTGTGACCCCTTGTCCTACCCCACCACAGTTACTGAAAGAAAAGTCCAAATCAGTGTCTGTCTGTGTTGGGCCTGTTCACTGCT
GTATAACGGCACAATACTAAACAACTTTCTGAAGCAGCCAGACAGATATAATTCTTGTGACGGGGAATGCATAGTTGTAATTGATTTCATAACTGGTGCTTTTGATGTCGT
AGTTACCTTCATCGGCCCCACTGCTGTGATTATATTTCTGTACGTGAGAGTATTTCTGGTGGCTGCGTCTCATGCTCAGGCTATGAGATCTCATGTTGCCTTTGTTACTTC
AAAGGGTTCAGTTCACATTAGGAAGTCTGAGAGAAAAGCAGCTACAACTATTGGTGTTGTTGTGGTTGTGTTTCTGATGTGTTTCTGTCCTTACTTTTATCCCTCGCTCG
CAGGGCAGGACACATCAACCAGTGTGGAATTTTCCGTTTTTGGGGTCTGGTTACTTTATTGTAATTCCTGTCTAAATCCACTTATCTATGCGTTTTTCTATCCCTGGTTTA
GGAAGACTGTTAAACTGATAGTTATGTTGCAGATTTTGCAGCCTGACTCCTGTGATGCCAACATACTGTAG
>contig109362-BriTARp.A013
ATGAGAGTATTTGTGGTGGCTGTGTCTCAGGCCCGTGCCATGCGCTCTCAGGTTACAGCTGTCACACTGCAGCTCTCAGTGACTCTAACAGCAAAGAAATCAGAGTTA
AAAGCAGCCAGGACACTGGGTGTTCTTGTTCTTGTGTTTCTAATATGTCTCTGTCCTCATTATTGTATTTTTCTTTTTGTCTCTGTCCTCATTATTGTATTTTTCTTTTTTGG
AGGAAAGGTGCTTAATAGCTCATCTGCAACTATTGTGATCTATCTGTACTATTTTAACTCCTGTCTAAATCCTTTGATTTATGCTATGTTCTACCCCTGGTTTAGAAAAGCTG
TGAAACTAATTGTCACACTACAGATACTGCAGCCTGGCTCCTGTGAGGTCAGCATACTG

>contig084868-BriTARs.A014
ATGGAGACCCAGGACGAAGCAGAGCTCTGCTTTCCACAACTCTTCAACACCTCCTGCAAGAAGCCTAAAACATCTCTGTCTCAAGTTTTGCTCCCTTACGTTGTGGTG
TTTTCAGTCTCTCTGCTAACAGTGGCTCTCAACCTTGTTGATATTTCTCTCTCTTCTTTCAGACAGCTCCACACACCCACTAACAGCCTCCTCCTCTCTCTGGCTGTCTC
AGACTTTCTCATTGGTCTGTTGATGATGCCGGCAAAAATCCTAGGAGACACAGCTTGTTGGTTTCTTGGTCAGCTCACATGTTCTCTGTATAGTTATATATGCTTCATCAT
TACCTCTGCCTCAGTGGGCATTATGGTGCTGATATCAGTCGACCGCTATGTGGCTATTTGTGACCCTCTGCATTACCCCACCAGAATCACAGAGAGAAGAGTGAAACTC
TGCGTCTGTCTGTGTTGGCTCTGCTCTGTTATTTACAACATACTATTTATAAAGAACGACCTGCTTCAAGGAGAACGACATACTTCCTGTTATGGAGAATGTGTATTTGTC
ATAGACTACATTGTAGGAACCACTGATATTGTTTTAACTTTTATTGCTCCAGTTACTGTCATCGTTGTTCTGTATATGAGAGTATTTGTGGTGGCTGTGTCTCAGGCCCGT
GCCATGCGCTCTCATGTTACAGCTGTCACACTGCAGCTCTCAGTGACTCTAACAGCAAAGAAATCAGAGATAAAAGCAGCCAGGACTCTGGGTGTTCTTGTTCTTGTG
TTTCTATTAGGTTTCTGCCCATATTACATTGTTTCACTTTTAGGAAACGAGTTGTTCAACAGCTCATCTGCATCCATTGCGATTTATCTGTATTATTTTAACTCCTGTCTAAAT
CCTTTGATTTACGCTATGTTCTACCCCTGGTTTAGAAAAGCTGTGAAATTCGTTGTCACTCTACAGATACTGCAGCCTGGCTCCTGTGAGGTCAGCATACTGtag
>contig084876-BriTARs.A015
ATGGATACACAGGACGTGGCAGAGCTCTGTTTTCCACAACTCTTCAACACCTCCTGCAAGAAACCTATAACTCCTCTGTCAGAATTTGTGTTCCTTCATGTTGTGTTGTC
CTCCATCTCGCTGCTAACTGTGACTCTCAACCTGCTCGTCATCATCTCAGTCTCCCACTACAGGCAGCTTCATACACCCACTAACATCCTCCTCCTCTCTCTGGCTGTC
TCAGACTTTCTTGTTGGTCTCCTGTTGATGCCTGGAGAAATCCTCCGAAATACAGCCTGCTGGTTTCTCGGTGACCTCACCTGTTTTATGTACGATTATATGTCTTTAATT
GTTACCTCTACCTCAGTGGGAGACATGGTGTTAATATCAATTGACCGCTATGTGGCTCTTTGTGACCCTCTGCATTACCCCACCAGAATCACAGACAGAAGAGTGAAAC
TCTCTGTCTGTCTGTGTTGGCTCTCTTCTGTTTTCTATAGCAGCCTGTTTGTAAAGGATGATCTAACTCATTCAGGGAAGCATAATTCCTGCTATGGAGAATGTACAATTG
TTGTTGACTTAATTACAGGAACGATTGACCTTCTTTTAACCTTTTTTGTTCCAGTTACTGTCATTGTAGTTCTGTATCTGAGAGTATTTGTGGTGGCTGTGTCTCAGGCTC
GCGCCATGCGCTCTCATGTTACAGCTGCTGCTCTGCAGCTTTCAGTGACTCTAACAACAAAGAGATCAGAGTTAAAAGCAGCCAGGACTCTGGGTGTTCTTGTAGTTG
TGTTTCTACTGTGTTTCTGCCCTTATTATTGTGTTACTCTTGCCAGGGACGACCTACTCAATAGTTCATCTGTATCCTTTTTGCTCTATCTGTTCTATTTTAACTCATGTCTA
AACCCTTTGATCTATGCACTGCTCTACCCCTGGTTTAGAAAAGCTGTGAAACTCATAATCTCTTTACACATACTGCAGCCTGGCTCCTGTGAGATTAGCATTCTGtaa
>contig084880-BriTARs.A016
ATGGAGATCCAGATGCATCCAGAGGCAGAGCTCTGTTTTCCAGAGCTACTGAACAGTTCCTGCAGGAAGCCAACACTTCACTGGTCCAAAACGGTGCTCCTGAACGT
TGGGCTTTCGTCCATCTCTCTGATCACTGCTGCTCTTAATCTGTTCATCATCATCTCGGTCTCCCACTTCAGGCAGCTCCACACTCCCAGTAACATCATCATCCTCTCTC
TGGCTGTCTCTGACTTTTTTGTCTGTTTTTTGTTGATGCCGGTAGAAATCTTCAAAAACACAGCCTGCTGGGTATTTGGTGATCTCATGTGTTCACTTTATACTTATCTGA
GCTGCATTCTTATCAATGCTTCGTTTGAAATGATTATTCTCGTATCAATTGACCGCTATGTGGCTATTTGTGACCCTCTGCATTACCCCACCAGAATCACTGTGCCGAGAG
TCAAACTCTGTGTTTTTCTGTGTTGGTTTTATGCTATTTTGTACAACATCATCTATACAAAGCATGCCCTGATAAACCCAGGCAGGTATGGTTCCTGCTATGGAGAGTGTG
TTTTTGTTGTTGATGATATTATTGGAATTGTTGACTTTGTTGTATCTCTTATAGTTCCAGTTACGATCATCGTAGTTCTGTATACGAGAGTGTTTGTGGTGGTTGTGTCTCA
GGCTCGTGCCATGCGCTCTCATGTTACAGCTGTTACACTTCAGCGTCCACTGAATCAAGCAAACAAATCTGAGCTGAAAGCAGCCAGGAACCTTGGGATTCTTGTAGT
AGTTTTTCTGGCGTGCTACTGTCCATTTTACTGCTACTTTTTTCTTGCAGGCAATGAAGTTAATGCTTCTTCTGCATCTTCTATCCTCATTGTTTATTATTTTAACTCTTGCC
TAAACCCATTAATGTATGCACTGTTTTACCCCTGGTTTAGAAATGCTGTTAAACTTATCATCACTCTGCAGATATTAAAGGCTAACAGCAGTGAGATCAACATACTAcagagat
aa
>contig084886-BriTARs.A017
ATGCAGAAAGAAGCCGAGCTCTGTTTCCCACAACTCATCAACAGTTCCTGCAGGAAGCCAACACTTCACTGGTCCAAAGCTGTGCTGCTGAACATTGTGCTGTCCTGC
ATCTCTCTGCTCACTGCTGCTCTAAACCTTCTCGTCATCATCTCAATCTCTTACTTCAGGCAGCTTCACACACCCACTAACATCCTCCTCCTTTCTCTGGCTGTCTCAGA
CTTTCTCACTGGTCTCCTTATGCTGCCACTAGAAATAATTAGAAACACGGCCTGTTGGGTACTTGGTGATCTTATGTGTTCTGTTTATACTTGTCTGACAACCGGTCTTTT
CTGTGTTTCAACTTGGAACGTAGTTCTCATTTCAGTTGACCGCTATGTAGCTATTTGTCACCCTCTTCATTACCCCACCAGAATTACCGTGGCAAGAGTCAAACTCTGTG
TTTGTCTGTGTTGGTTTTATTCAACCTTCTACGGCAGTCTTTTAACAAAGGATGTTCTAATTAAGCCGGCCAGGTATAATTCCTGTTATGGAGAGTGTGTGTTTGTCATTAA
TGATATTGCAGGGTTTATTGATCTTGTGTTATCTTTTATTGTTCCAGTTTCTGTCATCATAGTTCTGTATATGAGAGTGTTTGTGGTGGCTGTGTCTCAAGCTCGTGCCATG
CGCTCTCGTGTTACAGCTGTCACACTTCAGCGTTCAAGCAATCAAGCAAAGAAATCTGAACTGAAAGCAGCCAGGACTCTTGGAGTTCTTGTAGTTGGGTTTCTTGCAT
GTTACCTTCCATTTTACTGTTACTCTCTTATTGAAGTAAATGTGATCAATGACTCATCTACATTTTTTCTGATCATTGTGTTGTACTTTAACTCGTGTCTAAACCCACTGATGT
ATGCCCTTTTTTACTCTTGGTTTAGAAATGCTGTTAAACTTATTATCACTCTGCAGATTTTTAAGCATGACACTAGTGAAGCAAACATGttttaa
>contig084887-BriTARs.A018
ATGGAGATACCGAAAGGAGTCGAGCTCTGTTTCCCACAACTCCTCAACAGCTCCTGCAGGAAGCCGACACTTCACTGGTCCAAAGCTGTGCTCCTGAACATTGTGCT
CTCATGTATCTCTCTGCTCACTGCTGCTCTAAACCTTCTCGTCATCATCTCAGTCTCCCACTTCAGGCAGCTGCACACACCCAGTAATATCCTCCTCCTCTCTCTGGCC
GTCTCAGACTTTTTTGTGGGTCTCCTGTTGTTGCCTTTAGAAATTTTTAGAAAGACAAGCTGCTGGGTACTTGGTGATCGTATGTGTTCTGCTTATTGGTATTTGACCAG
CAACATTATCTGTGCTTCAATAGGGAACATTGTTCTAATATCAGTTGACCGCTATGTGGCTATTTGTGACCCTCTGCATTATCCCAGCAGAATTACTTTGGCGAAAGTCAA
ACTCAGTGTTTGTCTGTGTTGGTTTTATGCTTTTTTCTACAGCAATCTTTATACAAAGGATATCATGATTGAACCAAGCAGGTATAATTCTTGCTTTGGAGAGTGTGTATTTT
TTAGCAGCAATATTGCTATTGTTGTTGACCTTATTTATTCTTTTTTGTTCCAGTAACTGTTATCATAGTCTTGTATATGAGAGTATTTGTGGTGGCTGTGTCTCAAGCTCGT
GCCATGCGCTCTCATGTTACAGCTCTCACACTTCAGCGTTCACTGAATCAAACAAACAAATCTGAGCTGAAAGCAGCCAGGACTCTGGGGATTCTTGTAGTTGTGTTTC
TAGCATGCTTCTCTCCACTCTACTGCTACTCTCTTGTTGATGAAAATGCAATCATTGATCCAGCTGCATCTTTTGTGGTCATTATCTTTTACATTAACTCTTGTCTAAACCCT
TTGATCTATGCCTTGTTTTACCCCTGGTTTAGAAACGCTGTTAAACTTATCATCACGTTGGAGATCTTCAAGTATGACACAagtggtgccaacatactgtaa
>contig086337-BriTARs.A019
ATGGAGGAAACTGAACTCTGCTTTCCAAAACTCCTTAATATCTCCTGCAGGAGGCCAAAGCGTCCTCACTTTGAGATCATGCTGACTTACATTCTGCTCTCCTTCATTTC
TTTGCTTACTGTGATTCTTAACCTGCTGGTCATTATCTCCATCTCACACTTCAGGCAGCTCCACACCCCCACCAACTTCCTCCTTCTCTCTCTGGCTGTCGCTGATTTCT
TTGTAGGTTTCCTTATGTTCTTTCAAATAGTGCTCATCGATGGATGCTGGTTCCTCGGTGACATCATGTGCACTCTGTATCAGTATCTAGCATTCATTATTACTTCAGCCTC
AATAGGAACCATGGTGATCATATCTGCTGATAGGTATTTGGCTATTTGTTACCCTCTGCATTACTCCACCAAAATCACACAACAAAGAATTAATATATGTATAAGTTTGTGTT
GGTTTTTTTCTGTAATCTTTCAGAGTTTGATTGTGAAGGATAACTTGAAACAACCAGGAAAGTATAACTCCTGCATTGGAGAGTGTGTCTTTGTCGTTAACTACATTGCTG
GGATTTTTGATCTTTTGTTTTCCTTCATTGTTCCCATTACTGTGATTGTAGTTCTGTATCTGAGAGTGTTTGTGGTGGCTGTGACTCAGGCTCGTGCCATGAGGTGTCAA
CTTGCAGTCACTCACCAGCGATCAGTTACAGTAACTGTTATGAAATCGGAGCTGAAAGCAGCCCGTACTCTTGGTGTTGTTGTAGTTGTGTTTCTTATATGTATGTGCCC
ATATTACTGCGTTGCTCTCACAGGGCAAGATAACTTCCTAAATGCTTCATCTGCTGCCTTTGTAATATGTTTGGTGTACTTTAACTCTTGCCTAAACCCTATCATTTATGTCT
TTTTTTATCCCTGGTTCAGAAAGTCAATCAAACTTATTGCTACTCTTCAAATACTGCAGCCTGACTCCTGTGAGACTAACATGCATtaa
>contig086344-BriTARs.A020
ATGGAAGAAACTGAACTCTGCTTTCCACAACTATTCAACTCCTCTTGTGTGCGGCAAAAGCATTCACAAATTGAGGCTGTGTTTCTTTACACTCCACTGTCTTCCATCTC
TGTGCTTACTACAATTCTGAACCTGCTTGTGATCATCTCCATCGCTCACTTCAAGCAGCTGCACACCCCAACCAACCTCCTCCTCCTCTCTCTGGCCGTCTCAGATTTC
TTCGTGGGCCTCATCATGGCTTGTCAGATTAGTCTCCTAGATGGCTGCTGGTTTCTTGGTGACCACATGTGTGCTCTGTATACCACTTTAGATTACATTGTTACTTCTGCT
TCAGTAGGAACTATGGTATTCATATCAGCTGACCGCTATGTAGCCATTTGTGACCCTCTGCATTATCCCACCGAAATTACTATAAAAAGAATCTCAGTCTGTATTTGTACTT
GCTGGGCTTGTTCAATTCTGTATAACAGTCTTATCATGAAGGATAATTTCAAGCAGCCAGGGAGGTATAATTCTTGTTCTGGTGATTGTGCAGTTGTCATTGATTACTTTG
TGGGAATTTTTGACTTTGTTTTGACCTTTGTCGGCCCTGTGATTGTCATCATAGTTCTGTATCTAAGAGTATTTGTGGTGGCTGTGTCTCAGGCTCGGGCAATGCGCTCT
CATATCACTGCTCTCAGACTCCAGGGGTCAGAGACAGTGCATGCTAAGAAATCTGAGCTGAAAGCTGCCAGGACTCTCGGTGTACTTGTTATTGCCTTTCTGATATGTC
TTTTCCCATTTTTCTGTTCCTCAATGGTAGGCCAGAATAGTTTCTTTGATATTAGATCTGTACCTTTTGAGCGCTTGCTGTTCTACTTTAACTCTTGTCTGAACCCATTAATC
TACACTTTTTGCTACCCCTGGTTTCTGAAATCTATCAAGCTCATTGTGACATTTAAGATATTTAGGCATGGTTCCAGTGAGGCCAGTATACTAtag
>contig086351-BriTARs.A021
ATGGCAGAAACTGAACTCTGCTTTCCAAATCTCAACTCCTCCTGCAGTAGAGTAAAGCGTTCTCCCTCGGAGTCAGTGCTTATTTATGTGATTCTAACCATCATTTCTCTT
CTTACTGTGGCTCTAAACCTGCTGGTCATCATCTCCATCTCCCACTTCAAGCAGCTCCACACCCCTACAAACTACCTCCTCCTCTCCCTGGCAGTCTCTGATTTCTTTGT
GGGTTTCAATATGTGCTTTCAAAGTATGCTCATAGATGGTTGTTGGTATCTTGGTGACCTCATGTGTGTCTTGTATTATGTTTTCGAGATAGTTGTTACTTCTGCCTCAGTA
GGAACCATGGTGCTCATTTCAGTTGACCGTTATGTGGCCATTTGTGATCCTCTTCATTATCCCACCAAAGTCACTCCAAAAAGAGTTCAGACCTGTGTTTTAATATGTTG
GATTTGCTCGCTCCTAGTAGTCGGTGTGCTATTGAAGGATAACCTGGATAAACCAGGTAGGTTTAATTCCTGCTTTGGAGAGTGTGTGATTTATATTGACTTTGCAATACA
AGTTACCGATCTTATTTTTACAATCCTCCTTCCCATTACTGTCATCGTAATTTTGTATGTAAGAGTATTTGCTGTGGCCGTGTTTCAGATTCGGGCCATGCAACATCATGTT
GCAGCTGGCACACAGAGGGGGAAAGTAAGTCCAAAAAAATCTGAACTGAAAGCAGCTAGGACTCTTGGCATTGTTATTGTTGCATTTCTGATATGCCTATTCCCATATTA
CTGTGTAATACTCTCAGGCCAGGACACACTGCTTGATATTTTATCCCTTGCCTTTTTTTTATGTTTGTTTTATTTTAATTCCTTTCTCAACCCTATCATCTATGCCCTTTTCTA
CCCATGGTTTAGAAAATCTGTTAAACTTATTGTTACATTTCAAATAGTCAAATCTGGCTCCAGTGATGCCAGCATGCTGtga
>contig082565-BriTARs.A022
ATGGATACTCAGGATGGAGCAGAGCTCTGCTTTCCACAACTCTTCAACATCTCCTGCAAGAAACCTACAACTCCTCTGTCTCAAGTTTTGCTCATTTATACTGTTGTGTC
CTCAATGTCTCTGCTGACTGTGACTCTCAACCTGCTCGTCATCATTGCAGTCTCCCACTTCAGGCAGCTCCACACACCCACTAACATCCTGCTCCTTTCTCTGGCTGTC
ACTGACTTTCTCGTTGGTCTTCTGTTTATGCCTGGAGAAATCCTGCGAAATACAGCGTGTTGGTTTCTTGGTCAGCTCACATGTTCACTGTATAATTATGCATCCTACATC
ATTGCCTCTGCCTCAGTGGGCAACATGGTGCTGATATCAGTCGACCGCTATGTGGCTATTTGTGACCCTCTGCATTACCCCACCAGAATCACAGAGAGAAGAGTGAAA
CTCTGCGTCTGTCTGTGTTGGCTCTGCTCTGTTTTCTACAGCTATGTGATTTTAATAGATGATCTAAGTCAACCAGGCAAGCATAAATCTTGCTATGGAAAATGTATAATTT
TCATTGAATTCATTGCAGGATTTGTTGACCTTGTTTTAGCTTTTATTATTCCACTTACTGTCATCATTGTTCTGTATATGAGAGTGTTTGTGGTGGCTGTGTCTCAGGCCCG
TGCCATGCGCTCTCAGGTTACAGCTGTCACACTGCAGCTCTCAGTGACTCTAACAGCAAAGAAATCAGAGTTAAAAGCAGCCAGGACTCTGGGTGTTCTTGTTCTTGT
GTTTCTATTATGTTTCTGCTCATATTACATTATTTCACTTTTTGGAAACGAGTTGCTCAATAGCTCATCTGCATCGATTGTGATCTATCTGTACTATTTTAACTCCTGTCTAAA
CCCTTTGATTTATGCTATGTTCTACCCCTGGTTTAGAAAAGCTGTGAAACTAATTGTGACTCTACAGATACTGCAGCCTGGCTCCTGTGAGGTCAGCATACTGtag
>contig029634-BriTARe.A023
ATGGATAGCTCTGGAGGTCCTCCCCTCTGCTTCCCCAACCTCAACTCCTCCTGCAGGCGGCTGCTGCGACCCACCTCCCAGACCGTTGTTCTCTACACCCTGCTGGC
CTCCATCTCCCTGCTCACTGTGGTGCTCAACCTGCTTGTCGTCGTCTCCATCTCCCACTTCAGACAGCTTAACACCTCGACCAACACCCTGCTCCTGTCCCTGGCTGT
GTCTGACCTGCTGGTGGGGCTGCTGGTGATGCCCATCGAGGGCCTG
>contig084878-BriTARe.A024
ATGATGGAGAACAAGGACAGTATATGGTTTCCAGAAGTCTTCAACTCTTCCTGCAGGAGACCAGCATTTCACTGGTCTGAAGCTGTGCCCCTGAATACCCTGCTGTGC
TCCATCTCTGTAATCACCATAGCTCTGAACCTCCTTGTCATCATCTCAGTCTCCTACTTCAGGCAGATT
>contig011701-BriTARe.B027
TCTGGAGCAACTGCCAGTAAGATGGAGAAAAAGGCCACCAAGACTCTAGCTATTGTTATAGGAGTTTTTCTCTTATGTTGGACTCCTTACTTTCTTTGTATTATCTTTCAG

CCTATAACATATAATGAAACACCAATTGCAGTTATTGAAACACTTAACTGGCTTACATTGTCAAATTCAATGCTAAATCCATTCATTTATGCTTTCTTTTATAGCTGGTTCAGG
TCAGCTTTTACAACAATTATTTCTGGAAAAATATTTCAAAGTGATTGA

TetraodonTAAR gene sequences

>TETRENSTNIP2491
ATGGATAGCAACGGGGACCCTTGGCTCTGCTTCGCCAACCTTAACTCCTCCTGCAAGAGG
CTGAACCAAACCTTCTCTGAAACCGCTGTGCTGAACAGCCTGCTGGCCTCGATCTCCCTG
GTCACCGTCACACTCAACCTTCTCGTCATCGTCTCTATTTCTCACTTTAGGCAGCTGCAC
ACACCCACCAACGCCCTGCTCCTCTCTCTGGCTGTGTCGGATCTGCTGGTGGGCCTGCTG
GTGATGCCGATCGAGGGCCTGCGCTACGTGGAAACATGCTGGTTGCTGGGGAGTCTGATG
TGTGCTCTGACTCCTTATGTGTCCTACTCTGTACTTTCTGCGTCTGTTGGCAACATGGTG
CTCATTTCCATAGACCGTTACCTGGCTATCTGTGACCCACTGCTCTATTCCAATAAGGTG
ACTCTGAAAAGAGCCAAAATTGCAATCTGTGTCTGTTGGGCCGGTTCTCTTTTCTATAAC
GGATGTATTCTGATCGGCCACCTCAAACATCCAGACAGGTACAGTTCTTGTCACGGAGAG
TGTGTGGTTCTGATCGACCACATCTCTGGAACTGCTGATCTGTTCATTACAGTTGTTTTC
CCCTGTACCATCATGGTTGTGATGTACATGAGAGTGTTTGCAGCTGCTGTTGCTCAAATG
CGAGTCATTCGTTTGCAGAACGCCGCCGTCGCTGTGAACGCAGCGACCACCGTGAAAAAA
TCAGAGTGGAAAGCAGCCAGAACTCTGGGGATTGTGATCGCTGTGTATCTAATGTGTTTT
TGTCCCTATTACTATCCAGCTCTTGCAGGTGGAGACACCTCAAACAGCTTGTCCTACTTT
GCTGCTTTGTCTTGGATAATGATGATTAATTCTTGTGTGAACCCTCTGATTTATGCTCTG
TTCTATCCCTGGTTCAGGAGATCCATCAAACTCATCATCACACTCAGGATACTGCGTTCA
TACTCCAGCGAGATCAAGATCATTTAA

>TETRENSTNIP3943
ATGGATAGCAACGGGGACCCTTGGCTCTGCTTCGCCAACCTTAACTCCTCCTGCAAGAGG
CTGAACCGAACCTTCTCTGAAACCGCTGTGCTGAACAGCCTGCTGGCCTCGATCTCCCTG
GTCACCGTCACACTCAACCTTCTCGTCATCGTCTCTATTTCTCACTTTAGGCAGCTGCAC
ACACCCACCAACGCCCTGCTCCTCTCTCTGGCTGTGTCGGATCTGCTGGTGGGCCTGCTG
GTGATGCCGATCGAGGGCCTGCGCTACGTGGAAATGTGCTGGCGGTTAGGAAAGCTGATG
TGTGTTCTGGCTCCTTATGTGTCCTACTCTGTACTTTCTGCGTCTGTTGGCAACATGGTG
CTCATTTCCATAGACCGTTACCTGGCTATCTGTGACCCACTGCTCTATTCCAATAAGGTG
ACTCTGAAAAGAGCCAAAATTGCAGTCTGTGTCTGTTGGGCCGGTTCTGTACTCCACAAC
GGATGTATTCTGATCGGCCACCTCAAACATCCAGAAAGGTACAGTTCTTGTCACGGAGAG
TGCGTGGTTGTGATCGACCACATCTCTGGAACTGTTGATCTGTTTATAACCTTTTTTGCA
CCTTGTACCATCATGGTTGTGATGTACATGAGAGTGTTTGCAGCTGCTGTTGCTCAAATG
CGAGTCATTCGTTTGCAGAACGCCGCCGTCGCTGTGAACACAGCTACCACCGTGAAAAAA
TCAGAGTGGAAAGCAGCCAGAACTCTGGGGATTGTGGTCAGTGTGTTTCTAATGTGTTTT
TGTCCCTATTACTATCCAGCTTTCGCAGGTGAAGACACCTCAAACAACTCATCCTACTTT
GCTGCTTTGGTTTGGATAATGATGATTAATTCCTGTATGAACCCTCTGATTTATGCTCTG
TTCTATCCCTGGTTCAGGAGATCCATCAAACTCATCATCACACTCAGGATACTGCGTTCA
TACTCCA

>TETRENSTNIP3225
ATGGAGCCAGTCTTCTGTTTGGAGAAGAACATTTCCTGTGTAAAGACCGTCTATCCGCTG
CCCTTTCGTGTCATCCTTTACATGGTTTTAGGGGTCATGGTCATTGTGACTGTGTGTGGA
AACCTTTTGGTCACTGTTTCTATCATCTATTTTAAGCAGCTCCACACTCCAACAAACTAC
CTGTTGGTCTCTCTTGCTGTGTCTGACCTTTTCTTGGGGTTGTTGGTCATGTTACCCAAT
ATGATTCAGTCAGTAGAAACTTGCTGGTATTTTGGAGACATCTTGTGCAAAGTCTATTTG
AGTATCGGTGTGATGTTGTGCACAGCATCCATAATTAATCTGTCTTTAATATCAATTGAT
CGGTACAATGTGGTGATCCATCCTTTACGTTATAGAAGAAAAATGTCTGGTAATGTTATT
TTGTTAATGATTTCTTTCAGCTGGGGTGTTTCAGGTGTTGTAGGTTTTGGAATGATTTTT
TTCCGACTGAATATTTTGGGAATTGAAGATTTCTACAACTCTGTTGCATGTGAAGGAAGA
TGTATTTTTTTGCAGAGTGGTATGTCAAGTACAGTCTCGTCAGTGCTCTCCTTTTATATT
CCAGGAATAATAATGATCAGTTTATACCTGAAGATTTTCATTGTGGCAAAGAGACAATTC
CTCAGCATTCAGAATACAACCTGCATGGCATCAGCCAGAGATTCAAATAAAAAACAGACA
AAAGCCACTAAAACGCTTGTTGTGATCATGGGAGCATTTCTTTTATGCTGGGCTCCGTTT
TTTGTCTGTAATATAATTCATCCCTTTATCAGCTACTCGACACCAGCTGCATTGTTTGAA
ACTTTTTTATGGGCAGGTTATATTAATTCTACACTGAACCCTATCATTTATGCCTTTTTT
TACACCTGGTTTAGAAAAGCATTCCGTCTTTTCGTCTCAGGTAAGATCTTTAAAGATGAT
ATGTCAAACATTACCCTTTTC

>TETRENSTNIP1455
ATGGAGCCAGTTTTCTGTTATGAATCTAAGAACATTTCCTGTGTAAAGACCGTCTATCCG
CTGCCCATACGTGTCATCCTTTACATGGTTCTAGGGGTCATGGTTGTTGTGACTGTGTGT
GGAAACCTTTTGGTCACTGTTTCTATCATCTATTTTAAGCAGCTCCACACTCCAACCAAC
TACCTGTTGGTCTCTCTTGCTGTGTCTGACTTTCTCCTGGGGTTCTTTGTCATGTTACCC
AGTATGATTCAGTTGGTAGAGACTTGCTGGTATTTTGGTGATATCTTGTGTAAAGTCCAT
TTGAGTTTTGCTGTAATGTTGTGCACAGCATCCATAATTAATCTGTCTACAATATCAATT
GACCGATACAATGCGGTGATCCATCCTCTACTGTACAGAAGAAAAATGTCTGGTAATGTT
GTTTTCCTCATGATTTTTTTCAGCTGGAGTGTTTCAGGTGTTGTAGGTTTTGGAATGATT
TTTTTAAGACTGAACATTTTAGGAATTGAAGATTTCTACAACAACTTTGTTGCATGTGAA
GGAAGATGTGTTTTGTTACAGAGTGGTCTGTCAAGTACAGTCTCGTCAGTGCTCTCCTTT
TATATTCCAGGAATAATCATGATCAGTTTATACCTGAAGATTTTCTTTGTGGCAAAGAGA
CAATTCCTCAGCATTCAGAATACAAGCAGCATGACATCAGCCAGAGATTCAAATAAAAAA
CAGGCAAAAGCCACTAAAACTCTTGTTGTGATCATGGGAGCGTTTCTTTTATGCTGGGCT
CCATTTTTTCTCTGTAACATCATTGATCCTTACATCAGCTACTCAACACCAGCTGCATTG
ATTGAAACATTCCTATGGGTAGGTTATATTAATTCTACACTAAACCCTCTCATTTATGCA
TTTTTTTACACCTGGTTTCGAAAGGCGTTTTGCCTGTTCGCTTCAGGAAAGGTCTTTAAA
GAGGATATGTCAAACATTTCCCTTTTTACTGAT

>TETRENSTNIP7922
ATGGAGCCAGTTTTCTGTGTTGAATCTAAGAACATTTCCTGTGTAAAGACCGTCTATCCA
CTGCCCTTTCGTGTCATCCTTTACATGGTTCTAGGGGTCACGGTTATTGTGACTGTGTGT
GGAAACCTTTTGGTCACTGTTTCTATCATCTATTTTAAGCAGCTCCACACTCCAACCAAC
TACCTGTTGGTCTCTCTTGCTGTGTCTGACTTTCTCCTGGGGTTCTTGGTCATGTTACCC
GATATGATTCAGTTGGCAGAGACTTGCTGGTATTTTGGAGACATTTTGTGCAAAGTCCAT
TTGAGTTTTGCTGTAATGTTGTGCACAGCATCCATAATTAATCTGTCTTTAATATCAATT
GATCGATACAATGCGGTGATTCATCCTCTACTGTACAGAAGAAAAATGACTGGTAATGTT

GTTTTGCTGATGATTTTTGTCAGCTGGAGTATTTCAGGTGTTGTAGGTTTTGGAATGATT
TTTTTCAGGCTGAATATTTTGGGAATTGAAGATTTCTACAAGGACTTTGTTGCATGTGAA
GGAAGATGTATTTTGTTTCAAAGTGGTATGTCAAGCACAGTCTCGTCAGTGCTCTCCTTT
TATATTCCAGGAATAATCATGATTTGTGTATACCTGAAGATTTTCTTTGTGGCAAAGAGA
CAATTCCTCAGCATTCAGAATACAACCTGCATGACATCAGCACGAATTTCAAATAAAAAA
CAGACAAAAGCCACTAAAACTCTTGTTGTGATCATGGGAGCGTTTCTTTTATGCTGGGCT
CCATTTTTTCTCTGTAATGTCATTGATCCTTTCATCAGCTACTCAACACCAGCTGCATTG
ATGAAAACATTTGGATGGGTGGGCTTTATTAATTCTACACTAAACCCTCTCATTTATGCG
TTTTTTTACACCTGGCTCAGAAAGGCCTTTTGCCTGTTTGCTTCAGGGAAGGTCTTTAAA
GAGGATATGTCAAACATTACCCTTTTCACTGAA

>TETRENSTNIP7160
ATGGAGCCAGTCTTCTGTTATGAGTCCAAGAACTTTTCCTGTGTAAAGACCGTCTATCCA
CTGCCCATTCGTGTCATCCTTTACATGATTTTCGGGGTCATTGTCATTGTGACTGTGTGT
GGAAACCTTTTGGTCACTGTTTCTATCATCTATTTTAAGCAGCTCCACACTCCAACCAAC
TACCTGTTGGCCTCTCTTGCTGTGTCTGACTTTCTCCTGGGGTTCTTGGTCATGTTACCC
GATATGATTCAGTTGGTAGAGACTTGCTGGTATTTTGGAGACATCTTGTGCAAAGTCCAT
TTGAGTTTTGCTGTGATGTTGTGCACAACATCCATAATTAATCTGTCTTTAATATCAATT
GATCGATACAATGCAGTGATCCATCCTCTACTGTATAGAAGAAAAATGTCTGGTCATGTT
GTTTTGCTGATGATTTTTTTCAGCTGGAGTGTTTCAGGTGTTGTAGGTTTTGGAATGATT
TTTTTCAGACTGAATATTTTGGGAATTGAAGATTTCTACAACTTTGTTGCATGTGAAGGA
AGATGTGTTTTTTTGCAGAGTGGTATGTCAAGTACCCTCTCGTCAGTCCTCTCCTTTTAT
ATTCCAGGAATAATAATGATCAATTTATACCTGAAGATTTTCTTTGTGGCAAAGAGACAA
TTCCTCAGCATTCACAATACAACCTGCATGACATCAGCACAAATTTCAAATAACAAACAG
GCAAAAGCCACTAAAACTCTTGTTGTGATCATGGGAGTGTTTCTTTTATGCTGGGCTCCA
TTTTTTCTCTGTAACATCATTGATCCTTTCATTAGCTACTCAACACCAGCTGCACTGTTT
AAAACAATTTTATGGGGAGGTCTTTTTAATTCTACACTGAACCCTATCATTTATGCATTT
TTTTACACCTGGTTTAGAAAGGCATTTCATCTGTTTGTCTCAGGCAAGGTCTTTAAAGAT
GATATGTCAAACACTAGCCTTTTCGTGGAA

>TETRENSTNIP3518
ATGGAGCCAGTCTTCTGTTTCGAGAAGAACATTTCCTGTGTAAAGACCGTCTATCCGCTG
CCCTTTCGTGTCATTCTTTACATGGTTCTAGGGGTCATGGTCGTTGTGACTGTGTGTGGA
AACCTTTTGGTCACTGTTTCTATCATCTATTTTAAGCAGCTCCACACTCCAACCAACTAC
CTGTTGGTCTCTCTTGCTGTGTCTGACTTTCTCCTGGGGTTCTTGGTCATGTTACCTGAT
GTGATTCAGTCGGTAGAAACTTGCTGGTATTTTGGAGACATCTTGTGCAAAGTCCATTTG
AGTTCTGATGTGATGTTGTGCACAGCATCCATAATGAATCTGTCTTTAATAGCAATTGAT
CGATACAATGCGGTGATCCATCCTCTAGTGTATAGAAGAAAAATGTCTGGTCATGTTGTT
TTGCTGATGATTTTTTTCAGCTGGAGTGTTTCAGGTGTTGTAGGTTTTGGAATGATTTTT
TTCAGGCTAAACATTTTTGGAACTGAAGATTTCTACAACAACTTTGTTGCATGTGAAGGA
AAATGTCTTTTATTTCAAAGAGGTTTGTCAGGTACAGTCTTGTCAGTGCTCTCCTTTTAT
ATTCCAGGAATAATAATGATCAGTGTATACCTGAAGATTTTCTTTGTGGCAAAGAAACAA
TTCCTCAGCATTCAGAATACAACCTGCATGACATCAGCCAGAGATTCAGATAAAAAACAG
GCAAAAGCCACTAAAACTCTTGTTGTGATCATGGGAGCATTTCTTTTATGCTGGGCTCCA
TTTTTTCTCTGTCACATCATTGATCCTCTCATCAGCTACTCAACACCAGCTGCAATGTTT
GAAACATTTGTATGGCTAGGTTATATTAATTCTACACTAAACCCTCTCATTTATGCATTT
TCTTACACATGGTTTAGAAAGGCGTTTCGCCTGTTTGCTTCAGGAAAGGTCTTTAAAGCA
GGATATGTCAAACATTTCCCTTTTTACTCATTAAATGGC

>TETRENSTNIP7161
ATGGAGCCAGTTTTCTGTGTTGAATCTAAGAACATTTCCTGTGTAAAGACCGTCTATCCA
CTGCCCTTTCGTGTCATCCTTTACATGGTTCTAGGGGTCACGGTTATTGTGACTGTGTGT
GGAAACCTTTTGGTCACTGTTTCTATCATCTATTTTAAGCAGCTCCACACTCCAACCAAC
TACCTGTTGGTCTCTCTTGCTGTGTCTGACTTTCTCCTGGGGTTCTTGGTCATGTTACCC
GATATGATTCAGTTGGCAGAGACTTGCTGGTATTTTGGAGACATTTTGTGCAAAGTCCAT
TTGAGTTTTGCTGTAATGTTGTGCACAGCATCCATAATTAATCTGTCTTTAATATCAATT
GATCGATACAATGCGGTGATTCATCCTCTACTGTACAGAAGAAAAATGACTGGTAATGTT
GTTTTGCTGATGATTTTTGTCAGCTGGAGTATTTCAGGTGTTGTAGGTTTTGGAATGATT
TTTTTCAGGCTGAATATTTTGGGAATTGAAGATTTCTACAAGGACTTTGTTGCATGTGAA
GGAAGATGTATTTTGTTTCAAAGTGGTATGTCAAGCACAGTCTCGTCAGTGCTCTCCTTT
TATATTCCAGGAATAATCATGATTTGTGTATACCTGAAGATTTTCTTTGTGGCAAAGAGA
CAATTCCTCAGCATTCAGAATACAACCTGCATGACATCAGCACGAATTTCAAATAAAAAA
CAGACAAAAGCCACTAAAACTCTTGTTGTGATCATGGGAGCGTTTCTTTTATGCTGGGCT
CCATTTTTTCTCTGTAATGTCATTGATCCTTTCATCAGCTACTCAACACCAGCTGCATTG
ATGAAAACATTTGGATGGGTGGGCTTTATTAATTCTACACTAAACCCTCTCATTTATGCG
TTTTTTTACACCTGGCTCAGAAAGGCCTTTTGCCTGTTTGCTTCAGGGAAGGTCTTTAAA
GAGGATATGTCAAACATTACCCTTTTCACTGAA
>TETRENSTNIP7869
ATGGTTTTAGGGGTCATGGTCATTGTGACTGTGTGTGGAAACCTTTTGGTCACTGTTTCT
ATCATCTATTTTAAGCAGCTCCACACTCCAACCAACTACCTGTTGGTCTCTCTTGCTGTG
TCTGACTTTCTCCTGGGGTTCTTGGTCATGTTACCCAATATGATTCAGTTGGTGGAGACT
TGCTGGTATTTTGGGGACATCTTGTGCAAAGTCTATATGAGTTCTGATGTGATGTTGTGC
ACAGCATCCATAATTAATCTGTCTTTAATATCAATTGATCGATACAATGCGGTGATTCAT
CCTCTGCTGTATAGAAGAAAAATGTCTGGTCATGTTGTTTTGCTCATGATTTCTTTCAGC
TGGAGTGTTTCAGGTGTTGTAGGTTTTGGACAGATTTTTTTAAGGCTGAACATTTTGGGA
ATTGAAGATTTCTACAACAACTTTGTTGCATGTGAAGGAAGATGTGTTTTGTTACAAAGT
GGTCCGTCAAGTACAGTCTCGTCAGTGCTCTCCTTTTATATTCCAGGAATAATAATGATC
AGTTTATACCTGAAGATTTTCTTTGTGGCAAAGAGACAATTACTTAGCATTCAGAATACA
ACCTGCATGACATCAGCCAGAGATTCAGATAAAAAACAGGCAAAAGCCACTAAAACTCTT
GTTGTGATCATGGGAGTATTTCTTTTATGCTGGGCTCCGTTTTTTCTCTGTAACATCATT
GATCCTTTCATCAGCTACTCAACACCAACTGCATTGTTTGAAACATTTATATGGCTAGGT
TATATTAATTCTACACTAAACCCTCTCATTTATGCATTTTTTTACACCTGGTTTAGAAAG
GCATTTTGCCTGTTTGCTTCAGGAAAGGTCTTTAAAGAGGATATGTCAAACATTTCCCTT
TTTACTGAT

>TETRENSTNIP216
ATGGATAGCAACGGGGACCCTTGGCTCTGCTTCGCCAACCTTAACTCCTCCTGCAAGAGG
CTGAACCGAACCTTCTCTGAAACCGCTGTGCTGAACAGCCTGCTGGCCTCGATCTCCCTG
GTCACCGTCACACTCAACCTTCTCGTCATCGTCTCTATTTCTCACTTTAGGCAGCTGCAC
ACACCCACCAACGCCCTGCTCCTCTCTCTGGCTGTGTCGGATCTGCTGGTGGGCCTGCTG
GTGATGCCGATCGAGGGCCTGCGCTACGTGGAAATGTGCTGGCGGTTAGGAAAGCTGATG
TGTGTTCTGGCTCCTTATGTGTCCTACTCTGTACTTTCTGCGTCTGTTGGCAACATGGTG
CTCATTTCCATAGACCGTTACCTGGCTATCTGTGACCCACTGCTCTATTCCAATAAGGTG
ACTCTGAAAAGAGCCAAAATTGCAGTCTGTGTCTGTTGGGCCGGTTCTGTACTCCACAAC
GGATGTATTCTGATCGGCCACCTCAAACATCCAGAAAGGTACAGTTCTTGTCACGGAGAG
TGCGTGGTTGTGATCGACCACATCTCTGGAACTGTTGATCTGTTTATAACCTTTTTTGCA
CCTTGTACCATCATGGTTGTGATGTACATGAGAGTGTTTGCAGCTGCTGTTGCTCAAATG
CGAGTCATTCGTTTGCAGAACGCCGCCGTCGCTGTGAACACAGCTACCACCGTGAAAAAA
TCAGAGTGGAAAGCAGCCAGAACTCTGGGGATTGTGGTCAGTGTGTTTCTAATGTGTTTT
TGTCCCTATTACTATCCAGCTTTCGCAGGTGAAGACACCTCAAACAACTCATCCTACTTT

GCTGCTTTGGTTTGGATAATGATGATTAATTCCTGTATGAACCCTCTGATTTATGCTCTG
TTCTATCCCTGGTTCAGGAGATCCATCAAACTCATCATCACACTCAGGATACTGCGTTCA
TACTCC

>Chr1-Tetra.TARs.006
ATGAGATATACACTCTGTGTGCTGTTATTGTTTAATAATGATTCAACATGTGTAAATAAGTTGCTTCAAAAACTGAAGGCAGTCAATGTGTTGGTTATTTTTTTTTTTAATTGCAGTTAATTTGC
TTTTAAAGATATCAAGAACGTTTTCTCTCTTCAGGCACTTTCAGACCACCACCAATCTCATCCTGCTCTCCATGGCTGTGTCTGATTTTCTGGTGGGTCTTGCTGTGATGCCGCTGATGATCGT
CACCCTGGATTCCTGTCGAGGCACCAGTACAGTTGTGTGCTATCTTTATCACCTGTTTAGCTTTATTCTCACCTCTGCTTCTGTTGGGAACATGGTGCTGATATCAGTCGATCGTTACGTGGCC
ATCTGTTACCCTCTGCGTTATTCCTCCATCAAACCAAACAGAGTTAAAATCTGTGTGTCTCTGTGTTGGATCAGCTCTGTTATCTACAATTTTATACTTCTGAATGATTACCTTTCACAACTTG
ATTTTACAAGTTCCTGCTACAAAAAATGTATCCTTTACATTGATTATATTTTAGTTATTGTTGATGTAGTTATCACGTTTTGTGTCCCTCTCACTGTGATTATAGTTCTGTACAGCAGAGTGTT
TGTGGTGGCCGTCACACAGGCCCGTGCCATGAGGGCTCAGGTTTCAACCATCAGCTCACAAAGTGTGAGTGCAATGAAATCAGAGATGAGAGCTGCTAGAACACTCGGAATCATCATTCTCTTT
TTCCTGATGTCCTTTTTCCCATATTACATTTCTTCTTTAACAGGGCAAGGTTTAAGTGATGAAGCTTTAACAGGACAACTTTTACTGTTCTTTTGTAATTCTACAATCAATCCTATCATCTATG
CGTTTTTCTACCCCTGGTTTAGAAAGAGTTTAAAAGTCCTTGTCAGTGGCAAATACTGTGGACTGTAA

>Chr1-Tetra.TARs.002
ATGGTGATGTTGCTGGAAGATGGCTGCATCTCTCCGTTCAACACCTCCTGCAGATTCATGTCTGGTTCTTGGAAGTCCACACTCACCAGTGCAGCTCTGTTCTGCCTTT
CTCCGCTGACTGTGGTGCTGAATTTACTGGTTGTTTTCTCTATTTCTCATGTGAGGCACCTTCAGACCACCACCAATCTCATCCTGCTCTCCATGGCTGTGTCTGACCTT
CTGGTGGGCCTTGCTGTGATGCCGCTCATCATCGTCACCCTGGATTCCTGTCAAGGCACCAGTACATTTGAATGCTATCTTTATCAGCTGTTAGGCCTAATTCTCACCTC
TGCTTCTGTTGGGAACATGGTGCTGATATCAGTCGATCGTTACGTGGCCATCTGTTACCCTCTGCGTTATTCCTCCATCAAACCAAACAGAGTTAAACTCTGTGTGTCTC
TGTGCTGGATCTGTTCTGTTATCTACAGTTTTATACTTATGAAAGATAGCCTTTCACTTATTGATTTTTCTGTTACTTGCTACAAAAAATGTATTCTTTTTATAAACTTCATTTT
AGTGATTGTGGATGTAGTTATCACGTTTTATGTTCCTCTTACTGTGATTATAGTTCTGTATAGCAGAGTGTTTGTGGTGGCCGTCACACAGGCCCGTGCCATGAGGGCTC
AGGTTTCAACCATCAGTTCACAAAGTGTGAGTGCAATGAAATCAGAGATGAGAGCTGCTCGAACACTCGGAATCATCGTTCTCTTTTTCCTGATGTCCTTTCTCCCATAT
TACATTTCTTATTTAACGGGGCACAGTACAGATACCGAATCTGTAGCAAACCAACTGATACTGTTCTGTTGTAATTCTACAATAAATCCTATCATCTATGCCTTTTTCTACCC
CTGGTTTAGAAACAATGTAAAAGTCATTATCAGTGGCAAAGTTTTTGGACTGTAA

Cichlid TAAR sequences

>contig007512-TilTARs.A024
MMEETELCFPKLLNISCRRPKRPHFEIMLTYILLSFISLLTVILNLLVIISISHFRQLHTPTNFLLLSLAVADFFVGLLMFFQIVLIDGCWFLGDIMCTLYQYLAFIITSASIGTMVII
SADRYLAICYPLHYSTKITQQRVKICICLCWVFSVIFQSLIVKDNLKQPGKYNSCIGECVFVVNYIAGLFDLLFSFIVPITVIIVLYLRVFAVAVSQAHAMRCQHAVTHQRAVTV
TVTKSELKAARTLGVVVVVFLICMCPYYCVALTGQDNFLNASSAAFVICLVYFNSCVNPIIYVFFYPWFRKSIKLIATLQILQPDSHETNMHx
>contig007520-TilTARs.A025
MEMFEETELCFPQLLNFSCRKPMQPHSVSILIYIALSSISLLTVTLNLLVIISISHFKKLHTPTNLLLLSLAVSDCLVGLLMLFQIMIIDGCWFLGEFMCSMYFLLDYIITSASVG
TMVLISIDRYVAICYPLHYSTKVTPKRTKACVYLCWICSSVFQCLVLKDNLVQPGRYNSCYGECVVVVGHAFGVADLLLSFIGPVIVIVVLYLNVFVVAMTQARALRSHIAAL
THEGSVSTNVKKSEMKAVRTISVVIIVFLICLCPYFCVTLSGQDAMLSASSVAFVMCLFYLNSCLNPLIYALFYPWFRKSVKQIVTLKILKSGSCDTNIMx
>contig007524-TilTARs.A026
MKTFEETELCFPQLLNSSCRKTMRPYTLSILIYITLSSISLITVTLNLLVIISISHFEKLHTPTNLLLLSLAVSDCLVGLLMLFQIMIIDGCWFLGEFMCSMYFLLDYIITSASIGTM
VLISIDRYVAICYPLHYSTKVTPKRTKACVYLCWICSSVFQCLVLKDNLVQPGRYNSCYGECVVVVGHAFGVADLLLSFIGPVIVIVVLYLNVFVVAMTQARALRSHIAALTHE
GSVSTNVKKSEMKAVRTISVVIIVFLICLCPYFCVTLSGQDAMLSASSVAFVMCLFYLNSCLNPLIYALFYPWFRKSVKQIVTLKILKSGSCDTNIMx
>contig022324-TilTARs.A027
MEIQMHLEAEFCFPELLNSSCRKPTLHWSKTVLLNVGLSFISVITAALNLLIIISVSHFRQLHTPSNILLLSLAVSDFFVGFLLMPVEIFRSTACWVFGDLMCSLYIYLSGILMN
ASFEIIVFISVDRYVAICDPLHYPTRITVARVKLSVCLCWFYAIFYMSLYTKDVLIKPGRYASCYGECVFVIEDITGTVDLVLCFFVPVIIIIVLYTRVFVVAVSQARAMRSHVTAV
TLERSLNQTNKSELKAARNLGVLVIVFLASICPFYFYSLVDGSVLNASSATFLIIVYFNSFLNPLIYTLFYPWFRNAVKLIITLQIFKHNSSEANILx
>contig022330-TilTARs.A028
MEIQKEAELCFPQLLNISCRKPTLHWSKAVLLNIVLSCISLLTATLNLLVIISVSYFRKLHTPSNILLLSLAVSDFLMGLLLIPAEILRSMTCWVLGDLMCSVFFLTVNITCASI
GNIVLISIDRHVAICDPLHYSTRITVPRVKLSVCLCWFYSTFYSSLNTQEMLIVPGRYNSCYGECVLVISDFAGTLDLILFFILPVTVIIVLYMRVFVVAVSQARAMRSHVTAVTL
QRSPNQTNKSELKAARTLGVLVVVFLACYCPFYCYSLADENAVNDPAASFVVFVFYFNSCINPLMYALFYPWFRNAVKLIITLQIFKHNTCEANILx
>contig022334-TilTARs.A029
MEIQKEAELCFPQLLNISCRKPTLHWSKAVLLNIVLSCISLLTATLNLLVIISVSYFRKLHTPSNILLLSLAVSDFLMGLLLIPAEILRSMTCWVLGDLMCSVFFLTVNITCASI
GNIVLISIDRHVAICDPLHYPTRMTVPKIKLSVCLCWFYSTFYSSLNTKKILIEPGRYNSCYGECVLVSSNIAGTLDLILFFILPVTVIIVLYMRVFVVAVSQARAMRSHVTAVTL
QRSPNQTNKSELKAARTLGVLVVVFLACYCPFYCYSLADENAVNDPAASFVVFVFYFNSCINPLMYALFYPWFRNAVKLIITLQIFKHNTCEANILx
>contig022337-TilTARs.A031
MMEIEKGAELCFPQLLNSSCRKPTLHWSRALLLNIVLSCISLITAALNLLVIISVSYFRQLHTPSNIVLLSLAVSDFLIGLLKMPFEIIRNTGCWVLGDLMCSVYIFLTISLLCASI
LNIVLISIDRYVAICDPLHYPTRLTVARVKLSVCLCWFYSAFYSSLYAKNVLIEPGRYNFCFGECVFFSSNIAVVVDLVLFFIVPVSVIIVLYMRVFVVAVSQARAMRSHVTSVT
LQRSANQANKSELKAARTLGVLVVVFLATFCPFYCYSLVEENALNDPSTSTLITVFYLSNSCLNPLIYALFHPWFRNAVKLIITLQIFKCDTSEANILx
>contig022341-TilTARs.A032
MEIQKGVELCFPQLLNTSCRKPALHWSKAVLLNIVLSCVSLITAALNLLVIISVSYFRKLHTPTNILLLSLAVSDFLVGLLLMPFEIFRNMACWVLGDLMCSVSWYLTGNIAFA
STGNIVLISIDRYVAICHPLHYPTRITVARVKLSVSLFWLFAIFYSSLYMKDVLINPGRYNSCYGECVFIINDIAGIFDMVLSFFVPVPVIVILYMRVFVVAVSQARAMRSHVTAV
TLQRSLNQTNKSELKAARTLGILVVVFLICYCPFFCFSFGVEDMVNNPSTFFVIMVFYFNSCLNPLIYALFYPWFRNAVKLIITLQIFKHETSEANILx
>contig022343-TilTARs.A033
MEIEKGAELCFPQLLNSSCRKPTLHWSRALLLYIVLSCISLITAALNLLVIISVSYFRQLHTPSNIVLLSLAVSDFLIGLLMMPFEIIRNTGCWVLGDLMCSVYIFLTISLVCASIL
NIVLISIDRYVAICDPLHYPTRLTVARVKLSVCLCWFYSTFYSSLYAKNVLIEPGRYNFCFGECVFFSSNIAVVVDLVLFFIVPVSVIVVLYMRVFVVAVSQAHAMRSHVTSVTL
QRSANQANKSELKAARTLGVLVVVFLATFCPYYCYSLVDENAVNDPSASFVIIIFYINSCLNPLIYALFYPWFRNAVKLIITLQIFKCDTSEANILx
>contig022345-TilTARs.A034
MEMQKGTELCFPQLLNNSCRKPIRHWSKAVLLYIVLFCISLITAALNLLVIISISHFKQLHTPSNILLLSLAVSDFLVGLLLMPLEIFRSTACWVLGDLMCSVYIYMTVNITCASI
GNIVLISVDRYVAICDPLHYPTRITVARIKLSICFCWFYTIFYSSLYTKDILIKPGRYNSCCGECVLVINDIVGIVDLVLTFIVPVAVIIVLYMRVFVAAVSQARAMRSHVTAVTLQR
PLNQTNKSELKAARTLGVLVVVFLACYFPFYCYASVVEDMVNNPSALFVFMVFYFNSCLNPLIYALFYPWFRNAVKLIVTLQFLKHGTCEANILx
>contig022349-TilTARs.A035
MEIQKGAELCFPQLLNSSCRNPTLHWSRSLLLNIVLSCFSPITAALNLLVIISVSHFRQLHTPTNILLLSLAVSDFFVGLLLMPLEIFRNTTCWILGDIMCSVYWYLNFSFICV
TIGNILLISVDRYVAICYPLHYPNRITVARVKRSVCLCWFYAIFYCSFYTKDILIQPGRYNSCYGECVFFIDDMAGIIDLVLFFLIPVMVIIVLYTRVFVVAVSQARAMRSHVTAVT
LQRSANQANKSELKAARTLGVLVVVFLLCYCPSYFFTFFAEGMLNDPATVFFITVIYFNSCLNPLIYALFYSWFRNAVKRIITLQIVTHDTNEANILx
>contig022353-TilTARs.A036
MEIPKGVELCFPQLLNSSCRKPTLHWSKAVLLNIVLSCISLLTAALNLLVIISVSHFRQLHTPSNILLLSLAVSDFFVGLLLLPLEIFRNTSCWVLGDLMCSAYWYLTSNIICASI
GNIVLISVDRYVAICDPLHYPTRITLPKVKLGVCLCWFYAIFYSSLYTKDVMIEPGRYNSCFGECVFFSSNIAIVVDLILSFFGPVTVIIVLYMRVFVVAVSQARAMRSHVTSVT
LQRPLNQTNKSELKAARSLGVLVVVFLACFCPLYCYSLVDENAVNDPSASFVVIIFYINSCLNPLIYALFYPWFRNAVKLIISLEIFKYDTSGANILx
>contig022354-TilTARs.A037
MEIQEGPELCFPQLLNDSCRKQTLHYSKTMLLYIVMSCISLITTALNLLVIISVSHFRQLHTPSNILLLSLAVSDFLVGLLLMPLEILRSTTCWVLGDFMCFLYWYLTGNITCV
SIGNIVLISVDRYVAICDPLHYSTRITVAKVKLSVCLCWFYAIFSISLYAKDYLIESGRYNSCYGECAFVINDIARTIDLVLSFIFPITVIIVLYMRVFVVAVSQARAMRSHVTAVTL
QCSLNQANKSELKAARTLGILVVVFLLCFCPYYCVSLIREDFSSVAPIVISLFFSNSCLNPVIYALFYPWFRKALKLIVTLQILHSGSCEVSILx
>contig022354-TilTARs.A038
MEMQKGAELCFPQLLNSSCRKPTLHWSKAVLLNIVLSCISLLTAALNLLVIISVSYFRQLHTPTNILLLSLAVSDFLTGLLMLPLEIIRNTACWVLGDLMCSIYTYLTAGLFCAS
TGNIVLISVDRYVAICHPLHYPTRITVARVKLCVCLCWFCSTFYSSLLTKDVLIKPARYNSCYGECVFVINDIAGFIDLVLSFIVPVSVIIVLYMRVFVVAVSQARAMRSHVTAVT
LHHSSNQANKSELKAARTLGVLVVVFLACYLPFYCYSLIEVNVINDSSTFFLIIVFYFNSCLNPLIYALFYSWFRNAVKLIITLQIFKHDTSEANMFx
>contig022355-TilTARs.A039
MEIQKENKLCFPQLLNSSCRKPTLHWSKAVLLNTVLSCISLLTATLNLLVIISVSYFRKLHTPSNILLLSLAVSDFLVGVLLMPLGILRNTSCWVLGDIICSLYWYLTSNIVCASI
GNIVLISVDRYVAICDPLHYPSRITLVKVKLSVCLCWFYAIFYCSLYTKDILIEPGRDNSCYGECVFVINDIAVVVDLVFSFIVPVSVIVVLYMRVFVAAVSQARAMRSHVTSVT
LQRSLNQTNKSELKAARTLGVLVVVFLACFCPLYYFSLVDENAINDPSASFVVIIFYFNSCINPLIYALFYPWFRNAVKLIITLQIFKYNISEANILx
>contig022356-TilTARs.A040
MEIQKGAELCFPQFLNNSCRKPTLRSSKAVLLNIVFSCISLLTAALNLLVIISVSYFRKLHTPSNILLLSLAVSDFLVGLLLMPLEIFRSTTCWVLGDVMCSVYWYLTGNITCA
SIGNVVLISVDRYVAICDPLYYRTRITVERTKLSVCLCWFYAIFYWSLYIKNILIEPGRYNSCYGECMFVSSDIAGIIDLVLSFIVPVSVIIVLYMRVFVVAVSQARAMRSHVTAVT
LQHSNNQANKSELKAARTLGVLVAVFLSCYSPFYCYYLAEENVVNDPSASTVIIIFYFNSCINPLIYALFYPWFRNAVKLIITLQIFKHNTCEANILx
>contig022357-TilTARs.A041
MVIQKGAELCFPQLLNSSCRKPTLHWSKAVFLNIMLPCLSLTTAALNLLVIISVSHFRQLHTPTNILLLSLAVSDFLTGFLILPLEIIRNMACWVLGDLMCSVYTYLITSLFCAS
TGIIVLISVDRYVAICDPLHYPTRMTVVRSKLSVCLCWFYSTFYSSLQTKDVLINPGRYNSCDGECVFLISNIAGTIDLVLSFIFPVSVIIILYMRVFVVAVSQARATRSHVTAVT
LQSSLNQTNKSELKAARTLGILVVVFLACYCPFYCYFLVKENVVNDSATFFVIILYHVNSCLNPLIYALFYPWFRNAVKLIITFQIFTHDTSGANILx
>contig022362-TilTARs.A042
MEIQKGAELCFQQLLNNSCRKLTLNWSKTVLLYIVLFCISLITVALNLLVIISVSHFRQLHTPTNILLLSLAVSDFLVGLLLIPLEILRSTTCWVFGDVICSFYFYLTGNITCASTG
IIVLISVDRYVAICDPLHYPTRMTVARVKLSVCLCWFYAIFYVTLYLKDFLKPGRYNSCYGECILVINDTAGTADLVLSFFVPITVIIVLYMRIFVVAVSQARAMRSHVTAVAKSK
SELKAARTLGVLVVVFLASYCPFYCYTLVEENVISDSSAFFVVIAFYLNSFLNPMIYALFYPWFKKSVNLIITLQIFKRHSSDSNILx
>contig022363-TilTARs.A043
MEIQKGAELCFQQLLNNSCRKPTLHWSKAVLLYIVLFCISLITVALNLLVIISVSHFRQLHTPTNILLLSLAVSDFLVGLLLIPLEILRSTTCWVFGDVMCSVYWYLTGNITCASI
GNIVLISVDRYVAICDPLHYPTRMTVVRIKHWICLCWFYAIFYDTLYLKDLLIKPGRYNSCYGECVLVINDIAGTADLVGGSFGSYHRTSPWx
>contig022363-TilTARs.A044
MEIKKGDELCFVELNSSCKKPTLHWSKAVLLNIVLSCISLITAVLNLLVIISVSHFRQLHTPTNILLLSLAVSDFLVGFLLMPLEIFRSTTCWVFGDVMCSVYWYLTGNITCASI
GNIVLISVDRYVAICDPLHYPTRMTVVRIKHWICLCWFYAIFYDTLYLKDLLIKPGRYNSCYGECVLVINDIAGTADLVLSFCVPISVIIVLYMRVFLVAVSQARAMRSHVTTVTL
QRSLNQTNKSELKAARTLGILVVVFLLCFCPYYCVSLVRDRNSSVAPNLMSVFFLNSCLNPLIYTMFYPWFRKAVKLIVTLQILQTGSCEVSILx
>contig022365-TilTARs.A045
MEKGAELCFPQLNSSCRKPTLHWSKAVLLTIVLFCISLITVTLNILVIISVSHFRQLHTPSNILLPSLAVSDLLVGLLLMPLEIFRNTSCWVFGDLICSVYWYLSGNITSTSTGN
IVLISVDRYVAICDPLHYPTRMTVVRVKLSVCLCWFYYIFYISFYLKDILIEPGRYNSCKGECVLVINDIAGIIDLVIAFIVPVSVIIVLYMRVFVVAVSQARAMRSCVRAVTLQHS

LNQANKSELKAARTLGILVVVFLASFCPFYCYFFVGENVVSDSSAFFVIIAFYFNSFLNPMIYALFYPWFRNAVKLIITLQIFKRHSSDTNILx
>contig022368-TilTARs.A046
MEIQKGAELCFQQLLNNSCRKLTLHWSKAVLLYIVLFSISLITVALNLLVIISVSHFRQLHTPTNILLLSLAVSDFLVGLLLIPLEILRSTTCWVFGDVICSVYWYLTSNITCAST
GIIVLISVDRYVAICDPLHYPTRMTVARVKLSVCLCWFYAIFYVTLYLKDFLIKPGSYNSCYGECVLVINDTAGTADLVLSFFVPIIVITVLYMRVFVVAVSQARAMRSYVTAVTL
QRSLNQTNKSELKAARTLGVLVVVFLASYCPFYCYTLVEENVVSDSSAFFVVIAFYFNSFLNPMIYAFFYPWFRKSVKLIITLQIFKRHSSDSNILx
>contig022368-TilTARs.A047
MEKGAELCFPQRPNSSCGKPTLHWSKAVLLYSVLFCISLITVALNLLVIISVSHFRQLHTPSNILLLSLAVSDLLVGLLLMPLEILRSTTCWVFGDVICSVYWFLSGNIICASIG
NIVLISADRYVAICDPLHYPTRMTVVRVKLNVSLCWFYYVFYISLYLKDILIKPGRYNSCYGECVPVINDIAGIIDLVLTFIVPVSVIIVLYMRVFVAAVSQARAMRSHVTAITFQR
SLNQANKSELKAARTLGVLVIVFLACYCPFFCYTLVEEKVVSDSFVFFLIIAVYFNSFLNPLIYAFFYPWFRNAVKLIITLQIFKRHSSDANILx
>contig022375-TilTARs.A048
MEMQKGTELCFPDFLNTSCRKPTLHWSKAVLLNIVLSCISLITSALNLLVIISVSHFRQLHTPTNILLLSLAVSDFLVGLLLMPLEIFRSTACWVLGDLMCSVYWCLTGNIICA
SIGNIVLISIDRYVAICDPLHYPTRMTVARVRVCVCLCWFFAIFYSSLYTTDFLIDPGRYNSCYGECVFVISAIAGIFDLVVSFFVSVTVIIVLYMRVFVVAVSQARAMRSHVTTV
TLQRSLNQTNKSELKAARTLGILVVVFLLCFCPYYCVSLVRDENSSIASTVISVFFLNSCLNPLIYAMFYPWFRKAVKLTVTLQILRTGSCEVNILx
>contig022377-TilTARs.A050
MEKGAELCFPQLLNSSCRKPTLHWSKAVLLNIVLSCISLITAGLNLLVIISVSHFRQLHTPSNILLLSLAVSDFLVGLLLIPAEIFRITVCWVFGESMCSLYTYLGYIVVTSSISNI
VLISVDRYVAICDPLHYPSRITVARIKLSVCLCWFYSAFYSTLCTKNILIKPGRYNSCYGECVFITSDIAGILDLVLSFIVPVSIIIVLYIRVFVVAVSQARAMRSHVTAATLQSSLN
QTNKSELKAARTLGVLVVVFLASFCPFYCYFLVVEDIVSDSSASIVVIVYYFNSCLNPLIYALFYPWFRNAVKVIITFQIFKHDSSEVNILx
>contig022378-TilTARs.A051
MMENKDSICFPELFNSSCRRRALHWSETVPLNILLCSISAITIALNLLVIISVSYFRQLHTPTNILLLSLAVSDFLVGFLLLPGEIFLGIACWAFGNLLCSLFNYVSFIITSASVG
NMVLISVDRYVAICYPLHYSTRITVTRVKRSVCLCWLCCVLYSSALLKDELIQPGGHNSCYGECVFVINFIAGTVDLLLTFIVPISVIVVLYMRVFVVAVSQARAMRSHVTAVT
LQLSVNLTTKKSELKAGRTLGVLIVVFLMCFCPYYCASFAVEDSLSESSTSLVRYLFYFNSCLNPVIYALFYPWFRKAIKHIVMLQIFHTGSREANILx
>contig022379-TilTARs.A052
MDTQDVAELCFPQLFNTSCKKPITPLSEFVFLHVVLSSISLLTVTLNLLVIISVSHYRQLHTPTNILLLSLAVSDFLVGLLLMPGEILRNTACWFLGDLTCSMYNYMSFIVTST
SVGDMVLISIDRYLAICDPLHYPTRITDRRVKLSICLCWLSSVFYSSLFVKDDLTQPGKHNSCYGECTIVVDLITGIIDLLLTFFVPVTVIVVLYLRVFVVAVSQARAMRSHVTA
TALQLSVTLTTKKSELKAARTLGVLVVVFLLCFCPYYYCVTLARDDPLNSSSVSFVLYLFYFNSCLNPLIYALFYPWFRKAVKLIISLHILQPGSCEISILx
>contig022382-TilTARs.A053
MGTQEKSELCFPQLFNMSCKKPAIARSKAVFLHIVLSSVSFLTVALNLLVIISVSHFRQLHTPTNILLLSLAVSDFLVGLLLSPAEILRSTACWFLGQLTCLMYIFVSLTVTSAS
VGIMVLISADRYVAICDPLNYPIRITDRRVQLCVCLCWLCSIAFSCFLVRDDLHQTRKQNSCYGKCVVVVQYIAGVVDLILTFIVPVSVIIVLYMRVFVVAVSQARAMRSHVTA
VTSHLPVTLTKKSELKAARTLGVLVLVFLMCFCPYYCVSLVGEEFINSSSASFVAYLFGLNSCLNPLIYAMFYPWFRKAVKLVVTLQILQPGSCEVSILx
>contig022383-TilTARs.A054
METQDEAELCFPQLFNISCKKPKTSLSQVLLPYVVVYSVSLLTVALNLLVIVSVSHFRQLHTPTNILLLSLAVSDFLIGLLMMPAKILRDTACWFLGQLTCLIYNYICFIITSAS
VGIMVLISVDRYVAICDPLHYPTRITEKRVKLCVCLCWLCSVFYNILFIKDDLLQREQHTSCYGECVFVIDYIVGTTDIVVTFIAPVTVIVILYMRVFVVAVSQARAMRSHVTAV
TLQLSVTLTAKKSELKAARTLGVLVLVFLLCFCPYYIVSLLGNELFNSSSASIVIYLYYFNSCLNPLIYAMFYPWFRKAVKLVVTLQILQPGSCEVSILx
>contig022390-TilTARs.A055
MDTQEGAELCFPQLFNISCKKPTTPLSQVLLLYIVLSSMSLLTVTLNLLVIIAVSHFRRNTACWFLGQLTCSLYNYVSYIVASASVGNMVLISVDRYVAICDPLHYPSRITDKR
VKLCVCLCWLCSVFYSYVILIDDLSQPGKHNSCYGKCIIFIEFIAGFVDLVLSFIIPLTVIVVLYMRVFVVAVSQARAMRSHVTAVTLQLSVTLTAKKSELKAARTLGVLVLVFLI
CFCPYYIVSLFGDEFLNSSSASIVIYLYYFNSCVNPLIYAMFYPWFRKAVKLIVTLQILQPDSCEVSILX
>contig022390-TilTARs.A056
MDTQDGLELCFPQLFNISCKKPTTPLSQVLLPYIVVFSMSLLTVTLNLLVIIAVSHFRQLHTPTNILLLSLAVSDFLIGLLLMPANIIRKKVCWFLGQLTCLVYTYVSFIITSASV
GIMVLISVDRYVAICDPLHYPTRITHGRVKLCVCLCWLCSVIYNILLIKDELVKPGQHISCYGECKILVDYIAGTTDIVLTFIIPVTVIIVLYMRVFVVAVSQARAMRSHVTAVTMQ
LSVTLTTKKSELKAARTLGVLVLVFLICFCPHYCIFVFGGKLLSSSSATIVIYLYYFNSCVNPLIYAMFYPWFRKAVKLTVTLQILQPGSCEVSILx
>contig045088-TilTARs.A057
MEGVEFCFPHLLNSSCRKALRPVSVSMLIYMIISSISVLTATLNLLVIVSISHFKQLHTPTNFLLLSLAVSDFFVGLYLLFYIMFIDGCWYFGEFMCILYYVIGTINTSSSIGTMV
LISVDRYVAICDPLHYPIKVTAKRVQTCVSLCWSFSALAGSYLLKDNLKQQSRFNSCVGECVVHINFIEYVADLALNFLLPITVIIVLYLRIFVVVVSQVRAMRSHTAGVTYQC
SRKGNPKKSEMKAARTLGIVVIAFLSCTLPFYCVTLTGQNAFLNGSSSAFVLCLFYFNSCLNPIVYALFYPWFRKSIKFIVTFQILKSGSRNANIVKVTEx
>contig056134-TilTARs.A058
MEETELCFPKLLNTSCMRLRRPHFEIMLTYILLSSIALLTVTLNLFVIISISHFRQLHTRNNVLLLSLSAADFGVGLVMFFQIVLIDGCWFLGDIMCTLYQYLAYVITSASIGTM
VIISVDRYLAICYPLHYSTKITQQRVKIVVCLCWICSVIFQSLILMDNLKQPGRYNSCIGECVFVINYIAGLVDVTFSFIVPITVIVVLYLRVFVVAVSQAHAMRSQLAVTHQQSV
TVTAKKSELKAAWTLGIVVVVFLICMCPYYCVALTVKDNALSASSAAFVIYLFFFNSCLNPILYVFFYSWFRKSIKVIVTLQILQPDSSKANMLx
>contig022320-TilTARs.A059
MEIQKAAELCFPQLLNSSCRKPTLHWSKAVLLNIVLFCISVITAALNLLVIISVSHFRKLHTPSNILLVSLAVSDFLVGLLLMPLEIFRNTACWVLGDVMCSVYWYLTSNITCA
SIGNIVLISVDRYVAICDPLHYPTRITVVRVKLSVYLCWFFSIFYWSLYMKDILVEPGRYNSCYGECVLIINDIAGIVDLALSFIVPVTVIIVLYMRVFVVAVSQARAMRSHVTAV
TLQCSLNQANKSELKAARTLGVLVVVFLACYCPFYCYSLVDKNVVNDSSASFVVLVFYFNSCLNPLIYALFYPWFRKAIKCVITLLIFKHDSSEVNVIx
>contig007518-TilTARs.A019
MKTIDRAELCFSQLNSSCRKTMHPRSLSVLFYVILSSISVLTVALNLLVIISISHFRQLHTPTNLLLFSLAVSDCFVGLLTVFQILVIDGCWYLGEQLCILYYIFDYVVTNASIGT
MVLISVDRYVAICDPLYYPSKVTLKRVRTSVSLCWIFSLFCVFVLLKDNLGQPGSYNSCSGECVVLVYYVTGIVDILLSFIGPVIVIIALYLKVFMVVVTQARSMRSRIEAVAVQ
GSVTVTIKKSEIKAARVLGIVVVVFLACLLPYFCIGLTGDKTLLNASTATVFITLFYFNSCVNPLVYAFFYPWFRRTIKLIVTLKILNSRSCKANLLISx

>contig039639-TilTAR.A001
MDGSGGPPLCFPNLNSSCRRLLRPTSQAALLYTLMASVSLLTVVLNLLVVVSISHFKQLHTPTNTLLLSLAMSDLLVGLLVMPIEGLRYIETCWLLGRLMCALSPYLSYCLIS
VSLGSMVLISVDRYIAICDPLLYSSKITVNRVKLSVCVCWACSLLYNGCILMEHLGWPDRFSSCHGECVVFISRTSGTVDFFLSFVGPCGVMFVLYMRVFVVAVSQVRAIQ
SQVAVRAAPAAKKSELKAARTLGIVIVVFLMCSCPYYYPSFAGDDTSMSLPYYTLLFWMVLTNSCVNPVIYVLFYLWFRRAIRFIVTLRILQPHSREVNILx
>contig039640-TilTAR.A002
MDGSGGPPLCFPNLNSSCRRLLRPTSQAALLYTLLASVSLLTVVLNLLVVISISHFKQLHTPTNALLLSLAVSDLLLGLLVMPIEGLRYIETCWLLGRLMCALSPYLSYCFLSF
SLDSMVLISVDRYIAICDPLLYSSKITVNRVKLSVCFCWVCSLLYNGCILMEHLGSPDRFSSCHGECVVFISYTSGTVDLFLSFVAPFAVMFVLYMRVFVVAVSQVRAIQSQV
AVRAAPAAKTSELKAARTLGILIAVFLMCFCPYYYPSFTGDDTSTSLPYYAVLFWIMLINACVNPVIYVLFYPWFRRAIRFIVTLRILQPHSREVNILx
>contig039640-TilTAR.A003
MDGSGGPPLCFPNLNSSCRRLLRPTSQAALLYTLLASVSLLTVVLNLLVVISISHFKQLHTPTNALLLSLAVSDLLVGLLVMPIEGLRYIETCWLLGRLMCALSPYLSYCLISV
SLDSMVLISVDRYIAICDPLLYSSKITVNRVKLSVCVCWVCSLLYNGCILMEHLRWPKRFSSCHGECVVFISYTSGTIDLFLSFVGPCALMFVLYMRVFVVAVSQVRIIRSQV
AVRAAPAAKKSEMKAARTLGIVIAVFLMCFCPYYYPSLAGVDTSTSLPYYALFSWIMLTNSCVNPVIYALFYPWFRRAIRLIVTLRILQPHSREVNILx
>contig039642-TilTAR.A004
MDGSGGPPLCFPNLNSSCRRLLRPTSQAALLYTLLASVSLLTVVLNLLVVVSISHFKQLHTPTNALLLSLAVSDLLVGLLVMPIEGLRYIETCWLLGRLMCALSPYLSYCLIS
VSLGSMVLISVDRYIAICDPLLYSSKITVNRVKLSVCVCWACSLLYNGCILMEHLGWPDRLSSCHGECVVFISRTSGTVDFFFSFLGPCGVMFVLYMRVFVVAVSQVRAIQ
SQVAVRAAPAAKISELKAARTLGILIAVFVMCFCPYYYPSLAGVDTFLSLPSYAMVCWIMLTNACVNPVIYVLFYPWFRRAIRFIVTLRILQPHSREVNILx
>contig023443-TilTAR.A005
MMDSQDRTELCYPELANLSCRGLTRPQPEAVLLYTLFLSISVLTVALNVLVIISISHFRQLHMPTNVLLLSLAISDLLVGLLVMPVETMRFIETCWLLGDLMCALSYIIGFTLTS
ASVGNMVLISIDRYVAICYPLQYPTKITHSRAELSVTLCWACSLLYNGLILKEHLRQPDRHNTCHSQCLVVINYVSGAIDLVFTFIGPCSVIIILYMRVFVVAVSQAHAMRSHIT
AAAVRITAKKSEKKAARTLGVVIFVFLLSFCPYYYPSLAGQDISNSASSWAIVSWMLYFNSCLNPLIYALFYPWFRKAIWFIVSLKILEKGSSQANILx
>contig037879-TilTAR.B060
MHPCYESENATYTFTNNPSIACVILYIFLGSLSVVTIFGNLLVTIAIIYFKQLHTPTNYLILSLAVADLLVGVLVFPFSMAFTVTLCLDHEDLFCKIRDSFDVSLCTSSILNLCCISI
ERYYAVCQPLNYRSKINTQVTMFMILVSWGISVLIGICIIIAGFSQGMCEEVCSVDVVIANTMGPVFSFYLPAIIMLCIYLKIFLVAQKQVSTIQNAICTKSGATISKLERKATKTL
SIVMGVFLLCLSPYFICVVFQPLAHNPPPIPVIETLNWLTLSNSMLNPLIYAFFYSWFRSAYRLIVSGTILHCNLANSKLFx
>contig037889-TilTAR.B061
MTIIVNDLHPCYVIQDSKYMLTNSPSIICVFLYALLALLSVITICGNLLVIISVIYFKQLHTPTNSLILSLAVADLLIGIIAFPLSMAFSLSSCLYHEGLFCKVRGTFDISLSTCSILNL
CCISIDRYHAVCQPLTYQTKISHRAVVFMILMSWGVSGIIGISVTIAGLNNEKYEESFLIDVLMESTVGPMLSFYLPVIIMLCIYLKIFFVARRQARSIQNTTKCGQTASMMERK
ATKTLAIVLGVFILCWSPFFLCITFPPFTSDYQYQLQVPVPVIETLNWLTLANSMLNPFIYAFFYSWFRSAIRMIICGKIFQGDFANTKLTx
>contig002574-TilTAR.B062
MTNIVNDLHPCYVIQDSKYMLTNSPSIICVFLYAFLALLSVITICGNLLVIISVIYFKQLHTPTNSLILSLAVADLLVGIIAFPLSMAFSLSSCLYHEGLFCKVRGTFDISLSTCSILN

LCCISVDRYHAVCQPLTYQTKISHRVVVFMILMSWGVSGIIGISVTIAGFNNEKCEESCLIDVLMESTVGPMLSFYLPVTIMLCIYLKIFFVALKQASSIQNTTKCGQTASIMER
KATKTLAIVLGVFIFCWSPCFLCITFPPFTSDSVPVPVIETLNWLALANSMLNPFIYAFFYRWFRSAIRMIICGKIFQRDFANTNMTx
>contig037900-TilTAR.B063
MAPDVPVNRTDADDLHPCYKIDKGSYIVKSNPSTLCVLLHIFLGSLSLVTVCGNLLVIISIIYFKQLHTLTNSLVLSLAVADLVVGVLVFPLSMRFTVSVCLFYQDLFCKVRDSF
DVTLSTSSILNLCCISIDRYYAVCRPLTYRSKINVHVVVIMILVSWSVSILVAVGFLIAGLNHEKCKEGWISELLLANILGPVLSFYLPVIIMLCIYLKIFLVAEKHARSIQNTKCGA
TVVSKMEKKATKTLATVMGVFVMCWLPFFFCVTLQLLGHILIPLPVYETLNWLALCNSMLNPFIYAFFx

>contig035375-NyeTARs.A013
MDTQSRADLCFPQLFNTSCKKPTTPLSQVLLFHILMPSMCLLTVTLNLLVIIAISHFRQLHSHTNILLLSLAAADFLIGLLFMPGEILRNTVCWFLGQLTCSLYNYISFVVTSA
SVGIMVLISVDRYVAICDPLHYPTRITERRVKLCVCLCWLCSVFYCYMIVRDDLSQPGKHNSCFGECIIFVEFIAGFVDLVLSFIIPVTVIVFLYMRVFVVAVSQARAMRSQVT
AVTLQLSVTLTAKKSELKAARTLGVLVLVFLICFCPYYIVSLFGDEFLNSASASIVFYFFLLNSCLNPLIYAMFYPWFRKAVKLIMTLQILQPGSCEVSILx
>contig035376-NyeTARs.A014
MDTQDGAELCFPQLFNISCKKPTTPRSQVLLLYIVVSSMSLLTVTLNLLVIITVSHFRQLHTPTNILLLSLAVTDFLVGLLFMPGEILRNTACWFLGQLTCSLYNYVSYIIASAS
VGNMVLISVDRYVAICDPLHYPTRITERRVKLCVCLCWLCSVFYSYVILIDDLSQPGKHKSCYGRCIIFIEFIAGFVDLVLAFIIPLTVIIVLYMRVFVVAVSQARAMRSQVTAVT
LQLSVTLTATKSELKAARTLGVLVLVFLLCFCSYYIISLFGNELLNSSSASIVIYLYYFNSCLNPLIYAMFYPWFRKAVKLIVTLQILQPGSCEISIMx
>contig035381-NyeTARs.A015
MDTQDGAELCFPQLFNISCKKPTTPLSQVLLLYIVVSSMSLLTVTLNLLVIIAVSHFRQLHSPTNILLLSLAVADFLVGLLFMPGEILRNTACWFLGQLTCSLYNYVSYIITSAS
VGNMVLISVDRYVAICDPLHYPTRITERRVKLCVCLCWLCSVFYCYVILIDDLSQPGKHNSCYGKCIIIIEFIAGFVDLVLSFIIPLTVIIVLYMRVFVVAVSQARAMRSQVTAVTL
QLSVTLTAKKSELKAAKTLGVLVLVFLLCFCSYFIVSLFGNELLNSSSASIVIYLYYFNSCLNPLIYAMFYPWFRKAVKLIVTLQILQPGSCEVIILx

>contig046007-NyeTARs.A017
MQKENKLCFPQLLNSSCRKPTLHWSKDVLLNTVLSCISLLTAALNILVIISVSYFRKLHTPSNILLLSLAVSDFLVGLLLMPLGILRNTGCWVLGDIICSLYWYLTSNIVCASIG
NIVLISVDRYVAICDPLHYPSRITLAKVKLSVCLCWFYAFFYVSLYTKDILLEPGRDNSCYGECVFVINDIAVVVDLVFSFIVPVSVIVVLYIRVFVAAVSQARAMRSHVTSVTL
QRSLNQTNKSELKAARTLGILVVVFLACFCPLYYFSLVDENAINDPAASFVVIIFYFNSCLNPLIYALFYPWFRNAVKLIITLRIFKYNMSEANILx
>contig060105-NyeTARs.A018
MEETELCFPQLFNSSCVRQKHSQIEAVFIYTPLSSISVLTTILNLLVIISIAHFKQLHTPTNLLLLLSLAVSDFFVGLIMACQISLLDGCWFLGDHMCALYSSLDYIVTSASVGT
MVLISADRYVAICDPLHYPTKITIKRVSVSICTCWACSILYNSLIMKDNFKQPGRYNSCSGDCVVVIDYFVGIFDFVLTFVGPVIVIIVLYLRVFVVAVSQARAMRSHITALRLQ
GSETVHAKKSELKAARTLGVLVIAFLICLFPFFCSSMVGQNSFFDIRSVPFERLLFYFNSCLNPLIYTFCYPWFLKSIKLIVTFKIFRHGSSEASVLx
>contig046010-NyeTARs.A019
MQKEAELCFPQLLNSSCRKPTFHWSKAVLLNIVLCCISLLTAALNLLVIISVSYFRQLHTPTNILLLSLAVSDFLTGLLMLPLEIIRNTACWVLGDLMCSVYTYLTAGLFCAST
WNIVLISVDRYVAICHPLHYPTRITVARVKLCVCLCWFCSTFYGSLLTKDVLIKPARYNSCYGECVFVINDIAGFIDLVLSFIFPVSVIIVLYMRVFVVAVSQARSMRSHVTAVAL
QRSSNQANKSELKAARNLGVLVVVFLACYLPFYCYSLIEVNVINDSSTFFLIIVFYFNSCLNPLMYALFYSWFRNAVKLIITLQIFKHDTSEANMFx
>contig046013-NyeTARs.A021
MEIPKGVELCFPQLLNSSCRKPTLHWSKAVLLNTVLCCISLLTAALNLLVIISVSHFRQLHTPSNVLLLSLAVSDFFVGLLLLPLEIFRNTSCWVLGDRMCSAYWYLTSNIVC
ASIGNIVLISVDRYVAICDPLHYPTRITLANVKLSVCLCWFYAFFYSNLYTKDIMIEPGRYNSCFGECVFFGSNIAIVADLILFFFVPVTVIIVLYMRVFVVAVSQARAMRSHVTL
VTLQRSLNQTNKSELKAARTLGILVVVFLACFSPLYCYSLVDENAINDPAASFAVIIFYINSCLNPLIYALFYPWFRNAVKLIITLEIFKYDTSGANILx
>contig062039-NyeTARs.A022
MMENKDRICFPEVFNSSCRRPAFHWSEAVPLNTLLCSISVITIALNLLVIISVSYFRQLHTPTNILLLSLAVSDFLVGLLLLPGEIFLGIACWAFGNLLCSLFNYVSFIITSASVG
NMVLISVDRYVAICYPLHYSTRITVTRVKRSVCLCWLCCVLYSSVLLKDELIQPGRHNSCYGECVFVINFIAGTVDLLLTFIVPISVIVVLYIRVFAVAVSQARAMRSQLSVNLT
TKKSELKAGRTLGVLIVVFLMCFCPYYCVSLAGEDSLSESSTSIVRYLFYFNSCLNPLIYALFYPWFRKAIKHIVMLQIFHTGSHEDNILx
>contig035377-NyeTARs.A023
MDTQDGAELCFPQLFNISCKKPTTPLSQLLLPYIVVSSMSLLTVTLNLLVIIAVSHFRQLNTPTNILLLSLAVSDFFIGLLLMPANIIRKKVCWFLGQLACLVFTYVSFIITSASV
GIMVLISIDRYAAICDPLHYPTRITRERVKLCVCLCWLCSVFYNILFIKNDLLQRERHTSCYGECKILVDYIAGTTDIVLTFIAPVTVIIVLYMRVFVVAVSQARAMRSQVTAVTL
QLSVTLTTKKSELKAARTLGVLVLVFLICFCPHYCIFVFGGKVLNSSSATIVIYLYYFNSCLNPLIYAMFYPWFRKAVKLIVTLQILQPGSCEVSILx
>contig046014-NyeTARs.A024
MEIQKAAELCFPQLLNSSCRKPTLHWSKAVLLNIVLSCISLITAALNLLVIISVSHFRQLHTPSNILLVSLAVSDFLVGLLLMPLEIFRNTACWVLGDVMCSVYWYLTSNITCA
SIGNIVLISVDRYVAICDPLHYPTRITLVKVKLSVCLCWFFSIFYWSLYMKDILVEPGRYNSCYGECVLIINDIAGIVDLALSFIVPVTVIIVLYMRVFVVAVSQARAMRSHVTFVT
LQCSLNQANKSELKAARTLGVLVVVFLACYCPFYCYSLVDKNVVNDSSASFVVLVFYFNSCLNPLIYALFYPWFRKAIRCVITLLIFKHDSSEVNVIx
>contig058002-NyeTARs.A025
MEMFEETQLCFPQLLNFSCRKPIRPHSVSILIYITLSSISLLTVTLNLLVIISISHFKKLHTPTNLLLLSLAVSDCLVGLLILFQIMIIDGCWFLGEFMCSMYFLLDYIITSASVGTM
VLISIDRYVAICYPLHYSTKVTPKRTKACVYLCWICSSVDQCLLLKDNLVQPGRYNSCYGECVVVVGHAFGVADLLLSIIGPVTVIVVLYLNVFVVAMTQARALRSHIAALTH
ERSVSTNVKKSEMKAVRTISVLIIVFLICLCPYYCVTLSGQDAMLSASSVAFVMCLFYLNSCLNPLIYALFYPWFRKSVKQIVTLKILKSGSCDTNIMYTEx
>contig045999-NyeTARs.A026
MEIQMHPDAELCFPELLNSSCRKPTLHWSKTVLLNVGLSSISLITAALNLLIIISVSHFRQLHTPSNIIILSLAVSDFFVCFLLMPVEIFKNTACWVFGDLMCSLYTYLSCILINA
SFEMIILVSVDRYVAICDPLHYPTRITVPRVKLSVFLCWLYAILYNIIYTKHALINPGRYGSCYGECVFVVDDIIGIVDFVVSLIVPVTIIVVLYTRVFVVVVSQARAMRSHVTAVT
LQRPLNQANKSELKAARNLGILVVVFLACYCPFYCYFFLAGNEVNASSASSILIVYYFNSCLNPLMYALFYSWFRNAVKLIITLQILKANSSEINILQRx
>contig060292-NyeTARs.A027
MAETELCFPNLNSSCSRVKRSPSDSVLIYVILTIISLLTVALNLLVIISISHFKQLHTPTNHLLLSLAVSDFFVGLNMCFQSMLIDGCWYLGDLMCVLYYVFDIVATSASVGTM
VLISVDRYVAICDPLHYPTKVTPKRVQTCVLMCWICSLLVVGVLLKDNLDKPGRFNSCFGECVIYVDFAIQVTDLILTILLPITVIVILYVRVFAVAVFQIRAMQPHVAAVTQRGK
VSPKKSELKAARTLGIVIVAFLICLFPYYSVILSGQDTLLDILSVTFFLCLFYFNSCLNPIIYAFFYPWFRKSVKLIVTFQIVKSGSSDASMLx
>contig042499-NyeTARs.A028
MEGVEFCFPHLLNSSCRKALHPVSVSTLIYMIISSISVLTATLNLLVIISISHFKQLHNPTNFLLLSLAVSDFFVGLYLLFYIMFIDGCWYFGDFMCILYYVIATINTSSSIGTMVLI
SVDRYVAICDPLHYPTKVTAKRVQICVSLCWSFSALAGSFLLKDNLKQQSRFNSCVGECVVHINFIEYVADFALNFLLPITVIIVLYLRIFVVVVSQVRAMRSHTAGVTYQCS
RKGNPKKSEMKAARTLGIVVIAFLSCALPFYCVTLTGQNAFLNGSSSAFVLCLFYFNSCLNPIIYALFYPWFRKSVKLIVTFQILKSGSRNANIVKVTEx
>contig056200-NyeTARs.A029
MGTQEKSELCFPQLFNMSCKKPAIARSKAVFLHIVLSSVSFLTVALNLLVIISVSHFRQLHTPTNILLLSLAVSDFLVGLLLSPAEILRSTACWFLGQPTCLMYIFVSLTVTSAS
VGIMVLISADRYVAICDPLNYPIRITDRRVQLCVCLCWLCSIAFSCFLVRDDLHQKRKQNSCYGKCVVVVQYIAGVVDLILTFIVPVTVIIVLYMRVFVVAVSQARSMRSHATA
VRNHLPMTLTKKSELKAARTLGVLVLVFLMCFCPYYCVSLVGEEFINSSSASFVAYLFGLNSCLNPLIYAMFYPWFRKAVKLVVTLQILQPGSSEVSILx

>contig032272-NyeTAR.A004
MDRQDRIELCYPELANLSCRVLTRPQPEAVLLYTLFLSISVLTVALNVLVIISISHFRQLHMPTNVLLLSLAISDLLVGLLVMPVEAMRFIETCWLLGDLMCAFTYIIGFTLTSAS
VGNMVLISIDRYVAICYPLQYPTKITHSRAELSVTLCWACSLLYNGMILKEHLRQPDRHNTCHGQCLVVINYVPGAIDLVFTFIGPCSVIIILYMRVFVVAVSQAHAMRSHITAA
AAGTVKITAKKSEKKAARTLGVVIFVFLMSFCPYYYPSLAGQDISNSASSWAIVSWMLYFNSCLNPLIYAFFYPWFRKAIWFIVSLKILEKGSSQANILx
>contig038663-NyeTAR.A005
MDSSGGPPLCFPNLNSSCRRLLRPTSQTVVLYTLLASISLLTVVLNLLVVISISHFRQLNTSTNTLLLSLAVSDLLVGLLVMPIEGLYYIETCWLLGRLMCALSPYLSYCLISVS
LGSMVLISVDRYIAICDPLLYSSKITVNRVKLSVCVCWACSLLYNGCILMEHIGWPDRFGSCHGECVVFISRALGTIDLFFSFLGPCALMFVLYMRVFVVAVSQVRIIRSQAA
VRAAPAAKKSELKAARTLGILIAVFVMCFCPYYYPSFAGDDTSMSLPYYALFSWIMLTNSCVNPVIYALFYPWFKRAIRLIVTLRILQPHSRDVKILx
>contig052987-NyeTAR.B030
MELFNVTVNTVSFLLCDSQKNKLCVLLYVVLSSFMLLTICGNLLVIISIIYFRYLHTPTNYLILSMAVADLLIGALIFPLSMTVSLKPCLYIYSLLCNLRSTMDVTMGVSSLLNLC
CISVDRYYAVCHPLIYKTKITDCVAMKMGLGSWAVAILCGIFVFLLFFILDECDTSCVFALIAASVVVYYIPTIVLLFMYTKILVVALRQARSIHNTISQNTKSKAVSSTERKATKT
LTIVIGIFLIFWVPLFLSYSFVPLDSFILYVLLEPFNWFAISNSMLNPFIYAFFYTWFRRAFKMIISGKIFQGDVTNIKLHx

>contig061977-BurTARs.A012
MDTQDVAELCFPQLFNTSCKKPLSEFVFLHVVLSSISLLTVTLNLLVIISVSHYRQLHTPTNILLLSLAVSDFLVGLLLMPGEILRNTACWFLGDLTCSMYNYMSLIVTSTSV
GDMVLISIDRYVAICDPLHYPTRITDRRVKLCVCLCWLCSVFYSSLFVKDDLTQPGKHNSCYGECTIVVDLITGTIDLLLTFFVPVTVIVVLYLRVFVVAVSQARAMRSHVTVA
ALQLSVTLTTKKSELKAARTLGVLVVVFLLCFCPYYCVTLARDDLLNSSSVSFLLYLFYFNSCLNPLIYALLYPWFRKAVKLIISLHILQPGSCEISILx
>contig061433-BurTARs.A013
MEIQMHPEAELCFPELPNSSCRKPTLHWSKTVLLNVGLSSISVITAALNLLIIISVYHFRQLHTPSNILLLSLAVSDFFVGFLLLPVEIFKNTACWVFGDLMCSLYIYLSGILMN
ASIEIIVFISVDRYVAICDPLHYPTRITVTRVKLSVCLCWFYAIFYMSLYTKDVLIKPGRYASCYGECVFFIEDITGTVDIVLCFFVPVIIIIVLYTRVFVVAVSQARAMRSHVTAVT
LQRSLNQSNKSELKAARNLGVLVIVFLASICPFYFYSLVDGNVVKASSATFLIIVYFNACLNPLIYTLFYPWFRNAVKLIITLQIFKHNSSEANILx
>contig061091-BurTARs.A014
MEIPKGVELCFPQLLNSSCRKPTLHWSKAVLLNIVLSCISLLTAALNLLVIISVSHFRQLHTPSNILLLSLAVSDFFVGLLLLPLEIFRNTSCWVLGDRMCSAYWYLTSNIICAS
IGNIVLISVDRYVAICDPLHYPSRITLAKVKLSVCLCWFYAFFYSNLYTKNIMIEPGRYNSCYGECVFFSSNIAIVADLILFFFVPVTVIIVLYMRVFVVAVSQARAMRSHVTLVT
LQRSLNQTNKSELKAARTLGILVVVFLACFSPLYCYSLVDENAINNPAASFAVIIFYINSCLNPLIYALFYPWFRNAVKLIITLEIFKYDTSGANILx
>contig060707-BurTARs.A015
MEETELCFPQLFNSSCVRQKHSQIEAVFIYTPLSSISVLTTILNLLVIISIAHFKQLHTPTNLLLLSLAVSDFFVGLIMACQISLLDGCWFLGDHMCALYSSLDYIVTSASVGTM
VLISADRYVAICDPLHYPTKITMKRVSVSICTCWACSILYNSLIMKDNFKQPGRYNSCSGDCVVVIDYFVGIFDFVLTFVGPVIVIIVLYLRVFVVAVSQARAMRSHITALRLQG
SETVHAKKSELKAARTLGVLVIAFLICLFPFFCSSMVGQNSFFDIRSVPFELLLFYFNSCLNPLIYTFCYPWFLKSIKLIVTFKIFRHGSSEASILx
>contig059673-BurTARs.A016
MKTFEEAELCFPQLLNSSCRKTMRPYTFSILIYITLSSISLLTKLHTPTNLLLLSLAVSDCLVGLLILFQIMIIDGCWFLGEFMCSMYFLLDYIITSASIGTMVLISIDRYVAICYPL
HYSTKVTPKRTKACVYLCWICSSVFQCLLLKDNLVQPGRYNSCYGECVVVVGHAFGVADLLLSIIGPVTVIVVLYLNVFVVAMTQARALRSHIAALTHERSVSTNVKKSEM
KAVRTISVLIIVFLICLCPYFGVTLSGQDAMLSASSVAFVMCLFYLNSCLNPLIYALFYPWFRKSVKQIVTLKILKSGSCDTNIMx
>contig057301-BurTARs.A017
MEGVEFCFPHLLNSSCRKALHPVSVSTLIYMIISSISVLTATLNLLVIISISHFKQLHNPTNFLLLSLAVSDFFVGLYLLFYIMFIDGCWYFGEFMCILYYVIGTINTSSSIGTMVL
ISVDRYVAICDPLHYPIKVTAKRVQICVSLCWSFSALAGSFLLKDNLKQQSRFNSCVGECVVHINFIEYVADLALNFLLPITVIIVLYLRIFVVVVSQVRAMRTHTAGVTYQCS
RKGNPKKSEMKAARTLGIVVIAFLSCALPFYCVTLTGQNAFLNGSSSAFVLCLFYFNSCLNPIIYALFYPWFRKSIKLIVTFQILKSGSRNANIVKVTx
>contig057145-BurTARs.A018
MEIQKVPELCFPQFLNNSCRKPTLHWSKAVLLNIVFSCISLLTAALNLLVIISVSHFRKLHTPSNIVLLSLAVSDFLVGLLLMPAEILRSTTCWVLGDIMCSVYWYLTGNIICAS
IGNIVLISVDRYVAICDPLHYRTRITVKRTKLSVCLCWFYAIFYWSLYIKNILIEPGRYNSCYGECMFVSSDIAGIIDLFLSFIVPLSVIIVLYMRVFVAAVSQARAMHSHVTSVTL
QHSSNQAKKSELKAARTLGVLVVVFLSCYSPFYCYYLAEENVVNDPSASTVIIIFYINSCLNPLIYALFYPWFRNAVKLIVTLQIFKHGTCEANILx
>contig056023-BurTARs.A019
MEIQMHPEAGLCFPELLNSSCRKPTLHWSKTVLLNVGLSSISLITAALNLFIIISVSHFRQLHTPSNIIILSLAVSDFFVCFLLMPVEIFKNTACWVFGDLMCSLYTYLSCILINA
SFEMIILVSVDRYVAICDPLHYPTRITVPRVKLSVCLCWLYAILYNIIYTKHALINPGRYGSCYGECVFVVDDIIGIVDFVVSLIVPVTIIVVLYTRVFVVVVSQARAMRSHVTAVT
LQRPLNQANKSELKAARNLGILVVVFLACYCPFYCYFFLAGNEVNASSASSILIVYYFNSCLNPLMYALFYSWFRNAVKLIITLQILKANSSEINILQRx
>contig056021-BurTARs.A020
MEIQKGAELCFPQLLNISCRKPTLHWSKAVLLNIVLSCISLLTAALNLLVIISVSYFRKLHTPSNILLLSLAVSDFLMGLLLMPAEILRSMTCWVLGDLMCSVYFFLTVNLTCAS
IGNIVLISIDRYVAICDPLHYSTRITVARVKLSVCLCWFYSTFYCSLYTQEMLIEPGRYNSCYGECVLVISDFAGMVDLILFFILPVIIIIVLYTRVFVVAVSQARAMRSHVTAVTL
QRSLNQTNKSELKAARTLGVLVVVFLSCYCPFYCYSLTDKNAVNDPAASFVIFIFYFNSCINPLMYALFYPWFRNAVKLIITLQIFKHNSCEANILx
>contig056020-BurTARs.A021
MEIQKENKLCFPQLLNSSCRKPTLHWSKDVLLNTVLSCISLLTAALNLLVIISVSYFRKLHTPSNILLLSLAVSDFLVGLLLMPLGILRNTGCWVLGDIICSLYWYLTSNIVCAS
IGNIVLISVDRYVAICDPLHYPSRITLAKVKLSVCLCWFYAFFYVSLYTKDILIEPGRDNSCYGECAFVINDIAVVVDLVFSFIVPVSVIVVLYIRVFVAAVSQARAMRSHVTSVT
LQRSLNQTNKSELKAARTLGILVVVFLACFCPLYYFSLVDENAINDPAASFVVIIFYFNSCLNPLIYAFFYPWFRNAVKLIITLQIFKYNMSEANILx
>contig055697-BurTARs.A022
MMEEPELCFPKLLNISCSRPKRPHFEIMLTYILLSFISLLTVILNLLVIISISHFRQLHTPTNFLLLSLAVADFFVGLLMFFQIVLIDGCWFLGDIMCTLYQYLAFIITSASVGTMVI
ISADRYLAICYPLHYSTQITQQRVNICISLCWFFSVIFQSLIVKDNLKQPGKYNSCIGECVFVVNYIAGLFDLLFSFIVPITVIVVLYLRVFVVAVSQARAMRCQLAVTHQRSVT
VTVTKSELKAARTLGVVVVVFLICMCPYYCVALTGQDNFLNASSAAFVICLVYFNSCLNPIIYVFFYPWFRKSIKLIATLQILQPDSRETNMHx
>contig054630-BurTARs.A023
MENVYGQQFCFPQLPNTSCRKPLQHHNQTIFLYILLCCISLLTVTLNVLVIISISHFRQLHNQTNLFLLSLAVSDLLVGLLLMPPRILLLGGCWFLGTFMCGLFYYASFVLTSA
SVGNMVLISFDRYVAICDPLSYPTAVTERKVQISVCLCWACSLLYNGTILNNFLKQPDRYNSCDGECIVVINFITGAFDVVATFIGPTAVIIFLYMRVFLVAVSQAQAMRSHVA
FVTSKGSVHVAIKKSERKAATTIGVVVAVFLMCFCPYFYPSLAGQDTSTSVEFSVFGVWLLYCNSCLNPLIYAFFYPWFRKTVKLIVTLQILQPDSCDANILx
>contig049540-BurTARs.A024
MGTQEKSELCFPQLFNMSCKKPAIARSKAVFLHIVLSSVSFLTVALNLLVIISVSHFRQLHTPTNILLLSLAVSDFLVGLLLSPAEILRSTACWFLGQPTCLMYIFVSLTVTSAS
VGIMVLISADRYVAICDPLNYPIRITDRRVKLCVCLCWLCSIAFSCFLVRDDLHQKRKQNSCYGKCVVVVQYIAGVVDLILTFIVPVTVIIVLYMRVFVVAVSQARSMRSHATA
VRNHLPMTLTKKSELKAARTLGVLVLVFLMCFCPYYCVSLVGEEFINSSSASFVAYLFGLNSCLNPLIYAMFYPWFRKAVKLVVTLQILQPGSSEVSILx
>contig049534-BurTARs.A025
MEKGVELCFPQLLNNSCIKPTLHWSKAVLLNIVLSCISLITAGLNLLVIISVSHFRQLHTPSNILLLSLAVSDFLVGLLLIPAEIFRITVCWVFGESMCSLYTYLGYIVVTSSISNI
VLISVDRYVAICDPLHYPSRISVAKIRLSVCMCWFYSAFYSTLCTKNILIEPGRYNSCYGECVFITSDIAGIIDLVLSFIVPVSIIIVLYMRVFVVAVSQARAMHSHVTATLQRSLN
QTNKSELKAARTLGVLVVVFLASFCPFYCYFLVVEDIVSDSSASIVVIVYYFNSCLNPLIYALFYPWFRNAVKVIITFQIFKRDSSEANVIx
>contig041024-BurTARs.A026
MEIQKAAELCFPQLLNSSCRKPTLHWSKAVLLNIVLSCISLITAALNLLVIISVSHFRKLHTPSNILLLSLAVSDFLVGLLLMPLEIFRNTACWVLGDVMCSVYWYLTNNITCA
SIGNIVLISVDRYVAICDPLHYPTRITLAKVKLSVCLCWFFSIFYWSLYLKDILVEPGRYNSCYGECVLIINDIAGIVDLALSFIVPVTVIIVLYMRVFVVAVSQARAMRSHVTAVT
LQCSLNQANKSELKAARTLGVLVVVFLACYCPFYCYSLVDKNVVNDSSASFVVLVFYFNSCLNPLIYALFYPWFRKAIRCVITLLIFKHDSSEVNVIx
>contig034854-BurTARs.A027
MDTQGGAELCFPQLFNTSCKKPTTPLSQVLLFHILMPSMSLLTVTLNLLVIIAISHFRQLHSHTNILLLSLAAADFLNGLLFMPGEILRNTACWFLGQLTCSLYNYVSYIIASA
SVGNMVLISVDRYVAICDPLHYPTRITEGKVKLCVCLCWLCSVLYSYVILIDDLSQPGKHKSCYGKCIIFIEFIAGFVDLFISFIIPLTVIIVLYMRVFVVAVSQARAMRSQVTTVT
LQLSVTLTATKSELKAARTLGVLVLVFLLCFCPYYIVSLFGDEVLNSASESIAFYFFLLNSCLNPLIYAMFYPWFRKAVKLIVTLQILQPGSCEVSILx
>contig057148-BurTARs.A028
MQKEAELCFPQLLNSSCRKPTFHWSKAVLLNIVLCCISLLTAALNLLVIISVSYFRQLHTPTNILLLSLAVSDFLTGLLMLPLEIIRNTACWVLGDLMCSVYTYLTAGLFCAST
WNIVLISVDRYVAICHPLHYPTRITVARVKLCVCLCWFCSTFYGSLLTKDVLIKPARYNSCYGECVFVINDIAGFIDLVLSFIFPVSVIIVLYMRVFVVAVSQARAMRSHVTAVAL
QRSSNQANKSELKAARNLGVLVVVFLLCYLPFYCYSLIEVNVINDSSTFFLIIVFYFNSCLNPLMYALFYSWFRNAVKLIITLQIFKHDTTEANMFx
>contig059766-BurTARs.A029
MEETELCFQQLFNTSCMRPRRPHFEIMLTYILLSFISLLTVILNLLVIISVSHFRQLHTPTNLLLLSLAVADFYVGLLLFFQIVLIDGCWFLGDIMCTLYQYLAYVITSASIGTMVI
ISVDRYLAICYPLHYSTKITQQRVKIVVCLCWICSVIFQSLILMDNLEQPGRYNSCIGECVFVINYIAGLVDVTFSFIVPFTVIVVLYLRVFVVAVSQARAMRSQLAVTHQRSVT
VTAKKSELKAAWTLGIVVFVFLICMCPYYCVALTGQDNLPSASSLTFVLCLVYFNSCLNPIIYVFFYPWFRKSIKVIVTLQILQPDSCQATVLx
>contig065494-BurTARs.A030
MEETELCFQQLFNTSCMRPRRPHFEIMLTYILLSFISLLTVILNLLVIISVSHFRQLHTPTNLLLLSLAVADFYVGLLLFFQIVLIDGCWFLGDIMCTLYQYLAYVITSASIGTMVI
ISVDRYLAICYPLHYSTKITQQRVKIVVCLCWICSVIFQSLILMDNLEQPGRYNSCIGECVFVINYIAGLVDVTFSFIVPFTVIVVLYLRVFVVAVSQARAMRSQLAVTHQRSVT
VTAKKSELKAAWTLGIVVVVFLICMCPYYCVALTGQDSLPSASSLTFVLCLAYFNSCLNPIIYVFFYPWFRKSIKVIVTLQILQPDSSKVTMRx
>contig057305-BurTARs.A031
MAETELCFPNLNSSCSRVKRSPSDSVLIYVILTIISLLTVALNLLVIISISHFKQLHTPTNHLLLSLAVSDFFVGLNMCFQSMLVDGCWYLGDLMCVLYYVFDIVVTSASVGTM
VLISVDRYVAICDPLHYPTKVTPKRVQTCVLMCWICSLLVVGVLLKDNLDKPGRFNSCFGECVIYVDFAIQVTDLILTILLPITVIVILYVRVFAVAVFQIRAMQPHVPAVTQRGK
VSPKKSELKAARTLGIVIVAFLICLFPYYSVILSGQDTLLDILSVTFFLCLFYFNSCLNPIIYAFFYPWFRKSVKLIVTFQIVKSGSSDASMLx

>contig045302-BurTAR.A001
MDSSGGPPLCFPNLNSSCRRLLRPTSQTVVLYTLLASISLLTVVLNLLVVISISHFRQLNTSTNTLLLSLAVSDLLVGLLVMPIEGLYYIETCWLLGRLMCALSPYLSYCLISVS
LDSMVLISVDRYIAICDPLLYSSKITVNRVKLSVCVCWACSLLYNGCILMEHIGWPDRFSSCHGECVVFISRALGTIDLFFSFLGPCALMFVLYMRVFVVAVSQVRIIRSQAAV
RAPPAAKKSELKAARTLGILIAVFLMCFCPYYYPSFAGDDTSMNLPYYALFSWIMLTNSCVNPVIYALFYPWFRRAIRLIVTLRILQPHSRDVKILx

>contig020038-BurTAR.A002
MDRQDRIELCYPELANLSCRGLTRPQPEAFLLYTLFLSISVLTVALNVLVIISISHFRQLHMPTNVLLLSLAISDLLVGLLVMPVEAMRFIETCWMLGDLMCAFTYIIGFTLTSA
SVGNMVLISIDRYVAICYPLQYPTKITHSRAELSVTLCWACSLLYNGMILKEHLRQPDRHNTCHGQCLVVINYVSGAIDLVFTFIGPCSVIIILYMRVFVVALSQAHAMRSHITA
AAAGTVKITAKKSEKKAARTLGVVIFVFLMSFCPYYYPSLAGQDISNSASSWAIVSWMLYFNSCLNPLIYAFFYPWFRKAIRFIVSLKILEKGSSQANILx
>contig006087-BurTAR.B032
MELFNVTVNTVSFLLCDSQKNKLCVLLYVVLSSFMLLTICGNLLVIISIIYFRYLHTPTNYLILSMAVADLLIGALIFPLSMTVSLKPCLYIYSLLCNLRSTMDVTMGVSSLLNLC
CISVDRYYAVCHPLIYKTKITDCVAMKMGLGSWAVAILCGIFVFLLFFILDECDTSCLFALIAASVVVYYIPTIVLLFMYTKILVVALRQARSIHNTISQNTKSKAVSSTERKATKT
LTIVIGIFLIFWVPLFLSYSFVPLDSFILYVLLEPFNWFAISNSMLNPFIYAFFYTWFRRAFKMIISGKIFQGDVTNIKLHx

>contig084868-BriTARs.A014
METQDEAELCFPQLFNTSCKKPKTSLSQVLLPYVVVFSVSLLTVALNLVDISLSSFRQLHTPTNSLLLSLAVSDFLIGLLMMPAKILGDTACWFLGQLTCSLYSYICFIITSAS
VGIMVLISVDRYVAICDPLHYPTRITERRVKLCVCLCWLCSVIYNILFIKNDLLQGERHTSCYGECVFVIDYIVGTTDIVLTFIAPVTVIVVLYMRVFVVAVSQARAMRSHVTAV
TLQLSVTLTAKKSEIKAARTLGVLVLVFLLGFCPYYIVSLLGNELFNSSSASIAIYLYYFNSCLNPLIYAMFYPWFRKAVKFVVTLQILQPGSCEVSILx
>contig084876-BriTARs.A015
MDTQDVAELCFPQLFNTSCKKPITPLSEFVFLHVVLSSISLLTVTLNLLVIISVSHYRQLHTPTNILLLSLAVSDFLVGLLLMPGEILRNTACWFLGDLTCFMYDYMSLIVTST
SVGDMVLISIDRYVALCDPLHYPTRITDRRVKLSVCLCWLSSVFYSSLFVKDDLTHSGKHNSCYGECTIVVDLITGTIDLLLTFFVPVTVIVVLYLRVFVVAVSQARAMRSHVT
AAALQLSVTLTTKRSELKAARTLGVLVVVFLLCFCPYYCVTLARDDLLNSSSVSFLLYLFYFNSCLNPLIYALLYPWFRKAVKLIISLHILQPGSCEISILx
>contig084880-BriTARs.A016
MEIQMHPEAELCFPELLNSSCRKPTLHWSKTVLLNVGLSSISLITAALNLFIIISVSHFRQLHTPSNIIILSLAVSDFFVCFLLMPVEIFKNTACWVFGDLMCSLYTYLSCILINA
SFEMIILVSIDRYVAICDPLHYPTRITVPRVKLCVFLCWFYAILYNIIYTKHALINPGRYGSCYGECVFVVDDIIGIVDFVVSLIVPVTIIVVLYTRVFVVVVSQARAMRSHVTAVTL
QRPLNQANKSELKAARNLGILVVVFLACYCPFYCYFFLAGNEVNASSASSILIVYYFNSCLNPLMYALFYPWFRNAVKLIITLQILKANSSEINILQRx
>contig084886-BriTARs.A017
MQKEAELCFPQLINSSCRKPTLHWSKAVLLNIVLSCISLLTAALNLLVIISISYFRQLHTPTNILLLSLAVSDFLTGLLMLPLEIIRNTACWVLGDLMCSVYTCLTTGLFCVSTW
NVVLISVDRYVAICHPLHYPTRITVARVKLCVCLCWFYSTFYGSLLTKDVLIKPARYNSCYGECVFVINDIAGFIDLVLSFIVPVSVIIVLYMRVFVVAVSQARAMRSRVTAVTL
QRSSNQAKKSELKAARTLGVLVVGFLACYLPFYCYSLIEVNVINDSSTFFLIIVLYFNSCLNPLMYALFYSWFRNAVKLIITLQIFKHDTSEANMFx
>contig084887-BriTARs.A018
MEIPKGVELCFPQLLNSSCRKPTLHWSKAVLLNIVLSCISLLTAALNLLVIISVSHFRQLHTPSNILLLSLAVSDFFVGLLLLPLEIFRKTSCWVLGDRMCSAYWYLTSNIICAS
IGNIVLISVDRYVAICDPLHYPSRITLAKVKLSVCLCWFYAFFYSNLYTKDIMIEPSRYNSCFGECVFFSSNIAIVVDLILFFFVPVTVIIVLYMRVFVVAVSQARAMRSHVTALTL
QRSLNQTNKSELKAARTLGILVVVFLACFSPLYCYSLVDENAIIDPAASFVVIIFYINSCLNPLIYALFYPWFRNAVKLIITLEIFKYDTSGANILx
>contig086337-BriTARs.A019
MEETELCFPKLLNISCRRPKRPHFEIMLTYILLSFISLLTVILNLLVIISISHFRQLHTPTNFLLLSLAVADFFVGFLMFFQIVLIDGCWFLGDIMCTLYQYLAFIITSASIGTMVIIS
ADRYLAICYPLHYSTKITQQRINICISLCWFFSVIFQSLIVKDNLKQPGKYNSCIGECVFVVNYIAGIFDLLFSFIVPITVIVVLYLRVFVVAVTQARAMRCQLAVTHQRSVTVTV
MKSELKAARTLGVVVVVFLICMCPYYCVALTGQDNFLNASSAAFVICLVYFNSCLNPIIYVFFYPWFRKSIKLIATLQILQPDSCETNMHx
>contig086344-BriTARs.A020
MEETELCFPQLFNSSCVRQKHSQIEAVFLYTPLSSISVLTTILNLLVIISIAHFKQLHTPTNLLLLSLAVSDFFVGLIMACQISLLDGCWFLGDHMCALYTTLDYIVTSASVGTM
VFISADRYVAICDPLHYPTEITIKRISVCICTCWACSILYNSLIMKDNFKQPGRYNSCSGDCAVVIDYFVGIFDFVLTFVGPVIVIIVLYLRVFVVAVSQARAMRSHITALRLQGS
ETVHAKKSELKAARTLGVLVIAFLICLFPFFCSSMVGQNSFFDIRSVPFERLLFYFNSCLNPLIYTFCYPWFLKSIKLIVTFKIFRHGSSEASILx
>contig086351-BriTARs.A021
MAETELCFPNLNSSCSRVKRSPSESVLIYVILTIISLLTVALNLLVIISISHFKQLHTPTNYLLLSLAVSDFFVGFNMCFQSMLIDGCWYLGDLMCVLYYVFEIVVTSASVGTM
VLISVDRYVAICDPLHYPTKVTPKRVQTCVLICWICSLLVVGVLLKDNLDKPGRFNSCFGECVIYIDFAIQVTDLIFTILLPITVIVILYVRVFAVAVFQIRAMQHHVAAGTQRGK
VSPKKSELKAARTLGIVIVAFLICLFPYYCVILSGQDTLLDILSLAFFLCLFYFNSFLNPIIYALFYPWFRKSVKLIVTFQIVKSGSSDASMLx
>contig082565-BriTARs.A022
MDTQDGAELCFPQLFNISCKKPTTPLSQVLLIYTVVSSMSLLTVTLNLLVIIAVSHFRQLHTPTNILLLSLAVTDFLVGLLFMPGEILRNTACWFLGQLTCSLYNYASYIIASAS
VGNMVLISVDRYVAICDPLHYPTRITERRVKLCVCLCWLCSVFYSYVILIDDLSQPGKHKSCYGKCIIFIEFIAGFVDLVLAFIIPLTVIIVLYMRVFVVAVSQARAMRSQVTAVT
LQLSVTLTAKKSELKAARTLGVLVLVFLLCFCSYYIISLFGNELLNSSSASIVIYLYYFNSCLNPLIYAMFYPWFRKAVKLIVTLQILQPGSCEVSILx

>contig025313-BriTAR.A002
MDSQDRIELCYPELANLSCRGLTRPQPEAVLLYTLFLSISVLTVALNVLVIISISHFRQLHMPTNVLLLSLAISDLLVGLLVMPVETIRFIETCWLLGDLMCAFSYIIGFTLTSASV
GNMVLISIDRYVAICYPLQYPTKITHSRAELSVTLCWACSLLYNGLILKEHLRQPDRHNTCHGQCLVVINYVSGAIDLVFTFIGPCSVIIILYMRVFVVAVSQAHAMRSHITAAA
AGRVKITAKKSEKKAARTLGVVIFVFLMSFCPYYYPSLAGQDISNSASSWAIVSWMLYFNSCLNPLIYAFFYPWFRKAIWFIVSLKILEKGSSQANILx
>contig029633-BriTAR.A003
MDGTGGPPLCFPNLNSSCRRLLRPTSQAALLYTLLASVSLLTVVLKLLVVVSISHFKQLHTPTNALLLSLAMSDLLLGFLVMPIEGLCYIETCWLLGRLMCALSPYLSYCLLS
FSLGSMVLISVDRYIAICDPLLYSSKITVNRVKLSVCVCWACSLLYNGCILMGHIGWPDRFSSCHGECVVFISRTSGTVDFFLSFLGPCAVMFVMYMRVFVVAVSQVRAIQS
QAAVRAVPAAKKSELKAARTLGILIAVFVMCFCPYYYPSLAGVDTFLSLPSYAMVCWIMLINACVNPVIYVLFYPWFRRALRFIVTLRILQPHSRDVKILx
>contig035253-BriTAR.B025
MTNIVNDLHPCYVIQDSKYMLTNSPSIICVFLYAFLALLSVITICGNLLVIISVIYFKQLHTPTNYLILSLAVADLLVGIIAFPLSMAFSLSSCLYHEGLFCKVRGTFDISLSTCSILN
LCCISIDRYHAVCQPLTYQTKISPRVVVFMILMSWGVSGIIGISVTIAGFNNEKCEESCLIDVLIESTVGPMLSFYLPVTMMLFIYLKIFFVALRQARRIQNTTKCGQTASIMER
KATKTLAIVLGVFIFCWSPFFLSITFPPFTSDSVPVPVIETLNWLTLANSMLNPFIYAFFYSWFRSAIRMIICGKIFQGDFANTNMTx

>contig066890-ZebTARs.A014
MDTQDGEELCFPQLFNISCKKPTTPLSQLLLPYIVVFSMSLLTVILNLLVIIAVSHFRQLHTPTNFLLLSLAVSDFFIGLLLMPANIIRKKVCWFLGQLACLVFTYVSFIITSASV
GIMVLISVDRYAAICDPLHYPTRITRERVKLCVCLCWLCSVIYNILLIKDELVQPGQHNSCYGECKILVDYIAGTTDIVLTFIAPITVIIVLYMRVFVVAVSQARTMRSQVTAVTLQ
LSVTLTTKKSELKAARTLGVLVLVFLICFCPHYCIFVFGGKVLNSSSATIVIYLYYFNSCLNPLIYAMFYPWFRKAVKLIVTLQILQPGSCEVSILx
>contig066691-ZebTARs.A015
MEIPKGVELCFPQLLNSSCRKPTLHWSKAVLLNIVLSCISLLTAALNLLVIISVSHFRQLHTPSNILLLSLAVSDFFVGLLLLPLEIFRNTSCWVLGDRMCSAYWYLTSNIICAS
IGNIVLISVDRYVAICDPLHYPSRITLAKVKLSVCLCWFYAFFYSNLYTKNIMIEPGRYNSCFGECVFFSSNIAIVADLILFFFVPVTVIIALYMRVFVVAVSQARAMRSHVTLVT
LQRSLNQTNKSELKAARTLGILVVVFLACFSPLYCYSLVDENAINNPAASFAVIIFYINSCLNPLIYALFYPWFRNAVKLIITLEIFKYDTSGANILx
>contig066285-ZebTARs.A016
MEKGAELCFPHLFNNSCIKPTLHWSKAVLLNIVLSCISLITAGLNLLVIISVSHFRQLHTPSNILLLSLAVSDFLVGLLLIPAEIFRITVCWVFGESMCSLYTYLGYIVVTSSISNI
VLISVDRYVAICDPLHYPSRISVAKISLSVCMCWFYSAFYSTLCTKNILIEPGRYNSCYGECVFITSDIAGIIDLVLSFIVPVSIIIVLYMRVFVVAVSQARAMHSHVTATLQRSLN
QTNKSELKAARTLGVLVVVFLASFCPFYCYFLVVEDKVSDSSASIVVIVYYFNSCLNPLIYALFYPWFRNAVKVIITFQIFKHDSSEVNVIx
>contig066056-ZebTARs.A017
MEETELCFPQLLNSSCVRQKRPQIEAVCIYTLLSSISLLTAVLNLLVIISIAHFKQLHTPTNLLLLSLAVSDFFVGLIMAFEISLLDGCWFLGDHMCALYSSLDYIVTSASVGTM

VLISADRYVAICDPLHYPTKITIKRVSVSICTCWACSILYNSLIMKDNFKQPGRYNSCSGDCVVVIDYFVGIFDFVLTFVGPVIVIIVLYLRVFVVAVSQARAMRSHITALRLQGS
ETVHAKKSELKAARTLGVLVIAFLICLFPFFCSSMVGQNSFFDIRSVPFELLLFYFNSCLNPLIYTFCYPWFLKSIKLIVTFKIFRHGSSEASILx
>contig062677-ZebTARs.A018
MGTQEKSELCFPQLFNMSCKKPAIARSKAVFLHIVLSSVSFLTVALNLLVIISVSHFRQLHTPTNILLLSLAVSDFLVGLLLSPAEILRSTACWFLGQPTCLMYIFVSLTVTSAS
VGIMVLISADRYVAICDPLNYPIRITDRRVQLCVCLCWLCSITFSCFLVRDDLHQRRKQNSCYGKCVVVVQYIAGVVDLILTFIVPVTVIIVLYMRVFVVAVSQARSMRSHATA
VRNHLPMTLTKKSELKAARTLGVLVLVFLMCFCPYYCVSLVGEEFINSSSASFVAYLFGLNSCLNPLIYAMFYPWFRKAVKLVVTLQILQPGSSEVSILx
>contig062676-ZebTARs.A019
METQDEAELCFPQLFNISCKKPKTSLSQVLLPYVVVFSVSLLTVALNLLVIVSVSHFRQLHTPTNILLLSLAVSDFLIGLLLMPAKILRDTACWFLGQLTCSLYSYICFVVTSA
SVGIMVLISVDRYVAICDPLHYPTRITDRRVKLCVCLCWLCSVFYNILFIKDDLLQRERHTSCYGECVFVIDYIVGTTDIVLTFIAPVTVIVFLYMRVFVVAVSQARAMRSHVTA
VTLQLSVTLTAKKSELKAARTLGVLVLVFLLYFCPYYIVSFFGYELLNSSSASIVIYLYYFNSCLNPLIYAMFYPWFRKAVKLVVTLQILQPGSCEVSILx
>contig061417-ZebTARs.A020
MEIQKGAELCFPQLLNSSCRKPTLRWSKAVLLNIVLSCISLLTAALNLLVIISVSYFRKLHTPSNILLLSLAVSDFLMGLLLMPAEILRSMTCWVLGDLMCSVYFFLTVNITCA
SIGNIVLISIDRYVAICDPLHYSTRITVARVKLSVCLCWFYSTFYCSLYTQEMLIEPGRYNSCYGECVLVISDFAGMVDLILFFILPLSVIIVLYTRVFVVAVSQARAMRSHVTAV
TLQRSLNQTNKSELKAARTLGVLVVVFLSCYCPFYCYSLTDKNAVNDPATSSVIFIFYFNSCINPLIYALFYPWFRNAVKLIITLQIFKHNSCEANILx
>contig061410-ZebTARs.A021
MDTQDVAELCFPQLFNTSCKKPPLSEFVFLHVVLSSISLLTVTLNLLIIISVSHYRQLHTPTNILLLSLAVSDFLVGLLLMPGEILRNTACWFLGDLTCSMYNYMSLIVTSTSV
GDMVLISIDRYVAICDPLHYPTRITDRRVKLCVCLCWLCSVFYSSLFVKDDLTQPGKHNSCYGECTIDVDLITGTIDLLLTFFVPVTVIVVLYLRVFVVAVSQARAMRSHVTVA
ALQLSVTLTTKKSELKAARTLGVLVVVFLLCFCPYYCVTLARDDLLNSSSVSFLLYLFYFNSCLNPLIYALLYPWFRKAVKLIISLHILQPGSCEISILx
>contig059768-ZebTARs.A022
MENVYGQQFCFPQLLNTSCRKPLQHHNQTIFLYILLCCISLLTVTLNVLVIISISHFRQLHNQTNLFLLSLAVSDLLVGLLLMPPRILLLGGCWFLGTFMCGLFYYASFVLTSA
SVGNMVLISFDRYVAICDPLSYPTTVTERKVQISVCLCWACSLLYNGTILNNFLKQPDRYNSCDGECIVVINFITGAFDVVVTFIGPTAVIIFLYMRVFLVAVSQAQAMRSHVA
FVTSKGSVHVAIKKSERKAATTIGVVVAVFLMCFCPYFYPSLAGQDTSTSVEFSVFGVWLLYCNSCLNPLIYAFFYPWFRKTVKLIVTLQILQPDSCDANILx
>contig053139-ZebTARs.A023
MEEPELCFPKLLNISCIRPKRPHFEIMLTYILLSFISLLTVILNLLVIISISHFRQLHTPTNFLLLSLAVADFFVGLLMFFQIVLIDGCWFLGDIMCTLYQYLAFIITSASVGTMVIIS
ADRYLAICYPLHYSTQITQQRVNICISLCWFFSVIFQSLIVKDNLKQPGKYNSCIGECVFVVNYIAGLFDLLFSFIVPITVIVVLYLRVFVVAVSQARAMRCQLAVTHQRSVTVT
VTKSELKAARTLGVVVVVFLICMCPYYCVALTGQDNFLNASSAAFVICLVYFNSCLNPIIYVFFYPWFRKSIKLIATLQILQPDSRETNMHx
>contig030471-ZebTARs.A024
MEIQKAAELCFPQLLNSSCRKPTLHWSKAVLLNIVLSCISLITAALNLLVIISVSHFRKLHTPSNILLLSLAVSDFLVGLLLMPLEIFRNTACWVLGDRMCSAYWYLTINIICASI
GNIVLISVDRYVAICDPLHYPSRITLAKVKLSVCLCWFYAFFYSNLYTKNIMIEPGRYNSCFGECVFFSSNIAIVADLILFFFVPVTVIIALYMRVFVVAVSQARAMRSHVTSVTL
QCSLNQANKSELKAARTLGVLVVVFLACYCPFYCYALVDKNVVNDSSASFVVLVFYFNSCLNPLIYALFYPWFRKAIRCVITLLIFKHDSSEVNIIx
>contig030464-ZebTARs.A025
MQKEAELCFPQLLNSSCRKPTFHWSKAVLLNTVLCCISLLTAALNLLVIISVSYFRQLHTPTNILLLSLAVSDFLTGLLMLPLEIIRNTACWVLGDLMCSVYTYLTAGLFCAST
WHIVLISVDRYVAICHPLHYPTRITVARVKLCVCLCWFCSTFYGSLLTKDVLIKPARYNSCYGECVFVINDIAGFIDLVLSFIFPVSVIIVLYMRVFVVAVSQARSMRSHVTAVTL
QRSSNQANKSELKAARNLGVLVVVFLACYLPFYCYSLIEVNVINDSSTFFLIIVFYFNSCLNPLMYALFYSWFRNAVKLIITLQIFKHDTSEANMFx
>contig030445-ZebTARs.A026
MDTQDGAELCFPQLFNISCKKPTTPLSQVLLLYIVVSSMSLLTVTLNLLVIIAVSHFRQLHTPTNILLLSLAVTDFLVGLLFMPGEILRNTACWFLGQLTCSLYNYVSYIITSAS
VGNMVLISVDRYVAICDPLHYPTRITERRVKLCVCLCWLCSVFYCYVILIDDLSQPGKHNSCYGRCIIIIEFIAGFVDLFLSFIIPLTVIIVLYMRVFVVAVSQARAMRSQVTAVT
LQLSVTLTAKKSELKAARTLGVLVLVFLLCFCSYYFISLFGNDLLNSSSASIVIYLYYFNSCLNPLIYAMFYPWFRKAVKLIVTLQILQPGSCEVSVLx
>contig066330-ZebTARs.A027
MEEAELCFQQLFNTSCMRPRRPHFEIMLTYILLSFISLLTVILNLLVIISVSHFRQLHTPTNLLLLSLAVADFYVGLLLFFQIVLIDGCWFLGDIMCTLYQYLAYVITSASIGTMVI
ISVDRYLAICYPLHYSTKITQQRVKIVVCLCWICSVIFQSLILMDNLEQPGRYNSCIGECVFVINYIAGLVDVTFSFIVPFTVIVVLYLRVFVVAVSQARAMRSQLAVTHQRSVT
VTAKKSELKAAWTLGIVVVVFLICMCPYYCVALTGQDSLPSASSLTFVLCLAYFNSCLNPIIYVFFYPWFRKSIKVIVTLQILQPDSSKVTMRx
>contig053145-ZebTARs.A028
MEEAELCFQQLFNTSCMRPRRPHFEIMLTYILLSFISLLTVILNLLVIISVSHFRQLHTPTNLLLLSLAVADFYVGLLLFFQIVLIDGCWFLGDIMCTLYQYLAYVITSASIGTMVI
ISVDRYLAICYPLHYSTKITQQRVKIVVCLCWICSVIFQSLILMDNLEQPGRYNSCIGECVFVINYIAGLVDVTFSFIVPFTVIVVLYLRVFVVAVSQARAMRSQLAVTHQRSVT
VTAKKSELKAAWTLGIVVVVFLICMCPYYCVALTGQDNLPSASSLTFVLCLVYFNSCLNPIIYVFFYPWFRKSIKVIVTLQILQPDSCQATVLx
>contig030440-ZebTARs.A029
MDTQSRAELCFPQLFNTSCKKPTTPLSQVLLFHILMPSMSLLTVTLNLLVIIAVSHFRQLHTPTNILLLSLAAADFLIGLLFMPGEILRNTVCWFLGQLTCSLYNYISFVVTSA
SVGDMVLISVDRYVAICDPLHYPTRITERRVKLCVCLCWLCSVFYCYMIVRDDLSQPGKHNSCFGECIIFVEFIAGFVDLVLSFIIPVTVIVFLYMRVFVVAVSQARAMRSQV
TAVTLQLSVTLTATKSELKAARTLGVLVLVFLICFCPYYIVSLFGDEFLNSASASIVFYFFLLNSCLNPLIYAMFYPWFRKAVKLIMTLQILQPGSCEVSILx

>contig040586-ZebTAR.A001
MDSSGGPPLCFPNLNSSCRRLLRPTSQTVVLYTLLASISLLTVVLNLLVVISISHFRQLNTSTNTLLLSLAVSDLLVGLLVMPIEGLDYIETCWLLGRLMCALSPYLSYCLISVS
LDSMVLISVDRYIAICDPLLYSSKITVNRVKLSVCVCWACSLLYNGCILMEHIGWPDRFSSCHGECVVFISRALGTIDLFFSFLGPCALMFVLYMRVFVVAVSQVRIIRSQAAV
RAAPAAKKSELKAARTLGILIAVFLMCFCPYYYPSFAGDDTSMSLPYYALFSWIMLTNSCVNPVIYALFYPWFRRAIRLIVTLRILQPHSRDVKILx
>contig040586-ZebTAR.A002
MDGTGGPPLCFPNLNSSCRRLLRPTSQAALLYTLLASVSLLTVVLNLLVVVSISHFKQLHTPTNALLLSLAMSDLLVGLLVMPIEGLRYIETCWLLGRLMCALSPYLSYCLIS
VSLGSMVLISVDRYIAICDPLLYSSKITVNRVKLSVCVCWACSLLYNGCILMEHIGWPDRFSSCHGECVVFISQTSGTVDFFLSFLGPCGVMFVLYMRVFVVAVSQVRAIQS
QAAVRAAPAAKKSELKAARTLGILIAVFVMCFCPYYYPSLAGVDTFLSLPSYAMVCWIMLINACVNPVIYVLFYPWFRRAIRFIVSLRILQPHSRDVKILx
>contig003909-ZebTAR.A003
MDRQDRIELCYPELANLSCRGLTRPQPEAVLLYTLFLSISVLTVALNVLVIISISHFRQLHMPTNVLLLSLAISDLLVGLLVMPVEAMRFIETCWLLGDLMCAFTYIIGFTLTSAS
VGNMVLISIDRYVAICYPLQYPTKITHSRVELSVTLCWACSLLYNGMILKEHLRQPNRHNTCHGQCLVVINYVSGAIDLVFTFIGPCSVIIILYMRVFVVAVSQAHAMRSHITAA
AAGTVKITAKKSEKKAARTLGVVIFVFLMSFCPYYYPSLAGQDISNSASSWAIVSWMLYFNSCLNPLIYAFFYPWFRKAIWFIVSLKILEKGSSQANILx
>contig033536-ZebTAR.B029
MELFNVTVNTVSFLLCDSQKNKLCVLLYVVLSSFMLLTICGNLLVIISIIYFRYLHTPTNYLILSMAVADLLIGALIFPLSMTVSLKPCLYIYSLLCNLRSTMDVTMGVSSLLNLC
CISVDRYYAVCHPLIYKTKITDCVAMKMGLGSWAVAILCGIFVFLLFFILDECDTSCVFALIAASVVVYYIPTIVLLFMYTKILVVALRQARSIHNTISQNTKSKAVSSTERKATKT
LTIVIGIFLICWVPLFLSYSFVPLDSFILYVLLEPFNWFAISNSMLNPFIYAFFYTWFRRAFKMIISGKIFQGDVTNIKLHx

Tetraodon TAAR sequences

>TETRENSTNIP3225
MEPVFCLEKNISCVKTVYPLPFRVILYMVLGVMVIVTVCGNLLVTVSIIYFKQLHTPTNY
LLVSLAVSDLFLGLLVMLPNMIQSVETCWYFGDILCKVYLSIGVMLCTASIINLSLISID
RYNVVIHPLRYRRKMSGNVILLMISFSWGVSGVVGFGMIFFRLNILGIEDFYNSVACEGR
CIFLQSGMSSTVSSVLSFYIPGIIMISLYLKIFIVAKRQFLSIQNTTCMASARDSNKKQT
KATKTLVVIMGAFLLCWAPFFVCNIIHPFISYSTPAALFETFLWAGYINSTLNPIIYAFF
YTWFRKAFRLFVSGKIFKDDMSNITLF

>TETRENSTNIP1455
MEPVFCYESKNISCVKTVYPLPIRVILYMVLGVMVVVTVCGNLLVTVSIIYFKQLHTPTN
YLLVSLAVSDFLLGFFVMLPSMIQLVETCWYFGDILCKVHLSFAVMLCTASIINLSTISI

DRYNAVIHPLLYRRKMSGNVVFLMIFFSWSVSGVVGFGMIFLRLNILGIEDFYNNFVACE
GRCVLLQSGLSSTVSSVLSFYIPGIIMISLYLKIFFVAKRQFLSIQNTSSMTSARDSNKK
QAKATKTLVVIMGAFLLCWAPFFLCNIIDPYISYSTPAALIETFLWVGYINSTLNPLIYA
FFYTWFRKAFCLFASGKVFKEDMSNISLFTD
>TETRENSTNIP7922
MEPVFCFESQNISCVKTVYPLPIRVILYMVLGVMVIVTVCGNLLVTVSIIYFKQLHTPTN
YLLVSLAVSDFLLGFLVMLPNMIQSVETCWYFGDILCKVYMSSDVMLCTASIINLSLISI
DRYNAVIHPLLYRRKMSGHVVLLMISFSWSVSGVVGFGQIFLRLNILGIEDFYNNFVACE
GRCVLLQSGPSSTVSSVLSFYIPGIIMISLYLKIFFVAKRQLLSIQNTTCMTSARDSDKK
QAKATKTLVVIMGAFLLCWAPFFLCNIIDPFISYSTPAALFETFVWLGYINSTLNPLIYA
FFYTWFRKSFCLFASGKVFKEDMSNISLFTD

>TETRENSTNIP7160
MEPVFCYESKNFSCVKTVYPLPIRVILYMIFGVIVIVTVCGNLLVTVSIIYFKQLHTPTN
YLLASLAVSDFLLGFLVMLPDMIQLVETCWYFGDILCKVHLSFAVMLCTTSIINLSLISI
DRYNAVIHPLLYRRKMSGHVVLLMIFFSWSVSGVVGFGMIFFRLNILGIEDFYNFVACEG
RCVFLQSGMSSTLSSVLSFYIPGIIMINLYLKIFFVAKRQFLSIHNTTCMTSAQISNNKQ
AKATKTLVVIMGVFLLCWAPFFLCNIIDPFISYSTPAALFKTILWGGLFNSTLNPIIYAF
FYTWFRKAFHLFVSGKVFKDDMSNTSLFVE

>TETRENSTNIP3518
MEPVFCFEKNISCVKTVYPLPFRVILYMVLGVMVVVTVCGNLLVTVSIIYFKQLHTPTNY
LLVSLAVSDFLLGFLVMLPDVIQSVETCWYFGDILCKVHLSSDVMLCTASIMNLSLIAID
RYNAVIHPLVYRRKMSGHVVLLMIFFSWSVSGVVGFGMIFFRLNIFGTEDFYNNFVACEG
KCLLFQRGLSGTVLSVLSFYIPGIIMISVYLKIFFVAKKQFLSIQNTTCMTSARDSDKKQ
AKATKTLVVIMGAFLLCWAPFFLCHIIDPLISYSTPAAMFETFVWLGYINSTLNPLIYAF
SYTWFRKAFRLFASGKVFKAGYVKHFPFYSLNG

>TETRENSTNIP7161
MEPVFCVESKNISCVKTVYPLPFRVILYMVLGVTVIVTVCGNLLVTVSIIYFKQLHTPTN
YLLVSLAVSDFLLGFLVMLPDMIQLAETCWYFGDILCKVHLSFAVMLCTASIINLSLISI
DRYNAVIHPLLYRRKMTGNVVLLMIFVSWSISGVVGFGMIFFRLNILGIEDFYKDFVACE
GRCILFQSGMSSTVSSVLSFYIPGIIMICVYLKIFFVAKRQFLSIQNTTCMTSARISNKK
QTKATKTLVVIMGAFLLCWAPFFLCNVIDPFISYSTPAALMKTFGWVGFINSTLNPLIYA
FFYTWLRKAFCLFASGKVFKEDMSNITLFTE

>TETRENSTNIP7869
MVLGVMVIVTVCGNLLVTVSIIYFKQLHTPTNYLLVS
LAVSDFLLGFLVMLPNMIQLVETCWYFGDILCKVYMSSDVMLCTASIINLSLISIDRYNA
VIHPLLYRRKMSGHVVLLMISFSWSVSGVVGFGQIFLRLNILGIEDFYNNFVACEGRCVL
LQSGPSSTVSSVLSFYIPGIIMISLYLKIFFVAKRQLLSIQNTTCMTSARDSDKKQAKAT
KTLVVIMGVFLLCWAPFFLCNIIDPFISYSTPTALFETFIWLGYINSTLNPLIYAFFYTW
FRKAFCLFASGKVFKEDMSNISLFTD

>TETRENSTNIP2491
MDSNGDPWLCFANLNSSCKRLNQTFSETAVLNSLLASISLVTVTLNLLVIVSISHFRQLH
TPTNALLLSLAVSDLLVGLLVMPIEGLRYVETCWLLGSLMCALTPYVSYSVLSASVGNMV
LISIDRYLAICDPLLYSNKVTLKRAKIAICVCWAGSLFYNGCILIGHLKHPDRYSSCHGE
CVVLIDHISGTADLFITVVFPCTIMVVMYMRVFAAAVAQMRVIRLQNAAVAVNAATTVKK
SEWKAARTLGIVIAVYLMCFCPYYYPALAGGDTSNSLSYFAALSWIMMINSCVNPLIYAL
FYPWFRRSIKLIITLRILRSYSSEIKII

>TETRENSTNIP3943
MDSNGDPWLCFANLNSSCKRLNRTFSETAVLNSLLASISLVTVTLNLLVIVSIS
HFRQLHTPTNALLLSLAVSDLLVGLLVMPIEGLRYVEMCWRLGKLMCVLAPYVSYSVLSA
SVGNMVLISIDRYLAICDPLLYSNKVTLKRAKIAVCVCWAGSVLHNGCILIGHLKHPERY
SSCHGECVVVIDHISGTVDLFITFFAPCTIMVVMYMRVFAAAVAQMRVIRLQNAAVAVNT
ATTVKKSEWKAARTLGIVVSVFLMCFCPYYYPAFAGEDTSNNSSYFAALVWIMMINSCMN
PLIYALFYPWFRRSIKLIITLRILRSYSITFTLKKI

>TETRENSTNIP216
EPGLTVNDTSTLDEIHPCYTSHGKAYTFMHIASVKCVLFYIFLGLLSVVTVCGNVLVIIS
VVYFKQLHVRTNYLILSLAVADLLVGVLVFPFSMAFTVTSCWYQENSFCKIRIFLDVTLC
TASILNLCCISIDRYYAVCQPLTYKSKINDRIIGIMILVSWGVAASVAICIVYGENVDGK
CEESCLTDAVISTTLSCIFSFYLPVIVMLKIYMKIFFAARKQAKKIQNAKPGATVSKMER
KATKTLAIVMGVFLLCWTPYFLTIIFQPLIHNITSISVIEALSWLALLNSMLNPFIYAFF
YSWFRSAFRIIMSGKIFYSDYSNTKLL

>Chr1-Tetra.TARs.006
MRYTLCVLLLFNNDSTCVNKLLQKLKAVNVLVIFFLIAVNLLLKISRTFSLFRHFQTTTNLILLSMAVSDFLVGLAVMPLMIVTLDSCRGTSTVVCYLYHLFSFILTSASVGNMV
LISVDRYVAICYPLRYSSIKPNRVKICVSLCWISSVIYNFILLNDYLSQLDFTSSCYKKCILYIDYILVIVDVVITFCVPLTVIIVLYSRVFVVAVTQARAMRAQVSTISSQSVSAMK
SEMRAARTLGIIILFFLMSFFPYYISSLTGQGLSDEALTGQLLLFFCNSTINPIIYAFFYPWFRKSLKVLVSGKYCGL

>Chr1-Tetra.TARs.002
MVMLLEDGCISPFNTSCRFMSGSWKSTLTSAALFCLSPLTVVLNLLVVFSISHVRHLQTTTNLILLSMAVSDLLVGLAVMPLIIVTLDSCQGTSTFECYLYQLLGLILTSASVG
NMVLISVDRYVAICYPLRYSSIKPNRVKLCVSLCWICSVIYSFILMKDSLSLIDFSVTCYKKCILFINFILVIVDVVITFYVPLTVIIVLYSRVFVVAVTQARAMRAQVSTISSQSVS
AMKSEMRAARTLGIIVLFFLMSFLPYYISYLTGHSTDTESVANQLILFCCNSTINPIIYAFFYPWFRNNVKVIISGKVFGL
