# Supplementary material for: Identification and characterization of cichlid TAAR genes and comparison with other teleost TAAR repertoires
Source: BMC Genomics. 2015 Apr 23;16(1):335. doi: 10.1186/s12864-015-1478-4 (PMC4415300; doi:10.1186/s12864-015-1478-4)
Supplement: Additional file 6: — List of pairs and triplets of orthologous genes displaying at least 99% identity. [file 12864_2015_1478_MOESM6_ESM.pdf]

GENES_NAME	% ID AA	% ID DNA
contig056023-BurTARs.A019<br>contig045999-NyeTARs.A026<br>contig084880-BriTARs.A016	99%	99%
contig061433-BurTARs.A013<br>contig061414-ZebTARe.A006	99%	**78%**
contig057148-BurTARs.A028<br>contig046010-NyeTARs.A019<br>contig030464-ZebTARs.A025	99%	99%
contig061091-BurTARs.A014<br>contig066691-ZebTARs.A015	99%	99%
contig022371-TilTARe.A009<br>contig022368-TilTARe.A008	99%	99%
contig049540-BurTARs.A024<br>contig056200-NyeTARs.A029<br>contig062677-ZebTARs.A018	99%	99%
contig109361-BriTARe.A012<br>contig109362-BriTARe.A011	100%	100%
contig065494-BurTARs.A030<br>contig066330-ZebTARs.A027	99%	99%
contig053145-ZebTARs.A028<br>contig059766-BurTARs.A029	99%	99%
contig055697-BurTARs.A022<br>contig053139-ZebTARs.A023	99%	99%

contig057301-BurTARs.A017<br>contig061509-ZebTARe.A005	99%	99%
contig057305-BurTARs.A031<br>contig060292-NyeTARs.A027	99%	99%
contig066056-ZebTARs.A017<br>contig059670-BurTARe.A004	99%	99%
contig020038-BurTAR.A002<br>contig032272-NyeTAR.A004<br>contig003909-ZebTAR.A003	**98%**	**98%**
contig040586-ZebTAR.A001<br>contig045302-BurTAR.A001	99%	99%
contig006087-BurTAR.B032<br>contig052987-NyeTAR.B030<br>contig033536-ZebTAR.B029	99%	99%
