# Supplementary material for: Identification and characterization of cichlid TAAR genes and comparison with other teleost TAAR repertoires
Source: BMC Genomics. 2015 Apr 23;16(1):335. doi: 10.1186/s12864-015-1478-4 (PMC4415300; doi:10.1186/s12864-015-1478-4)
Supplement: Additional file 7: — List of contigs and scaffolds harboring TAAR genes, with their positions. [file 12864_2015_1478_MOESM7_ESM.pdf]

OR short names	Sub-fam	DNA Length	AA Length	Contig	Contig Length	Contig From	Contig To	Scaffold	Scaffold Length	LG	St	Groupe RH
TiITAR.A001	A6	978	325	ctg039639	5679	1597	2574	scaffold_195	1385799	LG16-21	+	RH2
TiITAR.A002	A6	975	325	ctg039640	11883	4619	5596	scaffold_195	1385799	LG16-21	+	RH2
TiITAR.A003	A6	975	325	ctg039640	11883	7891	8868	scaffold_195	1385799	LG16-21	+	RH2
TiITAR.A004	A6	975	325	ctg039642	47976	1045	2022	scaffold_195	1385799	LG16-21	+	RH2
TiITAR.A005	A5	984	328	ctg023443	44581	5727	6710	scaffold_84	2944784	LG16-21	-	RH2
TiITAR.B062	B1	963	321	ctg002574	25597	14156	15118	scaffold_005	9949877	LG16-21	+	RH2
TiITARes.A017	A2	165	55	ctg056115	1471	952	1471	scaffold_374	439512	LG16-21	+	RH2
TiITARe.A013	A2	549	183	ctg056116	1046	1	554	scaffold_374	439512	LG16-21	+	RH2
TiITARe.A015	A2	354	118	ctg056128	2735	2226	2735	scaffold_374	439512	LG16-21	+	RH2
TiITARe.A014	A2	549	183	ctg056129	1340	1	554	scaffold_374	439512	LG16-21	+	RH2
TiITARs.A058	A2	975	325	ctg056134	7268	1690	2815	scaffold_374	439512	LG16-21		RH2
TiITARs.A024	A2	975	325	ctg007512	6207	2223	3438	scaffold_16	6775405	LG16-21	+	RH2
TiITARs.A019	A2	894	298	ctg007518	16566	12546	13655	scaffold_16	6775405	LG16-21	-	RH2
TiITARs.A025	A2	987	329	ctg007520	18759	7551	8728	scaffold_16	6775405	LG16-21	-	RH2
TiITARs.A026	A2	987	329	ctg007524	9071	4463	5627	scaffold_16	6775405	LG16-21	-	RH2
TiITARp.A022	A3	975	325	ctg007525	5617	1246	2382	scaffold_16	6775405	LG16-21	+	RH2
TiITARp.A020	A2	1134	378	ctg007531	1503	234	1367	scaffold_16	6775405	LG16-21	-	RH2
TiITARs.A057	A2	990	330	ctg045088	4544	2084	3192	scaffold_242	989036	LG16-21	+	RH2
TiITARp.B065	B2	942	314	ctg037870	15198	11520	12463	scaffold_183	1538350	LG16-21	+	RH2
TiITARp.B066	B2	939	313	ctg037874	3428	586	1525	scaffold_183	1538350	LG16-21	+	RH2
TiITAR.B060	B1	951	316	ctg037879	21330	5684	6634	scaffold_183	1538350	LG16-21	-	RH2
TiITAR.B061	B1	981	326	ctg037889	5036	1097	2077	scaffold_183	1538350	LG16-21	+	RH2
TiITARe.B064	B1	330	110	ctg037890	1847	1518	1847	scaffold_183	1538350	LG16-21	+	RH2
TiITAR.B063	B1	903	301	ctg037900	3050	437	1339	scaffold_183	1538350	LG16-21	+	RH2
TiITARe.A016	A1	618	206	ctg072760	836	219	836	scaffold_1455	7045	LG16-21	-	RH2
TiITARe.A012	A1	327	109	ctg033907	7533	1	356	scaffold_155	1854179	LG16-21	+	RH2
TiITARs.A059	A1	984	328	ctg022320	24262	9244	10439	scaffold_78	3107833	LG16-21	+	RH2
TiITARe.A006	A1	840	280	ctg022321	10450	4	849	scaffold_78	3107833	LG16-21	+	RH2
TiITARe.A007	A1	777	259	ctg022322	6532	1	783	scaffold_78	3107833	LG16-21	+	RH2
TiITARs.A027	A1	987	329	ctg022324	9909	2838	4063	scaffold_78	3107833	LG16-21	+	RH2
TiITARs.A028	A1	984	328	ctg022330	1696	23	1215	scaffold_78	3107833	LG16-21	+	RH2
TiITARs.A029	A1	984	328	ctg022334	11402	919	2111	scaffold_78	3107833	LG16-21	+	RH2
TiITARs.A030	A1	930	310	ctg022334	11402	8257	9648	scaffold_78	3107833	LG16-21	+	RH2
TiITARs.A031	A1	990	330	ctg022337	6434	1770	3307	scaffold_78	3107833	LG16-21	+	RH2
TiITARs.A032	A1	984	328	ctg022341	7953	2856	4085	scaffold_78	3107833	LG16-21	+	RH2
TiITARs.A033	A1	984	328	ctg022343	5722	1737	3451	scaffold_78	3107833	LG16-21	+	RH2
TiITARs.A034	A1	984	328	ctg022345	3858	1661	2979	scaffold_78	3107833	LG16-21	+	RH2
TiITARs.A035	A1	984	328	ctg022349	9366	4051	5273	scaffold_78	3107833	LG16-21	+	RH2
TiITARs.A036	A1	966	322	ctg022353	10917	4558	5686	scaffold_78	3107833	LG16-21	+	RH2
TiITARs.A037	A1	978	326	ctg022354	14147	2468	3921	scaffold_78	3107833	LG16-21	+	RH2
TiITARs.A038	A1	981	327	ctg022354	14147	9500	10721	scaffold_78	3107833	LG16-21	+	RH2
TiITARs.A039	A1	984	328	ctg022355	8435	5539	6718	scaffold_78	3107833	LG16-21	+	RH2
TiITARs.A040	A1	984	328	ctg022356	12482	2874	4302	scaffold_78	3107833	LG16-21	+	RH2
TiITARp.A021	A1	1023	341	ctg022357	9326	3797	5098	scaffold_78	3107833	LG16-21	+	RH2
TiITARs.A041	A1	984	328	ctg022357	9326	6251	7462	scaffold_78	3107833	LG16-21	+	RH2
TiITARs.A042	A1	960	320	ctg022362	4873	1291	2677	scaffold_78	3107833	LG16-21	+	RH2
TiITARs.A043	A1	624	208	ctg022363	9125	2144	3172	scaffold_78	3107833	LG16-21	+	RH2
TiITARs.A044	A1	975	325	ctg022363	9125	6947	8135	scaffold_78	3107833	LG16-21	+	RH2
TiITARs.A045	A1	975	325	ctg022365	4640	900	2226	scaffold_78	3107833	LG16-21	-	RH2
TiITARs.A046	A1	984	328	ctg022368	18627	6723	8164	scaffold_78	3107833	LG16-21	+	RH2
TiITARs.A047	A1	978	326	ctg022368	18627	11645	12929	scaffold_78	3107833	LG16-21	+	RH2
TiITARe.A008	A1	720	240	ctg022368	18627	17678	18627	scaffold_78	3107833	LG16-21	+	RH2
TiITARe.A009	A1	720	240	ctg022371	1721	772	1721	scaffold_78	3107833	LG16-21	+	RH2
TiITARs.A048	A1	978	326	ctg022375	10343	1572	2770	scaffold_78	3107833	LG16-21	+	RH2
TiITARs.A050	A1	975	325	ctg022377	12552	5195	6356	scaffold_78	3107833	LG16-21	+	RH2
TiITARsp.A049	A1	978	326	ctg022377	12552	10671	11868	scaffold_78	3107833	LG16-21	+	RH2
TiITARs.A051	A1	984	328	ctg022378	7949	5164	6371	scaffold_78	3107833	LG16-21	+	RH2
TiITARs.A052	A1	990	330	ctg022379	10399	7185	8577	scaffold_78	3107833	LG16-21	+	RH2
TiITARp.A023	A1	747	249	ctg022380	10572	8012	10002	scaffold_78	3107833	LG16-21	+	RH2
TiITARs.A053	A1	984	328	ctg022382	16560	12664	13939	scaffold_78	3107833	LG16-21	+	RH2

TilTARe.A010	A1	852	284	ctg022383	6824	5729	6824	scaffold_78	3107833	LG16-21	**+**	RH2
TilTARs.A054	A1	987	329	ctg022383	6824	943	2234	scaffold_78	3107833	LG16-21	**+**	RH2
TilTARe.A011	A1	303	101	ctg022384	2484	3	308	scaffold_78	3107833	LG16-21	**+**	RH2
TilTARs.A055	A1	987	329	ctg022390	16691	881	1991	scaffold_78	3107833	LG16-21	**+**	RH2
TilTARs.A056	A1	987	329	ctg022390	16691	3919	5253	scaffold_78	3107833	LG16-21	**+**	RH2

OR short names	Fam. & Sub-fam	DNA Length	AA Length	Contig	Contig Length	Contig From	Contig To	Scaffold	Scaffold Length
BriTAR.A002	A5	990	330	contig025313	52845	31265	32254	scaffold_18	7756450
BriTAR.A003	A6	975	325	contig029633	42957	40336	39362	scaffold_25	6558494
BriTARe.A005	A6	444	148	contig029635	3022	2578	3021	scaffold_25	6558494
BriTAR.B025	B1	963	321	contig035253	7175	5226	6188	scaffold_31	5992636
BriTARep.A004	A2	444	148	contig086329	1134	519	1132	scaffold_166	1217179
BriTARe.A007	A2	453	151	contig086341	1650	1	664	scaffold_166	1217179
BriTARe.A008	A3	528	176	contig086348	1364	669	1363	scaffold_166	1217179
BriTARe.A001	A4	840	280	contig086354	5102	4963	4124	scaffold_166	1217179
BriTARe.A006	A1	843	281	contig082566	7962	7024	7866	scaffold_149	1395110
BriTARe.A009	A1	720	240	contig109361	5964	5123	5963	scaffold_1762	5964
BriTARe.A010	A1	771	257	contig116966	1143	3	990	scaffold_7877	1143
BriTARe.A011	A1	714	238	contig109362	5957	1	835	scaffold_1763	5957
BriTARp.A013	A1	393	131	contig109362	5957	5596	5956	scaffold_1763	5957
BriTARe.A012	A1	360	120	contig109361	5964	5123	5964	scaffold_1763	5964
BriTARe.A023	A6	261	87	contig029634	1976	1	261	scaffold_26	6558494
BriTARe.A024	A1	177	59	contig084878	4681	7	183	scaffold_161	1312651
BriTARe.B026	B2	870	290	contig011696	2878	2008	2877	scaffold_6	15066089
BriTARe.B027	B3	273	91	contig011701	969	697	969	scaffold_7	15066089
BriTARs.A014	A1	984	328	contig084868	6034	422	1662	scaffold_160	1312651
BriTARs.A015	A1	987	329	contig084876	12737	1012	2242	scaffold_160	1312651
BriTARs.A016	A1	990	330	contig084880	6950	4034	5237	scaffold_160	1312651
BriTARs.A017	A1	972	324	contig084886	3501	1062	2258	scaffold_160	1312651
BriTARs.A018	A1	966	322	contig084887	5446	3606	4631	scaffold_160	1312651
BriTARs.A019	A2	978	326	contig086337	3919	1489	2699	scaffold_166	1217179
BriTARs.A020	A3	981	327	contig086344	7241	5455	6581	scaffold_166	1217179
BriTARs.A021	A2	966	322	contig086351	6649	2142	3276	scaffold_166	1217179
BriTARs.A022	A1	987	329	contig082565	8642	2143	3276	scaffold_149	1395110

OR short names	Fam. & Sub-fam	DNA Length	AA Length	Contig	Contig Length	Contig From	Contig To	Scaffold	Scaffold Length
BurTAR.A001	A6	975	325	contig045302	5480	1796	2770	scaffold_612	299815
BurTARe.A005	A6	366	122	contig045302	5480	5113	5478	scaffold_612	299815
BurTAR.A002	A5	990	330	contig020038	15636	6098	7087	scaffold_128	1563649
BurTAR.B032	B2	945	314	contig006087	12251	5049	5993	scaffold_24	3272143
BurTARe.A003	A1	669	223	contig063687	1382	1	929	scaffold_3654	5883
BurTARe.A004	A3	819	273	contig059670	1963	3	984	scaffold_2074	14995
BurTARs.A016	A2	942	314	contig059673	4536	2062	2883	scaffold_2074	14995
BurTARe.A006	A1	837	279	contig034854	24963	80	916	scaffold_337	666956
BurTARs.A013	A1	987	329	contig061433	9564	989	2213	scaffold_2674	9564
BurTARs.A019	A1	990	330	contig056023	6343	2104	3333	scaffold_1343	56376
BurTARs.A020	A1	984	328	contig056021	5361	3087	4287	scaffold_1343	56376
BurTARs.A021	A1	987	329	contig056020	12247	5811	6966	scaffold_1343	56376
BurTARp.A009	A1	861	287	contig060903	8617	3670	4137	scaffold_2473	10912
BurTARp.A010	A1	813	271	contig057148	6327	6130	4855	scaffold_1542	46672
BurTARp.A011	A1	966	322	contig049535	10386	6840	8023	scaffold_802	179476
BurTARs.A012	A1	978	326	contig061977	5467	2787	4012	scaffold_2902	8394
BurTARs.A014	A1	984	328	contig061091	6806	3066	4266	scaffold_2545	10293
BurTARs.A015	A3	978	326	contig060707	8591	6136	7262	scaffold_2406	11478
BurTARs.A017	A2	987	329	contig057301	8691	6359	7472	scaffold_1575	45224
BurTARs.A018	A1	984	328	contig057145	4393	888	2311	scaffold_1542	46672
BurTARs.A028	A1	975	325	contig057148	6327	1887	2975	scaffold_1542	46672
BurTARs.A022	A2	975	325	contig055697	7670	2009	3225	scaffold_1303	59327
BurTARs.A023	A4	990	330	contig054630	11759	1117	2451	scaffold_1185	77959
BurTARs.A024	A1	984	328	contig049540	6683	4730	6002	scaffold_802	179476
BurTARs.A025	A1	975	325	contig049534	5273	2826	4019	scaffold_802	179476
BurTARs.A026	A1	984	328	contig041024	9427	3662	4892	scaffold_479	443485
BurTARs.A027	A1	987	329	contig034854	24963	5404	6503	scaffold_337	666956
BurTARs.A029	A2	975	325	contig059766	6209	388	1522	scaffold_2104	14652
BurTARs.A030	A2	975	325	contig065494	3633	1329	2472	scaffold_4685	3633
BurTARs.A031	A2	969	323	contig057305	7183	5298	6431	scaffold_1575	45224

OR short names	Fam. & Sub-fam	DNA Length	AA Length	Contig	Contig Length	Contig From	Contig To	Scaffold	Scaffold Length
NyeTAR.A004	A5	990	330	contig032272	45957	25441	26430	scaffold_133	1850505
NyeTAR.A005	A6	975	325	contig038663	20797	950	1924	scaffold_193	1173042
NyeTARp.A031	A6	975	325	contig038663	20797	4269	5242	scaffold_193	1173042
NyeTAR.B030	B2	942	314	contig052987	11035	1043	1984	scaffold_490	219585
NyeTARe.A001	A2	234	78	contig057511	1488	991	1486	scaffold_858	53252
NyeTARe.A003	A1	810	270	contig064861	2180	121	930	scaffold_4043	2180
NyeTARe.A006	A1	513	171	contig066211	1392	1	945	scaffold_5393	1392
NyeTARe.A007	A1	780	260	contig046002	1476	1	780	scaffold_295	622731
NyeTARs.A017	A3	981	327	contig046007	3028	1321	2494	scaffold_295	622731
NyeTARs.A019	A1	975	325	contig046010	3654	1464	2553	scaffold_295	622731
NyeTARs.A021	A1	984	328	contig046013	4647	3143	4362	scaffold_295	622731
NyeTARe.A008	A1	540	180	contig046003	1192	652	1192	scaffold_295	622731
NyeTARs.A024	A2	984	328	contig046014	3301	796	2025	scaffold_295	622731
NyeTARe.A009	A2	849	283	contig051547	7372	6360	7371	scaffold_431	306205
NyeTARe.A010	A2	309	103	contig063978	1482	1	309	scaffold_3406	3794
NyeTARs.A028	A1	990	330	contig042499	10941	9078	10182	scaffold_238	876280
NyeTARf.A011	A4	945	315	contig042497	16004	14601	15461	scaffold_238	876280
NyeTARp.A012	A2	975	325	contig058003	4885	1663	2873	scaffold_947	47935
NyeTARsP.A020	A3	978	326	contig057515	5985	4609	5750	scaffold_857	53252
NyeTARs.A013	A1	987	329	contig035375	14262	10993	12101	scaffold_159	1465909
NyeTARs.A014	A1	987	329	contig035376	9062	6029	7139	scaffold_159	1465909
NyeTARs.A015	A1	987	329	contig035381	6387	1857	2968	scaffold_159	1465909
NyeTARs.A018	A3	981	327	contig060105	6037	3795	4924	scaffold_1502	10601
NyeTARs.A022	A1	963	321	contig062039	3411	2059	3241	scaffold_2325	6191
NyeTARs.A023	A1	990	330	contig035377	5025	1542	2863	scaffold_159	1465909
NyeTARs.A025	A1	987	329	contig058002	9966	7309	8493	scaffold_947	47935
NyeTARs.A026	A2	990	330	contig045999	4861	1635	2873	scaffold_295	622731
NyeTARs.A027	A2	969	323	contig060292	9954	2878	4014	scaffold_1573	9954
NyeTARs.A029	A3	984	328	contig056200	4220	2052	3321	scaffold_698	90054

OR short names	Fam. & Sub-fam	DNA Length	AA Length	Contig	Contig Length	Contig From	Contig To	Scaffold	Scaffold Length
ZebTAR.A001	A6	978	325	contig040586	14923	2922	3899	scaffold00183	1398115
ZebTAR.A002	A6	978	325	contig040586	14923	6242	7219	scaffold00183	1398115
ZebTAR.A003	A5	993	330	contig003909	19494	14515	15507	scaffold00006	8803375
ZebTAR.B029	B2	945	314	contig033536	3835	1998	2942	scaffold00127	2089167
ZebTARe.A004	A3	543	181	contig063680	1644	3	694	scaffold01051	44567
ZebTARe.A005	A2	588	196	contig061509	1024	2	589	scaffold00685	104865
ZebTARe.A006	A1	504	168	contig061414	1394	1	504	scaffold00676	96250
ZebTARs.A020	A1	990	329	contig061417	4739	505	1708	scaffold00676	96250
ZebTARs.A021	A1	984	327	contig061410	4785	2260	3485	scaffold00676	96250
ZebTARe.A007	A2	381	127	contig053136	2491	2	382	scaffold00347	541917
ZebTARs.A028	A2	978	325	contig053145	2903	1238	2377	scaffold00347	541917
ZebTARe.A008	A2	324	108	contig053129	1048	1	534	scaffold00194	1095390
ZebTARep.A009	A1	429	143	contig030465	8200	1	912	scaffold00068	3427994
ZebTARep.A010	A1	399	133	contig067676	3294	9	407	scaffold02965	3294
ZebTARp.A012	A1	813	271	contig067676	3294	2392	3204	scaffold02965	3294
ZebTARp.A013	A1	810	270	contig030459	7445	7055	6248	scaffold00108	2324981
ZebTARs.A029	A1	990	329	contig030440	3790	374	1483	scaffold00108	2324981
ZebTARp.A030	A1	657	219	contig030469	1433	983	322	scaffold00108	2324981
ZebTARs.A024	A1	990	329	contig030471	9661	4832	6061	scaffold00108	2324981
ZebTARs.A025	A1	981	326	contig030464	4607	1666	2755	scaffold00108	2324981
ZebTARs.A026	A1	990	329	contig030445	8085	444	1554	scaffold00108	2324981
ZebTARs.A014	A1	990	329	contig066890	3962	529	1850	scaffold02487	3962
ZebTARs.A015	A1	987	328	contig066691	4173	865	2068	scaffold02369	4173
ZebTARs.A016	A1	978	325	contig066285	2664	621	1807	scaffold02115	4951
ZebTARs.A017	A3	981	326	contig066056	1582	344	1485	scaffold01981	5556
ZebTARs.A018	A1	987	328	contig062677	5038	3457	4741	scaffold00834	57291
ZebTARs.A019	A1	990	329	contig062676	3684	1872	3118	scaffold00834	57291
ZebTARs.A022	A4	993	330	contig059768	8884	3214	4539	scaffold00561	171875
ZebTARs.A023	A2	978	325	contig053139	9206	7266	8476	scaffold00347	541917
ZebTARs.A027	A2	978	325	contig066330	4928	1112	2248	scaffold02144	4928
