# Supplementary material for: Identification and characterization of cichlid TAAR genes and comparison with other teleost TAAR repertoires
Source: BMC Genomics. 2015 Apr 23;16(1):335. doi: 10.1186/s12864-015-1478-4 (PMC4415300; doi:10.1186/s12864-015-1478-4)
Supplement: Additional file 8: — Details of dN/dS ratios calculated for the gene pairs of families A and B. [file 12864_2015_1478_MOESM8_ESM.pdf]

Fam A				
		dN	dS	dN/dS
contig061091-BurTARs.A014	contig066691-ZebTARs.A015	0.003	0.023	0.122
contig055697-BurTARs.A022	contig053139-ZebTARs.A023	0.001	0.012	0.122
contig022390-TilTARs.A055	contig030445-ZebTARs.A026	0.016	0.083	0.190
contig035381-NyeTARs.A015	contig022390-TilTARs.A055	0.016	0.083	0.190
contig041024-BurTARs.A026	contig022320-TilTARs.A059	0.014	0.074	0.191
contig049540-BurTARs.A024	contig056200-NyeTARs.A029	0.001	0.007	0.192
contig045302-BurTAR.A001	contig040586-ZebTAR.A001	0.004	0.022	0.197
contig084880-BriTARs.A016	contig045999-NyeTARs.A026	0.008	0.042	0.201
contig007512-TilTARs.A024	contig066330-ZebTARs.A027	0.087	0.421	0.206
contig055697-BurTARs.A022	contig053145-ZebTARs.A028	0.081	0.386	0.208
contig007512-TilTARs.A024	contig053145-ZebTARs.A028	0.084	0.401	0.208
contig055697-BurTARs.A022	contig066330-ZebTARs.A027	0.085	0.406	0.210
contig057148-BurTARs.A028	contig046010-NyeTARs.A019	0.006	0.027	0.212
contig055697-BurTARs.A022	contig065494-BurTARs.A030	0.085	0.400	0.213
contig086337-BriTARs.A019	contig053145-ZebTARs.A028	0.082	0.381	0.214
contig065494-BurTARs.A030	contig007512-TilTARs.A024	0.089	0.416	0.215
contig086337-BriTARs.A019	contig065494-BurTARs.A030	0.085	0.394	0.215
contig055697-BurTARs.A022	contig059766-BurTARs.A029	0.082	0.381	0.216
contig086337-BriTARs.A019	contig059766-BurTARs.A029	0.082	0.375	0.218
contig007512-TilTARs.A024	contig056134-TilTARs.A058	0.094	0.432	0.218
contig046014-NyeTARs.A024	contig022320-TilTARs.A059	0.013	0.058	0.219
contig053139-ZebTARs.A023	contig053145-ZebTARs.A028	0.080	0.366	0.219
contig055697-BurTARs.A022	contig056134-TilTARs.A058	0.098	0.444	0.220
contig086337-BriTARs.A019	contig066330-ZebTARs.A027	0.089	0.404	0.221
contig059766-BurTARs.A029	contig007512-TilTARs.A024	0.088	0.396	0.221
contig084880-BriTARs.A016	contig056023-BurTARs.A019	0.008	0.038	0.222
contig056134-TilTARs.A058	contig053139-ZebTARs.A023	0.097	0.435	0.223
contig065494-BurTARs.A030	contig053139-ZebTARs.A023	0.085	0.379	0.224
contig053139-ZebTARs.A023	contig066330-ZebTARs.A027	0.088	0.389	0.226
contig059766-BurTARs.A029	contig053139-ZebTARs.A023	0.082	0.361	0.227
contig039639-TilTAR.A001	contig059768-ZebTARs.A022	0.361	1.584	0.228
contig086337-BriTARs.A019	contig056134-TilTARs.A058	0.097	0.425	0.228
contig049540-BurTARs.A024	contig062677-ZebTARs.A018	0.004	0.018	0.229
contig054630-BurTARs.A023	contig039639-TilTAR.A001	0.366	1.590	0.230
contig039640-TilTAR.A003	contig059768-ZebTARs.A022	0.342	1.470	0.233
contig054630-BurTARs.A023	contig039640-TilTAR.A003	0.346	1.475	0.235
contig025313-BriTAR.A002	contig023443-TilTAR.A005	0.010	0.042	0.236
contig025313-BriTAR.A002	contig022334-TilTARs.A030	0.334	1.409	0.237
contig025313-BriTAR.A002	contig059768-ZebTARs.A022	0.284	1.187	0.240
contig023443-TilTAR.A005	contig022334-TilTARs.A030	0.320	1.318	0.243
contig025313-BriTAR.A002	contig062039-NyeTARs.A022	0.279	1.143	0.244
contig032272-NyeTAR.A004	contig059768-ZebTARs.A022	0.289	1.186	0.244
contig022334-TilTARs.A030	contig003909-ZebTAR.A003	0.338	1.387	0.244
contig032272-NyeTAR.A004	contig022334-TilTARs.A030	0.343	1.389	0.247
contig003909-ZebTAR.A003	contig059768-ZebTARs.A022	0.289	1.169	0.247
contig025313-BriTAR.A002	contig054630-BurTARs.A023	0.286	1.152	0.249
contig040586-ZebTAR.A002	contig059768-ZebTARs.A022	0.376	1.503	0.250
contig059673-BurTARs.A016	contig007520-TilTARs.A025	0.029	0.116	0.251
contig065494-BurTARs.A030	contig066330-ZebTARs.A027	0.003	0.011	0.252
contig054630-BurTARs.A023	contig040586-ZebTAR.A002	0.380	1.508	0.252
contig020038-BurTAR.A002	contig059768-ZebTARs.A022	0.291	1.154	0.252

contig020038-BurTAR.A002	contig022334-TilTARs.A030	0.345	1.366	0.253
contig054630-BurTARs.A023	contig032272-NyeTAR.A004	0.291	1.151	0.253
contig039642-TilTAR.A004	contig059768-ZebTARs.A022	0.364	1.437	0.253
contig057145-BurTARs.A018	contig023443-TilTAR.A005	0.343	1.349	0.254
contig062039-NyeTARs.A022	contig023443-TilTAR.A005	0.286	1.120	0.255
contig056200-NyeTARs.A029	contig062677-ZebTARs.A018	0.003	0.011	0.255
contig062039-NyeTARs.A022	contig003909-ZebTAR.A003	0.288	1.125	0.256
contig054630-BurTARs.A023	contig039642-TilTAR.A004	0.369	1.437	0.257
contig054630-BurTARs.A023	contig003909-ZebTAR.A003	0.291	1.135	0.257
contig039640-TilTAR.A002	contig059768-ZebTARs.A022	0.369	1.435	0.257
contig054630-BurTARs.A023	contig039640-TilTAR.A002	0.373	1.439	0.259
contig032272-NyeTAR.A004	contig062039-NyeTARs.A022	0.289	1.113	0.260
contig020038-BurTAR.A002	contig062039-NyeTARs.A022	0.291	1.116	0.261
contig041024-BurTARs.A026	contig046014-NyeTARs.A024	0.010	0.038	0.261
contig039639-TilTAR.A001	contig022354-TilTARs.A038	0.421	1.612	0.261
contig029633-BriTAR.A003	contig059768-ZebTARs.A022	0.391	1.495	0.262
contig020038-BurTAR.A002	contig054630-BurTARs.A023	0.293	1.121	0.262
contig039640-TilTAR.A003	contig023443-TilTAR.A005	0.301	1.147	0.262
contig025313-BriTAR.A002	contig057145-BurTARs.A018	0.359	1.368	0.263
contig057145-BurTARs.A018	contig039640-TilTAR.A003	0.404	1.536	0.263
contig029633-BriTAR.A003	contig054630-BurTARs.A023	0.395	1.499	0.264
contig023443-TilTAR.A005	contig059768-ZebTARs.A022	0.292	1.106	0.264
contig045302-BurTAR.A001	contig059768-ZebTARs.A022	0.357	1.350	0.264
contig061977-BurTARs.A012	contig061410-ZebTARs.A021	0.003	0.011	0.265
contig039640-TilTAR.A003	contig003909-ZebTAR.A003	0.292	1.094	0.267
contig025313-BriTAR.A002	contig039640-TilTAR.A003	0.298	1.119	0.267
contig041024-BurTARs.A026	contig023443-TilTAR.A005	0.343	1.285	0.267
contig045302-BurTAR.A001	contig054630-BurTARs.A023	0.361	1.350	0.268
contig084886-BriTARs.A017	contig039639-TilTAR.A001	0.422	1.573	0.268
contig056020-BurTARs.A021	contig022355-TilTARs.A039	0.020	0.076	0.269
contig055697-BurTARs.A022	contig059768-ZebTARs.A022	0.292	1.087	0.269
contig054630-BurTARs.A023	contig023443-TilTAR.A005	0.294	1.090	0.270
contig046014-NyeTARs.A024	contig023443-TilTAR.A005	0.351	1.302	0.270
contig039642-TilTAR.A004	contig023443-TilTAR.A005	0.314	1.164	0.270
contig057145-BurTARs.A018	contig032272-NyeTAR.A004	0.367	1.360	0.270
contig025313-BriTAR.A002	contig022378-TilTARs.A051	0.294	1.086	0.271
contig035381-NyeTARs.A015	contig023443-TilTAR.A005	0.299	1.106	0.271
contig039640-TilTAR.A002	contig023443-TilTAR.A005	0.325	1.200	0.271
contig039639-TilTAR.A001	contig023443-TilTAR.A005	0.317	1.165	0.272
contig059768-ZebTARs.A022	contig053145-ZebTARs.A028	0.288	1.057	0.272
contig023443-TilTAR.A005	contig022378-TilTARs.A051	0.291	1.067	0.272
contig059766-BurTARs.A029	contig059768-ZebTARs.A022	0.292	1.072	0.273
contig038663-NyeTAR.A005	contig059768-ZebTARs.A022	0.364	1.332	0.273
contig055697-BurTARs.A022	contig054630-BurTARs.A023	0.297	1.087	0.273
contig066056-ZebTARs.A017	contig053139-ZebTARs.A023	0.254	0.930	0.273
contig059768-ZebTARs.A022	contig053139-ZebTARs.A023	0.297	1.086	0.273
contig023443-TilTAR.A005	contig030445-ZebTARs.A026	0.295	1.078	0.273
contig046010-NyeTARs.A019	contig022357-TilTARs.A041	0.103	0.377	0.274
contig020038-BurTAR.A002	contig039640-TilTAR.A003	0.300	1.096	0.274
contig082565-BriTARs.A022	contig023443-TilTAR.A005	0.298	1.087	0.274
contig057145-BurTARs.A018	contig039639-TilTAR.A001	0.411	1.500	0.274
contig057145-BurTARs.A018	contig003909-ZebTAR.A003	0.372	1.358	0.274
contig057148-BurTARs.A028	contig022357-TilTARs.A041	0.106	0.388	0.274

contig040586-ZebTAR.A001	contig059768-ZebTARs.A022	0.358	1.303	0.274
contig025313-BriTAR.A002	contig039642-TilTAR.A004	0.312	1.135	0.275
contig057148-BurTARs.A028	contig039639-TilTAR.A001	0.427	1.554	0.275
contig023443-TilTAR.A005	contig003909-ZebTAR.A003	0.017	0.062	0.275
contig086337-BriTARs.A019	contig066056-ZebTARs.A017	0.257	0.933	0.275
contig025313-BriTAR.A002	contig039640-TilTAR.A002	0.323	1.170	0.276
contig060707-BurTARs.A015	contig060105-NyeTARs.A018	0.004	0.015	0.276
contig054630-BurTARs.A023	contig038663-NyeTAR.A005	0.368	1.332	0.276
contig025313-BriTAR.A002	contig039639-TilTAR.A001	0.314	1.136	0.276
contig020038-BurTAR.A002	contig057145-BurTARs.A018	0.372	1.343	0.277
contig054630-BurTARs.A023	contig053139-ZebTARs.A023	0.301	1.086	0.277
contig007512-TilTARs.A024	contig059768-ZebTARs.A022	0.294	1.061	0.278
contig025313-BriTAR.A002	contig040586-ZebTAR.A001	0.315	1.134	0.278
contig054630-BurTARs.A023	contig040586-ZebTAR.A001	0.362	1.303	0.278
contig054630-BurTARs.A023	contig053145-ZebTARs.A028	0.294	1.057	0.278
contig054630-BurTARs.A023	contig059766-BurTARs.A029	0.299	1.072	0.278
contig039642-TilTAR.A004	contig003909-ZebTAR.A003	0.309	1.109	0.279
contig025313-BriTAR.A002	contig022341-TilTARs.A032	0.361	1.293	0.279
contig035376-NyeTARs.A014	contig023443-TilTAR.A005	0.304	1.090	0.279
contig032272-NyeTAR.A004	contig039640-TilTAR.A003	0.299	1.070	0.280
contig086337-BriTARs.A019	contig059768-ZebTARs.A022	0.299	1.071	0.280
contig039639-TilTAR.A001	contig030464-ZebTARs.A025	0.439	1.571	0.280
contig039640-TilTAR.A002	contig003909-ZebTAR.A003	0.320	1.144	0.280
contig022343-TilTARs.A033	contig022390-TilTARs.A055	0.183	0.652	0.280
contig061977-BurTARs.A012	contig023443-TilTAR.A005	0.286	1.019	0.280
contig046013-NyeTARs.A021	contig066691-ZebTARs.A015	0.014	0.050	0.280
contig039639-TilTAR.A001	contig003909-ZebTAR.A003	0.312	1.111	0.281
contig061091-BurTARs.A014	contig046013-NyeTARs.A021	0.014	0.050	0.281
contig055697-BurTARs.A022	contig066056-ZebTARs.A017	0.254	0.905	0.281
contig040586-ZebTAR.A001	contig003909-ZebTAR.A003	0.313	1.116	0.281
contig066330-ZebTARs.A027	contig053145-ZebTARs.A028	0.009	0.031	0.281
contig057145-BurTARs.A018	contig040586-ZebTAR.A002	0.416	1.478	0.281
contig020038-BurTAR.A002	contig039642-TilTAR.A004	0.313	1.112	0.282
contig025313-BriTAR.A002	contig061417-ZebTARs.A020	0.361	1.280	0.282
contig059673-BurTARs.A016	contig007524-TilTARs.A026	0.015	0.052	0.282
contig039640-TilTAR.A002	contig022354-TilTARs.A038	0.412	1.459	0.282
contig025313-BriTAR.A002	contig038663-NyeTAR.A005	0.319	1.132	0.282
contig020038-BurTAR.A002	contig039640-TilTAR.A002	0.324	1.146	0.283
contig046013-NyeTARs.A021	contig022353-TilTARs.A036	0.045	0.159	0.283
contig023443-TilTAR.A005	contig040586-ZebTAR.A002	0.317	1.117	0.284
contig035377-NyeTARs.A023	contig023443-TilTAR.A005	0.323	1.140	0.284
contig020038-BurTAR.A002	contig039639-TilTAR.A001	0.316	1.113	0.284
contig039640-TilTAR.A003	contig022354-TilTARs.A038	0.404	1.425	0.284
contig020038-BurTAR.A002	contig040586-ZebTAR.A001	0.318	1.119	0.284
contig084886-BriTARs.A017	contig039640-TilTAR.A002	0.412	1.452	0.284
contig025313-BriTAR.A002	contig035377-NyeTARs.A023	0.333	1.170	0.284
contig046010-NyeTARs.A019	contig039639-TilTAR.A001	0.431	1.515	0.284
contig022354-TilTARs.A038	contig040586-ZebTAR.A002	0.428	1.504	0.284
contig022378-TilTARs.A051	contig003909-ZebTAR.A003	0.301	1.056	0.285
contig054630-BurTARs.A023	contig030445-ZebTARs.A026	0.322	1.128	0.285
contig086337-BriTARs.A019	contig054630-BurTARs.A023	0.306	1.071	0.286
contig022357-TilTARs.A041	contig030464-ZebTARs.A025	0.108	0.379	0.286
contig054630-BurTARs.A023	contig035381-NyeTARs.A015	0.318	1.109	0.287

contig057145-BurTARs.A018	contig039642-TilTAR.A004	0.408	1.421	0.287
contig061977-BurTARs.A012	contig022379-TilTARs.A052	0.017	0.058	0.287
contig032272-NyeTAR.A004	contig040586-ZebTAR.A001	0.316	1.099	0.287
contig062039-NyeTARs.A022	contig039640-TilTAR.A003	0.339	1.181	0.287
contig025313-BriTAR.A002	contig035381-NyeTARs.A015	0.317	1.102	0.288
contig054630-BurTARs.A023	contig035376-NyeTARs.A014	0.331	1.149	0.288
contig020038-BurTAR.A002	contig038663-NyeTAR.A005	0.322	1.117	0.288
contig025313-BriTAR.A002	contig040586-ZebTAR.A002	0.314	1.090	0.288
contig022337-TilTARs.A031	contig022343-TilTARs.A033	0.028	0.096	0.289
contig054630-BurTARs.A023	contig007512-TilTARs.A024	0.306	1.059	0.289
contig025313-BriTAR.A002	contig046014-NyeTARs.A024	0.366	1.267	0.289
contig023443-TilTAR.A005	contig022320-TilTARs.A059	0.344	1.192	0.289
contig059768-ZebTARs.A022	contig030445-ZebTARs.A026	0.316	1.093	0.289
contig039639-TilTAR.A001	contig066056-ZebTARs.A017	0.385	1.332	0.289
contig084886-BriTARs.A017	contig039640-TilTAR.A003	0.404	1.399	0.289
contig032272-NyeTAR.A004	contig022378-TilTARs.A051	0.302	1.045	0.289
contig023443-TilTAR.A005	contig040586-ZebTAR.A001	0.317	1.097	0.289
contig038663-NyeTAR.A005	contig003909-ZebTAR.A003	0.318	1.098	0.289
contig023443-TilTAR.A005	contig022382-TilTARs.A053	0.320	1.106	0.290
contig057148-BurTARs.A028	contig022354-TilTARs.A038	0.022	0.074	0.290
contig020038-BurTAR.A002	contig022378-TilTARs.A051	0.304	1.048	0.290
contig035381-NyeTARs.A015	contig059768-ZebTARs.A022	0.312	1.076	0.290
contig025313-BriTAR.A002	contig066056-ZebTARs.A017	0.318	1.094	0.290
contig025313-BriTAR.A002	contig030445-ZebTARs.A026	0.312	1.075	0.290
contig025313-BriTAR.A002	contig041024-BurTARs.A026	0.358	1.233	0.290
contig056200-NyeTARs.A029	contig039639-TilTAR.A001	0.404	1.388	0.291
contig035381-NyeTARs.A015	contig003909-ZebTAR.A003	0.318	1.095	0.291
contig023443-TilTAR.A005	contig061417-ZebTARs.A020	0.347	1.192	0.291
contig025313-BriTAR.A002	contig061977-BurTARs.A012	0.298	1.024	0.291
contig025313-BriTAR.A002	contig082565-BriTARs.A022	0.315	1.083	0.291
contig057145-BurTARs.A018	contig039640-TilTAR.A002	0.408	1.401	0.291
contig086337-BriTARs.A019	contig060707-BurTARs.A015	0.264	0.905	0.291
contig032272-NyeTAR.A004	contig039642-TilTAR.A004	0.313	1.077	0.291
contig082565-BriTARs.A022	contig054630-BurTARs.A023	0.324	1.113	0.291
contig039642-TilTAR.A004	contig040586-ZebTAR.A002	0.017	0.060	0.291
contig035376-NyeTARs.A014	contig059768-ZebTARs.A022	0.325	1.114	0.292
contig032272-NyeTAR.A004	contig038663-NyeTAR.A005	0.320	1.097	0.292
contig039640-TilTAR.A002	contig022363-TilTARs.A043	0.423	1.449	0.292
contig060707-BurTARs.A015	contig053139-ZebTARs.A023	0.262	0.897	0.292
contig032272-NyeTAR.A004	contig035381-NyeTARs.A015	0.317	1.086	0.292
contig007520-TilTARs.A025	contig059768-ZebTARs.A022	0.333	1.140	0.292
contig032272-NyeTAR.A004	contig039640-TilTAR.A002	0.324	1.109	0.292
contig039640-TilTAR.A003	contig022365-TilTARs.A045	0.406	1.388	0.292
contig040586-ZebTAR.A002	contig003909-ZebTAR.A003	0.312	1.066	0.293
contig046014-NyeTARs.A024	contig003909-ZebTAR.A003	0.376	1.284	0.293
contig045302-BurTAR.A001	contig003909-ZebTAR.A003	0.319	1.089	0.293
contig032272-NyeTAR.A004	contig039639-TilTAR.A001	0.316	1.078	0.293
contig039642-TilTAR.A004	contig066056-ZebTARs.A017	0.386	1.317	0.293
contig003909-ZebTAR.A003	contig061417-ZebTARs.A020	0.370	1.261	0.294
contig003909-ZebTAR.A003	contig030445-ZebTARs.A026	0.314	1.068	0.294
contig049534-BurTARs.A025	contig022377-TilTARs.A050	0.024	0.083	0.294
contig025313-BriTAR.A002	contig022363-TilTARs.A043	0.378	1.286	0.294
contig032272-NyeTAR.A004	contig061417-ZebTARs.A020	0.370	1.260	0.294

contig038663-NyeTAR.A005	contig023443-TilTAR.A005	0.322	1.095	0.294
contig032272-NyeTAR.A004	contig062677-ZebTARs.A018	0.348	1.183	0.294
contig032272-NyeTAR.A004	contig023443-TilTAR.A005	0.017	0.058	0.294
contig082565-BriTARs.A022	contig003909-ZebTAR.A003	0.317	1.076	0.294
contig025313-BriTAR.A002	contig045302-BurTAR.A001	0.318	1.079	0.294
contig039640-TilTAR.A003	contig022363-TilTARs.A043	0.415	1.409	0.294
contig025313-BriTAR.A002	contig062677-ZebTARs.A018	0.342	1.160	0.295
contig022377-TilTARs.A050	contig066285-ZebTARs.A016	0.026	0.088	0.295
contig082565-BriTARs.A022	contig059768-ZebTARs.A022	0.318	1.079	0.295
contig041024-BurTARs.A026	contig003909-ZebTAR.A003	0.368	1.248	0.295
contig007512-TilTARs.A024	contig066056-ZebTARs.A017	0.263	0.891	0.295
contig029633-BriTAR.A003	contig066056-ZebTARs.A017	0.402	1.362	0.295
contig032272-NyeTAR.A004	contig030445-ZebTARs.A026	0.313	1.059	0.295
contig045302-BurTAR.A001	contig038663-NyeTAR.A005	0.009	0.029	0.295
contig025313-BriTAR.A002	contig056021-BurTARs.A020	0.363	1.228	0.295
contig049540-BurTARs.A024	contig039639-TilTAR.A001	0.400	1.354	0.295
contig003909-ZebTAR.A003	contig062677-ZebTARs.A018	0.344	1.164	0.295
contig082565-BriTARs.A022	contig032272-NyeTAR.A004	0.316	1.068	0.296
contig057148-BurTARs.A028	contig039640-TilTAR.A003	0.409	1.383	0.296
contig061433-BurTARs.A013	contig022324-TilTARs.A027	0.028	0.093	0.296
contig020038-BurTAR.A002	contig040586-ZebTAR.A002	0.316	1.068	0.296
contig061977-BurTARs.A012	contig032272-NyeTAR.A004	0.301	1.018	0.296
contig041024-BurTARs.A026	contig039639-TilTAR.A001	0.401	1.354	0.296
contig025313-BriTAR.A002	contig022345-TilTARs.A034	0.350	1.183	0.296
contig034854-BurTARs.A027	contig023443-TilTAR.A005	0.322	1.089	0.296
contig029633-BriTAR.A003	contig023443-TilTAR.A005	0.332	1.121	0.296
contig045302-BurTAR.A001	contig020038-BurTAR.A002	0.323	1.091	0.296
contig061977-BurTARs.A012	contig003909-ZebTAR.A003	0.301	1.017	0.296
contig023443-TilTAR.A005	contig061410-ZebTARs.A021	0.290	0.978	0.296
contig025313-BriTAR.A002	contig035376-NyeTARs.A014	0.322	1.086	0.297
contig039639-TilTAR.A001	contig062677-ZebTARs.A018	0.402	1.355	0.297
contig054630-BurTARs.A023	contig007520-TilTARs.A025	0.340	1.143	0.297
contig032272-NyeTAR.A004	contig022382-TilTARs.A053	0.336	1.132	0.297
contig056200-NyeTARs.A029	contig039640-TilTAR.A002	0.390	1.313	0.297
contig025313-BriTAR.A002	contig022382-TilTARs.A053	0.330	1.110	0.297
contig039640-TilTAR.A002	contig066056-ZebTARs.A017	0.393	1.321	0.297
contig023443-TilTAR.A005	contig062677-ZebTARs.A018	0.335	1.127	0.298
contig039639-TilTAR.A001	contig022382-TilTARs.A053	0.395	1.327	0.298
contig041024-BurTARs.A026	contig022341-TilTARs.A032	0.110	0.368	0.298
contig032272-NyeTAR.A004	contig046014-NyeTARs.A024	0.376	1.262	0.298
contig032272-NyeTAR.A004	contig003909-ZebTAR.A003	0.006	0.019	0.298
contig022382-TilTARs.A053	contig003909-ZebTAR.A003	0.332	1.114	0.298
contig022334-TilTARs.A030	contig022375-TilTARs.A048	0.107	0.357	0.299
contig046010-NyeTARs.A019	contig022354-TilTARs.A038	0.019	0.062	0.299
contig035376-NyeTARs.A014	contig022390-TilTARs.A055	0.032	0.106	0.299
contig039642-TilTAR.A004	contig022363-TilTARs.A043	0.421	1.408	0.299
contig025313-BriTAR.A002	contig022320-TilTARs.A059	0.359	1.199	0.299
contig023443-TilTAR.A005	contig022341-TilTARs.A032	0.356	1.187	0.300
contig020038-BurTAR.A002	contig035381-NyeTARs.A015	0.319	1.064	0.300
contig035377-NyeTARs.A023	contig003909-ZebTAR.A003	0.340	1.134	0.300
contig045302-BurTAR.A001	contig032272-NyeTAR.A004	0.321	1.072	0.300
contig023443-TilTAR.A005	contig062676-ZebTARs.A019	0.299	0.999	0.300
contig056134-TilTARs.A058	contig059768-ZebTARs.A022	0.311	1.037	0.300

contig023443-TilTAR.A005	contig066056-ZebTARs.A017	0.319	1.065	0.300
contig035376-NyeTARs.A014	contig003909-ZebTAR.A003	0.324	1.079	0.300
contig041024-BurTARs.A026	contig032272-NyeTAR.A004	0.368	1.228	0.300
contig056021-BurTARs.A020	contig023443-TilTAR.A005	0.349	1.162	0.300
contig032272-NyeTAR.A004	contig035377-NyeTARs.A023	0.340	1.133	0.300
contig039640-TilTAR.A003	contig030464-ZebTARs.A025	0.419	1.397	0.300
contig039640-TilTAR.A002	contig030464-ZebTARs.A025	0.429	1.430	0.300
contig020038-BurTAR.A002	contig046014-NyeTARs.A024	0.381	1.267	0.300
contig040586-ZebTAR.A002	contig066056-ZebTARs.A017	0.388	1.289	0.301
contig060707-BurTARs.A015	contig055697-BurTARs.A022	0.265	0.882	0.301
contig062039-NyeTARs.A022	contig039639-TilTAR.A001	0.350	1.161	0.301
contig039640-TilTAR.A003	contig022354-TilTARs.A037	0.373	1.238	0.301
contig020038-BurTAR.A002	contig061417-ZebTARs.A020	0.375	1.244	0.301
contig086351-BriTARs.A021	contig039639-TilTAR.A001	0.447	1.483	0.301
contig032272-NyeTAR.A004	contig035376-NyeTARs.A014	0.323	1.071	0.301
contig039642-TilTAR.A004	contig022354-TilTARs.A038	0.426	1.413	0.301
contig056200-NyeTARs.A029	contig039640-TilTAR.A003	0.382	1.268	0.302
contig049540-BurTARs.A024	contig039640-TilTAR.A002	0.387	1.282	0.302
contig062039-NyeTARs.A022	contig059768-ZebTARs.A022	0.314	1.039	0.302
contig032272-NyeTAR.A004	contig056200-NyeTARs.A029	0.349	1.156	0.302
contig045302-BurTAR.A001	contig023443-TilTAR.A005	0.323	1.069	0.302
contig020038-BurTAR.A002	contig041024-BurTARs.A026	0.372	1.232	0.302
contig020038-BurTAR.A002	contig030445-ZebTARs.A026	0.314	1.038	0.302
contig022341-TilTARs.A032	contig003909-ZebTAR.A003	0.371	1.227	0.302
contig025313-BriTAR.A002	contig056200-NyeTARs.A029	0.343	1.134	0.302
contig084868-BriTARs.A014	contig023443-TilTAR.A005	0.307	1.013	0.303
contig039639-TilTAR.A001	contig022334-TilTARs.A030	0.357	1.179	0.303
contig082565-BriTARs.A022	contig020038-BurTAR.A002	0.317	1.046	0.303
contig022363-TilTARs.A043	contig022390-TilTARs.A055	0.207	0.683	0.303
contig056200-NyeTARs.A029	contig003909-ZebTAR.A003	0.345	1.138	0.303
contig039640-TilTAR.A002	contig062677-ZebTARs.A018	0.399	1.315	0.304
contig003909-ZebTAR.A003	contig030440-ZebTARs.A029	0.336	1.107	0.304
contig039640-TilTAR.A003	contig022378-TilTARs.A051	0.346	1.141	0.304
contig056134-TilTARs.A058	contig066056-ZebTARs.A017	0.285	0.937	0.304
contig046010-NyeTARs.A019	contig039640-TilTAR.A003	0.411	1.352	0.304
contig022320-TilTARs.A059	contig003909-ZebTAR.A003	0.369	1.214	0.304
contig039640-TilTAR.A003	contig066056-ZebTARs.A017	0.384	1.265	0.304
contig061091-BurTARs.A014	contig023443-TilTAR.A005	0.381	1.254	0.304
contig054630-BurTARs.A023	contig022378-TilTARs.A051	0.318	1.047	0.304
contig056020-BurTARs.A021	contig041024-BurTARs.A026	0.099	0.326	0.304
contig046010-NyeTARs.A019	contig039640-TilTAR.A002	0.421	1.383	0.304
contig065494-BurTARs.A030	contig059768-ZebTARs.A022	0.297	0.978	0.304
contig023443-TilTAR.A005	contig030440-ZebTARs.A029	0.323	1.060	0.304
contig039639-TilTAR.A001	contig022378-TilTARs.A051	0.352	1.156	0.305
contig059766-BurTARs.A029	contig066330-ZebTARs.A027	0.013	0.042	0.305
contig057148-BurTARs.A028	contig039640-TilTAR.A002	0.417	1.368	0.305
contig025313-BriTAR.A002	contig030440-ZebTARs.A029	0.336	1.104	0.305
contig066056-ZebTARs.A017	contig053145-ZebTARs.A028	0.262	0.859	0.305
contig034854-BurTARs.A027	contig003909-ZebTAR.A003	0.342	1.123	0.305
contig039639-TilTAR.A001	contig022365-TilTARs.A045	0.429	1.408	0.305
contig025313-BriTAR.A002	contig034854-BurTARs.A027	0.340	1.117	0.305
contig023443-TilTAR.A005	contig022345-TilTARs.A034	0.338	1.109	0.305
contig022341-TilTARs.A032	contig022320-TilTARs.A059	0.109	0.356	0.305

contig025313-BriTAR.A002	contig059673-BurTARs.A016	0.371	1.215	0.305
contig039639-TilTAR.A001	contig053145-ZebTARs.A028	0.366	1.199	0.305
contig060292-NyeTARs.A027	contig039640-TilTAR.A002	0.458	1.500	0.305
contig056200-NyeTARs.A029	contig023443-TilTAR.A005	0.337	1.102	0.305
contig086337-BriTARs.A019	contig086344-BriTARs.A020	0.268	0.877	0.306
contig032272-NyeTAR.A004	contig040586-ZebTAR.A002	0.316	1.035	0.306
contig049540-BurTARs.A024	contig039640-TilTAR.A003	0.379	1.239	0.306
contig022341-TilTARs.A032	contig022390-TilTARs.A055	0.213	0.697	0.306
contig059766-BurTARs.A029	contig066056-ZebTARs.A017	0.260	0.849	0.306
contig054630-BurTARs.A023	contig056134-TilTARs.A058	0.317	1.037	0.306
contig062039-NyeTARs.A022	contig039642-TilTAR.A004	0.340	1.110	0.306
contig020038-BurTAR.A002	contig035377-NyeTARs.A023	0.341	1.112	0.306
contig057305-BurTARs.A031	contig039640-TilTAR.A002	0.463	1.509	0.307
contig022354-TilTARs.A038	contig022357-TilTARs.A041	0.096	0.313	0.307
contig086351-BriTARs.A021	contig039640-TilTAR.A002	0.447	1.456	0.307
contig054630-BurTARs.A023	contig062039-NyeTARs.A022	0.320	1.041	0.307
contig039640-TilTAR.A003	contig062677-ZebTARs.A018	0.381	1.240	0.307
contig034854-BurTARs.A027	contig032272-NyeTAR.A004	0.342	1.111	0.307
contig022378-TilTARs.A051	contig059768-ZebTARs.A022	0.312	1.015	0.307
contig022353-TilTARs.A036	contig022390-TilTARs.A055	0.197	0.640	0.308
contig025313-BriTAR.A002	contig061410-ZebTARs.A021	0.302	0.982	0.308
contig060707-BurTARs.A015	contig066056-ZebTARs.A017	0.016	0.051	0.308
contig032272-NyeTAR.A004	contig022341-TilTARs.A032	0.371	1.207	0.308
contig039640-TilTAR.A002	contig022354-TilTARs.A037	0.382	1.242	0.308
contig055697-BurTARs.A022	contig022383-TilTARs.A054	0.274	0.889	0.308
contig039639-TilTAR.A001	contig022363-TilTARs.A043	0.438	1.422	0.308
contig032272-NyeTAR.A004	contig022345-TilTARs.A034	0.358	1.162	0.308
contig039640-TilTAR.A002	contig022382-TilTARs.A053	0.397	1.288	0.308
contig039640-TilTAR.A003	contig022356-TilTARs.A040	0.408	1.322	0.308
contig056021-BurTARs.A020	contig003909-ZebTAR.A003	0.372	1.207	0.309
contig025313-BriTAR.A002	contig029633-BriTAR.A003	0.332	1.076	0.309
contig039639-TilTAR.A001	contig022368-TilTARs.A046	0.418	1.355	0.309
contig029633-BriTAR.A003	contig003909-ZebTAR.A003	0.332	1.077	0.309
contig020038-BurTAR.A002	contig035376-NyeTARs.A014	0.324	1.049	0.309
contig039640-TilTAR.A003	contig022382-TilTARs.A053	0.381	1.235	0.309
contig060707-BurTARs.A015	contig039639-TilTAR.A001	0.404	1.307	0.309
contig039639-TilTAR.A001	contig022354-TilTARs.A037	0.383	1.239	0.309
contig056021-BurTARs.A020	contig032272-NyeTAR.A004	0.373	1.206	0.309
contig059768-ZebTARs.A022	contig066330-ZebTARs.A027	0.298	0.965	0.309
contig022356-TilTARs.A040	contig061417-ZebTARs.A020	0.088	0.285	0.309
contig084886-BriTARs.A017	contig039642-TilTAR.A004	0.427	1.382	0.309
contig086344-BriTARs.A020	contig053139-ZebTARs.A023	0.268	0.868	0.309
contig032272-NyeTAR.A004	contig022320-TilTARs.A059	0.369	1.194	0.309
contig025313-BriTAR.A002	contig066890-ZebTARs.A014	0.337	1.091	0.309
contig055697-BurTARs.A022	contig062039-NyeTARs.A022	0.275	0.889	0.309
contig007524-TilTARs.A026	contig059768-ZebTARs.A022	0.340	1.100	0.309
contig065494-BurTARs.A030	contig066056-ZebTARs.A017	0.265	0.855	0.309
contig032272-NyeTAR.A004	contig030440-ZebTARs.A029	0.335	1.082	0.310
contig022345-TilTARs.A034	contig003909-ZebTAR.A003	0.358	1.157	0.310
contig045302-BurTAR.A001	contig066056-ZebTARs.A017	0.401	1.295	0.310
contig046007-NyeTARs.A017	contig022355-TilTARs.A039	0.024	0.079	0.310
contig039639-TilTAR.A001	contig022320-TilTARs.A059	0.411	1.325	0.310
contig039640-TilTAR.A003	contig022334-TilTARs.A030	0.364	1.174	0.310

contig020038-BurTAR.A002	contig022341-TilTARs.A032	0.375	1.211	0.310
contig059673-BurTARs.A016	contig003909-ZebTAR.A003	0.388	1.252	0.310
contig020038-BurTAR.A002	contig062677-ZebTARs.A018	0.352	1.135	0.310
contig020038-BurTAR.A002	contig061977-BurTARs.A012	0.306	0.987	0.310
contig022383-TilTARs.A054	contig059768-ZebTARs.A022	0.324	1.042	0.311
contig023443-TilTAR.A005	contig066890-ZebTARs.A014	0.330	1.064	0.311
contig054630-BurTARs.A023	contig065494-BurTARs.A030	0.304	0.978	0.311
contig056020-BurTARs.A021	contig046014-NyeTARs.A024	0.101	0.325	0.311
contig086351-BriTARs.A021	contig023443-TilTAR.A005	0.376	1.208	0.311
contig059766-BurTARs.A029	contig039639-TilTAR.A001	0.368	1.183	0.311
contig039640-TilTAR.A003	contig022368-TilTARs.A046	0.403	1.294	0.311
contig049540-BurTARs.A024	contig032272-NyeTAR.A004	0.349	1.123	0.311
contig060292-NyeTARs.A027	contig039639-TilTAR.A001	0.450	1.447	0.311
contig025313-BriTAR.A002	contig022368-TilTARs.A047	0.370	1.188	0.311
contig055697-BurTARs.A022	contig039639-TilTAR.A001	0.390	1.254	0.311
contig025313-BriTAR.A002	contig049540-BurTARs.A024	0.343	1.101	0.311
contig066056-ZebTARs.A017	contig066330-ZebTARs.A027	0.270	0.866	0.311
contig062039-NyeTARs.A022	contig040586-ZebTAR.A001	0.360	1.156	0.312
contig020038-BurTAR.A002	contig022320-TilTARs.A059	0.373	1.198	0.312
contig058002-NyeTARs.A025	contig059768-ZebTARs.A022	0.348	1.118	0.312
contig086337-BriTARs.A019	contig060105-NyeTARs.A018	0.267	0.857	0.312
contig023443-TilTAR.A005	contig066691-ZebTARs.A015	0.386	1.238	0.312
contig020038-BurTAR.A002	contig030440-ZebTARs.A029	0.336	1.078	0.312
contig086351-BriTARs.A021	contig032272-NyeTAR.A004	0.383	1.228	0.312
contig025313-BriTAR.A002	contig058002-NyeTARs.A025	0.339	1.088	0.312
contig059766-BurTARs.A029	contig023443-TilTAR.A005	0.333	1.069	0.312
contig029633-BriTAR.A003	contig020038-BurTAR.A002	0.337	1.079	0.312
contig060707-BurTARs.A015	contig053145-ZebTARs.A028	0.266	0.851	0.312
contig039639-TilTAR.A001	contig022362-TilTARs.A042	0.417	1.336	0.312
contig023443-TilTAR.A005	contig022383-TilTARs.A054	0.304	0.974	0.312
contig023443-TilTAR.A005	contig053145-ZebTARs.A028	0.338	1.082	0.312
contig057305-BurTARs.A031	contig039639-TilTAR.A001	0.455	1.456	0.312
contig062039-NyeTARs.A022	contig007512-TilTARs.A024	0.281	0.899	0.312
contig049540-BurTARs.A024	contig003909-ZebTAR.A003	0.345	1.105	0.312
contig039640-TilTAR.A003	contig022362-TilTARs.A042	0.399	1.276	0.313
contig062039-NyeTARs.A022	contig039640-TilTAR.A002	0.346	1.105	0.313
contig025313-BriTAR.A002	contig022368-TilTARs.A046	0.384	1.226	0.313
contig060105-NyeTARs.A018	contig053139-ZebTARs.A023	0.266	0.849	0.313
contig020038-BurTAR.A002	contig022345-TilTARs.A034	0.361	1.154	0.313
contig029633-BriTAR.A003	contig057145-BurTARs.A018	0.424	1.354	0.313
contig003909-ZebTAR.A003	contig061410-ZebTARs.A021	0.306	0.976	0.313
contig025313-BriTAR.A002	contig022365-TilTARs.A045	0.392	1.251	0.313
contig057148-BurTARs.A028	contig022337-TilTARs.A031	0.125	0.398	0.313
contig055697-BurTARs.A022	contig039640-TilTAR.A003	0.380	1.213	0.313
contig060707-BurTARs.A015	contig059766-BurTARs.A029	0.264	0.841	0.313
contig046010-NyeTARs.A019	contig022337-TilTARs.A031	0.121	0.387	0.313
contig049534-BurTARs.A025	contig041024-BurTARs.A026	0.130	0.415	0.313
contig057305-BurTARs.A031	contig032272-NyeTAR.A004	0.385	1.229	0.313
contig022378-TilTARs.A051	contig040586-ZebTAR.A001	0.356	1.134	0.314
contig020038-BurTAR.A002	contig034854-BurTARs.A027	0.342	1.091	0.314
contig023443-TilTAR.A005	contig022330-TilTARs.A028	0.348	1.109	0.314
contig065494-BurTARs.A030	contig039639-TilTAR.A001	0.369	1.175	0.314
contig054630-BurTARs.A023	contig007524-TilTARs.A026	0.347	1.103	0.314

contig025313-BriTAR.A002	contig061091-BurTARs.A014	0.388	1.233	0.314
contig046014-NyeTARs.A024	contig039639-TilTAR.A001	0.406	1.292	0.314
contig084887-BriTARs.A018	contig023443-TilTAR.A005	0.381	1.213	0.314
contig049540-BurTARs.A024	contig023443-TilTAR.A005	0.337	1.070	0.315
contig020038-BurTAR.A002	contig059673-BurTARs.A016	0.383	1.218	0.315
contig057305-BurTARs.A031	contig023443-TilTAR.A005	0.380	1.209	0.315
contig039639-TilTAR.A001	contig022337-TilTARs.A031	0.430	1.366	0.315
contig022337-TilTARs.A031	contig022390-TilTARs.A055	0.192	0.611	0.315
contig035375-NyeTARs.A013	contig003909-ZebTAR.A003	0.339	1.076	0.315
contig025313-BriTAR.A002	contig022330-TilTARs.A028	0.361	1.146	0.315
contig003909-ZebTAR.A003	contig066056-ZebTARs.A017	0.328	1.040	0.315
contig022363-TilTARs.A043	contig003909-ZebTAR.A003	0.382	1.213	0.315
contig059673-BurTARs.A016	contig023443-TilTAR.A005	0.367	1.165	0.315
contig035375-NyeTARs.A013	contig023443-TilTAR.A005	0.325	1.031	0.316
contig054630-BurTARs.A023	contig066330-ZebTARs.A027	0.305	0.965	0.316
contig039639-TilTAR.A001	contig066330-ZebTARs.A027	0.374	1.185	0.316
contig032272-NyeTAR.A004	contig066056-ZebTARs.A017	0.329	1.044	0.316
contig025313-BriTAR.A002	contig035375-NyeTARs.A013	0.339	1.073	0.316
contig032272-NyeTAR.A004	contig061410-ZebTARs.A021	0.311	0.984	0.316
contig062039-NyeTARs.A022	contig053139-ZebTARs.A023	0.281	0.889	0.316
contig040586-ZebTAR.A001	contig066056-ZebTARs.A017	0.404	1.278	0.316
contig039639-TilTAR.A001	contig022363-TilTARs.A044	0.399	1.264	0.316
contig003909-ZebTAR.A003	contig062676-ZebTARs.A019	0.321	1.016	0.316
contig084886-BriTARs.A017	contig022357-TilTARs.A041	0.107	0.337	0.316
contig057148-BurTARs.A028	contig039642-TilTAR.A004	0.432	1.366	0.316
contig060707-BurTARs.A015	contig056134-TilTARs.A058	0.292	0.922	0.316
contig020038-BurTAR.A002	contig056021-BurTARs.A020	0.377	1.192	0.316
contig056200-NyeTARs.A029	contig039642-TilTAR.A004	0.405	1.281	0.316
contig039640-TilTAR.A003	contig022337-TilTARs.A031	0.413	1.306	0.316
contig039642-TilTAR.A004	contig022334-TilTARs.A030	0.360	1.137	0.317
contig020038-BurTAR.A002	contig022382-TilTARs.A053	0.342	1.079	0.317
contig086351-BriTARs.A021	contig003909-ZebTAR.A003	0.381	1.204	0.317
contig062039-NyeTARs.A022	contig040586-ZebTAR.A002	0.347	1.097	0.317
contig022363-TilTARs.A043	contig040586-ZebTAR.A002	0.422	1.332	0.317
contig054630-BurTARs.A023	contig022383-TilTARs.A054	0.330	1.042	0.317
contig039640-TilTAR.A002	contig022334-TilTARs.A030	0.368	1.160	0.317
contig060707-BurTARs.A015	contig065494-BurTARs.A030	0.269	0.848	0.317
contig084876-BriTARs.A015	contig023443-TilTAR.A005	0.302	0.953	0.317
contig022368-TilTARs.A047	contig003909-ZebTAR.A003	0.377	1.189	0.317
contig041024-BurTARs.A026	contig066285-ZebTARs.A016	0.131	0.413	0.317
contig007520-TilTARs.A025	contig007524-TilTARs.A026	0.019	0.060	0.317
contig023443-TilTAR.A005	contig030471-ZebTARs.A024	0.380	1.199	0.317
contig046014-NyeTARs.A024	contig022390-TilTARs.A055	0.187	0.590	0.317
contig039640-TilTAR.A002	contig022378-TilTARs.A051	0.347	1.093	0.318
contig032272-NyeTAR.A004	contig022368-TilTARs.A047	0.377	1.188	0.318
contig046013-NyeTARs.A021	contig023443-TilTAR.A005	0.386	1.214	0.318
contig022383-TilTARs.A054	contig053139-ZebTARs.A023	0.274	0.862	0.318
contig046007-NyeTARs.A017	contig003909-ZebTAR.A003	0.384	1.208	0.318
contig039642-TilTAR.A004	contig062677-ZebTARs.A018	0.404	1.273	0.318
contig054630-BurTARs.A023	contig058002-NyeTARs.A025	0.355	1.118	0.318
contig060707-BurTARs.A015	contig039640-TilTAR.A002	0.412	1.296	0.318
contig045302-BurTAR.A001	contig022354-TilTARs.A038	0.432	1.360	0.318
contig059673-BurTARs.A016	contig032272-NyeTAR.A004	0.386	1.213	0.318

contig086344-BriTARs.A020	contig055697-BurTARs.A022	0.272	0.854	0.318
contig055697-BurTARs.A022	contig060105-NyeTARs.A018	0.265	0.832	0.318
contig057305-BurTARs.A031	contig003909-ZebTAR.A003	0.384	1.205	0.318
contig066691-ZebTARs.A015	contig030471-ZebTARs.A024	0.062	0.196	0.318
contig084886-BriTARs.A017	contig022390-TilTARs.A055	0.201	0.631	0.318
contig032272-NyeTAR.A004	contig060292-NyeTARs.A027	0.385	1.210	0.318
contig022365-TilTARs.A045	contig003909-ZebTAR.A003	0.396	1.242	0.319
contig038663-NyeTAR.A005	contig066056-ZebTARs.A017	0.406	1.275	0.319
contig023443-TilTAR.A005	contig007512-TilTARs.A024	0.337	1.057	0.319
contig023443-TilTAR.A005	contig022334-TilTARs.A029	0.352	1.103	0.319
contig045302-BurTAR.A001	contig057145-BurTARs.A018	0.425	1.334	0.319
contig060707-BurTARs.A015	contig066330-ZebTARs.A027	0.274	0.858	0.319
contig007512-TilTARs.A024	contig003909-ZebTAR.A003	0.349	1.093	0.319
contig086351-BriTARs.A021	contig045302-BurTAR.A001	0.434	1.360	0.319
contig023443-TilTAR.A005	contig022375-TilTARs.A048	0.345	1.081	0.319
contig060292-NyeTARs.A027	contig023443-TilTAR.A005	0.380	1.191	0.319
contig025313-BriTAR.A002	contig084876-BriTARs.A015	0.314	0.984	0.320
contig025313-BriTAR.A002	contig022334-TilTARs.A029	0.365	1.140	0.320
contig084868-BriTARs.A014	contig003909-ZebTAR.A003	0.329	1.028	0.320
contig060105-NyeTARs.A018	contig053145-ZebTARs.A028	0.265	0.829	0.320
contig058002-NyeTARs.A025	contig003909-ZebTAR.A003	0.352	1.099	0.320
contig060707-BurTARs.A015	contig007512-TilTARs.A024	0.274	0.856	0.320
contig025313-BriTAR.A002	contig062676-ZebTARs.A019	0.315	0.983	0.320
contig039639-TilTAR.A001	contig022341-TilTARs.A032	0.405	1.262	0.320
contig059766-BurTARs.A029	contig003909-ZebTAR.A003	0.338	1.054	0.321
contig086344-BriTARs.A020	contig039639-TilTAR.A001	0.416	1.299	0.321
contig039639-TilTAR.A001	contig053139-ZebTARs.A023	0.395	1.232	0.321
contig029633-BriTAR.A003	contig022354-TilTARs.A038	0.441	1.377	0.321
contig025313-BriTAR.A002	contig022362-TilTARs.A042	0.370	1.154	0.321
contig022354-TilTARs.A038	contig022390-TilTARs.A055	0.182	0.568	0.321
contig003909-ZebTAR.A003	contig053145-ZebTARs.A028	0.343	1.068	0.321
contig032272-NyeTAR.A004	contig035375-NyeTARs.A013	0.338	1.052	0.321
contig003909-ZebTAR.A003	contig053139-ZebTARs.A023	0.349	1.087	0.321
contig049540-BurTARs.A024	contig039642-TilTAR.A004	0.402	1.252	0.321
contig025313-BriTAR.A002	contig059766-BurTARs.A029	0.335	1.043	0.321
contig059766-BurTARs.A029	contig060105-NyeTARs.A018	0.263	0.820	0.321
contig057145-BurTARs.A018	contig040586-ZebTAR.A001	0.428	1.333	0.321
contig032272-NyeTAR.A004	contig022363-TilTARs.A043	0.391	1.217	0.321
contig054630-BurTARs.A023	contig034854-BurTARs.A027	0.340	1.058	0.321
contig040586-ZebTAR.A002	contig030464-ZebTARs.A025	0.457	1.421	0.321
contig039639-TilTAR.A001	contig007512-TilTARs.A024	0.400	1.246	0.321
contig025313-BriTAR.A002	contig053145-ZebTARs.A028	0.339	1.056	0.321
contig054630-BurTARs.A023	contig062676-ZebTARs.A019	0.331	1.030	0.322
contig061433-BurTARs.A013	contig039640-TilTAR.A002	0.441	1.371	0.322
contig023443-TilTAR.A005	contig022365-TilTARs.A045	0.377	1.171	0.322
contig086351-BriTARs.A021	contig040586-ZebTAR.A001	0.437	1.359	0.322
contig062676-ZebTARs.A019	contig053145-ZebTARs.A028	0.273	0.850	0.322
contig022390-TilTARs.A055	contig066285-ZebTARs.A016	0.242	0.753	0.322
contig020038-BurTAR.A002	contig056200-NyeTARs.A029	0.355	1.102	0.322
contig023443-TilTAR.A005	contig022379-TilTARs.A052	0.310	0.962	0.322
contig023443-TilTAR.A005	contig022356-TilTARs.A040	0.346	1.075	0.322
contig029633-BriTAR.A003	contig032272-NyeTAR.A004	0.337	1.045	0.322
contig055697-BurTARs.A022	contig039642-TilTAR.A004	0.390	1.211	0.322

contig032272-NyeTAR.A004	contig053139-ZebTARs.A023	0.351	1.089	0.322
contig039639-TilTAR.A001	contig022349-TilTARs.A035	0.433	1.342	0.322
contig025313-BriTAR.A002	contig066691-ZebTARs.A015	0.392	1.216	0.322
contig039642-TilTAR.A004	contig030464-ZebTARs.A025	0.445	1.380	0.322
contig039640-TilTAR.A003	contig053139-ZebTARs.A023	0.385	1.193	0.323
contig032272-NyeTAR.A004	contig007524-TilTARs.A026	0.361	1.118	0.323
contig022362-TilTARs.A042	contig040586-ZebTAR.A001	0.415	1.284	0.323
contig039640-TilTAR.A002	contig022362-TilTARs.A042	0.412	1.276	0.323
contig022345-TilTARs.A034	contig066056-ZebTARs.A017	0.282	0.873	0.323
contig025313-BriTAR.A002	contig060707-BurTARs.A015	0.329	1.019	0.323
contig020038-BurTAR.A002	contig035375-NyeTARs.A013	0.339	1.048	0.323
contig058002-NyeTARs.A025	contig039639-TilTAR.A001	0.427	1.322	0.323
contig041024-BurTARs.A026	contig059768-ZebTARs.A022	0.361	1.118	0.323
contig039642-TilTAR.A004	contig022382-TilTARs.A053	0.397	1.228	0.323
contig025313-BriTAR.A002	contig084868-BriTARs.A014	0.322	0.997	0.323
contig060292-NyeTARs.A027	contig003909-ZebTAR.A003	0.384	1.187	0.323
contig025313-BriTAR.A002	contig007524-TilTARs.A026	0.351	1.086	0.323
contig060707-BurTARs.A015	contig039642-TilTAR.A004	0.405	1.251	0.323
contig007512-TilTARs.A024	contig022383-TilTARs.A054	0.290	0.895	0.324
contig059766-BurTARs.A029	contig062676-ZebTARs.A019	0.275	0.851	0.324
contig022363-TilTARs.A043	contig040586-ZebTAR.A001	0.428	1.322	0.324
contig032272-NyeTAR.A004	contig046007-NyeTARs.A017	0.390	1.204	0.324
contig057148-BurTARs.A028	contig022320-TilTARs.A059	0.127	0.391	0.324
contig055697-BurTARs.A022	contig039640-TilTAR.A002	0.384	1.187	0.324
contig061433-BurTARs.A013	contig039639-TilTAR.A001	0.456	1.407	0.324
contig034854-BurTARs.A027	contig059768-ZebTARs.A022	0.333	1.028	0.324
contig056020-BurTARs.A021	contig039639-TilTAR.A001	0.435	1.342	0.324
contig022334-TilTARs.A029	contig022356-TilTARs.A040	0.091	0.281	0.324
contig086337-BriTARs.A019	contig022383-TilTARs.A054	0.276	0.852	0.324
contig032272-NyeTAR.A004	contig062676-ZebTARs.A019	0.321	0.989	0.324
contig055697-BurTARs.A022	contig023443-TilTAR.A005	0.342	1.056	0.324
contig025313-BriTAR.A002	contig086351-BriTARs.A021	0.377	1.162	0.324
contig025313-BriTAR.A002	contig022379-TilTARs.A052	0.322	0.993	0.324
contig022341-TilTARs.A032	contig022375-TilTARs.A048	0.122	0.376	0.324
contig023443-TilTAR.A005	contig066330-ZebTARs.A027	0.349	1.077	0.324
contig056020-BurTARs.A021	contig003909-ZebTAR.A003	0.380	1.172	0.325
contig039640-TilTAR.A003	contig007512-TilTARs.A024	0.390	1.202	0.325
contig065494-BurTARs.A030	contig023443-TilTAR.A005	0.342	1.054	0.325
contig023443-TilTAR.A005	contig007524-TilTARs.A026	0.346	1.064	0.325
contig056020-BurTARs.A021	contig032272-NyeTAR.A004	0.380	1.170	0.325
contig086351-BriTARs.A021	contig039640-TilTAR.A003	0.441	1.356	0.325
contig020038-BurTAR.A002	contig022368-TilTARs.A047	0.382	1.174	0.325
contig062676-ZebTARs.A019	contig059768-ZebTARs.A022	0.325	1.000	0.325
contig084876-BriTARs.A015	contig003909-ZebTAR.A003	0.318	0.977	0.325
contig007524-TilTARs.A026	contig003909-ZebTAR.A003	0.363	1.117	0.325
contig039642-TilTAR.A004	contig053145-ZebTARs.A028	0.371	1.140	0.325
contig029633-BriTAR.A003	contig062039-NyeTARs.A022	0.363	1.117	0.325
contig041024-BurTARs.A026	contig039640-TilTAR.A003	0.411	1.263	0.325
contig086337-BriTARs.A019	contig062039-NyeTARs.A022	0.286	0.877	0.325
contig058002-NyeTARs.A025	contig023443-TilTAR.A005	0.336	1.033	0.326
contig025313-BriTAR.A002	contig030471-ZebTARs.A024	0.395	1.212	0.326
contig029633-BriTAR.A003	contig060707-BurTARs.A015	0.421	1.292	0.326
contig020038-BurTAR.A002	contig066056-ZebTARs.A017	0.333	1.022	0.326

contig084887-BriTARs.A018	contig039639-TilTAR.A001	0.429	1.315	0.326
contig025313-BriTAR.A002	contig022356-TilTARs.A040	0.360	1.105	0.326
contig038663-NyeTAR.A005	contig062039-NyeTARs.A022	0.368	1.129	0.326
contig045302-BurTAR.A001	contig022378-TilTARs.A051	0.360	1.103	0.326
contig032272-NyeTAR.A004	contig007512-TilTARs.A024	0.356	1.092	0.326
contig056020-BurTARs.A021	contig039640-TilTAR.A003	0.431	1.321	0.326
contig046010-NyeTARs.A019	contig039642-TilTAR.A004	0.436	1.335	0.327
contig039639-TilTAR.A001	contig022375-TilTARs.A048	0.392	1.200	0.327
contig020038-BurTAR.A002	contig022365-TilTARs.A045	0.400	1.226	0.327
contig055697-BurTARs.A022	contig030445-ZebTARs.A026	0.258	0.791	0.327
contig032272-NyeTAR.A004	contig058002-NyeTARs.A025	0.349	1.069	0.327
contig045302-BurTAR.A001	contig062039-NyeTARs.A022	0.362	1.107	0.327
contig022383-TilTARs.A054	contig003909-ZebTAR.A003	0.324	0.990	0.327
contig020038-BurTAR.A002	contig058002-NyeTARs.A025	0.348	1.064	0.327
contig041024-BurTARs.A026	contig039640-TilTAR.A002	0.405	1.239	0.327
contig025313-BriTAR.A002	contig053139-ZebTARs.A023	0.347	1.063	0.327
contig023443-TilTAR.A005	contig022368-TilTARs.A047	0.363	1.109	0.327
contig061091-BurTARs.A014	contig003909-ZebTAR.A003	0.397	1.215	0.327
contig032272-NyeTAR.A004	contig066890-ZebTARs.A014	0.344	1.050	0.327
contig039640-TilTAR.A002	contig022363-TilTARs.A044	0.393	1.200	0.327
contig025313-BriTAR.A002	contig046007-NyeTARs.A017	0.382	1.168	0.327
contig025313-BriTAR.A002	contig007512-TilTARs.A024	0.353	1.079	0.327
contig025313-BriTAR.A002	contig056134-TilTARs.A058	0.348	1.064	0.327
contig046007-NyeTARs.A017	contig039639-TilTAR.A001	0.429	1.310	0.328
contig022368-TilTARs.A046	contig003909-ZebTAR.A003	0.394	1.203	0.328
contig022365-TilTARs.A045	contig040586-ZebTAR.A002	0.436	1.330	0.328
contig022354-TilTARs.A038	contig040586-ZebTAR.A001	0.428	1.306	0.328
contig022362-TilTARs.A042	contig022390-TilTARs.A055	0.239	0.729	0.328
contig039639-TilTAR.A001	contig022356-TilTARs.A040	0.424	1.294	0.328
contig084876-BriTARs.A015	contig032272-NyeTAR.A004	0.317	0.968	0.328
contig022324-TilTARs.A027	contig022368-TilTARs.A046	0.143	0.437	0.328
contig020038-BurTAR.A002	contig061410-ZebTARs.A021	0.311	0.947	0.328
contig003909-ZebTAR.A003	contig066890-ZebTARs.A014	0.344	1.049	0.328
contig060292-NyeTARs.A027	contig039640-TilTAR.A003	0.450	1.371	0.328
contig007520-TilTARs.A025	contig066056-ZebTARs.A017	0.280	0.853	0.328
contig065494-BurTARs.A030	contig062676-ZebTARs.A019	0.278	0.848	0.328
contig084868-BriTARs.A014	contig032272-NyeTAR.A004	0.328	1.001	0.328
contig054630-BurTARs.A023	contig041024-BurTARs.A026	0.368	1.121	0.328
contig039640-TilTAR.A002	contig053145-ZebTARs.A028	0.365	1.113	0.328
contig038663-NyeTAR.A005	contig022378-TilTARs.A051	0.364	1.108	0.328
contig023443-TilTAR.A005	contig022337-TilTARs.A031	0.377	1.148	0.328
contig029633-BriTAR.A003	contig056200-NyeTARs.A029	0.416	1.268	0.328
contig023443-TilTAR.A005	contig022349-TilTARs.A035	0.385	1.172	0.328
contig054630-BurTARs.A023	contig022334-TilTARs.A030	0.342	1.040	0.328
contig086351-BriTARs.A021	contig039642-TilTAR.A004	0.442	1.347	0.328
contig022337-TilTARs.A031	contig030464-ZebTARs.A025	0.127	0.387	0.328
contig041024-BurTARs.A026	contig053145-ZebTARs.A028	0.302	0.918	0.328
contig038663-NyeTAR.A005	contig022362-TilTARs.A042	0.416	1.268	0.328
contig056020-BurTARs.A021	contig007512-TilTARs.A024	0.312	0.949	0.329
contig022368-TilTARs.A047	contig022320-TilTARs.A059	0.115	0.349	0.329
contig041024-BurTARs.A026	contig057148-BurTARs.A028	0.131	0.398	0.329
contig022334-TilTARs.A030	contig040586-ZebTAR.A002	0.375	1.142	0.329
contig023443-TilTAR.A005	contig022354-TilTARs.A037	0.339	1.032	0.329

contig023443-TilTAR.A005	contig022368-TilTARs.A046	0.376	1.144	0.329
contig084886-BriTARs.A017	contig040586-ZebTAR.A002	0.446	1.356	0.329
contig060105-NyeTARs.A018	contig056134-TilTARs.A058	0.295	0.898	0.329
contig022330-TilTARs.A028	contig003909-ZebTAR.A003	0.371	1.128	0.329
contig032272-NyeTAR.A004	contig022365-TilTARs.A045	0.396	1.202	0.329
contig046007-NyeTARs.A017	contig039640-TilTAR.A003	0.427	1.297	0.329
contig057305-BurTARs.A031	contig039640-TilTAR.A003	0.454	1.379	0.329
contig084887-BriTARs.A018	contig039640-TilTAR.A003	0.420	1.274	0.330
contig032272-NyeTAR.A004	contig022330-TilTARs.A028	0.371	1.126	0.330
contig020038-BurTAR.A002	contig022363-TilTARs.A043	0.397	1.205	0.330
contig065494-BurTARs.A030	contig060105-NyeTARs.A018	0.272	0.826	0.330
contig045302-BurTAR.A001	contig060292-NyeTARs.A027	0.446	1.351	0.330
contig057148-BurTARs.A028	contig046014-NyeTARs.A024	0.131	0.398	0.330
contig003909-ZebTAR.A003	contig030471-ZebTARs.A024	0.405	1.228	0.330
contig046007-NyeTARs.A017	contig023443-TilTAR.A005	0.370	1.120	0.330
contig032272-NyeTAR.A004	contig007520-TilTARs.A025	0.345	1.045	0.330
contig022379-TilTARs.A052	contig003909-ZebTAR.A003	0.326	0.987	0.330
contig086344-BriTARs.A020	contig039642-TilTAR.A004	0.414	1.254	0.330
contig057148-BurTARs.A028	contig040586-ZebTAR.A002	0.453	1.371	0.330
contig060292-NyeTARs.A027	contig040586-ZebTAR.A001	0.454	1.374	0.330
contig022365-TilTARs.A045	contig022320-TilTARs.A059	0.113	0.342	0.331
contig060707-BurTARs.A015	contig062039-NyeTARs.A022	0.315	0.953	0.331
contig057145-BurTARs.A018	contig038663-NyeTAR.A005	0.435	1.316	0.331
contig023443-TilTAR.A005	contig053139-ZebTARs.A023	0.350	1.058	0.331
contig046014-NyeTARs.A024	contig022341-TilTARs.A032	0.114	0.345	0.331
contig025313-BriTAR.A002	contig057305-BurTARs.A031	0.379	1.145	0.331
contig084868-BriTARs.A014	contig054630-BurTARs.A023	0.340	1.026	0.331
contig039639-TilTAR.A001	contig022343-TilTARs.A033	0.414	1.251	0.331
contig023443-TilTAR.A005	contig056134-TilTARs.A058	0.342	1.032	0.331
contig025313-BriTAR.A002	contig022383-TilTARs.A054	0.317	0.959	0.331
contig045302-BurTAR.A001	contig022365-TilTARs.A045	0.408	1.230	0.331
contig065494-BurTARs.A030	contig039642-TilTAR.A004	0.371	1.121	0.331
contig055697-BurTARs.A022	contig062676-ZebTARs.A019	0.282	0.850	0.331
contig039642-TilTAR.A004	contig022378-TilTARs.A051	0.351	1.061	0.331
contig020038-BurTAR.A002	contig049540-BurTARs.A024	0.355	1.070	0.331
contig059766-BurTARs.A029	contig039642-TilTAR.A004	0.373	1.126	0.331
contig020038-BurTAR.A002	contig046007-NyeTARs.A017	0.394	1.190	0.331
contig086344-BriTARs.A020	contig007512-TilTARs.A024	0.283	0.853	0.331
contig060707-BurTARs.A015	contig022345-TilTARs.A034	0.297	0.897	0.331
contig039640-TilTAR.A003	contig053145-ZebTARs.A028	0.371	1.119	0.331
contig022368-TilTARs.A046	contig022390-TilTARs.A055	0.208	0.627	0.331
contig041024-BurTARs.A026	contig022324-TilTARs.A027	0.142	0.429	0.332
contig086351-BriTARs.A021	contig020038-BurTAR.A002	0.386	1.166	0.332
contig056134-TilTARs.A058	contig003909-ZebTAR.A003	0.352	1.060	0.332
contig039642-TilTAR.A004	contig053139-ZebTARs.A023	0.395	1.191	0.332
contig086337-BriTARs.A019	contig039640-TilTAR.A003	0.390	1.176	0.332
contig025313-BriTAR.A002	contig046013-NyeTARs.A021	0.393	1.185	0.332
contig025313-BriTAR.A002	contig022390-TilTARs.A056	0.333	1.003	0.332
contig060707-BurTARs.A015	contig040586-ZebTAR.A002	0.406	1.225	0.332
contig057305-BurTARs.A031	contig040586-ZebTAR.A001	0.458	1.382	0.332
contig060105-NyeTARs.A018	contig066330-ZebTARs.A027	0.277	0.836	0.332
contig020038-BurTAR.A002	contig062676-ZebTARs.A019	0.322	0.970	0.332
contig060105-NyeTARs.A018	contig039639-TilTAR.A001	0.411	1.239	0.332

contig086337-BriTARs.A019	contig039639-TilTAR.A001	0.401	1.209	0.332
contig062676-ZebTARs.A019	contig066330-ZebTARs.A027	0.280	0.843	0.332
contig020038-BurTAR.A002	contig007524-TilTARs.A026	0.359	1.081	0.332
contig022324-TilTARs.A027	contig022320-TilTARs.A059	0.140	0.421	0.332
contig041024-BurTARs.A026	contig059766-BurTARs.A029	0.306	0.920	0.332
contig022334-TilTARs.A030	contig059768-ZebTARs.A022	0.335	1.008	0.332
contig059766-BurTARs.A029	contig032272-NyeTAR.A004	0.340	1.024	0.332
contig022377-TilTARs.A050	contig022320-TilTARs.A059	0.125	0.376	0.333
contig046014-NyeTARs.A024	contig059768-ZebTARs.A022	0.363	1.093	0.333
contig061091-BurTARs.A014	contig032272-NyeTAR.A004	0.397	1.195	0.333
contig032272-NyeTAR.A004	contig022379-TilTARs.A052	0.325	0.977	0.333
contig084887-BriTARs.A018	contig065494-BurTARs.A030	0.319	0.958	0.333
contig025313-BriTAR.A002	contig022349-TilTARs.A035	0.397	1.194	0.333
contig032272-NyeTAR.A004	contig053145-ZebTARs.A028	0.345	1.036	0.333
contig065494-BurTARs.A030	contig053145-ZebTARs.A028	0.011	0.034	0.333
contig007520-TilTARs.A025	contig003909-ZebTAR.A003	0.347	1.043	0.333
contig039642-TilTAR.A004	contig066330-ZebTARs.A027	0.376	1.130	0.333
contig029633-BriTAR.A003	contig049540-BurTARs.A024	0.412	1.239	0.333
contig032272-NyeTAR.A004	contig022368-TilTARs.A046	0.394	1.183	0.333
contig045302-BurTAR.A001	contig057305-BurTARs.A031	0.453	1.359	0.333
contig003909-ZebTAR.A003	contig066330-ZebTARs.A027	0.354	1.062	0.333
contig039640-TilTAR.A002	contig053139-ZebTARs.A023	0.389	1.167	0.333
contig056134-TilTARs.A058	contig062676-ZebTARs.A019	0.294	0.882	0.333
contig039639-TilTAR.A001	contig066285-ZebTARs.A016	0.431	1.294	0.333
contig020038-BurTAR.A002	contig057305-BurTARs.A031	0.389	1.166	0.333
contig049534-BurTARs.A025	contig022345-TilTARs.A034	0.149	0.446	0.333
contig086344-BriTARs.A020	contig062039-NyeTARs.A022	0.323	0.969	0.333
contig039639-TilTAR.A001	contig022377-TilTARs.A050	0.417	1.251	0.333
contig086344-BriTARs.A020	contig039640-TilTAR.A002	0.426	1.278	0.333
contig065494-BurTARs.A030	contig003909-ZebTAR.A003	0.347	1.040	0.333
contig060707-BurTARs.A015	contig023443-TilTAR.A005	0.331	0.992	0.334
contig025313-BriTAR.A002	contig007520-TilTARs.A025	0.340	1.018	0.334
contig022345-TilTARs.A034	contig022377-TilTARs.A050	0.136	0.406	0.334
contig007520-TilTARs.A025	contig056134-TilTARs.A058	0.254	0.762	0.334
contig022356-TilTARs.A040	contig040586-ZebTAR.A002	0.425	1.274	0.334
contig025313-BriTAR.A002	contig066330-ZebTARs.A027	0.351	1.050	0.334
contig022362-TilTARs.A042	contig003909-ZebTAR.A003	0.378	1.133	0.334
contig046010-NyeTARs.A019	contig022320-TilTARs.A059	0.125	0.374	0.334
contig041024-BurTARs.A026	contig022356-TilTARs.A040	0.100	0.298	0.334
contig039642-TilTAR.A004	contig022354-TilTARs.A037	0.384	1.151	0.334
contig055697-BurTARs.A022	contig022378-TilTARs.A051	0.279	0.835	0.334
contig025313-BriTAR.A002	contig065494-BurTARs.A030	0.344	1.029	0.334
contig025313-BriTAR.A002	contig022375-TilTARs.A048	0.359	1.076	0.334
contig020038-BurTAR.A002	contig066890-ZebTARs.A014	0.344	1.031	0.334
contig020038-BurTAR.A002	contig053139-ZebTARs.A023	0.352	1.053	0.334
contig065494-BurTARs.A030	contig039640-TilTAR.A002	0.366	1.095	0.334
contig055697-BurTARs.A022	contig022368-TilTARs.A047	0.319	0.954	0.334
contig059766-BurTARs.A029	contig039640-TilTAR.A002	0.368	1.100	0.334
contig032272-NyeTAR.A004	contig022362-TilTARs.A042	0.378	1.131	0.334
contig086344-BriTARs.A020	contig022345-TilTARs.A034	0.298	0.891	0.334
contig032272-NyeTAR.A004	contig022334-TilTARs.A029	0.375	1.121	0.334
contig084887-BriTARs.A018	contig003909-ZebTAR.A003	0.393	1.175	0.334
contig041024-BurTARs.A026	contig056134-TilTARs.A058	0.320	0.957	0.334

contig007524-TilTARs.A026	contig056134-TilTARs.A058	0.261	0.779	0.335
contig046013-NyeTARs.A021	contig003909-ZebTAR.A003	0.403	1.204	0.335
contig020038-BurTAR.A002	contig059766-BurTARs.A029	0.341	1.019	0.335
contig029633-BriTAR.A003	contig062677-ZebTARs.A018	0.415	1.240	0.335
contig039639-TilTAR.A001	contig030471-ZebTARs.A024	0.434	1.298	0.335
contig025313-BriTAR.A002	contig022354-TilTARs.A038	0.368	1.099	0.335
contig084868-BriTARs.A014	contig059768-ZebTARs.A022	0.333	0.995	0.335
contig022362-TilTARs.A042	contig040586-ZebTAR.A002	0.424	1.266	0.335
contig023443-TilTAR.A005	contig007520-TilTARs.A025	0.334	0.997	0.335
contig066285-ZebTARs.A016	contig030471-ZebTARs.A024	0.138	0.411	0.335
contig056020-BurTARs.A021	contig023443-TilTAR.A005	0.366	1.091	0.335
contig039639-TilTAR.A001	contig007524-TilTARs.A026	0.432	1.290	0.335
contig084887-BriTARs.A018	contig066330-ZebTARs.A027	0.324	0.966	0.335
contig060707-BurTARs.A015	contig039640-TilTAR.A003	0.403	1.203	0.335
contig020038-BurTAR.A002	contig053145-ZebTARs.A028	0.346	1.032	0.335
contig041024-BurTARs.A026	contig022377-TilTARs.A050	0.121	0.363	0.335
contig022334-TilTARs.A029	contig003909-ZebTAR.A003	0.375	1.119	0.335
contig032272-NyeTAR.A004	contig022383-TilTARs.A054	0.323	0.965	0.335
contig003909-ZebTAR.A003	contig066691-ZebTARs.A015	0.402	1.199	0.335
contig020038-BurTAR.A002	contig061091-BurTARs.A014	0.402	1.199	0.335
contig039640-TilTAR.A003	contig022363-TilTARs.A044	0.393	1.172	0.335
contig056020-BurTARs.A021	contig055697-BurTARs.A022	0.304	0.905	0.335
contig032272-NyeTAR.A004	contig030471-ZebTARs.A024	0.405	1.207	0.336
contig039640-TilTAR.A002	contig022365-TilTARs.A045	0.419	1.249	0.336
contig020038-BurTAR.A002	contig022368-TilTARs.A046	0.399	1.187	0.336
contig039639-TilTAR.A001	contig007520-TilTARs.A025	0.425	1.267	0.336
contig039640-TilTAR.A002	contig066330-ZebTARs.A027	0.371	1.104	0.336
contig025313-BriTAR.A002	contig060292-NyeTARs.A027	0.379	1.128	0.336
contig084868-BriTARs.A014	contig020038-BurTAR.A002	0.330	0.981	0.336
contig056020-BurTARs.A021	contig022320-TilTARs.A059	0.106	0.315	0.336
contig049534-BurTARs.A025	contig030471-ZebTARs.A024	0.137	0.406	0.336
contig007512-TilTARs.A024	contig030445-ZebTARs.A026	0.264	0.785	0.336
contig023443-TilTAR.A005	contig022363-TilTARs.A043	0.390	1.161	0.336
contig022355-TilTARs.A039	contig022390-TilTARs.A055	0.222	0.661	0.336
contig039640-TilTAR.A002	contig022349-TilTARs.A035	0.426	1.266	0.336
contig039639-TilTAR.A001	contig056134-TilTARs.A058	0.373	1.108	0.337
contig025313-BriTAR.A002	contig022337-TilTARs.A031	0.389	1.155	0.337
contig084887-BriTARs.A018	contig059766-BurTARs.A029	0.320	0.951	0.337
contig060105-NyeTARs.A018	contig066056-ZebTARs.A017	0.017	0.051	0.337
contig041024-BurTARs.A026	contig022345-TilTARs.A034	0.112	0.333	0.337
contig039640-TilTAR.A002	contig022368-TilTARs.A046	0.413	1.227	0.337
contig029633-BriTAR.A003	contig086351-BriTARs.A021	0.451	1.338	0.337
contig086351-BriTARs.A021	contig038663-NyeTAR.A005	0.444	1.319	0.337
contig025313-BriTAR.A002	contig086344-BriTARs.A020	0.339	1.007	0.337
contig060707-BurTARs.A015	contig040586-ZebTAR.A001	0.423	1.254	0.337
contig022320-TilTARs.A059	contig059768-ZebTARs.A022	0.370	1.098	0.337
contig056200-NyeTARs.A029	contig040586-ZebTAR.A002	0.407	1.206	0.337
contig086337-BriTARs.A019	contig022354-TilTARs.A037	0.311	0.922	0.337
contig020038-BurTAR.A002	contig022330-TilTARs.A028	0.376	1.113	0.337
contig039640-TilTAR.A002	contig007512-TilTARs.A024	0.397	1.176	0.337
contig025313-BriTAR.A002	contig060105-NyeTARs.A018	0.332	0.984	0.337
contig039642-TilTAR.A004	contig022356-TilTARs.A040	0.416	1.234	0.337
contig022320-TilTARs.A059	contig066285-ZebTARs.A016	0.139	0.411	0.337

contig057148-BurTARs.A028	contig023443-TilTAR.A005	0.359	1.063	0.337
contig022354-TilTARs.A037	contig040586-ZebTAR.A002	0.397	1.175	0.337
contig084887-BriTARs.A018	contig053145-ZebTARs.A028	0.323	0.957	0.337
contig022368-TilTARs.A046	contig022320-TilTARs.A059	0.106	0.314	0.338
contig022341-TilTARs.A032	contig022357-TilTARs.A041	0.126	0.375	0.338
contig032272-NyeTAR.A004	contig022349-TilTARs.A035	0.406	1.203	0.338
contig029633-BriTAR.A003	contig086344-BriTARs.A020	0.430	1.274	0.338
contig039640-TilTAR.A002	contig022356-TilTARs.A040	0.417	1.234	0.338
contig059766-BurTARs.A029	contig039640-TilTAR.A003	0.373	1.105	0.338
contig056020-BurTARs.A021	contig066285-ZebTARs.A016	0.151	0.448	0.338
contig055697-BurTARs.A022	contig032272-NyeTAR.A004	0.354	1.049	0.338
contig054630-BurTARs.A023	contig046014-NyeTARs.A024	0.370	1.095	0.338
contig056020-BurTARs.A021	contig022324-TilTARs.A027	0.151	0.448	0.338
contig039639-TilTAR.A001	contig022330-TilTARs.A028	0.406	1.203	0.338
contig046010-NyeTARs.A019	contig022324-TilTARs.A027	0.154	0.456	0.338
contig022375-TilTARs.A048	contig003909-ZebTAR.A003	0.367	1.087	0.338
contig056021-BurTARs.A020	contig022324-TilTARs.A027	0.142	0.420	0.338
contig022345-TilTARs.A034	contig066285-ZebTARs.A016	0.153	0.452	0.338
contig020038-BurTAR.A002	contig030471-ZebTARs.A024	0.410	1.212	0.338
contig058002-NyeTARs.A025	contig066056-ZebTARs.A017	0.289	0.854	0.338
contig022365-TilTARs.A045	contig040586-ZebTAR.A001	0.421	1.246	0.338
contig046014-NyeTARs.A024	contig022355-TilTARs.A039	0.098	0.289	0.338
contig020038-BurTAR.A002	contig007512-TilTARs.A024	0.357	1.056	0.338
contig057148-BurTARs.A028	contig022343-TilTARs.A033	0.125	0.371	0.338
contig039640-TilTAR.A003	contig022343-TilTARs.A033	0.409	1.208	0.338
contig020038-BurTAR.A002	contig060292-NyeTARs.A027	0.389	1.149	0.338
contig039639-TilTAR.A001	contig022355-TilTARs.A039	0.430	1.271	0.339
contig022353-TilTARs.A036	contig003909-ZebTAR.A003	0.391	1.154	0.339
contig039640-TilTAR.A003	contig022341-TilTARs.A032	0.408	1.206	0.339
contig049534-BurTARs.A025	contig022320-TilTARs.A059	0.138	0.407	0.339
contig022334-TilTARs.A030	contig040586-ZebTAR.A001	0.371	1.096	0.339
contig041024-BurTARs.A026	contig046010-NyeTARs.A019	0.129	0.381	0.339
contig084887-BriTARs.A018	contig040586-ZebTAR.A002	0.432	1.274	0.339
contig039640-TilTAR.A002	contig022337-TilTARs.A031	0.427	1.260	0.339
contig046010-NyeTARs.A019	contig022343-TilTARs.A033	0.122	0.360	0.339
contig049534-BurTARs.A025	contig039639-TilTAR.A001	0.436	1.287	0.339
contig039640-TilTAR.A003	contig022330-TilTARs.A028	0.404	1.192	0.339
contig041024-BurTARs.A026	contig039642-TilTAR.A004	0.407	1.200	0.339
contig020038-BurTAR.A002	contig022349-TilTARs.A035	0.404	1.189	0.339
contig022368-TilTARs.A046	contig040586-ZebTAR.A002	0.428	1.260	0.339
contig039640-TilTAR.A002	contig022330-TilTARs.A028	0.403	1.188	0.340
contig046014-NyeTARs.A024	contig066285-ZebTARs.A016	0.131	0.387	0.340
contig020038-BurTAR.A002	contig007520-TilTARs.A025	0.343	1.011	0.340
contig039642-TilTAR.A004	contig007512-TilTARs.A024	0.396	1.164	0.340
contig032272-NyeTAR.A004	contig022356-TilTARs.A040	0.368	1.083	0.340
contig060707-BurTARs.A015	contig038663-NyeTAR.A005	0.425	1.251	0.340
contig022353-TilTARs.A036	contig053145-ZebTARs.A028	0.309	0.908	0.340
contig046010-NyeTARs.A019	contig046014-NyeTARs.A024	0.130	0.381	0.340
contig045302-BurTAR.A001	contig022363-TilTARs.A043	0.434	1.277	0.340
contig032272-NyeTAR.A004	contig046013-NyeTARs.A021	0.403	1.184	0.340
contig022383-TilTARs.A054	contig053145-ZebTARs.A028	0.278	0.817	0.340
contig039640-TilTAR.A003	contig022320-TilTARs.A059	0.421	1.236	0.340
contig084876-BriTARs.A015	contig020038-BurTAR.A002	0.323	0.949	0.340

contig025313-BriTAR.A002	contig057148-BurTARs.A028	0.375	1.102	0.340
contig039639-TilTAR.A001	contig022368-TilTARs.A047	0.417	1.224	0.340
contig038663-NyeTAR.A005	contig022354-TilTARs.A038	0.440	1.294	0.340
contig045302-BurTAR.A001	contig022334-TilTARs.A030	0.370	1.087	0.340
contig060707-BurTARs.A015	contig003909-ZebTAR.A003	0.340	0.997	0.340
contig057145-BurTARs.A018	contig022354-TilTARs.A037	0.157	0.460	0.340
contig053139-ZebTARs.A023	contig030445-ZebTARs.A026	0.259	0.762	0.341
contig039640-TilTAR.A003	contig022355-TilTARs.A039	0.427	1.252	0.341
contig060707-BurTARs.A015	contig032272-NyeTAR.A004	0.341	1.001	0.341
contig022378-TilTARs.A051	contig053139-ZebTARs.A023	0.284	0.835	0.341
contig056134-TilTARs.A058	contig053145-ZebTARs.A028	0.063	0.184	0.341
contig032272-NyeTAR.A004	contig066691-ZebTARs.A015	0.402	1.179	0.341
contig022353-TilTARs.A036	contig040586-ZebTAR.A002	0.415	1.219	0.341
contig049534-BurTARs.A025	contig046014-NyeTARs.A024	0.131	0.383	0.341
contig046010-NyeTARs.A019	contig022390-TilTARs.A055	0.201	0.589	0.341
contig057148-BurTARs.A028	contig022390-TilTARs.A055	0.201	0.589	0.341
contig059673-BurTARs.A016	contig059768-ZebTARs.A022	0.369	1.082	0.341
contig060292-NyeTARs.A027	contig039642-TilTAR.A004	0.457	1.339	0.341
contig025313-BriTAR.A002	contig086337-BriTARs.A019	0.351	1.028	0.341
contig066285-ZebTARs.A016	contig053145-ZebTARs.A028	0.318	0.933	0.341
contig045302-BurTAR.A001	contig057148-BurTARs.A028	0.445	1.305	0.341
contig046010-NyeTARs.A019	contig040586-ZebTAR.A002	0.457	1.340	0.341
contig023443-TilTAR.A005	contig007518-TilTARs.A019	0.380	1.112	0.341
contig086337-BriTARs.A019	contig023443-TilTAR.A005	0.349	1.024	0.341
contig039640-TilTAR.A003	contig022377-TilTARs.A050	0.410	1.202	0.341
contig022354-TilTARs.A038	contig003909-ZebTAR.A003	0.370	1.084	0.341
contig023443-TilTAR.A005	contig022390-TilTARs.A056	0.329	0.965	0.341
contig055697-BurTARs.A022	contig022354-TilTARs.A037	0.312	0.914	0.341
contig056020-BurTARs.A021	contig053139-ZebTARs.A023	0.305	0.894	0.341
contig023443-TilTAR.A005	contig022343-TilTARs.A033	0.362	1.060	0.341
contig084886-BriTARs.A017	contig022320-TilTARs.A059	0.129	0.377	0.341
contig038663-NyeTAR.A005	contig022363-TilTARs.A043	0.441	1.291	0.341
contig045302-BurTAR.A001	contig060707-BurTARs.A015	0.420	1.231	0.341
contig086337-BriTARs.A019	contig003909-ZebTAR.A003	0.354	1.038	0.341
contig060105-NyeTARs.A018	contig039640-TilTAR.A002	0.420	1.229	0.342
contig059673-BurTARs.A016	contig056134-TilTARs.A058	0.282	0.825	0.342
contig022390-TilTARs.A055	contig003909-ZebTAR.A003	0.370	1.082	0.342
contig032272-NyeTAR.A004	contig022390-TilTARs.A055	0.370	1.082	0.342
contig056020-BurTARs.A021	contig059766-BurTARs.A029	0.311	0.912	0.342
contig086337-BriTARs.A019	contig032272-NyeTAR.A004	0.356	1.042	0.342
contig057145-BurTARs.A018	contig022334-TilTARs.A029	0.102	0.299	0.342
contig049540-BurTARs.A024	contig040586-ZebTAR.A002	0.403	1.179	0.342
contig058002-NyeTARs.A025	contig039640-TilTAR.A003	0.420	1.230	0.342
contig056020-BurTARs.A021	contig040586-ZebTAR.A002	0.447	1.309	0.342
contig041024-BurTARs.A026	contig022355-TilTARs.A039	0.099	0.289	0.342
contig062676-ZebTARs.A019	contig053139-ZebTARs.A023	0.282	0.825	0.342
contig022368-TilTARs.A047	contig053139-ZebTARs.A023	0.318	0.930	0.342
contig038663-NyeTAR.A005	contig022334-TilTARs.A030	0.374	1.093	0.342
contig057148-BurTARs.A028	contig022324-TilTARs.A027	0.158	0.461	0.342
contig045302-BurTAR.A001	contig022368-TilTARs.A046	0.416	1.216	0.342
contig059766-BurTARs.A029	contig022383-TilTARs.A054	0.280	0.819	0.342
contig055697-BurTARs.A022	contig035377-NyeTARs.A023	0.287	0.838	0.342
contig022390-TilTARs.A055	contig030471-ZebTARs.A024	0.187	0.547	0.342

contig041024-BurTARs.A026	contig022390-TilTARs.A055	0.187	0.547	0.342
contig020038-BurTAR.A002	contig022334-TilTARs.A029	0.379	1.108	0.342
contig023443-TilTAR.A005	contig022354-TilTARs.A038	0.361	1.054	0.342
contig054630-BurTARs.A023	contig022320-TilTARs.A059	0.377	1.100	0.342
contig020038-BurTAR.A002	contig022362-TilTARs.A042	0.383	1.118	0.342
contig022349-TilTARs.A035	contig003909-ZebTAR.A003	0.406	1.186	0.342
contig056020-BurTARs.A021	contig065494-BurTARs.A030	0.310	0.906	0.342
contig056020-BurTARs.A021	contig053145-ZebTARs.A028	0.312	0.910	0.342
contig057305-BurTARs.A031	contig039642-TilTAR.A004	0.461	1.347	0.342
contig045302-BurTAR.A001	contig041024-BurTARs.A026	0.417	1.219	0.342
contig056020-BurTARs.A021	contig039642-TilTAR.A004	0.439	1.282	0.342
contig065494-BurTARs.A030	contig039640-TilTAR.A003	0.372	1.085	0.342
contig056020-BurTARs.A021	contig049534-BurTARs.A025	0.148	0.432	0.343
contig060105-NyeTARs.A018	contig007512-TilTARs.A024	0.278	0.811	0.343
contig041024-BurTARs.A026	contig065494-BurTARs.A030	0.313	0.914	0.343
contig025313-BriTAR.A002	contig084887-BriTARs.A018	0.419	1.223	0.343
contig022353-TilTARs.A036	contig066330-ZebTARs.A027	0.315	0.920	0.343
contig020038-BurTAR.A002	contig046013-NyeTARs.A021	0.407	1.189	0.343
contig046014-NyeTARs.A024	contig053145-ZebTARs.A028	0.306	0.894	0.343
contig041024-BurTARs.A026	contig040586-ZebTAR.A002	0.414	1.209	0.343
contig029633-BriTAR.A003	contig084886-BriTARs.A017	0.453	1.322	0.343
contig007512-TilTARs.A024	contig022354-TilTARs.A037	0.317	0.925	0.343
contig060105-NyeTARs.A018	contig039642-TilTAR.A004	0.408	1.188	0.343
contig084886-BriTARs.A017	contig041024-BurTARs.A026	0.127	0.369	0.343
contig086344-BriTARs.A020	contig023443-TilTAR.A005	0.341	0.995	0.343
contig022337-TilTARs.A031	contig003909-ZebTAR.A003	0.400	1.165	0.343
contig020038-BurTAR.A002	contig022383-TilTARs.A054	0.324	0.945	0.343
contig039640-TilTAR.A002	contig022341-TilTARs.A032	0.400	1.165	0.343
contig061091-BurTARs.A014	contig039639-TilTAR.A001	0.428	1.248	0.343
contig084868-BriTARs.A014	contig055697-BurTARs.A022	0.295	0.858	0.343
contig032272-NyeTAR.A004	contig022375-TilTARs.A048	0.367	1.070	0.343
contig041024-BurTARs.A026	contig030464-ZebTARs.A025	0.131	0.380	0.343
contig025313-BriTAR.A002	contig022343-TilTARs.A033	0.372	1.083	0.343
contig025313-BriTAR.A002	contig046010-NyeTARs.A019	0.385	1.120	0.343
contig039640-TilTAR.A002	contig022320-TilTARs.A059	0.413	1.203	0.343
contig045302-BurTAR.A001	contig030464-ZebTARs.A025	0.452	1.317	0.343
contig060105-NyeTARs.A018	contig022345-TilTARs.A034	0.301	0.876	0.344
contig020038-BurTAR.A002	contig066691-ZebTARs.A015	0.407	1.183	0.344
contig039640-TilTAR.A002	contig022377-TilTARs.A050	0.405	1.178	0.344
contig040586-ZebTAR.A002	contig062677-ZebTARs.A018	0.405	1.180	0.344
contig029633-BriTAR.A003	contig022378-TilTARs.A051	0.366	1.065	0.344
contig032272-NyeTAR.A004	contig022337-TilTARs.A031	0.400	1.164	0.344
contig039640-TilTAR.A002	contig062676-ZebTARs.A019	0.376	1.095	0.344
contig086344-BriTARs.A020	contig040586-ZebTAR.A002	0.416	1.209	0.344
contig032272-NyeTAR.A004	contig022390-TilTARs.A056	0.337	0.979	0.344
contig086344-BriTARs.A020	contig053145-ZebTARs.A028	0.276	0.803	0.344
contig029633-BriTAR.A003	contig060292-NyeTARs.A027	0.465	1.352	0.344
contig055697-BurTARs.A022	contig035381-NyeTARs.A015	0.263	0.765	0.344
contig020038-BurTAR.A002	contig022390-TilTARs.A055	0.362	1.053	0.344
contig029633-BriTAR.A003	contig057305-BurTARs.A031	0.465	1.352	0.344
contig046010-NyeTARs.A019	contig023443-TilTAR.A005	0.371	1.078	0.344
contig022324-TilTARs.A027	contig061417-ZebTARs.A020	0.157	0.455	0.344
contig039640-TilTAR.A003	contig066330-ZebTARs.A027	0.376	1.093	0.344

contig084887-BriTARs.A018	contig032272-NyeTAR.A004	0.398	1.155	0.344
contig066056-ZebTARs.A017	contig062677-ZebTARs.A018	0.314	0.912	0.344
contig041024-BurTARs.A026	contig066330-ZebTARs.A027	0.313	0.909	0.344
contig022324-TilTARs.A027	contig022334-TilTARs.A030	0.202	0.585	0.344
contig038663-NyeTAR.A005	contig022365-TilTARs.A045	0.427	1.240	0.344
contig022337-TilTARs.A031	contig022354-TilTARs.A038	0.114	0.332	0.345
contig022390-TilTARs.A056	contig003909-ZebTAR.A003	0.337	0.978	0.345
contig046014-NyeTARs.A024	contig039640-TilTAR.A003	0.416	1.208	0.345
contig039642-TilTAR.A004	contig022365-TilTARs.A045	0.430	1.246	0.345
contig056020-BurTARs.A021	contig066330-ZebTARs.A027	0.312	0.906	0.345
contig025313-BriTAR.A002	contig055697-BurTARs.A022	0.357	1.035	0.345
contig039642-TilTAR.A004	contig022337-TilTARs.A031	0.436	1.265	0.345
contig022356-TilTARs.A040	contig003909-ZebTAR.A003	0.373	1.082	0.345
contig059766-BurTARs.A029	contig066285-ZebTARs.A016	0.322	0.935	0.345
contig059673-BurTARs.A016	contig058002-NyeTARs.A025	0.024	0.069	0.345
contig022378-TilTARs.A051	contig040586-ZebTAR.A002	0.357	1.033	0.345
contig049534-BurTARs.A025	contig053145-ZebTARs.A028	0.319	0.924	0.345
contig039639-TilTAR.A001	contig022334-TilTARs.A029	0.416	1.206	0.345
contig029633-BriTAR.A003	contig022354-TilTARs.A037	0.401	1.162	0.345
contig022324-TilTARs.A027	contig022368-TilTARs.A047	0.158	0.457	0.345
contig020038-BurTAR.A002	contig022379-TilTARs.A052	0.331	0.958	0.345
contig061433-BurTARs.A013	contig022368-TilTARs.A046	0.152	0.441	0.345
contig086344-BriTARs.A020	contig059766-BurTARs.A029	0.274	0.793	0.345
contig058002-NyeTARs.A025	contig039640-TilTAR.A002	0.428	1.238	0.345
contig055697-BurTARs.A022	contig040586-ZebTAR.A001	0.406	1.175	0.346
contig032272-NyeTAR.A004	contig066330-ZebTARs.A027	0.356	1.031	0.346
contig046007-NyeTARs.A017	contig039642-TilTAR.A004	0.433	1.253	0.346
contig022324-TilTARs.A027	contig022363-TilTARs.A043	0.194	0.562	0.346
contig046013-NyeTARs.A021	contig039639-TilTAR.A001	0.434	1.256	0.346
contig062039-NyeTARs.A022	contig066056-ZebTARs.A017	0.319	0.923	0.346
contig065494-BurTARs.A030	contig032272-NyeTAR.A004	0.349	1.010	0.346
contig022345-TilTARs.A034	contig022354-TilTARs.A037	0.157	0.453	0.346
contig029633-BriTAR.A003	contig060105-NyeTARs.A018	0.424	1.225	0.346
contig055697-BurTARs.A022	contig035376-NyeTARs.A014	0.264	0.763	0.346
contig061977-BurTARs.A012	contig039640-TilTAR.A002	0.381	1.103	0.346
contig007512-TilTARs.A024	contig022368-TilTARs.A047	0.335	0.968	0.346
contig039640-TilTAR.A002	contig022343-TilTARs.A033	0.405	1.170	0.346
contig025313-BriTAR.A002	contig030464-ZebTARs.A025	0.387	1.119	0.346
contig022354-TilTARs.A037	contig053139-ZebTARs.A023	0.315	0.909	0.346
contig038663-NyeTAR.A005	contig060292-NyeTARs.A027	0.461	1.333	0.346
contig046014-NyeTARs.A024	contig039640-TilTAR.A002	0.410	1.185	0.346
contig057145-BurTARs.A018	contig022363-TilTARs.A043	0.155	0.449	0.346
contig055697-BurTARs.A022	contig003909-ZebTAR.A003	0.352	1.018	0.346
contig020038-BurTAR.A002	contig056134-TilTARs.A058	0.355	1.025	0.346
contig020038-BurTAR.A002	contig022375-TilTARs.A048	0.372	1.073	0.346
contig025313-BriTAR.A002	contig022390-TilTARs.A055	0.381	1.100	0.346
contig022354-TilTARs.A037	contig040586-ZebTAR.A001	0.398	1.148	0.346
contig025313-BriTAR.A002	contig056020-BurTARs.A021	0.408	1.177	0.347
contig022341-TilTARs.A032	contig022365-TilTARs.A045	0.133	0.384	0.347
contig022379-TilTARs.A052	contig066056-ZebTARs.A017	0.298	0.860	0.347
contig086337-BriTARs.A019	contig030445-ZebTARs.A026	0.263	0.758	0.347
contig045302-BurTAR.A001	contig055697-BurTARs.A022	0.412	1.188	0.347
contig059766-BurTARs.A029	contig046014-NyeTARs.A024	0.311	0.895	0.347

contig045302-BurTAR.A001	contig007512-TilTARs.A024	0.407	1.174	0.347
contig061091-BurTARs.A014	contig039640-TilTAR.A003	0.424	1.220	0.347
contig007512-TilTARs.A024	contig022390-TilTARs.A055	0.293	0.845	0.347
contig049534-BurTARs.A025	contig022341-TilTARs.A032	0.157	0.454	0.347
contig029633-BriTAR.A003	contig022382-TilTARs.A053	0.424	1.222	0.347
contig084887-BriTARs.A018	contig020038-BurTAR.A002	0.402	1.159	0.347
contig035377-NyeTARs.A023	contig053139-ZebTARs.A023	0.288	0.830	0.347
contig046007-NyeTARs.A017	contig066285-ZebTARs.A016	0.155	0.446	0.347
contig084887-BriTARs.A018	contig040586-ZebTAR.A001	0.436	1.256	0.347
contig020038-BurTAR.A002	contig022337-TilTARs.A031	0.400	1.150	0.347
contig058002-NyeTARs.A025	contig007524-TilTARs.A026	0.031	0.090	0.347
contig057305-BurTARs.A031	contig038663-NyeTAR.A005	0.466	1.340	0.347
contig086337-BriTARs.A019	contig022368-TilTARs.A047	0.317	0.913	0.348
contig059673-BurTARs.A016	contig054630-BurTARs.A023	0.376	1.082	0.348
contig045302-BurTAR.A001	contig022362-TilTARs.A042	0.420	1.209	0.348
contig086337-BriTARs.A019	contig022378-TilTARs.A051	0.286	0.821	0.348
contig039639-TilTAR.A001	contig022345-TilTARs.A034	0.401	1.153	0.348
contig084868-BriTARs.A014	contig053139-ZebTARs.A023	0.290	0.832	0.348
contig025313-BriTAR.A002	contig022353-TilTARs.A036	0.413	1.189	0.348
contig086337-BriTARs.A019	contig046014-NyeTARs.A024	0.318	0.915	0.348
contig054630-BurTARs.A023	contig022354-TilTARs.A037	0.341	0.980	0.348
contig020038-BurTAR.A002	contig066330-ZebTARs.A027	0.357	1.026	0.348
contig020038-BurTAR.A002	contig022356-TilTARs.A040	0.373	1.071	0.348
contig039640-TilTAR.A003	contig022334-TilTARs.A029	0.415	1.192	0.348
contig039639-TilTAR.A001	contig022353-TilTARs.A036	0.452	1.299	0.348
contig032272-NyeTAR.A004	contig022354-TilTARs.A038	0.374	1.075	0.348
contig020038-BurTAR.A002	contig065494-BurTARs.A030	0.350	1.006	0.348
contig022334-TilTARs.A030	contig022383-TilTARs.A054	0.229	0.656	0.348
contig086337-BriTARs.A019	contig039640-TilTAR.A002	0.393	1.128	0.348
contig056021-BurTARs.A020	contig022356-TilTARs.A040	0.096	0.275	0.348
contig086344-BriTARs.A020	contig056134-TilTARs.A058	0.303	0.869	0.348
contig039639-TilTAR.A001	contig061417-ZebTARs.A020	0.408	1.171	0.348
contig039640-TilTAR.A002	contig022334-TilTARs.A029	0.414	1.188	0.348
contig022324-TilTARs.A027	contig022345-TilTARs.A034	0.164	0.470	0.348
contig060105-NyeTARs.A018	contig023443-TilTAR.A005	0.334	0.958	0.348
contig022341-TilTARs.A032	contig030471-ZebTARs.A024	0.133	0.382	0.348
contig022320-TilTARs.A059	contig030464-ZebTARs.A025	0.131	0.375	0.348
contig059766-BurTARs.A029	contig022355-TilTARs.A039	0.321	0.922	0.348
contig056021-BurTARs.A020	contig039639-TilTAR.A001	0.408	1.172	0.349
contig061433-BurTARs.A013	contig053145-ZebTARs.A028	0.347	0.995	0.349
contig086337-BriTARs.A019	contig062676-ZebTARs.A019	0.284	0.815	0.349
contig032272-NyeTAR.A004	contig056134-TilTARs.A058	0.354	1.014	0.349
contig022345-TilTARs.A034	contig022382-TilTARs.A053	0.245	0.703	0.349
contig007512-TilTARs.A024	contig040586-ZebTAR.A001	0.406	1.163	0.349
contig046014-NyeTARs.A024	contig056134-TilTARs.A058	0.325	0.931	0.349
contig049534-BurTARs.A025	contig059766-BurTARs.A029	0.323	0.926	0.349
contig039640-TilTAR.A003	contig022353-TilTARs.A036	0.448	1.283	0.349
contig086344-BriTARs.A020	contig065494-BurTARs.A030	0.279	0.799	0.349
contig060105-NyeTARs.A018	contig062039-NyeTARs.A022	0.318	0.910	0.349
contig029633-BriTAR.A003	contig057148-BurTARs.A028	0.460	1.318	0.349
contig057148-BurTARs.A028	contig003909-ZebTAR.A003	0.379	1.086	0.349
contig022345-TilTARs.A034	contig022375-TilTARs.A048	0.132	0.377	0.349
contig055697-BurTARs.A022	contig046014-NyeTARs.A024	0.315	0.903	0.349

contig022355-TilTARs.A039	contig053145-ZebTARs.A028	0.322	0.921	0.349
contig058002-NyeTARs.A025	contig056134-TilTARs.A058	0.264	0.756	0.349
contig086344-BriTARs.A020	contig039640-TilTAR.A003	0.415	1.187	0.349
contig061433-BurTARs.A013	contig022320-TilTARs.A059	0.141	0.403	0.349
contig046007-NyeTARs.A017	contig040586-ZebTAR.A002	0.446	1.278	0.349
contig022368-TilTARs.A046	contig040586-ZebTAR.A001	0.430	1.230	0.349
contig059766-BurTARs.A029	contig056134-TilTARs.A058	0.063	0.179	0.349
contig039640-TilTAR.A002	contig056134-TilTARs.A058	0.365	1.045	0.349
contig049534-BurTARs.A025	contig039640-TilTAR.A003	0.432	1.235	0.350
contig082565-BriTARs.A022	contig055697-BurTARs.A022	0.273	0.781	0.350
contig054630-BurTARs.A023	contig030471-ZebTARs.A024	0.391	1.118	0.350
contig045302-BurTAR.A001	contig046010-NyeTARs.A019	0.447	1.277	0.350
contig039640-TilTAR.A003	contig022368-TilTARs.A047	0.414	1.182	0.350
contig084868-BriTARs.A014	contig086337-BriTARs.A019	0.292	0.834	0.350
contig022341-TilTARs.A032	contig022377-TilTARs.A050	0.142	0.407	0.350
contig061433-BurTARs.A013	contig059766-BurTARs.A029	0.349	0.997	0.350
contig046007-NyeTARs.A017	contig007512-TilTARs.A024	0.323	0.923	0.350
contig084886-BriTARs.A017	contig022337-TilTARs.A031	0.122	0.347	0.350
contig046007-NyeTARs.A017	contig046014-NyeTARs.A024	0.106	0.303	0.350
contig022390-TilTARs.A055	contig056134-TilTARs.A058	0.255	0.727	0.350
contig022356-TilTARs.A040	contig022368-TilTARs.A046	0.127	0.362	0.350
contig022343-TilTARs.A033	contig003909-ZebTAR.A003	0.383	1.091	0.351
contig023443-TilTAR.A005	contig030464-ZebTARs.A025	0.379	1.080	0.351
contig062039-NyeTARs.A022	contig053145-ZebTARs.A028	0.278	0.792	0.351
contig020038-BurTAR.A002	contig022390-TilTARs.A056	0.338	0.962	0.351
contig007518-TilTARs.A019	contig003909-ZebTAR.A003	0.393	1.121	0.351
contig045302-BurTAR.A001	contig053139-ZebTARs.A023	0.411	1.170	0.351
contig032272-NyeTAR.A004	contig022343-TilTARs.A033	0.383	1.090	0.351
contig057145-BurTARs.A018	contig054630-BurTARs.A023	0.377	1.073	0.351
contig059673-BurTARs.A016	contig039639-TilTAR.A001	0.458	1.305	0.351
contig057145-BurTARs.A018	contig061417-ZebTARs.A020	0.101	0.289	0.351
contig039640-TilTAR.A003	contig066285-ZebTARs.A016	0.429	1.222	0.351
contig086344-BriTARs.A020	contig066330-ZebTARs.A027	0.284	0.809	0.351
contig022324-TilTARs.A027	contig030464-ZebTARs.A025	0.160	0.456	0.351
contig039640-TilTAR.A003	contig022357-TilTARs.A041	0.419	1.192	0.351
contig020038-BurTAR.A002	contig060707-BurTARs.A015	0.345	0.981	0.351
contig045302-BurTAR.A001	contig022354-TilTARs.A037	0.394	1.121	0.352
contig060105-NyeTARs.A018	contig040586-ZebTAR.A002	0.409	1.164	0.352
contig061433-BurTARs.A013	contig039640-TilTAR.A003	0.436	1.239	0.352
contig022341-TilTARs.A032	contig066056-ZebTARs.A017	0.315	0.895	0.352
contig060292-NyeTARs.A027	contig040586-ZebTAR.A002	0.456	1.295	0.352
contig057145-BurTARs.A018	contig049534-BurTARs.A025	0.145	0.413	0.352
contig022354-TilTARs.A037	contig059768-ZebTARs.A022	0.335	0.950	0.352
contig057305-BurTARs.A031	contig040586-ZebTAR.A002	0.456	1.295	0.352
contig086351-BriTARs.A021	contig040586-ZebTAR.A002	0.444	1.261	0.352
contig039640-TilTAR.A002	contig022375-TilTARs.A048	0.390	1.108	0.352
contig086337-BriTARs.A019	contig039642-TilTAR.A004	0.400	1.137	0.352
contig020038-BurTAR.A002	contig022354-TilTARs.A038	0.379	1.076	0.352
contig061977-BurTARs.A012	contig066056-ZebTARs.A017	0.295	0.838	0.352
contig057148-BurTARs.A028	contig032272-NyeTAR.A004	0.378	1.074	0.352
contig086337-BriTARs.A019	contig041024-BurTARs.A026	0.318	0.902	0.352
contig059766-BurTARs.A029	contig062039-NyeTARs.A022	0.281	0.797	0.352
contig039640-TilTAR.A003	contig022349-TilTARs.A035	0.426	1.208	0.352

contig046010-NyeTARs.A019	contig003909-ZebTAR.A003	0.389	1.104	0.352
contig007512-TilTARs.A024	contig022378-TilTARs.A051	0.297	0.842	0.352
contig057148-BurTARs.A028	contig022349-TilTARs.A035	0.141	0.401	0.352
contig046014-NyeTARs.A024	contig022324-TilTARs.A027	0.144	0.409	0.352
contig086344-BriTARs.A020	contig040586-ZebTAR.A001	0.435	1.234	0.353
contig084886-BriTARs.A017	contig045302-BurTAR.A001	0.444	1.260	0.353
contig084887-BriTARs.A018	contig039642-TilTAR.A004	0.432	1.224	0.353
contig022324-TilTARs.A027	contig053145-ZebTARs.A028	0.341	0.966	0.353
contig086337-BriTARs.A019	contig035377-NyeTARs.A023	0.287	0.815	0.353
contig039640-TilTAR.A003	contig030471-ZebTARs.A024	0.440	1.247	0.353
contig039642-TilTAR.A004	contig022363-TilTARs.A044	0.404	1.145	0.353
contig056200-NyeTARs.A029	contig066056-ZebTARs.A017	0.315	0.893	0.353
contig007512-TilTARs.A024	contig062676-ZebTARs.A019	0.298	0.844	0.353
contig022382-TilTARs.A053	contig040586-ZebTAR.A002	0.410	1.161	0.353
contig039640-TilTAR.A002	contig066285-ZebTARs.A016	0.425	1.203	0.353
contig054630-BurTARs.A023	contig046011-NyeTARs.A016	0.409	1.159	0.353
contig035377-NyeTARs.A023	contig007512-TilTARs.A024	0.298	0.843	0.353
contig084868-BriTARs.A014	contig056134-TilTARs.A058	0.302	0.854	0.353
contig022356-TilTARs.A040	contig022363-TilTARs.A043	0.144	0.406	0.353
contig039640-TilTAR.A002	contig030445-ZebTARs.A026	0.363	1.029	0.353
contig057148-BurTARs.A028	contig007512-TilTARs.A024	0.330	0.935	0.353
contig022368-TilTARs.A047	contig053145-ZebTARs.A028	0.319	0.902	0.353
contig056020-BurTARs.A021	contig022354-TilTARs.A037	0.157	0.443	0.353
contig084887-BriTARs.A018	contig030471-ZebTARs.A024	0.065	0.183	0.353
contig039642-TilTAR.A004	contig022375-TilTARs.A048	0.397	1.124	0.354
contig035376-NyeTARs.A014	contig039640-TilTAR.A002	0.377	1.067	0.354
contig060707-BurTARs.A015	contig022341-TilTARs.A032	0.327	0.926	0.354
contig062039-NyeTARs.A022	contig022383-TilTARs.A054	0.223	0.631	0.354
contig055697-BurTARs.A022	contig041024-BurTARs.A026	0.315	0.890	0.354
contig022341-TilTARs.A032	contig066285-ZebTARs.A016	0.165	0.467	0.354
contig057145-BurTARs.A018	contig066285-ZebTARs.A016	0.149	0.421	0.354
contig039642-TilTAR.A004	contig022368-TilTARs.A046	0.417	1.179	0.354
contig022378-TilTARs.A051	contig053145-ZebTARs.A028	0.282	0.796	0.354
contig025313-BriTAR.A002	contig007518-TilTARs.A019	0.385	1.088	0.354
contig054630-BurTARs.A023	contig022356-TilTARs.A040	0.368	1.039	0.354
contig059766-BurTARs.A029	contig066691-ZebTARs.A015	0.336	0.948	0.354
contig061091-BurTARs.A014	contig022353-TilTARs.A036	0.042	0.119	0.354
contig023443-TilTAR.A005	contig022362-TilTARs.A042	0.371	1.047	0.354
contig086337-BriTARs.A019	contig020038-BurTAR.A002	0.357	1.008	0.354
contig084868-BriTARs.A014	contig059766-BurTARs.A029	0.289	0.816	0.354
contig007512-TilTARs.A024	contig022355-TilTARs.A039	0.312	0.881	0.354
contig059766-BurTARs.A029	contig022324-TilTARs.A027	0.343	0.968	0.354
contig057145-BurTARs.A018	contig022324-TilTARs.A027	0.167	0.471	0.354
contig056200-NyeTARs.A029	contig040586-ZebTAR.A001	0.409	1.154	0.354
contig023443-TilTAR.A005	contig022353-TilTARs.A036	0.406	1.145	0.354
contig056200-NyeTARs.A029	contig022345-TilTARs.A034	0.256	0.722	0.354
contig046014-NyeTARs.A024	contig030464-ZebTARs.A025	0.135	0.382	0.354
contig046013-NyeTARs.A021	contig039640-TilTAR.A003	0.426	1.201	0.354
contig041024-BurTARs.A026	contig040586-ZebTAR.A001	0.425	1.200	0.355
contig022345-TilTARs.A034	contig059768-ZebTARs.A022	0.343	0.967	0.355
contig084886-BriTARs.A017	contig046014-NyeTARs.A024	0.132	0.371	0.355
contig066691-ZebTARs.A015	contig053145-ZebTARs.A028	0.339	0.955	0.355
contig020038-BurTAR.A002	contig022343-TilTARs.A033	0.382	1.078	0.355

contig022334-TilTARs.A030	contig062676-ZebTARs.A019	0.220	0.621	0.355
contig022345-TilTARs.A034	contig022354-TilTARs.A038	0.124	0.350	0.355
contig025313-BriTAR.A002	contig022363-TilTARs.A044	0.376	1.059	0.355
contig084876-BriTARs.A015	contig039640-TilTAR.A002	0.398	1.123	0.355
contig022355-TilTARs.A039	contig059768-ZebTARs.A022	0.352	0.992	0.355
contig039642-TilTAR.A004	contig022320-TilTARs.A059	0.417	1.175	0.355
contig057148-BurTARs.A028	contig022345-TilTARs.A034	0.135	0.380	0.355
contig059768-ZebTARs.A022	contig030471-ZebTARs.A024	0.384	1.083	0.355
contig059766-BurTARs.A029	contig022368-TilTARs.A047	0.321	0.904	0.355
contig022343-TilTARs.A033	contig030464-ZebTARs.A025	0.128	0.360	0.355
contig086344-BriTARs.A020	contig032272-NyeTAR.A004	0.352	0.990	0.355
contig039640-TilTAR.A002	contig022379-TilTARs.A052	0.407	1.147	0.355
contig022343-TilTARs.A033	contig040586-ZebTAR.A002	0.426	1.200	0.355
contig056020-BurTARs.A021	contig039640-TilTAR.A002	0.430	1.211	0.355
contig040586-ZebTAR.A001	contig053139-ZebTARs.A023	0.411	1.155	0.355
contig060105-NyeTARs.A018	contig003909-ZebTAR.A003	0.342	0.963	0.355
contig049534-BurTARs.A025	contig039640-TilTAR.A002	0.427	1.201	0.356
contig086344-BriTARs.A020	contig038663-NyeTAR.A005	0.438	1.231	0.356
contig039640-TilTAR.A002	contig030440-ZebTARs.A029	0.375	1.055	0.356
contig039639-TilTAR.A001	contig066691-ZebTARs.A015	0.435	1.222	0.356
contig060707-BurTARs.A015	contig022378-TilTARs.A051	0.315	0.886	0.356
contig022341-TilTARs.A032	contig022356-TilTARs.A040	0.117	0.330	0.356
contig032272-NyeTAR.A004	contig060105-NyeTARs.A018	0.344	0.967	0.356
contig022324-TilTARs.A027	contig022341-TilTARs.A032	0.167	0.471	0.356
contig041024-BurTARs.A026	contig022365-TilTARs.A045	0.117	0.330	0.356
contig039640-TilTAR.A003	contig022375-TilTARs.A048	0.387	1.089	0.356
contig046010-NyeTARs.A019	contig022345-TilTARs.A034	0.131	0.369	0.356
contig057145-BurTARs.A018	contig059768-ZebTARs.A022	0.370	1.040	0.356
contig055697-BurTARs.A022	contig046007-NyeTARs.A017	0.313	0.879	0.356
contig045302-BurTAR.A001	contig056134-TilTARs.A058	0.393	1.103	0.356
contig065494-BurTARs.A030	contig022383-TilTARs.A054	0.283	0.794	0.356
contig020038-BurTAR.A002	contig055697-BurTARs.A022	0.361	1.013	0.356
contig086344-BriTARs.A020	contig003909-ZebTAR.A003	0.350	0.984	0.356
contig007524-TilTARs.A026	contig066056-ZebTARs.A017	0.285	0.800	0.356
contig007520-TilTARs.A025	contig053145-ZebTARs.A028	0.236	0.662	0.356
contig022368-TilTARs.A046	contig053145-ZebTARs.A028	0.313	0.877	0.356
contig055697-BurTARs.A022	contig038663-NyeTAR.A005	0.415	1.164	0.357
contig022354-TilTARs.A037	contig022356-TilTARs.A040	0.155	0.433	0.357
contig032272-NyeTAR.A004	contig007518-TilTARs.A019	0.388	1.087	0.357
contig065494-BurTARs.A030	contig022355-TilTARs.A039	0.325	0.912	0.357
contig025313-BriTAR.A002	contig022355-TilTARs.A039	0.380	1.064	0.357
contig039640-TilTAR.A003	contig007520-TilTARs.A025	0.421	1.180	0.357
contig039642-TilTAR.A004	contig022355-TilTARs.A039	0.434	1.217	0.357
contig022355-TilTARs.A039	contig040586-ZebTAR.A002	0.446	1.250	0.357
contig039642-TilTAR.A004	contig056134-TilTARs.A058	0.372	1.044	0.357
contig061091-BurTARs.A014	contig022390-TilTARs.A055	0.197	0.551	0.357
contig060105-NyeTARs.A018	contig039640-TilTAR.A003	0.408	1.144	0.357
contig029633-BriTAR.A003	contig030464-ZebTARs.A025	0.467	1.309	0.357
contig066056-ZebTARs.A017	contig061410-ZebTARs.A021	0.299	0.838	0.357
contig084868-BriTARs.A014	contig053145-ZebTARs.A028	0.287	0.804	0.357
contig022354-TilTARs.A038	contig022320-TilTARs.A059	0.118	0.332	0.357
contig059766-BurTARs.A029	contig022378-TilTARs.A051	0.285	0.799	0.357
contig029633-BriTAR.A003	contig022363-TilTARs.A043	0.458	1.282	0.357

contig041024-BurTARs.A026	contig022354-TilTARs.A038	0.121	0.338	0.357
contig041024-BurTARs.A026	contig046007-NyeTARs.A017	0.109	0.304	0.357
contig086344-BriTARs.A020	contig045302-BurTAR.A001	0.433	1.211	0.357
contig035377-NyeTARs.A023	contig056134-TilTARs.A058	0.307	0.860	0.357
contig022353-TilTARs.A036	contig066691-ZebTARs.A015	0.033	0.093	0.357
contig039642-TilTAR.A004	contig022362-TilTARs.A042	0.420	1.175	0.357
contig022341-TilTARs.A032	contig022368-TilTARs.A046	0.132	0.369	0.357
contig065494-BurTARs.A030	contig046014-NyeTARs.A024	0.318	0.890	0.358
contig084887-BriTARs.A018	contig066285-ZebTARs.A016	0.144	0.402	0.358
contig045302-BurTAR.A001	contig022356-TilTARs.A040	0.427	1.195	0.358
contig039639-TilTAR.A001	contig022379-TilTARs.A052	0.403	1.127	0.358
contig082565-BriTARs.A022	contig053139-ZebTARs.A023	0.269	0.753	0.358
contig059766-BurTARs.A029	contig007520-TilTARs.A025	0.234	0.654	0.358
contig057145-BurTARs.A018	contig066056-ZebTARs.A017	0.334	0.934	0.358
contig084887-BriTARs.A018	contig039640-TilTAR.A002	0.424	1.186	0.358
contig039640-TilTAR.A002	contig061410-ZebTARs.A021	0.388	1.086	0.358
contig020038-BurTAR.A002	contig007518-TilTARs.A019	0.392	1.095	0.358
contig049540-BurTARs.A024	contig066056-ZebTARs.A017	0.315	0.881	0.358
contig061977-BurTARs.A012	contig039639-TilTAR.A001	0.377	1.054	0.358
contig055697-BurTARs.A022	contig058002-NyeTARs.A025	0.233	0.652	0.358
contig022377-TilTARs.A050	contig040586-ZebTAR.A002	0.424	1.185	0.358
contig049534-BurTARs.A025	contig046007-NyeTARs.A017	0.154	0.431	0.358
contig022356-TilTARs.A040	contig022365-TilTARs.A045	0.126	0.353	0.358
contig065494-BurTARs.A030	contig066285-ZebTARs.A016	0.328	0.916	0.358
contig039640-TilTAR.A003	contig022345-TilTARs.A034	0.407	1.138	0.358
contig046014-NyeTARs.A024	contig053139-ZebTARs.A023	0.321	0.896	0.358
contig020038-BurTAR.A002	contig056020-BurTARs.A021	0.423	1.181	0.358
contig056134-TilTARs.A058	contig040586-ZebTAR.A001	0.390	1.089	0.358
contig022383-TilTARs.A054	contig056134-TilTARs.A058	0.295	0.823	0.358
contig046007-NyeTARs.A017	contig039640-TilTAR.A002	0.425	1.185	0.358
contig056021-BurTARs.A020	contig022330-TilTARs.A028	0.033	0.091	0.358
contig084876-BriTARs.A015	contig022334-TilTARs.A030	0.220	0.613	0.358
contig022345-TilTARs.A034	contig053145-ZebTARs.A028	0.322	0.897	0.358
contig057305-BurTARs.A031	contig066056-ZebTARs.A017	0.275	0.767	0.359
contig040586-ZebTAR.A001	contig030464-ZebTARs.A025	0.454	1.266	0.359
contig035381-NyeTARs.A015	contig053139-ZebTARs.A023	0.264	0.737	0.359
contig003909-ZebTAR.A003	contig030464-ZebTARs.A025	0.397	1.106	0.359
contig045302-BurTAR.A001	contig061433-BurTARs.A013	0.443	1.234	0.359
contig022320-TilTARs.A059	contig040586-ZebTAR.A002	0.424	1.183	0.359
contig046014-NyeTARs.A024	contig022345-TilTARs.A034	0.116	0.322	0.359
contig086344-BriTARs.A020	contig062677-ZebTARs.A018	0.323	0.901	0.359
contig054630-BurTARs.A023	contig022375-TilTARs.A048	0.352	0.980	0.359
contig022356-TilTARs.A040	contig059768-ZebTARs.A022	0.361	1.007	0.359
contig038663-NyeTAR.A005	contig056134-TilTARs.A058	0.399	1.112	0.359
contig065494-BurTARs.A030	contig056134-TilTARs.A058	0.063	0.174	0.359
contig022337-TilTARs.A031	contig040586-ZebTAR.A002	0.447	1.247	0.359
contig039642-TilTAR.A004	contig022330-TilTARs.A028	0.413	1.151	0.359
contig055697-BurTARs.A022	contig022390-TilTARs.A055	0.305	0.849	0.359
contig084868-BriTARs.A014	contig065494-BurTARs.A030	0.292	0.813	0.359
contig046011-NyeTARs.A016	contig059768-ZebTARs.A022	0.402	1.119	0.359
contig057145-BurTARs.A018	contig022377-TilTARs.A050	0.141	0.393	0.359
contig049540-BurTARs.A024	contig040586-ZebTAR.A001	0.405	1.128	0.359
contig054630-BurTARs.A023	contig022363-TilTARs.A044	0.354	0.985	0.359

contig046014-NyeTARs.A024	contig022356-TilTARs.A040	0.104	0.290	0.359
contig046014-NyeTARs.A024	contig039642-TilTAR.A004	0.413	1.149	0.359
contig032272-NyeTAR.A004	contig046010-NyeTARs.A019	0.393	1.095	0.359
contig025313-BriTAR.A002	contig022354-TilTARs.A037	0.357	0.993	0.359
contig046014-NyeTARs.A024	contig066330-ZebTARs.A027	0.318	0.885	0.359
contig038663-NyeTAR.A005	contig022368-TilTARs.A046	0.437	1.215	0.359
contig039640-TilTAR.A003	contig066691-ZebTARs.A015	0.430	1.195	0.359
contig084887-BriTARs.A018	contig038663-NyeTAR.A005	0.442	1.230	0.359
contig084887-BriTARs.A018	contig045302-BurTAR.A001	0.431	1.198	0.359
contig035375-NyeTARs.A013	contig039640-TilTAR.A002	0.374	1.038	0.360
contig066285-ZebTARs.A016	contig066330-ZebTARs.A027	0.328	0.911	0.360
contig022355-TilTARs.A039	contig022320-TilTARs.A059	0.102	0.284	0.360
contig061091-BurTARs.A014	contig059766-BurTARs.A029	0.336	0.933	0.360
contig040586-ZebTAR.A001	contig053145-ZebTARs.A028	0.390	1.083	0.360
contig039639-TilTAR.A001	contig022357-TilTARs.A041	0.434	1.205	0.360
contig055697-BurTARs.A022	contig022365-TilTARs.A045	0.315	0.876	0.360
contig022345-TilTARs.A034	contig062677-ZebTARs.A018	0.255	0.708	0.360
contig029633-BriTAR.A003	contig007512-TilTARs.A024	0.410	1.139	0.360
contig045302-BurTAR.A001	contig053145-ZebTARs.A028	0.395	1.096	0.360
contig054630-BurTARs.A023	contig022345-TilTARs.A034	0.349	0.970	0.360
contig038663-NyeTAR.A005	contig022354-TilTARs.A037	0.406	1.126	0.360
contig022355-TilTARs.A039	contig003909-ZebTAR.A003	0.389	1.081	0.360
contig084887-BriTARs.A018	contig054630-BurTARs.A023	0.391	1.085	0.360
contig020038-BurTAR.A002	contig057148-BurTARs.A028	0.388	1.078	0.360
contig029633-BriTAR.A003	contig046010-NyeTARs.A019	0.464	1.289	0.360
contig049540-BurTARs.A024	contig022345-TilTARs.A034	0.255	0.707	0.360
contig084868-BriTARs.A014	contig007512-TilTARs.A024	0.311	0.863	0.360
contig084887-BriTARs.A018	contig055697-BurTARs.A022	0.318	0.882	0.360
contig086344-BriTARs.A020	contig007520-TilTARs.A025	0.287	0.795	0.360
contig060707-BurTARs.A015	contig057305-BurTARs.A031	0.281	0.781	0.360
contig022383-TilTARs.A054	contig066330-ZebTARs.A027	0.285	0.789	0.361
contig007520-TilTARs.A025	contig022383-TilTARs.A054	0.303	0.840	0.361
contig061091-BurTARs.A014	contig053145-ZebTARs.A028	0.339	0.940	0.361
contig059766-BurTARs.A029	contig022368-TilTARs.A046	0.317	0.878	0.361
contig055697-BurTARs.A022	contig049534-BurTARs.A025	0.329	0.913	0.361
contig038663-NyeTAR.A005	contig053145-ZebTARs.A028	0.398	1.105	0.361
contig022390-TilTARs.A055	contig030464-ZebTARs.A025	0.211	0.586	0.361
contig061433-BurTARs.A013	contig022377-TilTARs.A050	0.146	0.404	0.361
contig086337-BriTARs.A019	contig058002-NyeTARs.A025	0.231	0.640	0.361
contig032272-NyeTAR.A004	contig022355-TilTARs.A039	0.389	1.079	0.361
contig046014-NyeTARs.A024	contig007512-TilTARs.A024	0.323	0.895	0.361
contig023443-TilTAR.A005	contig022363-TilTARs.A044	0.363	1.007	0.361
contig065494-BurTARs.A030	contig062039-NyeTARs.A022	0.282	0.781	0.361
contig035381-NyeTARs.A015	contig007512-TilTARs.A024	0.274	0.759	0.361
contig040586-ZebTAR.A001	contig062677-ZebTARs.A018	0.408	1.129	0.361
contig022355-TilTARs.A039	contig022356-TilTARs.A040	0.103	0.284	0.361
contig060105-NyeTARs.A018	contig040586-ZebTAR.A001	0.429	1.187	0.361
contig022356-TilTARs.A040	contig022368-TilTARs.A047	0.133	0.368	0.361
contig065494-BurTARs.A030	contig022368-TilTARs.A046	0.326	0.902	0.361
contig022324-TilTARs.A027	contig022356-TilTARs.A040	0.160	0.443	0.361
contig039640-TilTAR.A002	contig061417-ZebTARs.A020	0.402	1.112	0.361
contig060292-NyeTARs.A027	contig066056-ZebTARs.A017	0.277	0.767	0.361
contig022363-TilTARs.A044	contig059768-ZebTARs.A022	0.354	0.980	0.361

contig062039-NyeTARs.A022	contig056134-TilTARs.A058	0.290	0.804	0.361
contig022353-TilTARs.A036	contig030471-ZebTARs.A024	0.068	0.189	0.361
contig034854-BurTARs.A027	contig022390-TilTARs.A055	0.069	0.190	0.361
contig086337-BriTARs.A019	contig022375-TilTARs.A048	0.299	0.827	0.361
contig058002-NyeTARs.A025	contig053145-ZebTARs.A028	0.243	0.672	0.361
contig039640-TilTAR.A003	contig007524-TilTARs.A026	0.428	1.183	0.361
contig061977-BurTARs.A012	contig057148-BurTARs.A028	0.240	0.665	0.362
contig045302-BurTAR.A001	contig056200-NyeTARs.A029	0.406	1.122	0.362
contig046011-NyeTARs.A016	contig039640-TilTAR.A002	0.489	1.354	0.362
contig022324-TilTARs.A027	contig022355-TilTARs.A039	0.149	0.412	0.362
contig022383-TilTARs.A054	contig066056-ZebTARs.A017	0.293	0.810	0.362
contig084868-BriTARs.A014	contig066056-ZebTARs.A017	0.301	0.833	0.362
contig022324-TilTARs.A027	contig022349-TilTARs.A035	0.167	0.463	0.362
contig054630-BurTARs.A023	contig022355-TilTARs.A039	0.359	0.992	0.362
contig022375-TilTARs.A048	contig059768-ZebTARs.A022	0.345	0.953	0.362
contig032272-NyeTAR.A004	contig030464-ZebTARs.A025	0.396	1.094	0.362
contig056020-BurTARs.A021	contig022377-TilTARs.A050	0.139	0.383	0.362
contig039642-TilTAR.A004	contig022349-TilTARs.A035	0.438	1.211	0.362
contig022355-TilTARs.A039	contig061417-ZebTARs.A020	0.109	0.301	0.362
contig046010-NyeTARs.A019	contig007512-TilTARs.A024	0.333	0.920	0.362
contig060707-BurTARs.A015	contig007520-TilTARs.A025	0.283	0.782	0.362
contig086344-BriTARs.A020	contig022379-TilTARs.A052	0.307	0.848	0.362
contig025313-BriTAR.A002	contig084886-BriTARs.A017	0.381	1.054	0.362
contig022345-TilTARs.A034	contig022320-TilTARs.A059	0.112	0.308	0.362
contig022354-TilTARs.A037	contig056134-TilTARs.A058	0.315	0.868	0.362
contig055697-BurTARs.A022	contig066890-ZebTARs.A014	0.273	0.753	0.362
contig029633-BriTAR.A003	contig053145-ZebTARs.A028	0.402	1.111	0.362
contig039640-TilTAR.A003	contig061417-ZebTARs.A020	0.408	1.127	0.362
contig086344-BriTARs.A020	contig022378-TilTARs.A051	0.324	0.895	0.362
contig022377-TilTARs.A050	contig030471-ZebTARs.A024	0.127	0.351	0.362
contig025313-BriTAR.A002	contig046011-NyeTARs.A016	0.434	1.197	0.362
contig084886-BriTARs.A017	contig023443-TilTAR.A005	0.368	1.015	0.362
contig086337-BriTARs.A019	contig022365-TilTARs.A045	0.312	0.861	0.362
contig082565-BriTARs.A022	contig066056-ZebTARs.A017	0.303	0.835	0.362
contig056134-TilTARs.A058	contig030471-ZebTARs.A024	0.357	0.984	0.363
contig041024-BurTARs.A026	contig053139-ZebTARs.A023	0.320	0.883	0.363
contig059766-BurTARs.A029	contig022345-TilTARs.A034	0.321	0.886	0.363
contig059766-BurTARs.A029	contig058002-NyeTARs.A025	0.241	0.664	0.363
contig057148-BurTARs.A028	contig061410-ZebTARs.A021	0.240	0.663	0.363
contig022353-TilTARs.A036	contig040586-ZebTAR.A001	0.424	1.170	0.363
contig022368-TilTARs.A046	contig066330-ZebTARs.A027	0.325	0.897	0.363
contig046014-NyeTARs.A024	contig040586-ZebTAR.A002	0.420	1.157	0.363
contig057145-BurTARs.A018	contig022330-TilTARs.A028	0.110	0.303	0.363
contig061977-BurTARs.A012	contig022345-TilTARs.A034	0.227	0.625	0.363
contig029633-BriTAR.A003	contig065494-BurTARs.A030	0.396	1.091	0.363
contig029633-BriTAR.A003	contig059766-BurTARs.A029	0.398	1.096	0.363
contig065494-BurTARs.A030	contig022368-TilTARs.A047	0.331	0.911	0.363
contig057145-BurTARs.A018	contig041024-BurTARs.A026	0.113	0.312	0.363
contig057148-BurTARs.A028	contig022330-TilTARs.A028	0.131	0.359	0.363
contig086337-BriTARs.A019	contig022355-TilTARs.A039	0.303	0.833	0.363
contig056020-BurTARs.A021	contig030471-ZebTARs.A024	0.103	0.285	0.363
contig039639-TilTAR.A001	contig030445-ZebTARs.A026	0.365	1.005	0.363
contig055697-BurTARs.A022	contig022355-TilTARs.A039	0.303	0.833	0.363

contig020038-BurTAR.A002	contig046010-NyeTARs.A019	0.398	1.096	0.363
contig065494-BurTARs.A030	contig066691-ZebTARs.A015	0.340	0.936	0.364
contig022349-TilTARs.A035	contig040586-ZebTAR.A002	0.447	1.230	0.364
contig035376-NyeTARs.A014	contig053139-ZebTARs.A023	0.267	0.735	0.364
contig060105-NyeTARs.A018	contig022341-TilTARs.A032	0.333	0.916	0.364
contig060707-BurTARs.A015	contig022379-TilTARs.A052	0.302	0.831	0.364
contig082565-BriTARs.A022	contig039640-TilTAR.A002	0.367	1.008	0.364
contig022363-TilTARs.A044	contig040586-ZebTAR.A002	0.407	1.118	0.364
contig061091-BurTARs.A014	contig039640-TilTAR.A002	0.424	1.166	0.364
contig065494-BurTARs.A030	contig007520-TilTARs.A025	0.240	0.661	0.364
contig061433-BurTARs.A013	contig022368-TilTARs.A047	0.164	0.452	0.364
contig038663-NyeTAR.A005	contig060105-NyeTARs.A018	0.431	1.185	0.364
contig056021-BurTARs.A020	contig022355-TilTARs.A039	0.112	0.308	0.364
contig056021-BurTARs.A020	contig039640-TilTAR.A003	0.409	1.125	0.364
contig022382-TilTARs.A053	contig066056-ZebTARs.A017	0.315	0.866	0.364
contig057148-BurTARs.A028	contig022334-TilTARs.A029	0.133	0.364	0.364
contig086337-BriTARs.A019	contig082565-BriTARs.A022	0.273	0.749	0.364
contig022383-TilTARs.A054	contig062676-ZebTARs.A019	0.026	0.071	0.364
contig056021-BurTARs.A020	contig022334-TilTARs.A030	0.149	0.410	0.364
contig039640-TilTAR.A003	contig056134-TilTARs.A058	0.370	1.015	0.364
contig061433-BurTARs.A013	contig041024-BurTARs.A026	0.144	0.395	0.364
contig046010-NyeTARs.A019	contig022330-TilTARs.A028	0.127	0.349	0.364
contig054630-BurTARs.A023	contig066890-ZebTARs.A014	0.343	0.941	0.364
contig022355-TilTARs.A039	contig066330-ZebTARs.A027	0.332	0.909	0.365
contig055697-BurTARs.A022	contig066285-ZebTARs.A016	0.332	0.912	0.365
contig007512-TilTARs.A024	contig022365-TilTARs.A045	0.317	0.870	0.365
contig082565-BriTARs.A022	contig007512-TilTARs.A024	0.279	0.765	0.365
contig084887-BriTARs.A018	contig049534-BurTARs.A025	0.139	0.380	0.365
contig049534-BurTARs.A025	contig053139-ZebTARs.A023	0.326	0.894	0.365
contig049534-BurTARs.A025	contig065494-BurTARs.A030	0.331	0.907	0.365
contig046007-NyeTARs.A017	contig022324-TilTARs.A027	0.158	0.433	0.365
contig056021-BurTARs.A020	contig039640-TilTAR.A002	0.407	1.115	0.365
contig059768-ZebTARs.A022	contig030440-ZebTARs.A029	0.337	0.925	0.365
contig022363-TilTARs.A043	contig053145-ZebTARs.A028	0.345	0.945	0.365
contig022363-TilTARs.A043	contig066330-ZebTARs.A027	0.345	0.945	0.365
contig061091-BurTARs.A014	contig040586-ZebTAR.A002	0.438	1.200	0.365
contig059673-BurTARs.A016	contig053145-ZebTARs.A028	0.251	0.688	0.365
contig022355-TilTARs.A039	contig066285-ZebTARs.A016	0.151	0.412	0.365
contig022365-TilTARs.A045	contig022390-TilTARs.A055	0.211	0.577	0.365
contig007512-TilTARs.A024	contig022341-TilTARs.A032	0.327	0.897	0.365
contig086337-BriTARs.A019	contig035381-NyeTARs.A015	0.268	0.734	0.365
contig041024-BurTARs.A026	contig007512-TilTARs.A024	0.322	0.882	0.365
contig082565-BriTARs.A022	contig022334-TilTARs.A030	0.224	0.613	0.365
contig034854-BurTARs.A027	contig039640-TilTAR.A002	0.367	1.004	0.365
contig007520-TilTARs.A025	contig066330-ZebTARs.A027	0.244	0.667	0.365
contig046010-NyeTARs.A019	contig022334-TilTARs.A029	0.129	0.354	0.365
contig045302-BurTAR.A001	contig060105-NyeTARs.A018	0.426	1.167	0.365
contig066056-ZebTARs.A017	contig030445-ZebTARs.A026	0.305	0.834	0.365
contig046007-NyeTARs.A017	contig053139-ZebTARs.A023	0.317	0.867	0.365
contig084887-BriTARs.A018	contig059768-ZebTARs.A022	0.384	1.050	0.365
contig007512-TilTARs.A024	contig022354-TilTARs.A038	0.325	0.888	0.366
contig029633-BriTAR.A003	contig022334-TilTARs.A030	0.386	1.057	0.366
contig061091-BurTARs.A014	contig040586-ZebTAR.A001	0.436	1.193	0.366

contig061977-BurTARs.A012	contig022383-TilTARs.A054	0.136	0.373	0.366
contig086337-BriTARs.A019	contig022363-TilTARs.A043	0.339	0.927	0.366
contig086337-BriTARs.A019	contig049534-BurTARs.A025	0.327	0.893	0.366
contig055697-BurTARs.A022	contig022375-TilTARs.A048	0.303	0.829	0.366
contig035375-NyeTARs.A013	contig059768-ZebTARs.A022	0.337	0.920	0.366
contig038663-NyeTAR.A005	contig056200-NyeTARs.A029	0.416	1.137	0.366
contig084868-BriTARs.A014	contig007520-TilTARs.A025	0.317	0.867	0.366
contig038663-NyeTAR.A005	contig007512-TilTARs.A024	0.421	1.150	0.366
contig022363-TilTARs.A043	contig056134-TilTARs.A058	0.386	1.055	0.366
contig035376-NyeTARs.A014	contig039639-TilTAR.A001	0.381	1.042	0.366
contig086344-BriTARs.A020	contig057145-BurTARs.A018	0.348	0.950	0.366
contig030471-ZebTARs.A024	contig053145-ZebTARs.A028	0.348	0.951	0.366
contig055697-BurTARs.A022	contig040586-ZebTAR.A002	0.400	1.094	0.366
contig020038-BurTAR.A002	contig030464-ZebTARs.A025	0.401	1.095	0.366
contig022354-TilTARs.A037	contig003909-ZebTAR.A003	0.365	0.998	0.366
contig045302-BurTAR.A001	contig049540-BurTARs.A024	0.402	1.098	0.366
contig084868-BriTARs.A014	contig022334-TilTARs.A030	0.247	0.674	0.366
contig057148-BurTARs.A028	contig056134-TilTARs.A058	0.338	0.922	0.366
contig022363-TilTARs.A043	contig022363-TilTARs.A044	0.064	0.174	0.366
contig039642-TilTAR.A004	contig022343-TilTARs.A033	0.420	1.148	0.366
contig022334-TilTARs.A030	contig061410-ZebTARs.A021	0.219	0.597	0.366
contig041024-BurTARs.A026	contig038663-NyeTAR.A005	0.431	1.177	0.366
contig045302-BurTAR.A001	contig022324-TilTARs.A027	0.442	1.207	0.366
contig086344-BriTARs.A020	contig020038-BurTAR.A002	0.355	0.969	0.366
contig049534-BurTARs.A025	contig022355-TilTARs.A039	0.146	0.397	0.367
contig032272-NyeTAR.A004	contig022354-TilTARs.A037	0.365	0.997	0.367
contig059766-BurTARs.A029	contig040586-ZebTAR.A001	0.392	1.069	0.367
contig039640-TilTAR.A002	contig007520-TilTARs.A025	0.432	1.178	0.367
contig049534-BurTARs.A025	contig066330-ZebTARs.A027	0.331	0.902	0.367
contig057148-BurTARs.A028	contig059768-ZebTARs.A022	0.367	1.000	0.367
contig038663-NyeTAR.A005	contig053139-ZebTARs.A023	0.420	1.144	0.367
contig022382-TilTARs.A053	contig040586-ZebTAR.A001	0.420	1.144	0.367
contig022357-TilTARs.A041	contig003909-ZebTAR.A003	0.399	1.087	0.367
contig046007-NyeTARs.A017	contig022353-TilTARs.A036	0.099	0.269	0.367
contig039639-TilTAR.A001	contig030440-ZebTARs.A029	0.381	1.038	0.367
contig045302-BurTAR.A001	contig059766-BurTARs.A029	0.397	1.083	0.367
contig039642-TilTAR.A004	contig022334-TilTARs.A029	0.423	1.154	0.367
contig032272-NyeTAR.A004	contig022357-TilTARs.A041	0.394	1.073	0.367
contig022330-TilTARs.A028	contig022356-TilTARs.A040	0.100	0.272	0.367
contig020038-BurTAR.A002	contig060105-NyeTARs.A018	0.348	0.947	0.367
contig057305-BurTARs.A031	contig053145-ZebTARs.A028	0.274	0.747	0.367
contig022334-TilTARs.A029	contig022355-TilTARs.A039	0.105	0.287	0.367
contig007512-TilTARs.A024	contig040586-ZebTAR.A002	0.400	1.089	0.367
contig022355-TilTARs.A039	contig053139-ZebTARs.A023	0.306	0.834	0.367
contig046013-NyeTARs.A021	contig040586-ZebTAR.A002	0.440	1.198	0.367
contig007512-TilTARs.A024	contig030464-ZebTARs.A025	0.338	0.920	0.367
contig059766-BurTARs.A029	contig038663-NyeTAR.A005	0.401	1.091	0.367
contig059673-BurTARs.A016	contig059766-BurTARs.A029	0.253	0.689	0.367
contig055697-BurTARs.A022	contig022368-TilTARs.A046	0.311	0.848	0.367
contig022368-TilTARs.A047	contig066330-ZebTARs.A027	0.333	0.905	0.367
contig039640-TilTAR.A003	contig062676-ZebTARs.A019	0.385	1.049	0.367
contig066890-ZebTARs.A014	contig053139-ZebTARs.A023	0.274	0.746	0.368
contig045302-BurTAR.A001	contig046014-NyeTARs.A024	0.425	1.155	0.368

contig032272-NyeTAR.A004	contig022353-TilTARs.A036	0.424	1.153	0.368
contig007512-TilTARs.A024	contig066890-ZebTARs.A014	0.279	0.758	0.368
contig086344-BriTARs.A020	contig056200-NyeTARs.A029	0.324	0.882	0.368
contig056020-BurTARs.A021	contig061417-ZebTARs.A020	0.115	0.313	0.368
contig046007-NyeTARs.A017	contig022377-TilTARs.A050	0.141	0.383	0.368
contig039640-TilTAR.A002	contig022357-TilTARs.A041	0.421	1.146	0.368
contig086337-BriTARs.A019	contig022390-TilTARs.A055	0.283	0.769	0.368
contig056020-BurTARs.A021	contig056134-TilTARs.A058	0.333	0.906	0.368
contig041024-BurTARs.A026	contig022368-TilTARs.A046	0.105	0.287	0.368
contig022345-TilTARs.A034	contig030464-ZebTARs.A025	0.137	0.372	0.368
contig084886-BriTARs.A017	contig040586-ZebTAR.A001	0.446	1.213	0.368
contig035376-NyeTARs.A014	contig066056-ZebTARs.A017	0.314	0.855	0.368
contig054630-BurTARs.A023	contig057148-BurTARs.A028	0.379	1.030	0.368
contig022378-TilTARs.A051	contig056134-TilTARs.A058	0.292	0.793	0.368
contig022365-TilTARs.A045	contig053139-ZebTARs.A023	0.316	0.859	0.368
contig084887-BriTARs.A018	contig053139-ZebTARs.A023	0.320	0.869	0.368
contig035376-NyeTARs.A014	contig007512-TilTARs.A024	0.275	0.747	0.368
contig045302-BurTAR.A001	contig062677-ZebTARs.A018	0.404	1.099	0.368
contig060707-BurTARs.A015	contig060292-NyeTARs.A027	0.283	0.770	0.368
contig029633-BriTAR.A003	contig055697-BurTARs.A022	0.423	1.150	0.368
contig082565-BriTARs.A022	contig062039-NyeTARs.A022	0.208	0.564	0.368
contig039640-TilTAR.A002	contig022353-TilTARs.A036	0.451	1.225	0.368
contig057148-BurTARs.A028	contig066056-ZebTARs.A017	0.323	0.878	0.368
contig058002-NyeTARs.A025	contig007520-TilTARs.A025	0.018	0.050	0.368
contig084868-BriTARs.A014	contig039640-TilTAR.A002	0.382	1.037	0.368
contig084868-BriTARs.A014	contig066330-ZebTARs.A027	0.294	0.797	0.368
contig046014-NyeTARs.A024	contig022377-TilTARs.A050	0.127	0.344	0.368
contig022343-TilTARs.A033	contig022354-TilTARs.A038	0.115	0.312	0.368
contig056020-BurTARs.A021	contig040586-ZebTAR.A001	0.443	1.202	0.368
contig086351-BriTARs.A021	contig053145-ZebTARs.A028	0.273	0.742	0.368
contig086337-BriTARs.A019	contig059673-BurTARs.A016	0.245	0.666	0.368
contig039639-TilTAR.A001	contig007518-TilTARs.A019	0.466	1.265	0.368
contig061977-BurTARs.A012	contig062676-ZebTARs.A019	0.131	0.355	0.368
contig066890-ZebTARs.A014	contig059768-ZebTARs.A022	0.337	0.914	0.369
contig055697-BurTARs.A022	contig034854-BurTARs.A027	0.292	0.793	0.369
contig022324-TilTARs.A027	contig022377-TilTARs.A050	0.142	0.385	0.369
contig066285-ZebTARs.A016	contig053139-ZebTARs.A023	0.329	0.893	0.369
contig056134-TilTARs.A058	contig066330-ZebTARs.A027	0.067	0.181	0.369
contig057148-BurTARs.A028	contig066285-ZebTARs.A016	0.168	0.456	0.369
contig040586-ZebTAR.A002	contig053145-ZebTARs.A028	0.387	1.050	0.369
contig022349-TilTARs.A035	contig022390-TilTARs.A055	0.212	0.574	0.369
contig020038-BurTAR.A002	contig022355-TilTARs.A039	0.394	1.067	0.369
contig065494-BurTARs.A030	contig058002-NyeTARs.A025	0.247	0.670	0.369
contig045302-BurTAR.A001	contig022320-TilTARs.A059	0.427	1.158	0.369
contig055697-BurTARs.A022	contig057148-BurTARs.A028	0.332	0.899	0.369
contig084868-BriTARs.A014	contig007518-TilTARs.A019	0.361	0.978	0.369
contig039640-TilTAR.A002	contig022383-TilTARs.A054	0.380	1.030	0.369
contig039640-TilTAR.A003	contig022379-TilTARs.A052	0.401	1.087	0.369
contig049534-BurTARs.A025	contig007512-TilTARs.A024	0.342	0.927	0.369
contig055697-BurTARs.A022	contig022349-TilTARs.A035	0.327	0.885	0.369
contig086337-BriTARs.A019	contig045302-BurTAR.A001	0.416	1.126	0.369
contig084886-BriTARs.A017	contig032272-NyeTAR.A004	0.385	1.042	0.369
contig057145-BurTARs.A018	contig022341-TilTARs.A032	0.133	0.359	0.369

contig056021-BurTARs.A020	contig057148-BurTARs.A028	0.134	0.363	0.369
contig035377-NyeTARs.A023	contig053145-ZebTARs.A028	0.288	0.779	0.369
contig060707-BurTARs.A015	contig057145-BurTARs.A018	0.344	0.932	0.369
contig022349-TilTARs.A035	contig066056-ZebTARs.A017	0.339	0.918	0.370
contig046007-NyeTARs.A017	contig053145-ZebTARs.A028	0.324	0.877	0.370
contig038663-NyeTAR.A005	contig030464-ZebTARs.A025	0.461	1.248	0.370
contig046014-NyeTARs.A024	contig022354-TilTARs.A038	0.125	0.339	0.370
contig061091-BurTARs.A014	contig065494-BurTARs.A030	0.340	0.921	0.370
contig058002-NyeTARs.A025	contig022383-TilTARs.A054	0.303	0.821	0.370
contig046014-NyeTARs.A024	contig022365-TilTARs.A045	0.119	0.321	0.370
contig007512-TilTARs.A024	contig022349-TilTARs.A035	0.332	0.897	0.370
contig056021-BurTARs.A020	contig053145-ZebTARs.A028	0.329	0.891	0.370
contig057148-BurTARs.A028	contig040586-ZebTAR.A001	0.453	1.225	0.370
contig022368-TilTARs.A047	contig040586-ZebTAR.A002	0.434	1.174	0.370
contig062039-NyeTARs.A022	contig066330-ZebTARs.A027	0.285	0.771	0.370
contig061433-BurTARs.A013	contig040586-ZebTAR.A001	0.449	1.213	0.370
contig086337-BriTARs.A019	contig066285-ZebTARs.A016	0.330	0.892	0.370
contig059766-BurTARs.A029	contig030471-ZebTARs.A024	0.352	0.952	0.370
contig007524-TilTARs.A026	contig053145-ZebTARs.A028	0.242	0.653	0.370
contig049534-BurTARs.A025	contig066285-ZebTARs.A016	0.010	0.027	0.370
contig007520-TilTARs.A025	contig062676-ZebTARs.A019	0.309	0.836	0.370
contig039639-TilTAR.A001	contig061410-ZebTARs.A021	0.384	1.038	0.370
contig045302-BurTAR.A001	contig022330-TilTARs.A028	0.415	1.122	0.370
contig058002-NyeTARs.A025	contig039642-TilTAR.A004	0.425	1.148	0.370
contig084868-BriTARs.A014	contig058002-NyeTARs.A025	0.318	0.858	0.370
contig061977-BurTARs.A012	contig055697-BurTARs.A022	0.266	0.719	0.370
contig022354-TilTARs.A038	contig030464-ZebTARs.A025	0.020	0.054	0.370
contig055697-BurTARs.A022	contig022341-TilTARs.A032	0.314	0.848	0.370
contig057145-BurTARs.A018	contig022355-TilTARs.A039	0.111	0.300	0.370
contig065494-BurTARs.A030	contig022354-TilTARs.A037	0.312	0.842	0.370
contig029633-BriTAR.A003	contig022356-TilTARs.A040	0.434	1.171	0.370
contig061977-BurTARs.A012	contig046010-NyeTARs.A019	0.244	0.659	0.370
contig022324-TilTARs.A027	contig022330-TilTARs.A028	0.151	0.406	0.370
contig020038-BurTAR.A002	contig022353-TilTARs.A036	0.428	1.156	0.370
contig058002-NyeTARs.A025	contig066330-ZebTARs.A027	0.251	0.677	0.370
contig054630-BurTARs.A023	contig022349-TilTARs.A035	0.405	1.092	0.371
contig049540-BurTARs.A024	contig038663-NyeTAR.A005	0.412	1.113	0.371
contig022324-TilTARs.A027	contig066056-ZebTARs.A017	0.328	0.886	0.371
contig022353-TilTARs.A036	contig053139-ZebTARs.A023	0.303	0.817	0.371
contig039640-TilTAR.A002	contig030471-ZebTARs.A024	0.439	1.185	0.371
contig061091-BurTARs.A014	contig030471-ZebTARs.A024	0.066	0.177	0.371
contig029633-BriTAR.A003	contig066330-ZebTARs.A027	0.408	1.101	0.371
contig054630-BurTARs.A023	contig030440-ZebTARs.A029	0.344	0.927	0.371
contig055697-BurTARs.A022	contig061410-ZebTARs.A021	0.262	0.707	0.371
contig065494-BurTARs.A030	contig022378-TilTARs.A051	0.291	0.784	0.371
contig039640-TilTAR.A003	contig022324-TilTARs.A027	0.435	1.174	0.371
contig022356-TilTARs.A040	contig040586-ZebTAR.A001	0.432	1.164	0.371
contig035375-NyeTARs.A013	contig039639-TilTAR.A001	0.379	1.022	0.371
contig065494-BurTARs.A030	contig022353-TilTARs.A036	0.341	0.919	0.371
contig055697-BurTARs.A022	contig030440-ZebTARs.A029	0.274	0.739	0.371
contig039640-TilTAR.A002	contig022355-TilTARs.A039	0.424	1.144	0.371
contig086351-BriTARs.A021	contig056134-TilTARs.A058	0.302	0.813	0.371
contig022341-TilTARs.A032	contig053145-ZebTARs.A028	0.316	0.850	0.371

contig059766-BurTARs.A029	contig022353-TilTARs.A036	0.336	0.906	0.371
contig029633-BriTAR.A003	contig022337-TilTARs.A031	0.458	1.233	0.371
contig022378-TilTARs.A051	contig066056-ZebTARs.A017	0.319	0.860	0.371
contig059766-BurTARs.A029	contig057305-BurTARs.A031	0.274	0.738	0.371
contig084886-BriTARs.A017	contig022354-TilTARs.A038	0.026	0.070	0.371
contig059766-BurTARs.A029	contig007524-TilTARs.A026	0.240	0.646	0.371
contig082565-BriTARs.A022	contig039639-TilTAR.A001	0.371	0.999	0.371
contig062039-NyeTARs.A022	contig062676-ZebTARs.A019	0.222	0.599	0.371
contig059766-BurTARs.A029	contig035377-NyeTARs.A023	0.290	0.780	0.371
contig084886-BriTARs.A017	contig003909-ZebTAR.A003	0.386	1.039	0.371
contig060707-BurTARs.A015	contig022383-TilTARs.A054	0.299	0.805	0.371
contig061417-ZebTARs.A020	contig053145-ZebTARs.A028	0.324	0.871	0.371
contig057148-BurTARs.A028	contig022377-TilTARs.A050	0.150	0.405	0.372
contig022390-TilTARs.A055	contig022320-TilTARs.A059	0.206	0.555	0.372
contig086337-BriTARs.A019	contig046007-NyeTARs.A017	0.320	0.860	0.372
contig086337-BriTARs.A019	contig040586-ZebTAR.A001	0.413	1.111	0.372
contig022334-TilTARs.A030	contig061417-ZebTARs.A020	0.151	0.407	0.372
contig022324-TilTARs.A027	contig022362-TilTARs.A042	0.157	0.422	0.372
contig022356-TilTARs.A040	contig022362-TilTARs.A042	0.138	0.371	0.372
contig029633-BriTAR.A003	contig022365-TilTARs.A045	0.447	1.203	0.372
contig054630-BurTARs.A023	contig035375-NyeTARs.A013	0.343	0.922	0.372
contig084880-BriTARs.A016	contig039640-TilTAR.A003	0.440	1.185	0.372
contig046010-NyeTARs.A019	contig061410-ZebTARs.A021	0.244	0.657	0.372
contig022334-TilTARs.A030	contig022345-TilTARs.A034	0.154	0.414	0.372
contig060707-BurTARs.A015	contig062677-ZebTARs.A018	0.321	0.864	0.372
contig023443-TilTAR.A005	contig022355-TilTARs.A039	0.370	0.996	0.372
contig022334-TilTARs.A030	contig030445-ZebTARs.A026	0.217	0.583	0.372
contig007512-TilTARs.A024	contig022330-TilTARs.A028	0.320	0.860	0.372
contig035381-NyeTARs.A015	contig039640-TilTAR.A002	0.373	1.002	0.372
contig022345-TilTARs.A034	contig022390-TilTARs.A055	0.259	0.695	0.372
contig066285-ZebTARs.A016	contig066056-ZebTARs.A017	0.322	0.865	0.372
contig057305-BurTARs.A031	contig056134-TilTARs.A058	0.305	0.819	0.372
contig022337-TilTARs.A031	contig030471-ZebTARs.A024	0.121	0.325	0.372
contig086337-BriTARs.A019	contig022368-TilTARs.A046	0.313	0.841	0.372
contig039639-TilTAR.A001	contig022324-TilTARs.A027	0.457	1.228	0.372
contig057148-BurTARs.A028	contig022383-TilTARs.A054	0.252	0.678	0.372
contig022365-TilTARs.A045	contig053145-ZebTARs.A028	0.313	0.841	0.372
contig061977-BurTARs.A012	contig022334-TilTARs.A030	0.210	0.564	0.372
contig029633-BriTAR.A003	contig053139-ZebTARs.A023	0.422	1.133	0.372
contig049534-BurTARs.A025	contig022390-TilTARs.A055	0.262	0.704	0.372
contig035381-NyeTARs.A015	contig066056-ZebTARs.A017	0.307	0.824	0.372
contig084868-BriTARs.A014	contig060707-BurTARs.A015	0.305	0.818	0.372
contig038663-NyeTAR.A005	contig062677-ZebTARs.A018	0.415	1.114	0.372
contig082565-BriTARs.A022	contig060707-BurTARs.A015	0.303	0.814	0.372
contig039640-TilTAR.A002	contig022345-TilTARs.A034	0.395	1.061	0.372
contig049534-BurTARs.A025	contig066056-ZebTARs.A017	0.319	0.856	0.372
contig022390-TilTARs.A056	contig056134-TilTARs.A058	0.306	0.822	0.373
contig086351-BriTARs.A021	contig059766-BurTARs.A029	0.273	0.733	0.373
contig022330-TilTARs.A028	contig022334-TilTARs.A030	0.148	0.396	0.373
contig020038-BurTAR.A002	contig022354-TilTARs.A037	0.367	0.986	0.373
contig084887-BriTARs.A018	contig007512-TilTARs.A024	0.335	0.899	0.373
contig086344-BriTARs.A020	contig022341-TilTARs.A032	0.336	0.901	0.373
contig056021-BurTARs.A020	contig022320-TilTARs.A059	0.107	0.286	0.373

contig029633-BriTAR.A003	contig045088-TilTARs.A057	0.459	1.230	0.373
contig022334-TilTARs.A030	contig022379-TilTARs.A052	0.211	0.566	0.373
contig046011-NyeTARs.A016	contig039640-TilTAR.A003	0.493	1.322	0.373
contig054630-BurTARs.A023	contig035377-NyeTARs.A023	0.342	0.917	0.373
contig022379-TilTARs.A052	contig040586-ZebTAR.A002	0.417	1.119	0.373
contig035381-NyeTARs.A015	contig056134-TilTARs.A058	0.273	0.732	0.373
contig059766-BurTARs.A029	contig022363-TilTARs.A043	0.356	0.954	0.373
contig065494-BurTARs.A030	contig022363-TilTARs.A043	0.356	0.954	0.373
contig040586-ZebTAR.A002	contig066285-ZebTARs.A016	0.443	1.187	0.373
contig084876-BriTARs.A015	contig039639-TilTAR.A001	0.398	1.066	0.373
contig057145-BurTARs.A018	contig022320-TilTARs.A059	0.112	0.301	0.373
contig045302-BurTAR.A001	contig022341-TilTARs.A032	0.424	1.136	0.373
contig086337-BriTARs.A019	contig056020-BurTARs.A021	0.338	0.905	0.374
contig039639-TilTAR.A001	contig062676-ZebTARs.A019	0.381	1.020	0.374
contig084887-BriTARs.A018	contig022375-TilTARs.A048	0.131	0.351	0.374
contig059673-BurTARs.A016	contig065494-BurTARs.A030	0.260	0.696	0.374
contig084887-BriTARs.A018	contig086337-BriTARs.A019	0.322	0.861	0.374
contig059673-BurTARs.A016	contig066056-ZebTARs.A017	0.301	0.805	0.374
contig086337-BriTARs.A019	contig066890-ZebTARs.A014	0.273	0.732	0.374
contig056021-BurTARs.A020	contig059766-BurTARs.A029	0.334	0.892	0.374
contig022330-TilTARs.A028	contig022355-TilTARs.A039	0.107	0.286	0.374
contig084886-BriTARs.A017	contig007512-TilTARs.A024	0.330	0.882	0.374
contig055697-BurTARs.A022	contig022382-TilTARs.A053	0.297	0.796	0.374
contig055697-BurTARs.A022	contig022324-TilTARs.A027	0.325	0.868	0.374
contig046010-NyeTARs.A019	contig059768-ZebTARs.A022	0.369	0.987	0.374
contig061977-BurTARs.A012	contig039640-TilTAR.A003	0.377	1.007	0.374
contig049534-BurTARs.A025	contig032272-NyeTAR.A004	0.392	1.048	0.374
contig022363-TilTARs.A043	contig022320-TilTARs.A059	0.132	0.353	0.374
contig056020-BurTARs.A021	contig007518-TilTARs.A019	0.367	0.980	0.374
contig084876-BriTARs.A015	contig066056-ZebTARs.A017	0.315	0.843	0.374
contig084887-BriTARs.A018	contig007518-TilTARs.A019	0.359	0.960	0.374
contig059673-BurTARs.A016	contig066330-ZebTARs.A027	0.259	0.693	0.374
contig058002-NyeTARs.A025	contig062676-ZebTARs.A019	0.310	0.828	0.374
contig022334-TilTARs.A029	contig030471-ZebTARs.A024	0.108	0.289	0.374
contig055697-BurTARs.A022	contig022390-TilTARs.A056	0.285	0.762	0.374
contig060292-NyeTARs.A027	contig053145-ZebTARs.A028	0.272	0.727	0.374
contig046011-NyeTARs.A016	contig039639-TilTAR.A001	0.503	1.344	0.374
contig061977-BurTARs.A012	contig053139-ZebTARs.A023	0.261	0.698	0.374
contig046010-NyeTARs.A019	contig066056-ZebTARs.A017	0.324	0.865	0.375
contig023443-TilTAR.A005	contig022377-TilTARs.A050	0.363	0.968	0.375
contig060105-NyeTARs.A018	contig022378-TilTARs.A051	0.318	0.847	0.375
contig061433-BurTARs.A013	contig022355-TilTARs.A039	0.157	0.419	0.375
contig084887-BriTARs.A018	contig022334-TilTARs.A029	0.106	0.282	0.375
contig046013-NyeTARs.A021	contig039640-TilTAR.A002	0.424	1.130	0.375
contig057148-BurTARs.A028	contig045088-TilTARs.A057	0.372	0.992	0.375
contig022324-TilTARs.A027	contig022363-TilTARs.A044	0.186	0.497	0.375
contig059766-BurTARs.A029	contig065494-BurTARs.A030	0.011	0.030	0.375
contig022337-TilTARs.A031	contig022357-TilTARs.A041	0.136	0.362	0.375
contig066691-ZebTARs.A015	contig066330-ZebTARs.A027	0.350	0.934	0.375
contig059766-BurTARs.A029	contig040586-ZebTAR.A002	0.383	1.021	0.375
contig041024-BurTARs.A026	contig022334-TilTARs.A029	0.106	0.283	0.375
contig086344-BriTARs.A020	contig058002-NyeTARs.A025	0.300	0.799	0.375
contig022354-TilTARs.A037	contig053145-ZebTARs.A028	0.309	0.823	0.375

contig086337-BriTARs.A019	contig057148-BurTARs.A028	0.339	0.903	0.375
contig054630-BurTARs.A023	contig046010-NyeTARs.A019	0.382	1.017	0.375
contig061977-BurTARs.A012	contig022341-TilTARs.A032	0.229	0.610	0.375
contig055697-BurTARs.A022	contig030471-ZebTARs.A024	0.347	0.926	0.375
contig059673-BurTARs.A016	contig039640-TilTAR.A003	0.453	1.209	0.375
contig020038-BurTAR.A002	contig022357-TilTARs.A041	0.398	1.061	0.375
contig061977-BurTARs.A012	contig060707-BurTARs.A015	0.305	0.812	0.375
contig045302-BurTAR.A001	contig061091-BurTARs.A014	0.428	1.141	0.375
contig059766-BurTARs.A029	contig022341-TilTARs.A032	0.320	0.852	0.375
contig022356-TilTARs.A040	contig066285-ZebTARs.A016	0.152	0.406	0.375
contig060707-BurTARs.A015	contig030445-ZebTARs.A026	0.305	0.813	0.375
contig049534-BurTARs.A025	contig022365-TilTARs.A045	0.143	0.381	0.376
contig055697-BurTARs.A022	contig046010-NyeTARs.A019	0.332	0.885	0.376
contig049534-BurTARs.A025	contig040586-ZebTAR.A002	0.443	1.181	0.376
contig057145-BurTARs.A018	contig022365-TilTARs.A045	0.143	0.380	0.376
contig046007-NyeTARs.A017	contig040586-ZebTAR.A001	0.442	1.176	0.376
contig059766-BurTARs.A029	contig046007-NyeTARs.A017	0.330	0.878	0.376
contig059766-BurTARs.A029	contig061417-ZebTARs.A020	0.328	0.873	0.376
contig007512-TilTARs.A024	contig022363-TilTARs.A043	0.347	0.923	0.376
contig007512-TilTARs.A024	contig022375-TilTARs.A048	0.309	0.822	0.376
contig065494-BurTARs.A030	contig030471-ZebTARs.A024	0.356	0.946	0.376
contig022330-TilTARs.A028	contig040586-ZebTAR.A002	0.423	1.125	0.376
contig061977-BurTARs.A012	contig053145-ZebTARs.A028	0.261	0.693	0.376
contig039640-TilTAR.A003	contig007518-TilTARs.A019	0.458	1.219	0.376
contig007512-TilTARs.A024	contig066285-ZebTARs.A016	0.345	0.918	0.376
contig022349-TilTARs.A035	contig059768-ZebTARs.A022	0.398	1.058	0.376
contig022368-TilTARs.A046	contig053139-ZebTARs.A023	0.317	0.842	0.376
contig046010-NyeTARs.A019	contig066285-ZebTARs.A016	0.164	0.437	0.376
contig022345-TilTARs.A034	contig022363-TilTARs.A043	0.131	0.348	0.376
contig061977-BurTARs.A012	contig057145-BurTARs.A018	0.238	0.632	0.376
contig040586-ZebTAR.A002	contig053139-ZebTARs.A023	0.405	1.077	0.376
contig086337-BriTARs.A019	contig022349-TilTARs.A035	0.329	0.874	0.376
contig039642-TilTAR.A004	contig022353-TilTARs.A036	0.457	1.215	0.376
contig046007-NyeTARs.A017	contig007518-TilTARs.A019	0.365	0.971	0.376
contig057301-BurTARs.A017	contig053145-ZebTARs.A028	0.281	0.746	0.376
contig022375-TilTARs.A048	contig053139-ZebTARs.A023	0.304	0.809	0.376
contig057148-BurTARs.A028	contig042499-NyeTARs.A028	0.366	0.973	0.376
contig022330-TilTARs.A028	contig040586-ZebTAR.A001	0.421	1.119	0.376
contig066056-ZebTARs.A017	contig030464-ZebTARs.A025	0.326	0.867	0.376
contig035381-NyeTARs.A015	contig022334-TilTARs.A030	0.223	0.592	0.376
contig065494-BurTARs.A030	contig057305-BurTARs.A031	0.278	0.739	0.377
contig086344-BriTARs.A020	contig049540-BurTARs.A024	0.325	0.864	0.377
contig022353-TilTARs.A036	contig030464-ZebTARs.A025	0.140	0.372	0.377
contig022375-TilTARs.A048	contig040586-ZebTAR.A002	0.407	1.082	0.377
contig059766-BurTARs.A029	contig022365-TilTARs.A045	0.317	0.843	0.377
contig084880-BriTARs.A016	contig039640-TilTAR.A002	0.465	1.235	0.377
contig061977-BurTARs.A012	contig030464-ZebTARs.A025	0.248	0.659	0.377
contig049534-BurTARs.A025	contig057148-BurTARs.A028	0.168	0.446	0.377
contig025313-BriTAR.A002	contig022357-TilTARs.A041	0.401	1.064	0.377
contig059766-BurTARs.A029	contig022354-TilTARs.A037	0.311	0.825	0.377
contig060707-BurTARs.A015	contig058002-NyeTARs.A025	0.296	0.785	0.377
contig029633-BriTAR.A003	contig056020-BurTARs.A021	0.508	1.347	0.377
contig059673-BurTARs.A016	contig055697-BurTARs.A022	0.259	0.686	0.377

contig055697-BurTARs.A022	contig022354-TilTARs.A038	0.323	0.857	0.377
contig086337-BriTARs.A019	contig035376-NyeTARs.A014	0.274	0.727	0.377
contig035377-NyeTARs.A023	contig059768-ZebTARs.A022	0.336	0.891	0.377
contig065494-BurTARs.A030	contig040586-ZebTAR.A001	0.390	1.035	0.377
contig022324-TilTARs.A027	contig030471-ZebTARs.A024	0.155	0.410	0.377
contig055697-BurTARs.A022	contig022363-TilTARs.A043	0.354	0.939	0.377
contig007512-TilTARs.A024	contig022334-TilTARs.A030	0.342	0.907	0.377
contig022324-TilTARs.A027	contig003909-ZebTAR.A003	0.409	1.083	0.377
contig007512-TilTARs.A024	contig022368-TilTARs.A046	0.313	0.830	0.377
contig035381-NyeTARs.A015	contig039639-TilTAR.A001	0.374	0.993	0.377
contig084868-BriTARs.A014	contig061977-BurTARs.A012	0.151	0.399	0.377
contig046007-NyeTARs.A017	contig066330-ZebTARs.A027	0.328	0.869	0.377
contig022354-TilTARs.A037	contig066330-ZebTARs.A027	0.316	0.838	0.377
contig045302-BurTAR.A001	contig065494-BurTARs.A030	0.395	1.047	0.377
contig056134-TilTARs.A058	contig022320-TilTARs.A059	0.332	0.880	0.377
contig056021-BurTARs.A020	contig046010-NyeTARs.A019	0.135	0.356	0.377
contig056020-BurTARs.A021	contig022356-TilTARs.A040	0.117	0.311	0.378
contig084868-BriTARs.A014	contig062039-NyeTARs.A022	0.232	0.615	0.378
contig061433-BurTARs.A013	contig022341-TilTARs.A032	0.163	0.432	0.378
contig039639-TilTAR.A001	contig045088-TilTARs.A057	0.454	1.203	0.378
contig065494-BurTARs.A030	contig061417-ZebTARs.A020	0.337	0.893	0.378
contig053139-ZebTARs.A023	contig030440-ZebTARs.A029	0.274	0.726	0.378
contig065494-BurTARs.A030	contig007524-TilTARs.A026	0.246	0.652	0.378
contig022324-TilTARs.A027	contig053139-ZebTARs.A023	0.320	0.846	0.378
contig061091-BurTARs.A014	contig038663-NyeTAR.A005	0.442	1.170	0.378
contig030471-ZebTARs.A024	contig066330-ZebTARs.A027	0.356	0.941	0.378
contig035376-NyeTARs.A014	contig022334-TilTARs.A030	0.222	0.589	0.378
contig084886-BriTARs.A017	contig020038-BurTAR.A002	0.389	1.031	0.378
contig022349-TilTARs.A035	contig053139-ZebTARs.A023	0.332	0.878	0.378
contig040586-ZebTAR.A002	contig066691-ZebTARs.A015	0.444	1.175	0.378
contig054630-BurTARs.A023	contig030464-ZebTARs.A025	0.384	1.016	0.378
contig029633-BriTAR.A003	contig022363-TilTARs.A044	0.417	1.103	0.378
contig086351-BriTARs.A021	contig065494-BurTARs.A030	0.277	0.733	0.378
contig022363-TilTARs.A044	contig053145-ZebTARs.A028	0.315	0.833	0.378
contig022378-TilTARs.A051	contig066330-ZebTARs.A027	0.293	0.776	0.378
contig086337-BriTARs.A019	contig022341-TilTARs.A032	0.312	0.825	0.378
contig056134-TilTARs.A058	contig030445-ZebTARs.A026	0.269	0.712	0.378
contig061433-BurTARs.A013	contig039642-TilTAR.A004	0.447	1.182	0.378
contig065494-BurTARs.A030	contig038663-NyeTAR.A005	0.399	1.055	0.378
contig022356-TilTARs.A040	contig022320-TilTARs.A059	0.099	0.263	0.378
contig086337-BriTARs.A019	contig022345-TilTARs.A034	0.303	0.800	0.378
contig022341-TilTARs.A032	contig053139-ZebTARs.A023	0.312	0.824	0.378
contig038663-NyeTAR.A005	contig022382-TilTARs.A053	0.427	1.129	0.378
contig022349-TilTARs.A035	contig040586-ZebTAR.A001	0.441	1.165	0.378
contig029633-BriTAR.A003	contig086337-BriTARs.A019	0.421	1.112	0.378
contig035375-NyeTARs.A013	contig030440-ZebTARs.A029	0.012	0.032	0.378
contig040586-ZebTAR.A001	contig066691-ZebTARs.A015	0.442	1.169	0.379
contig058002-NyeTARs.A025	contig040586-ZebTAR.A001	0.428	1.131	0.379
contig022330-TilTARs.A028	contig053145-ZebTARs.A028	0.323	0.853	0.379
contig046007-NyeTARs.A017	contig022320-TilTARs.A059	0.111	0.293	0.379
contig056021-BurTARs.A020	contig055697-BurTARs.A022	0.324	0.855	0.379
contig045302-BurTAR.A001	contig022345-TilTARs.A034	0.399	1.055	0.379
contig059766-BurTARs.A029	contig060292-NyeTARs.A027	0.272	0.718	0.379

contig046007-NyeTARs.A017	contig022354-TilTARs.A037	0.171	0.452	0.379
contig057148-BurTARs.A028	contig053139-ZebTARs.A023	0.336	0.886	0.379
contig022343-TilTARs.A033	contig059768-ZebTARs.A022	0.357	0.944	0.379
contig084886-BriTARs.A017	contig038663-NyeTAR.A005	0.453	1.196	0.379
contig084887-BriTARs.A018	contig022324-TilTARs.A027	0.168	0.443	0.379
contig034854-BurTARs.A027	contig053139-ZebTARs.A023	0.291	0.767	0.379
contig046010-NyeTARs.A019	contig040586-ZebTAR.A001	0.454	1.199	0.379
contig057148-BurTARs.A028	contig022341-TilTARs.A032	0.127	0.336	0.379
contig046010-NyeTARs.A019	contig022377-TilTARs.A050	0.147	0.387	0.379
contig041024-BurTARs.A026	contig022368-TilTARs.A047	0.119	0.314	0.379
contig084887-BriTARs.A018	contig041024-BurTARs.A026	0.104	0.275	0.379
contig007524-TilTARs.A026	contig066330-ZebTARs.A027	0.250	0.658	0.379
contig061433-BurTARs.A013	contig022365-TilTARs.A045	0.167	0.441	0.379
contig007512-TilTARs.A024	contig022353-TilTARs.A036	0.341	0.898	0.379
contig066056-ZebTARs.A017	contig062676-ZebTARs.A019	0.296	0.781	0.379
contig055697-BurTARs.A022	contig022330-TilTARs.A028	0.313	0.825	0.379
contig040586-ZebTAR.A001	contig066330-ZebTARs.A027	0.395	1.043	0.379
contig061977-BurTARs.A012	contig059768-ZebTARs.A022	0.303	0.798	0.379
contig022341-TilTARs.A032	contig061410-ZebTARs.A021	0.231	0.610	0.379
contig029633-BriTAR.A003	contig022355-TilTARs.A039	0.461	1.217	0.379
contig045302-BurTAR.A001	contig066330-ZebTARs.A027	0.400	1.056	0.379
contig084887-BriTARs.A018	contig022320-TilTARs.A059	0.112	0.295	0.379
contig058002-NyeTARs.A025	contig053139-ZebTARs.A023	0.241	0.635	0.379
contig029633-BriTAR.A003	contig058002-NyeTARs.A025	0.443	1.169	0.379
contig039642-TilTAR.A004	contig022377-TilTARs.A050	0.414	1.092	0.379
contig025313-BriTAR.A002	contig049534-BurTARs.A025	0.389	1.024	0.379
contig046007-NyeTARs.A017	contig059768-ZebTARs.A022	0.349	0.918	0.380
contig086337-BriTARs.A019	contig030471-ZebTARs.A024	0.354	0.932	0.380
contig039640-TilTAR.A002	contig007524-TilTARs.A026	0.440	1.158	0.380
contig022345-TilTARs.A034	contig061410-ZebTARs.A021	0.234	0.615	0.380
contig084880-BriTARs.A016	contig022334-TilTARs.A030	0.212	0.558	0.380
contig022390-TilTARs.A056	contig053139-ZebTARs.A023	0.287	0.755	0.380
contig084886-BriTARs.A017	contig022324-TilTARs.A027	0.161	0.425	0.380
contig007512-TilTARs.A024	contig022390-TilTARs.A056	0.294	0.774	0.380
contig060707-BurTARs.A015	contig062676-ZebTARs.A019	0.305	0.803	0.380
contig049534-BurTARs.A025	contig022356-TilTARs.A040	0.149	0.392	0.380
contig057145-BurTARs.A018	contig060105-NyeTARs.A018	0.350	0.922	0.380
contig038663-NyeTAR.A005	contig066330-ZebTARs.A027	0.404	1.064	0.380
contig060292-NyeTARs.A027	contig056134-TilTARs.A058	0.303	0.797	0.380
contig058002-NyeTARs.A025	contig040586-ZebTAR.A002	0.431	1.133	0.380
contig060707-BurTARs.A015	contig007524-TilTARs.A026	0.289	0.761	0.380
contig038663-NyeTAR.A005	contig022349-TilTARs.A035	0.443	1.165	0.380
contig022341-TilTARs.A032	contig022349-TilTARs.A035	0.133	0.351	0.380
contig022368-TilTARs.A047	contig066056-ZebTARs.A017	0.318	0.837	0.380
contig060105-NyeTARs.A018	contig007520-TilTARs.A025	0.286	0.753	0.380
contig061433-BurTARs.A013	contig038663-NyeTAR.A005	0.454	1.194	0.380
contig055697-BurTARs.A022	contig022345-TilTARs.A034	0.307	0.809	0.380
contig049534-BurTARs.A025	contig003909-ZebTAR.A003	0.392	1.032	0.380
contig034854-BurTARs.A027	contig039639-TilTAR.A001	0.375	0.985	0.380
contig065494-BurTARs.A030	contig040586-ZebTAR.A002	0.381	1.002	0.380
contig039640-TilTAR.A003	contig030445-ZebTARs.A026	0.367	0.964	0.380
contig022353-TilTARs.A036	contig059768-ZebTARs.A022	0.373	0.982	0.380
contig022363-TilTARs.A043	contig053139-ZebTARs.A023	0.349	0.917	0.380

contig086344-BriTARs.A020	contig022382-TilTARs.A053	0.324	0.853	0.380
contig084886-BriTARs.A017	contig022343-TilTARs.A033	0.122	0.321	0.380
contig056020-BurTARs.A021	contig059768-ZebTARs.A022	0.351	0.921	0.380
contig022363-TilTARs.A043	contig022383-TilTARs.A054	0.280	0.735	0.381
contig045302-BurTAR.A001	contig022382-TilTARs.A053	0.419	1.100	0.381
contig039642-TilTAR.A004	contig022379-TilTARs.A052	0.407	1.069	0.381
contig046014-NyeTARs.A024	contig040586-ZebTAR.A001	0.431	1.132	0.381
contig061091-BurTARs.A014	contig039642-TilTAR.A004	0.437	1.147	0.381
contig086344-BriTARs.A020	contig061977-BurTARs.A012	0.303	0.796	0.381
contig056021-BurTARs.A020	contig065494-BurTARs.A030	0.342	0.897	0.381
contig061433-BurTARs.A013	contig022334-TilTARs.A030	0.211	0.553	0.381
contig055697-BurTARs.A022	contig030464-ZebTARs.A025	0.337	0.884	0.381
contig057148-BurTARs.A028	contig038663-NyeTAR.A005	0.460	1.208	0.381
contig084868-BriTARs.A014	contig086344-BriTARs.A020	0.316	0.828	0.381
contig039640-TilTAR.A002	contig022368-TilTARs.A047	0.423	1.109	0.381
contig061977-BurTARs.A012	contig059766-BurTARs.A029	0.265	0.694	0.381
contig039640-TilTAR.A003	contig045088-TilTARs.A057	0.457	1.199	0.381
contig029633-BriTAR.A003	contig022379-TilTARs.A052	0.424	1.112	0.381
contig057145-BurTARs.A018	contig022368-TilTARs.A046	0.140	0.369	0.381
contig056020-BurTARs.A021	contig038663-NyeTAR.A005	0.459	1.203	0.381
contig022320-TilTARs.A059	contig053145-ZebTARs.A028	0.313	0.822	0.381
contig022382-TilTARs.A053	contig062677-ZebTARs.A018	0.014	0.037	0.381
contig060707-BurTARs.A015	contig056200-NyeTARs.A029	0.322	0.846	0.381
contig061091-BurTARs.A014	contig066330-ZebTARs.A027	0.350	0.919	0.381
contig039642-TilTAR.A004	contig022341-TilTARs.A032	0.416	1.090	0.381
contig055697-BurTARs.A022	contig022363-TilTARs.A044	0.317	0.832	0.381
contig045302-BurTAR.A001	contig022349-TilTARs.A035	0.438	1.148	0.381
contig029633-BriTAR.A003	contig041024-BurTARs.A026	0.429	1.126	0.381
contig022343-TilTARs.A033	contig030471-ZebTARs.A024	0.110	0.290	0.382
contig046010-NyeTARs.A019	contig022349-TilTARs.A035	0.146	0.384	0.382
contig035381-NyeTARs.A015	contig022345-TilTARs.A034	0.242	0.634	0.382
contig022354-TilTARs.A038	contig066056-ZebTARs.A017	0.314	0.822	0.382
contig057301-BurTARs.A017	contig066330-ZebTARs.A027	0.287	0.751	0.382
contig055697-BurTARs.A022	contig061417-ZebTARs.A020	0.318	0.833	0.382
contig057305-BurTARs.A031	contig066330-ZebTARs.A027	0.284	0.744	0.382
contig045302-BurTAR.A001	contig022334-TilTARs.A029	0.428	1.122	0.382
contig086337-BriTARs.A019	contig046010-NyeTARs.A019	0.340	0.889	0.382
contig022362-TilTARs.A042	contig022320-TilTARs.A059	0.127	0.332	0.382
contig065494-BurTARs.A030	contig046007-NyeTARs.A017	0.332	0.868	0.382
contig038663-NyeTAR.A005	contig022356-TilTARs.A040	0.437	1.145	0.382
contig022382-TilTARs.A053	contig053139-ZebTARs.A023	0.297	0.777	0.382
contig022320-TilTARs.A059	contig040586-ZebTAR.A001	0.435	1.140	0.382
contig007512-TilTARs.A024	contig022334-TilTARs.A029	0.323	0.846	0.382
contig084868-BriTARs.A014	contig060105-NyeTARs.A018	0.310	0.812	0.382
contig039640-TilTAR.A002	contig045088-TilTARs.A057	0.459	1.203	0.382
contig029633-BriTAR.A003	contig057301-BurTARs.A017	0.464	1.215	0.382
contig041024-BurTARs.A026	contig022330-TilTARs.A028	0.108	0.282	0.382
contig057145-BurTARs.A018	contig022345-TilTARs.A034	0.116	0.305	0.382
contig022341-TilTARs.A032	contig040586-ZebTAR.A002	0.425	1.114	0.382
contig057301-BurTARs.A017	contig054630-BurTARs.A023	0.360	0.943	0.382
contig086344-BriTARs.A020	contig057305-BurTARs.A031	0.292	0.765	0.382
contig061417-ZebTARs.A020	contig066330-ZebTARs.A027	0.340	0.888	0.382
contig022320-TilTARs.A059	contig061417-ZebTARs.A020	0.107	0.280	0.382

contig022354-TilTARs.A038	contig061410-ZebTARs.A021	0.231	0.604	0.382
contig057301-BurTARs.A017	contig065494-BurTARs.A030	0.286	0.749	0.382
contig057301-BurTARs.A017	contig056134-TilTARs.A058	0.305	0.797	0.382
contig057301-BurTARs.A017	contig039640-TilTAR.A003	0.456	1.193	0.382
contig084886-BriTARs.A017	contig066056-ZebTARs.A017	0.317	0.828	0.382
contig061410-ZebTARs.A021	contig053145-ZebTARs.A028	0.263	0.687	0.382
contig061410-ZebTARs.A021	contig053139-ZebTARs.A023	0.262	0.686	0.382
contig054630-BurTARs.A023	contig022357-TilTARs.A041	0.398	1.040	0.382
contig086344-BriTARs.A020	contig022383-TilTARs.A054	0.306	0.800	0.382
contig042499-NyeTARs.A028	contig059768-ZebTARs.A022	0.348	0.911	0.382
contig056021-BurTARs.A020	contig030464-ZebTARs.A025	0.136	0.356	0.383
contig020038-BurTAR.A002	contig049534-BurTARs.A025	0.397	1.037	0.383
contig086351-BriTARs.A021	contig066056-ZebTARs.A017	0.287	0.749	0.383
contig060707-BurTARs.A015	contig035381-NyeTARs.A015	0.307	0.803	0.383
contig065494-BurTARs.A030	contig035377-NyeTARs.A023	0.293	0.765	0.383
contig065494-BurTARs.A030	contig022365-TilTARs.A045	0.325	0.849	0.383
contig022334-TilTARs.A029	contig030464-ZebTARs.A025	0.135	0.354	0.383
contig059766-BurTARs.A029	contig022330-TilTARs.A028	0.327	0.854	0.383
contig060105-NyeTARs.A018	contig022379-TilTARs.A052	0.306	0.799	0.383
contig059768-ZebTARs.A022	contig030464-ZebTARs.A025	0.377	0.985	0.383
contig040586-ZebTAR.A002	contig066330-ZebTARs.A027	0.393	1.026	0.383
contig060707-BurTARs.A015	contig035376-NyeTARs.A014	0.315	0.822	0.383
contig046013-NyeTARs.A021	contig039642-TilTAR.A004	0.439	1.146	0.383
contig022330-TilTARs.A028	contig030464-ZebTARs.A025	0.133	0.348	0.383
contig046010-NyeTARs.A019	contig022341-TilTARs.A032	0.128	0.334	0.383
contig042499-NyeTARs.A028	contig053145-ZebTARs.A028	0.278	0.727	0.383
contig029633-BriTAR.A003	contig022362-TilTARs.A042	0.440	1.149	0.383
contig086351-BriTARs.A021	contig066330-ZebTARs.A027	0.283	0.739	0.383
contig082565-BriTARs.A022	contig022390-TilTARs.A055	0.032	0.083	0.383
contig056134-TilTARs.A058	contig066890-ZebTARs.A014	0.302	0.789	0.383
contig086337-BriTARs.A019	contig038663-NyeTAR.A005	0.422	1.101	0.383
contig007520-TilTARs.A025	contig022343-TilTARs.A033	0.335	0.875	0.383
contig041024-BurTARs.A026	contig022337-TilTARs.A031	0.137	0.356	0.383
contig084868-BriTARs.A014	contig022345-TilTARs.A034	0.252	0.658	0.383
contig032272-NyeTAR.A004	contig022324-TilTARs.A027	0.409	1.066	0.383
contig039640-TilTAR.A002	contig007518-TilTARs.A019	0.463	1.208	0.383
contig035377-NyeTARs.A023	contig039639-TilTAR.A001	0.410	1.071	0.383
contig035376-NyeTARs.A014	contig039640-TilTAR.A003	0.383	0.999	0.383
contig022368-TilTARs.A046	contig022377-TilTARs.A050	0.147	0.384	0.383
contig061977-BurTARs.A012	contig058002-NyeTARs.A025	0.298	0.778	0.383
contig057301-BurTARs.A017	contig059766-BurTARs.A029	0.282	0.737	0.383
contig022343-TilTARs.A033	contig040586-ZebTAR.A001	0.428	1.117	0.383
contig059766-BurTARs.A029	contig042499-NyeTARs.A028	0.275	0.717	0.383
contig086337-BriTARs.A019	contig022354-TilTARs.A038	0.330	0.862	0.383
contig084880-BriTARs.A016	contig040586-ZebTAR.A001	0.447	1.166	0.384
contig029633-BriTAR.A003	contig022368-TilTARs.A046	0.443	1.154	0.384
contig041024-BurTARs.A026	contig066056-ZebTARs.A017	0.301	0.785	0.384
contig065494-BurTARs.A030	contig042499-NyeTARs.A028	0.279	0.728	0.384
contig055697-BurTARs.A022	contig007520-TilTARs.A025	0.227	0.591	0.384
contig059766-BurTARs.A029	contig022390-TilTARs.A056	0.287	0.747	0.384
contig046010-NyeTARs.A019	contig061417-ZebTARs.A020	0.130	0.338	0.384
contig086344-BriTARs.A020	contig061410-ZebTARs.A021	0.307	0.800	0.384
contig060105-NyeTARs.A018	contig062677-ZebTARs.A018	0.323	0.841	0.384

contig061977-BurTARs.A012	contig007520-TilTARs.A025	0.299	0.779	0.384
contig054630-BurTARs.A023	contig042499-NyeTARs.A028	0.349	0.910	0.384
contig057145-BurTARs.A018	contig046014-NyeTARs.A024	0.115	0.301	0.384
contig049534-BurTARs.A025	contig022354-TilTARs.A038	0.152	0.395	0.384
contig022337-TilTARs.A031	contig040586-ZebTAR.A001	0.441	1.148	0.384
contig022362-TilTARs.A042	contig022368-TilTARs.A046	0.038	0.100	0.384
contig065494-BurTARs.A030	contig060292-NyeTARs.A027	0.276	0.719	0.384
contig055697-BurTARs.A022	contig022379-TilTARs.A052	0.269	0.700	0.384
contig035377-NyeTARs.A023	contig066056-ZebTARs.A017	0.325	0.845	0.384
contig061433-BurTARs.A013	contig056021-BurTARs.A020	0.150	0.390	0.384
contig057145-BurTARs.A018	contig022375-TilTARs.A048	0.135	0.352	0.384
contig086337-BriTARs.A019	contig022330-TilTARs.A028	0.311	0.810	0.384
contig057145-BurTARs.A018	contig056021-BurTARs.A020	0.109	0.284	0.384
contig049534-BurTARs.A025	contig046010-NyeTARs.A019	0.164	0.427	0.384
contig053139-ZebTARs.A023	contig030471-ZebTARs.A024	0.353	0.918	0.384
contig022390-TilTARs.A056	contig053145-ZebTARs.A028	0.287	0.746	0.384
contig029633-BriTAR.A003	contig046007-NyeTARs.A017	0.475	1.234	0.385
contig054630-BurTARs.A023	contig022343-TilTARs.A033	0.364	0.946	0.385
contig059766-BurTARs.A029	contig022363-TilTARs.A044	0.316	0.821	0.385
contig057148-BurTARs.A028	contig061417-ZebTARs.A020	0.131	0.340	0.385
contig060105-NyeTARs.A018	contig030445-ZebTARs.A026	0.306	0.796	0.385
contig065494-BurTARs.A030	contig022324-TilTARs.A027	0.347	0.903	0.385
contig022365-TilTARs.A045	contig066330-ZebTARs.A027	0.325	0.844	0.385
contig035381-NyeTARs.A015	contig062039-NyeTARs.A022	0.207	0.537	0.385
contig022355-TilTARs.A039	contig040586-ZebTAR.A001	0.446	1.159	0.385
contig023443-TilTAR.A005	contig022357-TilTARs.A041	0.391	1.015	0.385
contig065494-BurTARs.A030	contig022390-TilTARs.A056	0.290	0.753	0.385
contig057148-BurTARs.A028	contig022362-TilTARs.A042	0.159	0.413	0.385
contig056021-BurTARs.A020	contig053139-ZebTARs.A023	0.323	0.839	0.385
contig022355-TilTARs.A039	contig056134-TilTARs.A058	0.337	0.876	0.385
contig045302-BurTAR.A001	contig022337-TilTARs.A031	0.439	1.140	0.385
contig061433-BurTARs.A013	contig057148-BurTARs.A028	0.165	0.429	0.385
contig049534-BurTARs.A025	contig023443-TilTAR.A005	0.387	1.006	0.385
contig045088-TilTARs.A057	contig040586-ZebTAR.A002	0.466	1.209	0.385
contig086337-BriTARs.A019	contig056021-BurTARs.A020	0.319	0.828	0.385
contig084886-BriTARs.A017	contig086337-BriTARs.A019	0.330	0.857	0.385
contig035377-NyeTARs.A023	contig022390-TilTARs.A056	0.045	0.117	0.385
contig022390-TilTARs.A055	contig066691-ZebTARs.A015	0.197	0.511	0.385
contig061433-BurTARs.A013	contig065494-BurTARs.A030	0.358	0.930	0.385
contig022354-TilTARs.A038	contig053139-ZebTARs.A023	0.322	0.837	0.385
contig022334-TilTARs.A029	contig040586-ZebTAR.A002	0.433	1.125	0.385
contig057305-BurTARs.A031	contig060105-NyeTARs.A018	0.285	0.740	0.385
contig046010-NyeTARs.A019	contig053139-ZebTARs.A023	0.336	0.873	0.385
contig022357-TilTARs.A041	contig040586-ZebTAR.A002	0.446	1.157	0.385
contig057145-BurTARs.A018	contig061410-ZebTARs.A021	0.240	0.622	0.385
contig056021-BurTARs.A020	contig066330-ZebTARs.A027	0.344	0.892	0.385
contig059766-BurTARs.A029	contig022320-TilTARs.A059	0.318	0.824	0.386
contig086337-BriTARs.A019	contig022320-TilTARs.A059	0.326	0.846	0.386
contig061410-ZebTARs.A021	contig030464-ZebTARs.A025	0.253	0.656	0.386
contig022320-TilTARs.A059	contig066056-ZebTARs.A017	0.310	0.802	0.386
contig022383-TilTARs.A054	contig061410-ZebTARs.A021	0.141	0.366	0.386
contig054630-BurTARs.A023	contig022390-TilTARs.A056	0.346	0.897	0.386
contig061977-BurTARs.A012	contig054630-BurTARs.A023	0.309	0.800	0.386

contig057148-BurTARs.A028	contig022356-TilTARs.A040	0.130	0.337	0.386
contig061433-BurTARs.A013	contig022357-TilTARs.A041	0.166	0.431	0.386
contig061977-BurTARs.A012	contig022354-TilTARs.A038	0.234	0.605	0.386
contig022353-TilTARs.A036	contig066285-ZebTARs.A016	0.147	0.381	0.386
contig022345-TilTARs.A034	contig022383-TilTARs.A054	0.241	0.624	0.386
contig086344-BriTARs.A020	contig007524-TilTARs.A026	0.295	0.763	0.386
contig055697-BurTARs.A022	contig022353-TilTARs.A036	0.326	0.844	0.386
contig022379-TilTARs.A052	contig022383-TilTARs.A054	0.144	0.373	0.386
contig022368-TilTARs.A047	contig056134-TilTARs.A058	0.356	0.922	0.386
contig057148-BurTARs.A028	contig022365-TilTARs.A045	0.152	0.393	0.386
contig061977-BurTARs.A012	contig022356-TilTARs.A040	0.241	0.624	0.386
contig025313-BriTAR.A002	contig022324-TilTARs.A027	0.406	1.050	0.386
contig061977-BurTARs.A012	contig040586-ZebTAR.A002	0.400	1.036	0.386
contig032272-NyeTAR.A004	contig022377-TilTARs.A050	0.381	0.985	0.386
contig060707-BurTARs.A015	contig049540-BurTARs.A024	0.322	0.834	0.386
contig061977-BurTARs.A012	contig056134-TilTARs.A058	0.285	0.738	0.387
contig022363-TilTARs.A044	contig003909-ZebTAR.A003	0.386	0.998	0.387
contig039642-TilTAR.A004	contig066285-ZebTARs.A016	0.436	1.127	0.387
contig086344-BriTARs.A020	contig030445-ZebTARs.A026	0.314	0.812	0.387
contig060707-BurTARs.A015	contig022349-TilTARs.A035	0.352	0.911	0.387
contig086337-BriTARs.A019	contig061977-BurTARs.A012	0.263	0.681	0.387
contig057301-BurTARs.A017	contig059768-ZebTARs.A022	0.354	0.915	0.387
contig029633-BriTAR.A003	contig084887-BriTARs.A018	0.478	1.235	0.387
contig022330-TilTARs.A028	contig022390-TilTARs.A055	0.203	0.524	0.387
contig022334-TilTARs.A029	contig022390-TilTARs.A055	0.203	0.524	0.387
contig056021-BurTARs.A020	contig022390-TilTARs.A055	0.203	0.524	0.387
contig086337-BriTARs.A019	contig007520-TilTARs.A025	0.224	0.579	0.387
contig061433-BurTARs.A013	contig046010-NyeTARs.A019	0.162	0.418	0.387
contig023443-TilTAR.A005	contig022324-TilTARs.A027	0.400	1.033	0.387
contig082565-BriTARs.A022	contig060105-NyeTARs.A018	0.308	0.797	0.387
contig086344-BriTARs.A020	contig066285-ZebTARs.A016	0.328	0.847	0.387
contig038663-NyeTAR.A005	contig022330-TilTARs.A028	0.428	1.106	0.387
contig055697-BurTARs.A022	contig022320-TilTARs.A059	0.323	0.836	0.387
contig086337-BriTARs.A019	contig030464-ZebTARs.A025	0.344	0.889	0.387
contig061433-BurTARs.A013	contig066330-ZebTARs.A027	0.360	0.929	0.387
contig035377-NyeTARs.A023	contig039640-TilTAR.A002	0.401	1.036	0.387
contig040586-ZebTAR.A002	contig030471-ZebTARs.A024	0.445	1.149	0.387
contig084887-BriTARs.A018	contig046014-NyeTARs.A024	0.106	0.274	0.387
contig059766-BurTARs.A029	contig046013-NyeTARs.A021	0.339	0.875	0.387
contig057148-BurTARs.A028	contig030471-ZebTARs.A024	0.152	0.393	0.387
contig022324-TilTARs.A027	contig022354-TilTARs.A038	0.154	0.398	0.387
contig035377-NyeTARs.A023	contig066330-ZebTARs.A027	0.295	0.761	0.387
contig022345-TilTARs.A034	contig022379-TilTARs.A052	0.225	0.582	0.387
contig029633-BriTAR.A003	contig022375-TilTARs.A048	0.414	1.070	0.387
contig022354-TilTARs.A038	contig022356-TilTARs.A040	0.114	0.295	0.388
contig086344-BriTARs.A020	contig022324-TilTARs.A027	0.338	0.872	0.388
contig084886-BriTARs.A017	contig055697-BurTARs.A022	0.329	0.849	0.388
contig084886-BriTARs.A017	contig030471-ZebTARs.A024	0.149	0.384	0.388
contig059766-BurTARs.A029	contig061410-ZebTARs.A021	0.267	0.688	0.388
contig056134-TilTARs.A058	contig066285-ZebTARs.A016	0.348	0.896	0.388
contig022357-TilTARs.A041	contig059768-ZebTARs.A022	0.391	1.008	0.388
contig056020-BurTARs.A021	contig022334-TilTARs.A029	0.118	0.304	0.388
contig022354-TilTARs.A038	contig066285-ZebTARs.A016	0.152	0.391	0.388

contig061977-BurTARs.A012	contig065494-BurTARs.A030	0.268	0.690	0.388
contig056020-BurTARs.A021	contig054630-BurTARs.A023	0.357	0.921	0.388
contig039640-TilTAR.A002	contig066691-ZebTARs.A015	0.430	1.109	0.388
contig086337-BriTARs.A019	contig022363-TilTARs.A044	0.304	0.782	0.388
contig059673-BurTARs.A016	contig039640-TilTAR.A002	0.462	1.191	0.388
contig041024-BurTARs.A026	contig022343-TilTARs.A033	0.124	0.319	0.388
contig022334-TilTARs.A029	contig040586-ZebTAR.A001	0.434	1.119	0.388
contig022330-TilTARs.A028	contig053139-ZebTARs.A023	0.317	0.815	0.388
contig039642-TilTAR.A004	contig030471-ZebTARs.A024	0.441	1.135	0.388
contig049534-BurTARs.A025	contig045088-TilTARs.A057	0.371	0.956	0.388
contig045302-BurTAR.A001	contig066691-ZebTARs.A015	0.434	1.118	0.388
contig060707-BurTARs.A015	contig057148-BurTARs.A028	0.341	0.877	0.388
contig058002-NyeTARs.A025	contig022343-TilTARs.A033	0.336	0.865	0.388
contig060707-BurTARs.A015	contig061410-ZebTARs.A021	0.308	0.794	0.389
contig042499-NyeTARs.A028	contig066330-ZebTARs.A027	0.285	0.732	0.389
contig007512-TilTARs.A024	contig022363-TilTARs.A044	0.326	0.838	0.389
contig022349-TilTARs.A035	contig053145-ZebTARs.A028	0.337	0.866	0.389
contig049534-BurTARs.A025	contig022343-TilTARs.A033	0.138	0.355	0.389
contig022334-TilTARs.A029	contig066691-ZebTARs.A015	0.110	0.283	0.389
contig045088-TilTARs.A057	contig056134-TilTARs.A058	0.301	0.773	0.389
contig054630-BurTARs.A023	contig022337-TilTARs.A031	0.382	0.983	0.389
contig086337-BriTARs.A019	contig022390-TilTARs.A056	0.287	0.737	0.389
contig049534-BurTARs.A025	contig039642-TilTAR.A004	0.436	1.121	0.389
contig084876-BriTARs.A015	contig055697-BurTARs.A022	0.281	0.722	0.389
contig045088-TilTARs.A057	contig059768-ZebTARs.A022	0.362	0.931	0.389
contig086344-BriTARs.A020	contig082565-BriTARs.A022	0.316	0.813	0.389
contig049534-BurTARs.A025	contig022337-TilTARs.A031	0.143	0.368	0.389
contig007512-TilTARs.A024	contig061417-ZebTARs.A020	0.326	0.838	0.389
contig007512-TilTARs.A024	contig022377-TilTARs.A050	0.347	0.892	0.389
contig022324-TilTARs.A027	contig040586-ZebTAR.A002	0.463	1.190	0.389
contig049534-BurTARs.A025	contig056134-TilTARs.A058	0.341	0.877	0.389
contig029633-BriTAR.A003	contig056134-TilTARs.A058	0.391	1.005	0.389
contig038663-NyeTAR.A005	contig058002-NyeTARs.A025	0.435	1.118	0.389
contig086337-BriTARs.A019	contig022334-TilTARs.A029	0.310	0.797	0.389
contig029633-BriTAR.A003	contig061977-BurTARs.A012	0.406	1.044	0.389
contig055697-BurTARs.A022	contig022334-TilTARs.A029	0.316	0.811	0.389
contig022324-TilTARs.A027	contig066330-ZebTARs.A027	0.349	0.898	0.389
contig042499-NyeTARs.A028	contig056134-TilTARs.A058	0.302	0.777	0.389
contig034854-BurTARs.A027	contig007512-TilTARs.A024	0.304	0.781	0.389
contig022334-TilTARs.A029	contig022334-TilTARs.A030	0.155	0.397	0.389
contig060292-NyeTARs.A027	contig066330-ZebTARs.A027	0.282	0.724	0.389
contig057145-BurTARs.A018	contig022368-TilTARs.A047	0.142	0.365	0.390
contig056020-BurTARs.A021	contig007520-TilTARs.A025	0.344	0.884	0.390
contig039640-TilTAR.A003	contig030440-ZebTARs.A029	0.377	0.968	0.390
contig046010-NyeTARs.A019	contig022355-TilTARs.A039	0.135	0.345	0.390
contig045302-BurTAR.A001	contig061977-BurTARs.A012	0.389	0.998	0.390
contig039642-TilTAR.A004	contig007520-TilTARs.A025	0.427	1.094	0.390
contig039639-TilTAR.A001	contig022383-TilTARs.A054	0.385	0.988	0.390
contig086351-BriTARs.A021	contig060707-BurTARs.A015	0.293	0.752	0.390
contig022334-TilTARs.A029	contig053145-ZebTARs.A028	0.326	0.837	0.390
contig045302-BurTAR.A001	contig022353-TilTARs.A036	0.450	1.155	0.390
contig022368-TilTARs.A046	contig056134-TilTARs.A058	0.344	0.881	0.390
contig022379-TilTARs.A052	contig061410-ZebTARs.A021	0.019	0.050	0.390

contig054630-BurTARs.A023	contig066691-ZebTARs.A015	0.384	0.984	0.390
contig061433-BurTARs.A013	contig046014-NyeTARs.A024	0.146	0.375	0.390
contig045302-BurTAR.A001	contig022355-TilTARs.A039	0.440	1.128	0.390
contig066890-ZebTARs.A014	contig053145-ZebTARs.A028	0.279	0.715	0.390
contig084876-BriTARs.A015	contig039640-TilTAR.A003	0.397	1.019	0.390
contig084886-BriTARs.A017	contig054630-BurTARs.A023	0.383	0.982	0.390
contig007520-TilTARs.A025	contig022355-TilTARs.A039	0.342	0.877	0.390
contig022390-TilTARs.A056	contig059768-ZebTARs.A022	0.340	0.872	0.390
contig039640-TilTAR.A002	contig022324-TilTARs.A027	0.449	1.152	0.390
contig086344-BriTARs.A020	contig060292-NyeTARs.A027	0.295	0.755	0.390
contig054630-BurTARs.A023	contig045088-TilTARs.A057	0.363	0.931	0.390
contig086337-BriTARs.A019	contig040586-ZebTAR.A002	0.407	1.041	0.390
contig058002-NyeTARs.A025	contig007512-TilTARs.A024	0.249	0.639	0.390
contig022345-TilTARs.A034	contig022365-TilTARs.A045	0.130	0.333	0.390
contig007512-TilTARs.A024	contig030471-ZebTARs.A024	0.351	0.898	0.390
contig057301-BurTARs.A017	contig039639-TilTAR.A001	0.465	1.192	0.391
contig007512-TilTARs.A024	contig022345-TilTARs.A034	0.313	0.802	0.391
contig061410-ZebTARs.A021	contig059768-ZebTARs.A022	0.312	0.798	0.391
contig046014-NyeTARs.A024	contig022354-TilTARs.A037	0.153	0.392	0.391
contig062039-NyeTARs.A022	contig030464-ZebTARs.A025	0.251	0.642	0.391
contig020038-BurTAR.A002	contig022363-TilTARs.A044	0.389	0.995	0.391
contig007524-TilTARs.A026	contig022383-TilTARs.A054	0.318	0.814	0.391
contig059673-BurTARs.A016	contig007512-TilTARs.A024	0.269	0.688	0.391
contig039642-TilTAR.A004	contig022368-TilTARs.A047	0.436	1.116	0.391
contig082565-BriTARs.A022	contig056134-TilTARs.A058	0.280	0.717	0.391
contig084868-BriTARs.A014	contig007524-TilTARs.A026	0.328	0.840	0.391
contig038663-NyeTAR.A005	contig066691-ZebTARs.A015	0.448	1.146	0.391
contig057148-BurTARs.A028	contig035381-NyeTARs.A015	0.246	0.628	0.391
contig084868-BriTARs.A014	contig057148-BurTARs.A028	0.268	0.685	0.391
contig022345-TilTARs.A034	contig053139-ZebTARs.A023	0.310	0.793	0.391
contig023443-TilTAR.A005	contig022390-TilTARs.A055	0.369	0.943	0.391
contig086337-BriTARs.A019	contig022324-TilTARs.A027	0.326	0.834	0.391
contig022341-TilTARs.A032	contig040586-ZebTAR.A001	0.430	1.099	0.391
contig022353-TilTARs.A036	contig022355-TilTARs.A039	0.078	0.199	0.391
contig032272-NyeTAR.A004	contig022363-TilTARs.A044	0.386	0.985	0.391
contig046013-NyeTARs.A021	contig040586-ZebTAR.A001	0.444	1.135	0.391
contig084868-BriTARs.A014	contig059673-BurTARs.A016	0.328	0.838	0.391
contig066285-ZebTARs.A016	contig030464-ZebTARs.A025	0.172	0.440	0.391
contig057145-BurTARs.A018	contig022343-TilTARs.A033	0.129	0.329	0.391
contig057148-BurTARs.A028	contig060105-NyeTARs.A018	0.341	0.870	0.391
contig084868-BriTARs.A014	contig039640-TilTAR.A003	0.384	0.981	0.391
contig057148-BurTARs.A028	contig022368-TilTARs.A047	0.150	0.383	0.391
contig045999-NyeTARs.A026	contig022334-TilTARs.A030	0.208	0.532	0.391
contig039642-TilTAR.A004	contig030445-ZebTARs.A026	0.367	0.938	0.391
contig061433-BurTARs.A013	contig030464-ZebTARs.A025	0.163	0.417	0.391
contig022356-TilTARs.A040	contig022377-TilTARs.A050	0.137	0.350	0.392
contig029633-BriTAR.A003	contig007520-TilTARs.A025	0.450	1.150	0.392
contig061091-BurTARs.A014	contig054630-BurTARs.A023	0.379	0.969	0.392
contig084887-BriTARs.A018	contig022377-TilTARs.A050	0.138	0.353	0.392
contig061433-BurTARs.A013	contig066691-ZebTARs.A015	0.162	0.414	0.392
contig022345-TilTARs.A034	contig022363-TilTARs.A044	0.155	0.395	0.392
contig061091-BurTARs.A014	contig066285-ZebTARs.A016	0.150	0.383	0.392
contig045302-BurTAR.A001	contig058002-NyeTARs.A025	0.429	1.094	0.392

contig045302-BurTAR.A001	contig022377-TilTARs.A050	0.426	1.087	0.392
contig007512-TilTARs.A024	contig022320-TilTARs.A059	0.327	0.833	0.392
contig039640-TilTAR.A003	contig061410-ZebTARs.A021	0.384	0.979	0.392
contig020038-BurTAR.A002	contig022324-TilTARs.A027	0.413	1.054	0.392
contig041024-BurTARs.A026	contig007518-TilTARs.A019	0.341	0.871	0.392
contig059766-BurTARs.A029	contig066890-ZebTARs.A014	0.281	0.716	0.392
contig029633-BriTAR.A003	contig042499-NyeTARs.A028	0.462	1.178	0.392
contig029633-BriTAR.A003	contig022353-TilTARs.A036	0.467	1.191	0.392
contig038663-NyeTAR.A005	contig022353-TilTARs.A036	0.465	1.185	0.392
contig084880-BriTARs.A016	contig022390-TilTARs.A055	0.315	0.803	0.392
contig065494-BurTARs.A030	contig022345-TilTARs.A034	0.327	0.833	0.392
contig007518-TilTARs.A019	contig062676-ZebTARs.A019	0.357	0.911	0.392
contig022345-TilTARs.A034	contig040586-ZebTAR.A001	0.406	1.034	0.392
contig058002-NyeTARs.A025	contig022355-TilTARs.A039	0.340	0.868	0.392
contig046014-NyeTARs.A024	contig007518-TilTARs.A019	0.348	0.887	0.392
contig061977-BurTARs.A012	contig059673-BurTARs.A016	0.317	0.809	0.392
contig084876-BriTARs.A015	contig060707-BurTARs.A015	0.320	0.815	0.392
contig084886-BriTARs.A017	contig056134-TilTARs.A058	0.342	0.871	0.392
contig084887-BriTARs.A018	contig056134-TilTARs.A058	0.343	0.874	0.392
contig046014-NyeTARs.A024	contig066056-ZebTARs.A017	0.305	0.778	0.392
contig045302-BurTAR.A001	contig022343-TilTARs.A033	0.429	1.093	0.392
contig057145-BurTARs.A018	contig035381-NyeTARs.A015	0.234	0.596	0.392
contig061977-BurTARs.A012	contig022365-TilTARs.A045	0.249	0.636	0.392
contig061433-BurTARs.A013	contig055697-BurTARs.A022	0.341	0.868	0.393
contig057148-BurTARs.A028	contig062039-NyeTARs.A022	0.254	0.648	0.393
contig022390-TilTARs.A056	contig066330-ZebTARs.A027	0.294	0.748	0.393
contig086344-BriTARs.A020	contig049534-BurTARs.A025	0.329	0.838	0.393
contig022377-TilTARs.A050	contig003909-ZebTAR.A003	0.381	0.970	0.393
contig022363-TilTARs.A044	contig053139-ZebTARs.A023	0.318	0.808	0.393
contig049534-BurTARs.A025	contig030464-ZebTARs.A025	0.168	0.427	0.393
contig059766-BurTARs.A029	contig022349-TilTARs.A035	0.341	0.868	0.393
contig084876-BriTARs.A015	contig086344-BriTARs.A020	0.322	0.821	0.393
contig022355-TilTARs.A039	contig030464-ZebTARs.A025	0.137	0.348	0.393
contig046011-NyeTARs.A016	contig040586-ZebTAR.A002	0.508	1.292	0.393
contig060707-BurTARs.A015	contig022382-TilTARs.A053	0.322	0.820	0.393
contig061091-BurTARs.A014	contig022334-TilTARs.A029	0.107	0.271	0.393
contig060707-BurTARs.A015	contig030464-ZebTARs.A025	0.339	0.863	0.393
contig046007-NyeTARs.A017	contig007520-TilTARs.A025	0.348	0.885	0.393
contig039639-TilTAR.A001	contig022390-TilTARs.A056	0.405	1.030	0.393
contig060707-BurTARs.A015	contig066285-ZebTARs.A016	0.328	0.835	0.393
contig065494-BurTARs.A030	contig022363-TilTARs.A044	0.321	0.816	0.393
contig038663-NyeTAR.A005	contig046014-NyeTARs.A024	0.437	1.111	0.393
contig057301-BurTARs.A017	contig039640-TilTAR.A002	0.466	1.186	0.393
contig022330-TilTARs.A028	contig022320-TilTARs.A059	0.109	0.278	0.393
contig046013-NyeTARs.A021	contig053145-ZebTARs.A028	0.342	0.869	0.393
contig061433-BurTARs.A013	contig046007-NyeTARs.A017	0.172	0.437	0.393
contig025313-BriTAR.A002	contig022377-TilTARs.A050	0.374	0.952	0.393
contig035376-NyeTARs.A014	contig039642-TilTAR.A004	0.383	0.973	0.393
contig061977-BurTARs.A012	contig039642-TilTAR.A004	0.390	0.991	0.393
contig060105-NyeTARs.A018	contig056200-NyeTARs.A029	0.324	0.824	0.393
contig039642-TilTAR.A004	contig061417-ZebTARs.A020	0.417	1.060	0.393
contig022354-TilTARs.A037	contig022368-TilTARs.A046	0.161	0.410	0.393
contig065494-BurTARs.A030	contig066890-ZebTARs.A014	0.284	0.721	0.394

contig045302-BurTAR.A001	contig046007-NyeTARs.A017	0.450	1.143	0.394
contig060105-NyeTARs.A018	contig060292-NyeTARs.A027	0.287	0.730	0.394
contig084876-BriTARs.A015	contig053139-ZebTARs.A023	0.276	0.700	0.394
contig046010-NyeTARs.A019	contig056134-TilTARs.A058	0.343	0.871	0.394
contig049540-BurTARs.A024	contig062039-NyeTARs.A022	0.253	0.643	0.394
contig066691-ZebTARs.A015	contig066285-ZebTARs.A016	0.153	0.390	0.394
contig086344-BriTARs.A020	contig062676-ZebTARs.A019	0.314	0.798	0.394
contig084876-BriTARs.A015	contig022345-TilTARs.A034	0.240	0.609	0.394
contig022337-TilTARs.A031	contig059768-ZebTARs.A022	0.376	0.954	0.394
contig022379-TilTARs.A052	contig040586-ZebTAR.A001	0.417	1.059	0.394
contig022365-TilTARs.A045	contig022377-TilTARs.A050	0.133	0.339	0.394
contig046010-NyeTARs.A019	contig062039-NyeTARs.A022	0.259	0.657	0.394
contig059673-BurTARs.A016	contig053139-ZebTARs.A023	0.259	0.658	0.394
contig082565-BriTARs.A022	contig039640-TilTAR.A003	0.373	0.946	0.394
contig056021-BurTARs.A020	contig039642-TilTAR.A004	0.417	1.060	0.394
contig086344-BriTARs.A020	contig035381-NyeTARs.A015	0.316	0.802	0.394
contig035375-NyeTARs.A013	contig039640-TilTAR.A003	0.376	0.953	0.394
contig041024-BurTARs.A026	contig022362-TilTARs.A042	0.126	0.320	0.394
contig039642-TilTAR.A004	contig066691-ZebTARs.A015	0.443	1.124	0.394
contig022345-TilTARs.A034	contig066330-ZebTARs.A027	0.331	0.840	0.394
contig060707-BurTARs.A015	contig035377-NyeTARs.A023	0.327	0.829	0.394
contig045302-BurTAR.A001	contig022379-TilTARs.A052	0.410	1.040	0.394
contig086344-BriTARs.A020	contig035376-NyeTARs.A014	0.324	0.821	0.394
contig084880-BriTARs.A016	contig038663-NyeTAR.A005	0.452	1.148	0.394
contig045302-BurTAR.A001	contig056020-BurTARs.A021	0.482	1.222	0.394
contig059766-BurTARs.A029	contig022334-TilTARs.A029	0.330	0.838	0.394
contig065494-BurTARs.A030	contig022330-TilTARs.A028	0.336	0.853	0.394
contig061977-BurTARs.A012	contig066330-ZebTARs.A027	0.270	0.684	0.394
contig038663-NyeTAR.A005	contig022355-TilTARs.A039	0.453	1.148	0.394
contig057301-BurTARs.A017	contig040586-ZebTAR.A002	0.471	1.194	0.394
contig038663-NyeTAR.A005	contig022320-TilTARs.A059	0.441	1.119	0.394
contig046007-NyeTARs.A017	contig030471-ZebTARs.A024	0.108	0.275	0.394
contig022324-TilTARs.A027	contig066691-ZebTARs.A015	0.162	0.410	0.394
contig030445-ZebTARs.A026	contig053145-ZebTARs.A028	0.248	0.630	0.394
contig040586-ZebTAR.A002	contig061417-ZebTARs.A020	0.427	1.082	0.394
contig060707-BurTARs.A015	contig022368-TilTARs.A047	0.330	0.836	0.394
contig045088-TilTARs.A057	contig053145-ZebTARs.A028	0.280	0.709	0.394
contig065494-BurTARs.A030	contig061410-ZebTARs.A021	0.270	0.683	0.395
contig055697-BurTARs.A022	contig022334-TilTARs.A030	0.344	0.871	0.395
contig061433-BurTARs.A013	contig022345-TilTARs.A034	0.170	0.430	0.395
contig056020-BurTARs.A021	contig022330-TilTARs.A028	0.120	0.303	0.395
contig022354-TilTARs.A038	contig022383-TilTARs.A054	0.241	0.611	0.395
contig046007-NyeTARs.A017	contig030464-ZebTARs.A025	0.139	0.352	0.395
contig045999-NyeTARs.A026	contig039640-TilTAR.A003	0.440	1.116	0.395
contig086344-BriTARs.A020	contig035377-NyeTARs.A023	0.330	0.835	0.395
contig022345-TilTARs.A034	contig062676-ZebTARs.A019	0.236	0.599	0.395
contig054630-BurTARs.A023	contig046007-NyeTARs.A017	0.361	0.915	0.395
contig035376-NyeTARs.A014	contig062039-NyeTARs.A022	0.215	0.545	0.395
contig061977-BurTARs.A012	contig040586-ZebTAR.A001	0.394	0.998	0.395
contig084868-BriTARs.A014	contig022383-TilTARs.A054	0.036	0.092	0.395
contig061417-ZebTARs.A020	contig053139-ZebTARs.A023	0.323	0.817	0.395
contig022377-TilTARs.A050	contig040586-ZebTAR.A001	0.432	1.095	0.395
contig022341-TilTARs.A032	contig022368-TilTARs.A047	0.148	0.375	0.395

contig058002-NyeTARs.A025	contig022362-TilTARs.A042	0.337	0.854	0.395
contig007512-TilTARs.A024	contig022362-TilTARs.A042	0.317	0.804	0.395
contig086337-BriTARs.A019	contig061410-ZebTARs.A021	0.264	0.669	0.395
contig059766-BurTARs.A029	contig045088-TilTARs.A057	0.276	0.700	0.395
contig084886-BriTARs.A017	contig049534-BurTARs.A025	0.164	0.415	0.395
contig007512-TilTARs.A024	contig022382-TilTARs.A053	0.308	0.779	0.395
contig039642-TilTAR.A004	contig007524-TilTARs.A026	0.433	1.097	0.395
contig086337-BriTARs.A019	contig022377-TilTARs.A050	0.338	0.855	0.395
contig086337-BriTARs.A019	contig061417-ZebTARs.A020	0.318	0.806	0.395
contig035377-NyeTARs.A023	contig007518-TilTARs.A019	0.340	0.860	0.395
contig056021-BurTARs.A020	contig040586-ZebTAR.A002	0.427	1.082	0.395
contig029633-BriTAR.A003	contig022343-TilTARs.A033	0.441	1.117	0.395
contig066691-ZebTARs.A015	contig059768-ZebTARs.A022	0.377	0.955	0.395
contig084886-BriTARs.A017	contig059768-ZebTARs.A022	0.376	0.953	0.395
contig065494-BurTARs.A030	contig045088-TilTARs.A057	0.280	0.710	0.395
contig060707-BurTARs.A015	contig046010-NyeTARs.A019	0.341	0.863	0.395
contig038663-NyeTAR.A005	contig046010-NyeTARs.A019	0.468	1.184	0.395
contig020038-BurTAR.A002	contig022377-TilTARs.A050	0.385	0.974	0.395
contig046011-NyeTARs.A016	contig053139-ZebTARs.A023	0.386	0.976	0.395
contig060707-BurTARs.A015	contig022320-TilTARs.A059	0.317	0.802	0.395
contig061977-BurTARs.A012	contig060105-NyeTARs.A018	0.309	0.781	0.395
contig061433-BurTARs.A013	contig040586-ZebTAR.A002	0.453	1.146	0.395
contig039642-TilTAR.A004	contig022324-TilTARs.A027	0.457	1.155	0.395
contig007520-TilTARs.A025	contig061410-ZebTARs.A021	0.299	0.757	0.395
contig039642-TilTAR.A004	contig030440-ZebTARs.A029	0.378	0.955	0.395
contig086337-BriTARs.A019	contig022334-TilTARs.A030	0.342	0.866	0.395
contig045302-BurTAR.A001	contig039640-TilTAR.A003	0.048	0.122	0.395
contig059673-BurTARs.A016	contig022383-TilTARs.A054	0.325	0.822	0.395
contig054630-BurTARs.A023	contig022382-TilTARs.A053	0.352	0.890	0.395
contig061433-BurTARs.A013	contig049534-BurTARs.A025	0.169	0.426	0.395
contig084886-BriTARs.A017	contig022345-TilTARs.A034	0.135	0.342	0.395
contig022363-TilTARs.A044	contig066330-ZebTARs.A027	0.327	0.826	0.396
contig061433-BurTARs.A013	contig066056-ZebTARs.A017	0.334	0.844	0.396
contig022354-TilTARs.A038	contig022368-TilTARs.A047	0.136	0.344	0.396
contig022341-TilTARs.A032	contig022379-TilTARs.A052	0.233	0.590	0.396
contig046010-NyeTARs.A019	contig007518-TilTARs.A019	0.363	0.919	0.396
contig060105-NyeTARs.A018	contig022349-TilTARs.A035	0.356	0.901	0.396
contig022354-TilTARs.A037	contig022355-TilTARs.A039	0.167	0.423	0.396
contig061091-BurTARs.A014	contig049534-BurTARs.A025	0.143	0.363	0.396
contig060105-NyeTARs.A018	contig058002-NyeTARs.A025	0.299	0.757	0.396
contig022354-TilTARs.A037	contig022363-TilTARs.A043	0.151	0.381	0.396
contig053139-ZebTARs.A023	contig030464-ZebTARs.A025	0.346	0.874	0.396
contig062039-NyeTARs.A022	contig030445-ZebTARs.A026	0.202	0.511	0.396
contig046011-NyeTARs.A016	contig007512-TilTARs.A024	0.383	0.967	0.396
contig062676-ZebTARs.A019	contig061410-ZebTARs.A021	0.136	0.343	0.396
contig086337-BriTARs.A019	contig061433-BurTARs.A013	0.335	0.847	0.396
contig039640-TilTAR.A003	contig022383-TilTARs.A054	0.385	0.972	0.396
contig022334-TilTARs.A030	contig030440-ZebTARs.A029	0.237	0.598	0.396
contig056021-BurTARs.A020	contig041024-BurTARs.A026	0.106	0.267	0.396
contig022354-TilTARs.A038	contig059768-ZebTARs.A022	0.362	0.915	0.396
contig049540-BurTARs.A024	contig056134-TilTARs.A058	0.359	0.908	0.396
contig038663-NyeTAR.A005	contig046007-NyeTARs.A017	0.464	1.172	0.396
contig061433-BurTARs.A013	contig053139-ZebTARs.A023	0.330	0.834	0.396

contig057301-BurTARs.A017	contig057148-BurTARs.A028	0.382	0.963	0.396
contig022379-TilTARs.A052	contig062676-ZebTARs.A019	0.139	0.350	0.396
contig038663-NyeTAR.A005	contig022341-TilTARs.A032	0.437	1.103	0.396
contig049534-BurTARs.A025	contig059768-ZebTARs.A022	0.384	0.969	0.396
contig022324-TilTARs.A027	contig022334-TilTARs.A029	0.152	0.383	0.396
contig022334-TilTARs.A029	contig066056-ZebTARs.A017	0.326	0.822	0.396
contig060105-NyeTARs.A018	contig022383-TilTARs.A054	0.303	0.764	0.396
contig022379-TilTARs.A052	contig053139-ZebTARs.A023	0.270	0.682	0.396
contig022349-TilTARs.A035	contig030464-ZebTARs.A025	0.153	0.385	0.396
contig049534-BurTARs.A025	contig035381-NyeTARs.A015	0.265	0.667	0.396
contig061091-BurTARs.A014	contig059768-ZebTARs.A022	0.373	0.940	0.397
contig057148-BurTARs.A028	contig066330-ZebTARs.A027	0.347	0.876	0.397
contig086337-BriTARs.A019	contig007524-TilTARs.A026	0.231	0.582	0.397
contig040586-ZebTAR.A002	contig062676-ZebTARs.A019	0.399	1.006	0.397
contig056021-BurTARs.A020	contig007512-TilTARs.A024	0.332	0.837	0.397
contig056134-TilTARs.A058	contig040586-ZebTAR.A002	0.380	0.959	0.397
contig022345-TilTARs.A034	contig030445-ZebTARs.A026	0.238	0.600	0.397
contig022320-TilTARs.A059	contig053139-ZebTARs.A023	0.329	0.829	0.397
contig022365-TilTARs.A045	contig066285-ZebTARs.A016	0.153	0.385	0.397
contig059766-BurTARs.A029	contig030445-ZebTARs.A026	0.250	0.630	0.397
contig022341-TilTARs.A032	contig022354-TilTARs.A037	0.169	0.426	0.397
contig022330-TilTARs.A028	contig066056-ZebTARs.A017	0.323	0.813	0.397
contig084886-BriTARs.A017	contig060707-BurTARs.A015	0.327	0.823	0.397
contig022375-TilTARs.A048	contig066056-ZebTARs.A017	0.323	0.815	0.397
contig055697-BurTARs.A022	contig022377-TilTARs.A050	0.346	0.870	0.397
contig057148-BurTARs.A028	contig022368-TilTARs.A046	0.149	0.375	0.397
contig084887-BriTARs.A018	contig061433-BurTARs.A013	0.163	0.409	0.397
contig022362-TilTARs.A042	contig059768-ZebTARs.A022	0.344	0.867	0.397
contig084876-BriTARs.A015	contig022341-TilTARs.A032	0.240	0.603	0.397
contig022324-TilTARs.A027	contig022365-TilTARs.A045	0.162	0.408	0.397
contig022377-TilTARs.A050	contig053145-ZebTARs.A028	0.335	0.844	0.397
contig065494-BurTARs.A030	contig022320-TilTARs.A059	0.325	0.819	0.397
contig022353-TilTARs.A036	contig056134-TilTARs.A058	0.335	0.843	0.397
contig061433-BurTARs.A013	contig022349-TilTARs.A035	0.174	0.437	0.397
contig065494-BurTARs.A030	contig022377-TilTARs.A050	0.338	0.852	0.397
contig054630-BurTARs.A023	contig061410-ZebTARs.A021	0.318	0.799	0.397
contig029633-BriTAR.A003	contig022330-TilTARs.A028	0.434	1.091	0.397
contig054630-BurTARs.A023	contig022324-TilTARs.A027	0.416	1.047	0.397
contig056023-BurTARs.A019	contig022334-TilTARs.A030	0.208	0.524	0.397
contig056134-TilTARs.A058	contig030440-ZebTARs.A029	0.291	0.732	0.397
contig022354-TilTARs.A038	contig022377-TilTARs.A050	0.133	0.336	0.397
contig084887-BriTARs.A018	contig022353-TilTARs.A036	0.031	0.078	0.397
contig065494-BurTARs.A030	contig046013-NyeTARs.A021	0.343	0.864	0.398
contig035381-NyeTARs.A015	contig060105-NyeTARs.A018	0.313	0.786	0.398
contig054630-BurTARs.A023	contig022354-TilTARs.A038	0.375	0.942	0.398
contig046007-NyeTARs.A017	contig056134-TilTARs.A058	0.347	0.871	0.398
contig022363-TilTARs.A044	contig040586-ZebTAR.A001	0.416	1.045	0.398
contig084886-BriTARs.A017	contig053139-ZebTARs.A023	0.333	0.837	0.398
contig022334-TilTARs.A029	contig022320-TilTARs.A059	0.111	0.279	0.398
contig061977-BurTARs.A012	contig041024-BurTARs.A026	0.236	0.592	0.398
contig049534-BurTARs.A025	contig066691-ZebTARs.A015	0.147	0.369	0.398
contig029633-BriTAR.A003	contig046013-NyeTARs.A021	0.448	1.127	0.398
contig057148-BurTARs.A028	contig053145-ZebTARs.A028	0.348	0.874	0.398

contig035377-NyeTARs.A023	contig058002-NyeTARs.A025	0.321	0.807	0.398
contig045302-BurTAR.A001	contig062676-ZebTARs.A019	0.411	1.033	0.398
contig035376-NyeTARs.A014	contig060105-NyeTARs.A018	0.320	0.805	0.398
contig054630-BurTARs.A023	contig007518-TilTARs.A019	0.381	0.957	0.398
contig056134-TilTARs.A058	contig061410-ZebTARs.A021	0.288	0.725	0.398
contig040586-ZebTAR.A001	contig062676-ZebTARs.A019	0.406	1.020	0.398
contig022353-TilTARs.A036	contig022320-TilTARs.A059	0.103	0.258	0.398
contig020038-BurTAR.A002	contig023443-TilTAR.A005	0.020	0.050	0.398
contig040586-ZebTAR.A001	contig066285-ZebTARs.A016	0.449	1.128	0.398
contig022377-TilTARs.A050	contig030464-ZebTARs.A025	0.153	0.385	0.398
contig022368-TilTARs.A047	contig040586-ZebTAR.A001	0.432	1.085	0.398
contig029633-BriTAR.A003	contig022349-TilTARs.A035	0.468	1.176	0.398
contig084876-BriTARs.A015	contig057148-BurTARs.A028	0.258	0.647	0.398
contig056023-BurTARs.A019	contig040586-ZebTAR.A001	0.441	1.107	0.398
contig022390-TilTARs.A055	contig066056-ZebTARs.A017	0.400	1.003	0.398
contig066890-ZebTARs.A014	contig066330-ZebTARs.A027	0.286	0.717	0.398
contig029633-BriTAR.A003	contig022320-TilTARs.A059	0.439	1.102	0.398
contig055697-BurTARs.A022	contig035375-NyeTARs.A013	0.280	0.702	0.399
contig060707-BurTARs.A015	contig059673-BurTARs.A016	0.305	0.765	0.399
contig022334-TilTARs.A029	contig053139-ZebTARs.A023	0.320	0.802	0.399
contig058002-NyeTARs.A025	contig022368-TilTARs.A046	0.343	0.860	0.399
contig061091-BurTARs.A014	contig022377-TilTARs.A050	0.140	0.352	0.399
contig082565-BriTARs.A022	contig039642-TilTAR.A004	0.373	0.935	0.399
contig039642-TilTAR.A004	contig045088-TilTARs.A057	0.457	1.146	0.399
contig045302-BurTAR.A001	contig061410-ZebTARs.A021	0.395	0.990	0.399
contig035376-NyeTARs.A014	contig056134-TilTARs.A058	0.288	0.722	0.399
contig057305-BurTARs.A031	contig062039-NyeTARs.A022	0.360	0.902	0.399
contig007520-TilTARs.A025	contig022390-TilTARs.A055	0.287	0.720	0.399
contig084876-BriTARs.A015	contig022383-TilTARs.A054	0.148	0.371	0.399
contig055697-BurTARs.A022	contig046011-NyeTARs.A016	0.391	0.981	0.399
contig060707-BurTARs.A015	contig022324-TilTARs.A027	0.335	0.841	0.399
contig057305-BurTARs.A031	contig058002-NyeTARs.A025	0.253	0.635	0.399
contig060707-BurTARs.A015	contig049534-BurTARs.A025	0.330	0.826	0.399
contig049540-BurTARs.A024	contig060105-NyeTARs.A018	0.324	0.813	0.399
contig046010-NyeTARs.A019	contig022356-TilTARs.A040	0.129	0.324	0.399
contig029633-BriTAR.A003	contig022390-TilTARs.A055	0.462	1.158	0.399
contig060105-NyeTARs.A018	contig066285-ZebTARs.A016	0.330	0.827	0.399
contig022330-TilTARs.A028	contig066330-ZebTARs.A027	0.338	0.848	0.399
contig060105-NyeTARs.A018	contig007524-TilTARs.A026	0.293	0.733	0.399
contig084868-BriTARs.A014	contig022363-TilTARs.A043	0.307	0.769	0.399
contig045302-BurTAR.A001	contig066285-ZebTARs.A016	0.445	1.116	0.399
contig022383-TilTARs.A054	contig062677-ZebTARs.A018	0.163	0.409	0.399
contig038663-NyeTAR.A005	contig022334-TilTARs.A029	0.441	1.106	0.399
contig040586-ZebTAR.A002	contig061410-ZebTARs.A021	0.407	1.020	0.399
contig042499-NyeTARs.A028	contig039640-TilTAR.A003	0.462	1.157	0.399
contig029633-BriTAR.A003	contig061091-BurTARs.A014	0.449	1.125	0.399
contig046007-NyeTARs.A017	contig046010-NyeTARs.A019	0.141	0.353	0.399
contig061433-BurTARs.A013	contig022362-TilTARs.A042	0.170	0.426	0.399
contig038663-NyeTAR.A005	contig022345-TilTARs.A034	0.411	1.030	0.399
contig057148-BurTARs.A028	contig065494-BurTARs.A030	0.350	0.876	0.399
contig039642-TilTAR.A004	contig062676-ZebTARs.A019	0.391	0.978	0.399
contig022356-TilTARs.A040	contig061410-ZebTARs.A021	0.244	0.612	0.399
contig086344-BriTARs.A020	contig059673-BurTARs.A016	0.311	0.779	0.399

contig022320-TilTARs.A059	contig066330-ZebTARs.A027	0.325	0.814	0.399
contig084876-BriTARs.A015	contig053145-ZebTARs.A028	0.276	0.692	0.399
contig022354-TilTARs.A037	contig022362-TilTARs.A042	0.170	0.424	0.399
contig022345-TilTARs.A034	contig040586-ZebTAR.A002	0.422	1.056	0.399
contig049534-BurTARs.A025	contig022324-TilTARs.A027	0.164	0.411	0.399
contig022334-TilTARs.A030	contig066056-ZebTARs.A017	0.337	0.844	0.399
contig022334-TilTARs.A030	contig053145-ZebTARs.A028	0.303	0.759	0.400
contig007524-TilTARs.A026	contig040586-ZebTAR.A002	0.439	1.099	0.400
contig055697-BurTARs.A022	contig049540-BurTARs.A024	0.332	0.831	0.400
contig046010-NyeTARs.A019	contig022362-TilTARs.A042	0.155	0.388	0.400
contig086337-BriTARs.A019	contig022379-TilTARs.A052	0.273	0.683	0.400
contig041024-BurTARs.A026	contig022354-TilTARs.A037	0.149	0.373	0.400
contig007518-TilTARs.A019	contig022383-TilTARs.A054	0.357	0.893	0.400
contig086344-BriTARs.A020	contig022320-TilTARs.A059	0.325	0.813	0.400
contig022382-TilTARs.A053	contig059768-ZebTARs.A022	0.346	0.865	0.400
contig060105-NyeTARs.A018	contig062676-ZebTARs.A019	0.309	0.773	0.400
contig049534-BurTARs.A025	contig022334-TilTARs.A029	0.145	0.363	0.400
contig022355-TilTARs.A039	contig030471-ZebTARs.A024	0.105	0.264	0.400
contig035375-NyeTARs.A013	contig039642-TilTAR.A004	0.376	0.940	0.400
contig040586-ZebTAR.A002	contig030445-ZebTARs.A026	0.378	0.946	0.400
contig046010-NyeTARs.A019	contig022368-TilTARs.A047	0.146	0.365	0.400
contig022355-TilTARs.A039	contig022365-TilTARs.A045	0.129	0.324	0.400
contig084887-BriTARs.A018	contig022345-TilTARs.A034	0.133	0.332	0.400
contig060105-NyeTARs.A018	contig030464-ZebTARs.A025	0.344	0.860	0.400
contig058002-NyeTARs.A025	contig061410-ZebTARs.A021	0.301	0.753	0.400
contig054630-BurTARs.A023	contig057305-BurTARs.A031	0.387	0.968	0.400
contig054630-BurTARs.A023	contig060292-NyeTARs.A027	0.387	0.968	0.400
contig045088-TilTARs.A057	contig066330-ZebTARs.A027	0.286	0.714	0.400
contig046010-NyeTARs.A019	contig030471-ZebTARs.A024	0.150	0.375	0.400
contig007520-TilTARs.A025	contig040586-ZebTAR.A002	0.432	1.080	0.400
contig039640-TilTAR.A002	contig022390-TilTARs.A055	0.432	1.080	0.400
contig039640-TilTAR.A002	contig022390-TilTARs.A056	0.402	1.004	0.400
contig035381-NyeTARs.A015	contig039640-TilTAR.A003	0.376	0.940	0.400
contig054630-BurTARs.A023	contig022365-TilTARs.A045	0.356	0.889	0.400
contig065494-BurTARs.A030	contig022341-TilTARs.A032	0.322	0.805	0.400
contig057145-BurTARs.A018	contig022362-TilTARs.A042	0.152	0.379	0.401
contig038663-NyeTAR.A005	contig022343-TilTARs.A033	0.439	1.096	0.401
contig045999-NyeTARs.A026	contig039640-TilTAR.A002	0.465	1.161	0.401
contig086337-BriTARs.A019	contig022353-TilTARs.A036	0.334	0.833	0.401
contig022341-TilTARs.A032	contig022362-TilTARs.A042	0.155	0.387	0.401
contig035377-NyeTARs.A023	contig039640-TilTAR.A003	0.400	0.998	0.401
contig007524-TilTARs.A026	contig062676-ZebTARs.A019	0.325	0.810	0.401
contig022355-TilTARs.A039	contig022377-TilTARs.A050	0.138	0.346	0.401
contig022345-TilTARs.A034	contig030471-ZebTARs.A024	0.126	0.315	0.401
contig022324-TilTARs.A027	contig040586-ZebTAR.A001	0.453	1.131	0.401
contig056023-BurTARs.A019	contig039640-TilTAR.A003	0.440	1.099	0.401
contig055697-BurTARs.A022	contig057305-BurTARs.A031	0.282	0.704	0.401
contig054630-BurTARs.A023	contig022341-TilTARs.A032	0.355	0.886	0.401
contig061410-ZebTARs.A021	contig066330-ZebTARs.A027	0.272	0.678	0.401
contig007520-TilTARs.A025	contig040586-ZebTAR.A001	0.433	1.079	0.401
contig060105-NyeTARs.A018	contig022320-TilTARs.A059	0.318	0.793	0.401
contig061091-BurTARs.A014	contig007518-TilTARs.A019	0.371	0.926	0.401
contig053145-ZebTARs.A028	contig030440-ZebTARs.A029	0.279	0.696	0.401

contig038663-NyeTAR.A005	contig022337-TilTARs.A031	0.452	1.126	0.401
contig022375-TilTARs.A048	contig056134-TilTARs.A058	0.346	0.862	0.401
contig045302-BurTAR.A001	contig046013-NyeTARs.A021	0.436	1.087	0.401
contig058002-NyeTARs.A025	contig060292-NyeTARs.A027	0.251	0.626	0.401
contig061977-BurTARs.A012	contig038663-NyeTAR.A005	0.398	0.991	0.401
contig022382-TilTARs.A053	contig056134-TilTARs.A058	0.353	0.879	0.401
contig061433-BurTARs.A013	contig061417-ZebTARs.A020	0.165	0.410	0.401
contig062039-NyeTARs.A022	contig060292-NyeTARs.A027	0.362	0.902	0.401
contig084880-BriTARs.A016	contig054630-BurTARs.A023	0.403	1.005	0.401
contig040586-ZebTAR.A001	contig030471-ZebTARs.A024	0.457	1.138	0.401
contig045302-BurTAR.A001	contig022363-TilTARs.A044	0.415	1.034	0.401
contig046010-NyeTARs.A019	contig022365-TilTARs.A045	0.148	0.369	0.401
contig022349-TilTARs.A035	contig056134-TilTARs.A058	0.377	0.939	0.401
contig046014-NyeTARs.A024	contig022368-TilTARs.A047	0.120	0.299	0.401
contig057148-BurTARs.A028	contig022355-TilTARs.A039	0.138	0.344	0.401
contig084887-BriTARs.A018	contig056021-BurTARs.A020	0.117	0.290	0.401
contig086344-BriTARs.A020	contig022368-TilTARs.A047	0.341	0.848	0.401
contig035376-NyeTARs.A014	contig040586-ZebTAR.A002	0.394	0.982	0.401
contig061977-BurTARs.A012	contig022375-TilTARs.A048	0.211	0.526	0.401
contig039642-TilTAR.A004	contig022357-TilTARs.A041	0.439	1.094	0.401
contig022354-TilTARs.A038	contig022365-TilTARs.A045	0.135	0.336	0.401
contig035381-NyeTARs.A015	contig066285-ZebTARs.A016	0.265	0.660	0.401
contig054630-BurTARs.A023	contig045999-NyeTARs.A026	0.401	1.000	0.402
contig022390-TilTARs.A055	contig053139-ZebTARs.A023	0.300	0.747	0.402
contig046011-NyeTARs.A016	contig023443-TilTAR.A005	0.429	1.069	0.402
contig059766-BurTARs.A029	contig022377-TilTARs.A050	0.340	0.846	0.402
contig057301-BurTARs.A017	contig049534-BurTARs.A025	0.376	0.936	0.402
contig035381-NyeTARs.A015	contig007520-TilTARs.A025	0.304	0.757	0.402
contig022357-TilTARs.A041	contig040586-ZebTAR.A001	0.440	1.095	0.402
contig086337-BriTARs.A019	contig046011-NyeTARs.A016	0.387	0.963	0.402
contig056021-BurTARs.A020	contig056134-TilTARs.A058	0.357	0.889	0.402
contig084886-BriTARs.A017	contig007518-TilTARs.A019	0.366	0.910	0.402
contig046007-NyeTARs.A017	contig061417-ZebTARs.A020	0.123	0.305	0.402
contig022365-TilTARs.A045	contig061410-ZebTARs.A021	0.252	0.626	0.402
contig022377-TilTARs.A050	contig066330-ZebTARs.A027	0.341	0.847	0.402
contig055697-BurTARs.A022	contig022362-TilTARs.A042	0.319	0.795	0.402
contig057148-BurTARs.A028	contig059766-BurTARs.A029	0.352	0.875	0.402
contig084876-BriTARs.A015	contig040586-ZebTAR.A002	0.421	1.047	0.402
contig059673-BurTARs.A016	contig062676-ZebTARs.A019	0.332	0.826	0.402
contig007524-TilTARs.A026	contig040586-ZebTAR.A001	0.438	1.089	0.402
contig029633-BriTAR.A003	contig061410-ZebTARs.A021	0.414	1.028	0.402
contig084886-BriTARs.A017	contig061977-BurTARs.A012	0.246	0.611	0.402
contig060105-NyeTARs.A018	contig046010-NyeTARs.A019	0.346	0.860	0.402
contig039642-TilTAR.A004	contig022345-TilTARs.A034	0.413	1.027	0.402
contig062039-NyeTARs.A022	contig062677-ZebTARs.A018	0.256	0.637	0.402
contig054630-BurTARs.A023	contig049534-BurTARs.A025	0.391	0.971	0.402
contig061091-BurTARs.A014	contig022355-TilTARs.A039	0.083	0.205	0.402
contig022363-TilTARs.A043	contig061417-ZebTARs.A020	0.151	0.374	0.403
contig022330-TilTARs.A028	contig022368-TilTARs.A046	0.133	0.330	0.403
contig061433-BurTARs.A013	contig061091-BurTARs.A014	0.159	0.395	0.403
contig084886-BriTARs.A017	contig022356-TilTARs.A040	0.130	0.324	0.403
contig039639-TilTAR.A001	contig066890-ZebTARs.A014	0.414	1.027	0.403
contig007520-TilTARs.A025	contig022354-TilTARs.A038	0.340	0.844	0.403

contig022365-TilTARs.A045	contig056134-TilTARs.A058	0.349	0.867	0.403
contig060707-BurTARs.A015	contig041024-BurTARs.A026	0.314	0.779	0.403
contig029633-BriTAR.A003	contig022341-TilTARs.A032	0.434	1.077	0.403
contig056020-BurTARs.A021	contig022368-TilTARs.A046	0.130	0.322	0.403
contig042499-NyeTARs.A028	contig039639-TilTAR.A001	0.466	1.156	0.403
contig039642-TilTAR.A004	contig022383-TilTARs.A054	0.389	0.965	0.403
contig022363-TilTARs.A044	contig056134-TilTARs.A058	0.358	0.888	0.403
contig061977-BurTARs.A012	contig007512-TilTARs.A024	0.276	0.685	0.403
contig022324-TilTARs.A027	contig059768-ZebTARs.A022	0.409	1.015	0.403
contig084868-BriTARs.A014	contig061410-ZebTARs.A021	0.155	0.386	0.403
contig022334-TilTARs.A030	contig066285-ZebTARs.A016	0.220	0.545	0.403
contig059766-BurTARs.A029	contig030440-ZebTARs.A029	0.281	0.697	0.403
contig046010-NyeTARs.A019	contig022378-TilTARs.A051	0.246	0.609	0.403
contig055697-BurTARs.A022	contig007524-TilTARs.A026	0.240	0.596	0.403
contig056021-BurTARs.A020	contig046007-NyeTARs.A017	0.130	0.323	0.403
contig035381-NyeTARs.A015	contig053145-ZebTARs.A028	0.254	0.630	0.403
contig084886-BriTARs.A017	contig066285-ZebTARs.A016	0.167	0.413	0.403
contig056020-BurTARs.A021	contig030464-ZebTARs.A025	0.136	0.337	0.404
contig035375-NyeTARs.A013	contig040586-ZebTAR.A002	0.386	0.956	0.404
contig054630-BurTARs.A023	contig022334-TilTARs.A029	0.353	0.875	0.404
contig055697-BurTARs.A022	contig060292-NyeTARs.A027	0.280	0.694	0.404
contig062039-NyeTARs.A022	contig035377-NyeTARs.A023	0.244	0.604	0.404
contig049534-BurTARs.A025	contig042499-NyeTARs.A028	0.378	0.935	0.404
contig040586-ZebTAR.A001	contig061410-ZebTARs.A021	0.400	0.991	0.404
contig022341-TilTARs.A032	contig022383-TilTARs.A054	0.250	0.619	0.404
contig054630-BurTARs.A023	contig022362-TilTARs.A042	0.351	0.869	0.404
contig056134-TilTARs.A058	contig062677-ZebTARs.A018	0.362	0.896	0.404
contig022378-TilTARs.A051	contig022383-TilTARs.A054	0.229	0.568	0.404
contig029633-BriTAR.A003	contig022377-TilTARs.A050	0.432	1.069	0.404
contig034854-BurTARs.A027	contig039642-TilTAR.A004	0.372	0.920	0.404
contig061091-BurTARs.A014	contig046007-NyeTARs.A017	0.090	0.224	0.404
contig054630-BurTARs.A023	contig062677-ZebTARs.A018	0.374	0.925	0.404
contig046014-NyeTARs.A024	contig022368-TilTARs.A046	0.111	0.275	0.404
contig038663-NyeTAR.A005	contig046013-NyeTARs.A021	0.450	1.113	0.404
contig056020-BurTARs.A021	contig022353-TilTARs.A036	0.085	0.211	0.404
contig084886-BriTARs.A017	contig060105-NyeTARs.A018	0.332	0.821	0.404
contig055697-BurTARs.A022	contig066691-ZebTARs.A015	0.358	0.885	0.404
contig007512-TilTARs.A024	contig022324-TilTARs.A027	0.336	0.832	0.404
contig057305-BurTARs.A031	contig022362-TilTARs.A042	0.386	0.955	0.404
contig060292-NyeTARs.A027	contig022362-TilTARs.A042	0.386	0.955	0.404
contig039640-TilTAR.A002	contig066890-ZebTARs.A014	0.402	0.994	0.404
contig056020-BurTARs.A021	contig066056-ZebTARs.A017	0.333	0.823	0.404
contig035381-NyeTARs.A015	contig022349-TilTARs.A035	0.251	0.622	0.404
contig055697-BurTARs.A022	contig022343-TilTARs.A033	0.316	0.782	0.404
contig022334-TilTARs.A030	contig053139-ZebTARs.A023	0.348	0.860	0.404
contig060707-BurTARs.A015	contig022354-TilTARs.A038	0.331	0.819	0.404
contig022341-TilTARs.A032	contig030464-ZebTARs.A025	0.135	0.333	0.404
contig029633-BriTAR.A003	contig046014-NyeTARs.A024	0.435	1.076	0.404
contig049534-BurTARs.A025	contig040586-ZebTAR.A001	0.454	1.124	0.404
contig022353-TilTARs.A036	contig022354-TilTARs.A038	0.127	0.314	0.404
contig086344-BriTARs.A020	contig054630-BurTARs.A023	0.352	0.870	0.405
contig035377-NyeTARs.A023	contig007520-TilTARs.A025	0.322	0.796	0.405
contig022343-TilTARs.A033	contig053145-ZebTARs.A028	0.333	0.822	0.405

contig084876-BriTARs.A015	contig059766-BurTARs.A029	0.280	0.693	0.405
contig007520-TilTARs.A025	contig022341-TilTARs.A032	0.347	0.857	0.405
contig049534-BurTARs.A025	contig060105-NyeTARs.A018	0.331	0.818	0.405
contig059766-BurTARs.A029	contig022334-TilTARs.A030	0.308	0.761	0.405
contig038663-NyeTAR.A005	contig022363-TilTARs.A044	0.421	1.041	0.405
contig084880-BriTARs.A016	contig039639-TilTAR.A001	0.450	1.112	0.405
contig022354-TilTARs.A037	contig022320-TilTARs.A059	0.155	0.384	0.405
contig084868-BriTARs.A014	contig039639-TilTAR.A001	0.392	0.968	0.405
contig057148-BurTARs.A028	contig022378-TilTARs.A051	0.243	0.601	0.405
contig061417-ZebTARs.A020	contig030464-ZebTARs.A025	0.136	0.337	0.405
contig029633-BriTAR.A003	contig084876-BriTARs.A015	0.428	1.056	0.405
contig035381-NyeTARs.A015	contig039642-TilTAR.A004	0.376	0.929	0.405
contig022334-TilTARs.A030	contig022341-TilTARs.A032	0.166	0.410	0.405
contig022341-TilTARs.A032	contig022355-TilTARs.A039	0.122	0.301	0.405
contig022362-TilTARs.A042	contig053145-ZebTARs.A028	0.322	0.795	0.405
contig084880-BriTARs.A016	contig046010-NyeTARs.A019	0.185	0.457	0.405
contig084876-BriTARs.A015	contig058002-NyeTARs.A025	0.310	0.764	0.405
contig084887-BriTARs.A018	contig046010-NyeTARs.A019	0.148	0.366	0.405
contig084887-BriTARs.A018	contig022330-TilTARs.A028	0.114	0.281	0.405
contig086337-BriTARs.A019	contig030440-ZebTARs.A029	0.283	0.698	0.405
contig084868-BriTARs.A014	contig022341-TilTARs.A032	0.263	0.649	0.405
contig022341-TilTARs.A032	contig066330-ZebTARs.A027	0.324	0.800	0.405
contig029633-BriTAR.A003	contig007524-TilTARs.A026	0.458	1.130	0.405
contig049534-BurTARs.A025	contig022368-TilTARs.A046	0.161	0.396	0.405
contig086351-BriTARs.A021	contig054630-BurTARs.A023	0.392	0.967	0.405
contig084876-BriTARs.A015	contig056134-TilTARs.A058	0.301	0.744	0.405
contig054630-BurTARs.A023	contig056200-NyeTARs.A029	0.376	0.926	0.405
contig084880-BriTARs.A016	contig059768-ZebTARs.A022	0.396	0.977	0.405
contig029633-BriTAR.A003	contig049534-BurTARs.A025	0.451	1.113	0.405
contig007520-TilTARs.A025	contig022334-TilTARs.A030	0.334	0.823	0.406
contig060105-NyeTARs.A018	contig022382-TilTARs.A053	0.324	0.799	0.406
contig045999-NyeTARs.A026	contig059768-ZebTARs.A022	0.394	0.972	0.406
contig086344-BriTARs.A020	contig022349-TilTARs.A035	0.358	0.881	0.406
contig060105-NyeTARs.A018	contig022368-TilTARs.A047	0.336	0.828	0.406
contig065494-BurTARs.A030	contig022334-TilTARs.A030	0.311	0.767	0.406
contig035375-NyeTARs.A013	contig053139-ZebTARs.A023	0.280	0.690	0.406
contig045302-BurTAR.A001	contig030471-ZebTARs.A024	0.449	1.106	0.406
contig022353-TilTARs.A036	contig022377-TilTARs.A050	0.139	0.343	0.406
contig061091-BurTARs.A014	contig022324-TilTARs.A027	0.158	0.391	0.406
contig056134-TilTARs.A058	contig030464-ZebTARs.A025	0.353	0.870	0.406
contig022341-TilTARs.A032	contig059768-ZebTARs.A022	0.349	0.861	0.406
contig059766-BurTARs.A029	contig035381-NyeTARs.A015	0.256	0.631	0.406
contig029633-BriTAR.A003	contig022334-TilTARs.A029	0.444	1.093	0.406
contig034854-BurTARs.A027	contig039640-TilTAR.A003	0.375	0.925	0.406
contig022345-TilTARs.A034	contig022356-TilTARs.A040	0.115	0.282	0.406
contig065494-BurTARs.A030	contig022334-TilTARs.A029	0.340	0.837	0.406
contig046013-NyeTARs.A021	contig066056-ZebTARs.A017	0.328	0.808	0.406
contig022377-TilTARs.A050	contig053139-ZebTARs.A023	0.345	0.849	0.406
contig041024-BurTARs.A026	contig061417-ZebTARs.A020	0.106	0.261	0.406
contig007512-TilTARs.A024	contig007520-TilTARs.A025	0.238	0.585	0.406
contig022337-TilTARs.A031	contig022365-TilTARs.A045	0.153	0.377	0.406
contig084876-BriTARs.A015	contig086337-BriTARs.A019	0.278	0.684	0.406
contig065494-BurTARs.A030	contig035381-NyeTARs.A015	0.259	0.637	0.406

contig022324-TilTARs.A027	contig022354-TilTARs.A037	0.201	0.494	0.406
contig084876-BriTARs.A015	contig007520-TilTARs.A025	0.311	0.764	0.406
contig061977-BurTARs.A012	contig022330-TilTARs.A028	0.230	0.565	0.406
contig058002-NyeTARs.A025	contig022379-TilTARs.A052	0.295	0.727	0.406
contig084886-BriTARs.A017	contig061410-ZebTARs.A021	0.248	0.610	0.406
contig039642-TilTAR.A004	contig061410-ZebTARs.A021	0.397	0.977	0.406
contig057145-BurTARs.A018	contig022337-TilTARs.A031	0.137	0.336	0.406
contig022365-TilTARs.A045	contig059768-ZebTARs.A022	0.351	0.864	0.406
contig057145-BurTARs.A018	contig053145-ZebTARs.A028	0.355	0.874	0.407
contig086344-BriTARs.A020	contig057148-BurTARs.A028	0.345	0.849	0.407
contig022337-TilTARs.A031	contig022368-TilTARs.A047	0.156	0.385	0.407
contig042499-NyeTARs.A028	contig040586-ZebTAR.A002	0.471	1.158	0.407
contig056023-BurTARs.A019	contig039640-TilTAR.A002	0.465	1.143	0.407
contig032272-NyeTAR.A004	contig066285-ZebTARs.A016	0.403	0.989	0.407
contig065494-BurTARs.A030	contig022379-TilTARs.A052	0.269	0.662	0.407
contig045999-NyeTARs.A026	contig040586-ZebTAR.A001	0.447	1.098	0.407
contig007520-TilTARs.A025	contig053139-ZebTARs.A023	0.234	0.575	0.407
contig082565-BriTARs.A022	contig040586-ZebTAR.A002	0.384	0.943	0.407
contig084876-BriTARs.A015	contig039642-TilTAR.A004	0.408	1.002	0.407
contig065494-BurTARs.A030	contig030445-ZebTARs.A026	0.255	0.627	0.407
contig035375-NyeTARs.A013	contig022334-TilTARs.A030	0.246	0.604	0.407
contig041024-BurTARs.A026	contig061410-ZebTARs.A021	0.238	0.584	0.407
contig057148-BurTARs.A028	contig062676-ZebTARs.A019	0.248	0.609	0.407
contig055697-BurTARs.A022	contig062677-ZebTARs.A018	0.334	0.820	0.407
contig022354-TilTARs.A038	contig056134-TilTARs.A058	0.337	0.827	0.407
contig035375-NyeTARs.A013	contig056134-TilTARs.A058	0.290	0.711	0.408
contig045088-TilTARs.A057	contig040586-ZebTAR.A001	0.492	1.208	0.408
contig065494-BurTARs.A030	contig022375-TilTARs.A048	0.316	0.775	0.408
contig086344-BriTARs.A020	contig041024-BurTARs.A026	0.322	0.790	0.408
contig045302-BurTAR.A001	contig049534-BurTARs.A025	0.448	1.098	0.408
contig007520-TilTARs.A025	contig022379-TilTARs.A052	0.296	0.726	0.408
contig084876-BriTARs.A015	contig046010-NyeTARs.A019	0.261	0.641	0.408
contig084887-BriTARs.A018	contig022354-TilTARs.A038	0.136	0.332	0.408
contig046010-NyeTARs.A019	contig022383-TilTARs.A054	0.256	0.628	0.408
contig045302-BurTAR.A001	contig056021-BurTARs.A020	0.422	1.035	0.408
contig084880-BriTARs.A016	contig030464-ZebTARs.A025	0.187	0.458	0.408
contig062039-NyeTARs.A022	contig056200-NyeTARs.A029	0.255	0.626	0.408
contig046010-NyeTARs.A019	contig045088-TilTARs.A057	0.374	0.916	0.408
contig007512-TilTARs.A024	contig007524-TilTARs.A026	0.248	0.608	0.408
contig007512-TilTARs.A024	contig030440-ZebTARs.A029	0.285	0.698	0.408
contig057305-BurTARs.A031	contig059768-ZebTARs.A022	0.383	0.939	0.408
contig060292-NyeTARs.A027	contig059768-ZebTARs.A022	0.383	0.939	0.408
contig046007-NyeTARs.A017	contig066056-ZebTARs.A017	0.337	0.826	0.408
contig049540-BurTARs.A024	contig053139-ZebTARs.A023	0.333	0.815	0.408
contig046013-NyeTARs.A021	contig066285-ZebTARs.A016	0.161	0.394	0.408
contig022383-TilTARs.A054	contig030464-ZebTARs.A025	0.263	0.645	0.408
contig084880-BriTARs.A016	contig045302-BurTAR.A001	0.458	1.123	0.408
contig045302-BurTAR.A001	contig022357-TilTARs.A041	0.443	1.087	0.408
contig046011-NyeTARs.A016	contig053145-ZebTARs.A028	0.378	0.927	0.408
contig046013-NyeTARs.A021	contig022390-TilTARs.A055	0.207	0.508	0.408
contig007512-TilTARs.A024	contig061410-ZebTARs.A021	0.273	0.668	0.408
contig084868-BriTARs.A014	contig022377-TilTARs.A050	0.260	0.636	0.408
contig042499-NyeTARs.A028	contig039640-TilTAR.A002	0.471	1.154	0.408

contig022390-TilTARs.A055	contig053145-ZebTARs.A028	0.264	0.646	0.408
contig022390-TilTARs.A055	contig066330-ZebTARs.A027	0.264	0.646	0.408
contig022334-TilTARs.A029	contig059768-ZebTARs.A022	0.347	0.849	0.408
contig057145-BurTARs.A018	contig059766-BurTARs.A029	0.357	0.875	0.408
contig056020-BurTARs.A021	contig046010-NyeTARs.A019	0.138	0.338	0.408
contig007520-TilTARs.A025	contig022365-TilTARs.A045	0.369	0.905	0.408
contig023443-TilTAR.A005	contig066285-ZebTARs.A016	0.393	0.962	0.408
contig039642-TilTAR.A004	contig007518-TilTARs.A019	0.473	1.159	0.408
contig038663-NyeTAR.A005	contig045088-TilTARs.A057	0.494	1.209	0.409
contig046011-NyeTARs.A016	contig003909-ZebTAR.A003	0.450	1.102	0.409
contig041024-BurTARs.A026	contig060105-NyeTARs.A018	0.315	0.771	0.409
contig061433-BurTARs.A013	contig003909-ZebTAR.A003	0.423	1.035	0.409
contig062677-ZebTARs.A018	contig059768-ZebTARs.A022	0.367	0.898	0.409
contig056021-BurTARs.A020	contig040586-ZebTAR.A001	0.428	1.047	0.409
contig046014-NyeTARs.A024	contig066691-ZebTARs.A015	0.112	0.274	0.409
contig022368-TilTARs.A046	contig059768-ZebTARs.A022	0.358	0.875	0.409
contig086337-BriTARs.A019	contig034854-BurTARs.A027	0.302	0.737	0.409
contig046007-NyeTARs.A017	contig022356-TilTARs.A040	0.124	0.302	0.409
contig054630-BurTARs.A023	contig022377-TilTARs.A050	0.371	0.907	0.409
contig046011-NyeTARs.A016	contig040586-ZebTAR.A001	0.510	1.248	0.409
contig084868-BriTARs.A014	contig022379-TilTARs.A052	0.158	0.387	0.409
contig060105-NyeTARs.A018	contig061410-ZebTARs.A021	0.312	0.764	0.409
contig029633-BriTAR.A003	contig066285-ZebTARs.A016	0.451	1.102	0.409
contig059766-BurTARs.A029	contig022343-TilTARs.A033	0.337	0.823	0.409
contig082565-BriTARs.A022	contig007520-TilTARs.A025	0.313	0.765	0.409
contig059673-BurTARs.A016	contig040586-ZebTAR.A002	0.462	1.128	0.409
contig032272-NyeTAR.A004	contig046011-NyeTARs.A016	0.451	1.101	0.409
contig022354-TilTARs.A038	contig061417-ZebTARs.A020	0.121	0.295	0.409
contig056023-BurTARs.A019	contig038663-NyeTAR.A005	0.446	1.090	0.409
contig022379-TilTARs.A052	contig066330-ZebTARs.A027	0.269	0.658	0.409
contig029633-BriTAR.A003	contig022324-TilTARs.A027	0.476	1.162	0.409
contig038663-NyeTAR.A005	contig022368-TilTARs.A047	0.439	1.073	0.409
contig059673-BurTARs.A016	contig040586-ZebTAR.A001	0.458	1.118	0.409
contig046010-NyeTARs.A019	contig042499-NyeTARs.A028	0.368	0.899	0.409
contig035381-NyeTARs.A015	contig040586-ZebTAR.A002	0.386	0.943	0.410
contig029633-BriTAR.A003	contig061433-BurTARs.A013	0.458	1.118	0.410
contig056200-NyeTARs.A029	contig056134-TilTARs.A058	0.362	0.883	0.410
contig046010-NyeTARs.A019	contig045999-NyeTARs.A026	0.181	0.442	0.410
contig046011-NyeTARs.A016	contig066330-ZebTARs.A027	0.388	0.946	0.410
contig022375-TilTARs.A048	contig040586-ZebTAR.A001	0.401	0.980	0.410
contig061433-BurTARs.A013	contig066285-ZebTARs.A016	0.173	0.423	0.410
contig058002-NyeTARs.A025	contig066890-ZebTARs.A014	0.325	0.793	0.410
contig022349-TilTARs.A035	contig022356-TilTARs.A040	0.143	0.350	0.410
contig059766-BurTARs.A029	contig046011-NyeTARs.A016	0.381	0.929	0.410
contig054630-BurTARs.A023	contig066056-ZebTARs.A017	0.347	0.846	0.410
contig045302-BurTAR.A001	contig061417-ZebTARs.A020	0.422	1.030	0.410
contig059766-BurTARs.A029	contig022362-TilTARs.A042	0.326	0.796	0.410
contig056023-BurTARs.A019	contig054630-BurTARs.A023	0.404	0.985	0.410
contig022383-TilTARs.A054	contig040586-ZebTAR.A002	0.398	0.972	0.410
contig061433-BurTARs.A013	contig023443-TilTAR.A005	0.405	0.987	0.410
contig045302-BurTAR.A001	contig007524-TilTARs.A026	0.438	1.069	0.410
contig040586-ZebTAR.A002	contig030440-ZebTARs.A029	0.388	0.945	0.410
contig035377-NyeTARs.A023	contig022341-TilTARs.A032	0.257	0.625	0.410

contig056200-NyeTARs.A029	contig059768-ZebTARs.A022	0.369	0.899	0.410
contig084887-BriTARs.A018	contig030464-ZebTARs.A025	0.150	0.366	0.410
contig038663-NyeTAR.A005	contig022379-TilTARs.A052	0.424	1.033	0.410
contig084887-BriTARs.A018	contig066056-ZebTARs.A017	0.316	0.770	0.410
contig022375-TilTARs.A048	contig066691-ZebTARs.A015	0.148	0.361	0.411
contig022337-TilTARs.A031	contig066285-ZebTARs.A016	0.151	0.367	0.411
contig059673-BurTARs.A016	contig039642-TilTAR.A004	0.455	1.109	0.411
contig084886-BriTARs.A017	contig022349-TilTARs.A035	0.149	0.362	0.411
contig022334-TilTARs.A030	contig022390-TilTARs.A055	0.260	0.633	0.411
contig022349-TilTARs.A035	contig022354-TilTARs.A037	0.175	0.425	0.411
contig086337-BriTARs.A019	contig022357-TilTARs.A041	0.369	0.898	0.411
contig045999-NyeTARs.A026	contig022390-TilTARs.A055	0.304	0.740	0.411
contig022334-TilTARs.A029	contig066330-ZebTARs.A027	0.342	0.832	0.411
contig057148-BurTARs.A028	contig046007-NyeTARs.A017	0.144	0.351	0.411
contig022334-TilTARs.A030	contig066330-ZebTARs.A027	0.313	0.762	0.411
contig022345-TilTARs.A034	contig056134-TilTARs.A058	0.354	0.860	0.411
contig057305-BurTARs.A031	contig022368-TilTARs.A046	0.393	0.957	0.411
contig060292-NyeTARs.A027	contig022368-TilTARs.A046	0.393	0.957	0.411
contig022375-TilTARs.A048	contig066285-ZebTARs.A016	0.174	0.423	0.411
contig038663-NyeTAR.A005	contig022324-TilTARs.A027	0.458	1.115	0.411
contig040586-ZebTAR.A001	contig061417-ZebTARs.A020	0.428	1.042	0.411
contig007518-TilTARs.A019	contig066691-ZebTARs.A015	0.375	0.912	0.411
contig086337-BriTARs.A019	contig022362-TilTARs.A042	0.323	0.786	0.411
contig022337-TilTARs.A031	contig022320-TilTARs.A059	0.141	0.343	0.411
contig045302-BurTAR.A001	contig056023-BurTARs.A019	0.452	1.099	0.411
contig057145-BurTARs.A018	contig046007-NyeTARs.A017	0.132	0.322	0.411
contig022356-TilTARs.A040	contig056134-TilTARs.A058	0.352	0.856	0.411
contig035381-NyeTARs.A015	contig066330-ZebTARs.A027	0.261	0.633	0.412
contig045302-BurTAR.A001	contig022375-TilTARs.A048	0.402	0.977	0.412
contig086344-BriTARs.A020	contig030464-ZebTARs.A025	0.344	0.836	0.412
contig084876-BriTARs.A015	contig065494-BurTARs.A030	0.283	0.688	0.412
contig061433-BurTARs.A013	contig057145-BurTARs.A018	0.172	0.418	0.412
contig057145-BurTARs.A018	contig057148-BurTARs.A028	0.144	0.351	0.412
contig060105-NyeTARs.A018	contig022354-TilTARs.A038	0.336	0.816	0.412
contig022356-TilTARs.A040	contig066056-ZebTARs.A017	0.333	0.808	0.412
contig022362-TilTARs.A042	contig053139-ZebTARs.A023	0.325	0.789	0.412
contig022375-TilTARs.A048	contig061410-ZebTARs.A021	0.214	0.519	0.412
contig007518-TilTARs.A019	contig059768-ZebTARs.A022	0.382	0.929	0.412
contig049534-BurTARs.A025	contig030445-ZebTARs.A026	0.257	0.623	0.412
contig049540-BurTARs.A024	contig022383-TilTARs.A054	0.163	0.397	0.412
contig061977-BurTARs.A012	contig046014-NyeTARs.A024	0.237	0.574	0.412
contig057145-BurTARs.A018	contig022383-TilTARs.A054	0.253	0.615	0.412
contig038663-NyeTAR.A005	contig022377-TilTARs.A050	0.439	1.067	0.412
contig061977-BurTARs.A012	contig062039-NyeTARs.A022	0.191	0.465	0.412
contig056020-BurTARs.A021	contig058002-NyeTARs.A025	0.345	0.838	0.412
contig007520-TilTARs.A025	contig022368-TilTARs.A047	0.357	0.867	0.412
contig061977-BurTARs.A012	contig022334-TilTARs.A029	0.230	0.558	0.412
contig060707-BurTARs.A015	contig046014-NyeTARs.A024	0.318	0.772	0.412
contig086351-BriTARs.A021	contig059768-ZebTARs.A022	0.387	0.940	0.412
contig061977-BurTARs.A012	contig022320-TilTARs.A059	0.238	0.578	0.412
contig022378-TilTARs.A051	contig030464-ZebTARs.A025	0.252	0.612	0.412
contig082565-BriTARs.A022	contig053145-ZebTARs.A028	0.261	0.634	0.412
contig045302-BurTAR.A001	contig022368-TilTARs.A047	0.431	1.045	0.412

contig029633-BriTAR.A003	contig046011-NyeTARs.A016	0.501	1.216	0.412
contig022354-TilTARs.A038	contig062677-ZebTARs.A018	0.260	0.632	0.412
contig057145-BurTARs.A018	contig056134-TilTARs.A058	0.361	0.875	0.412
contig038663-NyeTAR.A005	contig007520-TilTARs.A025	0.440	1.066	0.412
contig057301-BurTARs.A017	contig039642-TilTAR.A004	0.468	1.136	0.412
contig066890-ZebTARs.A014	contig066056-ZebTARs.A017	0.329	0.797	0.412
contig057305-BurTARs.A031	contig007520-TilTARs.A025	0.256	0.620	0.412
contig086337-BriTARs.A019	contig022343-TilTARs.A033	0.320	0.776	0.412
contig035381-NyeTARs.A015	contig058002-NyeTARs.A025	0.305	0.740	0.412
contig007518-TilTARs.A019	contig045088-TilTARs.A057	0.285	0.691	0.412
contig022324-TilTARs.A027	contig066285-ZebTARs.A016	0.168	0.409	0.412
contig007520-TilTARs.A025	contig022368-TilTARs.A046	0.340	0.823	0.412
contig061091-BurTARs.A014	contig066056-ZebTARs.A017	0.327	0.793	0.412
contig060707-BurTARs.A015	contig054630-BurTARs.A023	0.348	0.844	0.413
contig061433-BurTARs.A013	contig030471-ZebTARs.A024	0.155	0.376	0.413
contig046010-NyeTARs.A019	contig022368-TilTARs.A046	0.145	0.352	0.413
contig030445-ZebTARs.A026	contig066330-ZebTARs.A027	0.257	0.623	0.413
contig086351-BriTARs.A021	contig055697-BurTARs.A022	0.284	0.689	0.413
contig084886-BriTARs.A017	contig062039-NyeTARs.A022	0.257	0.624	0.413
contig061433-BurTARs.A013	contig022356-TilTARs.A040	0.168	0.406	0.413
contig049534-BurTARs.A025	contig046013-NyeTARs.A021	0.154	0.373	0.413
contig022334-TilTARs.A029	contig022345-TilTARs.A034	0.116	0.282	0.413
contig057145-BurTARs.A018	contig030445-ZebTARs.A026	0.226	0.548	0.413
contig022356-TilTARs.A040	contig053145-ZebTARs.A028	0.346	0.838	0.413
contig025313-BriTAR.A002	contig066285-ZebTARs.A016	0.399	0.966	0.413
contig084876-BriTARs.A015	contig060105-NyeTARs.A018	0.323	0.784	0.413
contig086344-BriTARs.A020	contig086351-BriTARs.A021	0.304	0.737	0.413
contig039639-TilTAR.A001	contig022390-TilTARs.A055	0.458	1.110	0.413
contig086351-BriTARs.A021	contig022362-TilTARs.A042	0.394	0.953	0.413
contig029633-BriTAR.A003	contig066691-ZebTARs.A015	0.455	1.103	0.413
contig061433-BurTARs.A013	contig022354-TilTARs.A038	0.157	0.381	0.413
contig022365-TilTARs.A045	contig030464-ZebTARs.A025	0.154	0.372	0.413
contig056021-BurTARs.A020	contig022368-TilTARs.A046	0.137	0.332	0.413
contig022343-TilTARs.A033	contig053139-ZebTARs.A023	0.315	0.764	0.413
contig038663-NyeTAR.A005	contig022357-TilTARs.A041	0.447	1.082	0.413
contig025313-BriTAR.A002	contig061433-BurTARs.A013	0.415	1.005	0.413
contig038663-NyeTAR.A005	contig062676-ZebTARs.A019	0.418	1.011	0.413
contig038663-NyeTAR.A005	contig007524-TilTARs.A026	0.445	1.077	0.413
contig057145-BurTARs.A018	contig007520-TilTARs.A025	0.335	0.811	0.413
contig066285-ZebTARs.A016	contig059768-ZebTARs.A022	0.394	0.954	0.413
contig055697-BurTARs.A022	contig056200-NyeTARs.A029	0.334	0.808	0.413
contig049534-BurTARs.A025	contig022375-TilTARs.A048	0.172	0.416	0.413
contig059673-BurTARs.A016	contig060105-NyeTARs.A018	0.309	0.747	0.413
contig022368-TilTARs.A047	contig030464-ZebTARs.A025	0.151	0.365	0.413
contig007520-TilTARs.A025	contig022345-TilTARs.A034	0.328	0.793	0.413
contig084876-BriTARs.A015	contig062676-ZebTARs.A019	0.145	0.351	0.413
contig007518-TilTARs.A019	contig022353-TilTARs.A036	0.401	0.969	0.414
contig061091-BurTARs.A014	contig022343-TilTARs.A033	0.103	0.250	0.414
contig054630-BurTARs.A023	contig049540-BurTARs.A024	0.378	0.914	0.414
contig046007-NyeTARs.A017	contig022365-TilTARs.A045	0.138	0.333	0.414
contig022345-TilTARs.A034	contig022368-TilTARs.A046	0.129	0.313	0.414
contig084886-BriTARs.A017	contig022377-TilTARs.A050	0.150	0.362	0.414
contig046010-NyeTARs.A019	contig062677-ZebTARs.A018	0.276	0.668	0.414

contig061091-BurTARs.A014	contig046014-NyeTARs.A024	0.109	0.263	0.414
contig086344-BriTARs.A020	contig046010-NyeTARs.A019	0.346	0.836	0.414
contig003909-ZebTAR.A003	contig066285-ZebTARs.A016	0.403	0.974	0.414
contig022324-TilTARs.A027	contig022357-TilTARs.A041	0.166	0.401	0.414
contig056020-BurTARs.A021	contig022341-TilTARs.A032	0.126	0.305	0.414
contig020038-BurTAR.A002	contig046011-NyeTARs.A016	0.450	1.088	0.414
contig057145-BurTARs.A018	contig035377-NyeTARs.A023	0.259	0.625	0.414
contig022330-TilTARs.A028	contig056134-TilTARs.A058	0.351	0.849	0.414
contig022377-TilTARs.A050	contig059768-ZebTARs.A022	0.365	0.881	0.414
contig007520-TilTARs.A025	contig022362-TilTARs.A042	0.339	0.818	0.414
contig061433-BurTARs.A013	contig056134-TilTARs.A058	0.378	0.914	0.414
contig022357-TilTARs.A041	contig053145-ZebTARs.A028	0.378	0.912	0.414
contig056023-BurTARs.A019	contig059768-ZebTARs.A022	0.397	0.958	0.414
contig035381-NyeTARs.A015	contig022377-TilTARs.A050	0.246	0.594	0.414
contig022379-TilTARs.A052	contig056134-TilTARs.A058	0.297	0.718	0.414
contig062039-NyeTARs.A022	contig007518-TilTARs.A019	0.340	0.821	0.414
contig057148-BurTARs.A028	contig007518-TilTARs.A019	0.373	0.901	0.414
contig045088-TilTARs.A057	contig030464-ZebTARs.A025	0.385	0.930	0.414
contig022334-TilTARs.A030	contig022362-TilTARs.A042	0.182	0.439	0.414
contig086337-BriTARs.A019	contig049540-BurTARs.A024	0.341	0.823	0.414
contig084868-BriTARs.A014	contig084876-BriTARs.A015	0.163	0.394	0.414
contig084876-BriTARs.A015	contig030464-ZebTARs.A025	0.265	0.641	0.414
contig059766-BurTARs.A029	contig022375-TilTARs.A048	0.308	0.742	0.414
contig057148-BurTARs.A028	contig022379-TilTARs.A052	0.245	0.590	0.414
contig038663-NyeTAR.A005	contig030471-ZebTARs.A024	0.463	1.117	0.414
contig061433-BurTARs.A013	contig046013-NyeTARs.A021	0.166	0.400	0.414
contig022334-TilTARs.A029	contig022377-TilTARs.A050	0.135	0.325	0.414
contig060707-BurTARs.A015	contig066890-ZebTARs.A014	0.328	0.792	0.414
contig082565-BriTARs.A022	contig059766-BurTARs.A029	0.263	0.635	0.414
contig057145-BurTARs.A018	contig022390-TilTARs.A055	0.242	0.583	0.414
contig057301-BurTARs.A017	contig040586-ZebTAR.A001	0.491	1.184	0.414
contig066056-ZebTARs.A017	contig059768-ZebTARs.A022	0.341	0.822	0.414
contig086344-BriTARs.A020	contig059768-ZebTARs.A022	0.350	0.844	0.415
contig029633-BriTAR.A003	contig062676-ZebTARs.A019	0.420	1.013	0.415
contig086351-BriTARs.A021	contig062039-NyeTARs.A022	0.362	0.874	0.415
contig084886-BriTARs.A017	contig053145-ZebTARs.A028	0.347	0.837	0.415
contig022354-TilTARs.A038	contig030471-ZebTARs.A024	0.139	0.335	0.415
contig022341-TilTARs.A032	contig062677-ZebTARs.A018	0.275	0.663	0.415
contig007518-TilTARs.A019	contig022320-TilTARs.A059	0.349	0.842	0.415
contig022377-TilTARs.A050	contig066691-ZebTARs.A015	0.144	0.346	0.415
contig082565-BriTARs.A022	contig058002-NyeTARs.A025	0.310	0.747	0.415
contig057301-BurTARs.A017	contig007518-TilTARs.A019	0.287	0.692	0.415
contig056020-BurTARs.A021	contig057148-BurTARs.A028	0.140	0.337	0.415
contig060292-NyeTARs.A027	contig007520-TilTARs.A025	0.254	0.611	0.415
contig030471-ZebTARs.A024	contig030464-ZebTARs.A025	0.156	0.377	0.415
contig055697-BurTARs.A022	contig046013-NyeTARs.A021	0.354	0.853	0.415
contig060105-NyeTARs.A018	contig035377-NyeTARs.A023	0.331	0.797	0.415
contig057148-BurTARs.A028	contig035376-NyeTARs.A014	0.247	0.596	0.415
contig022357-TilTARs.A041	contig056134-TilTARs.A058	0.380	0.916	0.415
contig007512-TilTARs.A024	contig066691-ZebTARs.A015	0.369	0.888	0.415
contig084887-BriTARs.A018	contig022343-TilTARs.A033	0.104	0.250	0.415
contig066691-ZebTARs.A015	contig053139-ZebTARs.A023	0.362	0.872	0.415
contig022330-TilTARs.A028	contig030471-ZebTARs.A024	0.119	0.288	0.415

contig086344-BriTARs.A020	contig061433-BurTARs.A013	0.344	0.829	0.415
contig022357-TilTARs.A041	contig022375-TilTARs.A048	0.164	0.396	0.415
contig086351-BriTARs.A021	contig022383-TilTARs.A054	0.347	0.836	0.415
contig041024-BurTARs.A026	contig066691-ZebTARs.A015	0.115	0.278	0.415
contig029633-BriTAR.A003	contig030445-ZebTARs.A026	0.391	0.943	0.415
contig039640-TilTAR.A003	contig022390-TilTARs.A056	0.403	0.970	0.415
contig029633-BriTAR.A003	contig059673-BurTARs.A016	0.474	1.141	0.415
contig061433-BurTARs.A013	contig032272-NyeTAR.A004	0.423	1.019	0.415
contig035381-NyeTARs.A015	contig022341-TilTARs.A032	0.246	0.593	0.415
contig057145-BurTARs.A018	contig065494-BurTARs.A030	0.366	0.880	0.415
contig022375-TilTARs.A048	contig053145-ZebTARs.A028	0.308	0.741	0.415
contig084880-BriTARs.A016	contig057148-BurTARs.A028	0.189	0.456	0.415
contig007512-TilTARs.A024	contig022343-TilTARs.A033	0.345	0.830	0.415
contig046007-NyeTARs.A017	contig058002-NyeTARs.A025	0.349	0.840	0.415
contig054630-BurTARs.A023	contig022368-TilTARs.A046	0.364	0.877	0.415
contig007520-TilTARs.A025	contig066890-ZebTARs.A014	0.327	0.787	0.415
contig038663-NyeTAR.A005	contig066285-ZebTARs.A016	0.456	1.099	0.415
contig054630-BurTARs.A023	contig022353-TilTARs.A036	0.417	1.004	0.415
contig062039-NyeTARs.A022	contig066890-ZebTARs.A014	0.235	0.566	0.416
contig086337-BriTARs.A019	contig057145-BurTARs.A018	0.339	0.817	0.416
contig038663-NyeTAR.A005	contig039639-TilTAR.A001	0.064	0.153	0.416
contig022334-TilTARs.A030	contig056134-TilTARs.A058	0.359	0.863	0.416
contig046013-NyeTARs.A021	contig066330-ZebTARs.A027	0.353	0.850	0.416
contig056020-BurTARs.A021	contig022368-TilTARs.A047	0.139	0.334	0.416
contig060707-BurTARs.A015	contig022334-TilTARs.A029	0.331	0.797	0.416
contig086337-BriTARs.A019	contig022382-TilTARs.A053	0.333	0.802	0.416
contig022368-TilTARs.A046	contig066056-ZebTARs.A017	0.336	0.807	0.416
contig058002-NyeTARs.A025	contig022354-TilTARs.A038	0.341	0.820	0.416
contig035381-NyeTARs.A015	contig022356-TilTARs.A040	0.241	0.580	0.416
contig042499-NyeTARs.A028	contig030464-ZebTARs.A025	0.379	0.912	0.416
contig061977-BurTARs.A012	contig007524-TilTARs.A026	0.311	0.748	0.416
contig062677-ZebTARs.A018	contig053139-ZebTARs.A023	0.335	0.805	0.416
contig084868-BriTARs.A014	contig086351-BriTARs.A021	0.366	0.880	0.416
contig061977-BurTARs.A012	contig022368-TilTARs.A047	0.253	0.609	0.416
contig029633-BriTAR.A003	contig035375-NyeTARs.A013	0.396	0.953	0.416
contig049540-BurTARs.A024	contig007512-TilTARs.A024	0.339	0.814	0.416
contig056200-NyeTARs.A029	contig022383-TilTARs.A054	0.165	0.397	0.416
contig045302-BurTAR.A001	contig007520-TilTARs.A025	0.433	1.041	0.416
contig022355-TilTARs.A039	contig022368-TilTARs.A046	0.131	0.314	0.416
contig022390-TilTARs.A055	contig040586-ZebTAR.A002	0.447	1.074	0.416
contig020038-BurTAR.A002	contig066285-ZebTARs.A016	0.407	0.978	0.416
contig060707-BurTARs.A015	contig022330-TilTARs.A028	0.328	0.788	0.416
contig065494-BurTARs.A030	contig046011-NyeTARs.A016	0.394	0.946	0.416
contig007518-TilTARs.A019	contig022390-TilTARs.A055	0.311	0.747	0.417
contig046014-NyeTARs.A024	contig022337-TilTARs.A031	0.139	0.333	0.417
contig022383-TilTARs.A054	contig040586-ZebTAR.A001	0.409	0.981	0.417
contig086344-BriTARs.A020	contig046014-NyeTARs.A024	0.326	0.783	0.417
contig086351-BriTARs.A021	contig060105-NyeTARs.A018	0.297	0.713	0.417
contig057145-BurTARs.A018	contig046010-NyeTARs.A019	0.144	0.346	0.417
contig022334-TilTARs.A029	contig022368-TilTARs.A046	0.132	0.316	0.417
contig022356-TilTARs.A040	contig030464-ZebTARs.A025	0.135	0.324	0.417
contig062039-NyeTARs.A022	contig022341-TilTARs.A032	0.235	0.565	0.417
contig058002-NyeTARs.A025	contig022330-TilTARs.A028	0.329	0.790	0.417

contig035377-NyeTARs.A023	contig022345-TilTARs.A034	0.259	0.621	0.417
contig045999-NyeTARs.A026	contig039639-TilTAR.A001	0.450	1.079	0.417
contig084868-BriTARs.A014	contig057145-BurTARs.A018	0.263	0.631	0.417
contig038663-NyeTAR.A005	contig022375-TilTARs.A048	0.407	0.976	0.417
contig066285-ZebTARs.A016	contig030445-ZebTARs.A026	0.257	0.616	0.417
contig022368-TilTARs.A046	contig045088-TilTARs.A057	0.367	0.880	0.417
contig084868-BriTARs.A014	contig022354-TilTARs.A038	0.259	0.621	0.417
contig007518-TilTARs.A019	contig022355-TilTARs.A039	0.383	0.918	0.417
contig062039-NyeTARs.A022	contig022334-TilTARs.A030	0.237	0.569	0.417
contig062039-NyeTARs.A022	contig022382-TilTARs.A053	0.248	0.595	0.417
contig084876-BriTARs.A015	contig045302-BurTAR.A001	0.410	0.982	0.417
contig022375-TilTARs.A048	contig022383-TilTARs.A054	0.225	0.539	0.417
contig060707-BurTARs.A015	contig059768-ZebTARs.A022	0.342	0.819	0.417
contig059766-BurTARs.A029	contig022356-TilTARs.A040	0.350	0.839	0.417
contig084886-BriTARs.A017	contig066330-ZebTARs.A027	0.351	0.841	0.417
contig049540-BurTARs.A024	contig022354-TilTARs.A038	0.262	0.629	0.417
contig022354-TilTARs.A038	contig053145-ZebTARs.A028	0.339	0.812	0.417
contig061091-BurTARs.A014	contig055697-BurTARs.A022	0.363	0.870	0.418
contig022349-TilTARs.A035	contig022354-TilTARs.A038	0.141	0.339	0.418
contig066285-ZebTARs.A016	contig062677-ZebTARs.A018	0.292	0.700	0.418
contig022341-TilTARs.A032	contig022343-TilTARs.A033	0.137	0.328	0.418
contig061433-BurTARs.A013	contig022337-TilTARs.A031	0.167	0.400	0.418
contig007518-TilTARs.A019	contig056134-TilTARs.A058	0.320	0.766	0.418
contig046007-NyeTARs.A017	contig022334-TilTARs.A029	0.124	0.296	0.418
contig034854-BurTARs.A027	contig022334-TilTARs.A030	0.244	0.585	0.418
contig060707-BurTARs.A015	contig022375-TilTARs.A048	0.334	0.799	0.418
contig060105-NyeTARs.A018	contig046014-NyeTARs.A024	0.319	0.764	0.418
contig057148-BurTARs.A028	contig030445-ZebTARs.A026	0.242	0.578	0.418
contig045302-BurTAR.A001	contig059673-BurTARs.A016	0.458	1.096	0.418
contig038663-NyeTAR.A005	contig061410-ZebTARs.A021	0.406	0.971	0.418
contig046010-NyeTARs.A019	contig022334-TilTARs.A030	0.188	0.451	0.418
contig054630-BurTARs.A023	contig046013-NyeTARs.A021	0.387	0.925	0.418
contig022356-TilTARs.A040	contig022375-TilTARs.A048	0.136	0.326	0.418
contig086337-BriTARs.A019	contig062677-ZebTARs.A018	0.342	0.817	0.418
contig084876-BriTARs.A015	contig066330-ZebTARs.A027	0.286	0.683	0.418
contig057148-BurTARs.A028	contig022334-TilTARs.A030	0.185	0.442	0.418
contig059766-BurTARs.A029	contig022357-TilTARs.A041	0.382	0.914	0.418
contig035381-NyeTARs.A015	contig022354-TilTARs.A038	0.243	0.581	0.418
contig022375-TilTARs.A048	contig066330-ZebTARs.A027	0.322	0.770	0.418
contig038663-NyeTAR.A005	contig045999-NyeTARs.A026	0.452	1.081	0.418
contig056021-BurTARs.A020	contig022375-TilTARs.A048	0.148	0.354	0.418
contig029633-BriTAR.A003	contig022357-TilTARs.A041	0.449	1.073	0.418
contig086337-BriTARs.A019	contig066691-ZebTARs.A015	0.365	0.872	0.418
contig084886-BriTARs.A017	contig086344-BriTARs.A020	0.334	0.798	0.418
contig084887-BriTARs.A018	contig057148-BurTARs.A028	0.150	0.359	0.418
contig049540-BurTARs.A024	contig059768-ZebTARs.A022	0.371	0.887	0.418
contig022354-TilTARs.A038	contig030440-ZebTARs.A029	0.249	0.596	0.418
contig022320-TilTARs.A059	contig066691-ZebTARs.A015	0.125	0.298	0.419
contig046010-NyeTARs.A019	contig007520-TilTARs.A025	0.348	0.831	0.419
contig022345-TilTARs.A034	contig066691-ZebTARs.A015	0.136	0.326	0.419
contig022357-TilTARs.A041	contig022390-TilTARs.A055	0.242	0.579	0.419
contig042499-NyeTARs.A028	contig022368-TilTARs.A046	0.365	0.873	0.419
contig057148-BurTARs.A028	contig030440-ZebTARs.A029	0.260	0.622	0.419

contig022379-TilTARs.A052	contig053145-ZebTARs.A028	0.276	0.660	0.419
contig007520-TilTARs.A025	contig030445-ZebTARs.A026	0.304	0.727	0.419
contig034854-BurTARs.A027	contig056134-TilTARs.A058	0.293	0.701	0.419
contig039639-TilTAR.A001	contig040586-ZebTAR.A001	0.060	0.144	0.419
contig022375-TilTARs.A048	contig061417-ZebTARs.A020	0.146	0.349	0.419
contig007512-TilTARs.A024	contig022379-TilTARs.A052	0.287	0.685	0.419
contig084887-BriTARs.A018	contig060707-BurTARs.A015	0.329	0.785	0.419
contig084886-BriTARs.A017	contig059766-BurTARs.A029	0.351	0.839	0.419
contig022363-TilTARs.A043	contig022377-TilTARs.A050	0.188	0.449	0.419
contig045088-TilTARs.A057	contig066285-ZebTARs.A016	0.384	0.917	0.419
contig066330-ZebTARs.A027	contig030440-ZebTARs.A029	0.281	0.670	0.419
contig086344-BriTARs.A020	contig066890-ZebTARs.A014	0.332	0.792	0.419
contig007512-TilTARs.A024	contig022357-TilTARs.A041	0.376	0.897	0.419
contig046011-NyeTARs.A016	contig039642-TilTAR.A004	0.504	1.203	0.419
contig022368-TilTARs.A047	contig059768-ZebTARs.A022	0.351	0.837	0.419
contig046013-NyeTARs.A021	contig022324-TilTARs.A027	0.166	0.395	0.419
contig022354-TilTARs.A037	contig022375-TilTARs.A048	0.113	0.269	0.419
contig056021-BurTARs.A020	contig022363-TilTARs.A043	0.158	0.376	0.419
contig045302-BurTAR.A001	contig035375-NyeTARs.A013	0.395	0.943	0.419
contig035377-NyeTARs.A023	contig039642-TilTAR.A004	0.413	0.984	0.419
contig054630-BurTARs.A023	contig066285-ZebTARs.A016	0.401	0.956	0.419
contig022355-TilTARs.A039	contig066056-ZebTARs.A017	0.327	0.779	0.420
contig057301-BurTARs.A017	contig038663-NyeTAR.A005	0.498	1.188	0.420
contig061977-BurTARs.A012	contig035377-NyeTARs.A023	0.154	0.367	0.420
contig056134-TilTARs.A058	contig066691-ZebTARs.A015	0.360	0.857	0.420
contig049534-BurTARs.A025	contig022368-TilTARs.A047	0.153	0.365	0.420
contig022377-TilTARs.A050	contig066056-ZebTARs.A017	0.310	0.738	0.420
contig061977-BurTARs.A012	contig030471-ZebTARs.A024	0.255	0.607	0.420
contig046010-NyeTARs.A019	contig022382-TilTARs.A053	0.273	0.651	0.420
contig029633-BriTAR.A003	contig082565-BriTARs.A022	0.394	0.939	0.420
contig022330-TilTARs.A028	contig022345-TilTARs.A034	0.118	0.281	0.420
contig084886-BriTARs.A017	contig065494-BurTARs.A030	0.353	0.841	0.420
contig084868-BriTARs.A014	contig030464-ZebTARs.A025	0.274	0.651	0.420
contig061091-BurTARs.A014	contig041024-BurTARs.A026	0.112	0.267	0.420
contig066691-ZebTARs.A015	contig066056-ZebTARs.A017	0.329	0.784	0.420
contig056021-BurTARs.A020	contig038663-NyeTAR.A005	0.435	1.035	0.420
contig058002-NyeTARs.A025	contig022390-TilTARs.A055	0.299	0.711	0.420
contig086337-BriTARs.A019	contig022356-TilTARs.A040	0.334	0.796	0.420
contig034854-BurTARs.A027	contig062039-NyeTARs.A022	0.234	0.556	0.420
contig058002-NyeTARs.A025	contig022377-TilTARs.A050	0.343	0.816	0.420
contig056020-BurTARs.A021	contig022365-TilTARs.A045	0.137	0.327	0.420
contig046014-NyeTARs.A024	contig022343-TilTARs.A033	0.125	0.299	0.420
contig082565-BriTARs.A022	contig065494-BurTARs.A030	0.266	0.633	0.420
contig082565-BriTARs.A022	contig022345-TilTARs.A034	0.245	0.582	0.420
contig022354-TilTARs.A037	contig030471-ZebTARs.A024	0.170	0.403	0.420
contig045302-BurTAR.A001	contig045999-NyeTARs.A026	0.458	1.090	0.420
contig061091-BurTARs.A014	contig060707-BurTARs.A015	0.340	0.809	0.420
contig057145-BurTARs.A018	contig030471-ZebTARs.A024	0.127	0.302	0.420
contig054630-BurTARs.A023	contig022368-TilTARs.A047	0.353	0.839	0.421
contig060105-NyeTARs.A018	contig022324-TilTARs.A027	0.341	0.811	0.421
contig057148-BurTARs.A028	contig022353-TilTARs.A036	0.162	0.385	0.421
contig061977-BurTARs.A012	contig066285-ZebTARs.A016	0.257	0.611	0.421
contig023443-TilTAR.A005	contig045088-TilTARs.A057	0.398	0.947	0.421

contig057145-BurTARs.A018	contig022354-TilTARs.A038	0.131	0.311	0.421
contig046010-NyeTARs.A019	contig056200-NyeTARs.A029	0.275	0.654	0.421
contig057145-BurTARs.A018	contig007524-TilTARs.A026	0.344	0.818	0.421
contig022330-TilTARs.A028	contig061410-ZebTARs.A021	0.233	0.553	0.421
contig056020-BurTARs.A021	contig022375-TilTARs.A048	0.148	0.351	0.421
contig046010-NyeTARs.A019	contig022353-TilTARs.A036	0.160	0.380	0.421
contig035376-NyeTARs.A014	contig053145-ZebTARs.A028	0.269	0.639	0.421
contig022368-TilTARs.A046	contig066285-ZebTARs.A016	0.169	0.401	0.421
contig022375-TilTARs.A048	contig022377-TilTARs.A050	0.162	0.384	0.421
contig061977-BurTARs.A012	contig056021-BurTARs.A020	0.234	0.556	0.421
contig007520-TilTARs.A025	contig022378-TilTARs.A051	0.339	0.805	0.421
contig022345-TilTARs.A034	contig022368-TilTARs.A047	0.128	0.303	0.421
contig059673-BurTARs.A016	contig038663-NyeTAR.A005	0.465	1.104	0.421
contig022355-TilTARs.A039	contig022368-TilTARs.A047	0.134	0.319	0.421
contig035376-NyeTARs.A014	contig066330-ZebTARs.A027	0.272	0.646	0.421
contig060707-BurTARs.A015	contig022334-TilTARs.A030	0.350	0.832	0.421
contig035375-NyeTARs.A013	contig062039-NyeTARs.A022	0.236	0.559	0.421
contig049534-BurTARs.A025	contig022362-TilTARs.A042	0.180	0.427	0.421
contig084876-BriTARs.A015	contig057145-BurTARs.A018	0.252	0.599	0.421
contig086351-BriTARs.A021	contig057148-BurTARs.A028	0.415	0.985	0.421
contig035375-NyeTARs.A013	contig040586-ZebTAR.A001	0.399	0.948	0.421
contig084868-BriTARs.A014	contig022320-TilTARs.A059	0.256	0.608	0.421
contig065494-BurTARs.A030	contig030440-ZebTARs.A029	0.284	0.674	0.421
contig061977-BurTARs.A012	contig030445-ZebTARs.A026	0.122	0.290	0.422
contig022343-TilTARs.A033	contig066691-ZebTARs.A015	0.103	0.245	0.422
contig029633-BriTAR.A003	contig061417-ZebTARs.A020	0.435	1.032	0.422
contig046010-NyeTARs.A019	contig053145-ZebTARs.A028	0.348	0.826	0.422
contig061091-BurTARs.A014	contig053139-ZebTARs.A023	0.362	0.859	0.422
contig058002-NyeTARs.A025	contig022345-TilTARs.A034	0.326	0.774	0.422
contig035377-NyeTARs.A023	contig022349-TilTARs.A035	0.277	0.657	0.422
contig046014-NyeTARs.A024	contig061410-ZebTARs.A021	0.239	0.566	0.422
contig061433-BurTARs.A013	contig022330-TilTARs.A028	0.158	0.374	0.422
contig049540-BurTARs.A024	contig022341-TilTARs.A032	0.274	0.649	0.422
contig007520-TilTARs.A025	contig022357-TilTARs.A041	0.379	0.898	0.422
contig022354-TilTARs.A038	contig066330-ZebTARs.A027	0.343	0.814	0.422
contig035376-NyeTARs.A014	contig022363-TilTARs.A043	0.270	0.640	0.422
contig057148-BurTARs.A028	contig022382-TilTARs.A053	0.271	0.643	0.422
contig057148-BurTARs.A028	contig062677-ZebTARs.A018	0.278	0.659	0.422
contig066056-ZebTARs.A017	contig030471-ZebTARs.A024	0.331	0.784	0.422
contig059673-BurTARs.A016	contig061410-ZebTARs.A021	0.321	0.760	0.422
contig049534-BurTARs.A025	contig038663-NyeTAR.A005	0.462	1.094	0.422
contig022320-TilTARs.A059	contig061410-ZebTARs.A021	0.240	0.570	0.422
contig065494-BurTARs.A030	contig022362-TilTARs.A042	0.336	0.795	0.422
contig065494-BurTARs.A030	contig022349-TilTARs.A035	0.344	0.816	0.422
contig056200-NyeTARs.A029	contig053139-ZebTARs.A023	0.335	0.793	0.422
contig065494-BurTARs.A030	contig035376-NyeTARs.A014	0.273	0.647	0.422
contig035377-NyeTARs.A023	contig040586-ZebTAR.A002	0.421	0.997	0.422
contig058002-NyeTARs.A025	contig022390-TilTARs.A056	0.327	0.773	0.422
contig055697-BurTARs.A022	contig022357-TilTARs.A041	0.375	0.888	0.422
contig056134-TilTARs.A058	contig061417-ZebTARs.A020	0.358	0.848	0.422
contig049534-BurTARs.A025	contig022383-TilTARs.A054	0.270	0.638	0.422
contig056021-BurTARs.A020	contig022354-TilTARs.A037	0.161	0.382	0.422
contig084886-BriTARs.A017	contig061433-BurTARs.A013	0.164	0.389	0.422

contig038663-NyeTAR.A005	contig061417-ZebTARs.A020	0.435	1.030	0.422
contig084876-BriTARs.A015	contig040586-ZebTAR.A001	0.415	0.983	0.422
contig046007-NyeTARs.A017	contig022341-TilTARs.A032	0.130	0.308	0.422
contig061977-BurTARs.A012	contig049534-BurTARs.A025	0.257	0.608	0.423
contig057305-BurTARs.A031	contig053139-ZebTARs.A023	0.288	0.681	0.423
contig084876-BriTARs.A015	contig022375-TilTARs.A048	0.220	0.521	0.423
contig061433-BurTARs.A013	contig022343-TilTARs.A033	0.164	0.387	0.423
contig057145-BurTARs.A018	contig066330-ZebTARs.A027	0.370	0.875	0.423
contig086337-BriTARs.A019	contig086351-BriTARs.A021	0.284	0.671	0.423
contig007518-TilTARs.A019	contig066056-ZebTARs.A017	0.319	0.754	0.423
contig057145-BurTARs.A018	contig030440-ZebTARs.A029	0.266	0.630	0.423
contig042499-NyeTARs.A028	contig039642-TilTAR.A004	0.468	1.106	0.423
contig084868-BriTARs.A014	contig057305-BurTARs.A031	0.351	0.830	0.423
contig057145-BurTARs.A018	contig062039-NyeTARs.A022	0.261	0.617	0.423
contig084880-BriTARs.A016	contig039642-TilTAR.A004	0.467	1.104	0.423
contig029633-BriTAR.A003	contig030440-ZebTARs.A029	0.398	0.941	0.423
contig022354-TilTARs.A038	contig022382-TilTARs.A053	0.254	0.600	0.423
contig042499-NyeTARs.A028	contig007518-TilTARs.A019	0.293	0.694	0.423
contig035376-NyeTARs.A014	contig007520-TilTARs.A025	0.311	0.736	0.423
contig061977-BurTARs.A012	contig035381-NyeTARs.A015	0.127	0.301	0.423
contig084886-BriTARs.A017	contig056021-BurTARs.A020	0.135	0.319	0.423
contig035376-NyeTARs.A014	contig022345-TilTARs.A034	0.244	0.577	0.423
contig066056-ZebTARs.A017	contig030440-ZebTARs.A029	0.329	0.777	0.423
contig086344-BriTARs.A020	contig022354-TilTARs.A038	0.336	0.794	0.423
contig046013-NyeTARs.A021	contig022345-TilTARs.A034	0.141	0.333	0.423
contig046013-NyeTARs.A021	contig059768-ZebTARs.A022	0.380	0.898	0.423
contig056023-BurTARs.A019	contig039639-TilTAR.A001	0.450	1.063	0.423
contig060707-BurTARs.A015	contig030440-ZebTARs.A029	0.329	0.778	0.423
contig059766-BurTARs.A029	contig035376-NyeTARs.A014	0.271	0.640	0.423
contig022341-TilTARs.A032	contig062676-ZebTARs.A019	0.247	0.583	0.423
contig029633-BriTAR.A003	contig030471-ZebTARs.A024	0.460	1.087	0.423
contig045302-BurTAR.A001	contig045088-TilTARs.A057	0.480	1.134	0.423
contig058002-NyeTARs.A025	contig030445-ZebTARs.A026	0.301	0.710	0.424
contig084886-BriTARs.A017	contig058002-NyeTARs.A025	0.345	0.814	0.424
contig084868-BriTARs.A014	contig022362-TilTARs.A042	0.275	0.650	0.424
contig084887-BriTARs.A018	contig086344-BriTARs.A020	0.338	0.797	0.424
contig084887-BriTARs.A018	contig022341-TilTARs.A032	0.131	0.310	0.424
contig057301-BurTARs.A017	contig023443-TilTAR.A005	0.404	0.953	0.424
contig049534-BurTARs.A025	contig058002-NyeTARs.A025	0.360	0.849	0.424
contig045302-BurTAR.A001	contig030445-ZebTARs.A026	0.384	0.905	0.424
contig049534-BurTARs.A025	contig022334-TilTARs.A030	0.224	0.527	0.424
contig054630-BurTARs.A023	contig060105-NyeTARs.A018	0.354	0.835	0.424
contig035377-NyeTARs.A023	contig022356-TilTARs.A040	0.258	0.609	0.424
contig007512-TilTARs.A024	contig062677-ZebTARs.A018	0.336	0.792	0.424
contig059766-BurTARs.A029	contig007518-TilTARs.A019	0.295	0.696	0.424
contig059766-BurTARs.A029	contig022379-TilTARs.A052	0.280	0.661	0.424
contig022362-TilTARs.A042	contig066330-ZebTARs.A027	0.335	0.791	0.424
contig086337-BriTARs.A019	contig057305-BurTARs.A031	0.291	0.686	0.424
contig084876-BriTARs.A015	contig022356-TilTARs.A040	0.254	0.598	0.424
contig086337-BriTARs.A019	contig056200-NyeTARs.A029	0.342	0.806	0.424
contig022349-TilTARs.A035	contig066330-ZebTARs.A027	0.344	0.811	0.424
contig084886-BriTARs.A017	contig007520-TilTARs.A025	0.346	0.815	0.424
contig007520-TilTARs.A025	contig022337-TilTARs.A031	0.355	0.837	0.424

contig057148-BurTARs.A028	contig022390-TilTARs.A056	0.264	0.622	0.424
contig046007-NyeTARs.A017	contig022368-TilTARs.A047	0.144	0.340	0.424
contig022330-TilTARs.A028	contig061417-ZebTARs.A020	0.039	0.091	0.424
contig057145-BurTARs.A018	contig058002-NyeTARs.A025	0.341	0.803	0.424
contig059673-BurTARs.A016	contig060292-NyeTARs.A027	0.269	0.635	0.424
contig020038-BurTAR.A002	contig061433-BurTARs.A013	0.428	1.008	0.424
contig065494-BurTARs.A030	contig022343-TilTARs.A033	0.347	0.818	0.424
contig065494-BurTARs.A030	contig022356-TilTARs.A040	0.358	0.844	0.424
contig035377-NyeTARs.A023	contig022337-TilTARs.A031	0.273	0.644	0.425
contig084887-BriTARs.A018	contig022355-TilTARs.A039	0.083	0.195	0.425
contig022353-TilTARs.A036	contig061417-ZebTARs.A020	0.114	0.269	0.425
contig046010-NyeTARs.A019	contig022379-TilTARs.A052	0.248	0.585	0.425
contig059673-BurTARs.A016	contig022362-TilTARs.A042	0.356	0.838	0.425
contig084887-BriTARs.A018	contig046007-NyeTARs.A017	0.087	0.204	0.425
contig056021-BurTARs.A020	contig062677-ZebTARs.A018	0.264	0.622	0.425
contig029633-BriTAR.A003	contig056021-BurTARs.A020	0.438	1.032	0.425
contig049540-BurTARs.A024	contig022382-TilTARs.A053	0.014	0.034	0.425
contig086351-BriTARs.A021	contig022368-TilTARs.A046	0.401	0.943	0.425
contig022337-TilTARs.A031	contig022345-TilTARs.A034	0.146	0.344	0.425
contig046007-NyeTARs.A017	contig022330-TilTARs.A028	0.126	0.295	0.425
contig022368-TilTARs.A046	contig030464-ZebTARs.A025	0.151	0.355	0.425
contig086344-BriTARs.A020	contig061091-BurTARs.A014	0.349	0.821	0.425
contig040586-ZebTAR.A001	contig030445-ZebTARs.A026	0.389	0.916	0.425
contig035377-NyeTARs.A023	contig022365-TilTARs.A045	0.275	0.646	0.425
contig062039-NyeTARs.A022	contig007520-TilTARs.A025	0.330	0.775	0.425
contig086344-BriTARs.A020	contig022354-TilTARs.A037	0.345	0.812	0.425
contig022363-TilTARs.A043	contig062676-ZebTARs.A019	0.275	0.647	0.425
contig022345-TilTARs.A034	contig022390-TilTARs.A056	0.255	0.599	0.425
contig022324-TilTARs.A027	contig022375-TilTARs.A048	0.186	0.438	0.425
contig086337-BriTARs.A019	contig061091-BurTARs.A014	0.365	0.858	0.425
contig007524-TilTARs.A026	contig022343-TilTARs.A033	0.339	0.797	0.425
contig062039-NyeTARs.A022	contig030440-ZebTARs.A029	0.242	0.568	0.425
contig035377-NyeTARs.A023	contig066285-ZebTARs.A016	0.271	0.636	0.425
contig060292-NyeTARs.A027	contig053139-ZebTARs.A023	0.285	0.671	0.425
contig060707-BurTARs.A015	contig046013-NyeTARs.A021	0.341	0.802	0.425
contig029633-BriTAR.A003	contig035376-NyeTARs.A014	0.405	0.952	0.425
contig022343-TilTARs.A033	contig066285-ZebTARs.A016	0.146	0.342	0.425
contig034854-BurTARs.A027	contig053145-ZebTARs.A028	0.286	0.673	0.426
contig022334-TilTARs.A030	contig022382-TilTARs.A053	0.233	0.547	0.426
contig007518-TilTARs.A019	contig022324-TilTARs.A027	0.377	0.887	0.426
contig082565-BriTARs.A022	contig066330-ZebTARs.A027	0.268	0.629	0.426
contig022341-TilTARs.A032	contig022363-TilTARs.A044	0.157	0.369	0.426
contig046013-NyeTARs.A021	contig053139-ZebTARs.A023	0.358	0.841	0.426
contig084876-BriTARs.A015	contig007512-TilTARs.A024	0.297	0.697	0.426
contig057145-BurTARs.A018	contig022379-TilTARs.A052	0.242	0.569	0.426
contig046010-NyeTARs.A019	contig066330-ZebTARs.A027	0.352	0.827	0.426
contig059673-BurTARs.A016	contig022343-TilTARs.A033	0.350	0.822	0.426
contig007520-TilTARs.A025	contig022390-TilTARs.A056	0.327	0.767	0.426
contig022368-TilTARs.A047	contig061410-ZebTARs.A021	0.256	0.600	0.426
contig022368-TilTARs.A046	contig022383-TilTARs.A054	0.262	0.614	0.426
contig022357-TilTARs.A041	contig053139-ZebTARs.A023	0.379	0.889	0.426
contig022334-TilTARs.A029	contig056134-TilTARs.A058	0.355	0.833	0.426
contig022362-TilTARs.A042	contig030464-ZebTARs.A025	0.165	0.388	0.426

contig059766-BurTARs.A029	contig022390-TilTARs.A055	0.275	0.646	0.426
contig065494-BurTARs.A030	contig022390-TilTARs.A055	0.275	0.646	0.426
contig056021-BurTARs.A020	contig049540-BurTARs.A024	0.263	0.617	0.426
contig082565-BriTARs.A022	contig049534-BurTARs.A025	0.268	0.628	0.426
contig056023-BurTARs.A019	contig046010-NyeTARs.A019	0.186	0.435	0.426
contig084876-BriTARs.A015	contig022354-TilTARs.A038	0.251	0.588	0.426
contig058002-NyeTARs.A025	contig022334-TilTARs.A029	0.329	0.772	0.426
contig084868-BriTARs.A014	contig022324-TilTARs.A027	0.278	0.652	0.426
contig034854-BurTARs.A027	contig066056-ZebTARs.A017	0.338	0.794	0.426
contig062039-NyeTARs.A022	contig061410-ZebTARs.A021	0.195	0.458	0.426
contig084886-BriTARs.A017	contig022330-TilTARs.A028	0.132	0.311	0.426
contig046010-NyeTARs.A019	contig058002-NyeTARs.A025	0.349	0.820	0.426
contig056200-NyeTARs.A029	contig022354-TilTARs.A038	0.264	0.620	0.426
contig060707-BurTARs.A015	contig034854-BurTARs.A027	0.339	0.795	0.426
contig025313-BriTAR.A002	contig003909-ZebTAR.A003	0.011	0.026	0.426
contig022363-TilTARs.A044	contig066056-ZebTARs.A017	0.339	0.795	0.426
contig061091-BurTARs.A014	contig056134-TilTARs.A058	0.360	0.844	0.427
contig056020-BurTARs.A021	contig066691-ZebTARs.A015	0.083	0.194	0.427
contig007518-TilTARs.A019	contig040586-ZebTAR.A001	0.484	1.136	0.427
contig022334-TilTARs.A029	contig061410-ZebTARs.A021	0.233	0.546	0.427
contig022334-TilTARs.A029	contig066285-ZebTARs.A016	0.149	0.350	0.427
contig084880-BriTARs.A016	contig040586-ZebTAR.A002	0.470	1.102	0.427
contig039642-TilTAR.A004	contig022390-TilTARs.A056	0.406	0.952	0.427
contig084886-BriTARs.A017	contig022334-TilTARs.A029	0.135	0.315	0.427
contig061433-BurTARs.A013	contig060707-BurTARs.A015	0.341	0.799	0.427
contig007524-TilTARs.A026	contig053139-ZebTARs.A023	0.245	0.575	0.427
contig060105-NyeTARs.A018	contig022375-TilTARs.A048	0.339	0.795	0.427
contig086351-BriTARs.A021	contig053139-ZebTARs.A023	0.284	0.666	0.427
contig007520-TilTARs.A025	contig022363-TilTARs.A043	0.362	0.847	0.427
contig086337-BriTARs.A019	contig060292-NyeTARs.A027	0.289	0.677	0.427
contig046013-NyeTARs.A021	contig056134-TilTARs.A058	0.358	0.838	0.427
contig060105-NyeTARs.A018	contig022334-TilTARs.A029	0.337	0.789	0.427
contig084868-BriTARs.A014	contig060292-NyeTARs.A027	0.355	0.831	0.427
contig084887-BriTARs.A018	contig022354-TilTARs.A037	0.160	0.375	0.427
contig084886-BriTARs.A017	contig022368-TilTARs.A046	0.149	0.349	0.428
contig084887-BriTARs.A018	contig056020-BurTARs.A021	0.085	0.198	0.428
contig030464-ZebTARs.A025	contig053145-ZebTARs.A028	0.353	0.825	0.428
contig039640-TilTAR.A003	contig040586-ZebTAR.A001	0.045	0.106	0.428
contig086337-BriTARs.A019	contig035375-NyeTARs.A013	0.286	0.668	0.428
contig022341-TilTARs.A032	contig022378-TilTARs.A051	0.224	0.523	0.428
contig035377-NyeTARs.A023	contig022334-TilTARs.A030	0.251	0.588	0.428
contig007520-TilTARs.A025	contig022330-TilTARs.A028	0.327	0.764	0.428
contig061977-BurTARs.A012	contig022368-TilTARs.A046	0.241	0.563	0.428
contig035376-NyeTARs.A014	contig040586-ZebTAR.A001	0.405	0.948	0.428
contig056021-BurTARs.A020	contig022354-TilTARs.A038	0.129	0.301	0.428
contig049540-BurTARs.A024	contig066285-ZebTARs.A016	0.292	0.682	0.428
contig035381-NyeTARs.A015	contig022383-TilTARs.A054	0.106	0.248	0.428
contig084868-BriTARs.A014	contig046010-NyeTARs.A019	0.272	0.634	0.428
contig046014-NyeTARs.A024	contig022334-TilTARs.A029	0.114	0.267	0.428
contig007518-TilTARs.A019	contig053145-ZebTARs.A028	0.295	0.690	0.428
contig084876-BriTARs.A015	contig022365-TilTARs.A045	0.263	0.615	0.428
contig060105-NyeTARs.A018	contig022330-TilTARs.A028	0.334	0.780	0.428
contig060707-BurTARs.A015	contig066691-ZebTARs.A015	0.342	0.799	0.428

contig039640-TilTAR.A003	contig066890-ZebTARs.A014	0.400	0.934	0.428
contig084887-BriTARs.A018	contig061417-ZebTARs.A020	0.117	0.272	0.428
contig045302-BurTAR.A001	contig030440-ZebTARs.A029	0.398	0.928	0.428
contig059766-BurTARs.A029	contig022354-TilTARs.A038	0.349	0.814	0.428
contig046013-NyeTARs.A021	contig022341-TilTARs.A032	0.137	0.319	0.428
contig022354-TilTARs.A038	contig045088-TilTARs.A057	0.364	0.850	0.428
contig084868-BriTARs.A014	contig039642-TilTAR.A004	0.397	0.926	0.428
contig007524-TilTARs.A026	contig022341-TilTARs.A032	0.348	0.812	0.428
contig084868-BriTARs.A014	contig049534-BurTARs.A025	0.279	0.651	0.428
contig058002-NyeTARs.A025	contig022363-TilTARs.A043	0.379	0.884	0.429
contig059673-BurTARs.A016	contig057301-BurTARs.A017	0.286	0.669	0.429
contig061091-BurTARs.A014	contig007512-TilTARs.A024	0.374	0.874	0.429
contig057301-BurTARs.A017	contig066285-ZebTARs.A016	0.384	0.896	0.429
contig057301-BurTARs.A017	contig022368-TilTARs.A046	0.370	0.862	0.429
contig058002-NyeTARs.A025	contig022368-TilTARs.A047	0.364	0.849	0.429
contig059673-BurTARs.A016	contig057305-BurTARs.A031	0.272	0.634	0.429
contig046007-NyeTARs.A017	contig022354-TilTARs.A038	0.133	0.309	0.429
contig045302-BurTAR.A001	contig039639-TilTAR.A001	0.064	0.148	0.429
contig046007-NyeTARs.A017	contig007524-TilTARs.A026	0.352	0.820	0.429
contig086351-BriTARs.A021	contig046010-NyeTARs.A019	0.416	0.970	0.429
contig022341-TilTARs.A032	contig056134-TilTARs.A058	0.356	0.830	0.429
contig035376-NyeTARs.A014	contig058002-NyeTARs.A025	0.308	0.719	0.429
contig022362-TilTARs.A042	contig022383-TilTARs.A054	0.266	0.620	0.429
contig060105-NyeTARs.A018	contig059768-ZebTARs.A022	0.348	0.811	0.429
contig049534-BurTARs.A025	contig062677-ZebTARs.A018	0.292	0.680	0.429
contig056023-BurTARs.A019	contig030464-ZebTARs.A025	0.187	0.436	0.429
contig022383-TilTARs.A054	contig066285-ZebTARs.A016	0.269	0.627	0.429
contig055697-BurTARs.A022	contig022356-TilTARs.A040	0.341	0.795	0.429
contig045302-BurTAR.A001	contig022383-TilTARs.A054	0.418	0.973	0.429
contig022363-TilTARs.A043	contig030445-ZebTARs.A026	0.264	0.615	0.429
contig022341-TilTARs.A032	contig061417-ZebTARs.A020	0.120	0.280	0.429
contig061091-BurTARs.A014	contig022345-TilTARs.A034	0.135	0.314	0.429
contig057148-BurTARs.A028	contig056200-NyeTARs.A029	0.277	0.645	0.429
contig035377-NyeTARs.A023	contig022320-TilTARs.A059	0.264	0.615	0.429
contig007518-TilTARs.A019	contig022349-TilTARs.A035	0.390	0.909	0.429
contig022365-TilTARs.A045	contig066056-ZebTARs.A017	0.344	0.801	0.429
contig022379-TilTARs.A052	contig066285-ZebTARs.A016	0.255	0.593	0.429
contig022334-TilTARs.A030	contig022354-TilTARs.A038	0.188	0.439	0.429
contig056200-NyeTARs.A029	contig066285-ZebTARs.A016	0.292	0.681	0.429
contig007520-TilTARs.A025	contig022354-TilTARs.A037	0.331	0.772	0.429
contig035375-NyeTARs.A013	contig066056-ZebTARs.A017	0.331	0.770	0.429
contig086337-BriTARs.A019	contig046013-NyeTARs.A021	0.361	0.841	0.429
contig057301-BurTARs.A017	contig030464-ZebTARs.A025	0.385	0.895	0.429
contig022356-TilTARs.A040	contig066330-ZebTARs.A027	0.361	0.839	0.430
contig022343-TilTARs.A033	contig066330-ZebTARs.A027	0.349	0.813	0.430
contig045302-BurTAR.A001	contig035376-NyeTARs.A014	0.402	0.937	0.430
contig060707-BurTARs.A015	contig035375-NyeTARs.A013	0.331	0.770	0.430
contig084886-BriTARs.A017	contig057145-BurTARs.A018	0.142	0.331	0.430
contig058002-NyeTARs.A025	contig045088-TilTARs.A057	0.273	0.634	0.430
contig082565-BriTARs.A022	contig045302-BurTAR.A001	0.392	0.912	0.430
contig084886-BriTARs.A017	contig022383-TilTARs.A054	0.259	0.603	0.430
contig022377-TilTARs.A050	contig056134-TilTARs.A058	0.346	0.805	0.430
contig034854-BurTARs.A027	contig059766-BurTARs.A029	0.289	0.672	0.430

contig022334-TilTARs.A030	contig022378-TilTARs.A051	0.233	0.541	0.430
contig084876-BriTARs.A015	contig059768-ZebTARs.A022	0.329	0.766	0.430
contig061410-ZebTARs.A021	contig030471-ZebTARs.A024	0.257	0.598	0.430
contig082565-BriTARs.A022	contig022349-TilTARs.A035	0.258	0.601	0.430
contig084868-BriTARs.A014	contig022368-TilTARs.A046	0.271	0.631	0.430
contig007518-TilTARs.A019	contig030464-ZebTARs.A025	0.382	0.890	0.430
contig086344-BriTARs.A020	contig022375-TilTARs.A048	0.345	0.801	0.430
contig058002-NyeTARs.A025	contig022341-TilTARs.A032	0.351	0.817	0.430
contig022337-TilTARs.A031	contig066056-ZebTARs.A017	0.350	0.814	0.430
contig057148-BurTARs.A028	contig035377-NyeTARs.A023	0.266	0.619	0.430
contig058002-NyeTARs.A025	contig022357-TilTARs.A041	0.368	0.855	0.430
contig056021-BurTARs.A020	contig022341-TilTARs.A032	0.123	0.287	0.430
contig086351-BriTARs.A021	contig058002-NyeTARs.A025	0.263	0.611	0.430
contig049540-BurTARs.A024	contig022378-TilTARs.A051	0.254	0.590	0.430
contig061091-BurTARs.A014	contig022375-TilTARs.A048	0.145	0.337	0.430
contig035381-NyeTARs.A015	contig061417-ZebTARs.A020	0.242	0.562	0.430
contig041024-BurTARs.A026	contig022363-TilTARs.A043	0.140	0.324	0.430
contig040586-ZebTAR.A001	contig030440-ZebTARs.A029	0.402	0.933	0.430
contig022354-TilTARs.A038	contig022368-TilTARs.A046	0.138	0.320	0.430
contig045302-BurTAR.A001	contig057301-BurTARs.A017	0.479	1.112	0.430
contig022343-TilTARs.A033	contig022320-TilTARs.A059	0.128	0.297	0.430
contig022337-TilTARs.A031	contig022377-TilTARs.A050	0.133	0.310	0.430
contig022330-TilTARs.A028	contig066691-ZebTARs.A015	0.121	0.282	0.430
contig060105-NyeTARs.A018	contig007518-TilTARs.A019	0.321	0.747	0.430
contig061977-BurTARs.A012	contig022362-TilTARs.A042	0.239	0.556	0.430
contig049540-BurTARs.A024	contig046010-NyeTARs.A019	0.278	0.646	0.430
contig056200-NyeTARs.A029	contig007512-TilTARs.A024	0.341	0.792	0.431
contig039640-TilTAR.A003	contig022390-TilTARs.A055	0.432	1.004	0.431
contig084880-BriTARs.A016	contig022375-TilTARs.A048	0.192	0.445	0.431
contig022330-TilTARs.A028	contig062677-ZebTARs.A018	0.259	0.601	0.431
contig084886-BriTARs.A017	contig046007-NyeTARs.A017	0.143	0.332	0.431
contig035381-NyeTARs.A015	contig030464-ZebTARs.A025	0.257	0.596	0.431
contig057301-BurTARs.A017	contig046010-NyeTARs.A019	0.383	0.890	0.431
contig060105-NyeTARs.A018	contig022334-TilTARs.A030	0.354	0.823	0.431
contig056200-NyeTARs.A029	contig022341-TilTARs.A032	0.276	0.641	0.431
contig022354-TilTARs.A037	contig022368-TilTARs.A047	0.185	0.429	0.431
contig065494-BurTARs.A030	contig022354-TilTARs.A038	0.351	0.814	0.431
contig022363-TilTARs.A044	contig022390-TilTARs.A055	0.258	0.598	0.431
contig007518-TilTARs.A019	contig022345-TilTARs.A034	0.366	0.850	0.431
contig056021-BurTARs.A020	contig056020-BurTARs.A021	0.148	0.343	0.431
contig066285-ZebTARs.A016	contig061410-ZebTARs.A021	0.259	0.602	0.431
contig007512-TilTARs.A024	contig022356-TilTARs.A040	0.340	0.789	0.431
contig084880-BriTARs.A016	contig022341-TilTARs.A032	0.183	0.425	0.431
contig086344-BriTARs.A020	contig022377-TilTARs.A050	0.320	0.742	0.431
contig056021-BurTARs.A020	contig035381-NyeTARs.A015	0.243	0.564	0.431
contig007524-TilTARs.A026	contig022355-TilTARs.A039	0.351	0.815	0.431
contig022334-TilTARs.A030	contig022368-TilTARs.A046	0.178	0.412	0.431
contig022349-TilTARs.A035	contig030445-ZebTARs.A026	0.249	0.576	0.431
contig061433-BurTARs.A013	contig007512-TilTARs.A024	0.351	0.815	0.431
contig056020-BurTARs.A021	contig007524-TilTARs.A026	0.354	0.820	0.431
contig084868-BriTARs.A014	contig022365-TilTARs.A045	0.283	0.656	0.431
contig007518-TilTARs.A019	contig040586-ZebTAR.A002	0.486	1.127	0.431
contig056021-BurTARs.A020	contig022382-TilTARs.A053	0.253	0.585	0.432

contig057305-BurTARs.A031	contig022383-TilTARs.A054	0.336	0.779	0.432
contig035377-NyeTARs.A023	contig022355-TilTARs.A039	0.276	0.640	0.432
contig035377-NyeTARs.A023	contig022390-TilTARs.A055	0.060	0.140	0.432
contig022354-TilTARs.A037	contig061417-ZebTARs.A020	0.162	0.374	0.432
contig082565-BriTARs.A022	contig066285-ZebTARs.A016	0.268	0.620	0.432
contig060707-BurTARs.A015	contig022356-TilTARs.A040	0.347	0.804	0.432
contig055697-BurTARs.A022	contig022337-TilTARs.A031	0.340	0.787	0.432
contig058002-NyeTARs.A025	contig022337-TilTARs.A031	0.358	0.830	0.432
contig022341-TilTARs.A032	contig022354-TilTARs.A038	0.121	0.279	0.432
contig022363-TilTARs.A044	contig022383-TilTARs.A054	0.236	0.547	0.432
contig022354-TilTARs.A038	contig022379-TilTARs.A052	0.238	0.551	0.432
contig022353-TilTARs.A036	contig022375-TilTARs.A048	0.136	0.314	0.432
contig035375-NyeTARs.A013	contig053145-ZebTARs.A028	0.284	0.658	0.432
contig030464-ZebTARs.A025	contig066330-ZebTARs.A027	0.357	0.827	0.432
contig056020-BurTARs.A021	contig060105-NyeTARs.A018	0.350	0.810	0.432
contig061091-BurTARs.A014	contig022320-TilTARs.A059	0.121	0.281	0.432
contig022379-TilTARs.A052	contig030440-ZebTARs.A029	0.134	0.309	0.432
contig022377-TilTARs.A050	contig022383-TilTARs.A054	0.254	0.587	0.432
contig084886-BriTARs.A017	contig045088-TilTARs.A057	0.367	0.849	0.432
contig056021-BurTARs.A020	contig022345-TilTARs.A034	0.121	0.279	0.432
contig084876-BriTARs.A015	contig059673-BurTARs.A016	0.334	0.773	0.432
contig035381-NyeTARs.A015	contig022330-TilTARs.A028	0.251	0.580	0.432
contig086344-BriTARs.A020	contig022334-TilTARs.A029	0.334	0.772	0.432
contig059766-BurTARs.A029	contig046010-NyeTARs.A019	0.358	0.828	0.432
contig084886-BriTARs.A017	contig022378-TilTARs.A051	0.250	0.579	0.432
contig061433-BurTARs.A013	contig022363-TilTARs.A044	0.201	0.465	0.432
contig042499-NyeTARs.A028	contig040586-ZebTAR.A001	0.497	1.148	0.432
contig061977-BurTARs.A012	contig061417-ZebTARs.A020	0.236	0.546	0.432
contig022334-TilTARs.A030	contig022390-TilTARs.A056	0.255	0.591	0.433
contig062677-ZebTARs.A018	contig061417-ZebTARs.A020	0.269	0.623	0.433
contig049534-BurTARs.A025	contig061410-ZebTARs.A021	0.259	0.599	0.433
contig038663-NyeTAR.A005	contig022383-TilTARs.A054	0.420	0.972	0.433
contig086344-BriTARs.A020	contig066691-ZebTARs.A015	0.351	0.811	0.433
contig038663-NyeTAR.A005	contig007518-TilTARs.A019	0.485	1.122	0.433
contig022375-TilTARs.A048	contig022379-TilTARs.A052	0.212	0.489	0.433
contig022356-TilTARs.A040	contig022379-TilTARs.A052	0.246	0.569	0.433
contig022341-TilTARs.A032	contig022345-TilTARs.A034	0.109	0.252	0.433
contig035381-NyeTARs.A015	contig022379-TilTARs.A052	0.129	0.299	0.433
contig035381-NyeTARs.A015	contig022354-TilTARs.A037	0.225	0.519	0.433
contig046013-NyeTARs.A021	contig007512-TilTARs.A024	0.370	0.856	0.433
contig022345-TilTARs.A034	contig030440-ZebTARs.A029	0.261	0.602	0.433
contig059673-BurTARs.A016	contig035381-NyeTARs.A015	0.324	0.748	0.433
contig030464-ZebTARs.A025	contig030440-ZebTARs.A029	0.261	0.603	0.433
contig022368-TilTARs.A046	contig061417-ZebTARs.A020	0.141	0.325	0.433
contig057148-BurTARs.A028	contig045999-NyeTARs.A026	0.185	0.427	0.433
contig022349-TilTARs.A035	contig061410-ZebTARs.A021	0.256	0.590	0.433
contig082565-BriTARs.A022	contig057145-BurTARs.A018	0.243	0.560	0.433
contig022382-TilTARs.A053	contig022383-TilTARs.A054	0.155	0.358	0.433
contig061091-BurTARs.A014	contig046010-NyeTARs.A019	0.158	0.365	0.433
contig059673-BurTARs.A016	contig045088-TilTARs.A057	0.285	0.657	0.433
contig084886-BriTARs.A017	contig061417-ZebTARs.A020	0.135	0.312	0.433
contig058002-NyeTARs.A025	contig022334-TilTARs.A030	0.337	0.779	0.433
contig084868-BriTARs.A014	contig022349-TilTARs.A035	0.282	0.652	0.433

contig034854-BurTARs.A027	contig040586-ZebTAR.A002	0.384	0.886	0.433
contig060707-BurTARs.A015	contig022363-TilTARs.A044	0.349	0.805	0.433
contig034854-BurTARs.A027	contig040586-ZebTAR.A001	0.394	0.909	0.433
contig022349-TilTARs.A035	contig022320-TilTARs.A059	0.141	0.326	0.433
contig049540-BurTARs.A024	contig022334-TilTARs.A030	0.240	0.553	0.434
contig038663-NyeTAR.A005	contig042499-NyeTARs.A028	0.498	1.149	0.434
contig084868-BriTARs.A014	contig040586-ZebTAR.A001	0.409	0.943	0.434
contig086344-BriTARs.A020	contig022356-TilTARs.A040	0.351	0.809	0.434
contig082565-BriTARs.A022	contig007518-TilTARs.A019	0.337	0.776	0.434
contig035377-NyeTARs.A023	contig066890-ZebTARs.A014	0.024	0.056	0.434
contig084886-BriTARs.A017	contig042499-NyeTARs.A028	0.361	0.833	0.434
contig042499-NyeTARs.A028	contig023443-TilTAR.A005	0.408	0.941	0.434
contig086344-BriTARs.A020	contig030440-ZebTARs.A029	0.339	0.782	0.434
contig022337-TilTARs.A031	contig030440-ZebTARs.A029	0.262	0.603	0.434
contig042499-NyeTARs.A028	contig022354-TilTARs.A038	0.368	0.847	0.434
contig060707-BurTARs.A015	contig022355-TilTARs.A039	0.336	0.773	0.434
contig082565-BriTARs.A022	contig057148-BurTARs.A028	0.256	0.590	0.434
contig029633-BriTAR.A003	contig035377-NyeTARs.A023	0.437	1.007	0.434
contig022334-TilTARs.A029	contig022354-TilTARs.A038	0.130	0.298	0.434
contig045999-NyeTARs.A026	contig022341-TilTARs.A032	0.182	0.420	0.434
contig082565-BriTARs.A022	contig022363-TilTARs.A043	0.277	0.637	0.434
contig056021-BurTARs.A020	contig066691-ZebTARs.A015	0.124	0.285	0.434
contig056021-BurTARs.A020	contig022343-TilTARs.A033	0.113	0.260	0.434
contig054630-BurTARs.A023	contig022330-TilTARs.A028	0.366	0.842	0.434
contig059766-BurTARs.A029	contig035375-NyeTARs.A013	0.286	0.659	0.434
contig029633-BriTAR.A003	contig022368-TilTARs.A047	0.447	1.029	0.434
contig065494-BurTARs.A030	contig007518-TilTARs.A019	0.300	0.692	0.434
contig022345-TilTARs.A034	contig061417-ZebTARs.A020	0.121	0.278	0.434
contig057148-BurTARs.A028	contig060292-NyeTARs.A027	0.408	0.939	0.434
contig029633-BriTAR.A003	contig022383-TilTARs.A054	0.420	0.966	0.434
contig057148-BurTARs.A028	contig007520-TilTARs.A025	0.347	0.799	0.434
contig038663-NyeTAR.A005	contig035375-NyeTARs.A013	0.406	0.935	0.435
contig022330-TilTARs.A028	contig022354-TilTARs.A038	0.128	0.294	0.435
contig061417-ZebTARs.A020	contig059768-ZebTARs.A022	0.370	0.852	0.435
contig040586-ZebTAR.A002	contig066890-ZebTARs.A014	0.422	0.970	0.435
contig046010-NyeTARs.A019	contig066691-ZebTARs.A015	0.157	0.362	0.435
contig022354-TilTARs.A038	contig022390-TilTARs.A056	0.254	0.584	0.435
contig049534-BurTARs.A025	contig022357-TilTARs.A041	0.160	0.369	0.435
contig041024-BurTARs.A026	contig022383-TilTARs.A054	0.247	0.569	0.435
contig059673-BurTARs.A016	contig022368-TilTARs.A046	0.361	0.832	0.435
contig061433-BurTARs.A013	contig007518-TilTARs.A019	0.369	0.850	0.435
contig049534-BurTARs.A025	contig035377-NyeTARs.A023	0.272	0.627	0.435
contig022365-TilTARs.A045	contig022379-TilTARs.A052	0.252	0.580	0.435
contig061977-BurTARs.A012	contig022349-TilTARs.A035	0.253	0.583	0.435
contig065494-BurTARs.A030	contig046010-NyeTARs.A019	0.360	0.828	0.435
contig022383-TilTARs.A054	contig022320-TilTARs.A059	0.247	0.567	0.435
contig084880-BriTARs.A016	contig022354-TilTARs.A038	0.183	0.422	0.435
contig022377-TilTARs.A050	contig062677-ZebTARs.A018	0.271	0.622	0.435
contig084886-BriTARs.A017	contig066691-ZebTARs.A015	0.156	0.359	0.435
contig022330-TilTARs.A028	contig022368-TilTARs.A047	0.149	0.341	0.435
contig054630-BurTARs.A023	contig061417-ZebTARs.A020	0.377	0.866	0.435
contig084868-BriTARs.A014	contig066285-ZebTARs.A016	0.278	0.639	0.435
contig056021-BurTARs.A020	contig056200-NyeTARs.A029	0.265	0.609	0.435

contig045999-NyeTARs.A026	contig030464-ZebTARs.A025	0.187	0.430	0.435
contig035381-NyeTARs.A015	contig022363-TilTARs.A043	0.275	0.631	0.435
contig035377-NyeTARs.A023	contig022362-TilTARs.A042	0.273	0.626	0.435
contig046014-NyeTARs.A024	contig022330-TilTARs.A028	0.116	0.266	0.435
contig022354-TilTARs.A038	contig022362-TilTARs.A042	0.145	0.332	0.435
contig042499-NyeTARs.A028	contig066285-ZebTARs.A016	0.391	0.898	0.435
contig062677-ZebTARs.A018	contig030464-ZebTARs.A025	0.283	0.650	0.436
contig056021-BurTARs.A020	contig058002-NyeTARs.A025	0.333	0.764	0.436
contig046010-NyeTARs.A019	contig046013-NyeTARs.A021	0.154	0.354	0.436
contig046013-NyeTARs.A021	contig007518-TilTARs.A019	0.374	0.859	0.436
contig082565-BriTARs.A022	contig022341-TilTARs.A032	0.253	0.581	0.436
contig041024-BurTARs.A026	contig022379-TilTARs.A052	0.240	0.551	0.436
contig056021-BurTARs.A020	contig066056-ZebTARs.A017	0.323	0.740	0.436
contig061433-BurTARs.A013	contig022354-TilTARs.A037	0.208	0.478	0.436
contig041024-BurTARs.A026	contig035377-NyeTARs.A023	0.261	0.600	0.436
contig022354-TilTARs.A037	contig022383-TilTARs.A054	0.225	0.517	0.436
contig060292-NyeTARs.A027	contig022383-TilTARs.A054	0.337	0.774	0.436
contig060707-BurTARs.A015	contig007518-TilTARs.A019	0.320	0.734	0.436
contig035377-NyeTARs.A023	contig022354-TilTARs.A038	0.257	0.589	0.436
contig062039-NyeTARs.A022	contig022390-TilTARs.A056	0.233	0.535	0.436
contig056021-BurTARs.A020	contig061410-ZebTARs.A021	0.237	0.544	0.436
contig061091-BurTARs.A014	contig022330-TilTARs.A028	0.118	0.271	0.436
contig084887-BriTARs.A018	contig022356-TilTARs.A040	0.118	0.270	0.436
contig045302-BurTAR.A001	contig035381-NyeTARs.A015	0.396	0.906	0.436
contig056021-BurTARs.A020	contig030471-ZebTARs.A024	0.121	0.277	0.436
contig022343-TilTARs.A033	contig061417-ZebTARs.A020	0.113	0.259	0.436
contig022330-TilTARs.A028	contig022341-TilTARs.A032	0.125	0.286	0.437
contig035376-NyeTARs.A014	contig022354-TilTARs.A038	0.242	0.555	0.437
contig046013-NyeTARs.A021	contig030471-ZebTARs.A024	0.075	0.171	0.437
contig007520-TilTARs.A025	contig022382-TilTARs.A053	0.316	0.724	0.437
contig035376-NyeTARs.A014	contig022349-TilTARs.A035	0.259	0.593	0.437
contig029633-BriTAR.A003	contig035381-NyeTARs.A015	0.399	0.914	0.437
contig056023-BurTARs.A019	contig057148-BurTARs.A028	0.189	0.434	0.437
contig065494-BurTARs.A030	contig022357-TilTARs.A041	0.392	0.898	0.437
contig086344-BriTARs.A020	contig034854-BurTARs.A027	0.349	0.799	0.437
contig056020-BurTARs.A021	contig022354-TilTARs.A038	0.128	0.294	0.437
contig022377-TilTARs.A050	contig030445-ZebTARs.A026	0.242	0.554	0.437
contig022357-TilTARs.A041	contig066056-ZebTARs.A017	0.339	0.776	0.437
contig022365-TilTARs.A045	contig022368-TilTARs.A046	0.090	0.206	0.437
contig057145-BurTARs.A018	contig030464-ZebTARs.A025	0.146	0.334	0.437
contig084876-BriTARs.A015	contig022330-TilTARs.A028	0.242	0.553	0.437
contig022354-TilTARs.A038	contig030445-ZebTARs.A026	0.234	0.536	0.437
contig084876-BriTARs.A015	contig054630-BurTARs.A023	0.335	0.767	0.437
contig022341-TilTARs.A032	contig022390-TilTARs.A056	0.258	0.590	0.437
contig056021-BurTARs.A020	contig007520-TilTARs.A025	0.325	0.743	0.437
contig084876-BriTARs.A015	contig038663-NyeTAR.A005	0.421	0.963	0.437
contig032272-NyeTAR.A004	contig045088-TilTARs.A057	0.410	0.937	0.437
contig049534-BurTARs.A025	contig035376-NyeTARs.A014	0.264	0.604	0.437
contig049534-BurTARs.A025	contig022330-TilTARs.A028	0.156	0.356	0.437
contig056020-BurTARs.A021	contig060292-NyeTARs.A027	0.403	0.921	0.437
contig045302-BurTAR.A001	contig046011-NyeTARs.A016	0.514	1.175	0.437
contig035377-NyeTARs.A023	contig022379-TilTARs.A052	0.159	0.364	0.437
contig035381-NyeTARs.A015	contig022343-TilTARs.A033	0.258	0.590	0.437

contig057145-BurTARs.A018	contig053139-ZebTARs.A023	0.348	0.795	0.437
contig061433-BurTARs.A013	contig056020-BurTARs.A021	0.195	0.445	0.438
contig057145-BurTARs.A018	contig035376-NyeTARs.A014	0.245	0.560	0.438
contig039642-TilTAR.A004	contig066890-ZebTARs.A014	0.413	0.944	0.438
contig007520-TilTARs.A025	contig030464-ZebTARs.A025	0.355	0.812	0.438
contig007520-TilTARs.A025	contig022334-TilTARs.A029	0.327	0.746	0.438
contig060707-BurTARs.A015	contig022377-TilTARs.A050	0.320	0.732	0.438
contig022390-TilTARs.A055	contig030440-ZebTARs.A029	0.080	0.183	0.438
contig007518-TilTARs.A019	contig022368-TilTARs.A046	0.365	0.835	0.438
contig084868-BriTARs.A014	contig041024-BurTARs.A026	0.259	0.592	0.438
contig049534-BurTARs.A025	contig007518-TilTARs.A019	0.383	0.875	0.438
contig022355-TilTARs.A039	contig066691-ZebTARs.A015	0.086	0.196	0.438
contig007520-TilTARs.A025	contig062677-ZebTARs.A018	0.333	0.760	0.438
contig060292-NyeTARs.A027	contig061410-ZebTARs.A021	0.317	0.723	0.438
contig035381-NyeTARs.A015	contig022334-TilTARs.A029	0.251	0.573	0.438
contig049540-BurTARs.A024	contig022330-TilTARs.A028	0.258	0.589	0.438
contig025313-BriTAR.A002	contig057301-BurTARs.A017	0.400	0.913	0.438
contig058002-NyeTARs.A025	contig066285-ZebTARs.A016	0.367	0.838	0.438
contig046007-NyeTARs.A017	contig022368-TilTARs.A046	0.142	0.323	0.438
contig022368-TilTARs.A046	contig061410-ZebTARs.A021	0.243	0.555	0.438
contig022368-TilTARs.A046	contig022375-TilTARs.A048	0.154	0.351	0.438
contig059766-BurTARs.A029	contig022382-TilTARs.A053	0.336	0.767	0.438
contig061091-BurTARs.A014	contig030464-ZebTARs.A025	0.160	0.364	0.438
contig046010-NyeTARs.A019	contig022390-TilTARs.A056	0.264	0.603	0.438
contig034854-BurTARs.A027	contig057148-BurTARs.A028	0.276	0.629	0.438
contig059766-BurTARs.A029	contig030464-ZebTARs.A025	0.363	0.827	0.438
contig035377-NyeTARs.A023	contig022377-TilTARs.A050	0.254	0.579	0.438
contig035376-NyeTARs.A014	contig022341-TilTARs.A032	0.252	0.574	0.438
contig084886-BriTARs.A017	contig022341-TilTARs.A032	0.135	0.308	0.438
contig086351-BriTARs.A021	contig042499-NyeTARs.A028	0.225	0.514	0.439
contig022349-TilTARs.A035	contig022383-TilTARs.A054	0.271	0.617	0.439
contig022330-TilTARs.A028	contig022375-TilTARs.A048	0.150	0.342	0.439
contig035381-NyeTARs.A015	contig046010-NyeTARs.A019	0.255	0.580	0.439
contig086344-BriTARs.A020	contig022330-TilTARs.A028	0.335	0.764	0.439
contig022353-TilTARs.A036	contig066056-ZebTARs.A017	0.314	0.716	0.439
contig061417-ZebTARs.A020	contig030471-ZebTARs.A024	0.121	0.276	0.439
contig022337-TilTARs.A031	contig022356-TilTARs.A040	0.133	0.303	0.439
contig057145-BurTARs.A018	contig022356-TilTARs.A040	0.039	0.090	0.439
contig049540-BurTARs.A024	contig057148-BurTARs.A028	0.280	0.637	0.439
contig022356-TilTARs.A040	contig053139-ZebTARs.A023	0.345	0.785	0.439
contig084880-BriTARs.A016	contig007518-TilTARs.A019	0.395	0.899	0.439
contig022382-TilTARs.A053	contig053145-ZebTARs.A028	0.336	0.766	0.439
contig035381-NyeTARs.A015	contig022337-TilTARs.A031	0.265	0.603	0.439
contig049540-BurTARs.A024	contig059766-BurTARs.A029	0.343	0.781	0.439
contig086337-BriTARs.A019	contig007518-TilTARs.A019	0.293	0.667	0.439
contig022341-TilTARs.A032	contig030445-ZebTARs.A026	0.246	0.561	0.439
contig038663-NyeTAR.A005	contig046011-NyeTARs.A016	0.521	1.186	0.439
contig057145-BurTARs.A018	contig022363-TilTARs.A044	0.160	0.365	0.439
contig058002-NyeTARs.A025	contig022365-TilTARs.A045	0.371	0.844	0.439
contig022379-TilTARs.A052	contig030464-ZebTARs.A025	0.257	0.585	0.439
contig084887-BriTARs.A018	contig060105-NyeTARs.A018	0.333	0.757	0.439
contig022362-TilTARs.A042	contig022377-TilTARs.A050	0.169	0.385	0.439
contig022330-TilTARs.A028	contig059768-ZebTARs.A022	0.359	0.818	0.439

contig084868-BriTARs.A014	contig022368-TilTARs.A047	0.284	0.647	0.439
contig022354-TilTARs.A038	contig066691-ZebTARs.A015	0.145	0.329	0.439
contig057305-BurTARs.A031	contig061410-ZebTARs.A021	0.316	0.718	0.439
contig022354-TilTARs.A037	contig022357-TilTARs.A041	0.188	0.427	0.439
contig022378-TilTARs.A051	contig062677-ZebTARs.A018	0.257	0.585	0.439
contig035381-NyeTARs.A015	contig007524-TilTARs.A026	0.319	0.726	0.440
contig086351-BriTARs.A021	contig062676-ZebTARs.A019	0.350	0.796	0.440
contig086344-BriTARs.A020	contig022363-TilTARs.A044	0.355	0.807	0.440
contig062039-NyeTARs.A022	contig022379-TilTARs.A052	0.195	0.445	0.440
contig060105-NyeTARs.A018	contig030440-ZebTARs.A029	0.335	0.761	0.440
contig061433-BurTARs.A013	contig054630-BurTARs.A023	0.423	0.963	0.440
contig022362-TilTARs.A042	contig066056-ZebTARs.A017	0.348	0.791	0.440
contig082565-BriTARs.A022	contig040586-ZebTAR.A001	0.395	0.898	0.440
contig022354-TilTARs.A038	contig022355-TilTARs.A039	0.125	0.285	0.440
contig035377-NyeTARs.A023	contig022334-TilTARs.A029	0.257	0.583	0.440
contig056021-BurTARs.A020	contig022368-TilTARs.A047	0.148	0.337	0.440
contig022362-TilTARs.A042	contig056134-TilTARs.A058	0.333	0.757	0.440
contig046013-NyeTARs.A021	contig022334-TilTARs.A029	0.117	0.265	0.440
contig049540-BurTARs.A024	contig053145-ZebTARs.A028	0.343	0.779	0.440
contig022365-TilTARs.A045	contig022375-TilTARs.A048	0.160	0.365	0.440
contig060105-NyeTARs.A018	contig066691-ZebTARs.A015	0.348	0.791	0.440
contig022343-TilTARs.A033	contig022375-TilTARs.A048	0.157	0.357	0.440
contig022390-TilTARs.A056	contig040586-ZebTAR.A002	0.416	0.945	0.440
contig049540-BurTARs.A024	contig061417-ZebTARs.A020	0.268	0.610	0.440
contig060707-BurTARs.A015	contig046007-NyeTARs.A017	0.357	0.812	0.440
contig061091-BurTARs.A014	contig056021-BurTARs.A020	0.120	0.274	0.440
contig022337-TilTARs.A031	contig053139-ZebTARs.A023	0.343	0.779	0.440
contig086344-BriTARs.A020	contig035375-NyeTARs.A013	0.341	0.774	0.440
contig022324-TilTARs.A027	contig056134-TilTARs.A058	0.377	0.857	0.440
contig022355-TilTARs.A039	contig062677-ZebTARs.A018	0.280	0.637	0.440
contig029633-BriTAR.A003	contig084880-BriTARs.A016	0.475	1.078	0.440
contig049534-BurTARs.A025	contig022379-TilTARs.A052	0.257	0.583	0.440
contig022354-TilTARs.A038	contig062676-ZebTARs.A019	0.240	0.544	0.441
contig035381-NyeTARs.A015	contig022363-TilTARs.A044	0.231	0.523	0.441
contig060105-NyeTARs.A018	contig066890-ZebTARs.A014	0.333	0.756	0.441
contig057148-BurTARs.A028	contig057305-BurTARs.A031	0.408	0.926	0.441
contig056020-BurTARs.A021	contig046013-NyeTARs.A021	0.083	0.189	0.441
contig065494-BurTARs.A030	contig022382-TilTARs.A053	0.341	0.775	0.441
contig022330-TilTARs.A028	contig022379-TilTARs.A052	0.235	0.534	0.441
contig022390-TilTARs.A055	contig061417-ZebTARs.A020	0.213	0.483	0.441
contig062039-NyeTARs.A022	contig022354-TilTARs.A038	0.256	0.580	0.441
contig065494-BurTARs.A030	contig030464-ZebTARs.A025	0.365	0.828	0.441
contig084886-BriTARs.A017	contig061091-BurTARs.A014	0.153	0.347	0.441
contig022343-TilTARs.A033	contig022345-TilTARs.A034	0.134	0.304	0.441
contig049534-BurTARs.A025	contig056200-NyeTARs.A029	0.292	0.662	0.441
contig049540-BurTARs.A024	contig049534-BurTARs.A025	0.292	0.662	0.441
contig046013-NyeTARs.A021	contig022355-TilTARs.A039	0.086	0.195	0.441
contig061977-BurTARs.A012	contig022357-TilTARs.A041	0.262	0.594	0.441
contig035377-NyeTARs.A023	contig022363-TilTARs.A044	0.247	0.560	0.441
contig022362-TilTARs.A042	contig061410-ZebTARs.A021	0.242	0.548	0.441
contig025313-BriTAR.A002	contig045088-TilTARs.A057	0.400	0.907	0.441
contig045302-BurTAR.A001	contig034854-BurTARs.A027	0.389	0.882	0.441
contig082565-BriTARs.A022	contig059673-BurTARs.A016	0.334	0.756	0.441

contig061091-BurTARs.A014	contig057148-BurTARs.A028	0.162	0.367	0.441
contig007518-TilTARs.A019	contig022337-TilTARs.A031	0.391	0.885	0.442
contig022377-TilTARs.A050	contig045088-TilTARs.A057	0.363	0.823	0.442
contig022356-TilTARs.A040	contig030471-ZebTARs.A024	0.124	0.280	0.442
contig022341-TilTARs.A032	contig022382-TilTARs.A053	0.269	0.609	0.442
contig059673-BurTARs.A016	contig022355-TilTARs.A039	0.364	0.824	0.442
contig059673-BurTARs.A016	contig057145-BurTARs.A018	0.360	0.814	0.442
contig022357-TilTARs.A041	contig066330-ZebTARs.A027	0.395	0.893	0.442
contig022362-TilTARs.A042	contig066285-ZebTARs.A016	0.189	0.428	0.442
contig086344-BriTARs.A020	contig046013-NyeTARs.A021	0.350	0.791	0.442
contig022334-TilTARs.A030	contig030464-ZebTARs.A025	0.199	0.451	0.442
contig022368-TilTARs.A047	contig061417-ZebTARs.A020	0.145	0.329	0.442
contig057145-BurTARs.A018	contig055697-BurTARs.A022	0.351	0.795	0.442
contig057305-BurTARs.A031	contig022378-TilTARs.A051	0.365	0.825	0.442
contig022362-TilTARs.A042	contig062677-ZebTARs.A018	0.268	0.606	0.442
contig046010-NyeTARs.A019	contig060292-NyeTARs.A027	0.409	0.924	0.442
contig007520-TilTARs.A025	contig022363-TilTARs.A044	0.354	0.801	0.442
contig060707-BurTARs.A015	contig030471-ZebTARs.A024	0.344	0.778	0.442
contig057301-BurTARs.A017	contig032272-NyeTAR.A004	0.415	0.940	0.442
contig049534-BurTARs.A025	contig022353-TilTARs.A036	0.167	0.377	0.442
contig086351-BriTARs.A021	contig045088-TilTARs.A057	0.229	0.519	0.442
contig084886-BriTARs.A017	contig022368-TilTARs.A047	0.151	0.341	0.442
contig082565-BriTARs.A022	contig061977-BurTARs.A012	0.129	0.291	0.442
contig084887-BriTARs.A018	contig022365-TilTARs.A045	0.141	0.319	0.442
contig022334-TilTARs.A029	contig062677-ZebTARs.A018	0.259	0.585	0.442
contig046014-NyeTARs.A024	contig022363-TilTARs.A043	0.138	0.312	0.442
contig056023-BurTARs.A019	contig022390-TilTARs.A055	0.327	0.740	0.442
contig057148-BurTARs.A028	contig058002-NyeTARs.A025	0.349	0.788	0.442
contig061091-BurTARs.A014	contig061417-ZebTARs.A020	0.121	0.273	0.442
contig041024-BurTARs.A026	contig030445-ZebTARs.A026	0.220	0.497	0.443
contig057301-BurTARs.A017	contig003909-ZebTAR.A003	0.410	0.927	0.443
contig029633-BriTAR.A003	contig034854-BurTARs.A027	0.392	0.885	0.443
contig034854-BurTARs.A027	contig060105-NyeTARs.A018	0.344	0.778	0.443
contig045302-BurTAR.A001	contig007518-TilTARs.A019	0.469	1.059	0.443
contig046014-NyeTARs.A024	contig022362-TilTARs.A042	0.133	0.300	0.443
contig022354-TilTARs.A038	contig022378-TilTARs.A051	0.240	0.543	0.443
contig061977-BurTARs.A012	contig060292-NyeTARs.A027	0.333	0.751	0.443
contig049540-BurTARs.A024	contig007520-TilTARs.A025	0.331	0.748	0.443
contig046013-NyeTARs.A021	contig030464-ZebTARs.A025	0.157	0.355	0.443
contig022334-TilTARs.A030	contig062677-ZebTARs.A018	0.242	0.546	0.443
contig035375-NyeTARs.A013	contig022345-TilTARs.A034	0.265	0.599	0.443
contig058002-NyeTARs.A025	contig042499-NyeTARs.A028	0.276	0.622	0.443
contig035376-NyeTARs.A014	contig066285-ZebTARs.A016	0.264	0.596	0.443
contig049540-BurTARs.A024	contig065494-BurTARs.A030	0.348	0.786	0.443
contig022382-TilTARs.A053	contig030464-ZebTARs.A025	0.281	0.634	0.443
contig057301-BurTARs.A017	contig007524-TilTARs.A026	0.280	0.631	0.443
contig022368-TilTARs.A047	contig022383-TilTARs.A054	0.272	0.614	0.443
contig057305-BurTARs.A031	contig007512-TilTARs.A024	0.292	0.658	0.443
contig056200-NyeTARs.A029	contig030464-ZebTARs.A025	0.282	0.637	0.443
contig029633-BriTAR.A003	contig084868-BriTARs.A014	0.417	0.941	0.443
contig022349-TilTARs.A035	contig062676-ZebTARs.A019	0.257	0.580	0.443
contig022343-TilTARs.A033	contig022356-TilTARs.A040	0.127	0.287	0.443
contig061091-BurTARs.A014	contig007520-TilTARs.A025	0.358	0.807	0.443

contig084886-BriTARs.A017	contig046013-NyeTARs.A021	0.158	0.357	0.443
contig046007-NyeTARs.A017	contig060105-NyeTARs.A018	0.357	0.806	0.443
contig022355-TilTARs.A039	contig022375-TilTARs.A048	0.150	0.337	0.443
contig045999-NyeTARs.A026	contig022375-TilTARs.A048	0.198	0.447	0.444
contig056020-BurTARs.A021	contig057305-BurTARs.A031	0.403	0.908	0.444
contig062039-NyeTARs.A022	contig045088-TilTARs.A057	0.342	0.770	0.444
contig086337-BriTARs.A019	contig022337-TilTARs.A031	0.346	0.779	0.444
contig056200-NyeTARs.A029	contig022334-TilTARs.A030	0.242	0.545	0.444
contig035377-NyeTARs.A023	contig061410-ZebTARs.A021	0.157	0.354	0.444
contig084868-BriTARs.A014	contig040586-ZebTAR.A002	0.405	0.913	0.444
contig060105-NyeTARs.A018	contig022356-TilTARs.A040	0.353	0.796	0.444
contig035377-NyeTARs.A023	contig022330-TilTARs.A028	0.260	0.585	0.444
contig038663-NyeTAR.A005	contig030440-ZebTARs.A029	0.409	0.921	0.444
contig022324-TilTARs.A027	contig062676-ZebTARs.A019	0.259	0.585	0.444
contig045088-TilTARs.A057	contig003909-ZebTAR.A003	0.411	0.925	0.444
contig056021-BurTARs.A020	contig022365-TilTARs.A045	0.152	0.342	0.444
contig022357-TilTARs.A041	contig066285-ZebTARs.A016	0.165	0.372	0.444
contig034854-BurTARs.A027	contig038663-NyeTAR.A005	0.400	0.901	0.444
contig084880-BriTARs.A016	contig066056-ZebTARs.A017	0.374	0.844	0.444
contig022365-TilTARs.A045	contig061417-ZebTARs.A020	0.148	0.334	0.444
contig059673-BurTARs.A016	contig022341-TilTARs.A032	0.357	0.805	0.444
contig057148-BurTARs.A028	contig022354-TilTARs.A037	0.189	0.426	0.444
contig022334-TilTARs.A029	contig022368-TilTARs.A047	0.147	0.330	0.444
contig038663-NyeTAR.A005	contig030445-ZebTARs.A026	0.396	0.892	0.444
contig057145-BurTARs.A018	contig049540-BurTARs.A024	0.277	0.625	0.444
contig022330-TilTARs.A028	contig022365-TilTARs.A045	0.145	0.327	0.444
contig084886-BriTARs.A017	contig022355-TilTARs.A039	0.137	0.308	0.444
contig061977-BurTARs.A012	contig057305-BurTARs.A031	0.331	0.746	0.444
contig057145-BurTARs.A018	contig062676-ZebTARs.A019	0.251	0.565	0.444
contig038663-NyeTAR.A005	contig035376-NyeTARs.A014	0.412	0.927	0.444
contig061091-BurTARs.A014	contig060105-NyeTARs.A018	0.346	0.779	0.444
contig061091-BurTARs.A014	contig022341-TilTARs.A032	0.130	0.292	0.444
contig041024-BurTARs.A026	contig007520-TilTARs.A025	0.323	0.727	0.444
contig007518-TilTARs.A019	contig066890-ZebTARs.A014	0.342	0.770	0.445
contig022330-TilTARs.A028	contig022377-TilTARs.A050	0.141	0.318	0.445
contig060707-BurTARs.A015	contig022368-TilTARs.A046	0.346	0.779	0.445
contig041024-BurTARs.A026	contig035381-NyeTARs.A015	0.227	0.511	0.445
contig022354-TilTARs.A037	contig030445-ZebTARs.A026	0.221	0.497	0.445
contig046010-NyeTARs.A019	contig030440-ZebTARs.A029	0.261	0.587	0.445
contig042499-NyeTARs.A028	contig022363-TilTARs.A043	0.408	0.919	0.445
contig058002-NyeTARs.A025	contig061417-ZebTARs.A020	0.333	0.749	0.445
contig060292-NyeTARs.A027	contig022378-TilTARs.A051	0.367	0.825	0.445
contig057148-BurTARs.A028	contig035375-NyeTARs.A013	0.270	0.607	0.445
contig084887-BriTARs.A018	contig057145-BurTARs.A018	0.124	0.278	0.445
contig029633-BriTAR.A003	contig022390-TilTARs.A056	0.432	0.970	0.445
contig022343-TilTARs.A033	contig022357-TilTARs.A041	0.129	0.289	0.445
contig029633-BriTAR.A003	contig022345-TilTARs.A034	0.432	0.970	0.445
contig061977-BurTARs.A012	contig022337-TilTARs.A031	0.256	0.575	0.445
contig007524-TilTARs.A026	contig045088-TilTARs.A057	0.275	0.618	0.445
contig057145-BurTARs.A018	contig022349-TilTARs.A035	0.151	0.340	0.445
contig007524-TilTARs.A026	contig061410-ZebTARs.A021	0.314	0.706	0.445
contig022356-TilTARs.A040	contig030445-ZebTARs.A026	0.237	0.534	0.445
contig057301-BurTARs.A017	contig062039-NyeTARs.A022	0.348	0.781	0.445

contig057305-BurTARs.A031	contig007524-TilTARs.A026	0.261	0.587	0.445
contig062677-ZebTARs.A018	contig062676-ZebTARs.A019	0.166	0.374	0.445
contig007518-TilTARs.A019	contig022354-TilTARs.A038	0.375	0.842	0.445
contig022324-TilTARs.A027	contig022383-TilTARs.A054	0.264	0.593	0.445
contig022357-TilTARs.A041	contig022363-TilTARs.A044	0.188	0.423	0.445
contig062039-NyeTARs.A022	contig058002-NyeTARs.A025	0.325	0.730	0.445
contig022362-TilTARs.A042	contig062676-ZebTARs.A019	0.261	0.587	0.445
contig029633-BriTAR.A003	contig066890-ZebTARs.A014	0.437	0.982	0.445
contig086344-BriTARs.A020	contig007518-TilTARs.A019	0.332	0.746	0.445
contig022363-TilTARs.A043	contig066285-ZebTARs.A016	0.215	0.482	0.445
contig007518-TilTARs.A019	contig030445-ZebTARs.A026	0.329	0.738	0.445
contig056021-BurTARs.A020	contig022379-TilTARs.A052	0.239	0.536	0.445
contig056200-NyeTARs.A029	contig022378-TilTARs.A051	0.256	0.574	0.446
contig035377-NyeTARs.A023	contig040586-ZebTAR.A001	0.424	0.952	0.446
contig084876-BriTARs.A015	contig046014-NyeTARs.A024	0.253	0.568	0.446
contig058002-NyeTARs.A025	contig030464-ZebTARs.A025	0.357	0.800	0.446
contig082565-BriTARs.A022	contig022377-TilTARs.A050	0.249	0.558	0.446
contig035375-NyeTARs.A013	contig007512-TilTARs.A024	0.296	0.664	0.446
contig084880-BriTARs.A016	contig057145-BurTARs.A018	0.195	0.438	0.446
contig022375-TilTARs.A048	contig022390-TilTARs.A055	0.276	0.619	0.446
contig022349-TilTARs.A035	contig022365-TilTARs.A045	0.166	0.372	0.446
contig059673-BurTARs.A016	contig042499-NyeTARs.A028	0.289	0.648	0.446
contig022353-TilTARs.A036	contig022356-TilTARs.A040	0.113	0.253	0.446
contig084876-BriTARs.A015	contig030445-ZebTARs.A026	0.135	0.302	0.446
contig060105-NyeTARs.A018	contig022355-TilTARs.A039	0.341	0.765	0.446
contig060707-BurTARs.A015	contig022365-TilTARs.A045	0.355	0.795	0.446
contig049540-BurTARs.A024	contig030464-ZebTARs.A025	0.280	0.628	0.446
contig061977-BurTARs.A012	contig022363-TilTARs.A044	0.223	0.500	0.446
contig035375-NyeTARs.A013	contig060105-NyeTARs.A018	0.336	0.754	0.446
contig046014-NyeTARs.A024	contig022379-TilTARs.A052	0.245	0.549	0.446
contig060292-NyeTARs.A027	contig022363-TilTARs.A044	0.403	0.903	0.446
contig022334-TilTARs.A029	contig022375-TilTARs.A048	0.149	0.334	0.446
contig057301-BurTARs.A017	contig007520-TilTARs.A025	0.276	0.619	0.446
contig007524-TilTARs.A026	contig022356-TilTARs.A040	0.351	0.786	0.446
contig022334-TilTARs.A029	contig022357-TilTARs.A041	0.131	0.293	0.446
contig060292-NyeTARs.A027	contig007512-TilTARs.A024	0.290	0.649	0.446
contig032272-NyeTAR.A004	contig042499-NyeTARs.A028	0.414	0.928	0.446
contig046007-NyeTARs.A017	contig066691-ZebTARs.A015	0.090	0.201	0.446
contig022341-TilTARs.A032	contig066691-ZebTARs.A015	0.133	0.298	0.447
contig057301-BurTARs.A017	contig058002-NyeTARs.A025	0.288	0.645	0.447
contig022349-TilTARs.A035	contig022379-TilTARs.A052	0.262	0.586	0.447
contig058002-NyeTARs.A025	contig022349-TilTARs.A035	0.358	0.803	0.447
contig086344-BriTARs.A020	contig030471-ZebTARs.A024	0.352	0.789	0.447
contig084876-BriTARs.A015	contig035381-NyeTARs.A015	0.140	0.313	0.447
contig041024-BurTARs.A026	contig022363-TilTARs.A044	0.153	0.344	0.447
contig035377-NyeTARs.A023	contig046014-NyeTARs.A024	0.264	0.591	0.447
contig035381-NyeTARs.A015	contig040586-ZebTAR.A001	0.399	0.892	0.447
contig086351-BriTARs.A021	contig057301-BurTARs.A017	0.229	0.513	0.447
contig049540-BurTARs.A024	contig066330-ZebTARs.A027	0.350	0.784	0.447
contig022368-TilTARs.A047	contig030471-ZebTARs.A024	0.147	0.329	0.447
contig086351-BriTARs.A021	contig022334-TilTARs.A030	0.394	0.881	0.447
contig041024-BurTARs.A026	contig058002-NyeTARs.A025	0.326	0.730	0.447
contig046010-NyeTARs.A019	contig062676-ZebTARs.A019	0.252	0.563	0.447

contig022334-TilTARs.A029	contig022379-TilTARs.A052	0.236	0.527	0.447
contig049534-BurTARs.A025	contig062676-ZebTARs.A019	0.263	0.588	0.447
contig022320-TilTARs.A059	contig062676-ZebTARs.A019	0.244	0.545	0.447
contig066691-ZebTARs.A015	contig030464-ZebTARs.A025	0.163	0.365	0.447
contig082565-BriTARs.A022	contig007524-TilTARs.A026	0.328	0.734	0.447
contig022363-TilTARs.A043	contig045088-TilTARs.A057	0.408	0.911	0.447
contig022356-TilTARs.A040	contig030440-ZebTARs.A029	0.265	0.592	0.447
contig029633-BriTAR.A003	contig007518-TilTARs.A019	0.496	1.110	0.447
contig049540-BurTARs.A024	contig022377-TilTARs.A050	0.271	0.605	0.447
contig007524-TilTARs.A026	contig022365-TilTARs.A045	0.375	0.838	0.448
contig035377-NyeTARs.A023	contig066691-ZebTARs.A015	0.278	0.620	0.448
contig060292-NyeTARs.A027	contig045088-TilTARs.A057	0.226	0.504	0.448
contig056200-NyeTARs.A029	contig022330-TilTARs.A028	0.260	0.581	0.448
contig084876-BriTARs.A015	contig041024-BurTARs.A026	0.248	0.555	0.448
contig022349-TilTARs.A035	contig030440-ZebTARs.A029	0.268	0.599	0.448
contig060292-NyeTARs.A027	contig042499-NyeTARs.A028	0.223	0.499	0.448
contig022390-TilTARs.A056	contig066056-ZebTARs.A017	0.335	0.747	0.448
contig059766-BurTARs.A029	contig062677-ZebTARs.A018	0.345	0.771	0.448
contig035381-NyeTARs.A015	contig007518-TilTARs.A019	0.330	0.737	0.448
contig039642-TilTAR.A004	contig022390-TilTARs.A055	0.447	0.999	0.448
contig084876-BriTARs.A015	contig022334-TilTARs.A029	0.243	0.542	0.448
contig084886-BriTARs.A017	contig057301-BurTARs.A017	0.366	0.818	0.448
contig061417-ZebTARs.A020	contig061410-ZebTARs.A021	0.239	0.535	0.448
contig057145-BurTARs.A018	contig056020-BurTARs.A021	0.152	0.340	0.448
contig022365-TilTARs.A045	contig066890-ZebTARs.A014	0.276	0.616	0.448
contig022379-TilTARs.A052	contig030445-ZebTARs.A026	0.124	0.278	0.448
contig058002-NyeTARs.A025	contig022378-TilTARs.A051	0.336	0.751	0.448
contig057301-BurTARs.A017	contig022354-TilTARs.A038	0.373	0.832	0.448
contig045302-BurTAR.A001	contig035377-NyeTARs.A023	0.423	0.945	0.448
contig035377-NyeTARs.A023	contig022343-TilTARs.A033	0.267	0.595	0.448
contig060292-NyeTARs.A027	contig007524-TilTARs.A026	0.259	0.579	0.448
contig049540-BurTARs.A024	contig022355-TilTARs.A039	0.279	0.623	0.448
contig086351-BriTARs.A021	contig007520-TilTARs.A025	0.267	0.596	0.448
contig045999-NyeTARs.A026	contig039642-TilTAR.A004	0.467	1.041	0.448
contig061977-BurTARs.A012	contig062677-ZebTARs.A018	0.162	0.362	0.448
contig022368-TilTARs.A047	contig066285-ZebTARs.A016	0.161	0.360	0.448
contig035381-NyeTARs.A015	contig022362-TilTARs.A042	0.262	0.585	0.448
contig007520-TilTARs.A025	contig022375-TilTARs.A048	0.334	0.746	0.448
contig061977-BurTARs.A012	contig035376-NyeTARs.A014	0.130	0.289	0.448
contig055697-BurTARs.A022	contig007518-TilTARs.A019	0.302	0.673	0.448
contig060105-NyeTARs.A018	contig022363-TilTARs.A044	0.356	0.793	0.448
contig057305-BurTARs.A031	contig045088-TilTARs.A057	0.229	0.512	0.448
contig022379-TilTARs.A052	contig059768-ZebTARs.A022	0.339	0.755	0.449
contig022354-TilTARs.A038	contig022375-TilTARs.A048	0.152	0.338	0.449
contig007518-TilTARs.A019	contig030471-ZebTARs.A024	0.382	0.852	0.449
contig057305-BurTARs.A031	contig042499-NyeTARs.A028	0.227	0.507	0.449
contig060105-NyeTARs.A018	contig022354-TilTARs.A037	0.348	0.776	0.449
contig025313-BriTAR.A002	contig042499-NyeTARs.A028	0.404	0.902	0.449
contig062677-ZebTARs.A018	contig053145-ZebTARs.A028	0.345	0.769	0.449
contig084880-BriTARs.A016	contig022320-TilTARs.A059	0.171	0.382	0.449
contig045302-BurTAR.A001	contig042499-NyeTARs.A028	0.484	1.080	0.449
contig046007-NyeTARs.A017	contig022375-TilTARs.A048	0.154	0.343	0.449
contig059673-BurTARs.A016	contig035377-NyeTARs.A023	0.348	0.776	0.449

contig022365-TilTARs.A045	contig022383-TilTARs.A054	0.274	0.609	0.449
contig062039-NyeTARs.A022	contig007524-TilTARs.A026	0.329	0.734	0.449
contig022382-TilTARs.A053	contig066330-ZebTARs.A027	0.346	0.770	0.449
contig086337-BriTARs.A019	contig057301-BurTARs.A017	0.289	0.643	0.449
contig056023-BurTARs.A019	contig007518-TilTARs.A019	0.399	0.888	0.449
contig022356-TilTARs.A040	contig022383-TilTARs.A054	0.260	0.578	0.449
contig084868-BriTARs.A014	contig045302-BurTAR.A001	0.420	0.936	0.449
contig034854-BurTARs.A027	contig065494-BurTARs.A030	0.292	0.650	0.449
contig022382-TilTARs.A053	contig061417-ZebTARs.A020	0.260	0.578	0.449
contig022337-TilTARs.A031	contig053145-ZebTARs.A028	0.355	0.790	0.449
contig045999-NyeTARs.A026	contig053145-ZebTARs.A028	0.407	0.905	0.449
contig057148-BurTARs.A028	contig066890-ZebTARs.A014	0.271	0.602	0.449
contig022337-TilTARs.A031	contig061410-ZebTARs.A021	0.258	0.575	0.449
contig060105-NyeTARs.A018	contig046013-NyeTARs.A021	0.347	0.772	0.449
contig022354-TilTARs.A038	contig066890-ZebTARs.A014	0.259	0.577	0.449
contig056020-BurTARs.A021	contig022343-TilTARs.A033	0.115	0.256	0.449
contig022390-TilTARs.A056	contig040586-ZebTAR.A001	0.426	0.947	0.449
contig056200-NyeTARs.A029	contig061417-ZebTARs.A020	0.270	0.602	0.449
contig057145-BurTARs.A018	contig007512-TilTARs.A024	0.355	0.789	0.450
contig007520-TilTARs.A025	contig022349-TilTARs.A035	0.352	0.783	0.450
contig084876-BriTARs.A015	contig035377-NyeTARs.A023	0.164	0.364	0.450
contig034854-BurTARs.A027	contig066330-ZebTARs.A027	0.293	0.651	0.450
contig086344-BriTARs.A020	contig022334-TilTARs.A030	0.356	0.792	0.450
contig061410-ZebTARs.A021	contig030445-ZebTARs.A026	0.125	0.279	0.450
contig046011-NyeTARs.A016	contig056134-TilTARs.A058	0.375	0.833	0.450
contig060707-BurTARs.A015	contig022337-TilTARs.A031	0.364	0.808	0.450
contig046013-NyeTARs.A021	contig046014-NyeTARs.A024	0.115	0.257	0.450
contig022330-TilTARs.A028	contig022382-TilTARs.A053	0.249	0.553	0.450
contig007520-TilTARs.A025	contig045088-TilTARs.A057	0.275	0.611	0.450
contig035381-NyeTARs.A015	contig062676-ZebTARs.A019	0.105	0.234	0.450
contig060105-NyeTARs.A018	contig022377-TilTARs.A050	0.326	0.724	0.450
contig057305-BurTARs.A031	contig022365-TilTARs.A045	0.425	0.944	0.450
contig022357-TilTARs.A041	contig062677-ZebTARs.A018	0.293	0.652	0.450
contig061433-BurTARs.A013	contig060105-NyeTARs.A018	0.347	0.771	0.450
contig056200-NyeTARs.A029	contig007520-TilTARs.A025	0.333	0.739	0.450
contig059673-BurTARs.A016	contig022379-TilTARs.A052	0.337	0.750	0.450
contig022330-TilTARs.A028	contig066285-ZebTARs.A016	0.160	0.355	0.450
contig057145-BurTARs.A018	contig062677-ZebTARs.A018	0.280	0.622	0.450
contig062676-ZebTARs.A019	contig030464-ZebTARs.A025	0.259	0.576	0.450
contig022390-TilTARs.A056	contig030464-ZebTARs.A025	0.272	0.603	0.450
contig082565-BriTARs.A022	contig022354-TilTARs.A038	0.246	0.546	0.450
contig084868-BriTARs.A014	contig046014-NyeTARs.A024	0.262	0.582	0.450
contig022337-TilTARs.A031	contig022349-TilTARs.A035	0.152	0.337	0.450
contig007520-TilTARs.A025	contig022353-TilTARs.A036	0.382	0.848	0.450
contig086344-BriTARs.A020	contig022365-TilTARs.A045	0.363	0.807	0.450
contig022320-TilTARs.A059	contig030445-ZebTARs.A026	0.226	0.503	0.450
contig007520-TilTARs.A025	contig022356-TilTARs.A040	0.341	0.758	0.451
contig030464-ZebTARs.A025	contig030445-ZebTARs.A026	0.247	0.549	0.451
contig049540-BurTARs.A024	contig045088-TilTARs.A057	0.363	0.806	0.451
contig056021-BurTARs.A020	contig046014-NyeTARs.A024	0.112	0.248	0.451
contig035381-NyeTARs.A015	contig061410-ZebTARs.A021	0.130	0.289	0.451
contig061417-ZebTARs.A020	contig030445-ZebTARs.A026	0.236	0.524	0.451
contig034854-BurTARs.A027	contig022354-TilTARs.A038	0.264	0.586	0.451

contig086344-BriTARs.A020	contig022355-TilTARs.A039	0.344	0.763	0.451
contig022349-TilTARs.A035	contig066890-ZebTARs.A014	0.283	0.627	0.451
contig061433-BurTARs.A013	contig022363-TilTARs.A043	0.211	0.469	0.451
contig060292-NyeTARs.A027	contig062676-ZebTARs.A019	0.339	0.752	0.451
contig038663-NyeTAR.A005	contig039640-TilTAR.A003	0.051	0.114	0.451
contig061977-BurTARs.A012	contig061433-BurTARs.A013	0.262	0.582	0.451
contig007524-TilTARs.A026	contig022345-TilTARs.A034	0.332	0.737	0.451
contig082565-BriTARs.A022	contig022343-TilTARs.A033	0.257	0.570	0.451
contig061433-BurTARs.A013	contig059768-ZebTARs.A022	0.421	0.934	0.451
contig057305-BurTARs.A031	contig022363-TilTARs.A044	0.402	0.892	0.451
contig022354-TilTARs.A037	contig022354-TilTARs.A038	0.182	0.403	0.451
contig022343-TilTARs.A033	contig022383-TilTARs.A054	0.265	0.587	0.451
contig007524-TilTARs.A026	contig022334-TilTARs.A030	0.339	0.752	0.451
contig059673-BurTARs.A016	contig022356-TilTARs.A040	0.360	0.798	0.451
contig086351-BriTARs.A021	contig007512-TilTARs.A024	0.287	0.635	0.451
contig061091-BurTARs.A014	contig022365-TilTARs.A045	0.145	0.322	0.451
contig057305-BurTARs.A031	contig022368-TilTARs.A047	0.421	0.933	0.451
contig060292-NyeTARs.A027	contig022368-TilTARs.A047	0.421	0.933	0.451
contig062677-ZebTARs.A018	contig061410-ZebTARs.A021	0.170	0.376	0.451
contig022357-TilTARs.A041	contig061410-ZebTARs.A021	0.264	0.585	0.451
contig056021-BurTARs.A020	contig030445-ZebTARs.A026	0.237	0.526	0.452
contig007518-TilTARs.A019	contig022362-TilTARs.A042	0.381	0.844	0.452
contig084868-BriTARs.A014	contig084880-BriTARs.A016	0.290	0.642	0.452
contig056020-BurTARs.A021	contig022390-TilTARs.A055	0.242	0.536	0.452
contig065494-BurTARs.A030	contig062677-ZebTARs.A018	0.351	0.776	0.452
contig035377-NyeTARs.A023	contig022375-TilTARs.A048	0.243	0.538	0.452
contig084868-BriTARs.A014	contig061433-BurTARs.A013	0.285	0.630	0.452
contig022379-TilTARs.A052	contig022382-TilTARs.A053	0.162	0.359	0.452
contig057145-BurTARs.A018	contig056200-NyeTARs.A029	0.279	0.618	0.452
contig029633-BriTAR.A003	contig039642-TilTAR.A004	0.031	0.070	0.452
contig022378-TilTARs.A051	contig062676-ZebTARs.A019	0.226	0.499	0.452
contig007520-TilTARs.A025	contig061417-ZebTARs.A020	0.330	0.729	0.452
contig035381-NyeTARs.A015	contig022320-TilTARs.A059	0.234	0.517	0.452
contig086344-BriTARs.A020	contig022368-TilTARs.A046	0.357	0.790	0.452
contig084876-BriTARs.A015	contig084886-BriTARs.A017	0.268	0.593	0.452
contig007518-TilTARs.A019	contig022365-TilTARs.A045	0.388	0.859	0.452
contig056200-NyeTARs.A029	contig022377-TilTARs.A050	0.272	0.601	0.452
contig035376-NyeTARs.A014	contig022377-TilTARs.A050	0.249	0.551	0.452
contig059673-BurTARs.A016	contig022354-TilTARs.A038	0.359	0.794	0.452
contig022343-TilTARs.A033	contig062677-ZebTARs.A018	0.285	0.631	0.452
contig022343-TilTARs.A033	contig056134-TilTARs.A058	0.349	0.771	0.452
contig007524-TilTARs.A026	contig022354-TilTARs.A038	0.352	0.779	0.452
contig007518-TilTARs.A019	contig066330-ZebTARs.A027	0.307	0.680	0.452
contig056021-BurTARs.A020	contig022349-TilTARs.A035	0.151	0.334	0.452
contig045999-NyeTARs.A026	contig040586-ZebTAR.A002	0.470	1.039	0.452
contig041024-BurTARs.A026	contig022353-TilTARs.A036	0.121	0.267	0.452
contig022337-TilTARs.A031	contig056134-TilTARs.A058	0.372	0.822	0.452
contig022330-TilTARs.A028	contig022362-TilTARs.A042	0.144	0.319	0.452
contig061433-BurTARs.A013	contig022353-TilTARs.A036	0.177	0.391	0.453
contig045999-NyeTARs.A026	contig003909-ZebTAR.A003	0.439	0.970	0.453
contig022345-TilTARs.A034	contig022362-TilTARs.A042	0.142	0.315	0.453
contig062039-NyeTARs.A022	contig042499-NyeTARs.A028	0.343	0.757	0.453
contig022320-TilTARs.A059	contig066890-ZebTARs.A014	0.266	0.587	0.453

contig057305-BurTARs.A031	contig035381-NyeTARs.A015	0.355	0.785	0.453
contig061433-BurTARs.A013	contig022375-TilTARs.A048	0.197	0.434	0.453
contig022330-TilTARs.A028	contig030445-ZebTARs.A026	0.245	0.541	0.453
contig025313-BriTAR.A002	contig045999-NyeTARs.A026	0.430	0.950	0.453
contig007520-TilTARs.A025	contig022320-TilTARs.A059	0.329	0.726	0.453
contig022362-TilTARs.A042	contig022382-TilTARs.A053	0.267	0.590	0.453
contig084868-BriTARs.A014	contig038663-NyeTAR.A005	0.423	0.934	0.453
contig084876-BriTARs.A015	contig007524-TilTARs.A026	0.325	0.717	0.453
contig046014-NyeTARs.A024	contig022357-TilTARs.A041	0.139	0.306	0.453
contig066285-ZebTARs.A016	contig062676-ZebTARs.A019	0.263	0.581	0.453
contig035376-NyeTARs.A014	contig030464-ZebTARs.A025	0.258	0.569	0.453
contig042499-NyeTARs.A028	contig003909-ZebTAR.A003	0.415	0.916	0.453
contig022365-TilTARs.A045	contig062676-ZebTARs.A019	0.265	0.585	0.453
contig046013-NyeTARs.A021	contig022377-TilTARs.A050	0.151	0.333	0.453
contig084880-BriTARs.A016	contig084886-BriTARs.A017	0.193	0.426	0.453
contig022365-TilTARs.A045	contig030471-ZebTARs.A024	0.147	0.323	0.453
contig022337-TilTARs.A031	contig022383-TilTARs.A054	0.277	0.611	0.453
contig049540-BurTARs.A024	contig022357-TilTARs.A041	0.293	0.645	0.453
contig061091-BurTARs.A014	contig022354-TilTARs.A038	0.145	0.320	0.453
contig022357-TilTARs.A041	contig022320-TilTARs.A059	0.137	0.301	0.453
contig022341-TilTARs.A032	contig022363-TilTARs.A043	0.161	0.355	0.454
contig022357-TilTARs.A041	contig022368-TilTARs.A046	0.149	0.328	0.454
contig022324-TilTARs.A027	contig022337-TilTARs.A031	0.173	0.382	0.454
contig022334-TilTARs.A029	contig022365-TilTARs.A045	0.144	0.317	0.454
contig059766-BurTARs.A029	contig045999-NyeTARs.A026	0.411	0.907	0.454
contig022368-TilTARs.A046	contig062676-ZebTARs.A019	0.256	0.564	0.454
contig057305-BurTARs.A031	contig007518-TilTARs.A019	0.282	0.621	0.454
contig084868-BriTARs.A014	contig022355-TilTARs.A039	0.278	0.614	0.454
contig046011-NyeTARs.A016	contig022345-TilTARs.A034	0.237	0.521	0.454
contig007518-TilTARs.A019	contig022334-TilTARs.A030	0.360	0.794	0.454
contig035377-NyeTARs.A023	contig007524-TilTARs.A026	0.337	0.744	0.454
contig066691-ZebTARs.A015	contig061417-ZebTARs.A020	0.124	0.273	0.454
contig022354-TilTARs.A037	contig066691-ZebTARs.A015	0.174	0.383	0.454
contig084868-BriTARs.A014	contig022356-TilTARs.A040	0.266	0.585	0.454
contig045302-BurTAR.A001	contig039640-TilTAR.A002	0.072	0.159	0.454
contig057301-BurTARs.A017	contig022363-TilTARs.A043	0.416	0.917	0.454
contig065494-BurTARs.A030	contig035375-NyeTARs.A013	0.289	0.637	0.454
contig061091-BurTARs.A014	contig035377-NyeTARs.A023	0.274	0.603	0.454
contig057148-BurTARs.A028	contig066691-ZebTARs.A015	0.161	0.355	0.454
contig035376-NyeTARs.A014	contig007518-TilTARs.A019	0.341	0.750	0.454
contig059766-BurTARs.A029	contig056200-NyeTARs.A029	0.345	0.760	0.454
contig022354-TilTARs.A037	contig066056-ZebTARs.A017	0.332	0.732	0.454
contig007524-TilTARs.A026	contig022368-TilTARs.A046	0.344	0.758	0.454
contig007512-TilTARs.A024	contig022337-TilTARs.A031	0.369	0.812	0.454
contig059766-BurTARs.A029	contig022337-TilTARs.A031	0.359	0.791	0.454
contig084886-BriTARs.A017	contig062677-ZebTARs.A018	0.280	0.615	0.454
contig007520-TilTARs.A025	contig066691-ZebTARs.A015	0.362	0.798	0.454
contig056020-BurTARs.A021	contig022345-TilTARs.A034	0.133	0.293	0.454
contig022354-TilTARs.A037	contig022363-TilTARs.A044	0.127	0.280	0.454
contig086337-BriTARs.A019	contig042499-NyeTARs.A028	0.285	0.626	0.454
contig061433-BurTARs.A013	contig061410-ZebTARs.A021	0.261	0.574	0.454
contig007518-TilTARs.A019	contig022343-TilTARs.A033	0.385	0.846	0.455
contig084868-BriTARs.A014	contig062677-ZebTARs.A018	0.183	0.402	0.455

contig084876-BriTARs.A015	contig022379-TilTARs.A052	0.021	0.046	0.455
contig060707-BurTARs.A015	contig022390-TilTARs.A056	0.335	0.737	0.455
contig007524-TilTARs.A026	contig022378-TilTARs.A051	0.338	0.744	0.455
contig056023-BurTARs.A019	contig039642-TilTAR.A004	0.467	1.026	0.455
contig056200-NyeTARs.A029	contig053145-ZebTARs.A028	0.345	0.759	0.455
contig084876-BriTARs.A015	contig066285-ZebTARs.A016	0.275	0.605	0.455
contig022390-TilTARs.A056	contig022320-TilTARs.A059	0.261	0.574	0.455
contig065494-BurTARs.A030	contig022337-TilTARs.A031	0.363	0.797	0.455
contig082565-BriTARs.A022	contig035381-NyeTARs.A015	0.020	0.044	0.455
contig045999-NyeTARs.A026	contig007518-TilTARs.A019	0.397	0.873	0.455
contig007524-TilTARs.A026	contig022379-TilTARs.A052	0.310	0.681	0.455
contig049540-BurTARs.A024	contig022334-TilTARs.A029	0.259	0.569	0.455
contig007518-TilTARs.A019	contig022356-TilTARs.A040	0.379	0.833	0.455
contig059673-BurTARs.A016	contig030445-ZebTARs.A026	0.326	0.717	0.455
contig040586-ZebTAR.A001	contig066890-ZebTARs.A014	0.428	0.939	0.455
contig060292-NyeTARs.A027	contig007518-TilTARs.A019	0.281	0.617	0.455
contig061977-BurTARs.A012	contig022343-TilTARs.A033	0.248	0.545	0.455
contig082565-BriTARs.A022	contig022378-TilTARs.A051	0.220	0.484	0.455
contig060105-NyeTARs.A018	contig022368-TilTARs.A046	0.354	0.777	0.455
contig057305-BurTARs.A031	contig046010-NyeTARs.A019	0.415	0.911	0.455
contig086344-BriTARs.A020	contig022390-TilTARs.A056	0.338	0.743	0.455
contig007524-TilTARs.A026	contig022362-TilTARs.A042	0.343	0.753	0.455
contig057145-BurTARs.A018	contig046013-NyeTARs.A021	0.131	0.288	0.455
contig057148-BurTARs.A028	contig046013-NyeTARs.A021	0.158	0.347	0.455
contig022334-TilTARs.A029	contig022341-TilTARs.A032	0.128	0.282	0.455
contig007518-TilTARs.A019	contig007520-TilTARs.A025	0.255	0.561	0.455
contig049540-BurTARs.A024	contig022343-TilTARs.A033	0.283	0.622	0.455
contig060707-BurTARs.A015	contig056020-BurTARs.A021	0.382	0.839	0.455
contig056021-BurTARs.A020	contig059768-ZebTARs.A022	0.368	0.808	0.455
contig022324-TilTARs.A027	contig022343-TilTARs.A033	0.164	0.361	0.455
contig062677-ZebTARs.A018	contig066330-ZebTARs.A027	0.352	0.774	0.456
contig022334-TilTARs.A030	contig022363-TilTARs.A044	0.136	0.298	0.456
contig045088-TilTARs.A057	contig062677-ZebTARs.A018	0.369	0.809	0.456
contig084887-BriTARs.A018	contig035377-NyeTARs.A023	0.277	0.607	0.456
contig084876-BriTARs.A015	contig022349-TilTARs.A035	0.266	0.584	0.456
contig022324-TilTARs.A027	contig022378-TilTARs.A051	0.265	0.582	0.456
contig057145-BurTARs.A018	contig046011-NyeTARs.A016	0.242	0.531	0.456
contig022337-TilTARs.A031	contig061417-ZebTARs.A020	0.122	0.267	0.456
contig054630-BurTARs.A023	contig022379-TilTARs.A052	0.345	0.757	0.456
contig035375-NyeTARs.A013	contig022337-TilTARs.A031	0.270	0.593	0.456
contig046011-NyeTARs.A016	contig022357-TilTARs.A041	0.245	0.537	0.456
contig022320-TilTARs.A059	contig030471-ZebTARs.A024	0.066	0.144	0.456
contig049540-BurTARs.A024	contig058002-NyeTARs.A025	0.334	0.732	0.456
contig082565-BriTARs.A022	contig022337-TilTARs.A031	0.266	0.583	0.456
contig056023-BurTARs.A019	contig022375-TilTARs.A048	0.199	0.437	0.456
contig041024-BurTARs.A026	contig046013-NyeTARs.A021	0.119	0.261	0.456
contig084876-BriTARs.A015	contig056021-BurTARs.A020	0.248	0.544	0.456
contig022330-TilTARs.A028	contig022357-TilTARs.A041	0.136	0.298	0.456
contig084880-BriTARs.A016	contig053145-ZebTARs.A028	0.404	0.885	0.456
contig056021-BurTARs.A020	contig054630-BurTARs.A023	0.375	0.821	0.456
contig058002-NyeTARs.A025	contig022382-TilTARs.A053	0.322	0.705	0.456
contig035377-NyeTARs.A023	contig030464-ZebTARs.A025	0.274	0.600	0.456
contig084886-BriTARs.A017	contig022362-TilTARs.A042	0.166	0.363	0.456

contig049540-BurTARs.A024	contig022362-TilTARs.A042	0.268	0.588	0.456
contig084876-BriTARs.A015	contig057305-BurTARs.A031	0.334	0.733	0.456
contig022330-TilTARs.A028	contig022363-TilTARs.A043	0.170	0.371	0.456
contig022379-TilTARs.A052	contig022320-TilTARs.A059	0.243	0.533	0.457
contig046010-NyeTARs.A019	contig035377-NyeTARs.A023	0.267	0.584	0.457
contig082565-BriTARs.A022	contig038663-NyeTAR.A005	0.401	0.879	0.457
contig086351-BriTARs.A021	contig022363-TilTARs.A044	0.407	0.892	0.457
contig046013-NyeTARs.A021	contig022320-TilTARs.A059	0.124	0.272	0.457
contig020038-BurTAR.A002	contig057301-BurTARs.A017	0.412	0.902	0.457
contig035375-NyeTARs.A013	contig066330-ZebTARs.A027	0.290	0.636	0.457
contig056021-BurTARs.A020	contig022383-TilTARs.A054	0.257	0.564	0.457
contig022368-TilTARs.A047	contig022377-TilTARs.A050	0.144	0.315	0.457
contig057301-BurTARs.A017	contig022377-TilTARs.A050	0.368	0.806	0.457
contig058002-NyeTARs.A025	contig022353-TilTARs.A036	0.383	0.839	0.457
contig061977-BurTARs.A012	contig022377-TilTARs.A050	0.243	0.531	0.457
contig061433-BurTARs.A013	contig022390-TilTARs.A055	0.340	0.744	0.457
contig022345-TilTARs.A034	contig022355-TilTARs.A039	0.126	0.276	0.457
contig086344-BriTARs.A020	contig046007-NyeTARs.A017	0.366	0.801	0.457
contig056023-BurTARs.A019	contig022341-TilTARs.A032	0.186	0.407	0.457
contig049534-BurTARs.A025	contig060292-NyeTARs.A027	0.446	0.976	0.457
contig035376-NyeTARs.A014	contig022356-TilTARs.A040	0.253	0.553	0.457
contig046013-NyeTARs.A021	contig022343-TilTARs.A033	0.107	0.233	0.457
contig057145-BurTARs.A018	contig022382-TilTARs.A053	0.269	0.590	0.457
contig060707-BurTARs.A015	contig022354-TilTARs.A037	0.340	0.743	0.457
contig066056-ZebTARs.A017	contig061417-ZebTARs.A020	0.329	0.719	0.457
contig084868-BriTARs.A014	contig084886-BriTARs.A017	0.275	0.601	0.457
contig045302-BurTAR.A001	contig022390-TilTARs.A056	0.429	0.939	0.457
contig035381-NyeTARs.A015	contig060292-NyeTARs.A027	0.356	0.779	0.457
contig022363-TilTARs.A044	contig061410-ZebTARs.A021	0.226	0.493	0.457
contig084876-BriTARs.A015	contig022320-TilTARs.A059	0.251	0.548	0.457
contig035377-NyeTARs.A023	contig022324-TilTARs.A027	0.281	0.614	0.457
contig039642-TilTAR.A004	contig040586-ZebTAR.A001	0.063	0.137	0.457
contig084876-BriTARs.A015	contig022368-TilTARs.A047	0.264	0.578	0.457
contig082565-BriTARs.A022	contig022356-TilTARs.A040	0.251	0.549	0.457
contig046010-NyeTARs.A019	contig022354-TilTARs.A037	0.193	0.421	0.457
contig056200-NyeTARs.A029	contig022355-TilTARs.A039	0.281	0.615	0.457
contig061977-BurTARs.A012	contig022324-TilTARs.A027	0.255	0.557	0.457
contig045302-BurTAR.A001	contig066890-ZebTARs.A014	0.426	0.932	0.457
contig029633-BriTAR.A003	contig040586-ZebTAR.A002	0.020	0.043	0.458
contig058002-NyeTARs.A025	contig022354-TilTARs.A037	0.335	0.732	0.458
contig022363-TilTARs.A043	contig022368-TilTARs.A047	0.124	0.271	0.458
contig007524-TilTARs.A026	contig022337-TilTARs.A031	0.359	0.784	0.458
contig022365-TilTARs.A045	contig066691-ZebTARs.A015	0.148	0.324	0.458
contig057145-BurTARs.A018	contig035375-NyeTARs.A013	0.274	0.599	0.458
contig034854-BurTARs.A027	contig007520-TilTARs.A025	0.345	0.753	0.458
contig038663-NyeTAR.A005	contig039642-TilTAR.A004	0.063	0.137	0.458
contig049534-BurTARs.A025	contig022382-TilTARs.A053	0.293	0.639	0.458
contig057145-BurTARs.A018	contig007518-TilTARs.A019	0.381	0.831	0.458
contig061433-BurTARs.A013	contig022383-TilTARs.A054	0.271	0.593	0.458
contig007524-TilTARs.A026	contig030445-ZebTARs.A026	0.319	0.697	0.458
contig049534-BurTARs.A025	contig022390-TilTARs.A056	0.272	0.595	0.458
contig065494-BurTARs.A030	contig056200-NyeTARs.A029	0.351	0.765	0.458
contig057301-BurTARs.A017	contig062677-ZebTARs.A018	0.365	0.796	0.458

contig022379-TilTARs.A052	contig062677-ZebTARs.A018	0.173	0.377	0.458
contig042499-NyeTARs.A028	contig022362-TilTARs.A042	0.360	0.786	0.458
contig084880-BriTARs.A016	contig022354-TilTARs.A037	0.225	0.490	0.458
contig022382-TilTARs.A053	contig066285-ZebTARs.A016	0.293	0.640	0.458
contig022379-TilTARs.A052	contig061417-ZebTARs.A020	0.241	0.526	0.458
contig058002-NyeTARs.A025	contig022320-TilTARs.A059	0.334	0.728	0.458
contig084887-BriTARs.A018	contig007520-TilTARs.A025	0.352	0.769	0.458
contig055697-BurTARs.A022	contig045088-TilTARs.A057	0.283	0.618	0.458
contig039640-TilTAR.A002	contig040586-ZebTAR.A001	0.069	0.151	0.458
contig060105-NyeTARs.A018	contig022365-TilTARs.A045	0.361	0.787	0.458
contig041024-BurTARs.A026	contig062039-NyeTARs.A022	0.253	0.553	0.458
contig084876-BriTARs.A015	contig062039-NyeTARs.A022	0.210	0.458	0.458
contig057305-BurTARs.A031	contig062676-ZebTARs.A019	0.338	0.737	0.459
contig022362-TilTARs.A042	contig045088-TilTARs.A057	0.363	0.790	0.459
contig007518-TilTARs.A019	contig022330-TilTARs.A028	0.385	0.839	0.459
contig057301-BurTARs.A017	contig022362-TilTARs.A042	0.365	0.796	0.459
contig061433-BurTARs.A013	contig022334-TilTARs.A029	0.160	0.348	0.459
contig022357-TilTARs.A041	contig022365-TilTARs.A045	0.146	0.319	0.459
contig046007-NyeTARs.A017	contig022343-TilTARs.A033	0.119	0.260	0.459
contig022363-TilTARs.A044	contig022377-TilTARs.A050	0.182	0.398	0.459
contig035381-NyeTARs.A015	contig022375-TilTARs.A048	0.216	0.471	0.459
contig086351-BriTARs.A021	contig022368-TilTARs.A047	0.429	0.934	0.459
contig022334-TilTARs.A029	contig030445-ZebTARs.A026	0.245	0.534	0.459
contig061977-BurTARs.A012	contig030440-ZebTARs.A029	0.132	0.288	0.459
contig084880-BriTARs.A016	contig022345-TilTARs.A034	0.187	0.408	0.459
contig060707-BurTARs.A015	contig042499-NyeTARs.A028	0.317	0.692	0.459
contig086351-BriTARs.A021	contig022378-TilTARs.A051	0.366	0.797	0.459
contig062039-NyeTARs.A022	contig066285-ZebTARs.A016	0.264	0.576	0.459
contig042499-NyeTARs.A028	contig066691-ZebTARs.A015	0.354	0.770	0.459
contig060105-NyeTARs.A018	contig022337-TilTARs.A031	0.367	0.800	0.459
contig046010-NyeTARs.A019	contig030445-ZebTARs.A026	0.245	0.534	0.459
contig020038-BurTAR.A002	contig045088-TilTARs.A057	0.412	0.898	0.459
contig060292-NyeTARs.A027	contig022365-TilTARs.A045	0.428	0.931	0.459
contig056023-BurTARs.A019	contig040586-ZebTAR.A002	0.470	1.024	0.459
contig049534-BurTARs.A025	contig066890-ZebTARs.A014	0.282	0.614	0.459
contig056021-BurTARs.A020	contig046013-NyeTARs.A021	0.126	0.275	0.459
contig056023-BurTARs.A019	contig053145-ZebTARs.A028	0.404	0.880	0.459
contig022377-TilTARs.A050	contig022390-TilTARs.A055	0.232	0.506	0.459
contig049534-BurTARs.A025	contig035375-NyeTARs.A013	0.292	0.636	0.459
contig032272-NyeTAR.A004	contig045999-NyeTARs.A026	0.439	0.956	0.459
contig035376-NyeTARs.A014	contig022343-TilTARs.A033	0.257	0.558	0.460
contig084880-BriTARs.A016	contig059673-BurTARs.A016	0.392	0.853	0.460
contig022334-TilTARs.A029	contig022343-TilTARs.A033	0.107	0.233	0.460
contig056021-BurTARs.A020	contig022377-TilTARs.A050	0.143	0.312	0.460
contig059673-BurTARs.A016	contig035376-NyeTARs.A014	0.332	0.723	0.460
contig007520-TilTARs.A025	contig022377-TilTARs.A050	0.349	0.759	0.460
contig022355-TilTARs.A039	contig022383-TilTARs.A054	0.269	0.586	0.460
contig035376-NyeTARs.A014	contig022337-TilTARs.A031	0.264	0.575	0.460
contig086351-BriTARs.A021	contig061410-ZebTARs.A021	0.323	0.703	0.460
contig022337-TilTARs.A031	contig062677-ZebTARs.A018	0.298	0.648	0.460
contig022383-TilTARs.A054	contig030445-ZebTARs.A026	0.109	0.238	0.460
contig056200-NyeTARs.A029	contig022362-TilTARs.A042	0.270	0.588	0.460
contig049540-BurTARs.A024	contig062676-ZebTARs.A019	0.166	0.362	0.460

contig022377-TilTARs.A050	contig062676-ZebTARs.A019	0.245	0.534	0.460
contig046014-NyeTARs.A024	contig022383-TilTARs.A054	0.250	0.544	0.460
contig062039-NyeTARs.A022	contig022354-TilTARs.A037	0.235	0.512	0.460
contig084868-BriTARs.A014	contig022354-TilTARs.A037	0.241	0.523	0.460
contig082565-BriTARs.A022	contig030464-ZebTARs.A025	0.258	0.560	0.460
contig056200-NyeTARs.A029	contig022334-TilTARs.A029	0.260	0.565	0.460
contig084868-BriTARs.A014	contig045999-NyeTARs.A026	0.290	0.631	0.460
contig056200-NyeTARs.A029	contig045088-TilTARs.A057	0.361	0.785	0.460
contig049534-BurTARs.A025	contig022363-TilTARs.A043	0.217	0.471	0.460
contig059673-BurTARs.A016	contig066890-ZebTARs.A014	0.350	0.760	0.460
contig022378-TilTARs.A051	contig022382-TilTARs.A053	0.245	0.532	0.460
contig056020-BurTARs.A021	contig022362-TilTARs.A042	0.154	0.335	0.460
contig035381-NyeTARs.A015	contig022378-TilTARs.A051	0.217	0.471	0.460
contig061091-BurTARs.A014	contig022354-TilTARs.A037	0.170	0.370	0.460
contig059673-BurTARs.A016	contig022377-TilTARs.A050	0.362	0.786	0.460
contig022337-TilTARs.A031	contig066330-ZebTARs.A027	0.365	0.793	0.460
contig084887-BriTARs.A018	contig042499-NyeTARs.A028	0.342	0.743	0.460
contig042499-NyeTARs.A028	contig007524-TilTARs.A026	0.282	0.612	0.460
contig082565-BriTARs.A022	contig045088-TilTARs.A057	0.351	0.762	0.460
contig060707-BurTARs.A015	contig022357-TilTARs.A041	0.353	0.766	0.461
contig084868-BriTARs.A014	contig022378-TilTARs.A051	0.239	0.520	0.461
contig084868-BriTARs.A014	contig049540-BurTARs.A024	0.184	0.399	0.461
contig007518-TilTARs.A019	contig022363-TilTARs.A044	0.369	0.801	0.461
contig045999-NyeTARs.A026	contig022377-TilTARs.A050	0.166	0.361	0.461
contig022368-TilTARs.A047	contig022379-TilTARs.A052	0.256	0.556	0.461
contig041024-BurTARs.A026	contig062676-ZebTARs.A019	0.247	0.537	0.461
contig035381-NyeTARs.A015	contig022355-TilTARs.A039	0.255	0.554	0.461
contig046007-NyeTARs.A017	contig046013-NyeTARs.A021	0.090	0.195	0.461
contig086337-BriTARs.A019	contig045088-TilTARs.A057	0.289	0.626	0.461
contig038663-NyeTAR.A005	contig035377-NyeTARs.A023	0.432	0.938	0.461
contig022368-TilTARs.A046	contig030471-ZebTARs.A024	0.128	0.278	0.461
contig086344-BriTARs.A020	contig022357-TilTARs.A041	0.356	0.772	0.461
contig041024-BurTARs.A026	contig022349-TilTARs.A035	0.140	0.303	0.461
contig061091-BurTARs.A014	contig042499-NyeTARs.A028	0.349	0.756	0.461
contig084876-BriTARs.A015	contig060292-NyeTARs.A027	0.336	0.728	0.461
contig022324-TilTARs.A027	contig061410-ZebTARs.A021	0.253	0.549	0.461
contig035381-NyeTARs.A015	contig022368-TilTARs.A047	0.270	0.586	0.461
contig084887-BriTARs.A018	contig022368-TilTARs.A047	0.155	0.337	0.461
contig035377-NyeTARs.A023	contig022363-TilTARs.A043	0.324	0.703	0.461
contig056200-NyeTARs.A029	contig022357-TilTARs.A041	0.294	0.638	0.461
contig084880-BriTARs.A016	contig035377-NyeTARs.A023	0.283	0.613	0.461
contig007524-TilTARs.A026	contig022354-TilTARs.A037	0.335	0.725	0.461
contig022349-TilTARs.A035	contig061417-ZebTARs.A020	0.152	0.330	0.461
contig057301-BurTARs.A017	contig049540-BurTARs.A024	0.366	0.792	0.461
contig061091-BurTARs.A014	contig058002-NyeTARs.A025	0.359	0.778	0.462
contig062039-NyeTARs.A022	contig022345-TilTARs.A034	0.255	0.552	0.462
contig045999-NyeTARs.A026	contig022354-TilTARs.A037	0.224	0.485	0.462
contig057301-BurTARs.A017	contig060292-NyeTARs.A027	0.230	0.499	0.462
contig049534-BurTARs.A025	contig030440-ZebTARs.A029	0.283	0.613	0.462
contig035376-NyeTARs.A014	contig046010-NyeTARs.A019	0.256	0.554	0.462
contig084876-BriTARs.A015	contig022368-TilTARs.A046	0.253	0.549	0.462
contig022363-TilTARs.A044	contig066890-ZebTARs.A014	0.240	0.520	0.462
contig034854-BurTARs.A027	contig022379-TilTARs.A052	0.154	0.335	0.462

contig022368-TilTARs.A047	contig045088-TilTARs.A057	0.393	0.851	0.462
contig022365-TilTARs.A045	contig022390-TilTARs.A056	0.268	0.581	0.462
contig084886-BriTARs.A017	contig022365-TilTARs.A045	0.152	0.329	0.462
contig056200-NyeTARs.A029	contig066330-ZebTARs.A027	0.352	0.763	0.462
contig022330-TilTARs.A028	contig022343-TilTARs.A033	0.117	0.253	0.462
contig084868-BriTARs.A014	contig022343-TilTARs.A033	0.271	0.586	0.462
contig045088-TilTARs.A057	contig066691-ZebTARs.A015	0.359	0.776	0.462
contig022334-TilTARs.A030	contig022365-TilTARs.A045	0.191	0.414	0.462
contig058002-NyeTARs.A025	contig022356-TilTARs.A040	0.347	0.750	0.462
contig007518-TilTARs.A019	contig022390-TilTARs.A056	0.351	0.760	0.462
contig057301-BurTARs.A017	contig057305-BurTARs.A031	0.234	0.506	0.462
contig046014-NyeTARs.A024	contig061417-ZebTARs.A020	0.112	0.242	0.463
contig022354-TilTARs.A037	contig022365-TilTARs.A045	0.178	0.385	0.463
contig082565-BriTARs.A022	contig022330-TilTARs.A028	0.256	0.553	0.463
contig041024-BurTARs.A026	contig045999-NyeTARs.A026	0.174	0.376	0.463
contig049540-BurTARs.A024	contig042499-NyeTARs.A028	0.364	0.786	0.463
contig022334-TilTARs.A029	contig022382-TilTARs.A053	0.249	0.538	0.463
contig045999-NyeTARs.A026	contig022320-TilTARs.A059	0.173	0.375	0.463
contig007518-TilTARs.A019	contig022334-TilTARs.A029	0.384	0.830	0.463
contig007518-TilTARs.A019	contig066285-ZebTARs.A016	0.401	0.866	0.463
contig035375-NyeTARs.A013	contig022354-TilTARs.A038	0.263	0.569	0.463
contig022334-TilTARs.A029	contig022363-TilTARs.A043	0.164	0.354	0.463
contig057301-BurTARs.A017	contig053139-ZebTARs.A023	0.294	0.634	0.463
contig086344-BriTARs.A020	contig066056-ZebTARs.A017	0.027	0.059	0.463
contig022383-TilTARs.A054	contig061417-ZebTARs.A020	0.260	0.561	0.463
contig035375-NyeTARs.A013	contig066285-ZebTARs.A016	0.293	0.633	0.463
contig086351-BriTARs.A021	contig022365-TilTARs.A045	0.430	0.929	0.463
contig084868-BriTARs.A014	contig022375-TilTARs.A048	0.242	0.523	0.463
contig049540-BurTARs.A024	contig022337-TilTARs.A031	0.296	0.639	0.463
contig046013-NyeTARs.A021	contig022330-TilTARs.A028	0.125	0.270	0.463
contig084876-BriTARs.A015	contig049534-BurTARs.A025	0.276	0.595	0.463
contig058002-NyeTARs.A025	contig022363-TilTARs.A044	0.357	0.771	0.463
contig060707-BurTARs.A015	contig057301-BurTARs.A017	0.320	0.690	0.463
contig055697-BurTARs.A022	contig042499-NyeTARs.A028	0.281	0.606	0.463
contig022324-TilTARs.A027	contig022379-TilTARs.A052	0.269	0.580	0.463
contig057305-BurTARs.A031	contig030445-ZebTARs.A026	0.352	0.759	0.463
contig007524-TilTARs.A026	contig022368-TilTARs.A047	0.362	0.782	0.463
contig058002-NyeTARs.A025	contig062677-ZebTARs.A018	0.335	0.724	0.463
contig022343-TilTARs.A033	contig022377-TilTARs.A050	0.129	0.279	0.463
contig084880-BriTARs.A016	contig041024-BurTARs.A026	0.172	0.372	0.463
contig082565-BriTARs.A022	contig022354-TilTARs.A037	0.230	0.497	0.463
contig057148-BurTARs.A028	contig022375-TilTARs.A048	0.163	0.352	0.463
contig084887-BriTARs.A018	contig045088-TilTARs.A057	0.347	0.749	0.464
contig045088-TilTARs.A057	contig053139-ZebTARs.A023	0.292	0.631	0.464
contig022345-TilTARs.A034	contig022357-TilTARs.A041	0.149	0.322	0.464
contig049534-BurTARs.A025	contig057305-BurTARs.A031	0.446	0.962	0.464
contig022343-TilTARs.A033	contig022355-TilTARs.A039	0.108	0.233	0.464
contig058002-NyeTARs.A025	contig056200-NyeTARs.A029	0.336	0.724	0.464
contig042499-NyeTARs.A028	contig007520-TilTARs.A025	0.279	0.601	0.464
contig041024-BurTARs.A026	contig035376-NyeTARs.A014	0.234	0.504	0.464
contig022363-TilTARs.A044	contig030445-ZebTARs.A026	0.229	0.493	0.464
contig084886-BriTARs.A017	contig022382-TilTARs.A053	0.275	0.592	0.464
contig022324-TilTARs.A027	contig062677-ZebTARs.A018	0.304	0.656	0.464

contig059673-BurTARs.A016	contig049534-BurTARs.A025	0.380	0.819	0.464
contig056023-BurTARs.A019	contig059766-BurTARs.A029	0.409	0.882	0.464
contig084880-BriTARs.A016	contig022383-TilTARs.A054	0.274	0.591	0.464
contig061977-BurTARs.A012	contig022382-TilTARs.A053	0.152	0.327	0.464
contig056023-BurTARs.A019	contig022354-TilTARs.A038	0.185	0.398	0.464
contig061091-BurTARs.A014	contig045088-TilTARs.A057	0.354	0.762	0.464
contig061433-BurTARs.A013	contig022379-TilTARs.A052	0.275	0.593	0.464
contig084868-BriTARs.A014	contig022337-TilTARs.A031	0.283	0.610	0.464
contig086351-BriTARs.A021	contig030464-ZebTARs.A025	0.415	0.895	0.464
contig084868-BriTARs.A014	contig056021-BurTARs.A020	0.270	0.582	0.464
contig062039-NyeTARs.A022	contig022324-TilTARs.A027	0.276	0.595	0.464
contig049534-BurTARs.A025	contig062039-NyeTARs.A022	0.264	0.568	0.464
contig042499-NyeTARs.A028	contig022377-TilTARs.A050	0.375	0.807	0.464
contig022343-TilTARs.A033	contig022365-TilTARs.A045	0.144	0.309	0.464
contig084887-BriTARs.A018	contig058002-NyeTARs.A025	0.353	0.761	0.464
contig084876-BriTARs.A015	contig086351-BriTARs.A021	0.342	0.737	0.464
contig007524-TilTARs.A026	contig022363-TilTARs.A044	0.358	0.770	0.464
contig086351-BriTARs.A021	contig061977-BurTARs.A012	0.339	0.731	0.464
contig022375-TilTARs.A048	contig030471-ZebTARs.A024	0.148	0.319	0.465
contig035376-NyeTARs.A014	contig022383-TilTARs.A054	0.110	0.237	0.465
contig042499-NyeTARs.A028	contig062677-ZebTARs.A018	0.368	0.792	0.465
contig022343-TilTARs.A033	contig061410-ZebTARs.A021	0.251	0.541	0.465
contig022341-TilTARs.A032	contig030440-ZebTARs.A029	0.264	0.567	0.465
contig038663-NyeTAR.A005	contig022390-TilTARs.A056	0.433	0.933	0.465
contig057148-BurTARs.A028	contig022363-TilTARs.A043	0.188	0.404	0.465
contig061977-BurTARs.A012	contig049540-BurTARs.A024	0.161	0.347	0.465
contig045999-NyeTARs.A026	contig022349-TilTARs.A035	0.197	0.425	0.465
contig035376-NyeTARs.A014	contig007524-TilTARs.A026	0.327	0.703	0.465
contig056200-NyeTARs.A029	contig062676-ZebTARs.A019	0.168	0.362	0.465
contig061433-BurTARs.A013	contig022378-TilTARs.A051	0.276	0.594	0.465
contig059673-BurTARs.A016	contig046007-NyeTARs.A017	0.367	0.790	0.465
contig022378-TilTARs.A051	contig066285-ZebTARs.A016	0.261	0.562	0.465
contig035377-NyeTARs.A023	contig022357-TilTARs.A041	0.273	0.588	0.465
contig084880-BriTARs.A016	contig022377-TilTARs.A050	0.167	0.360	0.465
contig056200-NyeTARs.A029	contig022343-TilTARs.A033	0.285	0.614	0.465
contig060292-NyeTARs.A027	contig030464-ZebTARs.A025	0.417	0.897	0.465
contig084868-BriTARs.A014	contig056200-NyeTARs.A029	0.185	0.399	0.465
contig059673-BurTARs.A016	contig022330-TilTARs.A028	0.347	0.747	0.465
contig045999-NyeTARs.A026	contig022354-TilTARs.A038	0.184	0.395	0.465
contig022334-TilTARs.A030	contig022377-TilTARs.A050	0.204	0.438	0.465
contig086351-BriTARs.A021	contig059673-BurTARs.A016	0.287	0.616	0.465
contig060707-BurTARs.A015	contig045088-TilTARs.A057	0.324	0.697	0.465
contig084880-BriTARs.A016	contig059766-BurTARs.A029	0.410	0.880	0.465
contig062039-NyeTARs.A022	contig022357-TilTARs.A041	0.283	0.609	0.465
contig056020-BurTARs.A021	contig035377-NyeTARs.A023	0.281	0.603	0.465
contig086351-BriTARs.A021	contig056020-BurTARs.A021	0.446	0.959	0.465
contig060292-NyeTARs.A027	contig022334-TilTARs.A030	0.390	0.837	0.465
contig066285-ZebTARs.A016	contig030440-ZebTARs.A029	0.284	0.610	0.465
contig046007-NyeTARs.A017	contig022345-TilTARs.A034	0.135	0.291	0.466
contig022337-TilTARs.A031	contig022375-TilTARs.A048	0.160	0.343	0.466
contig022362-TilTARs.A042	contig022375-TilTARs.A048	0.173	0.372	0.466
contig007524-TilTARs.A026	contig066890-ZebTARs.A014	0.342	0.735	0.466
contig084876-BriTARs.A015	contig022337-TilTARs.A031	0.271	0.582	0.466

contig007524-TilTARs.A026	contig022349-TilTARs.A035	0.356	0.764	0.466
contig046007-NyeTARs.A017	contig035377-NyeTARs.A023	0.285	0.611	0.466
contig022330-TilTARs.A028	contig022383-TilTARs.A054	0.260	0.559	0.466
contig035377-NyeTARs.A023	contig022368-TilTARs.A046	0.269	0.577	0.466
contig035381-NyeTARs.A015	contig030440-ZebTARs.A029	0.065	0.140	0.466
contig084880-BriTARs.A016	contig003909-ZebTAR.A003	0.439	0.942	0.466
contig061433-BurTARs.A013	contig062676-ZebTARs.A019	0.269	0.577	0.466
contig022341-TilTARs.A032	contig066890-ZebTARs.A014	0.265	0.568	0.466
contig084887-BriTARs.A018	contig022357-TilTARs.A041	0.144	0.310	0.466
contig049534-BurTARs.A025	contig022378-TilTARs.A051	0.259	0.555	0.466
contig022383-TilTARs.A054	contig030471-ZebTARs.A024	0.268	0.576	0.466
contig084880-BriTARs.A016	contig086344-BriTARs.A020	0.392	0.841	0.466
contig022345-TilTARs.A034	contig066890-ZebTARs.A014	0.263	0.565	0.466
contig084886-BriTARs.A017	contig056023-BurTARs.A019	0.189	0.405	0.467
contig084868-BriTARs.A014	contig056023-BurTARs.A019	0.290	0.623	0.467
contig035381-NyeTARs.A015	contig045088-TilTARs.A057	0.349	0.749	0.467
contig041024-BurTARs.A026	contig066890-ZebTARs.A014	0.267	0.572	0.467
contig038663-NyeTAR.A005	contig035381-NyeTARs.A015	0.406	0.869	0.467
contig029633-BriTAR.A003	contig045999-NyeTARs.A026	0.475	1.017	0.467
contig084886-BriTARs.A017	contig035381-NyeTARs.A015	0.254	0.544	0.467
contig045302-BurTAR.A001	contig039642-TilTAR.A004	0.066	0.142	0.467
contig056021-BurTARs.A020	contig007518-TilTARs.A019	0.358	0.766	0.467
contig060105-NyeTARs.A018	contig030471-ZebTARs.A024	0.350	0.749	0.467
contig045088-TilTARs.A057	contig066056-ZebTARs.A017	0.325	0.697	0.467
contig022368-TilTARs.A046	contig022368-TilTARs.A047	0.089	0.191	0.467
contig042499-NyeTARs.A028	contig066056-ZebTARs.A017	0.323	0.691	0.467
contig046014-NyeTARs.A024	contig030471-ZebTARs.A024	0.057	0.121	0.467
contig007524-TilTARs.A026	contig022357-TilTARs.A041	0.379	0.812	0.467
contig057145-BurTARs.A018	contig022390-TilTARs.A056	0.268	0.573	0.467
contig057305-BurTARs.A031	contig022334-TilTARs.A030	0.388	0.831	0.467
contig046014-NyeTARs.A024	contig022353-TilTARs.A036	0.120	0.256	0.467
contig022377-TilTARs.A050	contig022379-TilTARs.A052	0.245	0.523	0.467
contig022343-TilTARs.A033	contig066056-ZebTARs.A017	0.328	0.702	0.467
contig020038-BurTAR.A002	contig042499-NyeTARs.A028	0.417	0.891	0.467
contig022368-TilTARs.A047	contig022375-TilTARs.A048	0.166	0.354	0.467
contig049534-BurTARs.A025	contig022354-TilTARs.A037	0.199	0.425	0.468
contig022337-TilTARs.A031	contig022362-TilTARs.A042	0.174	0.373	0.468
contig049540-BurTARs.A024	contig061410-ZebTARs.A021	0.169	0.361	0.468
contig007524-TilTARs.A026	contig022330-TilTARs.A028	0.339	0.725	0.468
contig022375-TilTARs.A048	contig062676-ZebTARs.A019	0.227	0.486	0.468
contig034854-BurTARs.A027	contig022345-TilTARs.A034	0.268	0.572	0.468
contig022377-TilTARs.A050	contig061410-ZebTARs.A021	0.245	0.524	0.468
contig084880-BriTARs.A016	contig056021-BurTARs.A020	0.186	0.399	0.468
contig084868-BriTARs.A014	contig022363-TilTARs.A044	0.252	0.538	0.468
contig022362-TilTARs.A042	contig022390-TilTARs.A056	0.267	0.570	0.468
contig022330-TilTARs.A028	contig030440-ZebTARs.A029	0.264	0.564	0.468
contig045999-NyeTARs.A026	contig066056-ZebTARs.A017	0.377	0.806	0.468
contig022368-TilTARs.A047	contig062676-ZebTARs.A019	0.266	0.569	0.468
contig046007-NyeTARs.A017	contig062039-NyeTARs.A022	0.259	0.553	0.468
contig041024-BurTARs.A026	contig022357-TilTARs.A041	0.138	0.295	0.468
contig022343-TilTARs.A033	contig030445-ZebTARs.A026	0.254	0.543	0.468
contig057301-BurTARs.A017	contig055697-BurTARs.A022	0.294	0.628	0.468
contig007518-TilTARs.A019	contig022377-TilTARs.A050	0.374	0.798	0.468

contig065494-BurTARs.A030	contig045999-NyeTARs.A026	0.417	0.891	0.468
contig061433-BurTARs.A013	contig035377-NyeTARs.A023	0.282	0.602	0.468
contig046013-NyeTARs.A021	contig022365-TilTARs.A045	0.149	0.319	0.468
contig082565-BriTARs.A022	contig061417-ZebTARs.A020	0.250	0.535	0.468
contig059673-BurTARs.A016	contig022349-TilTARs.A035	0.378	0.808	0.468
contig084886-BriTARs.A017	contig059673-BurTARs.A016	0.363	0.776	0.468
contig022353-TilTARs.A036	contig062677-ZebTARs.A018	0.273	0.583	0.468
contig046014-NyeTARs.A024	contig030445-ZebTARs.A026	0.228	0.487	0.468
contig022354-TilTARs.A037	contig062676-ZebTARs.A019	0.230	0.491	0.468
contig022357-TilTARs.A041	contig022377-TilTARs.A050	0.140	0.300	0.468
contig042499-NyeTARs.A028	contig053139-ZebTARs.A023	0.289	0.618	0.468
contig022324-TilTARs.A027	contig022390-TilTARs.A055	0.343	0.732	0.469
contig084886-BriTARs.A017	contig084887-BriTARs.A018	0.171	0.365	0.469
contig035375-NyeTARs.A013	contig030464-ZebTARs.A025	0.277	0.592	0.469
contig022354-TilTARs.A037	contig030464-ZebTARs.A025	0.199	0.424	0.469
contig049534-BurTARs.A025	contig022363-TilTARs.A044	0.199	0.423	0.469
contig084886-BriTARs.A017	contig022353-TilTARs.A036	0.162	0.346	0.469
contig056021-BurTARs.A020	contig049534-BurTARs.A025	0.161	0.343	0.469
contig082565-BriTARs.A022	contig022334-TilTARs.A029	0.256	0.546	0.469
contig007512-TilTARs.A024	contig045088-TilTARs.A057	0.283	0.604	0.469
contig082565-BriTARs.A022	contig056021-BurTARs.A020	0.252	0.537	0.469
contig022382-TilTARs.A053	contig045088-TilTARs.A057	0.366	0.781	0.469
contig084876-BriTARs.A015	contig061417-ZebTARs.A020	0.250	0.534	0.469
contig062039-NyeTARs.A022	contig022337-TilTARs.A031	0.266	0.568	0.469
contig084886-BriTARs.A017	contig060292-NyeTARs.A027	0.401	0.856	0.469
contig022349-TilTARs.A035	contig022357-TilTARs.A041	0.164	0.349	0.469
contig022377-TilTARs.A050	contig066890-ZebTARs.A014	0.264	0.563	0.469
contig082565-BriTARs.A022	contig022379-TilTARs.A052	0.131	0.279	0.469
contig007518-TilTARs.A019	contig053139-ZebTARs.A023	0.309	0.657	0.469
contig049540-BurTARs.A024	contig022356-TilTARs.A040	0.287	0.611	0.469
contig022363-TilTARs.A044	contig062676-ZebTARs.A019	0.235	0.500	0.469
contig057301-BurTARs.A017	contig066691-ZebTARs.A015	0.356	0.758	0.470
contig020038-BurTAR.A002	contig045999-NyeTARs.A026	0.444	0.945	0.470
contig022349-TilTARs.A035	contig022390-TilTARs.A056	0.283	0.602	0.470
contig022337-TilTARs.A031	contig030445-ZebTARs.A026	0.261	0.555	0.470
contig041024-BurTARs.A026	contig042499-NyeTARs.A028	0.344	0.732	0.470
contig022356-TilTARs.A040	contig022363-TilTARs.A044	0.156	0.332	0.470
contig061977-BurTARs.A012	contig056200-NyeTARs.A029	0.163	0.347	0.470
contig060707-BurTARs.A015	contig056021-BurTARs.A020	0.332	0.708	0.470
contig061977-BurTARs.A012	contig022354-TilTARs.A037	0.224	0.477	0.470
contig035381-NyeTARs.A015	contig046014-NyeTARs.A024	0.236	0.502	0.470
contig041024-BurTARs.A026	contig022390-TilTARs.A056	0.262	0.558	0.470
contig056021-BurTARs.A020	contig030440-ZebTARs.A029	0.262	0.557	0.470
contig061977-BurTARs.A012	contig022355-TilTARs.A039	0.252	0.537	0.470
contig022353-TilTARs.A036	contig022368-TilTARs.A047	0.151	0.321	0.470
contig084876-BriTARs.A015	contig022362-TilTARs.A042	0.256	0.544	0.470
contig007520-TilTARs.A025	contig030471-ZebTARs.A024	0.355	0.756	0.470
contig086344-BriTARs.A020	contig022337-TilTARs.A031	0.369	0.783	0.470
contig059673-BurTARs.A016	contig022324-TilTARs.A027	0.392	0.832	0.471
contig035377-NyeTARs.A023	contig022368-TilTARs.A047	0.288	0.613	0.471
contig038663-NyeTAR.A005	contig066890-ZebTARs.A014	0.435	0.925	0.471
contig035381-NyeTARs.A015	contig022324-TilTARs.A027	0.278	0.591	0.471
contig057301-BurTARs.A017	contig007512-TilTARs.A024	0.293	0.623	0.471

contig022343-TilTARs.A033	contig022349-TilTARs.A035	0.153	0.326	0.471
contig035376-NyeTARs.A014	contig022330-TilTARs.A028	0.255	0.542	0.471
contig022356-TilTARs.A040	contig066890-ZebTARs.A014	0.269	0.573	0.471
contig057305-BurTARs.A031	contig022379-TilTARs.A052	0.346	0.735	0.471
contig084868-BriTARs.A014	contig066691-ZebTARs.A015	0.300	0.637	0.471
contig062039-NyeTARs.A022	contig061417-ZebTARs.A020	0.251	0.533	0.471
contig086351-BriTARs.A021	contig049534-BurTARs.A025	0.452	0.959	0.471
contig057145-BurTARs.A018	contig022334-TilTARs.A030	0.189	0.402	0.471
contig057301-BurTARs.A017	contig066056-ZebTARs.A017	0.325	0.690	0.471
contig022334-TilTARs.A029	contig022383-TilTARs.A054	0.262	0.555	0.471
contig056023-BurTARs.A019	contig003909-ZebTAR.A003	0.448	0.950	0.471
contig084887-BriTARs.A018	contig057301-BurTARs.A017	0.345	0.732	0.471
contig084876-BriTARs.A015	contig030471-ZebTARs.A024	0.268	0.568	0.471
contig034854-BurTARs.A027	contig030464-ZebTARs.A025	0.281	0.597	0.471
contig022355-TilTARs.A039	contig022362-TilTARs.A042	0.151	0.320	0.471
contig057301-BurTARs.A017	contig056200-NyeTARs.A029	0.363	0.771	0.471
contig082565-BriTARs.A022	contig061410-ZebTARs.A021	0.132	0.280	0.471
contig022343-TilTARs.A033	contig030440-ZebTARs.A029	0.254	0.539	0.471
contig084886-BriTARs.A017	contig086351-BriTARs.A021	0.400	0.850	0.471
contig046011-NyeTARs.A016	contig022362-TilTARs.A042	0.230	0.487	0.471
contig022363-TilTARs.A043	contig022368-TilTARs.A046	0.072	0.153	0.471
contig022357-TilTARs.A041	contig061417-ZebTARs.A020	0.137	0.290	0.471
contig061091-BurTARs.A014	contig057301-BurTARs.A017	0.351	0.745	0.471
contig084868-BriTARs.A014	contig022334-TilTARs.A029	0.271	0.574	0.471
contig046013-NyeTARs.A021	contig022354-TilTARs.A038	0.149	0.316	0.471
contig061417-ZebTARs.A020	contig030440-ZebTARs.A029	0.263	0.558	0.471
contig057305-BurTARs.A031	contig030464-ZebTARs.A025	0.417	0.885	0.472
contig059673-BurTARs.A016	contig041024-BurTARs.A026	0.341	0.722	0.472
contig042499-NyeTARs.A028	contig022368-TilTARs.A047	0.397	0.841	0.472
contig022341-TilTARs.A032	contig045088-TilTARs.A057	0.368	0.781	0.472
contig035381-NyeTARs.A015	contig066691-ZebTARs.A015	0.276	0.585	0.472
contig041024-BurTARs.A026	contig062677-ZebTARs.A018	0.264	0.560	0.472
contig022368-TilTARs.A046	contig066691-ZebTARs.A015	0.140	0.296	0.472
contig022324-TilTARs.A027	contig022353-TilTARs.A036	0.178	0.376	0.472
contig084886-BriTARs.A017	contig056200-NyeTARs.A029	0.281	0.595	0.472
contig007518-TilTARs.A019	contig007512-TilTARs.A024	0.309	0.654	0.472
contig022337-TilTARs.A031	contig022368-TilTARs.A046	0.163	0.346	0.472
contig049540-BurTARs.A024	contig022324-TilTARs.A027	0.303	0.643	0.472
contig084868-BriTARs.A014	contig061417-ZebTARs.A020	0.272	0.576	0.472
contig084886-BriTARs.A017	contig057148-BurTARs.A028	0.027	0.058	0.472
contig084880-BriTARs.A016	contig007512-TilTARs.A024	0.382	0.809	0.472
contig082565-BriTARs.A022	contig057301-BurTARs.A017	0.349	0.740	0.472
contig035377-NyeTARs.A023	contig030471-ZebTARs.A024	0.280	0.593	0.472
contig022363-TilTARs.A043	contig066056-ZebTARs.A017	0.385	0.814	0.472
contig035381-NyeTARs.A015	contig062677-ZebTARs.A018	0.162	0.343	0.472
contig022330-TilTARs.A028	contig066890-ZebTARs.A014	0.267	0.566	0.472
contig059673-BurTARs.A016	contig046010-NyeTARs.A019	0.371	0.786	0.472
contig022377-TilTARs.A050	contig022390-TilTARs.A056	0.255	0.539	0.472
contig056020-BurTARs.A021	contig062039-NyeTARs.A022	0.257	0.544	0.473
contig056200-NyeTARs.A029	contig061410-ZebTARs.A021	0.170	0.361	0.473
contig060292-NyeTARs.A027	contig030445-ZebTARs.A026	0.354	0.749	0.473
contig056200-NyeTARs.A029	contig022337-TilTARs.A031	0.298	0.630	0.473
contig056020-BurTARs.A021	contig022349-TilTARs.A035	0.156	0.331	0.473

contig058002-NyeTARs.A025	contig066691-ZebTARs.A015	0.363	0.768	0.473
contig084886-BriTARs.A017	contig030440-ZebTARs.A029	0.269	0.569	0.473
contig057145-BurTARs.A018	contig066691-ZebTARs.A015	0.126	0.267	0.473
contig082565-BriTARs.A022	contig042499-NyeTARs.A028	0.348	0.736	0.473
contig084868-BriTARs.A014	contig022357-TilTARs.A041	0.292	0.618	0.473
contig046007-NyeTARs.A017	contig060292-NyeTARs.A027	0.411	0.870	0.473
contig038663-NyeTAR.A005	contig039640-TilTAR.A002	0.075	0.159	0.473
contig022324-TilTARs.A027	contig066890-ZebTARs.A014	0.284	0.601	0.473
contig066890-ZebTARs.A014	contig030464-ZebTARs.A025	0.276	0.584	0.473
contig060105-NyeTARs.A018	contig022357-TilTARs.A041	0.359	0.758	0.473
contig086344-BriTARs.A020	contig060105-NyeTARs.A018	0.013	0.027	0.473
contig084880-BriTARs.A016	contig032272-NyeTAR.A004	0.439	0.929	0.473
contig007524-TilTARs.A026	contig022382-TilTARs.A053	0.333	0.704	0.473
contig084880-BriTARs.A016	contig022368-TilTARs.A047	0.203	0.430	0.473
contig034854-BurTARs.A027	contig035381-NyeTARs.A015	0.057	0.121	0.473
contig022363-TilTARs.A043	contig061410-ZebTARs.A021	0.266	0.563	0.473
contig059673-BurTARs.A016	contig066285-ZebTARs.A016	0.388	0.819	0.473
contig060292-NyeTARs.A027	contig022341-TilTARs.A032	0.409	0.863	0.473
contig046010-NyeTARs.A019	contig007524-TilTARs.A026	0.358	0.757	0.474
contig045999-NyeTARs.A026	contig066330-ZebTARs.A027	0.420	0.886	0.474
contig022334-TilTARs.A030	contig022355-TilTARs.A039	0.185	0.390	0.474
contig022368-TilTARs.A047	contig062677-ZebTARs.A018	0.295	0.623	0.474
contig084868-BriTARs.A014	contig022330-TilTARs.A028	0.273	0.577	0.474
contig046010-NyeTARs.A019	contig066890-ZebTARs.A014	0.269	0.568	0.474
contig084886-BriTARs.A017	contig045999-NyeTARs.A026	0.189	0.399	0.474
contig046014-NyeTARs.A024	contig058002-NyeTARs.A025	0.340	0.718	0.474
contig029633-BriTAR.A003	contig056023-BurTARs.A019	0.475	1.002	0.474
contig062039-NyeTARs.A022	contig022343-TilTARs.A033	0.267	0.564	0.474
contig022353-TilTARs.A036	contig022383-TilTARs.A054	0.278	0.586	0.474
contig057145-BurTARs.A018	contig066890-ZebTARs.A014	0.269	0.568	0.474
contig045999-NyeTARs.A026	contig022368-TilTARs.A047	0.204	0.431	0.474
contig022355-TilTARs.A039	contig022382-TilTARs.A053	0.274	0.578	0.474
contig007524-TilTARs.A026	contig062677-ZebTARs.A018	0.350	0.739	0.474
contig022353-TilTARs.A036	contig061410-ZebTARs.A021	0.250	0.528	0.474
contig086351-BriTARs.A021	contig022355-TilTARs.A039	0.419	0.883	0.474
contig060707-BurTARs.A015	contig022362-TilTARs.A042	0.357	0.753	0.474
contig022345-TilTARs.A034	contig045088-TilTARs.A057	0.356	0.751	0.474
contig084886-BriTARs.A017	contig022379-TilTARs.A052	0.253	0.533	0.474
contig046007-NyeTARs.A017	contig062677-ZebTARs.A018	0.291	0.613	0.474
contig086351-BriTARs.A021	contig007524-TilTARs.A026	0.272	0.574	0.474
contig046014-NyeTARs.A024	contig062676-ZebTARs.A019	0.250	0.528	0.474
contig022378-TilTARs.A051	contig030445-ZebTARs.A026	0.212	0.447	0.474
contig046014-NyeTARs.A024	contig022363-TilTARs.A044	0.154	0.325	0.474
contig084868-BriTARs.A014	contig030471-ZebTARs.A024	0.277	0.583	0.475
contig022357-TilTARs.A041	contig022363-TilTARs.A043	0.195	0.410	0.475
contig049540-BurTARs.A024	contig022379-TilTARs.A052	0.172	0.362	0.475
contig022343-TilTARs.A033	contig045088-TilTARs.A057	0.367	0.772	0.475
contig045999-NyeTARs.A026	contig023443-TilTAR.A005	0.433	0.912	0.475
contig022356-TilTARs.A040	contig066691-ZebTARs.A015	0.123	0.260	0.475
contig056023-BurTARs.A019	contig066056-ZebTARs.A017	0.377	0.795	0.475
contig057301-BurTARs.A017	contig022382-TilTARs.A053	0.363	0.765	0.475
contig058002-NyeTARs.A025	contig030440-ZebTARs.A029	0.332	0.700	0.475
contig022390-TilTARs.A055	contig040586-ZebTAR.A001	0.417	0.878	0.475

contig045302-BurTAR.A001	contig022390-TilTARs.A055	0.417	0.878	0.475
contig059673-BurTARs.A016	contig022337-TilTARs.A031	0.374	0.787	0.475
contig061091-BurTARs.A014	contig022368-TilTARs.A047	0.159	0.335	0.475
contig084886-BriTARs.A017	contig049540-BurTARs.A024	0.279	0.586	0.475
contig084886-BriTARs.A017	contig035377-NyeTARs.A023	0.267	0.563	0.475
contig056023-BurTARs.A019	contig022354-TilTARs.A037	0.224	0.471	0.475
contig022357-TilTARs.A041	contig022382-TilTARs.A053	0.288	0.605	0.475
contig049534-BurTARs.A025	contig061417-ZebTARs.A020	0.164	0.345	0.475
contig056023-BurTARs.A019	contig022377-TilTARs.A050	0.167	0.352	0.475
contig057145-BurTARs.A018	contig034854-BurTARs.A027	0.268	0.564	0.475
contig022353-TilTARs.A036	contig022354-TilTARs.A037	0.165	0.347	0.475
contig082565-BriTARs.A022	contig041024-BurTARs.A026	0.238	0.500	0.475
contig022382-TilTARs.A053	contig061410-ZebTARs.A021	0.159	0.334	0.475
contig084880-BriTARs.A016	contig056134-TilTARs.A058	0.403	0.847	0.475
contig061091-BurTARs.A014	contig056020-BurTARs.A021	0.108	0.227	0.475
contig084880-BriTARs.A016	contig065494-BurTARs.A030	0.414	0.871	0.475
contig034854-BurTARs.A027	contig058002-NyeTARs.A025	0.340	0.714	0.476
contig062677-ZebTARs.A018	contig030445-ZebTARs.A026	0.160	0.337	0.476
contig007518-TilTARs.A019	contig022375-TilTARs.A048	0.365	0.767	0.476
contig084886-BriTARs.A017	contig057305-BurTARs.A031	0.401	0.844	0.476
contig062039-NyeTARs.A022	contig022365-TilTARs.A045	0.254	0.534	0.476
contig086351-BriTARs.A021	contig046007-NyeTARs.A017	0.421	0.886	0.476
contig061433-BurTARs.A013	contig062039-NyeTARs.A022	0.287	0.602	0.476
contig022356-TilTARs.A040	contig062677-ZebTARs.A018	0.289	0.608	0.476
contig060707-BurTARs.A015	contig022390-TilTARs.A055	0.425	0.893	0.476
contig056021-BurTARs.A020	contig022362-TilTARs.A042	0.157	0.330	0.476
contig022334-TilTARs.A029	contig022362-TilTARs.A042	0.145	0.305	0.476
contig056021-BurTARs.A020	contig062039-NyeTARs.A022	0.259	0.544	0.476
contig060292-NyeTARs.A027	contig022354-TilTARs.A038	0.409	0.859	0.476
contig022377-TilTARs.A050	contig061417-ZebTARs.A020	0.145	0.305	0.476
contig055697-BurTARs.A022	contig045999-NyeTARs.A026	0.397	0.833	0.476
contig082565-BriTARs.A022	contig046010-NyeTARs.A019	0.260	0.545	0.476
contig022354-TilTARs.A037	contig066285-ZebTARs.A016	0.209	0.438	0.476
contig025313-BriTAR.A002	contig084880-BriTARs.A016	0.436	0.916	0.476
contig042499-NyeTARs.A028	contig007512-TilTARs.A024	0.289	0.607	0.476
contig059673-BurTARs.A016	contig022334-TilTARs.A029	0.347	0.729	0.476
contig007518-TilTARs.A019	contig022368-TilTARs.A047	0.384	0.806	0.476
contig041024-BurTARs.A026	contig045088-TilTARs.A057	0.342	0.718	0.477
contig022353-TilTARs.A036	contig030445-ZebTARs.A026	0.252	0.528	0.477
contig086344-BriTARs.A020	contig056021-BurTARs.A020	0.340	0.713	0.477
contig035376-NyeTARs.A014	contig061417-ZebTARs.A020	0.250	0.524	0.477
contig025313-BriTAR.A002	contig056023-BurTARs.A019	0.443	0.930	0.477
contig022334-TilTARs.A029	contig022354-TilTARs.A037	0.169	0.355	0.477
contig084886-BriTARs.A017	contig022334-TilTARs.A030	0.200	0.420	0.477
contig007520-TilTARs.A025	contig030440-ZebTARs.A029	0.338	0.708	0.477
contig084880-BriTARs.A016	contig023443-TilTAR.A005	0.427	0.895	0.477
contig035381-NyeTARs.A015	contig042499-NyeTARs.A028	0.346	0.726	0.477
contig022343-TilTARs.A033	contig022379-TilTARs.A052	0.249	0.523	0.477
contig084876-BriTARs.A015	contig022343-TilTARs.A033	0.263	0.552	0.477
contig061433-BurTARs.A013	contig035381-NyeTARs.A015	0.279	0.586	0.477
contig022343-TilTARs.A033	contig062676-ZebTARs.A019	0.263	0.552	0.477
contig022324-TilTARs.A027	contig022390-TilTARs.A056	0.281	0.590	0.477
contig059673-BurTARs.A016	contig022357-TilTARs.A041	0.387	0.810	0.477

contig084880-BriTARs.A016	contig046014-NyeTARs.A024	0.174	0.365	0.477
contig022355-TilTARs.A039	contig066890-ZebTARs.A014	0.280	0.587	0.477
contig022334-TilTARs.A030	contig022349-TilTARs.A035	0.197	0.414	0.477
contig084887-BriTARs.A018	contig022337-TilTARs.A031	0.118	0.247	0.477
contig035376-NyeTARs.A014	contig022334-TilTARs.A029	0.255	0.535	0.477
contig056020-BurTARs.A021	contig045999-NyeTARs.A026	0.184	0.386	0.477
contig056021-BurTARs.A020	contig035376-NyeTARs.A014	0.251	0.527	0.477
contig045999-NyeTARs.A026	contig022345-TilTARs.A034	0.187	0.391	0.477
contig059673-BurTARs.A016	contig022334-TilTARs.A030	0.355	0.744	0.478
contig056200-NyeTARs.A029	contig022356-TilTARs.A040	0.288	0.604	0.478
contig022363-TilTARs.A044	contig022390-TilTARs.A056	0.239	0.501	0.478
contig046014-NyeTARs.A024	contig007520-TilTARs.A025	0.337	0.706	0.478
contig022353-TilTARs.A036	contig022365-TilTARs.A045	0.137	0.286	0.478
contig084876-BriTARs.A015	contig022363-TilTARs.A044	0.235	0.492	0.478
contig035376-NyeTARs.A014	contig061410-ZebTARs.A021	0.133	0.278	0.478
contig035375-NyeTARs.A013	contig046010-NyeTARs.A019	0.275	0.576	0.478
contig084868-BriTARs.A014	contig061091-BurTARs.A014	0.296	0.619	0.478
contig062039-NyeTARs.A022	contig022377-TilTARs.A050	0.249	0.522	0.478
contig042499-NyeTARs.A028	contig056200-NyeTARs.A029	0.367	0.767	0.478
contig056021-BurTARs.A020	contig022337-TilTARs.A031	0.128	0.268	0.478
contig056023-BurTARs.A019	contig032272-NyeTAR.A004	0.448	0.936	0.478
contig022379-TilTARs.A052	contig030471-ZebTARs.A024	0.260	0.544	0.478
contig066890-ZebTARs.A014	contig066285-ZebTARs.A016	0.282	0.590	0.478
contig086351-BriTARs.A021	contig022354-TilTARs.A038	0.408	0.853	0.478
contig046014-NyeTARs.A024	contig066890-ZebTARs.A014	0.269	0.563	0.478
contig035376-NyeTARs.A014	contig022354-TilTARs.A037	0.233	0.488	0.478
contig022383-TilTARs.A054	contig022390-TilTARs.A055	0.141	0.294	0.478
contig084868-BriTARs.A014	contig045088-TilTARs.A057	0.370	0.773	0.478
contig086344-BriTARs.A020	contig042499-NyeTARs.A028	0.330	0.691	0.478
contig022377-TilTARs.A050	contig030440-ZebTARs.A029	0.264	0.551	0.478
contig049540-BurTARs.A024	contig066691-ZebTARs.A015	0.291	0.609	0.478
contig007524-TilTARs.A026	contig022334-TilTARs.A029	0.339	0.708	0.478
contig060105-NyeTARs.A018	contig022390-TilTARs.A056	0.339	0.709	0.479
contig022390-TilTARs.A056	contig066890-ZebTARs.A014	0.035	0.072	0.479
contig046010-NyeTARs.A019	contig022363-TilTARs.A043	0.185	0.386	0.479
contig022354-TilTARs.A037	contig030440-ZebTARs.A029	0.249	0.520	0.479
contig042499-NyeTARs.A028	contig022345-TilTARs.A034	0.355	0.742	0.479
contig022354-TilTARs.A037	contig061410-ZebTARs.A021	0.225	0.470	0.479
contig056023-BurTARs.A019	contig065494-BurTARs.A030	0.415	0.866	0.479
contig084880-BriTARs.A016	contig007520-TilTARs.A025	0.393	0.822	0.479
contig086351-BriTARs.A021	contig035381-NyeTARs.A015	0.368	0.769	0.479
contig061091-BurTARs.A014	contig022368-TilTARs.A046	0.136	0.285	0.479
contig022363-TilTARs.A044	contig022320-TilTARs.A059	0.155	0.323	0.479
contig042499-NyeTARs.A028	contig022343-TilTARs.A033	0.366	0.763	0.479
contig082565-BriTARs.A022	contig022355-TilTARs.A039	0.256	0.535	0.479
contig045999-NyeTARs.A026	contig022383-TilTARs.A054	0.274	0.573	0.479
contig057301-BurTARs.A017	contig022345-TilTARs.A034	0.360	0.751	0.479
contig059673-BurTARs.A016	contig022354-TilTARs.A037	0.349	0.729	0.479
contig057301-BurTARs.A017	contig022368-TilTARs.A047	0.397	0.828	0.479
contig056021-BurTARs.A020	contig022334-TilTARs.A029	0.046	0.096	0.479
contig084880-BriTARs.A016	contig022365-TilTARs.A045	0.185	0.385	0.479
contig022365-TilTARs.A045	contig022378-TilTARs.A051	0.257	0.536	0.479
contig049540-BurTARs.A024	contig007524-TilTARs.A026	0.349	0.727	0.479

contig046011-NyeTARs.A016	contig022355-TilTARs.A039	0.236	0.492	0.479
contig057301-BurTARs.A017	contig022343-TilTARs.A033	0.370	0.773	0.479
contig007524-TilTARs.A026	contig022390-TilTARs.A056	0.341	0.711	0.479
contig041024-BurTARs.A026	contig007524-TilTARs.A026	0.327	0.681	0.480
contig060292-NyeTARs.A027	contig022379-TilTARs.A052	0.346	0.722	0.480
contig056200-NyeTARs.A029	contig022379-TilTARs.A052	0.174	0.362	0.480
contig059673-BurTARs.A016	contig022363-TilTARs.A044	0.372	0.775	0.480
contig022334-TilTARs.A029	contig030440-ZebTARs.A029	0.265	0.553	0.480
contig034854-BurTARs.A027	contig022337-TilTARs.A031	0.278	0.579	0.480
contig056023-BurTARs.A019	contig041024-BurTARs.A026	0.173	0.361	0.480
contig084886-BriTARs.A017	contig007524-TilTARs.A026	0.356	0.743	0.480
contig061091-BurTARs.A014	contig007524-TilTARs.A026	0.365	0.761	0.480
contig046007-NyeTARs.A017	contig022362-TilTARs.A042	0.162	0.338	0.480
contig007520-TilTARs.A025	contig022324-TilTARs.A027	0.368	0.767	0.480
contig056020-BurTARs.A021	contig022383-TilTARs.A054	0.270	0.563	0.480
contig059673-BurTARs.A016	contig022363-TilTARs.A043	0.404	0.842	0.480
contig057305-BurTARs.A031	contig022341-TilTARs.A032	0.409	0.851	0.480
contig022357-TilTARs.A041	contig066691-ZebTARs.A015	0.147	0.307	0.480
contig062039-NyeTARs.A022	contig022356-TilTARs.A040	0.273	0.568	0.480
contig084887-BriTARs.A018	contig022368-TilTARs.A046	0.135	0.282	0.480
contig049540-BurTARs.A024	contig041024-BurTARs.A026	0.263	0.548	0.480
contig022334-TilTARs.A030	contig022357-TilTARs.A041	0.193	0.402	0.480
contig022363-TilTARs.A044	contig022368-TilTARs.A047	0.159	0.332	0.480
contig059673-BurTARs.A016	contig022368-TilTARs.A047	0.380	0.792	0.480
contig034854-BurTARs.A027	contig046010-NyeTARs.A019	0.279	0.581	0.480
contig022357-TilTARs.A041	contig022362-TilTARs.A042	0.170	0.353	0.480
contig022343-TilTARs.A033	contig022354-TilTARs.A037	0.185	0.385	0.480
contig086351-BriTARs.A021	contig022379-TilTARs.A052	0.353	0.734	0.480
contig056020-BurTARs.A021	contig062677-ZebTARs.A018	0.290	0.603	0.480
contig061091-BurTARs.A014	contig057145-BurTARs.A018	0.123	0.256	0.480
contig022362-TilTARs.A042	contig030445-ZebTARs.A026	0.258	0.537	0.480
contig058002-NyeTARs.A025	contig007518-TilTARs.A019	0.267	0.555	0.481
contig062039-NyeTARs.A022	contig022363-TilTARs.A044	0.243	0.505	0.481
contig022390-TilTARs.A056	contig066285-ZebTARs.A016	0.275	0.571	0.481
contig022365-TilTARs.A045	contig045088-TilTARs.A057	0.380	0.791	0.481
contig084880-BriTARs.A016	contig066330-ZebTARs.A027	0.417	0.866	0.481
contig084880-BriTARs.A016	contig022356-TilTARs.A040	0.192	0.399	0.481
contig022349-TilTARs.A035	contig022355-TilTARs.A039	0.150	0.313	0.481
contig082565-BriTARs.A022	contig057305-BurTARs.A031	0.353	0.733	0.481
contig022355-TilTARs.A039	contig022363-TilTARs.A044	0.164	0.340	0.481
contig057145-BurTARs.A018	contig022378-TilTARs.A051	0.259	0.538	0.481
contig022330-TilTARs.A028	contig022354-TilTARs.A037	0.168	0.348	0.481
contig084880-BriTARs.A016	contig055697-BurTARs.A022	0.397	0.825	0.482
contig084886-BriTARs.A017	contig062676-ZebTARs.A019	0.257	0.533	0.482
contig042499-NyeTARs.A028	contig022382-TilTARs.A053	0.366	0.761	0.482
contig046013-NyeTARs.A021	contig022354-TilTARs.A037	0.176	0.366	0.482
contig046014-NyeTARs.A024	contig022390-TilTARs.A056	0.265	0.550	0.482
contig056200-NyeTARs.A029	contig022324-TilTARs.A027	0.306	0.634	0.482
contig046007-NyeTARs.A017	contig022390-TilTARs.A055	0.265	0.551	0.482
contig057301-BurTARs.A017	contig035381-NyeTARs.A015	0.353	0.732	0.482
contig061977-BurTARs.A012	contig066890-ZebTARs.A014	0.160	0.331	0.482
contig007524-TilTARs.A026	contig022320-TilTARs.A059	0.332	0.689	0.482
contig060707-BurTARs.A015	contig022353-TilTARs.A036	0.358	0.743	0.482

contig022337-TilTARs.A031	contig062676-ZebTARs.A019	0.274	0.567	0.482
contig084880-BriTARs.A016	contig022368-TilTARs.A046	0.195	0.404	0.482
contig061091-BurTARs.A014	contig022356-TilTARs.A040	0.120	0.249	0.482
contig046014-NyeTARs.A024	contig062677-ZebTARs.A018	0.269	0.558	0.482
contig058002-NyeTARs.A025	contig022375-TilTARs.A048	0.337	0.700	0.482
contig022355-TilTARs.A039	contig062676-ZebTARs.A019	0.266	0.551	0.482
contig086344-BriTARs.A020	contig057301-BurTARs.A017	0.333	0.690	0.483
contig084880-BriTARs.A016	contig060707-BurTARs.A015	0.386	0.801	0.483
contig057145-BurTARs.A018	contig022353-TilTARs.A036	0.139	0.287	0.483
contig022343-TilTARs.A033	contig022378-TilTARs.A051	0.261	0.541	0.483
contig057305-BurTARs.A031	contig022354-TilTARs.A038	0.409	0.847	0.483
contig035381-NyeTARs.A015	contig022357-TilTARs.A041	0.279	0.579	0.483
contig056020-BurTARs.A021	contig046011-NyeTARs.A016	0.238	0.492	0.483
contig056021-BurTARs.A020	contig060105-NyeTARs.A018	0.338	0.701	0.483
contig084880-BriTARs.A016	contig022349-TilTARs.A035	0.203	0.419	0.483
contig084887-BriTARs.A018	contig035381-NyeTARs.A015	0.270	0.559	0.483
contig086344-BriTARs.A020	contig045088-TilTARs.A057	0.337	0.698	0.483
contig022345-TilTARs.A034	contig022349-TilTARs.A035	0.153	0.317	0.483
contig059673-BurTARs.A016	contig022382-TilTARs.A053	0.346	0.715	0.483
contig066691-ZebTARs.A015	contig062677-ZebTARs.A018	0.292	0.605	0.483
contig060292-NyeTARs.A027	contig022355-TilTARs.A039	0.417	0.864	0.483
contig049534-BurTARs.A025	contig007520-TilTARs.A025	0.379	0.783	0.483
contig022368-TilTARs.A047	contig022390-TilTARs.A056	0.281	0.580	0.483
contig049540-BurTARs.A024	contig035381-NyeTARs.A015	0.160	0.331	0.483
contig035375-NyeTARs.A013	contig022349-TilTARs.A035	0.277	0.572	0.483
contig084887-BriTARs.A018	contig022363-TilTARs.A044	0.167	0.344	0.483
contig022330-TilTARs.A028	contig045088-TilTARs.A057	0.366	0.757	0.483
contig061433-BurTARs.A013	contig030445-ZebTARs.A026	0.275	0.569	0.484
contig022356-TilTARs.A040	contig022390-TilTARs.A056	0.267	0.552	0.484
contig084880-BriTARs.A016	contig020038-BurTAR.A002	0.444	0.918	0.484
contig022363-TilTARs.A044	contig022375-TilTARs.A048	0.090	0.186	0.484
contig082565-BriTARs.A022	contig022363-TilTARs.A044	0.237	0.491	0.484
contig057305-BurTARs.A031	contig046007-NyeTARs.A017	0.417	0.861	0.484
contig084880-BriTARs.A016	contig062676-ZebTARs.A019	0.273	0.564	0.484
contig059673-BurTARs.A016	contig022320-TilTARs.A059	0.349	0.721	0.484
contig022356-TilTARs.A040	contig022390-TilTARs.A055	0.234	0.483	0.484
contig086351-BriTARs.A021	contig030445-ZebTARs.A026	0.360	0.743	0.484
contig041024-BurTARs.A026	contig022375-TilTARs.A048	0.139	0.287	0.484
contig056023-BurTARs.A019	contig022349-TilTARs.A035	0.199	0.411	0.484
contig056023-BurTARs.A019	contig066330-ZebTARs.A027	0.417	0.862	0.484
contig035375-NyeTARs.A013	contig022330-TilTARs.A028	0.274	0.565	0.484
contig062676-ZebTARs.A019	contig030445-ZebTARs.A026	0.109	0.225	0.484
contig022375-TilTARs.A048	contig030445-ZebTARs.A026	0.218	0.451	0.484
contig045999-NyeTARs.A026	contig007512-TilTARs.A024	0.385	0.795	0.484
contig035381-NyeTARs.A015	contig022365-TilTARs.A045	0.270	0.557	0.484
contig022363-TilTARs.A043	contig059768-ZebTARs.A022	0.376	0.777	0.484
contig022368-TilTARs.A047	contig066691-ZebTARs.A015	0.163	0.335	0.485
contig034854-BurTARs.A027	contig022354-TilTARs.A037	0.247	0.510	0.485
contig007518-TilTARs.A019	contig022357-TilTARs.A041	0.403	0.831	0.485
contig061091-BurTARs.A014	contig022337-TilTARs.A031	0.117	0.241	0.485
contig045999-NyeTARs.A026	contig056134-TilTARs.A058	0.403	0.831	0.485
contig022377-TilTARs.A050	contig022378-TilTARs.A051	0.243	0.502	0.485
contig056023-BurTARs.A019	contig022320-TilTARs.A059	0.174	0.359	0.485

contig035376-NyeTARs.A014	contig022379-TilTARs.A052	0.132	0.271	0.485
contig022334-TilTARs.A029	contig045088-TilTARs.A057	0.363	0.749	0.485
contig084880-BriTARs.A016	contig022357-TilTARs.A041	0.190	0.392	0.485
contig022356-TilTARs.A040	contig062676-ZebTARs.A019	0.258	0.531	0.485
contig022343-TilTARs.A033	contig022382-TilTARs.A053	0.276	0.569	0.485
contig046007-NyeTARs.A017	contig022349-TilTARs.A035	0.162	0.334	0.485
contig022356-TilTARs.A040	contig022382-TilTARs.A053	0.279	0.575	0.485
contig084868-BriTARs.A014	contig035381-NyeTARs.A015	0.122	0.252	0.485
contig022363-TilTARs.A043	contig022390-TilTARs.A056	0.291	0.600	0.485
contig035376-NyeTARs.A014	contig022320-TilTARs.A059	0.240	0.495	0.485
contig042499-NyeTARs.A028	contig022363-TilTARs.A044	0.382	0.787	0.485
contig049540-BurTARs.A024	contig022320-TilTARs.A059	0.265	0.545	0.485
contig057145-BurTARs.A018	contig056023-BurTARs.A019	0.195	0.401	0.485
contig059673-BurTARs.A016	contig056023-BurTARs.A019	0.400	0.824	0.485
contig061091-BurTARs.A014	contig035381-NyeTARs.A015	0.272	0.561	0.485
contig022378-TilTARs.A051	contig045088-TilTARs.A057	0.349	0.719	0.485
contig035376-NyeTARs.A014	contig022378-TilTARs.A051	0.225	0.463	0.485
contig035376-NyeTARs.A014	contig045088-TilTARs.A057	0.360	0.743	0.485
contig022334-TilTARs.A030	contig066890-ZebTARs.A014	0.264	0.543	0.485
contig035375-NyeTARs.A013	contig007520-TilTARs.A025	0.338	0.697	0.485
contig046011-NyeTARs.A016	contig022368-TilTARs.A047	0.253	0.522	0.486
contig022390-TilTARs.A055	contig045088-TilTARs.A057	0.361	0.744	0.486
contig022363-TilTARs.A044	contig022365-TilTARs.A045	0.162	0.334	0.486
contig060105-NyeTARs.A018	contig022362-TilTARs.A042	0.364	0.750	0.486
contig022377-TilTARs.A050	contig022382-TilTARs.A053	0.269	0.554	0.486
contig057145-BurTARs.A018	contig045999-NyeTARs.A026	0.195	0.401	0.486
contig061091-BurTARs.A014	contig049540-BurTARs.A024	0.287	0.592	0.486
contig056020-BurTARs.A021	contig022363-TilTARs.A044	0.164	0.337	0.486
contig056023-BurTARs.A019	contig022383-TilTARs.A054	0.275	0.565	0.486
contig056023-BurTARs.A019	contig056134-TilTARs.A058	0.398	0.820	0.486
contig035377-NyeTARs.A023	contig022378-TilTARs.A051	0.250	0.514	0.486
contig060292-NyeTARs.A027	contig066691-ZebTARs.A015	0.410	0.843	0.486
contig056023-BurTARs.A019	contig023443-TilTAR.A005	0.436	0.897	0.486
contig045999-NyeTARs.A026	contig022365-TilTARs.A045	0.187	0.384	0.486
contig022363-TilTARs.A044	contig022379-TilTARs.A052	0.232	0.477	0.486
contig022363-TilTARs.A044	contig045088-TilTARs.A057	0.388	0.797	0.486
contig022337-TilTARs.A031	contig022379-TilTARs.A052	0.261	0.537	0.486
contig056021-BurTARs.A020	contig045088-TilTARs.A057	0.360	0.741	0.486
contig046014-NyeTARs.A024	contig022375-TilTARs.A048	0.139	0.286	0.486
contig022382-TilTARs.A053	contig062676-ZebTARs.A019	0.158	0.325	0.486
contig007518-TilTARs.A019	contig022382-TilTARs.A053	0.359	0.739	0.486
contig007518-TilTARs.A019	contig022379-TilTARs.A052	0.340	0.699	0.486
contig086344-BriTARs.A020	contig056020-BurTARs.A021	0.388	0.798	0.486
contig022355-TilTARs.A039	contig061410-ZebTARs.A021	0.256	0.526	0.486
contig084880-BriTARs.A016	contig086337-BriTARs.A019	0.393	0.808	0.486
contig056020-BurTARs.A021	contig061410-ZebTARs.A021	0.252	0.518	0.487
contig022363-TilTARs.A043	contig030464-ZebTARs.A025	0.183	0.375	0.487
contig035375-NyeTARs.A013	contig022356-TilTARs.A040	0.276	0.566	0.487
contig084868-BriTARs.A014	contig022382-TilTARs.A053	0.175	0.360	0.487
contig022349-TilTARs.A035	contig062677-ZebTARs.A018	0.309	0.634	0.487
contig049540-BurTARs.A024	contig030445-ZebTARs.A026	0.158	0.325	0.487
contig022363-TilTARs.A044	contig066285-ZebTARs.A016	0.205	0.421	0.487
contig049540-BurTARs.A024	contig007518-TilTARs.A019	0.369	0.758	0.487

contig086344-BriTARs.A020	contig022353-TilTARs.A036	0.367	0.754	0.487
contig022320-TilTARs.A059	contig062677-ZebTARs.A018	0.268	0.550	0.487
contig082565-BriTARs.A022	contig066691-ZebTARs.A015	0.279	0.573	0.487
contig022353-TilTARs.A036	contig022368-TilTARs.A046	0.128	0.262	0.487
contig061091-BurTARs.A014	contig060292-NyeTARs.A027	0.410	0.842	0.487
contig084880-BriTARs.A016	contig053139-ZebTARs.A023	0.386	0.793	0.487
contig022362-TilTARs.A042	contig061417-ZebTARs.A020	0.157	0.323	0.487
contig022354-TilTARs.A037	contig022378-TilTARs.A051	0.224	0.459	0.487
contig082565-BriTARs.A022	contig062677-ZebTARs.A018	0.163	0.334	0.487
contig056020-BurTARs.A021	contig035381-NyeTARs.A015	0.262	0.538	0.487
contig084886-BriTARs.A017	contig046010-NyeTARs.A019	0.024	0.050	0.487
contig022363-TilTARs.A043	contig022365-TilTARs.A045	0.135	0.277	0.487
contig058002-NyeTARs.A025	contig022324-TilTARs.A027	0.374	0.768	0.487
contig059673-BurTARs.A016	contig022390-TilTARs.A056	0.356	0.731	0.487
contig049540-BurTARs.A024	contig030471-ZebTARs.A024	0.284	0.582	0.487
contig056200-NyeTARs.A029	contig007524-TilTARs.A026	0.350	0.719	0.487
contig056020-BurTARs.A021	contig042499-NyeTARs.A028	0.373	0.766	0.487
contig084880-BriTARs.A016	contig007524-TilTARs.A026	0.399	0.818	0.487
contig022383-TilTARs.A054	contig066691-ZebTARs.A015	0.293	0.600	0.487
contig046011-NyeTARs.A016	contig022356-TilTARs.A040	0.236	0.484	0.488
contig045999-NyeTARs.A026	contig022368-TilTARs.A046	0.193	0.396	0.488
contig007524-TilTARs.A026	contig022377-TilTARs.A050	0.354	0.726	0.488
contig061091-BurTARs.A014	contig022357-TilTARs.A041	0.144	0.295	0.488
contig035375-NyeTARs.A013	contig022341-TilTARs.A032	0.273	0.561	0.488
contig059673-BurTARs.A016	contig022365-TilTARs.A045	0.391	0.803	0.488
contig022378-TilTARs.A051	contig066890-ZebTARs.A014	0.237	0.487	0.488
contig007524-TilTARs.A026	contig022353-TilTARs.A036	0.390	0.799	0.488
contig007518-TilTARs.A019	contig022378-TilTARs.A051	0.375	0.769	0.488
contig046010-NyeTARs.A019	contig022375-TilTARs.A048	0.167	0.342	0.488
contig059673-BurTARs.A016	contig022345-TilTARs.A034	0.358	0.734	0.488
contig042499-NyeTARs.A028	contig022330-TilTARs.A028	0.365	0.748	0.488
contig022337-TilTARs.A031	contig022341-TilTARs.A032	0.148	0.303	0.488
contig035376-NyeTARs.A014	contig062676-ZebTARs.A019	0.110	0.226	0.488
contig056021-BurTARs.A020	contig035377-NyeTARs.A023	0.261	0.535	0.488
contig086344-BriTARs.A020	contig022390-TilTARs.A055	0.398	0.816	0.488
contig038663-NyeTAR.A005	contig022390-TilTARs.A055	0.431	0.882	0.488
contig022368-TilTARs.A046	contig022379-TilTARs.A052	0.250	0.512	0.488
contig042499-NyeTARs.A028	contig022390-TilTARs.A055	0.362	0.741	0.488
contig057301-BurTARs.A017	contig022390-TilTARs.A055	0.362	0.741	0.488
contig007518-TilTARs.A019	contig061417-ZebTARs.A020	0.373	0.763	0.488
contig035375-NyeTARs.A013	contig035381-NyeTARs.A015	0.068	0.139	0.488
contig086337-BriTARs.A019	contig045999-NyeTARs.A026	0.398	0.815	0.488
contig056200-NyeTARs.A029	contig066691-ZebTARs.A015	0.293	0.601	0.489
contig022349-TilTARs.A035	contig022375-TilTARs.A048	0.167	0.343	0.489
contig020038-BurTAR.A002	contig056023-BurTARs.A019	0.452	0.926	0.489
contig007524-TilTARs.A026	contig022324-TilTARs.A027	0.373	0.764	0.489
contig035381-NyeTARs.A015	contig056200-NyeTARs.A029	0.162	0.331	0.489
contig059673-BurTARs.A016	contig049540-BurTARs.A024	0.358	0.733	0.489
contig007524-TilTARs.A026	contig022375-TilTARs.A048	0.339	0.694	0.489
contig062039-NyeTARs.A022	contig022349-TilTARs.A035	0.286	0.584	0.489
contig022353-TilTARs.A036	contig066890-ZebTARs.A014	0.276	0.564	0.489
contig035381-NyeTARs.A015	contig030471-ZebTARs.A024	0.262	0.537	0.489
contig056020-BurTARs.A021	contig049540-BurTARs.A024	0.289	0.591	0.489

contig061410-ZebTARs.A021	contig030440-ZebTARs.A029	0.135	0.276	0.489
contig084880-BriTARs.A016	contig058002-NyeTARs.A025	0.390	0.798	0.489
contig086344-BriTARs.A020	contig022362-TilTARs.A042	0.367	0.750	0.489
contig045999-NyeTARs.A026	contig053139-ZebTARs.A023	0.391	0.800	0.489
contig022363-TilTARs.A044	contig061417-ZebTARs.A020	0.170	0.348	0.489
contig046013-NyeTARs.A021	contig022357-TilTARs.A041	0.149	0.305	0.489
contig042499-NyeTARs.A028	contig022334-TilTARs.A029	0.362	0.740	0.489
contig084887-BriTARs.A018	contig022390-TilTARs.A055	0.235	0.480	0.489
contig035377-NyeTARs.A023	contig022353-TilTARs.A036	0.299	0.612	0.490
contig022355-TilTARs.A039	contig022390-TilTARs.A056	0.272	0.555	0.490
contig056021-BurTARs.A020	contig022357-TilTARs.A041	0.140	0.286	0.490
contig084876-BriTARs.A015	contig061433-BurTARs.A013	0.279	0.569	0.490
contig061433-BurTARs.A013	contig062677-ZebTARs.A018	0.310	0.633	0.490
contig041024-BurTARs.A026	contig022334-TilTARs.A030	0.171	0.350	0.490
contig049534-BurTARs.A025	contig034854-BurTARs.A027	0.288	0.589	0.490
contig084880-BriTARs.A016	contig022330-TilTARs.A028	0.186	0.380	0.490
contig059673-BurTARs.A016	contig030464-ZebTARs.A025	0.374	0.763	0.490
contig060105-NyeTARs.A018	contig022343-TilTARs.A033	0.347	0.708	0.490
contig082565-BriTARs.A022	contig022320-TilTARs.A059	0.245	0.499	0.490
contig056021-BurTARs.A020	contig042499-NyeTARs.A028	0.355	0.725	0.490
contig035376-NyeTARs.A014	contig046014-NyeTARs.A024	0.242	0.494	0.490
contig057305-BurTARs.A031	contig022355-TilTARs.A039	0.417	0.852	0.490
contig046013-NyeTARs.A021	contig022368-TilTARs.A047	0.162	0.331	0.490
contig059673-BurTARs.A016	contig062677-ZebTARs.A018	0.360	0.735	0.490
contig022337-TilTARs.A031	contig066890-ZebTARs.A014	0.289	0.590	0.490
contig084876-BriTARs.A015	contig082565-BriTARs.A022	0.142	0.290	0.490
contig060707-BurTARs.A015	contig022343-TilTARs.A033	0.341	0.696	0.490
contig035375-NyeTARs.A013	contig022377-TilTARs.A050	0.273	0.557	0.490
contig039639-TilTAR.A001	contig040586-ZebTAR.A002	0.042	0.086	0.490
contig060292-NyeTARs.A027	contig066285-ZebTARs.A016	0.448	0.914	0.490
contig046013-NyeTARs.A021	contig061417-ZebTARs.A020	0.127	0.258	0.490
contig007524-TilTARs.A026	contig022390-TilTARs.A055	0.346	0.706	0.490
contig022349-TilTARs.A035	contig022382-TilTARs.A053	0.298	0.608	0.490
contig056020-BurTARs.A021	contig045088-TilTARs.A057	0.379	0.772	0.490
contig059673-BurTARs.A016	contig022353-TilTARs.A036	0.401	0.817	0.490
contig084880-BriTARs.A016	contig022355-TilTARs.A039	0.182	0.371	0.490
contig056023-BurTARs.A019	contig055697-BurTARs.A022	0.403	0.821	0.491
contig061091-BurTARs.A014	contig062677-ZebTARs.A018	0.288	0.588	0.491
contig041024-BurTARs.A026	contig056200-NyeTARs.A029	0.265	0.541	0.491
contig059673-BurTARs.A016	contig045999-NyeTARs.A026	0.400	0.815	0.491
contig056023-BurTARs.A019	contig022368-TilTARs.A047	0.204	0.415	0.491
contig035381-NyeTARs.A015	contig022368-TilTARs.A046	0.255	0.521	0.491
contig022368-TilTARs.A047	contig022390-TilTARs.A055	0.267	0.543	0.491
contig022357-TilTARs.A041	contig022378-TilTARs.A051	0.272	0.554	0.491
contig082565-BriTARs.A022	contig060292-NyeTARs.A027	0.355	0.723	0.491
contig049540-BurTARs.A024	contig046014-NyeTARs.A024	0.268	0.546	0.491
contig039640-TilTAR.A002	contig039640-TilTAR.A003	0.044	0.089	0.491
contig084876-BriTARs.A015	contig022377-TilTARs.A050	0.259	0.527	0.491
contig056021-BurTARs.A020	contig007524-TilTARs.A026	0.336	0.684	0.491
contig056021-BurTARs.A020	contig066285-ZebTARs.A016	0.165	0.336	0.491
contig059673-BurTARs.A016	contig057148-BurTARs.A028	0.370	0.754	0.491
contig042499-NyeTARs.A028	contig022365-TilTARs.A045	0.384	0.782	0.491
contig057148-BurTARs.A028	contig007524-TilTARs.A026	0.358	0.728	0.491

contig007518-TilTARs.A019	contig022354-TilTARs.A037	0.374	0.761	0.491
contig007524-TilTARs.A026	contig066691-ZebTARs.A015	0.370	0.752	0.491
contig056021-BurTARs.A020	contig045999-NyeTARs.A026	0.188	0.382	0.491
contig056023-BurTARs.A019	contig007512-TilTARs.A024	0.385	0.783	0.492
contig062039-NyeTARs.A022	contig022320-TilTARs.A059	0.254	0.516	0.492
contig056200-NyeTARs.A029	contig007518-TilTARs.A019	0.370	0.753	0.492
contig086344-BriTARs.A020	contig045999-NyeTARs.A026	0.395	0.803	0.492
contig056023-BurTARs.A019	contig022345-TilTARs.A034	0.187	0.381	0.492
contig084880-BriTARs.A016	contig066285-ZebTARs.A016	0.190	0.386	0.492
contig046013-NyeTARs.A021	contig022368-TilTARs.A046	0.141	0.286	0.492
contig049540-BurTARs.A024	contig046007-NyeTARs.A017	0.295	0.600	0.492
contig007524-TilTARs.A026	contig022363-TilTARs.A043	0.368	0.747	0.492
contig035376-NyeTARs.A014	contig022365-TilTARs.A045	0.266	0.542	0.492
contig007518-TilTARs.A019	contig007524-TilTARs.A026	0.251	0.510	0.492
contig046014-NyeTARs.A024	contig045999-NyeTARs.A026	0.176	0.358	0.492
contig084876-BriTARs.A015	contig022354-TilTARs.A037	0.229	0.466	0.492
contig056200-NyeTARs.A029	contig022368-TilTARs.A047	0.296	0.602	0.492
contig022324-TilTARs.A027	contig030445-ZebTARs.A026	0.274	0.557	0.492
contig062677-ZebTARs.A018	contig030471-ZebTARs.A024	0.285	0.578	0.492
contig056200-NyeTARs.A029	contig030445-ZebTARs.A026	0.160	0.325	0.492
contig060105-NyeTARs.A018	contig022353-TilTARs.A036	0.362	0.736	0.492
contig061977-BurTARs.A012	contig034854-BurTARs.A027	0.154	0.312	0.492
contig041024-BurTARs.A026	contig022378-TilTARs.A051	0.247	0.503	0.492
contig086351-BriTARs.A021	contig082565-BriTARs.A022	0.365	0.741	0.492
contig045999-NyeTARs.A026	contig022363-TilTARs.A044	0.220	0.447	0.492
contig061977-BurTARs.A012	contig022363-TilTARs.A043	0.272	0.551	0.492
contig046011-NyeTARs.A016	contig022349-TilTARs.A035	0.229	0.465	0.492
contig022354-TilTARs.A037	contig022377-TilTARs.A050	0.191	0.389	0.492
contig058002-NyeTARs.A025	contig030471-ZebTARs.A024	0.359	0.728	0.492
contig086351-BriTARs.A021	contig066691-ZebTARs.A015	0.417	0.846	0.493
contig022383-TilTARs.A054	contig045088-TilTARs.A057	0.356	0.722	0.493
contig022368-TilTARs.A047	contig066890-ZebTARs.A014	0.292	0.593	0.493
contig057305-BurTARs.A031	contig066691-ZebTARs.A015	0.410	0.832	0.493
contig042499-NyeTARs.A028	contig022357-TilTARs.A041	0.387	0.785	0.493
contig084886-BriTARs.A017	contig035375-NyeTARs.A013	0.272	0.551	0.493
contig022345-TilTARs.A034	contig022378-TilTARs.A051	0.244	0.496	0.493
contig046007-NyeTARs.A017	contig056200-NyeTARs.A029	0.292	0.592	0.493
contig057301-BurTARs.A017	contig022341-TilTARs.A032	0.373	0.757	0.493
contig022334-TilTARs.A030	contig022354-TilTARs.A037	0.151	0.305	0.493
contig084886-BriTARs.A017	contig022390-TilTARs.A056	0.271	0.550	0.493
contig061977-BurTARs.A012	contig022378-TilTARs.A051	0.202	0.410	0.493
contig061977-BurTARs.A012	contig046007-NyeTARs.A017	0.269	0.546	0.493
contig046013-NyeTARs.A021	contig042499-NyeTARs.A028	0.353	0.715	0.493
contig084876-BriTARs.A015	contig022357-TilTARs.A041	0.273	0.554	0.493
contig022337-TilTARs.A031	contig022382-TilTARs.A053	0.288	0.584	0.493
contig057145-BurTARs.A018	contig045088-TilTARs.A057	0.383	0.777	0.493
contig056021-BurTARs.A020	contig035375-NyeTARs.A013	0.272	0.551	0.493
contig084880-BriTARs.A016	contig022363-TilTARs.A044	0.218	0.442	0.493
contig061977-BurTARs.A012	contig007518-TilTARs.A019	0.334	0.678	0.493
contig035377-NyeTARs.A023	contig022354-TilTARs.A037	0.248	0.504	0.493
contig056200-NyeTARs.A029	contig022382-TilTARs.A053	0.013	0.026	0.494
contig086351-BriTARs.A021	contig061091-BurTARs.A014	0.417	0.844	0.494
contig022354-TilTARs.A037	contig066890-ZebTARs.A014	0.254	0.514	0.494

contig022355-TilTARs.A039	contig030445-ZebTARs.A026	0.251	0.509	0.494
contig057305-BurTARs.A031	contig030440-ZebTARs.A029	0.358	0.726	0.494
contig082565-BriTARs.A022	contig022368-TilTARs.A046	0.262	0.532	0.494
contig061091-BurTARs.A014	contig057305-BurTARs.A031	0.410	0.830	0.494
contig045088-TilTARs.A057	contig030445-ZebTARs.A026	0.354	0.716	0.494
contig046011-NyeTARs.A016	contig007520-TilTARs.A025	0.409	0.828	0.494
contig046013-NyeTARs.A021	contig035377-NyeTARs.A023	0.286	0.578	0.494
contig057305-BurTARs.A031	contig022345-TilTARs.A034	0.385	0.778	0.494
contig046011-NyeTARs.A016	contig022368-TilTARs.A046	0.229	0.463	0.494
contig035375-NyeTARs.A013	contig007518-TilTARs.A019	0.356	0.719	0.494
contig082565-BriTARs.A022	contig035376-NyeTARs.A014	0.014	0.027	0.494
contig022337-TilTARs.A031	contig066691-ZebTARs.A015	0.117	0.237	0.494
contig035377-NyeTARs.A023	contig061417-ZebTARs.A020	0.263	0.532	0.495
contig022365-TilTARs.A045	contig062677-ZebTARs.A018	0.300	0.606	0.495
contig061091-BurTARs.A014	contig022383-TilTARs.A054	0.289	0.583	0.495
contig066691-ZebTARs.A015	contig030445-ZebTARs.A026	0.270	0.546	0.495
contig007524-TilTARs.A026	contig030464-ZebTARs.A025	0.366	0.739	0.495
contig061433-BurTARs.A013	contig059673-BurTARs.A016	0.410	0.828	0.495
contig084876-BriTARs.A015	contig062677-ZebTARs.A018	0.177	0.357	0.495
contig022349-TilTARs.A035	contig022368-TilTARs.A047	0.166	0.336	0.495
contig060707-BurTARs.A015	contig061417-ZebTARs.A020	0.339	0.685	0.495
contig082565-BriTARs.A022	contig034854-BurTARs.A027	0.056	0.112	0.495
contig022337-TilTARs.A031	contig022378-TilTARs.A051	0.262	0.530	0.495
contig086351-BriTARs.A021	contig022345-TilTARs.A034	0.385	0.777	0.495
contig022334-TilTARs.A029	contig022353-TilTARs.A036	0.129	0.260	0.495
contig049540-BurTARs.A024	contig022368-TilTARs.A047	0.294	0.594	0.495
contig046007-NyeTARs.A017	contig061410-ZebTARs.A021	0.267	0.538	0.496
contig084880-BriTARs.A016	contig060105-NyeTARs.A018	0.393	0.793	0.496
contig034854-BurTARs.A027	contig022356-TilTARs.A040	0.276	0.556	0.496
contig045999-NyeTARs.A026	contig066285-ZebTARs.A016	0.189	0.381	0.496
contig086337-BriTARs.A019	contig056023-BurTARs.A019	0.398	0.804	0.496
contig046013-NyeTARs.A021	contig007520-TilTARs.A025	0.361	0.728	0.496
contig056200-NyeTARs.A029	contig022320-TilTARs.A059	0.267	0.538	0.496
contig046011-NyeTARs.A016	contig022341-TilTARs.A032	0.240	0.485	0.496
contig057148-BurTARs.A028	contig022363-TilTARs.A044	0.186	0.376	0.496
contig061091-BurTARs.A014	contig056200-NyeTARs.A029	0.289	0.584	0.496
contig022363-TilTARs.A044	contig022368-TilTARs.A046	0.141	0.283	0.496
contig082565-BriTARs.A022	contig022383-TilTARs.A054	0.106	0.214	0.496
contig056023-BurTARs.A019	contig053139-ZebTARs.A023	0.391	0.789	0.496
contig022356-TilTARs.A040	contig022378-TilTARs.A051	0.267	0.537	0.496
contig035375-NyeTARs.A013	contig022334-TilTARs.A029	0.275	0.554	0.496
contig034854-BurTARs.A027	contig022349-TilTARs.A035	0.286	0.575	0.496
contig007518-TilTARs.A019	contig022341-TilTARs.A032	0.382	0.769	0.496
contig022353-TilTARs.A036	contig022357-TilTARs.A041	0.139	0.281	0.496
contig022353-TilTARs.A036	contig062676-ZebTARs.A019	0.275	0.554	0.496
contig022334-TilTARs.A030	contig045088-TilTARs.A057	0.374	0.754	0.496
contig022363-TilTARs.A044	contig066691-ZebTARs.A015	0.170	0.343	0.496
contig034854-BurTARs.A027	contig066285-ZebTARs.A016	0.290	0.584	0.496
contig057301-BurTARs.A017	contig041024-BurTARs.A026	0.349	0.704	0.496
contig084876-BriTARs.A015	contig035376-NyeTARs.A014	0.143	0.288	0.496
contig084880-BriTARs.A016	contig061417-ZebTARs.A020	0.194	0.391	0.497
contig084887-BriTARs.A018	contig060292-NyeTARs.A027	0.406	0.818	0.497
contig057305-BurTARs.A031	contig022363-TilTARs.A043	0.459	0.925	0.497

contig045999-NyeTARs.A026	contig022357-TilTARs.A041	0.189	0.381	0.497
contig084887-BriTARs.A018	contig007524-TilTARs.A026	0.360	0.724	0.497
contig035375-NyeTARs.A013	contig058002-NyeTARs.A025	0.333	0.671	0.497
contig022363-TilTARs.A043	contig022375-TilTARs.A048	0.132	0.266	0.497
contig035381-NyeTARs.A015	contig022353-TilTARs.A036	0.290	0.583	0.497
contig082565-BriTARs.A022	contig022365-TilTARs.A045	0.271	0.546	0.497
contig035377-NyeTARs.A023	contig045999-NyeTARs.A026	0.290	0.584	0.497
contig060105-NyeTARs.A018	contig042499-NyeTARs.A028	0.323	0.649	0.497
contig084887-BriTARs.A018	contig022383-TilTARs.A054	0.292	0.588	0.497
contig057305-BurTARs.A031	contig066285-ZebTARs.A016	0.448	0.901	0.497
contig022355-TilTARs.A039	contig045088-TilTARs.A057	0.380	0.765	0.497
contig034854-BurTARs.A027	contig007518-TilTARs.A019	0.370	0.744	0.497
contig007518-TilTARs.A019	contig022363-TilTARs.A043	0.401	0.807	0.497
contig059673-BurTARs.A016	contig056200-NyeTARs.A029	0.360	0.725	0.497
contig042499-NyeTARs.A028	contig022375-TilTARs.A048	0.376	0.757	0.497
contig022368-TilTARs.A046	contig030445-ZebTARs.A026	0.252	0.506	0.497
contig046013-NyeTARs.A021	contig022356-TilTARs.A040	0.128	0.258	0.497
contig066285-ZebTARs.A016	contig061417-ZebTARs.A020	0.168	0.338	0.497
contig084876-BriTARs.A015	contig022324-TilTARs.A027	0.271	0.545	0.497
contig056200-NyeTARs.A029	contig030471-ZebTARs.A024	0.286	0.574	0.498
contig022375-TilTARs.A048	contig022390-TilTARs.A056	0.244	0.491	0.498
contig046013-NyeTARs.A021	contig022375-TilTARs.A048	0.155	0.312	0.498
contig022362-TilTARs.A042	contig030471-ZebTARs.A024	0.150	0.301	0.498
contig086351-BriTARs.A021	contig066285-ZebTARs.A016	0.454	0.912	0.498
contig041024-BurTARs.A026	contig022382-TilTARs.A053	0.259	0.520	0.498
contig022375-TilTARs.A048	contig066890-ZebTARs.A014	0.256	0.514	0.498
contig056021-BurTARs.A020	contig022363-TilTARs.A044	0.177	0.355	0.498
contig022356-TilTARs.A040	contig045088-TilTARs.A057	0.381	0.764	0.498
contig086337-BriTARs.A019	contig055697-BurTARs.A022	0.016	0.031	0.498
contig084887-BriTARs.A018	contig062677-ZebTARs.A018	0.286	0.574	0.498
contig061433-BurTARs.A013	contig049540-BurTARs.A024	0.309	0.620	0.498
contig022345-TilTARs.A034	contig022353-TilTARs.A036	0.149	0.298	0.498
contig035376-NyeTARs.A014	contig042499-NyeTARs.A028	0.358	0.717	0.499
contig060292-NyeTARs.A027	contig022345-TilTARs.A034	0.386	0.774	0.499
contig022343-TilTARs.A033	contig022368-TilTARs.A047	0.150	0.301	0.499
contig022355-TilTARs.A039	contig030440-ZebTARs.A029	0.266	0.533	0.499
contig062039-NyeTARs.A022	contig046014-NyeTARs.A024	0.259	0.519	0.499
contig060292-NyeTARs.A027	contig030440-ZebTARs.A029	0.359	0.721	0.499
contig086344-BriTARs.A020	contig056023-BurTARs.A019	0.395	0.792	0.499
contig046007-NyeTARs.A017	contig022383-TilTARs.A054	0.278	0.556	0.499
contig082565-BriTARs.A022	contig049540-BurTARs.A024	0.161	0.323	0.499
contig084886-BriTARs.A017	contig034854-BurTARs.A027	0.278	0.557	0.499
contig022357-TilTARs.A041	contig045088-TilTARs.A057	0.386	0.773	0.499
contig057301-BurTARs.A017	contig022363-TilTARs.A044	0.387	0.775	0.499
contig022362-TilTARs.A042	contig066691-ZebTARs.A015	0.162	0.325	0.499
contig062039-NyeTARs.A022	contig022330-TilTARs.A028	0.252	0.506	0.499
contig025313-BriTAR.A002	contig032272-NyeTAR.A004	0.011	0.022	0.499
contig057301-BurTARs.A017	contig022365-TilTARs.A045	0.384	0.770	0.499
contig057145-BurTARs.A018	contig042499-NyeTARs.A028	0.377	0.755	0.499
contig082565-BriTARs.A022	contig030440-ZebTARs.A029	0.070	0.140	0.499
contig022378-TilTARs.A051	contig061417-ZebTARs.A020	0.246	0.494	0.499
contig084876-BriTARs.A015	contig022382-TilTARs.A053	0.165	0.331	0.499
contig084868-BriTARs.A014	contig057301-BurTARs.A017	0.373	0.748	0.499

contig056020-BurTARs.A021	contig022334-TilTARs.A030	0.183	0.367	0.499
contig035376-NyeTARs.A014	contig022368-TilTARs.A047	0.277	0.555	0.499
contig030445-ZebTARs.A026	contig030440-ZebTARs.A029	0.070	0.140	0.500
contig046013-NyeTARs.A021	contig045088-TilTARs.A057	0.360	0.721	0.500
contig007520-TilTARs.A025	contig066285-ZebTARs.A016	0.386	0.773	0.500
contig056020-BurTARs.A021	contig056200-NyeTARs.A029	0.291	0.583	0.500
contig084887-BriTARs.A018	contig082565-BriTARs.A022	0.273	0.547	0.500
contig084876-BriTARs.A015	contig030440-ZebTARs.A029	0.145	0.289	0.500
contig061977-BurTARs.A012	contig066691-ZebTARs.A015	0.267	0.535	0.500
contig022330-TilTARs.A028	contig022349-TilTARs.A035	0.162	0.325	0.500
contig045999-NyeTARs.A026	contig062676-ZebTARs.A019	0.273	0.546	0.500
contig022355-TilTARs.A039	contig022379-TilTARs.A052	0.251	0.503	0.500
contig034854-BurTARs.A027	contig007524-TilTARs.A026	0.353	0.706	0.500
contig034854-BurTARs.A027	contig022378-TilTARs.A051	0.245	0.490	0.500
contig082565-BriTARs.A022	contig022368-TilTARs.A047	0.276	0.551	0.500
contig059673-BurTARs.A016	contig030440-ZebTARs.A029	0.353	0.706	0.500
contig084886-BriTARs.A017	contig030445-ZebTARs.A026	0.250	0.500	0.500
contig084880-BriTARs.A016	contig062039-NyeTARs.A022	0.302	0.603	0.500
contig046011-NyeTARs.A016	contig046007-NyeTARs.A017	0.246	0.492	0.500
contig056200-NyeTARs.A029	contig022349-TilTARs.A035	0.309	0.616	0.501
contig062039-NyeTARs.A022	contig045999-NyeTARs.A026	0.303	0.605	0.501
contig059673-BurTARs.A016	contig046014-NyeTARs.A024	0.356	0.710	0.501
contig045999-NyeTARs.A026	contig022355-TilTARs.A039	0.182	0.363	0.501
contig022353-TilTARs.A036	contig045088-TilTARs.A057	0.356	0.710	0.501
contig022357-TilTARs.A041	contig066890-ZebTARs.A014	0.277	0.552	0.501
contig082565-BriTARs.A022	contig061091-BurTARs.A014	0.275	0.549	0.501
contig084868-BriTARs.A014	contig046007-NyeTARs.A017	0.289	0.577	0.501
contig022334-TilTARs.A030	contig030471-ZebTARs.A024	0.186	0.372	0.501
contig022334-TilTARs.A030	contig022320-TilTARs.A059	0.172	0.343	0.501
contig046013-NyeTARs.A021	contig062677-ZebTARs.A018	0.297	0.593	0.501
contig035375-NyeTARs.A013	contig022343-TilTARs.A033	0.264	0.526	0.501
contig062039-NyeTARs.A022	contig022368-TilTARs.A047	0.286	0.571	0.501
contig046014-NyeTARs.A024	contig056200-NyeTARs.A029	0.270	0.539	0.502
contig057301-BurTARs.A017	contig022355-TilTARs.A039	0.379	0.756	0.502
contig059673-BurTARs.A016	contig056021-BurTARs.A020	0.347	0.691	0.502
contig022363-TilTARs.A043	contig030471-ZebTARs.A024	0.164	0.328	0.502
contig049540-BurTARs.A024	contig022354-TilTARs.A037	0.257	0.512	0.502
contig035375-NyeTARs.A013	contig061417-ZebTARs.A020	0.273	0.544	0.502
contig060105-NyeTARs.A018	contig045088-TilTARs.A057	0.329	0.656	0.502
contig086351-BriTARs.A021	contig022324-TilTARs.A027	0.431	0.859	0.502
contig057301-BurTARs.A017	contig022330-TilTARs.A028	0.370	0.737	0.502
contig046013-NyeTARs.A021	contig058002-NyeTARs.A025	0.362	0.721	0.502
contig035376-NyeTARs.A014	contig022363-TilTARs.A044	0.236	0.470	0.502
contig082565-BriTARs.A022	contig046014-NyeTARs.A024	0.247	0.491	0.502
contig056023-BurTARs.A019	contig045999-NyeTARs.A026	0.006	0.011	0.502
contig046014-NyeTARs.A024	contig045088-TilTARs.A057	0.350	0.697	0.502
contig086344-BriTARs.A020	contig061417-ZebTARs.A020	0.347	0.691	0.502
contig035376-NyeTARs.A014	contig066691-ZebTARs.A015	0.277	0.551	0.502
contig022330-TilTARs.A028	contig022390-TilTARs.A056	0.261	0.520	0.502
contig035377-NyeTARs.A023	contig062677-ZebTARs.A018	0.191	0.381	0.502
contig084887-BriTARs.A018	contig022334-TilTARs.A030	0.179	0.356	0.503
contig022365-TilTARs.A045	contig030440-ZebTARs.A029	0.290	0.578	0.503
contig056023-BurTARs.A019	contig062039-NyeTARs.A022	0.298	0.592	0.503

contig034854-BurTARs.A027	contig022330-TilTARs.A028	0.274	0.546	0.503
contig046011-NyeTARs.A016	contig022390-TilTARs.A055	0.294	0.584	0.503
contig022378-TilTARs.A051	contig030440-ZebTARs.A029	0.251	0.499	0.503
contig042499-NyeTARs.A028	contig061417-ZebTARs.A020	0.359	0.714	0.503
contig084887-BriTARs.A018	contig030445-ZebTARs.A026	0.262	0.521	0.503
contig057301-BurTARs.A017	contig022383-TilTARs.A054	0.367	0.730	0.503
contig046011-NyeTARs.A016	contig046014-NyeTARs.A024	0.221	0.440	0.503
contig084868-BriTARs.A014	contig042499-NyeTARs.A028	0.374	0.744	0.503
contig046007-NyeTARs.A017	contig022382-TilTARs.A053	0.284	0.565	0.503
contig022382-TilTARs.A053	contig022320-TilTARs.A059	0.260	0.517	0.503
contig057301-BurTARs.A017	contig022334-TilTARs.A029	0.367	0.729	0.503
contig022357-TilTARs.A041	contig022383-TilTARs.A054	0.272	0.539	0.504
contig061091-BurTARs.A014	contig059673-BurTARs.A016	0.375	0.744	0.504
contig057301-BurTARs.A017	contig035376-NyeTARs.A014	0.364	0.723	0.504
contig084887-BriTARs.A018	contig057305-BurTARs.A031	0.406	0.807	0.504
contig046011-NyeTARs.A016	contig022320-TilTARs.A059	0.220	0.437	0.504
contig084880-BriTARs.A016	contig030471-ZebTARs.A024	0.185	0.367	0.504
contig049540-BurTARs.A024	contig022365-TilTARs.A045	0.299	0.593	0.504
contig022368-TilTARs.A047	contig022378-TilTARs.A051	0.270	0.536	0.504
contig022363-TilTARs.A044	contig030471-ZebTARs.A024	0.180	0.357	0.504
contig061433-BurTARs.A013	contig035376-NyeTARs.A014	0.278	0.552	0.504
contig035375-NyeTARs.A013	contig022354-TilTARs.A037	0.257	0.511	0.504
contig056021-BurTARs.A020	contig062676-ZebTARs.A019	0.257	0.510	0.504
contig061977-BurTARs.A012	contig057301-BurTARs.A017	0.344	0.683	0.504
contig057305-BurTARs.A031	contig022324-TilTARs.A027	0.423	0.839	0.504
contig022353-TilTARs.A036	contig022382-TilTARs.A053	0.265	0.526	0.504
contig061091-BurTARs.A014	contig022363-TilTARs.A044	0.167	0.330	0.504
contig082565-BriTARs.A022	contig056200-NyeTARs.A029	0.163	0.323	0.504
contig056020-BurTARs.A021	contig062676-ZebTARs.A019	0.267	0.529	0.504
contig084886-BriTARs.A017	contig022375-TilTARs.A048	0.171	0.339	0.504
contig084876-BriTARs.A015	contig007518-TilTARs.A019	0.342	0.677	0.504
contig066890-ZebTARs.A014	contig066691-ZebTARs.A015	0.288	0.571	0.504
contig042499-NyeTARs.A028	contig022355-TilTARs.A039	0.377	0.747	0.504
contig056023-BurTARs.A019	contig022365-TilTARs.A045	0.186	0.368	0.504
contig022334-TilTARs.A029	contig022337-TilTARs.A031	0.119	0.236	0.504
contig007524-TilTARs.A026	contig061417-ZebTARs.A020	0.338	0.671	0.505
contig035376-NyeTARs.A014	contig030440-ZebTARs.A029	0.072	0.143	0.505
contig022357-TilTARs.A041	contig022379-TilTARs.A052	0.273	0.541	0.505
contig056021-BurTARs.A020	contig022378-TilTARs.A051	0.254	0.503	0.505
contig084887-BriTARs.A018	contig022362-TilTARs.A042	0.159	0.314	0.505
contig022349-TilTARs.A035	contig030471-ZebTARs.A024	0.148	0.293	0.505
contig061433-BurTARs.A013	contig066890-ZebTARs.A014	0.296	0.587	0.505
contig059673-BurTARs.A016	contig056020-BurTARs.A021	0.406	0.805	0.505
contig022353-TilTARs.A036	contig022379-TilTARs.A052	0.249	0.493	0.505
contig022324-TilTARs.A027	contig045088-TilTARs.A057	0.394	0.780	0.505
contig061977-BurTARs.A012	contig042499-NyeTARs.A028	0.339	0.670	0.505
contig084886-BriTARs.A017	contig035376-NyeTARs.A014	0.259	0.512	0.505
contig022365-TilTARs.A045	contig030445-ZebTARs.A026	0.266	0.526	0.505
contig022375-TilTARs.A048	contig030464-ZebTARs.A025	0.173	0.342	0.506
contig049534-BurTARs.A025	contig007524-TilTARs.A026	0.384	0.759	0.506
contig042499-NyeTARs.A028	contig022378-TilTARs.A051	0.357	0.707	0.506
contig007518-TilTARs.A019	contig062677-ZebTARs.A018	0.376	0.744	0.506
contig035377-NyeTARs.A023	contig045088-TilTARs.A057	0.381	0.753	0.506

contig084880-BriTARs.A016	contig035381-NyeTARs.A015	0.298	0.588	0.506
contig062039-NyeTARs.A022	contig022375-TilTARs.A048	0.244	0.482	0.506
contig056020-BurTARs.A021	contig022382-TilTARs.A053	0.281	0.555	0.506
contig084876-BriTARs.A015	contig022355-TilTARs.A039	0.265	0.524	0.506
contig035375-NyeTARs.A013	contig022378-TilTARs.A051	0.253	0.499	0.506
contig045088-TilTARs.A057	contig062676-ZebTARs.A019	0.367	0.725	0.506
contig057301-BurTARs.A017	contig030445-ZebTARs.A026	0.352	0.695	0.507
contig084887-BriTARs.A018	contig086351-BriTARs.A021	0.416	0.821	0.507
contig022334-TilTARs.A029	contig066890-ZebTARs.A014	0.270	0.533	0.507
contig084876-BriTARs.A015	contig049540-BurTARs.A024	0.175	0.345	0.507
contig061977-BurTARs.A012	contig022353-TilTARs.A036	0.277	0.547	0.507
contig057301-BurTARs.A017	contig022378-TilTARs.A051	0.358	0.706	0.507
contig022324-TilTARs.A027	contig022382-TilTARs.A053	0.297	0.585	0.507
contig022362-TilTARs.A042	contig066890-ZebTARs.A014	0.282	0.557	0.507
contig056023-BurTARs.A019	contig062676-ZebTARs.A019	0.273	0.539	0.507
contig022363-TilTARs.A043	contig066691-ZebTARs.A015	0.167	0.329	0.507
contig084886-BriTARs.A017	contig082565-BriTARs.A022	0.259	0.511	0.507
contig035375-NyeTARs.A013	contig022379-TilTARs.A052	0.144	0.283	0.507
contig022341-TilTARs.A032	contig022353-TilTARs.A036	0.149	0.293	0.507
contig061977-BurTARs.A012	contig061091-BurTARs.A014	0.263	0.519	0.507
contig061091-BurTARs.A014	contig022362-TilTARs.A042	0.159	0.313	0.507
contig022368-TilTARs.A047	contig022382-TilTARs.A053	0.289	0.571	0.507
contig084886-BriTARs.A017	contig022363-TilTARs.A043	0.199	0.393	0.507
contig022357-TilTARs.A041	contig022390-TilTARs.A056	0.269	0.530	0.507
contig022363-TilTARs.A044	contig030440-ZebTARs.A029	0.252	0.497	0.507
contig042499-NyeTARs.A028	contig030445-ZebTARs.A026	0.351	0.691	0.507
contig082565-BriTARs.A022	contig022375-TilTARs.A048	0.222	0.438	0.507
contig022362-TilTARs.A042	contig022379-TilTARs.A052	0.251	0.495	0.507
contig022362-TilTARs.A042	contig022378-TilTARs.A051	0.265	0.522	0.507
contig022334-TilTARs.A030	contig066691-ZebTARs.A015	0.191	0.376	0.507
contig007524-TilTARs.A026	contig030471-ZebTARs.A024	0.359	0.708	0.507
contig084887-BriTARs.A018	contig049540-BurTARs.A024	0.285	0.561	0.508
contig034854-BurTARs.A027	contig022334-TilTARs.A029	0.272	0.535	0.508
contig046007-NyeTARs.A017	contig022334-TilTARs.A030	0.191	0.377	0.508
contig057301-BurTARs.A017	contig060105-NyeTARs.A018	0.330	0.650	0.508
contig022378-TilTARs.A051	contig061410-ZebTARs.A021	0.205	0.403	0.508
contig035376-NyeTARs.A014	contig022355-TilTARs.A039	0.261	0.514	0.508
contig084887-BriTARs.A018	contig059673-BurTARs.A016	0.369	0.727	0.508
contig056021-BurTARs.A020	contig022353-TilTARs.A036	0.141	0.278	0.508
contig022334-TilTARs.A030	contig022343-TilTARs.A033	0.188	0.371	0.508
contig042499-NyeTARs.A028	contig022341-TilTARs.A032	0.378	0.744	0.508
contig061433-BurTARs.A013	contig022390-TilTARs.A056	0.290	0.571	0.508
contig046011-NyeTARs.A016	contig022383-TilTARs.A054	0.302	0.595	0.508
contig056023-BurTARs.A019	contig022368-TilTARs.A046	0.195	0.384	0.508
contig086351-BriTARs.A021	contig007518-TilTARs.A019	0.299	0.588	0.508
contig046014-NyeTARs.A024	contig007524-TilTARs.A026	0.341	0.670	0.508
contig062039-NyeTARs.A022	contig022368-TilTARs.A046	0.267	0.525	0.508
contig062676-ZebTARs.A019	contig030471-ZebTARs.A024	0.269	0.528	0.508
contig086351-BriTARs.A021	contig022341-TilTARs.A032	0.411	0.807	0.509
contig022337-TilTARs.A031	contig022390-TilTARs.A056	0.286	0.562	0.509
contig007518-TilTARs.A019	contig030440-ZebTARs.A029	0.360	0.707	0.509
contig046014-NyeTARs.A024	contig042499-NyeTARs.A028	0.352	0.691	0.509
contig045999-NyeTARs.A026	contig022356-TilTARs.A040	0.191	0.376	0.509

contig056020-BurTARs.A021	contig066890-ZebTARs.A014	0.284	0.559	0.509
contig060707-BurTARs.A015	contig045999-NyeTARs.A026	0.389	0.765	0.509
contig061433-BurTARs.A013	contig056200-NyeTARs.A029	0.311	0.612	0.509
contig060105-NyeTARs.A018	contig061417-ZebTARs.A020	0.345	0.679	0.509
contig046007-NyeTARs.A017	contig022378-TilTARs.A051	0.262	0.514	0.509
contig022330-TilTARs.A028	contig062676-ZebTARs.A019	0.259	0.509	0.509
contig035376-NyeTARs.A014	contig022362-TilTARs.A042	0.268	0.526	0.509
contig059673-BurTARs.A016	contig061417-ZebTARs.A020	0.345	0.677	0.509
contig084887-BriTARs.A018	contig066890-ZebTARs.A014	0.284	0.557	0.509
contig061091-BurTARs.A014	contig030445-ZebTARs.A026	0.266	0.522	0.509
contig045088-TilTARs.A057	contig061417-ZebTARs.A020	0.366	0.719	0.509
contig056023-BurTARs.A019	contig022355-TilTARs.A039	0.182	0.357	0.509
contig082565-BriTARs.A022	contig030445-ZebTARs.A026	0.019	0.037	0.510
contig022375-TilTARs.A048	contig045088-TilTARs.A057	0.380	0.745	0.510
contig082565-BriTARs.A022	contig022324-TilTARs.A027	0.282	0.553	0.510
contig022357-TilTARs.A041	contig030440-ZebTARs.A029	0.287	0.562	0.510
contig022353-TilTARs.A036	contig022363-TilTARs.A044	0.163	0.320	0.510
contig035381-NyeTARs.A015	contig046007-NyeTARs.A017	0.274	0.537	0.510
contig022362-TilTARs.A042	contig030440-ZebTARs.A029	0.283	0.555	0.510
contig035376-NyeTARs.A014	contig022324-TilTARs.A027	0.282	0.553	0.510
contig057301-BurTARs.A017	contig056021-BurTARs.A020	0.365	0.716	0.510
contig035381-NyeTARs.A015	contig046011-NyeTARs.A016	0.288	0.564	0.510
contig035376-NyeTARs.A014	contig022375-TilTARs.A048	0.222	0.434	0.510
contig049540-BurTARs.A024	contig046013-NyeTARs.A021	0.296	0.580	0.510
contig066890-ZebTARs.A014	contig061410-ZebTARs.A021	0.163	0.319	0.510
contig046013-NyeTARs.A021	contig022362-TilTARs.A042	0.163	0.320	0.510
contig057301-BurTARs.A017	contig057145-BurTARs.A018	0.387	0.757	0.510
contig084868-BriTARs.A014	contig046013-NyeTARs.A021	0.310	0.606	0.510
contig022334-TilTARs.A029	contig022349-TilTARs.A035	0.165	0.323	0.510
contig061433-BurTARs.A013	contig007520-TilTARs.A025	0.390	0.764	0.511
contig022362-TilTARs.A042	contig022363-TilTARs.A044	0.158	0.310	0.511
contig084886-BriTARs.A017	contig056020-BurTARs.A021	0.167	0.326	0.511
contig056023-BurTARs.A019	contig066285-ZebTARs.A016	0.190	0.372	0.511
contig054630-BurTARs.A023	contig022363-TilTARs.A043	0.392	0.767	0.511
contig056023-BurTARs.A019	contig035377-NyeTARs.A023	0.287	0.563	0.511
contig082565-BriTARs.A022	contig022362-TilTARs.A042	0.274	0.537	0.511
contig084876-BriTARs.A015	contig061977-BurTARs.A012	0.013	0.025	0.511
contig022354-TilTARs.A037	contig062677-ZebTARs.A018	0.260	0.508	0.511
contig056023-BurTARs.A019	contig046014-NyeTARs.A024	0.175	0.343	0.511
contig049540-BurTARs.A024	contig022349-TilTARs.A035	0.307	0.600	0.511
contig059673-BurTARs.A016	contig007518-TilTARs.A019	0.261	0.510	0.511
contig084887-BriTARs.A018	contig035376-NyeTARs.A014	0.268	0.523	0.511
contig046014-NyeTARs.A024	contig022334-TilTARs.A030	0.173	0.339	0.511
contig066691-ZebTARs.A015	contig061410-ZebTARs.A021	0.270	0.527	0.511
contig046014-NyeTARs.A024	contig022349-TilTARs.A035	0.143	0.280	0.511
contig022337-TilTARs.A031	contig022354-TilTARs.A037	0.192	0.374	0.511
contig056023-BurTARs.A019	contig022363-TilTARs.A044	0.222	0.434	0.511
contig022362-TilTARs.A042	contig022365-TilTARs.A045	0.110	0.214	0.511
contig035377-NyeTARs.A023	contig022383-TilTARs.A054	0.095	0.186	0.511
contig022363-TilTARs.A044	contig022378-TilTARs.A051	0.237	0.463	0.512
contig084880-BriTARs.A016	contig022334-TilTARs.A029	0.188	0.368	0.512
contig022357-TilTARs.A041	contig022368-TilTARs.A047	0.156	0.305	0.512
contig056020-BurTARs.A021	contig030440-ZebTARs.A029	0.273	0.533	0.512

contig084880-BriTARs.A016	contig049534-BurTARs.A025	0.190	0.371	0.512
contig084876-BriTARs.A015	contig056200-NyeTARs.A029	0.177	0.345	0.512
contig059673-BurTARs.A016	contig062039-NyeTARs.A022	0.346	0.676	0.512
contig061091-BurTARs.A014	contig066890-ZebTARs.A014	0.284	0.555	0.512
contig056021-BurTARs.A020	contig034854-BurTARs.A027	0.274	0.534	0.512
contig062039-NyeTARs.A022	contig022355-TilTARs.A039	0.261	0.510	0.512
contig082565-BriTARs.A022	contig022357-TilTARs.A041	0.282	0.551	0.512
contig082565-BriTARs.A022	contig030471-ZebTARs.A024	0.265	0.518	0.512
contig056020-BurTARs.A021	contig022390-TilTARs.A056	0.278	0.543	0.513
contig042499-NyeTARs.A028	contig022334-TilTARs.A030	0.378	0.737	0.513
contig022375-TilTARs.A048	contig022320-TilTARs.A059	0.139	0.271	0.513
contig060292-NyeTARs.A027	contig022324-TilTARs.A027	0.423	0.825	0.513
contig084886-BriTARs.A017	contig066890-ZebTARs.A014	0.275	0.536	0.513
contig056023-BurTARs.A019	contig056021-BurTARs.A020	0.189	0.369	0.513
contig082565-BriTARs.A022	contig061433-BurTARs.A013	0.281	0.548	0.513
contig035376-NyeTARs.A014	contig030471-ZebTARs.A024	0.261	0.508	0.513
contig042499-NyeTARs.A028	contig022356-TilTARs.A040	0.381	0.741	0.513
contig046007-NyeTARs.A017	contig042499-NyeTARs.A028	0.378	0.736	0.513
contig060105-NyeTARs.A018	contig022390-TilTARs.A055	0.425	0.828	0.513
contig042499-NyeTARs.A028	contig030471-ZebTARs.A024	0.373	0.726	0.513
contig022357-TilTARs.A041	contig030471-ZebTARs.A024	0.151	0.294	0.513
contig061977-BurTARs.A012	contig045088-TilTARs.A057	0.346	0.675	0.513
contig057301-BurTARs.A017	contig046013-NyeTARs.A021	0.363	0.706	0.513
contig035377-NyeTARs.A023	contig042499-NyeTARs.A028	0.375	0.730	0.514
contig056023-BurTARs.A019	contig007520-TilTARs.A025	0.403	0.785	0.514
contig041024-BurTARs.A026	contig046011-NyeTARs.A016	0.221	0.430	0.514
contig056200-NyeTARs.A029	contig022365-TilTARs.A045	0.301	0.585	0.514
contig086351-BriTARs.A021	contig022363-TilTARs.A043	0.452	0.879	0.514
contig084886-BriTARs.A017	contig022363-TilTARs.A044	0.192	0.374	0.515
contig022334-TilTARs.A029	contig062676-ZebTARs.A019	0.260	0.506	0.515
contig029633-BriTAR.A003	contig045302-BurTAR.A001	0.075	0.145	0.515
contig056023-BurTARs.A019	contig058002-NyeTARs.A025	0.398	0.773	0.515
contig035381-NyeTARs.A015	contig022382-TilTARs.A053	0.151	0.293	0.515
contig084876-BriTARs.A015	contig066890-ZebTARs.A014	0.170	0.330	0.515
contig062676-ZebTARs.A019	contig061417-ZebTARs.A020	0.262	0.508	0.515
contig034854-BurTARs.A027	contig061417-ZebTARs.A020	0.270	0.524	0.515
contig060292-NyeTARs.A027	contig022353-TilTARs.A036	0.432	0.838	0.515
contig061091-BurTARs.A014	contig022334-TilTARs.A030	0.187	0.363	0.516
contig060292-NyeTARs.A027	contig022363-TilTARs.A043	0.495	0.960	0.516
contig046007-NyeTARs.A017	contig022390-TilTARs.A056	0.283	0.548	0.516
contig007524-TilTARs.A026	contig066285-ZebTARs.A016	0.391	0.759	0.516
contig060292-NyeTARs.A027	contig022354-TilTARs.A037	0.395	0.766	0.516
contig049534-BurTARs.A025	contig045999-NyeTARs.A026	0.189	0.367	0.516
contig066691-ZebTARs.A015	contig062676-ZebTARs.A019	0.291	0.565	0.516
contig084880-BriTARs.A016	contig022337-TilTARs.A031	0.201	0.390	0.516
contig060707-BurTARs.A015	contig056023-BurTARs.A019	0.389	0.754	0.516
contig057305-BurTARs.A031	contig035376-NyeTARs.A014	0.363	0.703	0.516
contig045999-NyeTARs.A026	contig022379-TilTARs.A052	0.271	0.525	0.516
contig082565-BriTARs.A022	contig022353-TilTARs.A036	0.292	0.566	0.516
contig046007-NyeTARs.A017	contig045088-TilTARs.A057	0.383	0.742	0.516
contig059673-BurTARs.A016	contig066691-ZebTARs.A015	0.379	0.735	0.516
contig022368-TilTARs.A047	contig030445-ZebTARs.A026	0.275	0.532	0.516
contig060292-NyeTARs.A027	contig022349-TilTARs.A035	0.414	0.802	0.516

contig022349-TilTARs.A035	contig022363-TilTARs.A044	0.185	0.359	0.516
contig046011-NyeTARs.A016	contig060292-NyeTARs.A027	0.452	0.875	0.516
contig056020-BurTARs.A021	contig022337-TilTARs.A031	0.132	0.256	0.517
contig045088-TilTARs.A057	contig066890-ZebTARs.A014	0.380	0.735	0.517
contig061433-BurTARs.A013	contig007524-TilTARs.A026	0.393	0.760	0.517
contig022354-TilTARs.A038	contig022363-TilTARs.A043	0.182	0.353	0.517
contig022382-TilTARs.A053	contig030471-ZebTARs.A024	0.281	0.544	0.517
contig059673-BurTARs.A016	contig022378-TilTARs.A051	0.355	0.688	0.517
contig034854-BurTARs.A027	contig022343-TilTARs.A033	0.273	0.528	0.517
contig057301-BurTARs.A017	contig022375-TilTARs.A048	0.382	0.740	0.517
contig046014-NyeTARs.A024	contig022382-TilTARs.A053	0.264	0.510	0.517
contig022334-TilTARs.A030	contig022356-TilTARs.A040	0.187	0.362	0.517
contig084880-BriTARs.A016	contig022324-TilTARs.A027	0.111	0.214	0.517
contig057305-BurTARs.A031	contig035375-NyeTARs.A013	0.359	0.695	0.517
contig061091-BurTARs.A014	contig035376-NyeTARs.A014	0.273	0.528	0.517
contig084880-BriTARs.A016	contig061433-BurTARs.A013	0.107	0.207	0.517
contig022357-TilTARs.A041	contig030445-ZebTARs.A026	0.275	0.532	0.517
contig045999-NyeTARs.A026	contig007520-TilTARs.A025	0.401	0.775	0.518
contig045088-TilTARs.A057	contig030471-ZebTARs.A024	0.369	0.712	0.518
contig057301-BurTARs.A017	contig022357-TilTARs.A041	0.390	0.752	0.518
contig042499-NyeTARs.A028	contig022324-TilTARs.A027	0.397	0.766	0.518
contig060292-NyeTARs.A027	contig022357-TilTARs.A041	0.419	0.809	0.518
contig061433-BurTARs.A013	contig058002-NyeTARs.A025	0.396	0.765	0.518
contig046011-NyeTARs.A016	contig058002-NyeTARs.A025	0.407	0.786	0.518
contig022334-TilTARs.A030	contig022353-TilTARs.A036	0.204	0.394	0.518
contig058002-NyeTARs.A025	contig045999-NyeTARs.A026	0.396	0.764	0.518
contig061091-BurTARs.A014	contig022349-TilTARs.A035	0.151	0.291	0.518
contig061977-BurTARs.A012	contig045999-NyeTARs.A026	0.278	0.537	0.518
contig046007-NyeTARs.A017	contig062676-ZebTARs.A019	0.273	0.527	0.518
contig034854-BurTARs.A027	contig022363-TilTARs.A044	0.250	0.483	0.519
contig056020-BurTARs.A021	contig030445-ZebTARs.A026	0.256	0.494	0.519
contig084887-BriTARs.A018	contig056200-NyeTARs.A029	0.287	0.554	0.519
contig022353-TilTARs.A036	contig022390-TilTARs.A056	0.277	0.534	0.519
contig062039-NyeTARs.A022	contig022334-TilTARs.A029	0.260	0.501	0.519
contig022368-TilTARs.A046	contig066890-ZebTARs.A014	0.270	0.521	0.519
contig049540-BurTARs.A024	contig035377-NyeTARs.A023	0.191	0.369	0.519
contig022382-TilTARs.A053	contig030445-ZebTARs.A026	0.149	0.288	0.519
contig059673-BurTARs.A016	contig034854-BurTARs.A027	0.364	0.701	0.519
contig041024-BurTARs.A026	contig034854-BurTARs.A027	0.261	0.504	0.519
contig061091-BurTARs.A014	contig061410-ZebTARs.A021	0.266	0.512	0.519
contig007524-TilTARs.A026	contig030440-ZebTARs.A029	0.347	0.669	0.519
contig046013-NyeTARs.A021	contig022337-TilTARs.A031	0.122	0.235	0.519
contig022379-TilTARs.A052	contig066890-ZebTARs.A014	0.165	0.318	0.519
contig022363-TilTARs.A043	contig066890-ZebTARs.A014	0.302	0.582	0.519
contig045999-NyeTARs.A026	contig030471-ZebTARs.A024	0.187	0.360	0.519
contig084868-BriTARs.A014	contig030445-ZebTARs.A026	0.125	0.242	0.519
contig084887-BriTARs.A018	contig022363-TilTARs.A043	0.177	0.340	0.519
contig022390-TilTARs.A056	contig066691-ZebTARs.A015	0.287	0.553	0.519
contig084880-BriTARs.A016	contig022378-TilTARs.A051	0.285	0.549	0.520
contig084887-BriTARs.A018	contig022349-TilTARs.A035	0.147	0.284	0.520
contig046007-NyeTARs.A017	contig045999-NyeTARs.A026	0.194	0.374	0.520
contig084880-BriTARs.A016	contig022390-TilTARs.A056	0.296	0.570	0.520
contig022354-TilTARs.A037	contig045088-TilTARs.A057	0.355	0.684	0.520

contig060292-NyeTARs.A027	contig022330-TilTARs.A028	0.416	0.801	0.520
contig045999-NyeTARs.A026	contig022378-TilTARs.A051	0.287	0.551	0.520
contig042499-NyeTARs.A028	contig022337-TilTARs.A031	0.380	0.730	0.520
contig022343-TilTARs.A033	contig022368-TilTARs.A046	0.151	0.291	0.520
contig046010-NyeTARs.A019	contig022363-TilTARs.A044	0.190	0.365	0.520
contig056200-NyeTARs.A029	contig022354-TilTARs.A037	0.259	0.497	0.520
contig057301-BurTARs.A017	contig022356-TilTARs.A040	0.385	0.740	0.521
contig056020-BurTARs.A021	contig022378-TilTARs.A051	0.263	0.506	0.521
contig049540-BurTARs.A024	contig022353-TilTARs.A036	0.302	0.580	0.521
contig042499-NyeTARs.A028	contig022383-TilTARs.A054	0.368	0.707	0.521
contig059673-BurTARs.A016	contig030471-ZebTARs.A024	0.375	0.721	0.521
contig022330-TilTARs.A028	contig022353-TilTARs.A036	0.136	0.262	0.521
contig034854-BurTARs.A027	contig022341-TilTARs.A032	0.277	0.531	0.521
contig060292-NyeTARs.A027	contig022343-TilTARs.A033	0.406	0.779	0.521
contig084887-BriTARs.A018	contig022390-TilTARs.A056	0.280	0.538	0.521
contig060292-NyeTARs.A027	contig022375-TilTARs.A048	0.403	0.773	0.521
contig030471-ZebTARs.A024	contig030445-ZebTARs.A026	0.257	0.492	0.521
contig046013-NyeTARs.A021	contig056200-NyeTARs.A029	0.298	0.573	0.521
contig022354-TilTARs.A037	contig022379-TilTARs.A052	0.228	0.438	0.521
contig034854-BurTARs.A027	contig022363-TilTARs.A043	0.317	0.609	0.521
contig045088-TilTARs.A057	contig022320-TilTARs.A059	0.347	0.667	0.521
contig035376-NyeTARs.A014	contig022357-TilTARs.A041	0.282	0.540	0.521
contig022382-TilTARs.A053	contig066691-ZebTARs.A015	0.289	0.554	0.521
contig057301-BurTARs.A017	contig061410-ZebTARs.A021	0.351	0.672	0.521
contig022375-TilTARs.A048	contig030440-ZebTARs.A029	0.233	0.448	0.521
contig007518-TilTARs.A019	contig061410-ZebTARs.A021	0.346	0.664	0.522
contig045999-NyeTARs.A026	contig061417-ZebTARs.A020	0.195	0.374	0.522
contig086351-BriTARs.A021	contig030440-ZebTARs.A029	0.376	0.720	0.522
contig042499-NyeTARs.A028	contig062676-ZebTARs.A019	0.374	0.717	0.522
contig057305-BurTARs.A031	contig030471-ZebTARs.A024	0.422	0.809	0.522
contig060292-NyeTARs.A027	contig030471-ZebTARs.A024	0.422	0.809	0.522
contig062039-NyeTARs.A022	contig066691-ZebTARs.A015	0.286	0.548	0.522
contig056023-BurTARs.A019	contig022357-TilTARs.A041	0.189	0.363	0.522
contig057301-BurTARs.A017	contig022320-TilTARs.A059	0.349	0.669	0.522
contig034854-BurTARs.A027	contig061410-ZebTARs.A021	0.157	0.300	0.522
contig022354-TilTARs.A037	contig022390-TilTARs.A056	0.254	0.486	0.522
contig035375-NyeTARs.A013	contig060292-NyeTARs.A027	0.360	0.690	0.522
contig084880-BriTARs.A016	contig062677-ZebTARs.A018	0.330	0.631	0.522
contig084868-BriTARs.A014	contig035376-NyeTARs.A014	0.127	0.243	0.522
contig060105-NyeTARs.A018	contig045999-NyeTARs.A026	0.396	0.758	0.522
contig045999-NyeTARs.A026	contig061410-ZebTARs.A021	0.277	0.529	0.522
contig042499-NyeTARs.A028	contig061410-ZebTARs.A021	0.345	0.660	0.522
contig057305-BurTARs.A031	contig022353-TilTARs.A036	0.432	0.826	0.523
contig084880-BriTARs.A016	contig046007-NyeTARs.A017	0.199	0.381	0.523
contig057301-BurTARs.A017	contig061417-ZebTARs.A020	0.365	0.698	0.523
contig057305-BurTARs.A031	contig022354-TilTARs.A037	0.395	0.755	0.523
contig035381-NyeTARs.A015	contig045999-NyeTARs.A026	0.301	0.575	0.523
contig041024-BurTARs.A026	contig030440-ZebTARs.A029	0.258	0.494	0.523
contig022368-TilTARs.A047	contig030440-ZebTARs.A029	0.300	0.573	0.523
contig034854-BurTARs.A027	contig057305-BurTARs.A031	0.381	0.728	0.523
contig084887-BriTARs.A018	contig045999-NyeTARs.A026	0.191	0.366	0.523
contig057301-BurTARs.A017	contig046014-NyeTARs.A024	0.357	0.683	0.523
contig046007-NyeTARs.A017	contig022363-TilTARs.A044	0.175	0.335	0.523

contig086344-BriTARs.A020	contig022343-TilTARs.A033	0.348	0.666	0.523
contig056023-BurTARs.A019	contig007524-TilTARs.A026	0.409	0.781	0.523
contig057145-BurTARs.A018	contig022357-TilTARs.A041	0.154	0.295	0.523
contig022365-TilTARs.A045	contig022382-TilTARs.A053	0.294	0.562	0.523
contig056020-BurTARs.A021	contig035376-NyeTARs.A014	0.261	0.499	0.523
contig086344-BriTARs.A020	contig046011-NyeTARs.A016	0.463	0.885	0.523
contig034854-BurTARs.A027	contig022375-TilTARs.A048	0.232	0.444	0.523
contig061091-BurTARs.A014	contig062676-ZebTARs.A019	0.287	0.549	0.524
contig057305-BurTARs.A031	contig022349-TilTARs.A035	0.414	0.791	0.524
contig035376-NyeTARs.A014	contig062677-ZebTARs.A018	0.164	0.313	0.524
contig022354-TilTARs.A037	contig022390-TilTARs.A055	0.263	0.501	0.524
contig035377-NyeTARs.A023	contig056200-NyeTARs.A029	0.193	0.369	0.524
contig057301-BurTARs.A017	contig035377-NyeTARs.A023	0.385	0.734	0.524
contig056023-BurTARs.A019	contig022378-TilTARs.A051	0.282	0.538	0.524
contig045302-BurTAR.A001	contig040586-ZebTAR.A002	0.067	0.127	0.524
contig056021-BurTARs.A020	contig066890-ZebTARs.A014	0.270	0.516	0.524
contig057305-BurTARs.A031	contig046011-NyeTARs.A016	0.452	0.862	0.524
contig046011-NyeTARs.A016	contig030445-ZebTARs.A026	0.285	0.543	0.524
contig056023-BurTARs.A019	contig062677-ZebTARs.A018	0.323	0.616	0.524
contig034854-BurTARs.A027	contig022355-TilTARs.A039	0.274	0.523	0.524
contig057148-BurTARs.A028	contig030464-ZebTARs.A025	0.010	0.019	0.524
contig084880-BriTARs.A016	contig022343-TilTARs.A033	0.192	0.366	0.525
contig035375-NyeTARs.A013	contig022357-TilTARs.A041	0.292	0.557	0.525
contig057301-BurTARs.A017	contig062676-ZebTARs.A019	0.373	0.711	0.525
contig042499-NyeTARs.A028	contig022354-TilTARs.A037	0.356	0.678	0.525
contig086344-BriTARs.A020	contig060707-BurTARs.A015	0.014	0.027	0.525
contig046013-NyeTARs.A021	contig022363-TilTARs.A044	0.176	0.335	0.525
contig035376-NyeTARs.A014	contig022353-TilTARs.A036	0.286	0.544	0.525
contig057305-BurTARs.A031	contig022357-TilTARs.A041	0.419	0.798	0.525
contig022334-TilTARs.A030	contig022368-TilTARs.A047	0.196	0.373	0.525
contig022330-TilTARs.A028	contig022337-TilTARs.A031	0.129	0.245	0.525
contig086351-BriTARs.A021	contig022353-TilTARs.A036	0.441	0.840	0.525
contig022363-TilTARs.A043	contig062677-ZebTARs.A018	0.322	0.612	0.525
contig086351-BriTARs.A021	contig022343-TilTARs.A033	0.416	0.790	0.526
contig022343-TilTARs.A033	contig066890-ZebTARs.A014	0.282	0.537	0.526
contig084868-BriTARs.A014	contig056020-BurTARs.A021	0.314	0.597	0.526
contig041024-BurTARs.A026	contig057305-BurTARs.A031	0.405	0.770	0.526
contig041024-BurTARs.A026	contig060292-NyeTARs.A027	0.405	0.770	0.526
contig046011-NyeTARs.A016	contig066056-ZebTARs.A017	0.436	0.829	0.526
contig084887-BriTARs.A018	contig062039-NyeTARs.A022	0.281	0.535	0.526
contig056020-BurTARs.A021	contig022379-TilTARs.A052	0.251	0.477	0.526
contig057301-BurTARs.A017	contig022324-TilTARs.A027	0.397	0.754	0.526
contig084880-BriTARs.A016	contig022379-TilTARs.A052	0.267	0.508	0.526
contig082565-BriTARs.A022	contig046007-NyeTARs.A017	0.273	0.518	0.526
contig059673-BurTARs.A016	contig022375-TilTARs.A048	0.356	0.677	0.526
contig057305-BurTARs.A031	contig022375-TilTARs.A048	0.402	0.764	0.526
contig057301-BurTARs.A017	contig046007-NyeTARs.A017	0.392	0.745	0.526
contig035376-NyeTARs.A014	contig060292-NyeTARs.A027	0.365	0.694	0.527
contig084887-BriTARs.A018	contig062676-ZebTARs.A019	0.291	0.552	0.527
contig022356-TilTARs.A040	contig022357-TilTARs.A041	0.147	0.279	0.527
contig045999-NyeTARs.A026	contig007524-TilTARs.A026	0.406	0.771	0.527
contig046013-NyeTARs.A021	contig022383-TilTARs.A054	0.299	0.567	0.527
contig022349-TilTARs.A035	contig022378-TilTARs.A051	0.274	0.519	0.527

contig066890-ZebTARs.A014	contig061417-ZebTARs.A020	0.271	0.514	0.527
contig022343-TilTARs.A033	contig022390-TilTARs.A056	0.277	0.526	0.527
contig057305-BurTARs.A031	contig022330-TilTARs.A028	0.416	0.790	0.527
contig042499-NyeTARs.A028	contig066890-ZebTARs.A014	0.375	0.710	0.527
contig022353-TilTARs.A036	contig022363-TilTARs.A043	0.156	0.297	0.527
contig035375-NyeTARs.A013	contig022375-TilTARs.A048	0.242	0.459	0.527
contig061091-BurTARs.A014	contig022390-TilTARs.A056	0.283	0.537	0.528
contig084880-BriTARs.A016	contig022363-TilTARs.A043	0.239	0.453	0.528
contig022390-TilTARs.A056	contig030471-ZebTARs.A024	0.287	0.544	0.528
contig034854-BurTARs.A027	contig060292-NyeTARs.A027	0.382	0.723	0.528
contig057305-BurTARs.A031	contig022343-TilTARs.A033	0.406	0.769	0.528
contig057301-BurTARs.A017	contig022334-TilTARs.A030	0.383	0.725	0.528
contig035375-NyeTARs.A013	contig062677-ZebTARs.A018	0.169	0.321	0.528
contig022375-TilTARs.A048	contig062677-ZebTARs.A018	0.243	0.460	0.528
contig042499-NyeTARs.A028	contig022320-TilTARs.A059	0.349	0.661	0.528
contig084880-BriTARs.A016	contig061977-BurTARs.A012	0.275	0.520	0.528
contig022378-TilTARs.A051	contig022379-TilTARs.A052	0.206	0.390	0.529
contig084887-BriTARs.A018	contig061977-BurTARs.A012	0.264	0.500	0.529
contig022378-TilTARs.A051	contig022320-TilTARs.A059	0.248	0.468	0.529
contig045999-NyeTARs.A026	contig022330-TilTARs.A028	0.189	0.358	0.529
contig022343-TilTARs.A033	contig022363-TilTARs.A043	0.190	0.359	0.529
contig046011-NyeTARs.A016	contig060105-NyeTARs.A018	0.454	0.859	0.529
contig022368-TilTARs.A046	contig022390-TilTARs.A056	0.264	0.499	0.529
contig061977-BurTARs.A012	contig022390-TilTARs.A056	0.154	0.290	0.529
contig022349-TilTARs.A035	contig066691-ZebTARs.A015	0.154	0.291	0.529
contig084887-BriTARs.A018	contig046013-NyeTARs.A021	0.022	0.042	0.529
contig061091-BurTARs.A014	contig022382-TilTARs.A053	0.285	0.538	0.529
contig084880-BriTARs.A016	contig057305-BurTARs.A031	0.443	0.837	0.530
contig022337-TilTARs.A031	contig045088-TilTARs.A057	0.383	0.722	0.530
contig022362-TilTARs.A042	contig022368-TilTARs.A047	0.113	0.213	0.530
contig084880-BriTARs.A016	contig049540-BurTARs.A024	0.328	0.620	0.530
contig056023-BurTARs.A019	contig060105-NyeTARs.A018	0.396	0.747	0.530
contig086351-BriTARs.A021	contig022375-TilTARs.A048	0.407	0.767	0.530
contig022337-TilTARs.A031	contig022363-TilTARs.A043	0.201	0.379	0.530
contig057301-BurTARs.A017	contig066890-ZebTARs.A014	0.378	0.714	0.530
contig084868-BriTARs.A014	contig062676-ZebTARs.A019	0.038	0.071	0.530
contig084876-BriTARs.A015	contig046007-NyeTARs.A017	0.283	0.533	0.530
contig022334-TilTARs.A029	contig022390-TilTARs.A056	0.265	0.500	0.530
contig060105-NyeTARs.A018	contig022363-TilTARs.A043	0.417	0.786	0.530
contig022337-TilTARs.A031	contig022355-TilTARs.A039	0.126	0.238	0.530
contig049540-BurTARs.A024	contig022363-TilTARs.A043	0.326	0.614	0.531
contig066890-ZebTARs.A014	contig030471-ZebTARs.A024	0.292	0.550	0.531
contig045088-TilTARs.A057	contig061410-ZebTARs.A021	0.353	0.664	0.531
contig084880-BriTARs.A016	contig066890-ZebTARs.A014	0.303	0.570	0.531
contig034854-BurTARs.A027	contig022377-TilTARs.A050	0.277	0.522	0.531
contig056023-BurTARs.A019	contig022356-TilTARs.A040	0.193	0.363	0.531
contig022363-TilTARs.A043	contig022379-TilTARs.A052	0.272	0.512	0.531
contig086351-BriTARs.A021	contig035376-NyeTARs.A014	0.376	0.708	0.531
contig022330-TilTARs.A028	contig022378-TilTARs.A051	0.247	0.465	0.531
contig045999-NyeTARs.A026	contig022337-TilTARs.A031	0.203	0.382	0.532
contig056023-BurTARs.A019	contig049534-BurTARs.A025	0.190	0.358	0.532
contig060707-BurTARs.A015	contig022363-TilTARs.A043	0.414	0.779	0.532
contig056200-NyeTARs.A029	contig022353-TilTARs.A036	0.304	0.572	0.532

contig056023-BurTARs.A019	contig049540-BurTARs.A024	0.321	0.605	0.532
contig022330-TilTARs.A028	contig022363-TilTARs.A044	0.181	0.341	0.532
contig042499-NyeTARs.A028	contig022353-TilTARs.A036	0.385	0.723	0.532
contig035381-NyeTARs.A015	contig046013-NyeTARs.A021	0.285	0.535	0.532
contig060292-NyeTARs.A027	contig022356-TilTARs.A040	0.432	0.811	0.532
contig082565-BriTARs.A022	contig022382-TilTARs.A053	0.152	0.285	0.532
contig022368-TilTARs.A046	contig022378-TilTARs.A051	0.253	0.475	0.532
contig084880-BriTARs.A016	contig061410-ZebTARs.A021	0.273	0.513	0.533
contig030471-ZebTARs.A024	contig030440-ZebTARs.A029	0.280	0.526	0.533
contig084868-BriTARs.A014	contig084887-BriTARs.A018	0.339	0.636	0.533
contig057301-BurTARs.A017	contig030471-ZebTARs.A024	0.370	0.695	0.533
contig046011-NyeTARs.A016	contig030471-ZebTARs.A024	0.246	0.462	0.533
contig046011-NyeTARs.A016	contig007524-TilTARs.A026	0.413	0.775	0.533
contig022354-TilTARs.A038	contig022363-TilTARs.A044	0.180	0.337	0.533
contig084880-BriTARs.A016	contig056020-BurTARs.A021	0.216	0.405	0.533
contig084880-BriTARs.A016	contig066691-ZebTARs.A015	0.199	0.374	0.533
contig086351-BriTARs.A021	contig041024-BurTARs.A026	0.412	0.772	0.533
contig084886-BriTARs.A017	contig022354-TilTARs.A037	0.199	0.373	0.533
contig057301-BurTARs.A017	contig056020-BurTARs.A021	0.416	0.780	0.533
contig022334-TilTARs.A030	contig022337-TilTARs.A031	0.193	0.362	0.533
contig057305-BurTARs.A031	contig035377-NyeTARs.A023	0.367	0.687	0.534
contig082565-BriTARs.A022	contig046013-NyeTARs.A021	0.280	0.524	0.534
contig022343-TilTARs.A033	contig022363-TilTARs.A044	0.193	0.361	0.534
contig022363-TilTARs.A043	contig030440-ZebTARs.A029	0.310	0.580	0.534
contig022378-TilTARs.A051	contig022390-TilTARs.A056	0.237	0.443	0.534
contig086351-BriTARs.A021	contig034854-BurTARs.A027	0.392	0.733	0.534
contig046007-NyeTARs.A017	contig022379-TilTARs.A052	0.265	0.496	0.534
contig084876-BriTARs.A015	contig042499-NyeTARs.A028	0.354	0.662	0.535
contig035375-NyeTARs.A013	contig022365-TilTARs.A045	0.299	0.560	0.535
contig022383-TilTARs.A054	contig030440-ZebTARs.A029	0.135	0.252	0.535
contig082565-BriTARs.A022	contig062676-ZebTARs.A019	0.106	0.199	0.535
contig057301-BurTARs.A017	contig022337-TilTARs.A031	0.385	0.719	0.535
contig046007-NyeTARs.A017	contig030440-ZebTARs.A029	0.282	0.527	0.535
contig049534-BurTARs.A025	contig022349-TilTARs.A035	0.192	0.359	0.535
contig022363-TilTARs.A044	contig030464-ZebTARs.A025	0.196	0.366	0.535
contig084876-BriTARs.A015	contig066691-ZebTARs.A015	0.284	0.530	0.535
contig035376-NyeTARs.A014	contig022368-TilTARs.A046	0.260	0.485	0.535
contig035375-NyeTARs.A013	contig022363-TilTARs.A044	0.261	0.488	0.535
contig046007-NyeTARs.A017	contig030445-ZebTARs.A026	0.264	0.493	0.536
contig061433-BurTARs.A013	contig022382-TilTARs.A053	0.302	0.564	0.536
contig084880-BriTARs.A016	contig046013-NyeTARs.A021	0.202	0.376	0.536
contig035375-NyeTARs.A013	contig007524-TilTARs.A026	0.348	0.649	0.536
contig084876-BriTARs.A015	contig022363-TilTARs.A043	0.293	0.548	0.536
contig022357-TilTARs.A041	contig062676-ZebTARs.A019	0.277	0.517	0.536
contig062039-NyeTARs.A022	contig022362-TilTARs.A042	0.279	0.521	0.536
contig022354-TilTARs.A037	contig022382-TilTARs.A053	0.248	0.463	0.536
contig059673-BurTARs.A016	contig035375-NyeTARs.A013	0.356	0.665	0.536
contig046013-NyeTARs.A021	contig066890-ZebTARs.A014	0.287	0.536	0.536
contig060292-NyeTARs.A027	contig022334-TilTARs.A029	0.415	0.774	0.536
contig034854-BurTARs.A027	contig045088-TilTARs.A057	0.392	0.730	0.537
contig084876-BriTARs.A015	contig022353-TilTARs.A036	0.290	0.541	0.537
contig022383-TilTARs.A054	contig066890-ZebTARs.A014	0.108	0.201	0.537
contig056020-BurTARs.A021	contig022357-TilTARs.A041	0.157	0.293	0.537

contig084880-BriTARs.A016	contig060292-NyeTARs.A027	0.443	0.825	0.537
contig057301-BurTARs.A017	contig030440-ZebTARs.A029	0.383	0.714	0.537
contig056023-BurTARs.A019	contig022379-TilTARs.A052	0.272	0.506	0.537
contig022349-TilTARs.A035	contig045088-TilTARs.A057	0.380	0.708	0.537
contig049540-BurTARs.A024	contig035376-NyeTARs.A014	0.162	0.302	0.537
contig045999-NyeTARs.A026	contig022390-TilTARs.A056	0.299	0.558	0.537
contig061977-BurTARs.A012	contig046013-NyeTARs.A021	0.270	0.502	0.537
contig035375-NyeTARs.A013	contig022383-TilTARs.A054	0.137	0.256	0.537
contig056023-BurTARs.A019	contig035381-NyeTARs.A015	0.298	0.554	0.537
contig086351-BriTARs.A021	contig035377-NyeTARs.A023	0.382	0.710	0.538
contig057305-BurTARs.A031	contig022356-TilTARs.A040	0.431	0.801	0.538
contig056023-BurTARs.A019	contig022330-TilTARs.A028	0.189	0.352	0.538
contig086351-BriTARs.A021	contig022357-TilTARs.A041	0.424	0.788	0.538
contig084876-BriTARs.A015	contig045999-NyeTARs.A026	0.291	0.540	0.538
contig022334-TilTARs.A029	contig022363-TilTARs.A044	0.178	0.330	0.538
contig084876-BriTARs.A015	contig034854-BurTARs.A027	0.167	0.310	0.538
contig066691-ZebTARs.A015	contig030440-ZebTARs.A029	0.287	0.533	0.538
contig035375-NyeTARs.A013	contig022355-TilTARs.A039	0.276	0.512	0.539
contig045999-NyeTARs.A026	contig022362-TilTARs.A042	0.209	0.388	0.539
contig084868-BriTARs.A014	contig046011-NyeTARs.A016	0.341	0.633	0.539
contig060292-NyeTARs.A027	contig022337-TilTARs.A031	0.417	0.773	0.539
contig061977-BurTARs.A012	contig056023-BurTARs.A019	0.279	0.518	0.539
contig059673-BurTARs.A016	contig046011-NyeTARs.A016	0.458	0.850	0.539
contig056200-NyeTARs.A029	contig022363-TilTARs.A043	0.328	0.608	0.539
contig046007-NyeTARs.A017	contig066890-ZebTARs.A014	0.294	0.545	0.539
contig045999-NyeTARs.A026	contig066691-ZebTARs.A015	0.198	0.366	0.539
contig046011-NyeTARs.A016	contig042499-NyeTARs.A028	0.420	0.779	0.539
contig056021-BurTARs.A020	contig060292-NyeTARs.A027	0.414	0.768	0.539
contig056023-BurTARs.A019	contig030471-ZebTARs.A024	0.186	0.345	0.539
contig086351-BriTARs.A021	contig022356-TilTARs.A040	0.439	0.813	0.539
contig086351-BriTARs.A021	contig062677-ZebTARs.A018	0.378	0.701	0.540
contig022320-TilTARs.A059	contig030440-ZebTARs.A029	0.261	0.484	0.540
contig034854-BurTARs.A027	contig022320-TilTARs.A059	0.272	0.504	0.540
contig057145-BurTARs.A018	contig060292-NyeTARs.A027	0.428	0.793	0.540
contig046011-NyeTARs.A016	contig007518-TilTARs.A019	0.441	0.817	0.540
contig084880-BriTARs.A016	contig056200-NyeTARs.A029	0.331	0.611	0.541
contig034854-BurTARs.A027	contig046014-NyeTARs.A024	0.268	0.495	0.541
contig035375-NyeTARs.A013	contig022368-TilTARs.A047	0.309	0.571	0.541
contig045999-NyeTARs.A026	contig022343-TilTARs.A033	0.194	0.359	0.541
contig022353-TilTARs.A036	contig022362-TilTARs.A042	0.152	0.282	0.541
contig084868-BriTARs.A014	contig022353-TilTARs.A036	0.323	0.596	0.541
contig056021-BurTARs.A020	contig022390-TilTARs.A056	0.265	0.491	0.541
contig086351-BriTARs.A021	contig022354-TilTARs.A037	0.404	0.747	0.541
contig045088-TilTARs.A057	contig030440-ZebTARs.A029	0.387	0.716	0.541
contig022349-TilTARs.A035	contig022368-TilTARs.A046	0.159	0.294	0.541
contig084887-BriTARs.A018	contig061410-ZebTARs.A021	0.267	0.493	0.541
contig061977-BurTARs.A012	contig046011-NyeTARs.A016	0.302	0.557	0.541
contig022379-TilTARs.A052	contig066691-ZebTARs.A015	0.272	0.502	0.541
contig022349-TilTARs.A035	contig022377-TilTARs.A050	0.174	0.321	0.541
contig084887-BriTARs.A018	contig022382-TilTARs.A053	0.280	0.517	0.541
contig022353-TilTARs.A036	contig030440-ZebTARs.A029	0.269	0.497	0.542
contig049540-BurTARs.A024	contig035375-NyeTARs.A013	0.168	0.309	0.542
contig046013-NyeTARs.A021	contig045999-NyeTARs.A026	0.200	0.369	0.542

contig084880-BriTARs.A016	contig061091-BurTARs.A014	0.196	0.361	0.542
contig061977-BurTARs.A012	contig035375-NyeTARs.A013	0.142	0.263	0.542
contig046011-NyeTARs.A016	contig062676-ZebTARs.A019	0.312	0.575	0.542
contig057301-BurTARs.A017	contig022354-TilTARs.A037	0.360	0.663	0.542
contig022363-TilTARs.A043	contig022382-TilTARs.A053	0.313	0.578	0.542
contig034854-BurTARs.A027	contig035376-NyeTARs.A014	0.058	0.107	0.542
contig082565-BriTARs.A022	contig046011-NyeTARs.A016	0.301	0.555	0.543
contig084880-BriTARs.A016	contig030445-ZebTARs.A026	0.293	0.541	0.543
contig056023-BurTARs.A019	contig046007-NyeTARs.A017	0.199	0.367	0.543
contig056023-BurTARs.A019	contig056200-NyeTARs.A029	0.324	0.596	0.543
contig034854-BurTARs.A027	contig022357-TilTARs.A041	0.294	0.542	0.543
contig046014-NyeTARs.A024	contig030440-ZebTARs.A029	0.263	0.485	0.543
contig035376-NyeTARs.A014	contig056200-NyeTARs.A029	0.164	0.302	0.543
contig056021-BurTARs.A020	contig046011-NyeTARs.A016	0.243	0.448	0.543
contig046013-NyeTARs.A021	contig060292-NyeTARs.A027	0.410	0.754	0.543
contig022368-TilTARs.A046	contig062677-ZebTARs.A018	0.281	0.518	0.543
contig045999-NyeTARs.A026	contig022363-TilTARs.A043	0.249	0.459	0.543
contig084876-BriTARs.A015	contig022378-TilTARs.A051	0.219	0.403	0.543
contig056023-BurTARs.A019	contig061410-ZebTARs.A021	0.278	0.511	0.543
contig046007-NyeTARs.A017	contig022357-TilTARs.A041	0.161	0.296	0.543
contig084876-BriTARs.A015	contig061091-BurTARs.A014	0.280	0.515	0.544
contig057305-BurTARs.A031	contig022334-TilTARs.A029	0.415	0.764	0.544
contig045999-NyeTARs.A026	contig022324-TilTARs.A027	0.111	0.204	0.544
contig022334-TilTARs.A029	contig061417-ZebTARs.A020	0.052	0.096	0.544
contig034854-BurTARs.A027	contig062677-ZebTARs.A018	0.183	0.337	0.544
contig022375-TilTARs.A048	contig022382-TilTARs.A053	0.233	0.428	0.544
contig022334-TilTARs.A029	contig022378-TilTARs.A051	0.254	0.467	0.544
contig046011-NyeTARs.A016	contig061410-ZebTARs.A021	0.299	0.550	0.544
contig082565-BriTARs.A022	contig035375-NyeTARs.A013	0.075	0.139	0.544
contig035377-NyeTARs.A023	contig060292-NyeTARs.A027	0.369	0.678	0.544
contig086351-BriTARs.A021	contig066890-ZebTARs.A014	0.373	0.684	0.544
contig056023-BurTARs.A019	contig061417-ZebTARs.A020	0.197	0.362	0.545
contig049540-BurTARs.A024	contig022375-TilTARs.A048	0.243	0.447	0.545
contig035375-NyeTARs.A013	contig030445-ZebTARs.A026	0.075	0.139	0.545
contig086351-BriTARs.A021	contig022330-TilTARs.A028	0.424	0.778	0.545
contig056023-BurTARs.A019	contig022390-TilTARs.A056	0.297	0.545	0.545
contig056020-BurTARs.A021	contig035375-NyeTARs.A013	0.283	0.519	0.545
contig084880-BriTARs.A016	contig035376-NyeTARs.A014	0.298	0.547	0.545
contig060292-NyeTARs.A027	contig022377-TilTARs.A050	0.425	0.780	0.545
contig034854-BurTARs.A027	contig042499-NyeTARs.A028	0.386	0.707	0.545
contig046014-NyeTARs.A024	contig060292-NyeTARs.A027	0.413	0.758	0.545
contig057305-BurTARs.A031	contig046014-NyeTARs.A024	0.413	0.758	0.545
contig035375-NyeTARs.A013	contig022362-TilTARs.A042	0.291	0.534	0.545
contig022337-TilTARs.A031	contig022363-TilTARs.A044	0.190	0.348	0.545
contig022375-TilTARs.A048	contig022378-TilTARs.A051	0.248	0.455	0.546
contig056020-BurTARs.A021	contig034854-BurTARs.A027	0.280	0.513	0.546
contig046011-NyeTARs.A016	contig022375-TilTARs.A048	0.187	0.343	0.546
contig086351-BriTARs.A021	contig035375-NyeTARs.A013	0.377	0.689	0.546
contig061091-BurTARs.A014	contig062039-NyeTARs.A022	0.282	0.517	0.546
contig057305-BurTARs.A031	contig022337-TilTARs.A031	0.417	0.763	0.546
contig086351-BriTARs.A021	contig022349-TilTARs.A035	0.418	0.764	0.546
contig046007-NyeTARs.A017	contig022337-TilTARs.A031	0.136	0.249	0.546
contig084887-BriTARs.A018	contig056023-BurTARs.A019	0.193	0.354	0.546

contig042499-NyeTARs.A028	contig022349-TilTARs.A035	0.385	0.704	0.547
contig045999-NyeTARs.A026	contig062677-ZebTARs.A018	0.330	0.604	0.547
contig046011-NyeTARs.A016	contig022365-TilTARs.A045	0.250	0.458	0.547
contig056023-BurTARs.A019	contig057305-BurTARs.A031	0.449	0.821	0.547
contig086351-BriTARs.A021	contig049540-BurTARs.A024	0.383	0.700	0.547
contig046013-NyeTARs.A021	contig007524-TilTARs.A026	0.371	0.678	0.547
contig056021-BurTARs.A020	contig057305-BurTARs.A031	0.414	0.757	0.547
contig086351-BriTARs.A021	contig057145-BurTARs.A018	0.435	0.795	0.547
contig057301-BurTARs.A017	contig022353-TilTARs.A036	0.390	0.712	0.547
contig057145-BurTARs.A018	contig057305-BurTARs.A031	0.428	0.782	0.547
contig046011-NyeTARs.A016	contig061417-ZebTARs.A020	0.239	0.437	0.547
contig057305-BurTARs.A031	contig045999-NyeTARs.A026	0.444	0.810	0.548
contig035375-NyeTARs.A013	contig056200-NyeTARs.A029	0.169	0.309	0.548
contig034854-BurTARs.A027	contig022362-TilTARs.A042	0.290	0.529	0.548
contig035376-NyeTARs.A014	contig046007-NyeTARs.A017	0.274	0.500	0.548
contig057301-BurTARs.A017	contig022349-TilTARs.A035	0.385	0.703	0.548
contig084876-BriTARs.A015	contig057301-BurTARs.A017	0.360	0.657	0.548
contig086351-BriTARs.A021	contig030471-ZebTARs.A024	0.427	0.778	0.548
contig022390-TilTARs.A056	contig061417-ZebTARs.A020	0.268	0.489	0.548
contig061091-BurTARs.A014	contig045999-NyeTARs.A026	0.194	0.354	0.548
contig062039-NyeTARs.A022	contig030471-ZebTARs.A024	0.283	0.515	0.548
contig084876-BriTARs.A015	contig084880-BriTARs.A016	0.287	0.524	0.549
contig061091-BurTARs.A014	contig022363-TilTARs.A043	0.171	0.312	0.549
contig046011-NyeTARs.A016	contig022324-TilTARs.A027	0.280	0.509	0.550
contig061091-BurTARs.A014	contig022379-TilTARs.A052	0.268	0.487	0.550
contig046013-NyeTARs.A021	contig061410-ZebTARs.A021	0.272	0.495	0.550
contig084887-BriTARs.A018	contig022378-TilTARs.A051	0.270	0.492	0.550
contig022390-TilTARs.A056	contig045088-TilTARs.A057	0.383	0.697	0.550
contig034854-BurTARs.A027	contig030445-ZebTARs.A026	0.062	0.112	0.550
contig046011-NyeTARs.A016	contig022354-TilTARs.A038	0.243	0.442	0.550
contig084880-BriTARs.A016	contig086351-BriTARs.A021	0.450	0.818	0.550
contig056023-BurTARs.A019	contig022363-TilTARs.A043	0.252	0.458	0.550
contig057305-BurTARs.A031	contig046013-NyeTARs.A021	0.410	0.744	0.551
contig046011-NyeTARs.A016	contig066285-ZebTARs.A016	0.288	0.523	0.551
contig046014-NyeTARs.A024	contig022378-TilTARs.A051	0.256	0.465	0.551
contig056200-NyeTARs.A029	contig022375-TilTARs.A048	0.244	0.443	0.551
contig046013-NyeTARs.A021	contig022349-TilTARs.A035	0.159	0.289	0.551
contig034854-BurTARs.A027	contig046011-NyeTARs.A016	0.308	0.559	0.551
contig057305-BurTARs.A031	contig066890-ZebTARs.A014	0.360	0.653	0.551
contig086351-BriTARs.A021	contig022337-TilTARs.A031	0.427	0.774	0.551
contig035377-NyeTARs.A023	contig022382-TilTARs.A053	0.183	0.332	0.551
contig060707-BurTARs.A015	contig046011-NyeTARs.A016	0.453	0.821	0.551
contig057301-BurTARs.A017	contig034854-BurTARs.A027	0.393	0.712	0.551
contig046013-NyeTARs.A021	contig062676-ZebTARs.A019	0.296	0.537	0.552
contig046011-NyeTARs.A016	contig045999-NyeTARs.A026	0.305	0.553	0.552
contig022355-TilTARs.A039	contig022378-TilTARs.A051	0.258	0.467	0.552
contig046013-NyeTARs.A021	contig022363-TilTARs.A043	0.179	0.323	0.552
contig022349-TilTARs.A035	contig022363-TilTARs.A043	0.182	0.330	0.553
contig042499-NyeTARs.A028	contig030440-ZebTARs.A029	0.382	0.691	0.553
contig045999-NyeTARs.A026	contig022334-TilTARs.A029	0.191	0.346	0.553
contig057305-BurTARs.A031	contig022377-TilTARs.A050	0.425	0.770	0.553
contig086351-BriTARs.A021	contig046014-NyeTARs.A024	0.420	0.760	0.553
contig056023-BurTARs.A019	contig056020-BurTARs.A021	0.216	0.391	0.553

contig082565-BriTARs.A022	contig056020-BurTARs.A021	0.295	0.534	0.553
contig022349-TilTARs.A035	contig022353-TilTARs.A036	0.175	0.316	0.553
contig040586-ZebTAR.A001	contig040586-ZebTAR.A002	0.064	0.115	0.553
contig034854-BurTARs.A027	contig066691-ZebTARs.A015	0.288	0.520	0.553
contig061977-BurTARs.A012	contig056020-BurTARs.A021	0.286	0.517	0.553
contig022383-TilTARs.A054	contig022390-TilTARs.A056	0.102	0.184	0.553
contig046011-NyeTARs.A016	contig045088-TilTARs.A057	0.427	0.772	0.554
contig062677-ZebTARs.A018	contig030440-ZebTARs.A029	0.168	0.303	0.554
contig086351-BriTARs.A021	contig022334-TilTARs.A029	0.423	0.763	0.554
contig038663-NyeTAR.A005	contig040586-ZebTAR.A002	0.064	0.115	0.554
contig022368-TilTARs.A046	contig022382-TilTARs.A053	0.274	0.494	0.554
contig056023-BurTARs.A019	contig060292-NyeTARs.A027	0.449	0.810	0.554
contig086351-BriTARs.A021	contig022377-TilTARs.A050	0.429	0.775	0.554
contig056023-BurTARs.A019	contig022337-TilTARs.A031	0.205	0.370	0.554
contig066890-ZebTARs.A014	contig062677-ZebTARs.A018	0.193	0.348	0.555
contig049540-BurTARs.A024	contig045999-NyeTARs.A026	0.329	0.593	0.555
contig086351-BriTARs.A021	contig022320-TilTARs.A059	0.419	0.755	0.555
contig060292-NyeTARs.A027	contig022320-TilTARs.A059	0.412	0.743	0.555
contig045999-NyeTARs.A026	contig060292-NyeTARs.A027	0.444	0.799	0.555
contig060292-NyeTARs.A027	contig062677-ZebTARs.A018	0.369	0.664	0.555
contig022324-TilTARs.A027	contig030440-ZebTARs.A029	0.294	0.529	0.555
contig049540-BurTARs.A024	contig034854-BurTARs.A027	0.181	0.327	0.555
contig084880-BriTARs.A016	contig046011-NyeTARs.A016	0.310	0.558	0.555
contig034854-BurTARs.A027	contig030471-ZebTARs.A024	0.284	0.511	0.555
contig046013-NyeTARs.A021	contig022390-TilTARs.A056	0.288	0.518	0.556
contig046013-NyeTARs.A021	contig022382-TilTARs.A053	0.294	0.528	0.556
contig046011-NyeTARs.A016	contig022363-TilTARs.A043	0.258	0.464	0.556
contig062039-NyeTARs.A022	contig022363-TilTARs.A043	0.275	0.495	0.556
contig035377-NyeTARs.A023	contig030445-ZebTARs.A026	0.121	0.218	0.556
contig084868-BriTARs.A014	contig035375-NyeTARs.A013	0.150	0.270	0.557
contig086351-BriTARs.A021	contig046013-NyeTARs.A021	0.423	0.760	0.557
contig084876-BriTARs.A015	contig046011-NyeTARs.A016	0.311	0.559	0.557
contig035375-NyeTARs.A013	contig046011-NyeTARs.A016	0.314	0.563	0.557
contig056023-BurTARs.A019	contig022324-TilTARs.A027	0.111	0.199	0.557
contig057305-BurTARs.A031	contig060292-NyeTARs.A027	0.004	0.008	0.558
contig022355-TilTARs.A039	contig022357-TilTARs.A041	0.152	0.273	0.558
contig045999-NyeTARs.A026	contig066890-ZebTARs.A014	0.302	0.541	0.558
contig046011-NyeTARs.A016	contig030440-ZebTARs.A029	0.311	0.557	0.558
contig086351-BriTARs.A021	contig056200-NyeTARs.A029	0.381	0.682	0.558
contig061433-BurTARs.A013	contig045999-NyeTARs.A026	0.107	0.191	0.558
contig022349-TilTARs.A035	contig066285-ZebTARs.A016	0.196	0.352	0.558
contig022365-TilTARs.A045	contig022368-TilTARs.A047	0.078	0.139	0.559
contig086351-BriTARs.A021	contig056021-BurTARs.A020	0.419	0.749	0.559
contig035381-NyeTARs.A015	contig035377-NyeTARs.A023	0.124	0.223	0.559
contig084876-BriTARs.A015	contig045088-TilTARs.A057	0.362	0.648	0.559
contig060292-NyeTARs.A027	contig022382-TilTARs.A053	0.358	0.641	0.559
contig049540-BurTARs.A024	contig060292-NyeTARs.A027	0.371	0.664	0.559
contig084887-BriTARs.A018	contig046011-NyeTARs.A016	0.243	0.435	0.559
contig046013-NyeTARs.A021	contig030445-ZebTARs.A026	0.279	0.498	0.559
contig035376-NyeTARs.A014	contig046013-NyeTARs.A021	0.282	0.504	0.559
contig035375-NyeTARs.A013	contig022324-TilTARs.A027	0.299	0.534	0.559
contig084876-BriTARs.A015	contig056023-BurTARs.A019	0.292	0.522	0.559
contig084880-BriTARs.A016	contig082565-BriTARs.A022	0.302	0.540	0.559

contig084880-BriTARs.A016	contig022362-TilTARs.A042	0.211	0.376	0.560
contig057305-BurTARs.A031	contig022390-TilTARs.A056	0.365	0.651	0.560
contig057301-BurTARs.A017	contig022379-TilTARs.A052	0.362	0.646	0.560
contig062039-NyeTARs.A022	contig022353-TilTARs.A036	0.289	0.516	0.560
contig084887-BriTARs.A018	contig030440-ZebTARs.A029	0.282	0.503	0.560
contig045999-NyeTARs.A026	contig030445-ZebTARs.A026	0.297	0.529	0.561
contig061433-BurTARs.A013	contig046011-NyeTARs.A016	0.285	0.509	0.561
contig049534-BurTARs.A025	contig046011-NyeTARs.A016	0.277	0.494	0.561
contig034854-BurTARs.A027	contig056200-NyeTARs.A029	0.183	0.327	0.561
contig057305-BurTARs.A031	contig062677-ZebTARs.A018	0.370	0.660	0.561
contig086351-BriTARs.A021	contig022390-TilTARs.A056	0.378	0.673	0.561
contig059673-BurTARs.A016	contig046013-NyeTARs.A021	0.380	0.678	0.561
contig034854-BurTARs.A027	contig022365-TilTARs.A045	0.294	0.524	0.561
contig042499-NyeTARs.A028	contig022379-TilTARs.A052	0.356	0.634	0.562
contig086351-BriTARs.A021	contig022382-TilTARs.A053	0.370	0.658	0.562
contig061433-BurTARs.A013	contig030440-ZebTARs.A029	0.295	0.524	0.562
contig046010-NyeTARs.A019	contig030464-ZebTARs.A025	0.004	0.008	0.562
contig022390-TilTARs.A056	contig061410-ZebTARs.A021	0.157	0.279	0.562
contig056023-BurTARs.A019	contig022362-TilTARs.A042	0.211	0.375	0.562
contig060292-NyeTARs.A027	contig066890-ZebTARs.A014	0.362	0.644	0.562
contig056023-BurTARs.A019	contig022334-TilTARs.A029	0.191	0.340	0.563
contig022343-TilTARs.A033	contig022362-TilTARs.A042	0.167	0.297	0.563
contig057305-BurTARs.A031	contig022320-TilTARs.A059	0.412	0.733	0.563
contig061091-BurTARs.A014	contig030440-ZebTARs.A029	0.287	0.510	0.563
contig084876-BriTARs.A015	contig056020-BurTARs.A021	0.297	0.527	0.563
contig055697-BurTARs.A022	contig007512-TilTARs.A024	0.020	0.035	0.563
contig056023-BurTARs.A019	contig066691-ZebTARs.A015	0.199	0.354	0.563
contig035376-NyeTARs.A014	contig045999-NyeTARs.A026	0.301	0.535	0.563
contig022362-TilTARs.A042	contig022363-TilTARs.A043	0.098	0.173	0.564
contig046011-NyeTARs.A016	contig022334-TilTARs.A029	0.244	0.433	0.564
contig046011-NyeTARs.A016	contig062039-NyeTARs.A022	0.323	0.573	0.564
contig034854-BurTARs.A027	contig022368-TilTARs.A047	0.303	0.537	0.564
contig022378-TilTARs.A051	contig066691-ZebTARs.A015	0.283	0.501	0.564
contig035375-NyeTARs.A013	contig035376-NyeTARs.A014	0.082	0.146	0.564
contig057305-BurTARs.A031	contig022382-TilTARs.A053	0.359	0.636	0.565
contig049540-BurTARs.A024	contig057305-BurTARs.A031	0.372	0.659	0.565
contig056023-BurTARs.A019	contig022343-TilTARs.A033	0.196	0.347	0.565
contig022379-TilTARs.A052	contig045088-TilTARs.A057	0.359	0.636	0.565
contig056200-NyeTARs.A029	contig022368-TilTARs.A046	0.282	0.500	0.565
contig056023-BurTARs.A019	contig046013-NyeTARs.A021	0.202	0.357	0.566
contig035376-NyeTARs.A014	contig022382-TilTARs.A053	0.153	0.271	0.566
contig046011-NyeTARs.A016	contig022390-TilTARs.A056	0.326	0.576	0.566
contig084876-BriTARs.A015	contig022390-TilTARs.A056	0.164	0.289	0.566
contig045999-NyeTARs.A026	contig056200-NyeTARs.A029	0.331	0.585	0.566
contig062676-ZebTARs.A019	contig030440-ZebTARs.A029	0.124	0.219	0.566
contig054630-BurTARs.A023	contig059768-ZebTARs.A022	0.004	0.007	0.566
contig041024-BurTARs.A026	contig035375-NyeTARs.A013	0.267	0.471	0.567
contig022390-TilTARs.A056	contig062677-ZebTARs.A018	0.180	0.318	0.567
contig086351-BriTARs.A021	contig056023-BurTARs.A019	0.456	0.803	0.568
contig022368-TilTARs.A046	contig030440-ZebTARs.A029	0.283	0.498	0.568
contig049540-BurTARs.A024	contig030440-ZebTARs.A029	0.166	0.292	0.569
contig035375-NyeTARs.A013	contig062676-ZebTARs.A019	0.127	0.222	0.569
contig086351-BriTARs.A021	contig045999-NyeTARs.A026	0.451	0.792	0.569

contig022363-TilTARs.A044	contig062677-ZebTARs.A018	0.268	0.470	0.569
contig060292-NyeTARs.A027	contig061417-ZebTARs.A020	0.417	0.733	0.569
contig022343-TilTARs.A033	contig022353-TilTARs.A036	0.117	0.206	0.569
contig042499-NyeTARs.A028	contig022390-TilTARs.A056	0.384	0.673	0.570
contig022353-TilTARs.A036	contig022378-TilTARs.A051	0.260	0.456	0.570
contig061091-BurTARs.A014	contig034854-BurTARs.A027	0.284	0.498	0.570
contig046011-NyeTARs.A016	contig066691-ZebTARs.A015	0.248	0.435	0.570
contig046013-NyeTARs.A021	contig062039-NyeTARs.A022	0.280	0.492	0.571
contig066890-ZebTARs.A014	contig062676-ZebTARs.A019	0.108	0.190	0.571
contig022349-TilTARs.A035	contig022362-TilTARs.A042	0.180	0.315	0.571
contig060292-NyeTARs.A027	contig056200-NyeTARs.A029	0.369	0.646	0.571
contig060292-NyeTARs.A027	contig022390-TilTARs.A056	0.367	0.642	0.571
contig022337-TilTARs.A031	contig022353-TilTARs.A036	0.134	0.235	0.572
contig035375-NyeTARs.A013	contig066691-ZebTARs.A015	0.297	0.519	0.572
contig046011-NyeTARs.A016	contig022353-TilTARs.A036	0.240	0.419	0.572
contig057301-BurTARs.A017	contig022390-TilTARs.A056	0.387	0.677	0.572
contig084887-BriTARs.A018	contig022379-TilTARs.A052	0.263	0.459	0.573
contig046011-NyeTARs.A016	contig022330-TilTARs.A028	0.240	0.419	0.573
contig061091-BurTARs.A014	contig056023-BurTARs.A019	0.196	0.342	0.573
contig061433-BurTARs.A013	contig056023-BurTARs.A019	0.107	0.187	0.573
contig034854-BurTARs.A027	contig046007-NyeTARs.A017	0.292	0.510	0.573
contig056023-BurTARs.A019	contig066890-ZebTARs.A014	0.299	0.522	0.573
contig061091-BurTARs.A014	contig046011-NyeTARs.A016	0.249	0.435	0.573
contig049540-BurTARs.A024	contig022363-TilTARs.A044	0.266	0.463	0.574
contig086344-BriTARs.A020	contig022363-TilTARs.A043	0.421	0.734	0.574
contig049540-BurTARs.A024	contig066890-ZebTARs.A014	0.193	0.336	0.574
contig022355-TilTARs.A039	contig022363-TilTARs.A043	0.174	0.303	0.574
contig057301-BurTARs.A017	contig035375-NyeTARs.A013	0.383	0.667	0.574
contig056200-NyeTARs.A029	contig030440-ZebTARs.A029	0.168	0.292	0.575
contig049540-BurTARs.A024	contig046011-NyeTARs.A016	0.323	0.562	0.575
contig035375-NyeTARs.A013	contig022363-TilTARs.A043	0.332	0.578	0.575
contig035376-NyeTARs.A014	contig046011-NyeTARs.A016	0.311	0.541	0.575
contig035375-NyeTARs.A013	contig045088-TilTARs.A057	0.383	0.665	0.575
contig084876-BriTARs.A015	contig046013-NyeTARs.A021	0.287	0.498	0.575
contig046007-NyeTARs.A017	contig022363-TilTARs.A043	0.190	0.331	0.575
contig035375-NyeTARs.A013	contig030471-ZebTARs.A024	0.289	0.502	0.576
contig057301-BurTARs.A017	contig046011-NyeTARs.A016	0.431	0.749	0.576
contig022390-TilTARs.A055	contig062676-ZebTARs.A019	0.140	0.243	0.576
contig046011-NyeTARs.A016	contig066890-ZebTARs.A014	0.328	0.569	0.576
contig061433-BurTARs.A013	contig035375-NyeTARs.A013	0.303	0.526	0.576
contig022363-TilTARs.A044	contig022382-TilTARs.A053	0.255	0.443	0.576
contig086351-BriTARs.A021	contig061433-BurTARs.A013	0.440	0.763	0.577
contig057305-BurTARs.A031	contig056200-NyeTARs.A029	0.370	0.641	0.577
contig056023-BurTARs.A019	contig030445-ZebTARs.A026	0.294	0.509	0.577
contig057305-BurTARs.A031	contig061417-ZebTARs.A020	0.417	0.723	0.577
contig049540-BurTARs.A024	contig022368-TilTARs.A046	0.280	0.486	0.577
contig084868-BriTARs.A014	contig035377-NyeTARs.A023	0.113	0.196	0.578
contig057148-BurTARs.A028	contig046011-NyeTARs.A016	0.264	0.458	0.578
contig035375-NyeTARs.A013	contig061410-ZebTARs.A021	0.145	0.251	0.578
contig082565-BriTARs.A022	contig045999-NyeTARs.A026	0.305	0.528	0.578
contig029633-BriTAR.A003	contig038663-NyeTAR.A005	0.071	0.123	0.578
contig046011-NyeTARs.A016	contig062677-ZebTARs.A018	0.325	0.562	0.578
contig084868-BriTARs.A014	contig066890-ZebTARs.A014	0.128	0.221	0.579

contig035375-NyeTARs.A013	contig035377-NyeTARs.A023	0.149	0.257	0.579
contig084868-BriTARs.A014	contig022390-TilTARs.A056	0.119	0.206	0.579
contig084880-BriTARs.A016	contig022382-TilTARs.A053	0.325	0.560	0.579
contig056200-NyeTARs.A029	contig066890-ZebTARs.A014	0.195	0.336	0.579
contig056023-BurTARs.A019	contig035376-NyeTARs.A014	0.299	0.515	0.580
contig034854-BurTARs.A027	contig022324-TilTARs.A027	0.303	0.523	0.580
contig056200-NyeTARs.A029	contig022363-TilTARs.A044	0.267	0.460	0.580
contig022379-TilTARs.A052	contig022390-TilTARs.A056	0.160	0.275	0.581
contig084887-BriTARs.A018	contig034854-BurTARs.A027	0.288	0.496	0.581
contig046013-NyeTARs.A021	contig022334-TilTARs.A030	0.190	0.328	0.581
contig035375-NyeTARs.A013	contig022368-TilTARs.A046	0.288	0.496	0.581
contig035375-NyeTARs.A013	contig046007-NyeTARs.A017	0.298	0.513	0.581
contig086351-BriTARs.A021	contig061417-ZebTARs.A020	0.422	0.725	0.581
contig056023-BurTARs.A019	contig022382-TilTARs.A053	0.318	0.546	0.582
contig061433-BurTARs.A013	contig045088-TilTARs.A057	0.398	0.683	0.582
contig035375-NyeTARs.A013	contig022382-TilTARs.A053	0.159	0.272	0.582
contig046011-NyeTARs.A016	contig046013-NyeTARs.A021	0.247	0.424	0.583
contig084868-BriTARs.A014	contig030440-ZebTARs.A029	0.150	0.257	0.584
contig046011-NyeTARs.A016	contig030464-ZebTARs.A025	0.267	0.458	0.584
contig034854-BurTARs.A027	contig022368-TilTARs.A046	0.285	0.489	0.584
contig061433-BurTARs.A013	contig057305-BurTARs.A031	0.444	0.759	0.584
contig035375-NyeTARs.A013	contig042499-NyeTARs.A028	0.377	0.644	0.585
contig045999-NyeTARs.A026	contig042499-NyeTARs.A028	0.419	0.715	0.586
contig056020-BurTARs.A021	contig022363-TilTARs.A043	0.182	0.311	0.586
contig046011-NyeTARs.A016	contig022378-TilTARs.A051	0.314	0.536	0.586
contig084876-BriTARs.A015	contig035375-NyeTARs.A013	0.155	0.264	0.586
contig056023-BurTARs.A019	contig046011-NyeTARs.A016	0.314	0.536	0.586
contig084880-BriTARs.A016	contig042499-NyeTARs.A028	0.416	0.708	0.587
contig061433-BurTARs.A013	contig042499-NyeTARs.A028	0.392	0.668	0.587
contig022390-TilTARs.A056	contig062676-ZebTARs.A019	0.099	0.169	0.587
contig049540-BurTARs.A024	contig022390-TilTARs.A056	0.181	0.307	0.588
contig035375-NyeTARs.A013	contig046014-NyeTARs.A024	0.272	0.462	0.588
contig029633-BriTAR.A003	contig039639-TilTAR.A001	0.057	0.096	0.589
contig022390-TilTARs.A055	contig062677-ZebTARs.A018	0.191	0.324	0.590
contig034854-BurTARs.A027	contig062676-ZebTARs.A019	0.146	0.248	0.590
contig034854-BurTARs.A027	contig035377-NyeTARs.A023	0.155	0.263	0.590
contig029633-BriTAR.A003	contig040586-ZebTAR.A001	0.073	0.123	0.590
contig061091-BurTARs.A014	contig022378-TilTARs.A051	0.279	0.471	0.591
contig046013-NyeTARs.A021	contig022379-TilTARs.A052	0.274	0.464	0.591
contig084880-BriTARs.A016	contig084887-BriTARs.A018	0.222	0.376	0.592
contig061433-BurTARs.A013	contig060292-NyeTARs.A027	0.444	0.749	0.592
contig035376-NyeTARs.A014	contig035381-NyeTARs.A015	0.025	0.042	0.593
contig056200-NyeTARs.A029	contig022390-TilTARs.A056	0.182	0.307	0.593
contig046013-NyeTARs.A021	contig030440-ZebTARs.A029	0.290	0.489	0.594
contig035375-NyeTARs.A013	contig022320-TilTARs.A059	0.270	0.455	0.594
contig046011-NyeTARs.A016	contig022382-TilTARs.A053	0.315	0.531	0.594
contig022378-TilTARs.A051	contig030471-ZebTARs.A024	0.278	0.468	0.594
contig045999-NyeTARs.A026	contig045088-TilTARs.A057	0.426	0.716	0.594
contig082565-BriTARs.A022	contig035377-NyeTARs.A023	0.121	0.204	0.595
contig082565-BriTARs.A022	contig056023-BurTARs.A019	0.303	0.509	0.595
contig062039-NyeTARs.A022	contig022378-TilTARs.A051	0.028	0.047	0.595
contig046011-NyeTARs.A016	contig022379-TilTARs.A052	0.306	0.514	0.595
contig086351-BriTARs.A021	contig046011-NyeTARs.A016	0.475	0.797	0.596

contig084880-BriTARs.A016	contig045088-TilTARs.A057	0.423	0.709	0.596
contig034854-BurTARs.A027	contig022382-TilTARs.A053	0.172	0.289	0.596
contig035376-NyeTARs.A014	contig030445-ZebTARs.A026	0.021	0.035	0.596
contig046011-NyeTARs.A016	contig056200-NyeTARs.A029	0.325	0.546	0.596
contig061433-BurTARs.A013	contig034854-BurTARs.A027	0.312	0.522	0.597
contig084887-BriTARs.A018	contig035375-NyeTARs.A013	0.292	0.490	0.597
contig035377-NyeTARs.A023	contig062676-ZebTARs.A019	0.094	0.157	0.597
contig046011-NyeTARs.A016	contig046010-NyeTARs.A019	0.265	0.444	0.597
contig061091-BurTARs.A014	contig035375-NyeTARs.A013	0.297	0.496	0.598
contig084868-BriTARs.A014	contig082565-BriTARs.A022	0.123	0.205	0.599
contig084880-BriTARs.A016	contig030440-ZebTARs.A029	0.307	0.512	0.599
contig061433-BurTARs.A013	contig057301-BurTARs.A017	0.395	0.658	0.600
contig035377-NyeTARs.A023	contig030440-ZebTARs.A029	0.146	0.244	0.601
contig034854-BurTARs.A027	contig046013-NyeTARs.A021	0.291	0.484	0.602
contig022390-TilTARs.A055	contig066890-ZebTARs.A014	0.086	0.142	0.603
contig046011-NyeTARs.A016	contig022343-TilTARs.A033	0.257	0.426	0.603
contig022382-TilTARs.A053	contig022390-TilTARs.A055	0.186	0.308	0.603
contig049540-BurTARs.A024	contig022390-TilTARs.A055	0.186	0.308	0.603
contig056200-NyeTARs.A029	contig022390-TilTARs.A055	0.186	0.308	0.603
contig084880-BriTARs.A016	contig057301-BurTARs.A017	0.421	0.698	0.603
contig041024-BurTARs.A026	contig030471-ZebTARs.A024	0.053	0.088	0.605
contig045999-NyeTARs.A026	contig022353-TilTARs.A036	0.204	0.338	0.605
contig046011-NyeTARs.A016	contig022377-TilTARs.A050	0.275	0.454	0.605
contig056023-BurTARs.A019	contig042499-NyeTARs.A028	0.421	0.695	0.606
contig045999-NyeTARs.A026	contig030440-ZebTARs.A029	0.305	0.503	0.606
contig035375-NyeTARs.A013	contig022353-TilTARs.A036	0.309	0.509	0.607
contig045999-NyeTARs.A026	contig022382-TilTARs.A053	0.325	0.535	0.607
contig022379-TilTARs.A052	contig022390-TilTARs.A055	0.108	0.177	0.608
contig034854-BurTARs.A027	contig022383-TilTARs.A054	0.147	0.241	0.609
contig046011-NyeTARs.A016	contig022337-TilTARs.A031	0.263	0.431	0.611
contig035376-NyeTARs.A014	contig035377-NyeTARs.A023	0.127	0.207	0.612
contig084880-BriTARs.A016	contig022353-TilTARs.A036	0.204	0.333	0.613
contig046011-NyeTARs.A016	contig035377-NyeTARs.A023	0.325	0.530	0.613
contig022382-TilTARs.A053	contig030440-ZebTARs.A029	0.157	0.256	0.613
contig022382-TilTARs.A053	contig066890-ZebTARs.A014	0.184	0.300	0.614
contig084886-BriTARs.A017	contig030464-ZebTARs.A025	0.026	0.042	0.614
contig056023-BurTARs.A019	contig045088-TilTARs.A057	0.428	0.696	0.615
contig022363-TilTARs.A043	contig022378-TilTARs.A051	0.268	0.436	0.615
contig086351-BriTARs.A021	contig060292-NyeTARs.A027	0.022	0.035	0.616
contig046013-NyeTARs.A021	contig022378-TilTARs.A051	0.281	0.455	0.617
contig046011-NyeTARs.A016	contig022354-TilTARs.A037	0.076	0.122	0.619
contig056023-BurTARs.A019	contig030440-ZebTARs.A029	0.307	0.495	0.620
contig057301-BurTARs.A017	contig045999-NyeTARs.A026	0.441	0.711	0.620
contig039639-TilTAR.A001	contig039640-TilTAR.A002	0.047	0.076	0.620
contig084876-BriTARs.A015	contig084887-BriTARs.A018	0.309	0.498	0.621
contig035375-NyeTARs.A013	contig066890-ZebTARs.A014	0.151	0.242	0.621
contig035375-NyeTARs.A013	contig046013-NyeTARs.A021	0.301	0.483	0.622
contig046011-NyeTARs.A016	contig022363-TilTARs.A044	0.210	0.337	0.623
contig057305-BurTARs.A031	contig022390-TilTARs.A055	0.431	0.691	0.624
contig034854-BurTARs.A027	contig066890-ZebTARs.A014	0.155	0.248	0.625
contig059673-BurTARs.A016	contig022390-TilTARs.A055	0.512	0.815	0.628
contig084880-BriTARs.A016	contig035375-NyeTARs.A013	0.313	0.498	0.629
contig022382-TilTARs.A053	contig022390-TilTARs.A056	0.171	0.271	0.630

contig034854-BurTARs.A027	contig030440-ZebTARs.A029	0.051	0.080	0.632
contig056023-BurTARs.A019	contig022353-TilTARs.A036	0.206	0.326	0.632
contig084868-BriTARs.A014	contig022390-TilTARs.A055	0.214	0.339	0.633
contig062039-NyeTARs.A022	contig022390-TilTARs.A055	0.386	0.609	0.633
contig022390-TilTARs.A056	contig030440-ZebTARs.A029	0.140	0.222	0.633
contig084880-BriTARs.A016	contig034854-BurTARs.A027	0.327	0.515	0.635
contig035375-NyeTARs.A013	contig022390-TilTARs.A056	0.143	0.225	0.635
contig035375-NyeTARs.A013	contig045999-NyeTARs.A026	0.311	0.489	0.637
contig022390-TilTARs.A055	contig059768-ZebTARs.A022	0.377	0.593	0.637
contig084868-BriTARs.A014	contig034854-BurTARs.A027	0.157	0.245	0.639
contig057301-BurTARs.A017	contig056023-BurTARs.A019	0.443	0.691	0.641
contig034854-BurTARs.A027	contig045999-NyeTARs.A026	0.325	0.506	0.643
contig046011-NyeTARs.A016	contig022334-TilTARs.A030	0.237	0.369	0.643
contig056023-BurTARs.A019	contig035375-NyeTARs.A013	0.312	0.485	0.644
contig066890-ZebTARs.A014	contig030440-ZebTARs.A029	0.148	0.229	0.646
contig061977-BurTARs.A012	contig022390-TilTARs.A055	0.105	0.161	0.649
contig034854-BurTARs.A027	contig022353-TilTARs.A036	0.309	0.474	0.652
contig035381-NyeTARs.A015	contig022390-TilTARs.A056	0.117	0.179	0.652
contig039640-TilTAR.A002	contig040586-ZebTAR.A002	0.053	0.081	0.655
contig035381-NyeTARs.A015	contig066890-ZebTARs.A014	0.123	0.188	0.655
contig056023-BurTARs.A019	contig034854-BurTARs.A027	0.327	0.499	0.657
contig086351-BriTARs.A021	contig057305-BurTARs.A031	0.023	0.035	0.657
contig054630-BurTARs.A023	contig022390-TilTARs.A055	0.390	0.593	0.658
contig034854-BurTARs.A027	contig022390-TilTARs.A056	0.153	0.231	0.661
contig082565-BriTARs.A022	contig022390-TilTARs.A056	0.114	0.170	0.667
contig029633-BriTAR.A003	contig039640-TilTAR.A002	0.057	0.086	0.668
contig084886-BriTARs.A017	contig046011-NyeTARs.A016	0.280	0.419	0.669
contig039640-TilTAR.A003	contig040586-ZebTAR.A002	0.057	0.083	0.680
contig060292-NyeTARs.A027	contig022390-TilTARs.A055	0.432	0.635	0.681
contig086337-BriTARs.A019	contig007512-TilTARs.A024	0.024	0.035	0.683
contig007512-TilTARs.A024	contig053139-ZebTARs.A023	0.021	0.031	0.684
contig022390-TilTARs.A056	contig030445-ZebTARs.A026	0.114	0.166	0.686
contig066890-ZebTARs.A014	contig030445-ZebTARs.A026	0.120	0.175	0.688
contig022334-TilTARs.A030	contig022363-TilTARs.A043	0.293	0.425	0.690
contig086351-BriTARs.A021	contig022390-TilTARs.A055	0.425	0.611	0.695
contig035376-NyeTARs.A014	contig022390-TilTARs.A056	0.120	0.171	0.701
contig022390-TilTARs.A055	contig061410-ZebTARs.A021	0.112	0.158	0.708
contig084876-BriTARs.A015	contig022390-TilTARs.A055	0.108	0.152	0.713
contig082565-BriTARs.A022	contig066890-ZebTARs.A014	0.121	0.168	0.720
contig039639-TilTAR.A001	contig039642-TilTAR.A004	0.036	0.050	0.722
contig035381-NyeTARs.A015	contig030445-ZebTARs.A026	0.016	0.022	0.724
contig084876-BriTARs.A015	contig061410-ZebTARs.A021	0.016	0.022	0.732
contig084887-BriTARs.A018	contig066691-ZebTARs.A015	0.016	0.022	0.739
contig084887-BriTARs.A018	contig061091-BurTARs.A014	0.016	0.022	0.740
contig035376-NyeTARs.A014	contig066890-ZebTARs.A014	0.127	0.171	0.740
contig056021-BurTARs.A020	contig061417-ZebTARs.A020	0.014	0.019	0.745
contig020038-BurTAR.A002	contig003909-ZebTAR.A003	0.008	0.011	0.749
contig059766-BurTARs.A029	contig053145-ZebTARs.A028	0.003	0.004	0.760
contig039639-TilTAR.A001	contig039640-TilTAR.A003	0.049	0.064	0.762
contig029633-BriTAR.A003	contig039640-TilTAR.A003	0.070	0.092	0.764
contig056020-BurTARs.A021	contig046007-NyeTARs.A017	0.009	0.011	0.765
contig034854-BurTARs.A027	contig035375-NyeTARs.A013	0.052	0.067	0.774
contig035375-NyeTARs.A013	contig022390-TilTARs.A055	0.106	0.134	0.788

contig039640-TilTAR.A002	contig039642-TilTAR.A004	0.047	0.060	0.797
contig022378-TilTARs.A051	contig022390-TilTARs.A055	0.404	0.505	0.800
contig039640-TilTAR.A003	contig039642-TilTAR.A004	0.050	0.062	0.801
contig086337-BriTARs.A019	contig053139-ZebTARs.A023	0.016	0.019	0.803
contig022390-TilTARs.A055	contig022390-TilTARs.A056	0.073	0.083	0.874
contig025313-BriTAR.A002	contig020038-BurTAR.A002	0.014	0.015	0.941
contig038663-NyeTAR.A005	contig040586-ZebTAR.A001	0.007	0.007	0.995
contig057301-BurTARs.A017	contig042499-NyeTARs.A028	0.008	0.008	1.108
contig020038-BurTAR.A002	contig032272-NyeTAR.A004	0.008	0.007	1.127
contig057301-BurTARs.A017	contig045088-TilTARs.A057	0.014	0.011	1.239
contig022330-TilTARs.A028	contig022334-TilTARs.A029	0.015	0.011	1.368
contig042499-NyeTARs.A028	contig045088-TilTARs.A057	0.022	0.011	1.987

Fam B				
		dN	dS	dN/dS
contig037879-TilTAR.B060	contig037900-TilTAR.B063	0.238	0.736	0.324
contig037879-TilTAR.B060	contig002574-TilTAR.B062	0.240	0.689	0.348
contig035253-BriTAR.B025	contig037879-TilTAR.B060	0.239	0.680	0.352
contig006087-BurTAR.B032	contig033536-ZebTAR.B029	0.004	0.012	0.353
contig033536-ZebTAR.B029	contig052987-NyeTAR.B030	0.001	0.004	0.354
contig037879-TilTAR.B060	contig037889-TilTAR.B061	0.238	0.646	0.369
contig037889-TilTAR.B061	contig037900-TilTAR.B063	0.255	0.668	0.382
contig002574-TilTAR.B062	contig037900-TilTAR.B063	0.270	0.692	0.390
contig035253-BriTAR.B025	contig037900-TilTAR.B063	0.281	0.704	0.399
contig035253-BriTAR.B025	contig037889-TilTAR.B061	0.034	0.071	0.484
contig037879-TilTAR.B060	contig033536-ZebTAR.B029	0.460	0.936	0.491
contig006087-BurTAR.B032	contig037879-TilTAR.B060	0.459	0.931	0.493
contig037879-TilTAR.B060	contig052987-NyeTAR.B030	0.459	0.931	0.493
contig037900-TilTAR.B063	contig033536-ZebTAR.B029	0.449	0.867	0.518
contig035253-BriTAR.B025	contig006087-BurTAR.B032	0.462	0.880	0.525
contig037889-TilTAR.B061	contig002574-TilTAR.B062	0.033	0.063	0.526
contig035253-BriTAR.B025	contig033536-ZebTAR.B029	0.464	0.878	0.528
contig037900-TilTAR.B063	contig052987-NyeTAR.B030	0.452	0.854	0.529
contig035253-BriTAR.B025	contig052987-NyeTAR.B030	0.463	0.873	0.531
contig006087-BurTAR.B032	contig002574-TilTAR.B062	0.465	0.868	0.535
contig006087-BurTAR.B032	contig037900-TilTAR.B063	0.460	0.856	0.537
contig002574-TilTAR.B062	contig033536-ZebTAR.B029	0.466	0.867	0.538
contig002574-TilTAR.B062	contig052987-NyeTAR.B030	0.466	0.862	0.541
contig006087-BurTAR.B032	contig037889-TilTAR.B061	0.448	0.799	0.561
contig037889-TilTAR.B061	contig052987-NyeTAR.B030	0.449	0.793	0.567
contig037889-TilTAR.B061	contig033536-ZebTAR.B029	0.450	0.794	0.567
contig035253-BriTAR.B025	contig002574-TilTAR.B062	0.020	0.012	1.640
contig006087-BurTAR.B032	contig052987-NyeTAR.B030	0.001	0.000	>10
