# Supplementary material for: Identification and characterization of cichlid TAAR genes and comparison with other teleost TAAR repertoires
Source: BMC Genomics. 2015 Apr 23;16(1):335. doi: 10.1186/s12864-015-1478-4 (PMC4415300; doi:10.1186/s12864-015-1478-4)
Supplement: Additional file 9: — Prediction of the two-dimensional structure of cichlid TAARs by PolyPhobius [ 37 ]. [file 12864_2015_1478_MOESM9_ESM.pdf]

Phobius prediction

Prediction of contig066691-ZebTARs.A015\

```
ID   contig066691-ZebTARs.A015\
FT   TOPO_DOM      1     29       NON CYTOPLASMIC.
FT   TRANSMEM     30     54
FT   TOPO_DOM     55     65       CYTOPLASMIC.
FT   TRANSMEM     66     83
FT   TOPO_DOM     84    102       NON CYTOPLASMIC.
FT   TRANSMEM    103    124
FT   TOPO_DOM    125    144       CYTOPLASMIC.
FT   TRANSMEM    145    163
FT   TOPO_DOM    164    182       NON CYTOPLASMIC.
FT   TRANSMEM    183    214
FT   TOPO_DOM    215    249       CYTOPLASMIC.
FT   TRANSMEM    250    270
FT   TOPO_DOM    271    281       NON CYTOPLASMIC.
FT   TRANSMEM    282    305
FT   TOPO_DOM    306    329       CYTOPLASMIC.
//
```

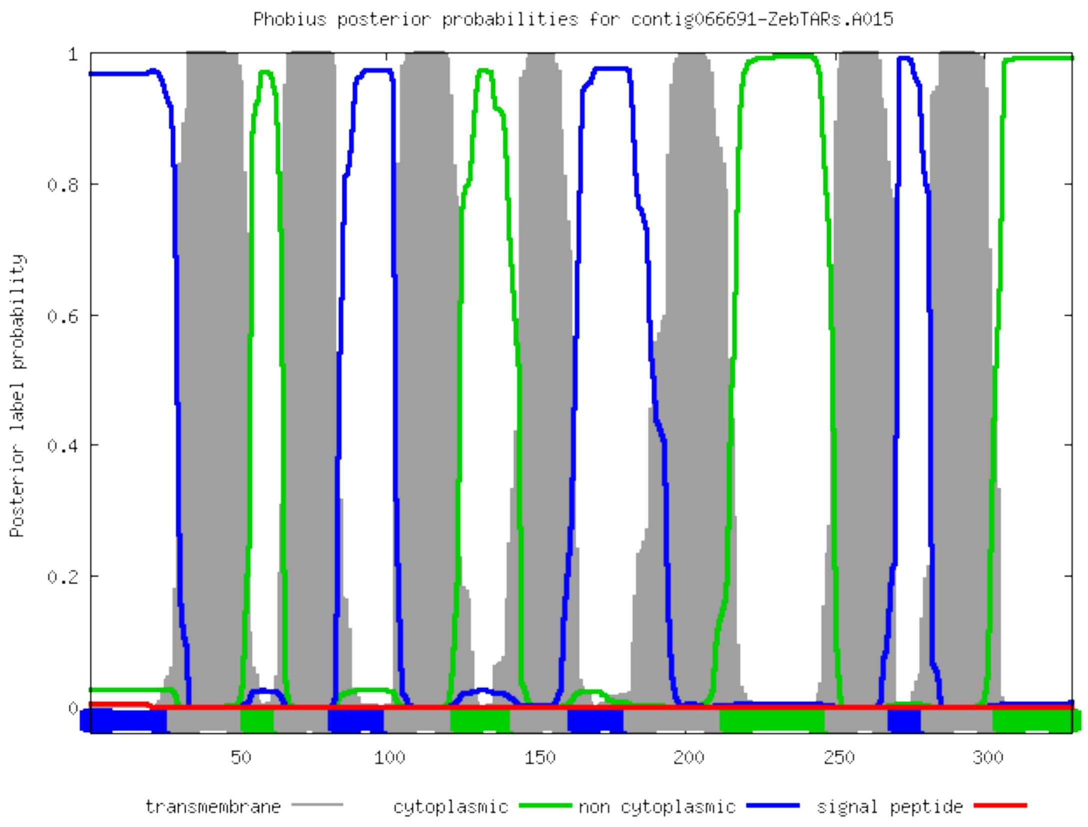

The probability data used in the plot is found here, and the gnuplot script is here.

Prediction of contig066285-ZebTARs.A016\

```
ID   contig066285-ZebTARs.A016\
FT   TOPO_DOM      1     27       NON CYTOPLASMIC.
FT   TRANSMEM     28     52
FT   TOPO_DOM     53     63       CYTOPLASMIC.
FT   TRANSMEM     64     81
```

```
FT   TOPO_DOM     82    100       NON CYTOPLASMIC.
FT   TRANSMEM    101    122
FT   TOPO_DOM    123    142       CYTOPLASMIC.
FT   TRANSMEM    143    166
FT   TOPO_DOM    167    185       NON CYTOPLASMIC.
FT   TRANSMEM    186    212
FT   TOPO_DOM    213    246       CYTOPLASMIC.
FT   TRANSMEM    247    267
FT   TOPO_DOM    268    278       NON CYTOPLASMIC.
FT   TRANSMEM    279    302
FT   TOPO_DOM    303    326       CYTOPLASMIC.
//
```

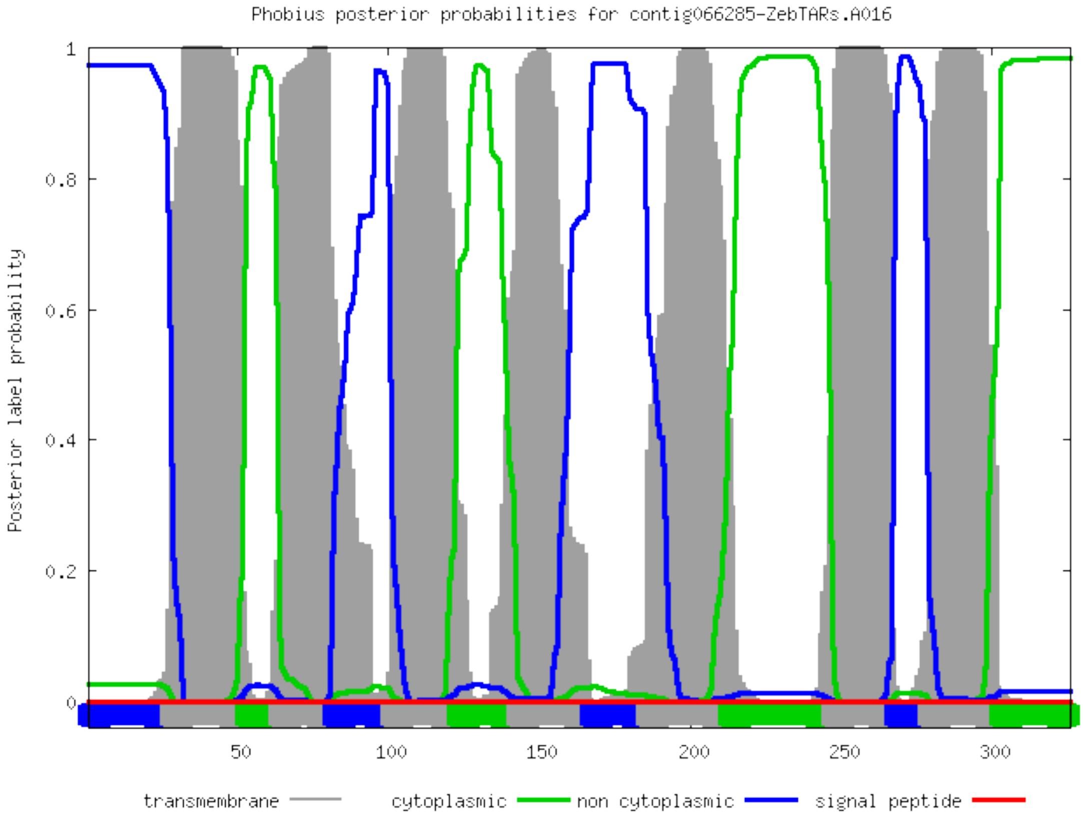

The probability data used in the plot is found here, and the gnuplot script is here.

Prediction of contig066056-ZebTARs.A017\

```
ID   contig066056-ZebTARs.A017\
FT   TOPO_DOM      1     28       NON CYTOPLASMIC.
FT   TRANSMEM     29     51
FT   TOPO_DOM     52     62       CYTOPLASMIC.
FT   TRANSMEM     63     86
FT   TOPO_DOM     87     97       NON CYTOPLASMIC.
FT   TRANSMEM     98    120
FT   TOPO_DOM    121    140       CYTOPLASMIC.
FT   TRANSMEM    141    160
FT   TOPO_DOM    161    179       NON CYTOPLASMIC.
FT   TRANSMEM    180    213
FT   TOPO_DOM    214    246       CYTOPLASMIC.
FT   TRANSMEM    247    268
FT   TOPO_DOM    269    282       NON CYTOPLASMIC.
FT   TRANSMEM    283    304
FT   TOPO_DOM    305    327       CYTOPLASMIC.
//
```

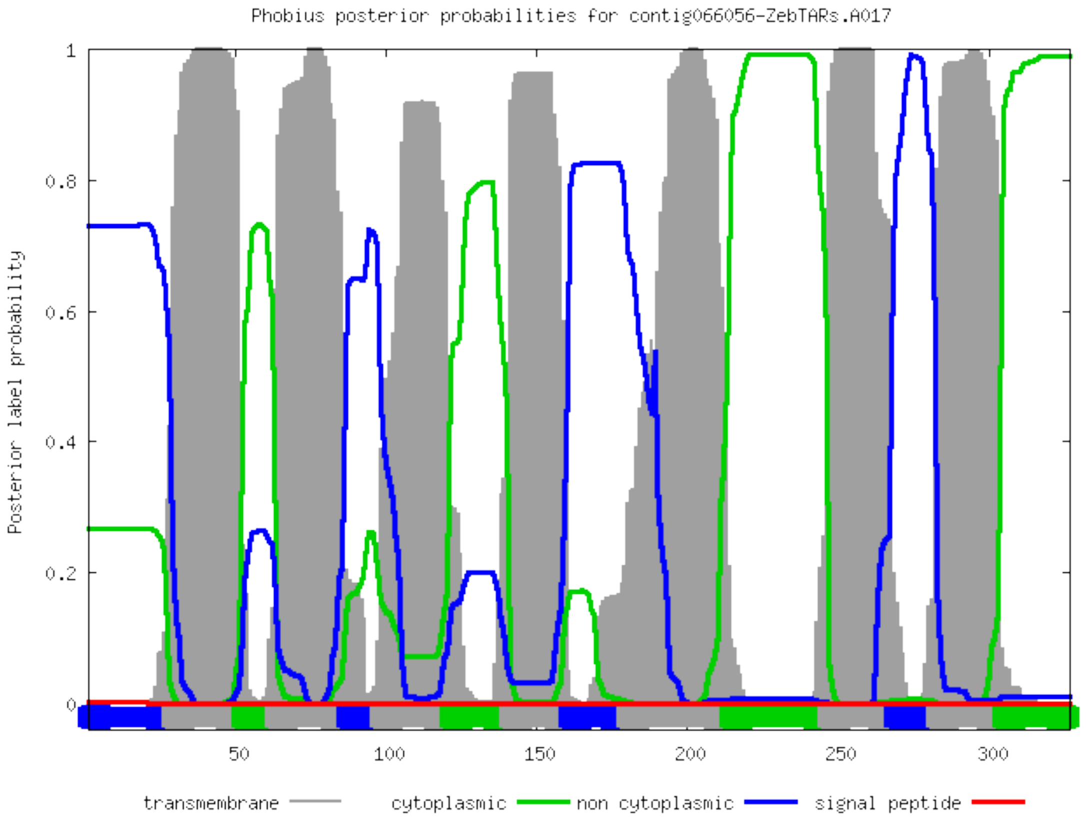

The probability data used in the plot is found here, and the gnuplot script is here.

Prediction of contig062677-ZebTARs.A018\

```
ID   contig062677-ZebTARs.A018\
FT   TOPO_DOM      1     29       NON CYTOPLASMIC.
FT   TRANSMEM     30     54
FT   TOPO_DOM     55     65       CYTOPLASMIC.
FT   TRANSMEM     66     82
FT   TOPO_DOM     83    101       NON CYTOPLASMIC.
FT   TRANSMEM    102    124
FT   TOPO_DOM    125    144       CYTOPLASMIC.
FT   TRANSMEM    145    165
FT   TOPO_DOM    166    184       NON CYTOPLASMIC.
FT   TRANSMEM    185    214
FT   TOPO_DOM    215    249       CYTOPLASMIC.
FT   TRANSMEM    250    270
FT   TOPO_DOM    271    281       NON CYTOPLASMIC.
FT   TRANSMEM    282    301
FT   TOPO_DOM    302    329       CYTOPLASMIC.
//
```

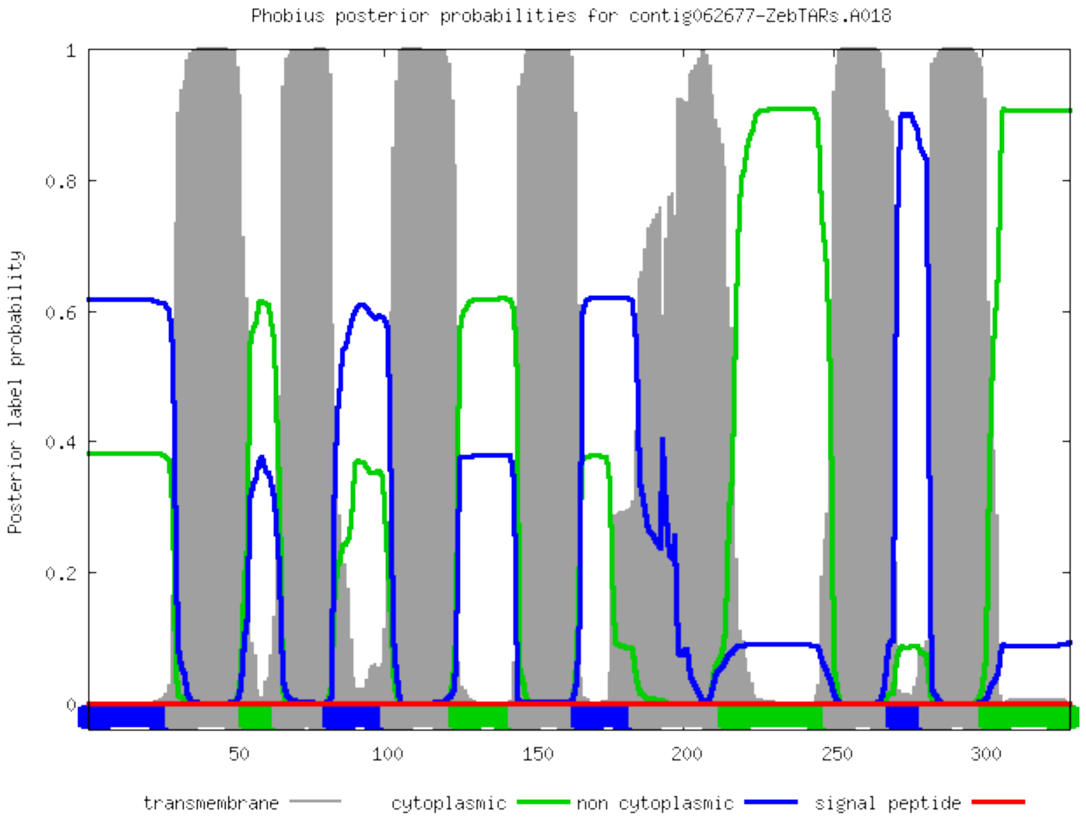

The probability data used in the plot is found here, and the gnuplot script is here.

Prediction of contig062676-ZebTARs.A019\

```
ID   contig062676-ZebTARs.A019\
FT   TOPO_DOM      1     29       NON CYTOPLASMIC.
FT   TRANSMEM     30     54
FT   TOPO_DOM     55     65       CYTOPLASMIC.
FT   TRANSMEM     66     83
FT   TOPO_DOM     84    102       NON CYTOPLASMIC.
FT   TRANSMEM    103    124
FT   TOPO_DOM    125    144       CYTOPLASMIC.
FT   TRANSMEM    145    164
FT   TOPO_DOM    165    193       NON CYTOPLASMIC.
FT   TRANSMEM    194    217
FT   TOPO_DOM    218    250       CYTOPLASMIC.
FT   TRANSMEM    251    273
FT   TOPO_DOM    274    284       NON CYTOPLASMIC.
FT   TRANSMEM    285    306
FT   TOPO_DOM    307    330       CYTOPLASMIC.
//
```

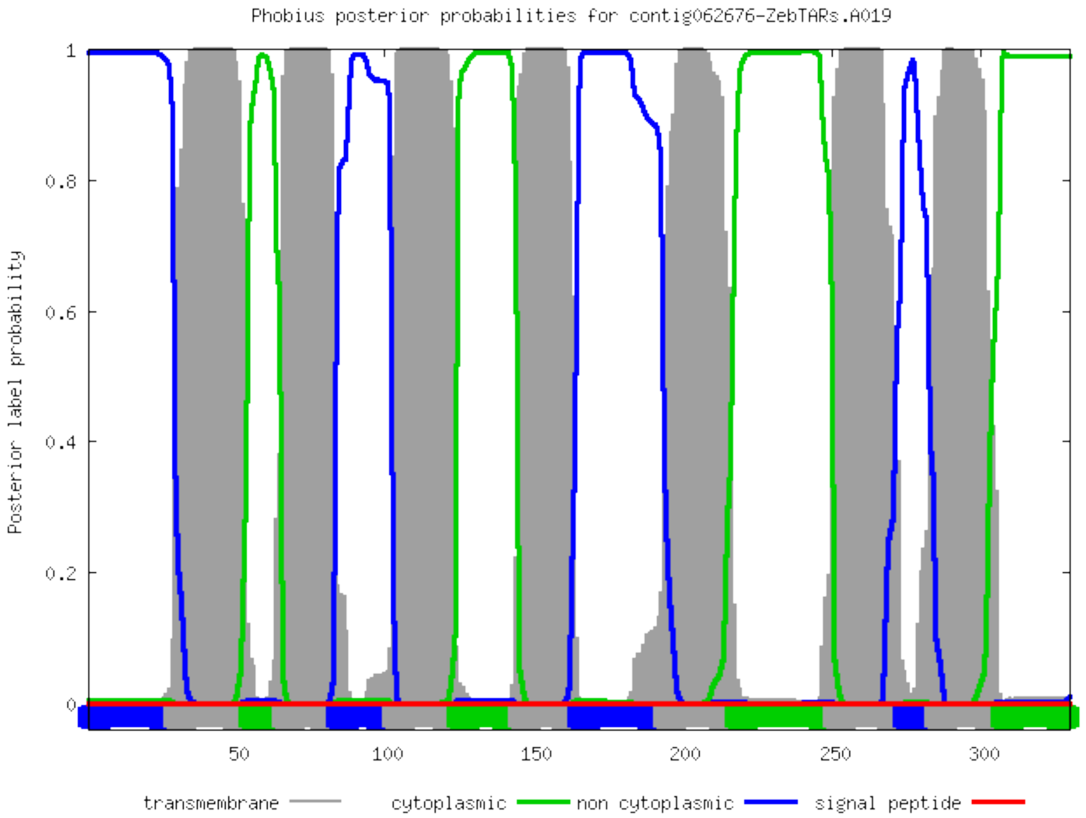

The probability data used in the plot is found here, and the gnuplot script is here.

Prediction of contig061417-ZebTARs.A020\

```
ID    contig061417-ZebTARs.A020\
FT    TOPO_DOM      1     29       NON CYTOPLASMIC.
FT    TRANSMEM     30     54
FT    TOPO_DOM     55     65       CYTOPLASMIC.
FT    TRANSMEM     66     83
FT    TOPO_DOM     84    102       NON CYTOPLASMIC.
FT    TRANSMEM    103    124
FT    TOPO_DOM    125    144       CYTOPLASMIC.
FT    TRANSMEM    145    164
FT    TOPO_DOM    165    193       NON CYTOPLASMIC.
FT    TRANSMEM    194    217
FT    TOPO_DOM    218    249       CYTOPLASMIC.
FT    TRANSMEM    250    270
FT    TOPO_DOM    271    281       NON CYTOPLASMIC.
FT    TRANSMEM    282    305
FT    TOPO_DOM    306    329       CYTOPLASMIC.
//
```

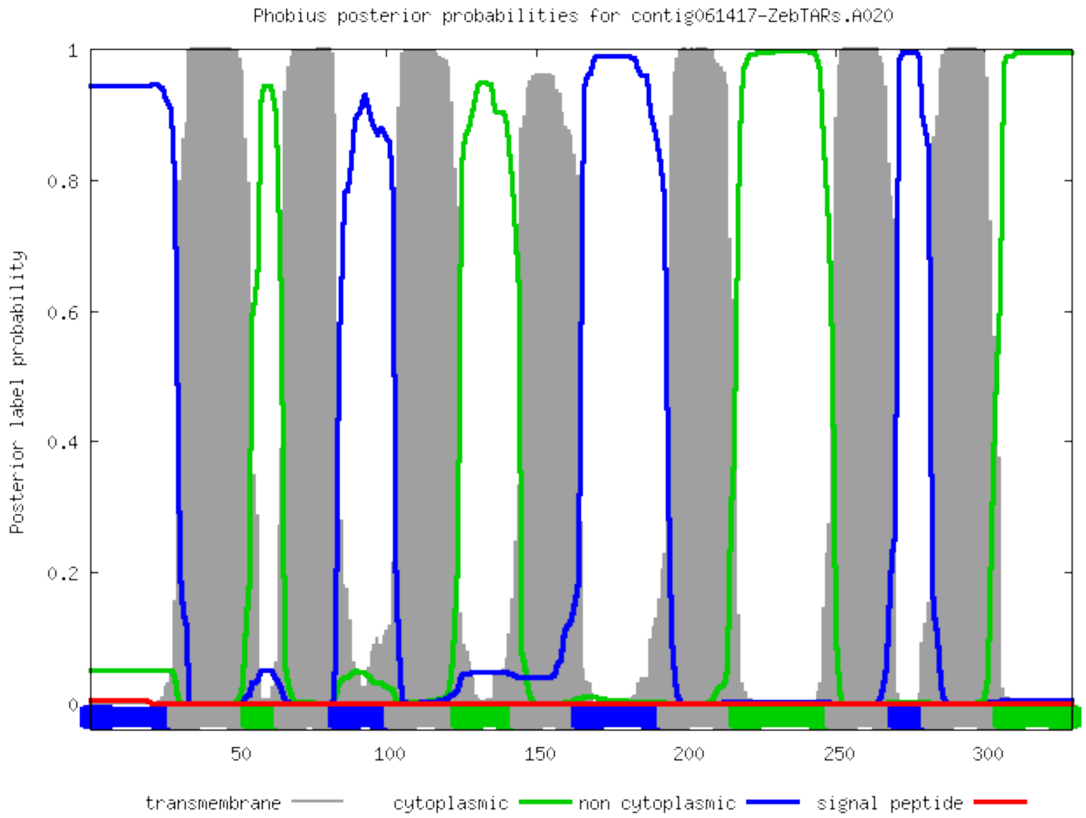

The probability data used in the plot is found here, and the gnuplot script is here.

Prediction of contig061410-ZebTARs.A021\

```
ID   contig061410-ZebTARs.A021\
FT   TOPO_DOM      1     27       NON CYTOPLASMIC.
FT   TRANSMEM     28     52
FT   TOPO_DOM     53     63       CYTOPLASMIC.
FT   TRANSMEM     64     81
FT   TOPO_DOM     82    100       NON CYTOPLASMIC.
FT   TRANSMEM    101    122
FT   TOPO_DOM    123    142       CYTOPLASMIC.
FT   TRANSMEM    143    162
FT   TOPO_DOM    163    191       NON CYTOPLASMIC.
FT   TRANSMEM    192    215
FT   TOPO_DOM    216    248       CYTOPLASMIC.
FT   TRANSMEM    249    269
FT   TOPO_DOM    270    280       NON CYTOPLASMIC.
FT   TRANSMEM    281    304
FT   TOPO_DOM    305    328       CYTOPLASMIC.
//
```

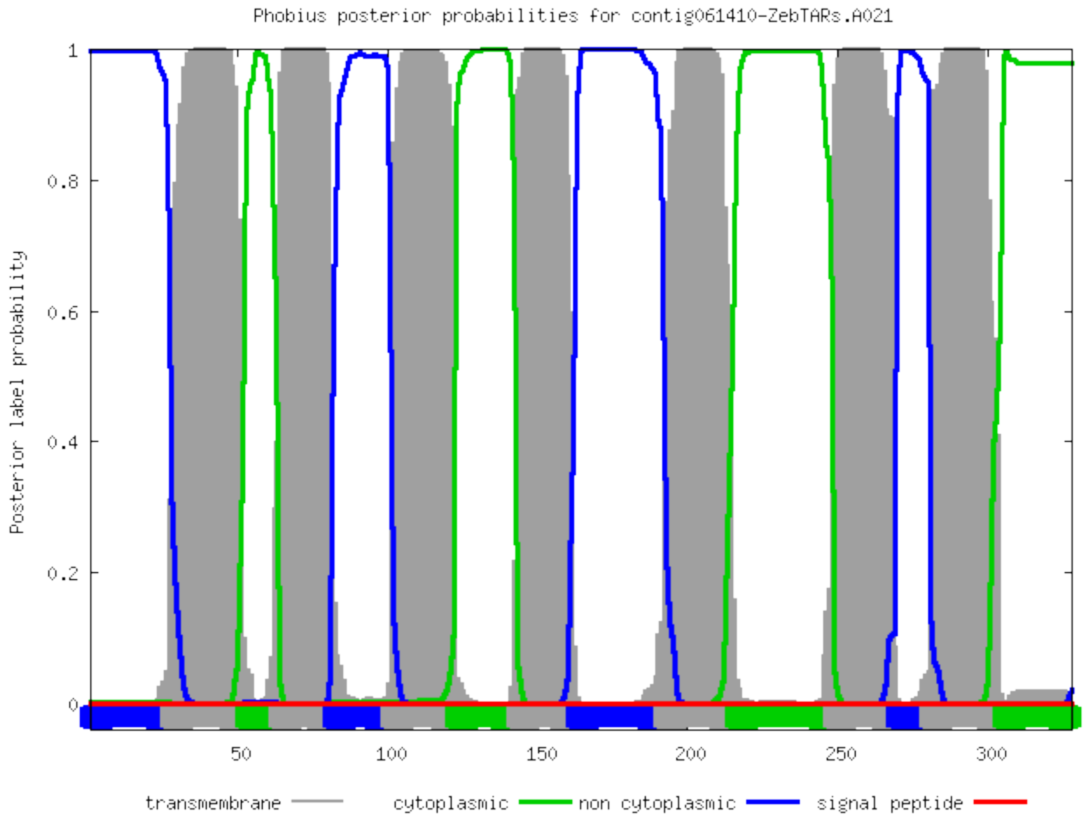

The probability data used in the plot is found here, and the gnuplot script is here.

Prediction of contig059768-ZebTARs.A022\

```
ID   contig059768-ZebTARs.A022\
FT   TOPO_DOM      1     29       NON CYTOPLASMIC.
FT   TRANSMEM     30     54
FT   TOPO_DOM     55     65       CYTOPLASMIC.
FT   TRANSMEM     66     83
FT   TOPO_DOM     84    102       NON CYTOPLASMIC.
FT   TRANSMEM    103    124
FT   TOPO_DOM    125    144       CYTOPLASMIC.
FT   TRANSMEM    145    164
FT   TOPO_DOM    165    183       NON CYTOPLASMIC.
FT   TRANSMEM    184    209
FT   TOPO_DOM    210    246       CYTOPLASMIC.
FT   TRANSMEM    247    267
FT   TOPO_DOM    268    286       NON CYTOPLASMIC.
FT   TRANSMEM    287    307
FT   TOPO_DOM    308    331       CYTOPLASMIC.
//
```

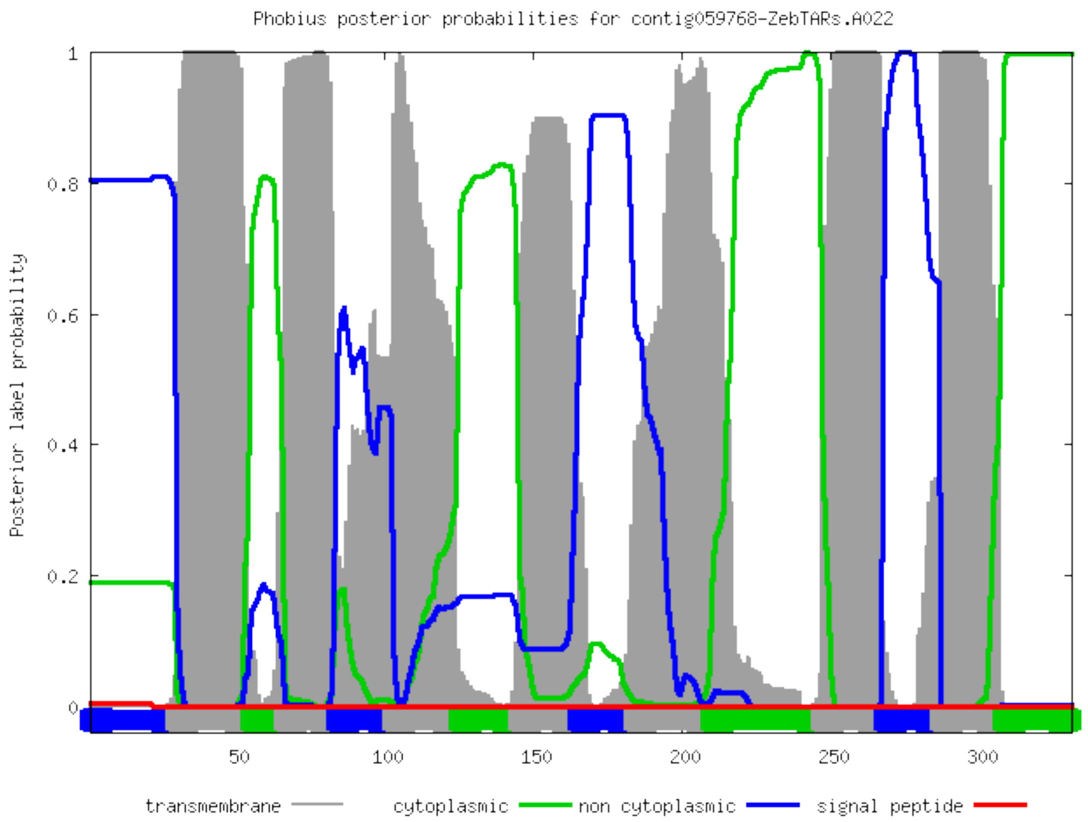

The probability data used in the plot is found here, and the gnuplot script is here.

Prediction of contig053139-ZebTARs.A023\

```
ID   contig053139-ZebTARs.A023\
FT   TOPO_DOM      1     26       NON CYTOPLASMIC.
FT   TRANSMEM     27     51
FT   TOPO_DOM     52     62       CYTOPLASMIC.
FT   TRANSMEM     63     93
FT   TOPO_DOM     94     98       NON CYTOPLASMIC.
FT   TRANSMEM     99    120
FT   TOPO_DOM    121    140       CYTOPLASMIC.
FT   TRANSMEM    141    160
FT   TOPO_DOM    161    179       NON CYTOPLASMIC.
FT   TRANSMEM    180    210
FT   TOPO_DOM    211    245       CYTOPLASMIC.
FT   TRANSMEM    246    266
FT   TOPO_DOM    267    277       NON CYTOPLASMIC.
FT   TRANSMEM    278    298
FT   TOPO_DOM    299    326       CYTOPLASMIC.
//
```

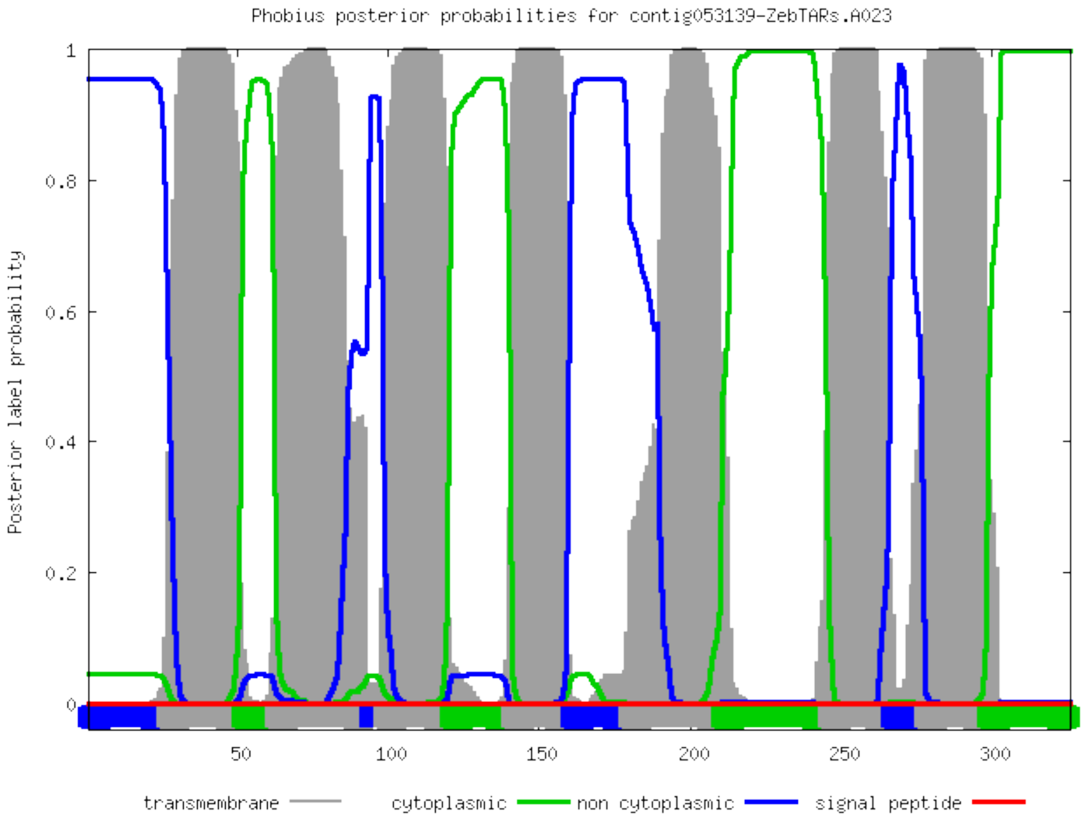

The probability data used in the plot is found here, and the gnuplot script is here.

Prediction of contig030471-ZebTARs.A024\

```
ID    contig030471-ZebTARs.A024\
FT    TOPO_DOM       1      29       NON CYTOPLASMIC.
FT    TRANSMEM      30      54
FT    TOPO_DOM      55      65       CYTOPLASMIC.
FT    TRANSMEM      66      83
FT    TOPO_DOM      84     102       NON CYTOPLASMIC.
FT    TRANSMEM     103     124
FT    TOPO_DOM     125     144       CYTOPLASMIC.
FT    TRANSMEM     145     163
FT    TOPO_DOM     164     182       NON CYTOPLASMIC.
FT    TRANSMEM     183     214
FT    TOPO_DOM     215     249       CYTOPLASMIC.
FT    TRANSMEM     250     270
FT    TOPO_DOM     271     281       NON CYTOPLASMIC.
FT    TRANSMEM     282     301
FT    TOPO_DOM     302     329       CYTOPLASMIC.
//
```

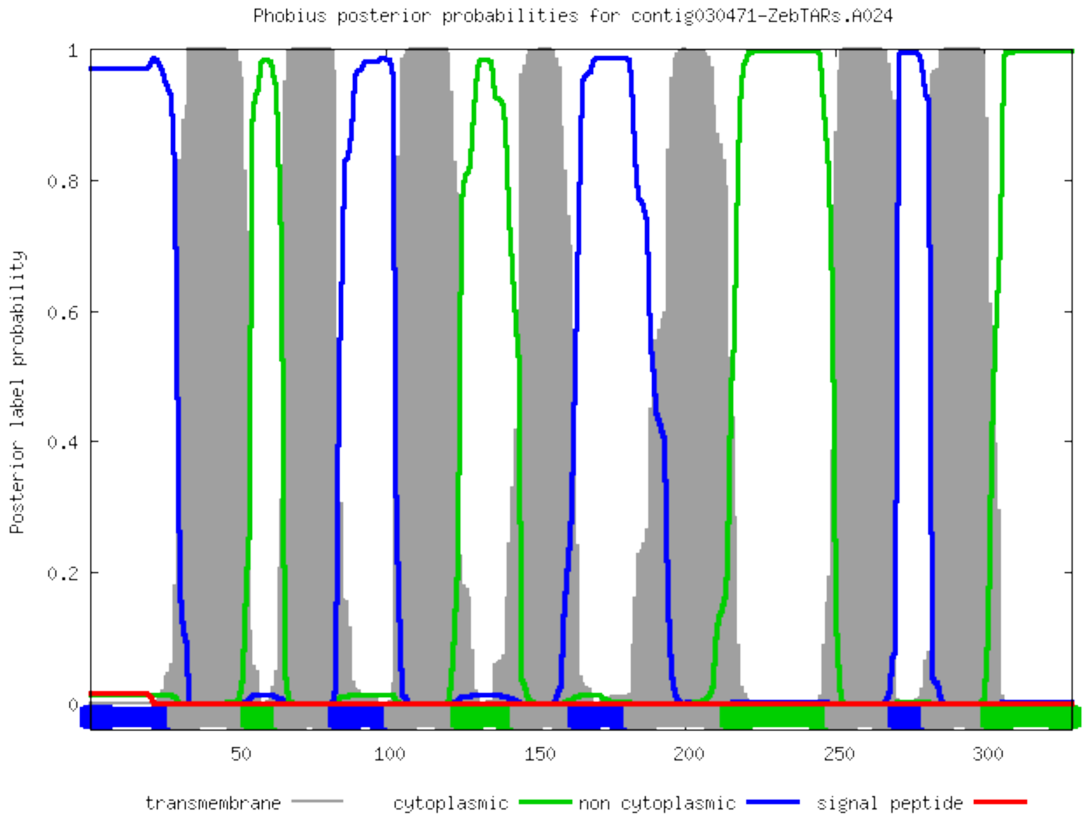

The probability data used in the plot is found here, and the gnuplot script is here.

Prediction of contig030464-ZebTARs.A025\

```
ID    contig030464-ZebTARs.A025\
FT    TOPO_DOM       1     27       NON CYTOPLASMIC.
FT    TRANSMEM      28     52
FT    TOPO_DOM      53     63       CYTOPLASMIC.
FT    TRANSMEM      64     81
FT    TOPO_DOM      82    100       NON CYTOPLASMIC.
FT    TRANSMEM     101    122
FT    TOPO_DOM     123    142       CYTOPLASMIC.
FT    TRANSMEM     143    162
FT    TOPO_DOM     163    191       NON CYTOPLASMIC.
FT    TRANSMEM     192    215
FT    TOPO_DOM     216    247       CYTOPLASMIC.
FT    TRANSMEM     248    268
FT    TOPO_DOM     269    279       NON CYTOPLASMIC.
FT    TRANSMEM     280    303
FT    TOPO_DOM     304    327       CYTOPLASMIC.
//
```

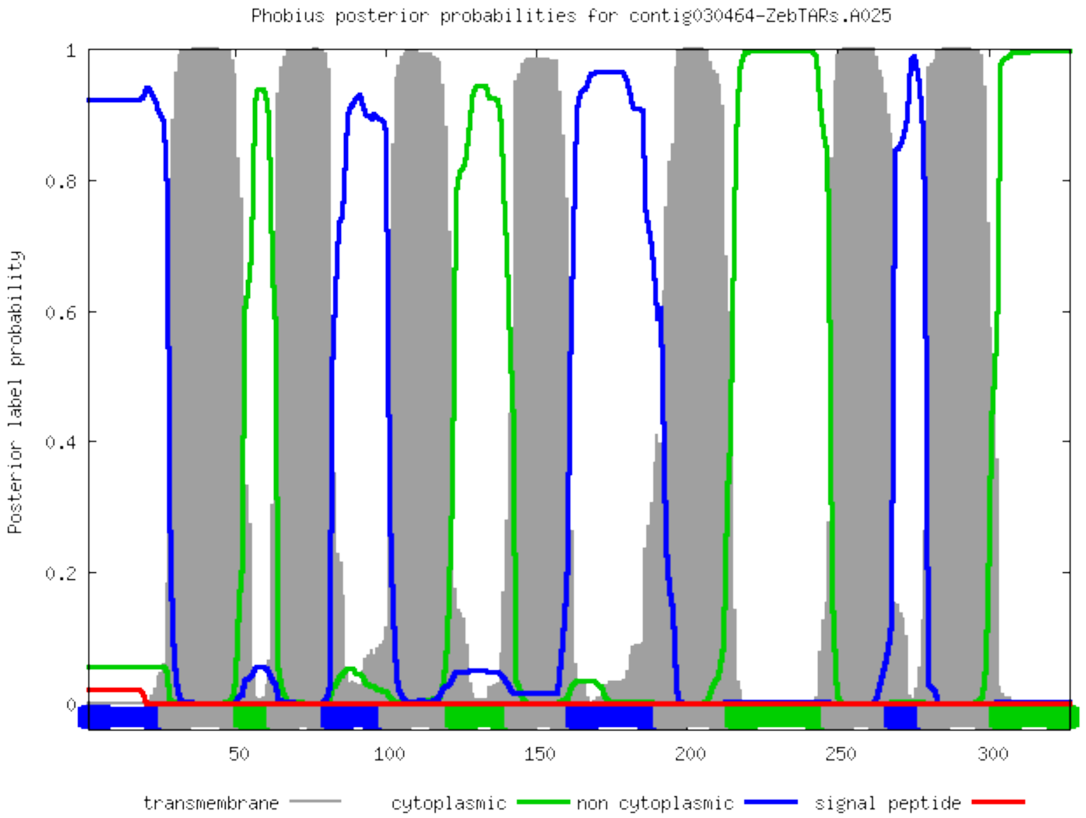

The probability data used in the plot is found here, and the gnuplot script is here.

Prediction of contig030445-ZebTARs.A026\

```
ID   contig030445-ZebTARs.A026\
FT   TOPO_DOM      1     29       NON CYTOPLASMIC.
FT   TRANSMEM     30     54
FT   TOPO_DOM     55     65       CYTOPLASMIC.
FT   TRANSMEM     66     83
FT   TOPO_DOM     84    102       NON CYTOPLASMIC.
FT   TRANSMEM    103    124
FT   TOPO_DOM    125    144       CYTOPLASMIC.
FT   TRANSMEM    145    165
FT   TOPO_DOM    166    193       NON CYTOPLASMIC.
FT   TRANSMEM    194    217
FT   TOPO_DOM    218    250       CYTOPLASMIC.
FT   TRANSMEM    251    271
FT   TOPO_DOM    272    282       NON CYTOPLASMIC.
FT   TRANSMEM    283    306
FT   TOPO_DOM    307    330       CYTOPLASMIC.
//
```

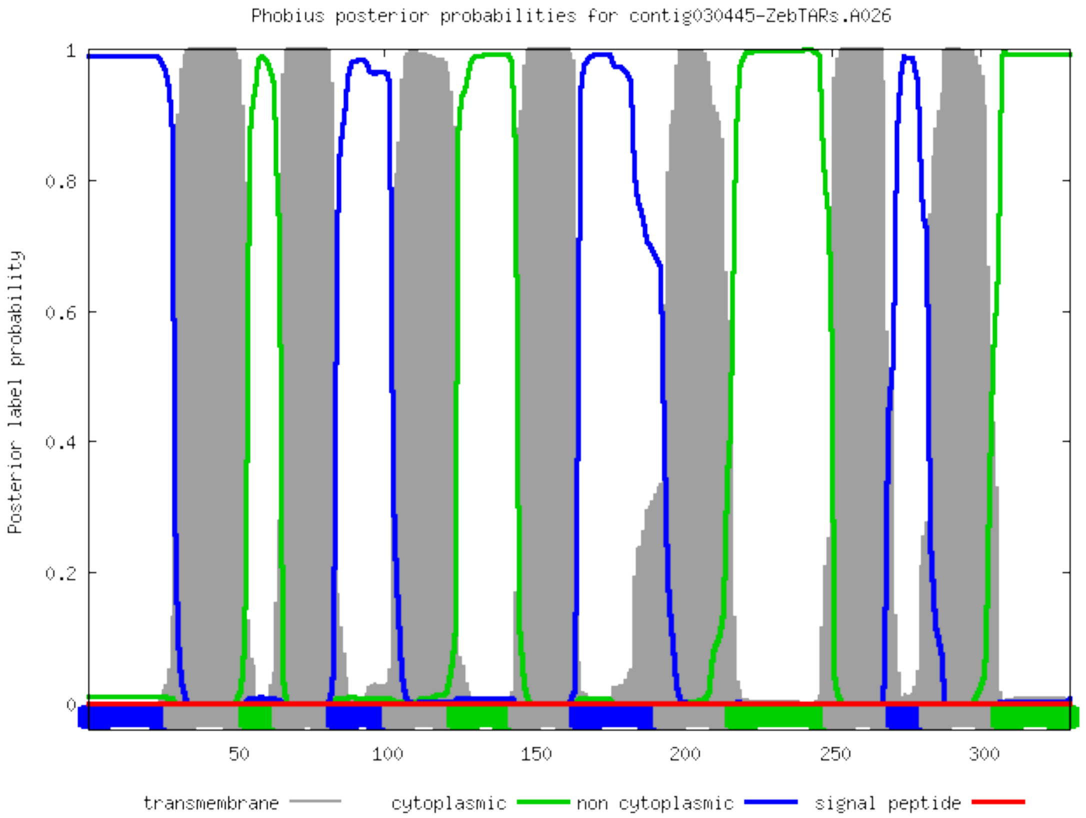

The probability data used in the plot is found here, and the gnuplot script is here.

Prediction of contig066330-ZebTARs.A027\

```
ID    contig066330-ZebTARs.A027\
FT    TOPO_DOM       1      26       NON CYTOPLASMIC.
FT    TRANSMEM      27      51
FT    TOPO_DOM      52      62       CYTOPLASMIC.
FT    TRANSMEM      63      93
FT    TOPO_DOM      94      98       NON CYTOPLASMIC.
FT    TRANSMEM      99     120
FT    TOPO_DOM     121     140       CYTOPLASMIC.
FT    TRANSMEM     141     161
FT    TOPO_DOM     162     191       NON CYTOPLASMIC.
FT    TRANSMEM     192     213
FT    TOPO_DOM     214     242       CYTOPLASMIC.
FT    TRANSMEM     243     266
FT    TOPO_DOM     267     277       NON CYTOPLASMIC.
FT    TRANSMEM     278     298
FT    TOPO_DOM     299     326       CYTOPLASMIC.
//
```

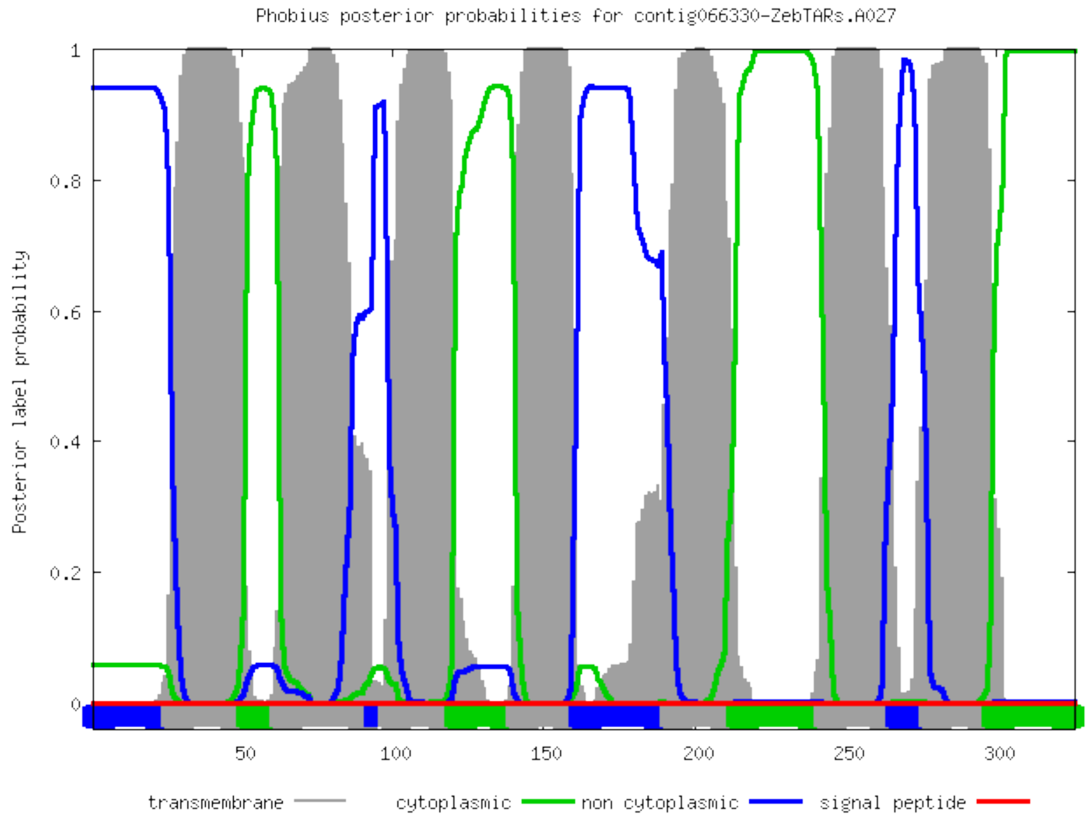

The probability data used in the plot is found here, and the gnuplot script is here.

Prediction of contig040586-ZebTAR.A001\

```
ID   contig040586-ZebTAR.A001\
FT   TOPO_DOM      1     28       NON CYTOPLASMIC.
FT   TRANSMEM     29     53
FT   TOPO_DOM     54     64       CYTOPLASMIC.
FT   TRANSMEM     65     86
FT   TOPO_DOM     87    105       NON CYTOPLASMIC.
FT   TRANSMEM    106    124
FT   TOPO_DOM    125    144       CYTOPLASMIC.
FT   TRANSMEM    145    166
FT   TOPO_DOM    167    193       NON CYTOPLASMIC.
FT   TRANSMEM    194    217
FT   TOPO_DOM    218    244       CYTOPLASMIC.
FT   TRANSMEM    245    262
FT   TOPO_DOM    263    273       NON CYTOPLASMIC.
FT   TRANSMEM    274    298
FT   TOPO_DOM    299    326       CYTOPLASMIC.
//
```

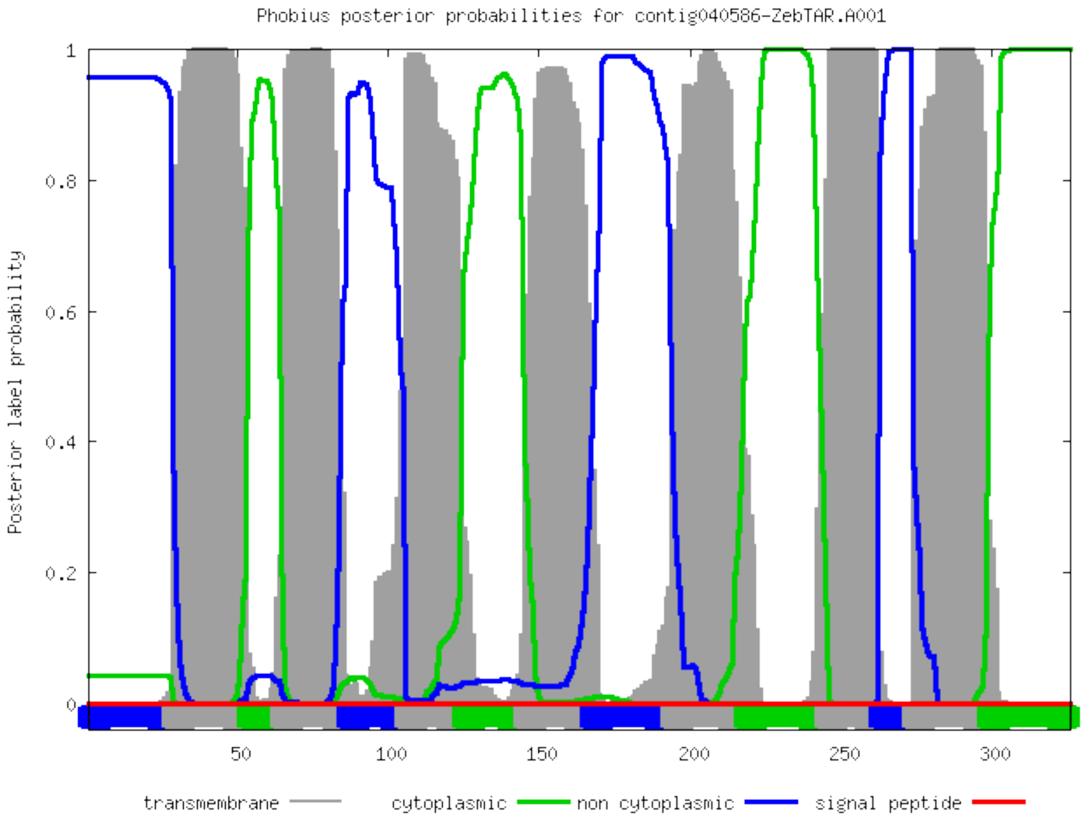

The probability data used in the plot is found here, and the gnuplot script is here.

Prediction of contig040586-ZebTAR.A002\

```
ID   contig040586-ZebTAR.A002\
FT   TOPO_DOM      1     28       NON CYTOPLASMIC.
FT   TRANSMEM     29     53
FT   TOPO_DOM     54     64       CYTOPLASMIC.
FT   TRANSMEM     65     86
FT   TOPO_DOM     87    105       NON CYTOPLASMIC.
FT   TRANSMEM    106    124
FT   TOPO_DOM    125    144       CYTOPLASMIC.
FT   TRANSMEM    145    166
FT   TOPO_DOM    167    193       NON CYTOPLASMIC.
FT   TRANSMEM    194    217
FT   TOPO_DOM    218    244       CYTOPLASMIC.
FT   TRANSMEM    245    262
FT   TOPO_DOM    263    273       NON CYTOPLASMIC.
FT   TRANSMEM    274    298
FT   TOPO_DOM    299    326       CYTOPLASMIC.
//
```

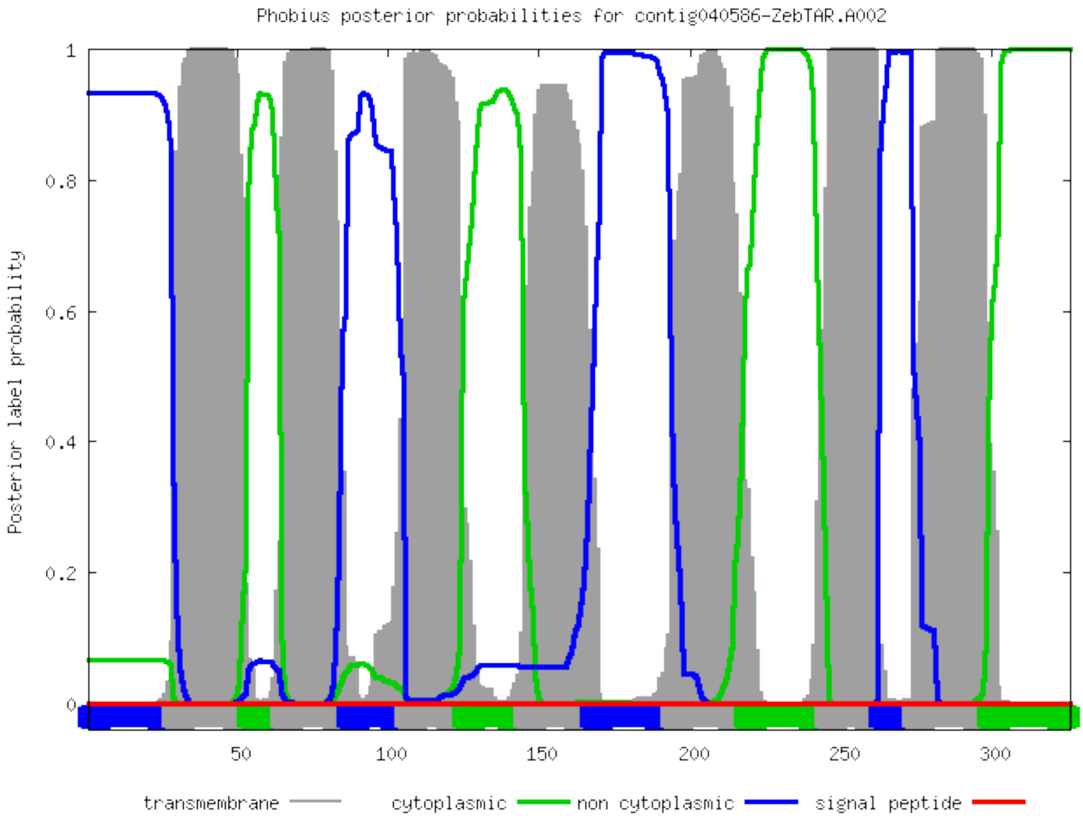

The probability data used in the plot is found here, and the gnuplot script is here.

Prediction of contig033536-ZebTAR.B029\

```
ID   contig033536-ZebTAR.B029\
FT   TOPO_DOM      1     24       NON CYTOPLASMIC.
FT   TRANSMEM     25     52
FT   TOPO_DOM     53     60       CYTOPLASMIC.
FT   TRANSMEM     61     85
FT   TOPO_DOM     86    104       NON CYTOPLASMIC.
FT   TRANSMEM    105    125
FT   TOPO_DOM    126    145       CYTOPLASMIC.
FT   TRANSMEM    146    166
FT   TOPO_DOM    167    171       NON CYTOPLASMIC.
FT   TRANSMEM    172    196
FT   TOPO_DOM    197    233       CYTOPLASMIC.
FT   TRANSMEM    234    257
FT   TOPO_DOM    258    268       NON CYTOPLASMIC.
FT   TRANSMEM    269    291
FT   TOPO_DOM    292    315       CYTOPLASMIC.
//
```

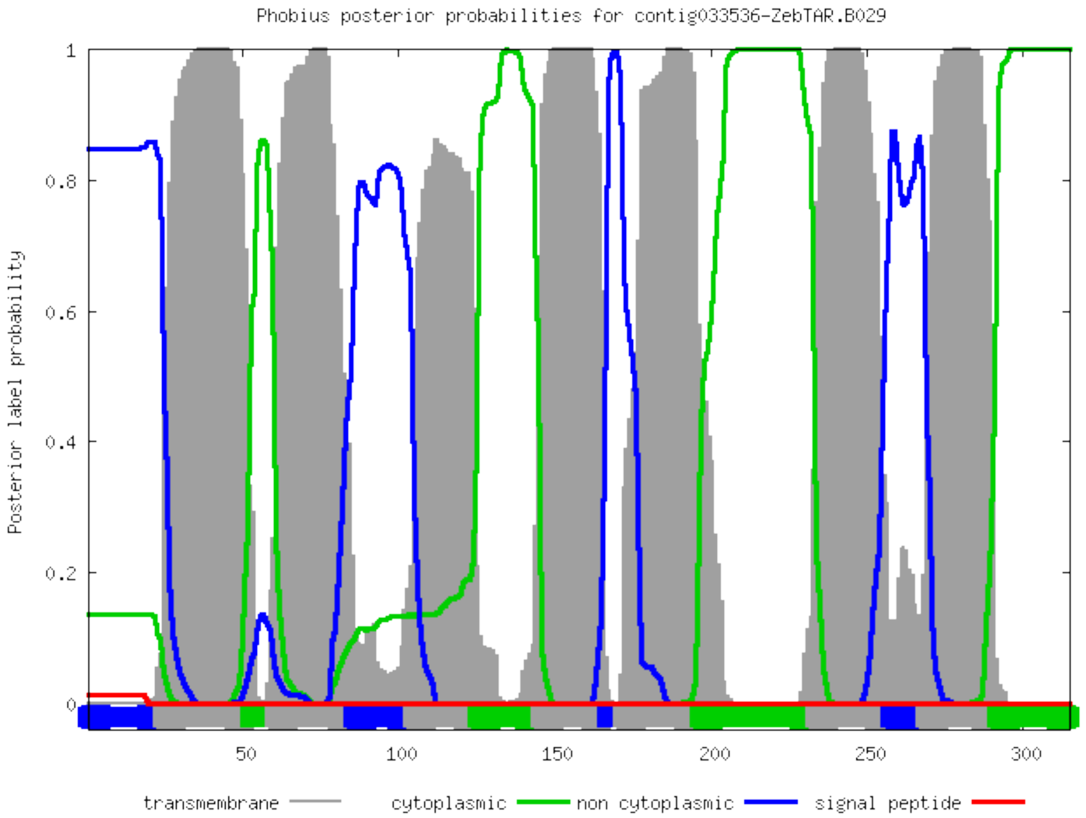

The probability data used in the plot is found here, and the gnuplot script is here.

Prediction of contig003909-ZebTAR.A003\

```
ID   contig003909-ZebTAR.A003\
FT   TOPO_DOM      1     29       NON CYTOPLASMIC.
FT   TRANSMEM     30     54
FT   TOPO_DOM     55     65       CYTOPLASMIC.
FT   TRANSMEM     66     83
FT   TOPO_DOM     84    102       NON CYTOPLASMIC.
FT   TRANSMEM    103    125
FT   TOPO_DOM    126    145       CYTOPLASMIC.
FT   TRANSMEM    146    165
FT   TOPO_DOM    166    194       NON CYTOPLASMIC.
FT   TRANSMEM    195    218
FT   TOPO_DOM    219    246       CYTOPLASMIC.
FT   TRANSMEM    247    267
FT   TOPO_DOM    268    286       NON CYTOPLASMIC.
FT   TRANSMEM    287    307
FT   TOPO_DOM    308    331       CYTOPLASMIC.
//
```

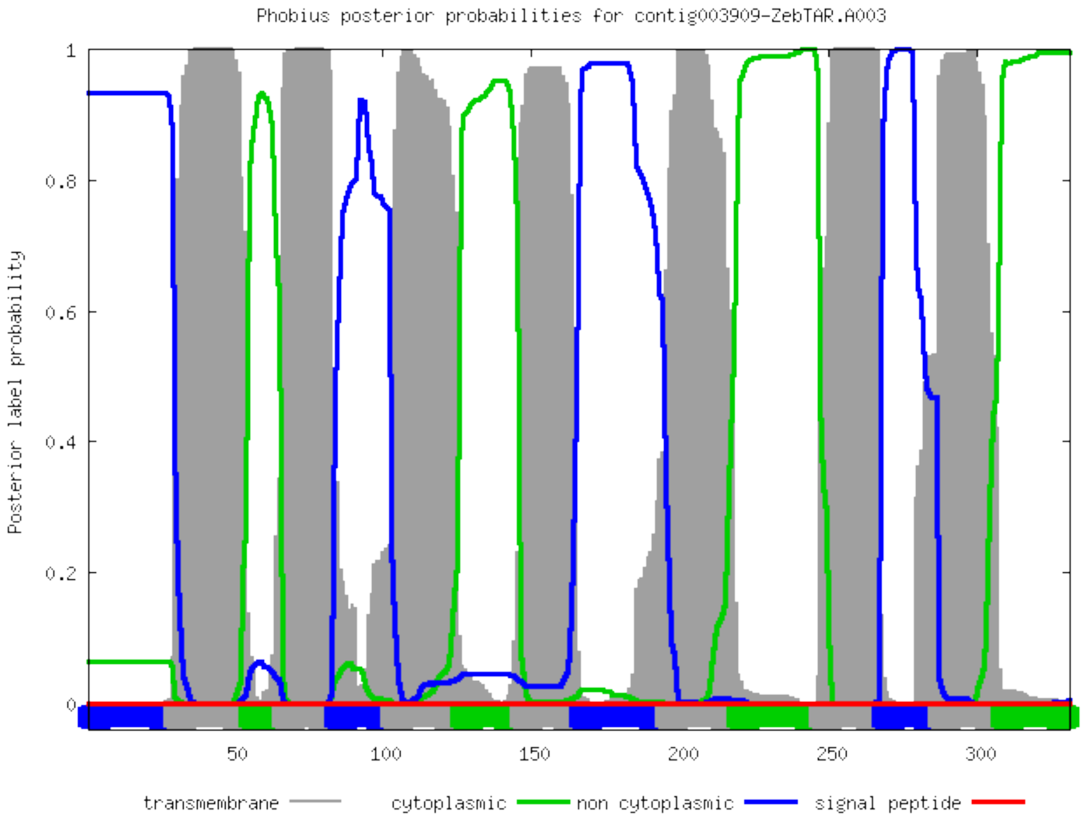

The probability data used in the plot is found here, and the gnuplot script is here.

Prediction of contig053145-ZebTARs.A028\

```
ID   contig053145-ZebTARs.A028\
FT   TOPO_DOM      1     26       NON CYTOPLASMIC.
FT   TRANSMEM     27     51
FT   TOPO_DOM     52     62       CYTOPLASMIC.
FT   TRANSMEM     63     93
FT   TOPO_DOM     94     98       NON CYTOPLASMIC.
FT   TRANSMEM     99    120
FT   TOPO_DOM    121    140       CYTOPLASMIC.
FT   TRANSMEM    141    161
FT   TOPO_DOM    162    191       NON CYTOPLASMIC.
FT   TRANSMEM    192    213
FT   TOPO_DOM    214    242       CYTOPLASMIC.
FT   TRANSMEM    243    266
FT   TOPO_DOM    267    277       NON CYTOPLASMIC.
FT   TRANSMEM    278    298
FT   TOPO_DOM    299    326       CYTOPLASMIC.
//
```

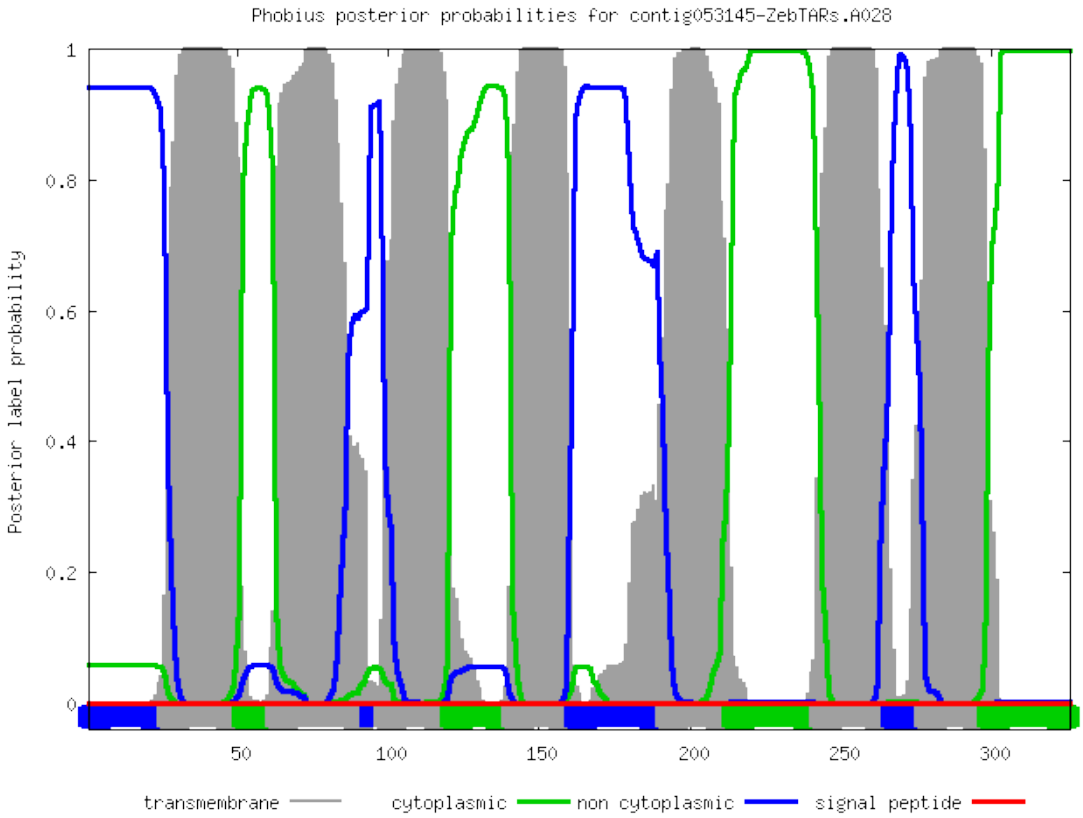

The probability data used in the plot is found here, and the gnuplot script is here.

Prediction of contig030440-ZebTARs.A029\

```
ID    contig030440-ZebTARs.A029\
FT    TOPO_DOM       1      29       NON CYTOPLASMIC.
FT    TRANSMEM      30      54
FT    TOPO_DOM      55      65       CYTOPLASMIC.
FT    TRANSMEM      66      83
FT    TOPO_DOM      84     102       NON CYTOPLASMIC.
FT    TRANSMEM     103     124
FT    TOPO_DOM     125     144       CYTOPLASMIC.
FT    TRANSMEM     145     164
FT    TOPO_DOM     165     193       NON CYTOPLASMIC.
FT    TRANSMEM     194     217
FT    TOPO_DOM     218     250       CYTOPLASMIC.
FT    TRANSMEM     251     271
FT    TOPO_DOM     272     282       NON CYTOPLASMIC.
FT    TRANSMEM     283     306
FT    TOPO_DOM     307     330       CYTOPLASMIC.
//
```

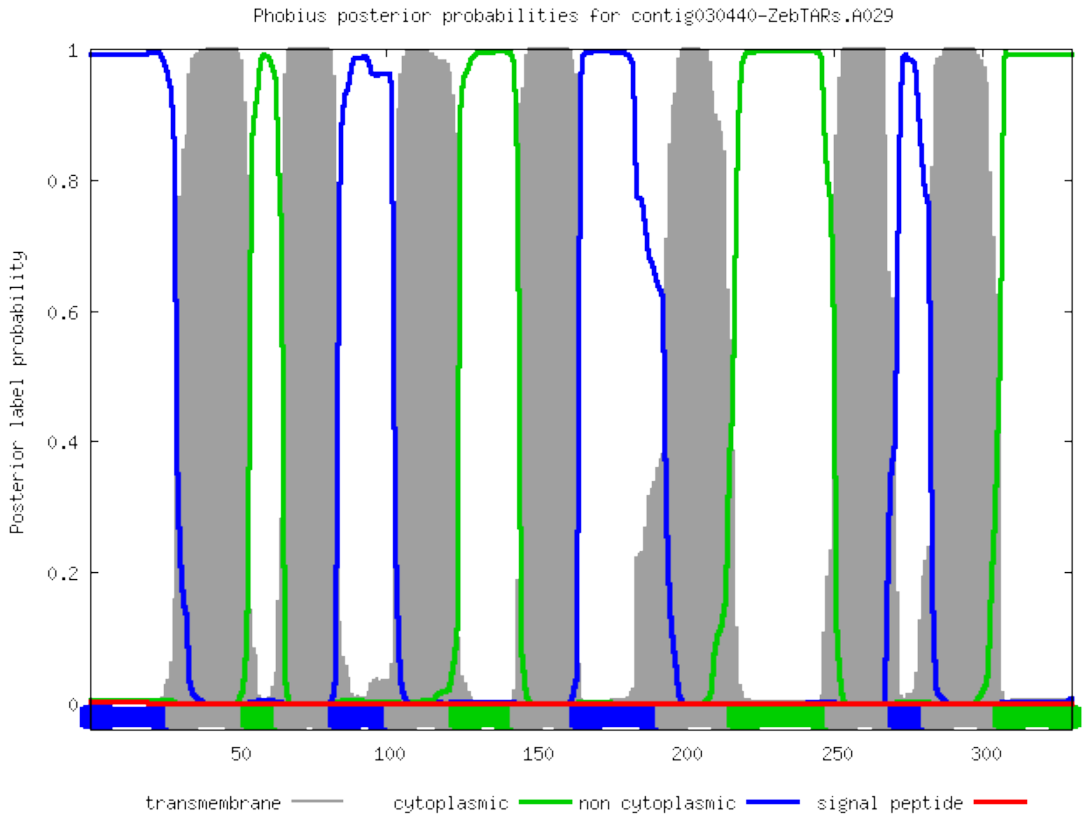

The probability data used in the plot is found here, and the gnuplot script is here.

Prediction of contig037879-TilTAR.B060\

```
ID   contig037879-TilTAR.B060\
FT   TOPO_DOM      1     22       NON CYTOPLASMIC.
FT   TRANSMEM     23     50
FT   TOPO_DOM     51     58       CYTOPLASMIC.
FT   TRANSMEM     59     80
FT   TOPO_DOM     81     99       NON CYTOPLASMIC.
FT   TRANSMEM    100    118
FT   TOPO_DOM    119    137       CYTOPLASMIC.
FT   TRANSMEM    138    162
FT   TOPO_DOM    163    181       NON CYTOPLASMIC.
FT   TRANSMEM    182    203
FT   TOPO_DOM    204    234       CYTOPLASMIC.
FT   TRANSMEM    235    259
FT   TOPO_DOM    260    270       NON CYTOPLASMIC.
FT   TRANSMEM    271    293
FT   TOPO_DOM    294    317       CYTOPLASMIC.
//
```

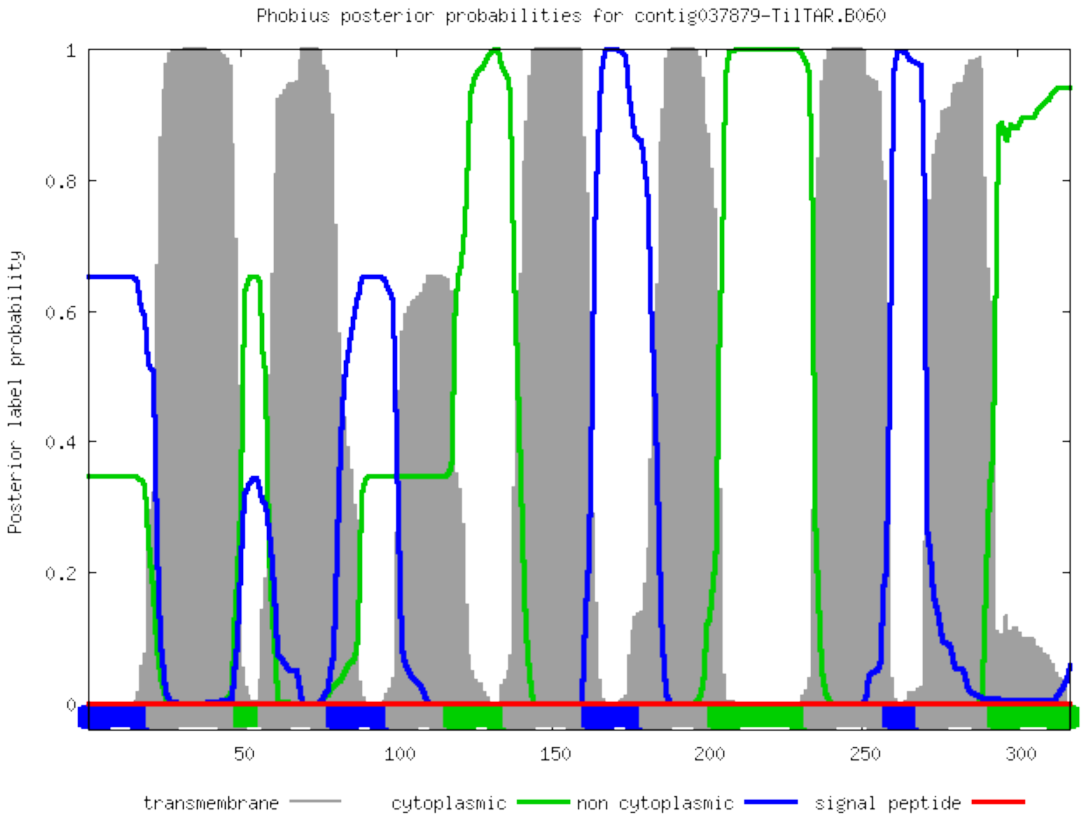

The probability data used in the plot is found here, and the gnuplot script is here.

Prediction of contig037889-TilTAR.B061\

```
ID   contig037889-TilTAR.B061\
FT   TOPO_DOM      1     29       NON CYTOPLASMIC.
FT   TRANSMEM     30     55
FT   TOPO_DOM     56     66       CYTOPLASMIC.
FT   TRANSMEM     67     87
FT   TOPO_DOM     88    106       NON CYTOPLASMIC.
FT   TRANSMEM    107    125
FT   TOPO_DOM    126    145       CYTOPLASMIC.
FT   TRANSMEM    146    169
FT   TOPO_DOM    170    188       NON CYTOPLASMIC.
FT   TRANSMEM    189    210
FT   TOPO_DOM    211    239       CYTOPLASMIC.
FT   TRANSMEM    240    263
FT   TOPO_DOM    264    282       NON CYTOPLASMIC.
FT   TRANSMEM    283    303
FT   TOPO_DOM    304    327       CYTOPLASMIC.
//
```

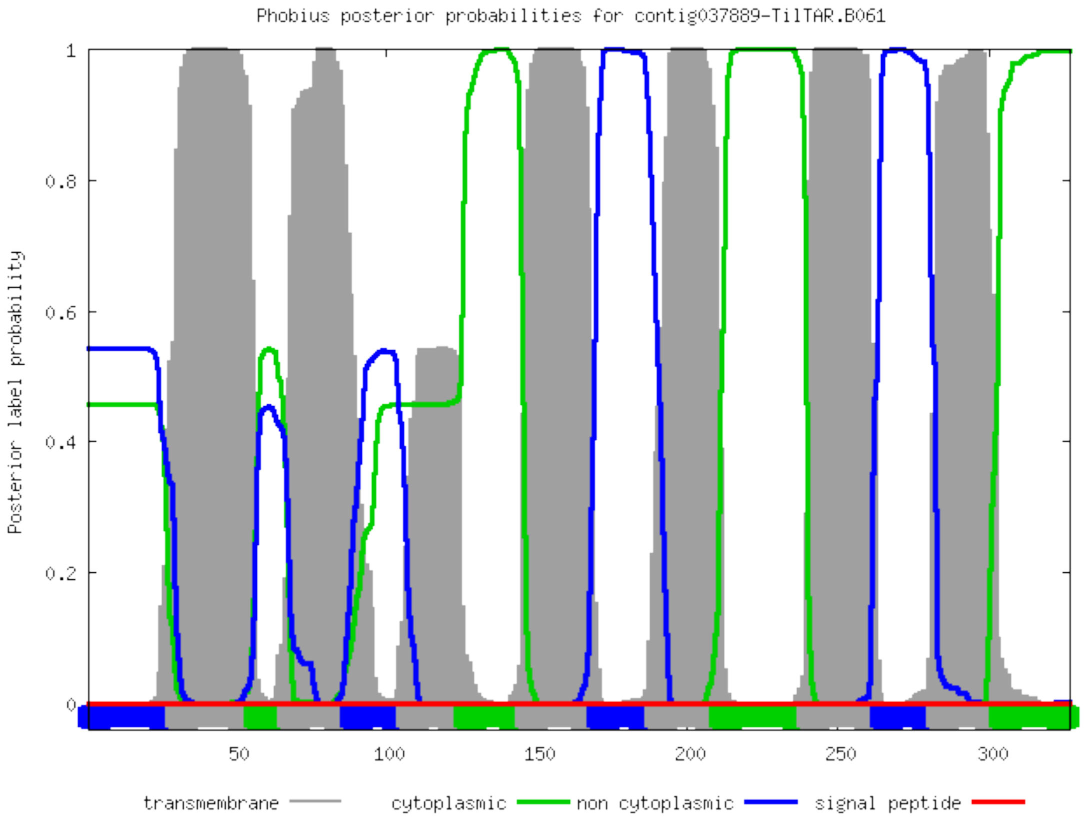

The probability data used in the plot is found here, and the gnuplot script is here.

Prediction of contig039639-TilTAR.A001\

```
ID    contig039639-TilTAR.A001\
FT    TOPO_DOM       1      28        NON CYTOPLASMIC.
FT    TRANSMEM      29      53
FT    TOPO_DOM      54      64        CYTOPLASMIC.
FT    TRANSMEM      65      86
FT    TOPO_DOM      87     105        NON CYTOPLASMIC.
FT    TRANSMEM     106     124
FT    TOPO_DOM     125     144        CYTOPLASMIC.
FT    TRANSMEM     145     166
FT    TOPO_DOM     167     193        NON CYTOPLASMIC.
FT    TRANSMEM     194     217
FT    TOPO_DOM     218     244        CYTOPLASMIC.
FT    TRANSMEM     245     262
FT    TOPO_DOM     263     273        NON CYTOPLASMIC.
FT    TRANSMEM     274     302
FT    TOPO_DOM     303     326        CYTOPLASMIC.
//
```

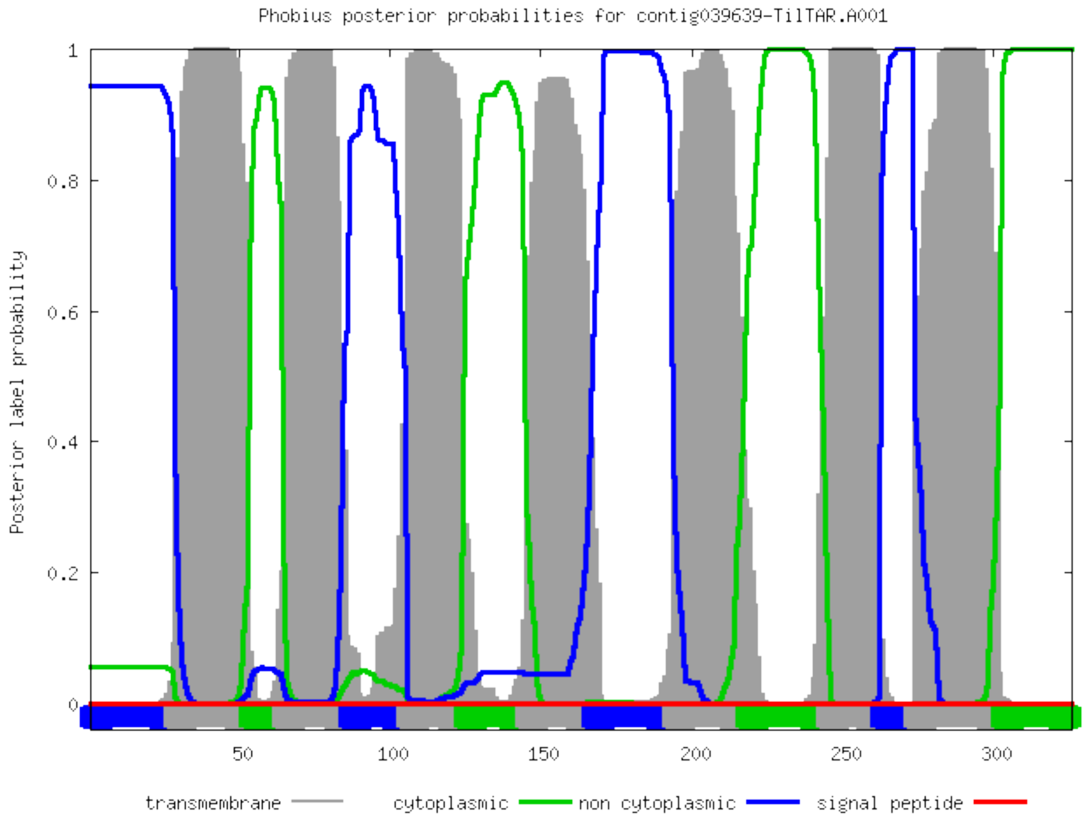

The probability data used in the plot is found here, and the gnuplot script is here.

Prediction of contig039640-TilTAR.A002\

```
ID    contig039640-TilTAR.A002\
FT    TOPO_DOM       1     28       NON CYTOPLASMIC.
FT    TRANSMEM      29     53
FT    TOPO_DOM      54     64       CYTOPLASMIC.
FT    TRANSMEM      65     86
FT    TOPO_DOM      87    105       NON CYTOPLASMIC.
FT    TRANSMEM     106    124
FT    TOPO_DOM     125    144       CYTOPLASMIC.
FT    TRANSMEM     145    166
FT    TOPO_DOM     167    193       NON CYTOPLASMIC.
FT    TRANSMEM     194    217
FT    TOPO_DOM     218    244       CYTOPLASMIC.
FT    TRANSMEM     245    262
FT    TOPO_DOM     263    273       NON CYTOPLASMIC.
FT    TRANSMEM     274    298
FT    TOPO_DOM     299    326       CYTOPLASMIC.
//
```

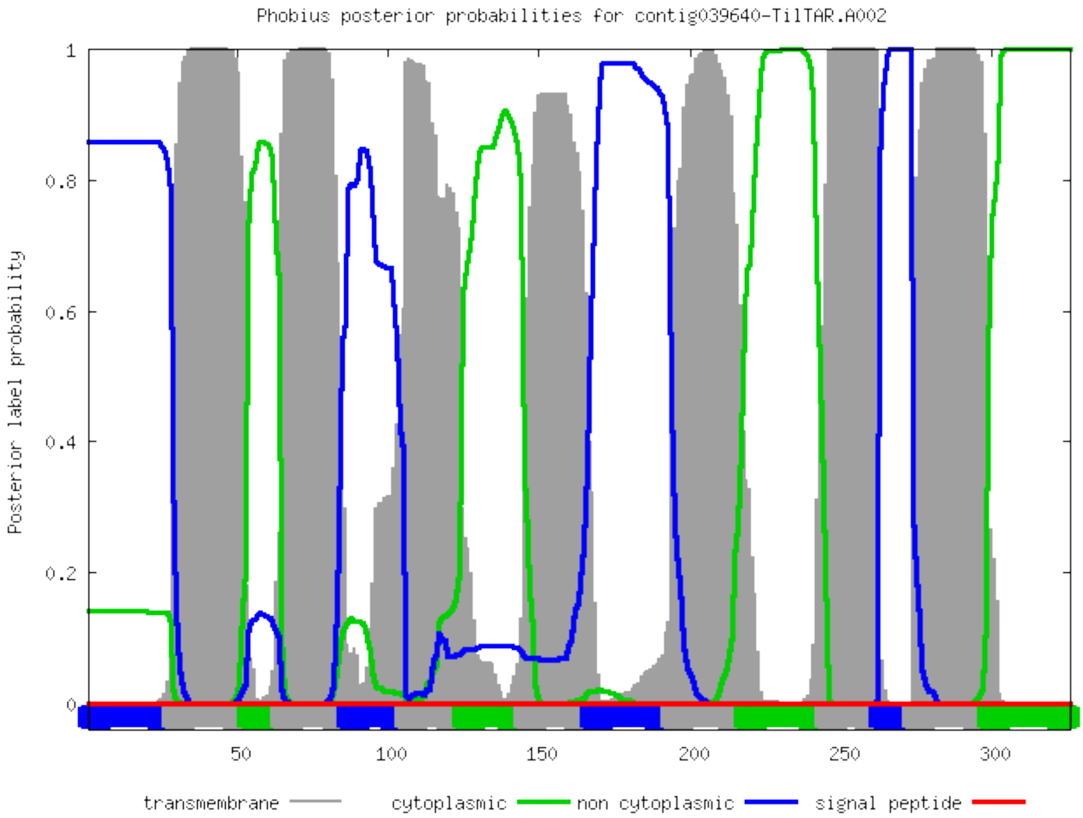

The probability data used in the plot is found here, and the gnuplot script is here.

Prediction of contig039640-TilTAR.A003\

```
ID    contig039640-TilTAR.A003\
FT    TOPO_DOM       1      28       NON CYTOPLASMIC.
FT    TRANSMEM      29      53
FT    TOPO_DOM      54      64       CYTOPLASMIC.
FT    TRANSMEM      65      86
FT    TOPO_DOM      87     105       NON CYTOPLASMIC.
FT    TRANSMEM     106     124
FT    TOPO_DOM     125     144       CYTOPLASMIC.
FT    TRANSMEM     145     166
FT    TOPO_DOM     167     193       NON CYTOPLASMIC.
FT    TRANSMEM     194     217
FT    TOPO_DOM     218     244       CYTOPLASMIC.
FT    TRANSMEM     245     262
FT    TOPO_DOM     263     273       NON CYTOPLASMIC.
FT    TRANSMEM     274     298
FT    TOPO_DOM     299     326       CYTOPLASMIC.
//
```

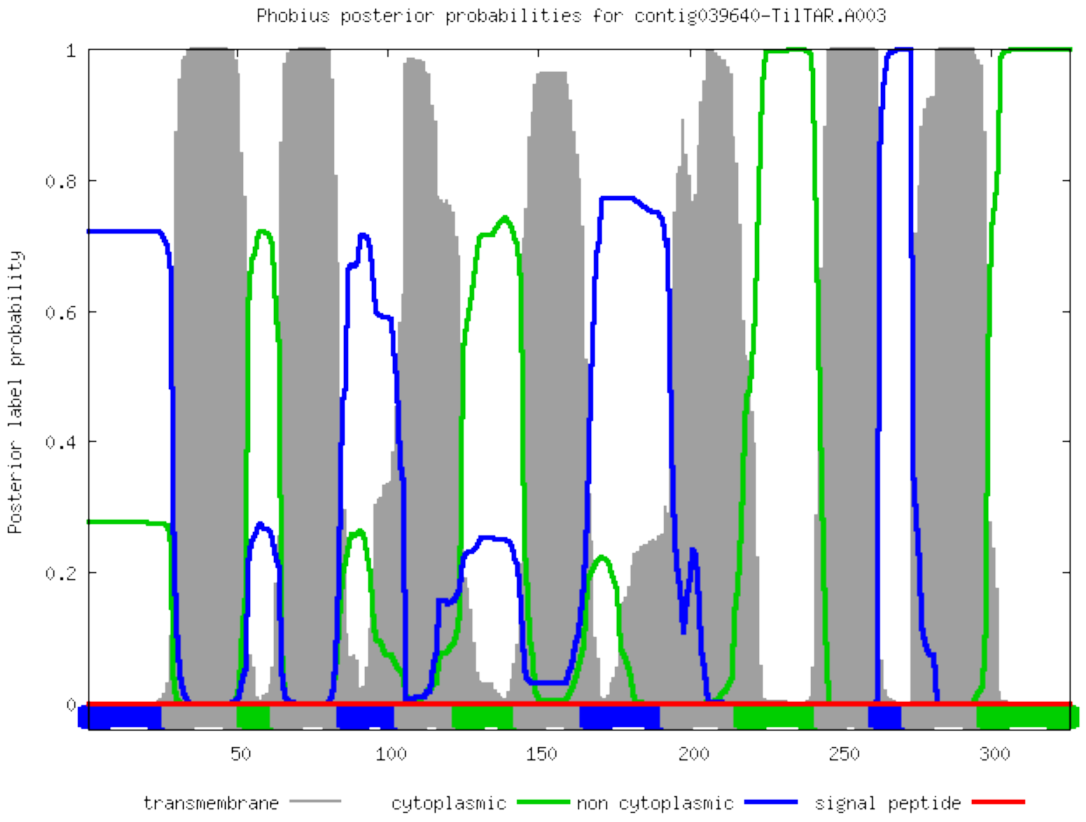

The probability data used in the plot is found here, and the gnuplot script is here.

Prediction of contig039642-TilTAR.A004\

```
ID    contig039642-TilTAR.A004\
FT    TOPO_DOM      1     28       NON CYTOPLASMIC.
FT    TRANSMEM     29     53
FT    TOPO_DOM     54     64       CYTOPLASMIC.
FT    TRANSMEM     65     86
FT    TOPO_DOM     87    105       NON CYTOPLASMIC.
FT    TRANSMEM    106    124
FT    TOPO_DOM    125    144       CYTOPLASMIC.
FT    TRANSMEM    145    166
FT    TOPO_DOM    167    193       NON CYTOPLASMIC.
FT    TRANSMEM    194    217
FT    TOPO_DOM    218    244       CYTOPLASMIC.
FT    TRANSMEM    245    262
FT    TOPO_DOM    263    273       NON CYTOPLASMIC.
FT    TRANSMEM    274    298
FT    TOPO_DOM    299    326       CYTOPLASMIC.
//
```

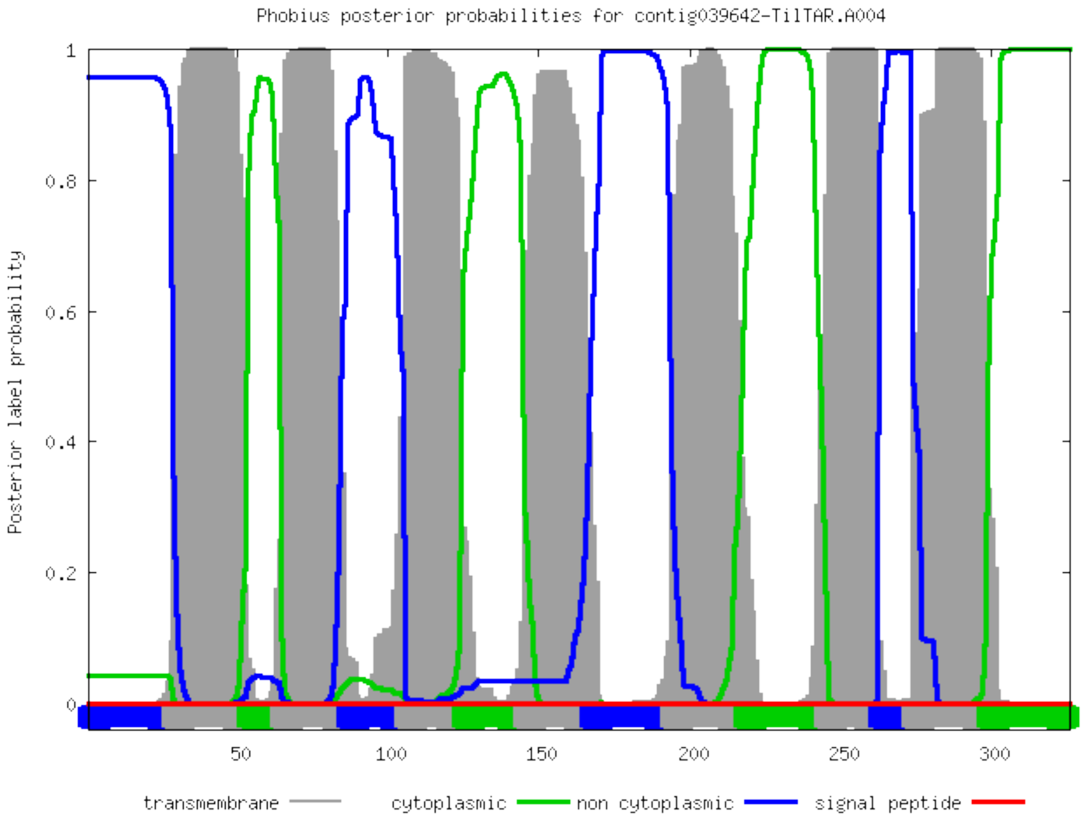

The probability data used in the plot is found here, and the gnuplot script is here.

Prediction of contig023443-TilTAR.A005\

```
ID   contig023443-TilTAR.A005\
FT   TOPO_DOM      1     30       NON CYTOPLASMIC.
FT   TRANSMEM     31     55
FT   TOPO_DOM     56     66       CYTOPLASMIC.
FT   TRANSMEM     67     84
FT   TOPO_DOM     85    103       NON CYTOPLASMIC.
FT   TRANSMEM    104    126
FT   TOPO_DOM    127    146       CYTOPLASMIC.
FT   TRANSMEM    147    166
FT   TOPO_DOM    167    195       NON CYTOPLASMIC.
FT   TRANSMEM    196    219
FT   TOPO_DOM    220    244       CYTOPLASMIC.
FT   TRANSMEM    245    265
FT   TOPO_DOM    266    284       NON CYTOPLASMIC.
FT   TRANSMEM    285    305
FT   TOPO_DOM    306    329       CYTOPLASMIC.
//
```

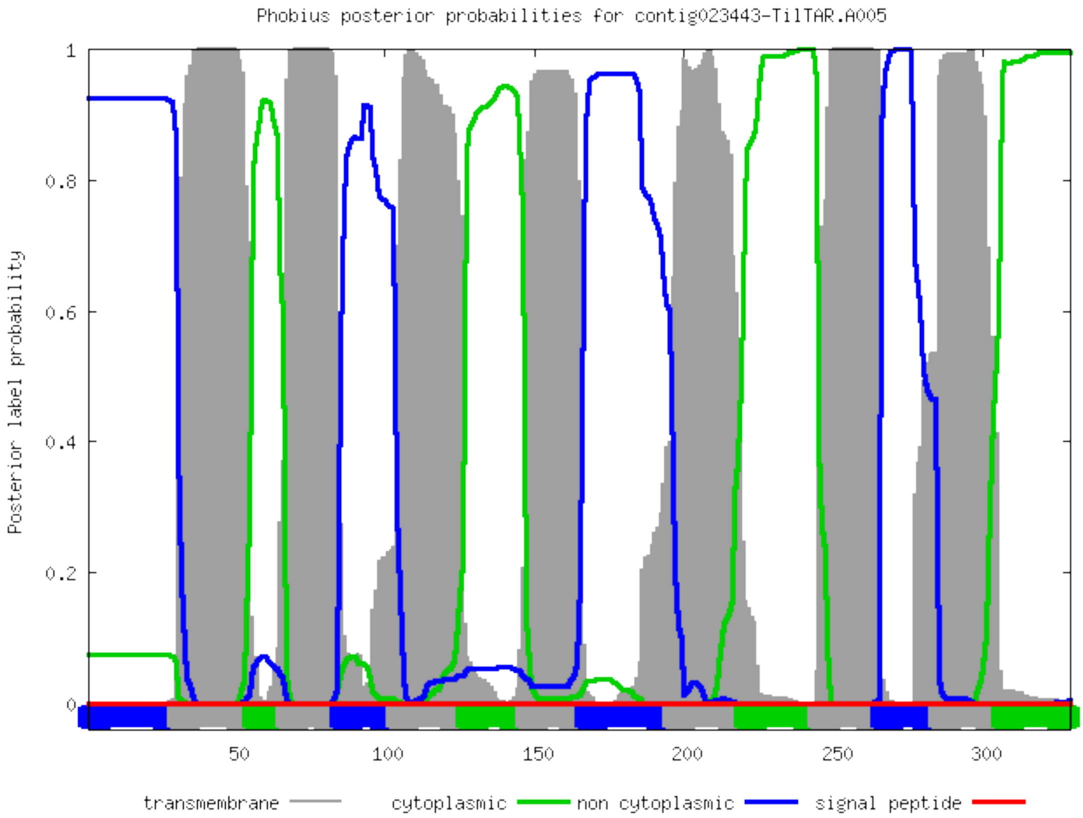

The probability data used in the plot is found here, and the gnuplot script is here.

Prediction of contig007512-TilTARs.A024\

```
ID   contig007512-TilTARs.A024\
FT   TOPO_DOM      1     27       NON CYTOPLASMIC.
FT   TRANSMEM     28     52
FT   TOPO_DOM     53     63       CYTOPLASMIC.
FT   TRANSMEM     64     94
FT   TOPO_DOM     95     99       NON CYTOPLASMIC.
FT   TRANSMEM    100    121
FT   TOPO_DOM    122    141       CYTOPLASMIC.
FT   TRANSMEM    142    161
FT   TOPO_DOM    162    180       NON CYTOPLASMIC.
FT   TRANSMEM    181    207
FT   TOPO_DOM    208    246       CYTOPLASMIC.
FT   TRANSMEM    247    267
FT   TOPO_DOM    268    278       NON CYTOPLASMIC.
FT   TRANSMEM    279    299
FT   TOPO_DOM    300    327       CYTOPLASMIC.
//
```

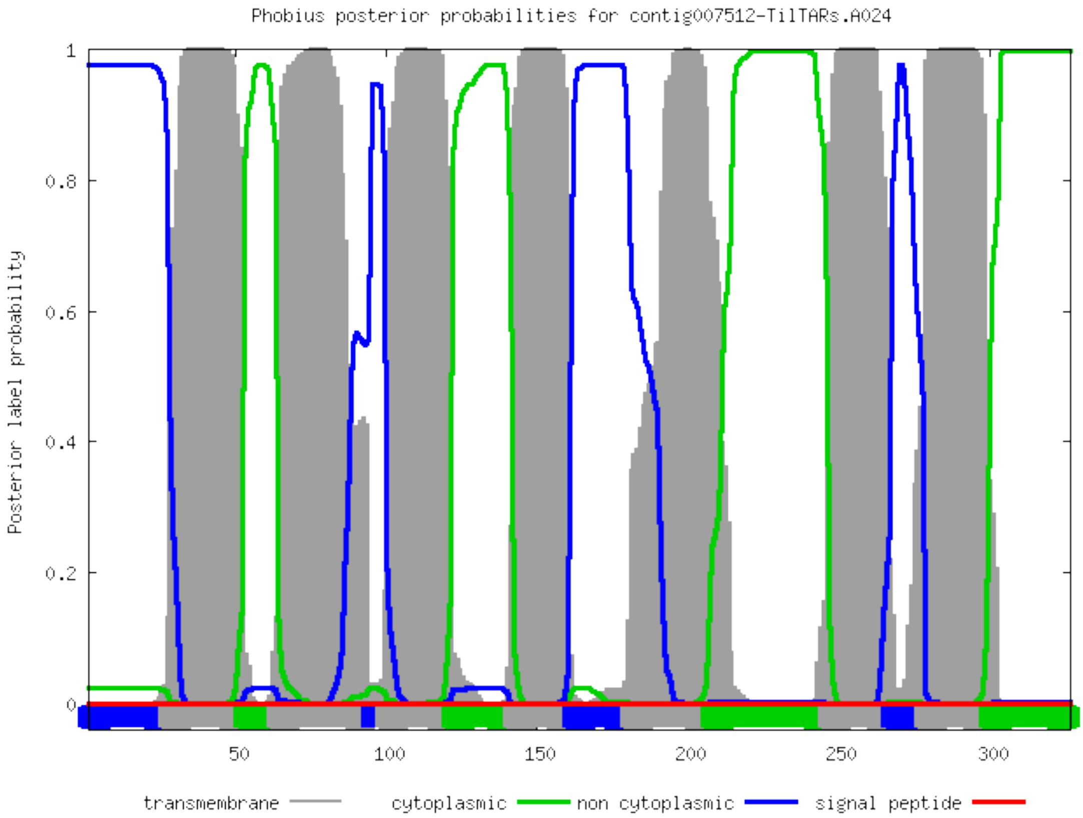

The probability data used in the plot is found here, and the gnuplot script is here.

Prediction of contig007520-TilTARs.A025\

```
ID   contig007520-TilTARs.A025\
FT   TOPO_DOM      1     29       NON CYTOPLASMIC.
FT   TRANSMEM     30     54
FT   TOPO_DOM     55     65       CYTOPLASMIC.
FT   TRANSMEM     66     88
FT   TOPO_DOM     89    107       NON CYTOPLASMIC.
FT   TRANSMEM    108    134
FT   TOPO_DOM    135    145       CYTOPLASMIC.
FT   TRANSMEM    146    164
FT   TOPO_DOM    165    192       NON CYTOPLASMIC.
FT   TRANSMEM    193    215
FT   TOPO_DOM    216    249       CYTOPLASMIC.
FT   TRANSMEM    250    270
FT   TOPO_DOM    271    281       NON CYTOPLASMIC.
FT   TRANSMEM    282    302
FT   TOPO_DOM    303    330       CYTOPLASMIC.
//
```

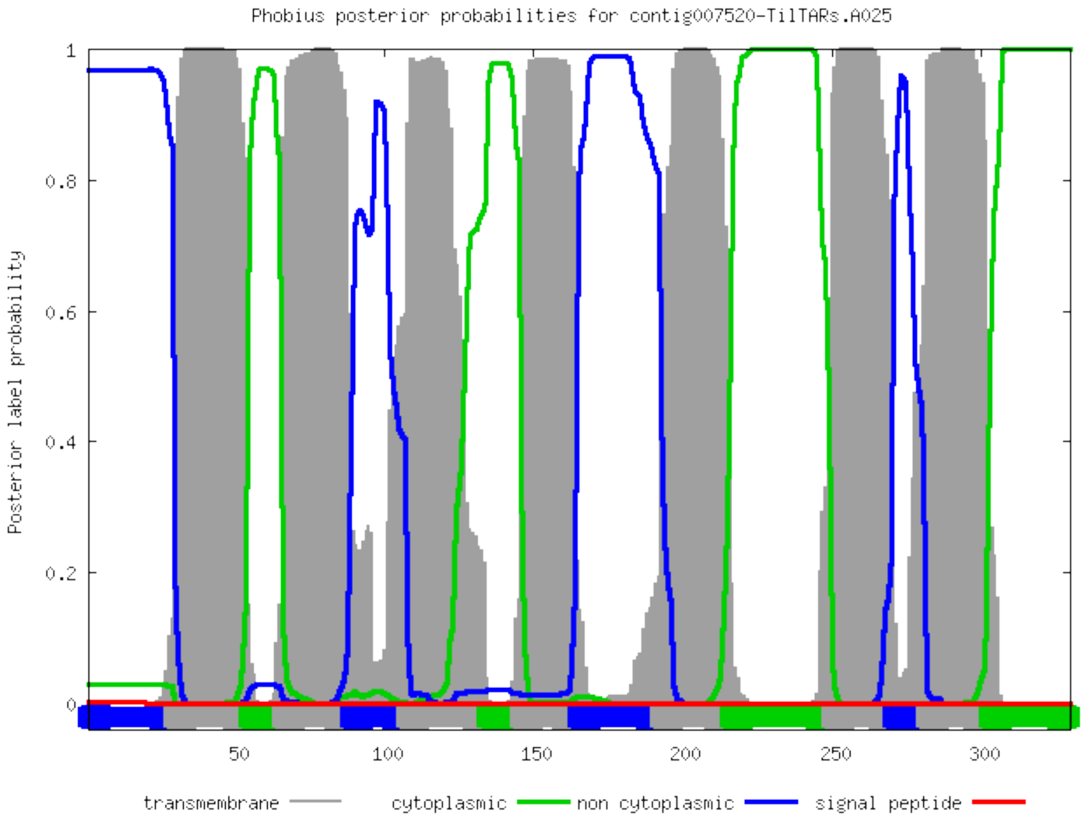

The probability data used in the plot is found here, and the gnuplot script is here.

Prediction of contig007524-TilTARs.A026\

```
ID   contig007524-TilTARs.A026\
FT   TOPO_DOM      1     29      NON CYTOPLASMIC.
FT   TRANSMEM     30     54
FT   TOPO_DOM     55     65      CYTOPLASMIC.
FT   TRANSMEM     66     88
FT   TOPO_DOM     89    107      NON CYTOPLASMIC.
FT   TRANSMEM    108    134
FT   TOPO_DOM    135    145      CYTOPLASMIC.
FT   TRANSMEM    146    164
FT   TOPO_DOM    165    192      NON CYTOPLASMIC.
FT   TRANSMEM    193    215
FT   TOPO_DOM    216    249      CYTOPLASMIC.
FT   TRANSMEM    250    270
FT   TOPO_DOM    271    281      NON CYTOPLASMIC.
FT   TRANSMEM    282    302
FT   TOPO_DOM    303    330      CYTOPLASMIC.
//
```

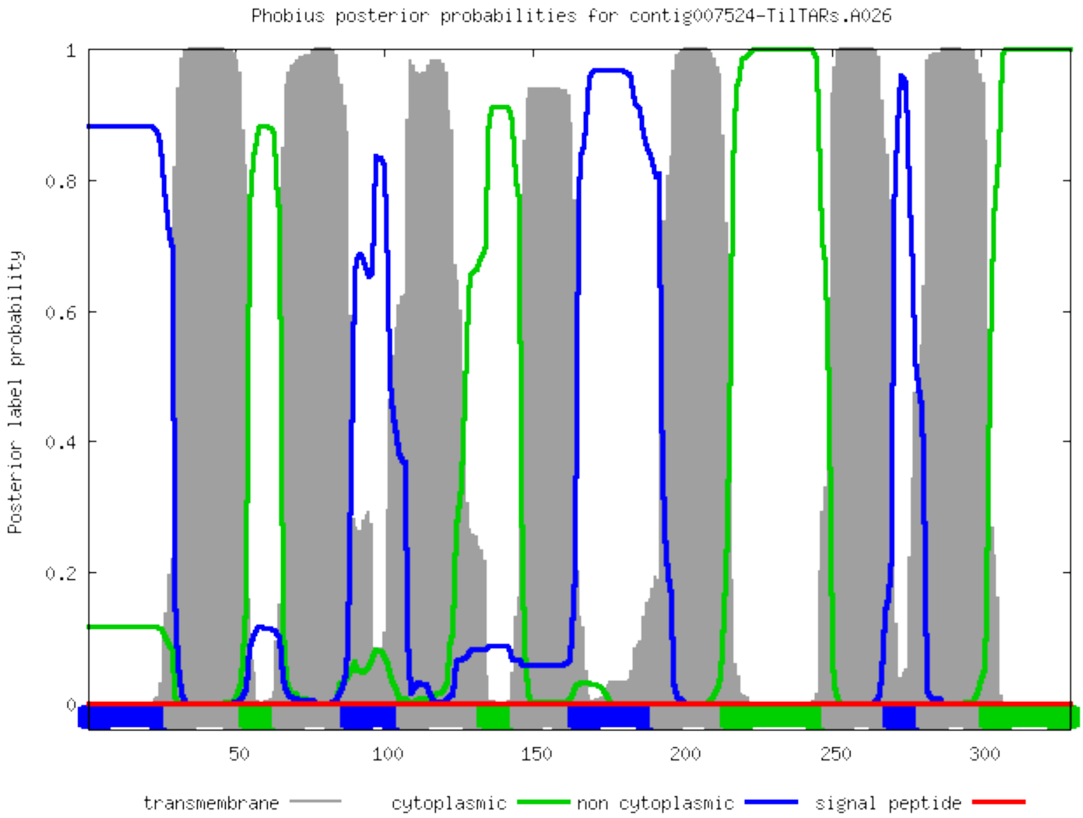

The probability data used in the plot is found here, and the gnuplot script is here.

Prediction of contig022324-TilTARs.A027\

```
ID   contig022324-TilTARs.A027\
FT   TOPO_DOM      1     31       NON CYTOPLASMIC.
FT   TRANSMEM     32     56
FT   TOPO_DOM     57     67       CYTOPLASMIC.
FT   TRANSMEM     68     85
FT   TOPO_DOM     86    104       NON CYTOPLASMIC.
FT   TRANSMEM    105    126
FT   TOPO_DOM    127    146       CYTOPLASMIC.
FT   TRANSMEM    147    165
FT   TOPO_DOM    166    199       NON CYTOPLASMIC.
FT   TRANSMEM    200    219
FT   TOPO_DOM    220    251       CYTOPLASMIC.
FT   TRANSMEM    252    272
FT   TOPO_DOM    273    283       NON CYTOPLASMIC.
FT   TRANSMEM    284    306
FT   TOPO_DOM    307    330       CYTOPLASMIC.
//
```

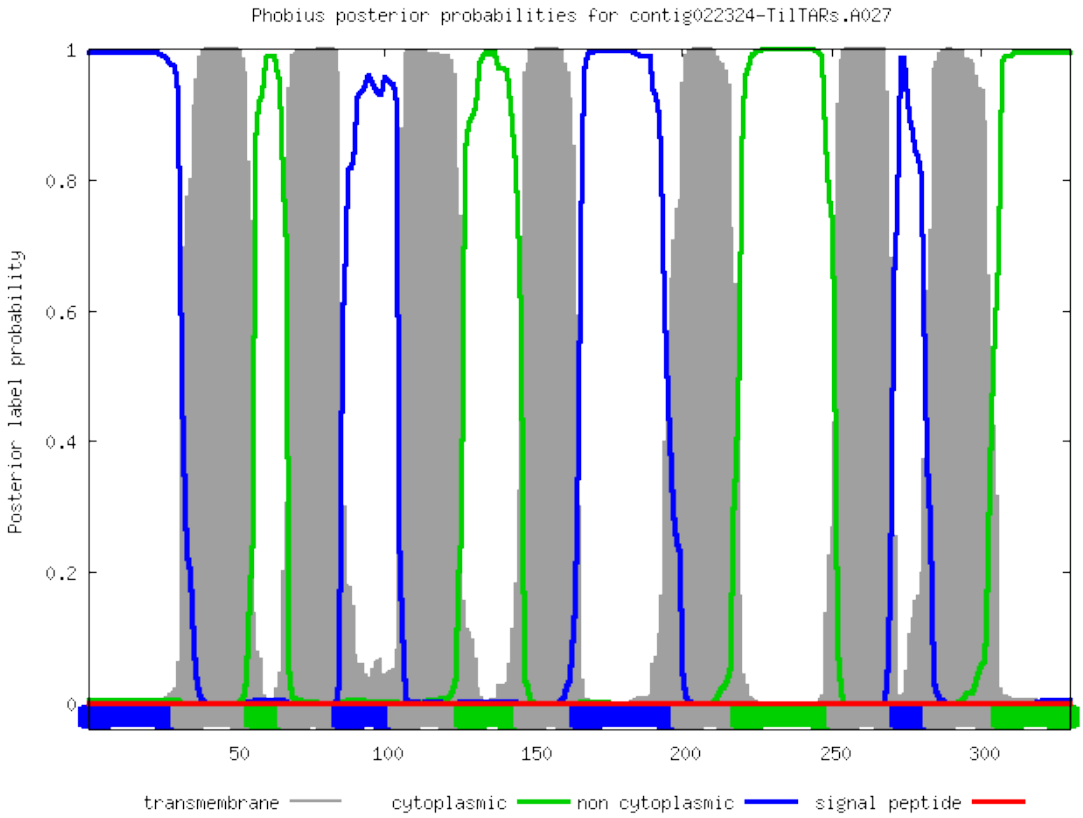

The probability data used in the plot is found here, and the gnuplot script is here.

Prediction of contig022330-TilTARs.A028\

```
ID   contig022330-TilTARs.A028\
FT   TOPO_DOM      1     29       NON CYTOPLASMIC.
FT   TRANSMEM     30     54
FT   TOPO_DOM     55     65       CYTOPLASMIC.
FT   TRANSMEM     66     83
FT   TOPO_DOM     84    102       NON CYTOPLASMIC.
FT   TRANSMEM    103    124
FT   TOPO_DOM    125    144       CYTOPLASMIC.
FT   TRANSMEM    145    162
FT   TOPO_DOM    163    193       NON CYTOPLASMIC.
FT   TRANSMEM    194    217
FT   TOPO_DOM    218    249       CYTOPLASMIC.
FT   TRANSMEM    250    270
FT   TOPO_DOM    271    281       NON CYTOPLASMIC.
FT   TRANSMEM    282    305
FT   TOPO_DOM    306    329       CYTOPLASMIC.
//
```

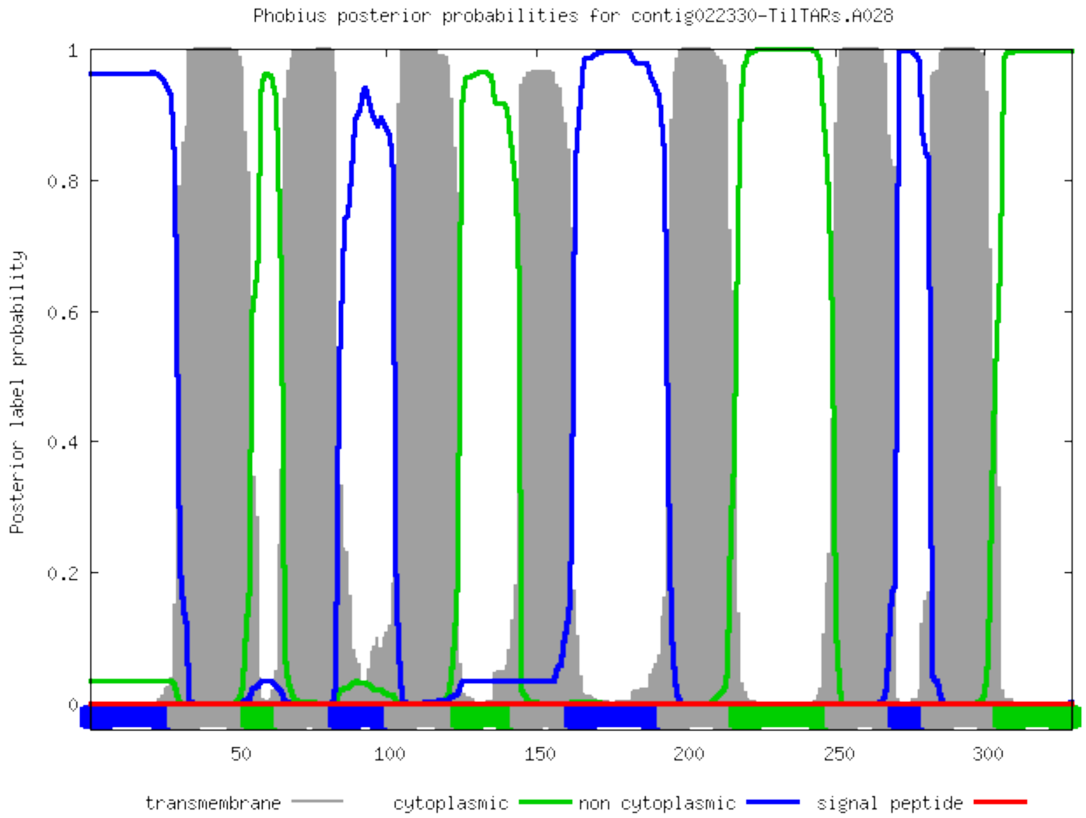

The probability data used in the plot is found here, and the gnuplot script is here.

Prediction of contig022334-TilTARs.A029\

```
ID   contig022334-TilTARs.A029\
FT   TOPO_DOM      1     29       NON CYTOPLASMIC.
FT   TRANSMEM     30     54
FT   TOPO_DOM     55     65       CYTOPLASMIC.
FT   TRANSMEM     66     83
FT   TOPO_DOM     84    102       NON CYTOPLASMIC.
FT   TRANSMEM    103    124
FT   TOPO_DOM    125    144       CYTOPLASMIC.
FT   TRANSMEM    145    162
FT   TOPO_DOM    163    193       NON CYTOPLASMIC.
FT   TRANSMEM    194    217
FT   TOPO_DOM    218    249       CYTOPLASMIC.
FT   TRANSMEM    250    270
FT   TOPO_DOM    271    281       NON CYTOPLASMIC.
FT   TRANSMEM    282    305
FT   TOPO_DOM    306    329       CYTOPLASMIC.
//
```

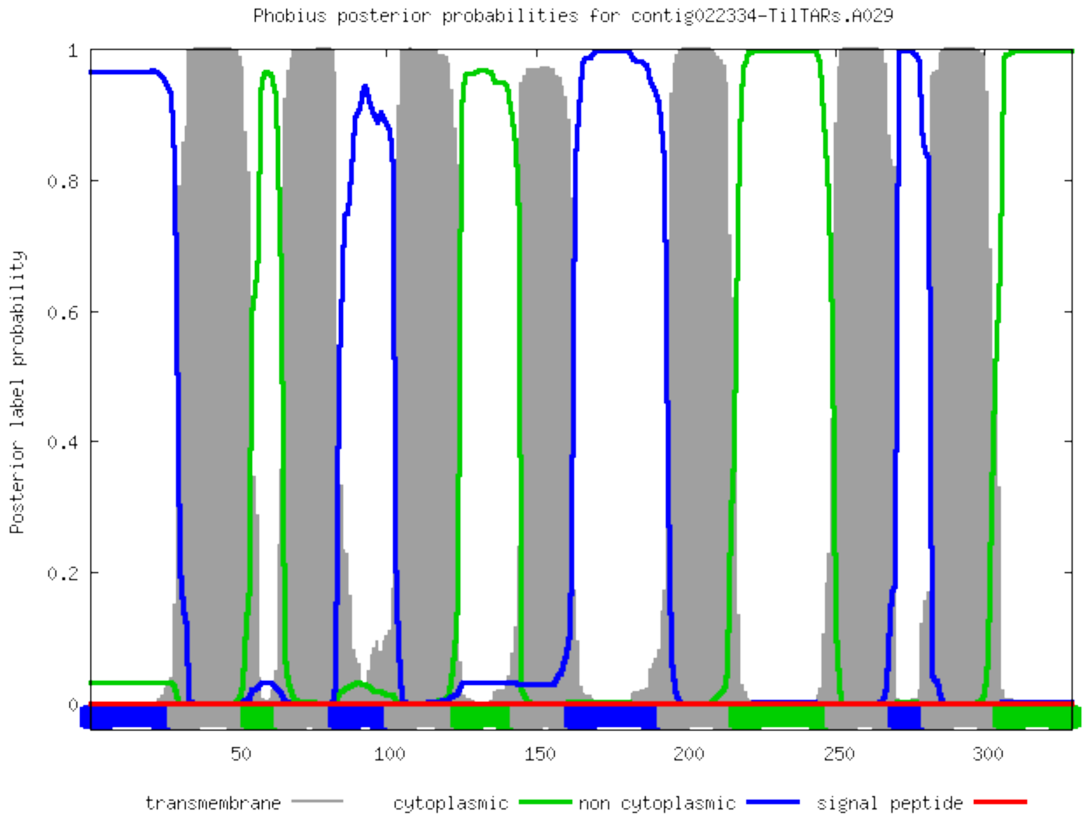

The probability data used in the plot is found here, and the gnuplot script is here.

Prediction of contig022334-TilTARs.A030\

```
ID    contig022334-TilTARs.A030\
FT    TOPO_DOM       1      30       NON CYTOPLASMIC.
FT    TRANSMEM      31      55
FT    TOPO_DOM      56      66       CYTOPLASMIC.
FT    TRANSMEM      67      84
FT    TOPO_DOM      85     103       NON CYTOPLASMIC.
FT    TRANSMEM     104     125
FT    TOPO_DOM     126     145       CYTOPLASMIC.
FT    TRANSMEM     146     176
FT    TOPO_DOM     177     181       NON CYTOPLASMIC.
FT    TRANSMEM     182     201
FT    TOPO_DOM     202     233       CYTOPLASMIC.
FT    TRANSMEM     234     254
FT    TOPO_DOM     255     259       NON CYTOPLASMIC.
FT    TRANSMEM     260     283
FT    TOPO_DOM     284     311       CYTOPLASMIC.
//
```

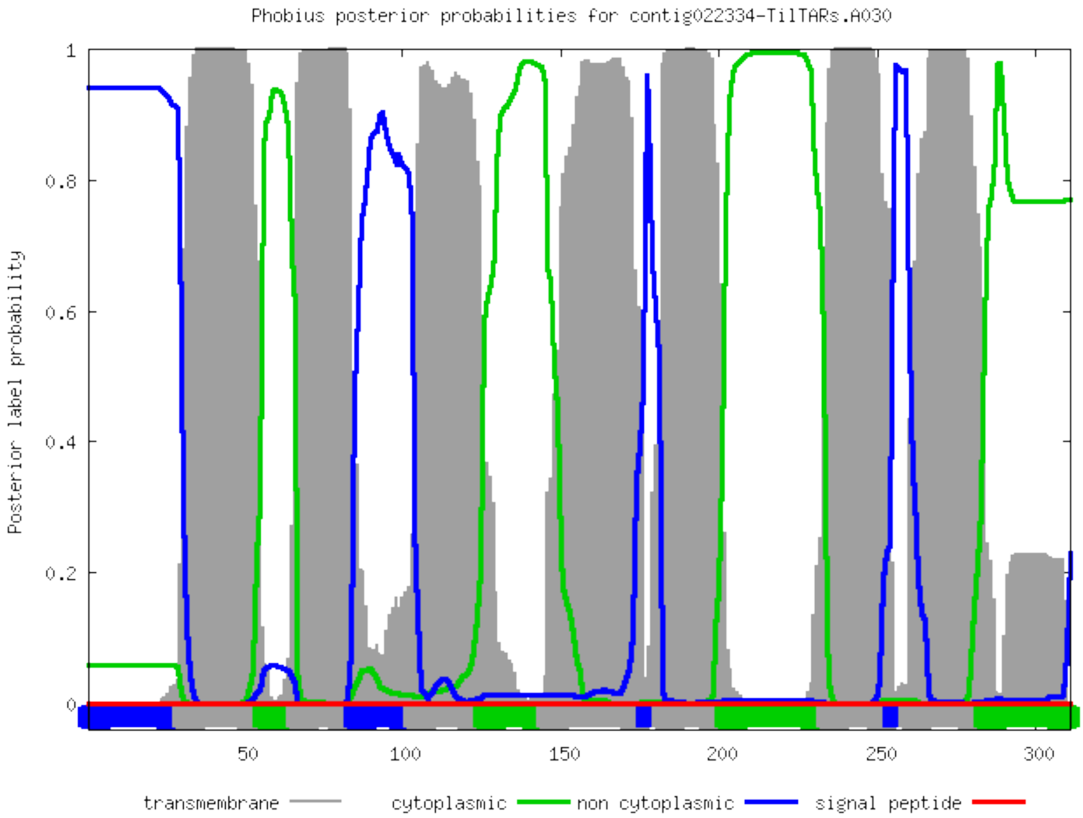

The probability data used in the plot is found here, and the gnuplot script is here.

Prediction of contig022337-TilTARs.A031\

```
ID   contig022337-TilTARs.A031\
FT   TOPO_DOM      1     30       NON CYTOPLASMIC.
FT   TRANSMEM     31     55
FT   TOPO_DOM     56     66       CYTOPLASMIC.
FT   TRANSMEM     67     84
FT   TOPO_DOM     85    103       NON CYTOPLASMIC.
FT   TRANSMEM    104    125
FT   TOPO_DOM    126    145       CYTOPLASMIC.
FT   TRANSMEM    146    165
FT   TOPO_DOM    166    194       NON CYTOPLASMIC.
FT   TRANSMEM    195    218
FT   TOPO_DOM    219    250       CYTOPLASMIC.
FT   TRANSMEM    251    271
FT   TOPO_DOM    272    282       NON CYTOPLASMIC.
FT   TRANSMEM    283    303
FT   TOPO_DOM    304    331       CYTOPLASMIC.
//
```

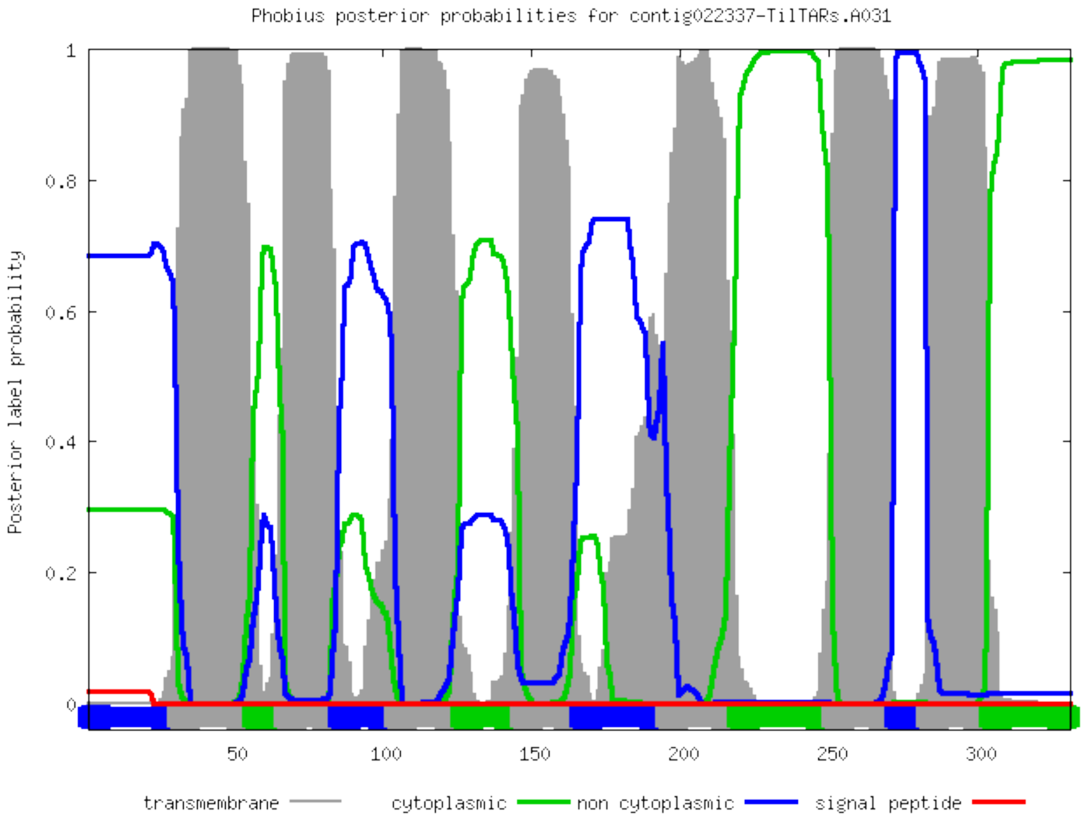

The probability data used in the plot is found here, and the gnuplot script is here.

Prediction of contig022341-TilTARs.A032\

```
ID   contig022341-TilTARs.A032\
FT   TOPO_DOM      1     29       NON CYTOPLASMIC.
FT   TRANSMEM     30     54
FT   TOPO_DOM     55     65       CYTOPLASMIC.
FT   TRANSMEM     66     85
FT   TOPO_DOM     86    104       NON CYTOPLASMIC.
FT   TRANSMEM    105    124
FT   TOPO_DOM    125    144       CYTOPLASMIC.
FT   TRANSMEM    145    164
FT   TOPO_DOM    165    194       NON CYTOPLASMIC.
FT   TRANSMEM    195    217
FT   TOPO_DOM    218    249       CYTOPLASMIC.
FT   TRANSMEM    250    270
FT   TOPO_DOM    271    281       NON CYTOPLASMIC.
FT   TRANSMEM    282    305
FT   TOPO_DOM    306    329       CYTOPLASMIC.
//
```

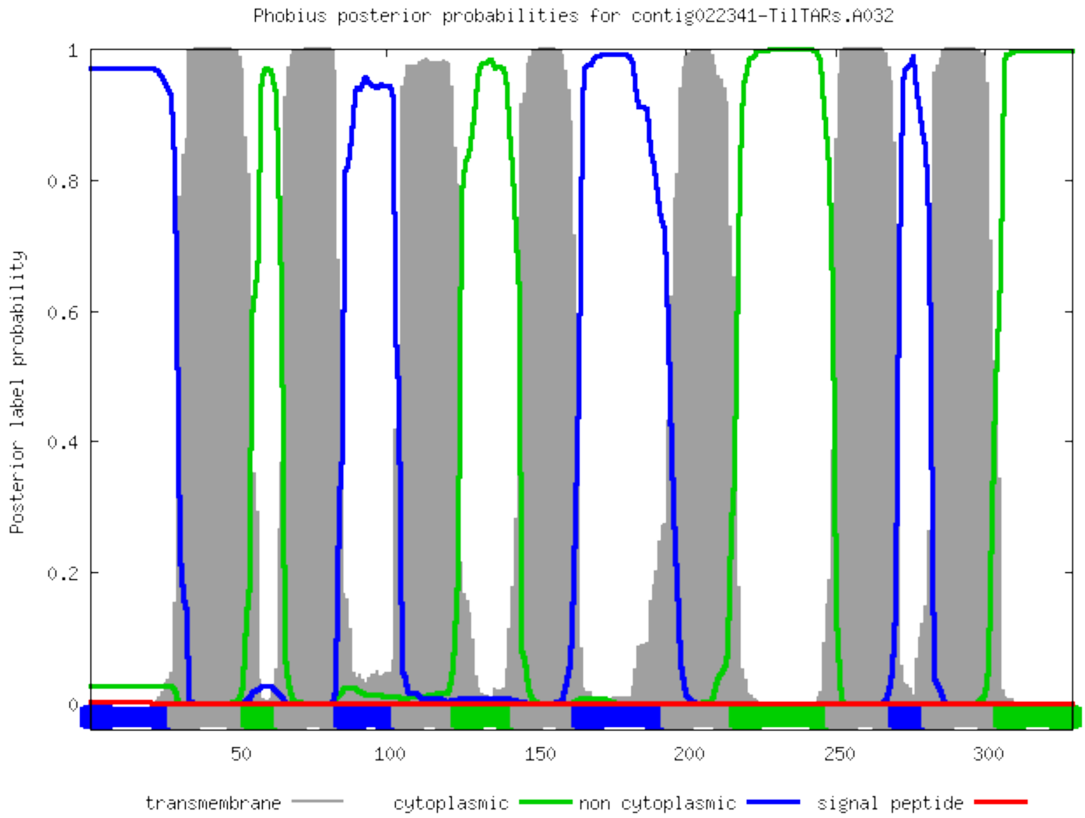

The probability data used in the plot is found here, and the gnuplot script is here.

Prediction of contig022343-TilTARs.A033\

```
ID    contig022343-TilTARs.A033\
FT    TOPO_DOM      1     29       NON CYTOPLASMIC.
FT    TRANSMEM     30     54
FT    TOPO_DOM     55     65       CYTOPLASMIC.
FT    TRANSMEM     66     83
FT    TOPO_DOM     84    102       NON CYTOPLASMIC.
FT    TRANSMEM    103    124
FT    TOPO_DOM    125    144       CYTOPLASMIC.
FT    TRANSMEM    145    164
FT    TOPO_DOM    165    183       NON CYTOPLASMIC.
FT    TRANSMEM    184    209
FT    TOPO_DOM    210    249       CYTOPLASMIC.
FT    TRANSMEM    250    270
FT    TOPO_DOM    271    281       NON CYTOPLASMIC.
FT    TRANSMEM    282    305
FT    TOPO_DOM    306    329       CYTOPLASMIC.
//
```

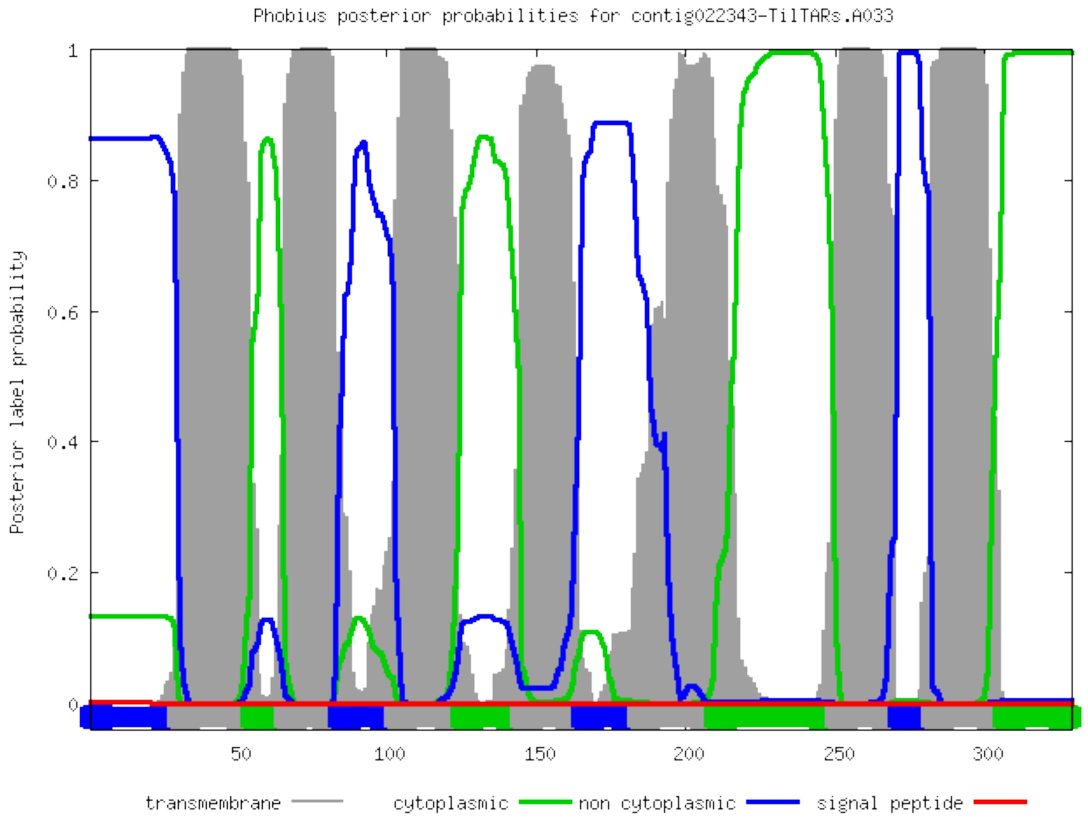

The probability data used in the plot is found here, and the gnuplot script is here.

Prediction of contig022345-TilTARs.A034\

```
ID    contig022345-TilTARs.A034\
FT    TOPO_DOM       1      29       NON CYTOPLASMIC.
FT    TRANSMEM      30      54
FT    TOPO_DOM      55      65       CYTOPLASMIC.
FT    TRANSMEM      66      83
FT    TOPO_DOM      84     102       NON CYTOPLASMIC.
FT    TRANSMEM     103     124
FT    TOPO_DOM     125     144       CYTOPLASMIC.
FT    TRANSMEM     145     163
FT    TOPO_DOM     164     193       NON CYTOPLASMIC.
FT    TRANSMEM     194     214
FT    TOPO_DOM     215     249       CYTOPLASMIC.
FT    TRANSMEM     250     270
FT    TOPO_DOM     271     281       NON CYTOPLASMIC.
FT    TRANSMEM     282     301
FT    TOPO_DOM     302     329       CYTOPLASMIC.
//
```

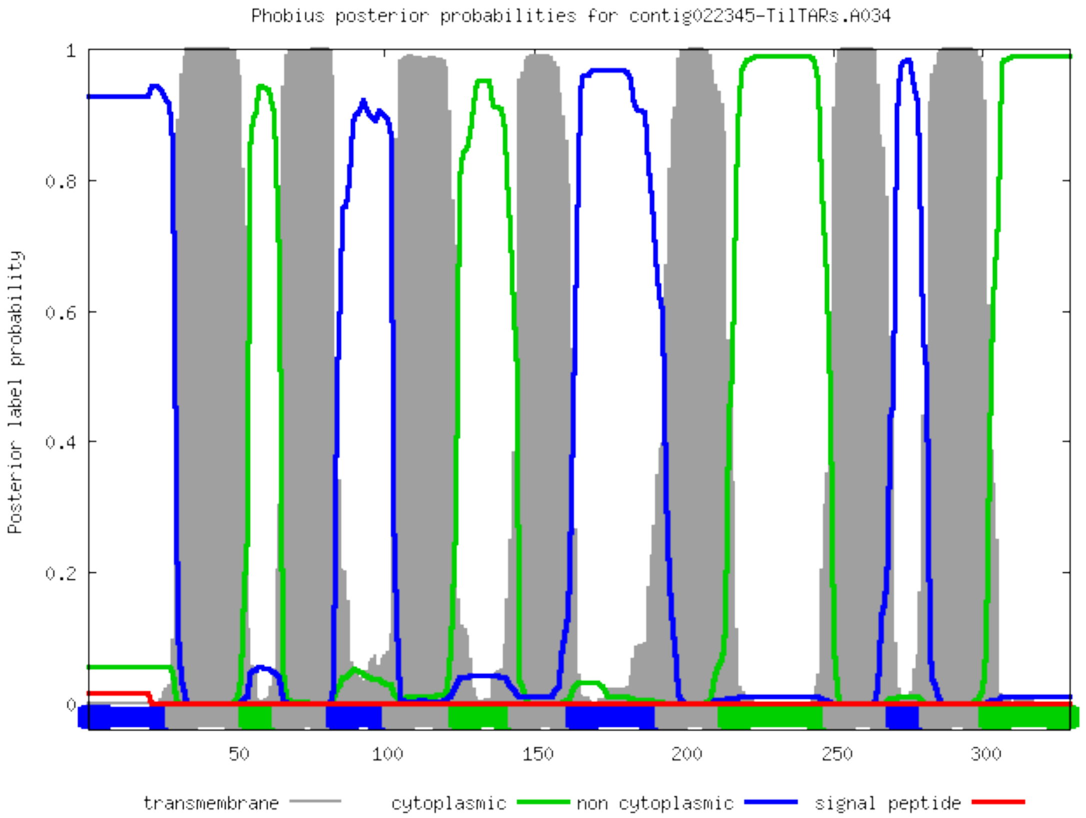

The probability data used in the plot is found here, and the gnuplot script is here.

Prediction of contig022349-TilTARs.A035\

```
ID   contig022349-TilTARs.A035\
FT   TOPO_DOM      1     29       NON CYTOPLASMIC.
FT   TRANSMEM     30     54
FT   TOPO_DOM     55     65       CYTOPLASMIC.
FT   TRANSMEM     66     83
FT   TOPO_DOM     84    102       NON CYTOPLASMIC.
FT   TRANSMEM    103    124
FT   TOPO_DOM    125    144       CYTOPLASMIC.
FT   TRANSMEM    145    164
FT   TOPO_DOM    165    193       NON CYTOPLASMIC.
FT   TRANSMEM    194    217
FT   TOPO_DOM    218    249       CYTOPLASMIC.
FT   TRANSMEM    250    270
FT   TOPO_DOM    271    281       NON CYTOPLASMIC.
FT   TRANSMEM    282    305
FT   TOPO_DOM    306    329       CYTOPLASMIC.
//
```

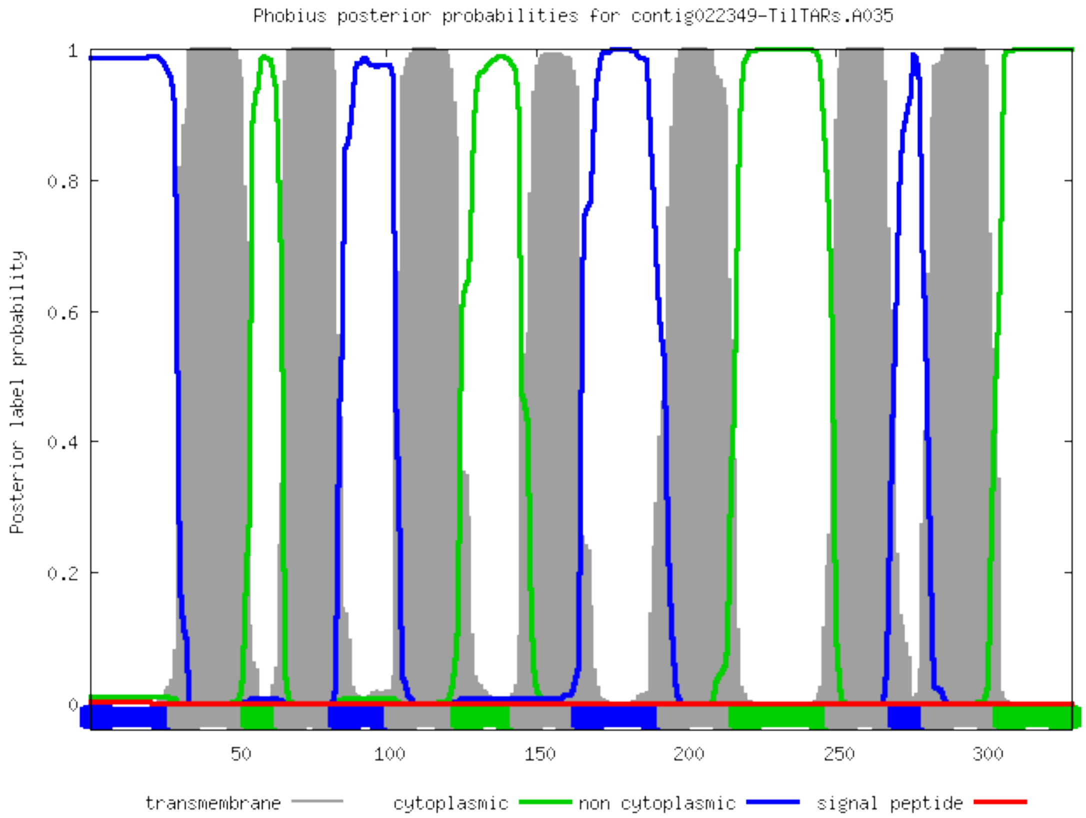

The probability data used in the plot is found here, and the gnuplot script is here.

Prediction of contig022353-TilTARs.A036\

```
ID    contig022353-TilTARs.A036\
FT    TOPO_DOM      1     29      NON CYTOPLASMIC.
FT    TRANSMEM     30     54
FT    TOPO_DOM     55     65      CYTOPLASMIC.
FT    TRANSMEM     66     83
FT    TOPO_DOM     84    102      NON CYTOPLASMIC.
FT    TRANSMEM    103    124
FT    TOPO_DOM    125    144      CYTOPLASMIC.
FT    TRANSMEM    145    163
FT    TOPO_DOM    164    182      NON CYTOPLASMIC.
FT    TRANSMEM    183    214
FT    TOPO_DOM    215    249      CYTOPLASMIC.
FT    TRANSMEM    250    270
FT    TOPO_DOM    271    281      NON CYTOPLASMIC.
FT    TRANSMEM    282    305
FT    TOPO_DOM    306    329      CYTOPLASMIC.
//
```

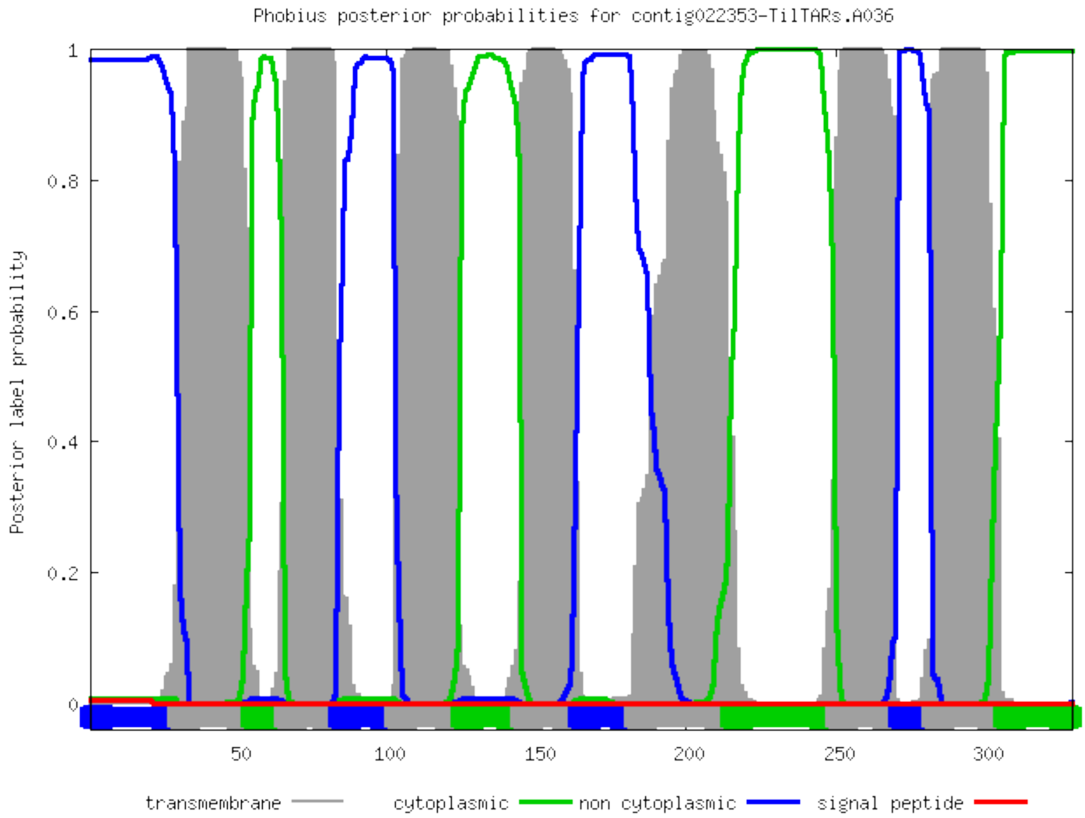

The probability data used in the plot is found here, and the gnuplot script is here.

Prediction of contig022354-TilTARs.A037\

```
ID    contig022354-TilTARs.A037\
FT    TOPO_DOM       1      29       NON CYTOPLASMIC.
FT    TRANSMEM      30      54
FT    TOPO_DOM      55      65       CYTOPLASMIC.
FT    TRANSMEM      66      83
FT    TOPO_DOM      84     102       NON CYTOPLASMIC.
FT    TRANSMEM     103     124
FT    TOPO_DOM     125     144       CYTOPLASMIC.
FT    TRANSMEM     145     164
FT    TOPO_DOM     165     193       NON CYTOPLASMIC.
FT    TRANSMEM     194     217
FT    TOPO_DOM     218     249       CYTOPLASMIC.
FT    TRANSMEM     250     270
FT    TOPO_DOM     271     275       NON CYTOPLASMIC.
FT    TRANSMEM     276     299
FT    TOPO_DOM     300     327       CYTOPLASMIC.
//
```

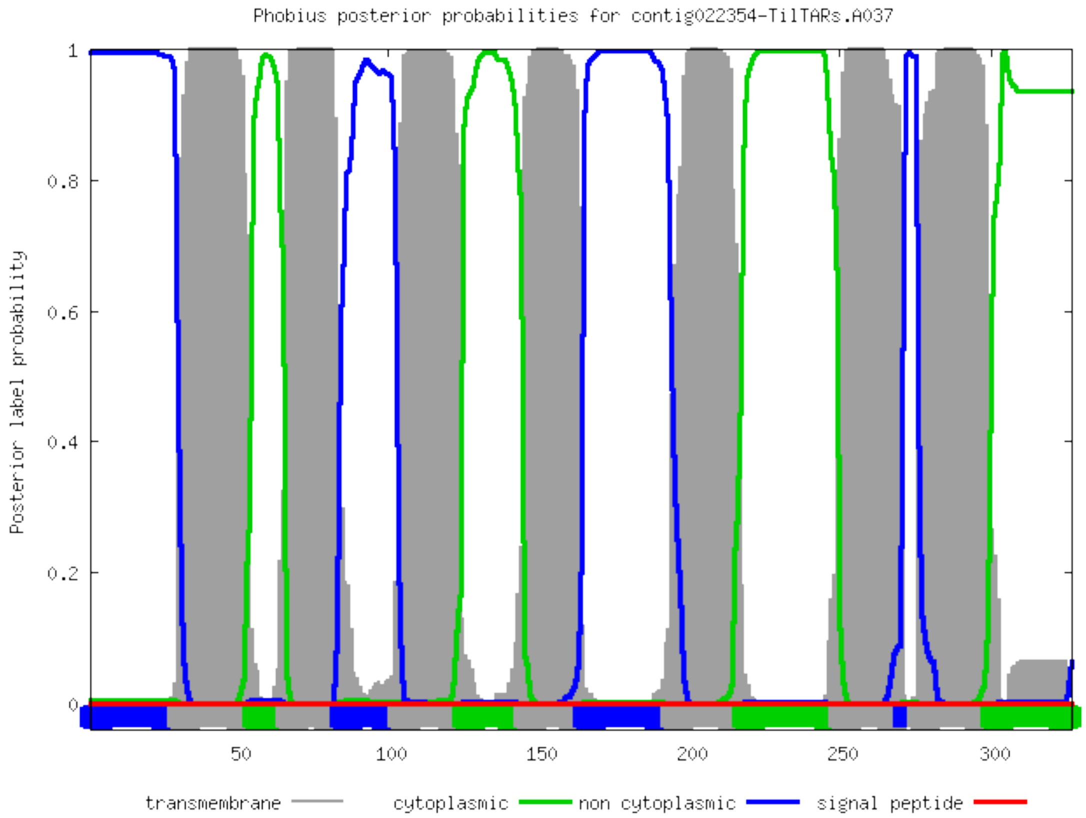

The probability data used in the plot is found here, and the gnuplot script is here.

Prediction of contig022354-TilTARs.A038\

```
ID    contig022354-TilTARs.A038\
FT    TOPO_DOM      1     29      NON CYTOPLASMIC.
FT    TRANSMEM     30     54
FT    TOPO_DOM     55     65      CYTOPLASMIC.
FT    TRANSMEM     66     83
FT    TOPO_DOM     84    102      NON CYTOPLASMIC.
FT    TRANSMEM    103    124
FT    TOPO_DOM    125    144      CYTOPLASMIC.
FT    TRANSMEM    145    163
FT    TOPO_DOM    164    193      NON CYTOPLASMIC.
FT    TRANSMEM    194    217
FT    TOPO_DOM    218    249      CYTOPLASMIC.
FT    TRANSMEM    250    270
FT    TOPO_DOM    271    281      NON CYTOPLASMIC.
FT    TRANSMEM    282    305
FT    TOPO_DOM    306    329      CYTOPLASMIC.
//
```

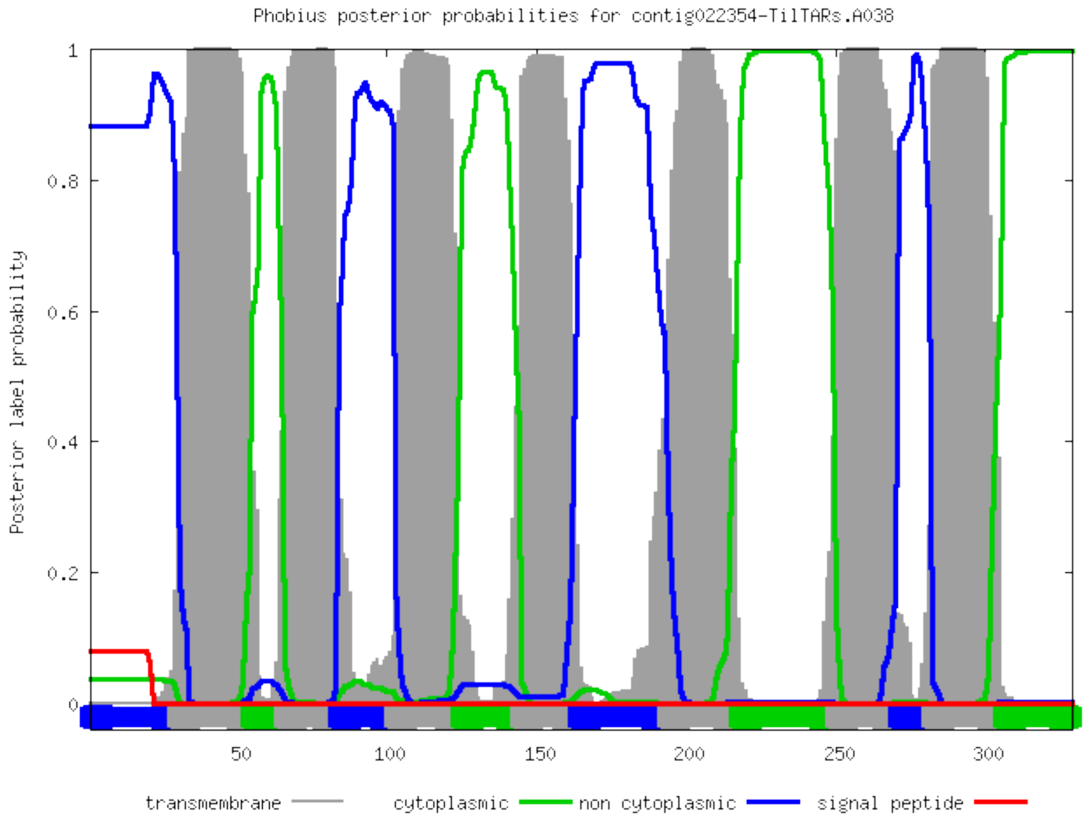

The probability data used in the plot is found here, and the gnuplot script is here.

Prediction of contig022355-TilTARs.A039\

```
ID   contig022355-TilTARs.A039\
FT   TOPO_DOM      1     29       NON CYTOPLASMIC.
FT   TRANSMEM     30     54
FT   TOPO_DOM     55     65       CYTOPLASMIC.
FT   TRANSMEM     66     88
FT   TOPO_DOM     89    102       NON CYTOPLASMIC.
FT   TRANSMEM    103    124
FT   TOPO_DOM    125    144       CYTOPLASMIC.
FT   TRANSMEM    145    164
FT   TOPO_DOM    165    193       NON CYTOPLASMIC.
FT   TRANSMEM    194    214
FT   TOPO_DOM    215    249       CYTOPLASMIC.
FT   TRANSMEM    250    268
FT   TOPO_DOM    269    279       NON CYTOPLASMIC.
FT   TRANSMEM    280    301
FT   TOPO_DOM    302    329       CYTOPLASMIC.
//
```

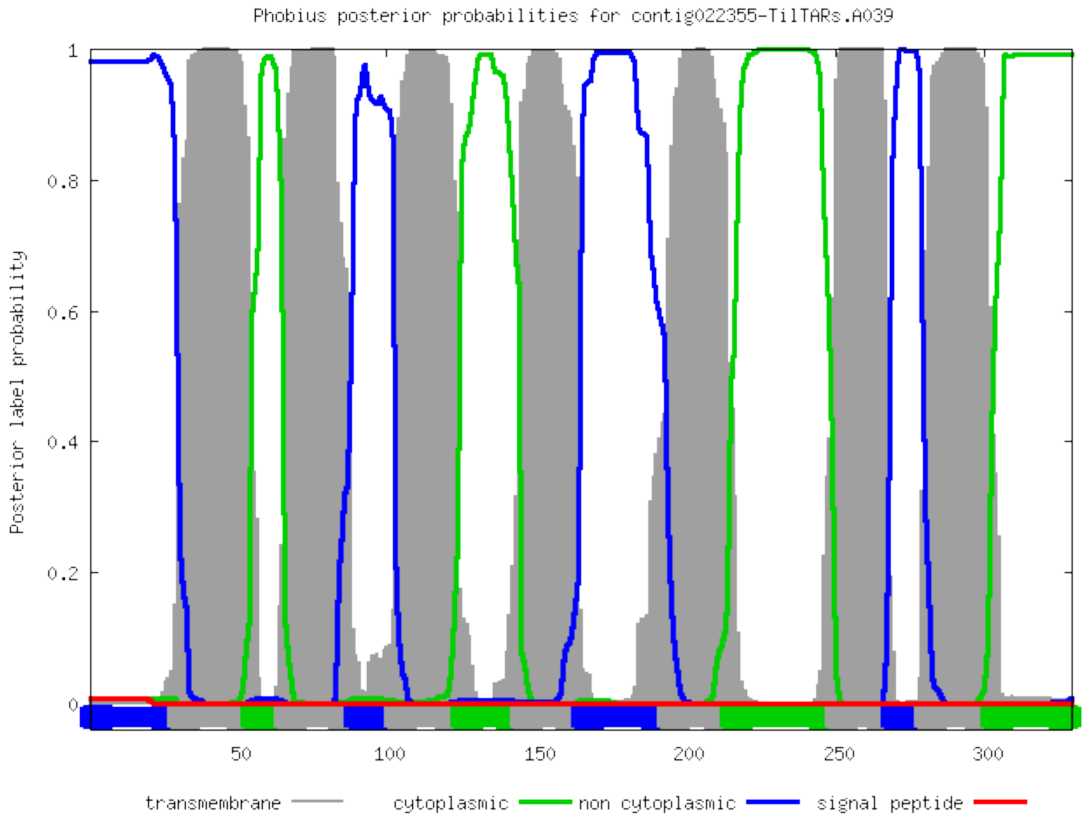

The probability data used in the plot is found here, and the gnuplot script is here.

Prediction of contig022356-TilTARs.A040\

```
ID   contig022356-TilTARs.A040\
FT   TOPO_DOM      1    29      NON CYTOPLASMIC.
FT   TRANSMEM     30    54
FT   TOPO_DOM     55    65      CYTOPLASMIC.
FT   TRANSMEM     66    83
FT   TOPO_DOM     84   102      NON CYTOPLASMIC.
FT   TRANSMEM    103   124
FT   TOPO_DOM    125   144      CYTOPLASMIC.
FT   TRANSMEM    145   164
FT   TOPO_DOM    165   187      NON CYTOPLASMIC.
FT   TRANSMEM    188   214
FT   TOPO_DOM    215   249      CYTOPLASMIC.
FT   TRANSMEM    250   270
FT   TOPO_DOM    271   281      NON CYTOPLASMIC.
FT   TRANSMEM    282   305
FT   TOPO_DOM    306   329      CYTOPLASMIC.
//
```

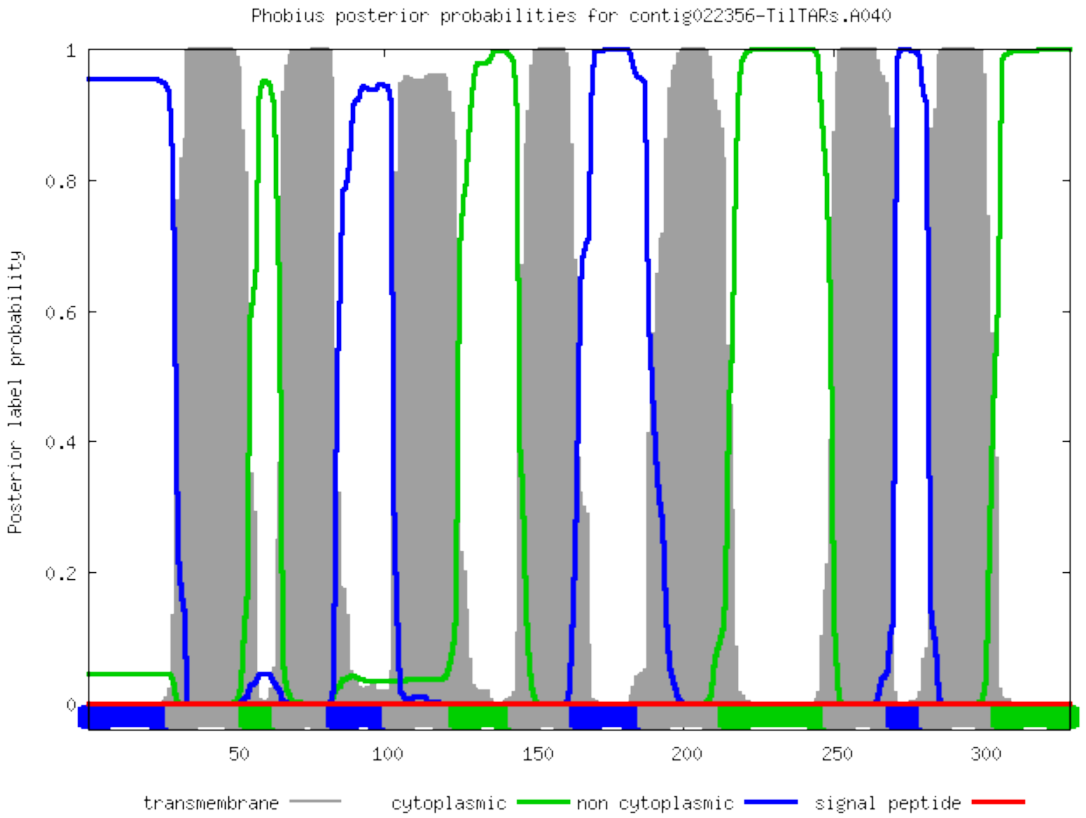

The probability data used in the plot is found here, and the gnuplot script is here.

Prediction of contig022357-TilTARs.A041\

```
ID   contig022357-TilTARs.A041\
FT   TOPO_DOM      1     29       NON CYTOPLASMIC.
FT   TRANSMEM     30     54
FT   TOPO_DOM     55     65       CYTOPLASMIC.
FT   TRANSMEM     66     83
FT   TOPO_DOM     84    102       NON CYTOPLASMIC.
FT   TRANSMEM    103    124
FT   TOPO_DOM    125    144       CYTOPLASMIC.
FT   TRANSMEM    145    162
FT   TOPO_DOM    163    181       NON CYTOPLASMIC.
FT   TRANSMEM    182    209
FT   TOPO_DOM    210    249       CYTOPLASMIC.
FT   TRANSMEM    250    270
FT   TOPO_DOM    271    281       NON CYTOPLASMIC.
FT   TRANSMEM    282    305
FT   TOPO_DOM    306    329       CYTOPLASMIC.
//
```

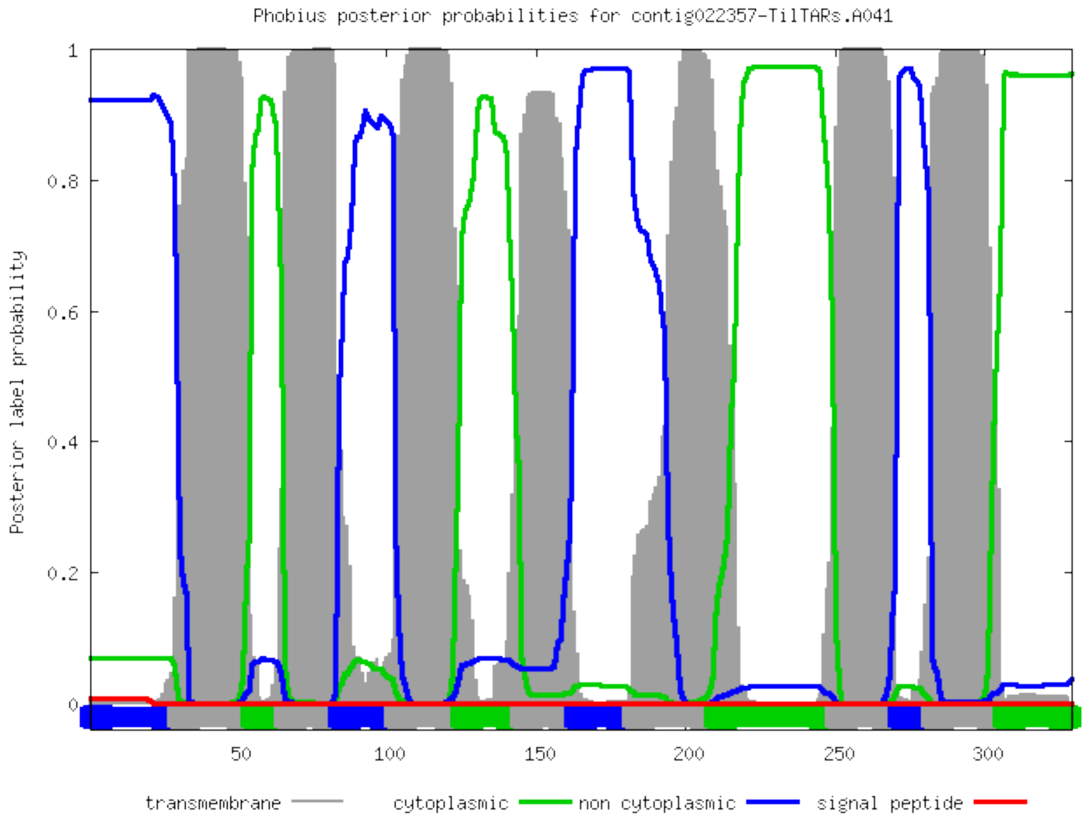

The probability data used in the plot is found here, and the gnuplot script is here.

Prediction of contig022362-TilTARs.A042\

```
ID   contig022362-TilTARs.A042\
FT   TOPO_DOM      1     29       NON CYTOPLASMIC.
FT   TRANSMEM     30     54
FT   TOPO_DOM     55     65       CYTOPLASMIC.
FT   TRANSMEM     66     83
FT   TOPO_DOM     84    102       NON CYTOPLASMIC.
FT   TRANSMEM    103    124
FT   TOPO_DOM    125    144       CYTOPLASMIC.
FT   TRANSMEM    145    164
FT   TOPO_DOM    165    192       NON CYTOPLASMIC.
FT   TRANSMEM    193    217
FT   TOPO_DOM    218    237       CYTOPLASMIC.
FT   TRANSMEM    238    262
FT   TOPO_DOM    263    273       NON CYTOPLASMIC.
FT   TRANSMEM    274    297
FT   TOPO_DOM    298    321       CYTOPLASMIC.
//
```

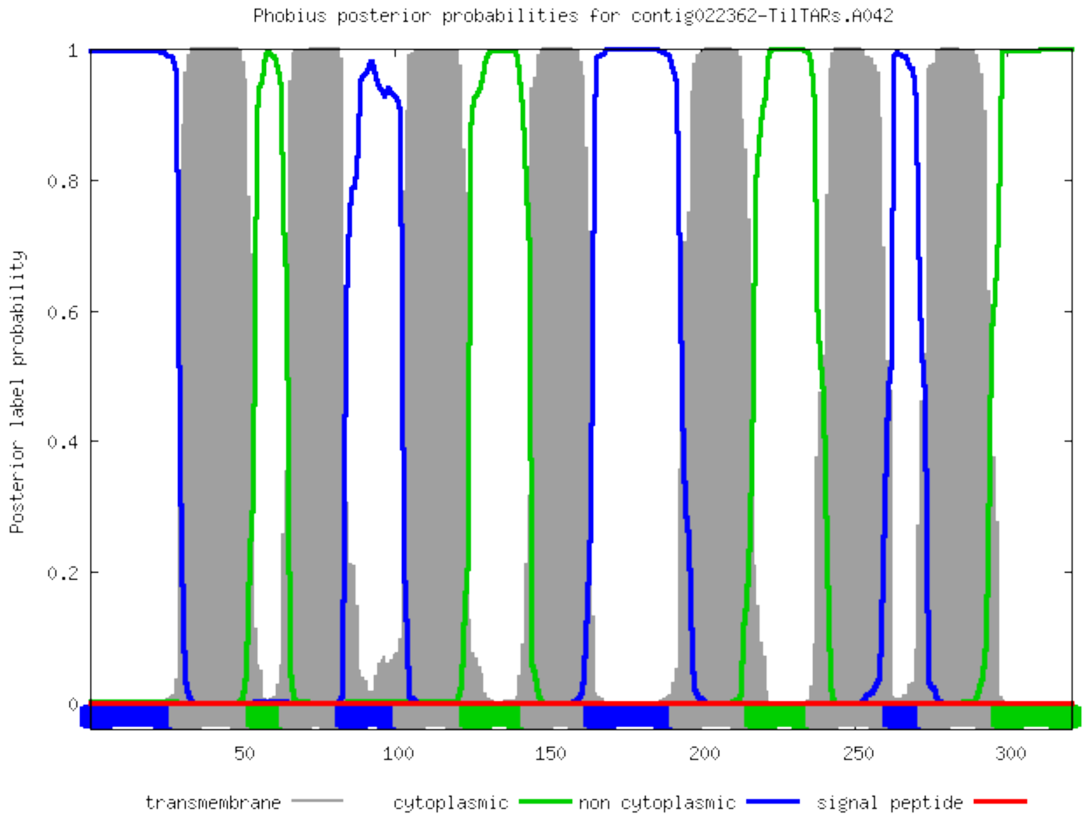

The probability data used in the plot is found here, and the gnuplot script is here.

Prediction of contig022363-TilTARs.A044\

```
ID   contig022363-TilTARs.A044\
FT   TOPO_DOM      1     28       NON CYTOPLASMIC.
FT   TRANSMEM     29     53
FT   TOPO_DOM     54     64       CYTOPLASMIC.
FT   TRANSMEM     65     82
FT   TOPO_DOM     83    101       NON CYTOPLASMIC.
FT   TRANSMEM    102    123
FT   TOPO_DOM    124    143       CYTOPLASMIC.
FT   TRANSMEM    144    163
FT   TOPO_DOM    164    192       NON CYTOPLASMIC.
FT   TRANSMEM    193    216
FT   TOPO_DOM    217    248       CYTOPLASMIC.
FT   TRANSMEM    249    269
FT   TOPO_DOM    270    280       NON CYTOPLASMIC.
FT   TRANSMEM    281    302
FT   TOPO_DOM    303    326       CYTOPLASMIC.
//
```

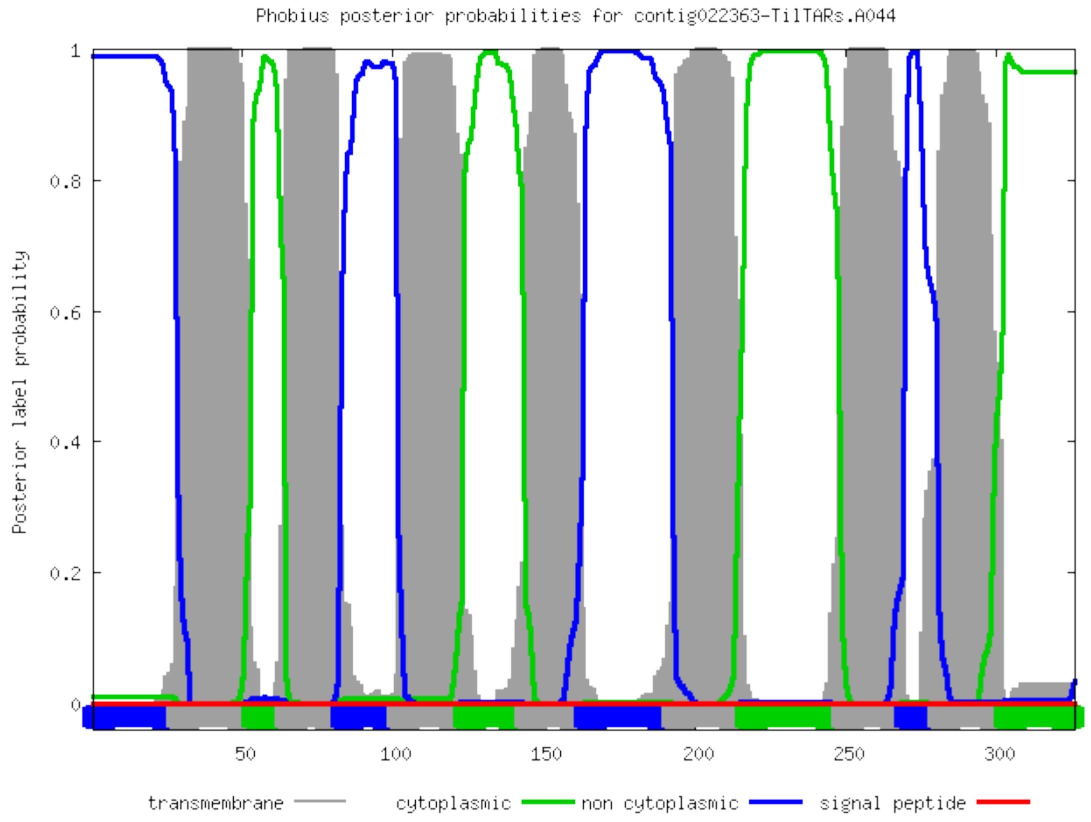

The probability data used in the plot is found here, and the gnuplot script is here.

Prediction of contig022365-TilTARs.A045\

```
ID    contig022365-TilTARs.A045\
FT    TOPO_DOM       1      26       NON CYTOPLASMIC.
FT    TRANSMEM      27      51
FT    TOPO_DOM      52      62       CYTOPLASMIC.
FT    TRANSMEM      63      80
FT    TOPO_DOM      81      99       NON CYTOPLASMIC.
FT    TRANSMEM     100     121
FT    TOPO_DOM     122     141       CYTOPLASMIC.
FT    TRANSMEM     142     161
FT    TOPO_DOM     162     184       NON CYTOPLASMIC.
FT    TRANSMEM     185     211
FT    TOPO_DOM     212     246       CYTOPLASMIC.
FT    TRANSMEM     247     267
FT    TOPO_DOM     268     278       NON CYTOPLASMIC.
FT    TRANSMEM     279     302
FT    TOPO_DOM     303     326       CYTOPLASMIC.
//
```

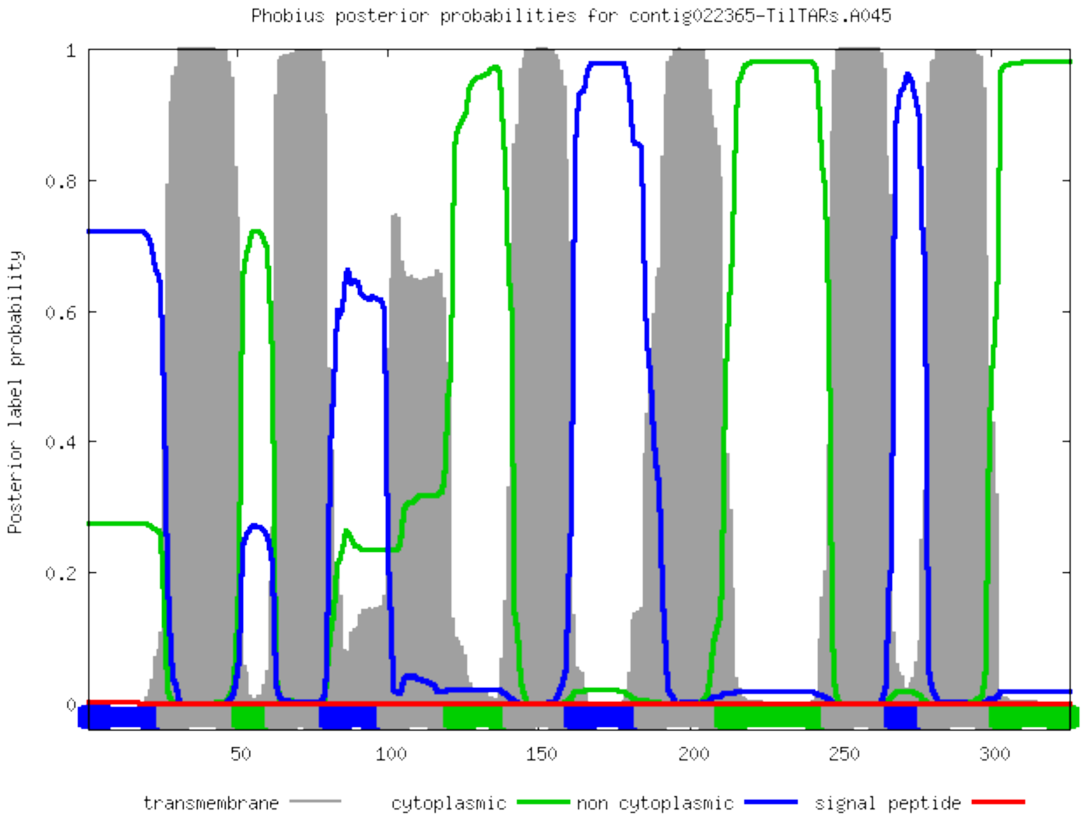

The probability data used in the plot is found here, and the gnuplot script is here.

Prediction of contig022368-TilTARs.A046\

```
ID   contig022368-TilTARs.A046\
FT   TOPO_DOM      1     29       NON CYTOPLASMIC.
FT   TRANSMEM     30     54
FT   TOPO_DOM     55     65       CYTOPLASMIC.
FT   TRANSMEM     66     83
FT   TOPO_DOM     84    102       NON CYTOPLASMIC.
FT   TRANSMEM    103    124
FT   TOPO_DOM    125    144       CYTOPLASMIC.
FT   TRANSMEM    145    164
FT   TOPO_DOM    165    193       NON CYTOPLASMIC.
FT   TRANSMEM    194    217
FT   TOPO_DOM    218    249       CYTOPLASMIC.
FT   TRANSMEM    250    270
FT   TOPO_DOM    271    281       NON CYTOPLASMIC.
FT   TRANSMEM    282    305
FT   TOPO_DOM    306    329       CYTOPLASMIC.
//
```

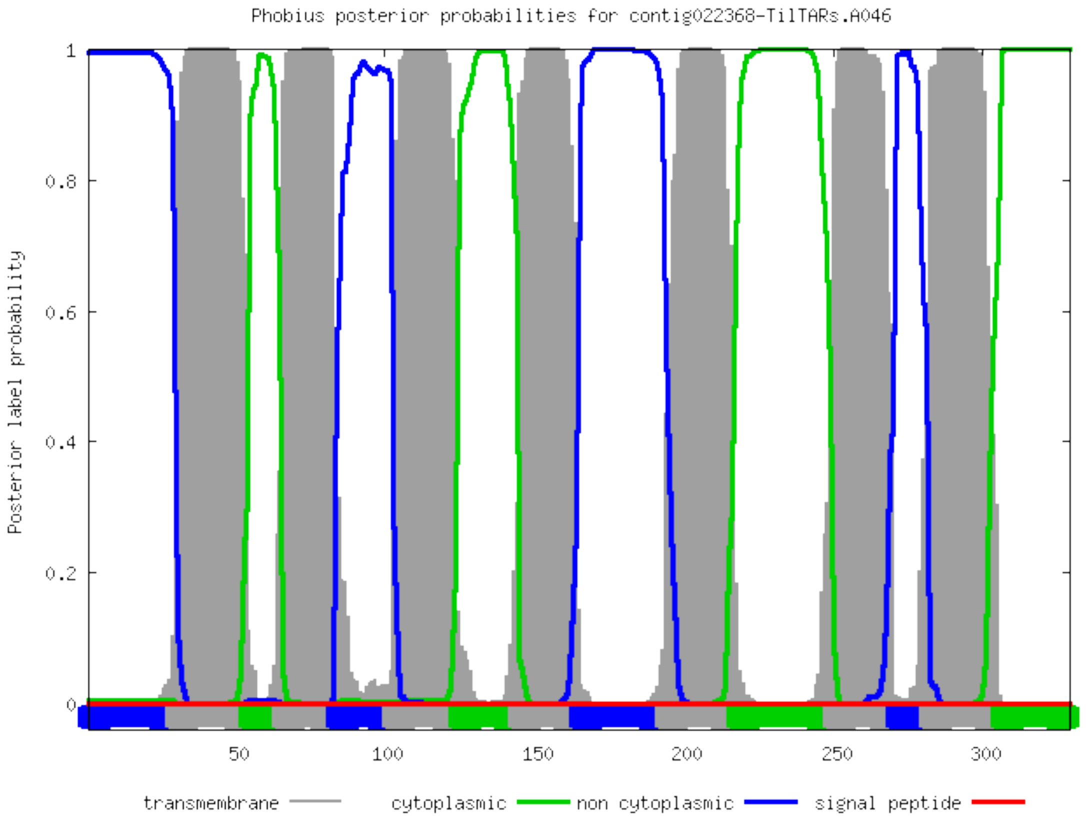

The probability data used in the plot is found here, and the gnuplot script is here.

Prediction of contig022368-TilTARs.A047\

```
ID   contig022368-TilTARs.A047\
FT   TOPO_DOM      1     27       NON CYTOPLASMIC.
FT   TRANSMEM     28     52
FT   TOPO_DOM     53     63       CYTOPLASMIC.
FT   TRANSMEM     64     81
FT   TOPO_DOM     82    100       NON CYTOPLASMIC.
FT   TRANSMEM    101    122
FT   TOPO_DOM    123    142       CYTOPLASMIC.
FT   TRANSMEM    143    162
FT   TOPO_DOM    163    185       NON CYTOPLASMIC.
FT   TRANSMEM    186    212
FT   TOPO_DOM    213    247       CYTOPLASMIC.
FT   TRANSMEM    248    268
FT   TOPO_DOM    269    279       NON CYTOPLASMIC.
FT   TRANSMEM    280    303
FT   TOPO_DOM    304    327       CYTOPLASMIC.
//
```

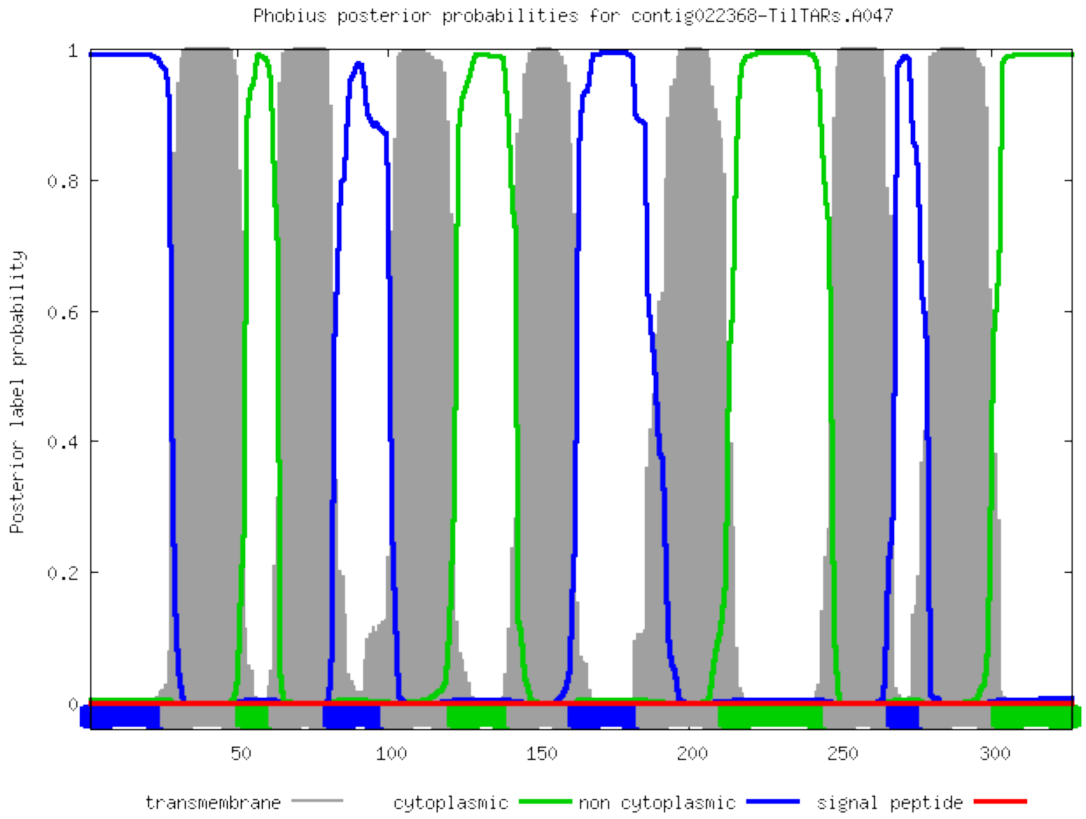

The probability data used in the plot is found here, and the gnuplot script is here.

Prediction of contig022375-TilTARs.A048\

```
ID   contig022375-TilTARs.A048\
FT   TOPO_DOM      1     29       NON CYTOPLASMIC.
FT   TRANSMEM     30     54
FT   TOPO_DOM     55     65       CYTOPLASMIC.
FT   TRANSMEM     66     82
FT   TOPO_DOM     83    101       NON CYTOPLASMIC.
FT   TRANSMEM    102    124
FT   TOPO_DOM    125    144       CYTOPLASMIC.
FT   TRANSMEM    145    163
FT   TOPO_DOM    164    182       NON CYTOPLASMIC.
FT   TRANSMEM    183    214
FT   TOPO_DOM    215    249       CYTOPLASMIC.
FT   TRANSMEM    250    270
FT   TOPO_DOM    271    275       NON CYTOPLASMIC.
FT   TRANSMEM    276    299
FT   TOPO_DOM    300    327       CYTOPLASMIC.
//
```

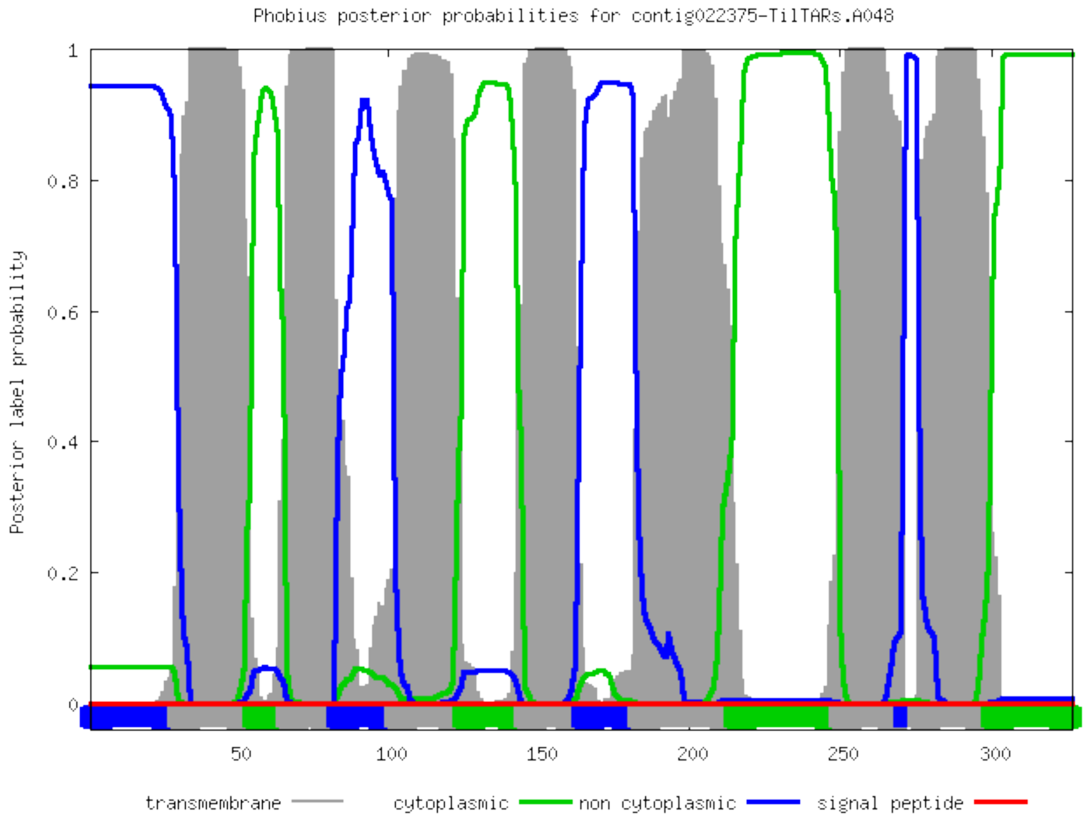

The probability data used in the plot is found here, and the gnuplot script is here.

Prediction of contig022377-TilTARs.A050\

```
ID    contig022377-TilTARs.A050\
FT    TOPO_DOM      1     27       NON CYTOPLASMIC.
FT    TRANSMEM     28     52
FT    TOPO_DOM     53     63       CYTOPLASMIC.
FT    TRANSMEM     64     81
FT    TOPO_DOM     82    100       NON CYTOPLASMIC.
FT    TRANSMEM    101    122
FT    TOPO_DOM    123    142       CYTOPLASMIC.
FT    TRANSMEM    143    166
FT    TOPO_DOM    167    185       NON CYTOPLASMIC.
FT    TRANSMEM    186    212
FT    TOPO_DOM    213    247       CYTOPLASMIC.
FT    TRANSMEM    248    268
FT    TOPO_DOM    269    279       NON CYTOPLASMIC.
FT    TRANSMEM    280    303
FT    TOPO_DOM    304    327       CYTOPLASMIC.
//
```

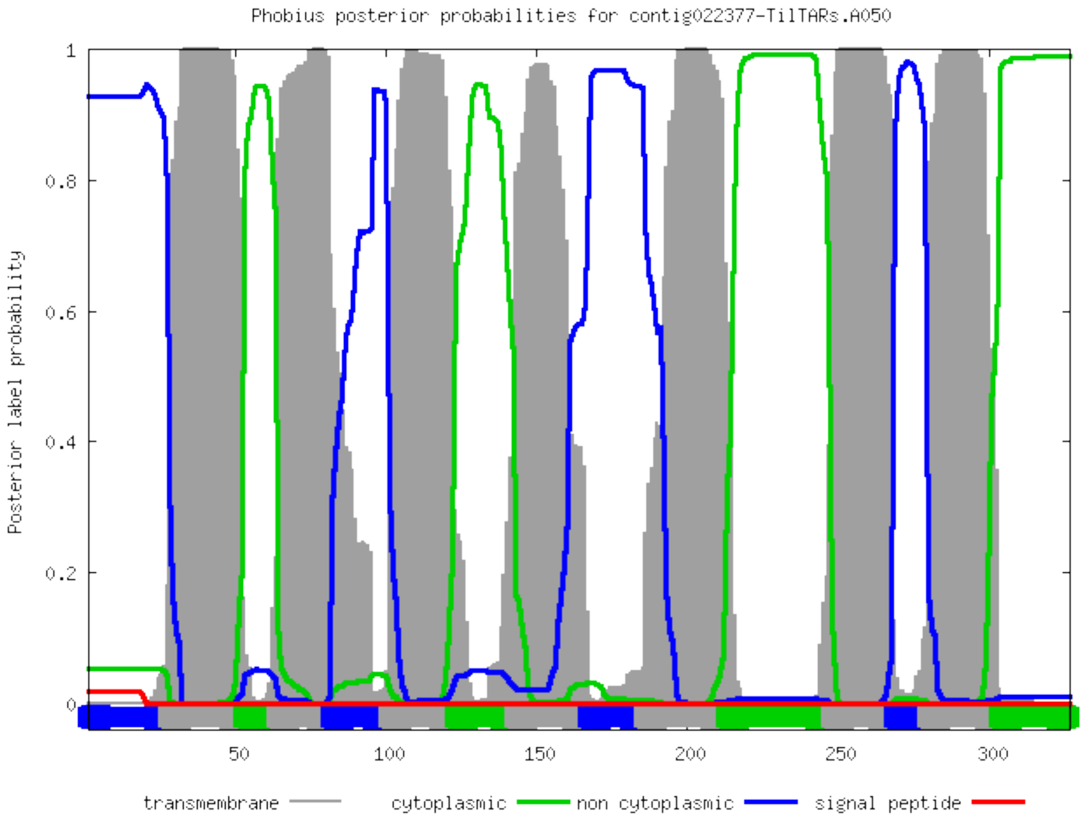

The probability data used in the plot is found here, and the gnuplot script is here.

Prediction of contig022378-TilTARs.A051\

```
ID    contig022378-TilTARs.A051\
FT    TOPO_DOM      1     30       NON CYTOPLASMIC.
FT    TRANSMEM     31     53
FT    TOPO_DOM     54     64       CYTOPLASMIC.
FT    TRANSMEM     65     96
FT    TOPO_DOM     97    101       NON CYTOPLASMIC.
FT    TRANSMEM    102    123
FT    TOPO_DOM    124    143       CYTOPLASMIC.
FT    TRANSMEM    144    163
FT    TOPO_DOM    164    182       NON CYTOPLASMIC.
FT    TRANSMEM    183    213
FT    TOPO_DOM    214    249       CYTOPLASMIC.
FT    TRANSMEM    250    271
FT    TOPO_DOM    272    282       NON CYTOPLASMIC.
FT    TRANSMEM    283    305
FT    TOPO_DOM    306    329       CYTOPLASMIC.
//
```

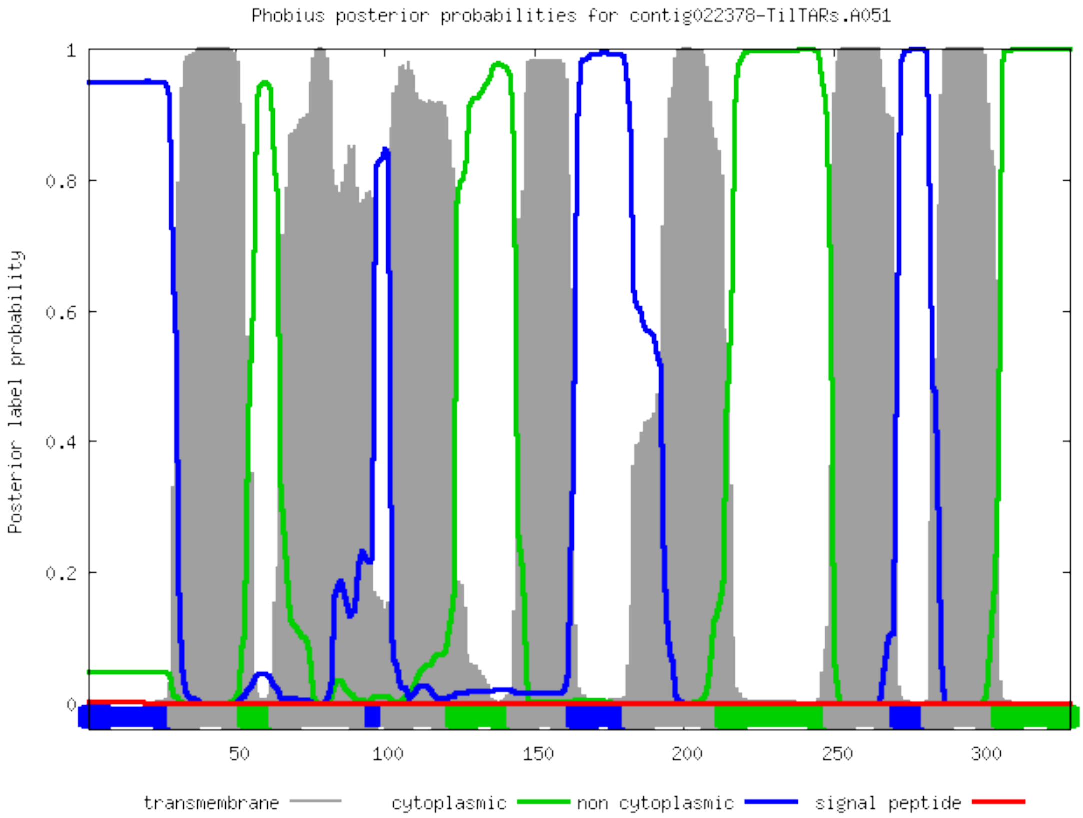

The probability data used in the plot is found here, and the gnuplot script is here.

Prediction of contig022379-TilTARs.A052\

```
ID   contig022379-TilTARs.A052\
FT   TOPO_DOM      1     29       NON CYTOPLASMIC.
FT   TRANSMEM     30     54
FT   TOPO_DOM     55     65       CYTOPLASMIC.
FT   TRANSMEM     66     83
FT   TOPO_DOM     84    102       NON CYTOPLASMIC.
FT   TRANSMEM    103    124
FT   TOPO_DOM    125    144       CYTOPLASMIC.
FT   TRANSMEM    145    164
FT   TOPO_DOM    165    193       NON CYTOPLASMIC.
FT   TRANSMEM    194    217
FT   TOPO_DOM    218    246       CYTOPLASMIC.
FT   TRANSMEM    247    267
FT   TOPO_DOM    268    286       NON CYTOPLASMIC.
FT   TRANSMEM    287    307
FT   TOPO_DOM    308    331       CYTOPLASMIC.
//
```

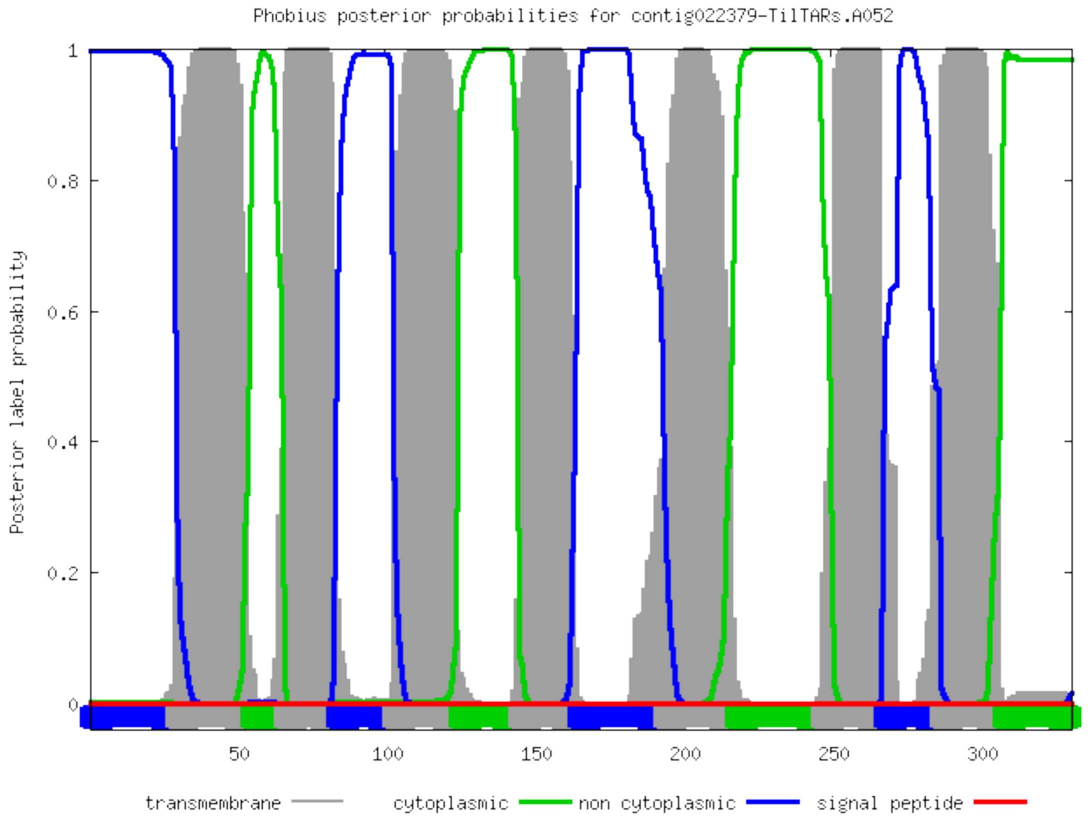

The probability data used in the plot is found here, and the gnuplot script is here.

Prediction of contig022382-TilTARs.A053\

```
ID   contig022382-TilTARs.A053\
FT   TOPO_DOM      1     29       NON CYTOPLASMIC.
FT   TRANSMEM     30     54
FT   TOPO_DOM     55     65       CYTOPLASMIC.
FT   TRANSMEM     66     82
FT   TOPO_DOM     83    101       NON CYTOPLASMIC.
FT   TRANSMEM    102    124
FT   TOPO_DOM    125    144       CYTOPLASMIC.
FT   TRANSMEM    145    165
FT   TOPO_DOM    166    184       NON CYTOPLASMIC.
FT   TRANSMEM    185    214
FT   TOPO_DOM    215    249       CYTOPLASMIC.
FT   TRANSMEM    250    270
FT   TOPO_DOM    271    281       NON CYTOPLASMIC.
FT   TRANSMEM    282    301
FT   TOPO_DOM    302    329       CYTOPLASMIC.
//
```

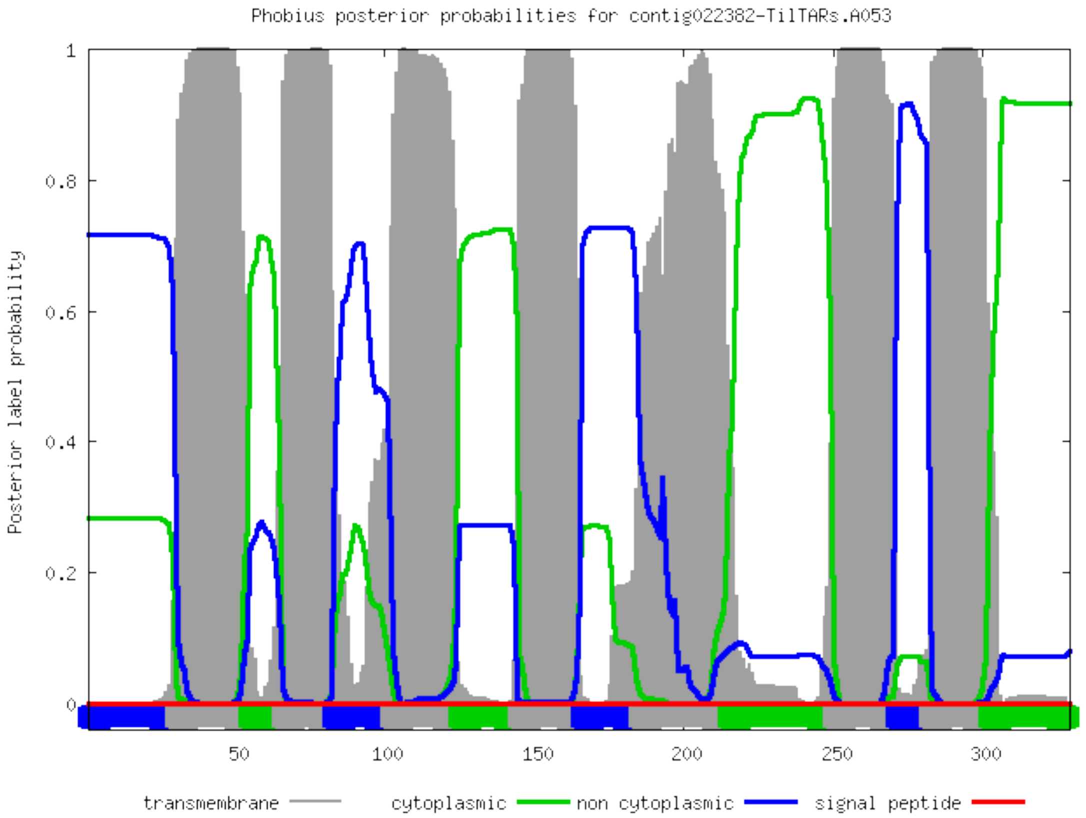

The probability data used in the plot is found here, and the gnuplot script is here.

Prediction of contig022383-TilTARs.A054\

```
ID    contig022383-TilTARs.A054\
FT    TOPO_DOM      1     29       NON CYTOPLASMIC.
FT    TRANSMEM     30     54
FT    TOPO_DOM     55     65       CYTOPLASMIC.
FT    TRANSMEM     66     83
FT    TOPO_DOM     84    102       NON CYTOPLASMIC.
FT    TRANSMEM    103    124
FT    TOPO_DOM    125    144       CYTOPLASMIC.
FT    TRANSMEM    145    164
FT    TOPO_DOM    165    193       NON CYTOPLASMIC.
FT    TRANSMEM    194    217
FT    TOPO_DOM    218    250       CYTOPLASMIC.
FT    TRANSMEM    251    271
FT    TOPO_DOM    272    282       NON CYTOPLASMIC.
FT    TRANSMEM    283    302
FT    TOPO_DOM    303    330       CYTOPLASMIC.
//
```

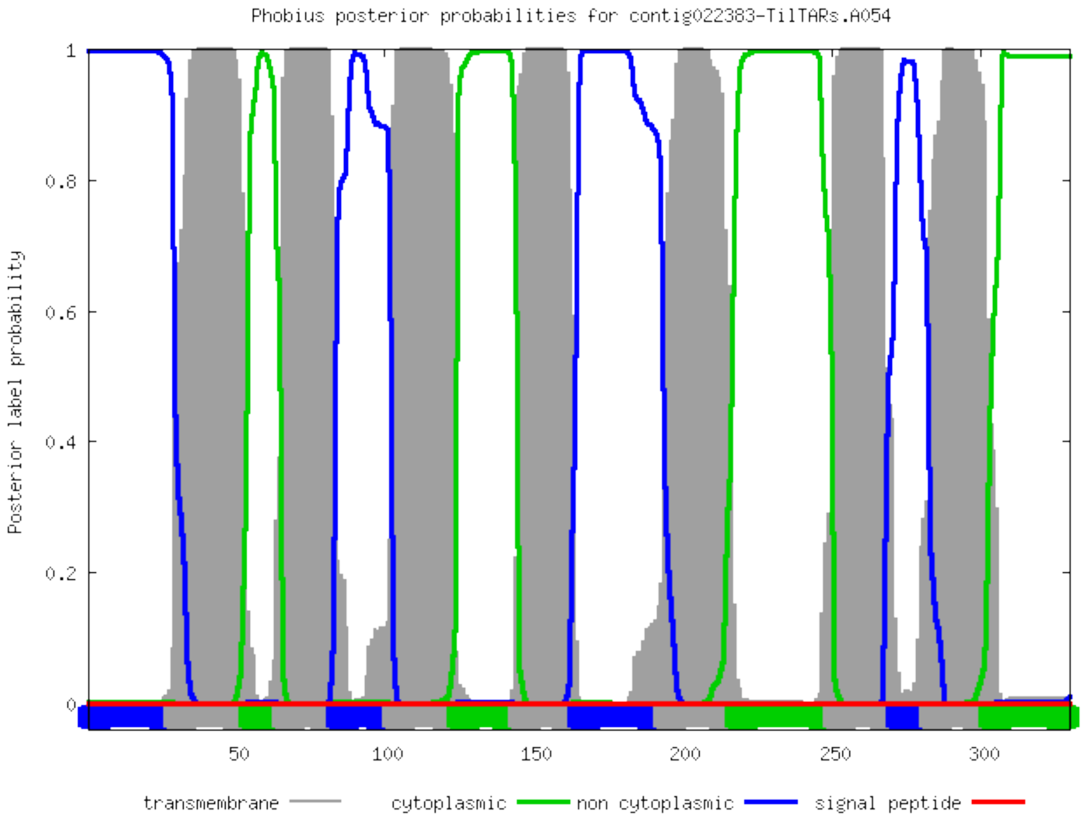

The probability data used in the plot is found here, and the gnuplot script is here.

Prediction of contig022390-TilTARs.A055\

```
ID    contig022390-TilTARs.A055\
FT    TOPO_DOM        1      29       CYTOPLASMIC.
FT    TRANSMEM       30      53
FT    TOPO_DOM       54      72       NON CYTOPLASMIC.
FT    TRANSMEM       73      94
FT    TOPO_DOM       95     114       CYTOPLASMIC.
FT    TRANSMEM      115     134
FT    TOPO_DOM      135     153       NON CYTOPLASMIC.
FT    TRANSMEM      154     184
FT    TOPO_DOM      185     220       CYTOPLASMIC.
FT    TRANSMEM      221     241
FT    TOPO_DOM      242     252       NON CYTOPLASMIC.
FT    TRANSMEM      253     276
FT    TOPO_DOM      277     300       CYTOPLASMIC.
//
```

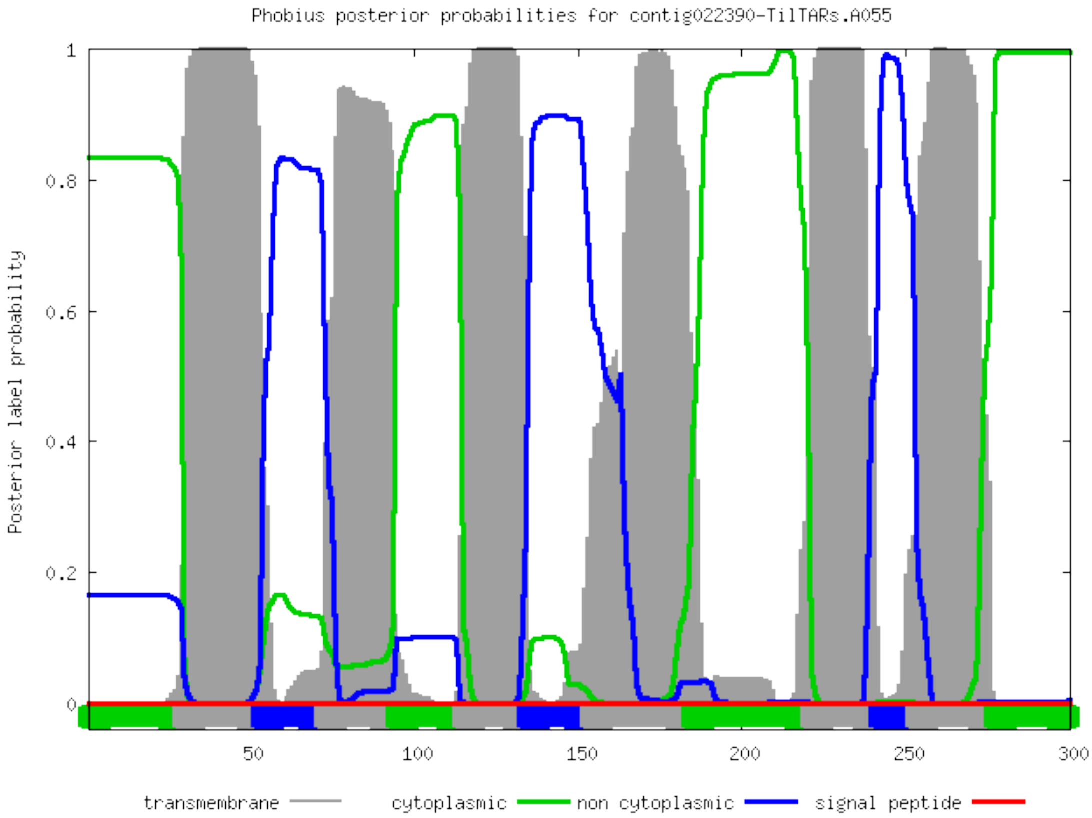

The probability data used in the plot is found here, and the gnuplot script is here.

Prediction of contig022390-TilTARs.A056\

```
ID    contig022390-TilTARs.A056\
FT    TOPO_DOM      1     29       NON CYTOPLASMIC.
FT    TRANSMEM     30     54
FT    TOPO_DOM     55     65       CYTOPLASMIC.
FT    TRANSMEM     66     83
FT    TOPO_DOM     84    102       NON CYTOPLASMIC.
FT    TRANSMEM    103    124
FT    TOPO_DOM    125    144       CYTOPLASMIC.
FT    TRANSMEM    145    164
FT    TOPO_DOM    165    193       NON CYTOPLASMIC.
FT    TRANSMEM    194    217
FT    TOPO_DOM    218    250       CYTOPLASMIC.
FT    TRANSMEM    251    271
FT    TOPO_DOM    272    282       NON CYTOPLASMIC.
FT    TRANSMEM    283    302
FT    TOPO_DOM    303    330       CYTOPLASMIC.
//
```

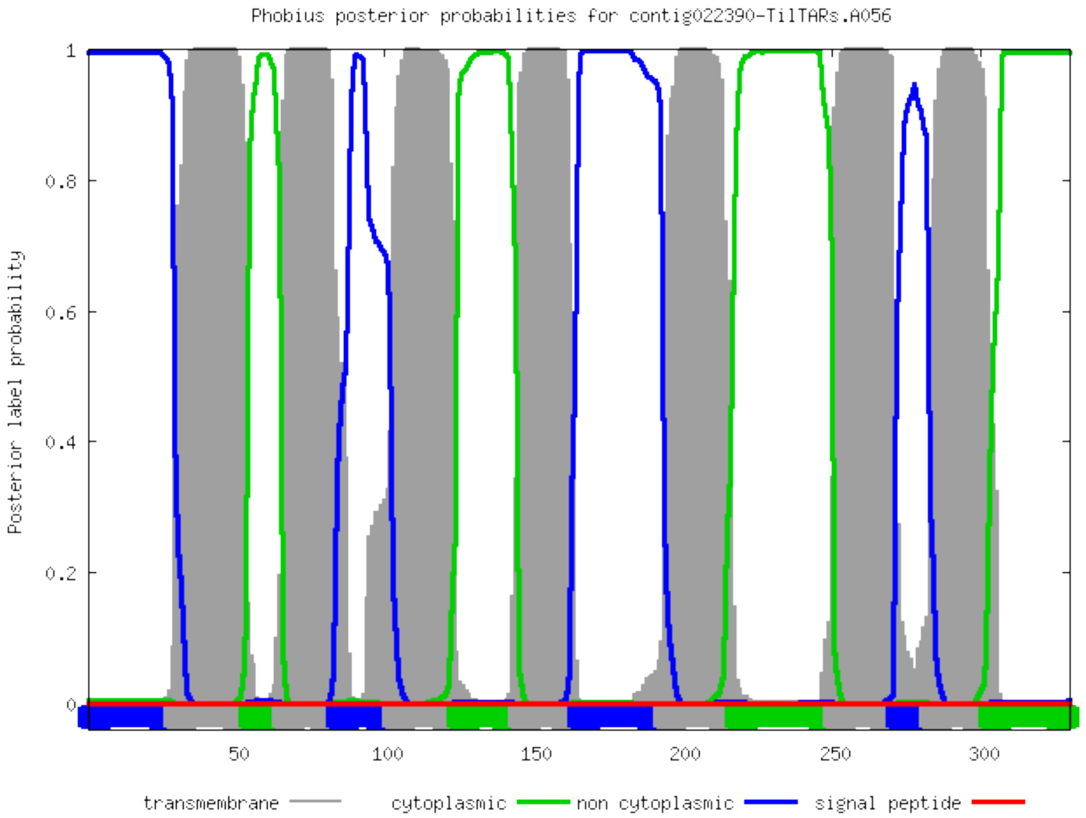

The probability data used in the plot is found here, and the gnuplot script is here.

Prediction of contig045088-TilTARs.A057\

```
ID   contig045088-TilTARs.A057\
FT   TOPO_DOM      1     26       NON CYTOPLASMIC.
FT   TRANSMEM     27     51
FT   TOPO_DOM     52     62       CYTOPLASMIC.
FT   TRANSMEM     63     86
FT   TOPO_DOM     87     97       NON CYTOPLASMIC.
FT   TRANSMEM     98    120
FT   TOPO_DOM    121    140       CYTOPLASMIC.
FT   TRANSMEM    141    160
FT   TOPO_DOM    161    193       NON CYTOPLASMIC.
FT   TRANSMEM    194    213
FT   TOPO_DOM    214    246       CYTOPLASMIC.
FT   TRANSMEM    247    267
FT   TOPO_DOM    268    278       NON CYTOPLASMIC.
FT   TRANSMEM    279    299
FT   TOPO_DOM    300    331       CYTOPLASMIC.
//
```

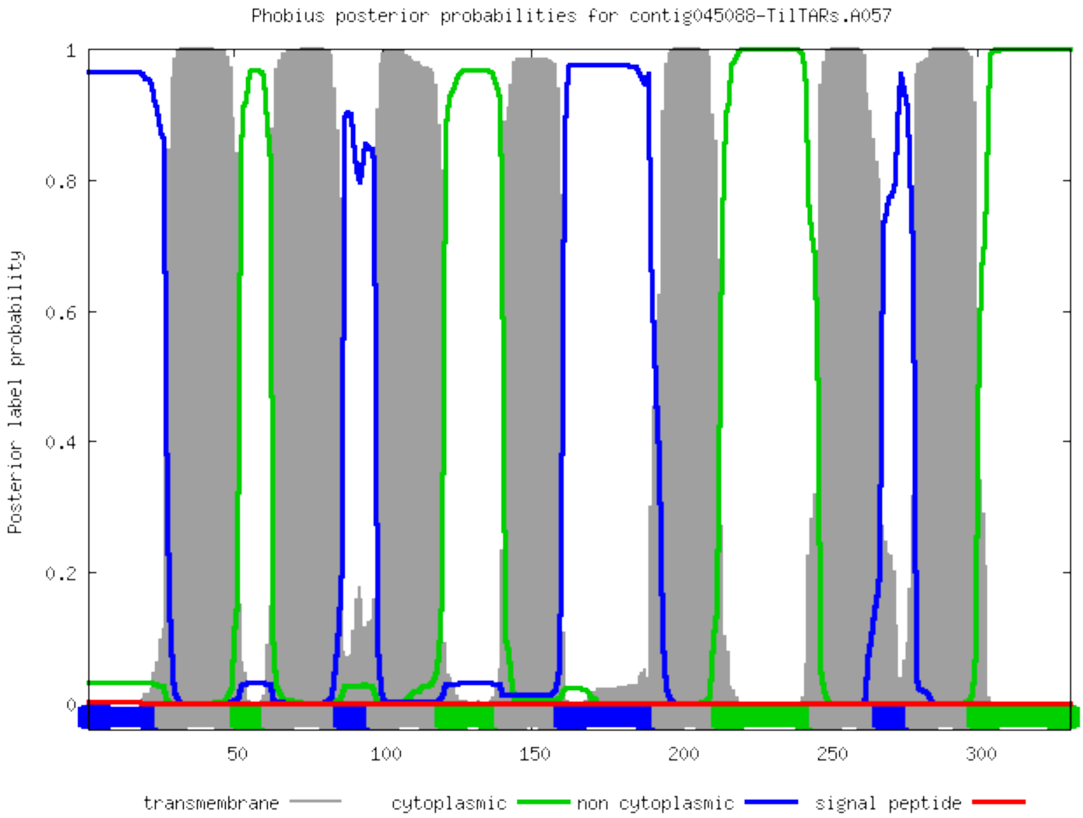

The probability data used in the plot is found here, and the gnuplot script is here.

Prediction of contig056134-TilTARs.A058\

```
ID   contig056134-TilTARs.A058\
FT   TOPO_DOM      1     26       NON CYTOPLASMIC.
FT   TRANSMEM     27     51
FT   TOPO_DOM     52     62       CYTOPLASMIC.
FT   TRANSMEM     63     93
FT   TOPO_DOM     94     98       NON CYTOPLASMIC.
FT   TRANSMEM     99    120
FT   TOPO_DOM    121    140       CYTOPLASMIC.
FT   TRANSMEM    141    161
FT   TOPO_DOM    162    191       NON CYTOPLASMIC.
FT   TRANSMEM    192    213
FT   TOPO_DOM    214    242       CYTOPLASMIC.
FT   TRANSMEM    243    267
FT   TOPO_DOM    268    278       NON CYTOPLASMIC.
FT   TRANSMEM    279    302
FT   TOPO_DOM    303    326       CYTOPLASMIC.
//
```

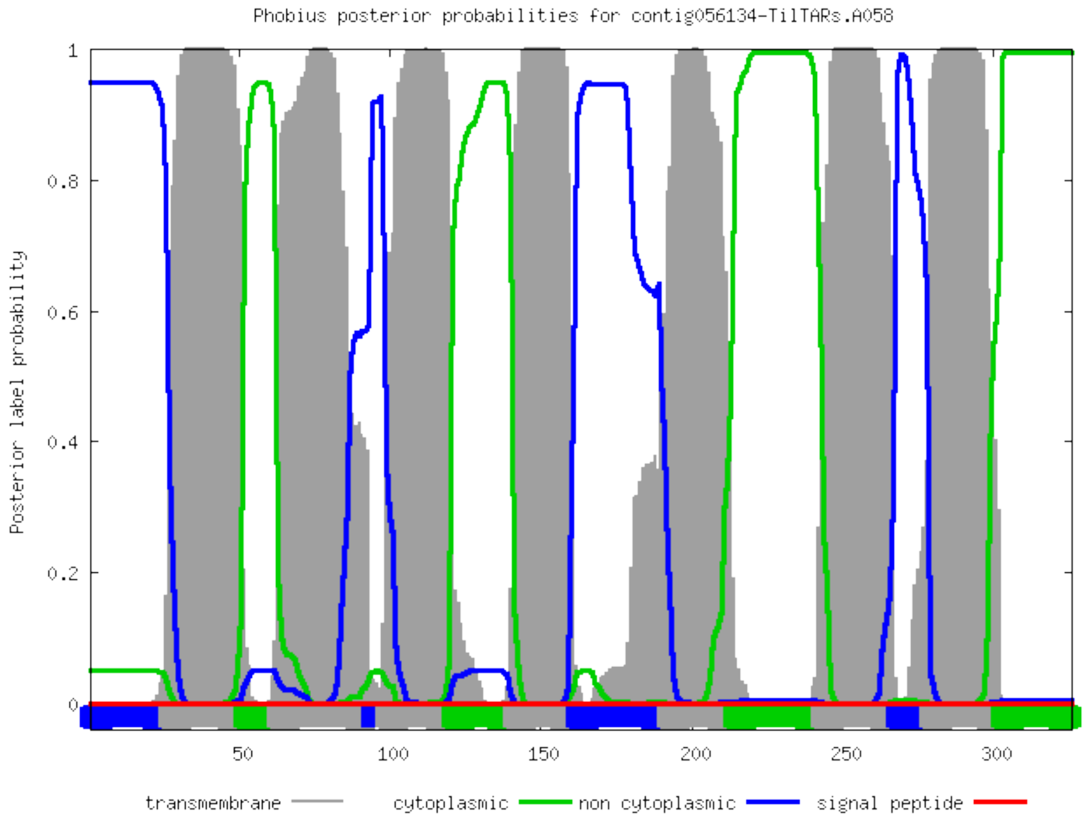

The probability data used in the plot is found here, and the gnuplot script is here.

Prediction of contig022320-TilTARs.A059\

```
ID   contig022320-TilTARs.A059\
FT   TOPO_DOM      1     29       NON CYTOPLASMIC.
FT   TRANSMEM     30     54
FT   TOPO_DOM     55     65       CYTOPLASMIC.
FT   TRANSMEM     66     83
FT   TOPO_DOM     84    102       NON CYTOPLASMIC.
FT   TRANSMEM    103    124
FT   TOPO_DOM    125    144       CYTOPLASMIC.
FT   TRANSMEM    145    163
FT   TOPO_DOM    164    193       NON CYTOPLASMIC.
FT   TRANSMEM    194    217
FT   TOPO_DOM    218    249       CYTOPLASMIC.
FT   TRANSMEM    250    270
FT   TOPO_DOM    271    281       NON CYTOPLASMIC.
FT   TRANSMEM    282    301
FT   TOPO_DOM    302    329       CYTOPLASMIC.
//
```

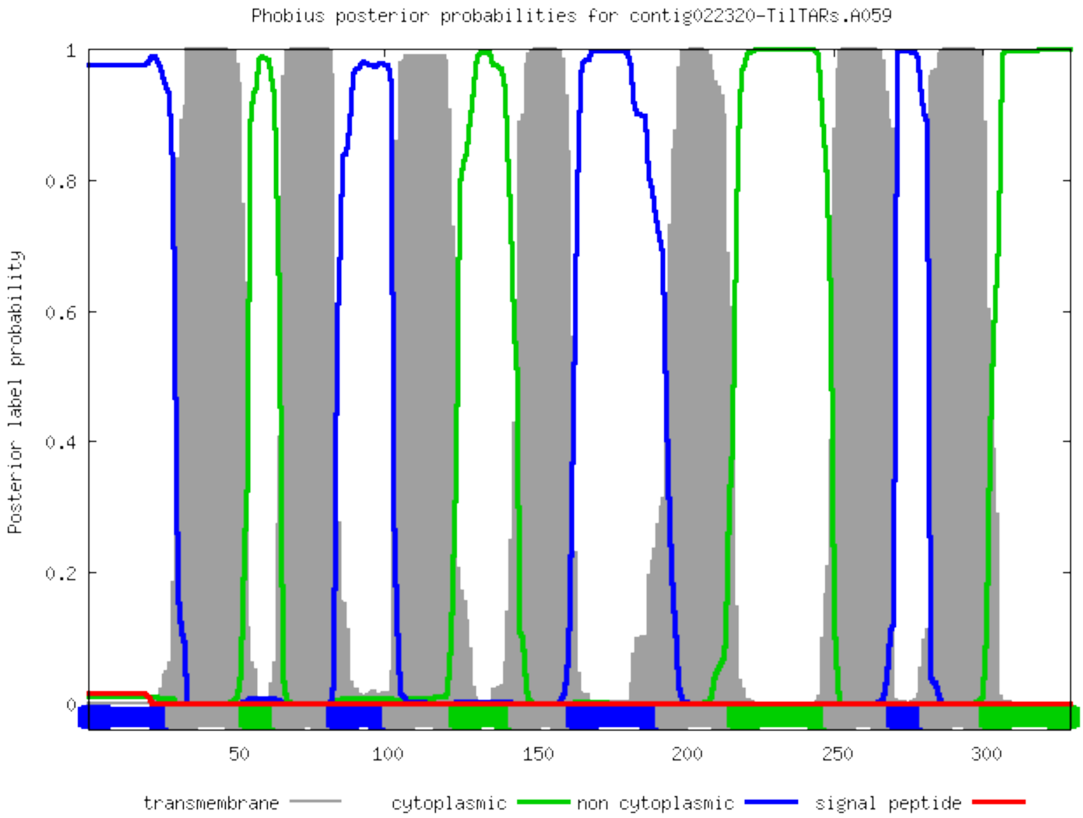

The probability data used in the plot is found here, and the gnuplot script is here.

Prediction of contig002574-TilTAR.B062\

```
ID   contig002574-TilTAR.B062\
FT   TOPO_DOM      1     26       CYTOPLASMIC.
FT   TRANSMEM     27     56
FT   TOPO_DOM     57     67       NON CYTOPLASMIC.
FT   TRANSMEM     68     91
FT   TOPO_DOM     92    144       CYTOPLASMIC.
FT   TRANSMEM    145    169
FT   TOPO_DOM    170    193       NON CYTOPLASMIC.
FT   TRANSMEM    194    213
FT   TOPO_DOM    214    239       CYTOPLASMIC.
FT   TRANSMEM    240    263
FT   TOPO_DOM    264    274       NON CYTOPLASMIC.
FT   TRANSMEM    275    294
FT   TOPO_DOM    295    322       CYTOPLASMIC.
//
```

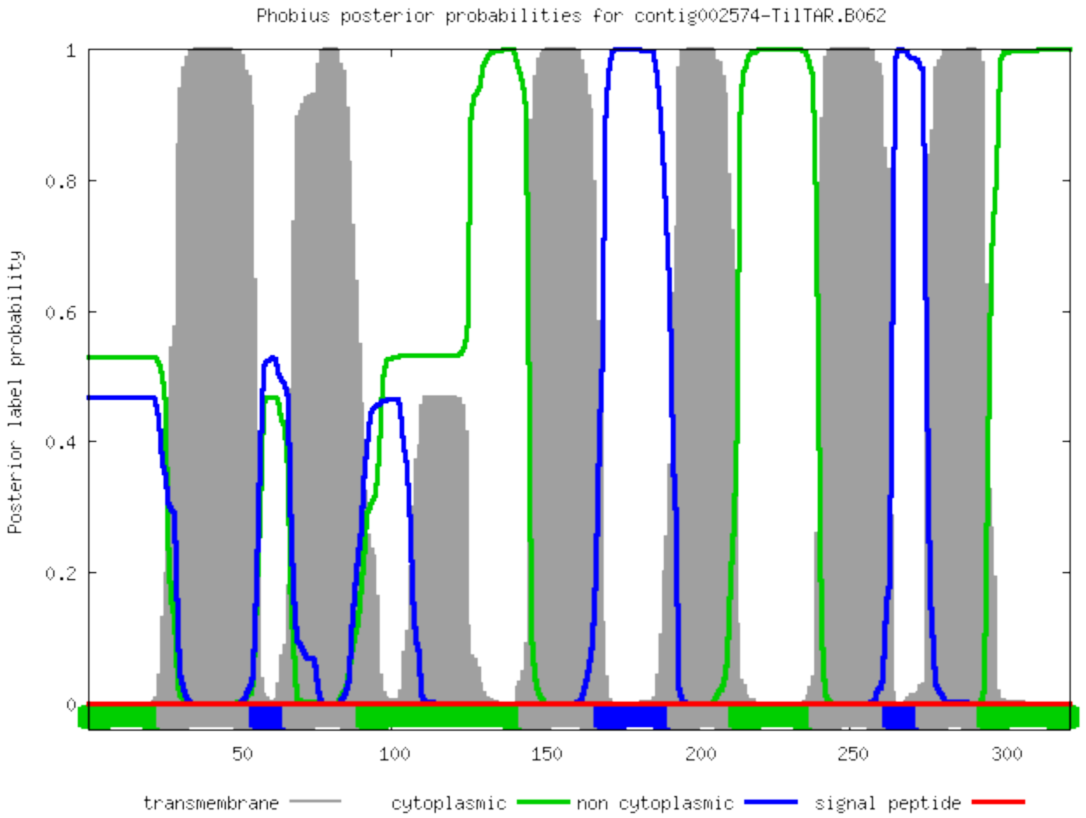

The probability data used in the plot is found here, and the gnuplot script is here.

Prediction of contig037900-TilTAR.B063\

```
ID    contig037900-TilTAR.B063\
FT    TOPO_DOM      1     36       NON CYTOPLASMIC.
FT    TRANSMEM     37     64
FT    TOPO_DOM     65     75       CYTOPLASMIC.
FT    TRANSMEM     76    105
FT    TOPO_DOM    106    116       NON CYTOPLASMIC.
FT    TRANSMEM    117    136
FT    TOPO_DOM    137    147       CYTOPLASMIC.
FT    TRANSMEM    148    174
FT    TOPO_DOM    175    193       NON CYTOPLASMIC.
FT    TRANSMEM    194    217
FT    TOPO_DOM    218    246       CYTOPLASMIC.
FT    TRANSMEM    247    270
FT    TOPO_DOM    271    281       NON CYTOPLASMIC.
FT    TRANSMEM    282    301
FT    TOPO_DOM    302    302       CYTOPLASMIC.
//
```

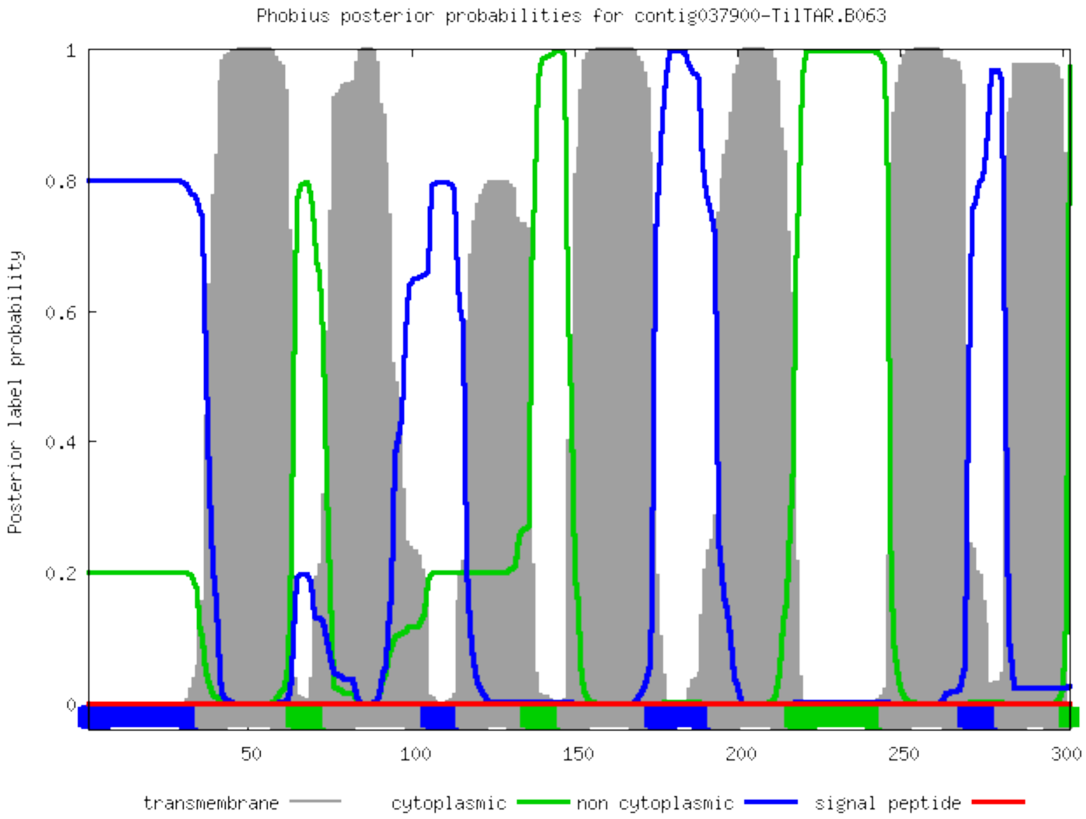

The probability data used in the plot is found here, and the gnuplot script is here.

Prediction of contig007518-TilTARs.A019\

```
ID   contig007518-TilTARs.A019\
FT   TOPO_DOM      1     28       NON CYTOPLASMIC.
FT   TRANSMEM     29     53
FT   TOPO_DOM     54     64       CYTOPLASMIC.
FT   TRANSMEM     65     88
FT   TOPO_DOM     89     99       NON CYTOPLASMIC.
FT   TRANSMEM    100    122
FT   TOPO_DOM    123    142       CYTOPLASMIC.
FT   TRANSMEM    143    162
FT   TOPO_DOM    163    181       NON CYTOPLASMIC.
FT   TRANSMEM    182    215
FT   TOPO_DOM    216    247       CYTOPLASMIC.
FT   TRANSMEM    248    270
FT   TOPO_DOM    271    281       NON CYTOPLASMIC.
FT   TRANSMEM    282    305
FT   TOPO_DOM    306    331       CYTOPLASMIC.
//
```

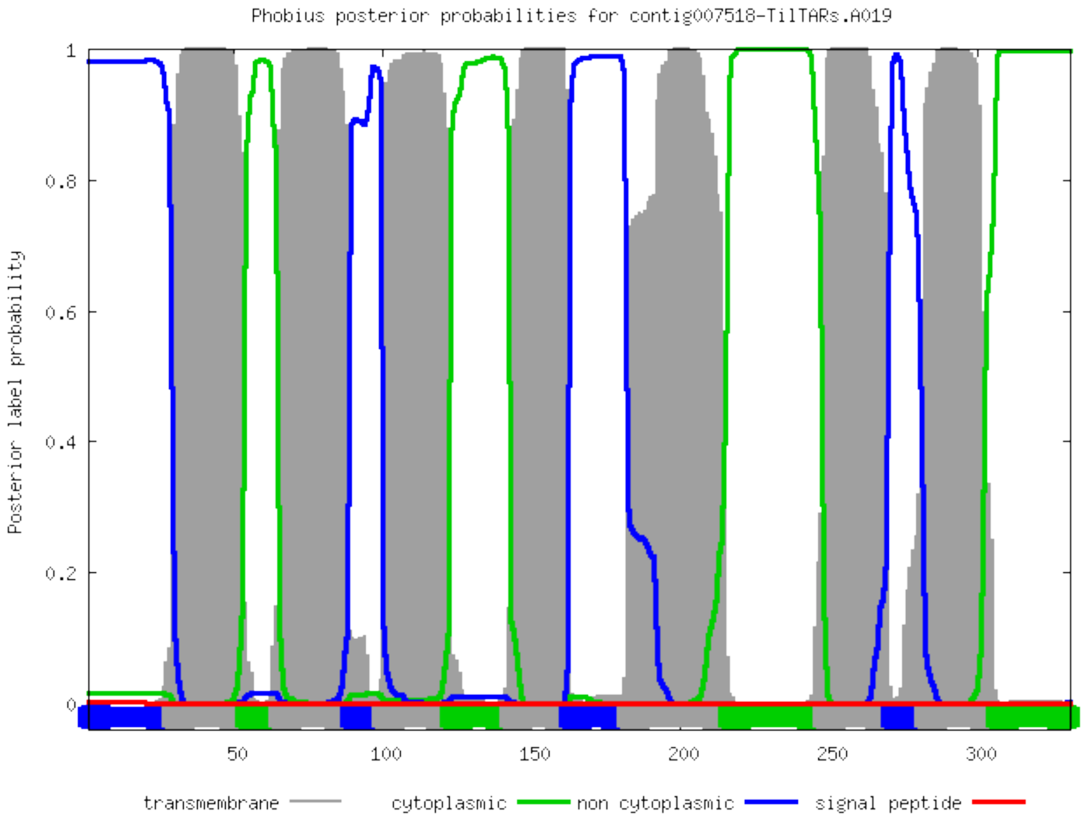

The probability data used in the plot is found here, and the gnuplot script is here.

Prediction of contig032272-NyeTAR.A004\

```
ID   contig032272-NyeTAR.A004\
FT   TOPO_DOM      1     29       NON CYTOPLASMIC.
FT   TRANSMEM     30     54
FT   TOPO_DOM     55     65       CYTOPLASMIC.
FT   TRANSMEM     66     83
FT   TOPO_DOM     84    102       NON CYTOPLASMIC.
FT   TRANSMEM    103    125
FT   TOPO_DOM    126    145       CYTOPLASMIC.
FT   TRANSMEM    146    165
FT   TOPO_DOM    166    194       NON CYTOPLASMIC.
FT   TRANSMEM    195    218
FT   TOPO_DOM    219    246       CYTOPLASMIC.
FT   TRANSMEM    247    267
FT   TOPO_DOM    268    286       NON CYTOPLASMIC.
FT   TRANSMEM    287    307
FT   TOPO_DOM    308    331       CYTOPLASMIC.
//
```

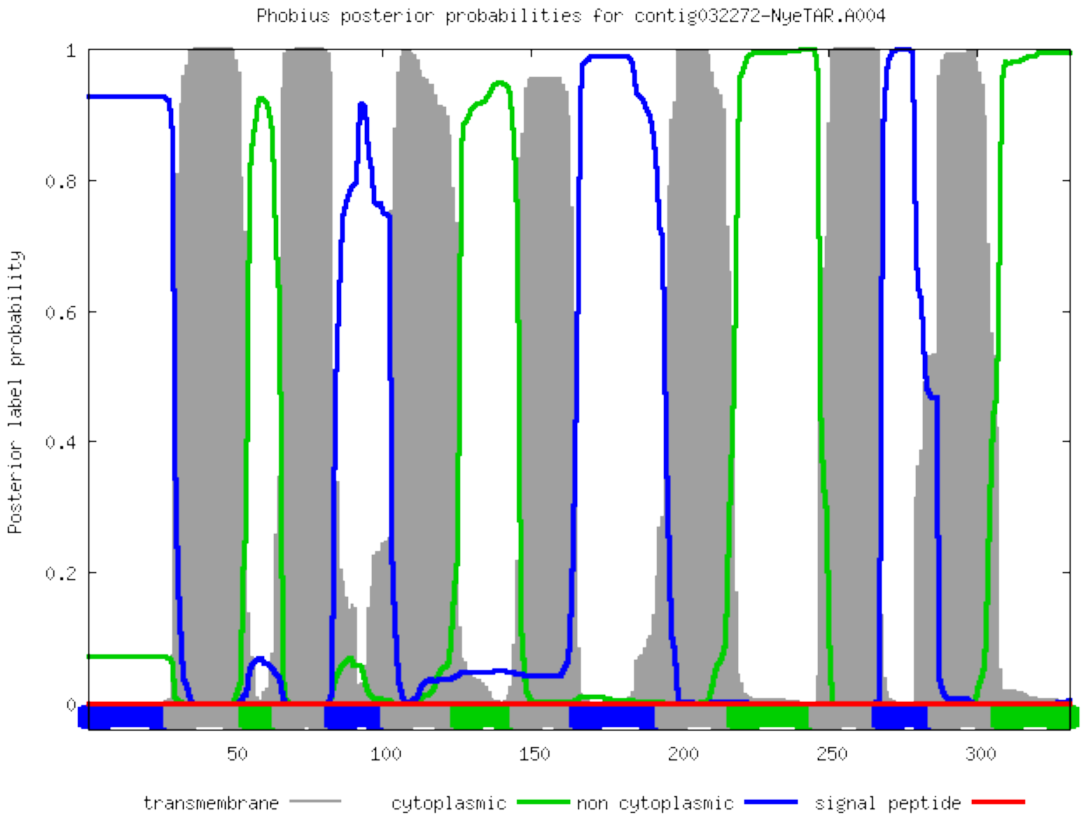

The probability data used in the plot is found here, and the gnuplot script is here.

Prediction of contig038663-NyeTAR.A005\

```
ID   contig038663-NyeTAR.A005\
FT   TOPO_DOM      1     28       NON CYTOPLASMIC.
FT   TRANSMEM     29     53
FT   TOPO_DOM     54     64       CYTOPLASMIC.
FT   TRANSMEM     65     90
FT   TOPO_DOM     91    105       NON CYTOPLASMIC.
FT   TRANSMEM    106    124
FT   TOPO_DOM    125    144       CYTOPLASMIC.
FT   TRANSMEM    145    166
FT   TOPO_DOM    167    193       NON CYTOPLASMIC.
FT   TRANSMEM    194    217
FT   TOPO_DOM    218    244       CYTOPLASMIC.
FT   TRANSMEM    245    262
FT   TOPO_DOM    263    273       NON CYTOPLASMIC.
FT   TRANSMEM    274    298
FT   TOPO_DOM    299    326       CYTOPLASMIC.
//
```

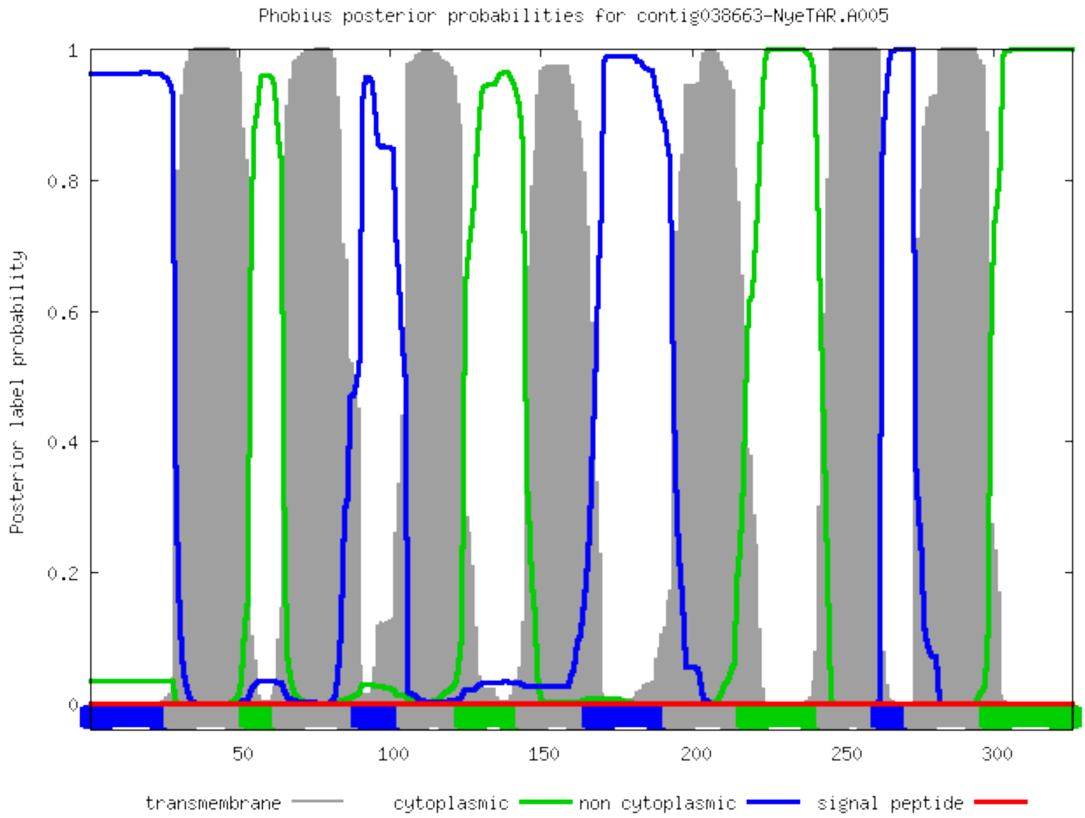

The probability data used in the plot is found here, and the gnuplot script is here.

Prediction of contig052987-NyeTAR.B030\

```
ID   contig052987-NyeTAR.B030\
FT   TOPO_DOM      1     24       NON CYTOPLASMIC.
FT   TRANSMEM     25     52
FT   TOPO_DOM     53     60       CYTOPLASMIC.
FT   TRANSMEM     61     85
FT   TOPO_DOM     86    104       NON CYTOPLASMIC.
FT   TRANSMEM    105    125
FT   TOPO_DOM    126    145       CYTOPLASMIC.
FT   TRANSMEM    146    166
FT   TOPO_DOM    167    171       NON CYTOPLASMIC.
FT   TRANSMEM    172    196
FT   TOPO_DOM    197    233       CYTOPLASMIC.
FT   TRANSMEM    234    257
FT   TOPO_DOM    258    268       NON CYTOPLASMIC.
FT   TRANSMEM    269    291
FT   TOPO_DOM    292    315       CYTOPLASMIC.
//
```

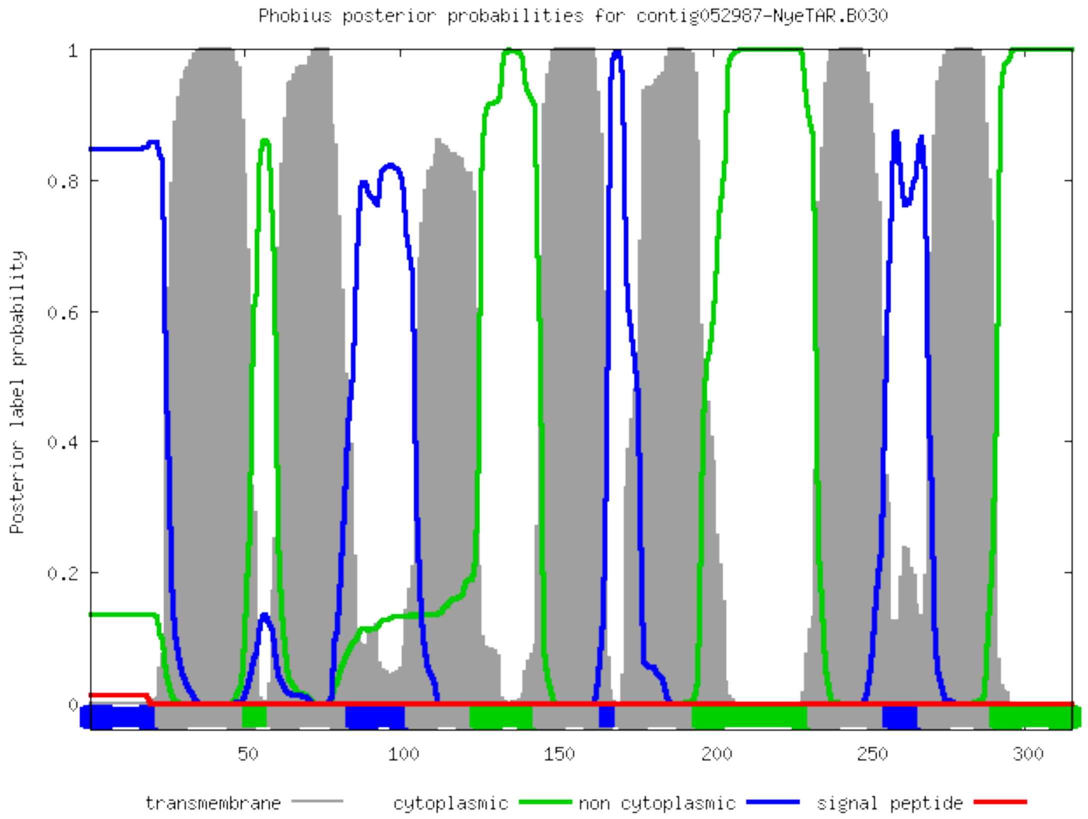

The probability data used in the plot is found here, and the gnuplot script is here.

Prediction of contig035375-NyeTARs.A013\

```
ID    contig035375-NyeTARs.A013\
FT    TOPO_DOM      1     29       NON CYTOPLASMIC.
FT    TRANSMEM     30     54
FT    TOPO_DOM     55     65       CYTOPLASMIC.
FT    TRANSMEM     66     83
FT    TOPO_DOM     84    102       NON CYTOPLASMIC.
FT    TRANSMEM    103    124
FT    TOPO_DOM    125    144       CYTOPLASMIC.
FT    TRANSMEM    145    164
FT    TOPO_DOM    165    193       NON CYTOPLASMIC.
FT    TRANSMEM    194    217
FT    TOPO_DOM    218    250       CYTOPLASMIC.
FT    TRANSMEM    251    271
FT    TOPO_DOM    272    282       NON CYTOPLASMIC.
FT    TRANSMEM    283    306
FT    TOPO_DOM    307    330       CYTOPLASMIC.
//
```

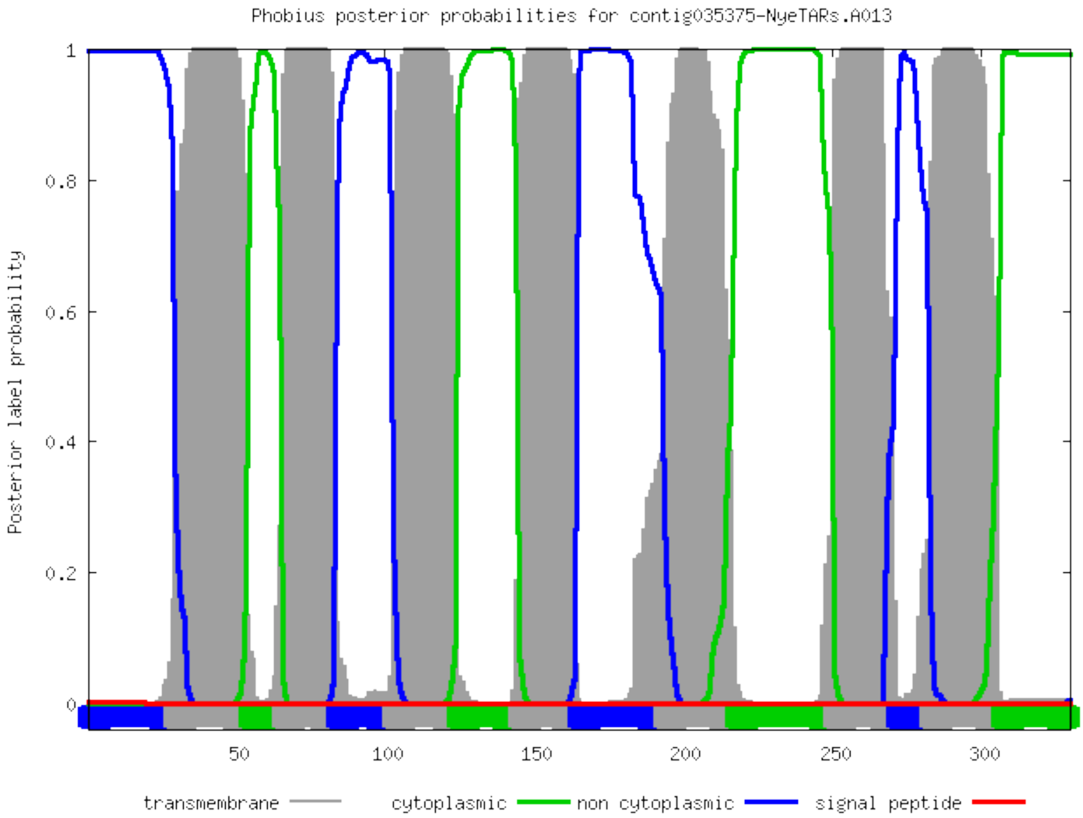

The probability data used in the plot is found here, and the gnuplot script is here.

Prediction of contig035376-NyeTARs.A014\

```
ID   contig035376-NyeTARs.A014\
FT   TOPO_DOM      1     29       NON CYTOPLASMIC.
FT   TRANSMEM     30     54
FT   TOPO_DOM     55     65       CYTOPLASMIC.
FT   TRANSMEM     66     83
FT   TOPO_DOM     84    102       NON CYTOPLASMIC.
FT   TRANSMEM    103    124
FT   TOPO_DOM    125    144       CYTOPLASMIC.
FT   TRANSMEM    145    164
FT   TOPO_DOM    165    183       NON CYTOPLASMIC.
FT   TRANSMEM    184    214
FT   TOPO_DOM    215    250       CYTOPLASMIC.
FT   TRANSMEM    251    271
FT   TOPO_DOM    272    282       NON CYTOPLASMIC.
FT   TRANSMEM    283    306
FT   TOPO_DOM    307    330       CYTOPLASMIC.
//
```

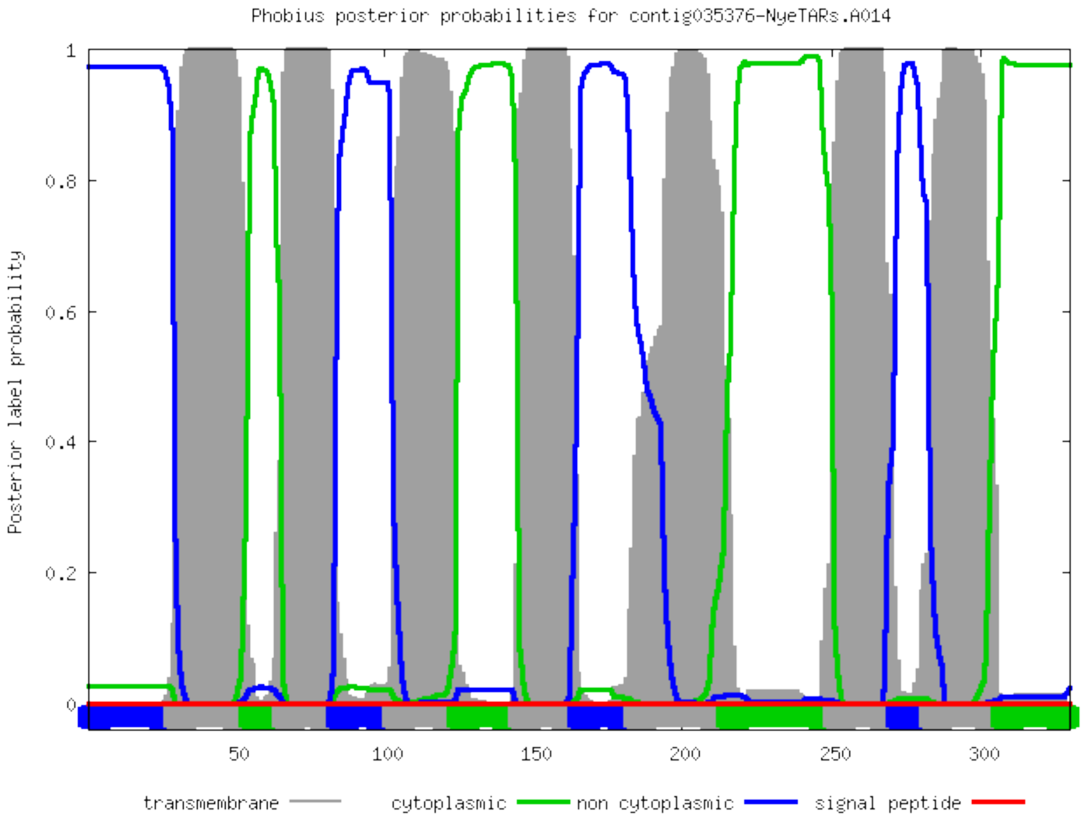

The probability data used in the plot is found here, and the gnuplot script is here.

Prediction of contig035381-NyeTARs.A015\

```
ID    contig035381-NyeTARs.A015\
FT    TOPO_DOM      1     29       NON CYTOPLASMIC.
FT    TRANSMEM     30     54
FT    TOPO_DOM     55     65       CYTOPLASMIC.
FT    TRANSMEM     66     83
FT    TOPO_DOM     84    102       NON CYTOPLASMIC.
FT    TRANSMEM    103    124
FT    TOPO_DOM    125    144       CYTOPLASMIC.
FT    TRANSMEM    145    164
FT    TOPO_DOM    165    183       NON CYTOPLASMIC.
FT    TRANSMEM    184    214
FT    TOPO_DOM    215    250       CYTOPLASMIC.
FT    TRANSMEM    251    271
FT    TOPO_DOM    272    282       NON CYTOPLASMIC.
FT    TRANSMEM    283    306
FT    TOPO_DOM    307    330       CYTOPLASMIC.
//
```

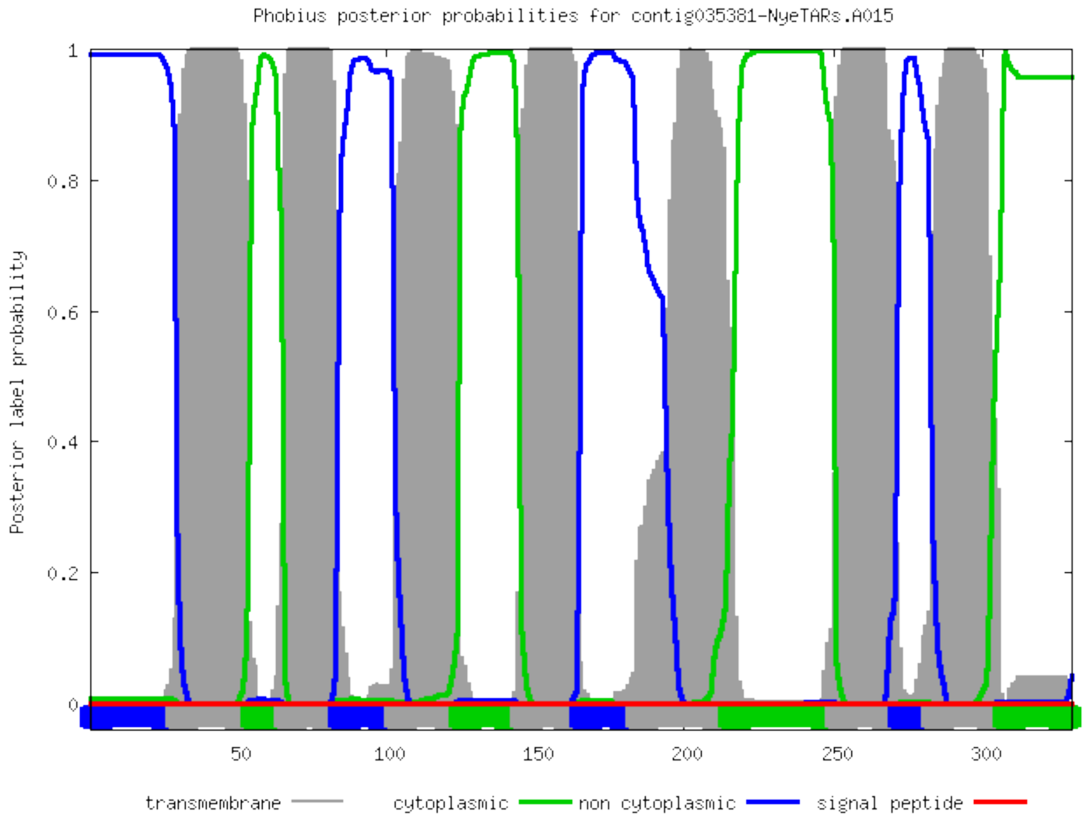

The probability data used in the plot is found here, and the gnuplot script is here.

Prediction of contig046011-NyeTARs.A016\

```
ID   contig046011-NyeTARs.A016\
FT   TOPO_DOM      1     29       CYTOPLASMIC.
FT   TRANSMEM     30     53
FT   TOPO_DOM     54     93       NON CYTOPLASMIC.
FT   TRANSMEM     94    115
FT   TOPO_DOM    116    135       CYTOPLASMIC.
FT   TRANSMEM    136    155
FT   TOPO_DOM    156    182       NON CYTOPLASMIC.
FT   TRANSMEM    183    206
FT   TOPO_DOM    207    238       CYTOPLASMIC.
FT   TRANSMEM    239    259
FT   TOPO_DOM    260    264       NON CYTOPLASMIC.
FT   TRANSMEM    265    288
FT   TOPO_DOM    289    306       CYTOPLASMIC.
//
```

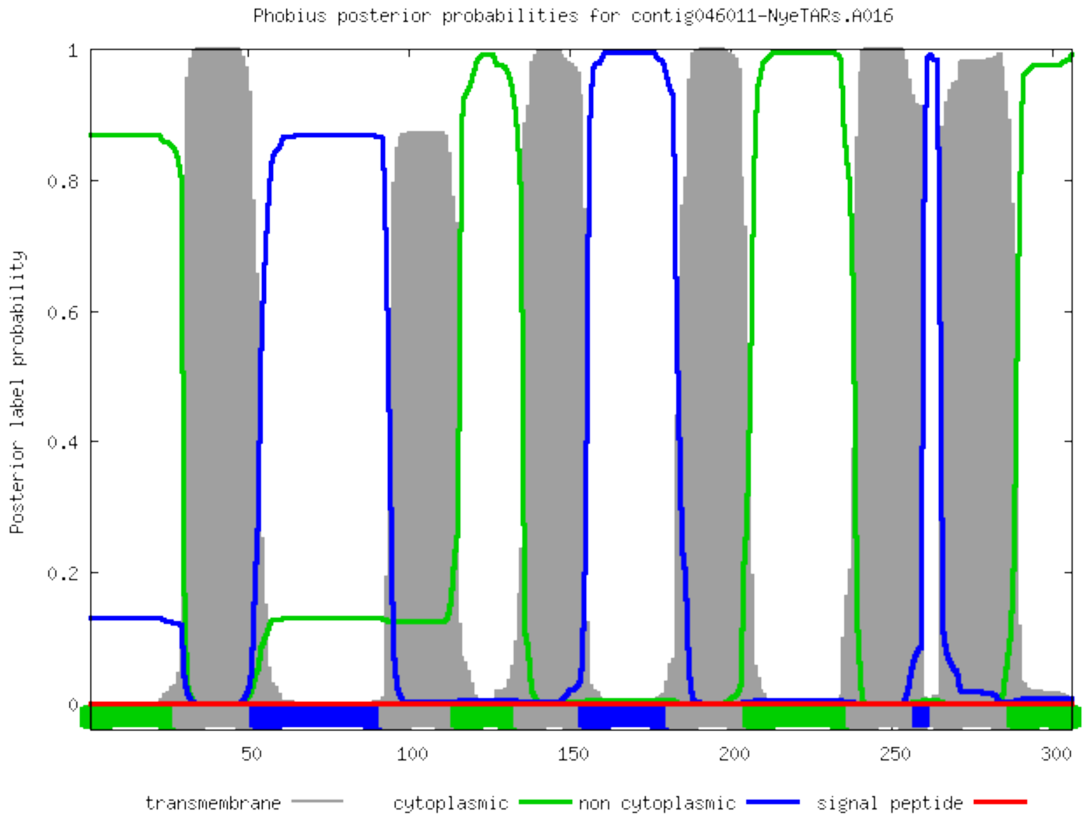

The probability data used in the plot is found here, and the gnuplot script is here.

Prediction of contig046007-NyeTARs.A017\

```
ID    contig046007-NyeTARs.A017\
FT    TOPO_DOM      1      27       NON CYTOPLASMIC.
FT    TRANSMEM     28      52
FT    TOPO_DOM     53      63       CYTOPLASMIC.
FT    TRANSMEM     64      86
FT    TOPO_DOM     87     100       NON CYTOPLASMIC.
FT    TRANSMEM    101     122
FT    TOPO_DOM    123     142       CYTOPLASMIC.
FT    TRANSMEM    143     162
FT    TOPO_DOM    163     191       NON CYTOPLASMIC.
FT    TRANSMEM    192     212
FT    TOPO_DOM    213     247       CYTOPLASMIC.
FT    TRANSMEM    248     265
FT    TOPO_DOM    266     276       NON CYTOPLASMIC.
FT    TRANSMEM    277     299
FT    TOPO_DOM    300     327       CYTOPLASMIC.
//
```

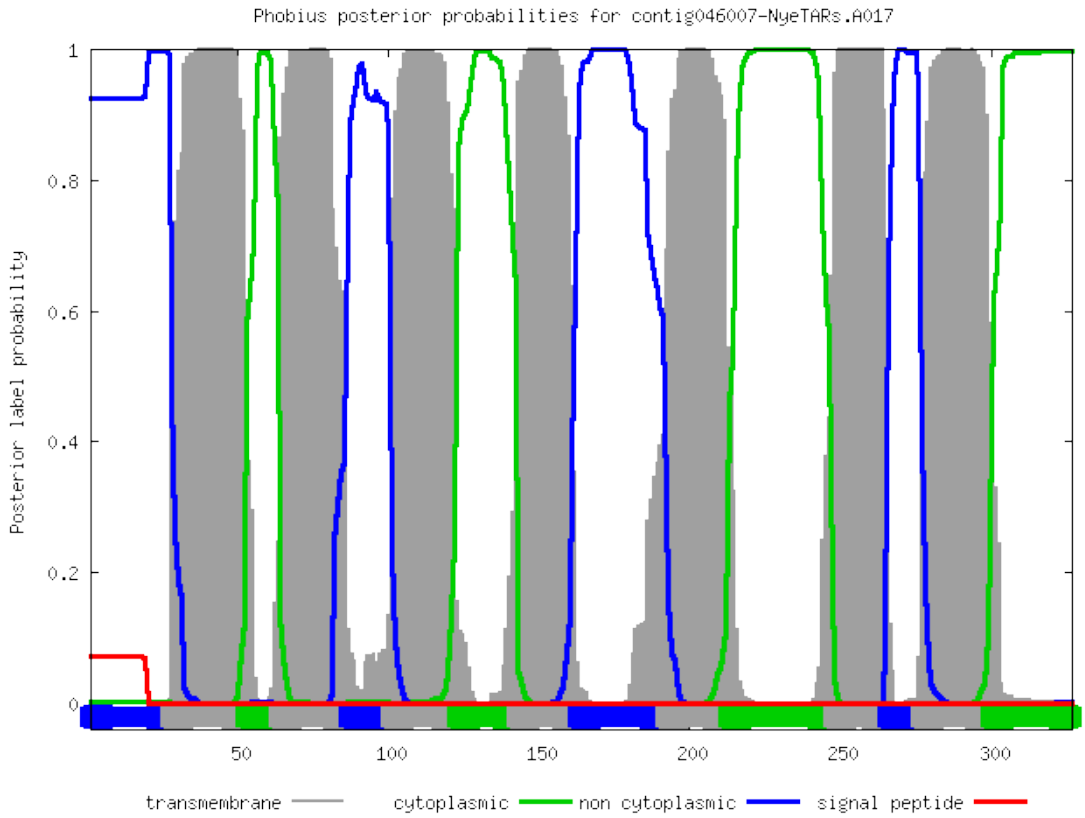

The probability data used in the plot is found here, and the gnuplot script is here.

Prediction of contig060105-NyeTARs.A018\

```
ID   contig060105-NyeTARs.A018\
FT   TOPO_DOM      1     26       NON CYTOPLASMIC.
FT   TRANSMEM     27     51
FT   TOPO_DOM     52     62       CYTOPLASMIC.
FT   TRANSMEM     63     87
FT   TOPO_DOM     88     98       NON CYTOPLASMIC.
FT   TRANSMEM     99    121
FT   TOPO_DOM    122    141       CYTOPLASMIC.
FT   TRANSMEM    142    161
FT   TOPO_DOM    162    180       NON CYTOPLASMIC.
FT   TRANSMEM    181    214
FT   TOPO_DOM    215    247       CYTOPLASMIC.
FT   TRANSMEM    248    269
FT   TOPO_DOM    270    283       NON CYTOPLASMIC.
FT   TRANSMEM    284    305
FT   TOPO_DOM    306    328       CYTOPLASMIC.
//
```

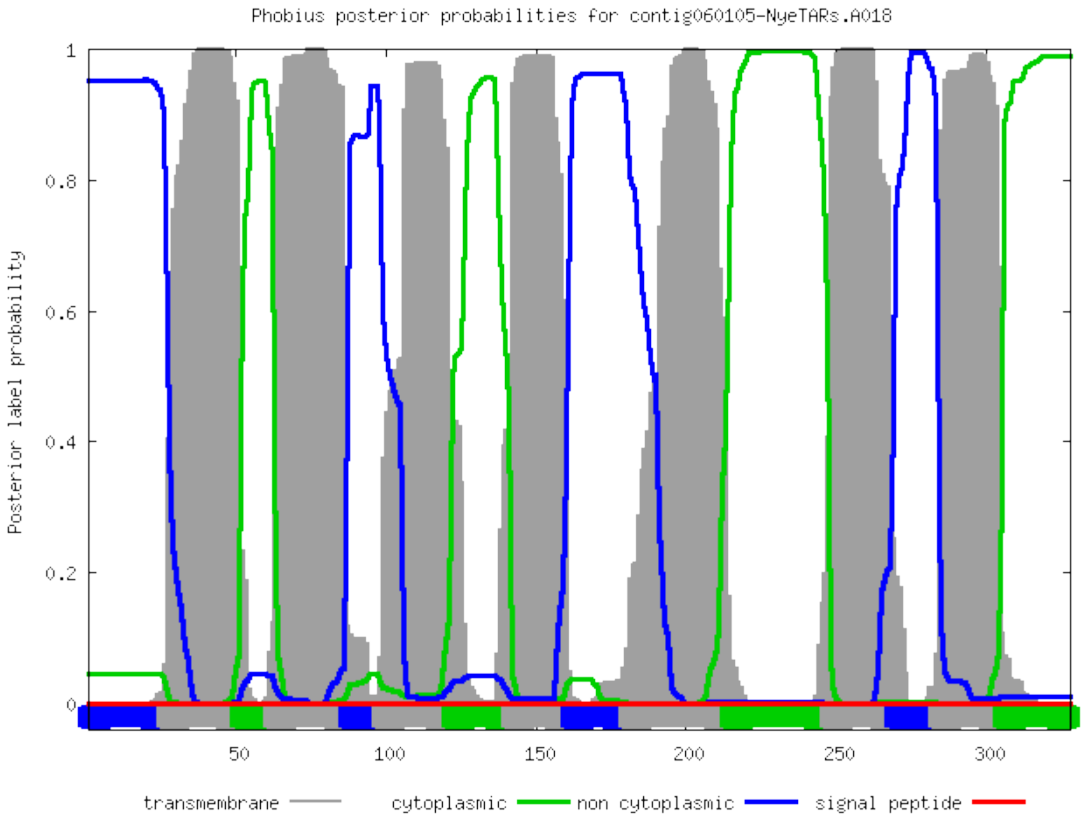

The probability data used in the plot is found here, and the gnuplot script is here.

Prediction of contig046010-NyeTARs.A019\

```
ID    contig046010-NyeTARs.A019\
FT    TOPO_DOM       1     27       NON CYTOPLASMIC.
FT    TRANSMEM      28     52
FT    TOPO_DOM      53     63       CYTOPLASMIC.
FT    TRANSMEM      64     81
FT    TOPO_DOM      82    100       NON CYTOPLASMIC.
FT    TRANSMEM     101    122
FT    TOPO_DOM     123    142       CYTOPLASMIC.
FT    TRANSMEM     143    162
FT    TOPO_DOM     163    191       NON CYTOPLASMIC.
FT    TRANSMEM     192    215
FT    TOPO_DOM     216    247       CYTOPLASMIC.
FT    TRANSMEM     248    268
FT    TOPO_DOM     269    279       NON CYTOPLASMIC.
FT    TRANSMEM     280    303
FT    TOPO_DOM     304    327       CYTOPLASMIC.
//
```

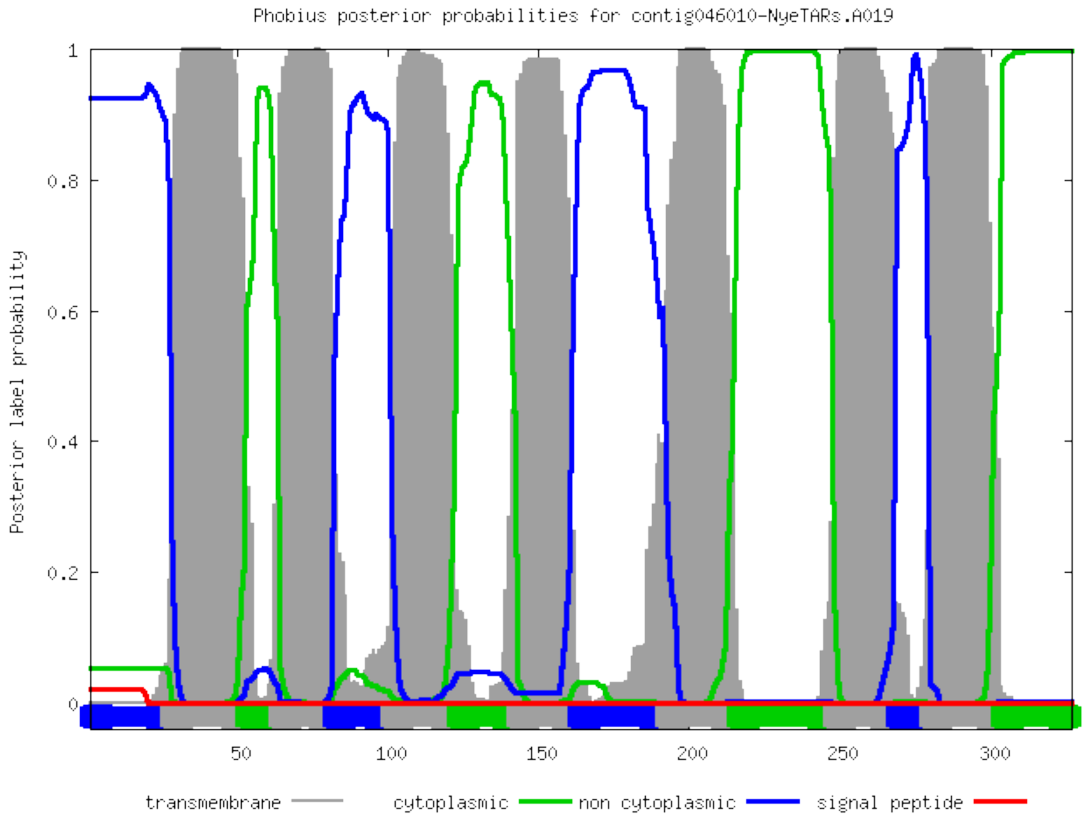

The probability data used in the plot is found here, and the gnuplot script is here.

Prediction of contig046013-NyeTARs.A021\

```
ID    contig046013-NyeTARs.A021\
FT    TOPO_DOM      1     29       NON CYTOPLASMIC.
FT    TRANSMEM     30     54
FT    TOPO_DOM     55     65       CYTOPLASMIC.
FT    TRANSMEM     66     83
FT    TOPO_DOM     84    102       NON CYTOPLASMIC.
FT    TRANSMEM    103    124
FT    TOPO_DOM    125    135       CYTOPLASMIC.
FT    TRANSMEM    136    158
FT    TOPO_DOM    159    193       NON CYTOPLASMIC.
FT    TRANSMEM    194    217
FT    TOPO_DOM    218    249       CYTOPLASMIC.
FT    TRANSMEM    250    270
FT    TOPO_DOM    271    281       NON CYTOPLASMIC.
FT    TRANSMEM    282    305
FT    TOPO_DOM    306    329       CYTOPLASMIC.
//
```

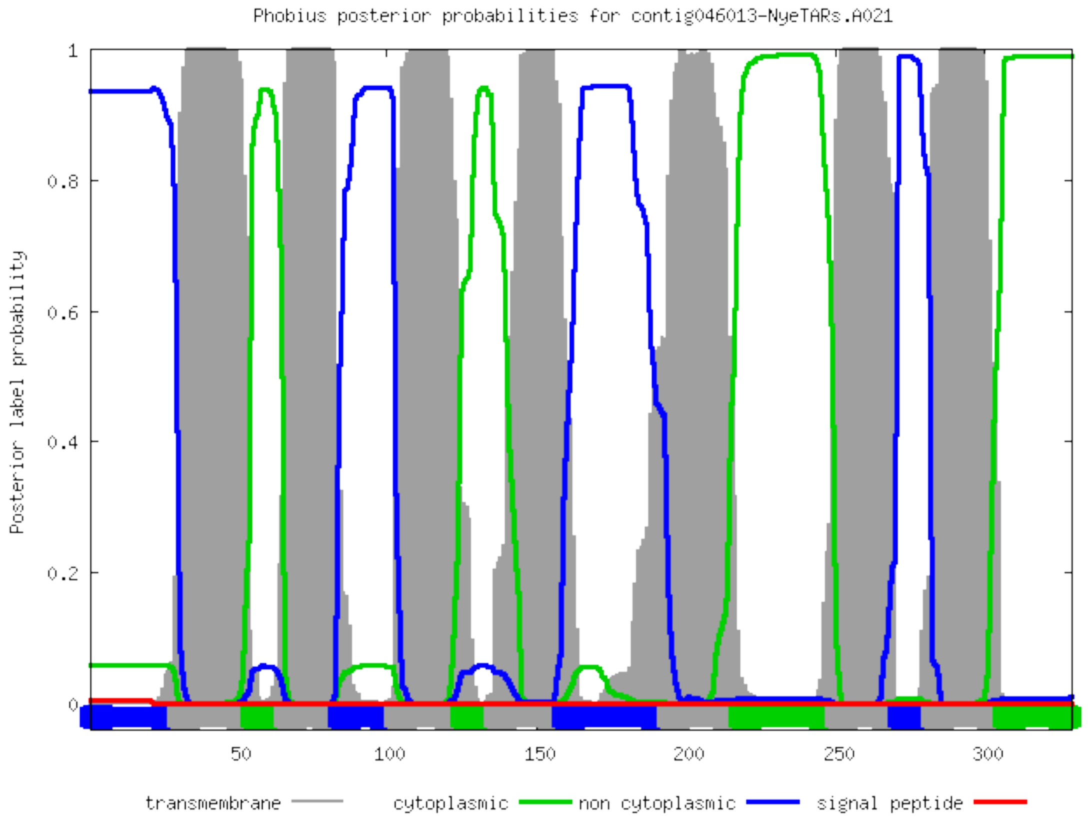

The probability data used in the plot is found here, and the gnuplot script is here.

Prediction of contig062039-NyeTARs.A022\

```
ID    contig062039-NyeTARs.A022\
FT    TOPO_DOM      1     30       NON CYTOPLASMIC.
FT    TRANSMEM     31     53
FT    TOPO_DOM     54     64       CYTOPLASMIC.
FT    TRANSMEM     65     96
FT    TOPO_DOM     97    101       NON CYTOPLASMIC.
FT    TRANSMEM    102    123
FT    TOPO_DOM    124    143       CYTOPLASMIC.
FT    TRANSMEM    144    163
FT    TOPO_DOM    164    182       NON CYTOPLASMIC.
FT    TRANSMEM    183    216
FT    TOPO_DOM    217    242       CYTOPLASMIC.
FT    TRANSMEM    243    263
FT    TOPO_DOM    264    274       NON CYTOPLASMIC.
FT    TRANSMEM    275    298
FT    TOPO_DOM    299    322       CYTOPLASMIC.
//
```

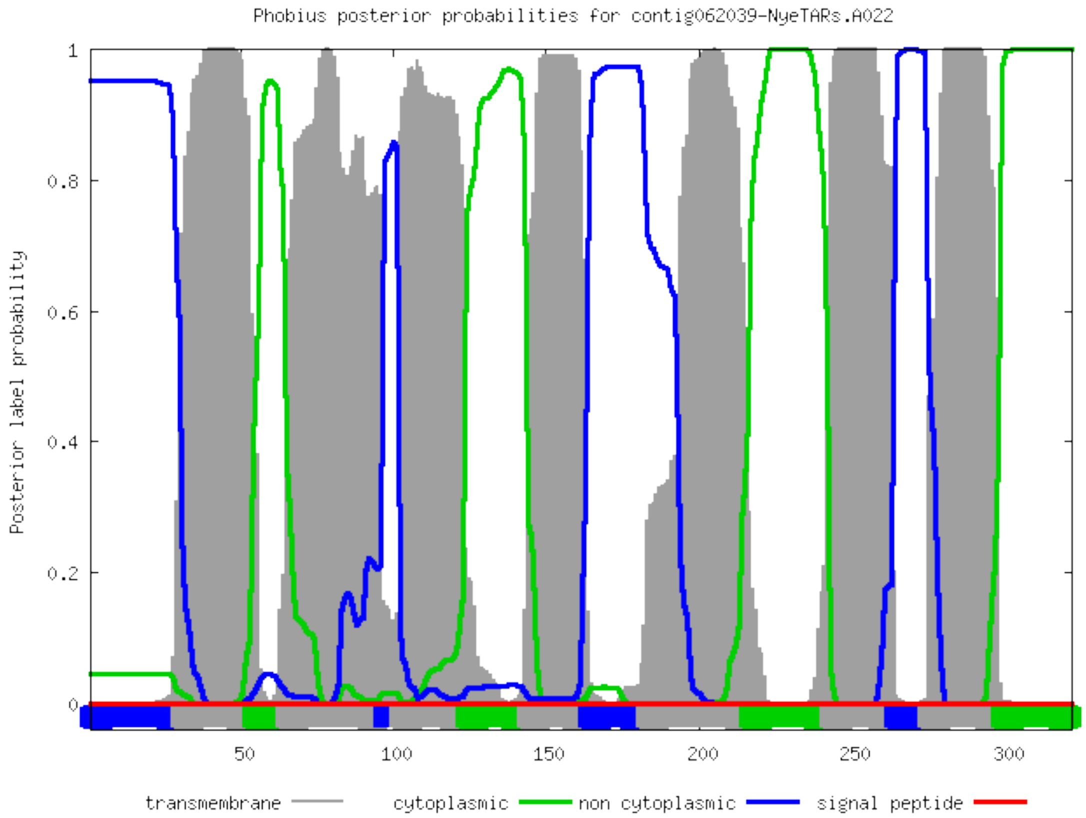

The probability data used in the plot is found here, and the gnuplot script is here.

Prediction of contig035377-NyeTARs.A023\

```
ID   contig035377-NyeTARs.A023\
FT   TOPO_DOM      1     29       NON CYTOPLASMIC.
FT   TRANSMEM     30     54
FT   TOPO_DOM     55     65       CYTOPLASMIC.
FT   TRANSMEM     66     88
FT   TOPO_DOM     89     93       NON CYTOPLASMIC.
FT   TRANSMEM     94    124
FT   TOPO_DOM    125    144       CYTOPLASMIC.
FT   TRANSMEM    145    164
FT   TOPO_DOM    165    193       NON CYTOPLASMIC.
FT   TRANSMEM    194    217
FT   TOPO_DOM    218    250       CYTOPLASMIC.
FT   TRANSMEM    251    271
FT   TOPO_DOM    272    282       NON CYTOPLASMIC.
FT   TRANSMEM    283    306
FT   TOPO_DOM    307    330       CYTOPLASMIC.
//
```

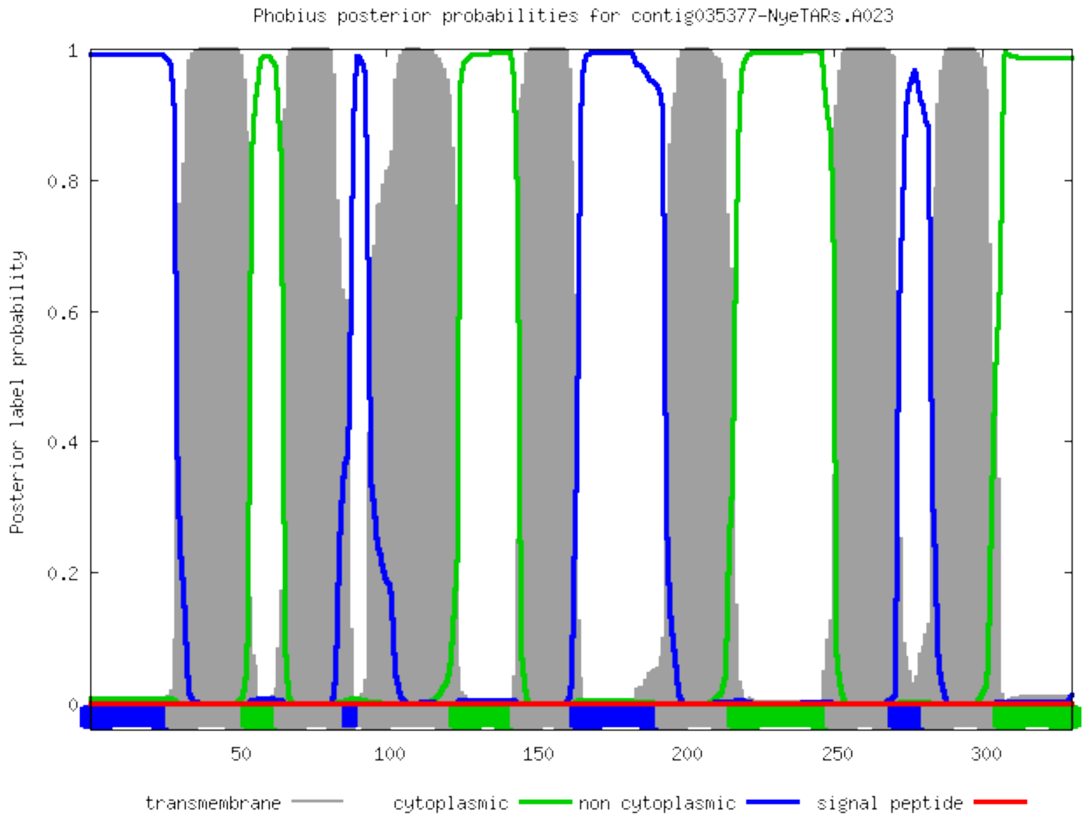

The probability data used in the plot is found here, and the gnuplot script is here.

Prediction of contig046014-NyeTARs.A024\

```
ID    contig046014-NyeTARs.A024\
FT    TOPO_DOM      1      29       NON CYTOPLASMIC.
FT    TRANSMEM     30      54
FT    TOPO_DOM     55      65       CYTOPLASMIC.
FT    TRANSMEM     66      83
FT    TOPO_DOM     84     102       NON CYTOPLASMIC.
FT    TRANSMEM    103     124
FT    TOPO_DOM    125     144       CYTOPLASMIC.
FT    TRANSMEM    145     163
FT    TOPO_DOM    164     193       NON CYTOPLASMIC.
FT    TRANSMEM    194     217
FT    TOPO_DOM    218     249       CYTOPLASMIC.
FT    TRANSMEM    250     270
FT    TOPO_DOM    271     281       NON CYTOPLASMIC.
FT    TRANSMEM    282     301
FT    TOPO_DOM    302     329       CYTOPLASMIC.
//
```

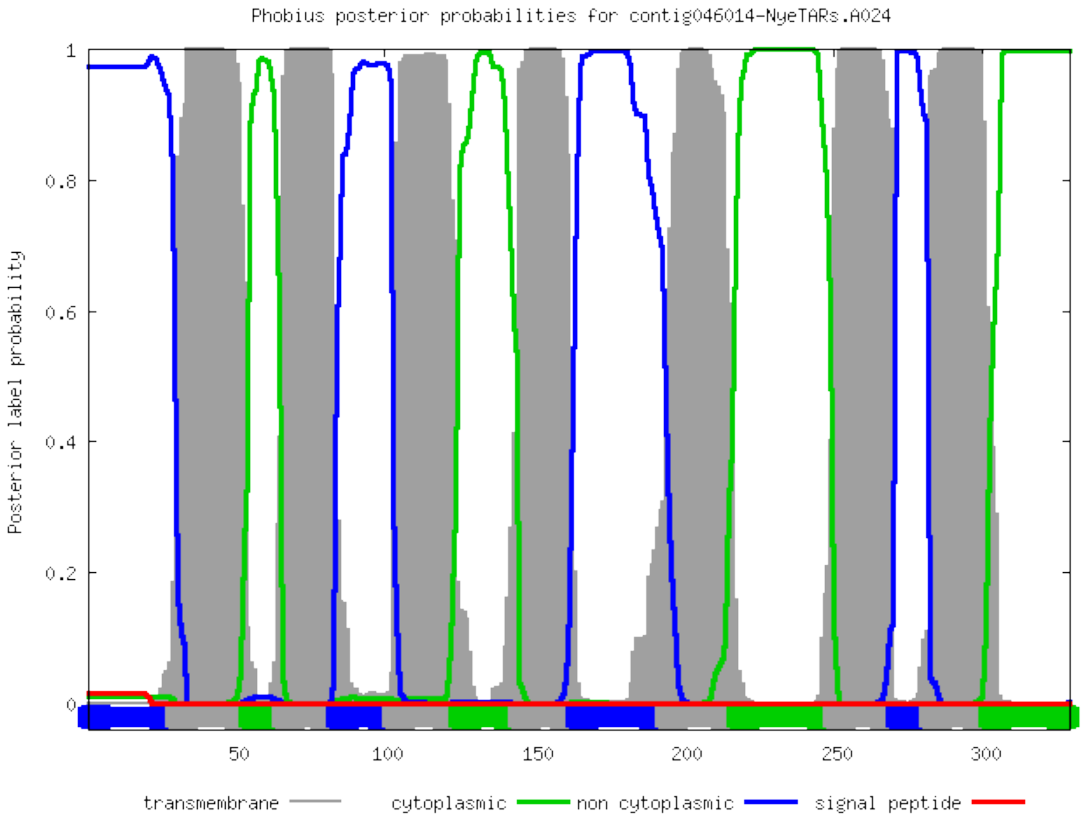

The probability data used in the plot is found here, and the gnuplot script is here.

Prediction of contig058002-NyeTARs.A025\

```
ID    contig058002-NyeTARs.A025\
FT    TOPO_DOM       1      29       CYTOPLASMIC.
FT    TRANSMEM      30      54
FT    TOPO_DOM      55      65       NON CYTOPLASMIC.
FT    TRANSMEM      66      89
FT    TOPO_DOM      90     100       CYTOPLASMIC.
FT    TRANSMEM     101     123
FT    TOPO_DOM     124     192       NON CYTOPLASMIC.
FT    TRANSMEM     193     215
FT    TOPO_DOM     216     249       CYTOPLASMIC.
FT    TRANSMEM     250     270
FT    TOPO_DOM     271     281       NON CYTOPLASMIC.
FT    TRANSMEM     282     302
FT    TOPO_DOM     303     333       CYTOPLASMIC.
//
```

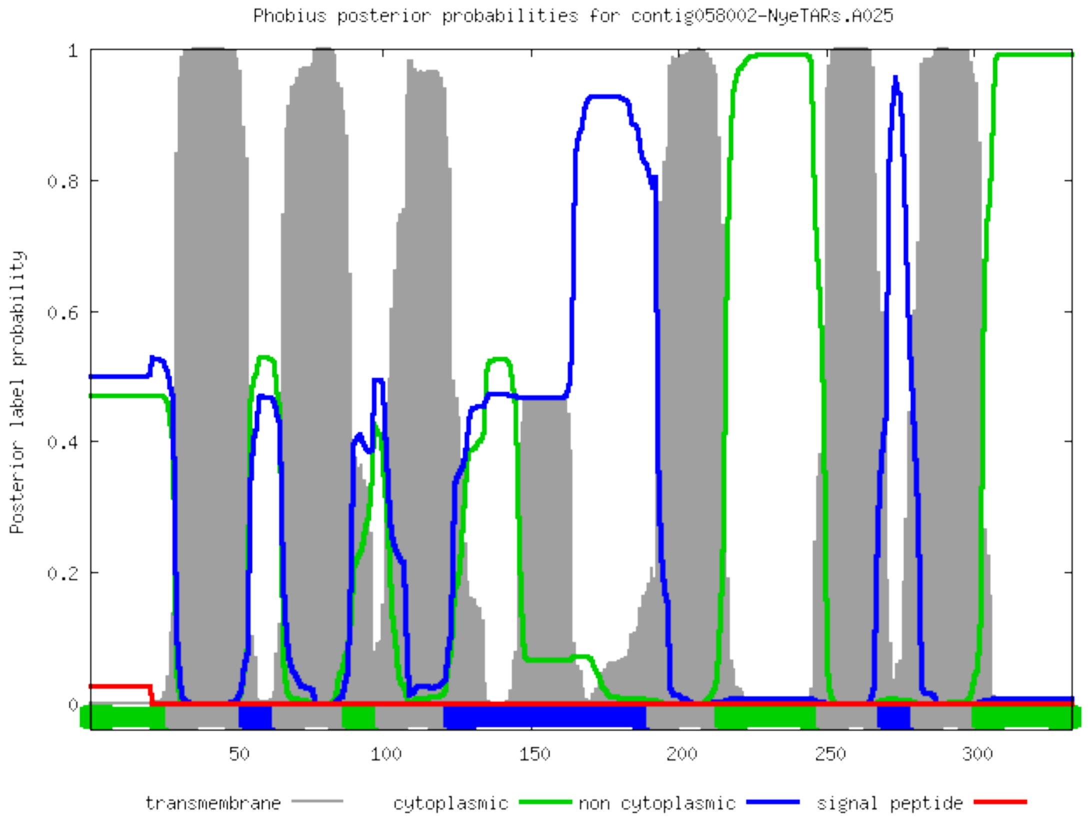

The probability data used in the plot is found here, and the gnuplot script is here.

Prediction of contig045999-NyeTARs.A026\

```
ID   contig045999-NyeTARs.A026\
FT   TOPO_DOM      1     31      NON CYTOPLASMIC.
FT   TRANSMEM     32     56
FT   TOPO_DOM     57     67      CYTOPLASMIC.
FT   TRANSMEM     68     85
FT   TOPO_DOM     86    104      NON CYTOPLASMIC.
FT   TRANSMEM    105    126
FT   TOPO_DOM    127    146      CYTOPLASMIC.
FT   TRANSMEM    147    165
FT   TOPO_DOM    166    195      NON CYTOPLASMIC.
FT   TRANSMEM    196    219
FT   TOPO_DOM    220    251      CYTOPLASMIC.
FT   TRANSMEM    252    272
FT   TOPO_DOM    273    283      NON CYTOPLASMIC.
FT   TRANSMEM    284    307
FT   TOPO_DOM    308    333      CYTOPLASMIC.
//
```

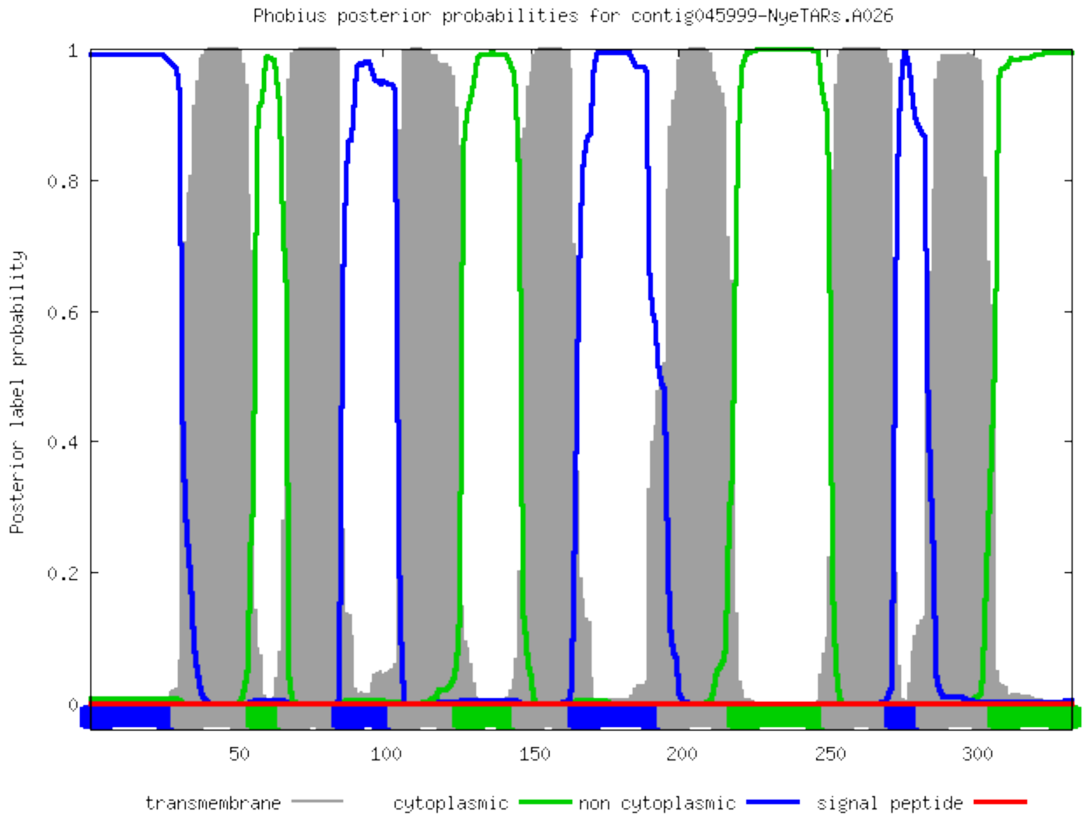

The probability data used in the plot is found here, and the gnuplot script is here.

Prediction of contig060292-NyeTARs.A027\

```
ID   contig060292-NyeTARs.A027\
FT   TOPO_DOM      1     25       NON CYTOPLASMIC.
FT   TRANSMEM     26     51
FT   TOPO_DOM     52     62       CYTOPLASMIC.
FT   TRANSMEM     63     85
FT   TOPO_DOM     86     96       NON CYTOPLASMIC.
FT   TRANSMEM     97    119
FT   TOPO_DOM    120    139       CYTOPLASMIC.
FT   TRANSMEM    140    159
FT   TOPO_DOM    160    188       NON CYTOPLASMIC.
FT   TRANSMEM    189    213
FT   TOPO_DOM    214    239       CYTOPLASMIC.
FT   TRANSMEM    240    260
FT   TOPO_DOM    261    271       NON CYTOPLASMIC.
FT   TRANSMEM    272    296
FT   TOPO_DOM    297    324       CYTOPLASMIC.
//
```

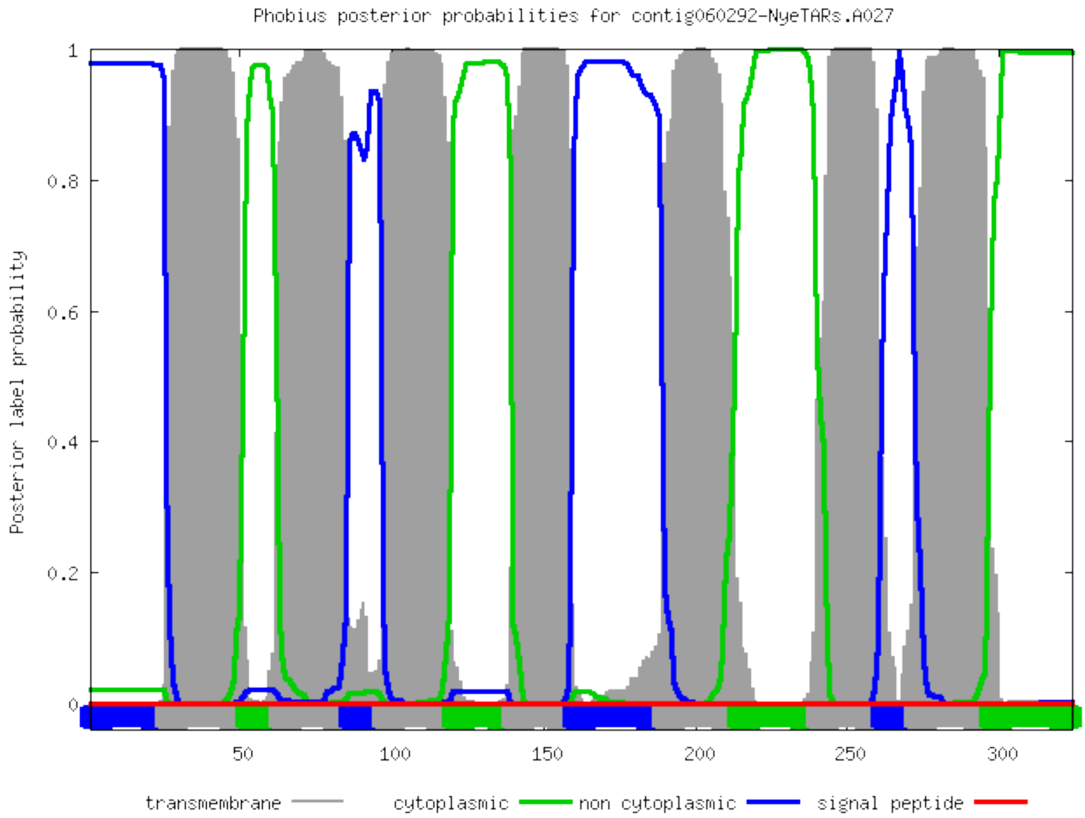

The probability data used in the plot is found here, and the gnuplot script is here.

Prediction of contig042499-NyeTARs.A028\

```
ID    contig042499-NyeTARs.A028\
FT    TOPO_DOM       1      27       NON CYTOPLASMIC.
FT    TRANSMEM      28      51
FT    TOPO_DOM      52      62       CYTOPLASMIC.
FT    TRANSMEM      63      86
FT    TOPO_DOM      87      97       NON CYTOPLASMIC.
FT    TRANSMEM      98     120
FT    TOPO_DOM     121     140       CYTOPLASMIC.
FT    TRANSMEM     141     160
FT    TOPO_DOM     161     189       NON CYTOPLASMIC.
FT    TRANSMEM     190     213
FT    TOPO_DOM     214     246       CYTOPLASMIC.
FT    TRANSMEM     247     267
FT    TOPO_DOM     268     278       NON CYTOPLASMIC.
FT    TRANSMEM     279     299
FT    TOPO_DOM     300     331       CYTOPLASMIC.
//
```

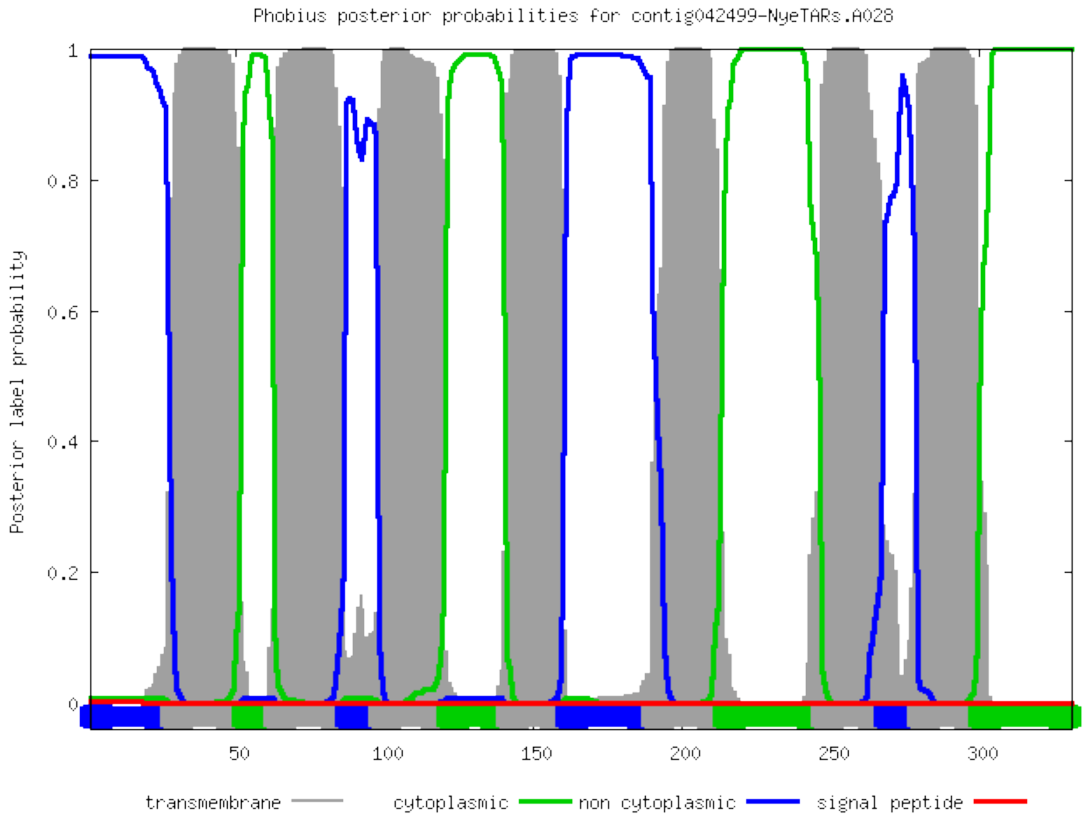

The probability data used in the plot is found here, and the gnuplot script is here.

Prediction of contig056200-NyeTARs.A029\

```
ID   contig056200-NyeTARs.A029\
FT   TOPO_DOM      1     29       NON CYTOPLASMIC.
FT   TRANSMEM     30     54
FT   TOPO_DOM     55     65       CYTOPLASMIC.
FT   TRANSMEM     66     82
FT   TOPO_DOM     83    101       NON CYTOPLASMIC.
FT   TRANSMEM    102    124
FT   TOPO_DOM    125    144       CYTOPLASMIC.
FT   TRANSMEM    145    165
FT   TOPO_DOM    166    184       NON CYTOPLASMIC.
FT   TRANSMEM    185    214
FT   TOPO_DOM    215    249       CYTOPLASMIC.
FT   TRANSMEM    250    270
FT   TOPO_DOM    271    281       NON CYTOPLASMIC.
FT   TRANSMEM    282    301
FT   TOPO_DOM    302    329       CYTOPLASMIC.
//
```

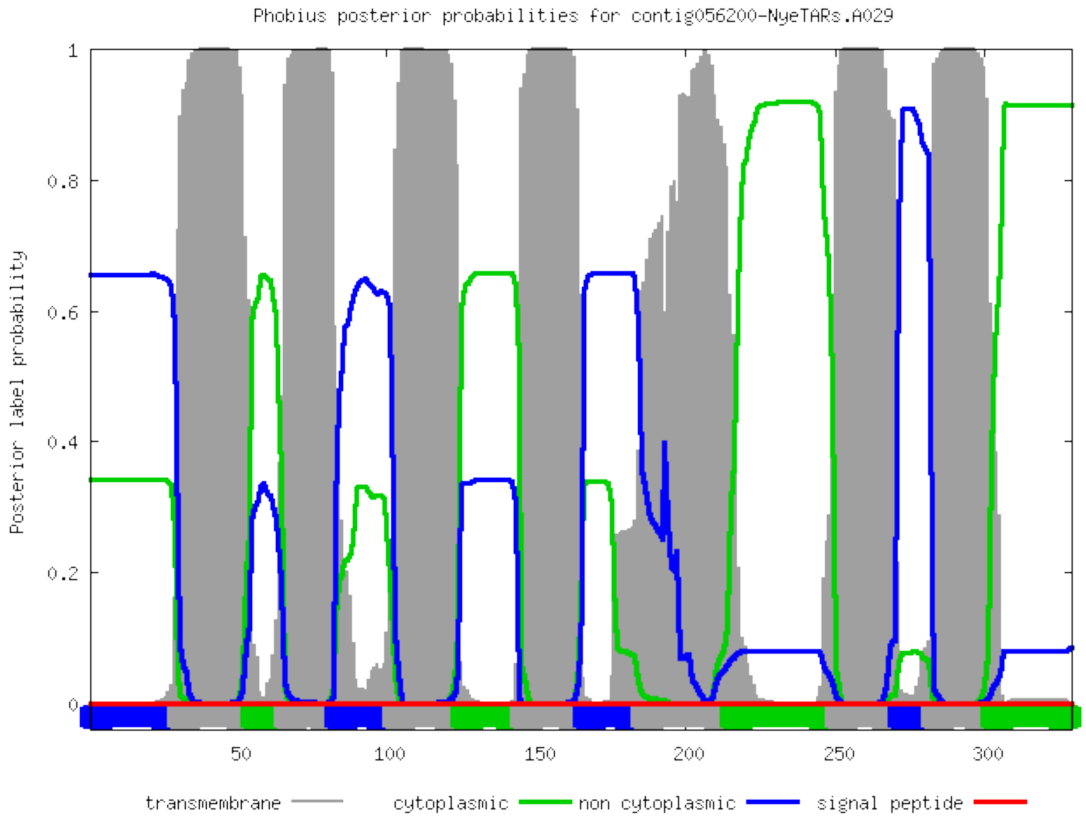

The probability data used in the plot is found here, and the gnuplot script is here.

Prediction of contig045302-BurTAR.A001\

```
ID    contig045302-BurTAR.A001\
FT    TOPO_DOM       1      28       NON CYTOPLASMIC.
FT    TRANSMEM      29      53
FT    TOPO_DOM      54      64       CYTOPLASMIC.
FT    TRANSMEM      65      90
FT    TOPO_DOM      91     105       NON CYTOPLASMIC.
FT    TRANSMEM     106     124
FT    TOPO_DOM     125     144       CYTOPLASMIC.
FT    TRANSMEM     145     166
FT    TOPO_DOM     167     193       NON CYTOPLASMIC.
FT    TRANSMEM     194     217
FT    TOPO_DOM     218     244       CYTOPLASMIC.
FT    TRANSMEM     245     262
FT    TOPO_DOM     263     273       NON CYTOPLASMIC.
FT    TRANSMEM     274     298
FT    TOPO_DOM     299     326       CYTOPLASMIC.
//
```

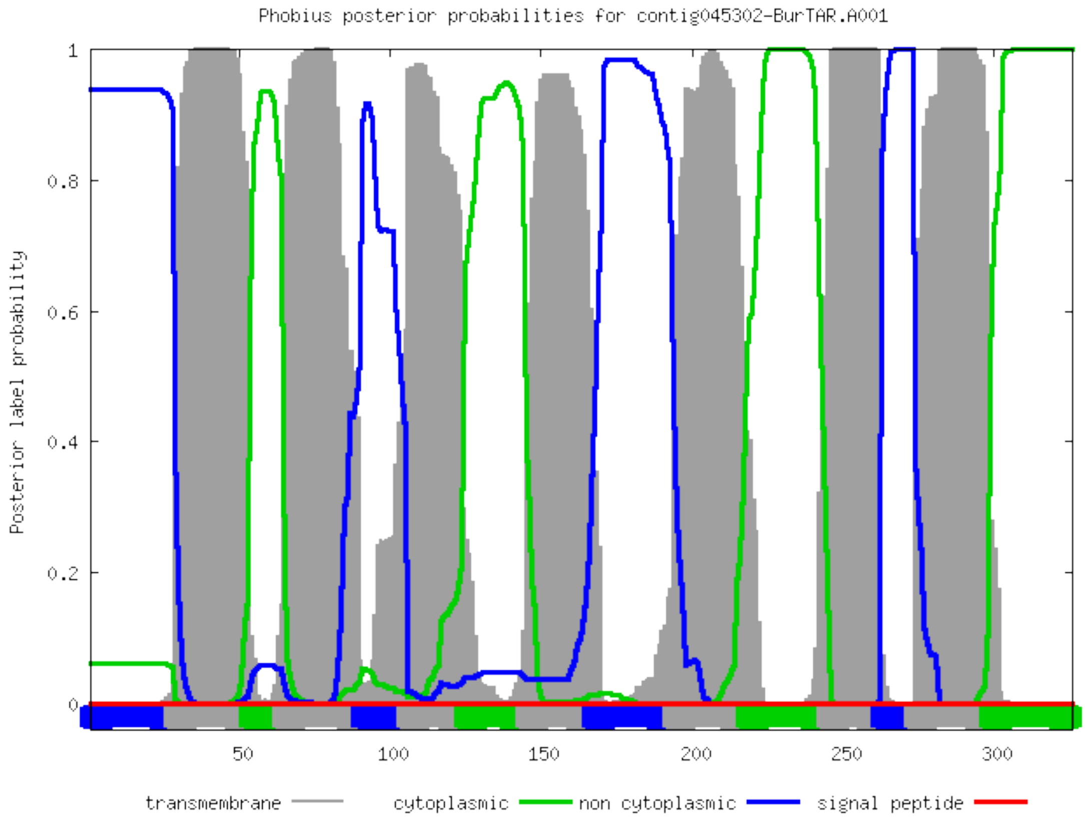

The probability data used in the plot is found here, and the gnuplot script is here.

Prediction of contig020038-BurTAR.A002\

```
ID   contig020038-BurTAR.A002\
FT   TOPO_DOM      1     29       NON CYTOPLASMIC.
FT   TRANSMEM     30     54
FT   TOPO_DOM     55     65       CYTOPLASMIC.
FT   TRANSMEM     66     83
FT   TOPO_DOM     84    102       NON CYTOPLASMIC.
FT   TRANSMEM    103    125
FT   TOPO_DOM    126    145       CYTOPLASMIC.
FT   TRANSMEM    146    165
FT   TOPO_DOM    166    194       NON CYTOPLASMIC.
FT   TRANSMEM    195    218
FT   TOPO_DOM    219    246       CYTOPLASMIC.
FT   TRANSMEM    247    267
FT   TOPO_DOM    268    286       NON CYTOPLASMIC.
FT   TRANSMEM    287    307
FT   TOPO_DOM    308    331       CYTOPLASMIC.
//
```

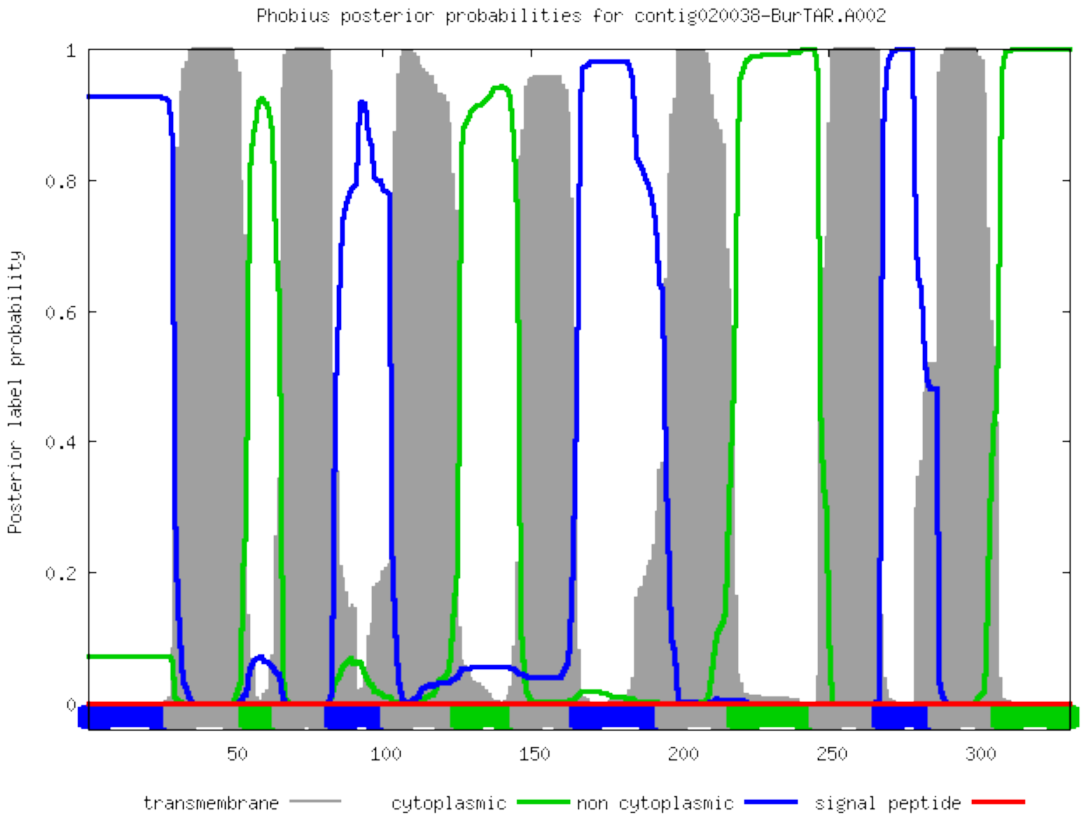

The probability data used in the plot is found here, and the gnuplot script is here.

Prediction of contig006087-BurTAR.B032\

```
ID   contig006087-BurTAR.B032\
FT   TOPO_DOM      1     24       NON CYTOPLASMIC.
FT   TRANSMEM     25     52
FT   TOPO_DOM     53     60       CYTOPLASMIC.
FT   TRANSMEM     61     85
FT   TOPO_DOM     86    104       NON CYTOPLASMIC.
FT   TRANSMEM    105    125
FT   TOPO_DOM    126    145       CYTOPLASMIC.
FT   TRANSMEM    146    166
FT   TOPO_DOM    167    171       NON CYTOPLASMIC.
FT   TRANSMEM    172    196
FT   TOPO_DOM    197    233       CYTOPLASMIC.
FT   TRANSMEM    234    257
FT   TOPO_DOM    258    268       NON CYTOPLASMIC.
FT   TRANSMEM    269    291
FT   TOPO_DOM    292    315       CYTOPLASMIC.
//
```

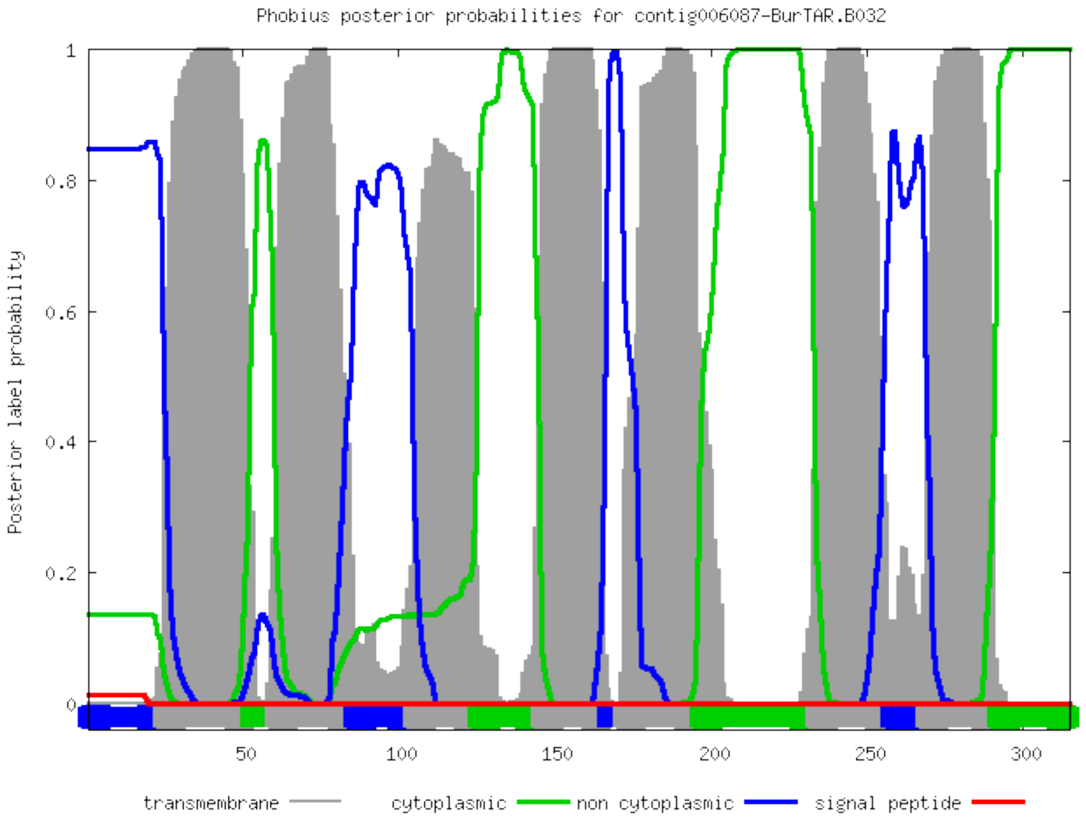

The probability data used in the plot is found here, and the gnuplot script is here.

Prediction of contig061977-BurTARs.A012\

```
ID    contig061977-BurTARs.A012\
FT    TOPO_DOM      1     26       NON CYTOPLASMIC.
FT    TRANSMEM     27     51
FT    TOPO_DOM     52     62       CYTOPLASMIC.
FT    TRANSMEM     63     80
FT    TOPO_DOM     81     99       NON CYTOPLASMIC.
FT    TRANSMEM    100    121
FT    TOPO_DOM    122    141       CYTOPLASMIC.
FT    TRANSMEM    142    161
FT    TOPO_DOM    162    190       NON CYTOPLASMIC.
FT    TRANSMEM    191    214
FT    TOPO_DOM    215    247       CYTOPLASMIC.
FT    TRANSMEM    248    268
FT    TOPO_DOM    269    279       NON CYTOPLASMIC.
FT    TRANSMEM    280    303
FT    TOPO_DOM    304    327       CYTOPLASMIC.
//
```

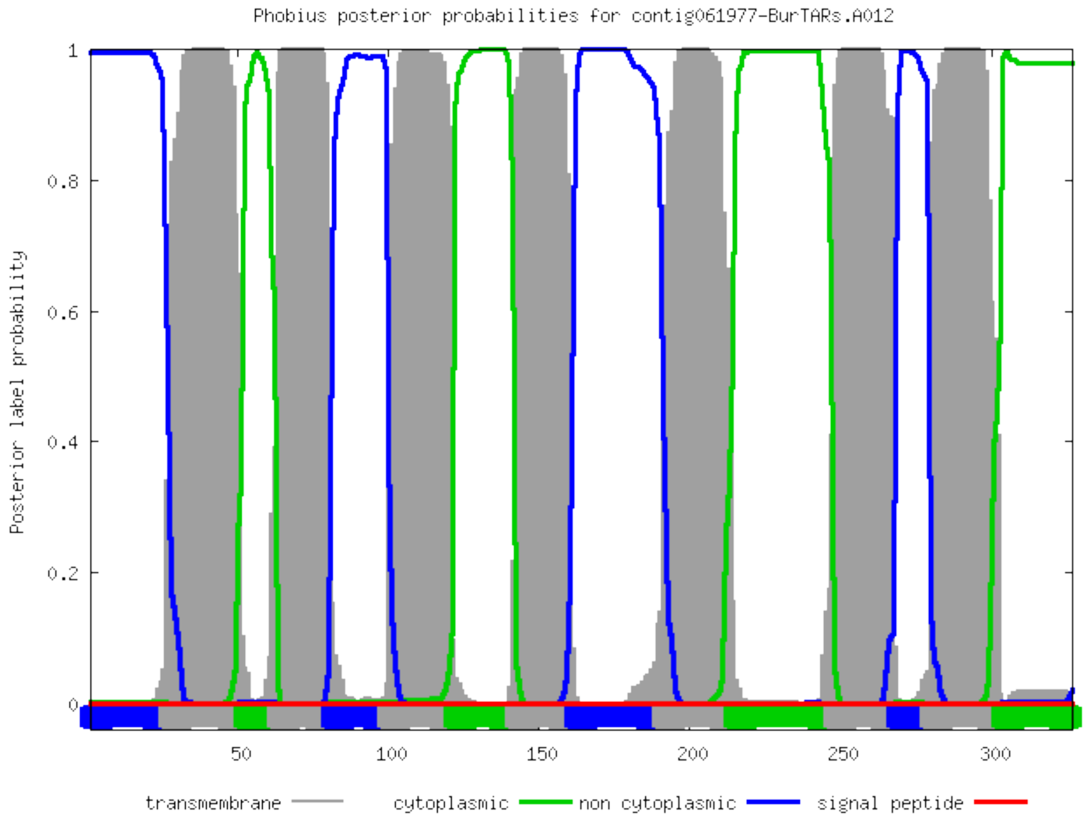

The probability data used in the plot is found here, and the gnuplot script is here.

Prediction of contig061433-BurTARs.A013\

```
ID    contig061433-BurTARs.A013\
FT    TOPO_DOM      1      31       NON CYTOPLASMIC.
FT    TRANSMEM     32      56
FT    TOPO_DOM     57      67       CYTOPLASMIC.
FT    TRANSMEM     68      85
FT    TOPO_DOM     86     104       NON CYTOPLASMIC.
FT    TRANSMEM    105     126
FT    TOPO_DOM    127     146       CYTOPLASMIC.
FT    TRANSMEM    147     165
FT    TOPO_DOM    166     193       NON CYTOPLASMIC.
FT    TRANSMEM    194     216
FT    TOPO_DOM    217     251       CYTOPLASMIC.
FT    TRANSMEM    252     272
FT    TOPO_DOM    273     283       NON CYTOPLASMIC.
FT    TRANSMEM    284     306
FT    TOPO_DOM    307     330       CYTOPLASMIC.
//
```

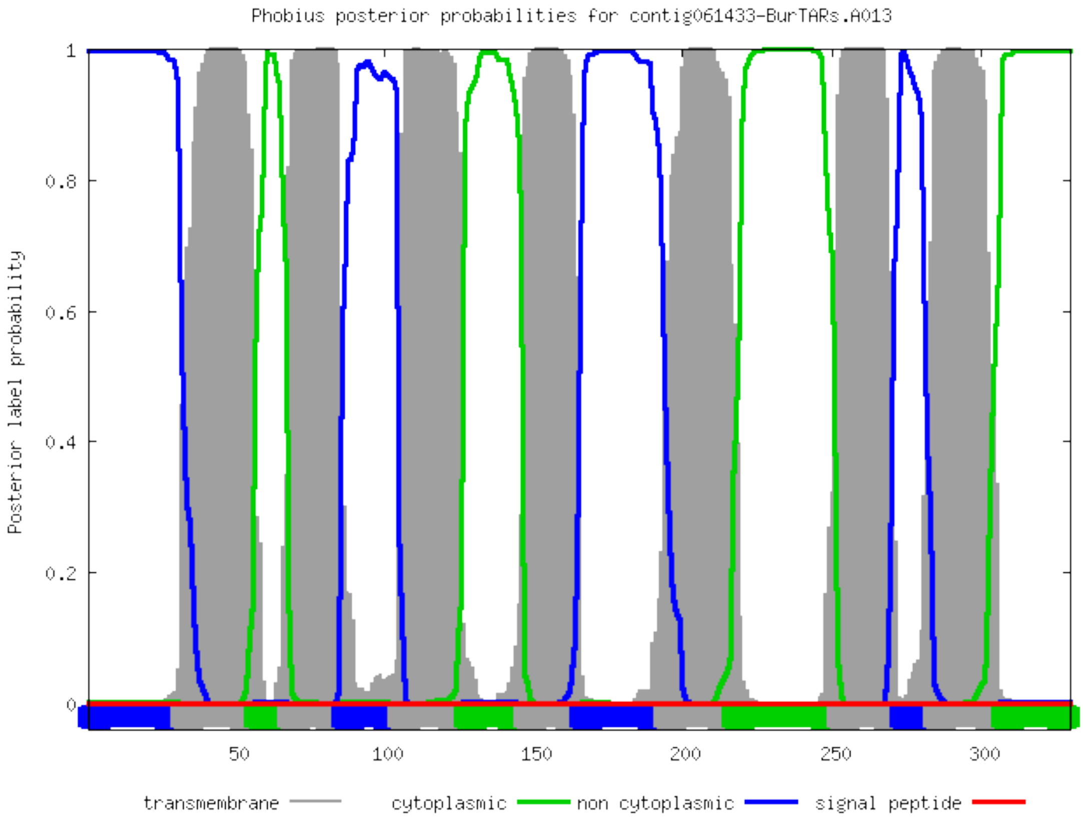

The probability data used in the plot is found here, and the gnuplot script is here.

Prediction of contig061091-BurTARs.A014\

```
ID   contig061091-BurTARs.A014\
FT   TOPO_DOM      1     29       NON CYTOPLASMIC.
FT   TRANSMEM     30     54
FT   TOPO_DOM     55     65       CYTOPLASMIC.
FT   TRANSMEM     66     83
FT   TOPO_DOM     84    102       NON CYTOPLASMIC.
FT   TRANSMEM    103    124
FT   TOPO_DOM    125    144       CYTOPLASMIC.
FT   TRANSMEM    145    163
FT   TOPO_DOM    164    182       NON CYTOPLASMIC.
FT   TRANSMEM    183    214
FT   TOPO_DOM    215    249       CYTOPLASMIC.
FT   TRANSMEM    250    270
FT   TOPO_DOM    271    281       NON CYTOPLASMIC.
FT   TRANSMEM    282    305
FT   TOPO_DOM    306    329       CYTOPLASMIC.
//
```

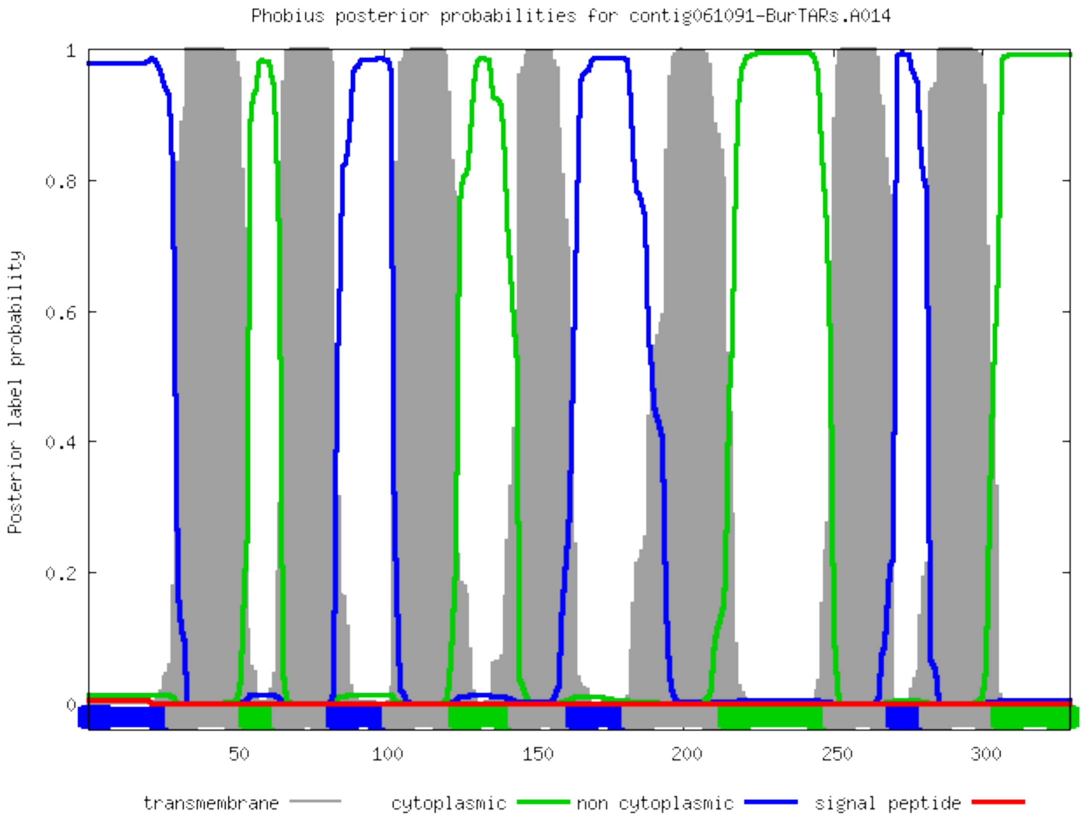

The probability data used in the plot is found here, and the gnuplot script is here.

Prediction of contig060707-BurTARs.A015\

```
ID   contig060707-BurTARs.A015\
FT   TOPO_DOM      1     26       NON CYTOPLASMIC.
FT   TRANSMEM     27     51
FT   TOPO_DOM     52     62       CYTOPLASMIC.
FT   TRANSMEM     63     86
FT   TOPO_DOM     87     97       NON CYTOPLASMIC.
FT   TRANSMEM     98    120
FT   TOPO_DOM    121    140       CYTOPLASMIC.
FT   TRANSMEM    141    160
FT   TOPO_DOM    161    179       NON CYTOPLASMIC.
FT   TRANSMEM    180    213
FT   TOPO_DOM    214    246       CYTOPLASMIC.
FT   TRANSMEM    247    268
FT   TOPO_DOM    269    282       NON CYTOPLASMIC.
FT   TRANSMEM    283    304
FT   TOPO_DOM    305    327       CYTOPLASMIC.
//
```

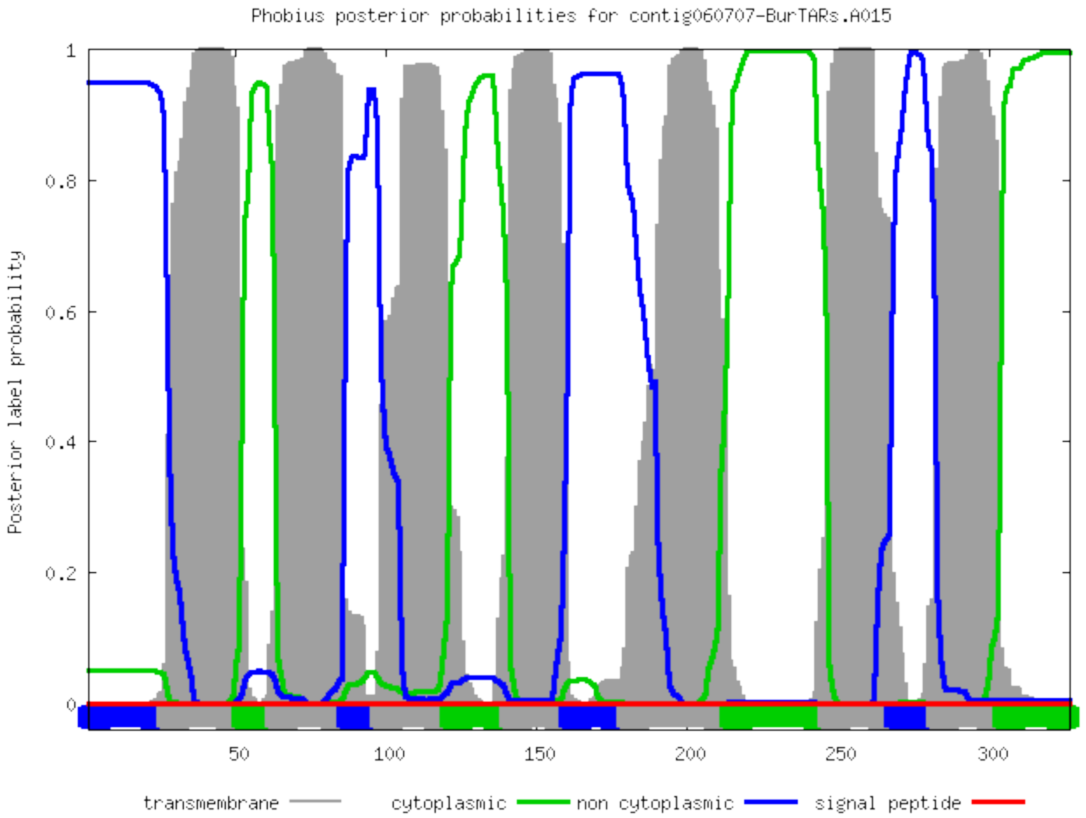

The probability data used in the plot is found here, and the gnuplot script is here.

Prediction of contig059673-BurTARs.A016\

```
ID   contig059673-BurTARs.A016\
FT   TOPO_DOM      1     25       NON CYTOPLASMIC.
FT   TRANSMEM     26     43
FT   TOPO_DOM     44     49       CYTOPLASMIC.
FT   TRANSMEM     50     73
FT   TOPO_DOM     74     92       NON CYTOPLASMIC.
FT   TRANSMEM     93    119
FT   TOPO_DOM    120    130       CYTOPLASMIC.
FT   TRANSMEM    131    149
FT   TOPO_DOM    150    177       NON CYTOPLASMIC.
FT   TRANSMEM    178    200
FT   TOPO_DOM    201    234       CYTOPLASMIC.
FT   TRANSMEM    235    254
FT   TOPO_DOM    255    265       NON CYTOPLASMIC.
FT   TRANSMEM    266    287
FT   TOPO_DOM    288    315       CYTOPLASMIC.
//
```

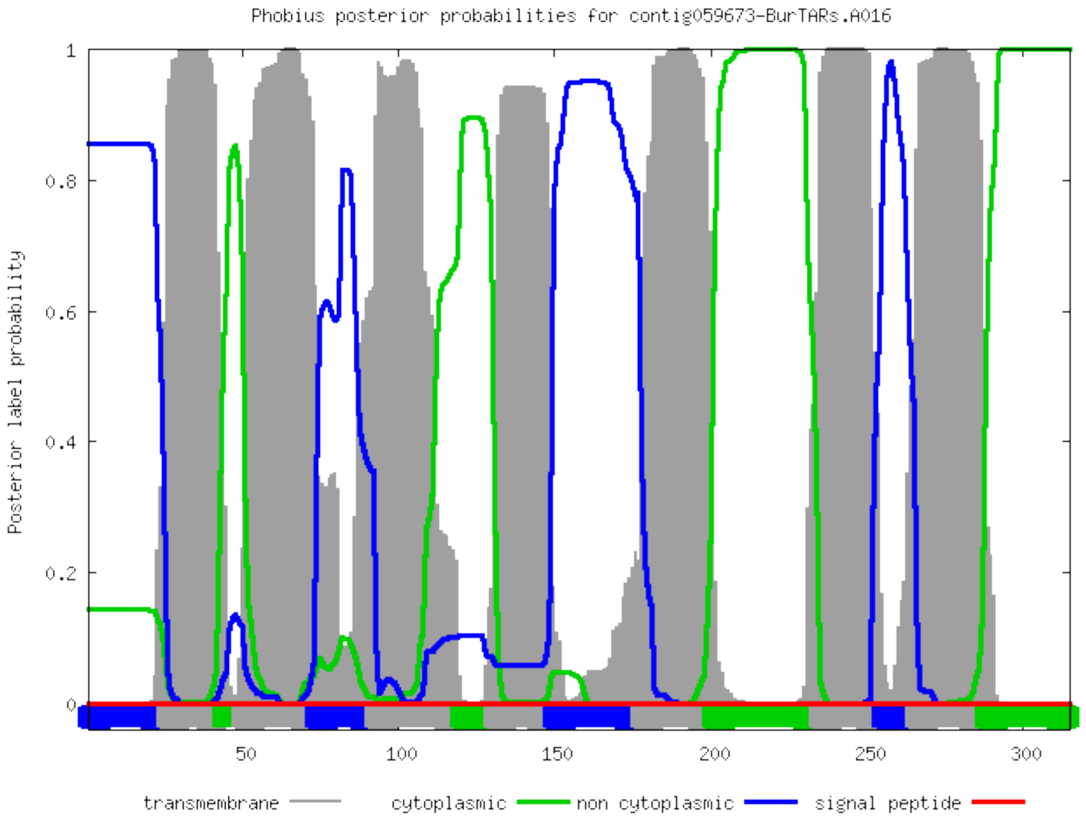

The probability data used in the plot is found here, and the gnuplot script is here.

Prediction of contig057301-BurTARs.A017\

```
ID    contig057301-BurTARs.A017\
FT    TOPO_DOM       1      27       NON CYTOPLASMIC.
FT    TRANSMEM      28      51
FT    TOPO_DOM      52      62       CYTOPLASMIC.
FT    TRANSMEM      63      86
FT    TOPO_DOM      87      97       NON CYTOPLASMIC.
FT    TRANSMEM      98     120
FT    TOPO_DOM     121     140       CYTOPLASMIC.
FT    TRANSMEM     141     160
FT    TOPO_DOM     161     193       NON CYTOPLASMIC.
FT    TRANSMEM     194     213
FT    TOPO_DOM     214     246       CYTOPLASMIC.
FT    TRANSMEM     247     267
FT    TOPO_DOM     268     278       NON CYTOPLASMIC.
FT    TRANSMEM     279     299
FT    TOPO_DOM     300     330       CYTOPLASMIC.
//
```

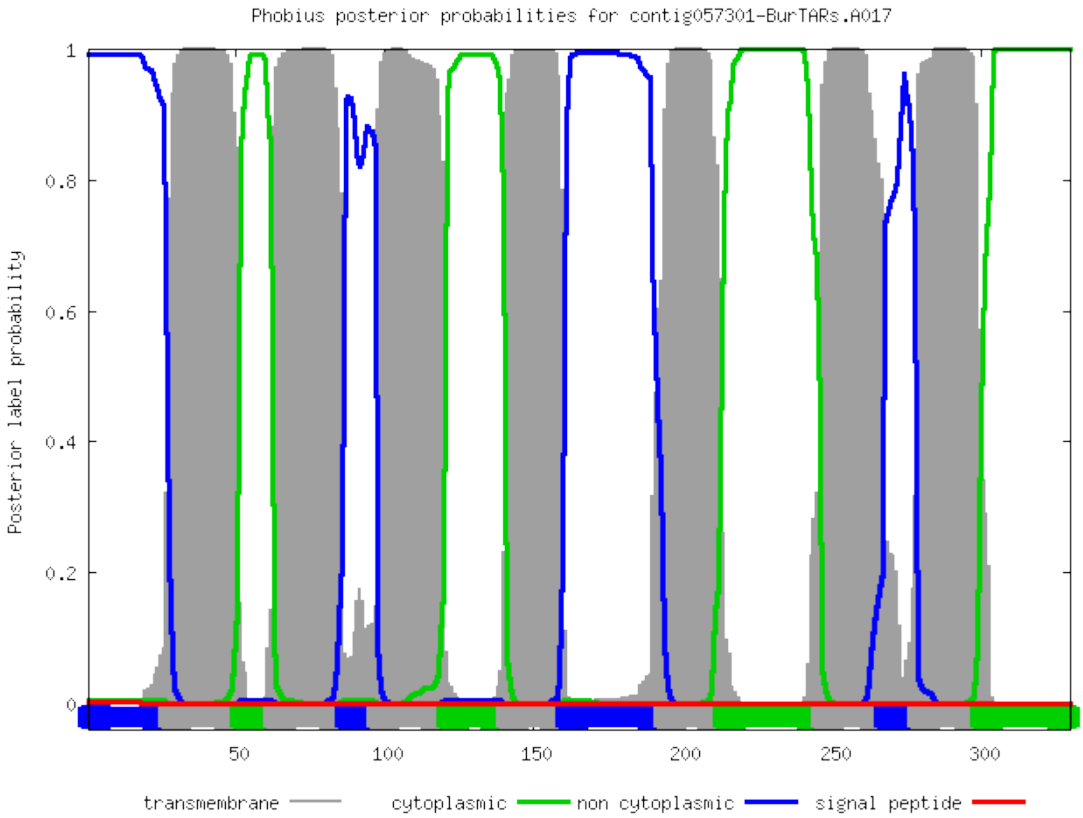

The probability data used in the plot is found here, and the gnuplot script is here.

Prediction of contig057145-BurTARs.A018\

```
ID    contig057145-BurTARs.A018\
FT    TOPO_DOM      1     29       NON CYTOPLASMIC.
FT    TRANSMEM     30     54
FT    TOPO_DOM     55     65       CYTOPLASMIC.
FT    TRANSMEM     66     83
FT    TOPO_DOM     84    102       NON CYTOPLASMIC.
FT    TRANSMEM    103    124
FT    TOPO_DOM    125    144       CYTOPLASMIC.
FT    TRANSMEM    145    164
FT    TOPO_DOM    165    187       NON CYTOPLASMIC.
FT    TRANSMEM    188    214
FT    TOPO_DOM    215    249       CYTOPLASMIC.
FT    TRANSMEM    250    270
FT    TOPO_DOM    271    281       NON CYTOPLASMIC.
FT    TRANSMEM    282    301
FT    TOPO_DOM    302    329       CYTOPLASMIC.
//
```

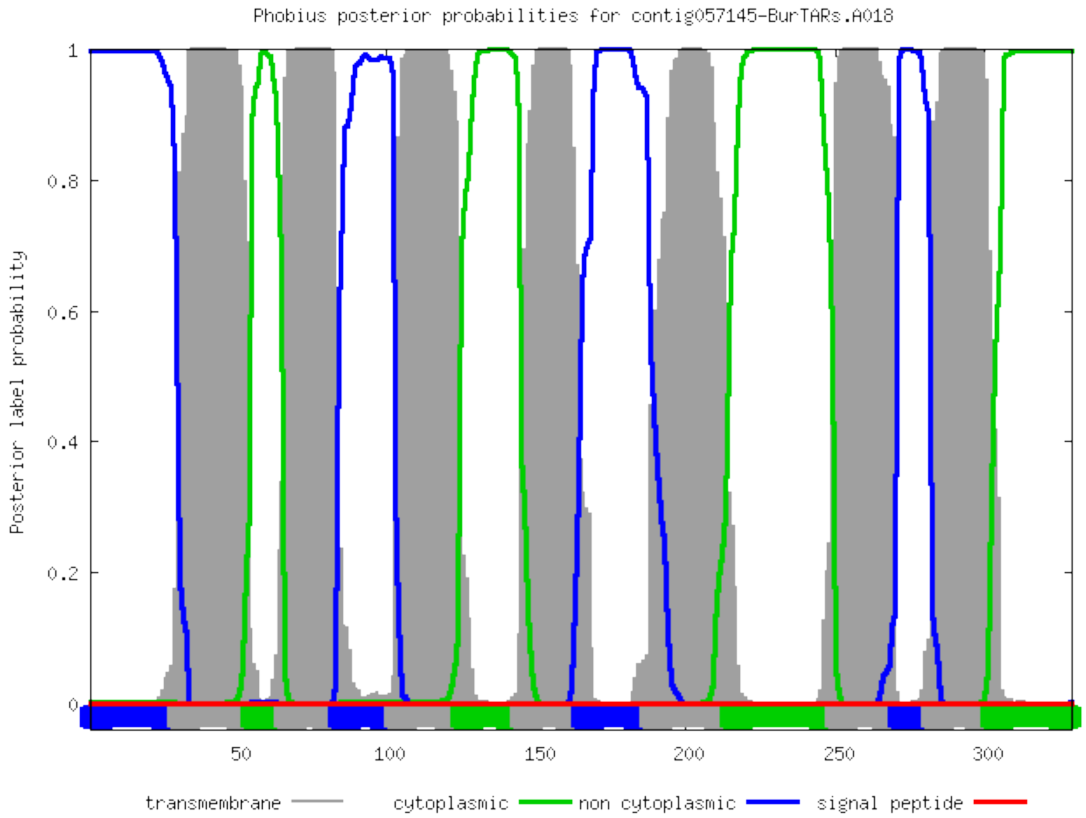

The probability data used in the plot is found here, and the gnuplot script is here.

Prediction of contig056023-BurTARs.A019\

```
ID   contig056023-BurTARs.A019\
FT   TOPO_DOM      1     31       NON CYTOPLASMIC.
FT   TRANSMEM     32     56
FT   TOPO_DOM     57     67       CYTOPLASMIC.
FT   TRANSMEM     68     85
FT   TOPO_DOM     86    104       NON CYTOPLASMIC.
FT   TRANSMEM    105    126
FT   TOPO_DOM    127    146       CYTOPLASMIC.
FT   TRANSMEM    147    165
FT   TOPO_DOM    166    195       NON CYTOPLASMIC.
FT   TRANSMEM    196    219
FT   TOPO_DOM    220    251       CYTOPLASMIC.
FT   TRANSMEM    252    272
FT   TOPO_DOM    273    283       NON CYTOPLASMIC.
FT   TRANSMEM    284    307
FT   TOPO_DOM    308    333       CYTOPLASMIC.
//
```

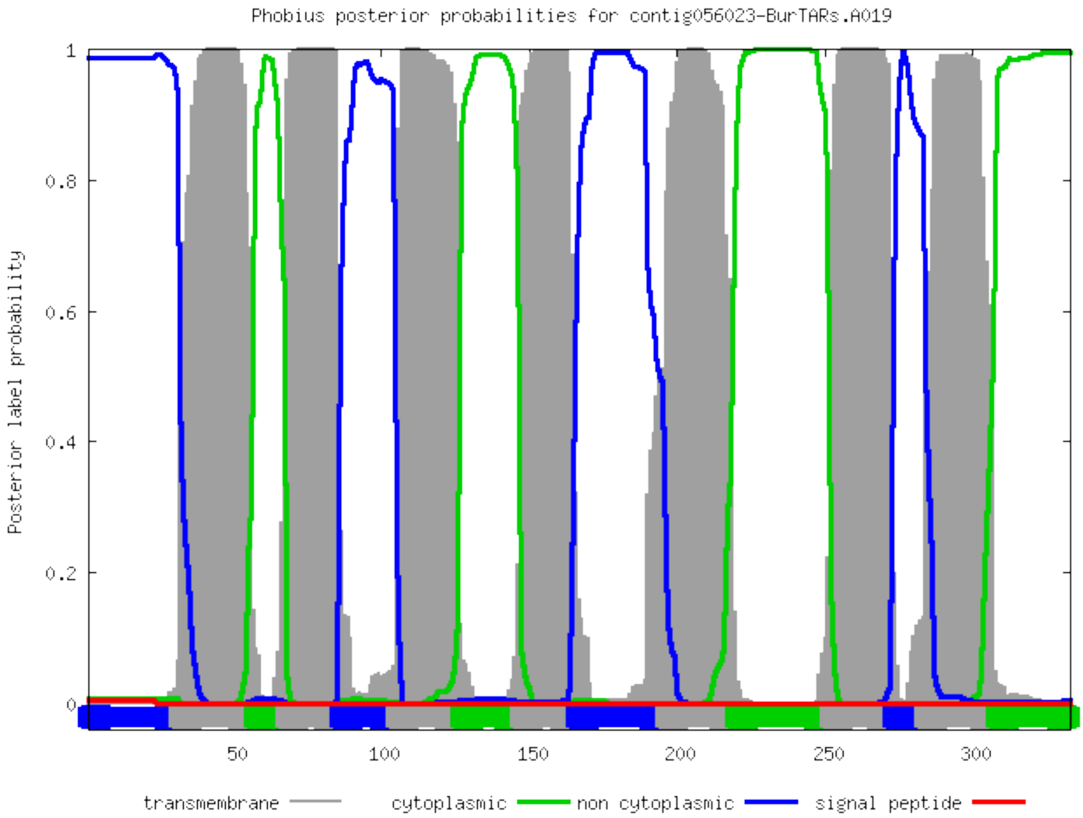

The probability data used in the plot is found here, and the gnuplot script is here.

Prediction of contig056021-BurTARs.A020\

```
ID    contig056021-BurTARs.A020\
FT    TOPO_DOM      1     29       NON CYTOPLASMIC.
FT    TRANSMEM     30     54
FT    TOPO_DOM     55     65       CYTOPLASMIC.
FT    TRANSMEM     66     83
FT    TOPO_DOM     84    102       NON CYTOPLASMIC.
FT    TRANSMEM    103    124
FT    TOPO_DOM    125    144       CYTOPLASMIC.
FT    TRANSMEM    145    164
FT    TOPO_DOM    165    193       NON CYTOPLASMIC.
FT    TRANSMEM    194    217
FT    TOPO_DOM    218    249       CYTOPLASMIC.
FT    TRANSMEM    250    270
FT    TOPO_DOM    271    281       NON CYTOPLASMIC.
FT    TRANSMEM    282    305
FT    TOPO_DOM    306    329       CYTOPLASMIC.
//
```

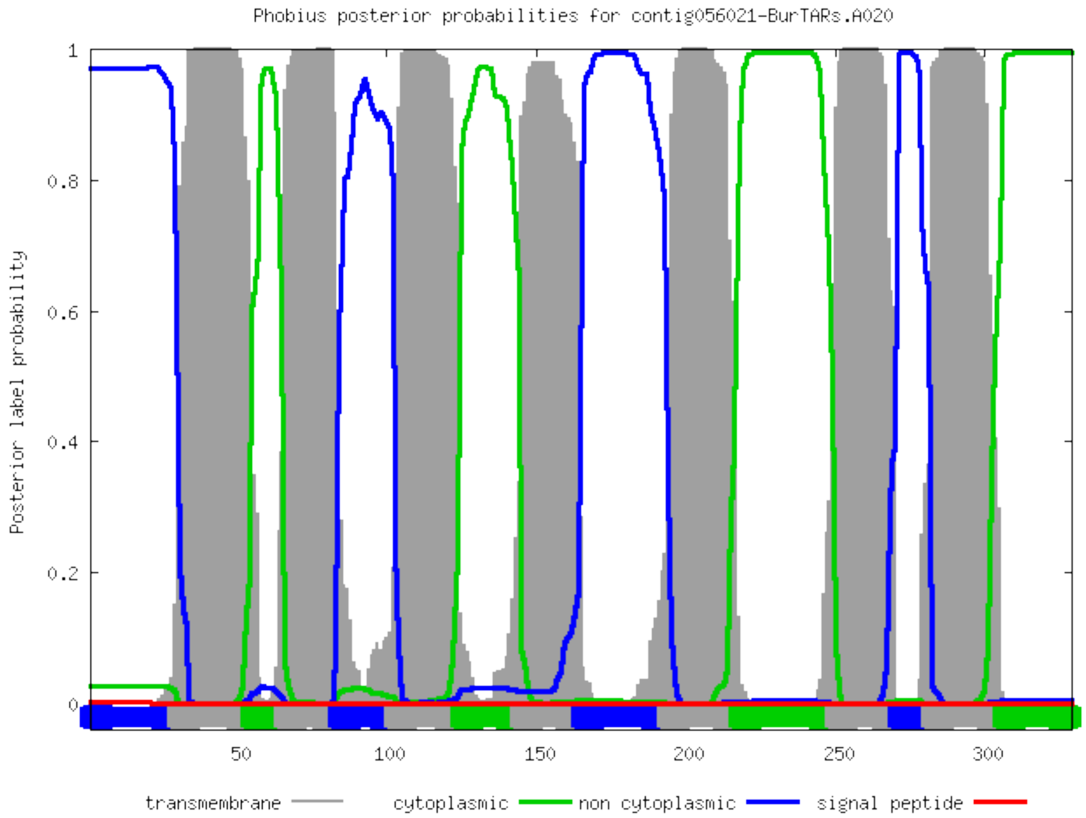

The probability data used in the plot is found here, and the gnuplot script is here.

Prediction of contig056020-BurTARs.A021\

```
ID    contig056020-BurTARs.A021\
FT    TOPO_DOM      1     29       NON CYTOPLASMIC.
FT    TRANSMEM     30     54
FT    TOPO_DOM     55     65       CYTOPLASMIC.
FT    TRANSMEM     66     88
FT    TOPO_DOM     89    102       NON CYTOPLASMIC.
FT    TRANSMEM    103    124
FT    TOPO_DOM    125    144       CYTOPLASMIC.
FT    TRANSMEM    145    164
FT    TOPO_DOM    165    193       NON CYTOPLASMIC.
FT    TRANSMEM    194    214
FT    TOPO_DOM    215    249       CYTOPLASMIC.
FT    TRANSMEM    250    267
FT    TOPO_DOM    268    278       NON CYTOPLASMIC.
FT    TRANSMEM    279    301
FT    TOPO_DOM    302    329       CYTOPLASMIC.
//
```

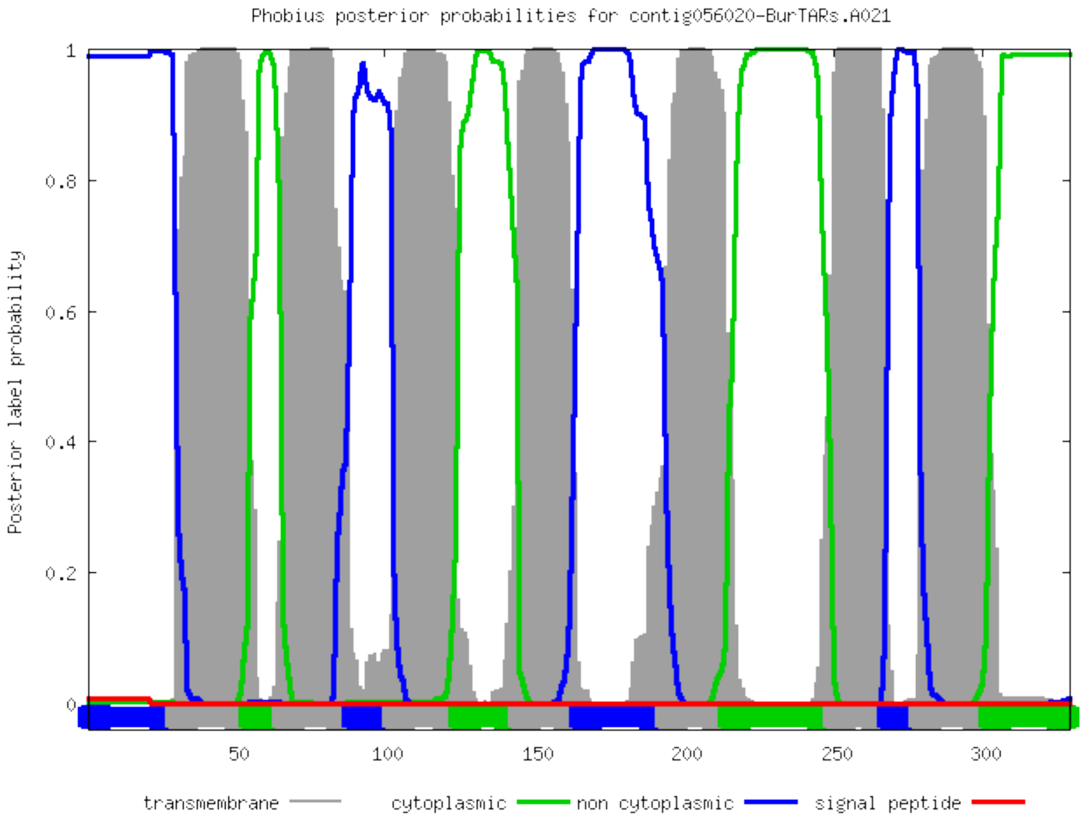

The probability data used in the plot is found here, and the gnuplot script is here.

Prediction of contig055697-BurTARs.A022\

```
ID    contig055697-BurTARs.A022\
FT    TOPO_DOM      1     27      NON CYTOPLASMIC.
FT    TRANSMEM     28     52
FT    TOPO_DOM     53     63      CYTOPLASMIC.
FT    TRANSMEM     64     94
FT    TOPO_DOM     95     99      NON CYTOPLASMIC.
FT    TRANSMEM    100    121
FT    TOPO_DOM    122    141      CYTOPLASMIC.
FT    TRANSMEM    142    161
FT    TOPO_DOM    162    180      NON CYTOPLASMIC.
FT    TRANSMEM    181    211
FT    TOPO_DOM    212    246      CYTOPLASMIC.
FT    TRANSMEM    247    267
FT    TOPO_DOM    268    278      NON CYTOPLASMIC.
FT    TRANSMEM    279    299
FT    TOPO_DOM    300    327      CYTOPLASMIC.
//
```

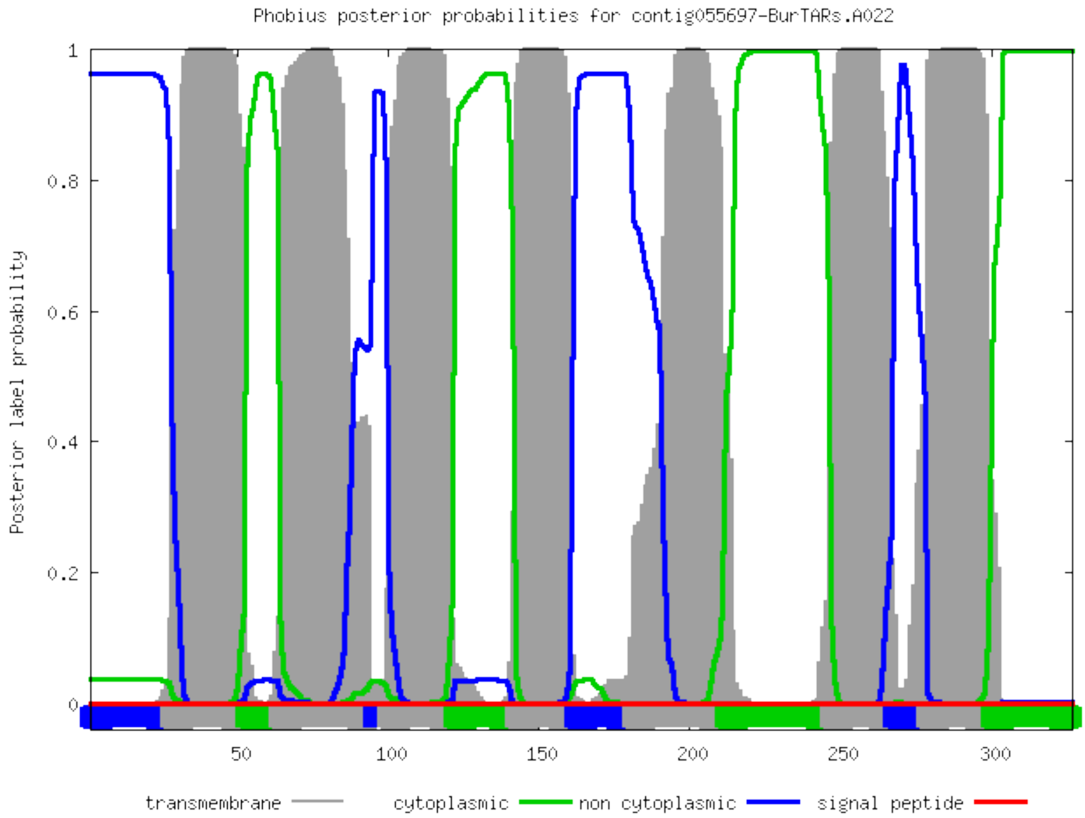

The probability data used in the plot is found here, and the gnuplot script is here.

Prediction of contig054630-BurTARs.A023\

```
ID   contig054630-BurTARs.A023\
FT   TOPO_DOM      1     29       NON CYTOPLASMIC.
FT   TRANSMEM     30     54
FT   TOPO_DOM     55     65       CYTOPLASMIC.
FT   TRANSMEM     66     83
FT   TOPO_DOM     84    102       NON CYTOPLASMIC.
FT   TRANSMEM    103    124
FT   TOPO_DOM    125    144       CYTOPLASMIC.
FT   TRANSMEM    145    164
FT   TOPO_DOM    165    183       NON CYTOPLASMIC.
FT   TRANSMEM    184    209
FT   TOPO_DOM    210    246       CYTOPLASMIC.
FT   TRANSMEM    247    267
FT   TOPO_DOM    268    286       NON CYTOPLASMIC.
FT   TRANSMEM    287    307
FT   TOPO_DOM    308    331       CYTOPLASMIC.
//
```

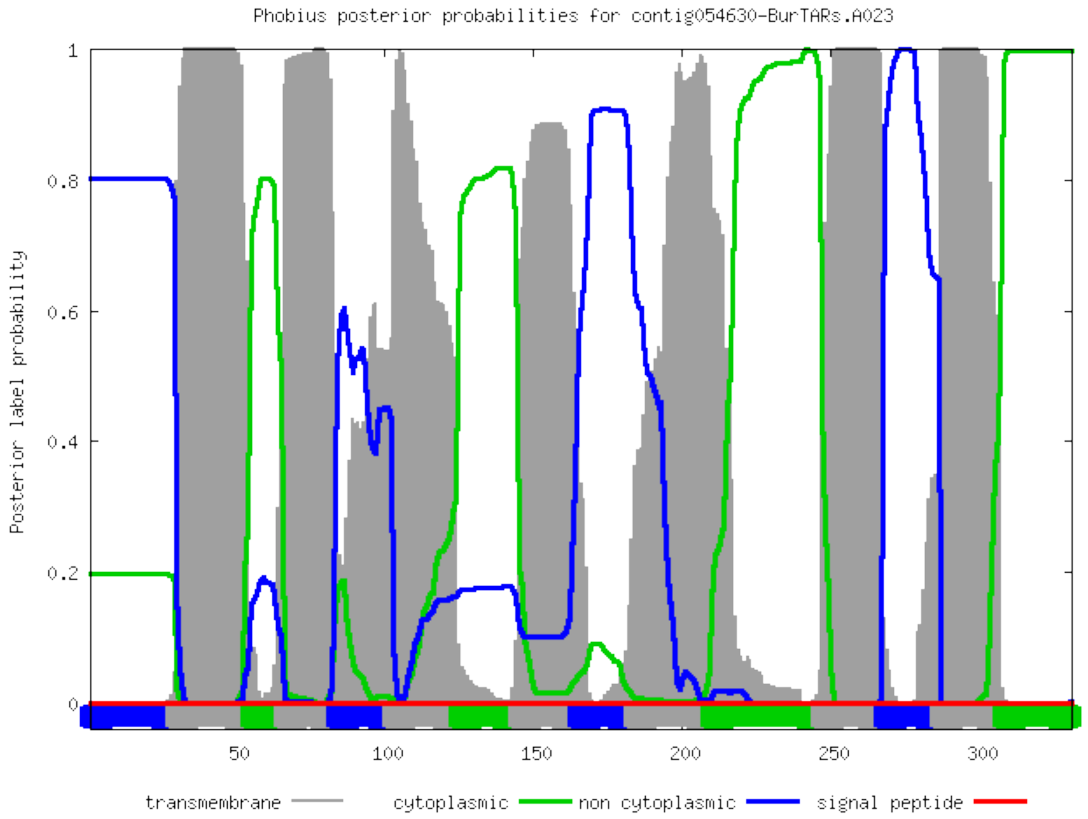

The probability data used in the plot is found here, and the gnuplot script is here.

Prediction of contig049540-BurTARs.A024\

```
ID    contig049540-BurTARs.A024\
FT    TOPO_DOM      1     29       NON CYTOPLASMIC.
FT    TRANSMEM     30     54
FT    TOPO_DOM     55     65       CYTOPLASMIC.
FT    TRANSMEM     66     82
FT    TOPO_DOM     83    101       NON CYTOPLASMIC.
FT    TRANSMEM    102    124
FT    TOPO_DOM    125    144       CYTOPLASMIC.
FT    TRANSMEM    145    165
FT    TOPO_DOM    166    184       NON CYTOPLASMIC.
FT    TRANSMEM    185    214
FT    TOPO_DOM    215    249       CYTOPLASMIC.
FT    TRANSMEM    250    270
FT    TOPO_DOM    271    281       NON CYTOPLASMIC.
FT    TRANSMEM    282    301
FT    TOPO_DOM    302    329       CYTOPLASMIC.
//
```

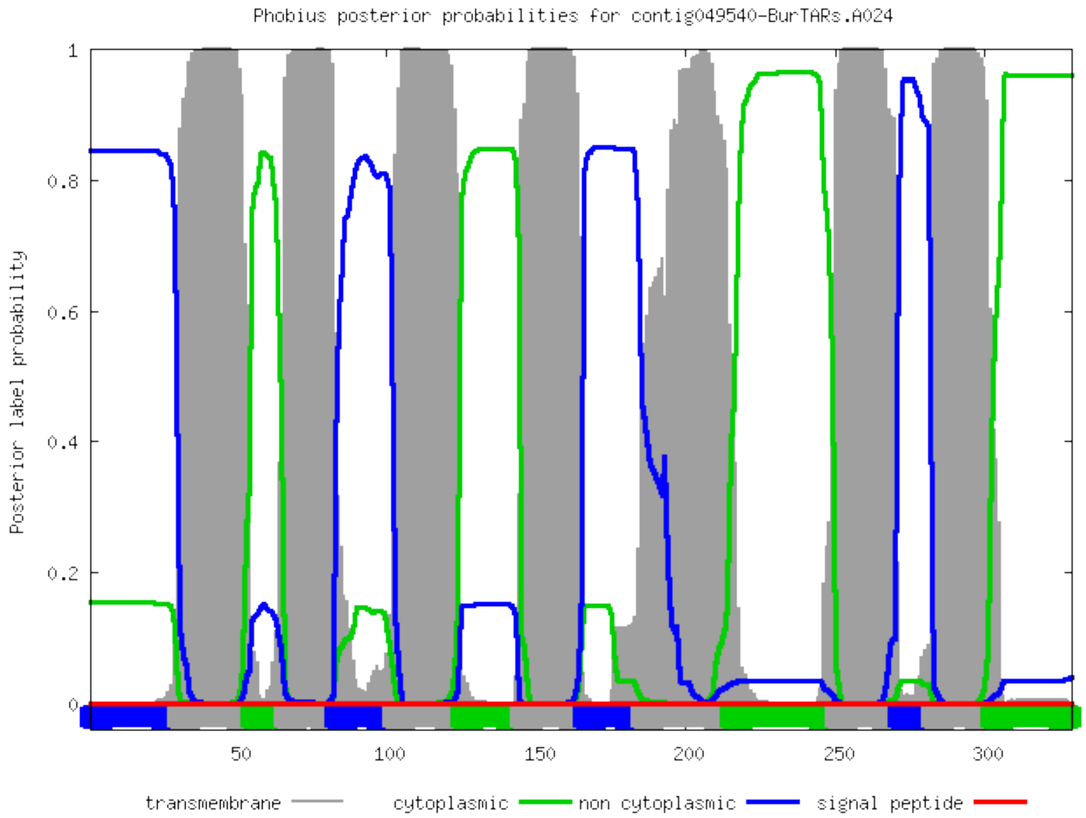

The probability data used in the plot is found here, and the gnuplot script is here.

Prediction of contig049534-BurTARs.A025\

```
ID    contig049534-BurTARs.A025\
FT    TOPO_DOM       1      27       NON CYTOPLASMIC.
FT    TRANSMEM      28      52
FT    TOPO_DOM      53      63       CYTOPLASMIC.
FT    TRANSMEM      64      81
FT    TOPO_DOM      82     100       NON CYTOPLASMIC.
FT    TRANSMEM     101     122
FT    TOPO_DOM     123     142       CYTOPLASMIC.
FT    TRANSMEM     143     166
FT    TOPO_DOM     167     185       NON CYTOPLASMIC.
FT    TRANSMEM     186     212
FT    TOPO_DOM     213     246       CYTOPLASMIC.
FT    TRANSMEM     247     267
FT    TOPO_DOM     268     278       NON CYTOPLASMIC.
FT    TRANSMEM     279     302
FT    TOPO_DOM     303     326       CYTOPLASMIC.
//
```

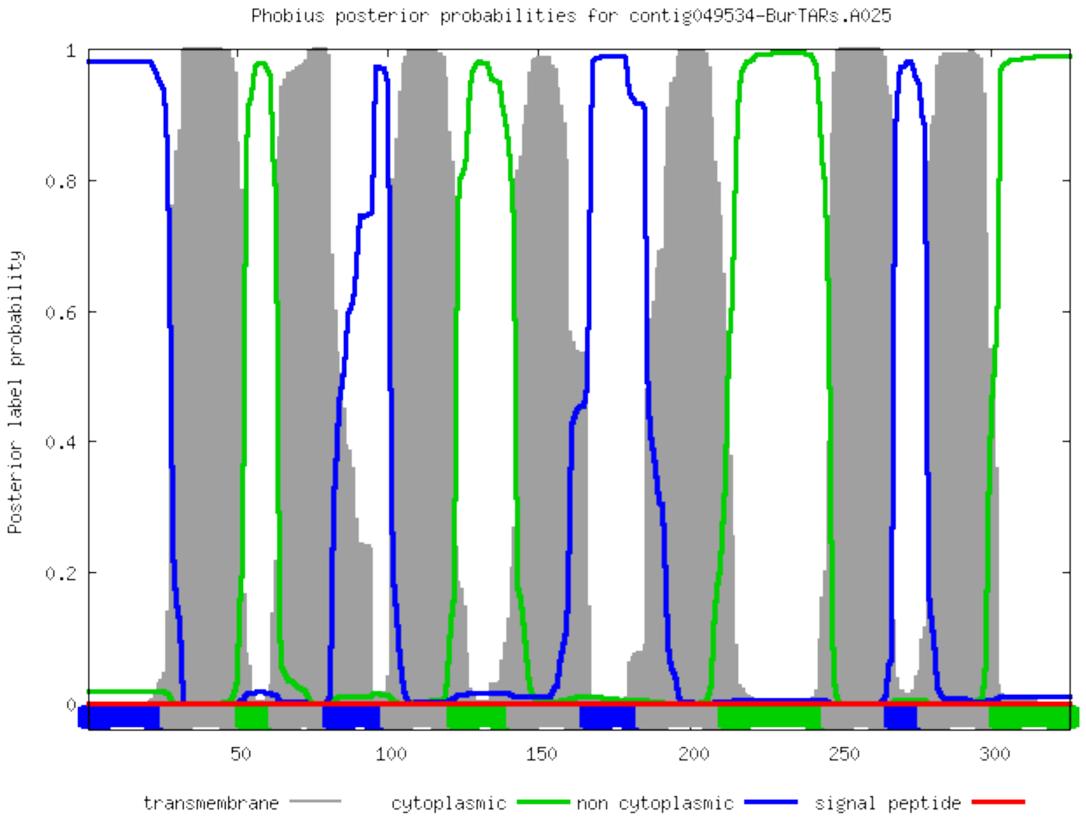

The probability data used in the plot is found here, and the gnuplot script is here.

Prediction of contig041024-BurTARs.A026\

```
ID   contig041024-BurTARs.A026\
FT   TOPO_DOM      1     29       NON CYTOPLASMIC.
FT   TRANSMEM     30     54
FT   TOPO_DOM     55     65       CYTOPLASMIC.
FT   TRANSMEM     66     83
FT   TOPO_DOM     84    102       NON CYTOPLASMIC.
FT   TRANSMEM    103    124
FT   TOPO_DOM    125    144       CYTOPLASMIC.
FT   TRANSMEM    145    163
FT   TOPO_DOM    164    193       NON CYTOPLASMIC.
FT   TRANSMEM    194    217
FT   TOPO_DOM    218    249       CYTOPLASMIC.
FT   TRANSMEM    250    270
FT   TOPO_DOM    271    281       NON CYTOPLASMIC.
FT   TRANSMEM    282    301
FT   TOPO_DOM    302    329       CYTOPLASMIC.
//
```

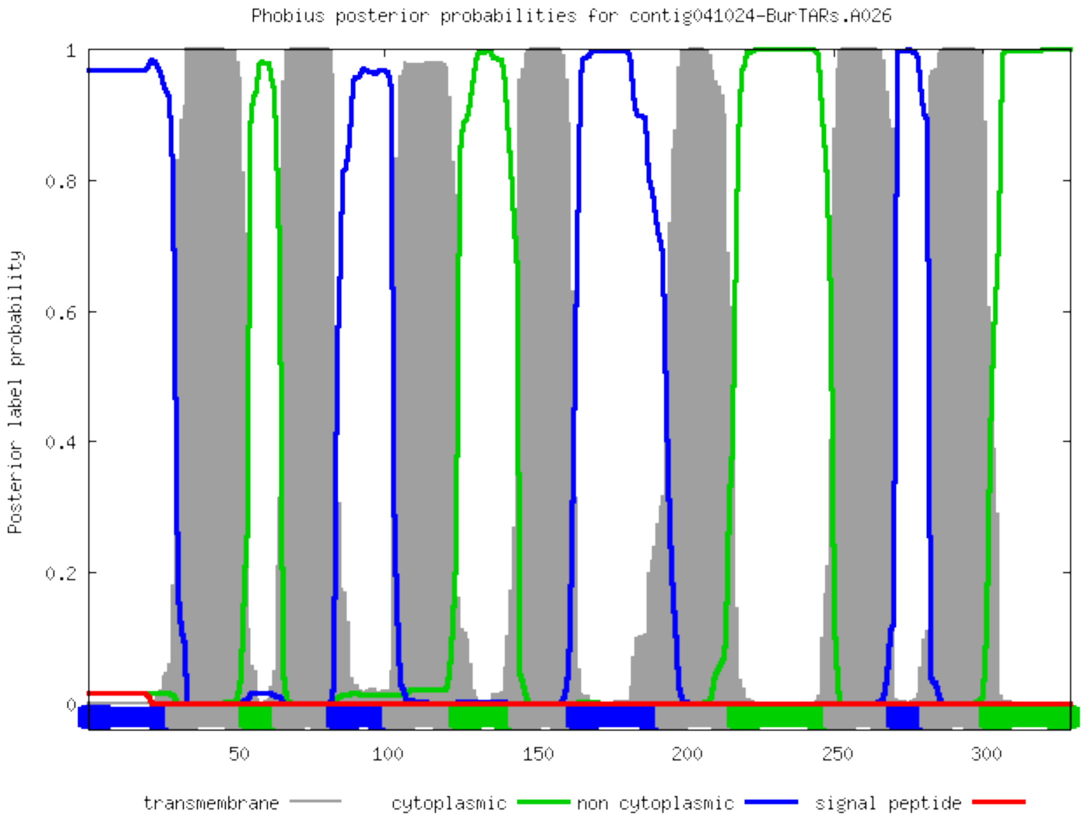

The probability data used in the plot is found here, and the gnuplot script is here.

Prediction of contig034854-BurTARs.A027\

```
ID   contig034854-BurTARs.A027\
FT   TOPO_DOM      1     29       NON CYTOPLASMIC.
FT   TRANSMEM     30     54
FT   TOPO_DOM     55     65       CYTOPLASMIC.
FT   TRANSMEM     66     83
FT   TOPO_DOM     84    102       NON CYTOPLASMIC.
FT   TRANSMEM    103    124
FT   TOPO_DOM    125    144       CYTOPLASMIC.
FT   TRANSMEM    145    165
FT   TOPO_DOM    166    193       NON CYTOPLASMIC.
FT   TRANSMEM    194    217
FT   TOPO_DOM    218    250       CYTOPLASMIC.
FT   TRANSMEM    251    271
FT   TOPO_DOM    272    282       NON CYTOPLASMIC.
FT   TRANSMEM    283    306
FT   TOPO_DOM    307    330       CYTOPLASMIC.
//
```

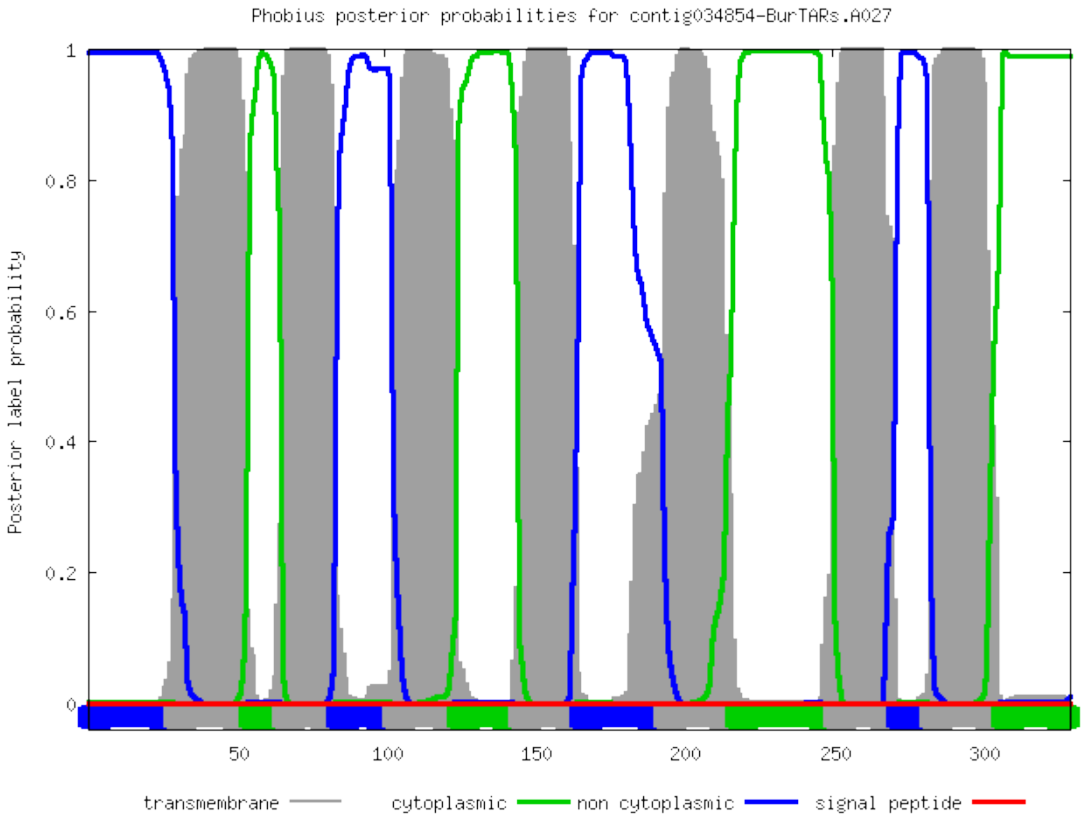

The probability data used in the plot is found here, and the gnuplot script is here.

Prediction of contig057148-BurTARs.A028\

```
ID    contig057148-BurTARs.A028\
FT    TOPO_DOM      1     27      NON CYTOPLASMIC.
FT    TRANSMEM     28     52
FT    TOPO_DOM     53     63      CYTOPLASMIC.
FT    TRANSMEM     64     81
FT    TOPO_DOM     82    100      NON CYTOPLASMIC.
FT    TRANSMEM    101    122
FT    TOPO_DOM    123    142      CYTOPLASMIC.
FT    TRANSMEM    143    162
FT    TOPO_DOM    163    191      NON CYTOPLASMIC.
FT    TRANSMEM    192    215
FT    TOPO_DOM    216    247      CYTOPLASMIC.
FT    TRANSMEM    248    268
FT    TOPO_DOM    269    279      NON CYTOPLASMIC.
FT    TRANSMEM    280    303
FT    TOPO_DOM    304    327      CYTOPLASMIC.
//
```

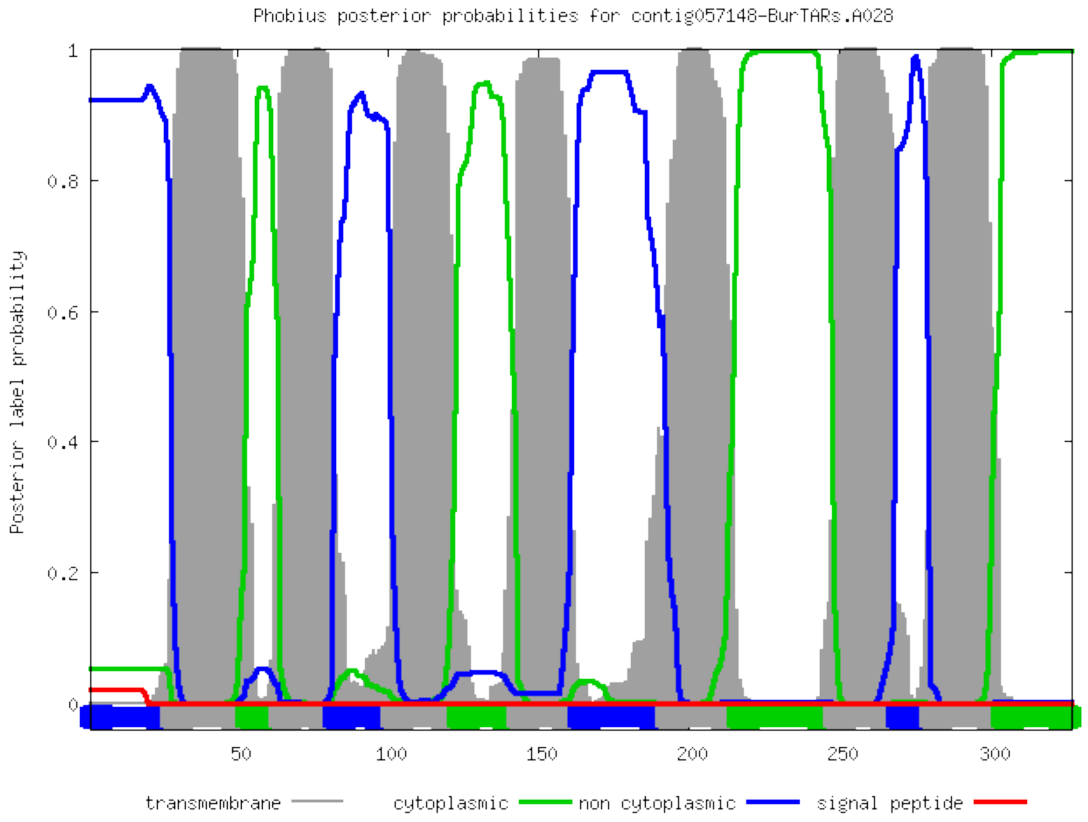

The probability data used in the plot is found here, and the gnuplot script is here.

Prediction of contig059766-BurTARs.A029\

```
ID   contig059766-BurTARs.A029\
FT   TOPO_DOM      1     26       NON CYTOPLASMIC.
FT   TRANSMEM     27     51
FT   TOPO_DOM     52     62       CYTOPLASMIC.
FT   TRANSMEM     63     93
FT   TOPO_DOM     94     98       NON CYTOPLASMIC.
FT   TRANSMEM     99    120
FT   TOPO_DOM    121    140       CYTOPLASMIC.
FT   TRANSMEM    141    161
FT   TOPO_DOM    162    191       NON CYTOPLASMIC.
FT   TRANSMEM    192    213
FT   TOPO_DOM    214    242       CYTOPLASMIC.
FT   TRANSMEM    243    266
FT   TOPO_DOM    267    277       NON CYTOPLASMIC.
FT   TRANSMEM    278    298
FT   TOPO_DOM    299    326       CYTOPLASMIC.
//
```

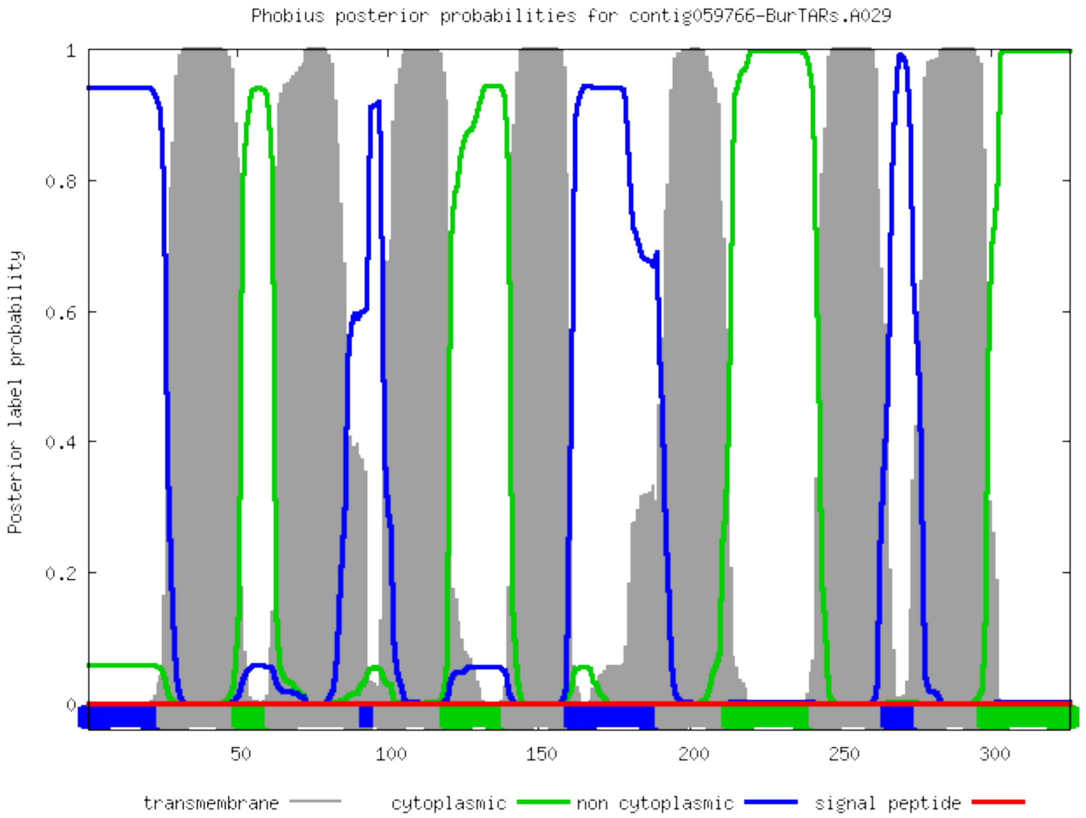

The probability data used in the plot is found here, and the gnuplot script is here.

Prediction of contig065494-BurTARs.A030\

```
ID   contig065494-BurTARs.A030\
FT   TOPO_DOM      1     26       NON CYTOPLASMIC.
FT   TRANSMEM     27     51
FT   TOPO_DOM     52     62       CYTOPLASMIC.
FT   TRANSMEM     63     93
FT   TOPO_DOM     94     98       NON CYTOPLASMIC.
FT   TRANSMEM     99    120
FT   TOPO_DOM    121    140       CYTOPLASMIC.
FT   TRANSMEM    141    161
FT   TOPO_DOM    162    191       NON CYTOPLASMIC.
FT   TRANSMEM    192    213
FT   TOPO_DOM    214    242       CYTOPLASMIC.
FT   TRANSMEM    243    266
FT   TOPO_DOM    267    277       NON CYTOPLASMIC.
FT   TRANSMEM    278    298
FT   TOPO_DOM    299    326       CYTOPLASMIC.
//
```

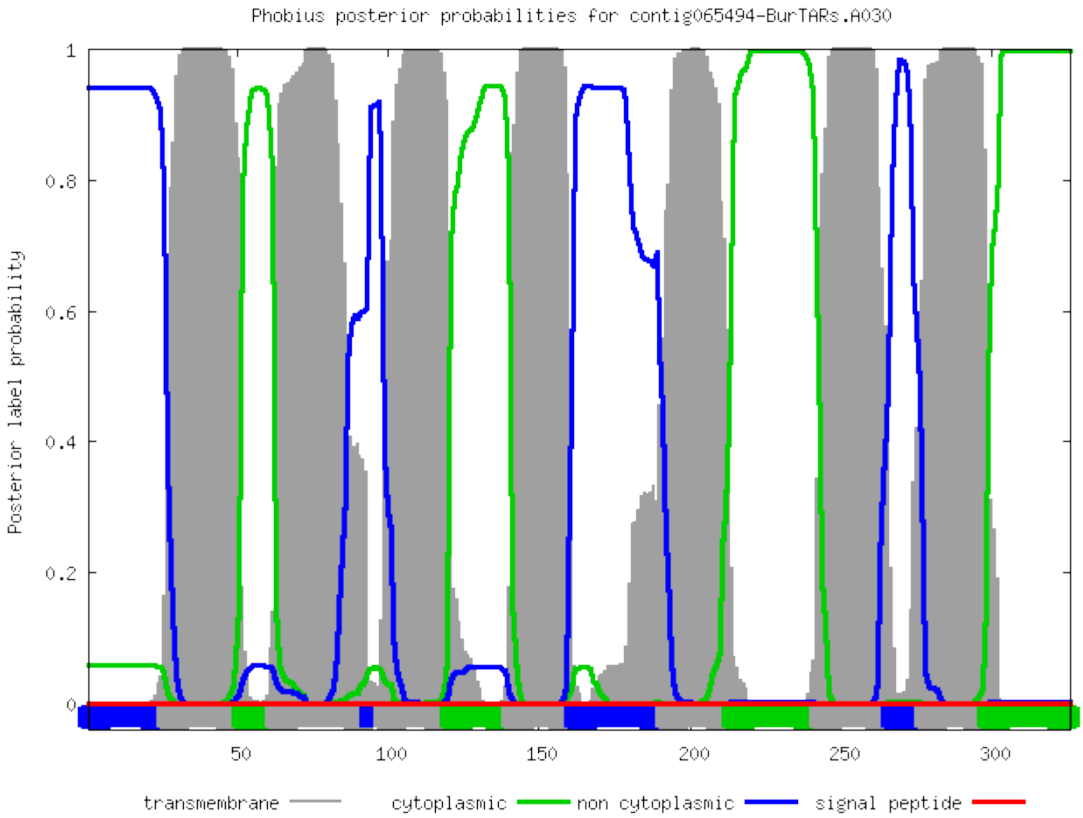

The probability data used in the plot is found here, and the gnuplot script is here.

Prediction of contig057305-BurTARs.A031\

```
ID   contig057305-BurTARs.A031\
FT   TOPO_DOM      1     25       NON CYTOPLASMIC.
FT   TRANSMEM     26     51
FT   TOPO_DOM     52     62       CYTOPLASMIC.
FT   TRANSMEM     63     85
FT   TOPO_DOM     86     96       NON CYTOPLASMIC.
FT   TRANSMEM     97    119
FT   TOPO_DOM    120    139       CYTOPLASMIC.
FT   TRANSMEM    140    159
FT   TOPO_DOM    160    188       NON CYTOPLASMIC.
FT   TRANSMEM    189    213
FT   TOPO_DOM    214    239       CYTOPLASMIC.
FT   TRANSMEM    240    260
FT   TOPO_DOM    261    271       NON CYTOPLASMIC.
FT   TRANSMEM    272    296
FT   TOPO_DOM    297    324       CYTOPLASMIC.
//
```

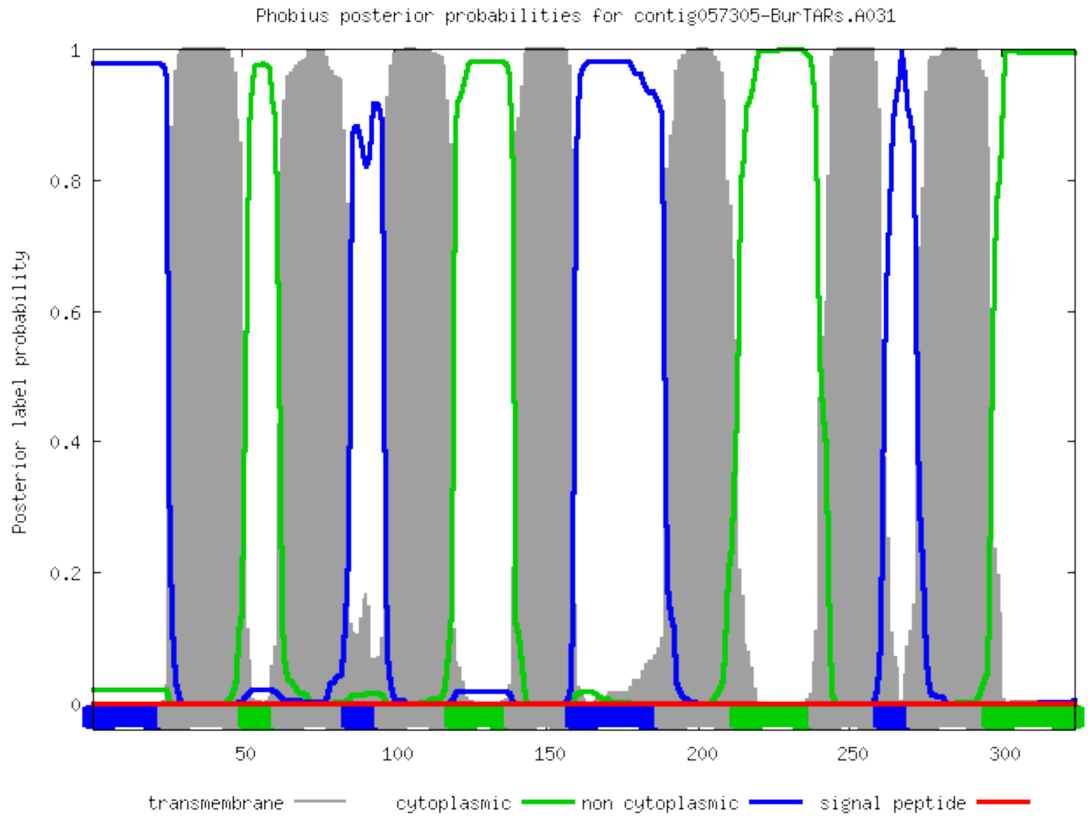

The probability data used in the plot is found here, and the gnuplot script is here.

Prediction of contig025313-BriTAR.A002\

```
ID   contig025313-BriTAR.A002\
FT   TOPO_DOM      1     29       NON CYTOPLASMIC.
FT   TRANSMEM     30     54
FT   TOPO_DOM     55     65       CYTOPLASMIC.
FT   TRANSMEM     66     83
FT   TOPO_DOM     84    102       NON CYTOPLASMIC.
FT   TRANSMEM    103    125
FT   TOPO_DOM    126    145       CYTOPLASMIC.
FT   TRANSMEM    146    165
FT   TOPO_DOM    166    194       NON CYTOPLASMIC.
FT   TRANSMEM    195    218
FT   TOPO_DOM    219    246       CYTOPLASMIC.
FT   TRANSMEM    247    267
FT   TOPO_DOM    268    286       NON CYTOPLASMIC.
FT   TRANSMEM    287    307
FT   TOPO_DOM    308    331       CYTOPLASMIC.
//
```

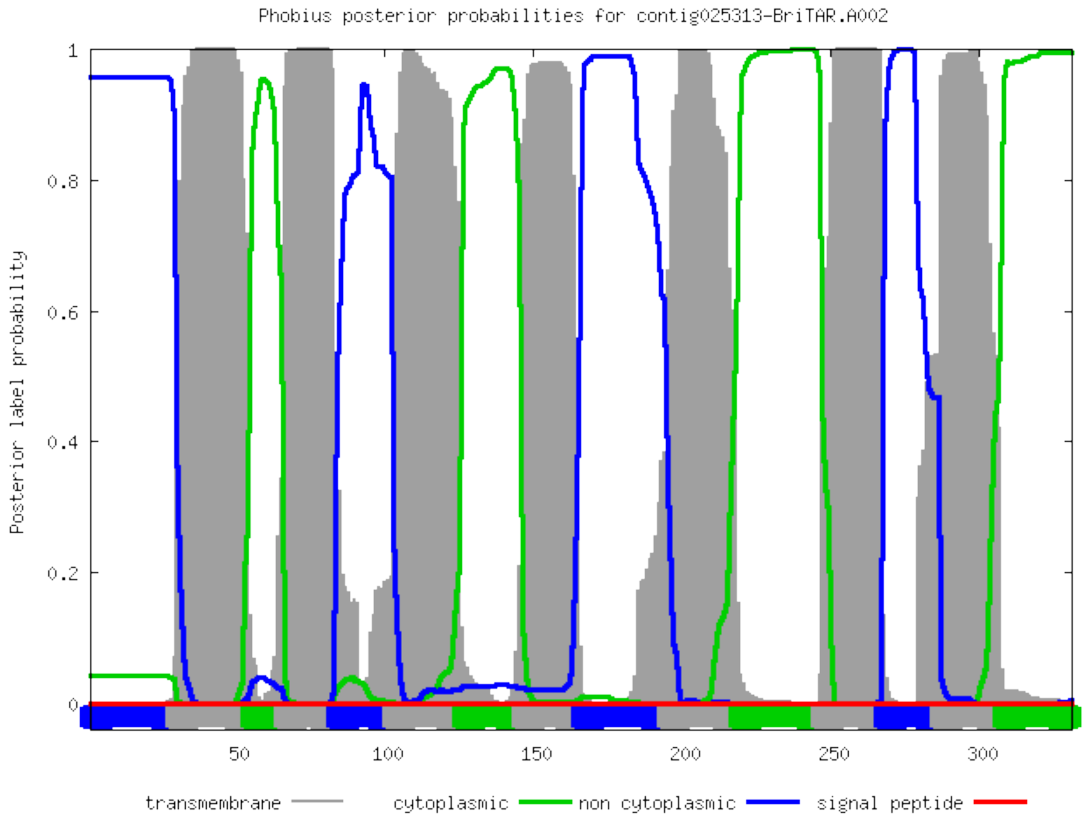

The probability data used in the plot is found here, and the gnuplot script is here.

Prediction of contig029633-BriTAR.A003\

```
ID    contig029633-BriTAR.A003\
FT    TOPO_DOM      1     28       NON CYTOPLASMIC.
FT    TRANSMEM     29     53
FT    TOPO_DOM     54     64       CYTOPLASMIC.
FT    TRANSMEM     65     86
FT    TOPO_DOM     87    105       NON CYTOPLASMIC.
FT    TRANSMEM    106    124
FT    TOPO_DOM    125    144       CYTOPLASMIC.
FT    TRANSMEM    145    170
FT    TOPO_DOM    171    193       NON CYTOPLASMIC.
FT    TRANSMEM    194    217
FT    TOPO_DOM    218    244       CYTOPLASMIC.
FT    TRANSMEM    245    262
FT    TOPO_DOM    263    273       NON CYTOPLASMIC.
FT    TRANSMEM    274    298
FT    TOPO_DOM    299    326       CYTOPLASMIC.
//
```

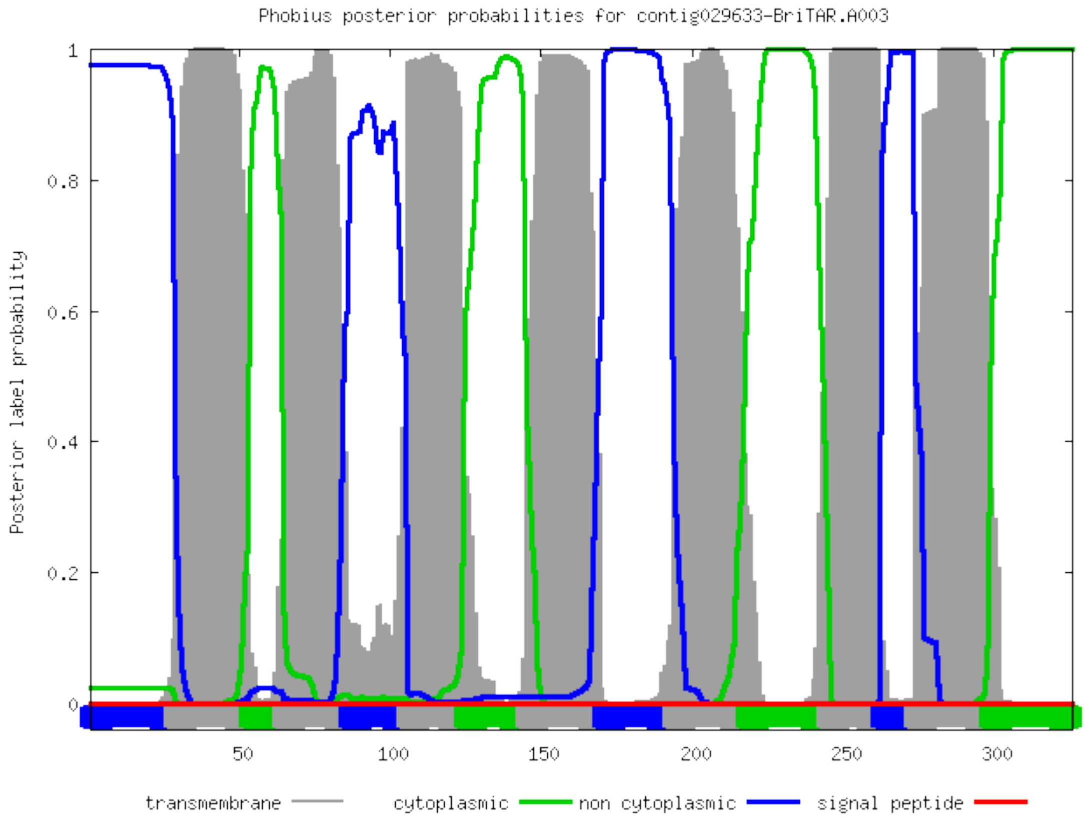

The probability data used in the plot is found here, and the gnuplot script is here.

Prediction of contig035253-BriTAR.B025\

```
ID   contig035253-BriTAR.B025\
FT   TOPO_DOM      1     29       NON CYTOPLASMIC.
FT   TRANSMEM     30     57
FT   TOPO_DOM     58     65       CYTOPLASMIC.
FT   TRANSMEM     66     86
FT   TOPO_DOM     87    105       NON CYTOPLASMIC.
FT   TRANSMEM    106    125
FT   TOPO_DOM    126    144       CYTOPLASMIC.
FT   TRANSMEM    145    169
FT   TOPO_DOM    170    193       NON CYTOPLASMIC.
FT   TRANSMEM    194    213
FT   TOPO_DOM    214    239       CYTOPLASMIC.
FT   TRANSMEM    240    258
FT   TOPO_DOM    259    277       NON CYTOPLASMIC.
FT   TRANSMEM    278    298
FT   TOPO_DOM    299    322       CYTOPLASMIC.
//
```

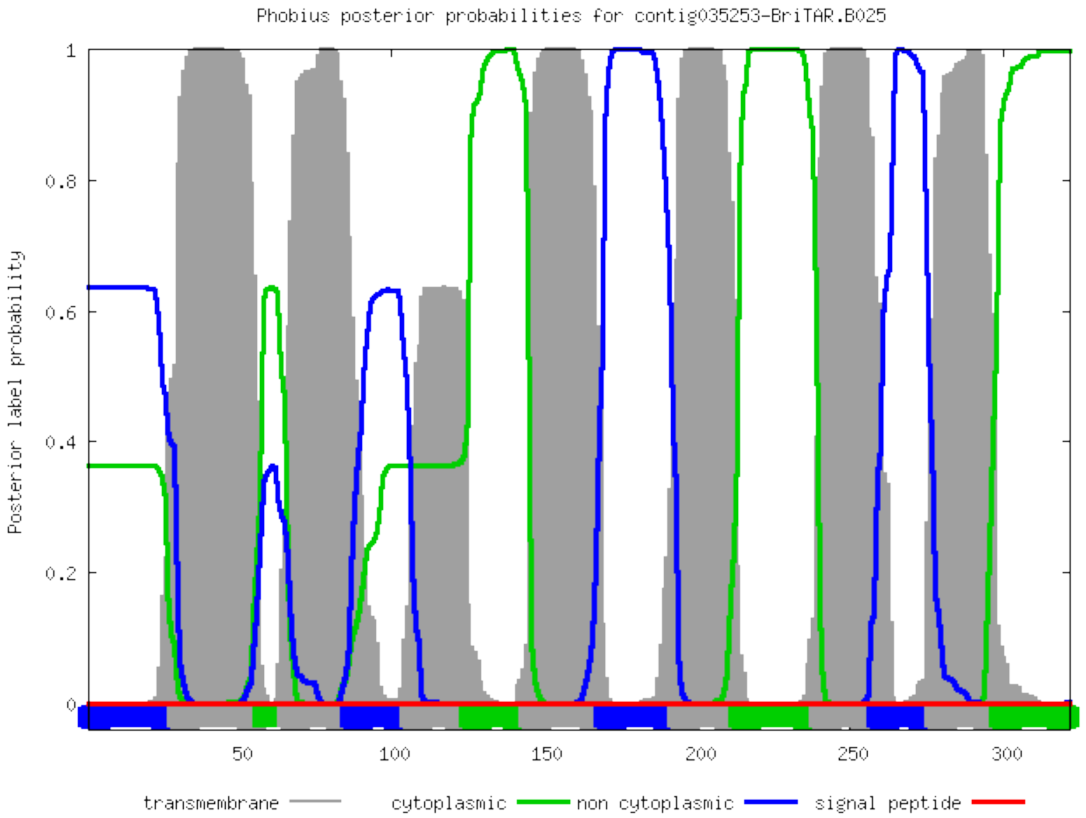

The probability data used in the plot is found here, and the gnuplot script is here.

Prediction of contig084868-BriTARs.A014\

```
ID   contig084868-BriTARs.A014\
FT   TOPO_DOM      1     29       NON CYTOPLASMIC.
FT   TRANSMEM     30     53
FT   TOPO_DOM     54     64       CYTOPLASMIC.
FT   TRANSMEM     65     82
FT   TOPO_DOM     83    101       NON CYTOPLASMIC.
FT   TRANSMEM    102    123
FT   TOPO_DOM    124    143       CYTOPLASMIC.
FT   TRANSMEM    144    163
FT   TOPO_DOM    164    192       NON CYTOPLASMIC.
FT   TRANSMEM    193    216
FT   TOPO_DOM    217    249       CYTOPLASMIC.
FT   TRANSMEM    250    270
FT   TOPO_DOM    271    281       NON CYTOPLASMIC.
FT   TRANSMEM    282    301
FT   TOPO_DOM    302    329       CYTOPLASMIC.
//
```

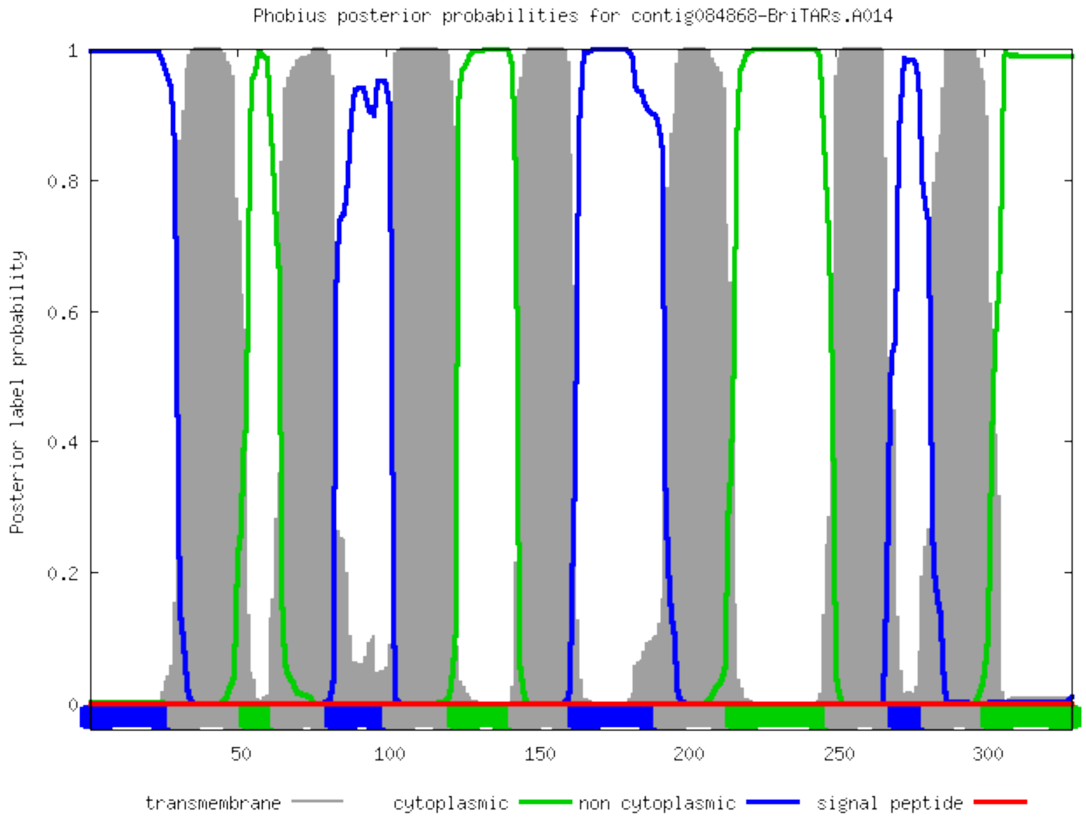

The probability data used in the plot is found here, and the gnuplot script is here.

Prediction of contig084876-BriTARs.A015\

```
ID   contig084876-BriTARs.A015\
FT   TOPO_DOM      1     29       NON CYTOPLASMIC.
FT   TRANSMEM     30     54
FT   TOPO_DOM     55     65       CYTOPLASMIC.
FT   TRANSMEM     66     83
FT   TOPO_DOM     84    102       NON CYTOPLASMIC.
FT   TRANSMEM    103    124
FT   TOPO_DOM    125    144       CYTOPLASMIC.
FT   TRANSMEM    145    164
FT   TOPO_DOM    165    193       NON CYTOPLASMIC.
FT   TRANSMEM    194    217
FT   TOPO_DOM    218    250       CYTOPLASMIC.
FT   TRANSMEM    251    271
FT   TOPO_DOM    272    282       NON CYTOPLASMIC.
FT   TRANSMEM    283    306
FT   TOPO_DOM    307    330       CYTOPLASMIC.
//
```

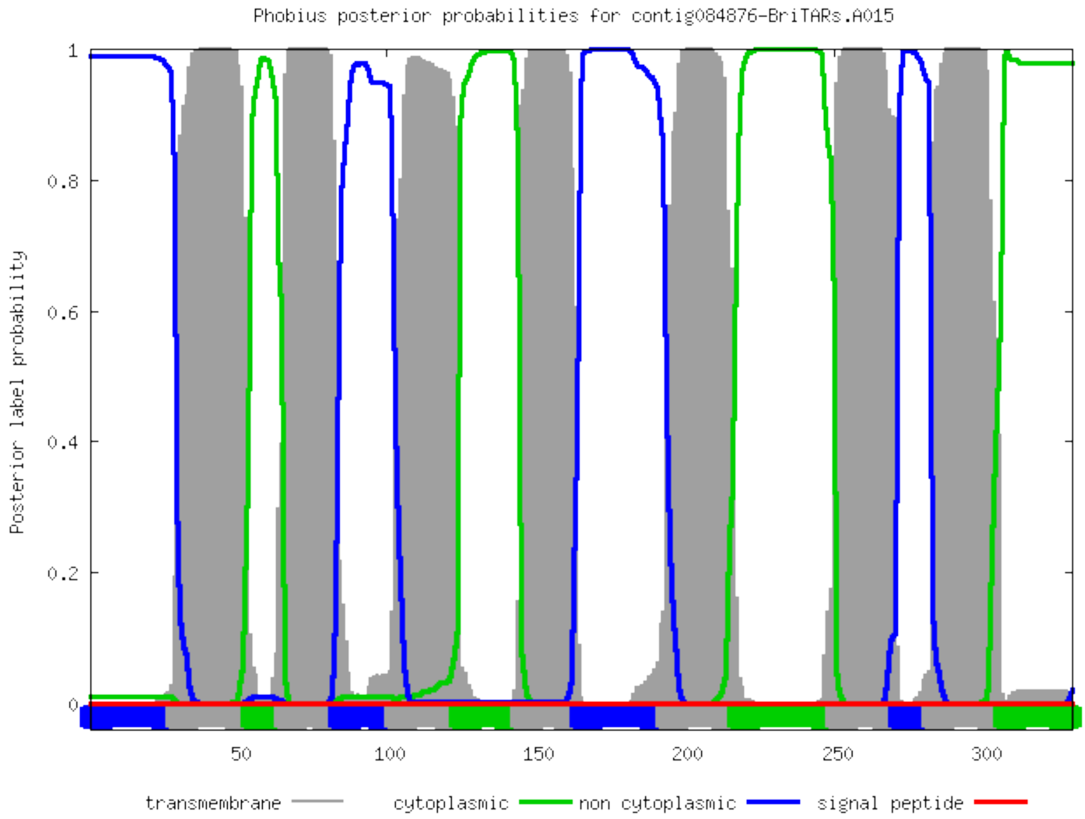

The probability data used in the plot is found here, and the gnuplot script is here.

Prediction of contig084880-BriTARs.A016\

```
ID   contig084880-BriTARs.A016\
FT   TOPO_DOM      1     31       NON CYTOPLASMIC.
FT   TRANSMEM     32     56
FT   TOPO_DOM     57     67       CYTOPLASMIC.
FT   TRANSMEM     68     85
FT   TOPO_DOM     86    104       NON CYTOPLASMIC.
FT   TRANSMEM    105    126
FT   TOPO_DOM    127    146       CYTOPLASMIC.
FT   TRANSMEM    147    165
FT   TOPO_DOM    166    195       NON CYTOPLASMIC.
FT   TRANSMEM    196    219
FT   TOPO_DOM    220    251       CYTOPLASMIC.
FT   TRANSMEM    252    272
FT   TOPO_DOM    273    283       NON CYTOPLASMIC.
FT   TRANSMEM    284    307
FT   TOPO_DOM    308    333       CYTOPLASMIC.
//
```

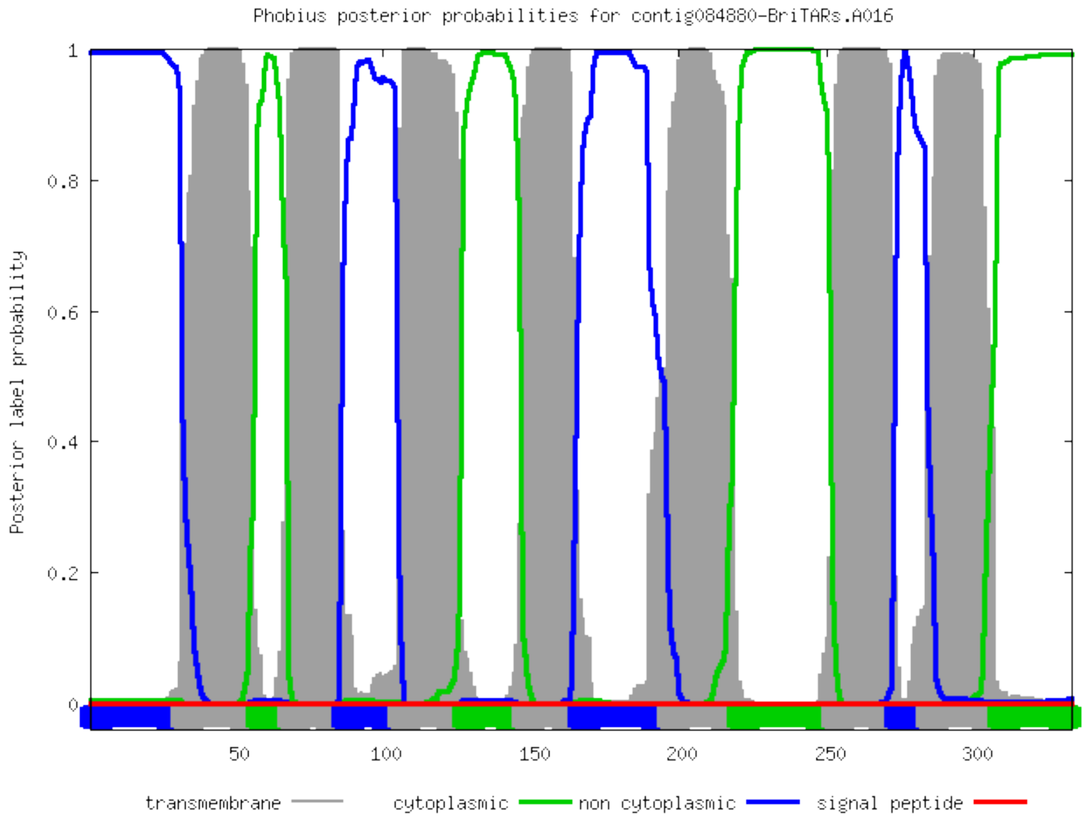

The probability data used in the plot is found here, and the gnuplot script is here.

Prediction of contig084886-BriTARs.A017\

```
ID   contig084886-BriTARs.A017\
FT   TOPO_DOM      1     27       CYTOPLASMIC.
FT   TRANSMEM     28     52
FT   TOPO_DOM     53     63       NON CYTOPLASMIC.
FT   TRANSMEM     64     83
FT   TOPO_DOM     84     89       CYTOPLASMIC.
FT   TRANSMEM     90    115
FT   TOPO_DOM    116    191       NON CYTOPLASMIC.
FT   TRANSMEM    192    215
FT   TOPO_DOM    216    247       CYTOPLASMIC.
FT   TRANSMEM    248    268
FT   TOPO_DOM    269    279       NON CYTOPLASMIC.
FT   TRANSMEM    280    303
FT   TOPO_DOM    304    327       CYTOPLASMIC.
//
```

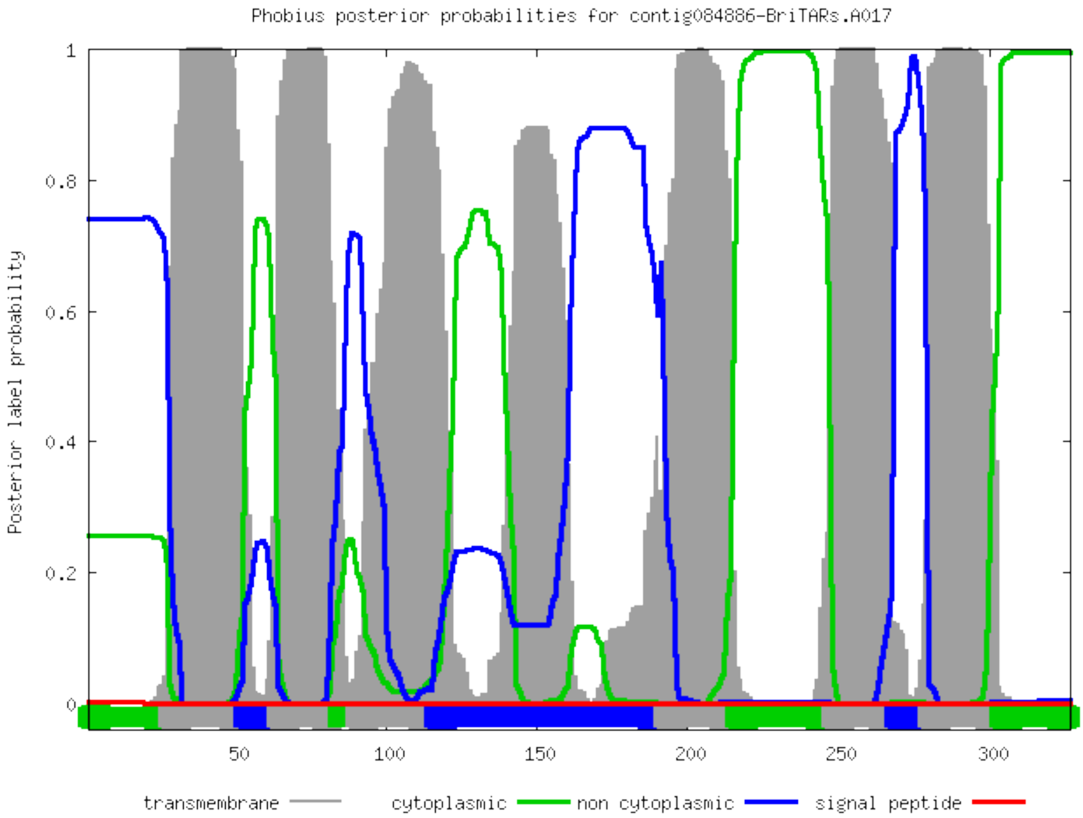

The probability data used in the plot is found here, and the gnuplot script is here.

Prediction of contig084887-BriTARs.A018\

```
ID   contig084887-BriTARs.A018\
FT   TOPO_DOM      1     29       NON CYTOPLASMIC.
FT   TRANSMEM     30     54
FT   TOPO_DOM     55     65       CYTOPLASMIC.
FT   TRANSMEM     66     83
FT   TOPO_DOM     84    102       NON CYTOPLASMIC.
FT   TRANSMEM    103    124
FT   TOPO_DOM    125    144       CYTOPLASMIC.
FT   TRANSMEM    145    163
FT   TOPO_DOM    164    182       NON CYTOPLASMIC.
FT   TRANSMEM    183    214
FT   TOPO_DOM    215    249       CYTOPLASMIC.
FT   TRANSMEM    250    270
FT   TOPO_DOM    271    281       NON CYTOPLASMIC.
FT   TRANSMEM    282    305
FT   TOPO_DOM    306    329       CYTOPLASMIC.
//
```

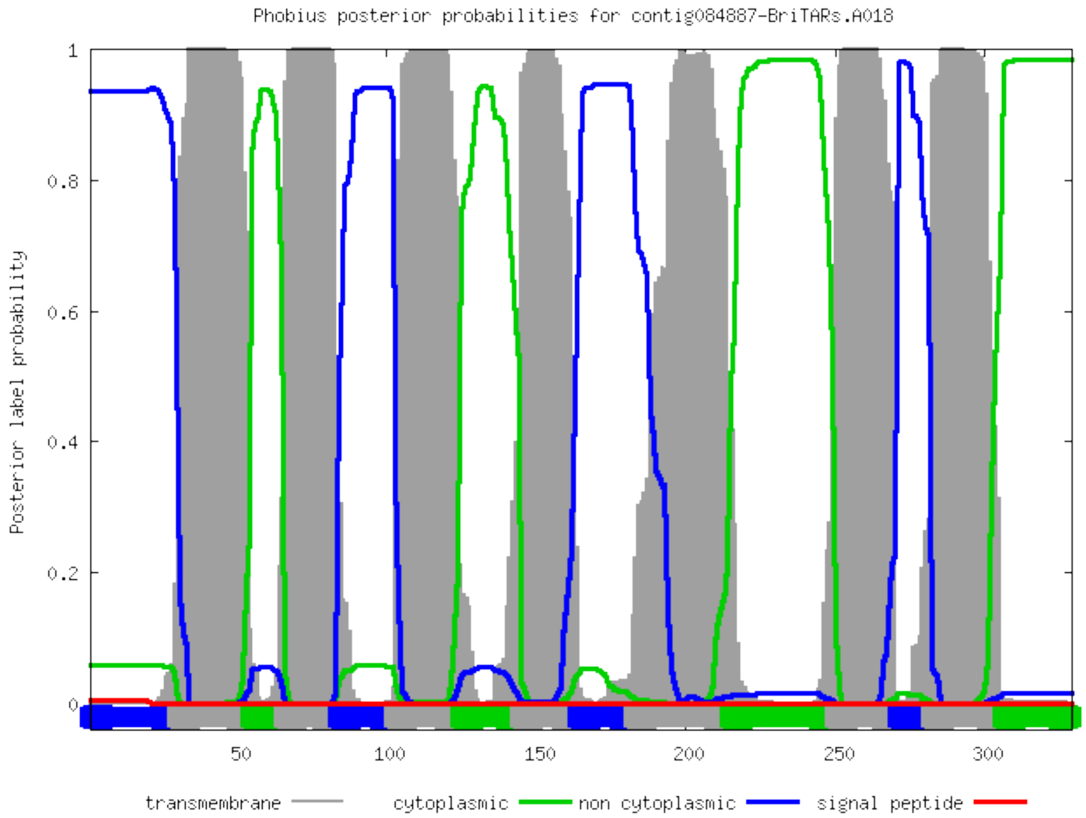

The probability data used in the plot is found here, and the gnuplot script is here.

Prediction of contig086337-BriTARs.A019\

```
ID   contig086337-BriTARs.A019\
FT   TOPO_DOM      1     26       NON CYTOPLASMIC.
FT   TRANSMEM     27     51
FT   TOPO_DOM     52     62       CYTOPLASMIC.
FT   TRANSMEM     63     93
FT   TOPO_DOM     94     98       NON CYTOPLASMIC.
FT   TRANSMEM     99    120
FT   TOPO_DOM    121    140       CYTOPLASMIC.
FT   TRANSMEM    141    160
FT   TOPO_DOM    161    179       NON CYTOPLASMIC.
FT   TRANSMEM    180    210
FT   TOPO_DOM    211    245       CYTOPLASMIC.
FT   TRANSMEM    246    266
FT   TOPO_DOM    267    277       NON CYTOPLASMIC.
FT   TRANSMEM    278    298
FT   TOPO_DOM    299    326       CYTOPLASMIC.
//
```

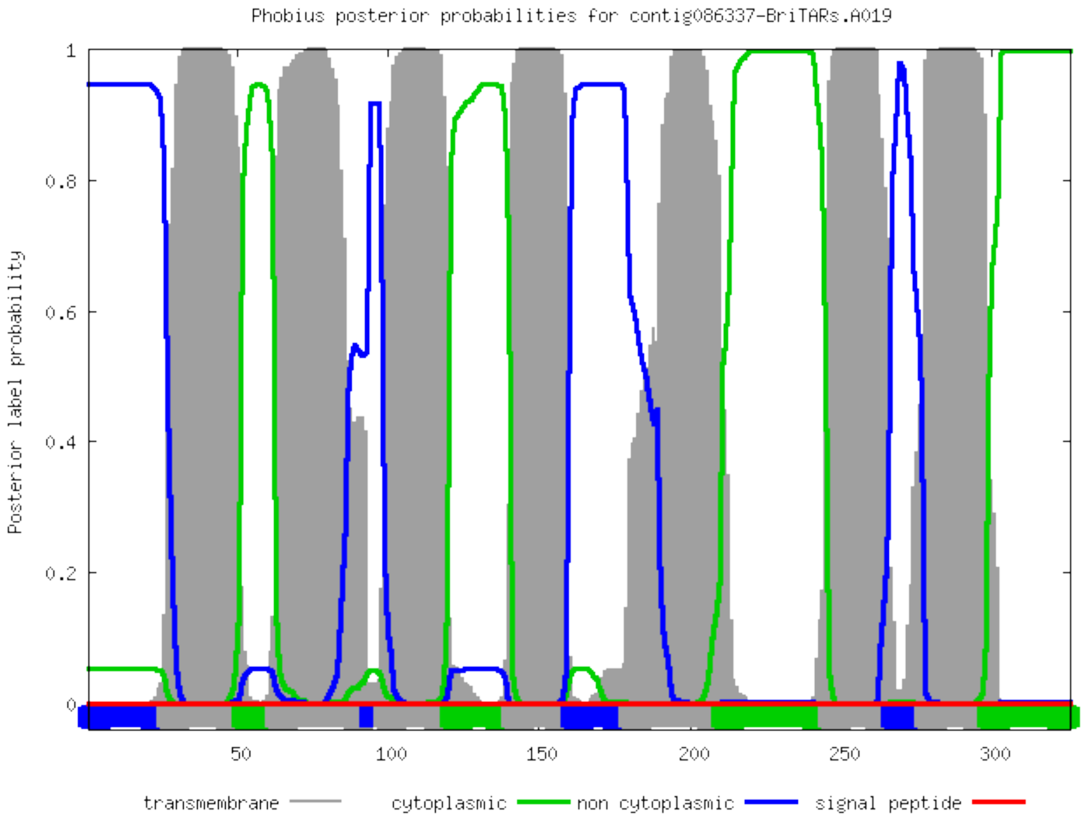

The probability data used in the plot is found here, and the gnuplot script is here.

Prediction of contig086344-BriTARs.A020\

```
ID    contig086344-BriTARs.A020\
FT    TOPO_DOM      1     26       NON CYTOPLASMIC.
FT    TRANSMEM     27     51
FT    TOPO_DOM     52     62       CYTOPLASMIC.
FT    TRANSMEM     63     86
FT    TOPO_DOM     87     97       NON CYTOPLASMIC.
FT    TRANSMEM     98    120
FT    TOPO_DOM    121    140       CYTOPLASMIC.
FT    TRANSMEM    141    160
FT    TOPO_DOM    161    179       NON CYTOPLASMIC.
FT    TRANSMEM    180    213
FT    TOPO_DOM    214    246       CYTOPLASMIC.
FT    TRANSMEM    247    268
FT    TOPO_DOM    269    282       NON CYTOPLASMIC.
FT    TRANSMEM    283    304
FT    TOPO_DOM    305    327       CYTOPLASMIC.
//
```

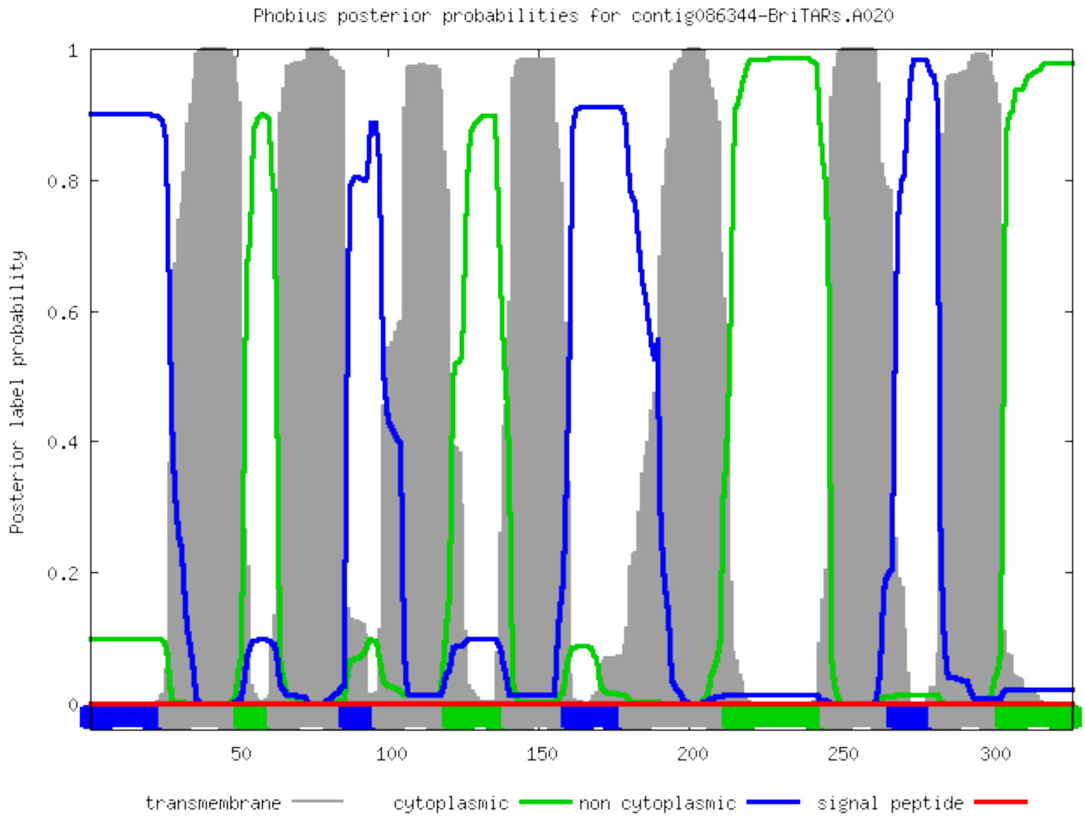

The probability data used in the plot is found here, and the gnuplot script is here.

Prediction of contig086351-BriTARs.A021\

```
ID   contig086351-BriTARs.A021\
FT   TOPO_DOM       1     25       NON CYTOPLASMIC.
FT   TRANSMEM      26     50
FT   TOPO_DOM      51     61       CYTOPLASMIC.
FT   TRANSMEM      62     85
FT   TOPO_DOM      86     96       NON CYTOPLASMIC.
FT   TRANSMEM      97    119
FT   TOPO_DOM     120    139       CYTOPLASMIC.
FT   TRANSMEM     140    159
FT   TOPO_DOM     160    188       NON CYTOPLASMIC.
FT   TRANSMEM     189    213
FT   TOPO_DOM     214    243       CYTOPLASMIC.
FT   TRANSMEM     244    266
FT   TOPO_DOM     267    271       NON CYTOPLASMIC.
FT   TRANSMEM     272    296
FT   TOPO_DOM     297    324       CYTOPLASMIC.
//
```

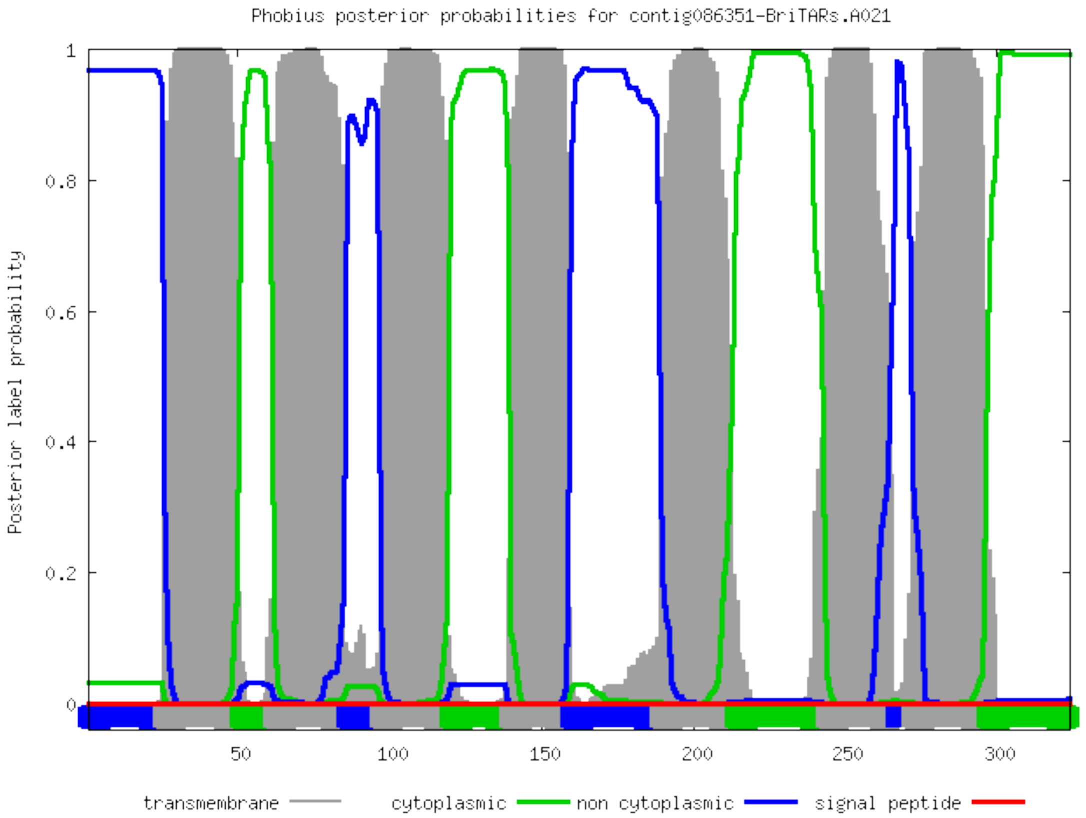

The probability data used in the plot is found here, and the gnuplot script is here.

Prediction of contig082565-BriTARs.A022\

```
ID   contig082565-BriTARs.A022\
FT   TOPO_DOM      1     29       NON CYTOPLASMIC.
FT   TRANSMEM     30     54
FT   TOPO_DOM     55     65       CYTOPLASMIC.
FT   TRANSMEM     66     83
FT   TOPO_DOM     84    102       NON CYTOPLASMIC.
FT   TRANSMEM    103    124
FT   TOPO_DOM    125    144       CYTOPLASMIC.
FT   TRANSMEM    145    164
FT   TOPO_DOM    165    183       NON CYTOPLASMIC.
FT   TRANSMEM    184    214
FT   TOPO_DOM    215    250       CYTOPLASMIC.
FT   TRANSMEM    251    271
FT   TOPO_DOM    272    282       NON CYTOPLASMIC.
FT   TRANSMEM    283    306
FT   TOPO_DOM    307    330       CYTOPLASMIC.
//
```

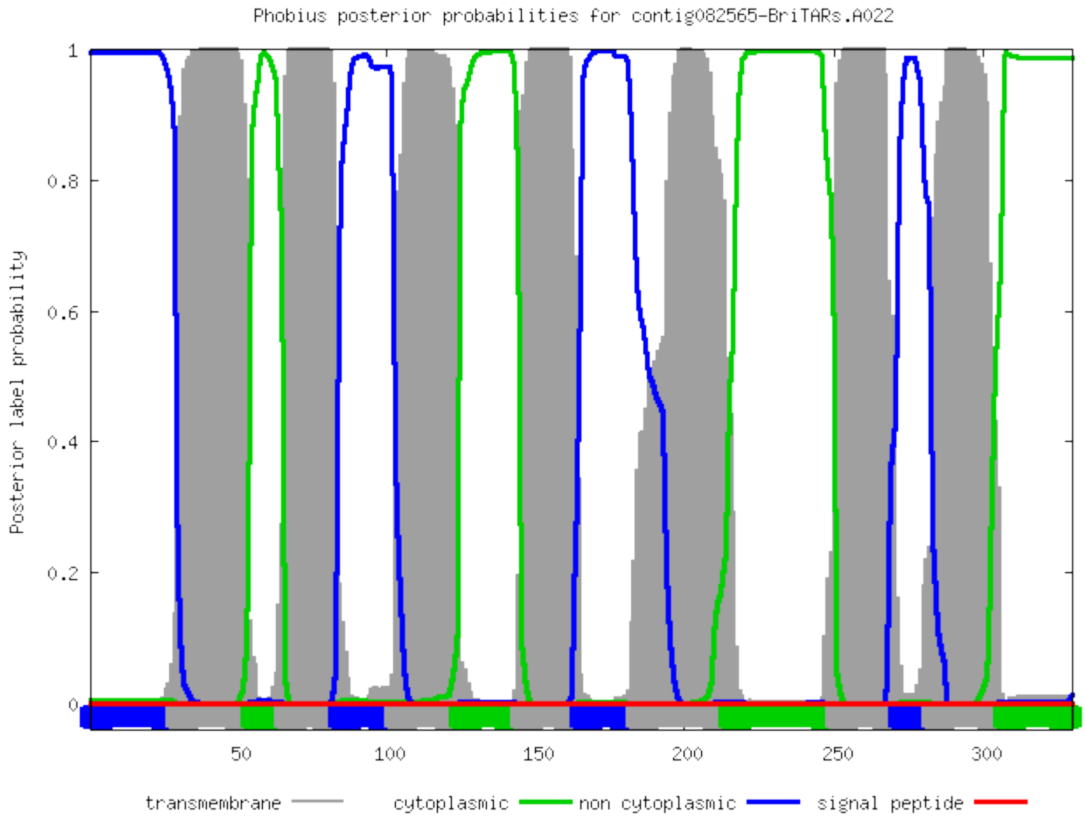

The probability data used in the plot is found here, and the gnuplot script is here.
