# Supplementary material for: Identification and characterization of cichlid TAAR genes and comparison with other teleost TAAR repertoires
Source: BMC Genomics. 2015 Apr 23;16(1):335. doi: 10.1186/s12864-015-1478-4 (PMC4415300; doi:10.1186/s12864-015-1478-4)
Supplement: Additional file 10: — Multiple alignment and LOGO presentation. Cichlid TAAR sequences were aligned with MAFFT [25]. The Logo was generated with Geneious [50]. [file 12864_2015_1478_MOESM10_ESM.pdf]

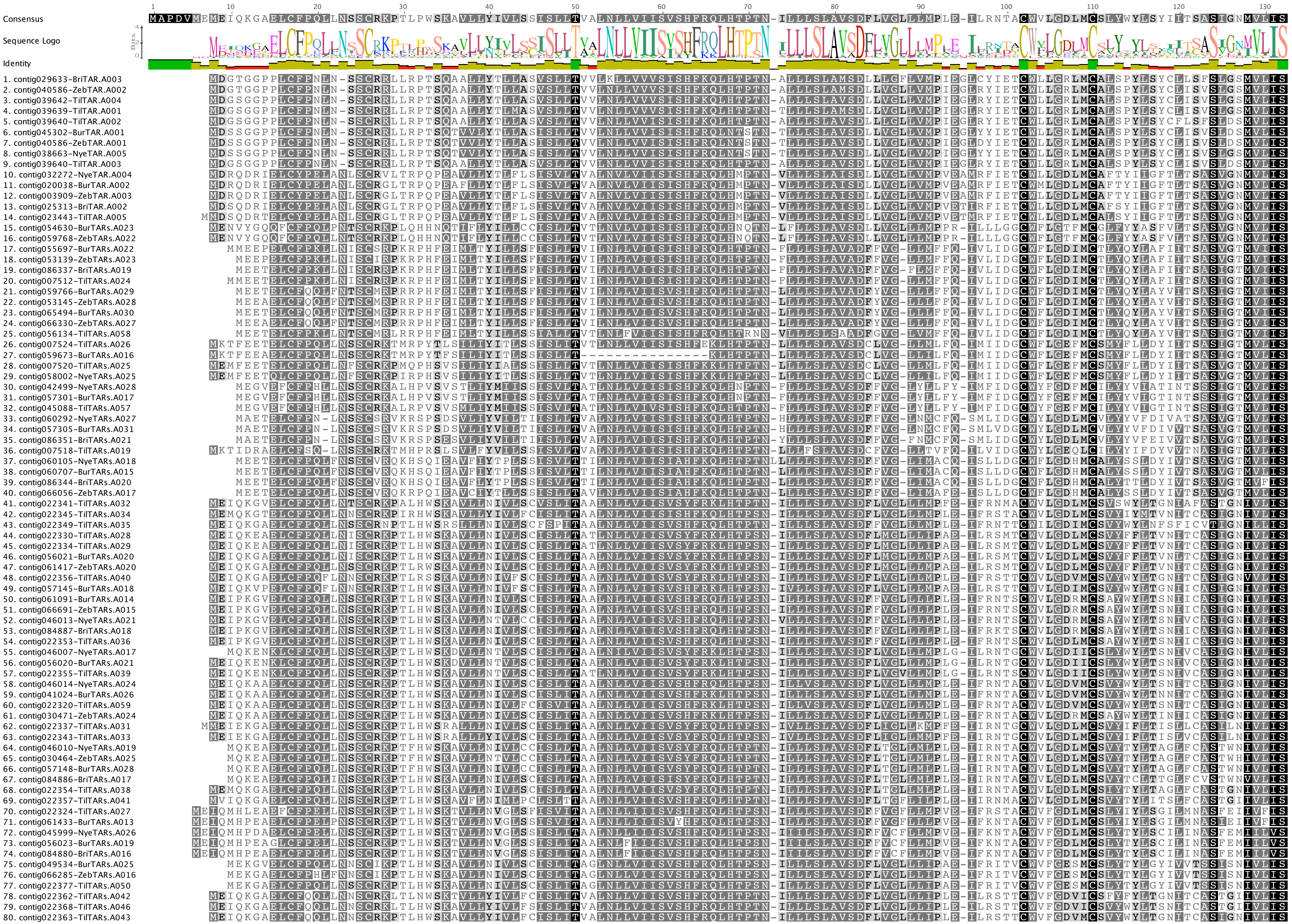

1
10
20
30
40
50
60
70
80
90
100
110
120
130
Consensus
Sequence Logo
Identity
1. contig029633-BriTAR.A003
2. contig040586-ZebTAR.A002
3. contig039642-TilTAR.A004
4. contig039639-TilTAR.A001
5. contig039640-TilTAR.A002
6. contig045302-BurTAR.A001
7. contig040586-ZebTAR.A001
8. contig038663-NyeTAR.A005
9. contig039640-TilTAR.A003
10. contig032272-NyeTAR.A004
11. contig020038-BurTAR.A002
12. contig003909-ZebTAR.A003
13. contig025313-BriTAR.A002
14. contig023443-TilTAR.A005
15. contig054630-BurTARs.A023
16. contig059768-ZebTARs.A022
17. contig055697-BurTARs.A022
18. contig053139-ZebTARs.A023
19. contig086337-BriTARs.A019
20. contig007512-TilTARs.A024
21. contig059766-BurTARs.A029
22. contig053145-ZebTARs.A028
23. contig065494-BurTARs.A030
24. contig066330-ZebTARs.A027
25. contig056134-TilTARs.A058
26. contig007524-TilTARs.A026
27. contig059673-BurTARs.A016
28. contig007520-TilTARs.A025
29. contig058002-NyeTARs.A025
30. contig042499-NyeTARs.A028
31. contig057301-BurTARs.A017
32. contig045088-TilTARs.A057
33. contig060292-NyeTARs.A027
34. contig057305-BurTARs.A031
35. contig086351-BriTARs.A021
36. contig007518-TilTARs.A019
37. contig060105-NyeTARs.A018
38. contig060707-BurTARs.A015
39. contig086344-BriTARs.A020
40. contig066056-ZebTARs.A017
41. contig022341-TilTARs.A032
42. contig022345-TilTARs.A034
43. contig022349-TilTARs.A035
44. contig022330-TilTARs.A028
45. contig022334-TilTARs.A029
46. contig056021-BurTARs.A020
47. contig061417-ZebTARs.A020
48. contig022356-TilTARs.A040
49. contig057145-BurTARs.A018
50. contig061091-BurTARs.A014
51. contig066691-ZebTARs.A015
52. contig046013-NyeTARs.A021
53. contig084887-BriTARs.A018
54. contig022353-TilTARs.A036
55. contig046007-NyeTARs.A017
56. contig056020-BurTARs.A021
57. contig022355-TilTARs.A039
58. contig046014-NyeTARs.A024
59. contig041024-BurTARs.A026
60. contig022320-TilTARs.A059
61. contig030471-ZebTARs.A024
62. contig022337-TilTARs.A031
63. contig022343-TilTARs.A033
64. contig046010-NyeTARs.A019
65. contig030464-ZebTARs.A025
66. contig057148-BurTARs.A028
67. contig084886-BriTARs.A017
68. contig022354-TilTARs.A038
69. contig022357-TilTARs.A041
70. contig022324-TilTARs.A027
71. contig061433-BurTARs.A013
72. contig045999-NyeTARs.A026
73. contig056023-BurTARs.A019
74. contig084880-BriTARs.A016
75. contig049534-BurTARs.A025
76. contig066285-ZebTARs.A016
77. contig022377-TilTARs.A050
78. contig022362-TilTARs.A042
79. contig022368-TilTARs.A046
80. contig022363-TilTARs.A043

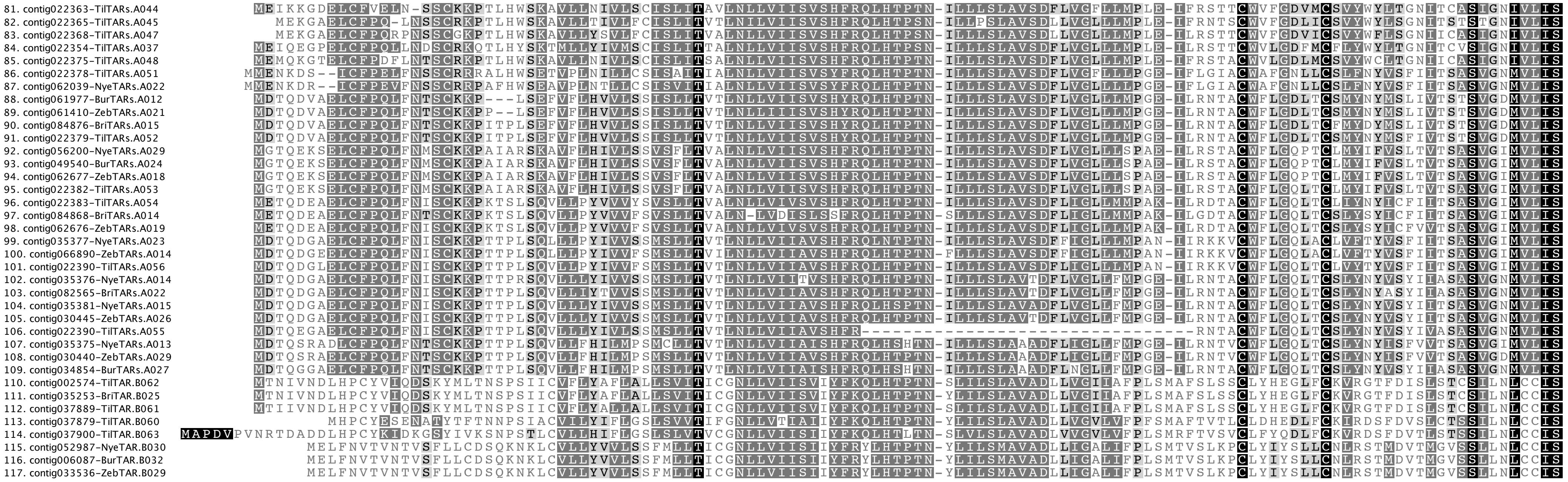
81. contig022363-TilTARs.A044
82. contig022365-TilTARs.A045
83. contig022368-TilTARs.A047
84. contig022354-TilTARs.A037
85. contig022375-TilTARs.A048
86. contig022378-TilTARs.A051
87. contig062039-NyeTARs.A022
88. contig061977-BurTARs.A012
89. contig061410-ZebTARs.A021
90. contig084876-BriTARs.A015
91. contig022379-TilTARs.A052
92. contig056200-NyeTARs.A029
93. contig049540-BurTARs.A024
94. contig062677-ZebTARs.A018
95. contig022382-TilTARs.A053
96. contig022383-TilTARs.A054
97. contig084868-BriTARs.A014
98. contig062676-ZebTARs.A019
99. contig035377-NyeTARs.A023
100. contig066890-ZebTARs.A014
101. contig022390-TilTARs.A056
102. contig035376-NyeTARs.A014
103. contig082565-BriTARs.A022
104. contig035381-NyeTARs.A015
105. contig030445-ZebTARs.A026
106. contig022390-TilTARs.A055
107. contig035375-NyeTARs.A013
108. contig030440-ZebTARs.A029
109. contig034854-BurTARs.A027
110. contig002574-TilTAR.B062
111. contig035253-BriTAR.B025
112. contig037889-TilTAR.B061
113. contig037879-TilTAR.B060
114. contig037900-TilTAR.B063
115. contig052987-NyeTAR.B030
116. contig006087-BurTAR.B032
117. contig033536-ZebTAR.B029

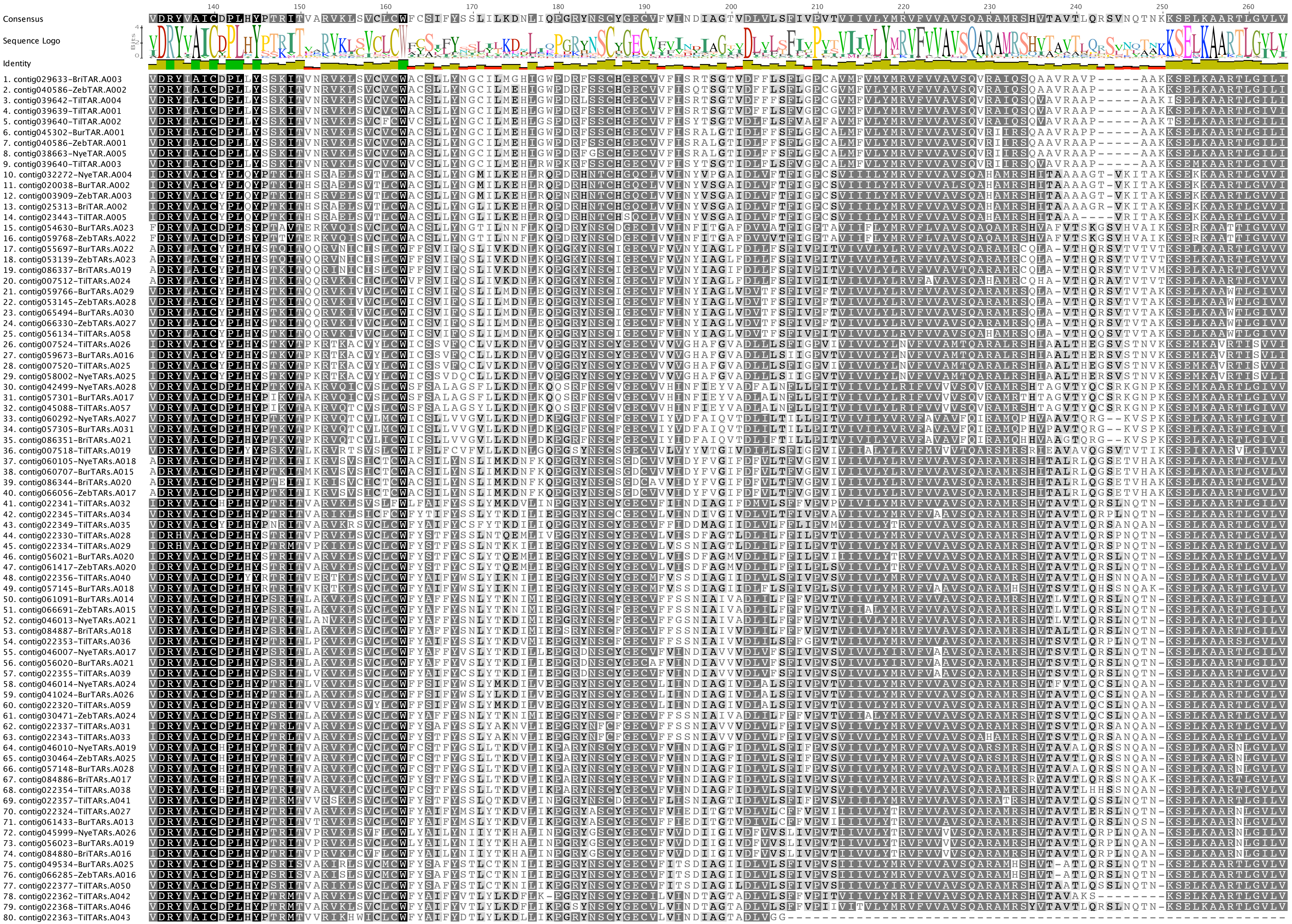

140
150
160
170
180
190
200
210
220
230
240
250
260
Consensus
VDRYVAICDPLHYPTRITVARVKLSVCLCWFCSIFYSSLILKDNLIQPGRYNSCYGECVFVINDIAGTVDLVLSFIVPVTVIIVLYMRVFVVAVSQARAMRSHVTAVTLQRSVNQTNKKSELKAARTLGVLV
Sequence Logo
Bits
Identity
1. contig029633-BriTAR.A003
2. contig040586-ZebTAR.A002
3. contig039642-TilTAR.A004
4. contig039639-TilTAR.A001
5. contig039640-TilTAR.A002
6. contig045302-BurTAR.A001
7. contig040586-ZebTAR.A001
8. contig038663-NyeTAR.A005
9. contig039640-TilTAR.A003
10. contig032272-NyeTAR.A004
11. contig020038-BurTAR.A002
12. contig003909-ZebTAR.A003
13. contig025313-BriTAR.A002
14. contig023443-TilTAR.A005
15. contig054630-BurTARs.A023
16. contig059768-ZebTARs.A022
17. contig055697-BurTARs.A022
18. contig053139-ZebTARs.A023
19. contig086337-BriTARs.A019
20. contig007512-TilTARs.A024
21. contig059766-BurTARs.A029
22. contig053145-ZebTARs.A028
23. contig065494-BurTARs.A030
24. contig066330-ZebTARs.A027
25. contig056134-TilTARs.A058
26. contig007524-TilTARs.A026
27. contig059673-BurTARs.A016
28. contig007520-TilTARs.A025
29. contig058002-NyeTARs.A025
30. contig042499-NyeTARs.A028
31. contig057301-BurTARs.A017
32. contig045088-TilTARs.A057
33. contig060292-NyeTARs.A027
34. contig057305-BurTARs.A031
35. contig086351-BriTARs.A021
36. contig007518-TilTARs.A019
37. contig060105-NyeTARs.A018
38. contig060707-BurTARs.A015
39. contig086344-BriTARs.A020
40. contig066056-ZebTARs.A017
41. contig022341-TilTARs.A032
42. contig022345-TilTARs.A034
43. contig022349-TilTARs.A035
44. contig022330-TilTARs.A028
45. contig022334-TilTARs.A029
46. contig056021-BurTARs.A020
47. contig061417-ZebTARs.A020
48. contig022356-TilTARs.A040
49. contig057145-BurTARs.A018
50. contig061091-BurTARs.A014
51. contig066691-ZebTARs.A015
52. contig046013-NyeTARs.A021
53. contig084887-BriTARs.A018
54. contig022353-TilTARs.A036
55. contig046007-NyeTARs.A017
56. contig056020-BurTARs.A021
57. contig022355-TilTARs.A039
58. contig046014-NyeTARs.A024
59. contig041024-BurTARs.A026
60. contig022320-TilTARs.A059
61. contig030471-ZebTARs.A024
62. contig022337-TilTARs.A031
63. contig022343-TilTARs.A033
64. contig046010-NyeTARs.A019
65. contig030464-ZebTARs.A025
66. contig057148-BurTARs.A028
67. contig084886-BriTARs.A017
68. contig022354-TilTARs.A038
69. contig022357-TilTARs.A041
70. contig022324-TilTARs.A027
71. contig061433-BurTARs.A013
72. contig045999-NyeTARs.A026
73. contig056023-BurTARs.A019
74. contig084880-BriTARs.A016
75. contig049534-BurTARs.A025
76. contig066285-ZebTARs.A016
77. contig022377-TilTARs.A050
78. contig022362-TilTARs.A042
79. contig022368-TilTARs.A046
80. contig022363-TilTARs.A043

81. contig022363-TilTARs.A044	VDRYVAICDPLHYPTRMTVVRIKHWICLCWFYAIFYDTLYLKDLLIKPGRYNSCYGECVLVINDIAGTADLVLSFCVPISVIIVLYMRVFLVAVSQARAMRSHVTTVTLQRSLNQTN-KSELKAARTLGILV
82. contig022365-TilTARs.A045	VDRYVAICDPLHYPTRMTVVRVKLSVCLCWFYYIFYISFYLKDILIEPGRYNSCKGECVLVINDIAGIIDLVIAFIVPVSVIIVLYMRVFVVAVSQARAMRSCVRAVTLQHSLNQAN-KSELKAARTLGILV
83. contig022368-TilTARs.A047	ADRYVAICDPLHYPTRMTVVRVKLNVSLCWFYYVFYISLYLKDILIKPGRYNSCYGECVPVINDIAGIIDLVLTFIVPVSVIIVLYMRVFVAAVSQARAMRSHVTAITFQRSLNQAN-KSELKAARTLGVLV
84. contig022354-TilTARs.A037	VDRYVAICDPLHYSTRITVAKVKLSVCLCWFYAIFSISLYAKDYLIESGRYNSCYGECAFVINDIARTIDLVLSFIFPITVIIVLYMRVFVVAVSQARAMRSHVTAVTLQCSLNQAN-KSELKAARTLGILV
85. contig022375-TilTARs.A048	IDRYVAICDPLHYPTRMTVARVRVCVCLCWFFAIFYSSLYTTDFLIDPGRYNSCYGECVFVISAIAGIFDLVVSFFVSVTVIIVLYMRVFVVAVSQARAMRSHVTTVTLQRSLNQTN-KSELKAARTLGILV
86. contig022378-TilTARs.A051	VDRYVAICYPLHYSTRITVTRVKRSVCLCWLCCVLYSSALLKDELIQPGGHNSCYGECVFVINFIAGTVDLLLTFIVPISVIVVLYMRVFVVAVSQARAMRSHVTAVTLQLSVNLTTKKSELKAGRTLGVLI
87. contig062039-NyeTARs.A022	VDRYVAICYPLHYSTRITVTRVKRSVCLCWLCCVLYSSVLLKDELIQPGRHNSCYGECVFVINFIAGTVDLLLTFIVPISVIVVLYIRVFAVAVSQARAMRS-------QLSVNLTTKKSELKAGRTLGVLI
88. contig061977-BurTARs.A012	IDRYVAICDPLHYPTRITDRRVKLCVCLCWLCSVFYSSLFVKDDLTQPGKHNSCYGECTIVVDLITGTIDLLLTFFVPVTVIVVLYLRVFVVAVSQARAMRSHVTVAALQLSVTLTTKKSELKAARTLGVLV
89. contig061410-ZebTARs.A021	IDRYVAICDPLHYPTRITDRRVKLCVCLCWLCSVFYSSLFVKDDLTQPGKHNSCYGECTIDVDLITGTIDLLLTFFVPVTVIVVLYLRVFVVAVSQARAMRSHVTVAALQLSVTLTTKKSELKAARTLGVLV
90. contig084876-BriTARs.A015	IDRYVALCDPLHYPTRITDRRVKLSVCLCWLSSVFYSSLFVKDDLTHSGKHNSCYGECTIVVDLITGTIDLLLTFFVPVTVIVVLYLRVFVVAVSQARAMRSHVTAAALQLSVTLTTKRSELKAARTLGVLV
91. contig022379-TilTARs.A052	IDRYLAICDPLHYPTRITDRRVKLSICLCWLSSVFYSSLFVKDDLTQPGKHNSCYGECTIVVDLITGIIDLLLTFFVPVTVIVVLYLRVFVVAVSQARAMRSHVTATALQLSVTLTTKKSELKAARTLGVLV
92. contig056200-NyeTARs.A029	ADRYVAICDPLNYPIRITDRRVQLCVCLCWLCSIAFSCFLVRDDLHQKRKQNSCYGKCVVVVQYIAGVVDLILTFIVPVTVIIVLYMRVFVVAVSQARSMRSHATAVRNHLPMTLTK-KSELKAARTLGVLV
93. contig049540-BurTARs.A024	ADRYVAICDPLNYPIRITDRRVKLCVCLCWLCSIAFSCFLVRDDLHQKRKQNSCYGKCVVVVQYIAGVVDLILTFIVPVTVIIVLYMRVFVVAVSQARSMRSHATAVRNHLPMTLTK-KSELKAARTLGVLV
94. contig062677-ZebTARs.A018	ADRYVAICDPLNYPIRITDRRVQLCVCLCWLCSITFSCFLVRDDLHQRRKQNSCYGKCVVVVQYIAGVVDLILTFIVPVTVIIVLYMRVFVVAVSQARSMRSHATAVRNHLPMTLTK-KSELKAARTLGVLV
95. contig022382-TilTARs.A053	ADRYVAICDPLNYPIRITDRRVQLCVCLCWLCSIAFSCFLVRDDLHQTRKQNSCYGKCVVVVQYIAGVVDLILTFIVPVSVIIVLYMRVFVVAVSQARAMRSHVTAVTSHLPVTLTK-KSELKAARTLGVLV
96. contig022383-TilTARs.A054	VDRYVAICDPLHYPTRITEKRVKLCVCLCWLCSVFYNILFIKDDLLQREQHTSCYGECVFVIDYIVGTTDIVVTFIAPVTVIVILYMRVFVVAVSQARAMRSHVTAVTLQLSVTLTAKKSELKAARTLGVLV
97. contig084868-BriTARs.A014	VDRYVAICDPLHYPTRITERRVKLCVCLCWLCSVIYNILFIKNDLLQGERHTSCYGECVFVIDYIVGTTDIVLTFIAPVTVIVVLYMRVFVVAVSQARAMRSHVTAVTLQLSVTLTAKKSEIKAARTLGVLV
98. contig062676-ZebTARs.A019	VDRYVAICDPLHYPTRITDRRVKLCVCLCWLCSVFYNILFIKDDLLQRERHTSCYGECVFVIDYIVGTTDIVLTFIAPVTVIVFLYMRVFVVAVSQARAMRSHVTAVTLQLSVTLTAKKSELKAARTLGVLV
99. contig035377-NyeTARs.A023	IDRYAAICDPLHYPTRITRERVKLCVCLCWLCSVFYNILFIKNDLLQRERHTSCYGECKILVDYIAGTTDIVLTFIAPVTVIIVLYMRVFVVAVSQARAMRSQVTAVTLQLSVTLTTKKSELKAARTLGVLV
100. contig066890-ZebTARs.A014	VDRYAAICDPLHYPTRITRERVKLCVCLCWLCSVIYNILLIKDELVQPGQHNSCYGECKILVDYIAGTTDIVLTFIAPITVIIVLYMRVFVVAVSQARTMRSQVTAVTLQLSVTLTTKKSELKAARTLGVLV
101. contig022390-TilTARs.A056	VDRYVAICDPLHYPTRITHGRVKLCVCLCWLCSVIYNILLIKDELVKPGQHISCYGECKILVDYIAGTTDIVLTFIIPVTVIIVLYMRVFVVAVSQARAMRSHVTAVTMQLSVTLTTKKSELKAARTLGVLV
102. contig035376-NyeTARs.A014	VDRYVAICDPLHYPTRITERRVKLCVCLCWLCSVFYSYVILIDDLSQPGKHKSCYGRCIIFIEFIAGFVDLVLAFIIPLTVIIVLYMRVFVVAVSQARAMRSQVTAVTLQLSVTLTATKSELKAARTLGVLV
103. contig082565-BriTARs.A022	VDRYVAICDPLHYPTRITERRVKLCVCLCWLCSVFYSYVILIDDLSQPGKHKSCYGKCIIFIEFIAGFVDLVLAFIIPLTVIIVLYMRVFVVAVSQARAMRSQVTAVTLQLSVTLTAKKSELKAARTLGVLV
104. contig035381-NyeTARs.A015	VDRYVAICDPLHYPTRITERRVKLCVCLCWLCSVFYCYVILIDDLSQPGKHNSCYGKCIIIIEFIAGFVDLVLSFIIPLTVIIVLYMRVFVVAVSQARAMRSQVTAVTLQLSVTLTAKKSELKAAKTLGVLV
105. contig030445-ZebTARs.A026	VDRYVAICDPLHYPTRITERRVKLCVCLCWLCSVFYCYVILIDDLSQPGKHNSCYGRCIIIIEFIAGFVDLFLSFIIPLTVIIVLYMRVFVVAVSQARAMRSQVTAVTLQLSVTLTAKKSELKAARTLGVLV
106. contig022390-TilTARs.A055	VDRYVAICDPLHYPSRITDKRVKLCVCLCWLCSVFYSYVILIDDLSQPGKHNSCYGKCIIFIEFIAGFVDLVLSFIIPLTVIVVLYMRVFVVAVSQARAMRSHVTAVTLQLSVTLTAKKSELKAARTLGVLV
107. contig035375-NyeTARs.A013	VDRYVAICDPLHYPTRITERRVKLCVCLCWLCSVFYCYMIVRDDLSQPGKHNSCFGECIIFVEFIAGFVDLVLSFIIPVTVIVFLYMRVFVVAVSQARAMRSQVTAVTLQLSVTLTAKKSELKAARTLGVLV
108. contig030440-ZebTARs.A029	VDRYVAICDPLHYPTRITERRVKLCVCLCWLCSVFYCYMIVRDDLSQPGKHNSCFGECIIFVEFIAGFVDLVLSFIIPVTVIVFLYMRVFVVAVSQARAMRSQVTAVTLQLSVTLTATKSELKAARTLGVLV
109. contig034854-BurTARs.A027	VDRYVAICDPLHYPTRITEGKVKLCVCLCWLCSVLYSYVILIDDLSQPGKHKSCYGKCIIFIEFIAGFVDLFISFIIPLTVIIVLYMRVFVVAVSQARAMRSQVTTVTLQLSVTLTATKSELKAARTLGVLV
110. contig002574-TilTAR.B062	VDRYHAVCQPLTYQTKISHRVVVFMILMSWGVSGIIGISVTIAGFNNEKCEESCLIDVLMESTVGP-----MLSFYLPVTIMLCIYLKIFFVALKQASSIQN--TTKCGQT-----ASIMERKATKTLAIVL
111. contig035253-BriTAR.B025	IDRYHAVCQPLTYQTKISPRVVVFMILMSWGVSGIIGISVTIAGFNNEKCEESCLIDVLIESTVGP-----MLSFYLPVTMMLFIYLKIFFVALRQARRIQN--TTKCGQT-----ASIMERKATKTLAIVL
112. contig037889-TilTAR.B061	IDRYHAVCQPLTYQTKISHRAVVFMILMSWGVSGIIGISVTIAGLNNEKYEESFLIDVLMESTVGP-----MLSFYLPVIIMLCIYLKIFFVARRQARSIQN--TTKCGQT-----ASMMERKATKTLAIVL
113. contig037879-TilTAR.B060	IERYYAVCQPLNYRSKINTQVTMFMILVSWGISVLIGICIIIAGFSQGMCEEVCSVDVVIANTMGP-----VFSFYLPAIIMLCIYLKIFLVAQKQVSTIQNAICTKSGAT-----ISKLERKATKTLSIVM
114. contig037900-TilTAR.B063	IDRYYAVCRPLTYRSKINVHVVVIMILVSWSVSILVAVGFLIAGLNHEKCKEGWISELLLANILGP-----VLSFYLPVIIMLCIYLKIFLVAEKHARSIQN---TKCGATV----VSKMEKKATKTLATVM
115. contig052987-NyeTAR.B030	VDRYYAVCHPLIYKTKITDCVAMKMGLGSWAVAILCGIFVFLLFFILDECDTSCVFALIAAS---------VVVYYIPTIVLLFMYTKILVVALRQARSIHNTISQNTKSKA----VSSTERKATKTLTIVI
116. contig006087-BurTAR.B032	VDRYYAVCHPLIYKTKITDCVAMKMGLGSWAVAILCGIFVFLLFFILDECDTSCLFALIAAS---------VVVYYIPTIVLLFMYTKILVVALRQARSIHNTISQNTKSKA----VSSTERKATKTLTIVI
117. contig033536-ZebTAR.B029	VDRYYAVCHPLIYKTKITDCVAMKMGLGSWAVAILCGIFVFLLFFILDECDTSCVFALIAAS---------VVVYYIPTIVLLFMYTKILVVALRQARSIHNTISQNTKSKA----VSSTERKATKTLTIVI

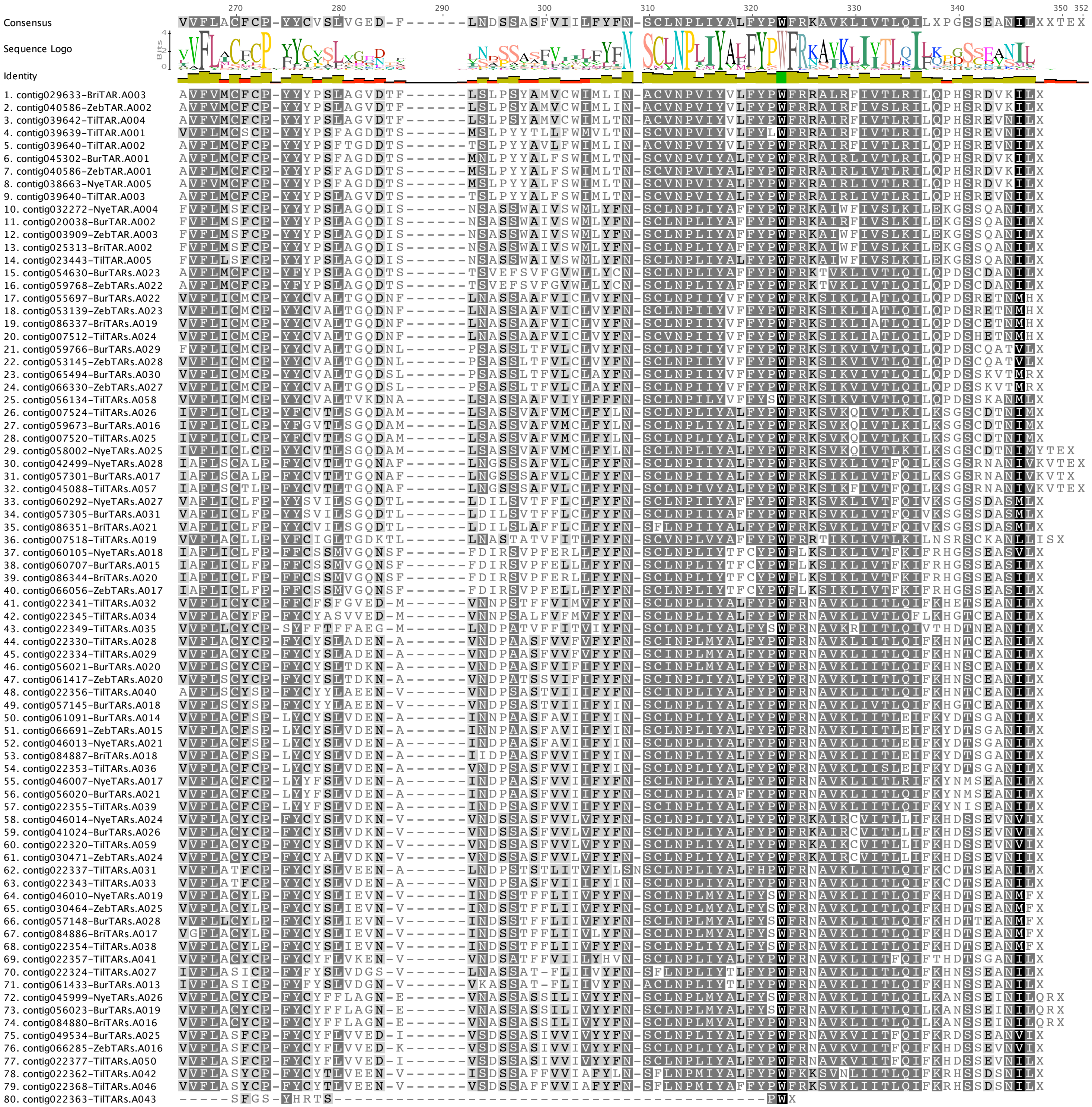
270
280
290
300
310
320
330
340
350
352
Consensus
Sequence Logo
Bits
Identity
1. contig029633-BriTAR.A003
2. contig040586-ZebTAR.A002
3. contig039642-TilTAR.A004
4. contig039639-TilTAR.A001
5. contig039640-TilTAR.A002
6. contig045302-BurTAR.A001
7. contig040586-ZebTAR.A001
8. contig038663-NyeTAR.A005
9. contig039640-TilTAR.A003
10. contig032272-NyeTAR.A004
11. contig020038-BurTAR.A002
12. contig003909-ZebTAR.A003
13. contig025313-BriTAR.A002
14. contig023443-TilTAR.A005
15. contig054630-BurTARs.A023
16. contig059768-ZebTARs.A022
17. contig055697-BurTARs.A022
18. contig053139-ZebTARs.A023
19. contig086337-BriTARs.A019
20. contig007512-TilTARs.A024
21. contig059766-BurTARs.A029
22. contig053145-ZebTARs.A028
23. contig065494-BurTARs.A030
24. contig066330-ZebTARs.A027
25. contig056134-TilTARs.A058
26. contig007524-TilTARs.A026
27. contig059673-BurTARs.A016
28. contig007520-TilTARs.A025
29. contig058002-NyeTARs.A025
30. contig042499-NyeTARs.A028
31. contig057301-BurTARs.A017
32. contig045088-TilTARs.A057
33. contig060292-NyeTARs.A027
34. contig057305-BurTARs.A031
35. contig086351-BriTARs.A021
36. contig007518-TilTARs.A019
37. contig060105-NyeTARs.A018
38. contig060707-BurTARs.A015
39. contig086344-BriTARs.A020
40. contig066056-ZebTARs.A017
41. contig022341-TilTARs.A032
42. contig022345-TilTARs.A034
43. contig022349-TilTARs.A035
44. contig022330-TilTARs.A028
45. contig022334-TilTARs.A029
46. contig056021-BurTARs.A020
47. contig061417-ZebTARs.A020
48. contig022356-TilTARs.A040
49. contig057145-BurTARs.A018
50. contig061091-BurTARs.A014
51. contig066691-ZebTARs.A015
52. contig046013-NyeTARs.A021
53. contig084887-BriTARs.A018
54. contig022353-TilTARs.A036
55. contig046007-NyeTARs.A017
56. contig056020-BurTARs.A021
57. contig022355-TilTARs.A039
58. contig046014-NyeTARs.A024
59. contig041024-BurTARs.A026
60. contig022320-TilTARs.A059
61. contig030471-ZebTARs.A024
62. contig022337-TilTARs.A031
63. contig022343-TilTARs.A033
64. contig046010-NyeTARs.A019
65. contig030464-ZebTARs.A025
66. contig057148-BurTARs.A028
67. contig084886-BriTARs.A017
68. contig022354-TilTARs.A038
69. contig022357-TilTARs.A041
70. contig022324-TilTARs.A027
71. contig061433-BurTARs.A013
72. contig045999-NyeTARs.A026
73. contig056023-BurTARs.A019
74. contig084880-BriTARs.A016
75. contig049534-BurTARs.A025
76. contig066285-ZebTARs.A016
77. contig022377-TilTARs.A050
78. contig022362-TilTARs.A042
79. contig022368-TilTARs.A046
80. contig022363-TilTARs.A043

```
81. contig022363-TilTARs.A044    VVFLLCFCP-YYCVSLVRD---------RNSSVAPNLMSVFFLN-SCLNPLIYTMFYPWFRKAVKLIVTLQILQTGSCEVSILX
82. contig022365-TilTARs.A045    VVFLASFCP-FYCYFFVGEN-V------VSDSSAFFVIIAFYFN-SFLNPMIYALFYPWFRNAVKLIITLQIFKRHSSDTNILX
83. contig022368-TilTARs.A047    IVFLACYCP-FFCYTLVEEK-V------VSDSFVFFLIIAVYFN-SFLNPLIYAFFYPWFRNAVKLIITLQIFKRHSSDANILX
84. contig022354-TilTARs.A037    VVFLLCFCP-YYCVSLIRED---------FSSVAPIVISLFFSN-SCLNPVIYALFYPWFRKALKLIVTLQILHSGSCEVSILX
85. contig022375-TilTARs.A048    VVFLLCFCP-YYCVSLVRDE---------NSSIASTVISVFFLN-SCLNPLIYAMFYPWFRKAVKLTVTLQILRTGSCEVNILX
86. contig022378-TilTARs.A051    VVFLMCFCP-YYCASFAVED-S------LSESSTSLVRYLFYFN-SCLNPVIYALFYPWFRKAIKHIVMLQIFHTGSREANILX
87. contig062039-NyeTARs.A022    VVFLMCFCP-YYCVSLAGED-S------LSESSTSIVRYLFYFN-SCLNPLIYALFYPWFRKAIKHIVMLQIFHTGSHEDNILX
88. contig061977-BurTARs.A012    VVFLLCFCP-YYCVTLARDD-L------LNSSSVSFLLYLFYFN-SCLNPLIYALLYPWFRKAVKLIISLHILQPGSCEISILX
89. contig061410-ZebTARs.A021    VVFLLCFCP-YYCVTLARDD-L------LNSSSVSFLLYLFYFN-SCLNPLIYALLYPWFRKAVKLIISLHILQPGSCEISILX
90. contig084876-BriTARs.A015    VVFLLCFCP-YYCVTLARDD-L------LNSSSVSFLLYLFYFN-SCLNPLIYALLYPWFRKAVKLIISLHILQPGSCEISILX
91. contig022379-TilTARs.A052    VVFLLCFCPYYYCVTLARDD-P------LNSSSVSFVLYLFYFN-SCLNPLIYALFYPWFRKAVKLIISLHILQPGSCEISILX
92. contig056200-NyeTARs.A029    LVFLMCFCP-YYCVSLVGEE-F------INSSSASFVAYLFGLN-SCLNPLIYAMFYPWFRKAVKLVVTLQILQPGSSEVSILX
93. contig049540-BurTARs.A024    LVFLMCFCP-YYCVSLVGEE-F------INSSSASFVAYLFGLN-SCLNPLIYAMFYPWFRKAVKLVVTLQILQPGSSEVSILX
94. contig062677-ZebTARs.A018    LVFLMCFCP-YYCVSLVGEE-F------INSSSASFVAYLFGLN-SCLNPLIYAMFYPWFRKAVKLVVTLQILQPGSSEVSILX
95. contig022382-TilTARs.A053    LVFLMCFCP-YYCVSLVGEE-F------INSSSASFVAYLFGLN-SCLNPLIYAMFYPWFRKAVKLVVTLQILQPGSCEVSILX
96. contig022383-TilTARs.A054    LVFLLCFCP-YYIVSLLGNE-L------FNSSSASIVIYLYYFN-SCLNPLIYAMFYPWFRKAVKLVVTLQILQPGSCEVSILX
97. contig084868-BriTARs.A014    LVFLLGFCP-YYIVSLLGNE-L------FNSSSASIAIYLYYFN-SCLNPLIYAMFYPWFRKAVKFVVTLQILQPGSCEVSILX
98. contig062676-ZebTARs.A019    LVFLLYFCP-YYIVSFFGYE-L------LNSSSASIVIYLYYFN-SCLNPLIYAMFYPWFRKAVKLVVTLQILQPGSCEVSILX
99. contig035377-NyeTARs.A023    LVFLICFCP-HYCIFVFGGK-V------LNSSSATIVIYLYYFN-SCLNPLIYAMFYPWFRKAVKLIVTLQILQPGSCEVSILX
100. contig066890-ZebTARs.A014   LVFLICFCP-HYCIFVFGGK-V------LNSSSATIVIYLYYFN-SCLNPLIYAMFYPWFRKAVKLIVTLQILQPGSCEVSILX
101. contig022390-TilTARs.A056   LVFLICFCP-HYCIFVFGGK-L------LSSSSATIVIYLYYFN-SCVNPLIYAMFYPWFRKAVKLTVTLQILQPGSCEVSILX
102. contig035376-NyeTARs.A014   LVFLLCFCS-YYIISLFGNE-L------LNSSSASIVIYLYYFN-SCLNPLIYAMFYPWFRKAVKLIVTLQILQPGSCEISIMX
103. contig082565-BriTARs.A022   LVFLLCFCS-YYIISLFGNE-L------LNSSSASIVIYLYYFN-SCLNPLIYAMFYPWFRKAVKLIVTLQILQPGSCEVSILX
104. contig035381-NyeTARs.A015   LVFLLCFCS-YFIVSLFGNE-L------LNSSSASIVIYLYYFN-SCLNPLIYAMFYPWFRKAVKLIVTLQILQPGSCEVIILX
105. contig030445-ZebTARs.A026   LVFLLCFCS-YYFISLFGND-L------LNSSSASIVIYLYYFN-SCLNPLIYAMFYPWFRKAVKLIVTLQILQPGSCEVSVLX
106. contig022390-TilTARs.A055   LVFLICFCP-YYIVSLFGDE-F------LNSSSASIVIYLYYFN-SCVNPLIYAMFYPWFRKAVKLIVTLQILQPDSCEVSILX
107. contig035375-NyeTARs.A013   LVFLICFCP-YYIVSLFGDE-F------LNSASASIVFYFFLLN-SCLNPLIYAMFYPWFRKAVKLIMTLQILQPGSCEVSILX
108. contig030440-ZebTARs.A029   LVFLICFCP-YYIVSLFGDE-F------LNSASASIVFYFFLLN-SCLNPLIYAMFYPWFRKAVKLIMTLQILQPGSCEVSILX
109. contig034854-BurTARs.A027   LVFLLCFCP-YYIVSLFGDE-V------LNSASESIAFYFFLLN-SCLNPLIYAMFYPWFRKAVKLIVTLQILQPGSCEVSILX
110. contig002574-TilTAR.B062    GVFIFCWSPCFLCITFPPFTSD-----SVPVPVIETLNWLALAN-SMLNPFIYAFFYRWFRSAIRMIICGKIFQRDFANTNMTX
111. contig035253-BriTAR.B025    GVFIFCWSPFFLSITFPPFTSD-----SVPVPVIETLNWLTLAN-SMLNPFIYAFFYSWFRSAIRMIICGKIFQGDFANTNMTX
112. contig037889-TilTAR.B061    GVFILCWSPFFLCITFPPFTSDYQYQLQVPVPVIETLNWLTLAN-SMLNPFIYAFFYSWFRSAIRMIICGKIFQGDFANTKLTX
113. contig037879-TilTAR.B060    GVFLLCLSPYFICVVFQPLAHN-----PPPIPVIETLNWLTLSN-SMLNPLIYAFFYSWFRSAYRLIVSGTILHCNLANSKLFX
114. contig037900-TilTAR.B063    GVFVMCWLPFFFCVTLQLLGHI-----LIPLPVYETLNWLALCN-SMLNPFIYAFFX
115. contig052987-NyeTAR.B030    GIFLIFWVPLFLSYSFVPLDSF------ILYVLLEPFNWFAISN-SMLNPFIYAFFYTWFRRAFKMIISGKIFQGDVTNIKLHX
116. contig006087-BurTAR.B032    GIFLIFWVPLFLSYSFVPLDSF------ILYVLLEPFNWFAISN-SMLNPFIYAFFYTWFRRAFKMIISGKIFQGDVTNIKLHX
117. contig033536-ZebTAR.B029    GIFLICWVPLFLSYSFVPLDSF------ILYVLLEPFNWFAISN-SMLNPFIYAFFYTWFRRAFKMIISGKIFQGDVTNIKLHX
```
