# Supplementary material for: Identification and characterization of cichlid TAAR genes and comparison with other teleost TAAR repertoires
Source: BMC Genomics. 2015 Apr 23;16(1):335. doi: 10.1186/s12864-015-1478-4 (PMC4415300; doi:10.1186/s12864-015-1478-4)
Supplement: Additional file 11: — N-glycosylation sites, as predicted by the NetNGly Server [ 38 ], for each cichlid TAAR. [file 12864_2015_1478_MOESM11_ESM.pdf]

NetNGlyc 1.0 Server - prediction results

Technical University of Denmark

Asn-Xaa-Ser/Thr sequons in the sequence output below are highlighted in **blue**.

Asparagines predicted to be N-glycosylated are highlighted in **red**.

Output for 'contig039639-TilTAR.A001'

```
##########################################################################################

    Warning: This sequence may not contain a signal peptide!!

    Proteins without signal peptides are unlikely to be exposed to
    the N-glycosylation machinery and thus may not be glycosylated
    (in vivo) even though they contain potential motifs.

    SignalP-NN euk predictions are as follows:

  # name                 Cmax  pos ?  Ymax  pos ?  Smax  pos ?  Smean ?  D     ?

    SignalP output is explained at http://www.cbs.dtu.dk/services/SignalP/output.html

##########################################################################################

Name:  contig039639-TilTAR.A001     Length:  326
MDGSGGPPLCFPNLNSSCRRLLRPTSQAALLYTLMASVSLLTVVLNLLVVVSISHFKQLHTPTNTLLLSLAMSDLLVGLL      80
VMPIEGLRYIETCWLLGRLMCALSPYLSYCLISVSLGSMVLISVDRYIAICDPLLYSSKITVNRVKLSVCVCWACSLLYN     160
GCILMEHLGWPDRFSSCHGECVVFISRTSGTVDFFLSFVGPCGVMFVLYMRVFVVAVSQVRAIQSQVAVRAAPAAKKSEL     240
KAARTLGIVIVVFLMCSCPYYYPSFAGDDTSMSLPYYTLLFWMVLTNSCVNPVIYVLFYLWFRRAIRFIVTLRILQPHSR     320
EVNILX
..............N.................................................................      80
................................................................................     160
................................................................................     240
................................................................................     320
......                                                                               400

(Threshold=0.5)
----------------------------------------------------------------------
SeqName      Position  Potential   Jury    N-Glyc
                      agreement result
----------------------------------------------------------------------
contig039639-TilTAR.A001   15 NSSC   0.6959    (9/9)   ++
----------------------------------------------------------------------
```

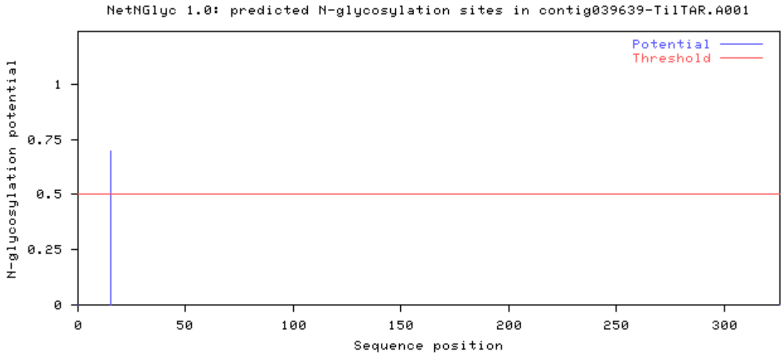

Graphics in PostScript

Output for 'contig039640-TilTAR.A002'

```
##########################################################################################

    Warning: This sequence may not contain a signal peptide!!

    Proteins without signal peptides are unlikely to be exposed to
    the N-glycosylation machinery and thus may not be glycosylated
    (in vivo) even though they contain potential motifs.

    SignalP-NN euk predictions are as follows:

  # name                 Cmax  pos ?  Ymax  pos ?  Smax  pos ?  Smean ?  D     ?

    SignalP output is explained at http://www.cbs.dtu.dk/services/SignalP/output.html

##########################################################################################

Name:  contig039640-TilTAR.A002     Length:  326
MDGSGGPPLCFPNLNSSCRRLLRPTSQAALLYTLLASVSLLTVVLNLLVVISISHFKQLHTPTNALLLSLAVSDLLLGLL      80
```

```
VMPIEGLRYIETCWLLGRLMCALSPYLSYCFLSFSLDSMVLISVDRYIAICDPLLYSSKITVNRVKLSVCFCWVCSLLYN     160
GCILMEHLGSPDRFSSCHGECVVFISYTSGTVDLFLSFVAPFAVMFVLYMRVFVVAVSQVRAIQSQVAVRAAPAAKTSEL     240
KAARTLGILIAVFLMCFCPYYYPSFTGDDTSTSLPYYAVLFWIMLINACVNPVIYVLFYPWFRRAIRFIVTLRILQPHSR     320
EVNILX
..............N.................................................................      80
................................................................................     160
................................................................................     240
................................................................................     320
......                                                                               400

(Threshold=0.5)
----------------------------------------------------------------------
SeqName      Position  Potential   Jury    N-Glyc
                                agreement result
----------------------------------------------------------------------
contig039640-TilTAR.A002   15 NSSC   0.6958     (9/9)   ++
----------------------------------------------------------------------
```

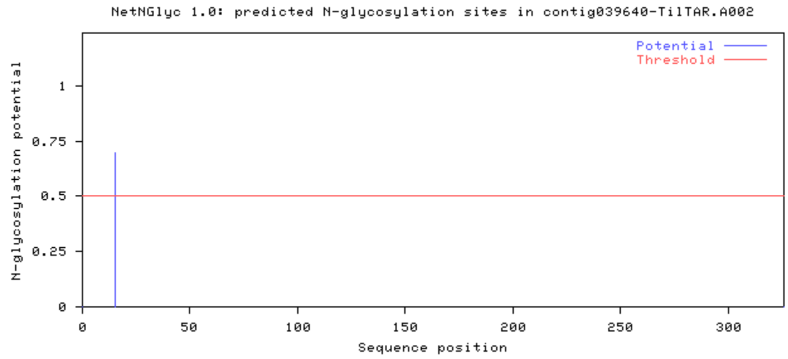

Graphics in PostScript

Output for 'contig039640-TilTAR.A003'

```
##########################################################################################

    Warning: This sequence may not contain a signal peptide!!

    Proteins without signal peptides are unlikely to be exposed to
    the N-glycosylation machinery and thus may not be glycosylated
    (in vivo) even though they contain potential motifs.

    SignalP-NN euk predictions are as follows:

  # name                Cmax  pos ?  Ymax  pos ?  Smax  pos ?  Smean ?  D     ?

    SignalP output is explained at http://www.cbs.dtu.dk/services/SignalP/output.html

##########################################################################################

Name:  contig039640-TilTAR.A003     Length:  326
MDGSGGPPLCFPNLNSSCRRLLRPTSQAALLYTLLASVSLLTVVLNLLVVISISHFKQLHTPTNALLLSLAVSDLLVGLL      80
VMPIEGLRYIETCWLLGRLMCALSPYLSYCLISVSLDSMVLISVDRYIAICDPLLYSSKITVNRVKLSVCVCWVCSLLYN     160
GCILMEHLRWPKRFSSCHGECVVFISYTSGTIDLFLSFVGPCALMFVLYMRVFVVAVSQVRIIRSQVAVRAAPAAKKSEM     240
KAARTLGIVIAVFLMCFCPYYYPSLAGVDTSTSLPYYALFSWIMLTNSCVNPVIYALFYPWFRRAIRLIVTLRILQPHSR     320
EVNILX
..............N.................................................................      80
................................................................................     160
................................................................................     240
................................................................................     320
......                                                                               400

(Threshold=0.5)
----------------------------------------------------------------------
SeqName      Position  Potential   Jury    N-Glyc
                                agreement result
----------------------------------------------------------------------
contig039640-TilTAR.A003   15 NSSC   0.6958     (9/9)   ++
----------------------------------------------------------------------
```

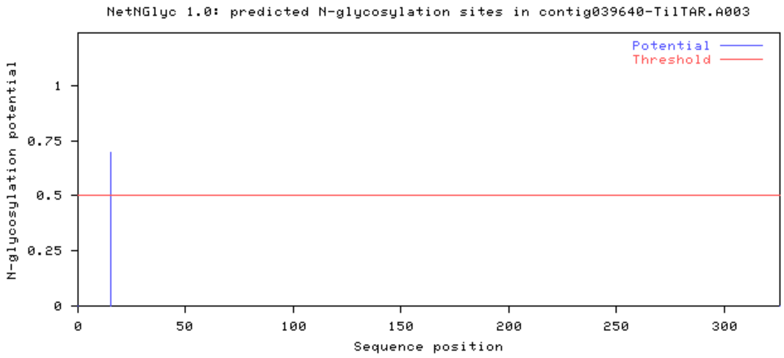

Graphics in PostScript

Output for 'contig039642-TilTAR.A004'

```
########################################################################################

    Warning: This sequence may not contain a signal peptide!!

    Proteins without signal peptides are unlikely to be exposed to
    the N-glycosylation machinery and thus may not be glycosylated
    (in vivo) even though they contain potential motifs.

    SignalP-NN euk predictions are as follows:

  # name                Cmax  pos ?  Ymax  pos ?  Smax  pos ?  Smean ?  D     ?

    SignalP output is explained at http://www.cbs.dtu.dk/services/SignalP/output.html

########################################################################################

Name:  contig039642-TilTAR.A004     Length:  326
MDGSGGPPLCFPNLNSSCRRLLRPTSQAALLYTLLASVSLLTVVLNLLVVVSISHFKQLHTPTNALLLSLAVSDLLVGLL      80
VMPIEGLRYIETCWLLGRLMCALSPYLSYCLISVSLGSMVLISVDRYIAICDPLLYSSKITVNRVKLSVCVCWACSLLYN     160
GCILMEHLGWPDRLSSCHGECVVFISRTSGTVDFFFSFLGPCGVMFVLYMRVFVVAVSQVRAIQSQVAVRAAPAAKISEL     240
KAARTLGILIAVFVMCFCPYYYPSLAGVDTFLSLPSYAMVCWIMLTNACVNPVIYVLFYPWFRRAIRFIVTLRILQPHSR     320
EVNILX
..............N.................................................................      80
................................................................................     160
................................................................................     240
................................................................................     320
......                                                                               400

(Threshold=0.5)
----------------------------------------------------------------------
SeqName      Position  Potential   Jury    N-Glyc
                                agreement result
----------------------------------------------------------------------
contig039642-TilTAR.A004   15 NSSC   0.6959     (9/9)   ++
----------------------------------------------------------------------
```

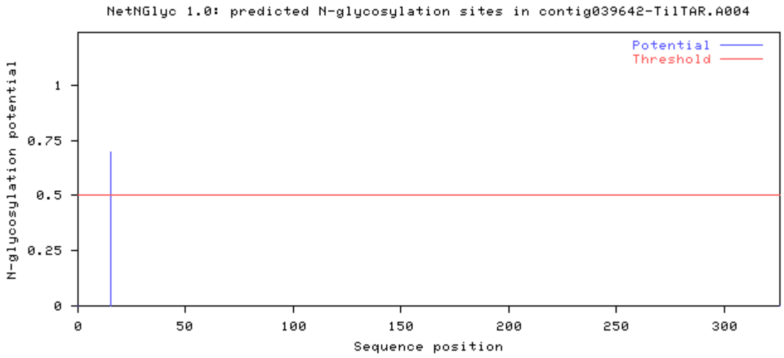

Graphics in PostScript

Output for 'contig023443-TilTAR.A005'

```
########################################################################################

    Warning: This sequence may not contain a signal peptide!!

    Proteins without signal peptides are unlikely to be exposed to
    the N-glycosylation machinery and thus may not be glycosylated
    (in vivo) even though they contain potential motifs.
```

```
    SignalP-NN euk predictions are as follows:

  # name                 Cmax  pos ?  Ymax  pos ?  Smax  pos ?  Smean ?  D     ?

    SignalP output is explained at http://www.cbs.dtu.dk/services/SignalP/output.html

##########################################################################################

Name:  contig023443-TilTAR.A005     Length:  329
MMDSQDRTELCYPELANLSCRGLTRPQPEAVLLYTLFLSISVLTVALNVLVIISISHFRQLHMPTNVLLLSLAISDLLVG      80
LLVMPVETMRFIETCWLLGDLMCALSYIIGFTLTSASVGNMVLISIDRYVAICYPLQYPTKITHSRAELSVTLCWACSLL     160
YNGLILKEHLRQPDRHNTCHSQCLVVINYVSGAIDLVFTFIGPCSVIIILYMRVFVVAVSQAHAMRSHITAAAVRITAKK     240
SEKKAARTLGVVIFVFLLSFCPYYYPSLAGQDISNSASSWAIVSWMLYFNSCLNPLIYALFYPWFRKAIWFIVSLKILEK     320
GSSQANILX
................N...............................................................      80
................................................................................     160
................................................................................     240
................................................................................     320
.........                                                                            400

(Threshold=0.5)
----------------------------------------------------------------------
SeqName      Position  Potential   Jury    N-Glyc
                                agreement result
----------------------------------------------------------------------
contig023443-TilTAR.A005    17 NLSC   0.6858     (9/9)    ++
----------------------------------------------------------------------
```

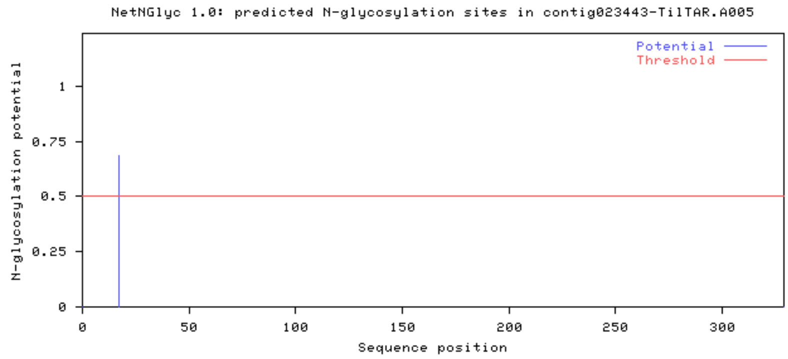

Graphics in PostScript

Output for 'contig007512-TilTARs.A024'

```
##########################################################################################

    Warning: This sequence may not contain a signal peptide!!

    Proteins without signal peptides are unlikely to be exposed to
    the N-glycosylation machinery and thus may not be glycosylated
    (in vivo) even though they contain potential motifs.

    SignalP-NN euk predictions are as follows:

  # name                 Cmax  pos ?  Ymax  pos ?  Smax  pos ?  Smean ?  D     ?

    SignalP output is explained at http://www.cbs.dtu.dk/services/SignalP/output.html

##########################################################################################

Name:  contig007512-TilTARs.A024    Length:  327
MMEETELCFPKLLNISCRRPKRPHFEIMLTYILLSFISLLTVILNLLVIISISHFRQLHTPTNFLLLSLAVADFFVGLLM      80
FFQIVLIDGCWFLGDIMCTLYQYLAFIITSASIGTMVIISADRYLAICYPLHYSTKITQQRVKICICLCWVFSVIFQSLI     160
VKDNLKQPGKYNSCIGECVFVVNYIAGLFDLLFSFIVPITVIIVLYLRVFAVAVSQAHAMRCQHAVTHQRAVTVTVTKSE     240
LKAARTLGVVVVVFLICMCPYYCVALTGQDNFLNASSAAFVICLVYFNSCVNPIIYVFFYPWFRKSIKLIATLQILQPDS     320
HETNMHX
.............N..................................................................      80
................................................................................     160
................................................................................     240
................................................................................     320
.......                                                                              400

(Threshold=0.5)
----------------------------------------------------------------------
SeqName      Position  Potential   Jury    N-Glyc
                                agreement result
----------------------------------------------------------------------
contig007512-TilTARs.A024   14 NISC   0.7886      (9/9)    +++
contig007512-TilTARs.A024  274 NASS   0.4884      (6/9)    -
----------------------------------------------------------------------
```

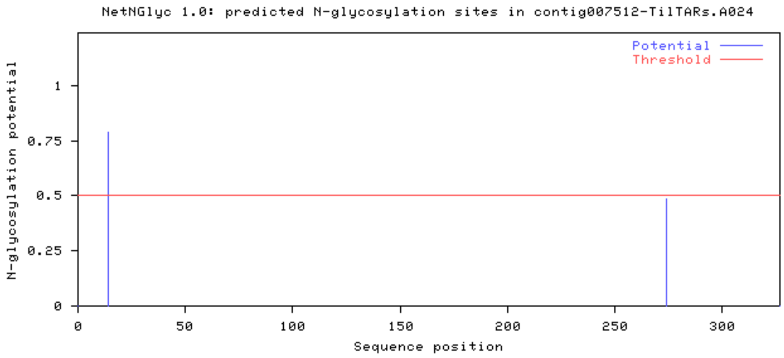

Graphics in PostScript

Output for 'contig007520-TilTARs.A025'

```
##########################################################################################

    Warning: This sequence may not contain a signal peptide!!

    Proteins without signal peptides are unlikely to be exposed to
    the N-glycosylation machinery and thus may not be glycosylated
    (in vivo) even though they contain potential motifs.

    SignalP-NN euk predictions are as follows:

  # name                Cmax  pos ?  Ymax  pos ?  Smax  pos ?  Smean ?  D     ?

    SignalP output is explained at http://www.cbs.dtu.dk/services/SignalP/output.html

##########################################################################################

Name:  contig007520-TilTARs.A025    Length:  330
MEMFEETELCFPQLLNFSCRKPMQPHSVSILIYIALSSISLLTVTLNLLVIISISHFKKLHTPTNLLLLSLAVSDCLVGL      80
LMLFQIMIIDGCWFLGEFMCSMYFLLDYIITSASVGTMVLISIDRYVAICYPLHYSTKVTPKRTKACVYLCWICSSVFQC     160
LVLKDNLVQPGRYNSCYGECVVVVGHAFGVADLLLSFIGPVIVIVVLYLNVFVVAMTQARALRSHIAALTHEGSVSTNVK     240
KSEMKAVRTISVVIIVFLICLCPYFCVTLSGQDAMLSASSVAFVMCLFYLNSCLNPLIYALFYPWFRKSVKQIVTLKILK     320
SGSCDTNIMX
...............N................................................................      80
................................................................................     160
................................................................................     240
................................................................................     320
..........                                                                           400

(Threshold=0.5)
----------------------------------------------------------------------
SeqName      Position  Potential   Jury    N-Glyc
                                 agreement result
----------------------------------------------------------------------
contig007520-TilTARs.A025   16 NFSC   0.6970     (9/9)   ++
----------------------------------------------------------------------
```

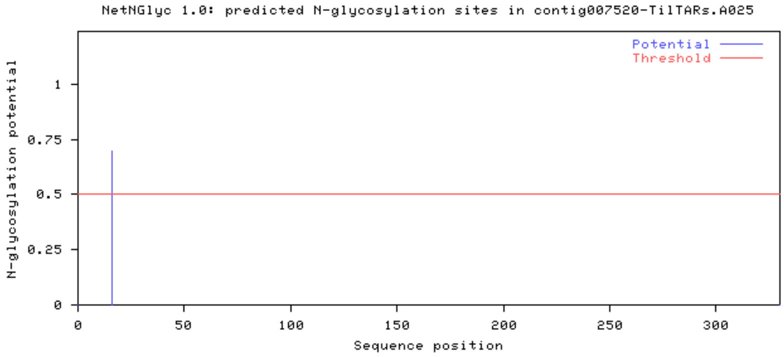

Graphics in PostScript

Output for 'contig007524-TilTARs.A026'

```
##########################################################################################

    Warning: This sequence may not contain a signal peptide!!

    Proteins without signal peptides are unlikely to be exposed to
    the N-glycosylation machinery and thus may not be glycosylated
    (in vivo) even though they contain potential motifs.
```

```
    SignalP-NN euk predictions are as follows:

  # name                 Cmax  pos ?  Ymax  pos ?  Smax  pos ?  Smean ?  D     ?

    SignalP output is explained at http://www.cbs.dtu.dk/services/SignalP/output.html

########################################################################################

Name:  contig007524-TilTARs.A026    Length:  330
MKTFEETELCFPQLLNSSCRKTMRPYTLSILIYITLSSISLITVTLNLLVIISISHFEKLHTPTNLLLLSLAVSDCLVGL      80
LMLFQIMIIDGCWFLGEFMCSMYFLLDYIITSASIGTMVLISIDRYVAICYPLHYSTKVTPKRTKACVYLCWICSSVFQC     160
LVLKDNLVQPGRYNSCYGECVVVVGHAFGVADLLLSFIGPVIVIVVLYLNVFVVAMTQARALRSHIAALTHEGSVSTNVK     240
KSEMKAVRTISVVIIVFLICLCPYFCVTLSGQDAMLSASSVAFVMCLFYLNSCLNPLIYALFYPWFRKSVKQIVTLKILK     320
SGSCDTNIMX
...............N................................................................      80
................................................................................     160
................................................................................     240
................................................................................     320
..........                                                                           400

(Threshold=0.5)
----------------------------------------------------------------------
SeqName      Position  Potential   Jury    N-Glyc
                                 agreement result
----------------------------------------------------------------------
contig007524-TilTARs.A026   16 NSSC   0.6550     (9/9)    ++
----------------------------------------------------------------------
```

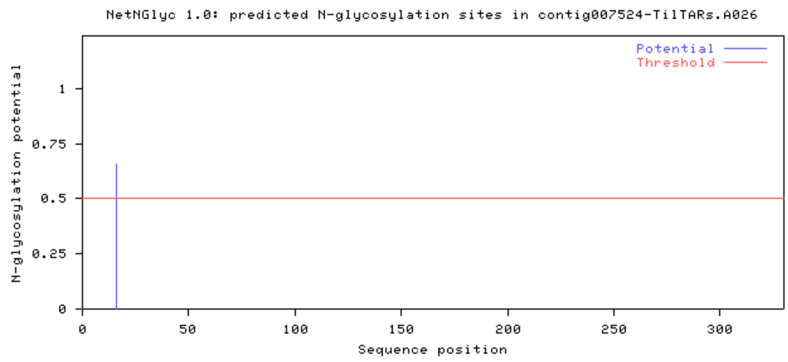

Graphics in PostScript

Output for 'contig022324-TilTARs.A027'

```
########################################################################################

    Warning: This sequence may not contain a signal peptide!!

    Proteins without signal peptides are unlikely to be exposed to
    the N-glycosylation machinery and thus may not be glycosylated
    (in vivo) even though they contain potential motifs.

    SignalP-NN euk predictions are as follows:

  # name                 Cmax  pos ?  Ymax  pos ?  Smax  pos ?  Smean ?  D     ?

    SignalP output is explained at http://www.cbs.dtu.dk/services/SignalP/output.html

########################################################################################

Name:  contig022324-TilTARs.A027    Length:  330
MEIQMHLEAEFCFPELLNSSCRKPTLHWSKTVLLNVGLSFISVITAALNLLIIISVSHFRQLHTPSNILLLSLAVSDFFV      80
GFLLMPVEIFRSTACWVFGDLMCSLYIYLSGILMNASFEIIVFISVDRYVAICDPLHYPTRITVARVKLSVCLCWFYAIF     160
YMSLYTKDVLIKPGRYASCYGECVFVIEDITGTVDLVLCFFVPVIIIIVLYTRVFVVAVSQARAMRSHVTAVTLERSLNQ     240
TNKSELKAARNLGVLVIVFLASICPFYFYSLVDGSVLNASSATFLIIVYFNSFLNPLIYTLFYPWFRNAVKLIITLQIFK     320
HNSSEANILX
.................N..............................................................      80
................................................................................     160
................................................................................     240
................................................................................     320
..........                                                                           400

(Threshold=0.5)
----------------------------------------------------------------------
SeqName      Position  Potential   Jury    N-Glyc
                                 agreement result
----------------------------------------------------------------------
contig022324-TilTARs.A027   18 NSSC   0.6778     (9/9)    ++
contig022324-TilTARs.A027  115 NASF   0.4697     (4/9)    -
contig022324-TilTARs.A027  239 NQTN   0.4744     (4/9)    -
contig022324-TilTARs.A027  242 NKSE   0.4208     (5/9)    -
contig022324-TilTARs.A027  278 NASS   0.4451     (6/9)    -
contig022324-TilTARs.A027  322 NSSE   0.4094     (6/9)    -
----------------------------------------------------------------------
```

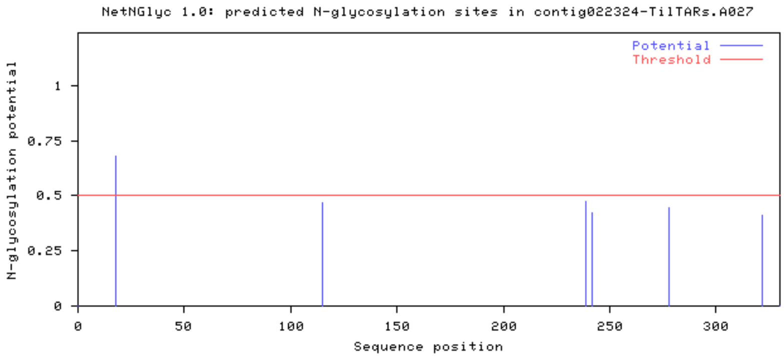

[Graphics in PostScript]

Output for 'contig022330-TilTARs.A028'

```
##########################################################################################

    Warning: This sequence may not contain a signal peptide!!

    Proteins without signal peptides are unlikely to be exposed to
    the N-glycosylation machinery and thus may not be glycosylated
    (in vivo) even though they contain potential motifs.

    SignalP-NN euk predictions are as follows:

  # name                Cmax  pos ?  Ymax  pos ?  Smax  pos ?  Smean ?  D     ?

    SignalP output is explained at http://www.cbs.dtu.dk/services/SignalP/output.html

##########################################################################################

Name:  contig022330-TilTARs.A028    Length:  329
MEIQKEAELCFPQLLNISCRKPTLHWSKAVLLNIVLSCISLLTATLNLLVIISVSYFRKLHTPSNILLLSLAVSDFLMGL      80
LLIPAEILRSMTCWVLGDLMCSVYFFLTVNITCASIGNIVLISIDRHVAICDPLHYSTRITVPRVKLSVCLCWFYSTFYS     160
SLNTQEMLIVPGRYNSCYGECVLVISDFAGTLDLILFFILPVTVIIVLYMRVFVVAVSQARAMRSHVTAVTLQRSPNQTN     240
KSELKAARTLGVLVVVFLACYCPFYCYSLADENAVNDPAASFVVFVFYFNSCINPLMYALFYPWFRNAVKLIITLQIFKH     320
NTCEANILX
...............N................................................................      80
.............................N..................................................     160
................................................................................     240
................................................................................     320
.........                                                                            400

(Threshold=0.5)
----------------------------------------------------------------------
SeqName      Position  Potential   Jury    N-Glyc
                               agreement result
----------------------------------------------------------------------
contig022330-TilTARs.A028   16 NISC   0.7508     (9/9)    +++
contig022330-TilTARs.A028  110 NITC   0.7488     (9/9)    ++
contig022330-TilTARs.A028  237 NQTN   0.4044     (7/9)    -
contig022330-TilTARs.A028  240 NKSE   0.4326     (5/9)    -
----------------------------------------------------------------------
```

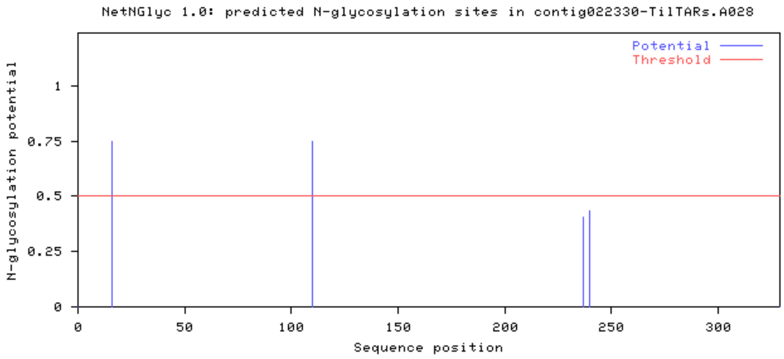

[Graphics in PostScript]

Output for 'contig022334-TilTARs.A029'

```
##########################################################################################

    Warning: This sequence may not contain a signal peptide!!

    Proteins without signal peptides are unlikely to be exposed to
```

```
    the N-glycosylation machinery and thus may not be glycosylated
    (in vivo) even though they contain potential motifs.

    SignalP-NN euk predictions are as follows:

  # name                 Cmax  pos ?  Ymax  pos ?  Smax  pos ?  Smean ?  D     ?

    SignalP output is explained at http://www.cbs.dtu.dk/services/SignalP/output.html

##########################################################################################

Name:  contig022334-TilTARs.A029    Length:  329
MEIQKEAELCFPQLLNISCRKPTLHWSKAVLLNIVLSCISLLTATLNLLVIISVSYFRKLHTPSNILLLSLAVSDFLMGL      80
LLIPAEILRSMTCWVLGDLMCSVYFFLTVNITCASIGNIVLISIDRHVAICDPLHYPTRMTVPKIKLSVCLCWFYSTFYS     160
SLNTKKILIEPGRYNSCYGECVLVSSNIAGTLDLILFFILPVTVIIVLYMRVFVVAVSQARAMRSHVTAVTLQRSPNQTN     240
KSELKAARTLGVLVVVFLACYCPFYCYSLADENAVNDPAASFVVFVFYFNSCINPLMYALFYPWFRNAVKLIITLQIFKH     320
NTCEANILX
...............N................................................................      80
.............................N..................................................     160
................................................................................     240
................................................................................     320
.........                                                                            400

(Threshold=0.5)
----------------------------------------------------------------------
SeqName      Position  Potential   Jury    N-Glyc
                                agreement result
----------------------------------------------------------------------
contig022334-TilTARs.A029   16 NISC   0.7508     (9/9)   +++
contig022334-TilTARs.A029  110 NITC   0.7488     (9/9)   ++
contig022334-TilTARs.A029  237 NQTN   0.4042     (7/9)   -
contig022334-TilTARs.A029  240 NKSE   0.4328     (5/9)   -
----------------------------------------------------------------------
```

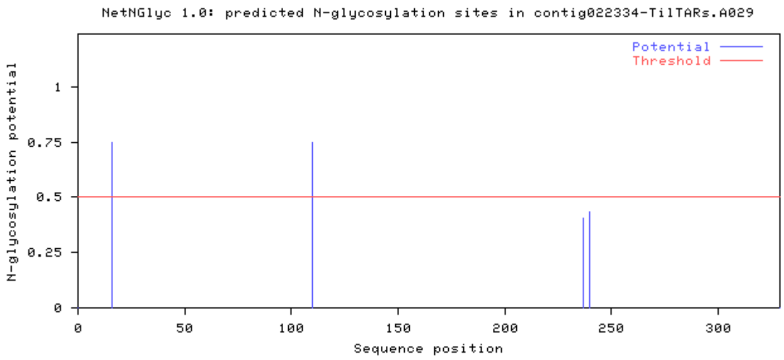

Graphics in PostScript

Output for 'contig022337-TilTARs.A031'

```
##########################################################################################

    Warning: This sequence may not contain a signal peptide!!

    Proteins without signal peptides are unlikely to be exposed to
    the N-glycosylation machinery and thus may not be glycosylated
    (in vivo) even though they contain potential motifs.

    SignalP-NN euk predictions are as follows:

  # name                 Cmax  pos ?  Ymax  pos ?  Smax  pos ?  Smean ?  D     ?

    SignalP output is explained at http://www.cbs.dtu.dk/services/SignalP/output.html

##########################################################################################

Name:  contig022337-TilTARs.A031    Length:  331
MMEIEKGAELCFPQLLNSSCRKPTLHWSRALLLNIVLSCISLITAALNLLVIISVSYFRQLHTPSNIVLLSLAVSDFLIG      80
LLKMPFEIIRNTGCWVLGDLMCSVYIFLTISLLCASILNIVLISIDRYVAICDPLHYPTRLTVARVKLSVCLCWFYSAFY     160
SSLYAKNVLIEPGRYNFCFGECVFFSSNIAVVVDLVLFFIVPVSVIIVLYMRVFVVAVSQARAMRSHVTSVTLQRSANQA     240
NKSELKAARTLGVLVVVFLATFCPFYCYSLVEENALNDPSTSTLITVFYLSNSCLNPLIYALFHPWFRNAVKLIITLQIF     320
KCDTSEANILX
................N...............................................................      80
................................................................................     160
................................................................................     240
................................................................................     320
...........                                                                          400

(Threshold=0.5)
----------------------------------------------------------------------
SeqName      Position  Potential   Jury    N-Glyc
                                agreement result
----------------------------------------------------------------------
contig022337-TilTARs.A031   17 NSSC   0.6940     (9/9)   ++
contig022337-TilTARs.A031  241 NKSE   0.3774     (7/9)   -
----------------------------------------------------------------------
```

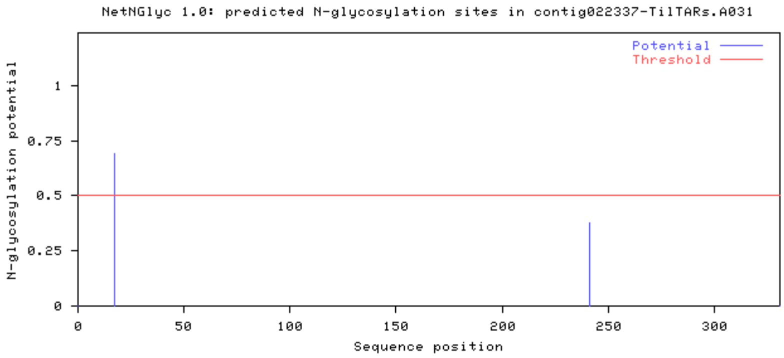

Graphics in PostScript

Output for 'contig022341-TilTARs.A032'

```
##########################################################################################

    Warning: This sequence may not contain a signal peptide!!

    Proteins without signal peptides are unlikely to be exposed to
    the N-glycosylation machinery and thus may not be glycosylated
    (in vivo) even though they contain potential motifs.

    SignalP-NN euk predictions are as follows:

  # name                Cmax  pos ?  Ymax  pos ?  Smax  pos ?  Smean ?  D     ?

    SignalP output is explained at http://www.cbs.dtu.dk/services/SignalP/output.html

##########################################################################################

Name:  contig022341-TilTARs.A032    Length:  329
MEIQKGVELCFPQLLNTSCRKPALHWSKAVLLNIVLSCVSLITAALNLLVIISVSYFRKLHTPTNILLLSLAVSDFLVGL      80
LLMPFEIFRNMACWVLGDLMCSVSWYLTGNIAFASTGNIVLISIDRYVAICHPLHYPTRITVARVKLSVSLFWLFAIFYS     160
SLYMKDVLINPGRYNSCYGECVFIINDIAGIFDMVLSFFVPVPVIVILYMRVFVVAVSQARAMRSHVTAVTLQRSLNQTN     240
KSELKAARTLGILVVVFLICYCPFFCFSFGVEDMVNNPSTFFVIMVFYFNSCLNPLIYALFYPWFRNAVKLIITLQIFKH     320
ETSEANILX
...............N................................................................      80
................................................................................     160
................................................................................     240
................................................................................     320
.........                                                                            400

(Threshold=0.5)
----------------------------------------------------------------------
SeqName      Position  Potential   Jury    N-Glyc
                                agreement result
----------------------------------------------------------------------
contig022341-TilTARs.A032    16 NTSC    0.6894     (9/9)    ++
contig022341-TilTARs.A032   237 NQTN    0.4559     (4/9)    -
contig022341-TilTARs.A032   240 NKSE    0.4188     (5/9)    -
contig022341-TilTARs.A032   277 NPST    0.4423     (6/9)    -
----------------------------------------------------------------------
```

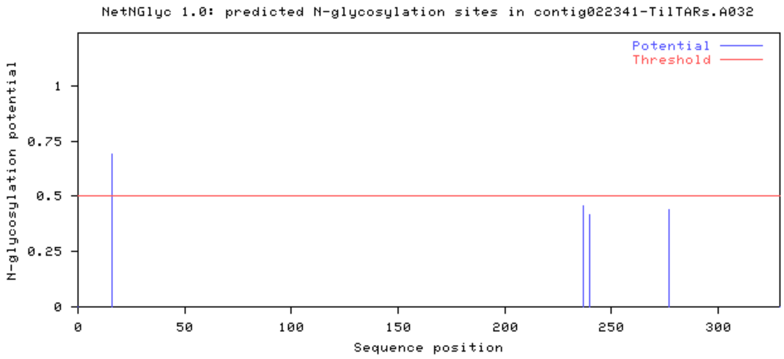

Graphics in PostScript

Output for 'contig022343-TilTARs.A033'

```
##########################################################################################

    Warning: This sequence may not contain a signal peptide!!

    Proteins without signal peptides are unlikely to be exposed to
```

```
    the N-glycosylation machinery and thus may not be glycosylated
    (in vivo) even though they contain potential motifs.

    SignalP-NN euk predictions are as follows:

  # name                 Cmax  pos ?  Ymax  pos ?  Smax  pos ?  Smean ?  D     ?

    SignalP output is explained at http://www.cbs.dtu.dk/services/SignalP/output.html

##########################################################################################

Name:  contig022343-TilTARs.A033    Length:  329
MEIEKGAELCFPQLLNSSCRKPTLHWSRALLLYIVLSCISLITAALNLLVIISVSYFRQLHTPSNIVLLSLAVSDFLIGL      80
LMMPFEIIRNTGCWVLGDLMCSVYIFLTISLVCASILNIVLISIDRYVAICDPLHYPTRLTVARVKLSVCLCWFYSTFYS     160
SLYAKNVLIEPGRYNFCFGECVFFSSNIAVVVDLVLFFIVPVSVIVVLYMRVFVVAVSQAHAMRSHVTSVTLQRSANQAN     240
KSELKAARTLGVLVVVFLATFCPYYCYSLVDENAVNDPSASFVIIIFYINSCLNPLIYALFYPWFRNAVKLIITLQIFKC     320
DTSEANILX
...............N................................................................      80
................................................................................     160
................................................................................     240
................................................................................     320
.........                                                                            400

(Threshold=0.5)
----------------------------------------------------------------------
SeqName      Position  Potential   Jury    N-Glyc
                                agreement result
----------------------------------------------------------------------
contig022343-TilTARs.A033   16 NSSC   0.6944     (9/9)   ++
contig022343-TilTARs.A033  240 NKSE   0.3776     (7/9)   -
----------------------------------------------------------------------
```

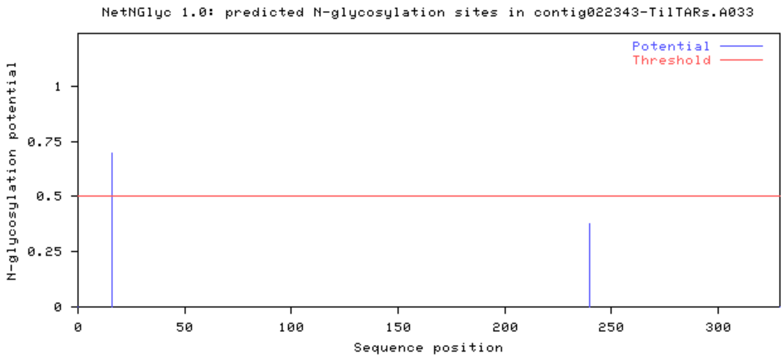

[Graphics in PostScript](#)

Output for 'contig022345-TilTARs.A034'

```
##########################################################################################

    Warning: This sequence may not contain a signal peptide!!

    Proteins without signal peptides are unlikely to be exposed to
    the N-glycosylation machinery and thus may not be glycosylated
    (in vivo) even though they contain potential motifs.

    SignalP-NN euk predictions are as follows:

  # name                 Cmax  pos ?  Ymax  pos ?  Smax  pos ?  Smean ?  D     ?

    SignalP output is explained at http://www.cbs.dtu.dk/services/SignalP/output.html

##########################################################################################

Name:  contig022345-TilTARs.A034    Length:  329
MEMQKGTELCFPQLLNNSCRKPIRHWSKAVLLYIVLFCISLITAALNLLVIISISHFKQLHTPSNILLLSLAVSDFLVGL      80
LLMPLEIFRSTACWVLGDLMCSVYIYMTVNITCASIGNIVLISVDRYVAICDPLHYPTRITVARIKLSICFCWFYTIFYS     160
SLYTKDILIEPGRYNSCCGECVLVINDIVGIVDLVLTFIVPVAVIIVLYMRVFVAAVSQARAMRSHVTAVTLQRPLNQTN     240
KSELKAARTLGVLVVVFLACYFPFYCYASVVEDMVNNPSALFVFMVFYFNSCLNPLIYALFYPWFRNAVKLIVTLQFLKH     320
GTCEANILX
...............N................................................................      80
.............................N..................................................     160
............................................................................N...     240
................................................................................     320
.........                                                                            400

(Threshold=0.5)
----------------------------------------------------------------------
SeqName      Position  Potential   Jury    N-Glyc
                                agreement result
----------------------------------------------------------------------
contig022345-TilTARs.A034   16 NNSC   0.6181     (9/9)   ++
contig022345-TilTARs.A034  110 NITC   0.7190     (9/9)   ++
contig022345-TilTARs.A034  237 NQTN   0.5031     (6/9)   +
contig022345-TilTARs.A034  240 NKSE   0.4268     (5/9)   -
contig022345-TilTARs.A034  277 NPSA   0.4033     (7/9)   -
----------------------------------------------------------------------
```

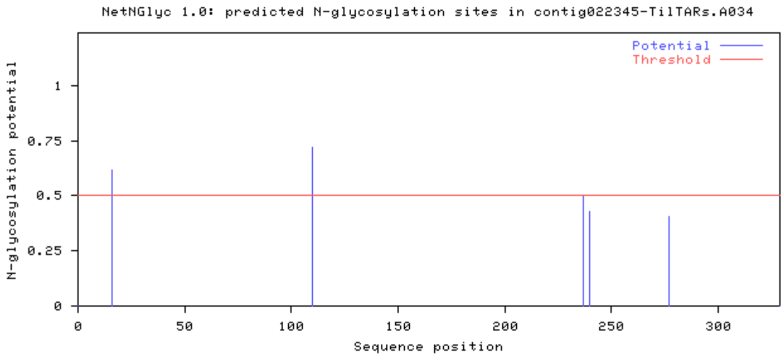

Graphics in PostScript

Output for 'contig022349-TilTARs.A035'

```
########################################################################################

    Warning: This sequence may not contain a signal peptide!!

    Proteins without signal peptides are unlikely to be exposed to
    the N-glycosylation machinery and thus may not be glycosylated
    (in vivo) even though they contain potential motifs.

    SignalP-NN euk predictions are as follows:

  # name                Cmax  pos ?  Ymax  pos ?  Smax  pos ?  Smean ?  D     ?

    SignalP output is explained at http://www.cbs.dtu.dk/services/SignalP/output.html

########################################################################################

Name:  contig022349-TilTARs.A035    Length:  329
MEIQKGAELCFPQLLNSSCRNPTLHWSRSLLLNIVLSCFSPITAALNLLVIISVSHFRQLHTPTNILLLSLAVSDFFVGL      80
LLMPLEIFRNTTCWILGDIMCSVYWYLNFSFICVTIGNILLISVDRYVAICYPLHYPNRITVARVKRSVCLCWFYAIFYC     160
SFYTKDILIQPGRYNSCYGECVFFIDDMAGIIDLVLFFLIPVMVIIVLYTRVFVVAVSQARAMRSHVTAVTLQRSANQAN     240
KSELKAARTLGVLVVVFLLCYCPSYFFTFFAEGMLNDPATVFFITVIYFNSCLNPLIYALFYSWFRNAVKRIITLQIVTH     320
DTNEANILX
...............N....N...........................................................      80
.........N.................N....................................................     160
................................................................................     240
................................................................................     320
.........                                                                            400

(Threshold=0.5)
----------------------------------------------------------------------
SeqName      Position  Potential   Jury    N-Glyc
                     agreement result
----------------------------------------------------------------------
contig022349-TilTARs.A035   16 NSSC   0.7122     (9/9)   ++
contig022349-TilTARs.A035   21 NPTL   0.6401     (8/9)   +    WARNING: PRO-X1.
contig022349-TilTARs.A035   90 NTTC   0.6680     (9/9)   ++
contig022349-TilTARs.A035  108 NFSF   0.7221     (9/9)   ++
contig022349-TilTARs.A035  240 NKSE   0.3777     (7/9)   -
----------------------------------------------------------------------
```

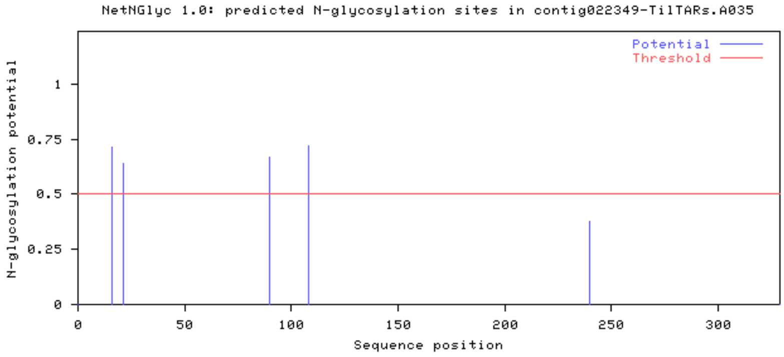

Graphics in PostScript

Output for 'contig022353-TilTARs.A036'

```
########################################################################################

    Warning: This sequence may not contain a signal peptide!!
```

```
    Proteins without signal peptides are unlikely to be exposed to
    the N-glycosylation machinery and thus may not be glycosylated
    (in vivo) even though they contain potential motifs.

    SignalP-NN euk predictions are as follows:

  # name                Cmax  pos ?  Ymax  pos ?  Smax  pos ?  Smean ?  D     ?

    SignalP output is explained at http://www.cbs.dtu.dk/services/SignalP/output.html

##########################################################################################

Name:  contig022353-TilTARs.A036    Length:  329
MEIPKGVELCFPQLLNSSCRKPTLHWSKAVLLNIVLSCISLLTAALNLLVIISVSHFRQLHTPSNILLLSLAVSDFFVGL      80
LLLPLEIFRNTSCWVLGDLMCSAYWYLTSNIICASIGNIVLISVDRYVAICDPLHYPTRITLPKVKLGVCLCWFYAIFYS     160
SLYTKDVMIEPGRYNSCFGECVFFSSNIAIVVDLILSFFGPVTVIIVLYMRVFVVAVSQARAMRSHVTSVTLQRPLNQTN     240
KSELKAARSLGVLVVVFLACFCPLYCYSLVDENAVNDPSASFVVIIFYINSCLNPLIYALFYPWFRNAVKLIISLEIFKY     320
DTSGANILX
...............N................................................................      80
.........N......................................................................     160
............................................................................N...     240
................................................................................     320
.........                                                                            400

(Threshold=0.5)
----------------------------------------------------------------------
SeqName      Position  Potential   Jury    N-Glyc
                                agreement result
----------------------------------------------------------------------
contig022353-TilTARs.A036   16 NSSC   0.6896     (9/9)   ++
contig022353-TilTARs.A036   90 NTSC   0.6279     (6/9)   +
contig022353-TilTARs.A036  237 NQTN   0.5013     (6/9)   +
contig022353-TilTARs.A036  240 NKSE   0.4303     (5/9)   -
----------------------------------------------------------------------
```

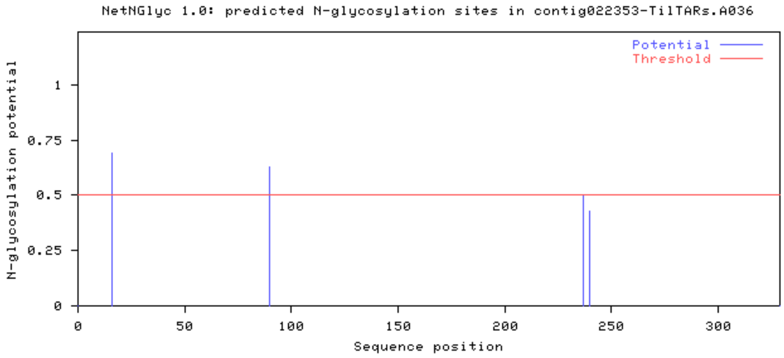

Graphics in PostScript

Output for 'contig022354-TilTARs.A037'

```
##########################################################################################

    Warning: This sequence may not contain a signal peptide!!

    Proteins without signal peptides are unlikely to be exposed to
    the N-glycosylation machinery and thus may not be glycosylated
    (in vivo) even though they contain potential motifs.

    SignalP-NN euk predictions are as follows:

  # name                Cmax  pos ?  Ymax  pos ?  Smax  pos ?  Smean ?  D     ?

    SignalP output is explained at http://www.cbs.dtu.dk/services/SignalP/output.html

##########################################################################################

Name:  contig022354-TilTARs.A037    Length:  327
MEIQEGPELCFPQLLNDSCRKQTLHYSKTMLLYIVMSCISLITTALNLLVIISVSHFRQLHTPSNILLLSLAVSDFLVGL      80
LLMPLEILRSTTCWVLGDFMCFLYWYLTGNITCVSIGNIVLISVDRYVAICDPLHYSTRITVAKVKLSVCLCWFYAIFSI     160
SLYAKDYLIESGRYNSCYGECAFVINDIARTIDLVLSFIFPITVIIVLYMRVFVVAVSQARAMRSHVTAVTLQCSLNQAN     240
KSELKAARTLGILVVVFLLCFCPYYCVSLIREDFSSVAPIVISLFFSNSCLNPVIYALFYPWFRKALKLIVTLQILHSGS     320
CEVSILX
...............N................................................................      80
.............................N..................................................     160
................................................................................     240
................................................................................     320
.......                                                                              400

(Threshold=0.5)
----------------------------------------------------------------------
SeqName      Position  Potential   Jury    N-Glyc
                                agreement result
----------------------------------------------------------------------
contig022354-TilTARs.A037   16 NDSC   0.6393     (8/9)   +
contig022354-TilTARs.A037  110 NITC   0.7857     (9/9)   +++
contig022354-TilTARs.A037  240 NKSE   0.3672     (8/9)   -
----------------------------------------------------------------------
```

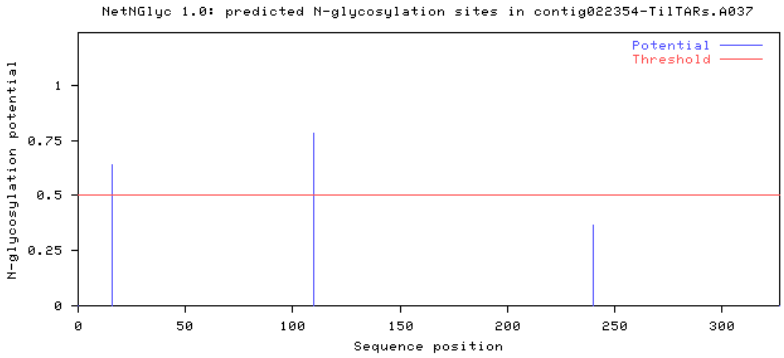

Graphics in PostScript

Output for 'contig022354-TilTARs.A038'

```
##########################################################################################

    Warning: This sequence may not contain a signal peptide!!

    Proteins without signal peptides are unlikely to be exposed to
    the N-glycosylation machinery and thus may not be glycosylated
    (in vivo) even though they contain potential motifs.

    SignalP-NN euk predictions are as follows:

  # name                Cmax  pos ?  Ymax  pos ?  Smax  pos ?  Smean ?  D     ?

    SignalP output is explained at http://www.cbs.dtu.dk/services/SignalP/output.html

##########################################################################################

Name:  contig022354-TilTARs.A038    Length:  329
MEMQKGAELCFPQLLNSSCRKPTLHWSKAVLLNIVLSCISLLTAALNLLVIISVSYFRQLHTPTNILLLSLAVSDFLTGL      80
LMLPLEIIRNTACWVLGDLMCSIYTYLTAGLFCASTGNIVLISVDRYVAICHPLHYPTRITVARVKLCVCLCWFCSTFYS     160
SLLTKDVLIKPARYNSCYGECVFVINDIAGFIDLVLSFIVPVSVIIVLYMRVFVVAVSQARAMRSHVTAVTLHHSSNQAN     240
KSELKAARTLGVLVVVFLACYLPFYCYSLIEVNVINDSSTFFLIIVFYFNSCLNPLIYALFYSWFRNAVKLIITLQIFKH     320
DTSEANMFX
...............N................................................................      80
................................................................................     160
................................................................................     240
................................................................................     320
.........                                                                            400

(Threshold=0.5)
----------------------------------------------------------------------
SeqName      Position  Potential   Jury    N-Glyc
                      agreement result
----------------------------------------------------------------------
contig022354-TilTARs.A038   16 NSSC   0.6944     (9/9)    ++
contig022354-TilTARs.A038  240 NKSE   0.3403     (8/9)    -
contig022354-TilTARs.A038  276 NDSS   0.3999     (7/9)    -
----------------------------------------------------------------------
```

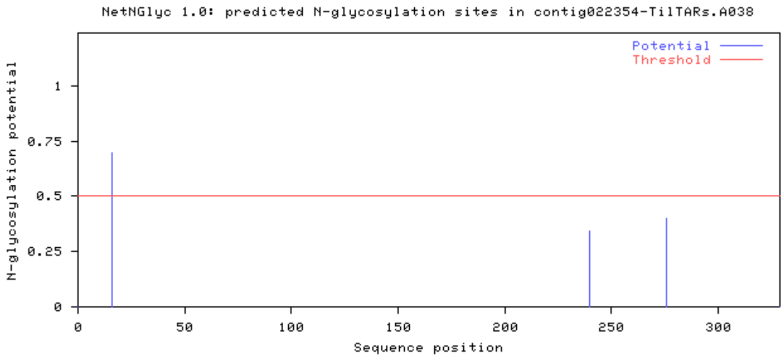

Graphics in PostScript

Output for 'contig022355-TilTARs.A039'

```
##########################################################################################

    Warning: This sequence may not contain a signal peptide!!

    Proteins without signal peptides are unlikely to be exposed to
    the N-glycosylation machinery and thus may not be glycosylated
```

```
    (in vivo) even though they contain potential motifs.

    SignalP-NN euk predictions are as follows:

  # name                 Cmax  pos ?  Ymax  pos ?  Smax  pos ?  Smean ?  D     ?
```

    SignalP output is explained at http://www.cbs.dtu.dk/services/SignalP/output.html

```
##########################################################################################

Name:  contig022355-TilTARs.A039    Length:  329
MEIQKENKLCFPQLLNSSCRKPTLHWSKAVLLNTVLSCISLLTATLNLLVIISVSYFRKLHTPSNILLLSLAVSDFLVGV      80
LLMPLGILRNTSCWVLGDIICSLYWYLTSNIVCASIGNIVLISVDRYVAICDPLHYPSRITLVKVKLSVCLCWFYAIFYC     160
SLYTKDILIEPGRDNSCYGECVFVINDIAVVVDLVFSFIVPVSVIVVLYMRVFVAAVSQARAMRSHVTSVTLQRSLNQTN     240
KSELKAARTLGVLVVVFLACFCPLYYFSLVDENAINDPSASFVVIIFYFNSCINPLIYALFYPWFRNAVKLIITLQIFKY     320
NISEANILX
...............N................................................................      80
.........N......................................................................     160
................................................................................     240
................................................................................     320
.........                                                                            400

(Threshold=0.5)
----------------------------------------------------------------------
SeqName      Position  Potential   Jury    N-Glyc
                                agreement result
----------------------------------------------------------------------
contig022355-TilTARs.A039   16 NSSC   0.6974     (9/9)   ++
contig022355-TilTARs.A039   90 NTSC   0.6288     (6/9)   +
contig022355-TilTARs.A039  237 NQTN   0.4539     (5/9)   -
contig022355-TilTARs.A039  240 NKSE   0.4183     (5/9)   -
contig022355-TilTARs.A039  321 NISE   0.4840     (5/9)   -
----------------------------------------------------------------------
```

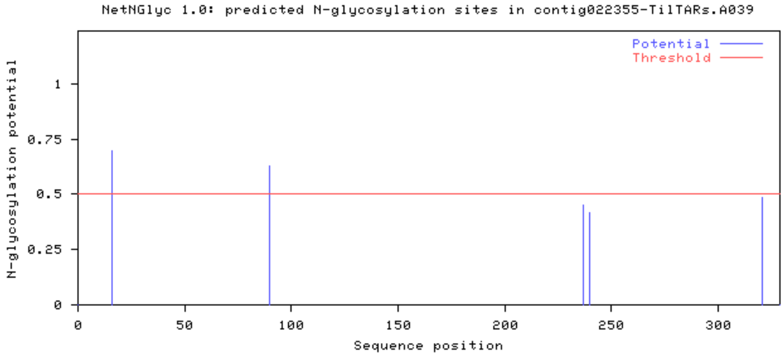

[Graphics in PostScript]

Output for 'contig022356-TilTARs.A040'

```
##########################################################################################

    Warning: This sequence may not contain a signal peptide!!

    Proteins without signal peptides are unlikely to be exposed to
    the N-glycosylation machinery and thus may not be glycosylated
    (in vivo) even though they contain potential motifs.

    SignalP-NN euk predictions are as follows:

  # name                 Cmax  pos ?  Ymax  pos ?  Smax  pos ?  Smean ?  D     ?
```

    SignalP output is explained at http://www.cbs.dtu.dk/services/SignalP/output.html

```
##########################################################################################

Name:  contig022356-TilTARs.A040    Length:  329
MEIQKGAELCFPQFLNNSCRKPTLRSSKAVLLNIVFSCISLLTAALNLLVIISVSYFRKLHTPSNILLLSLAVSDFLVGL      80
LLMPLEIFRSTTCWVLGDVMCSVYWYLTGNITCASIGNVVLISVDRYVAICDPLYYRTRITVERTKLSVCLCWFYAIFYW     160
SLYIKNILIEPGRYNSCYGECMFVSSDIAGIIDLVLSFIVPVSVIIVLYMRVFVVAVSQARAMRSHVTAVTLQHSNNQAN     240
KSELKAARTLGVLVAVFLSCYSPFYCYYLAEENVVNDPSASTVIIIFYFNSCINPLIYALFYPWFRNAVKLIITLQIFKH     320
NTCEANILX
...............N................................................................      80
.............................N..................................................     160
................................................................................     240
................................................................................     320
.........                                                                            400

(Threshold=0.5)
----------------------------------------------------------------------
SeqName      Position  Potential   Jury    N-Glyc
                                agreement result
----------------------------------------------------------------------
contig022356-TilTARs.A040   16 NNSC   0.5928     (6/9)   +
contig022356-TilTARs.A040  110 NITC   0.7801     (9/9)   +++
contig022356-TilTARs.A040  240 NKSE   0.3921     (8/9)   -
----------------------------------------------------------------------
```

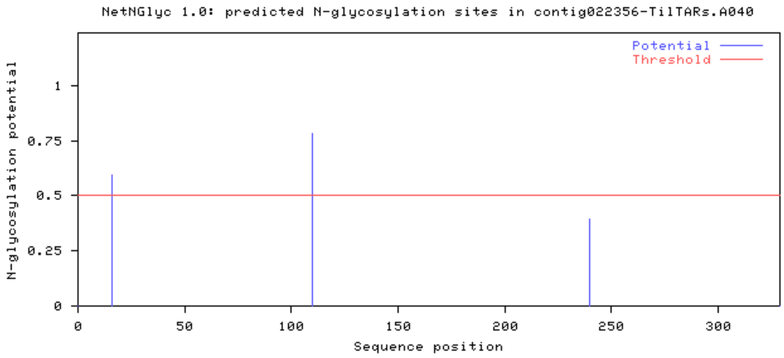

Graphics in PostScript

Output for 'contig022357-TilTARs.A041'

```
########################################################################################

    Warning: This sequence may not contain a signal peptide!!

    Proteins without signal peptides are unlikely to be exposed to
    the N-glycosylation machinery and thus may not be glycosylated
    (in vivo) even though they contain potential motifs.

    SignalP-NN euk predictions are as follows:

  # name                 Cmax  pos ?  Ymax  pos ?  Smax  pos ?  Smean ?  D     ?

    SignalP output is explained at http://www.cbs.dtu.dk/services/SignalP/output.html

########################################################################################

Name:  contig022357-TilTARs.A041    Length:  329
MVIQKGAELCFPQLLNSSCRKPTLHWSKAVFLNIMLPCLSLTTAALNLLVIISVSHFRQLHTPTNILLLSLAVSDFLTGF      80
LILPLEIIRNMACWVLGDLMCSVYTYLITSLFCASTGIIVLISVDRYVAICDPLHYPTRMTVVRSKLSVCLCWFYSTFYS     160
SLQTKDVLINPGRYNSCDGECVFLISNIAGTIDLVLSFIFPVSVIIILYMRVFVVAVSQARATRSHVTAVTLQSSLNQTN     240
KSELKAARTLGILVVVFLACYCPFYCYFLVKENVVNDSATFFVIILYHVNSCLNPLIYALFYPWFRNAVKLIITFQIFTH     320
DTSGANILX
...............N................................................................      80
................................................................................     160
................................................................................     240
................................................................................     320
.........                                                                            400

(Threshold=0.5)
----------------------------------------------------------------------
SeqName      Position  Potential   Jury    N-Glyc
                                agreement result
----------------------------------------------------------------------
contig022357-TilTARs.A041   16 NSSC   0.6946     (9/9)   ++
contig022357-TilTARs.A041  237 NQTN   0.4572     (6/9)   -
contig022357-TilTARs.A041  240 NKSE   0.4273     (6/9)   -
contig022357-TilTARs.A041  276 NDSA   0.4342     (6/9)   -
----------------------------------------------------------------------
```

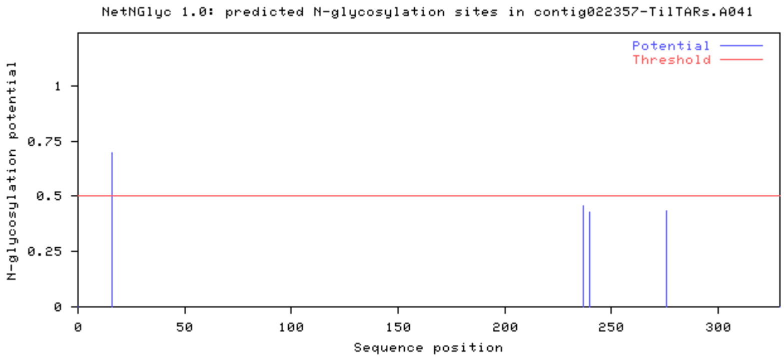

Graphics in PostScript

Output for 'contig022362-TilTARs.A042'

```
########################################################################################

    Warning: This sequence may not contain a signal peptide!!

    Proteins without signal peptides are unlikely to be exposed to
```

```
    the N-glycosylation machinery and thus may not be glycosylated
    (in vivo) even though they contain potential motifs.

    SignalP-NN euk predictions are as follows:

  # name                 Cmax  pos ?  Ymax  pos ?  Smax  pos ?  Smean ?  D     ?

    SignalP output is explained at http://www.cbs.dtu.dk/services/SignalP/output.html

##########################################################################################

Name:  contig022362-TilTARs.A042    Length:  321
MEIQKGAELCFQQLLNNSCRKLTLNWSKTVLLYIVLFCISLITVALNLLVIISVSHFRQLHTPTNILLLSLAVSDFLVGL      80
LLIPLEILRSTTCWVFGDVICSFYFYLTGNITCASTGIIVLISVDRYVAICDPLHYPTRMTVARVKLSVCLCWFYAIFYV     160
TLYLKDFLKPGRYNSCYGECILVINDTAGTADLVLSFFVPITVIIVLYMRIFVVAVSQARAMRSHVTAVAKSKSELKAAR     240
TLGVLVVVFLASYCPFYCYTLVEENVISDSSAFFVVIAFYLNSFLNPMIYALFYPWFKKSVNLIITLQIFKRHSSDSNIL     320
X
...............N........N.......................................................      80
.............................N..................................................     160
................................................................................     240
................................................................................     320
.                                                                                    400

(Threshold=0.5)
----------------------------------------------------------------------
SeqName      Position  Potential   Jury    N-Glyc
                      agreement result
----------------------------------------------------------------------
contig022362-TilTARs.A042   16 NNSC   0.6116     (9/9)    ++
contig022362-TilTARs.A042   25 NWSK   0.7681     (9/9)    +++
contig022362-TilTARs.A042  110 NITC   0.7682     (9/9)    +++
contig022362-TilTARs.A042  185 NDTA   0.4252     (7/9)    -
----------------------------------------------------------------------
```

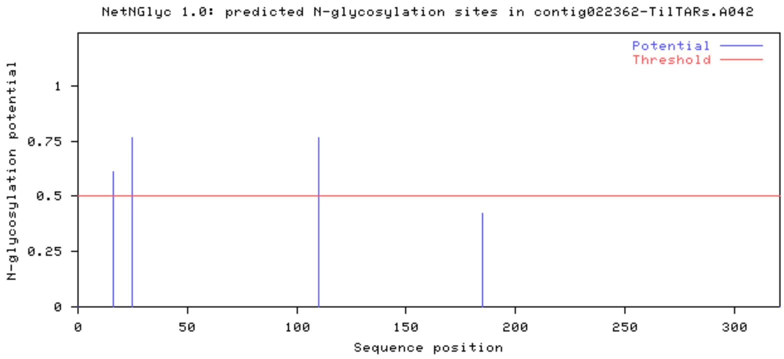

Graphics in PostScript

Output for 'contig022363-TilTARs.A043'

```
##########################################################################################

    Warning: This sequence may not contain a signal peptide!!

    Proteins without signal peptides are unlikely to be exposed to
    the N-glycosylation machinery and thus may not be glycosylated
    (in vivo) even though they contain potential motifs.

    SignalP-NN euk predictions are as follows:

  # name                 Cmax  pos ?  Ymax  pos ?  Smax  pos ?  Smean ?  D     ?

    SignalP output is explained at http://www.cbs.dtu.dk/services/SignalP/output.html

##########################################################################################

Name:  contig022363-TilTARs.A043    Length:  209
MEIQKGAELCFQQLLNNSCRKPTLHWSKAVLLYIVLFCISLITVALNLLVIISVSHFRQLHTPTNILLLSLAVSDFLVGL      80
LLIPLEILRSTTCWVFGDVMCSVYWYLTGNITCASIGNIVLISVDRYVAICDPLHYPTRMTVVRIKHWICLCWFYAIFYD     160
TLYLKDLLIKPGRYNSCYGECVLVINDIAGTADLVGGSFGSYHRTSPWX
...............N................................................................      80
.............................N..................................................     160
.................................................                                    240

(Threshold=0.5)
----------------------------------------------------------------------
SeqName      Position  Potential   Jury    N-Glyc
                      agreement result
----------------------------------------------------------------------
contig022363-TilTARs.A043   16 NNSC   0.6098     (8/9)    +
contig022363-TilTARs.A043  110 NITC   0.7527     (9/9)    +++
----------------------------------------------------------------------
```

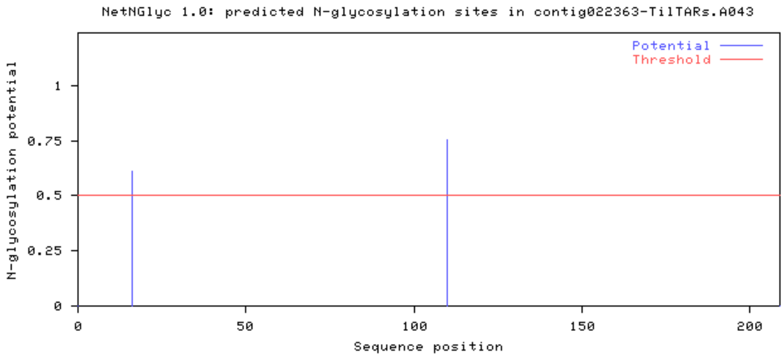

Graphics in PostScript

Output for 'contig022363-TilTARs.A044'

```
########################################################################################

    Warning: This sequence may not contain a signal peptide!!

    Proteins without signal peptides are unlikely to be exposed to
    the N-glycosylation machinery and thus may not be glycosylated
    (in vivo) even though they contain potential motifs.

    SignalP-NN euk predictions are as follows:

  # name                Cmax  pos ?  Ymax  pos ?  Smax  pos ?  Smean ?  D     ?

    SignalP output is explained at http://www.cbs.dtu.dk/services/SignalP/output.html

########################################################################################

Name:  contig022363-TilTARs.A044    Length:  326
MEIKKGDELCFVELNSSCKKPTLHWSKAVLLNIVLSCISLITAVLNLLVIISVSHFRQLHTPTNILLLSLAVSDFLVGFL      80
LMPLEIFRSTTCWVFGDVMCSVYWYLTGNITCASIGNIVLISVDRYVAICDPLHYPTRMTVVRIKHWICLCWFYAIFYDT     160
LYLKDLLIKPGRYNSCYGECVLVINDIAGTADLVLSFCVPISVIIVLYMRVFLVAVSQARAMRSHVTTVTLQRSLNQTNK     240
SELKAARTLGILVVVFLLCFCPYYCVSLVRDRNSSVAPNLMSVFFLNSCLNPLIYTMFYPWFRKAVKLIVTLQILQTGSC     320
EVSILX
..............N.................................................................      80
............................N...................................................     160
................................................................................     240
................................................................................     320
......                                                                               400

(Threshold=0.5)
----------------------------------------------------------------------
SeqName      Position  Potential   Jury    N-Glyc
                                 agreement result
----------------------------------------------------------------------
contig022363-TilTARs.A044   15 NSSC   0.6144     (7/9)   +
contig022363-TilTARs.A044  109 NITC   0.7689     (9/9)   +++
contig022363-TilTARs.A044  236 NQTN   0.4523     (5/9)   -
contig022363-TilTARs.A044  239 NKSE   0.4179     (5/9)   -
contig022363-TilTARs.A044  273 NSSV   0.3704     (7/9)   -
----------------------------------------------------------------------
```

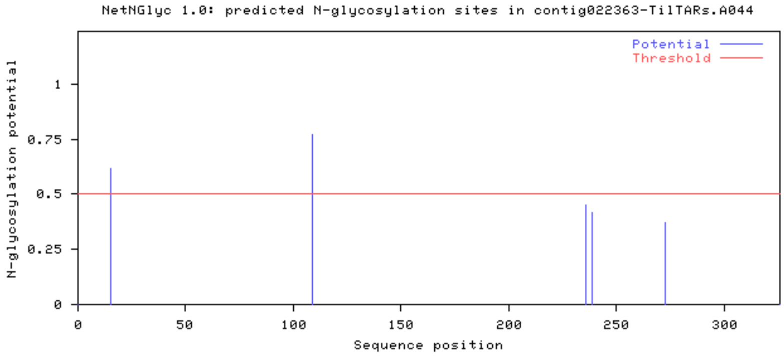

Graphics in PostScript

Output for 'contig022365-TilTARs.A045'

```
########################################################################################

    Warning: This sequence may not contain a signal peptide!!
```

```
    Proteins without signal peptides are unlikely to be exposed to
    the N-glycosylation machinery and thus may not be glycosylated
    (in vivo) even though they contain potential motifs.

    SignalP-NN euk predictions are as follows:

  # name                Cmax  pos ?  Ymax  pos ?  Smax  pos ?  Smean ?  D     ?

    SignalP output is explained at http://www.cbs.dtu.dk/services/SignalP/output.html

############################################################################

Name:  contig022365-TilTARs.A045    Length:  326
MEKGAELCFPQLNSSCRKPTLHWSKAVLLTIVLFCISLITVTLNILVIISVSHFRQLHTPSNILLPSLAVSDLLVGLLLM      80
PLEIFRNTSCWVFGDLICSVYWYLSGNITSTSTGNIVLISVDRYVAICDPLHYPTRMTVVRVKLSVCLCWFYYIFYISFY     160
LKDILIEPGRYNSCKGECVLVINDIAGIIDLVIAFIVPVSVIIVLYMRVFVVAVSQARAMRSCVRAVTLQHSLNQANKSE     240
LKAARTLGILVVVFLASFCPFYCYFFVGENVVSDSSAFFVIIAFYFNSFLNPMIYALFYPWFRNAVKLIITLQIFKRHSS     320
DTNILX
............N...................................................................      80
......N...................N.....................................................     160
................................................................................     240
................................................................................     320
......                                                                               400

(Threshold=0.5)
----------------------------------------------------------------------
SeqName      Position  Potential   Jury    N-Glyc
                                 agreement result
----------------------------------------------------------------------
contig022365-TilTARs.A045   13 NSSC   0.6494     (8/9)   +
contig022365-TilTARs.A045   87 NTSC   0.6447     (6/9)   +
contig022365-TilTARs.A045  107 NITS   0.7948     (9/9)   +++
contig022365-TilTARs.A045  237 NKSE   0.3742     (8/9)   -
----------------------------------------------------------------------
```

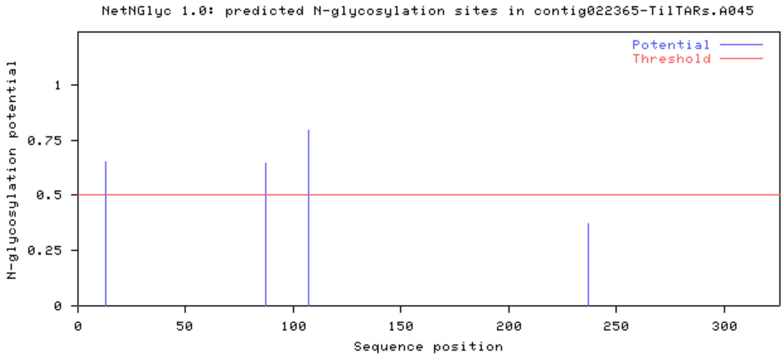

Graphics in PostScript

Output for 'contig022368-TilTARs.A046'

```
############################################################################

    Warning: This sequence may not contain a signal peptide!!

    Proteins without signal peptides are unlikely to be exposed to
    the N-glycosylation machinery and thus may not be glycosylated
    (in vivo) even though they contain potential motifs.

    SignalP-NN euk predictions are as follows:

  # name                Cmax  pos ?  Ymax  pos ?  Smax  pos ?  Smean ?  D     ?

    SignalP output is explained at http://www.cbs.dtu.dk/services/SignalP/output.html

############################################################################

Name:  contig022368-TilTARs.A046    Length:  329
MEIQKGAELCFQQLLNNSCRKLTLHWSKAVLLYIVLFSISLITVALNLLVIISVSHFRQLHTPTNILLLSLAVSDFLVGL      80
LLIPLEILRSTTCWVFGDVICSVYWYLTSNITCASTGIIVLISVDRYVAICDPLHYPTRMTVARVKLSVCLCWFYAIFYV     160
TLYLKDFLIKPGSYNSCYGECVLVINDTAGTADLVLSFFVPIIVITVLYMRVFVVAVSQARAMRSYVTAVTLQRSLNQTN     240
KSELKAARTLGVLVVVFLASYCPFYCYTLVEENVVSDSSAFFVVIAFYFNSFLNPMIYAFFYPWFRKSVKLIITLQIFKR     320
HSSDSNILX
...............N................................................................      80
.............................N..................................................     160
................................................................................     240
................................................................................     320
.........                                                                            400

(Threshold=0.5)
----------------------------------------------------------------------
SeqName      Position  Potential   Jury    N-Glyc
                                 agreement result
----------------------------------------------------------------------
contig022368-TilTARs.A046   16 NNSC   0.6107     (9/9)   ++
contig022368-TilTARs.A046  110 NITC   0.7127     (9/9)   ++
contig022368-TilTARs.A046  186 NDTA   0.4299     (7/9)   -
contig022368-TilTARs.A046  237 NQTN   0.4560     (4/9)   -
contig022368-TilTARs.A046  240 NKSE   0.4190     (5/9)   -
----------------------------------------------------------------------
```

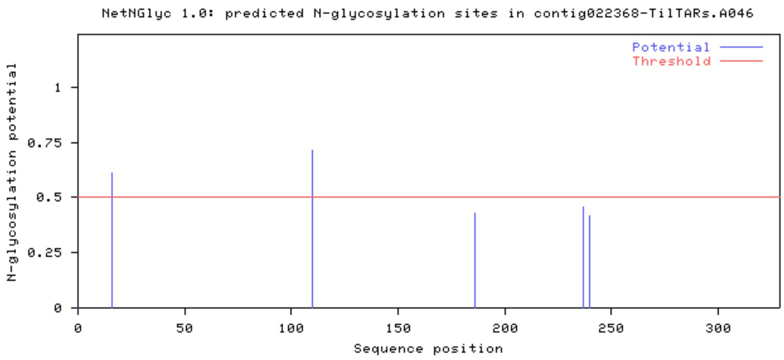

Graphics in PostScript

Output for 'contig022368-TilTARs.A047'

```
##########################################################################################

    Warning: This sequence may not contain a signal peptide!!

    Proteins without signal peptides are unlikely to be exposed to
    the N-glycosylation machinery and thus may not be glycosylated
    (in vivo) even though they contain potential motifs.

    SignalP-NN euk predictions are as follows:

  # name                Cmax  pos ?  Ymax  pos ?  Smax  pos ?  Smean ?  D     ?

    SignalP output is explained at http://www.cbs.dtu.dk/services/SignalP/output.html

##########################################################################################

Name:  contig022368-TilTARs.A047    Length:  327
MEKGAELCFPQRPNSSCGKPTLHWSKAVLLYSVLFCISLITVALNLLVIISVSHFRQLHTPSNILLLSLAVSDLLVGLLL      80
MPLEILRSTTCWVFGDVICSVYWFLSGNIICASIGNIVLISADRYVAICDPLHYPTRMTVVRVKLNVSLCWFYYVFYISL     160
YLKDILIKPGRYNSCYGECVPVINDIAGIIDLVLTFIVPVSVIIVLYMRVFVAAVSQARAMRSHVTAITFQRSLNQANKS     240
ELKAARTLGVLVIVFLACYCPFFCYTLVEEKVVSDSFVFFLIIAVYFNSFLNPLIYAFFYPWFRNAVKLIITLQIFKRHS     320
SDANILX
.............N..................................................................      80
.................................................................N..............     160
................................................................................     240
................................................................................     320
.......                                                                               400

(Threshold=0.5)
----------------------------------------------------------------------
SeqName      Position  Potential   Jury    N-Glyc
                       agreement result
----------------------------------------------------------------------
contig022368-TilTARs.A047   14 NSSC   0.6182     (9/9)    ++
contig022368-TilTARs.A047  146 NVSL   0.6366     (8/9)    +
contig022368-TilTARs.A047  238 NKSE   0.3684     (7/9)    -
----------------------------------------------------------------------
```

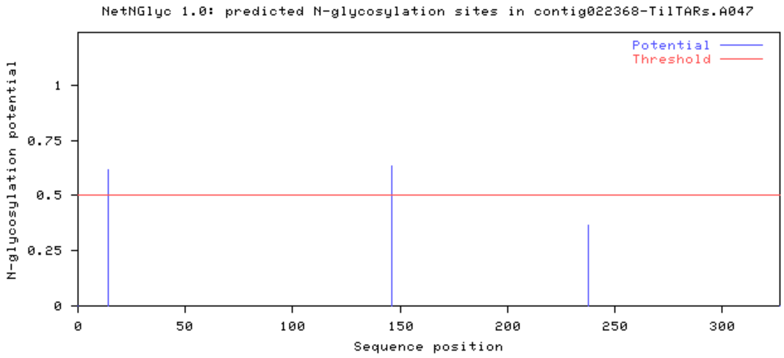

Graphics in PostScript

Output for 'contig022375-TilTARs.A048'

```
##########################################################################################

    Warning: This sequence may not contain a signal peptide!!
```

```
    Proteins without signal peptides are unlikely to be exposed to
    the N-glycosylation machinery and thus may not be glycosylated
    (in vivo) even though they contain potential motifs.

    SignalP-NN euk predictions are as follows:

  # name                Cmax  pos ?  Ymax  pos ?  Smax  pos ?  Smean ?  D     ?

    SignalP output is explained at http://www.cbs.dtu.dk/services/SignalP/output.html

########################################################################################

Name:  contig022375-TilTARs.A048    Length:  327
MEMQKGTELCFPDFLNTSCRKPTLHWSKAVLLNIVLSCISLITSALNLLVIISVSHFRQLHTPTNILLLSLAVSDFLVGL      80
LLMPLEIFRSTACWVLGDLMCSVYWCLTGNIICASIGNIVLISIDRYVAICDPLHYPTRMTVARVRVCVCLCWFFAIFYS     160
SLYTTDFLIDPGRYNSCYGECVFVISAIAGIFDLVVSFFVSVTVIIVLYMRVFVVAVSQARAMRSHVTTVTLQRSLNQTN     240
KSELKAARTLGILVVVFLLCFCPYYCVSLVRDENSSIASTVISVFFLNSCLNPLIYAMFYPWFRKAVKLTVTLQILRTGS     320
CEVNILX
...............N................................................................      80
................................................................................     160
................................................................................     240
................................................................................     320
.......                                                                              400

(Threshold=0.5)
----------------------------------------------------------------------
SeqName      Position  Potential   Jury    N-Glyc
                      agreement result
----------------------------------------------------------------------
contig022375-TilTARs.A048   16 NTSC   0.6017     (6/9)   +
contig022375-TilTARs.A048  237 NQTN   0.4526     (5/9)   -
contig022375-TilTARs.A048  240 NKSE   0.4179     (5/9)   -
contig022375-TilTARs.A048  274 NSSI   0.3514     (9/9)   --
----------------------------------------------------------------------
```

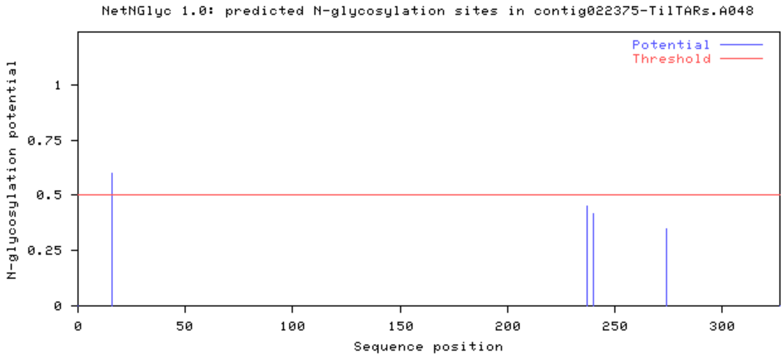

Graphics in PostScript

Output for 'contig022377-TilTARs.A050'

```
########################################################################################

    Warning: This sequence may not contain a signal peptide!!

    Proteins without signal peptides are unlikely to be exposed to
    the N-glycosylation machinery and thus may not be glycosylated
    (in vivo) even though they contain potential motifs.

    SignalP-NN euk predictions are as follows:

  # name                Cmax  pos ?  Ymax  pos ?  Smax  pos ?  Smean ?  D     ?

    SignalP output is explained at http://www.cbs.dtu.dk/services/SignalP/output.html

########################################################################################

Name:  contig022377-TilTARs.A050    Length:  327
MEKGAELCFPQLLNSSCRKPTLHWSKAVLLNIVLSCISLITAGLNLLVIISVSHFRQLHTPSNILLLSLAVSDFLVGLLL      80
IPAEIFRITVCWVFGESMCSLYTYLGYIVVTSSISNIVLISVDRYVAICDPLHYPSRITVARIKLSVCLCWFYSAFYSTL     160
CTKNILIKPGRYNSCYGECVFITSDIAGILDLVLSFIVPVSIIIVLYIRVFVVAVSQARAMRSHVTAATLQSSLNQTNKS     240
ELKAARTLGVLVVVFLASFCPFYCYFLVVEDIVSDSSASIVVIVYYFNSCLNPLIYALFYPWFRNAVKVIITFQIFKHDS     320
SEVNILX
.............N..................................................................      80
................................................................................     160
................................................................................     240
................................................................................     320
.......                                                                              400

(Threshold=0.5)
----------------------------------------------------------------------
SeqName      Position  Potential   Jury    N-Glyc
                      agreement result
----------------------------------------------------------------------
contig022377-TilTARs.A050   14 NSSC   0.6954     (9/9)   ++
contig022377-TilTARs.A050  235 NQTN   0.4534     (6/9)   -
contig022377-TilTARs.A050  238 NKSE   0.4340     (6/9)   -
----------------------------------------------------------------------
```

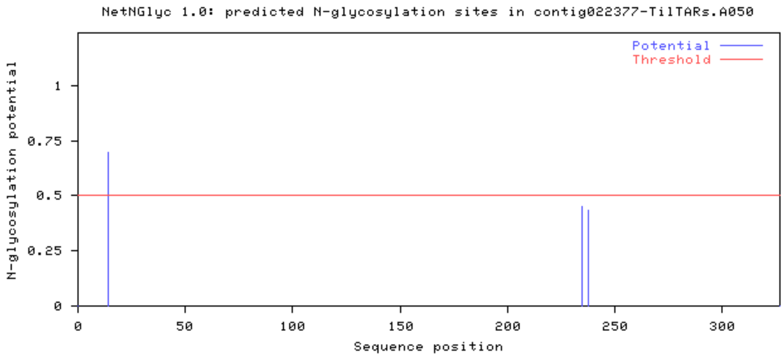

Graphics in PostScript

Output for 'contig022378-TilTARs.A051'

```
########################################################################################

    Warning: This sequence may not contain a signal peptide!!

    Proteins without signal peptides are unlikely to be exposed to
    the N-glycosylation machinery and thus may not be glycosylated
    (in vivo) even though they contain potential motifs.

    SignalP-NN euk predictions are as follows:

  # name                Cmax  pos ?  Ymax  pos ?  Smax  pos ?  Smean ?  D     ?

    SignalP output is explained at http://www.cbs.dtu.dk/services/SignalP/output.html

########################################################################################

Name:  contig022378-TilTARs.A051    Length:  329
MMENKDSICFPELFNSSCRRRALHWSETVPLNILLCSISAITIALNLLVIISVSYFRQLHTPTNILLLSLAVSDFLVGFL      80
LLPGEIFLGIACWAFGNLLCSLFNYVSFIITSASVGNMVLISVDRYVAICYPLHYSTRITVTRVKRSVCLCWLCCVLYSS     160
ALLKDELIQPGGHNSCYGECVFVINFIAGTVDLLLTFIVPISVIVVLYMRVFVVAVSQARAMRSHVTAVTLQLSVNLTTK     240
KSELKAGRTLGVLIVVFLMCFCPYYCASFAVEDSLSESSTSLVRYLFYFNSCLNPVIYALFYPWFRKAIKHIVMLQIFHT     320
GSREANILX
..............N.................................................................      80
................................................................................     160
...........................................................................N....     240
................................................................................     320
.........                                                                            400

(Threshold=0.5)
----------------------------------------------------------------------
SeqName      Position  Potential   Jury    N-Glyc
                      agreement result
----------------------------------------------------------------------
contig022378-TilTARs.A051   15 NSSC   0.6688     (9/9)   ++
contig022378-TilTARs.A051  236 NLTT   0.5283     (7/9)   +
----------------------------------------------------------------------
```

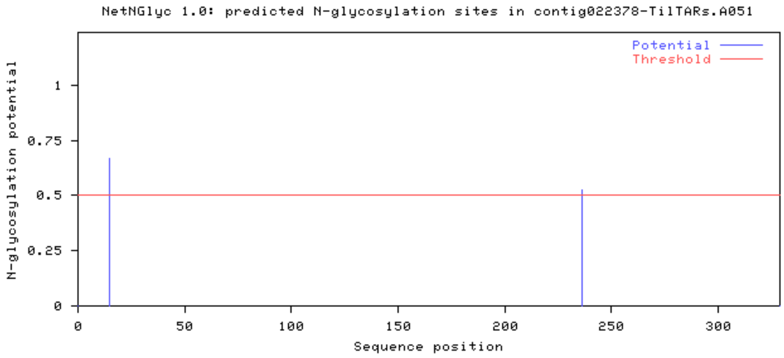

Graphics in PostScript

Output for 'contig022379-TilTARs.A052'

```
########################################################################################

    Warning: This sequence may not contain a signal peptide!!

    Proteins without signal peptides are unlikely to be exposed to
    the N-glycosylation machinery and thus may not be glycosylated
    (in vivo) even though they contain potential motifs.
```

```
    SignalP-NN euk predictions are as follows:

  # name                 Cmax  pos ?  Ymax  pos ?  Smax  pos ?  Smean ?  D     ?

    SignalP output is explained at http://www.cbs.dtu.dk/services/SignalP/output.html

##########################################################################################

Name:  contig022379-TilTARs.A052    Length:  331
MDTQDVAELCFPQLFNTSCKKPITPLSEFVFLHVVLSSISLLTVTLNLLVIISVSHYRQLHTPTNILLLSLAVSDFLVGL      80
LLMPGEILRNTACWFLGDLTCSMYNYMSFIVTSTSVGDMVLISIDRYLAICDPLHYPTRITDRRVKLSICLCWLSSVFYS     160
SLFVKDDLTQPGKHNSCYGECTIVVDLITGIIDLLLTFFVPVTVIVVLYLRVFVVAVSQARAMRSHVTATALQLSVTLTT     240
KKSELKAARTLGVLVVVFLLCFCPYYYCVTLARDDPLNSSSVSFVLYLFYFNSCLNPLIYALFYPWFRKAVKLIISLHIL     320
QPGSCEISILX
...............N................................................................      80
................................................................................     160
................................................................................     240
.....................................N..........................................     320
...........                                                                          400

(Threshold=0.5)
----------------------------------------------------------------------
SeqName      Position  Potential   Jury    N-Glyc
                      agreement result
----------------------------------------------------------------------
contig022379-TilTARs.A052   16 NTSC   0.5823     (7/9)   +
contig022379-TilTARs.A052  278 NSSS   0.5791     (5/9)   +
----------------------------------------------------------------------
```

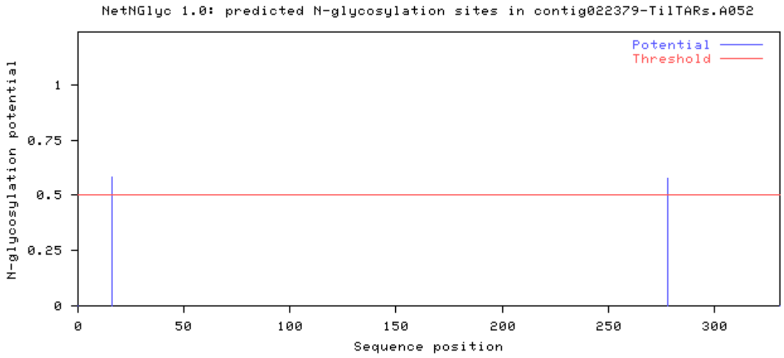

Graphics in PostScript

Output for 'contig022382-TilTARs.A053'

```
##########################################################################################

    Warning: This sequence may not contain a signal peptide!!

    Proteins without signal peptides are unlikely to be exposed to
    the N-glycosylation machinery and thus may not be glycosylated
    (in vivo) even though they contain potential motifs.

    SignalP-NN euk predictions are as follows:

  # name                 Cmax  pos ?  Ymax  pos ?  Smax  pos ?  Smean ?  D     ?

    SignalP output is explained at http://www.cbs.dtu.dk/services/SignalP/output.html

##########################################################################################

Name:  contig022382-TilTARs.A053    Length:  329
MGTQEKSELCFPQLFNMSCKKPAIARSKAVFLHIVLSSVSFLTVALNLLVIISVSHFRQLHTPTNILLLSLAVSDFLVGL      80
LLSPAEILRSTACWFLGQLTCLMYIFVSLTVTSASVGIMVLISADRYVAICDPLNYPIRITDRRVQLCVCLCWLCSIAFS     160
CFLVRDDLHQTRKQNSCYGKCVVVVQYIAGVVDLILTFIVPVSVIIVLYMRVFVVAVSQARAMRSHVTAVTSHLPVTLTK     240
KSELKAARTLGVLVLVFLMCFCPYYCVSLVGEEFINSSSASFVAYLFGLNSCLNPLIYAMFYPWFRKAVKLVVTLQILQP     320
GSCEVSILX
...............N................................................................      80
................................................................................     160
................................................................................     240
................................................................................     320
.........                                                                            400

(Threshold=0.5)
----------------------------------------------------------------------
SeqName      Position  Potential   Jury    N-Glyc
                      agreement result
----------------------------------------------------------------------
contig022382-TilTARs.A053   16 NMSC   0.5922     (8/9)   +
contig022382-TilTARs.A053  276 NSSS   0.4743     (5/9)   -
----------------------------------------------------------------------
```

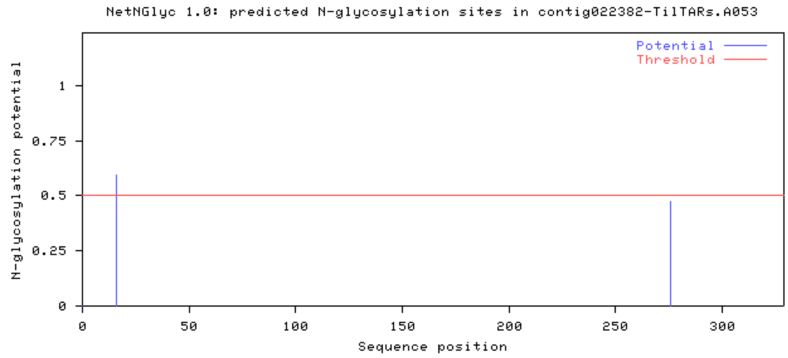

Graphics in PostScript

Output for 'contig022383-TilTARs.A054'

```
##########################################################################################

    Warning: This sequence may not contain a signal peptide!!

    Proteins without signal peptides are unlikely to be exposed to
    the N-glycosylation machinery and thus may not be glycosylated
    (in vivo) even though they contain potential motifs.

    SignalP-NN euk predictions are as follows:

  # name                Cmax  pos ?  Ymax  pos ?  Smax  pos ?  Smean ?  D     ?

    SignalP output is explained at http://www.cbs.dtu.dk/services/SignalP/output.html

##########################################################################################

Name:  contig022383-TilTARs.A054    Length:  330
METQDEAELCFPQLFNISCKKPKTSLSQVLLPYVVVYSVSLLTVALNLLVIVSVSHFRQLHTPTNILLLSLAVSDFLIGL      80
LMMPAKILRDTACWFLGQLTCLIYNYICFIITSASVGIMVLISVDRYVAICDPLHYPTRITEKRVKLCVCLCWLCSVFYN     160
ILFIKDDLLQREQHTSCYGECVFVIDYIVGTTDIVVTFIAPVTVIVILYMRVFVVAVSQARAMRSHVTAVTLQLSVTLTA     240
KKSELKAARTLGVLVLVFLLCFCPYYIVSLLGNELFNSSSASIVIYLYYFNSCLNPLIYAMFYPWFRKAVKLVVTLQILQ     320
PGSCEVSILX
...............N................................................................      80
................................................................................     160
................................................................................     240
................................................................................     320
..........                                                                           400

(Threshold=0.5)
----------------------------------------------------------------------
SeqName      Position  Potential   Jury    N-Glyc
                     agreement result
----------------------------------------------------------------------
contig022383-TilTARs.A054   16 NISC   0.6532     (8/9)   +
contig022383-TilTARs.A054  277 NSSS   0.4913     (4/9)   -
----------------------------------------------------------------------
```

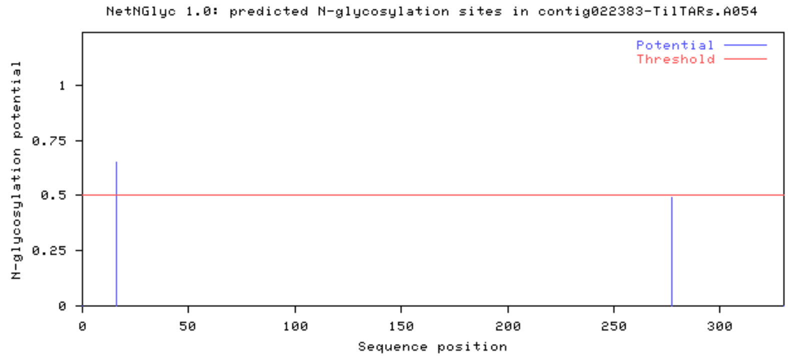

Graphics in PostScript

Output for 'contig022390-TilTARs.A055'

```
##########################################################################################

    Warning: This sequence may not contain a signal peptide!!

    Proteins without signal peptides are unlikely to be exposed to
    the N-glycosylation machinery and thus may not be glycosylated
    (in vivo) even though they contain potential motifs.
```

```
    SignalP-NN euk predictions are as follows:

  # name                Cmax  pos ?  Ymax  pos ?  Smax  pos ?  Smean ?  D     ?

    SignalP output is explained at http://www.cbs.dtu.dk/services/SignalP/output.html

##########################################################################################

Name:  contig022390-TilTARs.A055    Length:  300
MDTQEGAELCFPQLFNISCKKPTTPLSQVLLLYIVLSSMSLLTVTLNLLVIIAVSHFRRNTACWFLGQLTCSLYNYVSYI      80
VASASVGNMVLISVDRYVAICDPLHYPSRITDKRVKLCVCLCWLCSVFYSYVILIDDLSQPGKHNSCYGKCIIFIEFIAG     160
FVDLVLSFIIPLTVIVVLYMRVFVVAVSQARAMRSHVTAVTLQLSVTLTAKKSELKAARTLGVLVLVFLICFCPYYIVSL     240
FGDEFLNSSSASIVIYLYYFNSCVNPLIYAMFYPWFRKAVKLIVTLQILQPDSCEVSILX
...............N................................................................      80
................................................................................     160
................................................................................     240
......N.....................................................                         320

(Threshold=0.5)
----------------------------------------------------------------------
SeqName      Position  Potential   Jury    N-Glyc
                      agreement result
----------------------------------------------------------------------
contig022390-TilTARs.A055   16 NISC   0.6416     (8/9)    +
contig022390-TilTARs.A055  247 NSSS   0.5016     (4/9)    +
----------------------------------------------------------------------
```

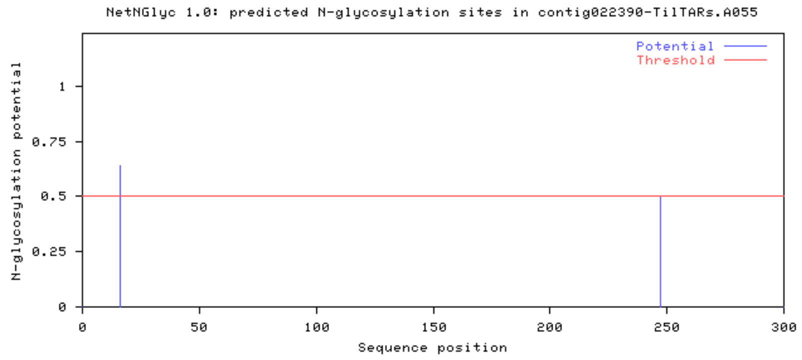

Graphics in PostScript

Output for 'contig022390-TilTARs.A056'

```
##########################################################################################

    Warning: This sequence may not contain a signal peptide!!

    Proteins without signal peptides are unlikely to be exposed to
    the N-glycosylation machinery and thus may not be glycosylated
    (in vivo) even though they contain potential motifs.

    SignalP-NN euk predictions are as follows:

  # name                Cmax  pos ?  Ymax  pos ?  Smax  pos ?  Smean ?  D     ?

    SignalP output is explained at http://www.cbs.dtu.dk/services/SignalP/output.html

##########################################################################################

Name:  contig022390-TilTARs.A056    Length:  330
MDTQDGLELCFPQLFNISCKKPTTPLSQVLLPYIVVFSMSLLTVTLNLLVIIAVSHFRQLHTPTNILLLSLAVSDFLIGL      80
LLMPANIIRKKVCWFLGQLTCLVYTYVSFIITSASVGIMVLISVDRYVAICDPLHYPTRITHGRVKLCVCLCWLCSVIYN     160
ILLIKDELVKPGQHISCYGECKILVDYIAGTTDIVLTFIIPVTVIIVLYMRVFVVAVSQARAMRSHVTAVTMQLSVTLTT     240
KKSELKAARTLGVLVLVFLICFCPHYCIFVFGGKLLSSSSATIVIYLYYFNSCVNPLIYAMFYPWFRKAVKLTVTLQILQ     320
PGSCEVSILX
...............N................................................................      80
................................................................................     160
................................................................................     240
................................................................................     320
..........                                                                           400

(Threshold=0.5)
----------------------------------------------------------------------
SeqName      Position  Potential   Jury    N-Glyc
                      agreement result
----------------------------------------------------------------------
contig022390-TilTARs.A056   16 NISC   0.6331     (8/9)    +
----------------------------------------------------------------------
```

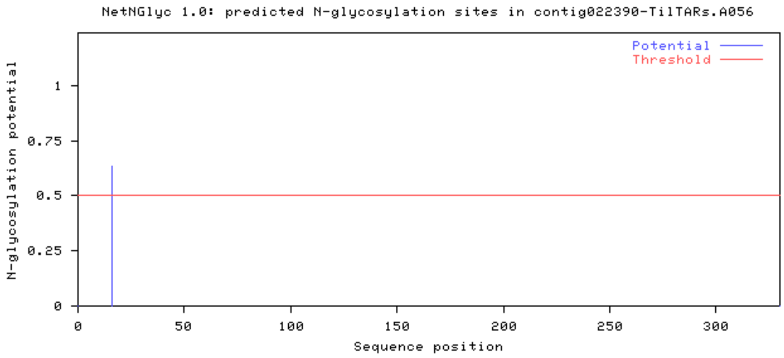

Graphics in PostScript

Output for 'contig045088-TilTARs.A057'

```
########################################################################################

    Warning: This sequence may not contain a signal peptide!!

    Proteins without signal peptides are unlikely to be exposed to
    the N-glycosylation machinery and thus may not be glycosylated
    (in vivo) even though they contain potential motifs.

    SignalP-NN euk predictions are as follows:

  # name                Cmax  pos ?  Ymax  pos ?  Smax  pos ?  Smean ?  D     ?

    SignalP output is explained at http://www.cbs.dtu.dk/services/SignalP/output.html

########################################################################################

Name:  contig045088-TilTARs.A057    Length:  331
MEGVEFCFPHLLNSSCRKALRPVSVSMLIYMIISSISVLTATLNLLVIVSISHFKQLHTPTNFLLLSLAVSDFFVGLYLL      80
FYIMFIDGCWYFGEFMCILYYVIGTINTSSSIGTMVLISVDRYVAICDPLHYPIKVTAKRVQTCVSLCWSFSALAGSYLL     160
KDNLKQQSRFNSCVGECVVHINFIEYVADLALNFLLPITVIIVLYLRIFVVVVSQVRAMRSHTAGVTYQCSRKGNPKKSE     240
MKAARTLGIVVIAFLSCTLPFYCVTLTGQNAFLNGSSSAFVLCLFYFNSCLNPIVYALFYPWFRKSIKFIVTFQILKSGS     320
RNANIVKVTEX
............N...................................................................      80
..........................N.....................................................     160
................................................................................     240
.................................N..............................................     320
...........                                                                          400

(Threshold=0.5)
----------------------------------------------------------------------
SeqName      Position  Potential   Jury    N-Glyc
                                 agreement result
----------------------------------------------------------------------
contig045088-TilTARs.A057   13 NSSC   0.6669     (9/9)    ++
contig045088-TilTARs.A057  107 NTSS   0.6154     (7/9)    +
contig045088-TilTARs.A057  274 NGSS   0.6617     (7/9)    +
----------------------------------------------------------------------
```

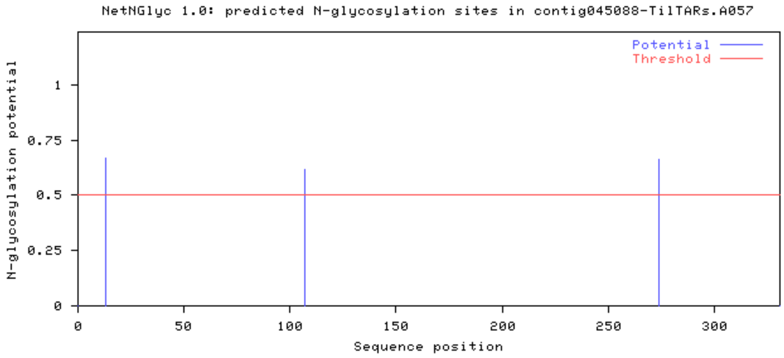

Graphics in PostScript

Output for 'contig056134-TilTARs.A058'

```
########################################################################################

    Warning: This sequence may not contain a signal peptide!!

    Proteins without signal peptides are unlikely to be exposed to
    the N-glycosylation machinery and thus may not be glycosylated
```

```
    (in vivo) even though they contain potential motifs.

    SignalP-NN euk predictions are as follows:

  # name                 Cmax  pos ?  Ymax  pos ?  Smax  pos ?  Smean ?  D     ?

    SignalP output is explained at http://www.cbs.dtu.dk/services/SignalP/output.html

##########################################################################################

Name:  contig056134-TilTARs.A058    Length:  326
MEETELCFPKLLNTSCMRLRRPHFEIMLTYILLSSIALLTVTLNLFVIISISHFRQLHTRNNVLLLSLSAADFGVGLVMF      80
FQIVLIDGCWFLGDIMCTLYQYLAYVITSASIGTMVIISVDRYLAICYPLHYSTKITQQRVKIVVCLCWICSVIFQSLIL     160
MDNLKQPGRYNSCIGECVFVINYIAGLVDVTFSFIVPITVIVVLYLRVFVVAVSQAHAMRSQLAVTHQQSVTVTAKKSEL     240
KAAWTLGIVVVVFLICMCPYYCVALTVKDNALSASSAAFVIYLFFFNSCLNPILYVFFYSWFRKSIKVIVTLQILQPDSS     320
KANMLX
............N...................................................................      80
................................................................................     160
................................................................................     240
................................................................................     320
......                                                                               400

(Threshold=0.5)
----------------------------------------------------------------------
SeqName      Position  Potential   Jury    N-Glyc
                                 agreement result
----------------------------------------------------------------------
contig056134-TilTARs.A058   13 NTSC   0.7543     (9/9)   +++
----------------------------------------------------------------------
```

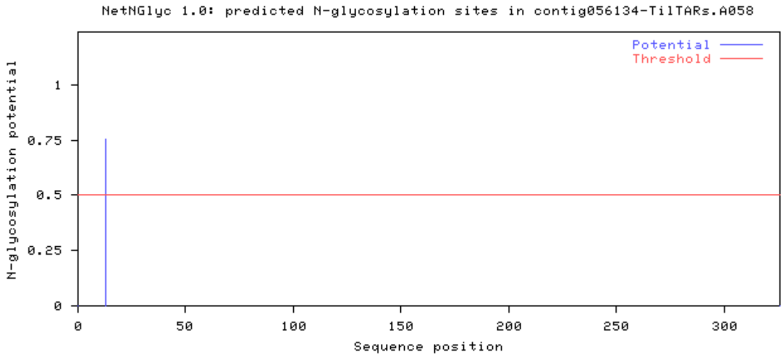

Graphics in PostScript

Output for 'contig022320-TilTARs.A059'

```
##########################################################################################

    Warning: This sequence may not contain a signal peptide!!

    Proteins without signal peptides are unlikely to be exposed to
    the N-glycosylation machinery and thus may not be glycosylated
    (in vivo) even though they contain potential motifs.

    SignalP-NN euk predictions are as follows:

  # name                 Cmax  pos ?  Ymax  pos ?  Smax  pos ?  Smean ?  D     ?

    SignalP output is explained at http://www.cbs.dtu.dk/services/SignalP/output.html

##########################################################################################

Name:  contig022320-TilTARs.A059    Length:  329
MEIQKAAELCFPQLLNSSCRKPTLHWSKAVLLNIVLFCISVITAALNLLVIISVSHFRKLHTPSNILLVSLAVSDFLVGL      80
LLMPLEIFRNTACWVLGDVMCSVYWYLTSNITCASIGNIVLISVDRYVAICDPLHYPTRITVVRVKLSVYLCWFFSIFYW     160
SLYMKDILVEPGRYNSCYGECVLIINDIAGIVDLALSFIVPVTVIIVLYMRVFVVAVSQARAMRSHVTAVTLQCSLNQAN     240
KSELKAARTLGVLVVVFLACYCPFYCYSLVDKNVVNDSSASFVVLVFYFNSCLNPLIYALFYPWFRKAIKCVITLLIFKH     320
DSSEVNVIX
...............N................................................................      80
.............................N..................................................     160
................................................................................     240
................................................................................     320
.........                                                                            400

(Threshold=0.5)
----------------------------------------------------------------------
SeqName      Position  Potential   Jury    N-Glyc
                                 agreement result
----------------------------------------------------------------------
contig022320-TilTARs.A059   16 NSSC   0.7031     (9/9)   ++
contig022320-TilTARs.A059  110 NITC   0.7317     (9/9)   ++
contig022320-TilTARs.A059  240 NKSE   0.3682     (8/9)   -
contig022320-TilTARs.A059  276 NDSS   0.3996     (7/9)   -
----------------------------------------------------------------------
```

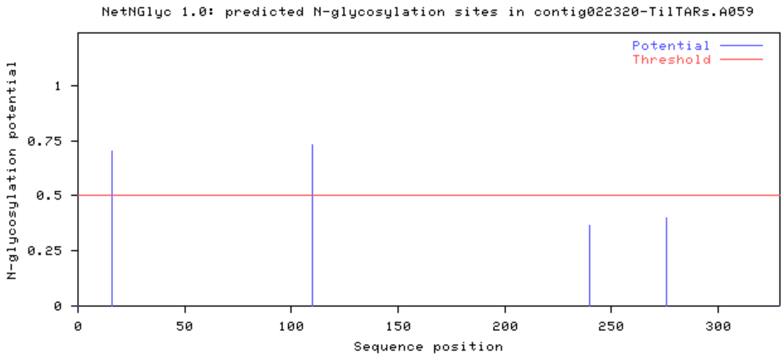

Graphics in PostScript

Output for 'contig007518-TilTARs.A019'

```
########################################################################################

    Warning: This sequence may not contain a signal peptide!!

    Proteins without signal peptides are unlikely to be exposed to
    the N-glycosylation machinery and thus may not be glycosylated
    (in vivo) even though they contain potential motifs.

    SignalP-NN euk predictions are as follows:

  # name                Cmax  pos ?  Ymax  pos ?  Smax  pos ?  Smean ?  D     ?

    SignalP output is explained at http://www.cbs.dtu.dk/services/SignalP/output.html

########################################################################################

Name:  contig007518-TilTARs.A019    Length:  331
MKTIDRAELCFSQLNSSCRKTMHPRSLSVLFYVILSSISVLTVALNLLVIISISHFRQLHTPTNLLLFSLAVSDCFVGLL      80
TVFQILVIDGCWYLGEQLCILYYIFDYVVTNASIGTMVLISVDRYVAICDPLYYPSKVTLKRVRTSVSLCWIFSLFCVFV     160
LLKDNLGQPGSYNSCSGECVVLVYYVTGIVDILLSFIGPVIVIIALYLKVFMVVVTQARSMRSRIEAVAVQGSVTVTIKK     240
SEIKAARVLGIVVVVFLACLLPYFCIGLTGDKTLLNASTATVFITLFYFNSCVNPLVYAFFYPWFRRTIKLIVTLKILNS     320
RSCKANLLISX
..............N.................................................................      80
................................................................................     160
................................................................................     240
................................................................................     320
...........                                                                          400

(Threshold=0.5)
----------------------------------------------------------------------
SeqName      Position  Potential   Jury    N-Glyc
                     agreement result
----------------------------------------------------------------------
contig007518-TilTARs.A019   15 NSSC   0.6466     (9/9)   ++
contig007518-TilTARs.A019  111 NASI   0.4902     (6/9)   -
contig007518-TilTARs.A019  276 NAST   0.4199     (7/9)   -
----------------------------------------------------------------------
```

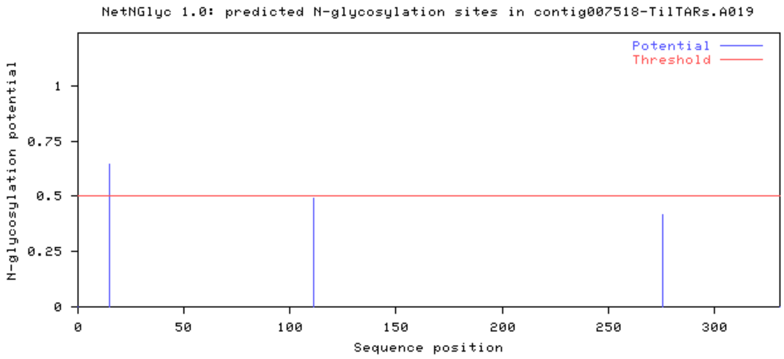

Graphics in PostScript

Output for 'contig032272-NyeTAR.A004'

```
########################################################################################

    Warning: This sequence may not contain a signal peptide!!

    Proteins without signal peptides are unlikely to be exposed to
    the N-glycosylation machinery and thus may not be glycosylated
```

```
    (in vivo) even though they contain potential motifs.

    SignalP-NN euk predictions are as follows:

  # name                 Cmax  pos ?  Ymax  pos ?  Smax  pos ?  Smean ?  D     ?

    SignalP output is explained at http://www.cbs.dtu.dk/services/SignalP/output.html

##########################################################################################

Name:  contig032272-NyeTAR.A004     Length:  331
MDRQDRIELCYPELANLSCRVLTRPQPEAVLLYTLFLSISVLTVALNVLVIISISHFRQLHMPTNVLLLSLAISDLLVGL      80
LVMPVEAMRFIETCWLLGDLMCAFTYIIGFTLTSASVGNMVLISIDRYVAICYPLQYPTKITHSRAELSVTLCWACSLLY     160
NGMILKEHLRQPDRHNTCHGQCLVVINYVPGAIDLVFTFIGPCSVIIILYMRVFVVAVSQAHAMRSHITAAAAGTVKITA     240
KKSEKKAARTLGVVIFVFLMSFCPYYYPSLAGQDISNSASSWAIVSWMLYFNSCLNPLIYAFFYPWFRKAIWFIVSLKIL     320
EKGSSQANILX
...............N................................................................      80
................................................................................     160
................................................................................     240
................................................................................     320
...........                                                                          400

(Threshold=0.5)
----------------------------------------------------------------------
SeqName      Position  Potential   Jury    N-Glyc
                      agreement result
----------------------------------------------------------------------
contig032272-NyeTAR.A004   16 NLSC   0.6882     (9/9)    ++
----------------------------------------------------------------------
```

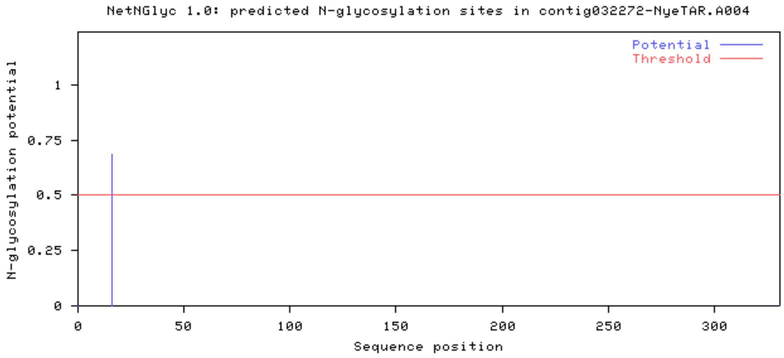

Graphics in PostScript

Output for 'contig037879-TilTAR.B060'

```
##########################################################################################

    Warning: This sequence may not contain a signal peptide!!

    Proteins without signal peptides are unlikely to be exposed to
    the N-glycosylation machinery and thus may not be glycosylated
    (in vivo) even though they contain potential motifs.

    SignalP-NN euk predictions are as follows:

  # name                 Cmax  pos ?  Ymax  pos ?  Smax  pos ?  Smean ?  D     ?

    SignalP output is explained at http://www.cbs.dtu.dk/services/SignalP/output.html

##########################################################################################

Name:  contig037879-TilTAR.B060     Length:  317
MHPCYESENATYTFTNNPSIACVILYIFLGSLSVVTIFGNLLVTIAIIYFKQLHTPTNYLILSLAVADLLVGVLVFPFSM      80
AFTVTLCLDHEDLFCKIRDSFDVSLCTSSILNLCCISIERYYAVCQPLNYRSKINTQVTMFMILVSWGISVLIGICIIIA     160
GFSQGMCEEVCSVDVVIANTMGPVFSFYLPAIIMLCIYLKIFLVAQKQVSTIQNAICTKSGATISKLERKATKTLSIVMG     240
VFLLCLSPYFICVVFQPLAHNPPPIPVIETLNWLTLSNSMLNPLIYAFFYSWFRSAYRLIVSGTILHCNLANSKLFX
........N.......N...............................................................      80
................................................................................     160
................................................................................     240
.............................................................................        320

(Threshold=0.5)
----------------------------------------------------------------------
SeqName      Position  Potential   Jury    N-Glyc
                      agreement result
----------------------------------------------------------------------
contig037879-TilTAR.B060    9 NATY   0.7432     (9/9)    ++
contig037879-TilTAR.B060   17 NPSI   0.6793     (8/9)    +     WARNING: PRO-X1.
----------------------------------------------------------------------
```

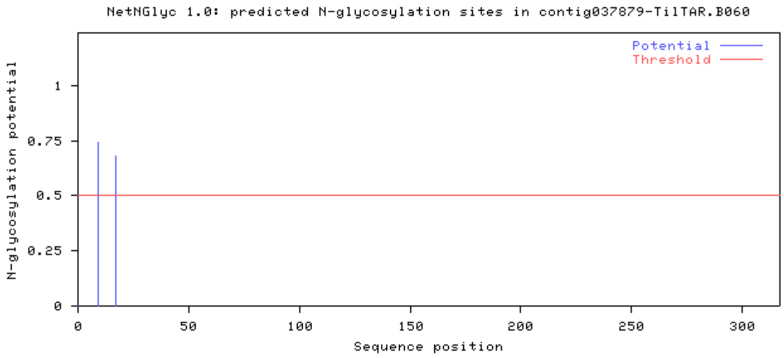

Graphics in PostScript

Output for 'contig037889-TilTAR.B061'

```
##########################################################################################

    Warning: This sequence may not contain a signal peptide!!

    Proteins without signal peptides are unlikely to be exposed to
    the N-glycosylation machinery and thus may not be glycosylated
    (in vivo) even though they contain potential motifs.

    SignalP-NN euk predictions are as follows:

  # name                Cmax  pos ?  Ymax  pos ?  Smax  pos ?  Smean ?  D     ?

    SignalP output is explained at http://www.cbs.dtu.dk/services/SignalP/output.html

##########################################################################################

Name:  contig037889-TilTAR.B061     Length:  327
MTIIVNDLHPCYVIQDSKYMLTNSPSIICVFLYALLALLSVITICGNLLVIISVIYFKQLHTPTNSLILSLAVADLLIGI      80
IAFPLSMAFSLSSCLYHEGLFCKVRGTFDISLSTCSILNLCCISIDRYHAVCQPLTYQTKISHRAVVFMILMSWGVSGII     160
GISVTIAGLNNEKYEESFLIDVLMESTVGPMLSFYLPVIIMLCIYLKIFFVARRQARSIQNTTKCGQTASMMERKATKTL     240
AIVLGVFILCWSPFFLCITFPPFTSDYQYQLQVPVPVIETLNWLTLANSMLNPFIYAFFYSWFRSAIRMIICGKIFQGDF     320
ANTKLTX
................................................................................      80
................................................................................     160
............................................................N...................     240
................................................................................     320
.......                                                                              400

(Threshold=0.5)
----------------------------------------------------------------------
SeqName      Position  Potential   Jury    N-Glyc
                      agreement result
----------------------------------------------------------------------
contig037889-TilTAR.B061  221 NTTK   0.5804     (6/9)   +
----------------------------------------------------------------------
```

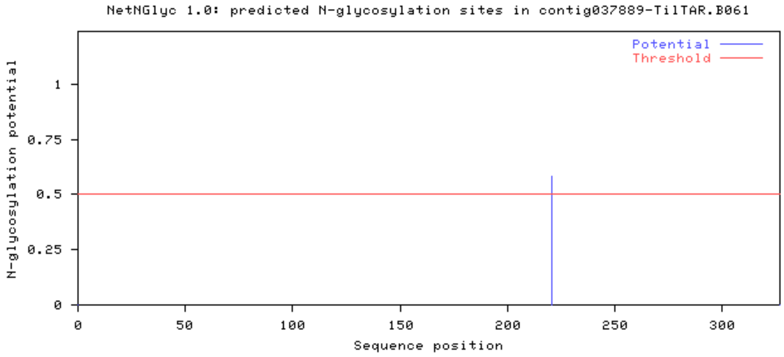

Graphics in PostScript

Output for 'contig002574-TilTAR.B062'

```
##########################################################################################

    Warning: This sequence may not contain a signal peptide!!

    Proteins without signal peptides are unlikely to be exposed to
    the N-glycosylation machinery and thus may not be glycosylated
    (in vivo) even though they contain potential motifs.
```

```
    SignalP-NN euk predictions are as follows:

  # name                  Cmax  pos ?  Ymax  pos ?  Smax  pos ?  Smean ?  D     ?

    SignalP output is explained at http://www.cbs.dtu.dk/services/SignalP/output.html

########################################################################################

Name:  contig002574-TilTAR.B062     Length:  322
MTNIVNDLHPCYVIQDSKYMLTNSPSIICVFLYAFLALLSVITICGNLLVIISVIYFKQLHTPTNSLILSLAVADLLVGI      80
IAFPLSMAFSLSSCLYHEGLFCKVRGTFDISLSTCSILNLCCISVDRYHAVCQPLTYQTKISHRVVVFMILMSWGVSGII     160
GISVTIAGFNNEKCEESCLIDVLMESTVGPMLSFYLPVTIMLCIYLKIFFVALKQASSIQNTTKCGQTASIMERKATKTL     240
AIVLGVFIFCWSPCFLCITFPPFTSDSVPVPVIETLNWLALANSMLNPFIYAFFYRWFRSAIRMIICGKIFQRDFANTNM     320
TX
................................................................................      80
................................................................................     160
............................................................N...................     240
................................................................................     320
..                                                                                   400

(Threshold=0.5)
----------------------------------------------------------------------
SeqName      Position  Potential   Jury    N-Glyc
                                 agreement result
----------------------------------------------------------------------
contig002574-TilTAR.B062  221 NTTK   0.5476     (4/9)   +
contig002574-TilTAR.B062  319 NMTX   0.4829     (6/9)   -
----------------------------------------------------------------------
```

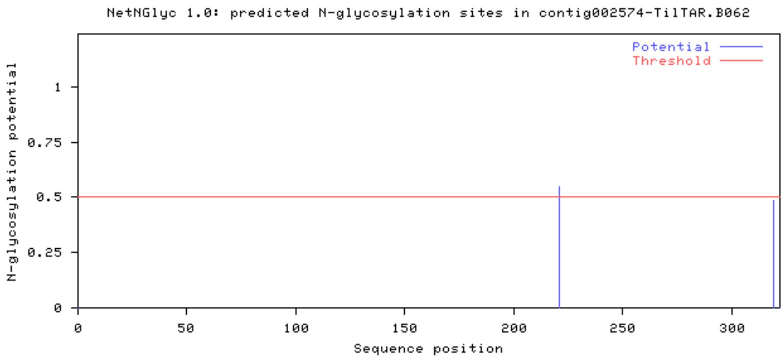

Graphics in PostScript

Output for 'contig037900-TilTAR.B063'

```
########################################################################################

    Warning: This sequence may not contain a signal peptide!!

    Proteins without signal peptides are unlikely to be exposed to
    the N-glycosylation machinery and thus may not be glycosylated
    (in vivo) even though they contain potential motifs.

    SignalP-NN euk predictions are as follows:

  # name                  Cmax  pos ?  Ymax  pos ?  Smax  pos ?  Smean ?  D     ?

    SignalP output is explained at http://www.cbs.dtu.dk/services/SignalP/output.html

########################################################################################

Name:  contig037900-TilTAR.B063     Length:  302
MAPDVPVNRTDADDLHPCYKIDKGSYIVKSNPSTLCVLLHIFLGSLSLVTVCGNLLVIISIIYFKQLHTLTNSLVLSLAV      80
ADLVVGVLVFPLSMRFTVSVCLFYQDLFCKVRDSFDVTLSTSSILNLCCISIDRYYAVCRPLTYRSKINVHVVVIMILVS     160
WSVSILVAVGFLIAGLNHEKCKEGWISELLLANILGPVLSFYLPVIIMLCIYLKIFLVAEKHARSIQNTKCGATVVSKME     240
KKATKTLATVMGVFVMCWLPFFFCVTLQLLGHILIPLPVYETLNWLALCNSMLNPFIYAFFX
.......N......................N.................................................      80
................................................................................     160
................................................................................     240
..............................................................                       320

(Threshold=0.5)
----------------------------------------------------------------------
SeqName      Position  Potential   Jury    N-Glyc
                                 agreement result
----------------------------------------------------------------------
contig037900-TilTAR.B063    8 NRTD   0.7521     (9/9)   +++
contig037900-TilTAR.B063   31 NPST   0.5144     (4/9)   +    WARNING: PRO-X1.
----------------------------------------------------------------------
```

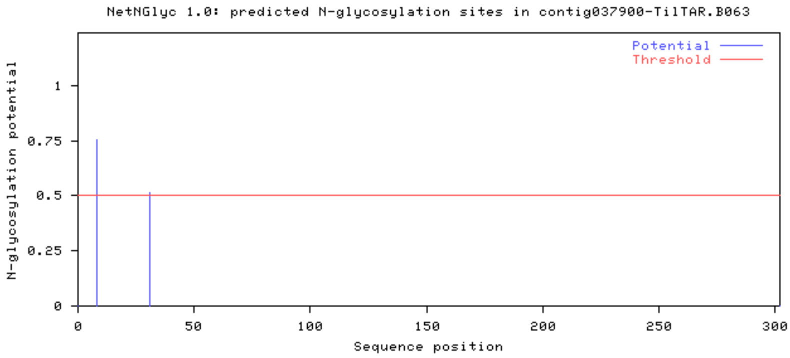

Graphics in PostScript

Output for 'contig066890-ZebTARs.A014'

```
##########################################################################################

    Warning: This sequence may not contain a signal peptide!!

    Proteins without signal peptides are unlikely to be exposed to
    the N-glycosylation machinery and thus may not be glycosylated
    (in vivo) even though they contain potential motifs.

    SignalP-NN euk predictions are as follows:

  # name                Cmax  pos ?  Ymax  pos ?  Smax  pos ?  Smean ?  D     ?

    SignalP output is explained at http://www.cbs.dtu.dk/services/SignalP/output.html

##########################################################################################

Name:  contig066890-ZebTARs.A014    Length:  330
MDTQDGEELCFPQLFNISCKKPTTPLSQLLLPYIVVFSMSLLTVILNLLVIIAVSHFRQLHTPTNFLLLSLAVSDFFIGL      80
LLMPANIIRKKVCWFLGQLACLVFTYVSFIITSASVGIMVLISVDRYAAICDPLHYPTRITRERVKLCVCLCWLCSVIYN     160
ILLIKDELVQPGQHNSCYGECKILVDYIAGTTDIVLTFIAPITVIIVLYMRVFVVAVSQARTMRSQVTAVTLQLSVTLTT     240
KKSELKAARTLGVLVLVFLICFCPHYCIFVFGGKVLNSSSATIVIYLYYFNSCLNPLIYAMFYPWFRKAVKLIVTLQILQ     320
PGSCEVSILX
...............N................................................................      80
................................................................................     160
................................................................................     240
....................................N...........................................     320
..........                                                                           400

(Threshold=0.5)
----------------------------------------------------------------------
SeqName      Position  Potential   Jury    N-Glyc
                      agreement result
----------------------------------------------------------------------
contig066890-ZebTARs.A014   16 NISC   0.6361     (8/9)   +
contig066890-ZebTARs.A014  277 NSSS   0.5306     (5/9)   +
----------------------------------------------------------------------
```

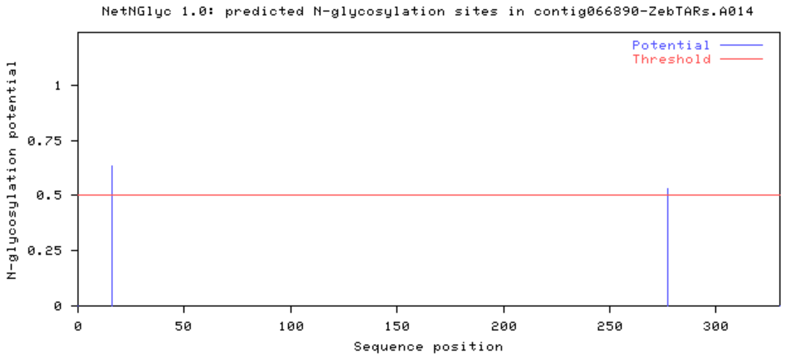

Graphics in PostScript

Output for 'contig066691-ZebTARs.A015'

```
##########################################################################################

    Warning: This sequence may not contain a signal peptide!!

    Proteins without signal peptides are unlikely to be exposed to
    the N-glycosylation machinery and thus may not be glycosylated
    (in vivo) even though they contain potential motifs.
```

```
    SignalP-NN euk predictions are as follows:

  # name                  Cmax  pos ?  Ymax  pos ?  Smax  pos ?  Smean ?  D     ?

    SignalP output is explained at http://www.cbs.dtu.dk/services/SignalP/output.html

##########################################################################################

Name:  contig066691-ZebTARs.A015    Length:  329
MEIPKGVELCFPQLLNSSCRKPTLHWSKAVLLNIVLSCISLLTAALNLLVIISVSHFRQLHTPSNILLLSLAVSDFFVGL      80
LLLPLEIFRNTSCWVLGDRMCSAYWYLTSNIICASIGNIVLISVDRYVAICDPLHYPSRITLAKVKLSVCLCWFYAFFYS     160
NLYTKNIMIEPGRYNSCFGECVFFSSNIAIVADLILFFFVPVTVIIALYMRVFVVAVSQARAMRSHVTLVTLQRSLNQTN     240
KSELKAARTLGILVVVFLACFSPLYCYSLVDENAINNPAASFAVIIFYINSCLNPLIYALFYPWFRNAVKLIITLEIFKY     320
DTSGANILX
...............N................................................................      80
.........N......................................................................     160
................................................................................     240
................................................................................     320
.........                                                                            400

(Threshold=0.5)
----------------------------------------------------------------------
SeqName      Position  Potential   Jury    N-Glyc
                                 agreement result
----------------------------------------------------------------------
contig066691-ZebTARs.A015   16 NSSC   0.6896     (9/9)    ++
contig066691-ZebTARs.A015   90 NTSC   0.6268     (6/9)    +
contig066691-ZebTARs.A015  237 NQTN   0.4542     (5/9)    -
contig066691-ZebTARs.A015  240 NKSE   0.4186     (5/9)    -
----------------------------------------------------------------------
```

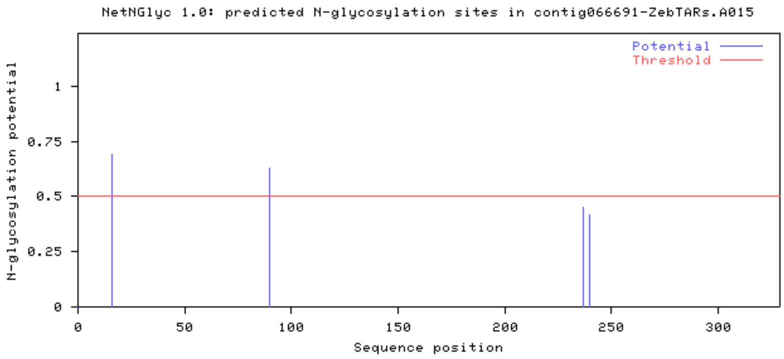

Graphics in PostScript

Output for 'contig066285-ZebTARs.A016'

```
##########################################################################################

    Warning: This sequence may not contain a signal peptide!!

    Proteins without signal peptides are unlikely to be exposed to
    the N-glycosylation machinery and thus may not be glycosylated
    (in vivo) even though they contain potential motifs.

    SignalP-NN euk predictions are as follows:

  # name                  Cmax  pos ?  Ymax  pos ?  Smax  pos ?  Smean ?  D     ?

    SignalP output is explained at http://www.cbs.dtu.dk/services/SignalP/output.html

##########################################################################################

Name:  contig066285-ZebTARs.A016    Length:  326
MEKGAELCFPHLFNNSCIKPTLHWSKAVLLNIVLSCISLITAGLNLLVIISVSHFRQLHTPSNILLLSLAVSDFLVGLLL      80
IPAEIFRITVCWVFGESMCSLYTYLGYIVVTSSISNIVLISVDRYVAICDPLHYPSRISVAKISLSVCMCWFYSAFYSTL     160
CTKNILIEPGRYNSCYGECVFITSDIAGIIDLVLSFIVPVSIIIVLYMRVFVVAVSQARAMHSHVTATLQRSLNQTNKSE     240
LKAARTLGVLVVVFLASFCPFYCYFLVVEDKVSDSSASIVVIVYYFNSCLNPLIYALFYPWFRNAVKVIITFQIFKHDSS     320
EVNVIX
.............N..................................................................      80
................................................................................     160
................................................................................     240
................................................................................     320
......                                                                               400

(Threshold=0.5)
----------------------------------------------------------------------
SeqName      Position  Potential   Jury    N-Glyc
                                 agreement result
----------------------------------------------------------------------
contig066285-ZebTARs.A016   14 NNSC   0.6030     (6/9)    +
contig066285-ZebTARs.A016  234 NQTN   0.4642     (4/9)    -
contig066285-ZebTARs.A016  237 NKSE   0.4248     (5/9)    -
----------------------------------------------------------------------
```

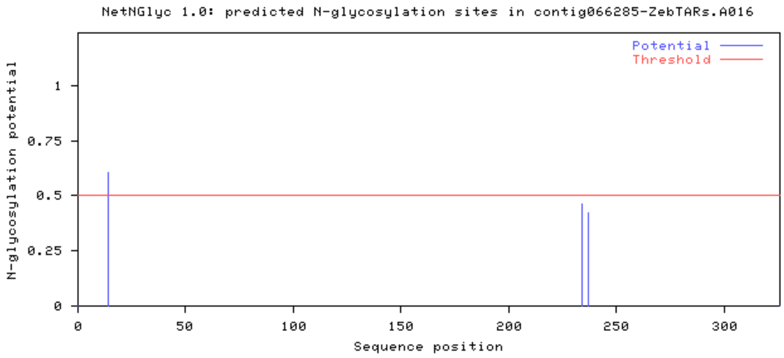

Graphics in PostScript

Output for 'contig066056-ZebTARs.A017'

```
##########################################################################################

    Warning: This sequence may not contain a signal peptide!!

    Proteins without signal peptides are unlikely to be exposed to
    the N-glycosylation machinery and thus may not be glycosylated
    (in vivo) even though they contain potential motifs.

    SignalP-NN euk predictions are as follows:

  # name                Cmax  pos ?  Ymax  pos ?  Smax  pos ?  Smean ?  D     ?

    SignalP output is explained at http://www.cbs.dtu.dk/services/SignalP/output.html

##########################################################################################

Name:  contig066056-ZebTARs.A017    Length:  327
MEETELCFPQLLNSSCVRQKRPQIEAVCIYTLLSSISLLTAVLNLLVIISIAHFKQLHTPTNLLLLSLAVSDFFVGLIMA      80
FEISLLDGCWFLGDHMCALYSSLDYIVTSASVGTMVLISADRYVAICDPLHYPTKITIKRVSVSICTCWACSILYNSLIM     160
KDNFKQPGRYNSCSGDCVVVIDYFVGIFDFVLTFVGPVIVIIVLYLRVFVVAVSQARAMRSHITALRLQGSETVHAKKSE     240
LKAARTLGVLVIAFLICLFPFFCSSMVGQNSFFDIRSVPFELLLFYFNSCLNPLIYTFCYPWFLKSIKLIVTFKIFRHGS     320
SEASILX
............N...................................................................      80
................................................................................     160
................................................................................     240
................................................................................     320
.......                                                                              400

(Threshold=0.5)
----------------------------------------------------------------------
SeqName      Position  Potential   Jury    N-Glyc
                                 agreement result
----------------------------------------------------------------------
contig066056-ZebTARs.A017   13 NSSC   0.7214     (9/9)    ++
----------------------------------------------------------------------
```

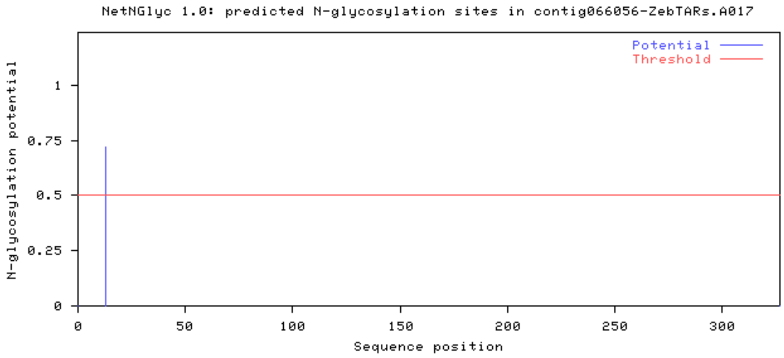

Graphics in PostScript

Output for 'contig062677-ZebTARs.A018'

```
##########################################################################################

    Warning: This sequence may not contain a signal peptide!!

    Proteins without signal peptides are unlikely to be exposed to
    the N-glycosylation machinery and thus may not be glycosylated
    (in vivo) even though they contain potential motifs.
```

```
    SignalP-NN euk predictions are as follows:

  # name                 Cmax  pos ?  Ymax  pos ?  Smax  pos ?  Smean ?  D     ?

    SignalP output is explained at http://www.cbs.dtu.dk/services/SignalP/output.html

########################################################################################

Name:  contig062677-ZebTARs.A018    Length:  329
MGTQEKSELCFPQLFNMSCKKPAIARSKAVFLHIVLSSVSFLTVALNLLVIISVSHFRQLHTPTNILLLSLAVSDFLVGL      80
LLSPAEILRSTACWFLGQPTCLMYIFVSLTVTSASVGIMVLISADRYVAICDPLNYPIRITDRRVQLCVCLCWLCSITFS     160
CFLVRDDLHQRRKQNSCYGKCVVVVQYIAGVVDLILTFIVPVTVIIVLYMRVFVVAVSQARSMRSHATAVRNHLPMTLTK     240
KSELKAARTLGVLVLVFLMCFCPYYCVSLVGEEFINSSSASFVAYLFGLNSCLNPLIYAMFYPWFRKAVKLVVTLQILQP     320
GSSEVSILX
...............N................................................................      80
................................................................................     160
................................................................................     240
................................................................................     320
.........                                                                            400

(Threshold=0.5)
----------------------------------------------------------------------
SeqName      Position  Potential   Jury    N-Glyc
                                 agreement result
----------------------------------------------------------------------
contig062677-ZebTARs.A018   16 NMSC   0.5924     (8/9)   +
contig062677-ZebTARs.A018  276 NSSS   0.4743     (5/9)   -
----------------------------------------------------------------------
```

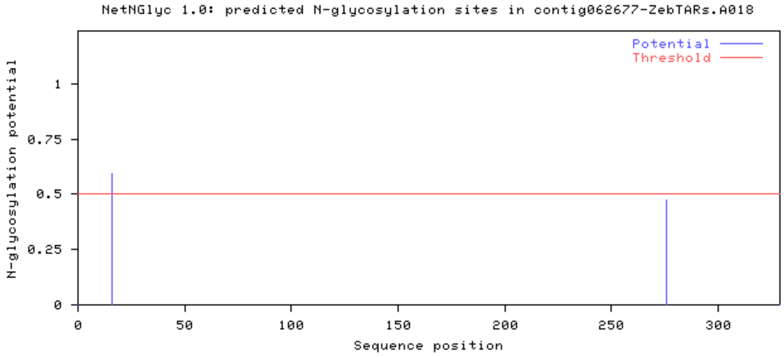

Graphics in PostScript

Output for 'contig062676-ZebTARs.A019'

```
########################################################################################

    Warning: This sequence may not contain a signal peptide!!

    Proteins without signal peptides are unlikely to be exposed to
    the N-glycosylation machinery and thus may not be glycosylated
    (in vivo) even though they contain potential motifs.

    SignalP-NN euk predictions are as follows:

  # name                 Cmax  pos ?  Ymax  pos ?  Smax  pos ?  Smean ?  D     ?

    SignalP output is explained at http://www.cbs.dtu.dk/services/SignalP/output.html

########################################################################################

Name:  contig062676-ZebTARs.A019    Length:  330
METQDEAELCFPQLFNISCKKPKTSLSQVLLPYVVVFSVSLLTVALNLLVIVSVSHFRQLHTPTNILLLSLAVSDFLIGL      80
LLMPAKILRDTACWFLGQLTCSLYSYICFVVTSASVGIMVLISVDRYVAICDPLHYPTRITDRRVKLCVCLCWLCSVFYN     160
ILFIKDDLLQRERHTSCYGECVFVIDYIVGTTDIVLTFIAPVTVIVFLYMRVFVVAVSQARAMRSHVTAVTLQLSVTLTA     240
KKSELKAARTLGVLVLVFLLYFCPYYIVSFFGYELLNSSSASIVIYLYYFNSCLNPLIYAMFYPWFRKAVKLVVTLQILQ     320
PGSCEVSILX
...............N................................................................      80
................................................................................     160
................................................................................     240
....................................N...........................................     320
..........                                                                           400

(Threshold=0.5)
----------------------------------------------------------------------
SeqName      Position  Potential   Jury    N-Glyc
                                 agreement result
----------------------------------------------------------------------
contig062676-ZebTARs.A019   16 NISC   0.6532     (8/9)   +
contig062676-ZebTARs.A019  277 NSSS   0.6216     (8/9)   +
----------------------------------------------------------------------
```

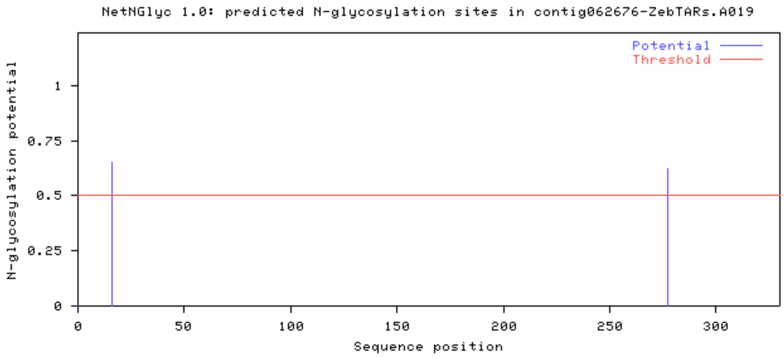

Graphics in PostScript

Output for 'contig061417-ZebTARs.A020'

```
########################################################################################

    Warning: This sequence may not contain a signal peptide!!

    Proteins without signal peptides are unlikely to be exposed to
    the N-glycosylation machinery and thus may not be glycosylated
    (in vivo) even though they contain potential motifs.

    SignalP-NN euk predictions are as follows:

  # name                Cmax  pos ?  Ymax  pos ?  Smax  pos ?  Smean ?  D     ?

    SignalP output is explained at http://www.cbs.dtu.dk/services/SignalP/output.html

########################################################################################

Name:  contig061417-ZebTARs.A020    Length:  329
MEIQKGAELCFPQLLNSSCRKPTLRWSKAVLLNIVLSCISLLTAALNLLVIISVSYFRKLHTPSNILLLSLAVSDFLMGL      80
LLMPAEILRSMTCWVLGDLMCSVYFFLTVNITCASIGNIVLISIDRYVAICDPLHYSTRITVARVKLSVCLCWFYSTFYC     160
SLYTQEMLIEPGRYNSCYGECVLVISDFAGMVDLILFFILPLSVIIVLYTRVFVVAVSQARAMRSHVTAVTLQRSLNQTN     240
KSELKAARTLGVLVVVFLSCYCPFYCYSLTDKNAVNDPATSSVIFIFYFNSCINPLIYALFYPWFRNAVKLIITLQIFKH     320
NSCEANILX
...............N................................................................      80
.............................N..................................................     160
................................................................................     240
................................................................................     320
.........                                                                            400

(Threshold=0.5)
----------------------------------------------------------------------
SeqName      Position  Potential   Jury    N-Glyc
                                 agreement result
----------------------------------------------------------------------
contig061417-ZebTARs.A020   16 NSSC   0.6949     (9/9)   ++
contig061417-ZebTARs.A020  110 NITC   0.7487     (9/9)   ++
contig061417-ZebTARs.A020  237 NQTN   0.4554     (4/9)   -
contig061417-ZebTARs.A020  240 NKSE   0.4186     (5/9)   -
----------------------------------------------------------------------
```

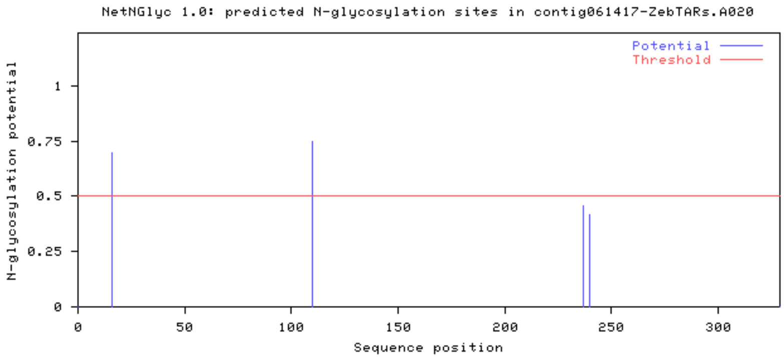

Graphics in PostScript

Output for 'contig061410-ZebTARs.A021'

```
########################################################################################

    Warning: This sequence may not contain a signal peptide!!

    Proteins without signal peptides are unlikely to be exposed to
```

```
    the N-glycosylation machinery and thus may not be glycosylated
    (in vivo) even though they contain potential motifs.

    SignalP-NN euk predictions are as follows:

  # name                 Cmax  pos ?  Ymax  pos ?  Smax  pos ?  Smean ?  D     ?

    SignalP output is explained at http://www.cbs.dtu.dk/services/SignalP/output.html

########################################################################################

Name:  contig061410-ZebTARs.A021    Length:  328
MDTQDVAELCFPQLFNTSCKKPPLSEFVFLHVVLSSISLLTVTLNLLIIISVSHYRQLHTPTNILLLSLAVSDFLVGLLL      80
MPGEILRNTACWFLGDLTCSMYNYMSLIVTSTSVGDMVLISIDRYVAICDPLHYPTRITDRRVKLCVCLCWLCSVFYSSL     160
FVKDDLTQPGKHNSCYGECTIDVDLITGTIDLLLTFFVPVTVIVVLYLRVFVVAVSQARAMRSHVTVAALQLSVTLTTKK     240
SELKAARTLGVLVVVFLLCFCPYYCVTLARDDLLNSSSVSFLLYLFYFNSCLNPLIYALLYPWFRKAVKLIISLHILQPG     320
SCEISILX
...............N................................................................      80
................................................................................     160
................................................................................     240
..................................N.............................................     320
........                                                                             400

(Threshold=0.5)
----------------------------------------------------------------------
SeqName      Position  Potential   Jury    N-Glyc
                                agreement result
----------------------------------------------------------------------
contig061410-ZebTARs.A021   16 NTSC   0.5988     (7/9)    +
contig061410-ZebTARs.A021  275 NSSS   0.5951     (6/9)    +
----------------------------------------------------------------------
```

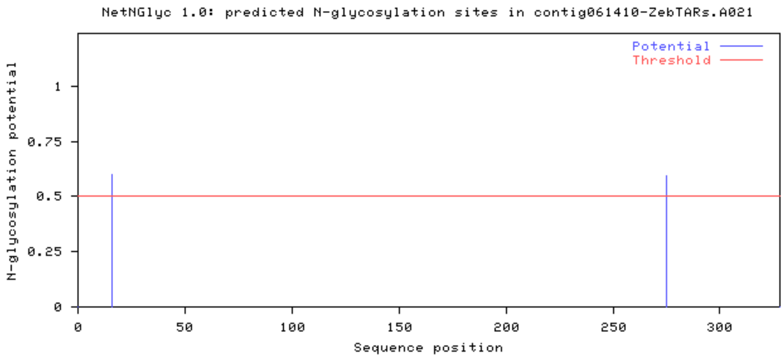

Graphics in PostScript

Output for 'contig059768-ZebTARs.A022'

```
########################################################################################

    Warning: This sequence may not contain a signal peptide!!

    Proteins without signal peptides are unlikely to be exposed to
    the N-glycosylation machinery and thus may not be glycosylated
    (in vivo) even though they contain potential motifs.

    SignalP-NN euk predictions are as follows:

  # name                 Cmax  pos ?  Ymax  pos ?  Smax  pos ?  Smean ?  D     ?

    SignalP output is explained at http://www.cbs.dtu.dk/services/SignalP/output.html

########################################################################################

Name:  contig059768-ZebTARs.A022    Length:  331
MENVYGQQFCFPQLLNTSCRKPLQHHNQTIFLYILLCCISLLTVTLNVLVIISISHFRQLHNQTNLFLLSLAVSDLLVGL      80
LLMPPRILLLGGCWFLGTFMCGLFYYASFVLTSASVGNMVLISFDRYVAICDPLSYPTTVTERKVQISVCLCWACSLLYN     160
GTILNNFLKQPDRYNSCDGECIVVINFITGAFDVVVTFIGPTAVIIFLYMRVFLVAVSQAQAMRSHVAFVTSKGSVHVAI     240
KKSERKAATTIGVVVAVFLMCFCPYFYPSLAGQDTSTSVEFSVFGVWLLYCNSCLNPLIYAFFYPWFRKTVKLIVTLQIL     320
QPDSCDANILX
...............N..........N..................................N..................      80
...............................................................................N     160
................................................................................     240
................................................................................     320
...........                                                                          400

(Threshold=0.5)
----------------------------------------------------------------------
SeqName      Position  Potential   Jury    N-Glyc
                                agreement result
----------------------------------------------------------------------
contig059768-ZebTARs.A022   16 NTSC   0.6682     (9/9)    ++
contig059768-ZebTARs.A022   27 NQTI   0.7472     (9/9)    ++
contig059768-ZebTARs.A022   62 NQTN   0.6788     (8/9)    +
contig059768-ZebTARs.A022  160 NGTI   0.7081     (9/9)    ++
----------------------------------------------------------------------
```

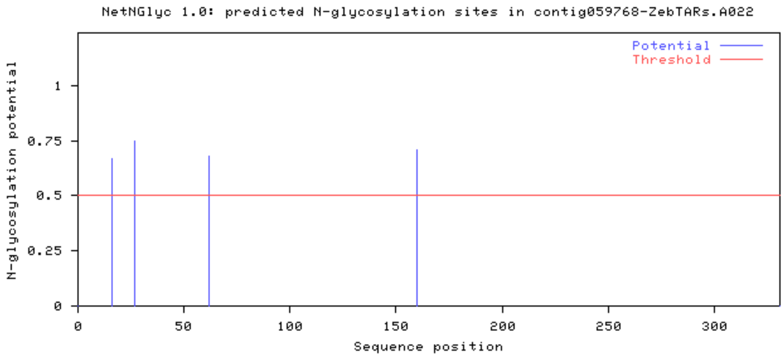

Graphics in PostScript

Output for 'contig053139-ZebTARs.A023'

```
########################################################################################

    Warning: This sequence may not contain a signal peptide!!

    Proteins without signal peptides are unlikely to be exposed to
    the N-glycosylation machinery and thus may not be glycosylated
    (in vivo) even though they contain potential motifs.

    SignalP-NN euk predictions are as follows:

  # name                Cmax   pos ?  Ymax   pos ?  Smax   pos ?  Smean ?  D      ?

    SignalP output is explained at http://www.cbs.dtu.dk/services/SignalP/output.html

########################################################################################

Name:  contig053139-ZebTARs.A023    Length:  326
MEEPELCFPKLLNISCIRPKRPHFEIMLTYILLSFISLLTVILNLLVIISISHFRQLHTPTNFLLLSLAVADFFVGLLMF      80
FQIVLIDGCWFLGDIMCTLYQYLAFIITSASVGTMVIISADRYLAICYPLHYSTQITQQRVNICISLCWFFSVIFQSLIV     160
KDNLKQPGKYNSCIGECVFVVNYIAGLFDLLFSFIVPITVIVVLYLRVFVVAVSQARAMRCQLAVTHQRSVTVTVTKSEL     240
KAARTLGVVVVVFLICMCPYYCVALTGQDNFLNASSAAFVICLVYFNSCLNPIIYVFFYPWFRKSIKLIATLQILQPDSR     320
ETNMHX
............N...................................................................      80
................................................................................     160
................................................................................     240
................................................................................     320
......                                                                               400

(Threshold=0.5)
----------------------------------------------------------------------
SeqName      Position  Potential   Jury    N-Glyc
                                agreement result
----------------------------------------------------------------------
contig053139-ZebTARs.A023   13 NISC   0.8040     (9/9)   +++
contig053139-ZebTARs.A023  273 NASS   0.4887     (6/9)   -
----------------------------------------------------------------------
```

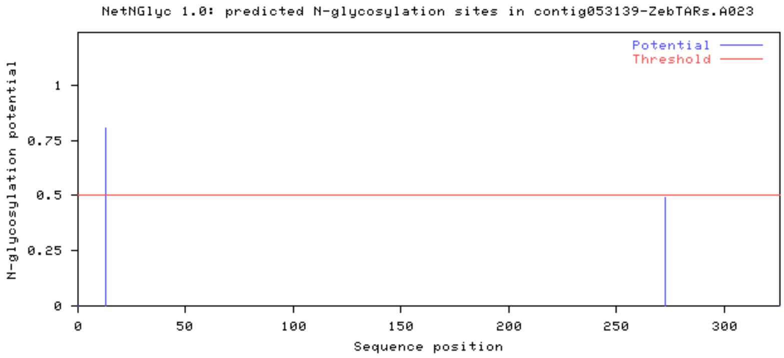

Graphics in PostScript

Output for 'contig030471-ZebTARs.A024'

```
########################################################################################

    Warning: This sequence may not contain a signal peptide!!

    Proteins without signal peptides are unlikely to be exposed to
    the N-glycosylation machinery and thus may not be glycosylated
    (in vivo) even though they contain potential motifs.
```

```
    SignalP-NN euk predictions are as follows:

  # name                 Cmax   pos ?  Ymax   pos ?  Smax   pos ?  Smean ?  D      ?

    SignalP output is explained at http://www.cbs.dtu.dk/services/SignalP/output.html

##########################################################################################

Name:  contig030471-ZebTARs.A024    Length:  329
MEIQKAAELCFPQLLNSSCRKPTLHWSKAVLLNIVLSCISLITAALNLLVIISVSHFRKLHTPSNILLLSLAVSDFLVGL      80
LLMPLEIFRNTACWVLGDRMCSAYWYLTINIICASIGNIVLISVDRYVAICDPLHYPSRITLAKVKLSVCLCWFYAFFYS     160
NLYTKNIMIEPGRYNSCFGECVFFSSNIAIVADLILFFFVPVTVIIALYMRVFVVAVSQARAMRSHVTSVTLQCSLNQAN     240
KSELKAARTLGVLVVVFLACYCPFYCYALVDKNVVNDSSASFVVLVFYFNSCLNPLIYALFYPWFRKAIRCVITLLIFKH     320
DSSEVNIIX
...............N................................................................      80
................................................................................     160
................................................................................     240
................................................................................     320
.........                                                                            400

(Threshold=0.5)
----------------------------------------------------------------------
SeqName      Position  Potential   Jury    N-Glyc
                      agreement result
----------------------------------------------------------------------
contig030471-ZebTARs.A024   16 NSSC   0.7032     (9/9)   ++
contig030471-ZebTARs.A024  240 NKSE   0.3682     (8/9)   -
contig030471-ZebTARs.A024  276 NDSS   0.4010     (7/9)   -
----------------------------------------------------------------------
```

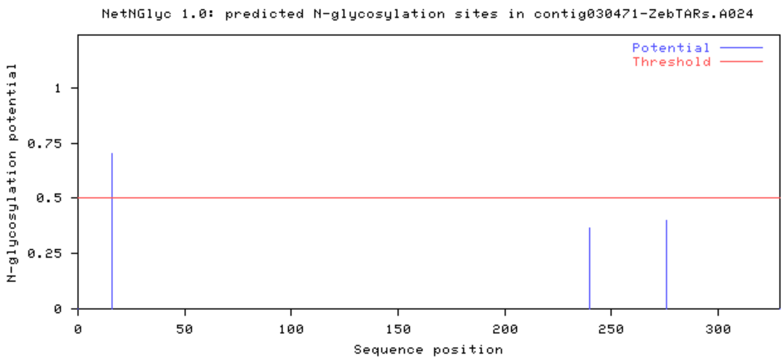

Graphics in PostScript

Output for 'contig030464-ZebTARs.A025'

```
##########################################################################################

    Warning: This sequence may not contain a signal peptide!!

    Proteins without signal peptides are unlikely to be exposed to
    the N-glycosylation machinery and thus may not be glycosylated
    (in vivo) even though they contain potential motifs.

    SignalP-NN euk predictions are as follows:

  # name                 Cmax   pos ?  Ymax   pos ?  Smax   pos ?  Smean ?  D      ?

    SignalP output is explained at http://www.cbs.dtu.dk/services/SignalP/output.html

##########################################################################################

Name:  contig030464-ZebTARs.A025    Length:  327
MQKEAELCFPQLLNSSCRKPTFHWSKAVLLNTVLCCISLLTAALNLLVIISVSYFRQLHTPTNILLLSLAVSDFLTGLLM      80
LPLEIIRNTACWVLGDLMCSVYTYLTAGLFCASTWHIVLISVDRYVAICHPLHYPTRITVARVKLCVCLCWFCSTFYGSL     160
LTKDVLIKPARYNSCYGECVFVINDIAGFIDLVLSFIFPVSVIIVLYMRVFVVAVSQARSMRSHVTAVTLQRSSNQANKS     240
ELKAARNLGVLVVVFLACYLPFYCYSLIEVNVINDSSTFFLIIVFYFNSCLNPLMYALFYSWFRNAVKLIITLQIFKHDT     320
SEANMFX
.............N..................................................................      80
................................................................................     160
................................................................................     240
................................................................................     320
.......                                                                              400

(Threshold=0.5)
----------------------------------------------------------------------
SeqName      Position  Potential   Jury    N-Glyc
                      agreement result
----------------------------------------------------------------------
contig030464-ZebTARs.A025   14 NSSC   0.6854     (9/9)   ++
contig030464-ZebTARs.A025  238 NKSE   0.3509     (8/9)   -
contig030464-ZebTARs.A025  274 NDSS   0.4003     (7/9)   -
----------------------------------------------------------------------
```

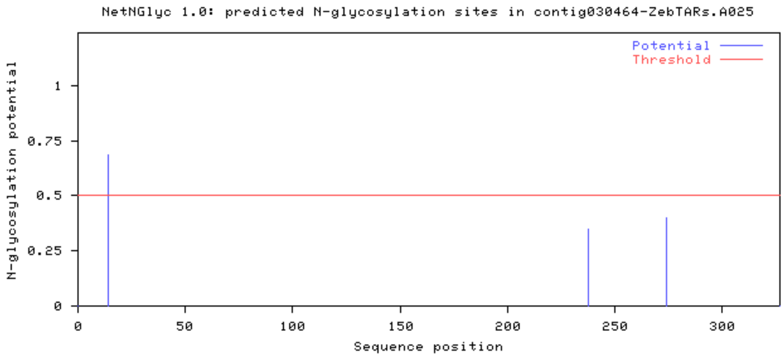

Graphics in PostScript

Output for 'contig030445-ZebTARs.A026'

```
##########################################################################

    Warning: This sequence may not contain a signal peptide!!

    Proteins without signal peptides are unlikely to be exposed to
    the N-glycosylation machinery and thus may not be glycosylated
    (in vivo) even though they contain potential motifs.

    SignalP-NN euk predictions are as follows:

  # name                Cmax   pos ?  Ymax   pos ?  Smax   pos ?  Smean ?  D     ?

    SignalP output is explained at http://www.cbs.dtu.dk/services/SignalP/output.html

##########################################################################

Name:  contig030445-ZebTARs.A026    Length:  330
MDTQDGAELCFPQLFNISCKKPTTPLSQVLLLYIVVSSMSLLTVTLNLLVIIAVSHFRQLHTPTNILLLSLAVTDFLVGL      80
LFMPGEILRNTACWFLGQLTCSLYNYVSYIITSASVGNMVLISVDRYVAICDPLHYPTRITERRVKLCVCLCWLCSVFYC     160
YVILIDDLSQPGKHNSCYGRCIIIIEFIAGFVDLFLSFIIPLTVIIVLYMRVFVVAVSQARAMRSQVTAVTLQLSVTLTA     240
KKSELKAARTLGVLVLVFLLCFCSYYFISLFGNDLLNSSSASIVIYLYYFNSCLNPLIYAMFYPWFRKAVKLIVTLQILQ     320
PGSCEVSVLX
...............N................................................................      80
................................................................................     160
................................................................................     240
....................................N...........................................     320
..........                                                                           400

(Threshold=0.5)
----------------------------------------------------------------------
SeqName      Position  Potential   Jury    N-Glyc
                     agreement result
----------------------------------------------------------------------
contig030445-ZebTARs.A026   16 NISC   0.6416     (8/9)   +
contig030445-ZebTARs.A026  277 NSSS   0.5511     (6/9)   +
----------------------------------------------------------------------
```

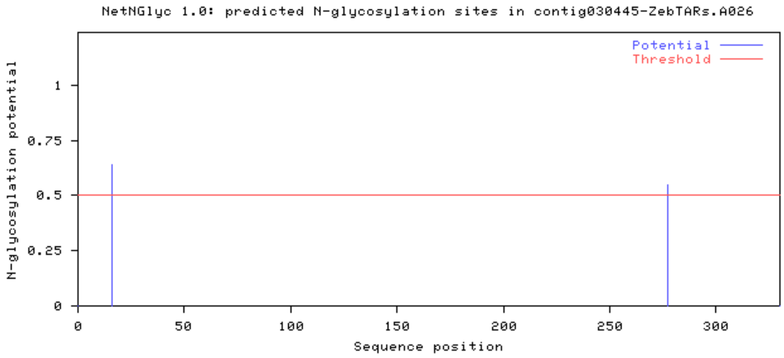

Graphics in PostScript

Output for 'contig066330-ZebTARs.A027'

```
##########################################################################

    Warning: This sequence may not contain a signal peptide!!

    Proteins without signal peptides are unlikely to be exposed to
    the N-glycosylation machinery and thus may not be glycosylated
    (in vivo) even though they contain potential motifs.
```

```
    SignalP-NN euk predictions are as follows:

  # name                  Cmax  pos ?  Ymax  pos ?  Smax  pos ?  Smean ?  D     ?

    SignalP output is explained at http://www.cbs.dtu.dk/services/SignalP/output.html

##########################################################################################

Name:  contig066330-ZebTARs.A027    Length:  326
MEEAELCFQQLFNTSCMRPRRPHFEIMLTYILLSFISLLTVILNLLVIISVSHFRQLHTPTNLLLLSLAVADFYVGLLLF      80
FQIVLIDGCWFLGDIMCTLYQYLAYVITSASIGTMVIISVDRYLAICYPLHYSTKITQQRVKIVVCLCWICSVIFQSLIL     160
MDNLEQPGRYNSCIGECVFVINYIAGLVDVTFSFIVPFTVIVVLYLRVFVVAVSQARAMRSQLAVTHQRSVTVTAKKSEL     240
KAAWTLGIVVVVFLICMCPYYCVALTGQDSLPSASSLTFVLCLAYFNSCLNPIIYVFFYPWFRKSIKVIVTLQILQPDSS     320
KVTMRX
............N...................................................................      80
................................................................................     160
................................................................................     240
................................................................................     320
......                                                                               400

(Threshold=0.5)
----------------------------------------------------------------------
SeqName      Position  Potential   Jury    N-Glyc
                      agreement result
----------------------------------------------------------------------
contig066330-ZebTARs.A027   13 NTSC   0.6403     (9/9)    ++
----------------------------------------------------------------------
```

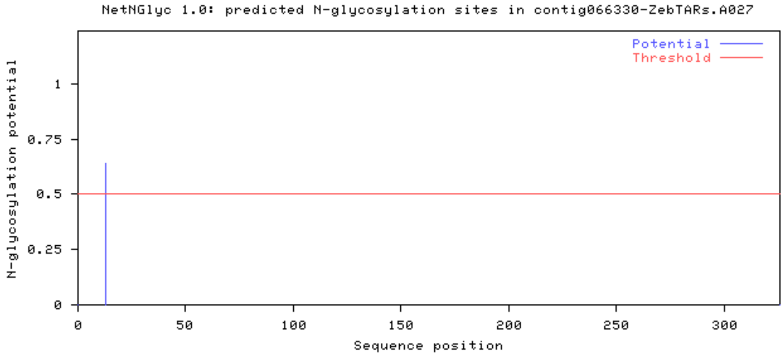

Graphics in PostScript

Output for 'contig040586-ZebTAR.A001'

```
##########################################################################################

    Warning: This sequence may not contain a signal peptide!!

    Proteins without signal peptides are unlikely to be exposed to
    the N-glycosylation machinery and thus may not be glycosylated
    (in vivo) even though they contain potential motifs.

    SignalP-NN euk predictions are as follows:

  # name                  Cmax  pos ?  Ymax  pos ?  Smax  pos ?  Smean ?  D     ?

    SignalP output is explained at http://www.cbs.dtu.dk/services/SignalP/output.html

##########################################################################################

Name:  contig040586-ZebTAR.A001     Length:  326
MDSSGGPPLCFPNLNSSCRRLLRPTSQTVVLYTLLASISLLTVVLNLLVVISISHFRQLNTSTNTLLLSLAVSDLLVGLL      80
VMPIEGLDYIETCWLLGRLMCALSPYLSYCLISVSLDSMVLISVDRYIAICDPLLYSSKITVNRVKLSVCVCWACSLLYN     160
GCILMEHIGWPDRFSSCHGECVVFISRALGTIDLFFSFLGPCALMFVLYMRVFVVAVSQVRIIRSQAAVRAAPAAKKSEL     240
KAARTLGILIAVFLMCFCPYYYPSFAGDDTSMSLPYYALFSWIMLTNSCVNPVIYALFYPWFRRAIRLIVTLRILQPHSR     320
DVKILX
..............N............................................N....................      80
................................................................................     160
................................................................................     240
................................................................................     320
......                                                                               400

(Threshold=0.5)
----------------------------------------------------------------------
SeqName      Position  Potential   Jury    N-Glyc
                      agreement result
----------------------------------------------------------------------
contig040586-ZebTAR.A001    15 NSSC   0.6957     (9/9)    ++
contig040586-ZebTAR.A001    60 NTST   0.6152     (8/9)    +
----------------------------------------------------------------------
```

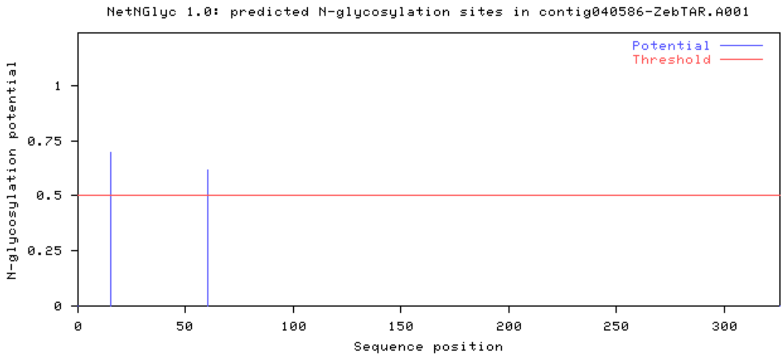

Graphics in PostScript

Output for 'contig040586-ZebTAR.A002'

```
########################################################################################

    Warning: This sequence may not contain a signal peptide!!

    Proteins without signal peptides are unlikely to be exposed to
    the N-glycosylation machinery and thus may not be glycosylated
    (in vivo) even though they contain potential motifs.

    SignalP-NN euk predictions are as follows:

  # name                Cmax  pos ?  Ymax  pos ?  Smax  pos ?  Smean ?  D     ?

    SignalP output is explained at http://www.cbs.dtu.dk/services/SignalP/output.html

########################################################################################

Name:  contig040586-ZebTAR.A002     Length:  326
MDGTGGPPLCFPNLNSSCRRLLRPTSQAALLYTLLASVSLLTVVLNLLVVVSISHFKQLHTPNALLLSLAMSDLLVGLL      80
VMPIEGLRYIETCWLLGRLMCALSPYLSYCLISVSLGSMVLISVDRYIAICDPLLYSSKITVNRVKLSVCVCWACSLLYN     160
GCILMEHIGWPDRFSSCHGECVVFISQTSGTVDFFLSFLGPCGVMFVLYMRVFVVAVSQVRAIQSQAAVRAAPAAKKSEL     240
KAARTLGILIAVFVMCFCPYYYPSLAGVDTFLSLPSYAMVCWIMLINACVNPVIYVLFYPWFRRAIRFIVSLRILQPHSR     320
DVKILX
..............N.................................................................      80
................................................................................     160
................................................................................     240
................................................................................     320
......                                                                               400

(Threshold=0.5)
----------------------------------------------------------------------
SeqName      Position  Potential   Jury    N-Glyc
                     agreement result
----------------------------------------------------------------------
contig040586-ZebTAR.A002   15 NSSC   0.6959     (9/9)    ++
----------------------------------------------------------------------
```

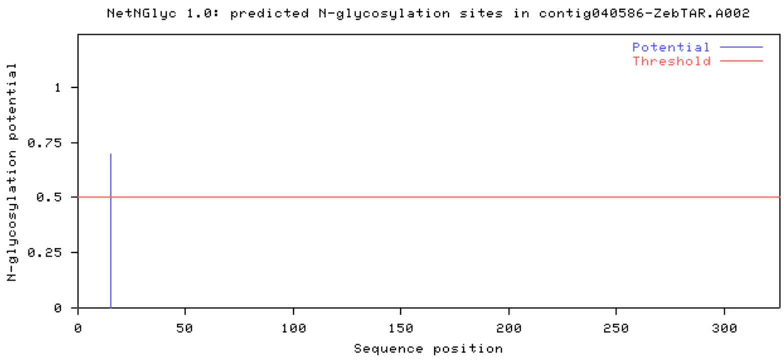

Graphics in PostScript

Output for 'contig003909-ZebTAR.A003'

```
########################################################################################

    Warning: This sequence may not contain a signal peptide!!

    Proteins without signal peptides are unlikely to be exposed to
    the N-glycosylation machinery and thus may not be glycosylated
    (in vivo) even though they contain potential motifs.
```

```
    SignalP-NN euk predictions are as follows:

  # name                 Cmax  pos ?  Ymax  pos ?  Smax  pos ?  Smean ?  D     ?

    SignalP output is explained at http://www.cbs.dtu.dk/services/SignalP/output.html

########################################################################################

Name:  contig003909-ZebTAR.A003     Length:  331
MDRQDRIELCYPELANLSCRGLTRPQPEAVLLYTLFLSISVLTVALNVLVIISISHFRQLHMPTNVLLLSLAISDLLVGL      80
LVMPVEAMRFIETCWLLGDLMCAFTYIIGFTLTSASVGNMVLISIDRYVAICYPLQYPTKITHSRVELSVTLCWACSLLY     160
NGMILKEHLRQPNRHNTCHGQCLVVINYVSGAIDLVFTFIGPCSVIIILYMRVFVVAVSQAHAMRSHITAAAAGTVKITA     240
KKSEKKAARTLGVVIFVFLMSFCPYYYPSLAGQDISNSASSWAIVSWMLYFNSCLNPLIYAFFYPWFRKAIWFIVSLKIL     320
EKGSSQANILX
...............N................................................................      80
................................................................................     160
................................................................................     240
................................................................................     320
...........                                                                          400

(Threshold=0.5)
----------------------------------------------------------------------
SeqName      Position  Potential   Jury    N-Glyc
                      agreement result
----------------------------------------------------------------------
contig003909-ZebTAR.A003   16 NLSC   0.6719     (8/9)    +
----------------------------------------------------------------------
```

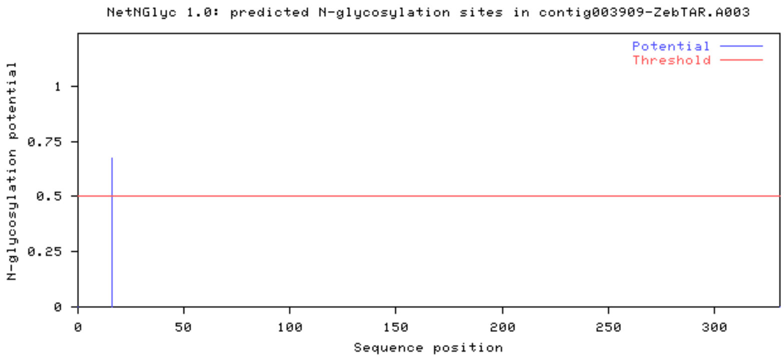

Graphics in PostScript

Output for 'contig053145-ZebTARs.A028'

```
########################################################################################

    Warning: This sequence may not contain a signal peptide!!

    Proteins without signal peptides are unlikely to be exposed to
    the N-glycosylation machinery and thus may not be glycosylated
    (in vivo) even though they contain potential motifs.

    SignalP-NN euk predictions are as follows:

  # name                 Cmax  pos ?  Ymax  pos ?  Smax  pos ?  Smean ?  D     ?

    SignalP output is explained at http://www.cbs.dtu.dk/services/SignalP/output.html

########################################################################################

Name:  contig053145-ZebTARs.A028    Length:  326
MEEAELCFQQLFNTSCMRPRRPHFEIMLTYILLSFISLLTVILNLLVIISVSHFRQLHTPTNLLLLSLAVADFYVGLLLF      80
FQIVLIDGCWFLGDIMCTLYQYLAYVITSASIGTMVIISVDRYLAICYPLHYSTKITQQRVKIVVCLCWICSVIFQSLIL     160
MDNLEQPGRYNSCIGECVFVINYIAGLVDVTFSFIVPFTVIVVLYLRVFVVAVSQARAMRSQLAVTHQRSVTVTAKKSEL     240
KAAWTLGIVVVVFLICMCPYYCVALTGQDNLPSASSLTFVLCLVYFNSCLNPIIYVFFYPWFRKSIKVIVTLQILQPDSC     320
QATVLX
............N...................................................................      80
................................................................................     160
................................................................................     240
................................................................................     320
......                                                                               400

(Threshold=0.5)
----------------------------------------------------------------------
SeqName      Position  Potential   Jury    N-Glyc
                      agreement result
----------------------------------------------------------------------
contig053145-ZebTARs.A028   13 NTSC   0.6403     (9/9)    ++
----------------------------------------------------------------------
```

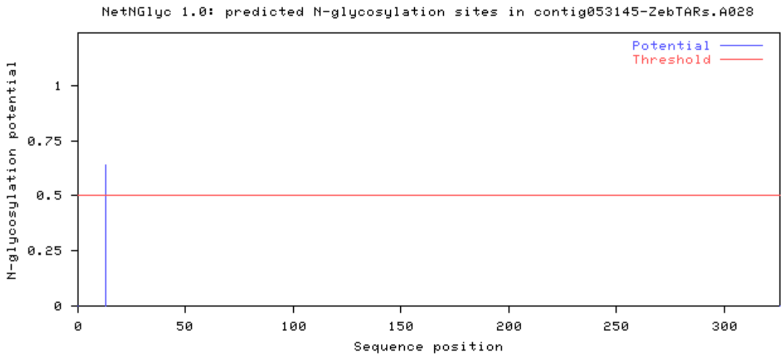

Graphics in PostScript

Output for 'contig030440-ZebTARs.A029'

```
##########################################################################################

    Warning: This sequence may not contain a signal peptide!!

    Proteins without signal peptides are unlikely to be exposed to
    the N-glycosylation machinery and thus may not be glycosylated
    (in vivo) even though they contain potential motifs.

    SignalP-NN euk predictions are as follows:

  # name                Cmax  pos ?  Ymax  pos ?  Smax  pos ?  Smean ?  D     ?

    SignalP output is explained at http://www.cbs.dtu.dk/services/SignalP/output.html

##########################################################################################

Name:  contig030440-ZebTARs.A029    Length:  330
MDTQSRAELCFPQLFNTSCKKPTTPLSQVLLFHILMPSMSLLTVTLNLLVIIAVSHFRQLHTPTNILLLSLAAADFLIGL      80
LFMPGEILRNTVCWFLGQLTCSLYNYISFVVTSASVGDMVLISVDRYVAICDPLHYPTRITERRVKLCVCLCWLCSVFYC     160
YMIVRDDLSQPGKHNSCFGECIIFVEFIAGFVDLVLSFIIPVTVIVFLYMRVFVVAVSQARAMRSQVTAVTLQLSVTLTA     240
TKSELKAARTLGVLVLVFLICFCPYYIVSLFGDEFLNSASASIVFYFFLLNSCLNPLIYAMFYPWFRKAVKLIMTLQILQ     320
PGSCEVSILX
...............N................................................................      80
................................................................................     160
................................................................................     240
................................................................................     320
..........                                                                           400

(Threshold=0.5)
----------------------------------------------------------------------
SeqName      Position  Potential   Jury    N-Glyc
                                  agreement result
----------------------------------------------------------------------
contig030440-ZebTARs.A029   16 NTSC   0.5784     (7/9)   +
----------------------------------------------------------------------
```

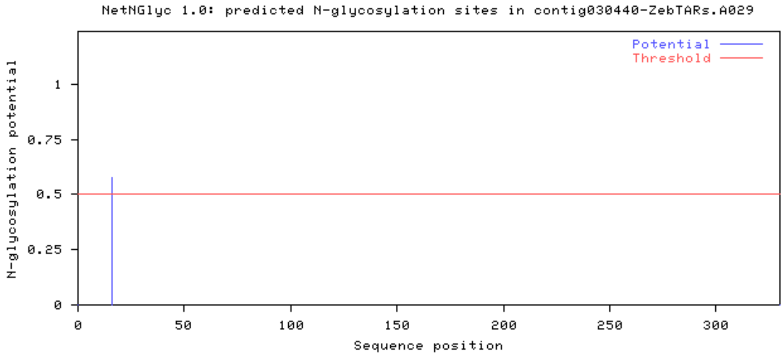

Graphics in PostScript

Output for 'contig033536-ZebTAR.B029'

```
##########################################################################################

    Warning: This sequence may not contain a signal peptide!!

    Proteins without signal peptides are unlikely to be exposed to
    the N-glycosylation machinery and thus may not be glycosylated
    (in vivo) even though they contain potential motifs.
```

```
    SignalP-NN euk predictions are as follows:

  # name                Cmax  pos ?  Ymax  pos ?  Smax  pos ?  Smean ?  D     ?

    SignalP output is explained at http://www.cbs.dtu.dk/services/SignalP/output.html

########################################################################################

Name:  contig033536-ZebTAR.B029     Length:  315
MELFNVTVNTVSFLLCDSQKNKLCVLLYVVLSSFMLLTICGNLLVIISIIYFRYLHTPTNYLILSMAVADLLIGALIFPL      80
SMTVSLKPCLYIYSLLCNLRSTMDVTMGVSSLLNLCCISVDRYYAVCHPLIYKTKITDCVAMKMGLGSWAVAILCGIFVF     160
LLFFILDECDTSCVFALIAASVVVYYIPTIVLLFMYTKILVVALRQARSIHNTISQNTKSKAVSSTERKATKTLTIVIGI     240
FLICWVPLFLSYSFVPLDSFILYVLLEPFNWFAISNSMLNPFIYAFFYTWFRRAFKMIISGKIFQGDVTNIKLHX
....N...........................................................................      80
................................................................................     160
................................................................................     240
...........................................................................          320

(Threshold=0.5)
----------------------------------------------------------------------
SeqName      Position  Potential   Jury    N-Glyc
                      agreement result
----------------------------------------------------------------------
contig033536-ZebTAR.B029     5 NVTV   0.7989     (9/9)    +++
----------------------------------------------------------------------
```

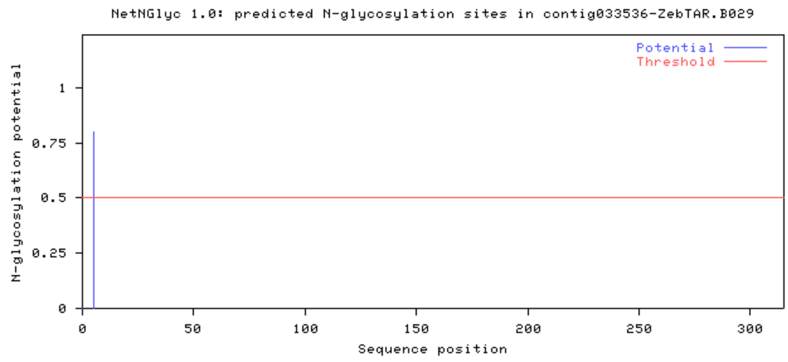

Graphics in PostScript

Output for 'contig038663-NyeTAR.A005'

```
########################################################################################

    Warning: This sequence may not contain a signal peptide!!

    Proteins without signal peptides are unlikely to be exposed to
    the N-glycosylation machinery and thus may not be glycosylated
    (in vivo) even though they contain potential motifs.

    SignalP-NN euk predictions are as follows:

  # name                Cmax  pos ?  Ymax  pos ?  Smax  pos ?  Smean ?  D     ?

    SignalP output is explained at http://www.cbs.dtu.dk/services/SignalP/output.html

########################################################################################

Name:  contig038663-NyeTAR.A005     Length:  326
MDSSGGPPLCFPNLNSSCRRLLRPTSQTVVLYTLLASISLLTVVLNLLVVISISHFRQLNTSTNTLLLSLAVSDLLVGLL      80
VMPIEGLYYIETCWLLGRLMCALSPYLSYCLISVSLGSMVLISVDRYIAICDPLLYSSKITVNRVKLSVCVCWACSLLYN     160
GCILMEHIGWPDRFGSCHGECVVFISRALGTIDLFFSFLGPCALMFVLYMRVFVVAVSQVRIIRSQAAVRAAPAAKKSEL     240
KAARTLGILIAVFVMCFCPYYYPSFAGDDTSMSLPYYALFSWIMLTNSCVNPVIYALFYPWFKRAIRLIVTLRILQPHSR     320
DVKILX
..............N............................................N....................      80
................................................................................     160
................................................................................     240
................................................................................     320
......                                                                               400

(Threshold=0.5)
----------------------------------------------------------------------
SeqName      Position  Potential   Jury    N-Glyc
                      agreement result
----------------------------------------------------------------------
contig038663-NyeTAR.A005    15 NSSC   0.6959     (9/9)    ++
contig038663-NyeTAR.A005    60 NTST   0.6153     (8/9)    +
----------------------------------------------------------------------
```

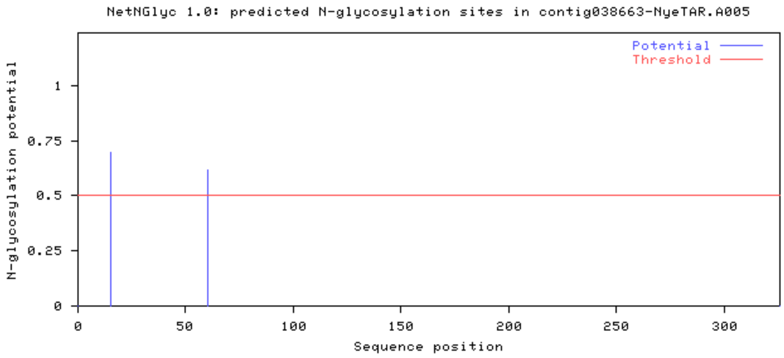

Graphics in PostScript

Output for 'contig035375-NyeTARs.A013'

```
##########################################################################################

    Warning: This sequence may not contain a signal peptide!!

    Proteins without signal peptides are unlikely to be exposed to
    the N-glycosylation machinery and thus may not be glycosylated
    (in vivo) even though they contain potential motifs.

    SignalP-NN euk predictions are as follows:

  # name                Cmax  pos ?  Ymax  pos ?  Smax  pos ?  Smean ?  D     ?

    SignalP output is explained at http://www.cbs.dtu.dk/services/SignalP/output.html

##########################################################################################

Name:  contig035375-NyeTARs.A013    Length:  330
MDTQSRADLCFPQLFNTSCKKPTTPLSQVLLFHILMPSMCLLTVTLNLLVIIAISHFRQLHSHTNILLLSLAAADFLIGL      80
LFMPGEILRNTVCWFLGQLTCSLYNYISFVVTSASVGIMVLISVDRYVAICDPLHYPTRITERRVKLCVCLCWLCSVFYC     160
YMIVRDDLSQPGKHNSCFGECIIFVEFIAGFVDLVLSFIIPVTVIVFLYMRVFVVAVSQARAMRSQVTAVTLQLSVTLTA     240
KKSELKAARTLGVLVLVFLICFCPYYIVSLFGDEFLNSASASIVFYFFLLNSCLNPLIYAMFYPWFRKAVKLIMTLQILQ     320
PGSCEVSILX
...............N................................................................      80
................................................................................     160
................................................................................     240
................................................................................     320
..........                                                                           400

(Threshold=0.5)
----------------------------------------------------------------------
SeqName      Position  Potential   Jury    N-Glyc
                      agreement result
----------------------------------------------------------------------
contig035375-NyeTARs.A013   16 NTSC   0.5803     (7/9)    +
----------------------------------------------------------------------
```

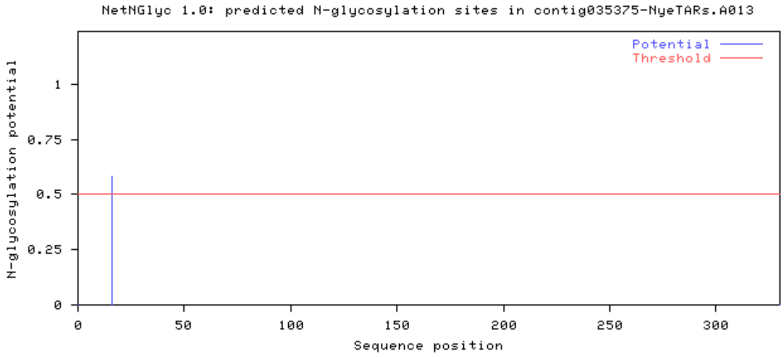

Graphics in PostScript

Output for 'contig035376-NyeTARs.A014'

```
##########################################################################################

    Warning: This sequence may not contain a signal peptide!!

    Proteins without signal peptides are unlikely to be exposed to
    the N-glycosylation machinery and thus may not be glycosylated
    (in vivo) even though they contain potential motifs.
```

```
    SignalP-NN euk predictions are as follows:

  # name                 Cmax  pos ?  Ymax  pos ?  Smax  pos ?  Smean ?  D     ?

    SignalP output is explained at http://www.cbs.dtu.dk/services/SignalP/output.html

########################################################################################

Name:  contig035376-NyeTARs.A014    Length:  330
MDTQDGAELCFPQLFNISCKKPTTPRSQVLLLYIVVSSMSLLTVTLNLLVIITVSHFRQLHTPTNILLLSLAVTDFLVGL      80
LFMPGEILRNTACWFLGQLTCSLYNYVSYIIASASVGNMVLISVDRYVAICDPLHYPTRITERRVKLCVCLCWLCSVFYS     160
YVILIDDLSQPGKHKSCYGRCIIFIEFIAGFVDLVLAFIIPLTVIIVLYMRVFVVAVSQARAMRSQVTAVTLQLSVTLTA     240
TKSELKAARTLGVLVLVFLLCFCSYYIISLFGNELLNSSSASIVIYLYYFNSCLNPLIYAMFYPWFRKAVKLIVTLQILQ     320
PGSCEISIMX
..............N.................................................................      80
................................................................................     160
................................................................................     240
....................................N...........................................     320
..........                                                                           400

(Threshold=0.5)
----------------------------------------------------------------------
SeqName      Position  Potential   Jury    N-Glyc
                                 agreement result
----------------------------------------------------------------------
contig035376-NyeTARs.A014    16 NISC   0.6353     (8/9)    +
contig035376-NyeTARs.A014   277 NSSS   0.5826     (6/9)    +
----------------------------------------------------------------------
```

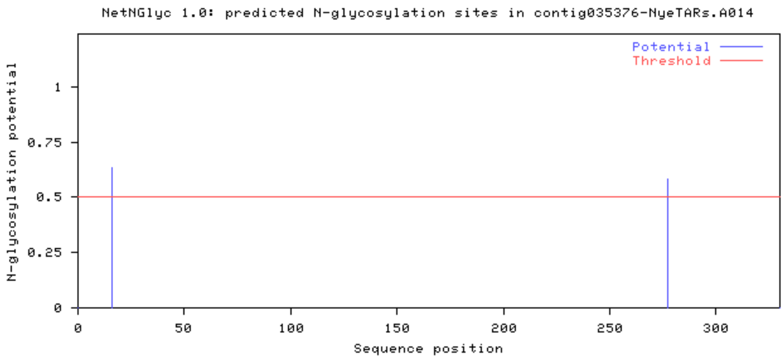

Graphics in PostScript

Output for 'contig035381-NyeTARs.A015'

```
########################################################################################

    Warning: This sequence may not contain a signal peptide!!

    Proteins without signal peptides are unlikely to be exposed to
    the N-glycosylation machinery and thus may not be glycosylated
    (in vivo) even though they contain potential motifs.

    SignalP-NN euk predictions are as follows:

  # name                 Cmax  pos ?  Ymax  pos ?  Smax  pos ?  Smean ?  D     ?

    SignalP output is explained at http://www.cbs.dtu.dk/services/SignalP/output.html

########################################################################################

Name:  contig035381-NyeTARs.A015    Length:  330
MDTQDGAELCFPQLFNISCKKPTTPLSQVLLLYIVVSSMSLLTVTLNLLVIIAVSHFRQLHSPTNILLLSLAVADFLVGL      80
LFMPGEILRNTACWFLGQLTCSLYNYVSYIITSASVGNMVLISVDRYVAICDPLHYPTRITERRVKLCVCLCWLCSVFYC     160
YVILIDDLSQPGKHNSCYGKCIIIIEFIAGFVDLVLSFIIPLTVIIVLYMRVFVVAVSQARAMRSQVTAVTLQLSVTLTA     240
KKSELKAAKTLGVLVLVFLLCFCSYFIVSLFGNELLNSSSASIVIYLYYFNSCLNPLIYAMFYPWFRKAVKLIVTLQILQ     320
PGSCEVIILX
..............N.................................................................      80
................................................................................     160
................................................................................     240
....................................N...........................................     320
..........                                                                           400

(Threshold=0.5)
----------------------------------------------------------------------
SeqName      Position  Potential   Jury    N-Glyc
                                 agreement result
----------------------------------------------------------------------
contig035381-NyeTARs.A015    16 NISC   0.6413     (8/9)    +
contig035381-NyeTARs.A015   277 NSSS   0.5993     (6/9)    +
----------------------------------------------------------------------
```

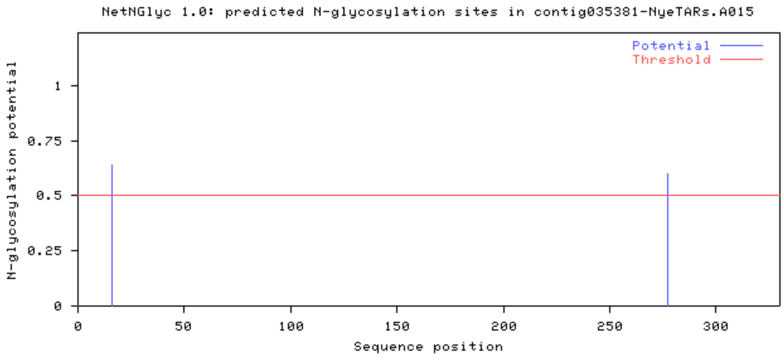

Graphics in PostScript

Output for 'contig046007-NyeTARs.A017'

```
##########################################################################################

    Warning: This sequence may not contain a signal peptide!!

    Proteins without signal peptides are unlikely to be exposed to
    the N-glycosylation machinery and thus may not be glycosylated
    (in vivo) even though they contain potential motifs.

    SignalP-NN euk predictions are as follows:

  # name                Cmax  pos ?  Ymax  pos ?  Smax  pos ?  Smean ?  D     ?

    SignalP output is explained at http://www.cbs.dtu.dk/services/SignalP/output.html

##########################################################################################

Name:  contig046007-NyeTARs.A017    Length:  327
MQKENKLCFPQLLNSSCRKPTLHWSKDVLLNTVLSCISLLTAALNILVIISVSYFRKLHTPSNILLLSLAVSDFLVGLLL      80
MPLGILRNTGCWVLGDIICSLYWYLTSNIVCASIGNIVLISVDRYVAICDPLHYPSRITLAKVKLSVCLCWFYAFFYVSL     160
YTKDILLEPGRDNSCYGECVFVINDIAVVVDLVFSFIVPVSVIVVLYIRVFVAAVSQARAMRSHVTSVTLQRSLNQTNKS     240
ELKAARTLGILVVVFLACFCPLYYFSLVDENAINDPAASFVVIIFYFNSCLNPLIYALFYPWFRNAVKLIITLRIFKYNM     320
SEANILX
.............N..................................................................      80
................................................................................     160
................................................................................     240
................................................................................     320
.......                                                                              400

(Threshold=0.5)
----------------------------------------------------------------------
SeqName      Position  Potential   Jury    N-Glyc
                                 agreement result
----------------------------------------------------------------------
contig046007-NyeTARs.A017   14 NSSC   0.6982     (9/9)   ++
contig046007-NyeTARs.A017  235 NQTN   0.4543     (5/9)   -
contig046007-NyeTARs.A017  238 NKSE   0.4189     (5/9)   -
contig046007-NyeTARs.A017  319 NMSE   0.4722     (4/9)   -
----------------------------------------------------------------------
```

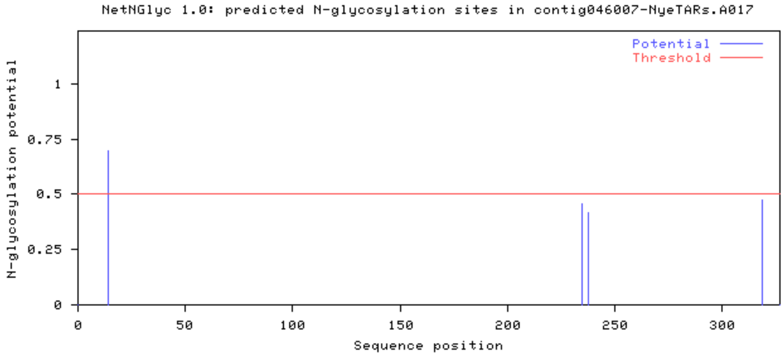

Graphics in PostScript

Output for 'contig060105-NyeTARs.A018'

```
##########################################################################################

    Warning: This sequence may not contain a signal peptide!!

    Proteins without signal peptides are unlikely to be exposed to
```

```
    the N-glycosylation machinery and thus may not be glycosylated
    (in vivo) even though they contain potential motifs.

    SignalP-NN euk predictions are as follows:

  # name                 Cmax   pos ?  Ymax   pos ?  Smax   pos ?  Smean ?  D     ?

    SignalP output is explained at http://www.cbs.dtu.dk/services/SignalP/output.html

##########################################################################################

Name:  contig060105-NyeTARs.A018    Length:  328
MEETELCFPQLFNSSCVRQKHSQIEAVFIYTPLSSISVLTTILNLLVIISIAHFKQLHTPTNLLLLLSLAVSDFFVGLIM      80
ACQISLLDGCWFLGDHMCALYSSLDYIVTSASVGTMVLISADRYVAICDPLHYPTKITIKRVSVSICTCWACSILYNSLI     160
MKDNFKQPGRYNSCSGDCVVVIDYFVGIFDFVLTFVGPVIVIIVLYLRVFVVAVSQARAMRSHITALRLQGSETVHAKKS     240
ELKAARTLGVLVIAFLICLFPFFCSSMVGQNSFFDIRSVPFERLLFYFNSCLNPLIYTFCYPWFLKSIKLIVTFKIFRHG     320
SSEASVLX
............N...................................................................      80
................................................................................     160
................................................................................     240
................................................................................     320
........                                                                             400

(Threshold=0.5)
----------------------------------------------------------------------
SeqName      Position  Potential   Jury    N-Glyc
                      agreement result
----------------------------------------------------------------------
contig060105-NyeTARs.A018    13 NSSC    0.6738     (9/9)    ++
----------------------------------------------------------------------
```

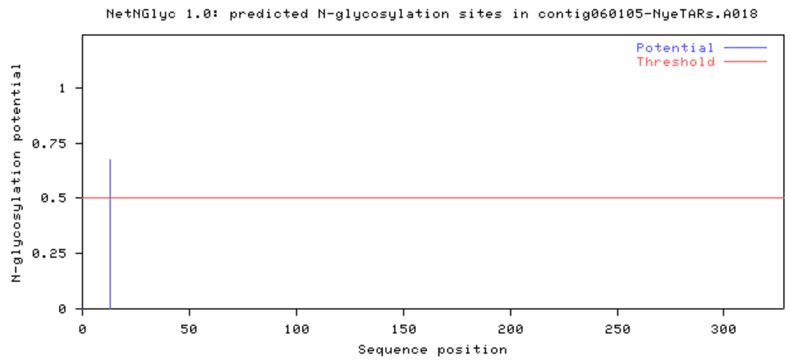

Graphics in PostScript

Output for 'contig046010-NyeTARs.A019'

```
##########################################################################################

    Warning: This sequence may not contain a signal peptide!!

    Proteins without signal peptides are unlikely to be exposed to
    the N-glycosylation machinery and thus may not be glycosylated
    (in vivo) even though they contain potential motifs.

    SignalP-NN euk predictions are as follows:

  # name                 Cmax   pos ?  Ymax   pos ?  Smax   pos ?  Smean ?  D     ?

    SignalP output is explained at http://www.cbs.dtu.dk/services/SignalP/output.html

##########################################################################################

Name:  contig046010-NyeTARs.A019    Length:  327
MQKEAELCFPQLLNSSCRKPTFHWSKAVLLNIVLCCISLLTAALNLLVIISVSYFRQLHTPTNILLLSLAVSDFLTGLLM      80
LPLEIIRNTACWVLGDLMCSVYTYLTAGLFCASTWNIVLISVDRYVAICHPLHYPTRITVARVKLCVCLCWFCSTFYGSL     160
LTKDVLIKPARYNSCYGECVFVINDIAGFIDLVLSFIFPVSVIIVLYMRVFVVAVSQARSMRSHVTAVALQRSSNQANKS     240
ELKAARNLGVLVVVFLACYLPFYCYSLIEVNVINDSSTFFLIIVFYFNSCLNPLMYALFYSWFRNAVKLIITLQIFKHDT     320
SEANMFX
.............N..................................................................      80
................................................................................     160
................................................................................     240
................................................................................     320
.......                                                                              400

(Threshold=0.5)
----------------------------------------------------------------------
SeqName      Position  Potential   Jury    N-Glyc
                      agreement result
----------------------------------------------------------------------
contig046010-NyeTARs.A019    14 NSSC    0.6857     (9/9)    ++
contig046010-NyeTARs.A019   238 NKSE    0.3591     (8/9)    -
contig046010-NyeTARs.A019   274 NDSS    0.4006     (7/9)    -
----------------------------------------------------------------------
```

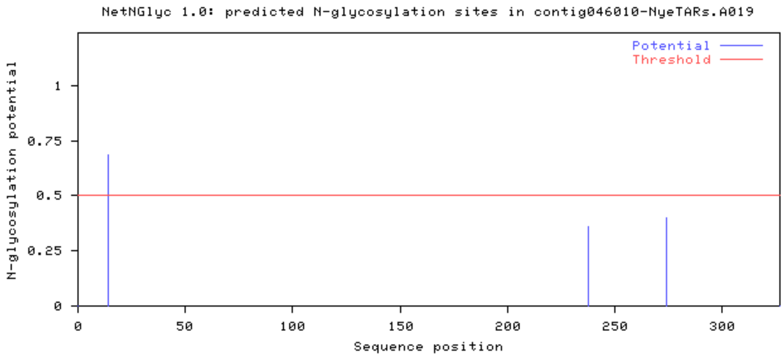

Graphics in PostScript

Output for 'contig046013-NyeTARs.A021'

```
########################################################################################

    Warning: This sequence may not contain a signal peptide!!

    Proteins without signal peptides are unlikely to be exposed to
    the N-glycosylation machinery and thus may not be glycosylated
    (in vivo) even though they contain potential motifs.

    SignalP-NN euk predictions are as follows:

  # name                 Cmax  pos ?  Ymax  pos ?  Smax  pos ?  Smean ?  D     ?

    SignalP output is explained at http://www.cbs.dtu.dk/services/SignalP/output.html

########################################################################################

Name:  contig046013-NyeTARs.A021    Length:  329
MEIPKGVELCFPQLLNSSCRKPTLHWSKAVLLNTVLCCISLLTAALNLLVIISVSHFRQLHTPSNVLLLSLAVSDFFVGL      80
LLLPLEIFRNTSCWVLGDRMCSAYWYLTSNIVCASIGNIVLISVDRYVAICDPLHYPTRITLANVKLSVCLCWFYAFFYS     160
NLYTKDIMIEPGRYNSCFGECVFFGSNIAIVADLILFFFVPVTVIIVLYMRVFVVAVSQARAMRSHVTLVTLQRSLNQTN     240
KSELKAARTLGILVVVFLACFSPLYCYSLVDENAINDPAASFAVIIFYINSCLNPLIYALFYPWFRNAVKLIITLEIFKY     320
DTSGANILX
...............N................................................................      80
.........N......................................................................     160
................................................................................     240
................................................................................     320
.........                                                                            400

(Threshold=0.5)
----------------------------------------------------------------------
SeqName      Position  Potential   Jury    N-Glyc
                      agreement result
----------------------------------------------------------------------
contig046013-NyeTARs.A021   16 NSSC   0.6896     (9/9)   ++
contig046013-NyeTARs.A021   90 NTSC   0.6269     (6/9)   +
contig046013-NyeTARs.A021  237 NQTN   0.4543     (5/9)   -
contig046013-NyeTARs.A021  240 NKSE   0.4190     (5/9)   -
----------------------------------------------------------------------
```

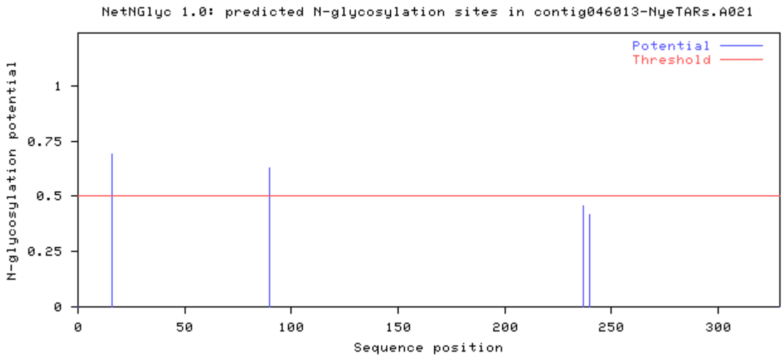

Graphics in PostScript

Output for 'contig062039-NyeTARs.A022'

```
########################################################################################

    Warning: This sequence may not contain a signal peptide!!

    Proteins without signal peptides are unlikely to be exposed to
```

```
    the N-glycosylation machinery and thus may not be glycosylated
    (in vivo) even though they contain potential motifs.

    SignalP-NN euk predictions are as follows:

  # name                 Cmax  pos ?  Ymax  pos ?  Smax  pos ?  Smean ?  D     ?

    SignalP output is explained at http://www.cbs.dtu.dk/services/SignalP/output.html

##########################################################################################

Name:  contig062039-NyeTARs.A022    Length:  322
MMENKDRICFPEVFNSSCRRPAFHWSEAVPLNTLLCSISVITIALNLLVIISVSYFRQLHTPTNILLLSLAVSDFLVGLL      80
LLPGEIFLGIACWAFGNLLCSLFNYVSFIITSASVGNMVLISVDRYVAICYPLHYSTRITVTRVKRSVCLCWLCCVLYSS     160
VLLKDELIQPGRHNSCYGECVFVINFIAGTVDLLLTFIVPISVIVVLYIRVFAVAVSQARAMRSQLSVNLTTKKSELKAG     240
RTLGVLIVVFLMCFCPYYCVSLAGEDSLSESSTSIVRYLFYFNSCLNPLIYALFYPWFRKAIKHIVMLQIFHTGSHEDNI     320
LX
..............N.................................................................      80
................................................................................     160
....................................................................N...........     240
................................................................................     320
..                                                                                   400

(Threshold=0.5)
----------------------------------------------------------------------
SeqName      Position  Potential   Jury    N-Glyc
                      agreement result
----------------------------------------------------------------------
contig062039-NyeTARs.A022   15 NSSC   0.5813     (8/9)   +
contig062039-NyeTARs.A022  229 NLTT   0.5718     (7/9)   +
----------------------------------------------------------------------
```

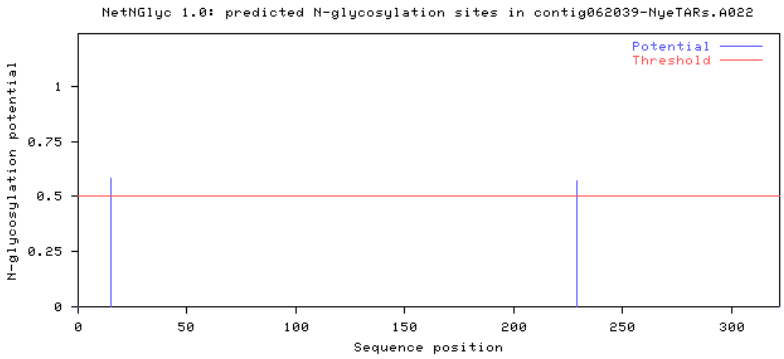

Graphics in PostScript

Output for 'contig035377-NyeTARs.A023'

```
##########################################################################################

    Warning: This sequence may not contain a signal peptide!!

    Proteins without signal peptides are unlikely to be exposed to
    the N-glycosylation machinery and thus may not be glycosylated
    (in vivo) even though they contain potential motifs.

    SignalP-NN euk predictions are as follows:

  # name                 Cmax  pos ?  Ymax  pos ?  Smax  pos ?  Smean ?  D     ?

    SignalP output is explained at http://www.cbs.dtu.dk/services/SignalP/output.html

##########################################################################################

Name:  contig035377-NyeTARs.A023    Length:  330
MDTQDGAELCFPQLFNISCKKPTTPLSQLLLPYIVVSSMSLLTVTLNLLVIIAVSHFRQLNTPTNILLLSLAVSDFFIGL      80
LLMPANIIRKKVCWFLGQLACLVFTYVSFIITSASVGIMVLISIDRYAAICDPLHYPTRITRERVKLCVCLCWLCSVFYN     160
ILFIKNDLLQRERHTSCYGECKILVDYIAGTTDIVLTFIAPVTVIIVLYMRVFVVAVSQARAMRSQVTAVTLQLSVTLTT     240
KKSELKAARTLGVLVLVFLICFCPHYCIFVFGGKVLNSSSATIVIYLYYFNSCLNPLIYAMFYPWFRKAVKLIVTLQILQ     320
PGSCEVSILX
...............N................................................................      80
................................................................................     160
................................................................................     240
....................................N...........................................     320
..........                                                                           400

(Threshold=0.5)
----------------------------------------------------------------------
SeqName      Position  Potential   Jury    N-Glyc
                      agreement result
----------------------------------------------------------------------
contig035377-NyeTARs.A023   16 NISC   0.6413     (8/9)   +
contig035377-NyeTARs.A023  277 NSSS   0.5306     (5/9)   +
----------------------------------------------------------------------
```

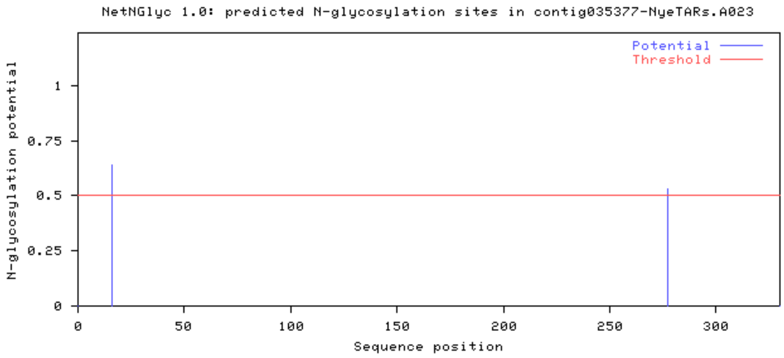

Graphics in PostScript

Output for 'contig046014-NyeTARs.A024'

```
##########################################################################################

    Warning: This sequence may not contain a signal peptide!!

    Proteins without signal peptides are unlikely to be exposed to
    the N-glycosylation machinery and thus may not be glycosylated
    (in vivo) even though they contain potential motifs.

    SignalP-NN euk predictions are as follows:

  # name                Cmax  pos ?  Ymax  pos ?  Smax  pos ?  Smean ?  D     ?

    SignalP output is explained at http://www.cbs.dtu.dk/services/SignalP/output.html

##########################################################################################

Name:  contig046014-NyeTARs.A024    Length:  329
MEIQKAAELCFPQLLNSSCRKPTLHWSKAVLLNIVLSCISLITAALNLLVIISVSHFRQLHTPSNILLVSLAVSDFLVGL      80
LLMPLEIFRNTACWVLGDVMCSVYWYLTSNITCASIGNIVLISVDRYVAICDPLHYPTRITLVKVKLSVCLCWFFSIFYW     160
SLYMKDILVEPGRYNSCYGECVLIINDIAGIVDLALSFIVPVTVIIVLYMRVFVVAVSQARAMRSHVTFVTLQCSLNQAN     240
KSELKAARTLGVLVVVFLACYCPFYCYSLVDKNVVNDSSASFVVLVFYFNSCLNPLIYALFYPWFRKAIRCVITLLIFKH     320
DSSEVNVIX
...............N................................................................      80
.............................N..................................................     160
................................................................................     240
................................................................................     320
.........                                                                            400

(Threshold=0.5)
----------------------------------------------------------------------
SeqName      Position  Potential   Jury    N-Glyc
                                 agreement result
----------------------------------------------------------------------
contig046014-NyeTARs.A024   16 NSSC   0.7030     (9/9)   ++
contig046014-NyeTARs.A024  110 NITC   0.7314     (9/9)   ++
contig046014-NyeTARs.A024  240 NKSE   0.3681     (8/9)   -
contig046014-NyeTARs.A024  276 NDSS   0.3994     (7/9)   -
----------------------------------------------------------------------
```

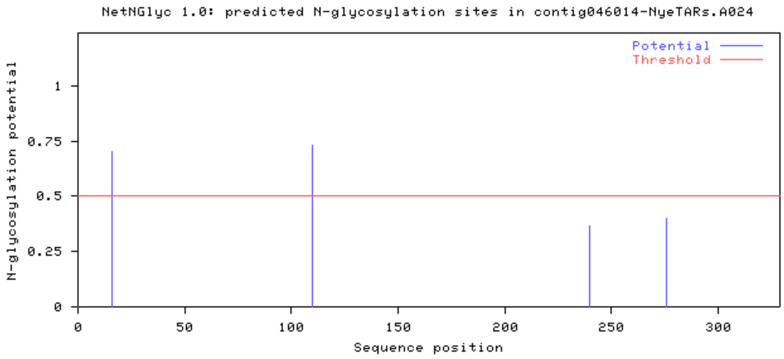

Graphics in PostScript

Output for 'contig058002-NyeTARs.A025'

```
##########################################################################################

    Warning: This sequence may not contain a signal peptide!!

    Proteins without signal peptides are unlikely to be exposed to
```

```
    the N-glycosylation machinery and thus may not be glycosylated
    (in vivo) even though they contain potential motifs.

    SignalP-NN euk predictions are as follows:

  # name                 Cmax  pos ?  Ymax  pos ?  Smax  pos ?  Smean ?  D     ?

    SignalP output is explained at http://www.cbs.dtu.dk/services/SignalP/output.html

##########################################################################################

Name:  contig058002-NyeTARs.A025    Length:  333
MEMFEETQLCFPQLLNFSCRKPIRPHSVSILIYITLSSISLLTVTLNLLVIISISHFKKLHTPTNLLLLSLAVSDCLVGL      80
LILFQIMIIDGCWFLGEFMCSMYFLLDYIITSASVGTMVLISIDRYVAICYPLHYSTKVTPKRTKACVYLCWICSSVDQC     160
LLLKDNLVQPGRYNSCYGECVVVVGHAFGVADLLLSIIGPVTVIVVLYLNVFVVAMTQARALRSHIAALTHERSVSTNVK     240
KSEMKAVRTISVLIIVFLICLCPYYCVTLSGQDAMLSASSVAFVMCLFYLNSCLNPLIYALFYPWFRKSVKQIVTLKILK     320
SGSCDTNIMYTEX
...............N................................................................      80
................................................................................     160
................................................................................     240
................................................................................     320
.............                                                                        400

(Threshold=0.5)
----------------------------------------------------------------------
SeqName      Position  Potential   Jury    N-Glyc
                      agreement result
----------------------------------------------------------------------
contig058002-NyeTARs.A025   16 NFSC   0.7008     (9/9)    ++
----------------------------------------------------------------------
```

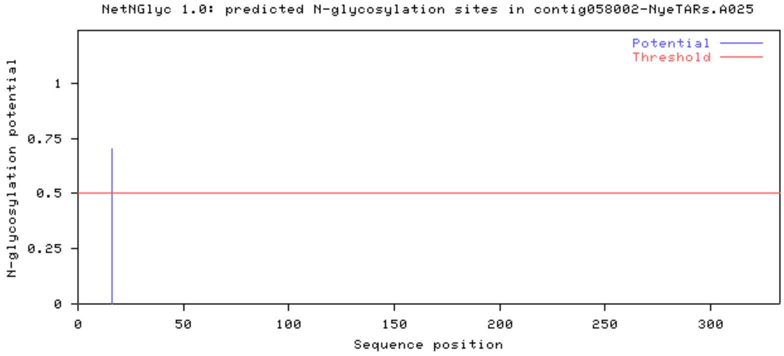

Graphics in PostScript

Output for 'contig045999-NyeTARs.A026'

```
##########################################################################################

    Warning: This sequence may not contain a signal peptide!!

    Proteins without signal peptides are unlikely to be exposed to
    the N-glycosylation machinery and thus may not be glycosylated
    (in vivo) even though they contain potential motifs.

    SignalP-NN euk predictions are as follows:

  # name                 Cmax  pos ?  Ymax  pos ?  Smax  pos ?  Smean ?  D     ?

    SignalP output is explained at http://www.cbs.dtu.dk/services/SignalP/output.html

##########################################################################################

Name:  contig045999-NyeTARs.A026    Length:  333
MEIQMHPDAELCFPELLNSSCRKPTLHWSKTVLLNVGLSSISLITAALNLLIIISVSHFRQLHTPSNIIILSLAVSDFFV      80
CFLLMPVEIFKNTACWVFGDLMCSLYTYLSCILINASFEMIILVSVDRYVAICDPLHYPTRITVPRVKLSVFLCWLYAIL     160
YNIIYTKHALINPGRYGSCYGECVFVVDDIIGIVDFVVSLIVPVTIIVVLYTRVFVVVVSQARAMRSHVTAVTLQRPLNQ     240
ANKSELKAARNLGILVVVFLACYCPFYCYFFLAGNEVNASSASSILIVYYFNSCLNPLMYALFYSWFRNAVKLIITLQIL     320
KANSSEINILQRX
.................N..............................................................      80
..................................N.............................................     160
................................................................................     240
................................................................................     320
.............                                                                        400

(Threshold=0.5)
----------------------------------------------------------------------
SeqName      Position  Potential   Jury    N-Glyc
                      agreement result
----------------------------------------------------------------------
contig045999-NyeTARs.A026   18 NSSC   0.6863     (9/9)    ++
contig045999-NyeTARs.A026  115 NASF   0.5099     (6/9)    +
contig045999-NyeTARs.A026  242 NKSE   0.3963     (7/9)    -
contig045999-NyeTARs.A026  278 NASS   0.3586     (8/9)    -
contig045999-NyeTARs.A026  323 NSSE   0.4093     (6/9)    -
----------------------------------------------------------------------
```

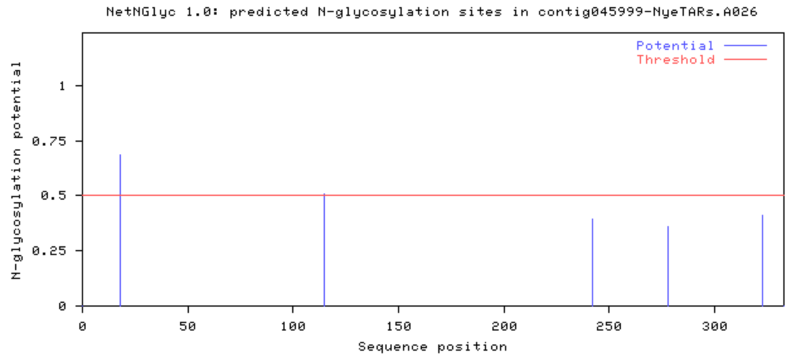

Graphics in PostScript

Output for 'contig060292-NyeTARs.A027'

```
##########################################################################################

    Warning: This sequence may not contain a signal peptide!!

    Proteins without signal peptides are unlikely to be exposed to
    the N-glycosylation machinery and thus may not be glycosylated
    (in vivo) even though they contain potential motifs.

    SignalP-NN euk predictions are as follows:

  # name                Cmax  pos ?  Ymax  pos ?  Smax  pos ?  Smean ?  D     ?

    SignalP output is explained at http://www.cbs.dtu.dk/services/SignalP/output.html

##########################################################################################

Name:  contig060292-NyeTARs.A027    Length:  324
MAETELCFPNLNSSCSRVKRSPSDSVLIYVILTIISLLTVALNLLVIISISHFKQLHTPTNHLLLSLAVSDFFVGLNMCF      80
QSMLIDGCWYLGDLMCVLYYVFDIVATSASVGTMVLISVDRYVAICDPLHYPTKVTPKRVQTCVLMCWICSLLVVGVLLK     160
DNLDKPGRFNSCFGECVIYVDFAIQVTDLILTILLPITVIVILYVRVFAVAVFQIRAMQPHVAAVTQRGKVSPKKSELKA     240
ARTLGIVIVAFLICLFPYYSVILSGQDTLLDILSVTFFLCLFYFNSCLNPIIYAFFYPWFRKSVKLIVTFQIVKSGSSDA     320
SMLX
...........N....................................................................      80
................................................................................     160
................................................................................     240
................................................................................     320
....                                                                                 400

(Threshold=0.5)
----------------------------------------------------------------------
SeqName      Position  Potential   Jury    N-Glyc
                       agreement result
----------------------------------------------------------------------
contig060292-NyeTARs.A027   12 NSSC   0.7291     (9/9)    ++
----------------------------------------------------------------------
```

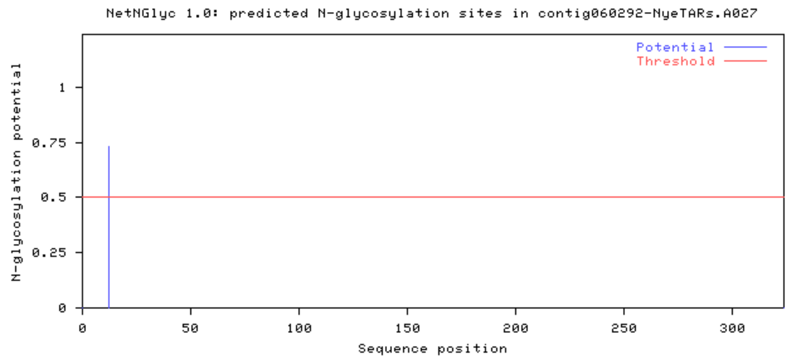

Graphics in PostScript

Output for 'contig042499-NyeTARs.A028'

```
##########################################################################################

    Warning: This sequence may not contain a signal peptide!!

    Proteins without signal peptides are unlikely to be exposed to
    the N-glycosylation machinery and thus may not be glycosylated
    (in vivo) even though they contain potential motifs.
```

```
    SignalP-NN euk predictions are as follows:

  # name                 Cmax  pos ?  Ymax  pos ?  Smax  pos ?  Smean ?  D     ?

    SignalP output is explained at http://www.cbs.dtu.dk/services/SignalP/output.html

########################################################################################

Name:  contig042499-NyeTARs.A028    Length:  331
MEGVEFCFPHLLNSSCRKALHPVSVSTLIYMIISSISVLTATLNLLVIISISHFKQLHNPTNFLLLSLAVSDFFVGLYLL      80
FYIMFIDGCWYFGDFMCILYYVIATINTSSSIGTMVLISVDRYVAICDPLHYPTKVTAKRVQICVSLCWSFSALAGSFLL     160
KDNLKQQSRFNSCVGECVVHINFIEYVADFALNFLLPITVIIVLYLRIFVVVVSQVRAMRSHTAGVTYQCSRKGNPKKSE     240
MKAARTLGIVVIAFLSCALPFYCVTLTGQNAFLNGSSSAFVLCLFYFNSCLNPIIYALFYPWFRKSVKLIVTFQILKSGS     320
RNANIVKVTEX
............N.............................................N.....................      80
................................................................................     160
................................................................................     240
.................................N..............................................     320
...........                                                                          400

(Threshold=0.5)
----------------------------------------------------------------------
SeqName      Position  Potential   Jury    N-Glyc
                                 agreement result
----------------------------------------------------------------------
contig042499-NyeTARs.A028   13 NSSC   0.6814     (9/9)   ++
contig042499-NyeTARs.A028   59 NPTN   0.7822     (9/9)   +++  WARNING: PRO-X1.
contig042499-NyeTARs.A028  107 NTSS   0.4988     (6/9)   -
contig042499-NyeTARs.A028  274 NGSS   0.6617     (7/9)   +
----------------------------------------------------------------------
```

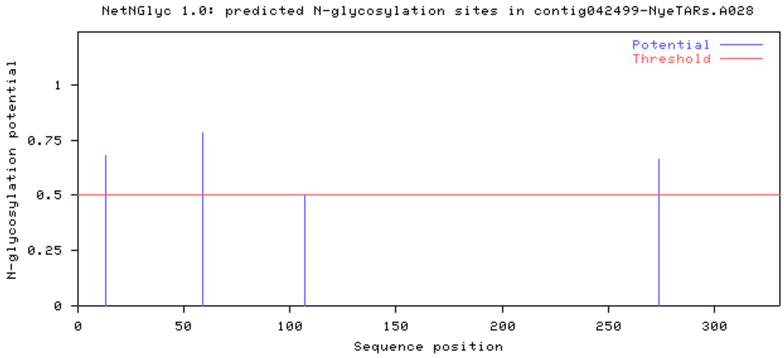

Graphics in PostScript

Output for 'contig056200-NyeTARs.A029'

```
########################################################################################

    Warning: This sequence may not contain a signal peptide!!

    Proteins without signal peptides are unlikely to be exposed to
    the N-glycosylation machinery and thus may not be glycosylated
    (in vivo) even though they contain potential motifs.

    SignalP-NN euk predictions are as follows:

  # name                 Cmax  pos ?  Ymax  pos ?  Smax  pos ?  Smean ?  D     ?

    SignalP output is explained at http://www.cbs.dtu.dk/services/SignalP/output.html

########################################################################################

Name:  contig056200-NyeTARs.A029    Length:  329
MGTQEKSELCFPQLFNMSCKKPAIARSKAVFLHIVLSSVSFLTVALNLLVIISVSHFRQLHTPTNILLLSLAVSDFLVGL      80
LLSPAEILRSTACWFLGQPTCLMYIFVSLTVTSASVGIMVLISADRYVAICDPLNYPIRITDRRVQLCVCLCWLCSIAFS     160
CFLVRDDLHQKRKQNSCYGKCVVVVQYIAGVVDLILTFIVPVTVIIVLYMRVFVVAVSQARSMRSHATAVRNHLPMTLTK     240
KSELKAARTLGVLVLVFLMCFCPYYCVSLVGEEFINSSSASFVAYLFGLNSCLNPLIYAMFYPWFRKAVKLVVTLQILQP     320
GSSEVSILX
...............N................................................................      80
................................................................................     160
................................................................................     240
................................................................................     320
.........                                                                            400

(Threshold=0.5)
----------------------------------------------------------------------
SeqName      Position  Potential   Jury    N-Glyc
                                 agreement result
----------------------------------------------------------------------
contig056200-NyeTARs.A029   16 NMSC   0.5922     (8/9)   +
contig056200-NyeTARs.A029  276 NSSS   0.4743     (5/9)   -
----------------------------------------------------------------------
```

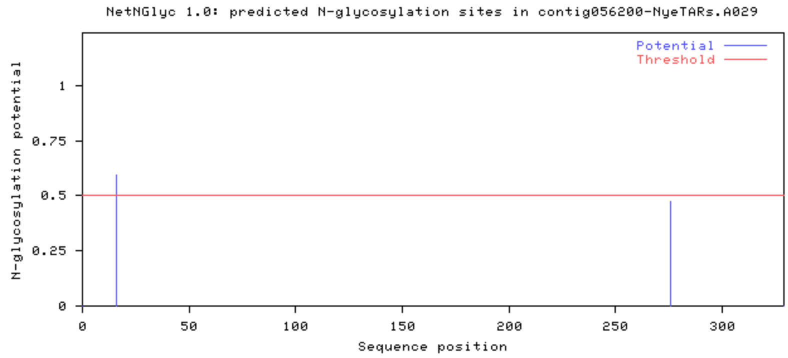

Graphics in PostScript

Output for 'contig052987-NyeTAR.B030'

```
##########################################################################################

    Warning: This sequence may not contain a signal peptide!!

    Proteins without signal peptides are unlikely to be exposed to
    the N-glycosylation machinery and thus may not be glycosylated
    (in vivo) even though they contain potential motifs.

    SignalP-NN euk predictions are as follows:

  # name                Cmax  pos ?  Ymax  pos ?  Smax  pos ?  Smean ?  D     ?

    SignalP output is explained at http://www.cbs.dtu.dk/services/SignalP/output.html

##########################################################################################

Name:  contig052987-NyeTAR.B030     Length:  315
MELFNVTVNTVSFLLCDSQKNKLCVLLYVVLSSFMLLTICGNLLVIISIIYFRYLHTPTNYLILSMAVADLLIGALIFPL      80
SMTVSLKPCLYIYSLLCNLRSTMDVTMGVSSLLNLCCISVDRYYAVCHPLIYKTKITDCVAMKMGLGSWAVAILCGIFVF     160
LLFFILDECDTSCVFALIAASVVVYYIPTIVLLFMYTKILVVALRQARSIHNTISQNTKSKAVSSTERKATKTLTIVIGI     240
FLIFWVPLFLSYSFVPLDSFILYVLLEPFNWFAISNSMLNPFIYAFFYTWFRRAFKMIISGKIFQGDVTNIKLHX
....N...........................................................................      80
................................................................................     160
................................................................................     240
...........................................................................          320

(Threshold=0.5)
----------------------------------------------------------------------
SeqName      Position  Potential   Jury    N-Glyc
                      agreement result
----------------------------------------------------------------------
contig052987-NyeTAR.B030     5 NVTV   0.7989     (9/9)   +++
----------------------------------------------------------------------
```

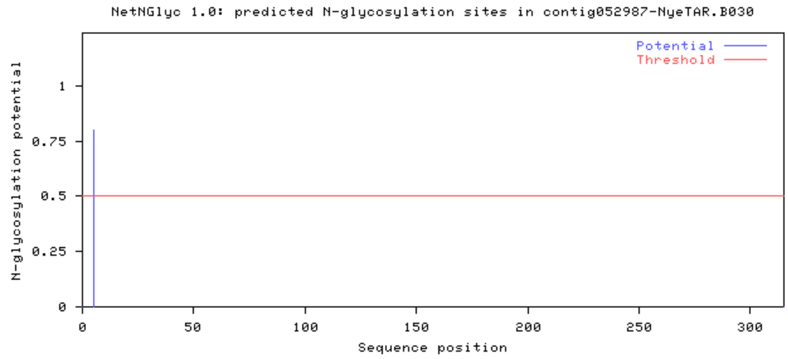

Graphics in PostScript

Output for 'contig045302-BurTAR.A001'

```
##########################################################################################

    Warning: This sequence may not contain a signal peptide!!

    Proteins without signal peptides are unlikely to be exposed to
    the N-glycosylation machinery and thus may not be glycosylated
    (in vivo) even though they contain potential motifs.

    SignalP-NN euk predictions are as follows:
```

```
 # name                 Cmax  pos ?  Ymax  pos ?  Smax  pos ?  Smean ?  D     ?

    SignalP output is explained at http://www.cbs.dtu.dk/services/SignalP/output.html

##########################################################################################

Name:  contig045302-BurTAR.A001     Length:  326
MDSSGGPPLCFPNLNSSCRRLLRPTSQTVVLYTLLASISLLTVVLNLLVVISISHFRQLNTSTNTLLLSLAVSDLLVGLL      80
VMPIEGLYYIETCWLLGRLMCALSPYLSYCLISVSLDSMVLISVDRYIAICDPLLYSSKITVNRVKLSVCVCWACSLLYN     160
GCILMEHIGWPDRFSSCHGECVVFISRALGTIDLFFSFLGPCALMFVLYMRVFVVAVSQVRIIRSQAAVRAPPAAKKSEL     240
KAARTLGILIAVFLMCFCPYYYPSFAGDDTSMNLPYYALFSWIMLTNSCVNPVIYALFYPWFRRAIRLIVTLRILQPHSR     320
DVKILX
..............N............................................N....................      80
................................................................................     160
................................................................................     240
................................................................................     320
......                                                                               400

(Threshold=0.5)
----------------------------------------------------------------------
SeqName      Position  Potential   Jury    N-Glyc
                       agreement result
----------------------------------------------------------------------
contig045302-BurTAR.A001   15 NSSC   0.6957     (9/9)    ++
contig045302-BurTAR.A001   60 NTST   0.6153     (8/9)    +
----------------------------------------------------------------------
```

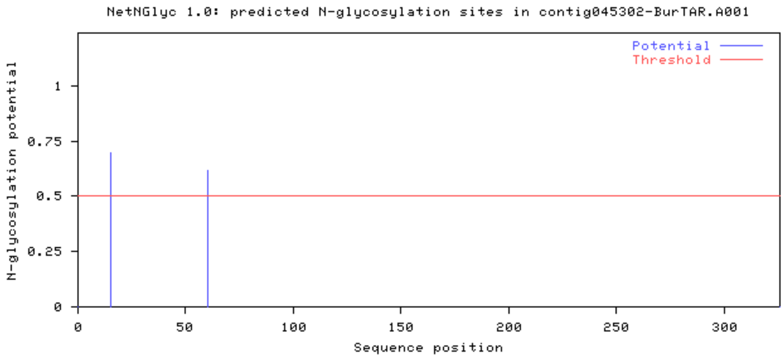

Graphics in PostScript

Output for 'contig020038-BurTAR.A002'

```
##########################################################################################

    Warning: This sequence may not contain a signal peptide!!

    Proteins without signal peptides are unlikely to be exposed to
    the N-glycosylation machinery and thus may not be glycosylated
    (in vivo) even though they contain potential motifs.

    SignalP-NN euk predictions are as follows:

 # name                 Cmax  pos ?  Ymax  pos ?  Smax  pos ?  Smean ?  D     ?

    SignalP output is explained at http://www.cbs.dtu.dk/services/SignalP/output.html

##########################################################################################

Name:  contig020038-BurTAR.A002     Length:  331
MDRQDRIELCYPELANLSCRGLTRPQPEAFLLYTLFLSISVLTVALNVLVIISISHFRQLHMPTNVLLLSLAISDLLVGL      80
LVMPVEAMRFIETCWMLGDLMCAFTYIIGFTLTSASVGNMVLISIDRYVAICYPLQYPTKITHSRAELSVTLCWACSLLY     160
NGMILKEHLRQPDRHNTCHGQCLVVINYVSGAIDLVFTFIGPCSVIIILYMRVFVVALSQAHAMRSHITAAAAGTVKITA     240
KKSEKKAARTLGVVIFVFLMSFCPYYYPSLAGQDISNSASSWAIVSWMLYFNSCLNPLIYAFFYPWFRKAIRFIVSLKIL     320
EKGSSQANILX
...............N................................................................      80
................................................................................     160
................................................................................     240
................................................................................     320
...........                                                                          400

(Threshold=0.5)
----------------------------------------------------------------------
SeqName      Position  Potential   Jury    N-Glyc
                       agreement result
----------------------------------------------------------------------
contig020038-BurTAR.A002   16 NLSC   0.6718     (8/9)    +
----------------------------------------------------------------------
```

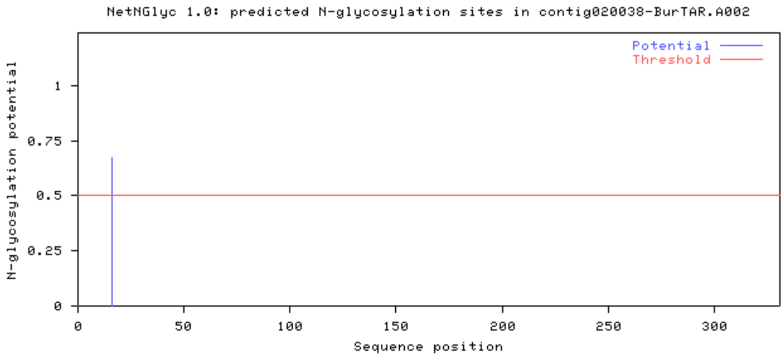

Graphics in PostScript

Output for 'contig061977-BurTARs.A012'

```
##########################################################################################

    Warning: This sequence may not contain a signal peptide!!

    Proteins without signal peptides are unlikely to be exposed to
    the N-glycosylation machinery and thus may not be glycosylated
    (in vivo) even though they contain potential motifs.

    SignalP-NN euk predictions are as follows:

  # name                Cmax  pos ?  Ymax  pos ?  Smax  pos ?  Smean ?  D     ?

    SignalP output is explained at http://www.cbs.dtu.dk/services/SignalP/output.html

##########################################################################################

Name:  contig061977-BurTARs.A012    Length:  327
MDTQDVAELCFPQLFNTSCKKPLSEFVFLHVVLSSISLLTVTLNLLVIISVSHYRQLHTPTNILLLSLAVSDFLVGLLLM      80
PGEILRNTACWFLGDLTCSMYNYMSLIVTSTSVGDMVLISIDRYVAICDPLHYPTRITDRRVKLCVCLCWLCSVFYSSLF     160
VKDDLTQPGKHNSCYGECTIVVDLITGTIDLLLTFFVPVTVIVVLYLRVFVVAVSQARAMRSHVTVAALQLSVTLTTKKS     240
ELKAARTLGVLVVVFLLCFCPYYCVTLARDDLLNSSSVSFLLYLFYFNSCLNPLIYALLYPWFRKAVKLIISLHILQPGS     320
CEISILX
...............N................................................................      80
................................................................................     160
................................................................................     240
.................................N..............................................     320
.......                                                                              400

(Threshold=0.5)
----------------------------------------------------------------------
SeqName      Position  Potential   Jury    N-Glyc
                                 agreement result
----------------------------------------------------------------------
contig061977-BurTARs.A012   16 NTSC   0.5843     (7/9)   +
contig061977-BurTARs.A012  274 NSSS   0.5953     (6/9)   +
----------------------------------------------------------------------
```

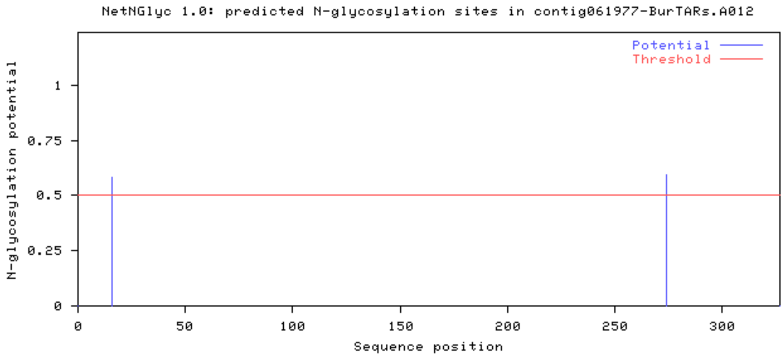

Graphics in PostScript

Output for 'contig061433-BurTARs.A013'

```
##########################################################################################

    Warning: This sequence may not contain a signal peptide!!

    Proteins without signal peptides are unlikely to be exposed to
    the N-glycosylation machinery and thus may not be glycosylated
    (in vivo) even though they contain potential motifs.
```

```
    SignalP-NN euk predictions are as follows:

  # name                 Cmax  pos ?  Ymax  pos ?  Smax  pos ?  Smean ?  D     ?

    SignalP output is explained at http://www.cbs.dtu.dk/services/SignalP/output.html

##########################################################################################

Name:  contig061433-BurTARs.A013    Length:  330
MEIQMHPEAELCFPELPNSSCRKPTLHWSKTVLLNVGLSSISVITAALNLLIIISVYHFRQLHTPSNILLLSLAVSDFFV      80
GFLLLPVEIFKNTACWVFGDLMCSLYIYLSGILMNASIEIIVFISVDRYVAICDPLHYPTRITVTRVKLSVCLCWFYAIF     160
YMSLYTKDVLIKPGRYASCYGECVFFIEDITGTVDIVLCFFVPVIIIIVLYTRVFVVAVSQARAMRSHVTAVTLQRSLNQ     240
SNKSELKAARNLGVLVIVFLASICPFYFYSLVDGNVVKASSATFLIIVYFNACLNPLIYTLFYPWFRNAVKLIITLQIFK     320
HNSSEANILX
.................N..............................................................      80
..................................N.............................................     160
................................................................................     240
................................................................................     320
..........                                                                           400

(Threshold=0.5)
----------------------------------------------------------------------
SeqName      Position  Potential   Jury    N-Glyc
                                 agreement result
----------------------------------------------------------------------
contig061433-BurTARs.A013   18 NSSC   0.6481     (9/9)   ++
contig061433-BurTARs.A013  115 NASI   0.5447     (6/9)   +
contig061433-BurTARs.A013  239 NQSN   0.3874     (7/9)   -
contig061433-BurTARs.A013  242 NKSE   0.4206     (7/9)   -
contig061433-BurTARs.A013  322 NSSE   0.4091     (6/9)   -
----------------------------------------------------------------------
```

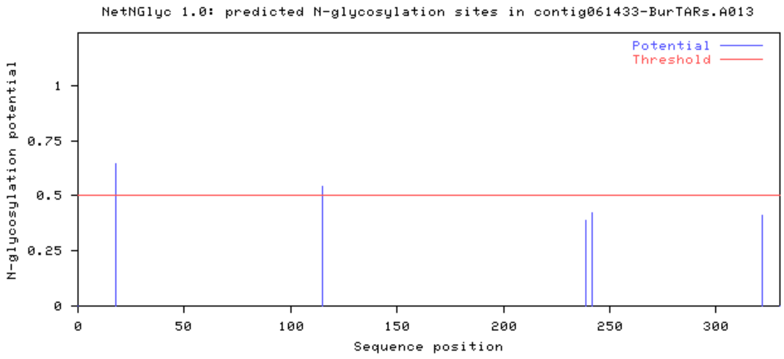

Graphics in PostScript

Output for 'contig061091-BurTARs.A014'

```
##########################################################################################

    Warning: This sequence may not contain a signal peptide!!

    Proteins without signal peptides are unlikely to be exposed to
    the N-glycosylation machinery and thus may not be glycosylated
    (in vivo) even though they contain potential motifs.

    SignalP-NN euk predictions are as follows:

  # name                 Cmax  pos ?  Ymax  pos ?  Smax  pos ?  Smean ?  D     ?

    SignalP output is explained at http://www.cbs.dtu.dk/services/SignalP/output.html

##########################################################################################

Name:  contig061091-BurTARs.A014    Length:  329
MEIPKGVELCFPQLLNSSCRKPTLHWSKAVLLNIVLSCISLLTAALNLLVIISVSHFRQLHTPSNILLLSLAVSDFFVGL      80
LLLPLEIFRNTSCWVLGDRMCSAYWYLTSNIICASIGNIVLISVDRYVAICDPLHYPSRITLAKVKLSVCLCWFYAFFYS     160
NLYTKNIMIEPGRYNSCYGECVFFSSNIAIVADLILFFFVPVTVIIVLYMRVFVVAVSQARAMRSHVTLVTLQRSLNQTN     240
KSELKAARTLGILVVVFLACFSPLYCYSLVDENAINNPAASFAVIIFYINSCLNPLIYALFYPWFRNAVKLIITLEIFKY     320
DTSGANILX
...............N................................................................      80
.........N......................................................................     160
................................................................................     240
................................................................................     320
.........                                                                            400

(Threshold=0.5)
----------------------------------------------------------------------
SeqName      Position  Potential   Jury    N-Glyc
                                 agreement result
----------------------------------------------------------------------
contig061091-BurTARs.A014   16 NSSC   0.6896     (9/9)   ++
contig061091-BurTARs.A014   90 NTSC   0.6268     (6/9)   +
contig061091-BurTARs.A014  237 NQTN   0.4541     (5/9)   -
contig061091-BurTARs.A014  240 NKSE   0.4186     (5/9)   -
----------------------------------------------------------------------
```

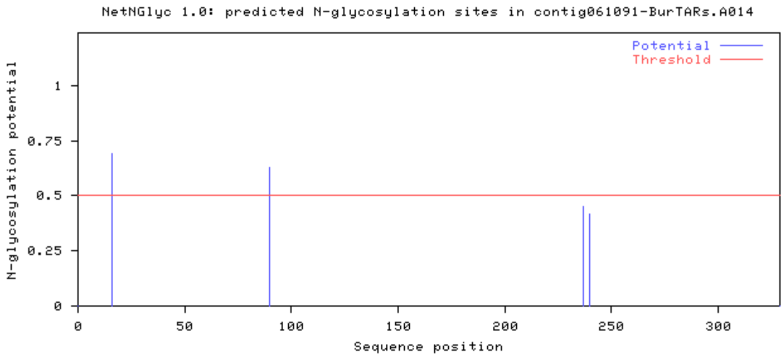

Graphics in PostScript

Output for 'contig060707-BurTARs.A015'

```
##########################################################################################

    Warning: This sequence may not contain a signal peptide!!

    Proteins without signal peptides are unlikely to be exposed to
    the N-glycosylation machinery and thus may not be glycosylated
    (in vivo) even though they contain potential motifs.

    SignalP-NN euk predictions are as follows:

  # name                Cmax  pos ?  Ymax  pos ?  Smax  pos ?  Smean ?  D     ?

    SignalP output is explained at http://www.cbs.dtu.dk/services/SignalP/output.html

##########################################################################################

Name:  contig060707-BurTARs.A015    Length:  327
MEETELCFPQLFNSSCVRQKHSQIEAVFIYTPLSSISVLTTILNLLVIISIAHFKQLHTPTNLLLLSLAVSDFFVGLIMA      80
CQISLLDGCWFLGDHMCALYSSLDYIVTSASVGTMVLISADRYVAICDPLHYPTKITMKRVSVSICTCWACSILYNSLIM     160
KDNFKQPGRYNSCSGDCVVVIDYFVGIFDFVLTFVGPVIVIIVLYLRVFVVAVSQARAMRSHITALRLQGSETVHAKKSE     240
LKAARTLGVLVIAFLICLFPFFCSSMVGQNSFFDIRSVPFELLLFYFNSCLNPLIYTFCYPWFLKSIKLIVTFKIFRHGS     320
SEASILX
............N...................................................................      80
................................................................................     160
................................................................................     240
................................................................................     320
.......                                                                              400

(Threshold=0.5)
----------------------------------------------------------------------
SeqName      Position  Potential   Jury    N-Glyc
                                 agreement result
----------------------------------------------------------------------
contig060707-BurTARs.A015   13 NSSC   0.6738     (9/9)    ++
----------------------------------------------------------------------
```

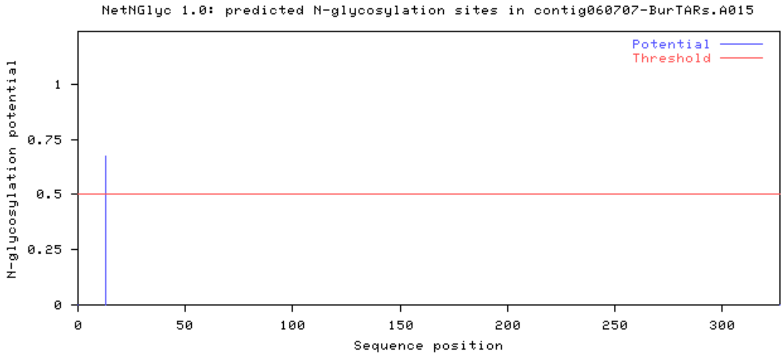

Graphics in PostScript

Output for 'contig059673-BurTARs.A016'

```
##########################################################################################

    Warning: This sequence may not contain a signal peptide!!

    Proteins without signal peptides are unlikely to be exposed to
    the N-glycosylation machinery and thus may not be glycosylated
    (in vivo) even though they contain potential motifs.
```

```
    SignalP-NN euk predictions are as follows:

  # name                 Cmax  pos ?  Ymax  pos ?  Smax  pos ?  Smean ?  D     ?

    SignalP output is explained at http://www.cbs.dtu.dk/services/SignalP/output.html

########################################################################################

Name:  contig059673-BurTARs.A016    Length:  315
MKTFEEAELCFPQLLNSSCRKTMRPYTFSILIYITLSSISLLTKLHTPTNLLLLSLAVSDCLVGLLILFQIMIIDGCWFL      80
GEFMCSMYFLLDYIITSASIGTMVLISIDRYVAICYPLHYSTKVTPKRTKACVYLCWICSSVFQCLLLKDNLVQPGRYNS     160
CYGECVVVVGHAFGVADLLLSIIGPVTVIVVLYLNVFVVAMTQARALRSHIAALTHERSVSTNVKKSEMKAVRTISVLII     240
VFLICLCPYFGVTLSGQDAMLSASSVAFVMCLFYLNSCLNPLIYALFYPWFRKSVKQIVTLKILKSGSCDTNIMX
...............N................................................................      80
................................................................................     160
................................................................................     240
...........................................................................          320

(Threshold=0.5)
----------------------------------------------------------------------
SeqName      Position  Potential   Jury    N-Glyc
                                  agreement result
----------------------------------------------------------------------
contig059673-BurTARs.A016   16 NSSC   0.6663     (9/9)    ++
----------------------------------------------------------------------
```

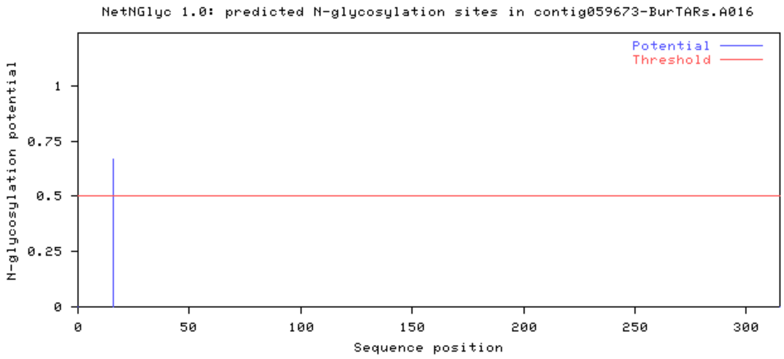

Graphics in PostScript

Output for 'contig057301-BurTARs.A017'

```
########################################################################################

    Warning: This sequence may not contain a signal peptide!!

    Proteins without signal peptides are unlikely to be exposed to
    the N-glycosylation machinery and thus may not be glycosylated
    (in vivo) even though they contain potential motifs.

    SignalP-NN euk predictions are as follows:

  # name                 Cmax  pos ?  Ymax  pos ?  Smax  pos ?  Smean ?  D     ?

    SignalP output is explained at http://www.cbs.dtu.dk/services/SignalP/output.html

########################################################################################

Name:  contig057301-BurTARs.A017    Length:  330
MEGVEFCFPHLLNSSCRKALHPVSVSTLIYMIISSISVLTATLNLLVIISISHFKQLHNPTNFLLLSLAVSDFFVGLYLL      80
FYIMFIDGCWYFGEFMCILYYVIGTINTSSSIGTMVLISVDRYVAICDPLHYPIKVTAKRVQICVSLCWSFSALAGSFLL     160
KDNLKQQSRFNSCVGECVVHINFIEYVADLALNFLLPITVIIVLYLRIFVVVVSQVRAMRTHTAGVTYQCSRKGNPKKSE     240
MKAARTLGIVVIAFLSCALPFYCVTLTGQNAFLNGSSSAFVLCLFYFNSCLNPIIYALFYPWFRKSIKLIVTFQILKSGS     320
RNANIVKVTX
............N.............................................N.....................      80
..........................N.....................................................     160
................................................................................     240
.................................N..............................................     320
..........                                                                           400

(Threshold=0.5)
----------------------------------------------------------------------
SeqName      Position  Potential   Jury    N-Glyc
                                  agreement result
----------------------------------------------------------------------
contig057301-BurTARs.A017   13 NSSC   0.6816     (9/9)    ++
contig057301-BurTARs.A017   59 NPTN   0.7821     (9/9)    +++  WARNING: PRO-X1.
contig057301-BurTARs.A017  107 NTSS   0.6152     (7/9)    +
contig057301-BurTARs.A017  274 NGSS   0.6612     (7/9)    +
----------------------------------------------------------------------
```

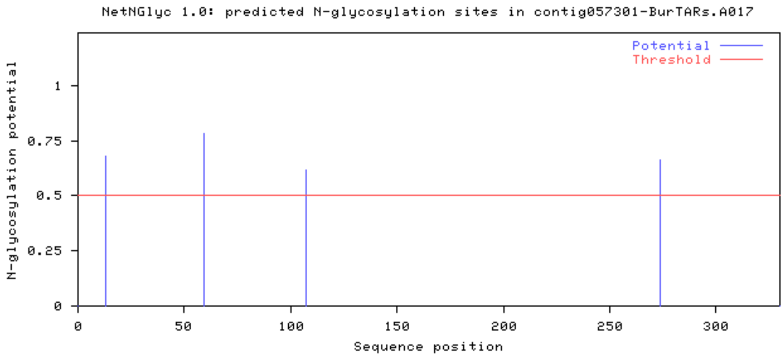

Graphics in PostScript

Output for 'contig057145-BurTARs.A018'

```
##########################################################################################

    Warning: This sequence may not contain a signal peptide!!

    Proteins without signal peptides are unlikely to be exposed to
    the N-glycosylation machinery and thus may not be glycosylated
    (in vivo) even though they contain potential motifs.

    SignalP-NN euk predictions are as follows:

  # name                Cmax  pos ?  Ymax  pos ?  Smax  pos ?  Smean ?  D     ?

    SignalP output is explained at http://www.cbs.dtu.dk/services/SignalP/output.html

##########################################################################################

Name:  contig057145-BurTARs.A018    Length:  329
MEIQKVPELCFPQFLNNSCRKPTLHWSKAVLLNIVFSCISLLTAALNLLVIISVSHFRKLHTPSNIVLLSLAVSDFLVGL      80
LLMPAEILRSTTCWVLGDIMCSVYWYLTGNIICASIGNIVLISVDRYVAICDPLHYRTRITVKRTKLSVCLCWFYAIFYW     160
SLYIKNILIEPGRYNSCYGECMFVSSDIAGIIDLFLSFIVPLSVIIVLYMRVFVAAVSQARAMHSHVTSVTLQHSSNQAK     240
KSELKAARTLGVLVVVFLSCYSPFYCYYLAEENVVNDPSASTVIIIFYINSCLNPLIYALFYPWFRNAVKLIVTLQIFKH     320
GTCEANILX
...............N................................................................      80
................................................................................     160
................................................................................     240
................................................................................     320
.........                                                                            400

(Threshold=0.5)
----------------------------------------------------------------------
SeqName      Position  Potential   Jury    N-Glyc
                    agreement result
----------------------------------------------------------------------
contig057145-BurTARs.A018   16 NNSC   0.5957     (6/9)   +
----------------------------------------------------------------------
```

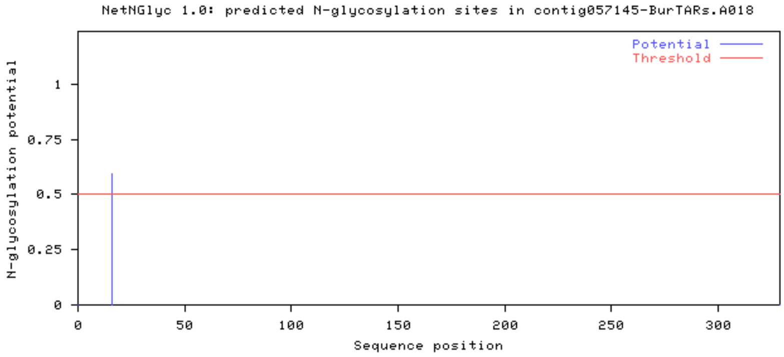

Graphics in PostScript

Output for 'contig056023-BurTARs.A019'

```
##########################################################################################

    Warning: This sequence may not contain a signal peptide!!

    Proteins without signal peptides are unlikely to be exposed to
    the N-glycosylation machinery and thus may not be glycosylated
    (in vivo) even though they contain potential motifs.
```

```
    SignalP-NN euk predictions are as follows:

  # name                 Cmax  pos ?  Ymax  pos ?  Smax  pos ?  Smean ?  D     ?

    SignalP output is explained at http://www.cbs.dtu.dk/services/SignalP/output.html

##########################################################################################

Name:  contig056023-BurTARs.A019    Length:  333
MEIQMHPEAGLCFPELLNSSCRKPTLHWSKTVLLNVGLSSISLITAALNLFIIISVSHFRQLHTPSNIIILSLAVSDFFV      80
CFLLMPVEIFKNTACWVFGDLMCSLYTYLSCILINASFEMIILVSVDRYVAICDPLHYPTRITVPRVKLSVCLCWLYAIL     160
YNIIYTKHALINPGRYGSCYGECVFVVDDIIGIVDFVVSLIVPVTIIVVLYTRVFVVVVSQARAMRSHVTAVTLQRPLNQ     240
ANKSELKAARNLGILVVVFLACYCPFYCYFFLAGNEVNASSASSILIVYYFNSCLNPLMYALFYSWFRNAVKLIITLQIL     320
KANSSEINILQRX
.................N..............................................................      80
..................................N.............................................     160
................................................................................     240
................................................................................     320
.............                                                                        400

(Threshold=0.5)
----------------------------------------------------------------------
SeqName      Position  Potential   Jury    N-Glyc
                      agreement result
----------------------------------------------------------------------
contig056023-BurTARs.A019   18 NSSC   0.6953     (9/9)   ++
contig056023-BurTARs.A019  115 NASF   0.5099     (6/9)   +
contig056023-BurTARs.A019  242 NKSE   0.3963     (7/9)   -
contig056023-BurTARs.A019  278 NASS   0.3587     (8/9)   -
contig056023-BurTARs.A019  323 NSSE   0.4093     (6/9)   -
----------------------------------------------------------------------
```

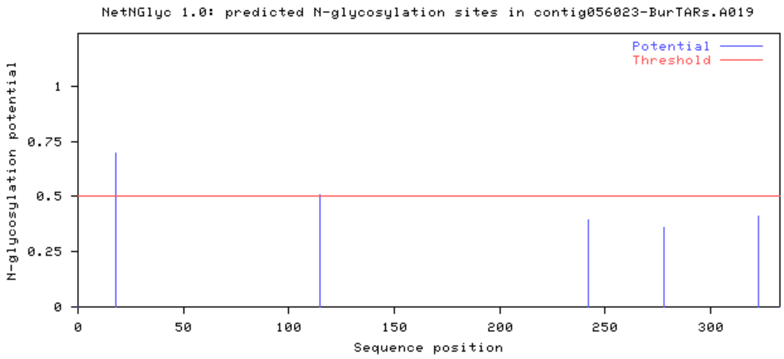

Graphics in PostScript

Output for 'contig056021-BurTARs.A020'

```
##########################################################################################

    Warning: This sequence may not contain a signal peptide!!

    Proteins without signal peptides are unlikely to be exposed to
    the N-glycosylation machinery and thus may not be glycosylated
    (in vivo) even though they contain potential motifs.

    SignalP-NN euk predictions are as follows:

  # name                 Cmax  pos ?  Ymax  pos ?  Smax  pos ?  Smean ?  D     ?

    SignalP output is explained at http://www.cbs.dtu.dk/services/SignalP/output.html

##########################################################################################

Name:  contig056021-BurTARs.A020    Length:  329
MEIQKGAELCFPQLLNISCRKPTLHWSKAVLLNIVLSCISLLTAALNLLVIISVSYFRKLHTPSNILLLSLAVSDFLMGL      80
LLMPAEILRSMTCWVLGDLMCSVYFFLTVNLTCASIGNIVLISIDRYVAICDPLHYSTRITVARVKLSVCLCWFYSTFYC     160
SLYTQEMLIEPGRYNSCYGECVLVISDFAGMVDLILFFILPVIIIIVLYTRVFVVAVSQARAMRSHVTAVTLQRSLNQTN     240
KSELKAARTLGVLVVVFLSCYCPFYCYSLTDKNAVNDPAASFVIFIFYFNSCINPLMYALFYPWFRNAVKLIITLQIFKH     320
NSCEANILX
...............N................................................................      80
.............................N..................................................     160
................................................................................     240
................................................................................     320
.........                                                                            400

(Threshold=0.5)
----------------------------------------------------------------------
SeqName      Position  Potential   Jury    N-Glyc
                      agreement result
----------------------------------------------------------------------
contig056021-BurTARs.A020   16 NISC   0.7473     (9/9)   ++
contig056021-BurTARs.A020  110 NLTC   0.7662     (9/9)   +++
contig056021-BurTARs.A020  237 NQTN   0.4556     (4/9)   -
contig056021-BurTARs.A020  240 NKSE   0.4186     (5/9)   -
----------------------------------------------------------------------
```

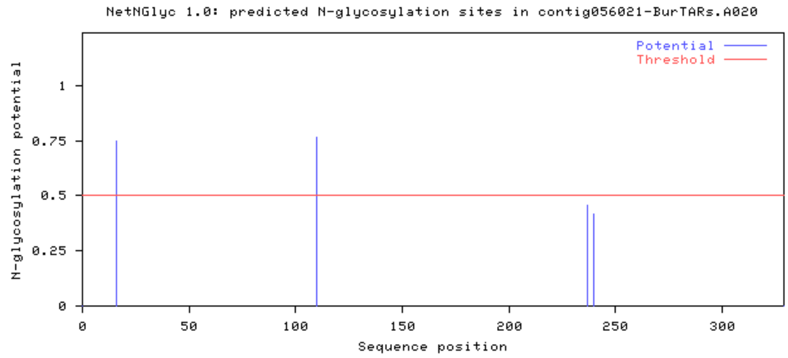

Graphics in PostScript

Output for 'contig056020-BurTARs.A021'

```
##########################################################################################

    Warning: This sequence may not contain a signal peptide!!

    Proteins without signal peptides are unlikely to be exposed to
    the N-glycosylation machinery and thus may not be glycosylated
    (in vivo) even though they contain potential motifs.

    SignalP-NN euk predictions are as follows:

  # name                Cmax  pos ?  Ymax  pos ?  Smax  pos ?  Smean ?  D     ?

    SignalP output is explained at http://www.cbs.dtu.dk/services/SignalP/output.html

##########################################################################################

Name:  contig056020-BurTARs.A021    Length:  329
MEIQKENKLCFPQLLNSSCRKPTLHWSKDVLLNTVLSCISLLTAALNLLVIISVSYFRKLHTPSNILLLSLAVSDFLVGL      80
LLMPLGILRNTGCWVLGDIICSLYWYLTSNIVCASIGNIVLISVDRYVAICDPLHYPSRITLAKVKLSVCLCWFYAFFYV     160
SLYTKDILIEPGRDNSCYGECAFVINDIAVVVDLVFSFIVPVSVIVVLYIRVFVAAVSQARAMRSHVTSVTLQRSLNQTN     240
KSELKAARTLGILVVVFLACFCPLYYFSLVDENAINDPAASFVVIIFYFNSCLNPLIYAFFYPWFRNAVKLIITLQIFKY     320
NMSEANILX
...............N................................................................      80
................................................................................     160
................................................................................     240
................................................................................     320
.........                                                                            400

(Threshold=0.5)
----------------------------------------------------------------------
SeqName      Position  Potential   Jury    N-Glyc
                                  agreement result
----------------------------------------------------------------------
contig056020-BurTARs.A021   16 NSSC   0.6976     (9/9)   ++
contig056020-BurTARs.A021  237 NQTN   0.4542     (5/9)   -
contig056020-BurTARs.A021  240 NKSE   0.4184     (5/9)   -
contig056020-BurTARs.A021  321 NMSE   0.4348     (4/9)   -
----------------------------------------------------------------------
```

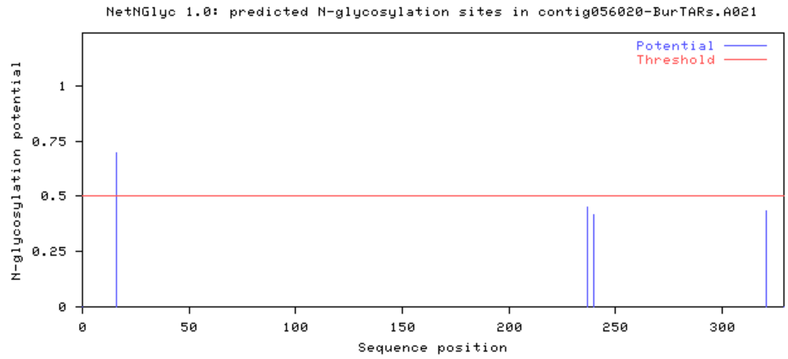

Graphics in PostScript

Output for 'contig055697-BurTARs.A022'

```
##########################################################################################

    Warning: This sequence may not contain a signal peptide!!

    Proteins without signal peptides are unlikely to be exposed to
```

```
    the N-glycosylation machinery and thus may not be glycosylated
    (in vivo) even though they contain potential motifs.

    SignalP-NN euk predictions are as follows:

  # name                 Cmax  pos ?  Ymax  pos ?  Smax  pos ?  Smean ?  D     ?

    SignalP output is explained at http://www.cbs.dtu.dk/services/SignalP/output.html

##########################################################################################

Name:  contig055697-BurTARs.A022    Length:  327
MMEEPELCFPKLLNISCSRPKRPHFEIMLTYILLSFISLLTVILNLLVIISISHFRQLHTPTNFLLLSLAVADFFVGLLM      80
FFQIVLIDGCWFLGDIMCTLYQYLAFIITSASVGTMVIISADRYLAICYPLHYSTQITQQRVNICISLCWFFSVIFQSLI     160
VKDNLKQPGKYNSCIGECVFVVNYIAGLFDLLFSFIVPITVIVVLYLRVFVVAVSQARAMRCQLAVTHQRSVTVTVTKSE     240
LKAARTLGVVVVVFLICMCPYYCVALTGQDNFLNASSAAFVICLVYFNSCLNPIIYVFFYPWFRKSIKLIATLQILQPDS     320
RETNMHX
.............N..................................................................      80
................................................................................     160
................................................................................     240
................................................................................     320
.......                                                                              400

(Threshold=0.5)
----------------------------------------------------------------------
SeqName      Position  Potential   Jury    N-Glyc
                      agreement result
----------------------------------------------------------------------
contig055697-BurTARs.A022   14 NISC   0.7858     (9/9)   +++
contig055697-BurTARs.A022  274 NASS   0.4886     (6/9)   -
----------------------------------------------------------------------
```

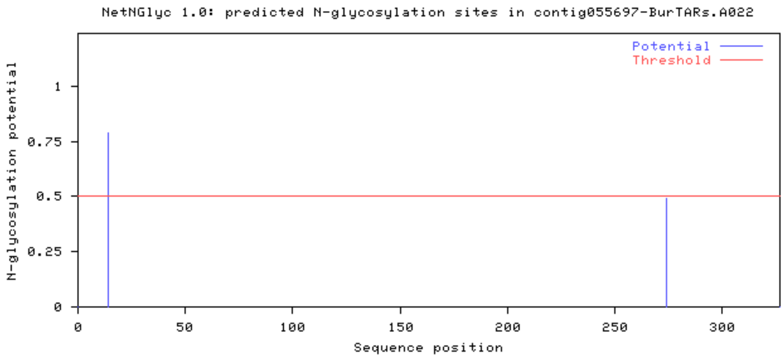

Graphics in PostScript

Output for 'contig054630-BurTARs.A023'

```
##########################################################################################

    Warning: This sequence may not contain a signal peptide!!

    Proteins without signal peptides are unlikely to be exposed to
    the N-glycosylation machinery and thus may not be glycosylated
    (in vivo) even though they contain potential motifs.

    SignalP-NN euk predictions are as follows:

  # name                 Cmax  pos ?  Ymax  pos ?  Smax  pos ?  Smean ?  D     ?

    SignalP output is explained at http://www.cbs.dtu.dk/services/SignalP/output.html

##########################################################################################

Name:  contig054630-BurTARs.A023    Length:  331
MENVYGQQFCFPQLPNTSCRKPLQHHNQTIFLYILLCCISLLTVTLNVLVIISISHFRQLHNQTNLFLLSLAVSDLLVGL      80
LLMPPRILLLGGCWFLGTFMCGLFYYASFVLTSASVGNMVLISFDRYVAICDPLSYPTAVTERKVQISVCLCWACSLLYN     160
GTILNNFLKQPDRYNSCDGECIVVINFITGAFDVVATFIGPTAVIIFLYMRVFLVAVSQAQAMRSHVAFVTSKGSVHVAI     240
KKSERKAATTIGVVVAVFLMCFCPYFYPSLAGQDTSTSVEFSVFGVWLLYCNSCLNPLIYAFFYPWFRKTVKLIVTLQIL     320
QPDSCDANILX
...............N..........N..................................N..................      80
...............................................................................N     160
................................................................................     240
................................................................................     320
...........                                                                          400

(Threshold=0.5)
----------------------------------------------------------------------
SeqName      Position  Potential   Jury    N-Glyc
                      agreement result
----------------------------------------------------------------------
contig054630-BurTARs.A023   16 NTSC   0.6222     (9/9)   ++
contig054630-BurTARs.A023   27 NQTI   0.7473     (9/9)   ++
contig054630-BurTARs.A023   62 NQTN   0.6788     (8/9)   +
contig054630-BurTARs.A023  160 NGTI   0.7078     (9/9)   ++
----------------------------------------------------------------------
```

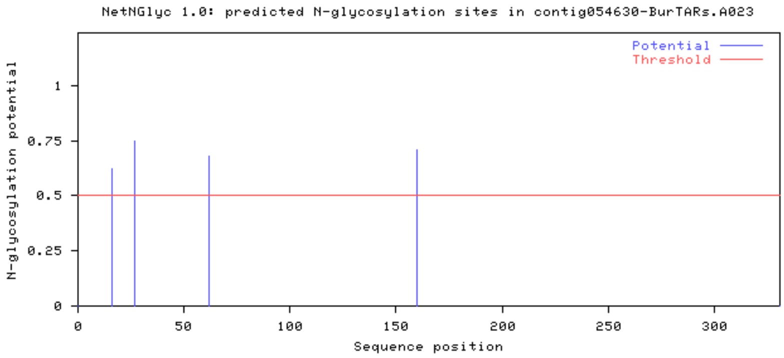

Graphics in PostScript

Output for 'contig049540-BurTARs.A024'

```
##########################################################################################

    Warning: This sequence may not contain a signal peptide!!

    Proteins without signal peptides are unlikely to be exposed to
    the N-glycosylation machinery and thus may not be glycosylated
    (in vivo) even though they contain potential motifs.

    SignalP-NN euk predictions are as follows:

  # name                Cmax  pos ?  Ymax  pos ?  Smax  pos ?  Smean ?  D     ?

    SignalP output is explained at http://www.cbs.dtu.dk/services/SignalP/output.html

##########################################################################################

Name:  contig049540-BurTARs.A024    Length:  329
MGTQEKSELCFPQLFNMSCKKPAIARSKAVFLHIVLSSVSFLTVALNLLVIISVSHFRQLHTPTNILLLSLAVSDFLVGL      80
LLSPAEILRSTACWFLGQPTCLMYIFVSLTVTSASVGIMVLISADRYVAICDPLNYPIRITDRRVKLCVCLCWLCSIAFS     160
CFLVRDDLHQKRKQNSCYGKCVVVVQYIAGVVDLILTFIVPVTVIIVLYMRVFVVAVSQARSMRSHATAVRNHLPMTLTK     240
KSELKAARTLGVLVLVFLMCFCPYYCVSLVGEEFINSSSASFVAYLFGLNSCLNPLIYAMFYPWFRKAVKLVVTLQILQP     320
GSSEVSILX
...............N................................................................      80
................................................................................     160
................................................................................     240
................................................................................     320
.........                                                                            400

(Threshold=0.5)
----------------------------------------------------------------------
SeqName      Position  Potential   Jury    N-Glyc
                       agreement  result
----------------------------------------------------------------------
contig049540-BurTARs.A024    16 NMSC    0.5922     (8/9)    +
contig049540-BurTARs.A024   276 NSSS    0.4743     (5/9)    -
----------------------------------------------------------------------
```

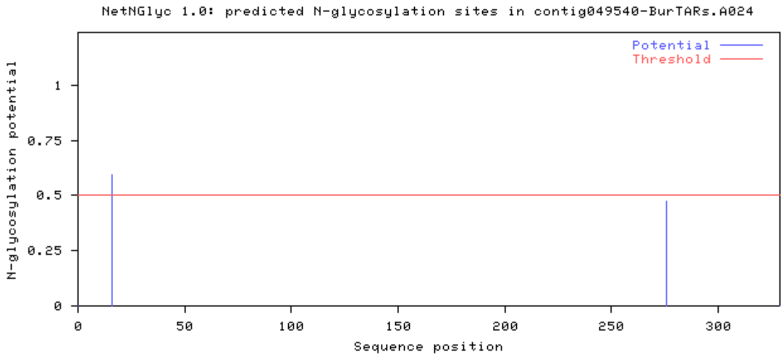

Graphics in PostScript

Output for 'contig049534-BurTARs.A025'

```
##########################################################################################

    Warning: This sequence may not contain a signal peptide!!

    Proteins without signal peptides are unlikely to be exposed to
    the N-glycosylation machinery and thus may not be glycosylated
    (in vivo) even though they contain potential motifs.
```

```
    SignalP-NN euk predictions are as follows:

  # name                 Cmax  pos ?  Ymax  pos ?  Smax  pos ?  Smean ?  D     ?

    SignalP output is explained at http://www.cbs.dtu.dk/services/SignalP/output.html

########################################################################################

Name:  contig049534-BurTARs.A025    Length:  326
MEKGVELCFPQLLNNSCIKPTLHWSKAVLLNIVLSCISLITAGLNLLVIISVSHFRQLHTPSNILLLSLAVSDFLVGLLL      80
IPAEIFRITVCWVFGESMCSLYTYLGYIVVTSSISNIVLISVDRYVAICDPLHYPSRISVAKIRLSVCMCWFYSAFYSTL     160
CTKNILIEPGRYNSCYGECVFITSDIAGIIDLVLSFIVPVSIIIVLYMRVFVVAVSQARAMHSHVTATLQRSLNQTNKSE     240
LKAARTLGVLVVVFLASFCPFYCYFLVVEDIVSDSSASIVVIVYYFNSCLNPLIYALFYPWFRNAVKVIITFQIFKRDSS     320
EANVIX
.............N..................................................................      80
................................................................................     160
................................................................................     240
................................................................................     320
......                                                                               400

(Threshold=0.5)
----------------------------------------------------------------------
SeqName      Position  Potential   Jury    N-Glyc
                                agreement result
----------------------------------------------------------------------
contig049534-BurTARs.A025   14 NNSC   0.6701     (9/9)   ++
contig049534-BurTARs.A025  234 NQTN   0.4641     (4/9)   -
contig049534-BurTARs.A025  237 NKSE   0.4248     (5/9)   -
----------------------------------------------------------------------
```

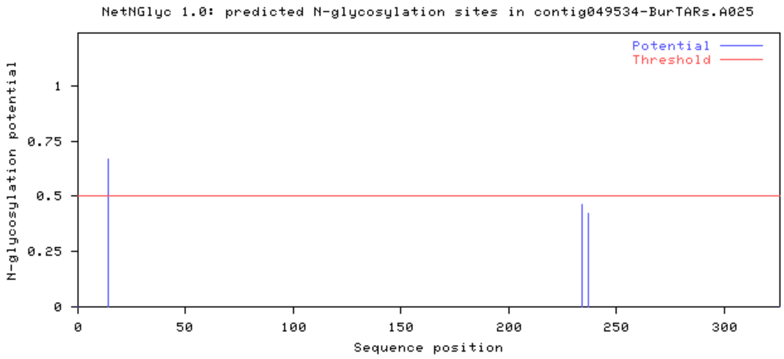

Graphics in PostScript

Output for 'contig041024-BurTARs.A026'

```
########################################################################################

    Warning: This sequence may not contain a signal peptide!!

    Proteins without signal peptides are unlikely to be exposed to
    the N-glycosylation machinery and thus may not be glycosylated
    (in vivo) even though they contain potential motifs.

    SignalP-NN euk predictions are as follows:

  # name                 Cmax  pos ?  Ymax  pos ?  Smax  pos ?  Smean ?  D     ?

    SignalP output is explained at http://www.cbs.dtu.dk/services/SignalP/output.html

########################################################################################

Name:  contig041024-BurTARs.A026    Length:  329
MEIQKAAELCFPQLLNSSCRKPTLHWSKAVLLNIVLSCISLITAALNLLVIISVSHFRKLHTPSNILLLSLAVSDFLVGL      80
LLMPLEIFRNTACWVLGDVMCSVYWYLTNNITCASIGNIVLISVDRYVAICDPLHYPTRITLAKVKLSVCLCWFFSIFYW     160
SLYLKDILVEPGRYNSCYGECVLIINDIAGIVDLALSFIVPVTVIIVLYMRVFVVAVSQARAMRSHVTAVTLQCSLNQAN     240
KSELKAARTLGVLVVVFLACYCPFYCYSLVDKNVVNDSSASFVVLVFYFNSCLNPLIYALFYPWFRKAIRCVITLLIFKH     320
DSSEVNVIX
...............N................................................................      80
.............................N..................................................     160
................................................................................     240
................................................................................     320
.........                                                                            400

(Threshold=0.5)
----------------------------------------------------------------------
SeqName      Position  Potential   Jury    N-Glyc
                                agreement result
----------------------------------------------------------------------
contig041024-BurTARs.A026   16 NSSC   0.7030     (9/9)   ++
contig041024-BurTARs.A026  110 NITC   0.7098     (9/9)   ++
contig041024-BurTARs.A026  240 NKSE   0.3682     (8/9)   -
contig041024-BurTARs.A026  276 NDSS   0.3992     (7/9)   -
----------------------------------------------------------------------
```

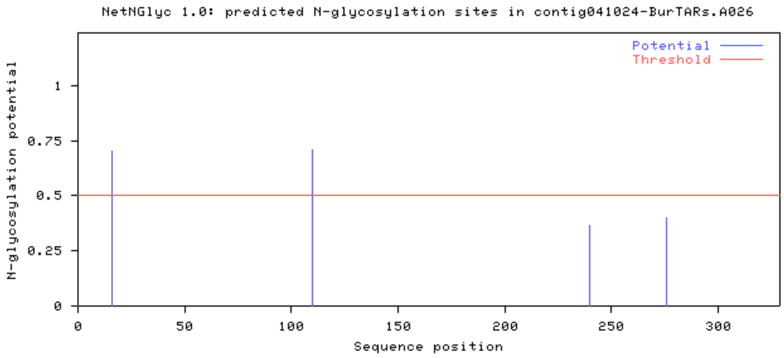

Graphics in PostScript

Output for 'contig034854-BurTARs.A027'

```
##########################################################################################

    Warning: This sequence may not contain a signal peptide!!

    Proteins without signal peptides are unlikely to be exposed to
    the N-glycosylation machinery and thus may not be glycosylated
    (in vivo) even though they contain potential motifs.

    SignalP-NN euk predictions are as follows:

  # name                Cmax  pos ?  Ymax  pos ?  Smax  pos ?  Smean ?  D     ?

    SignalP output is explained at http://www.cbs.dtu.dk/services/SignalP/output.html

##########################################################################################

Name:  contig034854-BurTARs.A027    Length:  330
MDTQGGAELCFPQLFNTSCKKPTTPLSQVLLFHILMPSMSLLTVTLNLLVIIAISHFRQLHSHTNILLLSLAAADFLNGL      80
LFMPGEILRNTACWFLGQLTCSLYNYVSYIIASASVGNMVLISVDRYVAICDPLHYPTRITEGKVKLCVCLCWLCSVLYS     160
YVILIDDLSQPGKHKSCYGKCIIFIEFIAGFVDLFISFIIPLTVIIVLYMRVFVVAVSQARAMRSQVTTVTLQLSVTLTA     240
TKSELKAARTLGVLVLVFLLCFCPYYIVSLFGDEVLNSASESIAFYFFLLNSCLNPLIYAMFYPWFRKAVKLIVTLQILQ     320
PGSCEVSILX
...............N................................................................      80
................................................................................     160
................................................................................     240
................................................................................     320
..........                                                                           400

(Threshold=0.5)
----------------------------------------------------------------------
SeqName      Position  Potential   Jury    N-Glyc
                     agreement result
----------------------------------------------------------------------
contig034854-BurTARs.A027   16 NTSC   0.5688     (7/9)    +
----------------------------------------------------------------------
```

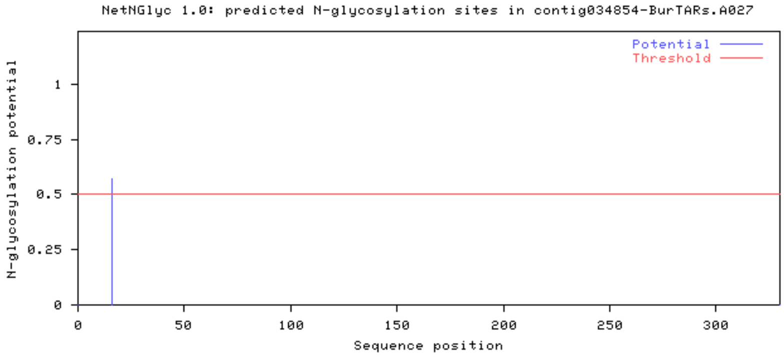

Graphics in PostScript

Output for 'contig057148-BurTARs.A028'

```
##########################################################################################

    Warning: This sequence may not contain a signal peptide!!

    Proteins without signal peptides are unlikely to be exposed to
    the N-glycosylation machinery and thus may not be glycosylated
    (in vivo) even though they contain potential motifs.
```

```
    SignalP-NN euk predictions are as follows:

  # name                 Cmax  pos ?  Ymax  pos ?  Smax  pos ?  Smean ?  D     ?

    SignalP output is explained at http://www.cbs.dtu.dk/services/SignalP/output.html

########################################################################################

Name:  contig057148-BurTARs.A028    Length:  327
MQKEAELCFPQLLNSSCRKPTFHWSKAVLLNIVLCCISLLTAALNLLVIISVSYFRQLHTPTNILLLSLAVSDFLTGLLM      80
LPLEIIRNTACWVLGDLMCSVYTYLTAGLFCASTWNIVLISVDRYVAICHPLHYPTRITVARVKLCVCLCWFCSTFYGSL     160
LTKDVLIKPARYNSCYGECVFVINDIAGFIDLVLSFIFPVSVIIVLYMRVFVVAVSQARAMRSHVTAVALQRSSNQANKS     240
ELKAARNLGVLVVVFLLCYLPFYCYSLIEVNVINDSSTFFLIIVFYFNSCLNPLMYALFYSWFRNAVKLIITLQIFKHDT     320
TEANMFX
.............N..................................................................      80
................................................................................     160
................................................................................     240
................................................................................     320
.......                                                                              400

(Threshold=0.5)
----------------------------------------------------------------------
SeqName      Position  Potential   Jury    N-Glyc
                      agreement result
----------------------------------------------------------------------
contig057148-BurTARs.A028   14 NSSC   0.6856     (9/9)    ++
contig057148-BurTARs.A028  238 NKSE   0.3593     (8/9)    -
contig057148-BurTARs.A028  274 NDSS   0.4007     (7/9)    -
----------------------------------------------------------------------
```

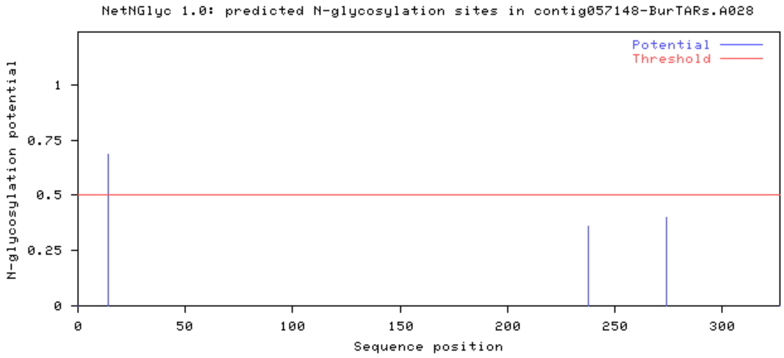

Graphics in PostScript

Output for 'contig059766-BurTARs.A029'

```
########################################################################################

    Warning: This sequence may not contain a signal peptide!!

    Proteins without signal peptides are unlikely to be exposed to
    the N-glycosylation machinery and thus may not be glycosylated
    (in vivo) even though they contain potential motifs.

    SignalP-NN euk predictions are as follows:

  # name                 Cmax  pos ?  Ymax  pos ?  Smax  pos ?  Smean ?  D     ?

    SignalP output is explained at http://www.cbs.dtu.dk/services/SignalP/output.html

########################################################################################

Name:  contig059766-BurTARs.A029    Length:  326
MEETELCFQQLFNTSCMRPRRPHFEIMLTYILLSFISLLTVILNLLVIISVSHFRQLHTPTNLLLLSLAVADFYVGLLLF      80
FQIVLIDGCWFLGDIMCTLYQYLAYVITSASIGTMVIISVDRYLAICYPLHYSTKITQQRVKIVVCLCWICSVIFQSLIL     160
MDNLEQPGRYNSCIGECVFVINYIAGLVDVTFSFIVPFTVIVVLYLRVFVVAVSQARAMRSQLAVTHQRSVTVTAKKSEL     240
KAAWTLGIVVFVFLICMCPYYCVALTGQDNLPSASSLTFVLCLVYFNSCLNPIIYVFFYPWFRKSIKVIVTLQILQPDSC     320
QATVLX
............N...................................................................      80
................................................................................     160
................................................................................     240
................................................................................     320
......                                                                               400

(Threshold=0.5)
----------------------------------------------------------------------
SeqName      Position  Potential   Jury    N-Glyc
                      agreement result
----------------------------------------------------------------------
contig059766-BurTARs.A029   13 NTSC   0.6332     (9/9)    ++
----------------------------------------------------------------------
```

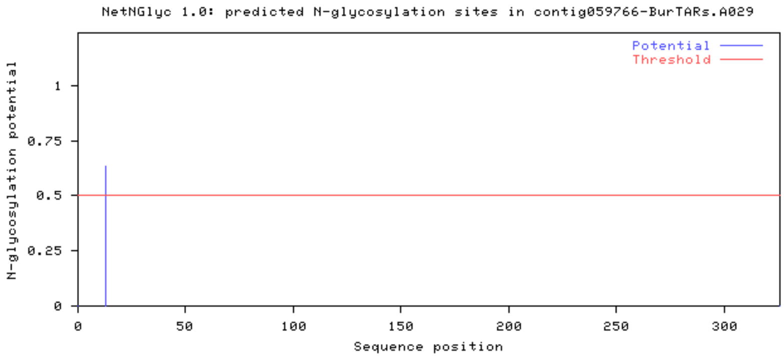

Graphics in PostScript

Output for 'contig065494-BurTARs.A030'

```
########################################################################################

    Warning: This sequence may not contain a signal peptide!!

    Proteins without signal peptides are unlikely to be exposed to
    the N-glycosylation machinery and thus may not be glycosylated
    (in vivo) even though they contain potential motifs.

    SignalP-NN euk predictions are as follows:

  # name                Cmax  pos ?  Ymax  pos ?  Smax  pos ?  Smean ?  D     ?

    SignalP output is explained at http://www.cbs.dtu.dk/services/SignalP/output.html

########################################################################################

Name:  contig065494-BurTARs.A030    Length:  326
MEETELCFQQLFNTSCMRPRRPHFEIMLTYILLSFISLLTVILNLLVIISVSHFRQLHTPTNLLLLSLAVADFYVGLLLF      80
FQIVLIDGCWFLGDIMCTLYQYLAYVITSASIGTMVIISVDRYLAICYPLHYSTKITQQRVKIVVCLCWICSVIFQSLIL     160
MDNLEQPGRYNSCIGECVFVINYIAGLVDVTFSFIVPFTVIVVLYLRVFVVAVSQARAMRSQLAVTHQRSVTVTAKKSEL     240
KAAWTLGIVVVVFLICMCPYYCVALTGQDSLPSASSLTFVLCLAYFNSCLNPIIYVFFYPWFRKSIKVIVTLQILQPDSS     320
KVTMRX
............N...................................................................      80
................................................................................     160
................................................................................     240
................................................................................     320
......                                                                               400

(Threshold=0.5)
----------------------------------------------------------------------
SeqName      Position  Potential   Jury    N-Glyc
                                agreement result
----------------------------------------------------------------------
contig065494-BurTARs.A030   13 NTSC   0.6332     (9/9)    ++
----------------------------------------------------------------------
```

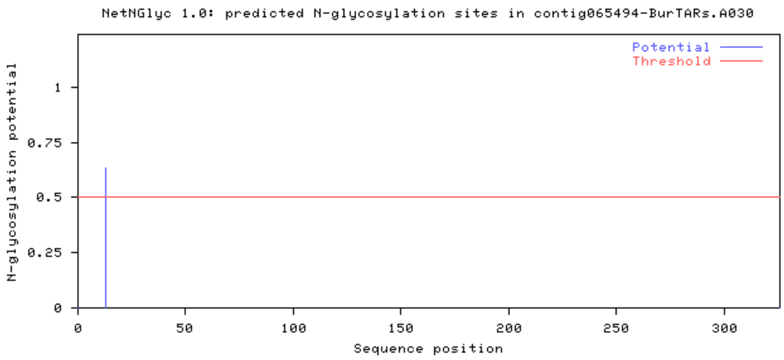

Graphics in PostScript

Output for 'contig057305-BurTARs.A031'

```
########################################################################################

    Warning: This sequence may not contain a signal peptide!!

    Proteins without signal peptides are unlikely to be exposed to
    the N-glycosylation machinery and thus may not be glycosylated
    (in vivo) even though they contain potential motifs.
```

```
    SignalP-NN euk predictions are as follows:

  # name                 Cmax  pos ?  Ymax  pos ?  Smax  pos ?  Smean ?  D     ?

    SignalP output is explained at http://www.cbs.dtu.dk/services/SignalP/output.html

##########################################################################################

Name:  contig057305-BurTARs.A031    Length:  324
MAETELCFPNLNSSCSRVKRSPSDSVLIYVILTIISLLTVALNLLVIISISHFKQLHTPTNHLLLSLAVSDFFVGLNMCF      80
QSMLVDGCWYLGDLMCVLYYVFDIVVTSASVGTMVLISVDRYVAICDPLHYPTKVTPKRVQTCVLMCWICSLLVVGVLLK     160
DNLDKPGRFNSCFGECVIYVDFAIQVTDLILTILLPITVIVILYVRVFAVAVFQIRAMQPHVPAVTQRGKVSPKKSELKA     240
ARTLGIVIVAFLICLFPYYSVILSGQDTLLDILSVTFFLCLFYFNSCLNPIIYAFFYPWFRKSVKLIVTFQIVKSGSSDA     320
SMLX
...........N....................................................................      80
................................................................................     160
................................................................................     240
................................................................................     320
....                                                                                 400

(Threshold=0.5)
----------------------------------------------------------------------
SeqName      Position  Potential   Jury    N-Glyc
                                agreement result
----------------------------------------------------------------------
contig057305-BurTARs.A031   12 NSSC   0.7291     (9/9)    ++
----------------------------------------------------------------------
```

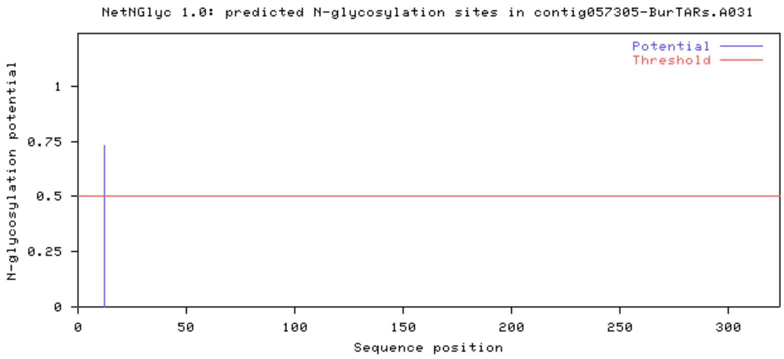

Graphics in PostScript

Output for 'contig006087-BurTAR.B032'

```
##########################################################################################

    Warning: This sequence may not contain a signal peptide!!

    Proteins without signal peptides are unlikely to be exposed to
    the N-glycosylation machinery and thus may not be glycosylated
    (in vivo) even though they contain potential motifs.

    SignalP-NN euk predictions are as follows:

  # name                 Cmax  pos ?  Ymax  pos ?  Smax  pos ?  Smean ?  D     ?

    SignalP output is explained at http://www.cbs.dtu.dk/services/SignalP/output.html

##########################################################################################

Name:  contig006087-BurTAR.B032     Length:  315
MELFNVTVNTVSFLLCDSQKNKLCVLLYVVLSSFMLLTICGNLLVIISIIYFRYLHTPTNYLILSMAVADLLIGALIFPL      80
SMTVSLKPCLYIYSLLCNLRSTMDVTMGVSSLLNLCCISVDRYYAVCHPLIYKTKITDCVAMKMGLGSWAVAILCGIFVF     160
LLFFILDECDTSCLFALIAASVVVYYIPTIVLLFMYTKILVVALRQARSIHNTISQNTKSKAVSSTERKATKTLTIVIGI     240
FLIFWVPLFLSYSFVPLDSFILYVLLEPFNWFAISNSMLNPFIYAFFYTWFRRAFKMIISGKIFQGDVTNIKLHX
....N...........................................................................      80
................................................................................     160
................................................................................     240
...........................................................................          320

(Threshold=0.5)
----------------------------------------------------------------------
SeqName      Position  Potential   Jury    N-Glyc
                                agreement result
----------------------------------------------------------------------
contig006087-BurTAR.B032     5 NVTV   0.7989     (9/9)    +++
----------------------------------------------------------------------
```

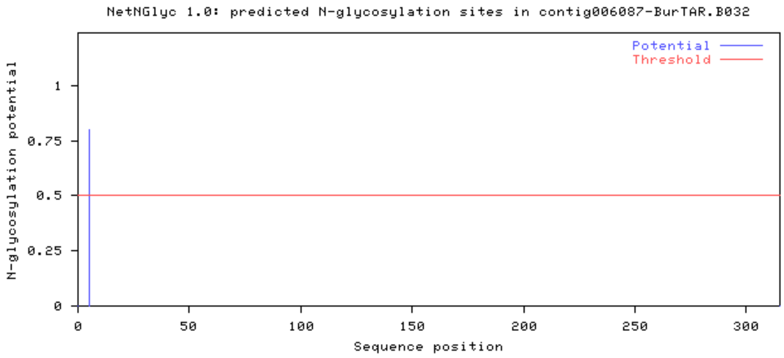

Graphics in PostScript

Output for 'contig025313-BriTAR.A002'

```
########################################################################################

    Warning: This sequence may not contain a signal peptide!!

    Proteins without signal peptides are unlikely to be exposed to
    the N-glycosylation machinery and thus may not be glycosylated
    (in vivo) even though they contain potential motifs.

    SignalP-NN euk predictions are as follows:

  # name                Cmax  pos ?  Ymax  pos ?  Smax  pos ?  Smean ?  D     ?

    SignalP output is explained at http://www.cbs.dtu.dk/services/SignalP/output.html

########################################################################################

Name:  contig025313-BriTAR.A002     Length:  331
MDSQDRIELCYPELANLSCRGLTRPQPEAVLLYTLFLSISVLTVALNVLVIISISHFRQLHMPTNVLLLSLAISDLLVGL      80
LVMPVETIRFIETCWLLGDLMCAFSYIIGFTLTSASVGNMVLISIDRYVAICYPLQYPTKITHSRAELSVTLCWACSLLY     160
NGLILKEHLRQPDRHNTCHGQCLVVINYVSGAIDLVFTFIGPCSVIIILYMRVFVVAVSQAHAMRSHITAAAAGRVKITA     240
KKSEKKAARTLGVVIFVFLMSFCPYYYPSLAGQDISNSASSWAIVSWMLYFNSCLNPLIYAFFYPWFRKAIWFIVSLKIL     320
EKGSSQANILX
...............N................................................................      80
................................................................................     160
................................................................................     240
................................................................................     320
...........                                                                          400

(Threshold=0.5)
----------------------------------------------------------------------
SeqName      Position  Potential   Jury    N-Glyc
                     agreement result
----------------------------------------------------------------------
contig025313-BriTAR.A002   16 NLSC   0.6719     (8/9)   +
----------------------------------------------------------------------
```

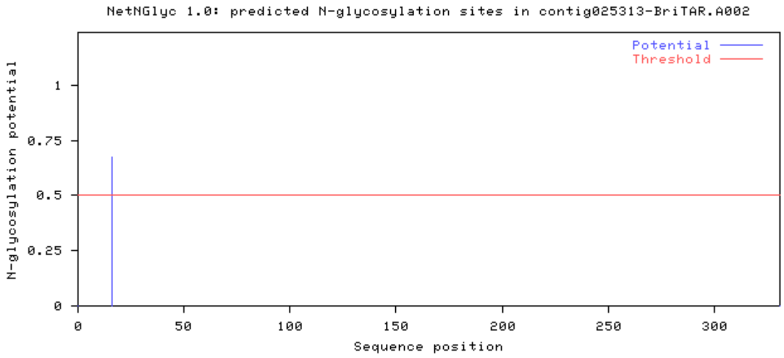

Graphics in PostScript

Output for 'contig029633-BriTAR.A003'

```
########################################################################################

    Warning: This sequence may not contain a signal peptide!!

    Proteins without signal peptides are unlikely to be exposed to
    the N-glycosylation machinery and thus may not be glycosylated
    (in vivo) even though they contain potential motifs.
```

```
    SignalP-NN euk predictions are as follows:

  # name                 Cmax  pos ?  Ymax  pos ?  Smax  pos ?  Smean ?  D     ?

    SignalP output is explained at http://www.cbs.dtu.dk/services/SignalP/output.html

##########################################################################################

Name:  contig029633-BriTAR.A003     Length:  326
MDGTGGPPLCFPNLNSSCRRLLRPTSQAALLYTLLASVSLLTVVLKLLVVVSISHFKQLHTPNALLLSLAMSDLLLGFL      80
VMPIEGLCYIETCWLLGRLMCALSPYLSYCLLSFSLGSMVLISVDRYIAICDPLLYSSKITVNRVKLSVCVCWACSLLYN     160
GCILMGHIGWPDRFSSCHGECVVFISRTSGTVDFFLSFLGPCAVMFVMYMRVFVVAVSQVRAIQSQAAVRAVPAAKKSEL     240
KAARTLGILIAVFVMCFCPYYYPSLAGVDTFLSLPSYAMVCWIMLINACVNPVIYVLFYPWFRRALRFIVTLRILQPHSR     320
DVKILX
..............N.................................................................      80
................................................................................     160
................................................................................     240
................................................................................     320
......                                                                               400

(Threshold=0.5)
----------------------------------------------------------------------
SeqName      Position  Potential   Jury    N-Glyc
                       agreement result
----------------------------------------------------------------------
contig029633-BriTAR.A003    15 NSSC    0.6958     (9/9)    ++
----------------------------------------------------------------------
```

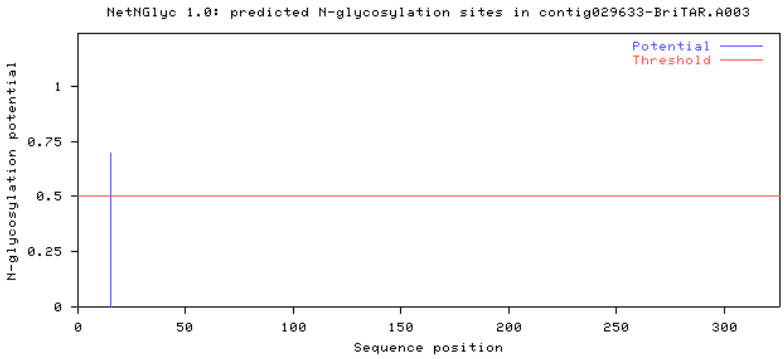

Graphics in PostScript

Output for 'contig084868-BriTARs.A014'

```
##########################################################################################

    Warning: This sequence may not contain a signal peptide!!

    Proteins without signal peptides are unlikely to be exposed to
    the N-glycosylation machinery and thus may not be glycosylated
    (in vivo) even though they contain potential motifs.

    SignalP-NN euk predictions are as follows:

  # name                 Cmax  pos ?  Ymax  pos ?  Smax  pos ?  Smean ?  D     ?

    SignalP output is explained at http://www.cbs.dtu.dk/services/SignalP/output.html

##########################################################################################

Name:  contig084868-BriTARs.A014    Length:  329
METQDEAELCFPQLFNTSCKKPKTSLSQVLLPYVVVFSVSLLTVALNLVDISLSSFRQLHTPTNSLLLSLAVSDFLIGLL      80
MMPAKILGDTACWFLGQLTCSLYSYICFIITSASVGIMVLISVDRYVAICDPLHYPTRITERRVKLCVCLCWLCSVIYNI     160
LFIKNDLLQGERHTSCYGECVFVIDYIVGTTDIVLTFIAPVTVIVVLYMRVFVVAVSQARAMRSHVTAVTLQLSVTLTAK     240
KSEIKAARTLGVLVLVFLLGFCPYYIVSLLGNELFNSSSASIAIYLYYFNSCLNPLIYAMFYPWFRKAVKFVVTLQILQP     320
GSCEVSILX
...............N................................................................      80
................................................................................     160
................................................................................     240
................................................................................     320
.........                                                                            400

(Threshold=0.5)
----------------------------------------------------------------------
SeqName      Position  Potential   Jury    N-Glyc
                       agreement result
----------------------------------------------------------------------
contig084868-BriTARs.A014   16 NTSC   0.5836     (7/9)   +
contig084868-BriTARs.A014  276 NSSS   0.4874     (4/9)   -
----------------------------------------------------------------------
```

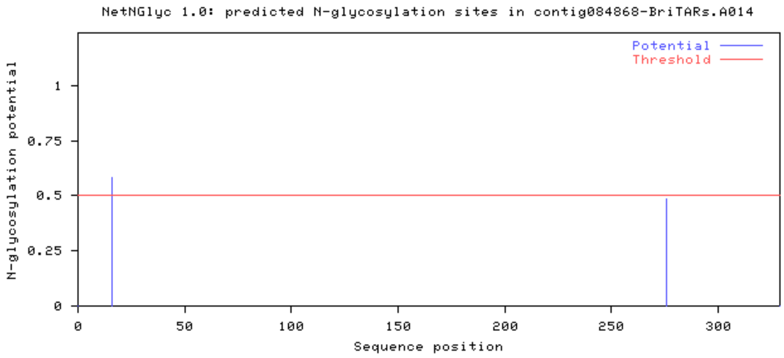

Graphics in PostScript

Output for 'contig084876-BriTARs.A015'

```
##########################################################################################

    Warning: This sequence may not contain a signal peptide!!

    Proteins without signal peptides are unlikely to be exposed to
    the N-glycosylation machinery and thus may not be glycosylated
    (in vivo) even though they contain potential motifs.

    SignalP-NN euk predictions are as follows:

  # name                Cmax  pos ?  Ymax  pos ?  Smax  pos ?  Smean ?  D     ?

    SignalP output is explained at http://www.cbs.dtu.dk/services/SignalP/output.html

##########################################################################################

Name:  contig084876-BriTARs.A015    Length:  330
MDTQDVAELCFPQLFNTSCKKPITPLSEFVFLHVVLSSISLLTVTLNLLVIISVSHYRQLHTPTNILLLSLAVSDFLVGL      80
LLMPGEILRNTACWFLGDLTCFMYDYMSLIVTSTSVGDMVLISIDRYVALCDPLHYPTRITDRRVKLSVCLCWLSSVFYS     160
SLFVKDDLTHSGKHNSCYGECTIVVDLITGTIDLLLTFFVPVTVIVVLYLRVFVVAVSQARAMRSHVTAAALQLSVTLTT     240
KRSELKAARTLGVLVVVFLLCFCPYYCVTLARDDLLNSSSVSFLLYLFYFNSCLNPLIYALLYPWFRKAVKLIISLHILQ     320
PGSCEISILX
...............N................................................................      80
................................................................................     160
................................................................................     240
....................................N...........................................     320
..........                                                                           400

(Threshold=0.5)
----------------------------------------------------------------------
SeqName      Position  Potential   Jury    N-Glyc
                      agreement result
----------------------------------------------------------------------
contig084876-BriTARs.A015    16 NTSC    0.5822     (7/9)    +
contig084876-BriTARs.A015   277 NSSS    0.5948     (6/9)    +
----------------------------------------------------------------------
```

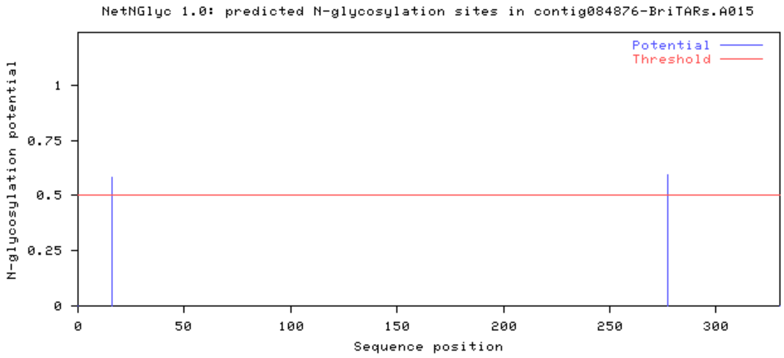

Graphics in PostScript

Output for 'contig084880-BriTARs.A016'

```
##########################################################################################

    Warning: This sequence may not contain a signal peptide!!

    Proteins without signal peptides are unlikely to be exposed to
    the N-glycosylation machinery and thus may not be glycosylated
    (in vivo) even though they contain potential motifs.
```

```
    SignalP-NN euk predictions are as follows:

  # name                Cmax  pos ?  Ymax  pos ?  Smax  pos ?  Smean ?  D     ?

    SignalP output is explained at http://www.cbs.dtu.dk/services/SignalP/output.html

########################################################################################

Name:  contig084880-BriTARs.A016    Length:  333
MEIQMHPEAELCFPELLNSSCRKPTLHWSKTVLLNVGLSSISLITAALNLFIIISVSHFRQLHTPSNIIILSLAVSDFFV      80
CFLLMPVEIFKNTACWVFGDLMCSLYTYLSCILINASFEMIILVSIDRYVAICDPLHYPTRITVPRVKLCVFLCWFYAIL     160
YNIIYTKHALINPGRYGSCYGECVFVVDDIIGIVDFVVSLIVPVTIIVVLYTRVFVVVVSQARAMRSHVTAVTLQRPLNQ     240
ANKSELKAARNLGILVVVFLACYCPFYCYFFLAGNEVNASSASSILIVYYFNSCLNPLMYALFYPWFRNAVKLIITLQIL     320
KANSSEINILQRX
.................N..............................................................      80
..................................N.............................................     160
................................................................................     240
................................................................................     320
.............                                                                        400

(Threshold=0.5)
----------------------------------------------------------------------
SeqName      Position  Potential   Jury    N-Glyc
                                agreement result
----------------------------------------------------------------------
contig084880-BriTARs.A016   18 NSSC   0.6893     (9/9)   ++
contig084880-BriTARs.A016  115 NASF   0.5100     (6/9)   +
contig084880-BriTARs.A016  242 NKSE   0.3964     (7/9)   -
contig084880-BriTARs.A016  278 NASS   0.3588     (8/9)   -
contig084880-BriTARs.A016  323 NSSE   0.4093     (6/9)   -
----------------------------------------------------------------------
```

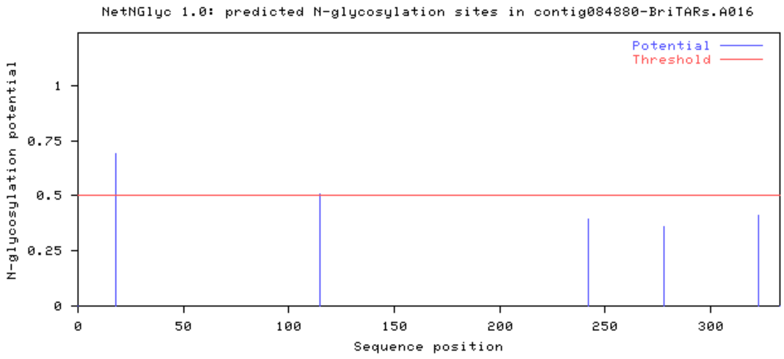

Graphics in PostScript

Output for 'contig084886-BriTARs.A017'

```
########################################################################################

    Warning: This sequence may not contain a signal peptide!!

    Proteins without signal peptides are unlikely to be exposed to
    the N-glycosylation machinery and thus may not be glycosylated
    (in vivo) even though they contain potential motifs.

    SignalP-NN euk predictions are as follows:

  # name                Cmax  pos ?  Ymax  pos ?  Smax  pos ?  Smean ?  D     ?

    SignalP output is explained at http://www.cbs.dtu.dk/services/SignalP/output.html

########################################################################################

Name:  contig084886-BriTARs.A017    Length:  327
MQKEAELCFPQLINSSCRKPTLHWSKAVLLNIVLSCISLLTAALNLLVIISISYFRQLHTPTNILLLSLAVSDFLTGLLM      80
LPLEIIRNTACWVLGDLMCSVYTCLTTGLFCVSTWNVVLISVDRYVAICHPLHYPTRITVARVKLCVCLCWFYSTFYGSL     160
LTKDVLIKPARYNSCYGECVFVINDIAGFIDLVLSFIVPVSVIIVLYMRVFVVAVSQARAMRSRVTAVTLQRSSNQAKKS     240
ELKAARTLGVLVVGFLACYLPFYCYSLIEVNVINDSSTFFLIIVLYFNSCLNPLMYALFYSWFRNAVKLIITLQIFKHDT     320
SEANMFX
.............N..................................................................      80
................................................................................     160
................................................................................     240
................................................................................     320
.......                                                                              400

(Threshold=0.5)
----------------------------------------------------------------------
SeqName      Position  Potential   Jury    N-Glyc
                                agreement result
----------------------------------------------------------------------
contig084886-BriTARs.A017   14 NSSC   0.6319     (9/9)   ++
contig084886-BriTARs.A017  274 NDSS   0.4004     (7/9)   -
----------------------------------------------------------------------
```

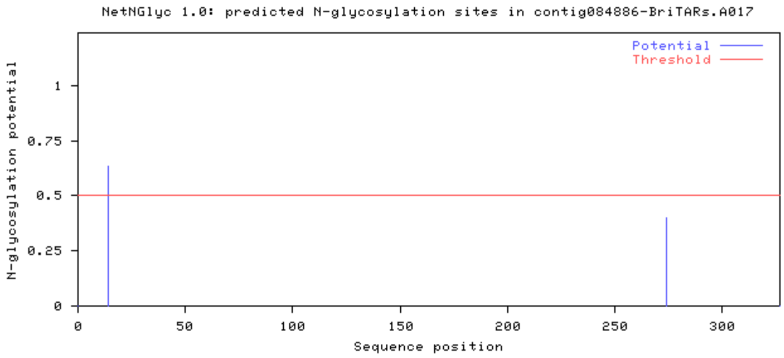

Graphics in PostScript

Output for 'contig084887-BriTARs.A018'

```
########################################################################################

    Warning: This sequence may not contain a signal peptide!!

    Proteins without signal peptides are unlikely to be exposed to
    the N-glycosylation machinery and thus may not be glycosylated
    (in vivo) even though they contain potential motifs.

    SignalP-NN euk predictions are as follows:

  # name                Cmax  pos ?  Ymax  pos ?  Smax  pos ?  Smean ?  D     ?

    SignalP output is explained at http://www.cbs.dtu.dk/services/SignalP/output.html

########################################################################################

Name:  contig084887-BriTARs.A018    Length:  329
MEIPKGVELCFPQLLNSSCRKPTLHWSKAVLLNIVLSCISLLTAALNLLVIISVSHFRQLHTPSNILLLSLAVSDFFVGL      80
LLLPLEIFRKTSCWVLGDRMCSAYWYLTSNIICASIGNIVLISVDRYVAICDPLHYPSRITLAKVKLSVCLCWFYAFFYS     160
NLYTKDIMIEPSRYNSCFGECVFFSSNIAIVVDLILFFFVPVTVIIVLYMRVFVVAVSQARAMRSHVTALTLQRSLNQTN     240
KSELKAARTLGILVVVFLACFSPLYCYSLVDENAIIDPAASFVVIIFYINSCLNPLIYALFYPWFRNAVKLIITLEIFKY     320
DTSGANILX
...............N................................................................      80
................................................................................     160
................................................................................     240
................................................................................     320
.........                                                                            400

(Threshold=0.5)
----------------------------------------------------------------------
SeqName      Position  Potential   Jury    N-Glyc
                     agreement result
----------------------------------------------------------------------
contig084887-BriTARs.A018   16 NSSC   0.6894     (9/9)   ++
contig084887-BriTARs.A018  237 NQTN   0.4627     (4/9)   -
contig084887-BriTARs.A018  240 NKSE   0.4129     (5/9)   -
----------------------------------------------------------------------
```

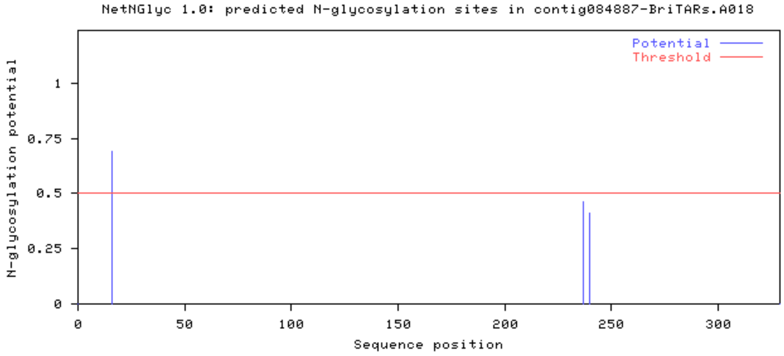

Graphics in PostScript

Output for 'contig086337-BriTARs.A019'

```
########################################################################################

    Warning: This sequence may not contain a signal peptide!!

    Proteins without signal peptides are unlikely to be exposed to
    the N-glycosylation machinery and thus may not be glycosylated
```

```
    (in vivo) even though they contain potential motifs.

    SignalP-NN euk predictions are as follows:

  # name                Cmax  pos ?  Ymax  pos ?  Smax  pos ?  Smean ?  D     ?

    SignalP output is explained at http://www.cbs.dtu.dk/services/SignalP/output.html

##########################################################################################

Name:  contig086337-BriTARs.A019    Length:  326
MEETELCFPKLLNISCRRPKRPHFEIMLTYILLSFISLLTVILNLLVIISISHFRQLHTPTNFLLLSLAVADFFVGFLMF      80
FQIVLIDGCWFLGDIMCTLYQYLAFIITSASIGTMVIISADRYLAICYPLHYSTKITQQRINICISLCWFFSVIFQSLIV     160
KDNLKQPGKYNSCIGECVFVVNYIAGIFDLLFSFIVPITVIVVLYLRVFVVAVTQARAMRCQLAVTHQRSVTVTVMKSEL     240
KAARTLGVVVVVFLICMCPYYCVALTGQDNFLNASSAAFVICLVYFNSCLNPIIYVFFYPWFRKSIKLIATLQILQPDSC     320
ETNMHX
............N...................................................................      80
................................................................................     160
................................................................................     240
................................................................................     320
......                                                                               400

(Threshold=0.5)
----------------------------------------------------------------------
SeqName      Position  Potential   Jury    N-Glyc
                      agreement result
----------------------------------------------------------------------
contig086337-BriTARs.A019    13 NISC   0.7890     (9/9)    +++
contig086337-BriTARs.A019   273 NASS   0.4887     (6/9)    -
----------------------------------------------------------------------
```

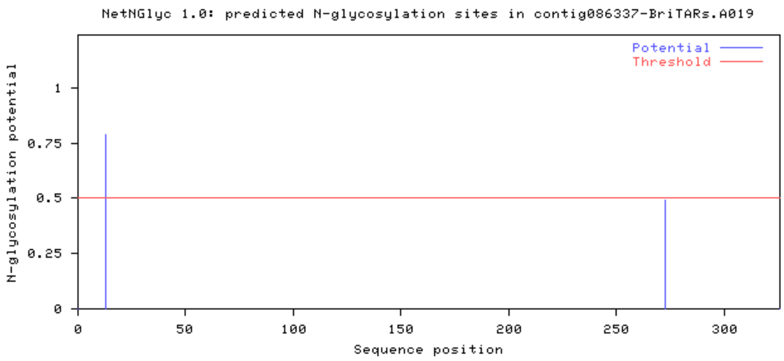

Graphics in PostScript

Output for 'contig086344-BriTARs.A020'

```
##########################################################################################

    Warning: This sequence may not contain a signal peptide!!

    Proteins without signal peptides are unlikely to be exposed to
    the N-glycosylation machinery and thus may not be glycosylated
    (in vivo) even though they contain potential motifs.

    SignalP-NN euk predictions are as follows:

  # name                Cmax  pos ?  Ymax  pos ?  Smax  pos ?  Smean ?  D     ?

    SignalP output is explained at http://www.cbs.dtu.dk/services/SignalP/output.html

##########################################################################################

Name:  contig086344-BriTARs.A020    Length:  327
MEETELCFPQLFNSSCVRQKHSQIEAVFLYTPLSSISVLTTILNLLVIISIAHFKQLHTPTNLLLLSLAVSDFFVGLIMA      80
CQISLLDGCWFLGDHMCALYTTLDYIVTSASVGTMVFISADRYVAICDPLHYPTEITIKRISVCICTCWACSILYNSLIM     160
KDNFKQPGRYNSCSGDCAVVIDYFVGIFDFVLTFVGPVIVIIVLYLRVFVVAVSQARAMRSHITALRLQGSETVHAKKSE     240
LKAARTLGVLVIAFLICLFPFFCSSMVGQNSFFDIRSVPFERLLFYFNSCLNPLIYTFCYPWFLKSIKLIVTFKIFRHGS     320
SEASILX
............N...................................................................      80
................................................................................     160
................................................................................     240
................................................................................     320
.......                                                                              400

(Threshold=0.5)
----------------------------------------------------------------------
SeqName      Position  Potential   Jury    N-Glyc
                      agreement result
----------------------------------------------------------------------
contig086344-BriTARs.A020    13 NSSC   0.6741     (9/9)    ++
----------------------------------------------------------------------
```

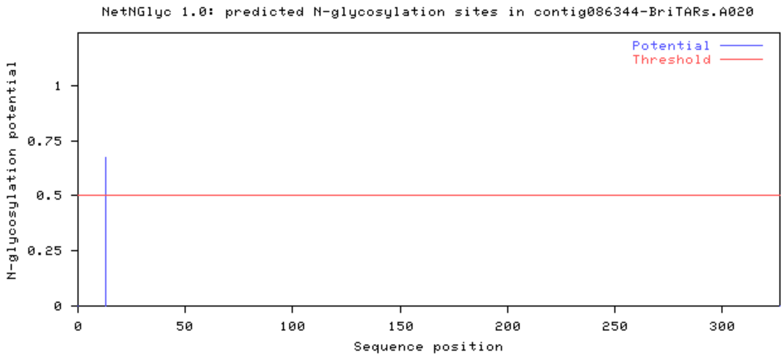

Graphics in PostScript

Output for 'contig086351-BriTARs.A021'

```
##########################################################################################

    Warning: This sequence may not contain a signal peptide!!

    Proteins without signal peptides are unlikely to be exposed to
    the N-glycosylation machinery and thus may not be glycosylated
    (in vivo) even though they contain potential motifs.

    SignalP-NN euk predictions are as follows:

  # name                Cmax  pos ?  Ymax  pos ?  Smax  pos ?  Smean ?  D     ?

    SignalP output is explained at http://www.cbs.dtu.dk/services/SignalP/output.html

##########################################################################################

Name:  contig086351-BriTARs.A021    Length:  324
MAETELCFPNLNSSCSRVKRSPSESVLIYVILTIISLLTVALNLLVIISISHFKQLHTPTNYLLLSLAVSDFFVGFNMCF      80
QSMLIDGCWYLGDLMCVLYYVFEIVVTSASVGTMVLISVDRYVAICDPLHYPTKVTPKRVQTCVLICWICSLLVVGVLLK     160
DNLDKPGRFNSCFGECVIYIDFAIQVTDLIFTILLPITVIVILYVRVFAVAVFQIRAMQHHVAAGTQRGKVSPKKSELKA     240
ARTLGIVIVAFLICLFPYYCVILSGQDTLLDILSLAFFLCLFYFNSFLNPIIYALFYPWFRKSVKLIVTFQIVKSGSSDA     320
SMLX
...........N....................................................................      80
................................................................................     160
................................................................................     240
................................................................................     320
....                                                                                 400

(Threshold=0.5)
----------------------------------------------------------------------
SeqName      Position  Potential   Jury    N-Glyc
                                agreement result
----------------------------------------------------------------------
contig086351-BriTARs.A021   12 NSSC   0.7292     (9/9)    ++
----------------------------------------------------------------------
```

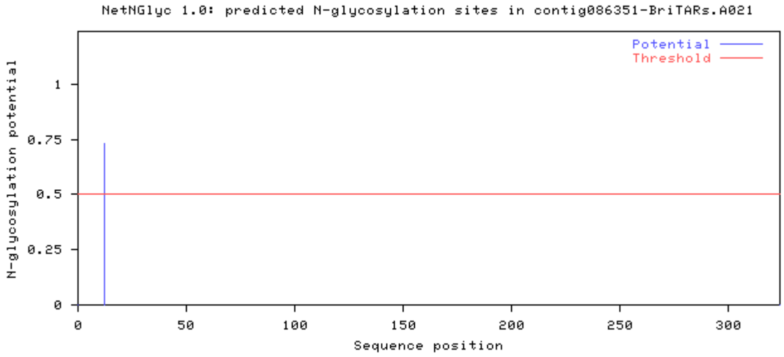

Graphics in PostScript

Output for 'contig082565-BriTARs.A022'

```
##########################################################################################

    Warning: This sequence may not contain a signal peptide!!

    Proteins without signal peptides are unlikely to be exposed to
    the N-glycosylation machinery and thus may not be glycosylated
    (in vivo) even though they contain potential motifs.
```

```
    SignalP-NN euk predictions are as follows:

  # name                 Cmax  pos ?  Ymax  pos ?  Smax  pos ?  Smean ?  D     ?

    SignalP output is explained at http://www.cbs.dtu.dk/services/SignalP/output.html

##########################################################################################

Name:  contig082565-BriTARs.A022    Length:  330
MDTQDGAELCFPQLFNISCKKPTTPLSQVLLIYTVVSSMSLLTVTLNLLVIIAVSHFRQLHTPTNILLLSLAVTDFLVGL      80
LFMPGEILRNTACWFLGQLTCSLYNYASYIIASASVGNMVLISVDRYVAICDPLHYPTRITERRVKLCVCLCWLCSVFYS     160
YVILIDDLSQPGKHKSCYGKCIIFIEFIAGFVDLVLAFIIPLTVIIVLYMRVFVVAVSQARAMRSQVTAVTLQLSVTLTA     240
KKSELKAARTLGVLVLVFLLCFCSYYIISLFGNELLNSSSASIVIYLYYFNSCLNPLIYAMFYPWFRKAVKLIVTLQILQ     320
PGSCEVSILX
...............N................................................................      80
................................................................................     160
................................................................................     240
....................................N...........................................     320
..........                                                                           400

(Threshold=0.5)
----------------------------------------------------------------------
SeqName      Position  Potential   Jury    N-Glyc
                      agreement result
----------------------------------------------------------------------
contig082565-BriTARs.A022    16 NISC   0.6414     (8/9)    +
contig082565-BriTARs.A022   277 NSSS   0.5827     (6/9)    +
----------------------------------------------------------------------
```

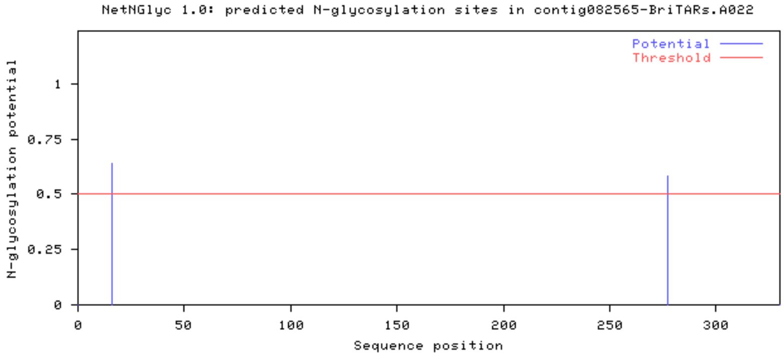

Graphics in PostScript

Output for 'contig035253-BriTAR.B025'

```
##########################################################################################

    Warning: This sequence may not contain a signal peptide!!

    Proteins without signal peptides are unlikely to be exposed to
    the N-glycosylation machinery and thus may not be glycosylated
    (in vivo) even though they contain potential motifs.

    SignalP-NN euk predictions are as follows:

  # name                 Cmax  pos ?  Ymax  pos ?  Smax  pos ?  Smean ?  D     ?

    SignalP output is explained at http://www.cbs.dtu.dk/services/SignalP/output.html

##########################################################################################

Name:  contig035253-BriTAR.B025     Length:  322
MTNIVNDLHPCYVIQDSKYMLTNSPSIICVFLYAFLALLSVITICGNLLVIISVIYFKQLHTPTNYLILSLAVADLLVGI      80
IAFPLSMAFSLSSCLYHEGLFCKVRGTFDISLSTCSILNLCCISIDRYHAVCQPLTYQTKISPRVVVFMILMSWGVSGII     160
GISVTIAGFNNEKCEESCLIDVLIESTVGPMLSFYLPVTMMLFIYLKIFFVALRQARRIQNTTKCGQTASIMERKATKTL     240
AIVLGVFIFCWSPFFLSITFPPFTSDSVPVPVIETLNWLTLANSMLNPFIYAFFYSWFRSAIRMIICGKIFQGDFANTNM     320
TX
................................................................................      80
................................................................................     160
............................................................N...................     240
................................................................................     320
..                                                                                   400

(Threshold=0.5)
----------------------------------------------------------------------
SeqName      Position  Potential   Jury    N-Glyc
                      agreement result
----------------------------------------------------------------------
contig035253-BriTAR.B025  221 NTTK   0.5736     (6/9)    +
contig035253-BriTAR.B025  319 NMTX   0.4856     (5/9)    -
----------------------------------------------------------------------
```

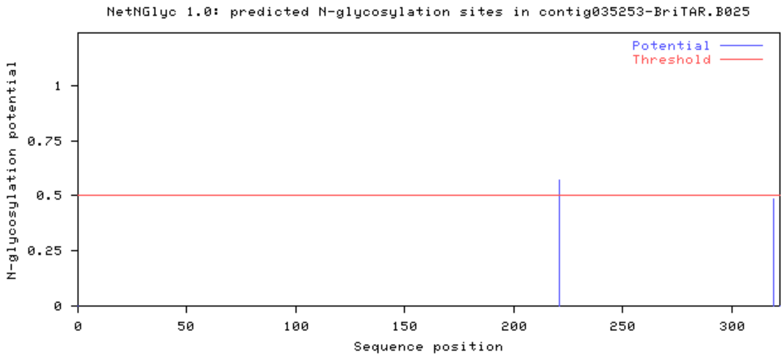

Graphics in PostScript

Explain the output. Go back.
